# Single-colony resolution of CRISPR–Cas adaptation in *E. coli* reveals altered spacer-source bias during solid-phase growth

**DOI:** 10.1093/nar/gkaf1044

**Published:** 2025-10-21

**Authors:** Jack Braithwaite, Christopher Cannon, Ronald Chalmers, Harry Edwards

**Affiliations:** School of Life Sciences, University of Nottingham, Queens Medical Centre, Nottingham NG7 2UK, United Kingdom; School of Life Sciences, University of Nottingham, Queens Medical Centre, Nottingham NG7 2UK, United Kingdom; School of Life Sciences, University of Nottingham, Queens Medical Centre, Nottingham NG7 2UK, United Kingdom; School of Life Sciences, University of Nottingham, Queens Medical Centre, Nottingham NG7 2UK, United Kingdom

## Abstract

CRISPR–Cas systems provide adaptive immunity by integrating short DNA fragments from mobile genetic elements into host arrays. While the core biochemical mechanism of adaptation is well defined, its modulation by physiological contexts is less well understood. Here, we present a visual papillation assay that enables single-colony detection of CRISPR–Cas adaptation in *Escherichia coli*. Spacer acquisition restores the reading frame of a disrupted *lacZ* gene, forming blue papillae on lactose X-gal plates. The assay is semi-quantitative, highly sensitive, capable of detecting single events among 10^9^ cells, and responds predictably to Cas1–Cas2 expression levels. Spacer mapping revealed a major shift in source bias: in liquid culture, 64% of spacers were plasmid-derived, but on solid medium this dropped to ∼9%. Adjusting inducer concentration to match liquid conditions did not restore plasmid bias, indicating a physiological basis linked to colony growth. Accounting for the molar excess of chromosomal DNA, the 9% plasmid share reflects near-neutral DNA source sampling rather than plasmid overrepresentation. These findings suggest that the spatial and metabolic structure of colonies strongly shapes the adaptation landscape. The assay provides a scalable platform for dissecting condition-specific features of CRISPR–Cas adaptation, including spacer origin, sequence features, and growth context.

## Graphical Abstract

**Graphical Abstract:**
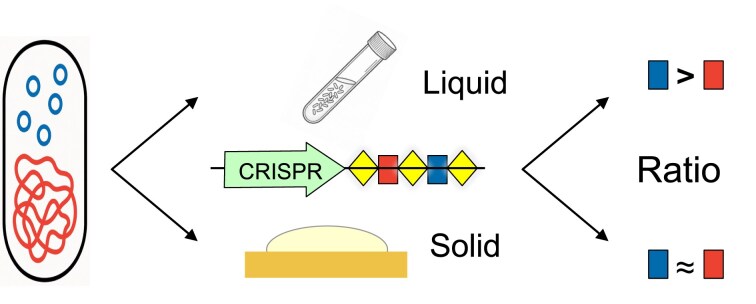

## Introduction

**Figure 1.**
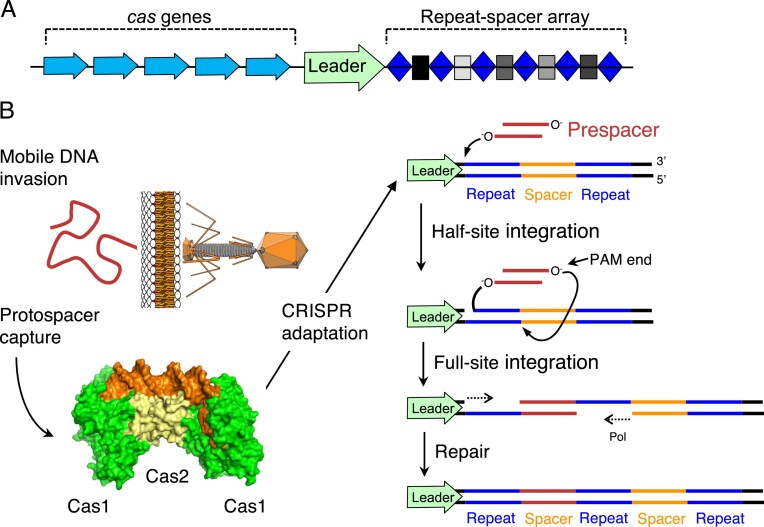
Spacer Integration by *Type I-E* CRISPR–Cas in *E. coli*. (**A**) A typical CRISPR–Cas system consists of a cluster of *cas* genes adjacent to a CRISPR array composed of short spacer sequences (squares) separated by identical direct repeats (diamonds). The upstream leader sequence orients the array and directs insertion of new spacers at the leader-proximal end. Spacers are captured from mobile genetic elements and later used to recognize and destroy matching sequences. (**B**) Upon entry of foreign DNA, Cas1–Cas2 captures a prespacer fragment and positions it at the first repeat via leader sequence interactions. The 3′ ends of the prespacer are inserted at opposite ends of the repeat by direct transesterification reactions. Host repair machinery fills the resulting single-stranded gaps, duplicating the repeat and completing integration. This mechanism of repeat duplication is chemically and mechanistically identical to the model first proposed in 1979 for the integration of bacterial transposons and later shown to apply to retroviral integrases [38–40]. Bilayer, CC0; phage, CC BY-SA 3.0, https://commons.wikimedia.org/wiki/File:PhageExterior.svg.

CRISPR–Cas systems enable prokaryotes to acquire heritable immunity against foreign genetic elements [1]. Each system comprises a cluster of *cas* genes next to a repeat–spacer array (Fig. 1A). Spacers are short DNA fragments captured from mobile genetic elements and used by effector proteins such as Cas9 to recognize and destroy matching sequences [2–4]. CRISPR–Cas systems are divided into two major classes and numerous types and subtypes, based on the effector functions encoded by their respective *cas* gene sets [5, 6]. In contrast to the diversity of effectors, all systems rely on the Cas1–Cas2 complex to capture foreign DNA fragments (Fig. 1B). In *Escherichia coli*, these are trimmed into prespacers by host nucleases [7, 8], after which Cas1–Cas2 binds the CRISPR leader sequence and inserts the processed fragment into the first repeat of the CRISPR array [9–11]. The mechanism of repeat-duplication is identical to that proposed in 1979 for the integration of bacterial transposons (Fig. 1B, legend).

A longstanding puzzle is how CRISPR–Cas systems avoid self-destructive autoimmunity. The integration machinery does not inherently distinguish between host and foreign DNA, and early surveys found that self-derived spacers are not rare: ∼0.4% of 23 550 catalogued spacers were chromosomal, and up to 18% of hosts carried at least one [12]. Plasmid bias appears to arise from interference-based selection rather than prespacer choice: in a *Type II-A* system, 68% of new spacers came from a plasmid representing only ∼1% of total DNA, but this enrichment disappeared when Cas9 was catalytically inactivated, yielding near-unbiased sampling [13]. In contrast, in the *Type I-E* system of *E. coli* K12, which lacks active interference, plasmid-derived spacers are ∼200-fold overrepresented relative to their DNA abundance [9], suggesting physiological influences on prespacer selection. Consistent with this, increasing Cas1–Cas2 expression raised the genomic spacer fraction from 2% to 23%, while RecBCD and Chi sites were found to shield the chromosome from prespacer capture [14]. Knockout of RecB, RecC, or RecD reduced overall adaptation by two-fold but increased the genomic fraction seven-fold, showing that Chi sites are protective but not the sole mechanism.

Because the Cas1–Cas2 complex does not discriminate between self and non-self DNA, spacer acquisition carries a fitness cost. There is therefore balancing selection on the adaptation rate: increased protection comes at the expense of greater risk of autoimmunity. The system is probably closest to its optimum when spacer acquisition is rare, offering a genetic jackpot that allows survivors to repopulate after phage infection eliminates their clone mates. Understanding how physiological conditions shape spacer-source bias is therefore central to understanding the emergence of CRISPR–Cas immunity.

Here, we present a visual papillation assay that detects CRISPR–Cas adaptation at single-colony resolution on solid medium. It enables clonal tracking of individual events and offers a sensitive, genetically tractable platform to dissect how physiological states shape spacer-source bias.

## Materials and methods

### Reagents and enzymes

Chemicals were obtained from Sigma–Aldrich, BDH, Fisher Scientific, and Thermo Scientific. Enzymes were purchased from New England Biolabs and used according to manufacturers’ instructions.

### Plasmids and molecular biology

The complete DNA sequences of all plasmids used in this study are provided in Supplementary Table S1. Plasmids were created using Gibson assembly [15], and all junctions were confirmed by Sanger sequencing.

The *E. coli* MG1655 *cas1–cas2* gene-pair was cloned downstream of a ribosome binding site and in-frame with the first ATG in pBAD [16], under control of P*_araBAD_*. The ampicillin resistance gene was replaced with a kanamycin marker to yield pRC1656. To lower the expression level, the *cas1–cas2* gene-pair was cloned in place of *yfp* in pAJM.677 [17], placing it under control of P*_araMari_*, yielding plasmid pRC2747.

Polymerase chain reaction (PCR) was performed using Q5 DNA polymerase (New England Biolabs). Amplicons were resolved on 1.0% agarose gels (TBE buffer, 0.3 μg/ml ethidium bromide) and visualized under UV transillumination. Where required, bands were excised and purified using the QIAquick Gel Extraction Kit (Qiagen). CRISPR array expansion was detected by PCR across the leader–repeat junction of the *lacZ* reporter in strain RC5311 using primers: 5′-ggctagcaggaggaattcacc and 5′-cgcatcgtaaccgtgcatctg.

### Bacterial growth conditions

*Escherichia coli* strains were cultured in Luria–Bertani (LB) Lennox medium (10 g/l tryptone, 5 g/l yeast extract, 5 g/l NaCl) at 37°C in aerated liquid cultures or on solid media containing 1.5% agar. For papillation assays, LB agar plates were supplemented with 0.1% l-lactose and 40 μg/ml X-gal (5-bromo-4-chloro-3-indolyl-β-d-galactopyranoside). Each plate was seeded with 25–50 colonies. Antibiotics were used at the following concentrations: ampicillin (100 μg/ml), kanamycin (50 μg/ml), and chloramphenicol (30 μg/ml). When indicated, cultures were supplemented with l-arabinose at concentrations listed in figure legends. For long-term storage, overnight LB cultures were mixed 1:1 with sterile glycerol (final concentration 50%) and stored at –80°C. Strains were recovered by streaking onto LB agar. Chromosomal markers were selected with 12.5 μg/ml tetracycline or 5 μg/ml chloramphenicol, and selection was removed in subsequent experiments unless otherwise stated.

### Genome engineering and bacterial strains

P1 transduction and λ Red recombineering were performed as previously described [18, 19]. When required, antibiotic resistance cassettes flanked by *frt* sites were excised using FLP recombinase. Non-polar single-gene knockouts were obtained from the Keio Collection [20], and the kanamycin marker was deleted by FLP recombination to eliminate downstream expression from the resistance promoter.

To construct the papillation reporter strain RC5311, the following steps were performed:

- Step 1: In *E. coli* MG1655, the entire coding region of *lacZ* (except for the first 15 codons) was deleted by λ Red recombination using a PCR product containing a chloramphenicol resistance gene positioned upstream of the *lac* promoter. The resistance cassette was removed by FLP recombination between flanking *frt* sites, yielding a non-polar *ΔlacZ* deletion that retained *lacY* and *lacA* expression from the native promoter.
- Step 2: In strain ER1793, the *CRISPR-II* locus was replaced with a kanamycin resistance gene using λ Red recombination. This modified allele was then P1 transduced into the strain from Step 1, and the resistance marker was removed by FLP recombination.
- Step 3: The *araBAD::T7 RNAP tetA* locus from BL21-AI was introduced by P1 transduction, disrupting the native arabinose operon and allowing use of pBAD-derived plasmids. The resulting intermediate strain was designated RC5310 (JB023).
- Step 4: A CRISPR reporter plasmid (pRC1651) was constructed by placing the synthetic constitutive promoter J23100 (sequence obtained from Promoters Catalog Anderson [https://parts.igem.org/Promoters/Catalog/Anderson; accessed 17 April 2024]) upstream of a ribosome binding site and start codon, driving transcription and translation through a *CRISPR-II* (T) leader–repeat region into a promoterless *lacZ* gene. This cassette was inserted at the *argE* locus in ER1793 by λ Red recombination and then P1 transduced into RC5310. The kanamycin marker was excised to yield the final reporter strain RC5311 (JB028; F^−^, λ^−^, *rph-1 ΔlacZ ΔCRISPR-II araBAD::T7 RNAP tetA argE::[J23100 CRISPR-II (T) Reporter]*).
- Step 5: RC5311 was submitted for whole-genome sequencing (MicrobesNG, Birmingham, UK). The resulting contigs covered all modified regions and were merged into the MG1655 reference genome to generate a complete genome sequence for RC5311 (see Supplementary Table S1).

It should be noted that the *CRISPR-I* locus in the reporter strain RC5311 was left intact, as *cysH* regulatory elements appear to reside within *cas3* or another more distant location within the *CRISPR-I* locus (see the “Discussion” section). However, the *CRISPR-I* locus is transcriptionally silent under standard conditions, and there is no evidence of interference or primed adaptation ([21, 22] and references therein). However, the array will acquire spacers if Cas1–Cas2 is supplied exogenously.

Other strains used in this study were: *E. coli* MG1655 (F^−^, λ^−^, *ilvG rfb-50 rph-1*); BL21-AI (Thermo Scientific; F^−^, λ^−^, *ompT hsdSB (rB^−^, mB^−^) gal dcm araB::T7RNAP-tetA*); ER1793 (New England Biolabs; F^−^, λ^−^, *fhuA2 Δ[lacZ]r1 glnV44 e14^−^[McrA^−^] trp-31 his-1 rpsL104 xyl-7 mtl-2 metB1 Δ(mcrC-mrr)114::IS10*), which was used for high-efficiency λ Red recombineering; and RC5045, derived from ER2566 (New England Biolabs; F^−^, λ^−^, *fhuA2 [lon] ompT lacZ::T7 gene1 gal sulA11 Δ(mcrC-mrr)114::IS10R(mcr-73::miniTn10)2 R(zgb-210::Tn10)1 endA1 [dcm]*), which served as the host for Tn5 transposase expression.

### Oxford nanopore sequencing of acquired spacers

**Figure 2.**
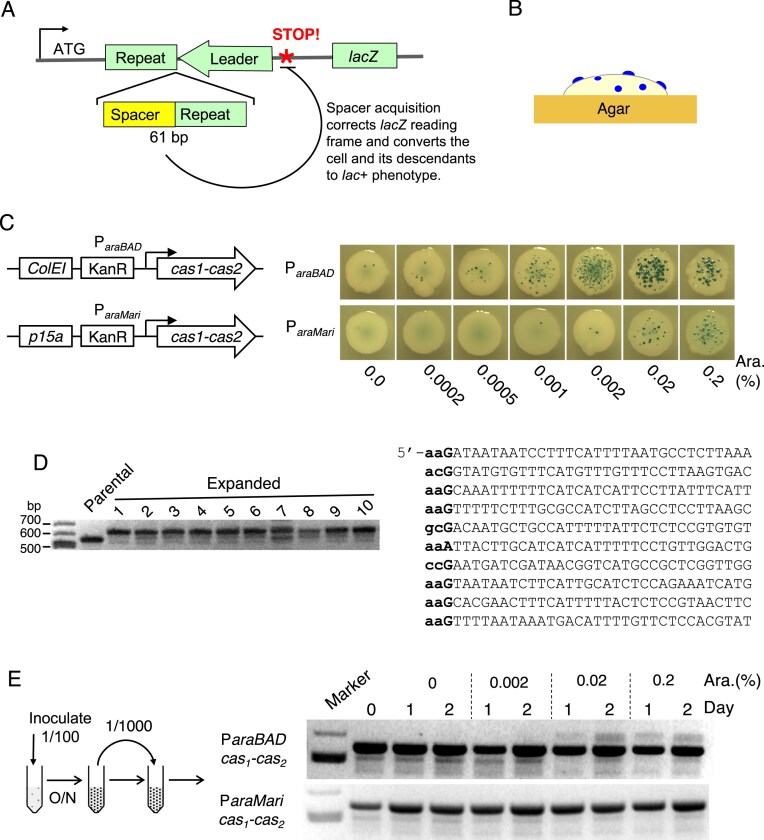
A Papillation Assay for Spacer Acquisition. (**A**) A constitutive promoter drives transcription through the CRISPR leader–repeat region into *lacZ*, which is initially inactive due to an in-frame stop codon. Spacer acquisition restores the reading frame, activating LacZ expression. (**B**) The assay is performed on LB agar containing 0.1% lactose and X-gal. Only colonies acquiring a spacer develop blue Lac^+^ papillae through lactose metabolism and X-gal cleavage. (**C**) Two Cas1–Cas2 plasmids, pRC1656 (P*_araBAD_*) and pRC2747 (P*_araMari_*), were introduced into RC5311. Arabinose concentration was varied to modulate expression. Papillation peaked at 0.002% l-arabinose for P*_araBAD_* and at 0.2% for P*_araMari_*. Representative colonies are shown. (**D**) Thirty Lac^+^ papillae were isolated by re-streaking for single colonies. CRISPR arrays were PCR-amplified and analyzed by agarose gel electrophoresis. All isolates displayed +1 repeat expansions. Ten representative examples are shown. Spacer sequences were confirmed by Sanger sequencing; uppercase letters denote spacer sequence; the PAM is in bold with the first two residues in lowercase to indicate that they are of genomic origin and not present in the CRISPR array. (**E**) Cells were passaged twice at 1:1000 dilution, and CRISPR array expansion was assessed by PCR. Bands were visible only at high P*_araBAD_* induction, demonstrating the greater sensitivity of the papillation assay compared to bulk PCR.

Strain RC5311 was transformed with plasmid pRC1656 to express Cas1–Cas2. For the liquid-phase assay, cells were passaged twice overnight (see Fig. 2E). The solid-phase assay was performed on standard papillation plates supplemented with 0.002% or 0.2% l-arabinose. After 5 days of incubation, colonies from 25 plates were pooled for analysis. The CRISPR locus was PCR-amplified using primers 5′-GGTCTTAATGAATGGCCGGG and 5′-CATGGATCCGAAGTCGAGC. PCR products were gel purified using Qiagen QIAquick PCR Purification Kit.

Oxford Nanopore Technologies (ONT) amplicon sequencing was performed by the University of Nottingham Deep Seq facility. Amplicon pool concentrations were measured using a Qubit 4 Fluorometer (Thermo Fisher Scientific) with a Qubit 1× dsDNA HS Assay Kit (Thermo Fisher Scientific; Q33231). Barcoded sequencing libraries were prepared for each amplicon pool using the Ligation Sequencing Amplicons – Native Barcoding Kit 96 V14 protocol (Oxford Nanopore Technologies; Version NBA_9170_v114_revL_15Sep2022) with a Native Barcoding Kit 96 V14 (Oxford Nanopore Technologies; SQK-NBD114.96). Barcoded libraries were pooled in equal amounts before the final sequencing-adapter ligation step. All purification steps were performed using AMPure XP beads (Beckman Coulter; A63882). The pooled library was loaded onto a PromethION R10.4.1M flow cell (Oxford Nanopore Technologies; FLO-PRO114M) and sequenced on a PromethION 24 platform (Oxford Nanopore Technologies). Default sequencing parameters were applied, except that barcode recognition was required at both ends of the read and raw data output was set to Fast5 format.

Spacer sequences were extracted from raw ONT reads and mapped to the genome and plasmid using Python 3.13.2 with the packages Biopython 1.85, Matplotlib 3.10.1, NumPy 2.2.4, and Pandas 2.2.3. Custom scripts are provided in Supplementary Table S2. The custom Python scripts used in this study were generated with the assistance of ChatGPT (OpenAI). The authors undertook extensive troubleshooting, testing, and refinement to ensure their correct functionality and integration into the analysis. Exact duplicate spacers were removed to generate chromosomal and plasmid maps, and sequence logos were created with Logomaker 0.8.7. All spacers were used to calculate chromosome-to-plasmid spacer ratios. For standard papillation plates with 0.002% l-arabinose, the ratio was 9.1% ± 0.5 (*n* = 2). Ratios for solid and liquid cultures with 0.2% l-arabinose were each *n* = 1, as these were controls for the standard papillation plates (0.002% l-arabinose) and for previous *E. coli* BL21 data, respectively. The spacer ratios for the papillation assays at 0.002% and 0.2% l-arabinose are very similar and if all three measurements are combined the average ratio is 8.7% ± 0.7 (*n* = 3).

### Transposon mutagenesis

A hyperactive triple-mutant Tn5 transposase was expressed from plasmid pRC2128 in strain RC5045 and purified as described [23]. Transposon DNA was excised from plasmid pRC1671 using PvuII, resolved on agarose gels, and purified by gel extraction. Transpososomes were assembled in a 20 μl reaction containing 50 ng/μl transposon DNA, 10 ng/μl Tn5 transposase, 25 mM Tris–acetate (pH 7.5), and 100 mM potassium glutamate. Reactions were incubated at 37°C for 30 min. One microliter of the transpososome mixture was electroporated into the target strain. After 1 h recovery in LB at 37°C, cells were plated on LB agar containing chloramphenicol and incubated overnight. Resistant colonies (∼15 000) were scraped from plates, pooled in LB, and stored at –80°C in 50% glycerol.

Transposon insertion sites in mutant strains were identified by plasmid rescue. Genomic DNA was purified, digested with ZraI and EcoRV, and the resulting blunt-ended fragments ligated. Because intramolecular ligation of blunt ends is much more efficient than intermolecular ligation, fragments carrying transposon insertions circularize to form plasmids. These plasmids were recovered by transformation into a host strain expressing the Pir protein. Since ZraI and EcoRV each recognize a six-base pair site, they are expected to cut approximately once every 3 kb, producing fragments small enough to be rescued as plasmids. Primer sites within the transposon were then used to sequence across the transposon–chromosome junctions.

## Results

### A visual papillation assay enables single-colony detection of CRISPR–Cas adaptation

We developed a clonal, semi-quantitative assay to measure CRISPR–Cas naive adaptation in individual colonies. In this system, a constitutive promoter and ribosome binding site drive transcription and translation through the CRISPR leader–repeat region into a chromosomal *lacZ* gene (Fig. 2A and Supplementary Fig. S1). However, an in-frame stop codon prevents LacZ expression. Acquisition of a single 61 bp spacer–repeat unit restores the reading frame, converting Lac^−^ cells to Lac^+^. To perform the assay, the Lac^−^ reporter strain is transformed with a Cas1–Cas2 expression plasmid and plated on LB agar supplemented with lactose and X-gal. Colonies appear after overnight incubation at 37°C, but growth slows significantly as carbon and energy sources become limiting. Cells in the colony that acquired a spacer during the first day of growth on LB are converted to a Lac^+^ phenotype. These cells continue to grow, using lactose as the primary carbon and energy source, and form papillae that stain blue due to X-gal cleavage (Fig. 2B).

To test the sensitivity of the assay to Cas1–Cas2 expression, we used two expression plasmids (Fig. 2C). The highest expression range was provided by a kanamycin-resistant derivative of pBAD, which encodes the *E. coli* arabinose operon promoter (P*_araBAD_*) [16]. The lower-expression system, P*_araMari_*, was engineered for reduced cell-to-cell variability and a broad, linear response to inducer concentration [17]. Papillation increased with rising Cas1–Cas2 levels, peaking with P*_araBAD_* at 0.002% arabinose. P*_araBAD_* therefore offers a sensitive setup to study genes or conditions that reduce the adaptation rate, whereas P*_araMari_* is better suited to explore conditions that increase the adaptation rate.

To confirm that blue papillae reflect genuine adaptation events, we isolated 30 papillae from across the inducer range, re-streaked them on X-gal plates, and analyzed the reporter loci by colony PCR. All products were consistent with the acquisition of a single 61 bp spacer-repeat unit, confirmed by DNA sequencing. Representative examples for 10 isolates are shown in Fig. 2D. Protospacer sites were identified using BLAST, and the associated protospacer adjacent motifs (PAMs) revealed that seven out of ten matched the canonical 5′-AAG PAM sequence, consistent with previous findings [22, 24]. These results demonstrate that the number of papillae on a colony serves as a visual readout of the spacer acquisition rate.

To benchmark the sensitivity of the assay, we performed a population-level PCR assay across the range of inducer concentrations (Fig. 2E). CRISPR array expansions were detectable only in P*_araBAD_* cultures at high inducer levels, confirming that the visual papillation assay is substantially more sensitive than bulk PCR. A single papillus corresponds to a detection threshold of approximately one adaptation event per 10^9^ cells. However, not all adaptation events restore *lacZ* function. Because spacer sequences may introduce stop codons, the theoretical detection limit of the assay is ∼62% (Supplementary Table S3).

### Spacer distribution reflects chromosomal hotspots and plasmid bias

**Figure 3.**
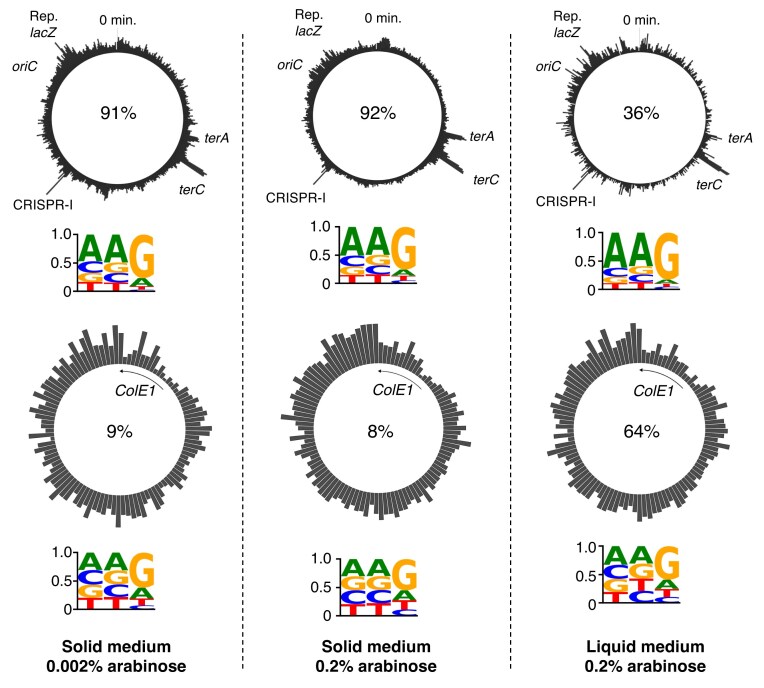
Spacer Acquisition Hotspots in Liquid and Solid Media. The reporter strain RC5311 carrying pRC1656 (P*_araBAD_*) was used for both assays. Colonies were pooled after 5 days (solid phase) or after two passages (liquid). CRISPR arrays were PCR-amplified and sequenced using Oxford Nanopore. Unique spacers were mapped to the genome (10 kb bins) and plasmid (50 bp bins). “0 min” marks the origin of transfer in *E. coli* HfrH; “Rep. *lacZ*”marks the reporter. Details of mapping procedures are given in the “Materials and Methods” section.

High-throughput spacer acquisition profiles previously revealed protospacer hotspots at *oriC*, the terminus, and the CRISPR locus in *E. coli* BL21 [14]. For comparison, we generated spacer distribution maps from both liquid cultures and papillation plates (Fig. 3). In liquid culture, the reporter strain was passaged twice in LB with 0.2% arabinose. For the solid-phase assay, papillation plates contained 0.1% lactose and either 0.002% or 0.2% arabinose. After 5 days of incubation, colonies were pooled, CRISPR arrays were amplified, and spacer sequences were extracted and mapped by BLAST (Fig. 3). Across conditions, chromosomal distributions were similar and aligned with known *E. coli* BL21 hotspot patterns, including strong signals at the terminus, the CRISPR locus, and the *lacZ* reporter. On papillation plates, the origin hotspot was less pronounced, replaced by a broader enrichment that extended into the adjacent reporter locus, particularly at higher Cas1–Cas2 expression levels.

Spacer origin analysis revealed condition-specific biases (Fig. 3). In liquid culture, 64% of spacers were plasmid-derived, consistent with the 77% observed in *E. coli* BL21 [14]. In contrast, only 9% of spacers from solid-phase cultures were plasmid-derived, indicating a major shift toward chromosomal targets under papillation conditions.

To examine PAM preferences, we extracted sequences flanking each spacer (–2 to +1) and visualized them as sequence logos (Fig. 3). All conditions favored the canonical 5′-AAG PAM. The consensus was strongest for chromosomal spacers from liquid culture and weaker for plasmid-derived sequences, likely due to saturation of the limited number of AAG sites on the plasmid.

### Functional enrichment of spacers via start codon capture

Close inspection of papillae reveals a range of sizes and X-gal staining intensities. Early-arising papillae likely have better access to nutrients. Some spacers may change *lacZ* expression by altering mRNA stability or the stability of the LacZ fusion protein. Another possibility is that certain spacers capture translational start signals, shortening the N-terminal fusion with LacZ, potentially increasing β-galactosidase activity by stabilizing the mRNA or the protein.

**Figure 4.**
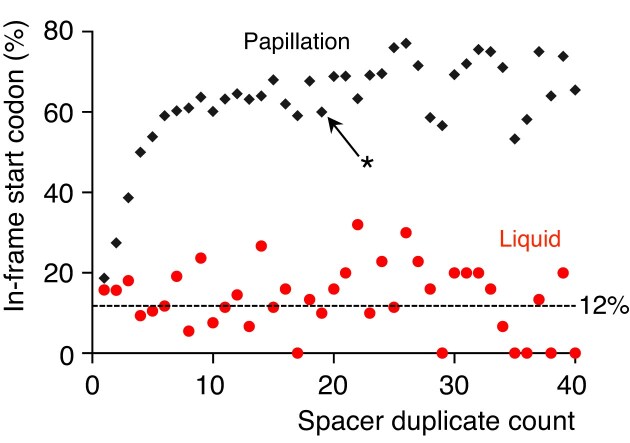
Spacer Duplication Correlates with Start Codon Enrichment. Spacers were grouped by duplication frequency, and the proportion in each bin containing an in-frame ATG was plotted. For example, the asterisk marks the 19-duplication bin, where 60% of spacers contained in-frame ATGs. Only 30 bp of each spacer could contribute an in-frame ATG due to overlap with the repeat. Assuming random composition, the probability of a 30 bp sequence containing an in-frame ATG lacking a downstream in-frame stop codon is 12% (dashed line and Supplementary Table S3).

To test for start codon enrichment, we grouped spacers by duplication frequency and calculated the proportion in each group containing an in-frame ATG codon (Fig. 4). The baseline probability for a 30 bp spacer to contain an in-frame ATG not followed by a stop codon is ∼12% (Supplementary Table S3). In liquid culture, where there is no lactose to enrich for in-frame start codons, spacers clustered around this baseline. However, papillation-enriched spacers showed a strong correlation between duplication frequency and ATG presence. This supports selective enrichment for functionally expressed LacZ fusions in the papillation assay.

### Proof-of-concept mutant detection

**Figure 5.**
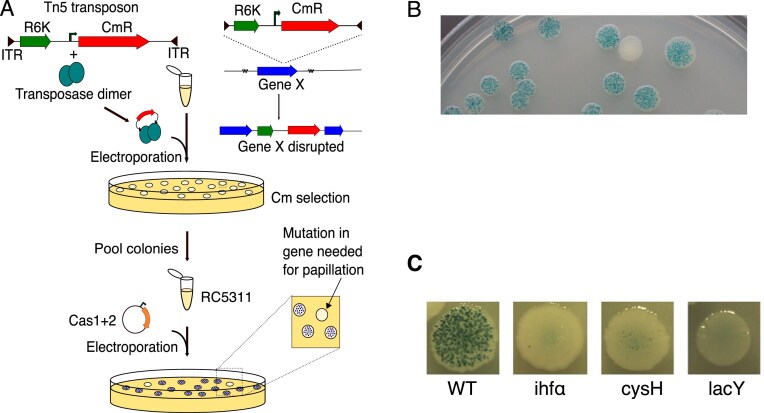
Transposon Mutagenesis Screen for Adaptation Regulators. (**A**) Workflow. Tn5 transpososomes carrying a chloramphenicol resistance marker and R6K origin were electroporated into RC5311. After selection, ∼15 000 colonies were pooled. The P*_araBAD_* Cas1–Cas2 plasmid (pRC1656) was introduced, and adaptation was assessed using the papillation assay (0.002% l-arabinose). (**B**) Representative screening plate. Hypo-papillating mutants were identified visually. Insertion sites were mapped by plasmid rescue using the R6K origin and the chloramphenicol resistance marker. (**C**) Selected hypo-papillating mutants are shown.

The clonal resolution of our assay allows straightforward identification of mutations that alter the rate of papillation. We performed a transposon mutagenesis screen using a Tn5 transposon carrying an R6K origin and chloramphenicol resistance marker (Fig. 5A). Approximately 15 000 chloramphenicol-resistant colonies were pooled to create a mutant library. After transformation with a Cas1–Cas2 expression plasmid, colonies with few or no papillae were identified by visual screening (Fig. 5B).

Insertion sites from hypo-papillating colonies were rescued as plasmids using the R6K origin and the chloramphenicol resistance gene. Three mutants, identified by sequencing from primers internal to the transposon, are shown in Fig. 5C. Integration host factor (IHF) is a known requirement for CRISPR–Cas adaptation in E. coli [25]. *lacY*, which encodes the lactose transporter, is required for papillae growth, while *cysH* was previously identified as having high CRISPRicity [26], a metric that quantifies genomic association with CRISPR loci (see the “Discussion” section).

## Discussion

### The papillation assay provides a sensitive and physiologically relevant readout

We have developed a semi-quantitative, clonal assay to measure CRISPR–Cas adaptation in *E. coli*, based on the classical papillation technique. This method exploits the conversion of cells from Lac^-^ to Lac^+^: cells that acquire a functional spacer–repeat unit during colony growth activate the reporter and form blue-stained papillae on X-gal plates (Fig. 2). Although papillae were first described in 1907, the lactose-based system emerged in the 1940s and was refined during studies of adaptive mutation in the 1980s ([27] and references therein). A major strength of this assay is its ability to detect and characterize mutations, in either Cas1–Cas2 or the host genome, that alter CRISPR–Cas adaptation rates.

The assay is highly sensitive and, in principle, can detect a single adaptation event in a colony of 10^9^ cells. Spacer acquisition peaks when Cas1–Cas2 expression is induced with 0.002% arabinose (Fig. 2C), even though the resulting adaptation is undetectable by population-level PCR (Fig. 2E). This underscores a broader issue in adaptation studies: overexpression of Cas1–Cas2 from strong promoters may distort physiological relevance. Our results emphasize the value of low-expression, near-physiological conditions for studying CRISPR–Cas adaptation.

### Physiological structure drives spacer-source bias

Liquid cultures are the default setting for many CRISPR–Cas studies but differ markedly from conditions *E. coli* encounters in natural environments. Shaking cultures are hyperoxic, spatially uniform, and lack the nutrient gradients present in colonies or biofilms. In contrast, growth on agar plates creates a structured environment with microaerobic zones, spatially restricted lineages, and metabolic heterogeneity. This structure likely contributes to the loss of plasmid bias we observe by slowing growth and increasing the availability of chromosomal prespacer fragments generated at stalled replication forks. These findings suggest that the influence of how spatial structure and growth state shape the natural dynamics of spacer acquisition may have been underestimated. Our plate-based papillation assay offers a tractable platform for dissecting CRISPR adaptation in a physiologically relevant context that better reflects, in some respects, natural microbial habitats. Supporting this view, CRISPR–Cas adaptation during phage challenge on semi-solid top agar has been shown to diverge markedly from liquid cultures, underscoring the impact of spatial structure on CRISPR dynamics [28].

### Papillation assay reproduces chromosomal hotspots but shifts spacer source bias

The most striking difference between liquid and solid-phase conditions was the shift in spacer source bias (Fig. 3). In liquid culture, 64% of spacers originated from the plasmid. On standard papillation plates containing 0.002% arabinose, this proportion dropped to just 9% (Fig. 3). This shift is significant because one of the central questions in CRISPR–Cas biology is how cells balance spacer acquisition from mobile genetic elements versus the chromosome, which is often lethal in the presence of a functional interference system [29–31].

To determine whether this change reflects altered Cas1–Cas2 activity, we repeated the papillation assay using 0.2% arabinose, the same inducer concentration used in liquid media (Fig. 3). The plasmid bias was not restored, indicating that the shift arises from physiological differences on solid media rather than from reduced Cas1–Cas2 expression. This interpretation is reinforced by earlier work in *E. coli* BL21, where increasing Cas1–Cas2 levels decreased plasmid-derived spacers from 98% to 77% [14]. If expression level were driving the change in our assay, we would expect a bias shift in the same direction, but we observe the opposite. Together, these findings point to the microenvironmental context of colony growth as the most likely cause.

Growth on a surface is, in many ways, a more natural condition for *E. coli*, a facultative aerobe that often exists in structured communities. In planktonic cultures, rapid mixing and abundant nutrients can trigger a phage bloom that sweeps through the population, creating strong selection for spacer acquisition from mobile genetic elements. On surfaces and within biofilms, restricted movement and the extracellular matrix impede phage dispersal, limiting epidemic spread [32–34]. With the threat reduced, the selective advantage of targeting foreign DNA diminishes. This rationale fits the shift we observed, from a strong plasmid bias in liquid culture to near-neutral sampling in the plate-based assay: although only 9% of spacers were plasmid-derived, the chromosomal DNA is present in a ∼22-fold molar excess (assuming a plasmid copy number of ∼40) (Data were obtained from BioNumbers [https://bionumbers.hms.harvard.edu/bionumber.aspx?id=107528&ver=2&trm=pet+copy+number&org=; accessed 12 August 2025]). The apparent plasmid underrepresentation therefore reflects near-neutral sampling of available DNA sources under these conditions. These results underscore the context-dependent nature of CRISPR–Cas adaptation and show how spatial structure can reshape the balance of spacer acquisition.

In addition to source bias, we examined PAM preferences under each condition. PAM logos revealed a dominant 5′-AAG consensus across all conditions (Fig. 3). The consensus was stronger in chromosomal spacers from liquid culture, while plasmid PAMs were more degenerate, likely due to saturation on the small plasmid, which has only 152 perfect motifs.

### Start codon enrichment indicates functional selection

We observed that the papillation assay frequently captured spacers containing in-frame ATG start codons upstream of the *lacZ* reporter gene (Fig. 4). This enrichment likely reflects enhanced LacZ translation, which allows faster growth on lactose. The effect was specific to the plate-based assay and not observed in liquid culture, where detection does not rely on LacZ activity.

Assuming unbiased base composition, the probability that a 30 bp spacer contains at least one in-frame ATG not followed by a stop codon is ∼12% (Supplementary Table S3). The actual frequency may be lower, as translation also depends on the presence of a ribosome binding site, although any A+G-rich sequence may suffice given the weak consensus in *E. coli*. This observation suggests that the assay selectively enriches for spacers that both restore the *lacZ* reading frame and enhance its translation. Thus, it should be broadly applicable to CRISPR–Cas systems whose repeat–spacer units are multiples of three bases, preserving the reporter’s reading frame. A conceptually similar fluorescent reporter was recently described in *Streptococcus pyogenes* [35], supporting the generality of this principle.

### Strengths and limitations of mutant identification with the papillation assay

We used Tn5 to generate a library of transposon insertion mutants in the reporter strain and screened for colonies with reduced papillation (Fig. 5). While a more extensive analysis and a parallel screen of the Keio Collection [20] will be reported elsewhere, this proof-of-concept highlights both what the assay can detect and where false negatives arise.

Here, we highlight three mutants, *ihfA*, *lacY*, and *cysH*, to illustrate both the utility and limitations of the papillation assay. IHF is essential for CRISPR–Cas adaptation in *E. coli* because it binds the CRISPR leader and directs site-specific spacer integration [25]. A transposon insertion in *ihfA* caused a strong defect, producing ≤2 papillae per colony. The *lacY* insertion blocked lactose import, preventing papilla growth.

The *cysH* mutant initially appeared promising because *cysH* has high CRISPRicity, a measure of genomic association with CRISPR loci [26]. However, the phenotype was fully rescued by supplementing papillation plates with 0.2 mM cystine [36], revealing that while carbon and energy are the primary growth-limiting factors in LB agar, cysteine availability can also limit papilla formation.

These cases underscore a key limitation: mutations affecting post-adaptation growth can mimic adaptation defects. Candidate genes from papillation screens should therefore be validated on M9 minimal glucose medium to distinguish genuine adaptation factors from growth-related artifacts.

### Implications and future directions

We have developed a sensitive, visual, and genetically tractable assay to measure naive CRISPR–Cas adaptation in *E. coli*. This system enables clonal tracking of individual adaptation events and supports high-throughput genetic screens. By linking adaptation to a live phenotypic output, it captures both direct and indirect modulators. The assay provides a platform to dissect adaptation dynamics, probe stress–response crosstalk, and map host factors that shape immune memory formation, including how plasmid replication modes influence spacer-source bias under different growth conditions. In parallel, our findings highlight the extent to which spacer-source bias itself is shaped by physiological context.

The system also offers a tractable route to investigate adaptation in less well-characterized CRISPR–Cas types. For example, *Type II-A* systems from *Streptococcus thermophilus* require Cas1, Cas2, Csn2, and Cas9 for spacer acquisition [13, 37], yet may perform poorly or fail to adapt when transplanted into *E. coli* (Gediminas Drabavicius, personal communication). This may reflect the absence of a required host factor or a physiological incompatibility in the heterologous setting. Because our assay sensitively detects single adaptation events in a genetically accessible host, it provides a means to systematically test such requirements. Combining heterologous Cas protein expression with host factor libraries could reveal whether these barriers are mechanistic or environmental. Since *E. coli* K12 lacks interference, it allows the underlying rules of spacer acquisition to be observed without the confounding effects of selection.

More broadly, our results demonstrate that plasmid-to-chromosome spacer-source bias is not a fixed property but an emergent feature of cellular physiology. This raises the question of what specific factors drive such shifts. Given the rarity and asynchrony of adaptation events, bulk-averaged approaches like transcriptomics are unlikely to detect relevant signatures, as signals from adapting cells are masked by the larger non-adapting population. Targeted analysis of candidate pathways, such as DNA repair, replication fork dynamics, and nucleoid-associated proteins, will therefore be necessary. Each may influence the availability or stability of prespacer substrates, thereby altering the balance between chromosomal and plasmid-derived acquisition. Dissecting these contributions through genetic perturbation and spacer mapping will be an important step toward understanding the physiological basis of spacer-source bias.

## Supplementary Material

gkaf1044_Supplemental_File

## Data Availability

Sequencing data associated with this study are available on NCBI SRA (PRJNA1305692). All code to replicate the analysis can be found in Supplemental Table 2.
