# Supplementary material for: Single-colony resolution of CRISPR–Cas adaptation in E. coli reveals altered spacer-source bias during solid-phase growth
Source: Nucleic Acids Res. 2025 Oct 21;53(19):gkaf1044. doi: 10.1093/nar/gkaf1044 (PMC12539621; doi:10.1093/nar/gkaf1044)
Supplement: gkaf1044_Supplemental_File [file gkaf1044_supplemental_file.pdf]

A

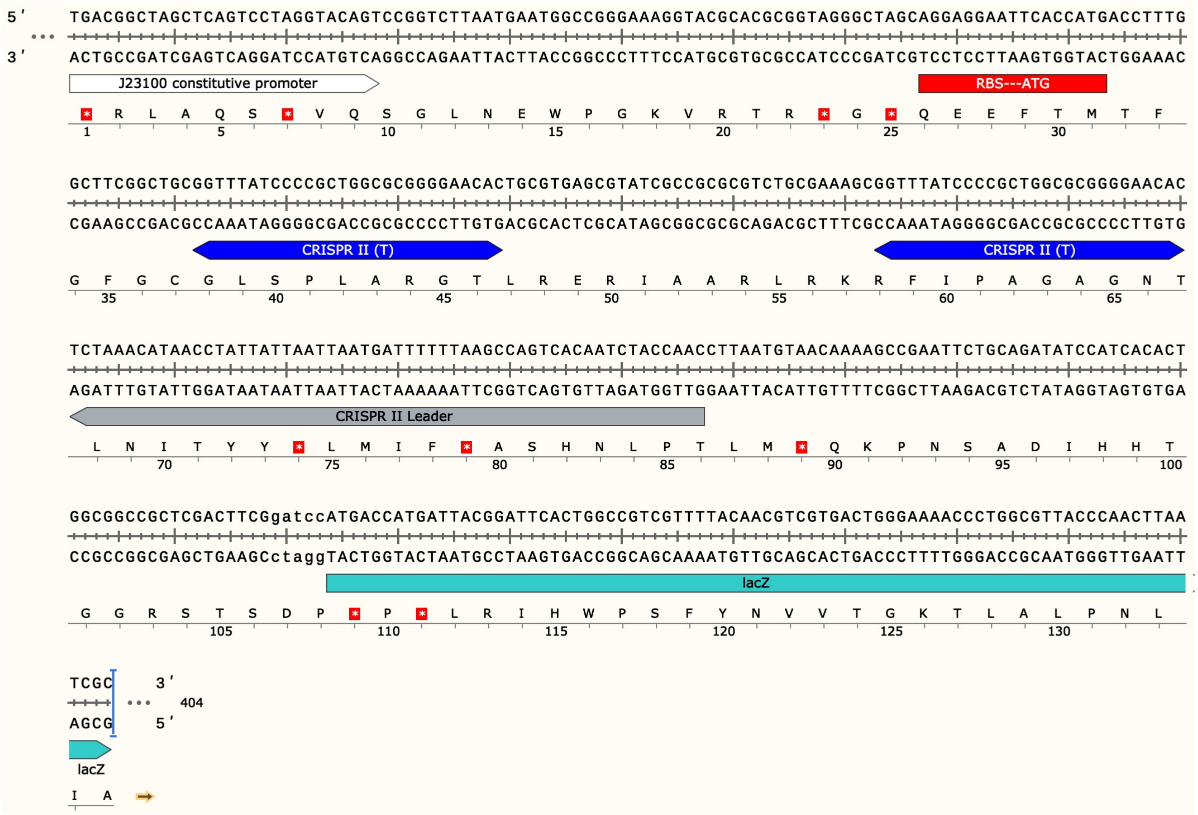
5'
TGACGGCTAGCTCAGTCCTAGGTACAGTCCGGTCTTAATGAATGGCCGGGAAAGGTACGCACGCGGTAGGGCTAGCAGGAGGAATTCACCATGACCTTTG
3'
J23100 constitutive promoter
RBS---ATG
GCTTCGGCTGCGGTTTATCCCCGCTGGCGCGGGGAACACTGCGTGAGCGTATCGCCGCGCGTCTGCGAAAGCGGTTTATCCCCGCTGGCGCGGGGAACAC
CRISPR II (T)
CRISPR II (T)
TCTAAACATAACCTATTATTAATTAATGATTTTTTAAGCCAGTCACAATCTACCAACCTTAATGTAACAAAAGCCGAATTCTGCAGATATCCATCACACT
CRISPR II Leader
GGCGGCCGCTCGACTTCGgatccATGACCATGATTACGGATTCACTGGCCGTCGTTTTACAACGTCGTGACTGGGAAAACCCTGGCGTTACCCAACTTAA
lacZ
TCGC
3'
404
5'
lacZ

B

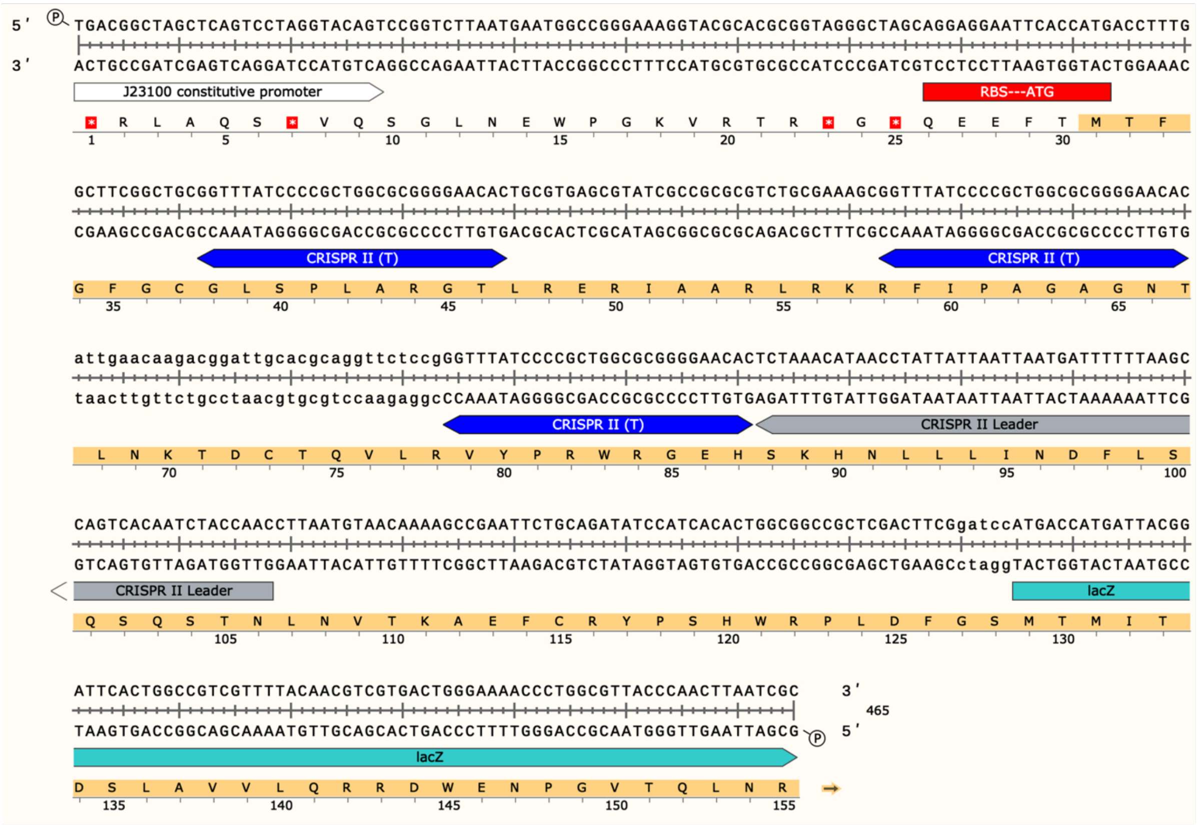

Supplemental Figure 1. Papillation reporter before and after spacer acquisition
The sequence of the papillation reporter is shown before and after acquisition of a 61 bp repeat–spacer segment. Transcription is driven by the constitutive J23100 promoter, and translation is initiated from a strong ribosome binding site (RBS) followed by an ATG start codon.
(A) Reporter sequence before spacer acquisition. The *lacZ* gene is out of frame with the ATG start codon, and three stop codons occur before the *lacZ* coding sequence.
(B) Reporter sequence after acquisition of a repeat–spacer segment. The *lacZ* gene is now in frame with the ATG start codon, and no intervening stop codons remain.

Supplemental Table 1, Plasmid and Genome sequences

pRC1656

AAGAAACCAATTGTCCATATTGCATCAGACATTGCCGTCACTGCGTCTTTTACTGGCTCTTCTCGC
TAACCAAACCGGTAACCCCGCTTATTAAAAGCATTCTGTAACAAAGCGGGACCAAAGCCATGACA
AAAACGCGTAACAAAAGTGTCTATAATCACGGCAGAAAAGTCCACATTGATTATTTGCACGGCGT
CACACTTTGCTATGCCATAGCATTTTTATCCATAAGATTAGCGGATCCTACCTGACGCTTTTTATC
GCAACTCTCTACTGTTTCTCCATACCCGTTTTTTGGGCTAACAGGAGGAATTAACCATGGCCTGG
CTTCCCCTTAATCCCATTCCACTCAAAGATCGCGTCTCCATGATCTTTCTGCAATATGGGCAGATC
GATGTAATAGATGGCGCGTTTGTACTTATCGACAAGACAGGGATCCGCACTCATATTCCTGTTGG
CTCGGTTGCCTGCATCATGCTGGAACCTGGTACACGGGTTTCGCATGCAGCTGTACGCCTGGCT
GCGCAAGTTGGAACATTGTTGGTATGGGTGGGGGAAGCGGGCGTTCGTGTTTATGCTTCTGGTC
AGCCTGGAGGTGCGCGTTCAGATAAGCTGCTCTATCAGGCAAAACTTGCTCTGGATGAAGATTT
GCGTCTGAAGGTCGTACGTAAAATGTTTGAACTTCGGTTTGGAGAACCTGCGCCTGCCCGGCGC
TCCGTAGAGCAACTCAGAGGTATAGAAGGCAGTCGCGTGCGGGCAACCTACGCACTTCTGGCG
AAGCAATACGGCGTGACATGGAATGGACGTCGCTACGATCCGAAAGACTGGGAAAAGGGCGAT
ACGATCAACCAATGCATTAGCGCTGCAACTTCCTGTTTATACGGCGTAACTGAAGCGGCGATACT
TGCAGCTGGTTATGCACCAGCTATTGGGTTTGTGCATACAGGAAAGCCTCTTTCCTTTGTTTACG
ATATTGCAGACATCATTAAATTTGACACTGTTGTACCGAAAGCTTTTGAGATAGCGCGTCGTAACC
CTGGTGAGCCGGACCGGGAAGTCCGTTTGGCGTGCAGGGATATTTTTCGCAGTAGTAAAACATT
AGCCAAATTGATTCCGCTTATAGAGGACGTGCTTGCCGCTGGAGAAATACAACCGCCGGCCCCA
CCTGAAGATGCACAGCCTGTTGCCATTCCGCTTCCTGTTTCACTGGGAGATGCAGGCCATCGGA
GTAGCTGAAATGAGTATGTTGGTCGTGGTCACTGAAAATGTACCTCCGCGCTTACGAGGCAGATT
AGCCATCTGGTTGTTGGAGGTACGTGCAGGGGTATATGTAGGTGATGTATCCGCAAAAATTCGT
GAAATGATCTGGGAACAAATAGCTGGACTGGCGGAAGAAGGCAATGTAGTGATGGCATGGGCAA
CGAATACGGAAACGGGATTTGAGTTCCAGACATTTGGGTTAAACAGGCGTACCCCGGTAGATTT
GGATGGTTTAAGGTTGGTGTCTTTTTTACCTGTTTGAGCGGCCGCCCATGGGGGGTTCTCATCAT
CATCATCATCATGGTATGGCTAGCATGACTGGTGGACAGCAAATGGGTCGGGATCTGTACGACG
ATGACGATAAGGATCGATGGGGATCCGAGCTCGAGATCTGCAGCTGGTACCATATGGGAATTCG
AAGCTTGGCTGTTTTGGCGGATGAGAGAAGATTTTCAGCCTGATACAGATTAAATCAGAACGCAG
AAGCGGTCTGATAAAACAGAATTTGCCTGGCGGCAGTAGCGCGGTGGTCCCACCTGACCCCATG
CCGAACTCAGAAGTGAAACGCCGTAGCGCCGATGGTAGTGTGGGGTCTCCCCATGCGAGAGTA
GGGAACTGCCAGGCATCAAATAAAACGAAAGGCTCAGTCGAAAGACTGGGCCTTTCGTTTTATCT
GTTGTTTGTCGGTGAACGCTCTCCTGAGTAGGACAAATCCGCCGGGAGCGGATTTGAACGTTGC
GAAGCAACGGCCCGGAGGGTGGCGGGCAGGACGCCCGCCATAAACTGCCAGGCATCAAATTAA
GCAGAAGGCCATCCTGACGGATGGCCTTTTTGCGTTTCTACAAACTCTTGTCTCAAAATCTCTGA
TGTTACATTGCACAAGATAAAAATATATCATCATGAACAATAAAACTGTCTGCTTACATAAACAGTA
ATACAAGGGGTGTTATGAGCCATATTCAACGGGAAACGTCTTGCTCGAGGCCGCGATTAAATTCC
AACATGGATGCTGATTTATATGGGTATAAATGGGCTCGCGATAATGTCGGGCAATCAGGTGCGA
CAATCTATCGATTGTATGGGAAGCCCGATGCGCCAGAGTTGTTTCTGAAACATGGCAAAGGTAG
CGTTGCCAATGATGTTACAGATGAGATGGTCAGACTAAACTGGCTGACGGAATTTATGCCTCTTC
CGACCATCAAGCATTTTATCCGTACTCCTGATGATGCATGGTTACTCACCACTGCGATCCCCGGG
AAAACAGCATTCCAGGTATTAGAAGAATATCCTGATTCAGGTGAAAATATTGTTGATGCGCTGGC
AGTGTTCCTGCGCCGGTTGCATTCGATTCCTGTTTGTAATTGTCCTTTTAACAGCGATCGCGTATT
TCGTCTCGCTCAGGCGCAATCACGAATGAATAACGGTTTGGTTGATGCGAGTGATTTTGATGACG
AGCGTAATGGCTGGCCTGTTGAACAAGTCTGGAAAGAAATGCATAAGCTTTTGCCATTCTCACCG
GATTCAGTCGTCACTCATGGTGATTTCTCACTTGATAACCTTATTTTTGACGAGGGGAAATTAATA
GGTTGTATTGATGTTGGACGAGTCGGAATCGCAGACCGATACCAGGATCTTGCCATCCTATGGA

ACTGCCTCGGTGAGTTTTCTCCTTCATTACAGAAACGGCTTTTTCAAAAATATGGTATTGATAATC
CTGATATGAATAAATTGCAGTTTCATTTGATGCTCGATGAGTTTTTCTAACTGTCAGACCAAGTTTA
CTCATATATACTTTAGATTGATTTAAAACTTCATTTTTAATTTAAAAGGATCTAGGTGAAGATCCTTT
TTGATAATCTCATGACCAAAATCCCTTAACGTGAGTTTTCGTTCCACTGAGCGTCAGACCCCGTA
GAAAAGATCAAAGGATCTTCTTGAGATCCTTTTTTTCTGCGCGTAATCTGCTGCTTGCAAACAAAA
AAACCACCGCTACCAGCGGTGGTTTGTTTGCCGGATCAAGAGCTACCAACTCTTTTTCCGAAGGT
AACTGGCTTCAGCAGAGCGCAGATACCAAATACTGTCCTTCTAGTGTAGCCGTAGTTAGGCCAC
CACTTCAAGAACTCTGTAGCACCGCCTACATACCTCGCTCTGCTAATCCTGTTACCAGTGGCTGC
TGCCAGTGGCGATAAGTCGTGTCTTACCGGGTTGGACTCAAGACGATAGTTACCGGATAAGGCG
CAGCGGTCGGGCTGAACGGGGGGTTCGTGCACACAGCCCAGCTTGGAGCGAACGACCTACACC
GAACTGAGATACCTACAGCGTGAGCTATGAGAAAGCGCCACGCTTCCCGAAGGGAGAAAGGCG
GACAGGTATCCGGTAAGCGGCAGGGTCGGAACAGGAGAGCGCACGAGGGAGCTTCCAGGGGG
AAACGCCTGGTATCTTTATAGTCCTGTCGGGTTTCGCCACCTCTGACTTGAGCGTCGATTTTTGT
GATGCTCGTCAGGGGGGCGGAGCCTATGGAAAAACGCCAGCAACGCGGCCTTTTTACGGTTCC
TGGCCTTTTGCTGGCCTTTTGCTCACATGTTCTTTCCTGCGTTATCCCCTGATTCTGTGGATAACC
GTATTACCGCCTTTGAGTGAGCTGATACCGCTCGCCGCAGCCGAACGACCGAGCGCAGCGAGT
CAGTGAGCGAGGAAGCGGAAGAGCGCCTGATGCGGTATTTTCTCCTTACGCATCTGTGCGGTAT
TTCACACCGCATATGGTGCACTCTCAGTACAATCTGCTCTGATGCCGCATAGTTAAGCCAGTATA
CACTCCGCTATCGCTACGTGACTGGGTCATGGCTGCGCCCCGACACCCGCCAACACCCGCTGA
CGCGCCCTGACGGGCTTGTCTGCTCCCGGCATCCGCTTACAGACAAGCTGTGACCGTCTCCGG
GAGCTGCATGTGTCAGAGGTTTTCACCGTCATCACCGAAACGCGCGAGGCAGCAGATCAATTCG
CGCGCGAAGGCGAAGCGGCATGCATAATGTGCCTGTCAAATGGACGAAGCAGGGATTCTGCAA
ACCCTATGCTACTCCGTCAAGCCGTCAATTGTCTGATTCGTTACCAATTATGACAACTTGACGGC
TACATCATTCACTTTTTCTTCACAACCGGCACGGAACTCGCTCGGGCTGGCCCCGGTGCATTTTT
TAAATACCCGCGAGAAATAGAGTTGATCGTCAAAACCAACATTGCGACCGACGGTGGCGATAGG
CATCCGGGTGGTGCTCAAAAGCAGCTTCGCCTGGCTGATACGTTGGTCCTCGCGCCAGCTTAAG
ACGCTAATCCCTAACTGCTGGCGGAAAAGATGTGACAGACGCGACGGCGACAAGCAAACATGCT
GTGCGACGCTGGCGATATCAAAATTGCTGTCTGCCAGGTGATCGCTGATGTACTGACAAGCCTC
GCGTACCCGATTATCCATCGGTGGATGGAGCGACTCGTTAATCGCTTCCATGCGCCGCAGTAAC
AATTGCTCAAGCAGATTTATCGCCAGCAGCTCCGAATAGCGCCCTTCCCCTTGCCCGGCGTTAAT
GATTTGCCCAAACAGGTCGCTGAAATGCGGCTGGTGCGCTTCATCCGGGCGAAAGAACCCCGTA
TTGGCAAATATTGACGGCCAGTTAAGCCATTCATGCCAGTAGGCGCGCGGACGAAAGTAAACCC
ACTGGTGATACCATTCGCGAGCCTCCGGATGACGACCGTAGTGATGAATCTCTCCTGGCGGGAA
CAGCAAAATATCACCCGGTCGGCAAACAAATTCTCGTCCCTGATTTTTCACCACCCCCTGACCGC
GAATGGTGAGATTGAGAATATAACCTTTCATTCCCAGCGGTCGGTCGATAAAAAAATCGAGATAA
CCGTTGGCCTCAATCGGCGTTAAACCCGCCACCAGATGGGCATTAAACGAGTATCCCGGCAGCA
GGGGATCATTTTGCGCTTCAGCCATACTTTTCATACTCCCGCCATTCAGAG

pRC1671

CTGTCTCTTATACACATCTCAACCATCATCGATGAATTGTGTCGTCTTGAGCGATTGTGTAGGCTG
GAGCTGCTTCGAAGTTCCTATACTTTCTAGAGAATAGGAACTTCGGAATAGGAACTTCATTTAAAT
GGCGCGCCTTACGCCCCGCCCTGCCACTCATCGCAGTACTGTTGTATTCATTAAGCATCTGCCG
ACATGGAAGCCATCACAAACGGCATGATGAACCTGAATCGCCAGCGGCATCAGCACCTTGTCGC
CTTGCGTATAATATTTGCCCATGGTGAAAACGGGGGCGAAGAAGTTGTCCATATTGGCCACGTTT
AAATCAAAACTGGTGAAACTCACCCAGGGATTGGCTGAGACGAAAAACATATTCTCAATAAACCC
TTTAGGGAAATAGGCCAGGTTTTCACCGTAACACGCCACATCTTGCGAATATATGTGTAGAAACT
GCCGGAAATCGTCGTGGTATTCACTCCAGAGCGATGAAAACGTTTCAGTTTGCTCATGGAAAACG
GTGTAACAAGGGTGAACACTATCCCATATCACCAGCTCACCGTCTTTCATTGCCATACGTAATTC

CGGATGAGCATTCATCAGGCGGGCAAGAATGTGAATAAAGGCCGGATAAAACTTGTGCTTATTTT
TCTTTACGGTCTTTAAAAAGGCCGTAATATCCAGTTGAACGGTCTGGTTATAGGTACATTGAGCAA
CTGACTGAAATGCCTCAAAATGTTCTTTACGATGCCATTGGGATATATCAACGGTGGTATATCCA
GTGATTTTTTTCTCCATTTTAGCTTCCTTAGCTCCTGAAAATCTCGACAACTCAAAAAATACGCCC
GGTAGTGATCTTATTTCATTATGGTGAAAGTTGGAACCTCTTACGTGCCGATCAACGTCTCATTTT
CGCCAAAAGTTGGCCCAGGGCTTCCCGGTATCAACAGGGACACCAGGATTTATTTATTCTGCGA
AGTGATCTTCCGTCACAGGTAGGCGCGCCGAAGTTCCTATACTTTCTAGAGAATAGGAACTTCGG
AATAGGAACTAAGGAGGATATTCATATGGACCATGGCTAATTACCGTTCCGTGGCAAAGCAAAAG
TTCAAAATCACCAACTGGTCCACCTACAACAAAGCTCTCATCAACCGTGGCGGGGATCCCAAGCT
TCTTCTAGAGGTACCGCATGCGATATCGAGCTCTCCCGGGAATTCCACAAATTGTTATCCGCTCA
CAATTCCACATGTGGAATTCCACATGTGGAATTCCCATGTCAGCCGTTAAGTGTTCCTGTGTCAC
TCAAAATTGCTTTGAGAGGCTCTAAGGGCTTCTCAGTGCGTTACATCCCTGGCTTGTTGTCCACA
ACCGTTAAACCTTAAAAGCTTTAAAAGCCTTATATATTCTTTTTTTTCTTATAAAACTTAAAACCTTA
GAGGCTATTTAAGTTGCTGATTTATATTAATTTTATTGTTCAAACATGAGAGCTTAGTACGTGAAAC
ATGAGAGCTTAGTACGTTAGCCATGAGAGCTTAGTACGTTAGCCATGAGGGTTTAGTTCGTTAAA
CATGAGAGCTTAGTACGTTAAACATGAGAGCTTAGTACGTGAAACATGAGAGCTTAGTACGTACT
ATCAACAGGTTGAACTGCTGATCTTCAGATCCTCTACGCCGGACGCATCGTGGCCGGATCCTCT
AGAGTCGACCTGCAGGCATGCAAGCTTCAGGGTTGAGATGTGTATAAGAGACAGCTGCATTAAT
GAATCGGCCAACGCGCGGGGAGAGGCGGTTTGCGTATTGGGCGCTCTTCCGCTTCCTCGCTCA
CTGACTCGCTGCGCTCGGTCGTTCGGCTGCGGCGAGCGGTATCAGCTCACTCAAAGGCGGTAA
TACGGTTATCCACAGAATCAGGGGATAACGCAGGAAAGAACATGTGAGCAAAAGGCCAGCAAAA
GGCCAGGAACCGTAAAAAGGCCGCGTTGCTGGCGTTTTTCCATAGGCTCCGCCCCCCTGACGA
GCATCACAAAAATCGACGCTCAAGTCAGAGGTGGCGAAACCCGACAGGACTATAAAGATACCAG
GCGTTTCCCCCTGGAAGCTCCCTCGTGCGCTCTCCTGTTCCGACCCTGCCGCTTACCGGATACC
TGTCCGCCTTTCTCCCTTCGGGAAGCGTGGCGCTTTCTCATAGCTCACGCTGTAGGTATCTCAGT
TCGGTGTAGGTCGTTCGCTCCAAGCTGGGCTGTGTGCACGAACCCCCCGTTCAGCCCGACCGC
TGCGCCTTATCCGGTAACTATCGTCTTGAGTCCAACCCGGTAAGACACGACTTATCGCCACTGG
CAGCAGCCACTGGTAACAGGATTAGCAGAGCGAGGTATGTAGGCGGTGCTACAGAGTTCTTGAA
GTGGTGGCCTAACTACGGCTACACTAGAAGGACAGTATTTGGTATCTGCGCTCTGCTGAAGCCA
GTTACCTTCGGAAAAAGAGTTGGTAGCTCTTGATCCGGCAAACAAACCACCGCTGGTAGCGGTG
GTTTTTTTGTTTGCAAGCAGCAGATTACGCGCAGAAAAAAAGGATCTCAAGAAGATCCTTTGATCT
TTTCTACGGGGTCTGACGCTCAGTGGAACGAAAACTCACGTTAAGGGATTTTGGTCATGAGATTA
TCAAAAAGGATCTTCACCTAGATCCTTTTAAATTAAAAATGAAGTTTTAAATCAATCTAAAGTATAT
ATGAGTAAACTTGGTCTGACAGTTACCAATGCTTAATCAGTGAGGCACCTATCTCAGCGATCTGT
CTATTTCGTTCATCCATAGTTGCCTGACTCCCCGTCGTGTAGATAACTACGATACGGGAGGGCTT
ACCATCTGGCCCCAGTGCTGCAATGATACCGCGAGACCCACGCTCACCGGCTCCAGATTTATCA
GCAATAAACCAGCCAGCCGGAAGGGCCGAGCGCAGAAGTGGTCCTGCAACTTTATCCGCCTCC
ATCCAGTCTATTAATTGTTGCCGGGAAGCTAGAGTAAGTAGTTCGCCAGTTAATAGTTTGCGCAA
CGTTGTTGCCATTGCTACAGGCATCGTGGTGTCACGCTCGTCGTTTGGTATGGCTTCATTCAGCT
CCGGTTCCCAACGATCAAGGCGAGTTACATGATCCCCCATGTTGTGCAAAAAAGCGGTTAGCTC
CTTCGGTCCTCCGATCGTTGTCAGAAGTAAGTTGGCCGCAGTGTTATCACTCATGGTTATGGCAG
CACTGCATAATTCTCTTACTGTCATGCCATCCGTAAGATGCTTTTCTGTGACTGGTGAGTACTCAA
CCAAGTCATTCTGAGAATAGTGTATGCGGCGACCGAGTTGCTCTTGCCCGGCGTCAATACGGGA
TAATACCGCGCCACATAGCAGAACTTTAAAAGTGCTCATCATTGGAAAACGTTCTTCGGGGCGAA
AACTCTCAAGGATCTTACCGCTGTTGAGATCCAGTTCGATGTAACCCACTCGTGCACCCAACTGA
TCTTCAGCATCTTTTACTTTCACCAGCGTTTCTGGGTGAGCAAAAACAGGAAGGCAAAATGCCGC
AAAAAAGGGAATAAGGGCGACACGGAAATGTTGAATACTCATACTCTTCCTTTTTCAATATTATTG
AAGCATTTATCAGGGTTATTGTCTCATGAGCGGATACATATTTGAATGTATTTAGAAAAATAAACA
AATAGGGGTTCCGCGCACATTTCCCCGAAAAGTGCCACCTAAATTGTAAGCGTTAATATTTTGTTA
AAATTCGCGTTAAATTTTTGTTAAATCAGCTCATTTTTTAACCAATAGGCCGAAATCGGCAAAATC

CCTTATAAATCAAAAGAATAGACCGAGATAGGGTTGAGTGTTGTTCCAGTTTGGAACAAGAGTCC
ACTATTAAAGAACGTGGACTCCAACGTCAAAGGGCGAAAAACCGTCTATCAGGGCGATGGCCCA
CTACGTGAACCATCACCCTAATCAAGTTTTTTGGGGTCGAGGTGCCGTAAAGCACTAAATCGGAA
CCCTAAAGGGAGCCCCCGATTTAGAGCTTGACGGGGAAAGCCGGCGAACGTGGCGAGAAAGGA
AGGGAAGAAAGCGAAAGGAGCGGGCGCTAGGGCGCTGGCAAGTGTAGCGGTCACGCTGCGCG
TAACCACCACACCCGCCGCGCTTAATGCGCCGCTACAGGGCGCGTCCCATTCGCCATTCAGGCT
GCGCAACTGTTGGGAAGGGCGATCGGTGCGGGCCTCTTCGCTATTACGCCAG

pRC2128

AAATCCGCCGGGAGCGGATTTGAACGTTGCGAAGCAACGGCCCGGAGGGTGGCGGGCAGGAC
GCCCGCCATAAACTGCCAGGAATTAATTCCCCAGGCATCAAATAAAACGAAAGGCTCAGTCGAAA
GACTGGGCCTTTCGTTTTATCTGTTGTTTGTCGGTGAACGCTCTCCTGAGTAGGACAAATCCGCC
GGGAGCGGATTTGAACGTTGCGAAGCAACGGCCCGGAGGGTGGCGGGCAGGACGCCCGCCAT
AAACTGCCAGGAATTAATTCCCCAGGCATCAAATAAAACGAAAGGCTCAGTCGAAAGACTGGGC
CTTTCGTTTTATCTGTTGTTTGTCGGTGAACGCTCTCCTGAGTAGGACAAATCCGCCGGGAGCGG
ATTTGAACGTTGCGAAGCAACGGCCCGGAGGGTGGCGGGCAGGACGCCCGCCATAAACTGCCA
GGAATTGGGGATCGGAATTAATTCCCGGTTTAAACCGGGGATCTCGATCCCGCGAAATTAATAC
GACTCACTATAGGGGAATTGTGAGCGGATAACAATTCCCCTCTAGAAATAATTTTGTTTAACTTTA
AGAAGGAGATATACATATGATAACTTCTGCTCTTCATCGTGCGGCCGACTGGGCTAAATCTGTGT
TCTCTTCGGCGGCGCTGGGTGATCCTCGCCGTACTGCCCGCTTGGTTAACGTCGCCGCCCAATT
GGCAAAATATTCTGGTAAATCAATAACCATCTCATCAGAGGGTAGTAAGGCCGCACAGGAAGGC
GCTTACCGATTTATCCGCAATCCCAACGTTTCTGCCGAGGCGATCAGAAAGGCTGGCGCCATGC
AAACAGTCAAGTTGGCTCAGGAGTTTCCCGAACTGCTGGCCATTGAGGACACCACCTCTTTGAG
TTATCGCCACCAGGTCGCCGAAGAGCTTGGCAAGCTGGGCTCTATTCAGGATAAATCCCGCGGA
TGGTGGGTTCACTCCGTTCTCTTGCTCGAGGCCACCACATTCCGCACCGTAGGATTACTGCATC
AGGAGTGGTGGATGCGCCCGGATGACCCTGCCGATGCGGATGAAAAGGAGAGTGGCAAATGGC
TGGCAGCGGCCGCAACTAGCCGGTTACGCATGGGCAGCATGATGAGCAACGTGATTGCGGTCT
GTGACCGCGAAGCCGATATTCATGCTTATCTGCAGGACAAACTGGCGCATAACGAGCGCTTCGT
GGTGCGCTCCAAGCACCCACGCAAGGACGTAGAGTCTGGGTTGTATCTGTACGACCATCTGAAG
AACCAACCGGAGTTGGGTGGCTATCAGATCAGCATTCCGCAAAAGGGCGTGGTGGATAAACGC
GGTAAACGTAAAAATCGACCAGCCCGCAAGGCGAGCTTGAGCCTGCGCAGTGGGCGCATCACG
CTAAAACAGGGGAATATCACGCTCAACGCGGTGCTGGCCGAGGAGATTAACCCGCCCAAGGGT
GAGACCCCGTTGAAATGGTTGTTGCTGACCAGCGAACCGGTCGAGTCGCTAGCCCAAGCCTTGC
GCGTCATCGACATTTATACCCATCGCTGGCGGATCGAGGAGTTCCATAAGGCATGGAAAACCGG
AGCAGGAGCCGAGAGGCAACGCATGGAGGAGCCGGATAATCTGGAGCGGATGGTCTCGATCCT
CTCGTTTGTTGCGGTCAGGCTGTTACAGCTCAGAGAAAGCTTCACGCCGCCGCAAGCACTCAGG
GCGCAAGGGCTGCTAAAGGAAGCGGAACACGTAGAAAGCCAGTCCGCAGAAACGGTGCTGACC
CCGGATGAATGTCAGCTACTGGGCTATCTGGACAAGGGAAAACGCAAGCGCAAAGAGAAAGCA
GGTAGCTTGCAGTGGGCTTACATGGCGATAGCTAGACTGGGCGGTTTTATGGACAGCAAGCGAA
CCGGAATTGCCAGCTGGGGCGCCCTCTGGGAAGGTTGGGAAGCCCTGCAAAGTAAACTGGATG
GCTTTCTTGCCGCCAAGGATCTGATGGCGCAGGGGATCAAGATCGGGGAATTCCTCGAGCCCG
GGTGCTTTGCCAAGGGTACCAATGTTTTAATGGCGGATGGGTCTATTGAATGTATTGAAAACATT
GAGGTTGGTAATAAGGTCATGGGTAAAGATGGCAGACCTCGTGAGGTAATTAAATTGCCCAGAG
GAAGAGAAACTATGTACAGCGTCGTGCAGAAAAGTCAGCACAGAGCCCACAAAAGTGACTCAAG
TCGTGAAGTGCCAGAATTACTCAAGTTTACGTGTAATGCGACCCATGAGTTGGTTGTTAGAACAC
CTCGTAGTGTCCGCCGTTTGTCTCGTACCATTAAGGGTGTCGAATATTTTGAAGTTATTACTTTTG
AGATGGGCCAAAAGAAAGCCCCCGACGGTAGAATTGTTGAGCTTGTCAAGGAAGTTTCAAAGAG
CTACCCAATATCTGAGGGGCCTGAGAGAGCCAACGAATTAGTAGAATCCTATAGAAAGGCTTCAA

ATAAAGCTTATTTTGAGTGGACTATTGAGGCCAGAGATCTTTCTCTGTTGGGTTCCCATGTTCGTA
AAGCTACCTACCAGACTTACGCTCCAATTCTTTATGAGAATGACCACTTTTTCGACTACATGCAAA
AAAGTAAGTTTCATCTCACCATTGAAGGTCCAAAAGTACTTGCTTATTTACTTGGTTTATGGATTG
GTGATGGATTGTCTGACAGGGCAACTTTTTCGGTTGATTCCAGAGATACTTCTTTGATGGAACGT
GTTACTGAATATGCTGAAAAGTTGAATTTGTGCGCCGAGTATAAGGACAGAAAAGAACCACAAGT
TGCCAAAACTGTTAATTTGTACTCTAAAGTTGTCAGAGGTAATGGTATTCGCAATAATCTTAATACT
GAGAATCCATTATGGGACGCTATTGTTGGCTTAGGATTCTTGAAGGACGGTGTCAAAAATATTCC
TTCTTTCTTGTCTACGGACAATATCGGTACTCGTGAAACATTTCTTGCTGGTCTAATTGATTCTGA
TGGCTATGTTACTGATGAGCATGGTATTAAAGCAACAATAAAGACAATTCATACTTCTGTCAGAGA
TGGTTTGGTTTCCCTTGCTCGTTCTTTAGGCTTAGTAGTCTCGGTTAACGCAGAACCTGCTAAGG
TTGACATGAATGTCACCAAACATAAAATTAGTTATGCTATTTATATGTCTGGTGGAGATGTTTTGCT
TAACGTTCTTTCGAAGTGTGCCGGCTCTAAAAAATTCAGGCCTGCTCCCGCCGCTGCTTTTGCAC
GTGAGTGCCGCGGATTTTATTTCGAGTTACAAGAATTGAAGGAAGACGATTATTATGGGATTACT
TTATCTGATGATTCTGATCATCAGTTTTTGCTTGGATCCCAGGTTGTCGTCCATGCATGCGGTGG
CCTGACCGGTCTGAACTCAGGCCTCACGACAAATCCTGGTGTATCCGCTTGGCAGGTCAACACA
GCTTATACTGCGGGACAATTGGTCACATATAACGGCAAGACGTATAAATGTTTGCAGCCCCACAC
CTCCTTGGCAGGATGGGAACCATCCAACGTTCCTGCCTTGTGGCAGCTTCAATGACTGCAGGAA
GGGGATCCGGCTGCTAACAAAGCCCGAAAGGAAGCTGAGTTGGCTGCTGCCACCGCTGAGCAA
TAACTAGCATAACCCCTTGGGGCCTCTAAACGGGTCTTGAGGGGTTTTTTGCTGAAAGGAGGAA
CTATATCCGGATAACTACGTCAGGTGGCACTTTTCGGGGAAATGTGCGCGGAACCCCTATTTGTT
TATTTTTCTAAATACATTCAAATATGTATCCGCTCATGAGACAATAACCCTGATAAATGCTTCAATA
ATATTGAAAAAGGAAGAGTATGAGTATTCAACATTTCCGTGTCGCCCTTATTCCCTTTTTTGCGGC
ATTTTGCCTTCCTGTTTTTGCTCACCCAGAAACGCTGGTGAAAGTAAAAGATGCTGAAGATCAGTT
GGGTGCACGAGTGGGTTACATCGAACTGGATCTCAACAGCGGTAAGATCCTTGAGAGTTTTCGC
CCCGAAGAACGTTCTCCAATGATGAGCACTTTTAAAGTTCTGCTATGTGGCGCGGTATTATCCCG
TGTTGACGCCGGGCAAGAGCAACTCGGTCGCCGCATACACTATTCTCAGAATGACTTGGTTGAG
TACTCACCAGTCACAGAAAAGCATCTTACGGATGGCATGACAGTAAGAGAATTATGCAGTGCTGC
CATAACCATGAGTGATAACACTGCGGCCAACTTACTTCTGACAACGATCGGAGGACCGAAGGAG
CTAACCGCTTTTTTGCACAACATGGGGGATCATGTAACTCGCCTTGATCGTTGGGAACCGGAGCT
GAATGAAGCCATACCAAACGACGAGCGTGACACCACGATGCCTGTAGCAATGGCAACAACGTTG
CGCAAACTATTAACTGGCGAACTACTTACTCTAGCTTCCCGGCAACAATTAATAGACTGGATGGA
GGCGGATAAAGTTGCAGGACCACTTCTGCGCTCGGCCCTTCCGGCTGGCTGGTTTATTGCTGAT
AAATCTGGAGCCGGTGAGCGTGGGTCTCGCGGTATCATTGCAGCACTGGGGCCAGATGGTAAG
CCCTCCCGTATCGTAGTTATCTACACGACGGGGAGTCAGGCAACTATGGATGAACGAAATAGAC
AGATCGCTGAGATAGGTGCCTCACTGATTAAGCATTGGTAACTGTCAGACCAAGTTTACTCATAT
ATACTTTAGATTGATTTACCCCGGTTGATAATCAGAAAAGCCCCAAAAACAGGAAGATTGTATAAG
CAAATATTTAAATTGTAAACGTTAATATTTTGTTAAAATTCGCGTTAAATTTTTGTTAAATCAGCTCA
TTTTTTAACCAATAGGCCGAAATCGGCAAAATCCCTTATAAATCAAAAGAATAGCCCGAGATAGG
GTTGAGTGTTGTTCCAGTTTGGAACAAGAGTCCACTATTAAAGAACGTGGACTCCAACGTCAAAG
GGCGAAAAACCGTCTATCAGGGCGATGGCCCACTACGTGAACCATCACCCAAATCAAGTTTTTTG
GGGTCGAGGTGCCGTAAAGCACTAAATCGGAACCCTAAAGGGAGCCCCCGATTTAGAGCTTGAC
GGGGAAAGCCGGCGAACGTGGCGAGAAAGGAAGGGAAGAAAGCGAAAGGAGCGGGCGCTAGG
GCGCTGGCAAGTGTAGCGGTCACGCTGCGCGTAACCACCACACCCGCCGCGCTTAATGCGCCG
CTACAGGGCGCGTAAAAGGATCTAGGTGAAGATCCTTTTTGATAATCTCATGACCAAAATCCCTT
AACGTGAGTTTTCGTTCCACTGAGCGTCAGACCCCGTAGAAAAGATCAAAGGATCTTCTTGAGAT
CCTTTTTTTCTGCGCGTAATCTGCTGCTTGCAAACAAAAAAACCACCGCTACCAGCGGTGGTTTG
TTTGCCGGATCAAGAGCTACCAACTCTTTTTCCGAAGGTAACTGGCTTCAGCAGAGCGCAGATAC
CAAATACTGTCCTTCTAGTGTAGCCGTAGTTAGGCCACCACTTCAAGAACTCTGTAGCACCGCCT
ACATACCTCGCTCTGCTAATCCTGTTACCAGTGGCTGCTGCCAGTGGCGATAAGTCGTGTCTTAC
CGGGTTGGACTCAAGACGATAGTTACCGGATAAGGCGCAGCGGTCGGGCTGAACGGGGGGTTC

GTGCACACAGCCCAGCTTGGAGCGAACGACCTACACCGAACTGAGATACCTACAGCGTGAGCTA
TGAGAAAGCGCCACGCTTCCCGAAGGGAGAAAGGCGGACAGGTATCCGGTAAGCGGCAGGGTC
GGAACAGGAGAGCGCACGAGGGAGCTTCCAGGGGGAAACGCCTGGTATCTTTATAGTCCTGTC
GGGTTTCGCCACCTCTGACTTGAGCGTCGATTTTTGTGATGCTCGTCAGGGGGGCGGAGCCTAT
GGAAAAACGCCAGCAACGCGGCCTTTTTACGGTTCCTGGCCTTTTGCTGGCCTTTTGCTCACATG
TTCTTTCCTGCGTTATCCCCTGATTCTGTGGATAACCGTATTACCGCCTTTGAGTGAGCTGATACC
GCTCGCCGCAGCCGAACGACCGAGCGCAGCGAGTCAGTGAGCGAGGAAGCTATGGTGCACTCT
CAGTACAATCTGCTCTGATGCCGCATAGTTAAGCCAGTATACACTCCGCTATCGCTACGTGACTG
GGTCATGGCTGCGCCCCGACACCCGCCAACACCCGCTGACGCGCCCTGACGGGCTTGTCTGCT
CCCGGCATCCGCTTACAGACAAGCTGTGACCGTCTCCGGGAGCTGCATGTGTCAGAGGTTTTCA
CCGTCATCACCGAAACGCGCGAGGCAGCTGCGGTAAAGCTCATCAGCGTGGTCGTGCAGCGAT
TCACAGATGTCTGCCTGTTCATCCGCGTCCAGCTCGTTGAGTTTCTCCAGAAGCGTTAATGTCTG
GCTTCTGATAAAGCGGGCCATGTTAAGGGCGGTTTTTTCCTGTTTGGTCACTGATGCCTCCGTGT
AAGGGGGATTTCTGTTCATGGGGGTAATGATACCGATGAAACGAGAGAGGATGCTCACGATACG
GGTTACTGATGATGAACATGCCCGGTTACTGGAACGTTGTGAGGGTAAACAACTGGCGGTATGG
ATGCGGCGGGACCAGAGAAAAATCACTCAGGGTCAATGCCAGCCGAACGCCAGCAAGACGTAG
CCCAGCGCGTCGGCCGCCATGCCGGCGATAATGGCCTGCTTCTCGCCGAAACGTTTGGTGGCG
GGACCAGTGACGAAGGCTTGAGCGAGGGCGTGCAAGATTCCGAATACCGCAAGCGACAGGCCG
ATCATCGTCGCGCTCCAGCGAAAGCGGTCCTCGCCGAAAATGACCCAGAGCGCTGCCGGCACC
TGTCCTACGAGTTGCATGATAAAGAAGACAGTCATAAGTGCGGCGACGATAGTCATGCCCCGCG
CCCACCGGAAGGAGCTGACTGGGTTGAAGGCTCTCAAGGGCATCGGTCGAGATCCCGGTGCCT
AATGAGTGAGCTAACTTACATTAATTGCGTTGCGCTCACTGCCCGCTTTCCAGTCGGGAAACCTG
TCGTGCCAGCTGCATTAATGAATCGGCCAACGCGCGGGGAGAGGCGGTTTGCGTATTGGGCGC
CAGGGTGGTTTTTCTTTTCACCAGTGAGACGGGCAACAGCTGATTGCCCTTCACCGCCTGGCCC
TGAGAGAGTTGCAGCAAGCGGTCCACGCTGGTTTGCCCCAGCAGGCGAAAATCCTGTTTGATGG
TGGTTAACGGCGGGATATAACATGAGCTGTCTTCGGTATCGTCGTATCCCACTACCGAGATATCC
GCACCAACGCGCAGCCCGGACTCGGTAATGGCGCGCATTGCGCCCAGCGCCATCTGATCGTTG
GCAACCAGCATCGCAGTGGGAACGATGCCCTCATTCAGCATTTGCATGGTTTGTTGAAAACCGG
ACATGGCACTCCAGTCGCCTTCCCGTTCCGCTATCGGCTGAATTTGATTGCGAGTGAGATATTTA
TGCCAGCCAGCCAGACGCAGACGCGCCGAGACAGAACTTAATGGGCCCGCTAACAGCGCGATT
TGCTGGTGACCCAATGCGACCAGATGCTCCACGCCCAGTCGCGTACCGTCTTCATGGGAGAAAA
TAATACTGTTGATGGGTGTCTGGTCAGAGACATCAAGAAATAACGCCGGAACATTAGTGCAGGCA
GCTTCCACAGCAATGGCATCCTGGTCATCCAGCGGATAGTTAATGATCAGCCCACTGACGCGTT
GCGCGAGAAGATTGTGCACCGCCGCTTTACAGGCTTCGACGCCGCTTCGTTCTACCATCGACAC
CACCACGCTGGCACCCAGTTGATCGGCGCGAGATTTAATCGCCGCGACAATTTGCGACGGCGC
GTGCAGGGCCAGACTGGAGGTGGCAACGCCAATCAGCAACGACTGTTTGCCCGCCAGTTGTTG
TGCCACGCGGTTGGGAATGTAATTCAGCTCCGCCATCGCCGCTTCCACTTTTTCCCGCGTTTTCG
CAGAAACGTGGCTGGCCTGGTTCACCACGCGGGAAACGGTCTGATAAGAGACACCGGCATACT
CTGCGACATCGTATAACGTTACTGGTTTCACATTCACCACCCTGAATTGACTCTCTTCCGGGCGC
TATCATGCCATACCGCGAAAGGTTTTGCGCCATTCGATGGTGTCCGGGATCTCGACGCTCTCCC
TTATGCGACTCCTGCATTAGGAAGCAGCCCAGTAGTAGGTTGAGGCCGTTGAGCACCGCCGCC
GCAAGGAATGGTGCATGCCGGCATGCCGCCCTTTCGTCTTCAAGAATTAATTCCCAATTCCCCAG
GCATCAAATAAAACGAAAGGCTCAGTCGAAAGACTGGGCCTTTCGTTTTATCTGTTGTTTGTCGG
TGAACGCTCTCCTGAGTAGGACAAATCCGCCGGGAGCGGATTTGAACGTTGCGAAGCAACGGC
CCGGAGGGTGGCGGGCAGGACGCCCGCCATAAACTGCCAGGAATTAATTCCCCAGGCATCAAA
TAAAACGAAAGGCTCAGTCGAAAGACTGGGCCTTTCGTTTTATCTGTTGTTTGTCGGTGAACGCT
CTCCTGAGTAGGACAAATCCGCCGGGAGCGGATTTGAACGTTGCGAAGCAACGGCCCGGAGGG
TGGCGGGCAGGACGCCCGCCATAAACTGCCAGGAATTAATTCCCCAGGCATCAAATAAAACGAA
AGGCTCAGTCGAAAGACTGGGCCTTTCGTTTTATCTGTTGTTTGTCGGTGAACGCTCTCCTGAGT
AGGAC

pRC2747

CTCGGTACCAAATTCCAGAAAAGAGGCCTCCCGAAAGGGGGGCCTTTTTTCGTTTTGGTCCAAT
GGCGGCGCGCCATCGAATGGTGCAAAACCTTTCGCGGTATGGCATGATAGCGCCCGGAAGAGA
GTCAATTCAGGGTGGTGAATATGGCTGAAGCGCAAAATGATCCCCTGCTGCCGGGATACTCGTT
TAATGCCCATCTGGTGGCGGGTTTAACGCCGATTGAGGCCAACGGTTATCTCGATTTTTTTATCG
ACCGACCGCTGGGAATGAAAGGTTATATTCTCAATCTCACCATTCGCGGTCAGGGGGTGGTGAA
AAATCAGGGACGAGAATTTGTTTGCCGACCGGGTGATATTTTGCTGTTCCCGCCAGGAGAGATT
CATCACTACGGTCGTCATCCGGAGGCTCGCGAATGGTATCACCAGTGGGTTTACTTTCGTCCGC
GCGCCTACTGGCATGAATGGCTTAACTGGCCGTCAATATTTGCCAATACGGGGTTCTTTCGCCC
GGATGAAGCGCACCAGCCGCATTTCAGCGACTTTTTTGGGCAAATCATTAACGCCGGGCAAGGG
GAAGGGCGCTATTCGGAGCTGCTGGCGATAAATCTGCTTGAGCAATTGTTACTGCGGCGCATGC
TAGCGATTAACGGATCGCTCCATCCACCGATGGATAATCGGGTACGCGAGGCTTGTCAGTACAT
CAGCGATCACCTGGCAGACAGCAATTTTGATATCGCCAGCGTCGCACAGCATGTTTGCTTGTCG
CCGTCGCGTCTGTCACATCTTTTCCGCCAGCAGTTAGGGATTAGCGTCTTAAGCTGGCGCGAGG
ACCAACGTATCAGCCAGGCGAAGCTGCTTTTGAGCACCACCCGGATGCCTATCGCCACCGTCGG
TCGCAATGTTGGTTTTGACGATCAACTCTATTTCTCGCGGGTATTTAAAAAATGCACCGGGGCCA
GCCCGAGCGAGTTCCGTGCCGGTTTGGAAGAAAAAGTGAATGATGTAGCCGTCAAGTTGTCATG
ATAAGATCCTATTCCAGCGGGATTAAAGAGGAGCGATTAAGCATGGTTACTATCAATACGGAATC
TGCTTTAACGCCACGTTCTTTGCGGGATACGCGGCGTATGAATATGTTTGTTTCGGTAGCTGCTG
CGGTCGCAGGATTGTTATTTGGTCTTGATATCGGCGTAATCGCCGGAGCGTTGCCGTTCATTACC
GATCACTTTGTGCTGACCAGTCGTTTGCAGGAATGGGTGGTTAGTAGCATGATGCTCGGTGCAG
CAATTGGTGCGCTGTTTAATGGTTGGCTGTCGTTCCGCCTGGGGCGTAAATACAGCCTGATGGC
GGGGGCCATCCTGTTTGTACTCGGTTCTATAGGGTCCGCTTTTGCGACCAGCGTAGAGATGTTA
ATCGCCGCTCGTGTGGTGCTGGGCATTGCTGTCGGGATCGCGTCTTACACCGCTCCTCTGTATC
TTTCTGAAATGGCAAGTGAAAACGTTCGCGGTAAGATGATCAGTATGTACCAGTTGATGGTCACA
CTCGGCATCGTGCTGGCGTTTTTATCCGATACAGCGTTCAGTTATAGCGGTAACTGGCGCGCAA
TGTTGGGGGTTCTTGCTTTACCAGCAGTTCTGCTGATTATTCTGGTAGTATTTCTGCCAAATAGCC
CGCGCTGGCTGGCGGAAAAGGGGCGTCATATTGAGGCGGAAGAAGTATTGCGTATGCTGCGCG
ATACGTCGGAAAAAGCGCGAGAAGAACTCAACGAAATTCGTGAAAGCCTGAAGTTAAAACAGGG
CGGTTGGGCACTGTTTAAGATCAACCGTAACGTCCGTCGTGCTGTGTTTCTCGGTATGTTGTTGC
AGGCGATGCAGCAGTTTACCGGTATGAACATCATCATGTACTACGCGCCGCGTATCTTCAAAATG
GCGGGCTTTACGACCACAGAACAACAGATGATTGCGACTCTGGTCGTAGGGCTGACCTTTATGT
TCGCCACCTTTATTGCGGTGTTTACGGTAGATAAAGCAGGGCGTAAACCGGCTCTGAAAATTGGT
TTCAGCGTGATGGCGTTAGGCACTCTGGTGCTGGGCTATTGCCTGATGCAGTTTGATAACGGTA
CGGCTTCCAGTGGCTTGTCCTGGCTCTCTGTTGGCATGACGATGATGTGTATTGCCGGTTATGC
GATGAGCGCCGCGCCAGTGGTGTGGATCCTGTGCTCTGAAATTCAGCCGCTGAAATGCCGCGA
TTTCGGTATTACCTGTTCGACCACCACGAACTGGGTGTCGAATATGATTATCGGCGCGACCTTCC
TGACACTGCTTGATAGCATTGGCGCTGCCGGTACGTTCTGGCTCTACACTGCGCTGAACATTGC
GTTTGTGGGCATTACTTTCTGGCTCATTCCGGAAACCAAAAATGTCACGCTGGAACATATCGAAC
GCAAACTGATGGCAGGCGAGAAGTTGAGAAATATCGGCGTCTGATAAGGATCCTAATTGGTAAC
GAATCAGACAATTGACGGCTCGAGGGAGTAGCATAGGGTTTGCAGAATCCCTGCTTCGTCCATT
TGACAGGCACATTATGCATCGATGATAAGCTGTCAAACATGAGCAGATCCTCTACGCCGGACGC
ATCGTGGCCGGCATCACCGGCGCCACAGGTGCGGTTGCTGGCGCCTATATCGCCGACATCACC
GATGGGGAAGATCGGGCTCGCCACTTCGGGCTCATGAGCAAATATTTTATCTGAGGTGCTTCCT
CGCTCACTGACTCGCTGCACGAGGCAGACCTCAGCGCTAGCGGAGTGTATACTGGCTTACTATG
TTGGCACTGATGAGGGTGTCAGTGAAGTGCTTCATGTGGCAGGAGAAAAAAGGCTGCACCGGTG
CGTCAGCAGAATATGTGATACAGGATATATTCCGCTTCCTCGCTCACTGACTCGCTACGCTCGGT
CGTTCGACTGCGGCGAGCGGAAATGGCTTACGAACGGGGCGGAGATTTCCTGGAAGATGCCAG

GAAGATACTTAACAGGGAAGTGAGAGGGCCGCGGCAAAGCCGTTTTTCCATAGGCTCCGCCCC
CCTGACAAGCATCACGAAATCTGACGCTCAAATCAGTGGTGGCGAAACCCGACAGGACTATAAA
GATACCAGGCGTTTCCCCCTGGCGGCTCCCTCGTGCGCTCTCCTGTTCCTGCCTTTCGGTTTAC
CGGTGTCATTCCGCTGTTATGGCCGCGTTTGTCTCATTCCACGCCTGACACTCAGTTCCGGGTA
GGCAGTTCGCTCCAAGCTGGACTGTATGCACGAACCCCCCGTTCAGTCCGACCGCTGCGCCTTA
TCCGGTAACTATCGTCTTGAGTCCAACCCGGAAAGACATGCAAAAGCACCACTGGCAGCAGCCA
CTGGTAATTGATTTAGAGGAGTTAGTCTTGAAGTCATGCGCCGGTTAAGGCTAAACTGAAAGGAC
AAGTTTTGGTGACTGCGCTCCTCCAAGCCAGTTACCTCGGTTCAAAGAGTTGGTAGCTCAGAGAA
CCTTCGAAAAACCGCCCTGCAAGGCGGTTTTTTCGTTTTCAGAGCAAGAGATTACGCGCAGACC
AAAACGATCTCAAGAAGATCATCTTATTAAGGGGTCTGACGCTCAGTGGAACGAAAAATCAATCT
AAAGTATATATGAGTAAACTTGGTCTGACAGTTACCTTAGAAAAACTCATCGAGCATCAAATGAAA
CTGCAATTTATTCATATCAGGATTATCAATACCATATTTTTGAAAAAGCCGTTTCTGTAATGAAGGA
GAAAACTCACCGAGGCAGTTCCATAGGATGGCAAGATCCTGGTATCGGTCTGCGATTCCGACTC
GTCCAACATCAATACAACCTATTAATTTCCCCTCGTCAAAAATAAGGTTATCAAGTGAGAAATCAC
CATGAGTGACGACTGAATCCGGTGAGAATGGCAAAAGCTTATGCATTTCTTTCCAGACTTGTTCA
ACAGGCCAGCCATTACGCTCGTCATCAAAATCACTCGCATCAACCAAACCGTTATTCATTCGTGA
TTGCGCCTGAGCGAGACGAAATACGCGATCGCTGTTAAAAGGACAATTACAAACAGGAATCGAA
TGCAACCGGCGCAGGAACACTGCCAGCGCATCAACAATATTTTCACCTGAATCAGGATATTCTTC
TAATACCTGGAATGCTGTTTTCCCGGGGATCGCAGTGGTGAGTAACCATGCATCATCAGGAGTA
CGGATAAAATGCTTGATGGTCGGAAGAGGCATAAATTCCGTCAGCCAGTTTAGTCTGACCATCTC
ATCTGTAACATCATTGGCAACGCTACCTTTGCCATGTTTCAGAAACAACTCTGGCGCATCGGGCT
TCCCATACAATCGATAGATTGTCGCACCTGATTGCCCGACATTATCGCGAGCCCATTTATACCCA
TATAAATCAGCATCCATGTTGGAATTTAATCGCGGCCTCGAGCAAGACGTTTCCCGTTGAATATG
GCTCATAACACCCCTTGTATTACTGTTTATGTAAGCAGACAGTTTTATTGTTCATGATGATATATTT
TTATCTTGTGCAATGTACATCAGAGATTTTGAGACACAACCAATTATTGAAGGCCTCCCTAACGG
GGGGCCTTTTTTTGTTTCTGGTCTCCCGCTTAACGATCGTTGGCTGAGAAACCAATTGTCCATATT
GCATCAGACATTGCCGTCACTGCGTCTTTTACTGGCTCTTCTCGCTAACCAAACCGGTAACCCCG
CTTATTAAAAGCATTCTGTAACAAAGCGGGACCAAAGCCATGACAAAAACGCGTAACAAAAGTGT
CTATAATCACGGCAGAAAAGTCCACATTGATTATTTGCACGGCGTCACACTTTGCTATGCCATAG
CATTTTTATCCATAAGATTAGCGGATCCTACCTGACGCTTTTTATCGCAACTCTCTACTGTTTCTC
CATACCCGAGCTGTCACCGGATGTGCTTTCCGGTCTGATGAGTCCGTGAGGACGAAACAGCCTC
TACAAATAATTTTGTTTAATACTAGAGAAAGAGGGGAAATACTAGATGGCCTGGCTTCCCCTTAAT
CCCATTCCACTCAAAGATCGCGTCTCCATGATCTTTCTGCAATATGGGCAGATCGATGTAATAGA
TGGCGCGTTTGTACTTATCGACAAGACAGGGATCCGCACTCATATTCCTGTTGGCTCGGTTGCCT
GCATCATGCTGGAACCTGGTACACGGGTTTCGCATGCAGCTGTACGCCTGGCTGCGCAAGTTGG
AACATTGTTGGTATGGGTGGGGGAAGCGGGCGTTCGTGTTTATGCTTCTGGTCAGCCTGGAGGT
GCGCGTTCAGATAAGCTGCTCTATCAGGCAAAACTTGCTCTGGATGAAGATTTGCGTCTGAAGGT
CGTACGTAAAATGTTTGAACTTCGGTTTGGAGAACCTGCGCCTGCCCGGCGCTCCGTAGAGCAA
CTCAGAGGTATAGAAGGCAGTCGCGTGCGGGCAACCTACGCACTTCTGGCGAAGCAATACGGC
GTGACATGGAATGGACGTCGCTACGATCCGAAAGACTGGGAAAAGGGCGATACGATCAACCAAT
GCATTAGCGCTGCAACTTCCTGTTTATACGGCGTAACTGAAGCGGCGATACTTGCAGCTGGTTAT
GCACCAGCTATTGGGTTTGTGCATACAGGAAAGCCTCTTTCCTTTGTTTACGATATTGCAGACAT
CATTAAATTTGACACTGTTGTACCGAAAGCTTTTGAGATAGCGCGTCGTAACCCTGGTGAGCCGG
ACCGGGAAGTCCGTTTGGCGTGCAGGGATATTTTTCGCAGTAGTAAAACATTAGCCAAATTGATT
CCGCTTATAGAGGACGTGCTTGCCGCTGGAGAAATACAACCGCCGGCCCCACCTGAAGATGCA
CAGCCTGTTGCCATTCCGCTTCCTGTTTCACTGGGAGATGCAGGCCATCGGAGTAGCTGAAATG
AGTATGTTGGTCGTGGTCACTGAAAATGTACCTCCGCGCTTACGAGGCAGATTAGCCATCTGGTT
GTTGGAGGTACGTGCAGGGGTATATGTAGGTGATGTATCCGCAAAAATTCGTGAAATGATCTGG
GAACAAATAGCTGGACTGGCGGAAGAAGGCAATGTAGTGATGGCATGGGCAACGAATACGGAAA

CGGGATTTGAGTTCCAGACATTTGGGTTAAACAGGCGTACCCCGGTAGATTTGGATGGTTTAAG
GTTGGTGTCTTTTTTACCTGTTTGAGCGG

RC5311 Papillation reporter genome sequence

tcaggaagcttggatcaaccggtagttatccaaagaacaactgttgttcagtttttgagttgtgtataacccctcattctgatcccagcttatacggtc
caggatcaccgatcattcacagttaatgatcctttccaggttgttgatcttaaaagccggatccttgttatccacagggcagtgcgatcctaataag
agatcacaatagaacagatctctaaataaatagatcttctttttaatacccaggatcccaggtctttctcaagccgacaaagttgagtagaatcca
cggcccgggcttcaatccattttcataccgcgttatgcgaggcaatcaccatgttttatccggatccttttgacgtcatcatcattggcgggggtcat
gcaggcaccgaggccgcgatggccgcggcgcgtatgggtcaacagactctgcttttgacacacaatatcgacactctggggcagatgagct
gcaacccggcgatcggcggtattgggaagggacatctggtaaaagaagtggatgcactcggcggtctgatggcgaaagcgatcgatcagg
cgggtatccagtttaggatactaaacgcaagcaaaggaccggcggttcgcgctacccgagctcaggcggatcgtgtgctctaccgtcaggcg
gtacgtacggcgctggagaaccaaccgaacctgatgatcttccagcaggcggttgaagatcttattgtcgaaaacgatcgcgtggtcggtgct
gttacccaaatgggactgaagttccgtgccaaagccgtcgtgctcaccgttgggacgttcctcgacggtaaaattcatatcggtctggataattac
agcggtggccgtgctggtgatccgccgtccattccgctttctcgccgtttgcgtgaactgccgctgcgcgttggtcgtctgaaaaccgggacacc
accgcgtattgatgctcgaaccatcgactttagcgtactggcgcaacagcatggcgataacccaatgccggtattctcgtttatgggcaatgcgt
cccagcatccccagcaggtgccgtgttatatcactcataccaacgagaaaacccatgatgtgatccgcagtaacctcgatcgtagcccaatgt
acgcaggggtgatcgaaggtgtcggcccacgctactgcccgtcgatcgaagacaaagtcatgcgcttcgccgacagaaatcagcatcagat
cttccttgaaccggaaggactgacctctaatgaaatttatccgaacggtatctccaccagcctgccgttcgatgtgcagatgcaaatcgtccgctc
tatgcaggggatggaaaacgcgaagatcgtgcgtccgggttatgccattgagtatgacttcttcgatcctcgcgacctgaaaccgacgctgga
gagcaagtttatccaggggctgttctttgctggtcagattaacggcactaccggttacgaagaagccgctgcgcaaggtttgctggccggtctta
acgctgcccgtctgtctgctgacaaagaaggttgggctccggcacgttctcaggcgtatctcggcgtactagttgatgacctgtgcactttaggaa
ccaaagaaccgtatcgtatgtttacttcgcgcgcagaatatcgtctgatgctacgcgaagataatgcggatctgcgtttgactgaaatcggtcgtg
aactgggcctggtggatgacgaacgttgggcgcgctttaacgagaaacttgagaatatcgagcgtgagcgtcagcgtctgaaatcgacctgg
gtaaccccgtcggcggaagctgcagccgaagtgaatgctcacctgactgcgccgctttcccgtgaagccagtggtgaagatctgctgcgtcgt
ccggaaatgacttatgaaaaattaaccacgctgacgccgtttgcccctgcgttgacagacgaacaggcggcggaacaggttgagattcaggt
taaatacgaaggttatatcgcgcgccagcaagatgagatcgaaaagcagctgcgtaacgagaacaccctgctacccgcgacactggattac
cgccaggtatccggtctttctaacgaagtgatcgccaaacttaacgatcacaaaccagcctctatcggccaagcttcgcgtatttctggcgtcac
gcctgcggccatctccattctgctggtgtggctgaaaaaacagggtatgctgcgtcgtagcgcataacgcattaaaaatgcctggtaagcaccc
gcttaccaggcaacgcatcaagaacaggtaatcaccgtgctcaacaaactctccttactgctgaaagacgcaggtatttcgcttaccgatcacc
agaaaaaccagcttattgcctacgtgaatatgctgcataaatggaacaaagcgtacaacctgacttcggtccgcgatcctaatgagatgctggt
acgccatattctcgatagcattgtggtggcaccgtatctgcaaggtgaacggtttatcgatgtcggcaccggaccaggactgccaggcattcca
ctctctatcgtgcgtcctgaagcccatttcactctgttggatagccttggtaaacgcgtgcgtttccttcgtcaggtgcaacatgagcttaaactgga
gaatattgaaccagtacagagcagggtagaagagtttccttcagagccgccatttgatggcgtaattagccgcgcttttgcctctctgaacgatat
ggtgagctggtgccaccatcttcctggtgagcaaggccgtttctacgcgctgaaagggcaaatgccggaagatgaaatcgctttgttgcccga
agaatatcaggtcgaatcagtggttaaacttcaggttccagccctggatggcgaacgtcatctggtggtgattaaagcaaataaaatttaattttta
tcaaaaaaatcataaaaaattgaccggttagactgttaacaacaaccaggttttctactgatataactggttacatttaacgccacgttcactctttt
gcatcaacaagataacgtggctttttttggtaagcagaaaataagtcattagtgaaaatatcagtctgctaaaaatcggcgctaagaaccatcat
tggctgttaaaacattattaaaaatgtcaatgggtggtttttgttgtgtaaatgtcatttattaaaacagtatctgtttttagactgaaatatcataaacttg
caaaggcatcatttgccaagtaaataaatatgctgtgcgcgaacatgcgcaatatgtgatctgaagcacgctttatcaccagtgtttacgcgttatt
tacagtttttcatgatcgaacagggttagcagaaaagtcgcaattgtatgcactggaaaaatatttaaacatttattcaccttttggctacttattgtttg
aaatcacgggggcgcaccgtataatttgaccgctttttgatgcttgactctaagccttaaagaaagttttatacgacacgcggcatacctcgaag
ggagcaggagtgaaaaacgtgatgtctgtgtcgctcgtgagtcgaaacgttgctcggaagcttctgctcgttcagttactggtggtgatagcaag
tggattgctgttcagcctcaaagacccccttctggggcgtctctgcaataagcgggggcctggcagtctttctgcctaacgttttgtttatgatatttgcc
tggcgtcaccaggcgcatacaccagcgaaaggccgggtggcctggacattcgcatttggcgaagctttcaaagttctggcgatgttggtgttac
tggtggtggcgttggcggttttaaaggcggtattcttgccgctgatcgttacgtgggttttggtgctggtggttcagatactggcaccggctgtaatta
acaacaaagggtaaaaggcatcatggcttcagaaaatatgacgccgcaggattacataggacaccacctgaataaccttcagctggacctg
cgtacattctcgctggtggatccacaaaaccccccagccaccttctggacaatcaatattgactccatgttcttctcggtggtgctgggtctgttgttc
ctggttttattccgtagcgtagccaaaaaggcgaccagcggtgtgccaggtaagtttcagaccgcgattgagctggtgatcggctttgttaatggt
agcgtgaaagacatgtaccatggcaaaagcaagctgattgctccgctggccctgacgatcttcgtctgggtattcctgatgaacctgatggattt
actgcctatcgacctgctgccgtacattgctgaacatgtactgggtctgcctgcactgcgtgtggttccgtctgcggacgtgaacgtaacgctgtct

atggcactgggcgtatttatcctgattctgttctacagcatcaaaatgaaaggcatcggcggcttcacgaaagagttgacgctgcagccgttcaa
tcactgggcgttcattcctgtcaacttaatccttgaaggggtaagcctgctgtccaaaccagtttcactcggtttgcgactgttcggtaacatgtatgc
cggtgagctgattttcattctgattgctggtctgttgccgtggtggtcacagtggatcctgaatgtgccgtgggccattttccacatcctgatcattacg
ctgcaagccttcatcttcatggttctgacgatcgtctatctgtcgatggcgtctgaagaacattaatttaccaacactactacgttttaactgaaacaa
actggagactgtcatggaaaacctgaatatggatctgctgtacatggctgccgctgtgatgatgggtctggcggcaatcggtgctgcgatcggta
tcggcatcctcgggggtaaattcctggaaggcgcagcgcgtcaacctgatctgattcctctgctgcgtactcagttctttatcgttatgggtctggtg
gatgctatcccgatgatcgctgtaggtctgggtctgtacgtgatgttcgctgtcgcgtagtaagcgttgcttttatttaaagagcaatatcagaacgtt
aactaaatagaggcattgtgctgtgaatcttaacgcaacaatcctcggccaggccatcgcgtttgtcctgttcgttctgttctgcatgaagtacgtat
ggccgccattaatggcagccatcgaaaaacgtcaaaaagaaattgctgacggccttgcttccgcagaacgagcacataaggaccttgacctt
gcaaaggccagcgcgaccgaccagctgaaaaaagcgaaagcggaagcccaggtaatcatcgagcaggcgaacaaacgccgctcgca
gattctggacgaagcgaaagctgaggcagaacaggaacgtactaaaatcgtggcccaggcgcaggcggaaattgaagccgagcgtaaa
cgtgcccgtgaagagctgcgtaagcaagttgctatcctggctgttgctggcgccgagaagatcatcgaacgttccgtggatgaagctgctaac
agcgacatcgtggataaacttgtcgctgaactgtaaggagggaggggctgatgtctgaatttattacggtagctcgcccctacgccaaagcag
cttttgactttgccgtcgaacaccaaagtgtagaacgctggcaggacatgctggcgtttgccgccgaggtaaccaaaaacgaacaaatggca
gagcttctctctggcgcgcttgcgccagaaacgctcgccgagtcgtttatcgcagtttgtggtgagcaactggacgaaaacggtcagaacctga
ttcgggttatggctgaaaatggtcgtcttaacgcgctcccggatgttctggagcagtttattcacctgcgtgccgtgagtgaggctaccgctgaggt
agacgtcatttccgctgccgcactgagtgaacaacagctcgcgaaaatttctgctgcgatggaaaaacgtctgtcacgcaaagttaagctgaa
ttgcaaaatcgataagtctgtaatggcaggcgttatcatccgagcgggtgatatggtcattgatggcagcgtacgcggtcgtcttgagcgccttgc
agacgtcttgcagtcttaaggggactggagcatgcaactgaattccaccgaaatcagcgaactgatcaagcagcgcattgctcagttcaatgtt
gtgagtgaagctcacaacgaaggtactattgtttctgtaagtgacggtgttatccgcattcacggcctggccgattgtatgcagggtgaaatgatc
tccctgccgggtaaccgttacgctatcgcactgaacctcgagcgcgactctgtaggtgcggttgttatgggtccgtacgctgaccttgccgaagg
catgaaagttaagtgtactggccgtatcctggaagttccggttggccgtggcctgctgggccgtgtggttaacactctgggtgcaccaatcgacg
gtaaaggtccgctggatcacgacggcttctctgctgtagaagcaatcgctccgggcgttatcgaacgtcagtccgtagatcagccggtacaga
ccggttataaagccgttgactccatgatcccaatcggtcgtggtcagcgtgaattgatcatcggtgaccgtcagacaggtaaaaccgcactggc
tatcgatgccatcatcaaccagcgcgattccggtatcaaatgtatctatgtcgctatcggccagaaagcgtccaccatttctaacgtggtacgtaa
actggaagagcacggcgcactggctaacaccatcgttgtggtagcaaccgcgtctgaatccgctgcactgcaatacctggcaccgtatgccg
gttgcgcaatgggcgaatacttccgtgaccgcggtgaagatgcgctgatcatttacgatgacctgtctaaacaggctgttgcttaccgtcagatct
ccctgctgctccgtcgtccgccaggacgtgaagcattcccgggcgacgttttctacctccactctcgtctgctggagcgtgctgcacgtgttaacg
ccgaatacgttgaagccttcaccaaaggtgaagtgaaagggaaaaccggttctctgaccgcactgccgattatcgaaactcaggcgggtga
cgtttctgcgttcgttccgaccaacgtaatctccattaccgatggtcagatcttcctggaaaccaacctgttcaacgccggtattcgtcctgcggtta
acccgggtatttccgtatcccgtgttggtggtgcagcacagaccaagatcatgaaaaaactgtccggtggtatccgtaccgctctggcacagtat
cgtgaactggcagcgttctctcagtttgcatccgaccttgacgatgcaacacgtaagcagcttgaccacggtcagaaagtgaccgaactgctg
aaacagaaacagtatgcgccgatgtccgttgcgcagcagtctctggttctgttcgcagcagaacgtggttacctggcggatgttgaactgtcga
aaattggcagcttcgaagccgctctgctggcttacgtcgaccgtgatcacgctccgttgatgcaagagatcaaccagaccggtggctacaacg
acgaaatcgaaggcaagctgaaaggcatcctcgattccttcaaagcaacccaatcctggtaacgtctggcggcttgccttagggcaggccgc
aaggcattgaggagaagctcatggccggcgcaaaagagatacgtagtaagatcgcaagcgtccagaacacgcaaaagatcactaaagc
gatggagatggtcgccgcttccaaaatgcgtaaatcgcaggatcgcatggcggccagccgtccttatgcagaaaccatgcgcaaagtgattg
gtcaccttgcacacggtaatctggaatataagcacccttacctggaagaccgcgacgttaaacgcgtgggctacctggtggtgtcgaccgacc
gtggtttgtgcggtggtttgaacattaacctgttcaaaaaactgctggcggaaatgaagacctggaccgacaaaggcgttcaatgcgacctcgc
aatgatcggctcgaaaggcgtgtcgttcttcaactccgtgggcggcaatgttgttgcccaggtcaccggcatgggggataacccttccctgtccg
aactgatcggtccggtaaaagtgatgttgcaggcctacgacgaaggccgtctggacaagctttacattgtcagcaacaaatttattaacaccat
gtctcaggttccgaccatcagccagctgctgccgttaccggcatcagatgatgatgatctgaaacataaatcctgggattacctgtacgaaccc
gatccgaaggcgttgctggataccctgctgcgtcgttatgtcgaatctcaggtttatcagggcgtggttgaaaacctggccagcgagcaggccg
cccgtatggtggcgatgaaagccgcgaccgacaatggcggcagcctgattaaagagctgcagttggtatacaacaaagctcgtcaggcca
gcattactcaggaactcaccgagatcgtctcgggggccgccgcggtttaaacaggttatttcgtagaggatttaagatggctactggaaagattg
tccaggtaatcggcgccgtagttgacgtcgaattccctcaggatgccgtaccgcgcgtgtacgatgctcttgaggtgcaaaatggtaatgagcg
tctggtgctggaagttcagcagcagctcggcggcggtatcgtacgtaccatcgcaatgggttcctccgacggtctgcgtcgcggtctggatgtaa
aagacctcgaacacccgattgaagtcccggtaggtaaagcgactctgggccgtatcatgaacgtactgggtgaaccggtcgacatgaaagg
cgagatcggtgaagaagagcgttgggcgattcaccgcgcagcaccttcctacgaagagctgtcaaactctcaggaactgctggaaaccggt
atcaaagttatcgacctgatgtgtccgttcgctaagggcggtaaagttggtctgttcggtggtgcgggtgtaggtaaaaccgtaaacatgatgga

gctcattcgtaacatcgcgatcgagcactccggttactctgtgtttgcgggcgtaggtgaacgtactcgtgagggtaacgacttctaccacgaaat
gaccgactccaacgttatcgacaaagtatccctggtgtatggccagatgaacgagccgccgggaaaccgtctgcgcgttgctctgaccggtct
gaccatggctgagaaattccgtgacgaaggtcgtgacgttctgctgttcgttgacaacatctatcgttacaccctggccggtacggaagtatccg
cactgctgggccgtatgccttcagcggtaggttatcagccgaccctggcggaagagatgggcgttctgcaggaacgtatcacctccaccaaa
actggttctatcacctccgtacaggcagtatacgtacctgcggatgacttgactgacccgtctccggcaaccacctttgcgcaccttgacgcaac
cgtggtactgagccgtcagatcgcgtctctgggtatctacccggccgttgacccgctggactccaccagccgtcagctggacccgctggtggtt
ggtcaggaacactacgacaccgcgcgtggcgttcagtccatcctgcaacgttatcaggaactgaaagacatcatcgccatcctgggtatggat
gaactgtctgaagaagacaaactggtggtagcgcgtgctcgtaagatccagcgcttcctgtcccagccgttcttcgtggcagaagtattcaccg
gttctccgggtaaatacgtctccctgaaagacaccatccgtggctttaaaggcatcatggaaggcgaatacgatcacctgccggagcaggcgt
tctacatggtcggttccatcgaagaagctgtggaaaaagccaaaaaactttaacgccttaatcggagggtgatatggcaatgacttaccacctg
gacgtcgtcagcgcagagcaacaaatgttctctggtctggtcgagaaaatccaggtaacgggtagcgaaggtgaactggggatctaccctgg
ccacgcaccgctgctcaccgccattaagcctggtatgattcgcatcgtgaaacagcacggtcacgaagagtttatctatctgtctggcggcattct
tgaagtgcagcctggcaacgtgaccgttctggccgacaccgcaattcgcggccaggatctcgacgaagcgcgagccatggaagcgaaac
gtaaggctgaagagcacattagcagctctcacggcgacgtagattacgctcaggcgtctgcggaactggccaaagcgatcgcgcagctgcg
cgttatcgagttgaccaaaaaagcgatgtaacaccggcttgaaaagcacaaaagccagtctggaaacaggctggcttttttttgcgcgtgtgac
ccgtcctgaatagcgttcacatagatcctgctgatataaaccccccctgttttcctgtttattcattgatcgaaataagagcaaaaacatccacctg
acgcttaaattaaggtactgccttaattttctgcagacaaaaggcgtgacgatggtcgaaaatggcgctttcgtcagcggggataatccgttattg
aacaatttatcctctgtccatttcacgatgaaaaaaatgtagtttttttcaaggtgaagcggtttaaattcgttctcaaattacagtcaggacgcgtatg
ttgaataatgctatgagcgtagtgatccttgccgcaggcaaaggcacgcgcatgtattccgatcttccgaaagtgctgcataccttgccgggaa
agcgatggttcagcatgtcattgatgctgcgaatgaattaggcgcagcgcacgttcacctggtgtacggtcacggcggcgatctgctaaaaca
ggcgctgaaagacgacaaccttaactgggtgcttcaggcagagcagctgggtacgggtcatgcaatgcagcaggccgcacctttctttgccg
atgatgaagacattttaatgctctacggcgacgtgccgctgatctctgtcgaaacactccagcgtctgcgtgatgctaaaccgcagggtggcatt
ggtctgctgacggtgaaactggatgatccgaccggttatggacgtatcacccgtgaaaacggcaaagttaccggcattgttgagcacaaagat
gccaccgacgagcagcgtcagattcaggagatcaacaccggcattctgattgccaacggcgcagatatgaaacgctggctggcgaagctg
accaacaataatgctcagggcgaatactacatcaccgacattattgcgctggcgtatcaggaagggcgtgaaatcgtcgccgttcatccgcaa
cgtttaagcgaagtagaaggcgtgaataaccgcctgcaactctcccgtctggagcgtgtttatcagtccgaacaggctgaaaaactgctgttag
caggcgttatgctgcgcgatccagcgcgttttgatctgcgtggtacgctaactcacgggcgcgatgttgaaattgatactaacgttatcatcgagg
gcaacgtgactctcggtcatcgcgtgaaaattggcaccggttgcgtgattaaaaacagcgtgattggcgatgattgcgaaatcagtccgtatac
cgttgtggaagatgcgaatctggcagcggcctgtaccattggcccgtttgcccgtttgcgtcctggtgctgagttgctggaaggtgctcacgtcggt
aacttcgttgagatgaaaaaagcgcgtctgggtaaaggctcgaaagctggtcatctgacttacctgggcgatgcggaaattggcgataacgtt
aacatcggcgcgggaaccattacctgcaactacgatggtgcgaataaatttaagaccattatcggcgacgatgtgtttgttggttccgacactca
gctggtggccccggtaacagtaggcaaaggcgcgaccattgctgcgggtacaactgtgacgcgtaatgtcggcgaaaatgcattagctatca
gccgtgtgccgcagactcagaaagaaggctggcgtcgtccggtaaagaaaaagtgattctggccggctaacccggtcacatgggatgagg
agataacataatctccctcccacaagcagtaactataaaaataaccccactctctacaaggctcggggcgcccgaaaaaacggcataca
ggttgaccgacaacgatataaatcggaatcaaaaactatgtgtggaattgttggcgcgatcgcgcaacgtgatgtagcagaaatccttcttgaa
ggtttacgtcgtctggaataccgcggatatgactctgccggtctggccgttgttgatgcagaaggtcatatgacccgcctgcgtcgcctcggtaaa
gtccagatgctggcacaggcagcggaagaacatcctctgcatggcggcactggtattgctcacactcgctgggcgacccacggtgaaccttc
agaagtgaatgcgcatccgcatgtttctgaacacattgtggtggtgcataacggcatcatcgaaaaccatgaaccgctgcgtgaagagctaaa
agcgcgtggctataccttcgtttctgaaaccgacaccgaagtgattgcccatctggtgaactgggagctgaaacaaggcgggactctgcgtga
ggccgttctgcgtgctatcccgcagctgcgtggtgcgtacggtacagtgatcatggactcccgtcacccggataccctgctggcggcacgttctg
gtagtccgctggtgattggcctggggatgggcgaaaactttatcgcttctgaccagctggcgctgttgccggtgacccgtcgctttatcttccttgaa
gagggcgatattgcggaaatcactcgccgttcggtaaacatcttcgataaaactggcgcggaagtaaaacgtcaggatatcgaatccaatctg
caatatgacgcgggcgataaaggcatttaccgtcactacatgcagaaagagatctacgaacagccgaacgcgatcaaaaacacccttacc
ggacgcatcagccacggtcaggttgatttaagcgagctgggaccgaacgccgacgaactgctgtcgaaggttgagcatattcagatcctcgc
ctgtggtacttcttataactccggtatggtttcccgctactggtttgaatcgctagcaggtattccgtgcgacgtcgaaatcgcctctgaattccgctat
cgcaaatctgccgtgcgtcgtaacagcctgatgatcaccttgtcacagtctggcgaaaccgcggataccctggctggcctgcgtctgtcgaaag
agctgggttaccttggttcactggcaatctgtaacgttccgggttcttctctggtgcgcgaatccgatctggcgctaatgaccaacgcgggtacag
aaatcggcgtggcatccactaaagcattcaccactcagttaactgtgctgttgatgctggtggcgaagctgtctcgcctgaaaggtctggatgcct
ccattgaacatgacatcgtgcatggtctgcaggcgctgccgagccgtattgagcagatgctgtctcaggacaaacgcattgaagcgctggcag
aagatttctctgacaaacatcacgcgctgttcctgggccgtggcgatcagtacccaatcgcgctggaaggcgcattgaagttgaaagagatctc

ttacattcacgctgaagcctacgctgctggcgaactgaaacacggtccgctggcgctaattgatgccgatatgccggttattgttgttgcaccgaa
caacgaattgctggaaaaactgaaatccaacattgaagaagttcgcgcgcgtggcggtcagttgtatgtcttcgccgatcaggatgcgggttttg
taagtagcgataacatgcacatcatcgagatgccgcatgtggaagaggtgattgcaccgatcttctacaccgttccgctgcagctgctggcttac
catgtcgcgctgatcaaaggcaccgacgttgaccagccgcgtaacctggcaaaatcggttacggttgagtaataaatggatgccctgcgtaa
gcggggcatttttcttcctgttatgtttttaatcaaacatcctgccaactccatgtgacaaaccgtcatcttcggctactttttctctgtcacagaatgaa
aatttttctgtcatctcttcgttattaatgtttgtaattgactgaatatcaacgcttatttaaatcagactgaagactttatctctctgtcataaaactgtcat
attccttacatataactgtcacctgtttgtcctattttgcttctcgtagccaacaaacaatgctttatgaatcctcccaggagacattatgaaagttatgc
gtaccaccgtcgcaactgttgtcgccgcgaccttatcgatgagtgctttctctgtgtttgcagaagcaagcctgacaggtgcaggtgcaaccttcc
ctgcgccggtgtatgccaaatgggctgacacttaccagaaagaaaccggtaataaagttaactaccagggtatcggttcttccggtggcgtaa
aacagattatcgctaataccgttgattttggtgcctctgacgcgccgctgtctgacgaaaaactggctcaggaaggtctgttccagttcccgaccg
tgattggcggcgtggtgctggcggttaacattccagggctgaagtctggcgaactggtgctggatggtaaaaccctcggcgacatctacctggg
caaaatcaagaagtgggatgatgaagccatcgccaaactgaatccgggtctgaaactgccttcacaaaacattgctgtagtacgccgcgca
gatggctccgggacttccttcgtcttcaccagctacctggcgaaagtgaacgaagagtggaaaaacaacgttggtactggctctaccgtaaaa
tggccgatcggtctgggcggtaaaggtaacgacggtatcgccgcgttcgttcagcgtctgccgggtgcaattggttatgttgaatatgcttacgcg
aagcagaacaacctggcgtacaccaaactgatctccgctgatggtaaaccggttagtccgaccgaagaaaacttcgctaatgcagcaaaag
gtgcagactggagcaaaaccttcgctcaggatctgaccaaccagaaaggcgaagatgcatggcctattacctctaccacgttcattctgatcc
acaaagatcagaagaaaccagaacaaggcacagaagtgctgaaattcttcgactgggcgtacaaaaccggggctaaacaggcgaacga
cctggattacgccagcctgccggatagtgtagttgaacaggttcgcgctgcgtggaagaccaatattaaagacagtagcggtaagccgctgta
ctaataaaactccaggccgggtacggtgttttacgccgcatccggcattacaaaatgactttgtaaacgcgtttaactgaagagtaacttatggct
gcaaccaagcctgcttttaacccaccgggtaaaaagggcgacataattttcagcgtgctggtaaaactggcggcgctgattgtgctattgatgtt
gggtggcattattgtctctctgatcatctcctcctggccgagcattcagaaatttggtctggctttcctatggaccaaagagtgggatgcaccgaac
gatatctacggggcgctggtgccgatctacggtacgttggtgacttcgtttatcgcgctgctgatcgccgtcccggtgagtttcggtatcgccctgtt
cctgactgagcttgcgcctggctggctgaaacgcccgctgggtatcgccattgagctgctggcagccattccaagtatcgtttacggcatgtggg
gcctgtttatctttgcgccgctgttcgccgtttactttcaggagccggtcggcaatatcatgtcgaatatcccgattgttggcgcgctgttctctggccc
cgcatttggtatcggtatcctcgcggcaggcgtgatcctcgccatcatgattattccgtacattgcggcggtaatgcgtgatgtgttcgaacaaacc
ccggtgatgatgaaagagtcggcctacggtattggctgcaccacctgggaagttatctggcgtatcgttcttccgttcaccaaaaatggtgttatc
ggcggcatcatgctggggctgggccgcgcgctcggtgaaaccatggcggtgacctttatcatcggtaacacctaccagctcgacagcgcctc
gctgtatatgccgggcaacagtatcacctctgcgctggcgaacgaatttgcggaagcggaatccggtctgcacgttgccgcactgatggaact
gggcctgatcctgtttgtgattaccttcatcgtcctcgccgcatcgaagtttatgattatgcgcctggctaagaatgaggggcacgctaatggcta
tggttgaaatgcaaaccactgcggcgctggctgaatctcgccgcaaaatgcaggcgcgtcgccgcctcaaaaaccgtattgcgctgacgctct
cgatggcgacgatggccttcggcctgttctggctgatctggattttaatgtccaccatcactcgcggtatcgacggtatgtcgctggcgctgttcact
gaaatgacgccgccgcccaatacggaaggtggtggtctggcgaacgctctggcgggtagcgggctgttaattttgtgggccacggtattcggt
acgccgctgggcattatggcggggatttatctggcggaatatggtcgtaaatcctggctggcagaagtgattcgcttcattaacgacattctgctct
ctgcgccgtcgattgtggttggtctgtttgtttacaccattgtggtggcgcagatggagcacttctccggctgggcgggcgtgattgccctggcgttg
ttgcaggtgccgattgttatccgcaccaccgagaacatgctgaaactggtgccgtacagcctgcgtgaagcggcttatgcgctgggtacaccg
aagtggaagatgatctctgcgattacgctgaaagcgtcggtgtccgggattatgaccggtatcctgctggcgattgcccgtattgctggtgaaac
cgcgccgctgctgtttaccgcgctctccaaccagttctggagcacggacatgatgcagccgatcgccaacctgccggtgacgatctttaagtttg
cgatgagcccgtttgcggaatggcagcaattggcctgggccggggtattgatcattaccctgtgcgtactgctgctgaacattctggcgcgcgttg
ttttgcgaagaataaacacggttgatattgctgacacggtttcccctcaccctaaccctctccccagaggggcgaggggaccgaccgagcgc
ctttttgactctgtacacggttaacactttgccggatgcggcgtgaacgcctgatccggcctacggtaagcctgattagcgaagtgcatcaggca
cgatgaggaaaagattgcaatgagtatggttgaaactgccccgagtaaaattcaggttcgtaatttgaacttctactacggcaaattccatgccct
gaaaaacatcaacctggatatcgctaaaaaccaggtaacggcgtttatcgggccgtccggctgcggtaaatcgacgctgctgcgtaccttcaa
caaaatgtttgaactgtacccggagcagcgtgcggaaggtgaaattctgcttgatggcgacaacatcctgaccaactctcaggatatcgcact
gctgcgtgcgaaagtgggcatggtgttccagaaaccgacgccgtttccgatgtccatctacgacaacatcgcttttggcgttcgtctgtttgagaa
gctctcccgtgccgacatggacgagcgcgtgcagtgggcattgaccaaagccgcattgtggaacgaaaccaaagataaattgcaccagag
cggttactctctctctggtggtcagcaacagcgtctgtgtattgcgcgtggtatcgccattcgcccggaagtgctgctgctcgacgaaccgtgttcg
gcgctcgaccctatctctaccgggcgtattgaagagctgatcaccgaactgaagcaggattacaccgtggtgatcgtcacccacaacatgca
gcaggctgcgcgttgttccgaccacacggcgtttatgtacctgggcgaattgattgagttcagcaacacggacgatctgttcaccaagccagcg
aagaaacaaacagaagactacatcaccggtcgttacggttgattcaggagtgcgttatggacagtctcaatcttaataaacatatttccggcca
gttcaacgccgaactggaaagtatccgcacgcaggtgatgaccatgggcggcatggtggagcagcagctttctgatgcaatcaccgcgatgc

ataaccaggacagcgatctggcgaagcgcgtcatcgaaggcgacaagaacgtcaacatgatggaagtggcgatcgatgaagcctgcgtg
cgcattatcgccaaacgtcagccgacggcgagcgacctgcgactggttatggtgatcagtaaaaccattgccgagctggagcgtattggcga
cgtggcggacaaaatctgccgtactgcgctggagaaattctcccagcagcatcagccgttgctggtaagtctggagtcgctgggccgtcatac
catccagatgctgcacgacgtgctggacgcgttcgcgcggatggacattgacgaagcggtacgtatttatcgtgaagataaaaaagtcgatca
ggaatacgaaggtattgttcgtcaactgatgacctacatgatggaagattcgcgtaccattccgagcgtacttactgcgctgttctgcgcgcgttct
atcgaacgtattggcgaccgctgccagaatatttgtgagtttatcttctactacgtgaaggggcaggatttccgtcacgtcggtggcgatgagctg
gataaactgctggcggggaaagatagcgacaaataattcaccagacaaatcccaataacttaattattgggatttgttatatataactttataaatt
cctaaaattacacaaagttaataactgcgagcatggtcatatttttatcaatagcgcattgctattttctctgcacgcaattaaattaatttccgaacct
ggatgttcgttataaaaaccattaataaatgactggattgttactgcattcgcaggcaaaacctgacataaccagagaatactggtgaagtcgg
gtttttttgtttataaaaaaggtccttgctatgaacatgcaaatcaccaaaattctcaacaataatgttgtggtggttattgatgatcaacagcgggaa
aaagtcgtcatggggcgcggaattggctttcaaaaacgcgctggcgaaagaattaactcaagtggaatagaaaaagagtatgccttgagca
gtcatgaactgaacgggcgattaagcgaactcttaagtcatattcctcttgaggtgatggcaacctgtgatcgtattatctctttagcgcaggagcg
cttgggaaaattacaggacagtatttatatctcgctaactgaccattgccagtttgcgattaaacgctttcagcaaaacgtgttgctgcccaacccg
ttgctgtgggatatccagcggctttacccgaaagagttccagctaggggaagaagcgttaaccattattgataaacggttgggcgtgcagttacc
gaaagatgaagtgggctttattgccatgcatctggtcagtgcccaaatgagcggaaatatggaggatgttgcaggtgtcacgcagttaatgcgc
gaaatgctgcaattaataaaatttcagttcagccttaattaccaggaagaaagcttgagttatcagcgactggttacacatctgaagtttttatcctg
gcgtattcttgaacatgcttcaattaacgatagtgatgaatcattacaacaagcagtaaaacaaaattacccgcaagcatggcaatgtgcgga
acggatcgccatttttattggtttgcagtatcaacgtaaaatttcacccgcagagattatgtttttagccataaatatagagcgcgtgcgcaaagaa
cactgaaatattattactgagtaaaggattgttaccgcactaagcgggcaaaacctgaaaaaaattgcttgattcacgtcaggccgtttttttcag
gtttttttttggagttttgccgcaaagcggtagagggcaagttatgacggagttagccagaaaaatagtcgcaggagtcgggggcgcagataac
attgtgagtctgatgcattgcgcaacgcgattacgttttaaattaaaggatgaaagcaaagcgcaagcagaggtactgaaaaagacccccgg
tattattatggtggtggaaagcggtggccagtttcaggtggtcataggtaaccatgtggccgatgtcttcctggcggttaacagtgtggcaggcctt
gacgaaaaagcgcaacaggcaccggaaaatgatgataaaggtaatctgctaaaccgctttgtttatgttatttcaggtatttttacgcctctgatc
ggtttgatggcggcaaccgggatcttgaaaggtatgctggctctggcgctcacttttcagtggacgaccgaacaaagtggtacttatttaattttatt
cagcgccagtgatgccttgttttggttcttcccgataatcctgggatacaccgcggggaaacgcttcggcggtaatccatttactgccatggtgatt
ggtggagcgttagtgcatccattaattctgactgctttcgagaacgggcaaaaagcggatgcgctggggctggatttcctgggtattccggtcac
attgttgaattactcgtcatcggttattcccattatttttctgcctggttgtgcagcattctggaacgccgacttaatgcgtggttaccgtcggcaatca
aaaatttcttcacaccattgctatgtctgatggttatcacacccgtcacctttctgctggtggggccgctatcaacctggataagcgaactgattgcc
gccggttatctctggctttatcaggcggttcctgcatttgcgggcgcggtaatgggcggcttctggcaaatcttcgtcatgttcggactgcactgggg
cctggtgccgctgtgtatcaataacttcaccgtgctgggctacgacaccatgatcccgctgttaatgcccgccattatggcgcaggtcggggcgg
cgctcggcgtcttcctctgcgaacgcgatgcgcagaaaaaagtggtggcgggatcagcggcgttgacgagtctgtttggtatcaccgaaccag
cggtatatggcgtcaacctgccgcgtaagtacccctttgttatcgcctgtatcagtggggctttgggggccaccattattggctacgcgcaaacga
aagtctactcctttggtttgccaagtattttcaccttcatgcaaaccatcccgtcaacgggaattgatttcaccgtctgggccagcgttattggcggtg
tcattgccatcggttgcgcatttgtcggtacggtgatgcttcatttcatcaccgctaaacgtcagccagcgcagggtgccccgcaagagaaaac
accagaggttattacaccacctgagcagggcggtatctgttcaccgatgacgggagagattgtgccgctcattcacgtcgctgataccacgtttg
ccagtggcctgttgggtaaaggtattgccattctgccctcggttggtgaagtgcgttctccggttgcgggtcgaattgcttcgttgttcgccacattac
acgccattggcattgagtcagatgatggtgtggagatcctgattcatgtcggtatcgacaccgtaaaactggacggcaaattcttttccgctcacg
tcaacgtgggtgacaaggtcaatacaggcgatcggctgatttcttttgatatccctgctattcgcgaggccggatttgatctgacgacgccggtatt
aatcagtaatagcgatgattttacggacgtattaccccacggcacggcgcagataagcgcaggtgaaccgctgttatccatcattcgctaacga
taaaaggagttaattatgaaagcatttccagaaacatttctttggggtggcgcaacagctgccaatcaggtggaaggtgcctggcaggaagat
ggcaaagggatctcgacctcagatttacagcctcatggcgtaatgggaaaaatggaaccgcgcatcctggggaaagagaatatcaaagat
gtcgccatcgatttttatcaccgttacccggaagatatcgcgttatttgccgagatgggcttcacctgtctgcgtatttccattgcctgggcgcgaattt
tccctcagggcgacgaagtcgaaccgaatgaagcggggttagcgttttacgatcggctgtttgatgaaatggcgcaggcggggatcaagccg
ctggtaacgttatcccattacgaaatgccatatgggctggtgaaaaactacggcggttgggctaatcgagcggtcatcgatcacttcgagcatta
cgcccgcacggtctttactcgctaccaacataaagtggcgttatggctgacgtttaatgaaatcaacatgtcgttacacgcgccattcacgggcg
tggggctggcagaagagagtggcgaggcggaagtttatcaggctatccaccatcaactggttgccagtgcgcgggcagttaaagcctgtcat
agcctgctccccgaagcgaaaatcggcaatatgcttctcggtgggctggtttacccccctcacctgccagccacaggatatgttgcaggccatgg
aagagaaccggcgctggatgttctttggtgatgttcaggcgcgtggccagtatcccggctatatgcagcgtttcttccgcgaccacaatatcacc
attgagatgactgaaagtgacgcagaagatttaaaacataccgtcgatttcatctcttttagttattacatgactggttgtgtttcccacgacgaaag
cattaataaaaatgcgcagggcaacatactgaatatgatccccaatccgcatctgaaaagttcagagtgggggtggcaaattgatccggttgg

attacgggttctgttaaatacgctttgggatcgttatcaaaaaccgttatttattgtcgagaacggattaggcgcaaaagacagcgttgaagcgga
tggttcgatacaggacgattatcgaattgcctatttaaacgatcacctggtacaggtaaatgaagcgattgccgatggtgtggatattatggggta
caccagttgggggccaattgatttagtcagtgcatctcattcacaaatgtctaagcgctacggctttatttatgtggatcgtgatgataatggcgaa
ggaagcctcacaagaacacgcaagaaaagcttcggatggtatgcagaggtgatcaagacgcgggggctgtcattaaaaaaaaataaccatt
aaagcaccttaattatcgtcgcattcagaacagtctggatgcgatgcgttaattctttctttgcaccataaagggatattatgtttagacgaaatctta
ttacctctgccatcttattaatggcaccgttagccttttctgcacaatcattggctgaatcattaacggtggaacaacgccttgagttattagaaaag
gcgttaagagaaacgcaaagcgaactcaaaaagtataaagatgaagagaagaaaaagtatacgccagcgacggtgaatcgtagcgtaa
gtacgaatgatcaagggtatgccgccaatccgttcccgaccagtagtgccgcaaaacctgatgctgtactggtcaaaaatgaagagaaaaat
gccagtgagacaggctcgatttattcttccatgactctgaaagatttcagtaaatttgtgaaagatgaaattggctttagttacaacggctattaccg
ttctggttgggggaccgcctctcatggttcacctaaatcatgggcgattggttctctgggccgctttggtaacgaatactccggctggtttgattgca
gttaaaacaacgtgtctacaacgaaaacggcaaacgggttgatgccgttgtgatgatggatggtaacgttggtcagcagtactctaccggctg
gtttggcgataacgccggtggcgagaactatatgcagttctccgatatgtacgttaccaccaaaggtttcctgccctttgcgccagaggctgatttc
tgggtgggtaaacacggtgcgccgaaaattgaaatccagatgcttgactggaaaacgcagcgtactgatgccgcagcgggtgtaggtctgg
aaaactggaaagtcggtccgggtaaaattgatatcgcgctggttcgcgaagatgtcgatgattacgatcgcagcctgcaaaacaaacagcag
attaataccaataccattgatttacgctataaagatatcccgttatgggataaagccaccttaatggtaagtggtcgttatgtcacggcaaacgaa
agcgcatcggaaaaagataatcaggataataacgggtattatgactggaaagatacctggatgtttggcacatctttaacgcagaaatttgata
aaggtggcttcaacgaattctccttcctggtcgcgaataactctatcgccagtaactttggccgttatgctggcgcaagtccatttaccacctttaat
ggtcgttattatggtgatcacaccggcggaacagcggtacgtctgacttcgcagggcgaagcctatattggcgatcatttcattgtagctaacgc
gattgtttactccttcggtaacgatatatatagctacgaaacaggcgcccactctgatttcgaatctattcgtgcggttgttcgcccggcctatatttgg
gaccaatataaccagacaggtgttgaactgggctatttcacccagcaaaacaaagatgcgaatagtaataaatttaatgagtctggttataaaa
ccacgctcttccatacctttaaagtcaataccagtatgttgacctcgcgtccggaaattcgtttctacgccacgtatatcaaagccctggaaaacg
aactggatggcttcacctttgaagacaataaagacgaccagtttgctgtcggtgcccaggctgaaatctggtggtaatggaacttaatgaaagg
aatattacgatgaatataaaaattgctgctttaacgctggctattgccagcggtatttctgctcagtgggccatagcagcggatatgccagccagc
ccggcacccactatcccggtgaaacagtatgtgactcaggtcaatgccgataacagcgtgacctttcgctactttgcccctggggcaaaaaat
gtctctgtagtggtgggtgttccggttccggacaatattcacccgatgaccaaagacgaagcaggagtctggtcgtggcgcacacctatcctga
aaggcaacctgtacgagtatttttcaatgttgatggtgtacgcagcattgatacaggcaccgcaatgactaatcctcagcgccaggttaactcc
agtatgattctggtgccaggcagttatctggatacgcgttctgttgcgcatggtgatttgatcgccataacttaccactccaacgcattgcaatctga
acgtcagatgtatgtctggaccccgccaggatacaccggcatgggcgagcctttgccagtgctctatttctatcacggctttggtgataccggac
gttccgctatcgatcaggggcgtatcccgcaaatcatggataacctgcttgctgaagggaaaattaaaccgatgctggtggtgatcccggatac
agaaaccgatgcgaagggcattattcccgaagatttcgtgcctcaggaaagacgtaaagtcttttatccgctgaatgctaaagcggcagatcg
cgaactgatgaacgatattatcccgctgattagcaagcgttttaatgtccgtaaagatgccgatggccgcgcgctggcagggctttcacaaggc
gggtaccaggcgctggtttccggaatgaatcatctggaaagctttggctggctggccacattcagtggtgttaccacgacaaccgtaccggatg
aaggtgtcgcggcccgactgaacgatccggcagctatcaaccagcaactacgtaattttactgtggttgtggggataaagatgtcgtaaccg
gcaaggatatcgccgggctgaaaactgagcttgagcagaaaaaaattaactttgattaccaggaatatccgggtctgaaccatgaaatggat
gtctggcgaccagcctatgcagcctttgtacagaaattatttaaataaaataaggcattaagatgaaattaatcattaccgaagattaccaggaa
atgagccgtgtcgcggcacaccatctgcttggttatatgtcgaagacgcgtcgtgttaacctggcaattaccgccggtagcacgcccaaaggc
atgtatgaatacctcaccaccctggttaaaggtaagccctggtacgataactgctatttctataatttgatgaaattccatttcgcggcaaagagg
gagaaggcgtaacgattaccaatctgcgtaatctgtttttcacccctgcggggatcaaagaagagaatatccagaagctcactattgataacta
ccgcgagcatgatcagaaactggcgcgtgaaggcggactggatttggtggtgctgggattaggtgcagatggtcatttttgtggcaatttgccga
atacgacccatttccatgagcaaaccgtggagttcccgatccagggagagatggttgatattgtggcgcatggcgagttgggcggcgacttttc
gttagtgcctgacagctacgtgacgatggggccgaaaagcatcatggcggcgaaaaacttgctgataattgtcagcggcgcgggtaaagcg
caggcactgaaaaatgttctgcaaggtccggttaccgaagatgttccagcttccgtgttgcagctgcatccttcgctgatggtaatcgctgataaa
gccgctgccgcagaattagcactcggttaagcttaagaaataaggccggatgtgtgaattacgctccggcctgttctcattatttaaataactacg
aaaaatcaccccacagtctcagcttgccgcagtggagacagcggaagagataaccctgacaatgaccacccttttgcaggcatttagctaga
tcagaatttctaataccgaaattctcacaatcttcttcaaggttggcaaattcatcgaggcgatcttttatatcattccagcccacatagccgataaa
aacacagaaatcgccacaatgcgcgagccagaattcctgctgccatccatgataacctggcgtgcgttcaaccaactctttcagcatttcatca
ggatatgtgttcttaataccggcaaattcgtcctcttcatcatactcaaattcaacaccttctatgctggcatcatcctgaaaactacctgcaaactttt
cagcagcagaaccgtccgcaatacaccacggacagagatgttcaacttcatcaacgcaataaaagggacccgagtaataaactgacgtct
gttgttcacagcaatcgcactctacggttttatcctgttcaaatgcgcctgtttccagtggcttgggatgatatttgaattggggtaacggcctgatattt
tgagtcatagaaacatcctgtctgcttttgagtagcccaaagataaagtatcccgcttaacttaaagagtctgtttcaggacatgaaatatccaatc

cggtagacgggtaatgattctgctcatcaccacacctgccagcaatgcaggaatgatgcggacaacgagactgtcaccagaaagaatgttt
tcaaacaactccgaggaagccatgcccttaatatgtacaataactgcacaatagatctcggtgataacgacgccacctatgccaccagccag
acaacgataacgtttctgtgacccgatcttttccggcagacaggcaaccattacaccggttaacaacgcaggaatagctcccgtagttatcgac
aataagagaaaaattggatgtattatcacccaaagaaccagcggttcttttgcgaagaatatgtagaggacaagaaacagaacaccaattaa
cggtcctaaaactgcaaaataaagtccgtgatgtattacccgacgtgaaacagacatatcaaactccctttacttacctttgagcgccaatttaag
cggggtaagtgaagggagtgtgaagaactatgccgtaatatcccaaccacgcgctttccacagttcaggtaactgcgaaagatgggtaaag
gtggtgactttcgggtgaacgatcggcttattgtgcgggtcggcgcagaagtagaacacttccatacctgcgtcgataccagattgtgcaccgg
caactgagtcatcaaccagaatgcagttttctacatttacattcatcgcttttgccgcatggaacattaacgccgggtctggcttccagcgctgaat
atcgtagccgctgaacagtttatccgggaagtagtgcaacatattcagcttgcccatagaatgctgcattttgttatttggccgttagataccaca
cacattggcgcagtgatcgctgacaggagcgctccagccccttcgatggcttccagttctgaatcgaacagccgagcgacttctgcacggtaa
acgtgttcagcttctgttttcgctaacgtaacaccatgttcaagggaaacaatatcgataatttcgtacagttttacacctttgaaacgtttgaatacct
cttcaggatcgagcgtaataccaaattcctgaaacatcgttacatatgcgcgagagcaaatgacttcactgtcgaccagcgtaccgtcgcagtc
gaaaaataccgcttctatccgggacatgcctttcctttttaatcagtttaacgttttcgtagagccgatttgcgcaacgcaatcgttgccgtataagta
aatccaataaaaaagtctctcacaaacgcgcctattgtcgcattttggtatacgatagcgacggattttccctccttgttcggaaatagataatgag
tcatcaacataccacccagacttctggtcaggggatgctggaacgcgtgtttaaactgcgcgaacatggcacgacggcacggaccgaagtg
atcgccggttttaccaccttcctgacgatggtttacatcgtttttgttaacccgcaaattcttggcgttgctggcatggataccagcgccgtcttcgtca
ctacctgtctgatcgctgcattcggcagtattatgatgggactgtttgctaacctgccagttgcactggcacccgctatgggcctgaatgcgttcttc
gcttttgtcgttgtacaggcgatgggcttgccgtggcaggtcgggatgggcgcaatcttctggggcgcgataggtctgctgttactgacgattttcc
gcgttcgctactggatgatagccaacattccggtgagtctgcgtgtgggtattaccagcggtatcggtctgttcattggcatgatggggctgaaaa
acgcaggtgtgattgtcgctaacccggaaacgctggtgagcatcggtaatctgacttctcacagcgtacttctgggtatcctcggcttcttcatcatt
gctattctggcctcgcgcaacattcacgcagcggtgctggtttctatcgtggtgacgacgctgctgggctggatgctgggtgatgtgcactacaat
ggcatcgtttctgcgccgccgagcgtaatgacagttgtgggtcatgtagatttagccgggtcgtttaacctcgggctggcaggggtgattttctcttt
catgttggtcaacttgtttgactcctccggtacgctgattggcgtgaccgataaagcaggtctggcggatgagaaggggaaattcccgcgcatg
aagcaggcgctgtatgtcgacagtatctcttccgtgaccggttcgtttatcggtacttcttccgttacggcttatattgagtcctcttccggcgtatcggt
tggcggtcgtaccggtctgacggcagtggttgttggtctgctgttcctgctggttatctttctgtcgccgctggcggggatggtgccaggctacgctg
cagctggtgcgctgatttacgttggcgtgctgatgacttccagtctggcacgcgtgaactggcaggatcttactgaatctgttccggcgtttattacc
gccgtgatgatgccgttcagcttctcgattaccgaaggtatcgcgctgggctttatctcctactgcgtgatgaaaatcggtaccggacgtctgcgtg
atctaagcccgtgcgtaatcatcgttgcgctgctgtttatcctgaagattgtctttatcgacgctcactaaaatcgatagcgcgttaaaaacccgcc
atgcatgattgctggcgggtttttatttagatcttaactcgctgaataaactcaccaaatgcggtcaattgcccggtcaggtggtccagcgtaccct
gatcaatcacttctccggtttgcggatcaactttgttctgaatcacgccgcccataaattccggcttgttcatcaccattgcatcgaggaaaaccag
aatctggcgcaggtgatactgacagcgcgcgccgccaatcacgcccattgagctggtctgaatcaataccggtttaccggccagcggttgatc
cggcaggcgggaaagccagtcgatggcatttttcagcccacccggtaccgagtagttatattccggcgtgacgatcaccacaccgtcagcctg
acggatctgttccgccagagcttcaaccgttgctggaaaaccttcttcctgctgtacgtcagcgtcatacaagggaatgtcggcaatggatggta
acgcattgacttccatgctcgccggagcaattttcggcagggtacgtgcaaccatgccattaaatgagcctttgcgcaggctccccagtaacgt
aaccacctgcaatttttcagacatgatgactcctgtggttgtctatcaatttggcgaaaaagccttcacggtagagagttgggcaaaacgcatcat
ccggctggtaggattgttggcgcggctgtgaatatccggcaggcttttccagccgtgtttgccatctaactcccagacactgagcttttcaatcgtg
ggcgtgaccgccacggaccacaccagcacgtcttccgcgtcgcataacgcttctacatgatttacatgagcacatttcaggcgtccggcaatg
ggcagcagctgcagatcgcgcggatcgtcattcaaaacatcaccggttagttttagcacgcttcggcgcagcgtgatgagctgcgcccgcgct
tcgttaggatcgttttgtttactgatccatagcgattcattgctggaaaaggtgtggttatcgggcgcgtgtgggctatgaaacccgcgcgtcgcac
gctgtagttccatatcgaggccacattcaccttcggttgttaacgccacgccaaccatattcccggcataggaaatggaaaacgaaggcaaatt
tttatcactgaaaaccggtttacctttcggcagggtgacgatttccggcaattcgccaatgccatacagcatgaacatcagttctgcgagtaaacc
tctggacgccagaaaacggctttgacgatgagggggaatgtttcgggcttcttgatgacactgggaggggagacgaacagaaatcagatgtc
cttccgttaaaatccctcgggcaaagtgagtcgccatttttcgctccatgcttaatggtccattatgaatacaagagtaatcatttactggaatgatg
gacagactaaagcggctaaaagttcgattcagacaagcttattacaactttttttacattttattgtgaaattgggtcgcgtttcatacgattcggttatg
ccatcgacgcgtaaaggtttttaatggtttcccgtaaccagacgatcttcggattatggctgttccgtttatgccacagcagggtaaaaggaacttc
cagctttttttgctggctttcgtcaaacgggagaggaagagcaaccaacggcagttgatggagttgattgtagtactgacagtagcgcggcgcg
gtcgccagtagcagattgtcgggttgcgctgccataaacagtgactgctcgaattccggcaggctcatagcaatcgtgcgttcgcgtcccagct
cctgtaacacattgtccagcgcccaggtatcgctctgttcccagcaaatgctgatatgcggataacgtaagaaggtgtccagattccacgtttgat
gcagtgccggatgatctttgcgtaaccagacgcagggcacatcactaaacagcacttcataatcaatggctaacggtagcgagcttaacagct
cccgcgagcgaggatggctttcgcgaccggaaaaaccgatatccacttcaccacgagtaatggcatctaaggaatcgtaatcccagttacgt

aatttgatggtcgcctgcgggtaacgttggtagatctgtttcgacagcgcattaagcatgatcatcatcagcggtgattccgccgccagctcaaac
ttcaggccgcgcggtgtctggtggtgcggtttatccagcagcaggttgctcatttgcatccactccgccagattttgctccatgctgaccatcagcg
gtgtgggcgacagacccagcggtgagttcacaaagagcgggtcgtcaaaccacgctcttaactttgccagcgacttactcaccgccgaaggt
gtcacgtttatccgcttcgccgctttggttacgctgcgctcctgcatcagcagttgcagacaaagcagcagattgagatcaagcgtggtgatggat
ttcttcatgagcggcaacggggcgtccaggcgcgacgaacataatcagcaacaggctcactatgctacaggcaatcagaatcccgatcagc
atattccatgcgccgataccaaccaccgctgccagccaaatccacagtgacgaaccgcaaacctgcgcaatacctaaggtcgagctggcta
cgcccgcgcgtaatgaaaacggccctaacgcctgactcatcgccacaccaaaacctaccgagaaaccggcgcaaatcagcgtgatacca
aacagagaaaccgcatgggaaggtgaaacggcaagagtgatccccgccgccaggaataacacctgcgaggtgatcatcaacgtacgtg
gcttaaaaattcccagcgcaaatggcgtggagaatgaaacggtcatgctgacgccagcggtcagcgccataatggtggcgtattcaccgcgc
tcaaaccccatgatttccatcagcaataccggtgacgtgttgacgaaagtgaggatcaccgaaacgctgagggtggtgataacaacacggct
gaggaaaaaacggttaagcagcgactcgctattttctcgtggtttatccgaagctgcgggggccgctgggcgcgtttcttttaaaataaacaaag
acaacatcagtaccgcgatgcccatcattgccatcgcccagaacagactctgccacgggaatttaagcataatcagatgtccgagcactggc
gctaacaccggaatgatgcaggtaataccgttgagtaatgacagcactttagcccgacgtcgatcatccagcgtgtcgcgcaaaatagcgaa
cgccactacgtaacaacagcctgcgcccaacccctgtagaaatcggcctgcaagaaataacgtgctggtttcagccagtgaacagaacacc
gaggcaataataaatagcgccgcgccgggtatggcgaccggctttctccctgaacgatcggccactttaccggcaaataacatcgcagctgc
catccccgccagatatacggagaacgcaatatgcaactgcgcttcgctggcattgagatcggcggcgatgcgcggtaaaccaacgaggtac
atatcaatcccggcgggataaagtaaaaccagggcaaaactacaaatcaaaaagcgggacatgggctaaagcgtcctgatagtaaggtg
acgcaaagcatacgtggtgaaggaggatcaggcgagttgccatttggacataagtgatttcctgagaggcaagaagccagcgaatggctgg
cttcttgaaggatttagccaaatttaggtaacacgttaaagacgttgccgaaccagcacaaaatcacggttataccaaagagaatgacaatcg
ccggaataagattgccgccccagaccgtgaacatctgattgggaaacttcttgcgagctttgattgcaagcactgcgggaataatgaccgccc
agatggtggcgcacagcccggcaccgccgatcccgtaaataaagccgttcgggaagatcagatacaacaacgcaggtggcaggaaggtt
aacagcacggttttgaaacgcccgccgtgggagttatcaatcttaaacaggtccgccagataatcgaacaaccccagcgtgacaccaaaga
acgaactggcaacagctaagttagagaacaccagcaggcaaaactcgataatgccgtgctgtttggtgccgaggaacgatttcaccagcga
atcaacgttgccgcctgaggagataatcgccttaaagctttctcgcggaatattccccatggtgcaatagagccagaagagataaatcaccag
cgccagcagcgaaccaaataccacgcttttgattaacttatctttgcgttttccatagcaaataatcaggctgggaatattgccgtggaaaccaa
atgacgccagacacaccggcaaagccataaagatatacgggaagtaagacgttcccgcagtggagctggtggcgtcgcgcagaatggag
taatcgacctggaagaaaaaagaaccaaacacgatcacaaaggagataatcttcagcccgaggaacaatgaggtaatacgactggcggc
taacgaacttaaccacaatacgctggcaacgaaaatggctgtgcagatcccgacaatacgtggattagcgtgataacccaaattcattgatat
cgtttcactaatgatcgcaccattagcagagatataggcataagtgaggatatagagaacgaaggcaacggtaataccgctgataatgttcca
ggtgttaccgattaaatctttggtgatggtgttaaaactggagccgacgggataatttaaatttgcttctaataacaataacccggaatgaagcatt
gaaaaccaggcaatgataaggataaaggcaccccagaaaaaccaggcaccggcaagatcaacaggtaaagcaaacatacctccacca
attactgtacctgctataaccataacaccccaaaatgcagagtgctttttttcagcttgatcagtcatgatgccacctttagaggaaggctatttttgtt
attgaggatgtagggtaagagagtggctaacatccttatagccactctgtagtattaattaaacttctttaagttttgcggtgaagtgacgcaatactt
tcggttcgtacgtaaaggttaatcctttaatattcgccgcgttctctttcacatgtttaaaggcttcaataatgaagtccatatgtgtttgagtatatgttg
cgcgcggaatggttaaacgcagcagttcagccgggcatggcagttgtttaccggttttcggatcgcggcctaacaggaaagagccaatttcta
ccgcacggataccggcgactttatacagctcgcaggccagcgcctgtgccgggaactggtctgccgggatatgcggcaacagtttaccggca
tcaacgaatgccgcgtgaccgcccgcctgctggcagacaacgccaatctcttccagaccatcgaccagatactgtacctgcgcgatacgata
agccagccagtcgagattcatgccgtcatacagacctaccgccagacgctccatcgcgccgccttccaggccgccatatgtcgggaagcctt
cctgcaccacgcaaagggttctgcactcggtgtacacatcaaagaagctgtcgtctttcatgcacagcaggccgcccatcggcaccatcgcat
ctttcttggcggacatcgccagcatatcggcatatttgtaggtttcgcgggtgatctgctcgatggtccagtctttgtattctgcttcacgctgcttgatg
aaataggcgttttcagcaaagcgcgcggagtccattaccaccggaatatcgtatttcttcgcgatgctgtacatcgcttttaagtttgccagtgaaa
ccggctgaccacctgcagagttactggtgatggttgcaacgatatacggcacgttattcggaccaacttcttcaataccgcgttctaatccctcaa
ggtcaaagttgcctttaaagtcgtaacgcacgcccgtatcgaaggcttctttgatatagacgttacgcacggtacagccgttgatctggctatggc
cctgcgtggtatcaaagaaatagttagagaacgccaccattttgctgcgatccaggcctttttcctgctcgcgtttttttaatcagtaccggaatatag
atttgctctgcgccacggccctggtgagtcggaatggtgtattgataaccaaagatatttttcactgactcggctaacgcatagtagctacgactg
ccgctgtaggcttcgtcgccgcgcatcatcgcagcctgcatgctctgcgtcaccgccccggtgccgctgtcggtcagtaaatcgataaaaacat
cttcgctatccagcaggaacgggttcataccggatttaataattgcctcttcacgataagcgcgagtggtacgttttactggctcaataacacgaa
tgcggaacggttcagggagatgtttaaagttttccattacataatccttcatttattttaattacagtgatccctgtgaatattacatctgctataccgatt
aattcgccagatatatatgcaagcaccatcatttactaaaacaagtaaataaacgagagatgacctttgcaaaaggcaaaattaagatatagc
aaacaaatagatcacattgaatctaatcggtttggctctggtgatggctacagaagggcaaatcaagggcggtgatcgacaattttgttgtcaat

attgaaccattttgaggtcacacatatatgtaagatattcataatgcacttatcctcgcaagacacgaggctacattattacactcagagtttttgtct
attctgaaattgtttaaatgtgaatcgaatcacaatcgttcggggagcaatattaaatgctttttttaaagattataaattacgtaagaattttaatgttta
aaaaaagggttgaggaataacaggagtggtaacaatgaagcgaagccgcatctgacagtcagaatgcggcttcgtaagcgcggttacttac
caatacagaagctggagaaaatccgccccagcaggtcgtctgaagtaaattccccggtgatttcgcttaagttctgctgtgccagacgcaactc
ttccgccagcagttcacctgcccaggctcccaacagttgcgctttgccctgttgtagatgttccgctgcctgttccagcgcctgtaggtggcgacg
acgcgccaggaagccgccttccatgttggtgtcaaagcccatgctctgtttgagatggttacgcagcacgtccacgccttcaccagtccttgccg
agagacgaattaacgcgtgaccgttcacttcactcattcccagcgtttcgccggtgatatcggctttattgcgcaccacggtgatcggcagtttcg
ctggcagacgggcaataaattccggccagatctctgccggatccacggcgtctgttgtggtgccatcgaccataaacagcacgcggtcggcct
gttcaatttcctgccacgcgcgctcgataccaatacgttctacttcgtcactggcttcacgtagcccggcggtatcgatgatatgcagcggcattcc
gtcaatgtggatatgctcacgcagcacgtcacgcgtagttccggcgatatcggttacgattgccgcttcacgccccgccagcgcgtttaacagg
ctcgatttaccggcgttaggacgtccggcaatcaccactttcatcccttcgcgcaacaaactaccctgacgtgcttcagcacgcactgcatcaag
atcggcaataacgtcattgagctgggcttcaatttttccgtcggagaggaaatcgatctcttcatcggggaaatcaattgccgcttcgacgtaaat
gcgcaagtgggtgagggcttctaccagatgattaacccgtgcggagaatgcgccttgcagcgagttaagtgccgaacggccgcctgttccg
aactggcgtcgataagatcggcaatcgcctcggcctgggctaagtcaagtttatcgttaagaaacgcgcgttcggaaaactcaccagggcga
gcaatccgcaggccgggaatggtcagaatgcgttttaacagcaggtcgaggatcaccggaccgccatgaccttgcagttccagcacatcttc
gccggtgaacgagttcgggccagggaaccatagcgcaatcccctgatcgagcacgctgccgtcggcgtctttaaacggaagataatcggcg
tagcgcggcttaggcagtttacccagcacggtttcggcaacttcacgggctttgaagccggagatgcgcaggatgccaacgccgccacgtcc
cggaggcgtggcctgggctacgatagtgtcattatcgctcatgatgttcctgttgctttgtgtggcggatgcgcggtgcttatccgccctacgaaaa
gaaaaaaggcggtcaactgaccgcccttattttagcgaaaactcaccgaatcaggatttttttcttctcgcggctatgcaggccacgtttttccaga
ccacggtaaatcagctgctgctgaataatggttaccaggttgctgacgatatagtacagcaccagacctgacgggaaccacaggaagaaca
cggtgaagatgaccggcataaaggtcatgatcttctgctgcatcgggtcggtcactgtggtcggcgacatcttctgaatgaagaacatcgttacg
cccatcaggatcggcaggatgtagtacgggtcctgtgccgacaggtcgtggatccacagtgcaaacggtgcctgacgcagttcaacggaac
ccatcagcatgtagtacaacgccaggaagattggcatctggatcagcagcgggaagcagccgcccagcgggttaaccttctcagctttgtac
agcgccatcatttcctggctgatacgctgtttgtcatcgcccagacgctcacgcattgcctgaatcttcggctgcaacatacgcatcttcgccatgg
aggtgtactgcgctttggtcagcgggtacatgatgccacgaacgataaaggtgatgatgataatggagaagccccagttacccacaaagctat
ggatccatttcagcagtttgaacagcggctgagagatgaaccacaaccaaccgtaatcaacggtcagatccaggtgcggagcaacagctgc
cattttgtcctggatttccgggccaacccacagggtgctgttcatcgcgccagtctgaccaggctgaaccagtaccggctgagatttatagccga
tagcggcgatgccgttacccagattagcggtatagaagttgttggtaccgtcgttatgcgggatccacgccgtcgcgaaatactgttgcagcatc
gccacccaaccacctttcgaagagatgttcaggttttcgttatcggcaatggtatcgaacttgtatttctcatacttctcgtcaggcgtggagtacgc
cgcgccacggaaggtgtgcagtgcgaagttgctgcttccggtatcgagatgcggtggcagagtgatggattgcttcaactgaccaaacgagg
agatttccagcggtttctcgccagcgttctgcacgttgtagttgacgttgacagcgtaatcaccacgtttcaggacaaacgttttggtaaacgtgttg
cctgccgcgtcggtatacgtcatcggcacctgcagttcgttttgaccttcagccagcacataagcgtctttttcaacgttatacagcggacgcggg
ccgttagccgggttatccgggccatcacgaccggtcagaccgctctgtgcctgataaataaactgcggtgaagtttccaacagctggaacggc
tgggtagagttcagctctttcgggtaagcaggcagcagagcttgctcaacatcaccaccacgggtgttgatggtcagatcaagcacgtcggtct
taaccgagatcagtttcccctggccactggccggtacgccctggtcggcggcgctacccgctgcggtggtcgttgtctgcgtggtctgttgggcct
gaggttgcgggtttttatcctgctcccaggcttgccagatcatgaaagacacgaacagcaaagcgatgactaaaagattgcgttgcgaatccat
cgttagtgttctctggtatcaaatggtccgggcgggacgggatcgtcaccaccagggtgtaaagggtggcattttaatacgcgtttcaccgtcaa
ccaactgccttttatcactccaaacctgcgcaatgcctcaattccgtagcttgaacaggttggagtgaaacgacaatgcggcccgagtagcgg
actaatcaggcgttgatagacccgaatgagggctatcaggacccgcgagccaggcgacagtggcggcgccataatttttccaacgcttccga
gagagcacggttatcgaggtcggcaacccctttttcgccaccaccacgaaatccatagccgggagttcatgttggcgcagacggaagctttc
acgcgtcagacgtttaatccgattgcgttcatgggcgcgtcgaacgtttttcttggcgactgtaagaccgatacggggatgccccagcgaattca
ggcggccgagaatggtaatttgcggcgtgccagcccgttgtggctgctggaagacgaatgtgaattgactgggagttaacaagcgtaactccc
tgggaaatgcgagcttaaccactcaggggttagctttattacttagaaacggtcagacgagcgcggcctttagcacgacgacgtgccagaac
ctgacgaccatttttagtagccatacgagcacggaagccgtgagaacggttgcgcttcagtacagacggttgaaaagtgcgtttcatggcgattt
ctacctaaacttgaataaattcaatggctttattggatatccgccgaaaaatgaaacgatggacaccgaagccatgggtgattaaagaggccg
gattgtaataattgtacactccggagtcaattctctttccttatttaccgcgcttttccgcaccttttcgcagggaaaatgtacgacctcacaccagtg
gaaaccagcatggcgcgccgggtggaggattatacgggctgatgggtaaagcgcaaggatcgtcctggatctttattagatcgattaagcca
atttttgtctatggtcattaaattttccaatatgcggcgtaaatcgtgcccgcctcgcggcaggatcgtttacacttagcgagttctggaaagtcctgt
ggataaatcgggaaaatctgtgagaaacagaagatctcttgcgcagtttaggctatgatccgcggtcccgatcgttttgcaggatcttgatcggg
catataaccgcagacagcggttcgtgcgtcaccctcaagcagggtcttttcgacgtacgtcaacaatcatgaatgtttcagccttagtcattatcg

acttttgttcgagtggagtccgccgtgtcactttcgctttggcagcagtgtcttgcccgattgcaggatgagttaccagccacagaattcagtatgtg
gatacgcccattgcaggcggaactgagcgataacacgctggccctgtacgcgccaaaccgttttgtcctcgattgggtacgggacaagtacct
taataatatcaatggactgctaaccagtttctgcggagcggatgccccacagctgcgttttgaagtcggcaccaaaccggtgacgcaaacgcc
acaagcggcagtgacgagcaacgtcgcggcccctgcacaggtggcgcaaacgcagccgcaacgtgctgcgccttctacgcgctcaggttg
ggataacgtcccggccccggcagaaccgacctatcgttctaacgtaaacgtcaaacacacgtttgataacttcgttgaaggtaaatctaacca
actggcgcgcgcggcggctcgccaggtggcggataaccctggcggtgcctataacccgttgttcctttatggcggcacgggtctgggtaaaac
tcacctgctgcatgcggtgggtaacggcattatggcgcgcaagccgaatgccaaagtggtttatatgcactccgagcgctttgttcaggacatg
gttaaagccctgcaaaacaacgcgatcgaagagtttaaacgctactaccgttccgtagatgcactgctgatcgacgatattcagtttttgctaat
aaagaacgatctcaggaagagtttttccacaccttcaacgccctgctggaaggtaatcaacagatcattctcacctcggatcgctatccgaaag
agatcaacggcgttgaggatcgtttgaaatcccgcttcggttggggactgactgtggcgatcgaaccgccagagctggaaacccgtgtggcg
atcctgatgaaaaaggccgacgaaaacgacattcgtttgccgggcgaagtggcgttctttatcgccaagcgtctacgatctaacgtacgtgag
ctggaaggggcgctgaaccgcgtcattgccaatgccaactttaccggacgggcgatcaccatcgacttcgtgcgtgaggcgctgcgcgactt
gctggcattgcaggaaaaactggtcaccatcgacaatattcagaagacggtggcggagtactacaagatcaaagtcgcggatctcctttcca
agcgtcgatcccgctcggtggcgcgtccgcgccagatggcgatggcgctggcgaaagagctgactaaccacagtctgccggagattggcg
atgcgtttggtggccgtgaccacacgacggtgcttcatgcctgccgtaagatcgagcagttgcgtgaagagagccacgatatcaaagaagatt
tttcaaatttaatcagaacattgtcatcgtaaacctatgaaatttaccgtagaacgtgagcatttattaaaaccgctacaacaggtgagcggtccgt
taggtggtcgtcctacgctaccgattctcggtaatctgctgttacaggttgctgacggtacgttgtcgctgaccggtactgatctcgagatggaaat
ggtggcacgtgttgcgctggttcagccacacgagccaggagcgacgaccgttccggcgcgcaaattctttgatatctgccgtggtctgcctgaa
ggcgcggaaattgccgtgcagctggaaggtgaacggatgctggtacgctccgggcgtagccgtttttcgctgtctaccctgccagcggcggatt
tcccgaacctcgatgactggcagagtgaagtcgaatttaccctgccgcaggcaacgatgaagcgtctgattgaagcgacccagttttctatggc
gcatcaggacgttcgctattacttaaatggtatgctgtttgaaaccgaaggtgaagaactgcgcaccgtggcaaccgacggccaccgtctggc
ggtctgttcaatgccaattggtcaatctttgccaagccattcggtgatcgtaccgcgtaaaggcgtgattgaactgatgcgtatgctcgacggcgg
cgacaatccgctgcgcgtacagattggcagcaacaacattcgcgcccacgttggcgactttatcttcacctccaaactggtggatggtcgcttcc
cggattatcgccgcgttctgccgaagaacccggacaaacatctggaagctggctgcgatctgctcaagcaggcgtttgctcgcgcggcgattc
tctctaacgagaaattccgcggcgtacgtctttatgtcagcgaaaaccagctgaaaatcaccgccaacaacccggaacaggaagaagcgg
aagagatcctcgacgttacctatagcggtgcggagatggaaatcggcttcaacgtcagttatgtgctggatgttctgaacgcgctgaaatgcga
aaacgtccgcatgatgctgaccgattcggtttccagcgtgcagattgaagatgcggccagccagagcgcggcttatgttgtcatgccaatgag
actgtaatgtccctcacccgcttgttgatccgcgatttccgcaacattgaaaccgcggatctcgccttatctcccggctttaactttctggtaggtgcc
aacggcagtggcaaaaccagcgtgctggaagccatctatacgctcggccatggtcgggcgtttcgcagtttgcagattggtcgcgtcattcgcc
atgagcaggaggcgtttgttctccacgggcgattacagggcgaagagcgcgagacagcgattggcttaaccaaagacaaacagggcgac
agcaaagtccgcatcgacggtacagacgggcataaggtcgcggaactggcgcacctgatgccaatgcagttgataacgccagaagggttt
actttactcaacggcggccccaaatacagaagagcattcctcgactggggatgctttcacaacgaacccggatttttcaccgcctggagcaatc
tcaagcgattgctcaagcagcgcaatgcggcgctgcgccaggtgacacgttacgaacagctacgcccgtgggataaagagctgatcccgct
ggcggagcaaatcagcacctggcgcgcggagtatagcgccggtatcgcggccgatatggctgatacctgtaagcaatttctccctgagttttct
ctgactttctctttccagcgcggctgggagaaagagacagaatatgctgaggtgctggaacgtaattttgaacgcgatcgccagctaacctaca
ccgcgcacggcccgcacaaagcggacttacgcattcgcgccgacggtgcgccggtggaagataccttatcgcgtgggcagcttaagctgttg
atgtgcgccttacgtctggcgcaaggagagttcctcacccgtgaaagcgggcggcggtgtctctacctgatagatgatttgcctctgagcttgat
gatgagcgtcgcgggctgcttgccagccgcttaaaagcgacgcaatcacaggtctttgtcagcgcgatcagtgctgaacacgttatagacatgt
cggacgaaaattcgaagatgtttaccgtggaaaagggtaaaataacggattaacccaagtataaatgagcgagaaacgttgatgtcgaattc
ttatgactcctccagtatcaaagtcctgaaagggctggatgcggtgcgtaagcgcccgggtatgtatatcggcgacacggatgacggcaccg
gtctgcaccacatggtattcgaggtggtagataacgctatcgacgaagcgctcgcgggtcactgtaaagaaattatcgtcaccattcacgccga
taactctgtctctgtacaggatgacgggcgcggcattccgaccggtattcacccggaagagggcgtatcggcggcggaagtgatcatgaccgt
tctgcacgcaggcggtaaatttgacgataactcctataaagtgtccggcggtctgcacggcgttggtgtttcggtagtaaacgccctgtcgcaaa
aactggagctggttatccagcgcgagggtaaaattcaccgtcagatctacgaacacggtgtaccgcaggccccgctggcggttaccggcga
gactgaaaaaaccggcaccatggtgcgtttctggcccagcctcgaaaccttcaccaatgtgaccgagttcgaatatgaaattctggcgaaacg
tctgcgtgagttgtcgttcctcaactccggcgtttccattcgtctgcgcgacaagcgcgacggcaaagaagaccacttccactatgaaggcggc
atcaaggcgttcgttgaatatctgaacaagaacaaaacgccgatccacccgaatatcttctacttctccactgaaaaagacggtattggcgtcg
aagtggcgttgcagtggaacgatggcttccaggaaaacatctactgctttaccaacaacattccgcagcgtgacggcggtactcacctggcag
gcttccgtgcggcgatgacccgtaccctgaacgcctacatggacaaagaaggctacagcaaaaaagccaaagtcagcgccaccggtgac
gatgcgcgtgaaggcctgattgcggtcgtttccgtgaaagtgccggacccgaaattctcctcccagaccaaagacaaactggtttcttctgaggt

gaaatcggcggttgaacagcagatgaacgaactgctggcagaatacctgctggaaaacccaaccgacgcgaaaatcgtggttggcaaaat
tatcgatgctgcccgtgcccgtgaagcggcgcgtcgcgcgcgtgaaatgacccgccgtaaaggtgcgctcgacttagcgggcctgccgggc
aaactggcagactgccaggaacgcgatccggcgctttccgaactgtacctggtggaaggggactccgcgggcggctctgcgaagcagggg
cgtaaccgcaagaaccaggcgattctgccgctgaagggtaaaatcctcaacgtcgagaaagcgcgcttcgataagatgctctcttctcagga
agtggcgacgcttatcaccgcgcttggctgtggtatcggtcgtgacgagtacaacccggacaaactgcgttatcacagcatcatcatcatgacc
gatgcggacgtcgacggctcgcacattcgtacgctgctgttgaccttcttctatcgtcagatgccggaaatcgttgaacgcggtcacgtctacatc
gctcagccgccgctgtacaaagtgaagaaaggcaagcaggaacagtacattaaagacgacgaagcgatggatcagtaccagatctctatc
gcgctggacggcgcaacgctgcacaccaacgccagtgcaccggcattggctggcgaagcgttagagaaactggtatctgagtacaacgcg
acgcagaaaatgatcaatcgtatggagcgtcgttatccgaaagcaatgctgaaagagcttatctatcagccgacgttgacggaagctgacctttt
ctgatgagcagaccgttacccgctgggtgaacgcgctggtcagcgaactgaacgacaaagaacagcacggcagccagtggaagtttgatg
ttcacaccaatgctgagcaaaacctgttcgagccgattgttcgcgtgcgtacccacggtgtggatactgactatccgctggatcacgagtttatca
ccggtggcgaatatcgtcgtatctgcacgctgggtgagaaactgcgtggcttgctggaagaagatgcgtttatcgaacgtggcgagcgtcgtca
gccggtagccagcttcgagcaggcgctggactggctggtgaaagagtcccgtcgcggcctctccatccagcgttataaaggtctgggcgaga
tgaacccggaacagctgtgggaaaccactatggacccggaaagtcgtcgtatgctgcgcgttaccgttaaagatgcgattgctgccgaccagt
tgttcaccacgctgatgggcgacgccgttgaaccgcgccgtgcgtttattgaagagaacgccctgaaagcggcgaatatcgatatttaatggc
gctaaccatgcgagcgtgcctgatgcgctacgcttatcaggcctacgaaaatcttgcaatgtattgaattttcatgattttgtaggccggataaggc
gttcacgccgcatccggcaagaagctataagaaaagggcggagatcatctccgcccttttttatttctgcaatccgcaattatcatcccgcaaatc
tatactttttgggtcaacacgtaacgctttacaggtgattatcatggggctttttgatgaagttgtcggtgcctttctgaaaggcgatgcggggaaata
tcaggctattttaagttgggttgaggagcagggcggcattcaggtgctgctggaaaaactgcaaagtggcggcttaggggccattctctcaacct
ggctgagtaatcaacagggcaatcaatcggttagtggcgagcaactggaatcggcgctcggcacaaatgcggtgtccgatctcgggcaaaa
acttggcgtggataccagtacagcttccagtttactggcagaacaattgccgaagattattgatgcgctctcaccgcaaggtgaagtgtcaccac
aagccaataacgatctgctttccgcaggcatggaactgctgaaagggaaactcttccgctaagcaaaaagggagcacgcaacgcgcgttgc
atccccccttttccctcgggtgttgtactgatttttgagcggaatcgcgttagcatgggtcaggaaccaatctacctggggaactcatggctattaaac
tcattgctatcgatatggatggcacccttctgctgcccgatcacaccatttcacccgccgttaaaaatgcgattgccgcagctcgcgcccgtggc
gtgaatgtcgtgctaacgacgggtcgcccgtatgcaggtgtgcacaactacctgaaagagctgcatatggaacagccgggcgactactgcat
tacttataacggcgcgctggtacagaaggccgctgatggtagcaccgtggcgcaaactgctctcagctatgacgactatcgtttcctggaaaaa
ctctctcgcgaagtcggttctcatttccacgccctggaccgcaccacgctgtacaccgccaaccgtgatatcagctactacacggtgcatgaatc
cttcgttgccaccattccgctggtgttctgcgaagcggagaaaatggaccccaatacccagttcctgaaagtgatgatgattgatgaacccgcc
atcctcgaccaggctatcgcgcgtattccgcaggaagtgaaagagaaatataccgtgctgaaaagtgcgccgtacttcctcgaaatcctcgat
aaacgcgttaacaaaggtacgggggtgaaatcactggccgacgtgttaggtattaaaccggaagaaatcatggcgattggcgatcaggaaa
acgatatcgcaatgattgaatatgcaggcgtcggtgtggcgatggataacgctattccttcagtgaaagaagtggcgaactttgtcaccaaatct
aaccttgaagatggcgtggcgtttgctattgagaagtatgtgctgaattaatctgtgggcgggcaaacagcataaacgcgtttgcccgcttactg
attattggtttactgcatatacaaccagaaattgcagcggtgcagtaatgatgtccagcgtaacagtcactggcagtaatgctgcggaaacacc
atccggaatgcctctgggtgaatagtattcataaaatcgcactttaaatggatggtagaacgccatcacctttgagtaatccatgtttttatttttctcat
ggattgtgccttttaggttttccagtgacctggtacagtgttgaacaccacaggcaaaaccataatgcgttgccagtgccttttcttcttcgttattcca
gtagtagtaaagcgatattgtgccggtaaatttctttttccccgcatttaaaacaaatcttgcgtcatcggcaacctgaatattgtgccggttaagtgc
tggatcattgagcatcttaacgatgtcatcggcaccatcagtgataaggtaatccagcgattcccctacaaaaacatagccttttgttccattactat
cttttgcctgcgatagcccagtgatggtgtctgactgcgattgatactttcctgaactaccctttgcggtatgggaaaccagagcatacgagatac
agcctgaaagcatcagcgccagtgggaataaaccggcagccttaaaaaatcccttaaaattcaacttcatgattaatcctttaaacgagtagct
aagccgttgaaagcatactcttctttccctcctgtttagcgcctgatatcccttttcagatttctgcccgacgcatgtcattttttttatgcattgttctttttgtg
atctaaattgtagtacaacaatataagtttgtactacattacacgcacggcaaacgcgaacgtcatcacgctggtactacaaagttgccgcgtta
tgcatcgatcggggtaaagtagagaagaacatacagagcacaaggactctccatgactctcaataaaaccgatcgcattgtcattacgctgg
gtaaacagatcgttcacggcaaatacgtgccaggctcgccgcttccggctgaggcggaactctgtgaggagtttgcaacctcgcgcaacatc
atccgtgaggtgttccgttcgctgatggcgaagcggctgattgaaatgaaacgttatcgcggggcgtttgtggcaccgcgtaaccagtggaatta
cctcgacactgacgtactgcaatgggtgctggaaaatgactacgacccacggcttatcagtgccatgagcgaagtgcgaaatctggtggaac
cggcgattgcccgttgggcagcagagcgcgcgacttccagcgatctggcgcagattgaatcggcgctgaacgagatgattgccaacaatca
ggaccgcgaagcgtttaacgaagcggatattcgctaccacgaggcggtgctgcagtcggtgcataacccggtgttacagcaacttagcattgc
gatcagttcgctgcagcgggcggtttttgaacgaacctggatgggcgatgaggccaacatgccgcaaacgctccaggaacataaggcgctg
ttcgatgcgatacgacatcaggacggcgatgcggcagagcaggcggcgcttaccatgatcgccagctcgacacgaaggttaaaggaaatc
acatgacagctcgctacatcgcaattgactggggatcgaccaatctgcgcgcctggctttatcagggcgaccactgcctggagagcaggcaa

tcagaagcaggcgtcacgcgcctgaacggaaaatctccggctgcggtgttagcagaagtcacgaccgactggcgtgaagagaaaacgcc
agtggtaatggcaggaatggttggcagcaacgtcggctggaaagttgcaccgtatttatctgttcctgcctgtttttcgtctattggcgaacaattaa
cgtcagttggcgacaatatctggattattcccggattatgtgtctctcatgacgataaccacaatgtgatgcgcggcgaagaaacacaattgatc
ggcgcgcgagctctggctccttcctctctttatgtcatgcccggaacccattgcaaatgggtgcaggccgatagccagcaaatcaacgattttcg
caccgtgatgaccggtgaattacatcatttactgttaaatcactcattgattggcgcaggtttgccgccgcaggaaaactctgccgatgccttcac
agctggccttgagcgtggtcttaatacgcccgccatattgccgcagctttttgaagttcgcgcctcgcatgtgctgggaacacttccccgcgaaca
ggtcagcgaatttctctctggtttgttgattggcgcagaggtcgccagtatgcgcgactatgtggcccatcaacacgccatcacccttgtcgccgg
aacatcgctgaccgcgcgctaccagcaagcctttcaggcgatgggttgcgacgtgacggcggtggcgggcgacacggcatttcaggctggt
ataaggagcatcgctcatgcagtggcaaactaaactcccgctgatcgccattttgcgcggtattacgcccgacgaggcgctggcgcatgttgg
cgcggtgattgacgccgggttcgacgcggttgaaatcccgctgaattccccacaatgggagcaaagcattcccgccatcgttgatgcgtacgg
cgacaaggcgttgattggcgcaggtacggtactgaaacctgaacaggtcgatgcgctcgccaggatgggctgtcagctcatcgttacgccca
atatccatagtgaagtgatccgccgtgcggtgggctacggcatgaccgtctgccccggctgcgcgacggcgaccgaagcctttaccgcgctc
gaagcgggcgcgcaggcgctgaaaatatttccgtcatcggcttttggtccgcaatacatcaaagcgttaaaagcggtattgccatcggacatc
gcagtctttgccgttggcggcgtgacgccagaaaacctggcgcagtggatagacgcaggttgtgcaggggcgggcttaggcagcgatctcta
tcgcgccgggcaatccgtagagcgcaccgcgcagcaggcagcagcatttgttaaggcgtatcgagaggcagtgcaatgaaaatcaccaaa
attaccacgtatcgtttacctccccgctggatgttcctgaaaattgaaaccgatgaaggcgtggtcggttggggcgagcccgtgatcgaaggcc
gcgcccgtacggtggaagcggcagttcacgagctgggtgactatttgattggtcaggatccatcgcgcatcaatgacttatggcaagtgatgtat
cgcgccggattctatcgcggcggtccgatcctgatgagcgccatcgccgggattgaccaggcgttatgggatatcaaaggtaaagtgctgaat
gcgccggtctggcaactgatgggcggcctggttcgcgacaaaattaaagcctacagttgggttggcggcgatcgtccggcggatgttatcgac
ggcattaaaacgctacgcgaaatcggcttcgataccttcaaactgaacggttgtgaagaactggggctaattgataactcccgcgcggtagat
gcggcggttaacaccgtggcacaaattcgtgaagcttttggcaatcagattgagtttggtcttgatttccacggtcgcgtcagcgcgccgatggc
gaaagtgctgattaaagaactggagccgtatcgcccgctgtttattgaggagccggtgctggcggaacaagccgaatactacccgaaactgg
cggcacaaacgcatattccactggcggcgggtgaacgcatgttctcacgcttcgattttaaacgcgtgctggaggcaggtggtatttcgattctgc
aaccggatctctcccacgcgggcggtattaccgaatgctacaaaatcgccggaatggcagaagcctatgacgtgacccttgcgccgcactgt
ccgctcggaccgattgcactggcggcttgcctgcatatcgactttgtttcctataacgccgtacttcaggaacaaagtatgggaattcattacaac
aaaggcgcggagttactcgactttgtgaaaaacaaagaagacttcagcatggtcggcggcttctttaaaccgttaacgaaaccgggcttaggc
gtggaaatcgacgaagctaaagtgattgagttcagtaaaaatgccccggactggcgtaatccgctctggcgtcatgaagataacagcgtagc
agagtggtaattcctgccacgtaagcccctcatcgggcactaaaacagcaatacaaaaatataaccctctgtaaattacagggcatggtgag
cggcttcgctatgcccaaaatctggagaaagctcgctatggatattcccgttaatgcagcaaagccggggcgtcggcgttatctgacgctggtg
atgatctttattacggtagtcattgttatgtcgaccgcgccaacctggccgtggcttccgcccatattcaggaagagttcggcattaccaaagcgg
aaatgggctatgtattttcggccttcgcctggctttatacgctatgtcagatccccggcggttggtttttagatcgcgtaggttctcgcgtgacttatttta
ttgcgatatttggctggtcagtggcgactttattccagggctttgccacgggcttaatgtcattaattggtctgcgcgcgataaccggtattttcgaag
cgcctgctttcccgaccaataaccggatggtgaccagctggttcccggaacatgaacgcgcttctgccgttggtttttatacgtctggtcagtttgtc
ggtctggcgtttctgacgccgctgctgatctggattcaggagatgttgagctggcactgggtgttcattgtcactggtggtatcggcattatctggtcg
ctgatttggtttaaggtttatcagccgccgcgcctgaccaaaggtatcagcaaagctgaactggattacattcgtgatggcggcggtctggtggat
ggtgatgcgccggtgaagaaagaggcgcgtcagccgttaacagccaaagactggaaactggtgttccatcgtaaactgatcggcgtttatctt
gggcaatttgcggtggcttctacactgtggtttttcttaacctggttcccgaactatttaacccaggaaaaaggaatcacggcgctgaaagcagg
ctttatgaccacggtgccattcctcgcggcgtttgtcggcgtcctgctctctggctgggtagcggatctgctggtacgtaagggcttttcactgggctt
tgcgcgtaaaacgccgattatctgcggcttgctgatctccacctgcattatgggcgctaactacactaacgatccgatgatgattatgtgcctgatg
gcgctggcattcttcggtaacggttttgcttcgattacctggtcgctggtttcttctctggcaccgatgcgcctgattggtttaaccggcggcgtgtttaa
cttcgccggtggtctgggcggcatcaccgttccgctggtggtggggtacctggcgcagggttacggtttcgcacctgcactggtttatatctccgcc
gtcgcgttgattggcgcgctctcttatatcctgctggtgggcgatgtgaagcgcgttggctaatccttcaactgtggaatatgtgccacaccactgc
gcataatccactttcttaaagccggtgcggtcaggcatcggctttttactatcttgccgaagagttttaaacgcagtttgcgggtggcgcgccagta
agcggtattgagcttctctggctgcttcagtaacaccgaacgcagaatgtctgtgctatccagcgcatagctaatcccctccagcgagctggcgc
tgataaatcccgccgcttcaccaatcagaaaggcgttgtccttaccgcagacaaaatcctgccagcgcgagggaaacagcaccgtgcattttt
cgcttttcaccgtcttaccaaactggaactgaaaggcgctcattttctctttcagcgtcgtgaaacgcgtctgaccgtctttcattggataggcaccg
ccaaagataaaataaccgtctttgctgatactccatgaataacagttagttatcgaattatcaaagatgcaggagtagaacggcaccggatgttt
ctccgcgaaccactgctggatagcgacatatttacggatttgatgatccgggtagagatgtcgccgcaccatcgagtttgcgccatcggcaccg
accagatagcgggcggtaatatgttgctcccagccgtctgcacgaaaaatgacatgccatttatcatcctcacgccagatttttcggcacaggct
gtcgtgataaacctcaacgctggcggggatcagcgatttcatccacaagtcgaaagcatggcgattaatattgatatagcttcgctggtagttac

gcgtgagcgatgcggcgacgtcgacggttttgacgctgaaaatctgcggattagcgatcacatcgacaggaagcgtcagcccatcgcgaat
aaaagatcgctgcgcgtccggtgccagcagaccgccgcagggtttgctgaaaccttcagtaccacactggtgctttttatccagcgcgatcacc
tgcattttgcctgctaactttcgtgccaacgccgatcctgccgggccgaggccaataatcgccacgtcgaaatgttccatcttttcactcacattcat
cacgatggcaaaagactattgtctgaaagtgaagtccttgtgaagtatgatggcgggacccgttttgtatcaggaccgcgccatgaaacagat
aacctttgctccccgtaatcacctgctcaccaataccaatacctggacgcccgacagccagtggctggtatttgacgtgcgtccttctggcgcgtc
gtttaccggcgagaccattgagcgtgtgaatatccataccggcgaggtcgaggttatctatcgcgcgtcacagggcgcacacgtcggcgtggt
gaccgttcatccgaagtcagagaaatatgttttcatccacggcccggaaaatcctgatgaaacatggcattacgacttccatcatcggcgcgga
gtgattgttgaaggcggcaagatgagtaaccttgatgcgatggatattaccgcgccgtatacgccaggagtgctgcgcggcggcagccatgtg
catgtctttagcccgaacggtgaaagggtgagctttacctataacgaccatgtaatgcatgaactcgatccggcgctggatttgcgaaacgtcg
gtgttgctgcgccgtttggcccggtcaacgtacaaaagcagcatccgcgtgaatacagcggtagccactggtgcgtgctggtgagcaaaacc
acgcccacgccacagcctggcagtgatgaaatcaatcgtgcttatgaagaaggatgggtaggaaatcacgcgctggcatttattggcgacac
actttcgccaaagggcgagaaagtgccggagctgtttatcgttgagttaccgcaagatgaagctggctggaaagcggcaggtgatgcgccgtt
aagcggaacggaaacaaccctgcccgcgccaccgcgtggcgtcgtgcagcgacgtttaacctttacccaccatcgggcttatccggggttag
tcaacgtcccgcgccactgggtgcgctgtaatccgcagggtacacaaatcgcgtttttaatgcgagatgataacggcattgtgcaactgtggctt
atctcgccacagggcggcgagccgcgccagttaacccataacaaaacggatattcagtctgcatttaactggcatccgtcaggagaatggttg
ggctttgtgctggataatcgaattgcttgtgcccatgcgcaaagcggcgaggttgagtatttaaccgaacaccacgccaattcaccttctgcgga
cgccgtggtcttctcgccggatggtcagtggctggcgtggatggaaggcggccagctgtggatcaccgaaactgatcgctaaatcagttatca
ggcattctggccggtggaataaccgcattggtggcttgcgcgttggcttcgctttttttcaacgcgcgagcgcaccgagctgtctttgcggaacaca
tcccacggcagtaaaagcgtgtccatcacagcggtaaacggcatatcgagaatcgccagcgacttagtgccccagttggtttcatcgtcgcca
atcatggttgcgctggcgcgggtgccgggatacgttccttctttccctccggtatgggacatcacgctggagcatcctccgagtaatgtcattccgc
tgcatatcatgaacgctaacaacacatttcttatcataataaattcatctgttgatcgtgggtgttggcctgatgagttatagcgatcccttgctgaaa
ataacatcatcattacgtcgcactgtggcggctatcgcactttaacgtttcgtgctgccccctcagtctatgcaatagaccataaactgcaaaaaa
aagtccgctgataaggcttgaaaagttcatttccagacccatttttacatcgtagccgatgaggacgcgcctgatgggtgttctggctacctgacct
gtccattgtggaaggtcttacattctcgctgatttcaggagctattgattatgcgtaactttgatttatccccgctttaccgttctgctattggatttgaccg
tttgtttaaccacttagaaaacaaccagagccagagtaatggcggctaccctccgtataacgttgaactggtagacgaaaaccattaccgcatt
gctatcgctgtggctggttttgctgagagcgaactggaaattaccgcccaggataatctgctggtggtgaaaggtgctcacgccgacgaacaa
aaagagcgcacctatctgtaccagggcatcgctgaacgcaactttgaacgcaaattccagttagctgagaacattcatgttcgtggtgctaacct
ggtaaatggtttgctgtatatcgatctcgaacgcgtgattccggaagcgaaaaaaccgcgccgtatcgaaatcaactaattccctaaggccgcc
tggcgcggcctgacatctccatgctcgccgtcagggagcatatgcgaatcttcggatttgcaggtacttactcgcttcttagaaggagaaatgact
atgcgtaacttcgatttatccccactgatgcgtcaatggatcggttttgacaaactggccaacgcactgcaaaacgccggtgaaagccagagct
tcccgccgtacaacattgagaaaagcgacgataaccactaccgcattacccttgcgctggcaggtttccgtcaggaagatttagagattcaact
ggaaggtacgcgcctgagcgtaaaaggcacgccggagcagccaaaagaagagaaaaaatggctgcatcaagggcttatgaatcagcca
tttagcctgagctttacgctggctgaaaatatggaagtctctggcgcaaccttcgtaaacggtttactgcatattgatttaattcgtaatgagcctgaa
cccatcgcagcgcagcgtatcgctatcagcgaacgtcccgcgttaaatagctaactagctattctctttgccccgccattctggtggggctttttttgt
cttacttactcaacaatgcgttaaatttcgactgtttaagatatttcggcacgttctttcccgtgataatgtgcgctataaggcaaatctgcttcaccga
ttcagccataatccgtagcaattaaaatgttatctcaagagaaatggacgatgagtgatatagcattaacggtcagtattctggctttggtggcagt
cgtcggtttgtttatcggcaacgtcaaatttcgcggcataggattaggtattggcggcgtgctgtttggtgggatcatcgtcggccattttgtttctcag
gcggggatgacattaagtagcgatatgctgcatgttattcaggaatttggcctgatcctgttcgtttatactatcgggattcaggtagggccgggctt
ctttgcctcattgcgcgtctccggattacgcctcaacctgtttgctgttctgatcgtcatcatcggtggtctggttaccgccatcctgcataaactgtttg
atattccactgccggtagtgctggggattttctccggtgcggttaccaatacgccagcgctgggggcagggcagcagattttgcgcgacctgggt
acaccaatggaaatggtcgatcagatggggatgagttacgcgatggcgtatccattcggcatttgcgggattttgttcaccatgtggatgttgcgg
gttattttccgcgtcaatgtcgagacagaagctcagcagcacgagtcttcacgcaccaatggcggcgcgctgatcaagactatcaatattcgcg
ttgagaaccctaacctgcatgatttagccattaaagatgtaccgattctcaacggcgacaaaattatctgctcgcgtctgaaacgcgaagaaac
cctaaaagttccttcgccagataccattatccaactgggcgatttgctgcatctggtgggtcagccagcggatttacataatgcgcaactggtgatt
ggtcaggaggtcgatacttcgctgtccacgaaaggcactgatttgcgcgtcgagcgtgtggtggtcaccaatgaaaacgtgctcggaaaacgt
attcgcgacctgcactttaaagaacgctatgacgttgttatctcgcgcctgaaccgtgccggggtcgaactggtcgccagtggcgatatcagcct
gcagttcggcgatatcctcaatctggtggggcgtccgtccgcaattgatgccgttgccaatgtgctggggaatgcgcagcaaaaactgcaaca
ggttcagatgctgccagtgtttattggcatcgggctaggcgtattgttaggttctattcccgtctttgtgccaggattcccggccgcgttgaaactggg
gctggcgggcggaccgctgattatggcgttgatcctcgggcgtatcggcagtatcggcaagctgtactggtttatgccgccaagcgccaacctc
gcgctgcgggagctggggatcgtgctgttcctctcggtcgttggtctgaaatctggtggggattttgtgaataccctggtcaatggcgaagggcta

agctggattggttatggtgccctgatcaccgccgttccgctgattactgttggcattctggcgcggatgttagccaaaatgaattacctgaccatgt
gcgggatgctggcaggttccatgaccgatcctccggcgctggcgtttgctaataatcttcatccaaccagcggtgcggcggcgctctcttacgcc
actgtctatccgttggtaatgttcctgcgcattatcaccccccaattactggcggtgctcttctggagtatcggttaactcttttccgggtggagtcgcc
gtaaatggtactccacctggtattcgctggcatttctgaacatcactgaataattcaaaaactccccgctctcgctatatgacaacgaggtgatcc
gcagcagtggcgtgtgttccgctaccttcatttgccgcgccagcagtctatcggcgagcactggcgtcaggctttcataattaccgccaatcaaa
atcccgcactcttgttcaatatattcaaacttcgacccttccagatgttgcagcgaaagattacggaacagttttaccggcatatagctgtcttccag
catcagcggtttcccttccacaaaacgaacgcgacgggagaagtagatctgctcgttgatttgaatccgtaactggctggcaatggccggagg
cgcaggcatgatttcaaaaatcagcacctggctggtgaccgtttttccctgccgttttaacacctccaccagcccggtcagactggcggtttgatg
cagtacatctttgcgcaccaggtaagtgccactaccgtggcggcggaccaccagcccccacgctaccagcaggtcaatggctttacggatgg
tcatccgcgataccgcaaactcttccgccagcttttttcaccgggaagaaggctgtttagcgtgaaatctgcggagttaagtcgaattcttaaccg
ctccgcaatgcttttgtagatcatctctagatctctttttgcctgcaagcccgggtaatgcgcacacaagttctgcttaatcgattgaaaaaacatctt
ttttttaaagatgtgttcgatgctgtgtacctgtgctcacagaggtctactttttcgcgaaaacgtagatctctaccgcccaacgaaaagcatgaaa
gcgatcacgaatcccattgggtcgtcatgttctgccgatcgcatcttcctatcctcgctccaggcctgccgcataaccaatcaggcttcctacttac
agaattgagaaaagaggatgtggaaatgctcagtcaaattcaacgctttggcggcgcgatgttcacgccagtgctgctgtttcccttcgccggg
attgtggtgggtcttgccatcttgctgcaaaacccgatgtttgtcggggaatcactgaccgatccgaacagtttattcgcgcaaatcgtacacatta
ttgaagagggcggttggacggtattccgtaatatgccgctgatttttgctgtcggtttacccattggccttgctaagcaagcgcaggggcgtgcttgt
ctggcggtgatggtgagtttcctgacctggaactatttcatcaacgcgatgggaatgacctggggaagctacttcggcgtcgatttcactcagga
cgcggtggcaggtagcggtctgacaatgatggccgggattaaaaccctcgataccagcattatcggcgcaattatcatttccggcattgtgacg
gcgctgcataaccgtctgttcgataaaaaactgccggtttttctcggcattttccaggggacgtcttatgtggtgattatcgccttcctggtgatgatcc
cctgtgcctggctgacgttgctcggctggccaaaagtacaaatggggattgaatctctgcaagcgttcctgcgttcggcgggtgcacttggggtct
gggtttacaccttcctcgaacgtattctgatcccaaccggtttacaccacttcatctacggacagtttatctttggtccggcagctgttgaaggcggc
attcagatgtactgggcgcagcatctgcaagagttcagtttgagcgccgagccgctgaaatcgttgttcccggaaggcggttttgccctgcacgg
taactcaaaaatctttggtgccgtgggcatttctttagcgatgtacttcactgccgcaccggaaaatcgggtaaaagtggcgggcttgctgattcc
cgcaaccttaaccgccatgctggtgggaattaccgaaccgctggaatttaccttcctgttcatttcaccgttgctgtttgcggtacacgccgtgctgg
cggcctcaatgtcgaccgtaatgtatctctttggtgtggtgggcaacatgggcggaggtctgattgactaggttttaccgcaaaactggatcccga
tgttcagcaaccacgcggatatgatgctgacccaaatcgccattgggttgtgctttaccctgctgtacttcgtggttttccgcacactgattctgcagt
tcaacatgtgcacgccgggacgtgaagatgcggaagtgaaactctactcaaaagccgaatacaaagcctcgcgaggccaaaccaccgcg
gcagagccaaaaaaagagctggatcaggctgccggtatcctgcaagccctgggcggggtcggcaatatctccagcattaacaattgcgcga
cgcgtttacgtattgcactgcatgacatgtcacaaacgctggatgacgaagtctttaaaaagctgggagcgcacggcgtcttccgtagtggcga
tgccattcaggtgatcattggtctgcatgtatctcagctgcgtgaacagctcgatagcttaattaattctcatcaatcagcagaaaatgttgccatta
cggaggcagtataatgaccaaattctcagtggttgtcgcaggcggtggaagcacctttacgccaggcatcgtgttgatgctcctggcgaatcag
gaccgtttcccgcttcgtgcactgaaattttatgataacgatggtgcgcggcaggaagtgattgccgaagcctgtaaagtcatccttaaagaaaa
agcgccggacattgcgtttagttacaccaccgatcctgaagtggcattcagcgacgttgattttgtcatggcgcacatccgcgtcggcaaatacc
cgatgcgcgaactggatgaaaaaatcccgctgcgccacggcgttgttggtcaggaaacttgcggacccggcggaatagcgtacggcatgcg
ttccattggcggcgtcctggaactggtggattatatggaaaaatattcaccaaatgcctggatgctcaactactccaacccggcagccattgtcg
cagaagccacgcgtcgtctgcgcccgaatgcgaaaatcctcaacatctgtgacatgccaatcggtattgaaagccggatggcgcaaattgttg
ggctgcaagatcgcaaacagatgcgcgtgcgctactacggcctgaaccactggtggtcggcgatatcccgcagtttcagaaagggttaatga
gtcagcaagtggcggtggaaaaactggtcgtggacgcctgggaacagcgttcatatcagcacctgtggcaggcgattacgttgtcgaaaacg
gtaccgagcgcctcggtcgccaaagctattctggatgagttgctggaggccaacaaagcgtactggccagagttacgttaattgtgtggactgg
catccgttgggtgctggtccgtattctttagcacggctgacggcgcataaccaaatttatgtttaaaggctttgctgaaatgaaaggagtcgaaga
agttgagcgtatcagcgacttcgccaaccgagtttccttgttgctttaatagcgatagcgccagatccagacgcgcatcgaggtaatattctttcgg
cgtcttgccggtatagcgtaaaaacagacgacgcaaccaggcttcgctacaggggatcgtggcagccatatcagctacgctccagcgttgttg
cagactggcatgtaaagtggcaattaatttttcaatttgccgccgttgtggatctttttttaccatctgcgtacataagacaaatccactgataaataatt
ttcgtcagaaaagcgactgccagattatttttattgcttctggtgaagttattaactcagcaacttcggtgagttcctgattataaatttcgccattata
aataacgctttgctgaccaacgggaatatccatcatactggtgggggtaaattccatccagtactgttcccagactaaaccttcacagtgataag
agtgaatatccattggctttaagaatataatacagttgccatgcagtgttatttgttcgccattctttaaaagaattttcccacatccttgtacggtatac
accgcaacccatgagttggcgataagcggtttcttcttgtcccgcggccagatcacccggtaatgctcgtcggcgtaggtatgccagagcgag
atcagagaaattccgctggagatcacattttcgttaatcaataagggaatattgtacggagtttcgtttttttacatctgaactttgcaattttccattcatt
ttaatatccctccggcgaattatgctcacaacctgaacttaacagaaaacaatccgttcagggatacgacgtttgtattttaagaatctgactgcct
gacccgacgcattttacctctccctatattcatgcgtccgggacataaacataaataagggctatgagatgaattcgttacaaatcttgagttttgtc

ggttttacgctgctggtggcggtgatcacctggtggaaggtccgcaaaacagataccggatcgcaacaaggctattttcttgccggacgttcact
aaaagcgccggttattgccgcttcgttaatgctaaccaacctttccacggaacaactggtcggcctttccgggcaggcctacaaaagcggcat
gtcggtgatgggctgggaagtgacttcagcggtgacgctgatcttcctcgcgctaatctttttaccgcgctatctgaagcgcggcattgccaccat
ccccgattttctggaggaacgttatgataaaacgacgcgtattatcatcgacttctgcttcctaattgccaccggcgtctgctttctgccgattgttctc
tactccggcgcgttggcgctcaacagcctgtttcacgtcggggaatcgctacagatttctcacggtgcggctatctggctactggtaattttgcttgg
tctggcgggaattttgtatgcggtgatcggcggactgcgcgcaatggcagtggcggactccatcaacggtattgggctggttatcggcgggttga
tggtgccggtatttggcctaatcgcgatgggcaagggcagctttatgcagggcattgagcaactcaccaccgttcacgccgagaaattaaactc
aatcggtggcccgaccgatcccttgccgattggcgcggcatttaccggtttgattctggtgaacaccttttactggtgtacaaatcagggcatcgtg
caacgcacgctggcgtcaaaaagcctggcggaagggcaaaagggggcgctgttaacggcggtgctgaaaatgctcgacccgctggtact
ggtgctgccagggttgattgcgtttcatctgtatcaggatttaccgaaagccgacatggcctacccgacgctggtcaataacgttctgccagtgcc
aatggtgggtttcttcggcgcggtgttatttggtgcggtgatcagtaccttcaacggctttctgaatagcgccagtacgttattcagtatgggtatttac
cgtcgcatcattaaccagaatgccgagccgcagcagctggtcaccgtcgggcgcaaatttggtttctttatcgctatcgtttcggtgctggtcgcg
ccgtggatcgccaacgcgccgcaggggctgtatagctggatgaaacagctcaacggcatttacaacgtgccgctggttaccatcatcattatg
ggctttttcttcccgcgcatcccggcgctggcggcaaaagtagcgatggggattggcataatcagctacatcaccatcaactatctggtgaagtt
cgacttccatttcctctatgtgctggcctgtacgttctgcatcaacgtggtcgtgatgctggtgatcggttttatcaaaccgcgcgccacgccgttcac
cttcaaagatgcgtttgcggtggacatgaaaccgtggaaaaacgtcaagatcgcgtcaattggcatcctgttcgcgatgattggcgtctatgccg
ggctggctgaattcggcggctacggtacgcgctggttagcgatgatcagttatttcattgccgccgtagtgattgtctacctgatttttgacagctgg
cggcatcgtcacgacccagccgtaacctttactcccgacgggaaggatagcctatgaaacgccccaattttctgttcgtcatgaccgataccca
ggccaccaatatggtcggttgctatagcggtaaaccgctgaatacgcaaaatattgatagtctggcggcggaaggtattcgctttaattccgcct
acacctgttcaccggtttgtacgcctgcacgggccggactatttaccggtatctacgctaaccagtccggcccgtggaccaacaacgtcgcgcc
aggcaaaaacatctccactatggggcgctactttaaggatgccggctatcacacctgttacatcggcaaatggcatctcgacggtcatgactatt
tcggcactggcgagtgtccgccggagtgggacgctgattactggttcgatggggcgaactatcttagcgaactgacggaaaaagagattagc
ctgtggcgcaatggcctaaacagcgtcgaagatttacaggcgaaccatatcgacgaaaccttcacctgggcgcatcgtatcagcaatcgggc
ggtggattttctgcaacagcctgcgcgcgcagacgagcctttcctgatggtggtttcgtatgatgagccgcatcacccgttcacctgtccggtgga
gtatttagagaaatacgctgatttttactacgagctgggcgagaaagcacaggatgacctggctaacaaaccggaacatcaccgcttatgggc
gcaggcgatgccatcgccagtcggtgatgacgggctttatcaccatccgctctattttgcctgtaatgactttgttgatgaccaaatcggacgggtc
atcaatgccttaacgccagagcaacgtgaaaatacgtgggttatttatacctccgatcacggcgaaatgatgggcgcacataagctgatcagt
aaaggggcggcgatgtatgacgacatcacccgcattccgctgatcatccgttcgccgcaaggggagcggcgacaggtcgatacgccagtc
agtcatatcgatttactgccgacaatgatggcgctggcagatattgaaaaaccagagattctgccgggggaaaatatccttgccgtgaaagag
ccacgcggcgtgatggtggaatttaaccgctacgagattgagcatgacagctttggcggttttattccggtgcgttgctgggtgacggatgacttta
aactggtactcaacctcttcaccagtgatgaactttacgatcgccgtaatgacccaaatgaaatgcataacctgatcgatgatatccgttttgcag
acgttcgcagcaaaatgcatgacgccttattggattacatggacaaaattcgcgatccgttccgcagttaccaatggagtctgcgtccgtggcgt
aaagatgcacggccgcgctggatgggggcgtttcgtccacgtccacaagatggctattcgccagttgtacgcgactatgacaccggcctacc
gacacaaggggtgaaggtggaggagaaaaaacagaagttctgacgataatgcccgctgatgatcacccggcgggcattattcaggctgctt
aaaccagtgaaccgccggtcaggaaggaaaagataaggaatatcagcaaaatgctgcacacgatgccgataccaaaaagcaaggtatg
gaaagccagcgtcccgccgccaaaaaccagcgcgccgcgaatgccccagcgattttccttacgcataccgccaataaatgcgcaaagtag
cgcagcaatggccagtgcgataccgctttgattgaggattttatcgacatctacgtcctctttttctccacggtggtaatttttcgccttttattccggc
gagaagacctcttttaatttcagccgttttgtccgccacgatgctttcaaacgttggcggtggtgcggagaaagggccgaatgaaaagtggata
atccccatcattaaagcgatggcaccgaacagcatgcccaatgaactgattttattttgagctatgatacccatctgacctccttgtagcggatgg
caaaagcatatcaggaaacgggcggacgaaattcttaaaaaaaccgctaaaggtagagcgttagtgaaattgcaaaggagcaacaatga
aaatttcccgcctcggagaagcgcctgattaccgcttctcgctggctaatgagcgtacttttctggcgtggatccgtactgcactaggatttctggc
ggcgggggttgggcttgatcaacttgcgccagatttcgccacgcccgtcattcgcgaactgctggcgttgttgttgtgtctgttttccggcgggttag
cgatgtatggctacttgcgctggttacgcaatgaaaaagcgatgcgtctgaaagaggacctgccctacaccaacagcttattaatcattagctta
atattgatggtcgtggcggtgatcgttatgggactggtgttgtatgccggatagccgcaaagccagacgcattgccgacccaggactgcagcc
ggagcgcacatcactggcgtggtttcgtaccatgctgggctacggcgcgttgatggcgttggctatcaaacacaactggcaccaggcgggcat
gttattctggatttcgattggcattctcgccatcgtggcgctgatcctctggcactacacccgtaatcgcaatttaatggatgtcacgaatagcgatttt
tcccaatttcacgtagtccgtgacaaatttttgatctccctcgcggtgttatctctcgcaatactgtttgctgtaacgcatatacatcaacttatcgtattt
attgagagagtcgcatgacaggaagtcaggtcattgatgctgaagaggacaggcataaactggttgtagagtacaaggatgctctgcaacct
gccgatttttatcacaatttcaaacagcggggcatccgctccgtacagctcattccctatcttgaatttgatgaccggggcgatctgacggccgctt
cggtgacagcagagctgtgggggaaatttttaattgccctgttcgagtgttgggtgcgggcagatatcagtcgtatctcgattgagctttttgacgc

caccttacaaaaatggtgtggcagcgaaaacccgcaaccgcgctgtgactgccaggcgtgcgactggcatcgcctgtgcccgcacgctcgg
caggagacgccagacagcgtgctttgtgcgggctatcaggcgttttattcttactccgcaccgcatatgcgggtgatgcgtgatctgataaagca
gcatcgttctcccatggagctgatgacgatgctgcggtgacgtgcgcttaaacgggctgcccctgatgcgacatccgcgtcgccagcggcagc
cagcacagcacgatcaacaatcccattaaggtcatcaacaaccccaggctgccctgaccggtttgcggcaacatcgcagagagcgacgcc
agcacgccggaaccaatgttttgcagaccgccgaccagcgcgccagccgtgcccgccaggaaggggaacggctccatcgcgccgctggt
cgccagcggaaacagcatcccggcaccgaaaaagaacagcgcggcgggaacgagcagcgtccagacattcatcacgccaaaccagtc
ggggatccacatcagcaagccagccagcaggcagcagataaccgactgccacattaacgtggagaagcgtttattgggacgtccggcaaa
ccatgcgccaaaaaacgctgccggaatcggcagaataaacaaaatactgaccgtcatactgctcagccctaacaccgcgcccatcagcac
gccggagcaggcttcaaaggcggcaatcccggccagaccgccaatcagcatcagcaaataacagttaaaaccgctgttaccgaaaagcg
ttttataactggtaagcaggcgcgtgcgcggtgcatcgaccggacgcgtttccggcatccagcgggccatactgaaggtcacaccagcacaa
agaaccaacaaaaagagataacaggcgcgccagttccacatcgtatccagcagaccgccgattagcggtgcgagcaacggactgacga
gaatccccatgtttaacaggctgttagcatggcgcaactgtgtccgttcatataaatctcgcggtaaagtacgcgccattacgccgccaacgcc
ggtgcccatcccctgcatcgcgctggcggcaatcaacaccgtcaaactggaggtcgtgaccgcgaccagcgttgccagcataaaaatggac
attccgacgaggatcaccggtcggcggcccacgcggtcggaaatcgggccataaaacagctgtgagacaccgtaagtcagcagataagc
gcccattacgctctgcaccgccccttcacggacgttgagatcgcgcgccatatcggcaatagctggaatataaatggtttgcgccatctgaccg
acggccacgagtaataccaacatcaataacaaattgacgtttctttgccttttcattatcacggatgcttttataaaaataattagaataagtcacta
cgcattcccataaatgcgggagaggaatctatcacattgatgaatgttagccagatatacgccaggaatggggaattgtttagcggcagaatat
gtaaacaaaagcggcaataaatgttgccgggagagatggtagggcggataagatgcgtcagcatcgcatccgacaccaacccgcacgct
aaatataaaaggggagcggtttcccgctcccctttggtgcgacttgaatctgaattacttcaggtatttcagaacagcatcaagcagttgcagtgc
tgcaacaatgagtttgaggataagaatggcgatatccaccaggttcatacgcgtctcctgtggttcaggagcactgccagctgacgtacctttcc
gctgcctgttgccagctgttgtgcacgcggtatctcaaggagaaacgttgtgcttgttgacataacacagtgtgctcacgcggggttaaggcgct
gacggcaccacccgtttcagccaggacttagtgcgccgggtaacgatgcggcccccgcataacacattgcgtacagtgatattatagaagttt
actgtataaataaacagtaatatttggacaaaacgcaaactgtgatcgatactcaactatgcactaaatacgtcaaaattcgtgccgaaattgc
gcgttctgcgcggaacacgtatactttcagtgttgacataatacagtgtgctttgcggttaccagccgcaggcgactgacgaaacctcgctccgg
cggggtttttgttatctgcaattcagtacaaaacgtgatcaacccctcaattttcccttgctgaaaaattttccattgtctcccctgtaaagctgtgcttg
tataaatattgttaaacacaaaaccaacaaggtccccaatgactacttccatgctcaacgcaaaactactaccaactgcgccatccgccgcag
tggtcgtcgtgcgtgtggtggtggtcgtcggcaatgcgccgtagggactggaacaacacacgattccaaaaccccgccggcgcaaaccggg
cggggtttttcgtttaagcacctcccggaaagtcggcccagaagaaaaggactggagcatggcaagttcgggcacaacatcgacgcgtaag
cgctttaccggcgcagaatttatcgttcatttcctggaacagcagggcattaagattgtgacaggcattccgggcggttctatcctgcctgtttacga
tgccttaagccaaagcacgcaaatccgccatattctggcccgtcatgaacagggcgcgggctttatcgctcagggaatggcgcgcaccgacg
gtaaaccggcggtctgtatggcctgtagcggaccgggtgcgactaacctggtgaccgccattgccgatgcgcggctggactccatcccgctga
tttgcatcactggtcaggttcccgcctcgatgatcggcaccgacgccttccaggaagtggacacctacggcatctctatccccatcaccaaaca
caactatctggtcagacatatcgaagaactcccgcaggtcatgagcgatgccttccgcattgcgcaatcaggccgcccaggcccggtgtggat
agacattcctaaggatgtgcaaacggcagtttttgagattgaaacacagcccgctatggcagaaaaagccgccgcccccgcctttagcgaag
aaagcattcgtgacgcagcggcgatgattaacgctgccaaacgcccggtgctttatctgggcggcggtgtgatcaatgcgcccgcacgggtg
cgtgaactggcggagaaagcgcaactgcctaccaccatgactttaatggcgctgggcatgttgccaaaagcgcatccgttgtcgctgggtatg
ctggggatgcacggcgtgcgcagcaccaactatattttgcaggaggcggatttgttgatagtgctcggtgcgcgttttgatgaccggcgattgg
caaaaccgagcagttctgtccgaatgccaaaatcattcatgtcgatatcgaccgtgcagagctgggtaaaatcaagcagccgcacgtggcga
ttcaggcggatgttgatgacgtgctggcgcagttgatcccgctggtggaagcgcaaccgcgtgcagagtggcaccagttggtagcggatttgc
agcgtgagtttccgtgtccaatcccgaaagcgtgcgatccgttaagccattacggcctgatcaacgccgttgccgcctgtgtcgatgacaatgc
aattatcaccaccgacgttggtcagcatcagatgtggaccgcgcaagcttatccgctcaatcgcccacgccagtggctgacctccggtgggct
gggcacgatgggttttggcctgcctgcggcgattggcgctgcgctggcgaacccggatcgcaaagtgttgtgtttctccggcgacggcagcctg
atgatgaatattcaggagatggcgaccgccagtgaaaatcagctggatgtcaaaatcattctgatgaacaacgaagcgctggggctggtgca
tcagcaacagagtctgttctacgagcaaggcgtttttgccgccacctatccgggcaaaatcaactttatgcagattgccgccggattcggcctcg
aaacctgtgatttgaataacgaagccgatccgcaggcttcattgcaggaaatcatcaatcgccctggcccggcgctgatccatgtgcgcattga
tgccgaagaaaaagtttacccgatggtgccgccaggtgcggcgaatactgaaatggtgggggaataagccatgcaaaacacaactcatga
caacgtaattctggagctcaccgttcgcaaccatccgggcgtaatgacccacgtttgtggcctttttgcccgccgcgcttttaacgttgaaggcatt
ctttgtctgccgattcaggacagcgacaaaagccatatctggctactggtcaatgacgaccagcgtctggagcagatgataagccaaatcgat
aagctggaagatgtcgtgaaagtgcagcgtaatcagtccgatccgacgatgtttaacaagatcgcggtgtttttcagtaaccgctcaaggcttg
aacaacatcgcgcttatcgttaaggtaagcgcgtatttttttacccgccaggacaagaccatgatcaccgttgcccttatagacgatcacctcat

cgtccgctccggctttgcgcagctgctggggctggaacctgatttgcaggtagttgccgagtttggttcggggcgcgaggcgctggcggggctg
ccggggcgcggtgtgcaggtgtgtatttgcgatatctccatgcccgatatctccggtctggagctgctaagccagctgccgaaaggtatggcga
cgattatgctctccgttcacgacagtcctgcgctggttgagcaggcgcttaacgcgggggcacgcggctttctttccaaacgctgtagcccggat
gaactcattgctgcggtgcatacggttgccacgggcggctgttatctgacgccggatattgccattaaactggcatccggtcgtcaggacccgct
aaccaaacgtgaacgccaggtggcggaaaaactggcgcaaggaatggcggtgaaagagattgccgccgaactgggcttgtcaccgaaa
acggtacacgtccatcgcgccaatctgatggaaaaactgggcgtcagtaacgacgtagagctggcgcgccgcatgtttgatggctggtgatg
aagacgttgttctcccgcttaattaccgttattgcctgctttttatcttctctgccgcatggttttgcctgtggagtatcagcctgcatctggttgagcgcc
ctgatatggcggtgctgttatttccgtttggtctgcgtctggggctaatgctgcaatgcccgcgcggatactggcccgtattgctgggcgcggagtg
gctgctgatttactggctaacgcaggcggtcggtttaacccattttccgttattgatgatcggtagtttactgacgttactgcccgtagcgctgatctcg
cgctatcgccatcagcgtgactggcgcaccttgctgttacagggggcggcgttaacggcggcggcgttgttgcagtcgctgccctggctttggc
acggcaaagagtcgtggaatgcgctgttgctgactttaactggcggcctgacgctggccccgatatgtctggtgttctggcactatctcgccaata
acacctggctgccgctcggtccgtcactggtttctcagccaatcaactggcgcgggcgacatctggtctggtacttgctgctgtttgttatcagtctct
ggctccagttgggattgccggacgaactgtcgcgctttacgccattctgtctggcgctgccgattatcgcgctggcctggcactatggttggcaag
gggcgctgattgcgacgttgatgaacgccatcgcgctgatcgccagtcaaacctggcgcgatcatccggtggatttattgctctcgctgctggtg
caaagtctgacagggttgttgcttggcgctggcatccagcggttgcgtgaacttaaccagtcgctgcaaaaggaactggcgcgcaatcagcat
ctggctgaacggttgctggaaaccgaagagagcgtgcgccgtgatgtggcgcgtgagctgcatgatgatatcggtcagaccatcactgctatt
cgtactcaggcgggcattgttcagcggctggcggcagataacgccagcgtgaagcagagcgggcagctcatcgaacaactatcgctgggc
gtttacgacgcggtgcgccgtttgttgggtcggttacgtccgcgccagttggatgatctcaccctggagcaggccatccgctcactgatgcggga
aatggagctggaagggcgcggtattgtcagccatctcgaatggcgaatcgatgaatcagcgttaagcgaaaaccagcgcgtgacgctgtttc
gtgtctgccaggaagggctgaacaacattgtgaaacatgctgatgccagcgcggtcaccctgcaaggctggcagcaggatgaacggttgat
gctggttattgaagacgatggcagcggtttgccgccgggttccgggcaacaaggttttggcctcaccggaatgcgcgagcgcgtaacggcgct
gggtggcacattacacatttcctgtctgcacggcacgcgtgtcagcgtttctctacctcaacgctatgtctaaggtttgatgatgttgccgtttctgaa
agcgcctgccgatgcgccattaatgactgataaatatgaaattgatgcccgctatcgctactggcgtcggcatattctgctgaccatctggctggg
ttacgcgctgttttacttcacgcggaaaagttttaacgccgccgtaccagaaatccttgctaacggcgtgctcagccgtagcgatatcggcctgtt
agcgaccctgttttacattacctatggcgtgtcgaagtttgtctccggcattgtcagcgatcgctcaaatgcccgttattttatggggatagggcttat
cgccacgggcattatcaacattctgtttggcttctcgacgtcgctatgggcgtttgccgtgctctgggtgctgaacgcctttttccagggctggggttc
accggtgtgtgcgcgtctgttaacggcctggtattcacgtaccgagcgcggcggttggtgggcattatggaacacggcgcataacgtcggcgg
cgcactcattcccattgtgatggcagcggctgcgctgcattacggctggcgtgccgggatgatgattgctggttgtatggcgatagtcgtggggat
tttctctgctggcggctacgcgatcgcccgcaggcgttaggtttaccggcggtcggtgaatggcgacacgacgcgctggaaattgctcaacaa
caagaaggggcagggttgacgcgtaaagagatcctcaccaaatatgtgttgctgaatccgtatatctggctgctttcgttttgctatgtgctggtcta
tgtggtccgggcggcgatcaacgactggggcaatttgtatatgtccgagacactgggcgtcgatctggtcacggcgaatacggcagtgacgat
gtttgaactgggcggatttatcggtgcgctggtagccggttggggctcggacaaattgtttaacggcaaccgagggccgatgaatttgattttcgc
cgccggaattttgctttcagtcggctccctgtggctgatgccatttgccagctacgtgatgcaggcaacctgcttcttcaccattggtttttttgtctttgg
cccacagatgttaatcggtatggcggcggcagagtgttcccacaaagaggcggcaggggcggcgacggggtttgtcggcttgtttgcttatctg
ggggcgtcgcttgctggttggccgctggcgaaagtactcgatacctggcactggagcggatttttttgtggttatctctatcgccgccgggatttccg
cactgctgttactgcccttttttgaacgcccagacaccgcgcgaagcgtgatgcatctcaccttttcacttcatatccggcaaaactaagaaattttc
caggttttgcctggacgctatctcaggcctgatttgctgctgatttttacaatgcatgcctcacgcaggtattcatttcaggagtaacccatgctggctt
tcttaaaccaggttcgcaagccgaccctggaccttccgctcgaagtgcggcgcaaaatgtggttcaaaccgttcatgcaatcctacctggtggtc
tttatcggctacctgacgatgtacctgattcgcaagaactttaacatcgcgcagaacgatatgatttcgacctacgggttgagcatgacgcagct
ggggatgatcggcctgggtttctccatcacttatggcgtgggtaaaacgctggtttcctactacgccgacggcaaaaacaccaaacaattcctg
ccgttcatgctgatcctctctgctatttgtatgctgggcttcagtgccagtatgggcagcggctcggttagcctgttcctgatgattgccttctacgcctt
aagcggctttttccagagtaccggcggttcgtgcagttactccaccatcaccaaatggacgccgcgtcgtaaacgcgggacattcctcggtttct
ggaatatttctcacaaccttggcggtgcaggcgcagcaggtgtggcgctgttcggggcaaattacctgttcgatggccatgtcatcggcatgttta
tcttcccgtcgattatcgcgctgattgtcggttttatcggcctgcgttacggcagcgactccccggaatcttatggcctcggcaaagctgaagaact
gttcggcgaggagatcagcgaagaggacaaagagacagaatctaccgatatgaccaagtggcagatctttgttgagtatgtgctgaaaaac
aaagtgatctggctgctgtgcttcgccaacattttcctctatgtggtacgtattggtatcgaccagtggtcaaccgtatacgcgttccaggaactga
aactctctaaagcggtggcgattcagggctttacgctgtttgaagctggtgcgctggtcggtacgctgctgtggggctggctctctgacctggcga
acggtcgccgtggcctggtggcctgcatcgcgctggcgctgattatcgccacgctcggtgtgtatcaacatgccagtaacgaatatatctatctg
gcttctctctttgcgttgggtttcctggtctttggcccgcaattgttgattggtgtggctgctgttggctttgtacctaaaaaagcgattggcgctgccgat
ggtattaaaggcacctttgcttacctgattggtgacagctttgccaagttaggtctgggaatgattgccgatgggacgccggtattcggccttaccg

gctgggcaggcaccttcgccgcgctggatatcgccgcgattggttgtatctgcctgatggcgatagtggcggtaatggaagaacgcaaaatcc
gccgcgagaaaaaaattcagcagttgacagtggcataaacgtaactggtgacttttgcccggcatgacgccgggcttttttattattccgtgactt
ccagcgtagtgaaggcaaacttctcgccatcaaatagcccctgactggttagttttagcgcggggatcactggcagagaaagaaacgccatct
gaataaacggctcatcgggtaacggaccgcattcacgggcggcggctttcaaggcgtcaatttgttccgccagcgactgcgccgtgtcggtgc
tcatcagcccggcaatgggtaacggcagatgactttgtacctggccgttacgtaccacgcacagcccgccgccatcctgaatcacctgattga
ccgccagcgccatctcttcggcactgcgaccgatcaccacaatattatggctgtcatggctgaccgtcgcagccagcgcaccttcattcagtcc
aaagccgccaagcaaaccacaagccggagccagccgttgcccgtaacgctcaagtacggcaataaagctcacatcatcgcgatcaaaac
cattttcgctgtagacgctggagtgggagtgcgtaatcaattcgttatggatgacgtcaatgacccgatagcgttttccgggcgtaaattgcaggg
caaagtcgctggcggaaactggctggcgggcaatggtgttgccatatggcggagcggattgtgccagtctcgccgactcttccgcctgtaaggt
ttgcgcatcaatcggctcgcctttcaccagtacctgctgcaccgtgaccttacgcgcatcgctcaacaggacgatatcggcctgcttgccgggtg
ccagtaagccgaggtgattcagaccaaagtggcgcgccgtcgaccagctggcgacgcgatatgccacatgcagcggcacattgtgttgttcg
atcaggcggcgaattaaggcatcgatgtgtccttcatgggcgatctcccacgggttacggtcatcggtacagagcatgcattgcgggctgttaa
attcgttgatcaacggtgccagcgcgttgagattgcgggcagcggacccttcgcggatcatcaacgacatgccgagttgtaatttccggcgtcct
tcttccagctgataactttcgtggcagttttcaatacccgcagtaatataggcgttaagttctttaccacccaaacccgggcagtgaccgtccagc
gtcaggtggcgaaatgcatccagtttatcgagcagcgcattctgcccgctaattacgccagggtagtccatcatttctgcaaggccggtaacctg
cggatggtcccgccaggcgagcatctgttcaagggtaaaactggcaccgttaacatcgcagccttccagggcgggtacgcaagagctgacc
tgtaagtactggttttgccttgcctgttcggcacagcgggcaaaccaggcgaatccggcttcgcccatcacgttgacgatttcatgagggtcgca
aataacggtcgtcaggccgcgcggcagggtagcggtttcaaaagtgaccggcgtcatcatgctggattcaatatgcaggtgagcatcaataa
accctggcaccgccgttgcgccgcgagcatcaatccgctgcaaagccggagcatcagtgtattctgcgccaacaccggcaatgtaacgtcct
ttaatcacaattgggccggaaatttctccgccattgatcaggtcgagaatagagacattatcaataatataatcggcaacagcgtcgccacggg
aaacggctaacaattcctggtattcagcccggctaatgtgatgaaatttatggttaatagaattattcatatttttctcattttgattttatcaatttgattatt
atttatcataaagaaacgtcgcgagattattaaagaatatccattaatgtgcaattgaaatgtgataattatcactgaaccgttgttgtcatttgtttta
caaaagcaagggattaaatctcctccaatggacaaaaaaatgaataatgacaataccgattacgtgagtaatgaatcagggacgctttcgcg
attatttaaactacctcagcatggaccaccgtccgcacagaattgattgcggggatgaccactttttaaccatggtgtacatcgtttttgtgaacc
cgcaaatcctcggcgcggcacaaatggacccgaaagtggtgtttgttaccacctgtttgattgccggtatcggcagtattgcgatggggatatttg
ctaacttacccgtggcgctggctccggcaatggggctgaacgccttctttgccttcgtggtcgtgggggcgatgggcatctcctggcagaccgg
gatgggcgcaatattctggggcgcagttggactatttttgctcacgctgtttcgtatccggtactggatgatctccaacattcccttaagtttacgtatt
ggtatcaccagcggaattggattatttatcgccttaatgggattaaaaaatactggcgttattgtcgccaataaagacacgctggtgatgattggc
gatttaagttctcacggcgtgttgttaggtattttagggtttttattataaccgtgttgtcatcacgtcattttcatgccgcggtgctggtttctattgtggtg
acgtcttgctgtggattatttttcggtgatgttcattttagcggcgtctattccattccgcctgatattagcggcgtcattggtgaagtagatttgagcggc
gcgttaacacttgaactcgccggtatcattttctcctttatgctgatcaacctatttgattcatcaggaacattaattggtgtaactgataaagcgggct
taatagatggtaacggtaaattccccaatatgaataaggcgctgtatgttgatagcgtcagttcggtggcgggtgcgtttatcggcacctcgtctgt
tactgcctatattgaaagtacttctggtgtggcagtcggtggccgcacggggctgactgcggttgtggttggcgttatgttcctgttggttatgttcttct
caccgctggtggcgatagttcctccttacgcaaccgccggagcgttaatctttgttggcgtgctgatgacttcgagcctggcgcgcgttaactggg
atgattttaccgaatcggtgcctgcgtttattaccacggtgatgatgccctttactttctcgatcaccgaagggattgcactcggctttatgtcgtactg
catcatgaaagtatgcaccgggcgctggcgcgatctgaacctgtgtgtggtggtggtcgcagctctgtttgcactgaagattattctggtggattag
taactggcgagggtttccagatatcatgagttctgattacgcaggagaactcatgatctggataatgctcgccacgctggcggtagtgtttgtggtt
ggttttcgggtgctgacatccggggccagaaaagcgattcgccgtctcagcgatcggctgaacatcgatgtcgtacccgtggagtcgatggtcg
atcaaatgggaaagtcagctggtgacgaatttttacgttatttgcatcgtccggatgagtcgcacctgcaaaacgccgcgcaggtgttgctcatct
ggcaaattgtcattgtcgatggtagcgaacagaacctgctgcaatggcatcggattttacaaaaagctcgcctcgccgcgccgattaccgacg
ctcaggtcaggctggcgctaggttttctgcgcgaaaccgaacctgaaatgcaggatattaatgcttttcagatgcgctataacgcgttctttcagcc
tgccgagggcgttcactggttgcattgaaaccgtttcgtcttatcagcctacagaccgcgcctgacctgtcgggtggataagacgttcacgccgc
atccggtaatctttgccctgttccgtttcgatcttaactccagctcaagcaacaccgtaaaagttgcaaaaacgttaaatgcgtcacacatttcaat
gttaaagtggtcggcttttcccctgaaacatgccacgggtaacaccatgagtgaatttattgccgaaaaccgcggcgcggatgccatcacccg
accgaactggtcagccgttttctcggtggcgttttgtgtcgcctgtctgattatcgttgagtttttgcccgtcagtttgttgacgccaatggcccaggattt
aggcatttcggaaggggttgccgggcaatcggtgaccgtgaccgcctttgtggcaatgtttgccagtttgtttattacccagacaattcaggctact
gaccgccgctacgttgttattttgtttgccgttttgctgacgctctcctgcttgctggtttcctttgctaactcattcagtttgcttttaatcggtcgtgcctgtc
tggggctggcgctgggcgggttctgggcgatgtcggcgtcgctgaccatgcgtctggtgccgccgcgtacggtgccgaaggcgctgtcggtga
tcttcggcgcggtttctattgcgctggtgattgccgcgccgttgggcagtttttaggcgagcttatcggttggcgcaatgtctttaatgcggcggcgg
tgatgggcgtgctgtgtattttctggattatcaaatcattgccttcactgccaggcgaaccctcgcatcagaaacaaaatactttccgcttattacaa

cgtccgggtgtgatggcagggatgatcgccatcttcatgtctttcgccgggcagtttgctttcttcacgtatattcgcccggtgtatatgaacctggcg
ggattcggcgtggatggcttaacgctggtgctgttgagttttggtatcgccagctttattggtacgtcgctttcgtcgttcattcttaaacgttcggtaaa
actggccttagcaggcgcgccgttaatactggctgtgagtgcgttggtactgacgttgtggggaagcgataaaatcgttgctaccggcgtggcg
attatctgggggctaacttttgcattggttcccgtcggctggtcaacgtggatcacccgctcgctggccgatcaggcagaaaaagccgggtctatt
caggtggcggttattcagcttgctaatacctgtggcgcggcaatcggcggttatgcgctggataatattggtctgacttcgccgttgatgttgtccgg
cacattgatgttgctgactgcattgttggttactgcaaaggtgaaaatgaagaaatcctgatatcaggtaaagaagcgttggtcagtagttgttgct
gttcttacatatccgggcattctaccttacccaacgcttcccaataattgctctgattattacgttccatcgccacgatggcctcgcgtatatgttgtttat
tgttttcatctgacaacattaacttttcccacgcttcatctgataatgacgcctgtggcgtgcaaattttacgtaaatcttgtaataccgtggttttctctttt
gcggactgcagtgagctaaaaggcgactcggcgtatacgatgcctggcatagcgaaaaaagtgaaaataagcagtagtgtcgttggtttcat
ggattcaaccttttacaaatgtgtatcgaatgaagcataagtgtagtttgctttcatcgctctcatcatgctgtacgaaacgcgcttgcgtggtcagta
agaagtgccagactttatattccacttttattcctttttattcttatcgatagcgtttcgtttttttaaaccgcagcgaccttaccgctatagtcaggtaatca
ttaataaaaggataaaaaaatgaaactgacaacacatcatctacggacaggggccgcattattgctggccggaattctgctggcaggttgcg
accagagtagcagcgatgcaaaacacattaaagttggcgttataaatggcgcagaacaagatgtcgcggaagtcgctaaaaaggtggcaa
aagagaaatatggcctcgatgttgaactggtaggatttagcggttcgttgctaccgaacgatgcgacaaatcatggcgaacttgatgcgaatgt
cttccagcatcgaccctttcttgaacaagataatcaggcgcatggttataaactggtagcggtgggaaatacgtttgtcttccctatggcgggttatt
ccaaaaaaatcaaaaccgtggcgcaaataaaagaaggcgcgacagtggcgatccccaacgacccgaccaaccttggtcgcgcactttta
ctgttgcaaaaagagaagctaattactctgaaagagggaaaagggttattacctaccgcgctggatataaccgataatcctcgtcatttgcaga
ttatggaactcgagggggcgcagctcccgcgcgtactggatgatcccaaagttgatgtagcgattatcagcaccacttacattcagcagaccg
ggctttctccggtgcacgacagcgtatttattgaagataagaattcgccgtatgtgaatattttggtggcacgggaagataataagaatgcagaa
aacgtgaaggaatttctgcaatcttatcaatcacccgaagtcgctaaagcggcagaaaccatttttaacggtggcgcggtgcctggctggtaatt
ctcacttatgccgcgccggacgatttatttttctggcgcggcgacgggaatccattgaaatcaaaatcaccgatgacagaatcagcagcgttcc
cagccagtccggtaatgtgaacgtgatccccagcaacagcaaagagagtagcgcgctacttaatggttctgcacagctcaaaatgctggctttt
ggaccgccaattaattgtgctcctttcaggtacagactaaatgtcagggacgtaccaatgaccaccaaataaaaaaacgccagaatcaaact
gccgttaacgacaaagtttgttccttgtctggcataaaaaggcaacagaatcagaccgccaatcagcatactccagccgacgactggtaacgt
gccatagcgggcaattagcgttgagggataggtggtatagaatgcagcagcaaatgccgaggcaatgccccagaacaacgcggcaggag
agatcgataatgacgtcggattaccgtgtgtcaccaataaaaaagtcccgaccagcgatgtcaaaatagcgcagaaaaccagaatgcccg
ggcgcgatttacgcaccagtgagaaccaggcgacgataatcgtcggtgagaggaattgcagcaccgttgccgtggctgcgttcgatttttcgat
ggttagcaaaaaagtgagctgtacagttagcgcgccaaccacggaaaaaatcagcaggctaatggcatctttatgattgttaataatagaaaa
gattttatcgccatgaacaaatgacagcgtcagtagaatcaaaccggcgaatatcaaacgcgtcatagtcaaaaactgcgacgacatctggc
tttgctccatgatgtattgcgcgcagacccctgaacttccccacaacacggcggcaatcagaacgttcagcatcccctttctggtggaacccattt
ttccctctgcatactgttttgtcgtttatgtatgcaggcatgatagcaaaaatggcgaggatggctctgtttgcgccacgccgatttttcttagcccag
actggtgagctaaatatctttaataaacagcaggcaaatcatcgcaatacccaacatccctatcgccagccagaacaacgcatggtagctcc
agatttcaaccataattccgtcaacagagccggcgataatccagccgacgcgtgaagtatttgcatataacgttgtcgcagagcctatttttttcag
gcatcaagtcctgaaaataaagcatgccgataccacagagaataccaaggaagatggcattaagaatttgcaattccagctcaaccgccgg
agtcgtcgccatgagtacgctggcgtaaaaacacattccactcacgatagcaatgagcattaatagtcgcttgccaatacgtttcatgtaatagc
ctgcgattaacatcatcggaatttccagaccggcagcgataccaatcatttctccagtcagtttatcggttagatgcagttcatcaataataaatag
cggcatatttatcatgtagagattattcgccgcccacatcattgaacagaccacaaaaagtagccgcgtatcccgcttcctgtgggtggagggta
aaatttctacgggttgggtaacgacaggaatatttctttgtattgatggcaaaaacaaccagactatcagtccgcaaacaacaaatgcgatggc
agcggtgagatacatcactttaaaactaaatcccattgccagttcataagcgagcggtggcccgataacccaggcaagcgagatctgagcac
gtaaaaatgtactgaacatgaccgtctcacggcctgttctgtcggcgtgttcacgggcgagggcgaacatttgcgggtttgcggtggaagcaaa
actactcagaagtacgcccgttgagaggagaatgaagtagttgcgattccacgcaaaaagcgtgcaggccagcactccaaataagcaaca
tagcagaatcagtaatttacggtcgccttgtttatcggagtgccttgccagaaattgactgaccagaattcccataatagcgctaccggtgaaga
aaaaacctaccattataggacgggctttcagttcatctgcgaggaatatacttagggtaggagtctgaagagcgcccgcaatacccgtcagaa
aggcgacaagtaaaaatgccgcggcagtgagatcaagtatttttgatggagtggtagccgtttttttgcattcttctgaatcaccgggcgaaaaaa
tataactgagttacaaataagtaccagtcgaatgagtattgtattaagaatttttgagtatcgctgatacaaccagaaagcaatgctagcgcaata
ttatagggccagcaattgagattattagtaaaagcatgttaagaattaattaccgaatttatatattaattatagtaaggcaatgagctatttttgata
gtaaaaataaaacaagttgatttgatatattaaatgaacaaattaatcttgatggcagtctgattattggtgaagtttatagtcagtttttttcgccagc
ggttaaagaaatagaatagtcgtataaaaaagggtgttctggggcgagtaaaacaacatcttcaccttaatggcgatacagttggctgagagt
cagcagggggcaaatcacctgcaaataaacacggcgcatactggcgtgaatatcgcggatgcgttcgaggacagaagaaccgacagg
cggattcttcagccgctcatcgtccatcacaaaaccttagaccaggtattcctgaagccgattgaatattgagcagtagaaaatatctggattga

cagtattacaaggggttggagaacgtcaggagaggcattttggcggaagatcacaggagtcgaacctgcccgggaccgctggcggcccca
actggatttgaagtccagccgcctcaccggagacgacgatcttccgcgcctcgattgctacatggaggcggggcgcattatagctacttccttg
agtttctacatcccccagatcagtttttccgcctaatacacgcagcgtattatcacgaaagcattatagttcatataaaacccatattttacaatgat
atatgagcggaattaacgctaccacgatcacataaaacaagaatcataaattaataaccagatatcggaatattcgctctcacagggatggttt
acaaaatgcgttatcggtctcagcacccctatagcatcaaggaaaagcagatgaagagtgaagtgttgtccgttaaagagaaaattggttatg
gcatgggagacgccgccagccacattattttcgataacgtaatgttatatatgatgttcttttataccgatatttttggcattcctgccggttttgtcgga
accatgtttttggtcgctcgtgcactggatgcgatttccgatccttgcatggggttgttggccgatcgaacgcgctctcgctggggtaaatttcgtccg
tgggtactgtttggcgcactgccattcgggatcgtctgtgtactggcctatagcacgccagatctcagtatgaacggcaaaatgatctatgcagc
aattacttacaccctacttaccttactttataccgtcgtcaatatcccttactgcgcattgggtggtgtaatcaccaatgacccgactcagcgtatctc
gctgcaatcctggcgttttgtgctggcgaccgcgggaggcatgctttctactgttctgatgatgccactggttaatttaattggcggtgataataaac
cactcggtttccagggcggtatcgcggtcctttccgtggtggcattcatgatgctggcattttgtttcttcaccactaaagaacgcgttgaagcacca
cctacaacaacgtctatgcgggaagatttacgtgatatctggcaaaacgaccagtggcggattgtcggtttactaaccattttcaatatcctggcg
gtgtgcgtacgcggtggggcgatgatgtattacgtcacatggattttgggcacgccggaagtgtttgtcgcttttctcaccacttattgcgtgggtaa
cctgattggttccgcactggcaaaacctctgaccgactggaaatgtaaagtcactatcttctggtggacgaacgccctgctggcagtgattagcc
tcgcgatgttctttgttcccatgcaggccagcatcactatgtttgtcttcatcttcgtgattggtgtgttgcatcaactggtgacacctatccagtgggta
atgatgtccgataccgtcgactacggcgagtggtgcaatggtaaacgcctgaccgggatcagttttgctggcacgctgtttgtgctcaaactggg
gttggccttcggcggcgctcttatcggctggatgctggcttatggcggatatgatgcggcagaaaaagcgcagaacagcgccacgattagcat
cattattgcgctattcacgattgttccggcgatctgttatttgctgagcgcgattatcgctaaacgctactactcactcacgacgcacaatctgaaaa
ccgttatggaacagctggctcagggtaaacgccgttgccagcaacaattcacctctcaagaagtgcagaactaaggaacggcaatgaaaat
tagcgatggaaactggttgattcaacctggcctcaatttgattcacccgcttcaggtgttcgaggttgaacagcaggataatgaaatggtggtcta
tgctgcccccccgtgatgtgcgtgaacgtacctggcagcttgatacgcctttatttacgttgcgctttttctccccacaggaaggtattgtcggtgtgcg
gattgagcattttcagggggcgctgaataacggtcctcattatccgctcaatattttgcaggacgtgaaggtcacaatcgaaaacacagaacgtt
atgctgagtttaaaagtggcaacttaagcgcgcgtgtcagcaaaggtgagttctggtcactggattttctgcgcaacggcgaacgtattaccggt
agtcaggtgaaaaataatggctacgtgcaggacacgaataatcaacgcaattatatgtttgagcggcttgatcttggcgttggcgaaacagttta
cggtctgggagagcgctttactgccctggtgcgcaatggccagacggtagagacctggaaccgggacggcggcacaagtactgaacaggc
gtataaaaatatcccgttctacatgactaaccgtggttatggggtactggtcaatcatccccagtgtgtctcttttgaagtgggatcggagaaagtct
ccaaagtgcagttcagcgttgagagtgaatatctcgaatactttgttatcgacggcccgacgccgaaagcggtacttgatcgttatacccgcttta
ctggtcgtccggcgctgccgcccgcgtggtccttcggcctgtggctaaccacttcatttaccaccaactacgacgaagcgacggtaaacagctt
tatcgatggtatggcggaacgcaatctgccgctgcatgttttccactttgactgtttctggatgaaagccttccagtggtgcgattttgagtgggacc
cgctgactttccctgacccggaagggatgatccgccgcctgaaagcgaaaggactgaaaatctgcgtctggattaacccctatatcggtcaaa
aatcccccgtctttaaagagttacaagagaaaggctatttactcaaacgcccggacggttcgctatggcagtgggataaatggcagccaggtc
tggcgatttatgactttaccaatccggatgcctgcaaatggtacgccgacaaactgaaaggtctggtcgcgatgggcgttgattgctttaagacc
gactttggcgaacgtatcccaactgatgttcagtggtttgacggttccgatccgcagaaaatgcataaccattatgcgtacatctacaacgaact
ggtgtggaacgtgctcaaggacaccgttggtgaggaagaagctgtcttgtttgcccgctcggcctccgtcggtgcgcagaaattcccggtacac
tggggtggcgattgttacgctaactacgaatcaatggcggaaagcctgcgcggtggtttgtctattggcctttcaggttttggcttctggagccacg
atatcggcggctttgaaaataccgctccggcgcacgtttacaaacgctggtgcgcgtttggtttgctctccagccatagccgtttacacggtagca
aatcttatcgtgtgccgtgggcctacgatgatgagtcctgtgatgtggtgcgcttcttcacgcaactgaaatgccgcatgatgccgtatctgtatcgt
gaagctgcgcgtgcgaacgcgcggggtacgccgatgatgcgggccatgatgatggagttcccggacgatccggcttgtgattaccttgaccgt
caatacatgttaggcgacaacgtgatggttgcgccggtgttcactgaagcgggcgatgtgcagttctacctgccggaaggtcgctggacacac
ctgtggcacaacgatgaactcgacggtagtcgctggcataaacagcagcacggcttcctgagtctgcccgtttatgtgcgtgataacactctact
ggcgctgggcaacaacgatcaacgtcccgattacgtgtggcacgaaggcacggcattccacctcttcaatctgcaagacgggcatgaagcc
gtctgtgaagtgcccgctgctgacggatcggtgatctttactttaaaagcagcacgtactggcaacacgattactgtgactggtgcgggcgagg
cgaagaactggacactgtgcctgcgcaatgttgtgaaagtaaatggtctgcaagacggttcgcaggctgaaagtgagcaggggctggtggtg
aagcctcaagggaatgcgctgacaattacgttgtaatctattgatggaatgccgggtacgtctgagtcgtacccggcgagattgatgtttaccgc
accggctccgtcgatgtaacttcaccgctggaaacgatgccagcattcatggtttgagtcacttgctgctttgcggcgttcacggcatgcaattgc
ccgctaaccgtaggtttcaacgggacattggcctgaatatcgccactggcggtaagctgaatattgccgtcgccagtcaacggtaattcaggcc
atccccactgttgcaaaatattcaccggcacaccgcgtccattcaggctgatatgtgtctgacgttgtggcgtttgtgaaacactggcggtggcttc
cagaatgcctttttcagtaaatgcactcagttcgctgatattcaccgtgctgctgttggcggtcagcgccagcgacgggcgacgaacatcaaca
cgattgaatgtcgcggcggcggcattcagattcgccgagccactccagacgccccatttatgatcggtaaccagcgtcaggttggcaccgtaa
ccatcgagcgcggtgagctgccacgggaagtcagggtcgatatcaatgatcagattgcggctggcgctaaatctcttcagttgcaggctgttta

accaaccgggtgtcgtttccatccacaactgttgccagtttttcggcaaggtatattccagcccggcaattgccgcatcatcaaggatcaacgtttt
cccgtcacgcagccagttccctgacgttctgaccatacccccttcccagcggctggtgaactggcgcagcgctacgccctgcggggaaaattc
cgtgtttataatcgggtcaaataaatgcagcgaaccataaatgaactcgctagcgttcatcgacagtttgccttcctgtgtctgccagtcatctttact
gaaggtcatgttgcgcaagctgagatcgaggtcggtcaccgcccagtccggaccttgcaaacgagcatcgatcacttccaggcgaccaatct
gcaacgacgggacagagcgtaatggcgcaaagaagtcggttagcgatttttcgctttgtagacgtatatccgccatgcgcaggttttccacttgc
cagctgccgtcggcgttacgctgcgcgtttccggttaatgtcccgcgggcgatgtcggcacccaggttagtcagcgtaacgcgatcgttatcaat
actgccttcaatcagtacattggtggcaggaacatcattgagcgaaagcgatccggcactaaactgaatctgcgccttcgtacccagcacttta
ccggcttttggtgaccacggaactacgccgccatttacccgctgcgcgctcagtttccattcgctattcgggctattaaacgccatatcacgcagtt
gcagacgatcggctttgaacggtagcggcgcggtctggtcggtgagattcagcgtcccgttttccagcaggatggtatcgacatggcgtggttc
ggtcagttgccgactgcttagcgcaatgtcgacacttttggccaccagggtcgcgggctggccatcacgaccaaacgtgacgttctccagcac
gatatgagatggcgcggaaaaacggtgatccatcgccccgaaggccagatgatagtcgctattctcggaaacccatgcgctgatatgttctgc
tccccagcgggtttgcagaagaaaatagaggccagcgatcgccactaacagagcgatgagaatgtagagaagcagcttcccaataaatttc
atggtcttccatcccgtgaaatgcacataagggagttatgcacgatttacgcgcaatcctcaaggcgggaatggttaaagaggtgagactgcg
gcggtaattatcttaccgccgccagtgaattactgtttttctggtgggaaaatcagattcagcacgatggcagtaataccgcccgcggcgatccc
ggaggagagcaggtttttcagccattcaggggcaaactgcaaaatcagcggctgctgagacacgcccagaccaaccgccagcgacagcg
cgataatcagaatcgcccgacggttcagcggctcacgagaaacgatacgcacaccggaggcggcgatggtgccaaacattacaagcgttg
cgccgcccagaaccggttctggaatgtgttgtacaaaaccgctcactgccgggaacagacccagcacgatcaacatcagcgcgacgacaa
aaccgacatagcggctggcaacaccagtcaactggatcactccgttgttttgcccgaagcaggagttcgggaaggtgttaaacaccgccgaa
acaaacgagttcaggccgtttgccagcacgccgcctttcaggcgtttcatgtacagcggaccggacactggctgttcggaaacgtcagaggtc
gccgtgatatcgccaatggtttccagcgaagtgatcataaagaccagcatcagcggcagcagcagactccattcaatgccaagaccgtaata
gagcggcgttggcaccataatcagttcttgcgtcataggttcgttgctttctggcaacatgcccataaaccacgccagcgcatatccggccgcca
tcgcaattaccagtgaggccacgcgtaagtaagggttacgttgacggttaagcaggataattaaggctaagaccacgcctgccagcagcag
atttttcggtgcgccgaaggtgttatcgctcatggctgcgtaaccgccgccaatggacgttaacccaacctgaattagcgacaggccgataatc
atcaccacaacgccagaaaccagcggcgtaataatgcggcgcgccagatgcagaacgcgggagatcaccatctcggtgcaacttgccag
catcaacgtgccgaacaaagccgccatcatggtaggaacatcagcaccaccggttttcagcgcggtaccgcccataatcagcggggcaac
aaagttgaagctggtgccctgaatagacaacagcccggagccaaccggaccccaggccttaatttgaataatcgatgccacaccggaggc
aaacagcgacatactaataatgtgttgcgtgtcttgtgccggtaaacccagcgcctggcagattaatagcgctggcgtgatcaccgcaacgaa
catcgccagcagatgctgacaggcggcaaacagggtttgaggaagcggcggacgatcttcaagacggtaaatcagttcgctgttttgagtctg
cgcaaccggttgcgcattttctgactcgagggtggaaacagacatcgaaaacaatcccgtggtagcaaagcgggcattttagctgaccgaaa
gagaaaagcaaacgtttgccacatcctcatctaaatatttaaatgagataaatgcactttttatataacttttggtatttttctgcttaaaatccatgcc
atttgttcattaacagctgagaatttgacccaggaaagcataactcgatacccgtcatagttcatgaagttgccactgcgcgatgaagtatgacg
agtatgaaagagtgatgcggatacaaaggagtaactatgtttcatctcgatactttagcaacgcttgttgccgcaacgctgacgttgctgctcggg
cgtaagttggtccattccgtctcctttttgaagaaatacaccataccggaacctgttgcgggtggtttgttggtggcgctggcgctactagtactgaa
aaaaagcatgggctgggaagtcaactttgatatgtccctgcgcgatccgttaatgctggctttcttcgccaccattggcctgaacgccaacattgc
cagtttgcgtgccggtgggcgtgtggttggcatcttcttgattgtggttgttggtctgttggtgatgcaaaatgccattggcattggtatggctagcttgtt
agggcttgatccgctgatggggctgttggccggttctattactctttccggcggtcacggtacgggcgctgcgtggagtaaattgttcattgaacgtt
atggcttcaccaatgcgacggaagtggcgatggcctgtgcaacgttcggtctggtgctgggcggcttgattggcggtccggtggcgcgctatct
ggtgaaacactccaccacgccgaacggtattccggatgaccaggaagtcccgacggcgtttgaaaagccggatgtgggacgcatgatcac
ctcgttggtgctgattgaaactatcgcgctgattgctatctgcctgacggtggggaaaattgttgcgcaacttttggctggcactgcttttgaactgcc
gaccttcgtctgtgtactgtttgttggcgtgattctgagcaacggtctgtcaataatgggcttttaccgcgtctttgagcgtgcggtatccgtgctgggt
aacgtaagcttgtcgttgttcctggcgatggcgttgatggggctgaaactgtgggagctggcttcgctggcgctgccgatgctggcgattctggtg
gtacagaccatcttcatggcgttgtatgccatcttcgttacctggcgcatgatgggcaaaaactacgatgcggcagtgctggctgcgggtcactgt
ggttttggcctcggtgcaacgccaacggcaatcgccaacatgcaggcgatcactgaacgctttggcccgtcgcacatggcgtttttggtggtgc
cgatggtcggtgcgttctttatcgatatcgtcaatgcgctggtaattaagttgtatttgatgttgccgatttttgccggttaaccgatgaagcggcggta
gaagtgccgccgcaacaaagacaaatgcctgatacgcttcgcttatcaagcctgcgtattgattcataatttattgaatttgtagggtgatgaatcg
catccggcaggaaggtagggtaacctgaaatggcggtcttctcactgccgccttttacgcattcgagtaacgttccgtctccggcatccagcgttc
tatcagggcttttgcctgttgtgggtaacgttcgtgaatatggcgtgccaggcgctgaacttccgggatcatcgcctgatcgcgcagtaaatccgc
cactttaaattcagcattacccgtctgacgcgtgcctaacaattcgccagggccgcgaatctccagatctttttgcgcaatcacaaaaccgtcgtt
actgtcgcgcagcacttgcaggcgaatttgcgccgttttagaaagcggcgttttgtagagcagcacgcagtgagaagccaccgcgccacgac
ctacgcgcccgcgcagctggtgtaactgcgccagacccagacgctccgggttttcgataatcatcagactggcgttaggcacatcaacgccg

acttcaataacggttgtggcaaccagcaggtgtagctcaccttgtttaaacgacgccatcaccgcctgtttctcggcaggtttcatccgcccgtgta
ccaggccaacgttcaactctggtagcgccagtttcaactcttcccaggtagcttccgccgcctgcgcttccagcaattccgactcttcaatcaacg
tacaaacccagtatgcctgacgaccttcagttatgcaggcgtggtgcacgcggtcaatgatgtcggtacggcgggtatcaggaatagcgacc
gtagtcactggcgtgcggcctggcggcagctcatctatcaccgaggtatcgagatcggcatacgcagtcattgccagcgtgcgggggatcgg
cgtggcggtcatgatcaactgatgcggatggaagccctgctgctggcctttctcccacaatgccagacgctgatgcacgccaaaacgatgctg
ttcgtcgataatcaccagcgccaggccgttaaactgcacctgttcctggaagatggcgtgtgtaccgacaatcatctgcacctgaccgctggcg
atggcttcctgctgtgccagccgtgctttacctttctgcttaccggcgagccagcccacttcgataccgagcggtgcaaaccagttgcggaagtta
ttggcgtgctgctcggcgagtaattcggttggtgccatcaatgctacctgtttgccgtgggcaatcgcacgcaacgcggcgagggcggcgacc
agcgttttaccggaacctacatcgccctgcaccagacgcatcatcggcacatccagcgccatatcgcgctcgatctccgccactacgcgtgcc
tgtgcgcccgttggcttgaacggtaaggcggcgaggagtttatttttcagcgtgtcattggcgctcagcggctgggcatgaaaacgctgtgctcc
ggcacgtaaggctaacatgctgaggttgtgcgccagcagttcttccagaatcagacgacgttgcgccggatgctgcccggtttccagatcgcta
agctgtagcgtcggtggcgggcggtgcaaagtgcgcaacgcttccggtagcgtcatcattccttgtgacagttccggcggcaggagttcttcaat
ggcgcaggtgtcgagcagatccagcgcctggtcggttaatttacgcagcgtggcctgctttacgccttccgttgttggataaaccggcgtgagcg
tttcctgtaattctggcgtgctgagatcgccctgcacgcggtattccgggtggatcatctccgcaccatatttaccgcgctttgcttcgccataagcc
agtacacggcggcccgccgccaggctatttttcattgccgcgctgaaattgaaaaagcgcatggtgaggatgccggaaccgtcgctgatctgg
caggtcatcatccgccgaccgccgaaggagatattgcagttcagcacttcgccttccaccgtggcataaacgcccggcagtagttctccgatg
gggtagagatgggtgcgatcttcgtagcgcaggggaaggtgtaagagtagatcctgcacggtatgcaggttgattttcgccagtttgttactaagt
gctgcgccaacgcccgttagggaactgagtgggacagcatctaacaggcgacctttcatggcacttaccctgcagcctgcatagtagcccac
cagtcggcatcagcttcgatctcgccttgctgattgacgtggggataaggcaggcctttgcgttttgcgactttcgccagcaccggatagccgcct
tcaaacaacaggcgttgttgctctgcttccggcaacatgctgttttcacgcaggtacatgcctgcattttgccgctgacgctgggcttcgtaaagaa
tgagggctgaggcaacggaaacattcagcgactgcaccatgccgatcatcggaatgatgatgtcctgatccgccagggccaatgcttcctgc
gtgatgcccgttttctcctgtcccatcaaaatgcaggtcgggcgagtgtaatcaatttcgcggaaatcgacagcgttatcagaaagatgggttgc
cagaatctgcatgccctggcctttgagatgagcgacggcatcgccaatggtgcggtgtgttttcacctgtacccagctgttactacccgccgctgc
cgaagccatggtgcgcatgcggctaccaggccagacggcgtgaacttcatgtacgccaacggcatctgcggtacgaataatcgcagaaac
gttatgaggtttgtggacctgctccatgcagacggtcagatcaggctgccgcctggcgagcatttcgcagatgcgtgcataacgtgttgggttcat
aaaacattaatttcggtttcgggtgactttaatcacgtctggcatcacgcggattttgcgcatgatattcgccagatgcacacggtcacgagcggt
cagacgaataaaggcgctgtagacgcgaccatctttctcttccgtattcaaactttgaatattcgaagtcgtggtgttaattgccgccgtcaggtttg
ccagcgcaccctgatgattgaacatctccaccttgatttcggtgatgaactcctgcgccgtctctttatcccattccacagccataaacttctctggct
ctttctggtagccacggatattacggcaggattcatggtggatcaccagacctttaccggggctgacgtgggcgataatcgggtcgccaggaat
agggcggcagcatttcgcaaaggtgatcagcacgccatcggcacctttaatgggcagatgtccgtggctttgggttgccggtggaatggaggc
gtccccatgttgcagatttttcgcgaccaccacgctcattgcgttaccaagtccgatttctgccagcagatcgtcaagcgttgccagcttcatgcga
tccagctcgcgctgaatattttcctgcgggatttcattcagcttacggctaccacccaaagcatggttgagcagacgacggcccaggcttacag
aatcatcacgcttgaggtttttcagcaactgacgaattttggcgcgcgctttcgagctaacgacaaagttcagccaagcggcattcgggcgagc
gcccggagcggtaatgatttcaacggtttgaccgctggtaagcggctgcgacagcgggtaaggctggcggtcaacgcgtgcgcccacgcag
gcatgaccgatatcggtatgcactgcataagcgaagtcgacgggcgttgcaccggcaggcagctcgacaatgcgcccttccggtgtgaaaa
cgtaaatctcatccgggaagagatcggatttaacgctctcgataaattcaaacgaactaccggcgctctgttgcagctccagcaggctttgcatc
cagcgctgggcgcggatttgtgcggtagtactggtttcgccgtgctctttataagcccagtgcgcggcaacacccatctccgccatctggtccata
tcttcggtacggatctggacctcaaccggcacaccgtgcgggccgatcatcgaggtgtgcaaagactgatagccgttcgcttttggaatggcga
tatagtctttcacgcggcccggacgcggcttgtacaggctgtgcatctggcccagcacgcgataacaggtgtcagaatcattgacgatcacgc
ggaaagcgtagatgtccatgatcgagtgaaaacgctgctctttgagcaccattttgcagtaaatcgaataaagatgcttctcgcgaccactgac
gcggcacggtattcccgcttcctgcaaacgcccttcgatttcagaaagaatcttctggatcatctctttacggttgccgcgcgcggctttcaccactt
ctttgattacgcgataacggttgggatacagcgcctcaaaacccagctcttcgagttcggttttaatgtggtggatacctaaacggtgcgccagc
gggctataaatttcgagagtttcacgggcgatgcggcgacgtttgtccgggcgaagtgagcccagcgtgcgcatgttgtgggtacggtcggca
agtttgatgaggatgacgcggatatcctgcaccatcgccataatcatcttgcgaaagttttcggcctgcgcctctttcttatcgcggaacttgagttta
tcaagtttcgacaccccctctaccagctcggcgacgcttttaccaaaaagctgttccatatcctggtaggtggcgggagtatcttcaatcacgtcat
gcagcagcgccgccatcagcgtttcatagtcgagtttcatctcggccagaatgcaggcaaccgctaccgggtgcgtgatatagggttcaccgc
ttgaacgtgtttgcccctcgtgagcatcacgtgcaacgagatacgcctgccgcagacgcttgatttggtcttccggcaggtaggtttgaatcagttg
attcaggctttcaaacagatacaagggcgacccgctttgtgattaacgacgaccttcagcaatagcggtaacggcttgtaattcagcggcttcct
gctcttgctgttcctggcgttcgcgaacgtcgaggatctggttgttgatcagaccttcttcgatttcgcgcagcgcgattacagtggttttatcgttttctt
ccggtaccagcggatcctttccgcctacctgcatctgacgagcgcgacgcgcggcgaccagtaccaggtcaaaacggttaccaattttctcta

cagcgtcctgaacagttacgcgtgccatacttaaaaagctccacaggtgaagaaatgactgggcatgatactgaaatcaggttcagtctgcca
acaatttgctgattaaagcgtcatgacgctgcttttggcggctcatgcgcagacgttcggcgcgaataatggtcttcaaatcggtcaacgcggtat
cgaagtcatcattcacaatcagataatcatattcggcgtaatggctcatttctgcaacagcttgcgccatacgctttgcaatgacctcttcgctgtcc
tgaccgcgaccgcgtagacggcggtccagttcaattttggacggcggtaaaataaagatactccgcgcgtgcggcatcttctggcgaatttgct
gcgcgccctgccagtcgatatcgagaaaaacatcgacaccggtcgccagtacttgctcaatggcctcacgcgaagtgccatagtaattacca
aaaacttctgcgtgttcgaggaacgcatctctgctaatcatttctttaaattcatcatgattaacaaagaaataatgttcaccgtggacttcaccagg
acgcggttggcgtgtggtgtgtgaaacagaaacctgggtgtcatacaacggttgggtttttaataaagcctgaatcaggctggatttacccgcgc
cactgggggcagaaacaatataaagcgtgccttgagccatgagtatctttcgtatgtgattagcgaaataaagcctacatacgagcttattatac
acggcgcggccatgtgacgtagcccttgctcacactttttgcgcaaagttgttgcgttttgcgtagctgcttccgcatttgcctgtcattcactgcaat
aaagacgattttcaggaagcgtcctcgcagactccgtttttgccactcggctttgctttcatcacgttgtatatctgcctgactatctacagcggaggt
gatgatgaaagtatggatggcgatattaataggtatcttgtgctggcaatcatctgtgtgggcggtctgtccggcctggtcgccagccagagcac
aggaagaaatttcccgcctgcaacagcaaataaaacagtgggacgatgactactggaaggaaggaaaaagtgaggtggaagacggtgtt
tacgatcagttaagcgcccgtcttacgcagtggcaacgctgctttgggagcgagccccgcgacgtgatgatgccgcctttaaatggtgcggttat
gcatccggttgcccatacgggtgtacgcaaaatggtggataaaaacgcattaagtctgtggatgcgagaacgtagcgatctttgggtgcagcc
aaaagttgatggcgttgcggtaaccctggtttatcgggacgggaaactgaacaaagcaatcagtcgcggtaatggcctgaaaggcgaggac
tggacgcagaaagttagcttaatttccgctgtgccgcaaaccgttagcgggcctttagccaacagtacgcttcaggggggaaatatttctccagcg
cgaggggcatatccaacaacaaatgggggggaataaatgcccgcgcaaaagttgctggcttgatgatgcgccaggacgatagcgacacgct
gaattctctgggcgtttttgtctgggcatggccggatggaccgcagttaatgtctgatcgtttaaaagagctggctaccgcaggttttactctaacgc
agacgtatacccgtgcggtgaaaaatgctgatgaagttgcgcgcgtacgcaatgagtggtggaaagcggaattacccttcgtcaccgatggc
gtagttgtacgagcggcgaaagagccagaatcccgcccattggctaccgggccaggcagagtggctggtggcctggaaatatcaacctgtag
ctcaggttgccgaagtgaaggcaattcagtttgcggtgggtaagagcggtaaaatatcggtggttgcgtcactcgcacctgtcatgctggatgat
aaaaaagtccagcgggtgaatattggttccgtcaggcgctggcaggagtgggatattgcgcctggtgatcagattctcgtcagccttgccggtc
agggtattcctcgcattgatgatgtggtgtggcgcggtgcagaacgtacaaaaccgacaccgccagaaaaccgctttaactcgttgacctgcta
ctttgcttctgatgtttgtcaggaacagttcatttcacgcttagtctggctgggggcaaaacaggttcttgggctggatggcattggtgaggccggtt
ggcgcgcgctgcatcagactcatcgctttgaacatatcttttcctggcttttattaacgccagagcaattacagaacacgccggggatcgcgaaa
agtaaaagtgcgcagctatggcatcagtttaatctggctcgtaagcagccttttactcgctgggtgatggcaatgggaataccgctaacccggg
cggcgcttaatgccagtgatgaacggtcctggtcgcaacttttatttagcacggagcagttctggcagcagctgccggggactggatcggac
gcgccagacaggttattgaatggaaggaaaatgcgcaaatcaagaagctgggcagttggctggctgcccagcagatcacaggttttgaacct
tagtgtccttcgtggctgtagtaaaagaccggtaatcccagcttaaggcgtagcgccagtaagcgggcaaaaaagccgaataccagggtgg
agataattaccacatcatggttagaaacatagtgttgcagcgcgatgtacagcacggcggaggcaaatgagacaccggcatacaactctttct
ggaataccagtgggatacgtttacagaacatatcgcgcaaaacgccgccaaacacgccggtggttaccgccgcgacaacggcaataattg
ggccgtgccccatatccagtgcaacctgtgccccgatgatagaaaagaccaccagtccgagcgcatcgagcaccagaaacactttgcgca
ggtaaggcattacaggagcaacgatagtggtgagtactgcggcggtagcgacgataatcacatattccgggtgtttgacccagccgagcgga
tagtggcccagcagaatatcgcgcactgaccctccgccaattgcggtggcggtcgcaataataattacgccaaatgtatccatgcgccgtcgtc
cggccgccagcgcccccgtcatggcttcggcagtaatgccaaccaaatacaaaatgtgtaacagcatggttctctccctaaaaatctgtctggc
agggtagcgatttgtgcgcattgtcacgattgagattttttaaggggaggggaaattgataacttgtagttatgtgaaatcgctattttctgtagcaga
gatgcattcttctgacttcttgtctgagaaaatgcataagaaaaaaataggttaaaactgagggggaaaagaaaggtgcaatatttagccattgtg
ttcactgcacctggaccttggcttacgcatcgggtgtctcaatcaacaaaaagagtttttagtttctgctatttcttctctgtaatttttacactgttttccaa
ctgcttgatgctgacctgcggggtcggcaactcctcaggcatggttccgccaagttcctgaatggtttgcctcaccttgcgacccacgtcaaagtg
tgtggtgtttgcttgctgttttgaattcacgccatcccgcttgagtttttcttctgtttgggtagctcgaaagagattagccgccagttctgttgaacccat
atgatccaggatcttctgattctttttcagccccttccgctgatgaatagctttctgatctaatccaccatacagcccctggtaaccatgattctgaaat
atggcgaaatcagtagctgtcgctaccccagcttgctgtgctgcctcaaccaattgtttgttatgttctttcaactcatttctcagaaacagacgctttt
cgtcttcacgaagttgcttaaatgcttcatcgtcagcaagctcctgccgtcgggtctggatagcaaaataagtctgccctgccgcaatgaccggtt
tcgcagggtcgccgttttgtactaccaggtaacaggcatagcgggagagtacgatgtcttttaatgctcttttggcatttgagccaatttgaccatat
cgaggatctcctcgaaatggtcatcaattgtgtgaccactgttctcacaggcttctttggcgcgcgttaatacaggtataaagtgacgatactctga
ataacccagtagtttcccaagttgtcttgctgaccattgttctgctccgtttgcattaatcagttttatctcttcaaaaggttgatgatgttcgttcattgccc
actccatgtgatatcaattgcaaagaaagtaaatctgtaactgtatttatatacagttgtgagtatctcacgttctcagtgtgctgggcaaaaaaac
atcgcaaaatctggagcaggcacgcaaaacagaattgaatgttgcggggaagaaaaatgccgctctgtttgagtggcggcatttgtgaaggc
ggaaaagtaagaattggcgttacgagttattcgatgttctgaatctgctcgcgcatctgctcaatcaacactttcagctcgatggcggagtttgtca
cttcggcattgatagacttcgacgcaagagtgttcgactcgcggttgaactcctgcatcataaaatccagacgacgaccaaccgcttctttttctt

cagaatgttgtaggtctctttgacatgcgcttcgaggcgatccagttcttcggcaacgtcaattcgttgtgccagcagaaccagttcctgctccaga
cggttgttttccagttgcacctgagcatcttccagcttcgcgaccagacgctcacgctgccattgcaggatttccggcatatgggagcggactttg
accacttcggcggtgacgccttccagacgctgctcgatcaatgctttcagtgcctgaccttcggtttcgcgcgcgacaataaagtcgtccagcgt
accatccagcgccgcgagaatttcagcggcaatggcgtcaagatcctgctcctgggctgccatcacgcccggccagcgtagaatatcaacc
gggttgatttccccttcgtcactctgcatttttacccagttcgcggcagttaccagctgtttagccagtttttcgttgaggatcagctcaccttgcgcgct
aacatctggctcatagcgcagggtacattccactttaccgcgcgtcaggcgagaacgaatacgctcgcgaacgacaggttcaaggctacgg
aactgctccggcagacgaaagtaagtttccagataacgctggtttaccgagcgcatttcccaggttgcgctcccccattcacccttgatttcacgc
cgggcgtaggcggtcatactgcggatcatagacgttcctgtttataaaaggagaggtggaaggattatagccatcgatgccttgtcaggatagg
aataaccgccggaagtccgtataatgcgcagccacatttgtttcaagccggagatttcaatatgcgtccagcaggccgtagcaataatcaggt
gcgtcccgttaccctgactcgtaactatacaaaacatgcagaaggctcggtgctggtcgaatttggcgataccaaagtgttgtgtaccgcctctat
tgaagaaggcgtgccgcgcttcctgaaaggtcagggccagggctggatcaccgcagagtacggcatgctgccacgttctacccacacccgt
aacgctcgtgaagcggcgaaaggtaagcagggtggacgcacaatggaaatccagcgtctgatcgcccgtgctcttcgcgcggcagtagatt
tgaaagcgctgggtgagttcaccattacgctggactgcgacgtgcttcaggctgatggtggcacgcgtaccgcgtcgattacgggtgcctgcgt
ggcgctggtagatgcgctacagaagctggtggaaaacggcaagctgaaaaccaatccgatgaaagggatggtagccgcagtttctgtcgg
aattgtgaacggcgaagcggtttgcgatctggaatacgttgaagactctgccgcagagaccgacatgaacgtagtgatgaccgaagacggg
cgcatcattgaagtgcaggggacggcagaaggcgagccgttcacccatgaagagctactcatcttgttggctctggcccgaggggaatcga
atccattgtagcgacgcagaaggcggcgctggcaaactgatttttaaggcgactgatgagtcgcctttttttgtctgtagaaaagtaagatgagg
agcgaaggcatgaaaccatatcagcgccagtttattgaatttgcgcttagcaagcaggtgttaaagtttggcgagtttacgctgaaatccgggc
gcaaaagcccctatttcttcaacgccgggctgtttaataccgggcgcgatctggcactgttaggccgtttttacgctgaagcgttggtggattccgg
cattgagttcgatctgctgtttggccctgcttacaaagggatcccgattgccaccacaaccgctgtggcactggcggagcatcacgacctggac
ctgccgtactgctttaaccgcaaagaagcaaaagaccacggtgaaggcggcaatctggttggtagcgcgttacaaggacgcgtaatgctggt
agatgatgtgatcaccgccggaacggcgattcgcgagtcgatggagattattcaggccaatggcgcgacgcttgctggcgtgttgatttcgctc
gatcgtcaggaacgcgggcgcggcgagatttcggcgattcaggaagttgagcgtgattacaactgcaaagtgatctctatcatcaccctgaaa
gacctgattgcttacctggaagagaagccggaaatggcggaacatctggcggcggttaaggcctatcgcgaagagtttggcgtttaaagaaa
ctcgccggatgaaaagtcatccggcgtcatattactgcaactgtgccgcaattagcggccagcgggcgtcaaaatcatccgtcgggcggtattt
aaattcgctgcggacaaaacgtgacagcataccttcacagaaggccaggatctggcttgccagcagggtttcatcggtggtgtaaccttcacc
ctcacgcattctcttttcacgcaatacctggcgcagctgcgcttcaatacgctcgaacagctggttgatgcgcccttgcaggcgatcctgttcaaa
cattagcgcatgaccagtgaggatgcgggtcaggccaggattacgctcaccaaaaccgagaagcagcaacacaatcagacgcaggcgc
gctgtggtgtctttctcatctttcagaatcaggttgatgcgagtaatcaggctatcttcgataaactcaatcaggctatcgaacatgcgggtcttactg
gggaagtggcgatacagtgccgcttcggaaacgccgacagaggcggccagttttgccgtcgtgatacgttggcttccatcgctggattccagc
atcagcgccagagactgaagtatttcctcgcgacggttccttttcgcagtttgtttttctgccatgttacaaaataccccctgaaaataagcacttgcc
aggcggcacccacgctatgaccgcaaacgaaatgtttgcggctatgttatgacgttattcggatgcgtatgtgttactgacgaccagagtgacc
aaagccgccttcaccgcggtcggtggcgtcgaaatcttccaccagattaaattcagcctgtactaccggaacaaaaatcatctgggcgatgcg
ttcgccaggttgaatggtgaagctgtcctgaccacggttccacacggaaatcatcaactggccctgatagtcagaatcgatcaatcctaccagg
ttaccaagcacgataccgtgcttatgtcccaatccggagcgcggcagcatcattgccgccagtgaaggatcggcaatatgaatcgccagccc
ggtcggaaccagcgtagtgtcacccggagccagttctacggcgtcgttgagacaggcacgcaggtcaagtccggcagagccagaggtggc
ataagtcgggagcggaaattccttcccaacgcgcgggtccagaatcttaacgtcgatttttttcatcataacgggtcacgatctcgtcgagtaata
attggccaaggagctctttgcgctcaagcggtaagactttatctccgtcctgccagaaaaggtgtaatgcgttgttgtcgctgttaaatccttgagtt
ggctgggaaacatcgttcgcgcagatcagatcaaggtttttacggatacgtttttgccgggcgtattcttccacattatttgtttcggcggcaaatcc
aacgacgtagggtcgatggtcttttagtgcggcaacgcctgcgacgatatcggggtttttaaccatttttattgttaattcatcaccctgcgtggcctg
ctttttgattttctctggggccacggtagctgcgcgataatccgccacggcggcgcagccgataaaaatatttgctgctgtacagaagcattcac
ggcggcttccatttccagcgcggtcatcacatcaacacgtttaacaaacggtggcgtcggtagtgaaaccggacctgataccagcgtgacgtt
cgcgccacgacgggcagcggcggcggcgatagcaaaacccatcttgccggagctgtgattagagatataacgcaccggatcgagcggttc
acgcgtcgggccggcggtaatcataatgttcagatgtttcaggtcgttgacgggcgaaaaatgcgctaccgccatatccacaatggttaacgg
atcgagcattcgcccaggaccgatatcaccacaagcctgactgccactgtctggcccccagatgagcaaaccacgggaagcaagcacctc
taaattatgctgcgtggcagcggcacggtacatctgctggttcatggcggggagcacggctacaggcgcaggtgtagccagacaaatcgtcg
ataccaggtcattcgccattccggcagcaacacgggcaatcaaatctgccgtggcaggggcgagaatcactaaatcagcccatttacccagc
tcaatatggcccatagcggcttcggctgccgggtccagcagactgtcggaaacgggataaccagaaaccgcctgcaagctaagtggggtga
taaaggcttttgccgcttcggtcatggctacgcggacgtcggccccgcgatcgcgcaaacgacgcaccagttcaggggttttataggcagcaa
taccgccgctaacgccgagaacgatttttttaccggccaggctcatgatgatttttcctgtagggtgacaccagaagttggcgattttatcacaata

cttttgttgtcgtgccttcacctgagattcactttgcgaggcgctttccaggattgaaaactggccgtcgatttaacgaaacggctatgacaggatg
cgagcaccacaaaggaggtgaaggtgaaaaacaattcacagctgttgatgccgcgcgaaaaaatgctgaagtttggtattagcgccttaac
ggatgtcgagctgctggcgctatttctgcgtaccggaacgcgcggtaaagatgtattaaccctggcaaaagagatgctggagaatttcggctct
ctttatggcttgttaacctctgaatatgaacaatttagtggcgttcatggaattggcgtggcgaaatttgcccagttaaaggggattgctgaactggc
gcggcgttactacaacgtgcggatgcgtgaagaaagccctttactcagcccggagatgacgagggaatttttacaaagccagctcacgggtg
aggagcgggagatctttatggtgatctttctcgactcccaacaccgggttataacgcataggcgtctttttccggcacgctaaaccatgttgaagt
ccatcctcgggaaattatccgcgaagcgataaaaataaacgcctcggcgctgatccttgcacataatcacccttcgggttgtgctgaacccagt
aaagcggataaactcattactgaacggataataaagagttgtcagttcatggatttacgcgtgctcgaccatatcgtgattgggcgtggagagta
tgtttcttttgccgaacgcggctggatttaacccgctatgcgcgatccttcgggatctttgtctgttcgggacttgagcacatcgctgagtcagcgtat
actacgccacctttgagaatctcgggtttggcatttgggcctggcaatcgagagttcacatagaactgcgatgaccggctgtaaagcctgacg
aggcgccaataccccatacgaagctcgagctaatttgatttttggagaatagacatgtcccgagtctgccaagttactggcaagcgtccggtga
ccggtaacaaccgttcccacgcactgaacgcgactaaacgccgtttcctgccgaacctgcactctcaccgtttctgggttgagagcgagaagc
gttttgtcaccctgcgcgtatctgctaaaggtatgcgtgtaatcgataaaaaaggcatcgatacagttctggctgaactgcgtgcccgtggcgaa
aagtactaagtacttagaggaaataaatcatggctaaaggtattcgtgagaaaatcaagctggtttcttctgctggtactggtcacttctataccac
tacgaagaacaaacgtactaagccggaaaaactggaactgaaaaaattcgatccagttgttcgccagcacgtgatctacaaagaagcgaa
aatcaaataattctcgctttgatgtaacaaaaaacctcgctccggcggggtttttttgttatctgcttgcccccatattgactgcatctgttcattcctgga
gatgctatgcctgaattacccgaagttgaaaccagccgccgcggcatagaaccgcatctcgttggtgcaaccattcttcatgcagtggtgcgca
acggacgcttgcgctggccggtttcagaagagatctaccgtttaagcgaccaaccagtgcttagcgtgcagcggcgggctaaatatctgctgct
ggagctgcctgagggctggattatcattcatttagggatgtctggcagcctgcgcatccttccagaagaacttccccctgaaaagcatgaccatg
tggatttggtgatgagcaacggcaaagtgctgcgctacaccgatccgcgccgctttggtgcctggctgtggaccaaagagctggaagggcat
aatgtgctgacccatcttggaccggagccgcttagcgacgatttcaatggtgagtatctgcatcagaagtgcgcgaagaaaaaaacggcgatt
aaaccgtggctgatggataacaagctggtggtaggggtagggaatatctatgccagcgaatcactgtttgcggcggggatccatccggatcg
gctggcgtcatcactgtcgctggcagagtgtgaattgttagctcgggtgattaaagcggtgttgctgcgttcgattgagcagggtggtacaacgct
gaaagattttctgcaaagtgatggtaaaccgggctatttcgctcaggaattgcaggtttacgggcgaaaaggtgagccgtgtcgggtgtgcggt
acgccgattgtggcgactaaacatgcgcagcgggcaacgttttattgtcggcagtgccagaagtaattcatgcgcgccggatggcataccatc
cggcataaacgctacgctaacttcgccatcagcgcctgatggacattctccggcaggaaatgggtgacatcgccctgatggcgcgccacctct
ttcaccaacgatgaagagataaacgaccactctttcgacggcatcagaaacacactttccagttccggcattaagtggcgattcatatgcgcca
gctgcatttcatattcaaaatctgccaccgcacgcaggccacgaatcagcaccgtagcgtgttgattacgggcgaagttcgccattaaatcact
aaacccgaccacttccacgttccccagatgcgcggttgcctgctgtgccagtgccacacgctcttccagggtaaacatcggtttttttactggggct
ggcggcaatcgccagaataacgtgatcgaacatctgcgtggcgcgcgtcacgatatcgatatgaccattggtaatgggatcgaaagtacccg
gataaatcgcccgtttttgcataacaacctcaatgcgttttcggtggcaggtaaggttccagcagttgaagcagacgctgtagcgcgccctggttt
tgatacagtacttcaacggcatgacggccatagaaactacggtaatcggcgtcggtgagtaaagaggaaacctcttttgcaagcgtagtggca
tcggtaacggtaatcagcccgcttgcctgctccagccgcgcgcaaatgtctttaaagttaaaagtatgcggccccatcaataccggaatagcgt
gtgcggcagcttccagcggattatgcccaccacgttcaaccagtgaaccgccaacaaaggcgagatcggcaatgccatacagtaacatca
actcgcccatcgtatcgccaaccacaacctgcgtgctggtggaggggacttcccctgaagagcgtgtgatatagcttagtccagcctggcgga
caaggttaatcgcatccgggaagcgttccggatgacggggtaccaggatgagcaataaattcgggaattgctgtaacaatgcctgatgtgcg
gcgatcaccacactctcttcgccttcgtgagtgctggtggcaatccataccgggcggtgtggtgcccactggcggcgcagcgtcacggctttag
cagccaactgcggcgttacagaaatatcgaatttcaggctaccggtaacggtcacctgattattttttgcgcccagcgccacaaaacgtgcacc
atcttcttcattttgcgcagcaatcagcgtaatacgacgcagcaagcgacggacgaatttacccagtttggcataacctgcggccgagcgggc
agagagtcgcgcgttagcgatcaccagcggaattttacgtttatgtagcgccgcaatcaggttaggccatagttcggtttccataatcaacacca
gtttagggtcgactttattcaggaaacggttgagtgcatcgggcagatcatacggcagataaacgtgctgaacatccttcccgaaagccgattgt
acgcgctccgaaccggttggcgtcatggttgttacggtaatcggtaaatcaggataacgatgacgcagcgcgcgcaccaacgggattgccgc
cagagtttcaccgacggagacggagtgcagcataatgccgcctggttttagcggatggcggtaaaaaccgtaacgttcaccccagcgttttcg
ataggccggagccttacgtccgcgcacccagagccgtatccagatcagcggctgaataaggtagagaagggcggtgtaaagcaattcgag
catagtaaatagctgacttatggatgtgctggggattctatgtatttagctgtggctttaccattacttttcccgtttttgacttaaatagcttcagtttggtc
tgatctgccgctacatcttcatttttttttgtatttttatgcgattcattgaaactcggccccattttcaaatctacataggccgtactgacattatcgaaatg
ctattttttatctatttgatttttatgattaaagtatattttgtgtataaaaatcattcgggtcggattgctgcgaaagaaatgatacactagcacgtcaaa
gtaagtgcgttatcagtattcaggtagctgttgagcctggggcggtagcgtgctttttttctgcttaacttaaccagacaatcacacaaaagagtcgc
tagtggaaaagccatttcgaaaaatcctggtcataaagatgcgatatcatggggatatgttattaactactcctgtcatcagtacgctcaagcag
aattatcctgatgcaaaaatcgatatgctgctttatcaggacaccatccctattttgtctgaaaacccggaaattaatgcgctctatgggataagca

ataaaggtgcgggaactttcgataaaattaaaaatgtgctttcgttgataaaaactctgcgtgcgaataattatgacctggtcattaatcttacggat
cagtggatggtggcgctgctggtacgttgtttacctgcacggatgaaaatatcgcaactttatggtcatcggcagcatggtatttggaaaaaaag
cttcacacacttagcgccaatacacggtacacatattgttgagcgtaatttatcggtccttgagccattaggtattaccgatttctacaccgacaca
acaatgagttacgccgaagattgctggaagaagatgcgccgggaattagatgccctgggcgtaaaagatcattatgttgtcatccaaccgaca
gcgcgtcagatatttaagtgttgggataacgataaattttctaaggttatcgatgcgctgcaacagcgaggctatcaggttgtgctaacctgtggg
ccctcggcagatgatctcgcttgtgtagatgagattgcacgaggttgcgaaacaaaacccattactggccttgcaggtaaaacacgttttcctga
actgggtgcattaattgatcatgcagtgctttttattggtgtggattctgcgccgggacatattgcagcggcagtgaaaacgccagtcattagtctat
ttggtgcaacggatcacgtattctggcgtccctggaccgagaatattattcaattctgggcggggaattatcagaaaatgccgacccggcatga
acttgaccgcaacaaaaaatatctttctgttatcccagcggaggatgtgatcgccgctacggaaaagctgttgccagaagatgccccttcagct
gacaggaatgcacaattatgatcgtggcgttttgtttatataaatattttccatttggtgggcttcaacgtgactttatgcgcattgcatcaacagttgc
cgcacggggccaccatgttcgggtatatacacagtcgtgggaaggcgattgcccgaaagcatttgagcttattcaggtgccagttaagtcccat
accaaccatggacgcaatgcagaatattatgcctgggtacaaaatcatctcaaagagcatcccgcagatcgcgttgttgggtttaataagatgc
ctggcctggatgtttattttgccgctgatgtttgttacgccgagaaagttgcgcaagaaaaaggtttttttatatcgtttaacatcacgatatcgccatta
tgccgcatttgagcgagcgactttcgagcagggtaaatcgacgaaacttatgatgctgaccgataagcaaatcgccgatttccagaagcattat
caaactgaacctgaacgttttcaaattcttcctcccggtatttatccggacagaaaatacagtgagcaaatcccaaacagccgtgaaatttatcg
ccagaaaaatggcataaaagagcaacaaaacttattactgcaggttggatcagattttggccgtaaaggtgtagatcgctcaattgaagctttg
gcatcgttaccggaatcattacgtcacaatacgcttttatttgttgttggtcaggataagccgcgaaaatttgaagcgctggcagaaaaactcggc
gtgcggagcaatgtgcatttcttctccggtcgcaatgatgtgtcagaattaatggcagccgctgatttattactgcatcccgcttatcaggaagccg
cgggtatcgttcttctagaagcgatcactgctgggttacctgttttaacaacagcggtatgtgggtacgcgcattatattgcggatgccaattgtgga
acggtcatcgctgaacctttctctcaggaacaattaaatgaagttttacgtaaagcgttaactcagtcgccattgcgaatggcctgggcggagaa
tgctcgccattatgccgatactcaggatttgtatagcttgccagaaaaagccgcggatatcattacaggtggtttagatggttgaattaaaagagc
cgcttgccacactttggcgtggtaaagatgcttttgcagaggtcaaaaaactgaacggcgaggtctttcgtgaactggagactcgtcgtacatta
cgctttgaactgtccgggaaaagctattttcttaaatggcacaaggggacgacattaaaagagattataaaaaatctactctcattgcggatgcc
cgttttgggcgcagacagagagtggcacgctattcatcgcctgagtgatgttggcgttgatacaatgaagggcattgggtttggcgaaaaaggg
ttaaatccattaactcgcgcatcatttattattaccgaagatctcactcccacaattagccttgaagattattgtgccgattgggcagtcaacccgcc
tgatatacgtgttaagcgtatgctgatcgcacgtgtagcaactatggtgcgtaaaatgcatactgcagggataaatcaccgcgactgttacatttg
ccactttttgcttcatttgccatttactggccgggaagatgaattaaaaatttcagttatcgatctgcatcgggcacagatacgtgcaaaagtaccg
cgccgctggcgcgataaagacctgattggtttatatttctcatcaatgaatattggcctgacgcaaagagatatctggcgatttatgaaggtttatttt
gggatgcctttacgcaaaatattgtctcttgaacagaatttattgaacatggcaagcgttaaggccgagcgtattaaagagcgaacacaacgc
aaaggattataaaaatatttattagtatgtactggaactaactttattatgactatttattttataaattgggttgcagattatgaattaaaaatgattcag
tacctgaaaaaaaagtacaagataaaaaatataacaacaccaaaaaaatataactggattaataaaaaaatatcaaaaatagggatggat
aatgcatggctggggcgattattcatcaagcattacctcaatgatattaaaaaagatgacataattatcattaatgacagtgttgttaataaaggta
tcaataaacaaatattaaaaaatattaactgtcataaagttttgcttctaagaaatactgttggcgaagattttattcttgataacgcgaattatttcga
tattatatatgattttgagcatagatttatagggaatgaaaagatcaaggcaatagaacaattttttcctataggaatggatgaaattagaaattata
gtttatctgacaaaaataacagccagccaatatgtttttttccttggtcgtgataaagggcgtcttcaaataattaatgagttagccgagagattaac
aacactaggatgtaagctagattttaatgttgtcaaagataagacatcatctacgacttcaaagtatttgattgaaaaacaaatatcttatgaaga
gaatattagaagaacattaaatgctaatataattgttgatataaccaaagaaaaccaatctggctggactcttcgtatacttgaggcactatttttca
ataaaaaattaattactaataatataaatgtatttggttcagaaatctatagtgaatctcgctttttataataggtcatgatgattgggataagttaga
gtattttattaactcatctgttaaaccaatggattatgatagtttatataaattcagtccagataaaatgatgtcaacgattgtttctgattttatagacaa
ataaaaccgttaaaacagtgagttttactcaattaatgagagtttctatgaaaatagcgtttatcggcgaagctgtttctggatttggcggcatgga
aactgtcatccgtgatgttattactaccttcaggcaacaacatatccaaagtgaaatgtttttcttttgtcgtaatgacaaaatggataagggatggc
tggaaggtattaaatactcatgttcattttccaatattcgtttaggtttttttgcgtcgggcgaaacatattcatgctctaagtaaatggcttcaggagtac
caaccagatatcgttatttgtattgatgtgatctcctgcctgtttgccgcaaaagcacgtaaaaaatcaggaattgatatgccagtattttcatggcc
acatttttctttagatcacaaaaaacatgctgaatatatcacctgtgccgactatcatttagcaatcagctctggcattaaacagcaaatgattaatc
gaggtgtagcagaatcgacgataaatgttatttttaatcccgtcgaaactaaagactcagtcattcccgcgcccgaggaaggtgaaacggcaa
cgtttatttatgttgggcgtatgaaatttgaaggccagaaaagagttaaagacctgcttgatggcttatcacaagcgaaaggtaactggaaactc
catgtattgggtgatggctccgatttcgaaaaatgccaggcttatggtagagaattaaatattgatgatcgaattgtctggtatggttggcaacaat
atccctgggaattggtccagcaagatattgaaaaagtgagtgcgttattgctcacgtcatcttttgaaggtttcccaatgaccctacttgaagccttg
tcgtgggggattccttgtatcagtgccgattgtgtctccgggcctgctgacattattcaacctgatgtcaatggccatctgtatcaaccgggagatat
tgctggttttgtaactcttctaaataaatatatagcaggtgaaattcatatcgaacatgaaaaaaattcctgcgtcgatcgatgaattttaccaatcga

agtattacgatagattacataaggtaattatttctgctatttcccggaggaaataatgcagcaggtgtttttccaggaaactgagtttcttaattcagtt
attgattatgatcataaagtagaaacagaaaatctttgtctggacatcgcttatggaactgacaaaaactttctatttggttgcggaatttcgattgctt
caatattaaaatataatgaaggaagtcgattgtgtttccatatttttactgattattttggtgatgatgatcgtaagtattttgatgccctggcgttgcagt
ataaaaccagaattaaaatatatctgattaatggtgatcgactgcgctcattgcctagcactaagaattggactcatgcaatatattttcgttttgttat
tgcagattactttattaataaggcgcctaaagtcctttatctggatgcagatatcatttgtcaggggactattgagccactaattaatttctcatttcctg
acgataaagtcgcaatggttgttacagaagggcaagctgactggtgggaaaaacgcgcccattcgttaggtgttgcgggaattgctaaaggtt
actttaactccggtttttttattgattaatactgcccaatgggcggctcagcaggtttctgcacgagctattgcaatgctaaatgagccagaaataatc
aaaaaaataacacatcctgatcaggatgtgttaaatatgttgctggcggataaacttattttcgctgatattaaatataacacccagttcagcttaa
attatcaactcaaagaaagctttataaacccagtaaccaacgatactatttttatccattatatcgggccaaccaagccctggcatgattgggcct
gggattatcctgtatcacaagcctttatggaggcaaaaaatgcttcgccatggaagaatacggcgttgctgaaaccgaacaatagcaatcaatt
aagatatagcgcaaaacatatgcttaaaaagcatagatatctaaaaggatttagcaactatctttttttattttattgaaaagataaagcattaaaac
tgggaaactataaagtaatgatataagggtagcattgtggactcatttcctgccatagagatagataaagttaaagcctgggattttcggctagct
aatataaatacttctgaatgtttaaatgttgcctatggtgtcgatgctaattatcttgatggtgtaggtgtttccatcacatcaattgtcctaaataatcga
catattaatcttgatttttatattattgctgatgtttataatgatggttttttcaaaaaattgcaaagcttgcagagcaaaaccaattaagaatcacgtta
tatagaattaacactgataagctccagtgcttgccttgtacacaggtctggtcaagagcaatgtacttccgtttatttgcatttcaattattgggtttaac
gcttgatcgtttgctttatcttgatgcagatgttgtttgtaaaggcgatattagccaactattacatctgggtttaaatggagcggttgctgctgttgttaa
agatgttgagccaatgcaagaaaaggcggtatccaggttgtctgatcctgaattacttgggcagtactttaattccggtgtcgtttatttagacttaa
aaaaatgggctgacgcaaaactaacagaaaaagcgttgtctattcttatgagtaaagataatgtttataaataccctgatcaggatgttatgaat
gtcttactgaaagggatgacattatttttgcctcgtgagtacaatacaatttatacaattaaaagtgaattaaaagacaaaacacatcaaaactat
aaaaagctgattacagaaagtactcttcttattcattatacaggtgcaactaaaccgtggcacaaatgggcaatttatccatcggtaaaatactat
aaaatagctttagaaaattccccctggaaagatgactctccacgagatgcgaaatcaattattgaatttaaaaaaagatataaacatcttttagtg
caacatcattatatctcaggaattatagcaggagtctgttatctttgccgaaaatattaccgtaaataacatttaactggtttattatgattcagaaga
gcaagatcaaagacttggttgtttttaccgatgaaaacaattcaaagtacctcaatgtattaaatgacttcttgtcttataatataaatatcatcaagg
tttttcgttctattgatgatacaaaagttatgcttattgataccgattacggtaaattgattcttaaggtttttttctccgaaagttaagcgtaacgaacgttt
ctttaagtctctgttaaaaggtgattattacgaacgccttttttgagcaaacccaaaaagtacgaaatgaagggttaaatacactcaatgacttttatt
tattggctgaacggaaaaccttacgttttgtccatacttatatcatgatcatcgagtatattgatggcatagagttgtgtgatatgcccgatattgatga
tgcgctaaaaaataaaattcagcaatcaattaatgccttacatcaacatggcatggtttctggcgacccccatcgtggtaacttcattataaaaaa
tggtgaggttcgaattatcgatctctccggaaagcgtgcttcagcgcagcgtaaagcgaaagatcgtattgacttagagcgtcattacggtatta
aaaatgagattagagatctaggctattatcttttagtatatcgtaaaaaaatgcgcaattttatgcggcgtttgaaagggaaaccagcgcgctaa
aaaaaatcccacaattacatgtcttcaccaggacagttttaagcggagttatttatttcacaggtttatcatgaagaatattagatacattgacaaa
aaagatgttgaaaacctgattgaaaataaaatatctgatgatgttattatttttctttcgggccctacatcgcaaaaaacacctttgtcagtattacga
acaaaggatattattgctgtcaatggttctgcgcaatatctgttaagtaataatatcgttccttttatatatgtacttacagatgtccgatttttacatcag
cgtcgtgatgatttttataaatttagccagagaagtcgttataccatagtaaacgttgacgtttatgaacacgcttccaaagaagataaactttatat
cctccagaactgcctcgtattacggtccttttaccgtcgggaaaaaggtggttttataaagaagattaaatttaatattttgagacagattcacaaa
gaactgctgatctctgtaccgttgtctaaaaaaggtcgtctggttggattttgcaaggacattagtcttggttattgctcatgccatactattgcctttgct
gcaattcaaatcgcatattcacttaagtatgcgcggattatttgttctggtcttgatttaacgggtagctgttctcgtttctatgatgagaataaaaatcc
catgccctcggaattaagtagggatttattcaaaatattgccatttttttcgttttatgcatgataatgtaaaagatattaatatttataatttatcagatgat
acagctatttcttacgatgtaattccatttattaaacttcaagacatcagtgcagaagaatcaaaagatatgacaagaaaaaaaaatgcaatatag
aacttcaaccgattcttatgctaattaatcatcctgaaactaaaataatatggtataaaaatgcgcttaggaacttttcacaaaaagaaaagatttt
atataaacaaaatcaagattaatttcctttctttttttatttcgtaataaaataaataatcaaattacagatccagcacaagttaaatcatgccttattatt
catgataataataaacttggtgatctaattgtattaagttcgatttatcgtgaactttatagtaaaggggttaaaataactcttctcacaaatagaaag
ggtggggaatttctctctaataataaaaatatatttgagttctgtattaaagaatcaaccggttttcttgaaatgcttactctatgtaagcatctgcggg
atttacagttcgatattgtattagaccccctttgaaactatgccctcatttaagcatagcttaatattatcaagtttaaaagattcttacattctgggttttga
tcattggtataagcgttactattcattttaccatccccacgatgaatgtttaaaagaacatatgagtacacgagcaatagaaatactgaagcatatt
tatggtgagggtaaattttccaccaactacgacttgcatttaccagtagatgttgaagataaaataaaagaatttattggagatacaagaatagtc
attattaatccattaggtgcaaaaaaaaatatgccgtcttacgtttgagcaaataaaagttatatatcaagaagtgaaaacacactttgaaaattat
cggattatatttaccgggttaccgcaagatttattgacaataccaatacttgaaatcgaaacactaccatttgatgagtttatttataccgttgcgttg
accaagtatagtgattttgtcatttctgttgatacagctcttgttcatatcgctgcggcttatcataaaccaacgcttgcattttaccctaattcacgtact
ccggaatatccctcgcatttaatttggtccccgaatcatcataaatctatacaaatagtatccccaacatatacggtaaaagatatcgatactgaa
actttaactaattcagtaaagagattaagttgtatagataagaagtgagttttaactcacttcttaaacttgtttattcttaattaattgtattgttacgatta

ttaatgacgagtaagaggactatagcggatataatgataattggaatgctgcgtgcccatatgatcacatcactgagtccaatccccacaatgc
caagcgttaatatcaacaaacccaaagctcgtttttatatgctatataaaatagtgaaaaatagaggaaaagtgtggaaaaaattcccatcag
acctttcagtgaccctgcttcaattatctcattatgtagatgtacgttagaaaactccaatgcccctcttagcctattgtgttctgcaactaacaaattc
atactttcagcgcgtgactctgctgatctaaatgaaaaaggtgactttatgaatatatttaaaccaatttcgtacattgccagtcttgcacctaggga
agtaacactattagcattggtataactgtttaagtcatttaatgcttcattatagcgattctgtattggtttattaaatataataacaatgctagctaatat
agcaattagtagaacaatagaggaagtgaatttcttgggtgatttattataataagctattaatgcagcaacacaaattatagggaacaggagta
gggttgctctggtttgtgttagcgccagaacataaagtaccgcgcaactatttaataaaaataaaaaaggatgatttttcttagtataaagaatcgc
aacgccactaactatccctattagcattgttgaatatgctgctcctgttgccgttcctacaccaaaagaaatgcggtcattttcatgaatgctattaat
atacattgcatatccagcaattagaaatgacagagaatacaaagtgtataatacactctcttttttgattttagctggctagttagtgtcaagaaaa
caataaaagaaccaaatataaatattttggcagtatttaaataactatggtaagtagcacgaaatggcgaattatctactttaaacgcagaatac
caaattaaatcaagcaagcctattaaaaatatagaaaggggaagaataaggtttttttatattataattttcttgtctgccacgtaaaattagtgaca
ataagcaaactatagcagtgatattatatatttttatgctgaaagtattatcgacgaatgcaattatcattgaaaaaaaacataatatataagttatta
tctctaaaatcattgatgatttcagagtgtaaggtttcaatgaatgaagtttaaaggatgttagcatgttttacctttataatgatgataacttttccaaa
actgcttgactgttgaggttaattaaattttctcttggagccctacataccatctgattcttcccatacccaccaattaatcccggatcggttggtccat
aaaccgtgatattgggtctatccagtgccgccgttaaatggcttaaccccgtatccaccgacactacaaatttagccccggccagcacgcggg
caacgccttccagactcatcttcggcaatacttcaacataagcaaatccttccgccagtcgtttcgcccgttcttcctcatgcggcgcgccccacg
gaagtttaatccgtattcctgaatcagccagtaaaccaatcaattctcgccagtgttcttccggccagtgtttatcatcacgggtcgtcgcatgaag
aaatacggcatattcgccagcatctgtaggcaggttcgtcagaaaatgctgtgcgatagcataatcgccctgggtttgcggtttgctatagccca
aacttttggcaaacagttcgcgggtgcgttctacggcgtgctgctgttttgcaatatgatgcttacgattgtaaaacaggctggctaaaggttcgcg
agcggtttgccagtccatgccatgctttacgccatgcgccagacgcgtcaccagcgccgcgctttttaccagcccctgagcgtcgataactgcgt
catagttctctgcttgtagcgcttcacgaaacgctttgcgttccgcttttatgggggccgagaaccaggctttacgccagcgacgtattgccacag
gaataactcgctcaacggcagcgtgccaggaaggaatctgtgcgaacccttcttccaccacccagtcaaacttaatccctgggattgcctgctg
ggcatcagtgagtgcgggcaacgtatggagaacatcgcccatcgacgatgttttaacgatcaaaacccgcatccgtcaggcttcctcttgtaac
aatagcgcgttgagttcttccagtacgcgctggggagtaatgtcgattaagctctggtgataaccctccgcagcgtcacctttacgcactttgtgat
agccggtaatcaaacggatcacgcgcgctttatgggatagcggcggtgtgaagtccgggctactcggaccatacagggcaaccagcggac
gattgagcgccgccgcaacatgcatcaggccagaatcgttagtgacaatggctttacaggctgcaatcaggataaccgcttgatcaagctgtg
tttcccccgccaggttccgacaccatgcctgctgctcggtattcaaagcggcaagaatctcattgcccgcttcatgatctttcgccgagccaaaca
gaaccacctgataaccttcatcaatcagctgctttgccagctccgcatagtggtagtgtggccagcgttttgccggaccaaactccgcacccgg
gcaaaaaccaatcatcggacgttctgatgaaagcgaaaattgattacaggtatatgatttttcaccttcgctcacctgcaactgcggccataaca
atggctgcggcagatcttgtgctgtgcgcataatgcctttgtcataggccagcgctatatagcgttccaccattagcggccaggcttctttatcgag
cacgcgtacatcgttgagtaaaccgtagcgcatctcgccgcgccagccggtgcgatgaggaatacccgcgaagaaaggcactaatgcaga
tttgaaggagttgggtaagacgtaggcgcggtcgtagcgcttttcacgcaggctatgacccagtttgcggcgttcgccgatttccagcgctccgtg
accgagaggcataggaatagcttcgttaacttccggcatccgcgataataatggacggcaccatgccggtgccatcacatcgattatcgcctg
gggatagcgcgcctggagcgtgcgatagagactttgcgacatcatcatgtcgccaacccaagacgggccgatcaccagtattttcatgcaga
gctcttatgcgtcgcgattcagccaggccatgtattccgttacaccttcagcaacggttttgaacggtttgtcgtaacccgccgcgcgcagatttgtc
agatctgcctgagtgaacgcctgatagcggcctttcagtttatccgggaacggaatgtattcgatctggcctttcttgtgataagccagcgtagcat
cagctaccgcctggaaggattccgcacgaccagtaccgagattgaagatgccggaaacgccattttccaggaaccacagattcacatcagc
cacgtcgcccacatagacgaagtcgcgtttgaagttctcgctaccttcaaacagcttcggtgattcaccgttgttaagctgggtgttgagatggaa
agcgacgctcgccatgctgcctttatggccttcacgcggtccataaacgttgaaataacggaagccaacaatctgcgagttcgcttcaggcag
gatttgacgaacatattcatcaaacaggaattttgagtaaccgtagacgttcaacggtttttcgtactcgcgggattcaataaagtcggaggtgcg
tccgccgtaggtggctgcggaagaagcgtacaggaacgggatttcacgctccaggcagtagtgcagcagctccttggagtattgatagttgtta
tccatcatatacttgccgtcccactcggtggtggaagagcacgcgccttcgtggaaaatcgcttcgacatcgccgaactcttcgccagccataat
ctggatcaggaagtcttccttatccatatagtctgcgatattcagatccaccaggttcacaaacttggtgccgtctttcaggttgtccaccaccaga
atatcggtgatgcctttatcattcagggctttaacgatgttgctgccgataaagcccgcgccgccggtaacgatgatcataactgtaaccttcgaat
tatggagtcagagacaatctcagacacgaatgtctgtaatcataccatcacatccgaatcccttcagccctttacatagaggtgcaaggatgcta
attgcaacaaactagtttgggttttagctagtccttcatggcggatgttggttttgcatgtgaatagtgctttagtatcaatattccgatgtatgataaat
aacggaagcatttgaatggataatatacagtatacattctattaatatttatgatataattcttttttttatattaatggtcttattagtaatgaaatcatcca
ccacaattatcactgcatattttgacattggtagaggggattggactgctaataaagggttccgtgaaaaactagctcgttcagttgatgtttatttta
gttactttgagcgtctggccgcacttgaaaatgagatgattattttcacttctcccgatttgaaaccaagggttgaagcgattcgcaacggaaaac
caacaacggttatcgttatcgatataaaaaagaaatttagatatatcagaagtcgtatcgaaaaaattcaaaaagatgagtcgtttacaaatag

acttgagcctcgacaattaaaaaacccagagtactggtcaccagagtatgttttggtatgtaaccttaaggcctattttgtaaataaagctatcaac
atgggcttagtgaaaacgcctcttgttgcgtggatagattttggctattgccataagccaaatgtaactcgaggattgaaaatttgggatttcccattt
gatgaaagtaagatgcatcttttcactataaaaaaagggcttactgtcacatctcaacaacaggtttttgatttcatgattggcaatcatgtatatatt
attgggggcgcaattgttggttcacagcataaatggaaagaattctataaattagttctggaaagtcaaaaaataacactaaataataacattgt
ggacgatgaccagggcatttttgtcatgtgctattacaaacgccccgatctcttcaacctaaactatctggggagaggaaaatggttcgatttgttt
cgctgcttcaggagtaacactttaggggcaaaaatgcaggcgctgagaattttcctttcaagaaaataggtttccctccattagtgggtagcgtttt
gataccaactgagataggaaaaatgatgctttggcatcatttttctttttatatcaatgcagtaaaatcaatcatcattatgcccagccaaaactgt
acagtttttctactatctgtaggtgttgagagacatcaagttacacgttatttatcctgaattttgcagaagtgttaacgcgttatctcgtcgcgacctat
aagtttgggtaatatgtgctggaatttgccctgtctggagaatcgcaatgcgtggagaattttatcagcagttaaccaacgatctggaaaccgca
cgggcggaagggttgtttaaagaagagcgcattattacgtctgcgcagcaagcagatatcactgtggctgatggaagccacgtcattaactttt
gtgccaacaactatctcgggctggcgaatcatcctgatctgattgcggcggcaaaggcgggaatggattctcacggtttcggcatggcttcggt
gcgttttatttgcggcactcaggacagccataaagagcttgaacaaaaactggcggccttcctggggatggaagatgcgattctctactcttcct
gctttgatgctaacggtggcctgtttgaaacgcttctgggtgcggaagacgccattatctccgacgcactgaaccacgcgtctattattgatggtgt
gcgtctgtgcaaagctaaacgctatcgctatgccaacaacgatatgcaggagctggaagcacgtctgaaagaagcgcgtgaagccggtgc
gcgtcatgtgctgatcgccaccgatggtgtgttctcaatggacggcgtgattgccaacctgaagggcgtttgcgatctggcagataaatatgatg
ccctggtgatggtagacgactcccacgcggtcggttttgtcggtgaaaatggtcgtggttcccatgaatactgcgatgtgatgggccgggtcgat
attatcaccggtacgcttggtaaagcgctgggcggggcttctggtggttataccgcggcgcgcaaagaagtggttgagtggctgcgccagcgtt
ctcgtccgtacctgttctccaactcgctggcaccggccattgttgccgcgtccatcaaagtactggagatggtcgaagcgggcagcgaactgcg
tgaccgtctgtgggcgaacgcgcgtcagttccgtgagcaaatgtcggcggcgggctttaccctggcgggagccgatcacgccattattccggt
catgcttggtgatgcggtagtggcgcagaaatttgcccgtgagctgcaaaaagagggcatttacgttaccggtttcttctatccggtcgttccgaa
aggtcaggcgcgtattcgtacccagatgtctgcggcgcataccccctgagcaaattacgcgtgcagtagaagcatttacgcgtattggtaaaca
actgggcgttatcgcctgaggatgtgagatgaaagcgttatccaaactgaaagcggaagagggcatctggatgaccgacgttcctgtaccgg
aactcgggcataacgatctgctgattaaaatccgtaaaacagccatctgcgggactgacgttcacatctataactgggatgagtggtcgcaaa
aaaccatcccggtgccgatggtcgtgggccatgaatatgtcggtgaagtggtaggtattggtcaggaagtgaaaggcttcaagatcggcgatc
gcgtttctggcgaaggccatatcacctgtggtcattgccgcaactgtcgtggtggtcgtacccatttgtgccgcaacacgataggcgttggtgttaa
tcgcccgggctgctttgccgaatatctggtgatcccggcattcaacgccttcaaaatccccgacaatatttccgatgacttagccgcaatttttgatc
ccttcggtaacgccgtgcataccgcgctgtcgtttgatctggtgggcgaagatgtgctggtttctggtgcaggcccgattggtattatggcagcgg
cggtggcgaaacacgttggtgcacgcaatgtggtgatcactgatgttaacgaataccgccttgagctggcgcgtaaaatgggtatcacccgtg
cggttaacgtcgccaaagaaaatctcaatgacgtgatggcggagttaggcatgaccgaaggttttgatgtcggtctggaaatgtccggtgcgcc
gccagcgtttcgtaccatgcttgacaccatgaatcacggcggccgtattgcgatgctgggtattccgccgtctgatatgtctatcgactggaccaa
agtgatctttaaaggcttgttcattaaaggtatttacggtcgtgagatgtttgaaacctggtacaagatggcggcgctgattcagtctggcctcgatc
tttcgccgatcattacccatcgtttctctatcgatgatttccagaagggctttgacgctatgcgttcgggccagtccgggaaagttattctgagctggg
attaacacgaacaagggctggtattccagcccttttatctgaggataatctgttaaatatgtaaaatcctgtcagtgtaataaagagttcgtaattgt
gctgatctcttatatagctgctctcattatctctctaccctgaagtgactctctcacctgtaaaaataatatctcacaggcttaatagtttcttaatacaa
agcctgtaaaacgtcaggataacttcagaggtcgtcggtaatttatgatgaacagcaccaataaacttagtgttattattccgttatataatgcggg
cgatgatttccgcacttgtatggaatctttaattacgcaaacctggactgctctggaaatcattattattaacgatggttcaacggataattctgttga
aatagcaaagtattacgcagaaaactatccgcacgttcgtttgttgcatcaggcgaatgctggcgcatcggtggcgcgtaatcgtgggattgaa
gtggcaacgggcaaatatgtcgcttttgtcgatgctgacgatgaagtctatcccaccatgtacgaaacgctgatgaccatggcgttagaggacg
acctcgacgtggcgcagtgcaacgctgactggtgttttcgtgaaacgggagaaacctggcaatccatccccaccgatcgccttcgctcaaccg
gcgtattaaccggcccggactggctgcggatggggctttcttcgcgccgttggactcacgttgtctggatgggggtttatcgccgtgatgttattgtt
aaaaataacattaaatttattgccggattacatcatcaggatattgtctggacaacagaattcatgtttaacgcgctgcgtgcgcgatataccgag
caatcattatataaatattatctgcataatacgtcagtgagtcggttgcatagacaagggaataaaaaccttaattatcaacgtcactatattaaga
ttacccgcctgctggagaaattaaatcgaaattatgccgacaaaattatgatttatccggaatttcatcagcaaataacttacgaggcattgcgtgt
ttgccatgcggtgcgcaaagagccggatattcttacccgccaacggatgattgccgagatatttacttccggtatgtataagcgcctgattaccaa
tgtgcgcagcgtgaaggtcggttaccaggcgttactgtggtctttccgcttatggcaatggcgcgacaaaacgcggtcgcaccatcgcattacg
cgtagcgcctttaatttgcgctagcgttaaacttcgccgcttcgggctgtttgccccagccttgccactgatgctggaagtaaacgatcagcgtgct
ctggctgatgctttcgcttaatacttcaaagaagcgattagcataaacaggttctatcggtttcttcggtttgcacagcttcacgccacggaacgga
ttacgcggtgcatcaggcacagcgtttttcggcggtgtagaagtatcaacctgcggttcattcagcaagctgctggctttcaccagcgtaatgtctg
gcggcaggttataaaccatctgttgcaacacgcgtaccgttgcagggtgaggatgcccaatggcaatggtcgaaccgttgcgacgcgccagg
tcaattgcgcgattaaattgcacacggatgtccgcttcattttgcgaatcgtcgaggaataccttccgtttgatcaccttcacgccagtgccttgcgc

ggcgcgcatcgcctgggtattaccgatggttacgctgtcgaggaagtaaagattgtaacgctccagcgcctgcatcactttctgcataccaaac
aggttagaggtcatcttgctgcccatgtggttgttgatccccacggcatagggcacgttattgaccgcactacgaataatgcgctcaatttcgtcgc
tgctcatctccgggcgtagcgtatttttctccagcggctgtttactcaacggtgccatcgggagatgaatcaacacttcgtgcccgctgttatgcgct
ttggtcgccatctctctggcgtgcggtgaatcgggtaatacagcgacggagatagcggaaggcatcgccagcacctggttttcgttgtgcgggc
gatacccaaaatcatcaatgacgatggcaagtttgccagcaagtacgggggaggagagcgccaacagagcggcaaatgcaagaacgtta
cgacgaaatggaaacaaaacttatcttcccaaccacggctgtggattgaccgcctgaccctggcggcgaatttcgaaatagagtgaaggccg
accctgaccgccactgctgcccaccagtgcaatcggctggcccgcgcgaacctgcgaaccaacgctcaccagtgcgctctgattatagccgt
aaagactcatgtcgcctttaccatgctcaaccaccaccaccagaccgtagccttgcagccagtcagccagaatcacccgaccgtcggcaatc
gctttaacttcagtaccttcagaagcaccgataaccatacctttccagcgtaattcaccctgtagctgttcgccatagcgatgcagcgtcggcccg
cgaaccggccagaatgcctgaccacgcggcgcgcccagaccaccggtacgggacatcagcgatttttcgctttcggtcggtttgtaggtggtg
cctttgcgcgtcgcttctttctggcggtcgcgaaccgcctgggcctcacgtgcttctcgttctgcacgcgctttcgccgcggcttccgcacgggcaa
tgctgttacgcagacgggattcgttggcgcgcagctcgctcaactgttgctgaccttgctggatggaagactccagccctgccagcgtctttttac
gctcgttcagcgcctgagtcagtttcgcctgttgggcgcgctgctcatataacagcgtttgttgctcgctctgtttctcttccagttcagcacgctgcat
ggcgacttcttcacgcgtttgcttcaactgggcaatggtttcttgtcgcgcctggttgagatagccgaaataagcctgtaaacgctgtccacgctgg
ctttcttcaccgctgagaatcagctgaataccggtatgctcgccctgacggaatgcggcatccagttgtgcggcgaggctgcgctcctgggcgg
ctttttgctgctccagtttggcaatcgacgcgttcatctcatcaatctgtttattgagttgattgagcgtgttttgcgtttcgcgcagcttacgggtggcttc
agagatcgcttcttcctgctttttcaattgtgcgagcaggcttgcgcgttgttgttgcttttggcgtaccgcgcgctcttttgcggcgatgtcagcctgaa
tagatttgagttggtcacgctcatccgcgtgggcggaaaaggcgcacaacaatacgccagcgctaagaacgctggcgtagatgatgggcct
gattgcaaacctgcgcggtttcacggcccgtgtcatggtattaatcgcctttcccctcatggggagggattattccacgatgaacagcggcttacc
agtcatctcttgcgggatttccatacccatcagcgacaacatggtcggcgcgatgtcagaaagtttgccgccttcaaccgctttcacgttcttatca
ccaacgtaaatcagcggaactggcaggttggtgtgtgccgtgtgtgcctgacccgttgccggatcgcgcatctgctcagcgttaccgtggtcag
cggtgatcagcagttgtccacccacggactcaaccgctttcgcgacttcttccacgcagtgatccagcgcttcaaccgctttaaccgccgcttcc
atcaccccggtgtgacctaccatgtcgccgttcggatagttacagatgatggtgtcgtatttgccgctcttgatggccgcaaccagtttttcggtcag
ctctgcggagctcatttccggttgcagatcgtaggtagccactttcggtgagttgatcagaatgcgatcttcgcctttgaacgactcttctacgccac
cgttgaagaagaaagtaacgtgggcatatttttcggtttcggaaatacgcaactgagttttgtcgtttttcgccatccactcgccgaaggtgttaacc
agggaagcgggtgggtaagcaaccgcagttttgatgtcagcggcgtattcggtcagcatcacgaaatcgacgttaaccactttcttacgcgcg
aagccatcgaaatcagcgttcacgaaagcacgagtgatttcacgcgcgcggtcagcacggaagttcatgaaaatcagtgcatcgccgtcttc
cattgccgcgtctggctgaccttcagcacggataacggtcgctttcacgaattcatcattttcgtcgcgagcataagcagcctgcaaaccagcaa
cggcggtatcggcctggaactcgccctgcgccagagtcagcaggtcataagctttttctacgcgatcccaacggttatcgcggtccatcgcgta
gtaacgaccaatgatggacgctacgcggcctttgcccagcgcggcaaatttttcttcgaatttttcagcgaggattcagcactgcgcggcggag
tgtcgcgaccgtcaaggaatgcgtgcaggtagatttttctgcgccgcgttcagctgccagttctaccatcgccatgatgtgatcttcgtggctgtgt
acgccgcctgcggagagcagacccataatgtgtaccgctttgcctgcgtttttcgctttatctaccgcgccagtcagcaccggattagcaaagaa
agcgcgatctttgatttcaacgtccagacgagtcaggtcctgatacacgatgcggccggcacccaggttaacgtggcctacttcggagttaccc
atctgacggtcaggcagaccgacttccagaccggaagcgtcgattagggtatgcggacgattggcccacagtgcatccattaccggggtttta
gcactaaaaatggcgttatcctgctgttcttcgcgatagccatagccatccagaatcaccagtaccataggtttttagaaaccaacattgcgaca
acctcatactcaagagtcaaaatttgcgtaattttactacagctgaatcgataaaatagcctctgaagatcaaaatatacgaccgctcgcggca
agccgatgccagatttacgcttttttttcgtcgcgccccgcaaaattgcattccagttaacgcgctggctgtatttgccgcaccgcgcaggtatactc
ctttcctggtttttttaatcactacgtcgggagttgttacccccccatgcaagaaattatgcaatttgttggccgtcatcccatactgagtatcgcctggat
cgcgttactggtggcggttcttgtgactacgtttaagagcctgacctcgaaagtgaaggtgattactcgtggtgaagctacgcgtctgatcaacaa
agaagacgctgtggttgtggatttacgtcagcgtgatgacttccgtaaaggccatatcgcaggttctattaatctgttgccgagcgaaatcaaagc
caacaatgttggtgagcttgagaagcacaaagacaaaccggttatcgtggtagacggttctggcatgcagtgccaggagcctgcaaacgca
ctgacgaaagctggttttgcgcaagtattcgtgctgaaagaaggcgtcgctggctgggctggcgaaaacttgcctttggtgcgcggcaaataatt
tacctgtcgtctttcatgatgcaggccgttagctgcaattaggcccccagcgacttattgttataagcttctggggacactccattttgctgccgccct
gcaacatgaaatttcagggtaaaaaaacaggagctaatttatggccaatgttgaaatctataccaaagaaacctgcccgtattgccatcgtgca
aaagcactgctgagcagcaagggcgtgagtttccaggagctgccgatcgatggcaacgccgccaagcgtgaagagatgatcaaacgcag
cggtcgcaccacggttccccagatttttattgacgcacagcacattggcggctgtgatgacttgtatgcattggatgcacgtggtggactggatcc
cctgctgaaataacgtgtgaacgttggcattacattgcgcagtatttaaggacaacacttaagggttttctacacatgtcagaacaaaacaacac
tgaaatgactttccagatccaacgtatttataccaaggatatctctttcgaagcgccgaacgcgccgcacgttttccagaaagattggcaaccag
aagttaaacttgatctggatacggcatcttcccaactggcagatgacgtatacgaagtggtactgcgtgttaccgtaacggcctctttgggcgaa
gaaaccgcgttcctgtgtgaagttcagcagggcggtattttctccatcgcgggtatcgaaggcacccagatggcgcattgcctgggagcatact

gcccgaacattctgttcccgtatgctcgtgagtgcatcaccagcatggtatcccgcggtacattcccgcaactgaaccttgcgccggttaacttcg
atgcgctgttcatgaactatttgcagcagcaggctggcgaaggtactgaagaacatcaggatgcctgatgaaccaacgtaatgcttcaatgact
gtgatcggtgccggctcgtacggcaccgctcttgccatcaccctggcaagaaatggccacgaggttgtcctctggggccatgaccctgaacat
atcgcaacgcttgaacgcgaccgctgtaacgccgcgtttctccccgatgtgccttttcccgatacgctccatcttgaaagcgatctcgccactgcg
ctggcagccagccgtaatattctcgtcgtcgtacccagccatgtctttggtgaagtgctgcgccagattaaaccactgatgcgtcctgatgcgcgt
ctggtgtgggcgaccaaagggctggaagcggaaaccggacgtctgttacaggacgtggcgcgtgaggccttaggcgatcaaattccgctgg
cggttatctctggcccaacgtttgcgaaagaactggcggcaggtttaccgacagctatttcgctggcctcgaccgatcagacctttgccgatgat
ctccagcagctgctgcactgcggcaaaagtttccgcgtttacagcaatccggatttcattggcgtgcagcttggcggcgcggtgaaaaacgttat
tgccattggtgcggggatgtccgacggtatcggttttggtgcgaatgcgcgtacggcgctgatcacccgtgggctggctgaaatgtcgcgtcttg
gtgcggcgctgggtgccgaccctgccacctttatgggcatggcggggcttggcgatctggtgcttacctgtaccgacaaccagtcgcgtaaccg
ccgttttggcatgatgctcggtcagggcatggatgtacaaagcgcgcaggagaagattggtcaggtggtggaaggctaccgcaatacgaaa
gaagtccgcgaactggcgcatcgcttcggcgttgaaatgccaataaccgaggaaatttatcaagtattatattgcggaaaaaacgcgcgcga
ggcagcattgactttactaggtcgtgcacgcaaggacgagcgcagcagccactaaccccagggaacctttgttaccgctatgacccggcccg
cgcagaacgggccggtcattatctcatcgtgtggagtaagcaatgtcgtgtgaagaactggaaattgtctggaacaatattaaagccgaagcc
agaacgctggcggactgtgagccaatgctggccagtttttaccacgcgacgctactcaagcacgaaaaccttggcagtgcactgagctacat
gctggcgaacaagctgtcatcgccaattatgcctgctattgctatccgtgaagtggtggaagaagcctacgccgctgacccggaaatgatcgc
ctctgcggcctgtgatattcaggcggtgcgtacccgcgacccggcagtcgataaatactcaaccccgttgttatacctgaagggttttcatgcctt
gcaggcctatcgcatcggtcactggttgtggaatcaggggcgtcgcgcactggcaatctttctgcaaaaccaggtttctgtgacgttccaggtcg
atattcacccggcagcaaaaattggtcgcggtatcatgcttgaccacgcgacaggcatcgtcgttggtgaaacggcggtgattgaaaacgac
gtatcgattctgcaatctgtgacgcttggcggtacgggtaaatctggtggtgaccgtcacccgaaaattcgtgaaggtgtgatgattggcgcggg
cgcgaaaatcctcggcaatattgaagttgggcgcggcgcgaagattggcgcaggttccgtggtgctgcaaccggtgccgccgcataccacc
gccgctggcgttccggctcgtattgtcggtaaaccagacagcgataagccatcaatggatatggaccagcatttcaacggtattaaccatacatt
tgagtatggggatgggatctaatgtcctgtgatcgtgccggatgcgatgtaatcatctatccggcctacagtaactaatctctcaataccgctcccg
gataccccaactgccgccaggcttcatacaccactaccgacaccgcattggacagattcatgctgcggctgtccggcaccatcggaatgcga
atttttgttcagcgggcagggcatcaagaatgctcgctggcaggccgcgtgtttccgggccgaacatcagataatcgccatcctgatagcttac
ggcgctgtgagcaggtgtacctttcgtggtgagggcgaacaggcgctggggattttctgcttcgaggaacgcgcgatagtcatgatgacgcgta
acggcggtaaactcgtgatagtccagccccgcgcggcgcaggcgcttatcgtcccaggcaaatcccatcggttcgatgatatgcagacgaa
agccggtattagcgcaaagacggatgatgttgccagtatttggcggaatttctggttcgtaaagtacgatgtttagcatactgccccccttagtgcgg
gggcaggatagcagaaaattggtgagcagtgcggccttttcccctcaccccggccctctccccggaggggcgagggagaaaaaaagagtg
cggtgtcgtttcagagtgagggagtacatacagcgccgaacggtcccctctccctgagggagagggttagggtgagggggcgcaaacgact
atgccgcattccctttcgccatgggagccagtgccgcaggcaactctttacccagcccctgcaccagcgaatcttgcgtaatttcgctgatcgatt
tcgcgccagtcagcgtcatcgccactttcatctctttttcgatcagatttagcaggttagctacacccgcctggcccgctgttgccagcgcatacaa
gaaagcacgacccagcagtacggtgtcggcaccgagcgcaatcatacgcacgacatcaagcccgttacgaattccgctatccgccagaat
ggctatatcacctttcaccgcatctgcaatagcaggcagtgcacgggcggaagagagtacaccgtccagctggcggccaccgtggttagaa
accacaattccatcagcaccaaaacgtactgcatcgcgcgcatcttccggatcgaggatccctttgatcaccatcgggccatcccagaaatcg
cggatccattcaaggtctttccatgagatggacggatcgaagttattccccagccagccgatgtaatcttccagtccggtcggtttgccgagataa
gctgagatattacctaaatcatgtggacgaccgttcaggcccacatcccacgcccattgcggatgtgtcaccgcttgcaagtagcggcgcattg
ccgcgttcgggccgctcatacctgaatgcgcatcacggtagcgtgcgcccggtgtcggcatatccacggtgaaaaccagcgtcgaacaacc
cgctgcttttgctcgctccagcgcgttacgcataaagccgcgatcgcgcagtacataaagctggaaccacattgggcgcttgatggctggcgc
gacttcttcaatcgggcaaacggaaaccgtcgagagagtaaacggaataccatgcgcgtccgccgctttggctgcctgaacttcgccacgac
gcgcatacatgccacacaaacccaccggagccagtgccaccggcatcgacaatttctcattaaacagcgtcgtttccaggcttaagtcggac
atgtttttcagaatacgctggcgcagcgccacttctgacaaatcttccacgttgcggcgcagcgtgtattcagaatatgcaccaccatccatatag
tggaacaggaacggcggcagaatgcgttgcgctgcggcgcgataatcgctggctgcggaaataatcatgcgtttttctccctcgaatgctcatt
atgctcaccgggcaggcgggtaatccgtgcgtggcgagcctgatcttcatcgaatcgtttcatggtggtgtgaacaaaactaaggtgcgccatc
attgctttacgcgccccgtcagcatcaccggcaaaaatggcgtcaatgacagcctgatgttgttcggtcagttgtgaaaaaaccggtggcacca
gatacatccgctgacggctatgcttcactgaggattgcaggacatcgaagaaaccgcgcatggtttgcagcagcacgatgttatgtgaggcttc
ggcaatcgccagatgaaaacgaacgtccgcttgtgaggcgatatccgggtcttcacttagcgttgcttcaaagcaaagctgaatcttttctttgtc
gccaggtgtggcgcgcattgccgcatgccatgcggtgctggcttcaatggcgtagcgggcttccagaatatcgaaactgtaatccggatcatcg
gccatcagtgtttttagcggctggacgatgttttgctccgaccatgtgtcatgacgccagcgaataaacgtcccgccgccgcgtcgactgagca
gcacgccttcactcaccagttttgccagcgcctcgcgcagtgaattacgtgatacgccgagttgcatcgccagttggcgctcagcgggcaactt

catgcccgcttccaggttttttcatcaatcagcgcccgcacacgatcggcaacctcgtctgacaggcgtctgggtaaaacaatcattaaggaat
catccacgttaagacataagcctgaagcgtggtgatcacgcccactatacaggtgaagatcaggctgtgtttgacagtaaagcggaacaaat
cagactctttgcccaccaggcctaccgccgcacaggcgatagcgatagattgcggggagatcatcttaccggtgacgccaccggtggtattg
gcggcaaccagcaacagatcagagacgccaatttgttgtgctgcggtggcttgcagcgcggcgaacagggcgttagatgaggtatccgacc
cggtcaggaatacccccagccagccgaggaacggcgagaagaaggtgaatgcatgaccggtgtgcgccagtgccagcgccagtgttgat
gacagtccggaatagttcgaaataaaggcgaatgccagcaccataccgatggagtagatgggcagagccagttctttcagcgtgctgccga
aggtgctgatagcgtcagacggtttcatcttcagccagacaatcgagagcagtgcagcaaacagaatggcggtgccggtggcagagaacc
agtcaaacttaaacacggcggcataggctgtagcctcgctgaccactggcggcatacgggcaaccagtttatcgaggtacggcaccggaat
attgatcacccactcatacagcgcgccacccgatgcgaacagggctttaaacggcgggatactccacagtgttacggtagctgtcaggaaca
ggaacggtgtccaggcacgtaacacctgacccgcagtgtaaccggtgtgggccagcgtcatatcaacctgtgacgcccccaaatcaccaaa
acggaatacacgcactggctgccagcgtttgaggaacagcgtcaggcagagcagtgataccagcgaagagataatgtccggcagctccg
gcccaatgaagttagagctaaggtactgagcgatggcaaacgagccgcccgcaaccacgaccgcaggccacgtctctttgataccgcgcc
agccgtccataatcgccatgatccagaacagcacgataatggtcataaacggtagctgccgccccaccatctgaccaatctcaaagctgtcg
atacctgttacctgtccggcaaccaggattggaatgcccatcgcaccaaatgccactggcgcggtgttaacaatcaggcacagcccggcggc
gtacagcggtttaaaacccaggccgaccagcaatgcggcggtaattgctaccggtgcgccaaagcctgcggctccttcaaggaacgcgccg
aaacagaaaccgacgatcagcatttgcagacgctggtcaggggttatcgaaagaatagacgagcgaatgatgtcaaactgcccggttttcac
cgagatcttatagacgaacaccgctgcaataatgatccacgcgatgggccacaacccgtagaagaaaccataaaccaccgaggccagcg
cgttagcgaccggcattttatagaacagcaaagccacggcaagggcgattgccaccgtccacgaggcggcgacgtatcctttcagtttgagct
taatcagcgcaaagaagaaaaacaggatgggaagcgatgctatcagactggaaagccagatattcccggcgggatcgtagttttgttgcca
gagattcattgcaggtctcctggagtccacgcacatcgtgtaatgagtctgttgctcatctccttgtcacacgttgtgtaaaagtggcactgccaatt
gtgttgtgtagggataatgacaatgaatggttaactaaatgttatgtattggcaacattgatgtgagttttaattgggaaattgtgaaccagaggtca
gataagcgaagaattggtagggccaattcttgtggggggagatgtctggtaaagaaaagggcaatcctcatcagagaattgcccttaggttgtt
gatccagagcgccttatcttgccggatgcggcgtgaacaccttactcggcctataggaatgtgcagaatcaccctgtagatactgtgttcttaaaa
cgcggtctttgaaaacccagtcatctcctgcaggcccatttcgcggcccagtgcggtcattgggtgcacaacgaccagtccgcgaacacttttct
tcagcttgcccatatcagcctgctctttttggtaattgcgcgctggaatggcatcttcatcagcttctgtgcttctttactcagtttttggctatgaacttca
cgcaggcgagcaatttctgcttccagcgtggctttctctttttccagctcggcatatttgtctgcggcttcaaccagcgaaagatcggcctgctggtg
gcggatggcgtccaggcgatcgctgaggcgtttgatttcgttttttcgacttctttcatgtgatggcttctgataaagataaatttatagcccctagtat
gcgcgaccaggtgctttgttcacaactaatgacattgtaatagttctgtaacgtaatcattaacatgctcaaaaactgcgcaattcgttgattaatta
agaaatctatagaaaaaagataataaacagcagcggatgattattgaggattcagcacgttttccttaatgtcatctttaccgggaacctaaact
aaaattggcggccgcgcattcccggcgcggggtaatacggagatatcatcatgggcaaattaggtgaaaacgttccgcttcttatcgataaag
ccgtagatttcatggcatcaagccaggcgttccgggagtatctgaaaaaacttcctccccgtaacgcgattccgtccggaatacccgatgaaa
gcgtgccgttatatctacaacgtctggagtattatcgtcggctttatcggccgaagcaggtagaggggcagtaagcagacgttgcttacttttgaa
aggctttttttaagctgattttggaaatcagctcagtcagtgaaaggaccattgttgatcgcacagcctgttgatagcgctgaatttgcattgcatac
aaactggagtctgctggttcaaactgtggcggcggtggtaacgccgccacgcagtgcagttcaccaaagggtccaaggatttcgtcgtcggta
aaggcgtactcgttgccgtcgtgatttagctcttcacgcaatgccatcagcagttccgcatcttcgtattcctggcggttaatgacgcccaacccgt
aaatgagttttaaacgcacagaaagatcgcccagcggaccatcgccgtcgagtaacggttctacagcatacttcaccgcgtaatcgtctttgcg
gaatacctgaagcaccagaagatttaccgcctcggtcaggagctcgacggcggtgatcagaaagcttcgcacggttttgccagcattcagac
gctcaagcacacggttttcaaaggcctgggtttgttccattgttgcctgcatatctgacaatctgttattcgggcgcagggtgtcctgcgcctggtcc
accattattgcattgctttataagcggttaccgcctcggatacaacctcgctgttggcatcaagaccggaaatctgtgccagcgccgcctgcgga
cctttgtcagcgatcagtgctgccagttcctgagcctgcggatcatcttcactgcggaagtgcattgcagcggcaataccttcaatcaggtttttatg
tggcagaccatattccagcgtaccgagcagtggcttgatcagacggtcgccagcactcagtttacgcagtggctgacggcctacgcgctctac
atcatctttcagatacgggttctcaaaacggccgagaattttctggatgtacgccgcatgcttgtcagcgtcaaagccgtagcgcttgatcaatact
gcaccactttcttccatcgcaccttttaccaccgcgcggattttctcgtcgagaatcgcgtcacgaatggtctgatgaccggccagttttccgaggt
acgcggttatagcatgacccgtgttcagggtgaagagtttacgttcgacaaatgccatcaggttgtcggttaactccatgcctgggatgttcggca
gtgcgcctttgaactgcgttttatcgacaatccattcgctgaaggtttctaccgtcacttccagcggatcgttagttgccgaagccgaaggcggtac
gatgcggtcaacggcggaatcgacaaagccaacgtgttcttctacccacgctttggcgtcttccggcagggcgttcatcacatggcctttcagct
gcgtggtaccgcgtaccatgttttcacaggcgatgatgttcagcggggattcattaccttgttctttacgtttcacctgccctttggcgattgccggag
caatacgttccagcacaaccgggccaacggcggtagtgactaaatcaacctgagcaatcagatcaacgacatcatcaccaatgctgctgac
agcattgacgccggaaacggtatctacctgctcggtttcaccaaccacatgtacctgatagctatgacgggcattcagggcatcaagtaccac
ctgattgacatcggcaaacgtcagttggatacccgcgtctgccagcagtttaccgataaagccacgaccgatattacctgcgccaaaatgtaat

gctttcatagtattaaccttcatcaatgtttttacccgagagggctggggtgaggcttttccctcacccaaaccctctccgtgaggagaggggcag
cgccggatgcggcgtaagcgccttatccggcctacaagaacgtgcaaattcaacaaattgtgatgcatcctgtaggcctgataagcgtagcgc
atcaggcaattaaccccaccttctccatgtggagagggtgggattggattacttacgacctgccagcagttccagcacttcatccacgctggtgg
tgtgtgccagacgctcgatgacggactcatcatccagtgcattggtcaggctggtgataacctgaatgtgctcgttgttacgggcagcaatacca
atcaccagacgggcaatgtcatcttcttcttcaccgaagcgcacgccttccgggtactggcagaacacgacgcccgttttcagtacgcgatcttt
cgcttcaaccgtaccgtgtggcaccgcgatagactcacccagataagtcggggtcagtttttcacgatccagcatcgcctgaacgtattccggct
caacgtaaccgcctttcaccagctgctcgccagcaaaacgaatcgcttcttctttggttgccgctttgcgaccgaggaagatgttctccgcgccta
gcttgaacaggttagcactggaatcgtcaaagctgtctttcaggctgtcttttactttctcttcgtttgccgtgtggcgttgggcagcaaccagacgttc
ggtcaggctggtgtacaggccgctgtcgaggaagttggtcagcgaaatatgctgtgcctgcggaacctggcgcatagcgcgttcggtcaggtc
acggtgagtgatgacgaggtccacatctggcggcaggttgttgatcgcgctgttagtaacagaaatctgcgacagacctgcatcctgaattttctt
acgcagaacgcctgcgcccatcgcactggaacccataccggcgtcacaggcaacgatgattttacgtacgtggctcaggtcgttagtcacatc
gccagcagacagcggagatgcgcctttagactcagctttcatgtcctgcatacgacgagttgctgcttcaatatcatcttcttctttcactttgctggtt
ttcagcaaaatagcagagacaacgaaggagacagccatcgccgcacacacacccgcgatgttagcgaagtaagcaccttttggtgtcatcg
ccagtacagcaaggatagaacccggagatgccggagaaaccagaccaccgcccaggatagtcagcgtgaacacgccagtcataccgcc
gaggatgactgcgaggatcagacgcggattcatcagcacatacgggaagtagatttcgtggatacccccaggaagtggatgattgccgca
ccgcccgcagactgtttagcgctaccacgaccaaagaacatgtacgccagcagcacgcccatacctggacctgggttagcttcaatcagga
agaagattgatttacccagttcatgggactgctgaatacccagcggcgagaagataccgtggttaatggcgttgttgaggaacaggattttcgc
cggttcaacaaagatagacgccagcggcagcatgtcatggacaaccatgaagttaacgcccgcagccagcattttggacagggcttcaaca
atcgggccaatgccgaggaatgccagaatagcgaggatcatcccgatgatgcctgcggagaagttattcaccagcatctcaaaaccggattt
gatcttaccgtctacccagcggtcgaagtgcttaatgcaccagccgcccagcggacctgcaatcatagaaccgaggaacatcggcatgtctg
cgccgacgataacgcccatggtggtgatggcaccgactacgccgccacgttcgccgcctaccagcttaccaccggtataaccgatcagcag
cggcaggagataagtgatcatcggcccgaccagcttcgccagcgtctcgttcggtaaccaccctgttggaataaataacgcggtgatgatacc
ccacgcgataaacgcgccgatatttggcatcaccatgttgctgaggaaacgaccaaagctttgcactttgatcttaatatcggatgacataaaa
acacccccttcttatgtttgctgtcgcgcaggctggaagcccgaggtttttttgttaatgtggcggcagaggtagccggaccttgtagatactgcgaa
atctggcactgaatcggttaactgtccagtcgacggccttttgtgtgatatcaatcacataaatataggggggtagagaggtaattgacgtgacgtt
catcacaaaacgcttgtgtaaaaaagcaacaaggcggcgattttcgctaaaaatggcatttaaatgtgagtagtgtcacatttttgttttgatggttt
gttgaatctttgtgatctgaatcacaagatattttcttacggcgttcacctgatgtgatctacagcatgttatgccttcgtcatacaatcgttatgtaacta
agcaactcatcacctctaaaaattattctttggtgttatggcgagaatcgcgaacccggattattgtcaaaatatcagcctgactcccttctggctgc
cagcccaaaaacaacactgataaacgtgaatccgaggcagatatgtttctaaactatttcgcgttgggagtgctgatcttcgtctttctggtgattttt
tatggaatcatcgcgatacatgacatcccttatctgattgccaaaaagcgcaaccatccccatgccgacgctattcatacggcgggctgggtga
gcctgtttactctgcatgttatctggccgtttctgtggatctgggcgacgctctatcaaccggagcgtggctggggtatgcagtcacatgttgcgtcg
caggagaaagcgactgacccggaaatcgccgcactttctgaccgaatttcccggctggagcatcaactcgccgccgagaaaaagactgac
tattccacgttcccggagatctaattatggatctattgattgttttaacttacgtggcgctggcgtgggcggtctttaaaatcttccgcattccggtaaat
cagtggacgctggcgacggcggcgctgggaggcgtgtttctggtgagtggtttgattttgttgatgaactacaaccacccttacacttttaccgcg
caaaaggcagtgatagcgatccctatcacgccacaggtgacgggaattgttactgaagtcactgacaagaataatcagcttattcaaaaggg
cgaggtgctttttaagctcgacccggttcgttaccaggcgcgagttgacagacttcaggctgacctgatgacggcgacgcataatataaagac
gctgcgtgcgcagctcactgaagcgcaggccaacaccacccaggtttcagcggagcgcgaccgtctgtttaaaaattatcaacgttacttgaa
aggcagccaggcggcggtgaatccgttctcggaacgtgacatcgacgatgcgcggcaaaatttcctcgcgcaggatgcgctggtgaaaggc
tcggtggcggagcaggcgcagatccagagccagctcgacagtatggttaacggcgagcaatcgcagattgtgagcttaagagcgcaactta
ctgaagcaaaatataaccttgagcagactgtcattcgcgcgccgagcaatggctacgttactcaggtactgatccgcccaggtacatacgcag
ctgccttgccgctgcgtccggtgatggtcttcatccccgagcaaaaacggcaaattgtcgcccaatttcggcaaaactcgctgttacgtctgaaa
cctggcgatgatgcggaagtggtgtttaacgcgctacctgggcaggtgtttcacggcaaactgactagtattttacctgtcgtgccaggcggttctt
atcaggcgcagggggtattgcaatcattaacggtcgtgcccggcacggacggtgtgctgggaaccattgaactggaccctaacgatgatatc
gatgccttacccgacggcatctacgcccaggtggcggtttactccgaccatttcagccatgtttcggtgatgcggaaagtgctgctaagaatgac
cagctggatgcattatctttatttggatcattgaggggggaggggggagattatggaactttgctccttgtaaggactaattatttttgcatattacttcat
tagcttcgatgatttttctttaatattcattatgttaataagttctggatgagtgtgctctcattggatttttttatccgtcttgttctgccgcaggatgagactat
acatcgtcgagatcgcaatgatgatatgagttgttcctcctgcgacggctctaatcaaaaaagagcaggagtgcttttccatctattgcgatgatg
gctttattatctgaaggatgacagtcacatttaaaaaatatttaatctatagatcacaatgtagggtcatggaatatatgtatacaactattttcttcttc
acttttaattatcgtattcattataaaactaagttaaaaaaactcctggagagtgagttattaatatatttttaaataaaattaagttatcattcatcgatg
cactttatagaatcatcaatggccataatagtactattaactctcaattttatatttttatcatcactacaaatattaactgaatttatatcaatgattttttt

attcttatcttttattttatttactgtttcaatgcaatcagcggcaagttggagtggatttagatttttcagatatggtcttatgatctttcccttacttaataaat
agcatgtataggattcggaaagtccagcatctattttgtacctcattatattagataaggatgtgagaaattccttgttatcattcgcagccatatatg
ccagtccgagttcagcctctgcggccttacaggcaaaatagcttagtgaacacgcttttcgtccctctttagtactggctgatattagtgcccataat
attgctttgtctgccaaatctaactctctctgaaattgtcttgcagtaactgcactcgcagaaatagagaaaaaaatacaattgttaataaccttgcc
atattttttagtccgtaactgttaatgtgctatctggctctgaaaatattaactcatttaatttttgcatgtcattagataaaccagttatacaaccctcgg
aacagaatcgaggtcctaaagacggaccaaaagcgtttaaacaactatgtattgaaaatccattgcgattataagttcctgtattaactgacggt
actaatcgtatgccgtcaggttttctattgcttgctcccgggtcattaacattccaactccataggccttgaggtattggccatctatttataaatgtgta
caacggggattatttttacattgcatattattaccagaagcgactggaatactaagcacatcataaaaattatcatcacatggtatatctgaaaca
cacgaaagcatacctaaagttatgctgtataaacaggtggctaatcccaaggggatcgattccagaaatcgggttcaacggatactgataaa
aattccacctaccttccagcccaatcggagcctgagtgttataccgcccctgctgcggttaatgttaccgataatagttatagtacagccctgattc
atcatcatactactgctgcggcagtctgatgaactattgcttttacggattctccttattcttccttctatccattcgctttagctttttgagctcttgaagtttg
ctctttgcatcaatattaccgttatttacagcgaattccatccaataaatagctctattcaaattataatattctgataatgttggatttgtatctgataaa
aagaccccataattaaactgcgctgtaacattcccctgagatgctgatctttccaagaatcgcaattgtttgtaaatgttatttttagtaaagcagtaa
tattgatatagtcgaaatgatgcctctgaatcaccatgttgtgatttatacttcagtttcatcaacttttcttcacttaaatcataggtcattgctgtagacc
ccatcatactttcgttttgttttattttcacatcagcaccatgcaattgacaaataaaagagaaataaaaaaatagtaacaagcatgctttcatacat
ctacaacctatcttatatcactgggtttttctggcgtaccttgcatattagaaatggggtgcccagatggcccatatgttatccaattaccaaggtcttt
tgttgttatagccattagatccttatttgaattaaggttcgttggtgctgttagcactgtatccatttttaacttaccattagttcctcgcccccaccacgtc
catagcgtgtcaggtcctattacatttgtatgtaactctttagctaattagctagcaaatgaattttgtcctttacctgtattacaagaaaataaccaaa
ctgtcatcccatctttataattcccatcaaatttaattagatatgctagatctttagctgtcatgatactgcgcgttgcggattcaatagatgtgggggta
ccatgccccccgattgtgaaaacgcctgggatattaatctcatcagctacagaatggataagatcactttcggggtataaatttatatcaaccaa
accttgcggatctacatttatgaccggattcaacggatactgataaaaattccatcccccttcagtccaatcggatcctgagtgatataccgccc
cagcagcgggtcataatagcggtggcggttgtaatacaggccggactcctcatcatactgctgacccggcaggcgaatgagctgctgcagat
gatgcgggttctcatcactcagcaggttgccccattcatcatattctgcgcaccacgctgttgccccttccgtgctgatgagcgccagcggcagg
ccgcgatggtcgcagtggtacaggtggatttttcgcgccggcgtgtacaccgggtccatctggtttttcatctgctccacggtcaggccgcacgat
gccagccagcggcggctttcctcactcacccggtcagccaggatttcactttccagccggtcgagcatctgcaccgtgccggcgggaatcccc
cactgtgcataggcaactcacgctatgaacgcttcagcttatcaatagcggaagttataatttcattgtcatcaaacttgtacaacatagtatctaa
aacaggaagtagcgttttatcacctaattcacccgcagcttcaatgatatcatcataaacagtattttttttaactcatcgcataaaacagataaaa
ccctttttatcttttctgtaggaaagtccgattattgcttcaatacggacttcctcatttttatcctgaagcatctccacaaaacaatccctaatatcactat
tatcatatttattgatatttattgcaaatgcggcccagtttctgacatctccatttggatcttttaacagattaatcaatagtggaattgttgctttatcattg
ataacagaaatagcaaatgctgtagcacgtctgacattagtcgatttatcaaaagcagtaatttgagattgttctactattttaggtgaataaattgg
gtttttcttgcatcgctgagccgttgactcgattgccgtagctcgaacgcaagcgctcttatcattcaatgccatattgttcaaaatattaaaaacatt
atcttcgcatttttttgcaaatttttatttgcccgagtataaatgctccgatatctctacggatataatttttatcagagcagaactcaattgccaatctaac
agcgtcttgcccacctcttaactgtaatactctggcagatgaaatccttttcaaggaattgtgatcatcaagtaagcgaaataattcatcatcattta
gtttcttacattgattatataaaccatactctttacttgcctttcttttctggtatgtatttgacattgcaggaacctccacaagggctatcttttttttatcgtttg
actgtctggagtgacgacaattccaggctttctgtgtgctttttgcatctttagcactaatatcaccacaatcaattagttcctgaagtacgtcacatct
gtcgggtttttttcccatcactccagaatctgtcatttattatgtcattgacacgatcagttatccctgtgtctgcaacatttccacgagaagctatcattgt
tatatctaatgctagtcccggattattcaatattagagagtcaaatattctttgtgatgctgtatcgttccaccattttgtcaatgcttttgcagcattatcat
ctgcttgctgctgtgcagggcttttgggccatggaattggcaaggggaatggtctaggaaaaacttctaaccccagaggatctgtattcgtaactg
gattcaacggatactgataaaaattccatcccccttcagcccaatcggatcctgagtgatataccgcccctgcagcgggtcataatagcggtg
gcggttgtaatacaggccggactcctcatcatactgctgccccggcaggcggataagctgctgcagctgatgcgggttctcttcattcagcaggt
tgccccattcatcgtattctgcgcaccattctgttgtcccttccttgctgataagggccagcggcaggccgcgatggtcgcagtggtacaggtgga
tttttcgcgccggcgtgtacaccgggtccatctggttttgcatctgctccacggtcaggccgcacgatgccagccagcggcggctttcctcactca
cccggtcagccaggatttcactttccagccggtcgagcatctgcaccagcaccggcgggaacaccacactgccaccgtcttcgccgccgga
ctgctgaagcgcatccgccaggctgcggcgctgcgttttcgccagctcaccggtggcggtttcaactctgatgagtggcgtgaagctccccggc
tgataaatcgtctggatgcgggttctgtcgttctgtattgtggtcagccggtcgccgtcccagccgtaccaggtcacttgcggtttccgtgacagcg
acatccagcccgtcaggtcccgttcacgccgccacacccgttttgccaccctgcggcccagcgggtcgtaaagatagcgactttcgaccagc
ggctcttcatattgtgtccgcgtgtagtgcaccagccggtgctgactgtcgtaatggtaccggtgagtccgctcatcatccgtgcggataacccctt
ccgggatgaggtcggttttctctgtcagcctgccgtgacggtcataccggtaaagatagtgcgcgtcacgggcgatacggttatccggccacat
gctgagggtgctgtccgggtgcagctccgggtcgggcaggcggttacctgccgggtctgtggcatacgggatgcggatatccagattcgctgc
ggtggtgtgaacgccggtcagcctgccggtggtgctgtagctgtaactccgggtctggcgcgggctgctgatgcggatgagttcgccgttgtcgt

tccaggtgtaatcgcggtcagacagcaggctgttcaggtgctggctctgtaactgcccggcaggggtataagcggtggtgagttcataacggc
cgaagctgcgcagcgtttcccggtgcaggcggtcgcgggtgtactccaccagcggtgtgtcgccgagtttcatgcctgccaggtaaccgctgc
cgtaggtcagccattccacggcgggcaggctgtccggtatacagcggttcgccagcccctgcgcgttgtacgcatgtctggtctcatgctgcca
gagcagtgcttccgtctgcgggtgatgcaccgtctgacgctcaccggtcagccggcctttctcatcatacctgtaatgcaccgccacccggtgcc
cttcgctgatatggctgatgtctgtcagccagccacgttcgtcatactgccaccgctctgcggtttcacccttcacggtgcggtgcgtgaggcggtc
tgcttcgtcatagtgccagtgggtgaccagaccctcatcctcgctgcggataagtttgccggtcaggtcgtggtgataacgctgtgtgcggccgtc
aaagccggtttcctgtatcagccggtcaagtacatcgtaacggaaggtggtgtggctgccgttttcactggtcaggcggatgacccgtccggca
gcatcgtattccatactgcgcgttagcccgccctgcgtggtacggacggcctttccccacgcatcgtactgtgtcccgtttctgctgccgtccgggg
caatgacggcggtcaggtcaccggcgatgttgtattcataccgcgtttcatggccctgcgtgtctttcacggcaattaactgtccacggctgtcgta
tgcgcggtactgactcagcccttcctcgcggtgcaccgccgtcatctgcccgaagcggtcatggtcataacgggttacataaccggaacagtc
ggtgaagctcagcaactgaccgtaacggctccacgtcatggttttccggctgccggtggcatcttccgttgcgcagggtaagtcactgtgtggatt
atcataacggtagcgggtgatatcgccgtcaggggcagtttcctgaatcagacggcccaattcatcatattcccggcgcaattccagcccgtca
ggcccggtggctgacgttaactggttgtggtggttatagtaaaacgccgatgccctgccatccggcgtggttatgcgcgtgatgaggcccgtcac
cacatccgggctgtactctgttgtcctgcctgcggcatccgtctgtgccctgagcctgcccacggcgtcaaactgactctgcgtgacgctgccgtc
cgcgtgttcctttttcaccacccgcttcagcccggcttcgccctgcgtgtgcagcacttcacggcggtccaggctgtcggtgatggtgatgcggtct
ttctcatactgatacgtgtagcttaagcctgccgggtttagctgttctgtcacccgcccgtcgctgtcgtaacggtaacggatttccggccggcccgt
gtgacggtgcgccaccatccggccccggtatttatcatcgtaagtaaagctgcgcacctgtttgccactacggtcatacaccaccgccagttcg
ccgcgtggcgtccagccatagcgcaccagcggcgcggcaggtaaattctccgggtattccgggtcgtgcgtcagccacacggcagacaga
cggatgccgttgtcccggccatattcggtgtaacccggcagggtatcaggaaaagcggacggttccgtcccgccggaaatggcctgctgccg
ggcttcttctgcccgctgcgcctgcgtggtcagtaccagccggaagtgacgccaggcaccatccgtcacgccggtgatttcgccgctgaattca
ccggcggcttcgcggtggaacgtctgtgtgcgcccgaagcggtccaccagcccggtcagtacccggtacggcggcagcggcgcaggcag
cacctcatccgcttccggcacccgctcacaccaaccgagcagccaccacggcccctgcggactgtttgtcgccagataacgatgcggactta
agcggagttcttccggcagcgcctgccagagtgcggccagccggtgaccttcatccagtttcgccacgccgccgcgcaccagccacagtga
ctcgctgcggctgtaaccgtcctcaccgggaaacaggtgctcaaaatacaggcttctgccgccgttatcactgagtatcagtgtgttatcgcgca
gctgtaagcggatatccgcaggcattttccagccggggccgaggctccccaccggcgcgggcgtttttgtccggtaactgctgtaggtgcggg
agaggatgaacggcagcgggccgggcagggcgatgtcggtttcaccgggaaggacctttgcaccgagcaggggattgaccggatggccg
gacgtcactccgccggggcacaccgaacaggccacgccggtgggggcaccaatgcgcaccccggctgaaccctgaacaatgctaccgc
catactgcgtcatgtcgccctgacgcgctgccggttttccgctcatttctcatccttgttttatcgggttatttgtctgctgacaggcgaaaccctacag
atttataagaaagcctgaagtgagatatccatcagagtgaaataattaggaaaatatttatatcaccatgataaatatgtgatttatttggaagtaa
caaaccatcaacattttctgccctttttcccatcaccaggccatactgatatttttcctcgcacaaaggactcggcatgaaactcgtcggtagctac
accagcccgtttgtacgcaaactttctattctgttgttagaaaagggcataactttcgaatttattaatgaactgccctataacgcggacaacggcg
tggcgcaatttaacccgttaggaaaagtgccggtgctggtgaccgaagagggcgaatgctggtttgattcgccgatcatcgctgaatatattga
attaatgaatgtcgctccggcgatgttgccgcgcgatccgctggagtcgttgcgggtgcgcaaaattgaggcactggcggatggcattatggat
gccgggctggtatcggtgcgtgaacaggcgcgtccagcggcgcagcagtctgaagatgaattgttacgccagcgggagaaaatcaaccgc
agtctggatgtgctggaaggatatctggtcgatggcacactcaaaaccgatacggtcaatctggcgactatcgccattgcctgtgccgtcggat
atctcaatttccgccgcgttgcgccaggttggtgtgtcgaccgcccacacttagtcaaactggtcgaaaacctgtttagccgcgaaagttttgctc
gcaccgaaccgccaaaggcttgatgcgcgatatgtcctcctgacccatctcacgttacaatccgtggttatgttaaacgcccttctccgtgtgaga
gggccttgatcagccaggtttcctatgacaaccgaaacgcgttccctctatagtcaacttccggctattgatcgcttattgcgcgatagctccttcctt
tctttgcgtgatacttatggtcacacccgcgtggtggaattgttgcgtcagatgctcgacgaagcgcgagaagtgattcgtggcagccagacgct
gcctgcgtggtgtgaaaactgggcgcaagaagtcgatgcccggttgacgaaagaagcgcagagcgcgctgcgtccggtgatcaacctgac
gggaaccgtgctgcataccaaccttgggcgagctttacaggcggaagccgcggtggaagccgttgcgcaggctatgcgttcgccagtgacc
ctcgagtatgatctggacgacgccggacgcggacatcgcgatcgggcgctggcgcagctgctgtgccgtattacgggggcggaagatgcct
gtatcgtcaataacaatgcggcggcggtgttattgatgttggcggccactgccagcggaaaagaggtggtggtatctcgcggcgaactggtgg
agattggcggcgcgtttcgtattcccgatgttatgcgtcaggcaggctgcaccctacacgaagtagggaccaccaaccgcacgcacgcgaat
gattatcgtcaggcggtgaatgaaaataccgcactgttgatgaaagtacataccagtaactacagcattcaggggttcaccaaagcgatagat
gaagcggaactggtggcgctcggcaaagagctggatgttcccgtagtgactgatttaggcagtggctcgctggtcgatcttagccagtacggttt
gccgaaagagccaatgccgcaggagttgattgcggcgggcgtcagtctggtgagtttctccggcgacaagttgttaggcgggccgcaggca
ggaattattgttggtaaaaaagagatgatcgcccgcctgcaaagccacccgctgaagcgtgcattacgcgcggataaaatgaccctcgcggc
gctggaagccacgttgcgtctttatttacaccctgaagctctgagtgaaaaattaccgaccctgcgcctgcttacccgcagcgcagaggtcattc
aaatccaggcacaacgtttacaggccccccttgccgcacattacggcgcggagtttgcggtacaggttatgccatgtctttcgcagattggcagt

ggttcgctgccggttgatcgcctgccgagcgcggcattaacgtttacaccccatgatggacgcggtagccaccttgagtcattagccgcccgct
ggcgtgaattgccagtgccggtgattggtcgtatttatgacggacgattgtggctggatttacgctgccttgaagatgagcaacggtttttggagat
gttgttgaaatgattattgcgactgccggacacgttgaccacggcaaaacaaccttattgcaggcgattactggcgtaaatgctgaccgtctgcc
ggaagaaaaaaagcgcggcatgaccatcgatctcggctatgcctactggccgcagccggatggtcgcgtgcctggttttatcgacgttcccgg
tcatgaaaagtttctttccaacatgctggcgggcgttggtggtatcgatcacgcgctgttggtggtggcgtgcgatgacggcgtgatggcacaga
cccgtgagcatctggcgattttgcagctgaccggtaacccgatgctgacagtggcgctgaccaaagccgatcgcgtggacgaagcgcgtgtt
gatgaggttgaacgccaggtaaaggaggttctgcgggaatacggttttgctgaggcaaaactgtttatcaccgcagcaaccgaaggtcgggg
aatggatgccctgcgcgagcatctgcttcagttgccggaacgcgagcacgccagccaacatagtttccgcctcgcgattgaccgcgcatttac
cgtaaaaggtgccgggctggtcgtcaccggtacggcgttaagcggggaagtgaaggtaggcgattcactctggctgactggtgtaaataaac
cgatgcgtgtacgtgcgctgcatgcgcaaaaccagccaacagaaaccgccaatgccgggcagcgtatcgcgcttaacatcgcgggtgatg
cggaaaaagagcagattaaccgtggcgactggctgcttgccgatgtgccgccagagccgttcacacgggtgattgtcgagcttcaaacccat
acaccgctgacccagtggcagccgctgcatattcaccacgccgccagccacgtcacgggacgcgtttcactgctggaagataaccttgctga
actggtcttcgacaccccgttatggctggcagataacgaccgcctggtattgcgcgatatctctgcccgcaacacgctggccggagcgcgcgt
cgtgatgcttaacccgccgcgtcgcggtaaacgtaagccggaatatctgcaatggctggcgtctcttgcacgggcgcagagcgatgccgatg
cgttatctgttcatctggaacgcggcgcggttaaccttgcggatttcgcctgggcgcgccagctcaacggcgaagggatgcgcgaattgctgca
acagcctggttatattcaggctggttatagcttgttgaatgcgccggttgccgcccgctggcagcggaaaattctcgacacattagcgacttatcat
gagcaacatcgcgatgaacctggccctgggcgcgaacgtctgcgacgtatggcgttgccaatggaagatgaagcgctggtactgttgctgatt
gaaaagatgcgcgaaagcggcgacatccacagccatcacggctggctgcatctgccagatcacaaagcgggcttcagcgaagagcagc
aggccatctggcaaaaagcagagccactgtttggtgacgaaccgtggtgggtgcgtgacctggcaaaagagacgggaaccgacgagcag
gcaatgcgcctgactctacgccaggcggcgcagcaaggaataattaccgcgatcgttaaagatcgttattaccgtaacgatcggattgtcgag
tttgccaatatgatccgcgatctcgatcaggagtgtggttcaacctgcgcggcggatttccgcgatcgcttaggcgtaggccgaaagctggcaat
tcagattctggaatattttgaccgcattggctttacgcgtcgtcgtggaaatgatcatttattgcgcgacgcattattatttccggaaaaataaggaa
atgattaattaagttttaaaataaattaatacaaaattcttatgaatttaaaaaaagcacattgtttaatgaatacaatgtgctttttattagattaattttg
ataatcaataatactgatcatattttcgaatgaaaatcctcagtaagctgcccgcccttttttacactttcaggagtgtgttatggcagcttcaacgttc
tttattccttctgtgaatgtcatcggcgctgattcattgactgatgcaatgaatatgatggcagattatggatttacccgtaccttaattgtcactgacaa
tatgttaacgaaattaggtatggcgggcgatgtgcaaaaagcactggaagaacgcaatatttttagcgttatttatgatggcacccaacctaacc
ccaccacggaaaacgtcgccgcaggtttgaaattacttaaagagaataattgcgatagcgtgatctccttaggcggtggttctccacacgactg
cgcaaaaggtattgcgctggtggcagccaatggcggcgatattcgcgattacgaaggcgttgaccgctctgcaaaaccgcagctgccgatga
tcgccatcaataccacggcgggtacggcctctgaaatgacccgtttctgcatcatcactgacgaagcgcgtcatatcaaaatggcgattgttgat
aaacatgtcactccgctgctttctgtcaatgactcctctctgatgattggtatgccgaagtcactgaccgccgcaacgggtatggatgccttaacg
cacgctatcgaagcatatgtttctattgccgccacgccgatcactgacgcttgtgcactgaaagccgtgaccatgattgccgaaaacctgccgtt
agccgttgaagatggcagtaatgcgaaagcgcgtgaagcaatggcttatgcccagttcctcgccggtatggcgttcaataatgcttctctgggtt
atgttcatgcgatggcgcaccagctgggcggtttctacaacctgccacacggtgtatgtaacgccgttttgctgccgcacgttcaggtattcaaca
gcaaagtcgccgctgcacgtctgcgtgactgtgccgctgcaatgggcgtgaacgtgacaggtaaaaacgacgcggaaggtgctgaagcct
gcattaacgccatccgtgaactggcgaagaaagtggatatcccggcaggcctacgcgacctgaacgtgaaagaagaagatttcgcggtatt
ggcgactaatgccctgaaagatgcctgtggctttactaacccgatccaggcaactcacgaagaaattgtggcgatttatcgcgcagcgatgta
atcatcatttccacaacggctggcaaattgttagccgctttttcaactatctctgtaacccttgcccgtaaattcgtgatagctgtcgtaaagctgttac
cgactggcgaagatttcgccagtcacgtctacccttgttatacctcacaccgcaaggagacgatcatgaccaataatccccttcagcacagat
taagcccggcgagtatggtttccccctcaagttaaaagcccgctatgacaactttattggcggcgaatgggtagcccctgccgacggcgagtat
taccagaatctgacgccggtgaccgggcagctgctgtgcgaagtggcgtcttcgggcaaacgagacatcgatctggcgctggatgctgcgca
caaagtgaaagataaatgggcgcacacctcggtgcaggatcgtgcggcgattctgtttaagattgccgatcgaatggaacaaaacctcgagc
tgttagcgacagctgaaacctgggataacggcaaacccattcgcgaaaccagtgctgcggatgtaccgctggcgattgaccatttccgctattt
cgcctcgtgtattcgggcgcaggaaggtgggatcagtgaagttgatagcgaaaccgtggcctatcatttccatgaaccgttaggcgtggtgggg
cagattatcccgtggaacttcccgctgctgatggcgagctggaaaatggctcccgcgctggcggcgggcaactgtgtggtgctgaaacccgc
acgtcttaccccgctttctgtactgctgctaatggaaattgtcggtgatttactgccgccgggcgtggtgaacgtggtcaatggcgcaggtggggt
aattggcgaatatctggcgacctcgaaacgcatcgccaaagtggcgtttaccggctcaacggaagtgggccaacaaattatgcaatacgca
acgcaaaacattattccggtgacgctggagttgggcggtaagtcgccaaatatcttctttgctgatgtgatggatgaagaagatgcctttttcgata
aagcgctggaaggctttgcactgtttgcctttaaccagggcgaagtttgcacctgtccgagtcgtgctttagtgcaggaatctatctacgaacgctt
tatggaacgcgccatccgccgtgtcgaaagcattcgtagcggtaacccgctcgacagcgtgacgcaaatgggcgcgcaggtttctcacggg
caactggaaaccatcctcaactacattgatatcggtaaaaaagagggcgctgacgtgctcacaggcgggcggcgcaagctgctggaaggt

gaactgaaagacggctactacctcgaaccgacgattctgtttggtcagaacaatatgcgggtgttccaggaggagatttttggcccggtgctgg
cggtgaccaccttcaaaacgatggaagaagcgctggagctggcgaacgatacgcaatatggcctgggcgcgggcgtctggagccgcaac
ggtaatctggcctataagatggggcgcggcatacaggctgggcgcgtgtggaccaactgttatcacgcttacccggcacatgcggcgtttggt
ggctacaaacaatcaggtatcggtcgcgaaacccacaagatgatgctggagcattaccagcaaaccaagtgcctgctggtgagctactcgg
ataaaccgttggggctgttctgatataagaagctggtcgcattgggtattcattgcctgatgcgacgcttacgcgtcttatcatgcctacgggaacc
tgaccgtaggccggataaggcgtacacgccgcatccggcataaacaaagcgcacgttgttacaatttacgttttaaacctacgtgcgcaataa
cccagactacgccaacagaagtacgtttctggggcgtgttttgtcaggagtttaatctcgcgtttcgatggtgagcgggcgaaccaggccgtaa
atcaccattggtgatccgaggccaatcagcaataaacggtagataattgaaggtttaatacgcttaatctgtactgtggatgttttactggagttatt
cattagtttggccaatagttccactatttcaatatgataaacctcgtcctgttgaaaaaccagacagcacatttaagaaagtatataaaaaacaat
atgtcgttatataaataaggaaattgcactgcatcatggcatttcataatatgcgttaattgttctttttatattctttgagggaaatatgttcctggacta
ttttgcactgggagtgcttatttttgtatttctggtgatcttctatgggatcattattttacatgatattccctacctgattgccaaaaaacgtaatcatcctc
atgccgacgccattcatgttgctggttgggtgagtctttttacgttacatgttatctggccgtttttgtggatttgggccacgctttaccgcccggagcg
gggatggggaatgcaaagccatgattcatccgttatgcaactgcaacagcgcattgccgggctggagaaacagctcgccgacatcaaatcct
cttctgccgagtaatacttatggatttactgattattttgacctatgtggcttttgcatgggcaatgtttaagatcttcaaaattcccgtaaataaatgga
ccattcccacagcggccctgggtggaatatttattgtcagcggtctaattctgttaatgaactataaccatccgtatacctttaaagcgcaaaaagc
ggttatttctattcctgttgtcccacaggtgactggtgtggtgattgaagtgacggataagaaaaatacgctgattaaaaaggtgaggtgctattt
cgactggacccgacgcgttatcaggcgcgggtggatcggctgatggcggatatcgttaccgcagaacataaacagcgggcgttgggcgca
gagttagatgagatggcggcgaatactcagcaggcaaaggccacgcgggataaattcgctaaagagtatcagcgttacgcacgtggcagc
caggcgaaagtaaacccgttttcagaacgcgatatcgatgtggcgcggcaaaattatctggcgcaggaagcctccgtgaagtcatcggcgg
cggaacaaaaacagatccagagccagctggatagcctggtgttgggtgaacattctcaaatcgccagcctgaaagcacagctcgcggaag
caaaatataaccttgagcagacgatagtgcgtgcgccgagcgatggttatgtgacccaggtactgattcgtccgggtacctatgccgcgtcgct
gccgctacgtccggtgatggtgtttatacccgatcagaaacgacaaatcgtggcgcagttccgtcagaactccttgctgcgactggctcctggc
gacgatgcggaagtggtgtttaatgctctgccaggtaaggtattcagcggtaagctggcagccattagtccagccgttcccggcggagcttatc
agtcgaccggcaccttacagacgttaaacacagcgccgggttcagatggcgttatcgcgaccattgaactggatgagcacactgatttgagcg
ctttaccagacggtatttacgcccaggtggcggtttactctgatcatttcagccatgtctcggtgatgcgcaaagtgctattacgcatgaccagctg
ggtgcattacctttatctcgatcattaatcttcgctttccgccgcttgttcaggccgcagttgctgcgactgagcaagctggtggcgaatttcacgca
gcatttcatccagcacatactggacccgccagggctggtattcccgtttacggaagatagccaccagatccagccaccattcatagggttgctg
aaagcagggcaccagcgtgccgtcttgcaggtcgctctgcacacttttgtctggcgcaaacacaatccccaggtgatttcgtgccagctccagc
gcagattgcgtgttgtcgcaaacgtaatttcccttcacgcgataatcccgcacctctttactcccggcgacgttgaagcgccagatattcgcgtcgt
cgatcatcatcgagtcgatcaaaatacaggagtgatgttcgagttcgtcaggacggctaatgggatgtttctcaagatagcgctggctggcata
agcggttacggcatattgggtaataaaactggcaaccagcgattcatcttttggtggagcgtagctgattaaaacatcacaatcatcaggaaatt
cgacgccttcataaaaggcattacgctcaagattgcaggtttttagcgataaggtgatatcaccgatatctttaattttatcgattacatgtttggata
aataggtaattatgccggttggggcgtagatggtgacccgaccacgtttctcatgcttataatctgcaataaaattattaagttgctcgtttctgtcca
gcatgtcgttgatgtacggtaacagcgcggtgccaaaaggtgtgagcatcagctgccgggtggttcggtcaaagacttttaaacccacttttgatt
caaaatcagcaagatatttgctgacgttggcctgtgcgatgccaagtacagtcgcggcatggctgatattttcactggcagcgattaccgagata
atttttaactcccggtacttaagttgtaattttgtcatcgcaacagcccaattatatataattttatatattactatataaatatgaatcttgcaccggaga
aattgtaagcattattatgccgactctttttatcttattttaaaatggaataccggacatgttaattaatcgcaatattgtggcgttatttgcgttgcctttta
tggcaagcgcaactgcttctgaattatccattggtgctggtgcggcttataatgaatcgccttatcgcggttataatgaaaatacgaaggcaattcc
gctgattagttatgaaggtgatactttttatgttcgtcagaccacgttaggttttattctgtcgcaaagtgaaaaaaatgaacttagcctgaccgcatc
ctggatgccgctggaatttgaccctaccgataatgacgattatgccatgcaacagcttgataagcgtgatagtacggctatggcgggggttgcct
ggtatcaccacgagcgttggggaaccgtgaaagcctctgcagctgcggacgttctggataacagcaacggctgggtgggggagctatcggt
attccacaaaatgcagataggtcgtctgtcgctgacacctgcgctgggcgttctctattatgacgagaatttcagtgactattactatggcatttcag
agagtgagtcccgtcgtagcggtctggcaagttattccgcgcaggatgcctgggtgccctatgtcagcctgacggcaaaatacccgataggag
agcacgtcgtattgatggcgagcgcaggatacagcgagctgccggaagagattaccgacagcccgatgattgatcgtaatgagagttttacct
ttgtcaccggggtgagctggcgtttttaattcaccggtggatgtcggtgcggctggtgctccgtcgtaatgccacaaaggttgttgctctggattgct
ctgttcataccgggaacctggcgtctgaatcaggatcttatggctgaacaatcccttgccttcaaatgtcgtaaatggcgcatcggcgggcagca
acaggttatgcagtgactcgacgttgtcggcctgctccatgcaatacggtttaatgacgttggctatttccggcagatttttcagaaacatactggttt
aacgccagcagcgtggaagcggcgttacagcaaggaacatatcaattcgtagtgccggggcgatgaagccccggcgtgagggattactgc
ccgtaataggcattggccccatgcttacgcaggtagtgcttatccagcagttcgttttgcatcgcagggagctgcggcgcaagctggcgcgaga
atagacccatataggcgcattcttcgagtactacggcgttatgcacggcatcggcggcgtttttaccccatgcgaacgggccgtgagaatgcac

cagcaccgccgggatttgtgccggactcctgccacgttcttcgaaggtttcaatgatcacttcgccggtctgatattcatattcgccgttaatctcctc
tgcggtcatctgtcgcgtgcaggggatggcaccgtaaaaataatcggcgtgggtggtgccccaggcggggagatccagcccggcctgtgac
cagatggtggcgtggcgcgagtgggtatgcacaataccgccaatttcggcatagcgacggtagagcgccagatgcgttggtgtatcggaaga
gggtttttgctgccttccaccaccttaccgctggctatctcaaccaccaccatatcgtcggcggtcatcacgtcgtactcgacgccggaaggtttg
attaccatccattgccgcgtttcgtctaccgcgctgacattgccccaggtgaacgtcaccagatggtgagcgggaagcgccagattcgccgcc
agcacgtcggctttcagttgctctaacatataaatccagcctcctgcatacgcgcttcaatccaacgccgcgcctgaataatctccagcaccggc
tctttggctttttcggtccacatctcaatcaggaaagaaccgcgatagttcagtttatgcagcgttttgaagatgccaacgaaatcgacgcagcctt
cgccaaacggcacgtcgcggaactgtccgggactttgcccggtgactggctgggtgtctttcaggtggatcgcggcgatacggtcaatgccca
gtttcagttcggcgggaacatcattgccccaggcgctgaggttgccgacgtccgggtagacggtgaaccacggtgaggcgagcatctcgtcc
catttttccatttgctgatggagttcataaacgcggtatccataatctccaccgccagcattacttgtgatgccgccgcctgttcgactgcccacgc
cagcccttcagcaaaacgttgccgggtgccttcgtcgtggtcttcgtaatagacgtcataacctgccagctggatggtgcgaatgccgagatcg
cgcgccaggcgaatcgctttactcatgatttcccgcgcccgttcgcgcactgcctcgtcacggctaccaaagggaaagcgacgatgtgcgga
caggcacatcgacgggatcccaacacctgtttcgatcatcgcggcaaccagcgaagtcctttgtgcggcgctccagtcaagacgtgagagcc
gttcgtcggtttcatccaccgacatttcgacaaaatcaaaaccgcagctttttgccagcaccagccgctccggccaggagagatctttcgccag
cgctttttcataaatccctaactgatgattacgcacgcgcacctccccagatagcgtcgatttgcgcatggaaatcgccagccacttgcgccgga
tttgccgcgcctgccagtgcccgcccggcaataaacgctttcacgcggatatctttaaacagcggcaggtcagcaggggtaatcccgccagta
atggaaagctcaaggccgatatctgaaagcgccttcatgcgtgccagatcggcttcgccccactgttgcccgctggcctgtgcatcacgaccg
cgatgataaatggcctgccgcacgccaatacgatgccagtcgcgggcgtcgtccagcgtccagttaccgaacagctctatctgaatttcaccc
ccgcagcgttgtgccattgcgtggcctttttctaccgtcgcgagcggcgcggcgcagatgatggtcatccagttagcgcctgcgccaaacgcct
gttgcgcgagcgtttcaccagcgtcggcgaccttccagtcagcaacgatgattttgtccgggcactgttcgcgcaaggctttcaccgcgccaag
cccttcgtttaaacagagaatggtgcccgcttcgacgatatcgacgctgtcttttaacagcgtcacgtcgcgctgcgcggcttcaagtgatgagtg
gtcgagggccagttgcagaagtggtcggctcataatgtgtgctccttaatgcgggcgtgaaagccctgaagtgcggcaatgagatgctgataa
cgttgatatttttttggtaaagctgatgggcggtcatatctggcagcagggtgcgcaccgggtgtcgcaagtcacgttgggcttcgctgaagttgt
gataaaccccggtgccgacgcgggcggcaagggccgcaccaaagcagccggtttcttccacctgcggcagctcgatacgcagaccgctg
acgtccgccagcatttgcatccagacatcggagtgcgccgggccgccagtgacgcgtagggtgtgaacatcagtaaaacgttcgcgcattcg
gttgaggtgggtcatatggctgaacaccaccccttcatagatggcctgcaacaggtgcgcgcgggtgtgaatggcctgcatcccgtagaaacc
actggtcatctcgagtccggcgttgctgccgtacaggaacggcaggaaaaagagatcgcccccagccttcggcaagctggcaacggcctg
attgatctcatcaaacgagatttctccccactgtgcggtaaaccattcgaggttgccggaagaggtagggctggcttcgtgaacgataaattcac
catcgttaacgtagcgaccatagacatacggatgcgcttcaccgtcacgtaaaccgcgggttatgccgctggtcaccgcccaggtccccatca
ccgcattgagggtaaattcgtcttcgatcccggcgcagagtgcggtggaaaccacatcaaacaggccgccaacaacgggcgtacccgctttc
agaccggtcagggcggctgtctgagcggtgatctccccgcagatttcggcagatccgacaacaggcggcagggcgtgattgatttcagcgat
ccccagccagtcggtgaggcacgggtcatattccccaagactcatgttgtagaggttggactcggaaatattgctctcttcacagcctttgacgc
cagttaaacaccagcgcaggtagtcgtgcgtcatcatcacgcagccaatttgcgcgtagcgttctggttcgtgctctttcagccagcgtaacagc
gacaccggatgcccggtccacaaggtttgtcgggtcagcgggtagagtttttccgggatgccatcttcctgccagcgacgaacgatttccatcg
cccggcggtccgaggacaaaatagcattcccgagcggtttgtcgtttttatccagcaaaaacaagccctttccctgtgcggagatgccgatacc
gacaatttgttccccgctaacaccagaatgagtaagcagggcgcgaatgacagccatgcagcattgccacagttctgccatatcgcgctctgc
ccagcctggctgcgggcttaatgcgcacagcggcaggcgctgcacgcctgcctcccggccttcgcggtcatacagcccggctttcagccagc
taccgccacaatctaaccccagccagtattgcgtcatggtttattgcacctcatccacggctttcagcagatcagctccctgcggcacatctttaac
aaacatgtcgcgaacctgattccccagtgcgtcgctaaaggctttgcggtcgagatcggtgatgacttccacgcccgcttctttcatgccgtcgat
gattttttgctgatcttcagcaaccagtttgcgctgatagttacccgcttcttgtgcagatgaaactagcgcctgctggaactccggacttaagccat
caaacttcgctttgttgatcaccaccagaagcggggaataggcgtggtgcgtcagagaaaggaacttctgcacttcgaaaaattttgctgacca
gacgacgttgatcgggtgttcctgagcgtcgatagtgcgggtttccagcccggtatagacttcggcaaacggcatcgggatcgggttagcgcc
aaagactttgaatgcggcgatattcatcgggctattgttggtgcggattttcagccctttcaggtcggcgggggttttaaccggtgcgcgcgagttg
gtgacatcgcgccagccgttttcccagtaggccagtactttcagtcctttaccttcaagtgaggctttcagatcatcaccgactttgccgtcgagcgt
tttatgcgcgtgagcggtatcgcggaacaggaaagggacatcaagcaagttcatcactggtgataacccggcaaagttattcgagccggaca
tttccatatcgatggtgccgccacgtacgccgctgatcatcgcctgcgcgttaccgagagtgctgtccgggaacagttttaatttcagctcgcctttg
gttctctcctgcaataaatcattgaatttttcgccgcaatatgttgcgagtcggtttgtgatgtttcataaccgaaacgtaaagattgtgccgccaga
gaagatgtgctgaatgcagccaggccagcaatgaataatgcgtaggttacagagcgtaatttcataatattttcctttaattaatccatttcaaag
ggaggatgatgagatcgggaataaaaacaaatacgactaataatgaatataaaaccaggacgtaagggaaaacgcctctgactgcatcat
cgaatttgagttttgccacccctgaaataacgttaagtacattaccgataggcggtgtaattaagccgattgagcagttaatgataaacatgaca

ccgaaataaatcggatcgattcctgcctctttaactaaaggcattaataccggggtaagaattaataccgtcggcgttaaatccatgaccatgcc
gacaatcaggatagccaccataatgacgataaacagcagacgcggtgaatcgaccaacggttgcagtaaatcagaaaccatcatcggca
gttcagcaatggtaatcagccaggcggaaacttgtgccgaggccaccagaaacataaccactgaggtggttttcgccgcgccaatgagtaca
tgccaaagtgtggcgaaggtcatttcacggtaaataactgtggcgacaaacagcgcatagaaagcagcaacggccccggcttcggtaggg
gtaaacaggccggagcgaaacccgccaataatgatgactggaagaaacagcgcccagataccggagacaaaagagtgccagatttcct
gcatcgttgctttttgctggcggggtaaattaaggcgactggcctgccaccaccaggtaagcatcagcgttgcgcccatcatcatgccaggggc
aatgcccgccataaacagcttactgatggataatccgctggaaacgccgaagataataaacggaatggaaggggggaataattggcgcgat
aatgccgccagaggcaatcagccccgccgcccggttgaccggatagttggcgctgcgcatcatcggcaccagtagagcggcaacggcgg
cggtatccgcaacggcagaaccagaaaggctcgccataatcatagctgccagcacgccgacgtagcccagcccgcctggtttatgccccac
cagtttcatcggcaggtcaacaatgcgttttgacaagccgcccgcattcatgatttcacccgccagcacaaagaacggaatcgccagcaggg
agaagctatcggctccgttcaccagcgtttgtgccatgatctggacatcaaacatgtccagccagaacattaacgccgccccgcacaacaac
agtgcccaggcaataggcaaaccgatagcaataccacccaacagacagcccagaaaaatcagcacagccatgattaagctccttgcggt
ggcgacgttagagaattgctacgcgtgatgagttgatataaatgacgcagttcaaagaatgcgataacgaggctggtgggcagacaagcgg
catacatcaggccgatgggtaaaccgaggatcggtgaataatcgctccagtcctgaattgtttttagcgttgcgccccaggccagtgcgccaca
aataaataagattaaggaatgggtaaccagagcgactcgtcgctgccatgcgggggagagtttctccaccagaaaggtgacctgaacgtgg
gcgttatccataaaagctacaatcgcgccaataaacgttaaccagacaaataaataacgtgacaattcatcaacagataaaatgcttgtctga
aaaccatatcttaaaataatgtttataaatacaatacaggaaagtacggcgagattaatcgccagtattgcttcgagtattttttcatagctattcctt
gaggctatgtctgtcataattcaagtcgcatgtgcagcaacctgaattattaatatagttagagcaataacattacccaggcgttctggcgtagac
gattattcgattaattcagcgccgttaatgcgactttaaccacaatttttcgtatctcagaggctgtttgcataatacaacctggtcggtgaacatcct
gcggaaagaaaattgcgtagctgcccggtatcatttctataaatgattcatgttcactgtcgtgataaaaaataatattgcgctgctctaatagtgat
tcgctgactttattatttcccgtatcaatagcaatgccgattttctcttcgccccacgccagaaactgaatatcgatataccgacgatgcacttccgg
acggtttaccaccgcttcgcgtgtggttaaatcgataatttgcgtataaatatttttgccgtcgatttcgacaacgcccggctccagggcgttgaaat
cggtagcgcgcagaaaatcgagcgccttttcaatggcggcgggcaaacggcacggattgggctgcgcgatatgtccaaaaatcatgacttat
ctcctcataacgcctggattttggcccacacgctgtcatcaacagtgatgccgttacggcggttttcggccagcagggtagtaaattcatggccg
ggtaagcgaatggcctgattttcgtcagcacgctcggcactagtaacgtaatccatgatgcgttgcagcttggcatcgcgggtgggaccgtcga
taagcttgtccacttcaatggcaataaaaatttgtgaaatgccgtattcgtcgctgttgtcctgggtgacttcggcaacggatgcgccgtcggaaa
ggagagtagcgatcatatccagcacaatcgacatgccagaacctttccagtagcccatcggcaaaatgcggcgattcttctcgataacgcca
ggttctttggtcaaattgccctcatcatcaaagccaccatcgaccgggagctgacgacctgccagacggttaacttctaacatgccgtaagaga
acatcgacatcgacatatcgaccatggtgatcggcgtggaaggaatggcgacgatcagcgggttagtgcctatgcgacactcttttgcgcccc
acggcggcattacggcgatggagttggtccagcaaatgccaatatagcctttttccgccgcctgccagccgtagctgccgccgcgcatccagt
ggttggcattacgtagtgccaccagaccaataccgtgatcggcagccagttcaatggcgcgatccatcatctttttcgctgtcaggttaccgatcg
aacgctgggcgtcccactgttcaattgcgccgaggctggttatacgtttgggttgggcatcaggaatgatatcgccgttttccagttgttgaatgaa
acgagggaaacgattaacgccgtgagaataaacgccggattcggtggtgcgggcgaacatctctgcacaggcgtcagccgtttcgctgtca
acgccgcgtgaaattaagacccgattaaaggctgcttttaactgctcaaatgtcactttcatcccaggcttccttgtttttttgatcgctttttttggcttaa
aatttcaatatgcgaaacttgatttcaaatatatcgatcacttttttaacaaggcaatctgattgatgaatttcaaaagacataaaaatcaattggttat
aaattatctgtcgagatcgtgaactacggcacactttgcgctaccatcaggacgcgacaaaatggggaaagaagtgatgggaaaaaaaga
gaacgagatggcgcaggaaaaagagcgtccagccggaagccagagtctgtttcgcgggttgatgctgattgagattttgagcaactatccaa
acggttgtccgttggcgcatctttcggagctggctggtttaaataagagtaccgtccatcgcttattgcagggattacagtcctgcggctatgtgac
caccgcgcctgccgcagggagttatcgcctgaccaccaaatttattgccgtcgggcagaaggcgctgtcttcgctgaatatcattcatatcgccg
ctccgcatcttgaggcactgaacatcgccactggtgaaaccattaacttctccagccgcgaagacgatcacgctattttgatttataagctggaa
cccacaaccgggatgctgcgaacccgtgcctatattggccagcatatgccgctctactgttccgcaatgggcaagatctacatggcgtttggtca
cccggactacgtgaagtcatactgggaaagccatcagcatgagatccagccgttaacccgcaataccattaccgagctgcccgcgatgttcg
acgaactggcgcacattcgtgaaagcggagcggcgatggacagagaagaaaacgaactcggcgtctcctgtattgctgttccggtgtttgata
ttcatgggcgggtgccgtacgccgtgtcgatttcgctttcgacatcacgtctgaaacaggtgggagagaaaaatctcctgaaaccactgcgtga
aaccgcgcaggctatttctaatgaactgggatttactgtcagggatgacctgggcgcgatcacataacgctttggacaagtgccaaaactttaa
catttccttcgttggatcaaagcagtagggacgcgctctctggcactctgctgttttagtgcaaaggagtgatcatgaaccggtttattattgcggat
gcgacgaaatgtatcggttgccgtacctgtgaagtggcttgcgcagtgtcgcatcatgagaatcaggattgcgctgcgttgtcaccagacgagtt
tatttcccgtattcgtgtcattaaagaccactgctggaccacggcagtagcctgtcatcagtgtgaagatgcaccgtgcgcgaatgtctgccctgtt
gacgcgataagccgcgaacatgggcatattttcgttgaacaaacacgttgcattggctgtaaaagctgtatgctggcttgcccgtttggtgcgatg
gaggtcgtttcttcgcgcaaaaaggcgagggcgattaagtgcgatctgtgctggcatcgggagacgggaccggcctgtgttgaagcctgccc

gacaaaggcgttgcagtgcatggatgtcgagaaagtgcagcggcatcggctacggcagcagcctgtttgaaacgttttccggcgtttctcaac
gccggaacagtgcgctttgtttatgccagatgcggcgtaaacgccttatccggcctacaaattcttccaaattcaatatattgcagaaatcatgta
ggcctgataagcgtagcgcatcaggcaatttttagtgactttcagcccaggctctttctatctcttccgccagaatcttcacccccgcctcaattttct
ccggctctggtacgtagttcatgcgcatacattgatgcgtatgcggccacggtttatccagccctgggaagaagttgtgccccggcaccatcag
cacgccgcgtgctttcaggcgctgatagagctgcttggtcgtaatgggcaaatccttaaaccatagccagaggaaaatggctccttccggtttat
gaatcaggcagcgattttccggtaaatagcggcgaatgatggcgatagtttcctgaacacgctggtagtaaaacggtttgatgactgtttcagac
aggcgcagcagatcgttacgcttaatcatttcacacatcatcgccggaccaataccgccaggtgccaggctgataatgccgttcatattggtgat
ggcggtgatgattttttcattggcgatgataatgccgcagcgggagccaggtagacccagcttggaaagactcatgcacagcacgatattcgg
attccatagcgggcgcgcttcactgaagatgatacccgggaacgggacgccataagcgttatcaatcaccagcggaatgccgtgttgattcgc
cagcgcgtcaagcttcagcaactcttcgtcagtaatcacattgcctgttggattcgtcggccgggagacgcaaatcatcccggtttcttcgccaat
atgcagatgctcaaaatcgacgtggtatttaaactggccttccggcagcagttcaatattcggacgcgcagagacaaacagatcttcttccagtc
cggcgtcagcatagccaatgtattccggtgcaagcgggaacagcactttttgacccgaccatcggcacggcgtccggcaaacaggttaaat
aagtagaaaaacgcgctctggctgccgtttgttagtgcaatattctgtggttcgatatcccaacccaacttctcgcgcagcattccggcaagcagt
gtgagtagctccgttttcccctgtggaccgtcgtagttacacagtgcatcagtcgctttgccactttccagcatgtcggtcagtagcgtctggaagta
gtcctgcatttccgggatctgcgccggattaccgccgccgagcataatcgcgccaggcgtgcgtaaaccgtcgttcagatcttccatcaacagc
gtaatgccggagtggcgggtaaatttgtcaccaaaaagggagaatgtcatagcgggatatctgtcgaactctgtagcaaggaaggtaacaat
aacgctacatttgctggggtgcaaatcggtctgttgaagagtgagcggtgctttatcctgcaacgctgattagggctgacattttatcctggttggcg
gcttcccgaggtactgacgaacgggaagccggaaaagttactgttgccctgcccagacgaccagcactttatcgtcgccatgctcacgaaca
aagccgtagccctgcttcagcaaaagtgtcgtttgtttgcccgcgccaattgcgggatggcgggcgcggaactggctgattttctgccagtgcgc
gacgctggcggcagatttaccgctaacatcctgccagttcatatccgaacgtgtaccttgcagcggatcagaacctgtaggaccgaacggac
gcgaggattcatcaccataaaagatttgtaccgcgcctggcgctaatagtaataactctgctgctttgtcgccccccttcacggaacaggcgggta
tcatgcgacgagaggtagctcaacacgttgaaaccctgcaatttctccgccatttgctgccaggtcgtatccatctgcgccagacagtcgactgc
tttcgccgcctgctcctgataatcgaaattgatcatcgcatcgaagccgtggcgatagtagtcactttgcatcacgccgtggccccaggcttcacc
ggtcatccagaaaggtttgtcatctaatgctttgtcggggttagctttttccattcgcgaagcgcggcgctggcttcggttttcagttgctgccaggc
gggcaactcaacatgtttggcggtatcgacccgaaaaccatcaatcccatagtcgcggacccactgacttaaccagtgggttaagtaatcgcg
cggcgtatagccgtcaatggctttggcgtgggtatccattttgtttttatagaacaccggcagaccagaagcggtagttgattcggttttgatatccg
gcaaaaaggctagcgacatagtgagatcgtcgaatccaggattgtcgtaatcgccgatatccgttctgatccagtttttccccaccatttatccca
gcctgttttgtcgctgaaattaatgtaatcgttaaagctatgccaggtttgcccggcggcaggtttccagtcgctccagcgttcacccagcgattttt
cacttcgtcaccagaaagatataacgcgccaaactgatactcctgcatatccgccagcgtggcatagccggtgtggttcatcacgacatcaaa
gagaatacgaataccgcgctgatgtgcgctatcaaccagcgtccgtagatcggcttcgttgcccatattggcatcaagattcgtccagtcctgtgt
gtaataaccgtggtaggcataatgcgggaaatcgccttttgtaccgccgccgacccagccgtgaatttgctcaaatggggcgcttatccataaa
gcattaacgcccaactgctggaggtaatccagtttgttggtcaggccgcgtaaatcgccgccgtgaaaagtgccaatttccgccataccgtcttt
atgacgtccgtaactctggtcattactgggatcgccgttttcgaaacgatctgtcagcacaaagtaaaccgtggcgttatgccagtcgaaaggg
gcggatgtgtcagtttctgcccgttccagcaggagtaaaccgttgctggtggcagcgggttgtaacattatttgaccgttcttcactatcgcaatttg
ctggctgtaataatcccgtacggcggctccttccgggaaagtggcgctgacatccactgtgagcggtaatccatcccatttcgggcattcacgg
accaggcttgctaccggttcggcggcgttctggatggaaatcatcaatgttggcgtaccggagcgggtgtctatttgcagcgtatattcgccgtcc
ctgaacaatcgccattgaggcggcgtgttgctacaaggttgcagggaaagcatctgattgagttttatcgcatccgcaggctgccagcactgttg
gtcaaaatttagcgttagtggacgcgtacctttgggcaactgcgcgtggctgacaaatgttcctgtcccctgttcgctaaaggcgggaaaccccg
gagaagtccagctggcggcaacggcgaagccaggaaggagtgtcagaaaacaggcggcgagtttcatggcggatgagtccttattgctgc
gatttcagacagtttgccagcgataagccaaacaaaactcatccctgagcggtaagtccacaggatgagaagcaagggtgagcgatctcgc
gcaaaaaacggctgaattttgcgataacccccacatttctgcgatttagcgccaatctgaatcgttaacacgtgacatagtttcagatttggacta
ttccttggggtgctcttgacagctatttttgatatgatttgagattcaactctcaaatttgtgaaaaatataaggtgttggaatgattaaatccgaccag
gagacctgatgattttgactcccatacgacgatatggggcgatgattcttatgttactcactctggtgttttcgagtgaggtgttagcgaagacgcac
acaacaacagcgagtcaaaagtcccacttaactaaagctagtaataaacaggtaagcagtaaacaagagtattctcgcaatagtgcaaag
agtagttcacttcctgatttgcgaaaatacccttccggaacaccaaggaaaaaggcgtttctccggaccgtaatgccttacattaccagccaaa
atgcggccattactgcggaacgtaactggctcatttcaaaacagtatcagggccaatggtcacctgctgagcgtgcgcgtctgaaagacatcg
ccaaacgctacaaggtgaagtggtccggtaatacgcgaaaaatcccgtggaataccttgcttgaacgcgtagacattatccccaccagtatgg
tggcgacgatggctgcagcagaaagcggttggggaacgtcgaagctggcgcgcaacaacaacaacctgttcggcatgaaatgcatgaaa
ggacgttgtaccaatgcgccaggtaaagtgaaagggtactcacagtttagttctgtcaaagaatcggtgagcgcctatgtcactaacctgaata
cgcacccggcttactcttcgttccgtaaatcgcgtgcgcagctgcgtaaagcggatcaggaagtgactgccacagcgatgattcacaagctga

agggctactcgaccaaagggaagagttataacaactacctgttcgcaatgtaccaggataaccaacggttaatcgcggcgcatatgtaattgc
atttccttgagccttatccgacttgtcagtcggataaggctttttactttgtctcaggcagttgagctacgagcctgaagcgttgttggtgcgttttatcat
gcctggcgggtaggtcggataaggcgttcacgccgcatccgacaaccacgcagcgttacctgatgtgacgccgacaattctcatcatcgctac
aacatgacctcgctatttacatcgcgatactcttttggcgtcgtgtcatatgctttttaaaaacagagtagaaatattgcagcgatggataaccgca
catttgcgatatctcattgatcgacaaggtggttgaaatcagcagactgcgcgctttctccagcttctcggcatgaatcatggcatggatggtttca
cccacctcttctttaaaacgcttctcaagattggagcgcgagatcccgaccgcatccagtacctgatccactttaatccctttacaggcgtgattac
gaatgtaatgcatggcctgaataacggcgggatcggtcagcgagcgataatctgttgagcgccgttcaatgacgcgaactggtgggaccaaa
attcgctgtagcggcatttcttctttatctaataatcgatgcaacagttttgccgcctgatagcccatttgccgcgcgccctgagcgaccgaagaaa
gggcgacacgcgacagatagcgggtcagttcttcgttatcgatgccaatcacgcataatttttccggtacgggaatatgtagatgttcacatactt
gcagaatatgccgcgctcgggcgtcagtaacggcaataatcccggtttgcggtggtagcgtttgtagccagtctgccagccgattttgcgcgtgtt
gccagttctctggcgcggtttctaacccctgataaaccactccgcgatacttttcttcggcgacaagctgacgaaatgcatattcgcgctcagtgg
cccaacgtttgccgcttgattccggaagaccataaaaagcaaagcggttaacgcctttctcttttaaatgcaaaaatgcgctttcaaccagcgca
tagttatcggtggcaatgtaatgaacgggtgggtaactttctgcaaggtgatacgagccgccaaccccaacaatggggacgtcgacatcagc
cagcgcttgctcgatctgtttgtcgtcgaagtcggcaatgacgccatctcctaaccagtccttgattttatcaatgcgggcgcggaaatcttcttcaa
tgaaaatatcccattccgattgtgacgcctgtaaatattcccctacgccttctactacctgccggtcataggctttattggcattgaacagtaatgtga
tgcggtgacgtttagtaaacatggttcttttcctgctgaatcatgcaaaaactcaaaaccggtaatacgtaaccggctttgagaaaatttttatcaa
aatcaagaacggcgtttggttgcggagtccatccatactgccagcaacagaatcgcacctttaacgatatactgccagaaggtcggtacatcc
atcatactcatgccgttatccagtgaagccatgataaatgcccccattactgctccggcaacgcttcccacaccgccagccaggctggtgccgc
caatcacgcatgctgcaattgcgtccagttcggcgatatttcccgcagaaggtgaaccagcgccaagtcgagaactaaggattaatccggcg
atggctaccattaatccgttaatcgcgaacacggcaagtttggtgcgttcaacgttaatcccggagagacgtgctgcttccagattgccgccgat
ggcataaatgcgtcgtccaaatgccgtccgcgttgccataaacattccgccgagtaacagcaacgtcagcagcagaacaggagtgggaac
gccacggtaatcattcaacagccagattgcgcctaatacgatgatagcggttaaagcctggcgaccgactactgcggtagaggccggagact
gtaaacccaaagcctgacggcgcattcttccgcgccattgccaaccaacaaaagccattaagccaagcgcgccaatgatgaagccggtact
ggcggggagatagctttgcccaatttgtgacatcgcggcgctggtgggggatacagtcgtgccgttggtgatgccaatgagtatgccgcgaaat
gccaacatgcccgcgagggtgacaataaatgaagggactttacggtacgcgacccaccatccgttccaggcaccgagaagcagtcccag
aaccagcgtcacaatgatggtaagtggcaaaggccagcctaaccagacgtcacaaatcgccgcgacgccacctaacagccccatcattga
gccgacggaaaggtcgatttcagcagaaattatgacgaacaccattcctaccgcgaggatgccggtaatcgcggtctggcgtaacaggttgg
agacgttacgggcgcttaagtaggcaccatcggtggtccaggtaaagaacagcatgattgcgatgatagctgcaatcatcacgaagacctgc
aaattcagtgatttcagcccggagaagccaccggatgtcggtacggccaatttcacttcagacggattgcttttcgacatgatgttcgctcctcaat
gcggcttccatcacctgctcctgagtcaggttatgatttatcaggttggcttttagtttcccttcatgcatcaccagtacacgatcgctaaggccgag
cacttcaggtaattcggaagagatgacaataacggcaataccctgctggacgagttggttaattaatttgtagatctcgtatttcgcgccaatatcg
ataccccctggtgggttcatcaagaatgagaatgcgcgggttaagtaacagacagcgagcgaggatcgctttttgctgattgccgccgctcaaa
cgtccaatagcaaggtcgggggacgacgttttaactttgagttgctggattgattccagaatacatttttgctctgccgcgtcatcaagctggctaat
gccaccggtaaatttattgagtgcggcgagggtaatatttttaccaaccgccattaccggaacgatgccgtcgcgctttctgtcttcggggaccat
cgcaatcccctgggcgatggcttgctgacagttacgaatatctacctgtttgccatcaatataaatttttccttcccattgtccgggccacacaccaa
acaggcactgaatggtctcggtacgtccggcaccaacgagtccggcaatacccaatatttcgccacgtttcagggaaaacgagacatcatta
actcgtttaatatgacgattaaccggatgccatgccgtcagatgttcaatacgtaatatttcatctccggtggtatgtggttcattagggtaaagcgc
ggttaactctcgcccgaccatcatggtgataatatcgtcttcactcattccggcagcatcacgcgtaccaatgtgctgtccgtcgcgaataacgca
aatcgtatcggaaatcgctttgacttcgttgagtttgtgcgaaatataaatacaggcgataccgtgctgttgtagatcgcgaataatatccagtaaa
atcgacgtttcctgctcagttaatgaggctgtcggttcatcgagaattaacaagcgcacctgtttattaagtgccttggcaatttcaaccagttgttgtt
gcccaagccctaaatcgccaacgcgggtatcaggtgaaatggataaactgacctgtgcgagcagcttctgacagcgtagcgtcatcaggtca
taatccataatgccattgtgggttatttcgttacccaggaagatattttccagcacggtcaattctttcaccagggccaattcctgatgaatgatggc
gatacctttgcgttcggtatcgcggatgtgactcgcctgaatctcttctcccgcaaaaataatttcgccttcgtaggagccatggggataaatacca
cacagcactttcatcagcgttgatttaccagacccattttccccacaaagtgagacgatttcgccagcattcaaccgcaagcagacgttatcaat
cgccttcacactgccgaaggttttggtaatgttcttcatttcaagtagataaggcataacgactccacctaagccaattcattcacgcggcatgga
gagaaatcacgcccccgctccgcgctggggcgtaacgcttacagctcgctctctttgtggaatccgtctttaattaccgtatctttgatgttgtttttatt
cacatcgatcggtgtcaggaggcgggaggggacatctttcaggccattattcagtgtggtatctgcttttggttcctgaccattgcccaactcaacg
gcaatttctgcggcagtatttgccaacaacgtaataggtttatacaccgtcatagtttgcgtaccggcagcaatacgtttaatacctgcgagatcc
gcatcctggccggagattgctactttccctgataaaccttgcgcgcttaatgcctgaattgccccacctgcggtggcatcgtttgaggcaactaca
gcatcaattttgttattattggcggttagcgcgttttccataattttcaatgcgttttccggtaaccagccatcaacccattggtcaccaacgactttaatt

tttccggaatcaacgtaaggttttaacactttcatttgtccggcgcggaacagcttggcgttgttatctaccggcgagccgcccatcaggaagtaat
taccttgcggaacaatatcgaccagggcttttgcctgcagttcaccgactttttcgttatcgaaagaaatataaaaatcgatatccgcatcgttaat
catacggtcgtaagctaatactttaatgccttcttgtttggcttcttttacaacgttacttaatacctgaccgttatacggaataatgacaagaacatcg
acaccccggtttatcatgttttcaatctgcgacatttgtgtttcttcattgccatttgcagactgtacaaatactttcgcgccgagagattctgccttttca
caaagatatctcgatctttttgccagcgttcaagacggagatcatcaatcgccatacctattttgacttctttggcgtgtgcagcaacgttggtaagc
aggagtgaggtgcaaagggtgagtagaatgttctttattttcatggtgtagggccttctgtagttagaggacagttttaataagtaacaatcaccgc
gataaacgtaaccaatttttagcaactaaacaggggaaaacaattacagatttttatctttcgattacgatttttggtttatttcttgatttatgaccgag
atcttacttttgttgcgcaattgtacttattgcatttttctcttcgaggaattacccagtttcatcattccattttattttgcgagcgagcgcacacttgtgaat
tatctcaatagcagtgtgaaataacataattgagcaactgaaagggagtgcccaatattacgacatcatccatcacccgcggcattacctgatt
atggagttcaatatgcaagcctattttgaccagctcgatcgcgttcgttatgaaggctcaaaatcctcaaacccgttagcattccgtcactacaatc
ccgacgaactggtgttgggtaagcgtatggaagagcacttgcgttttgccgcctgctactggcacaccttctgctggaacggggcggatatgttt
ggtgtgggggcgtttaatcgtccgtggcagcagcctggtgaggcactggcgttggcgaagcgtaaagcagatgtcgcatttgagtttttccacaa
gttacatgtgccattttattgcttccacgatgtggatgtttcccctgagggcgcgtcgttaaaagagtacatcaataattttgcgcaaatggttgatgtc
ctggcaggcaagcaagaagagagcggcgtgaagctgctgtggggaacggccaactgctttacaaaccctcgctacggcgcgggtgcggc
gacgaacccagatcctgaagtcttcagctgggcggcaacgcaagttgttacagcgatggaagcaacccataaattgggcggtgaaaactat
gtcctgtggggcggtcgtgaaggttacgaaacgctgttaaataccgacttgcgtcaggagcgtgaacaactgggccgctttatgcagatggtgg
ttgagcataaacataaaatcggtttccagggcacgttgcttatcgaaccgaaaccgcaagaaccgaccaaacatcaatatgattacgatgcc
gcgacggtctatggcttcctgaaacagtttggtctggaaaaagagattaaactgaacattgaagctaaccacgcgacgctggcaggtcactctt
tccatcatgaaatagccaccgccattgcgcttggcctgttcggttctgtcgacgccaaccgtggcgatgcgcaactgggctgggacaccgacc
agttcccgaacagtgtggaagagaatgcgctggtgatgtatgaaattctcaaagcaggcggtttcaccaccggtggtctgaacttcgatgccaa
agtacgtcgtcaaagtactgataaatatgatctgttttacggtcatatcggcgcgatggatacgatggcactggcgctgaaaattgcagcgcgca
tgattgaagatggcgagctggataaacgcatcgcgcagcgttattccggctggaatagcgaattgggccagcaaatcctgaaaggccaaatg
tcactggcagatttagccaaatatgctcaggaacatcatttgtctccggtgcatcagagtggtcgccaggaacaactggaaaatctggtaaacc
attatctgttcgacaaataacggctaactgtgcagtccgttggcccggttatcggtagcgataccgggcatttttttaaggaacgatcgatatgtata
tcgggatagatcttggcacctcgggcgtaaaagttattttgctcaacgagcagggtgaggtggttgctgcgcaaacggaaaagctgaccgtttc
gcgcccgcatccactctggtcggaacaagacccggaacagtggtggcaggcaactgatcgcgcaatgaaagctctgggcgatcagcattct
ctgcaggacgttaaagcattgggtattgccggccagatgcacggagcaaccttgctggatgctcagcaacgggtgttacgccctgccattttgtg
gaacgacgggcgctgtgcgcaagagtgcactttgctggaagcgcgagttccgcaatcgcgggtgattaccggcaacctgatgatgcccggat
ttactgcgcctaaattgctatgggttcagcggcatgagccggagatattccgtcaaatcgacaaagtattattaccgaaagattacttgcgtctgc
gtatgacgggggagtttgccagcgatatgtctgacgcagctggcaccatgtggctggatgtcgcaaagcgtgactggagtgacgtcatgctgc
aggcttgcgacttatctcgtgaccagatgcccgcattatacgaaggcagcgaaattactggtgctttgttacctgaagttgcgaaagcgtggggt
atggcgacggtgccagttgtcgcaggcggtggcgacaatgcagctggtgcagttggtgtgggaatggttgatgctaatcaggcaatgttatcgc
tggggacgtcgggggtctattttgctgtcagcgaagggttcttaagcaagccagaaagcgccgtacatagcttttgccatgcgctaccgcaacg
ttggcatttaatgtctgtgatgctgagtgcagcgtcgtgtctggattgggccgcgaaattaaccggcctgagcaatgtcccagctttaatcgctgca
gctcaacaggctgatgaaagtgccgagccagtttggtttctgccttatctttccggcgagcgtacgccacacaataatccccaggcgaagggg
gttttctttggtttgactcatcaacatggccccaatgaactggcgcgagcagtgctggaaggcgtgggttatgcgctggcagatggcatggatgtc
gtgcatgcctgcggtattaaaccgcaaagtgttacgttgattgggggcggggcgcgtagtgagtactggcgtcagatgctggcggatatcagc
ggtcagcagctcgattaccgtacgggggggatgtggggccagcactgggcgcagcaaggctggcgcagatcgcggcgaatccagaga
aatcgctcattgaattgttgccgcaactaccgttagaacagtcgcatctaccagatgcgcagcgttatgccgcttatcagccacgacgagaaac
gttccgtcgcctctatcagcaacttctgccattaatggcgtaaacgttatcccctgcctgaccgggtgggggataattcacatctatatatctcagta
attaattaatatttagtatgaatttattctgaaaatcatttgttaatggcatttttcagttttgtctttcgttggttactcgtaatgtatcgctggtagatatgga
gatcgttatgaaaacctcaaagactgtggcaaaactattatttgttgtcggggcgctggtttatctggttgggctatggatctcatgcccattgttaag
tggaaaaggctattttcttggcgtgttaatgacagcaacttttggcaactatgcatatcttcgcgcagaaaaactcgggcaactggatgatttttta
cccatatctgccagttagttgcgttaatcactatcggtctcttgtttatcggtgttttaaacgcacctatcaatacttatgaaatggtgatctatcccatcg
cctttttgtctgcttgtttggtcaaatgcgtttgtttcgctcggcatgagcaacataaagctcttacatattcaggaatgaaaggaatactgtgatgga
caacaaaatatcaacctattcaccggcctttagtattgtgtcatggatagctctcgttggtggtatcgttacctatctgttagggctatggaatgcaga
gatgcagttaaatgaaaaaggatattattttgccgtactggtattaggactgttttctgcggcgtcttatcaaaagaccgttcgggacaagtatgaa
ggcataccgaccacttccatttattatatgacctgcctgactgtctttattatctctgttgcattactgatggtaggtctgtggaatgcgacattattactc
agcgaaaaaggtttttatggactggctttcttcttaagcttgtttggtgcagtagcggtgcagaaaaatattcgtgatgccggaataaacccacca
aaagaaacacaggttacccaggaagaatacagcgaataactcacgtaagcccggtcagtccaatgtgaccgggcttttacttaactcactaa

tctgtttctgtcgattcgttgtaccagcatagaaagtaacaaactcgctgccaacgtcgcgcaaaagatccaaataatatccagtattggccaatt
tttaagctcaattccccgggtgcgcagcgcatggataatcaaggcgtggaatccgtatatacccaatgaatggcgggagattaagccaagtcc
gcgaatggtacgcgtatccagcgtgtttttaaccagagtcaatagcgcgattgcgcagataaaaaccatcggcccacagtaaagataccagg
tatcggcaaaatttccgcgccactgcaattcatataatgtcccgcgagagataataaaaacccccgtcgcaaacagcgcggcgctcacccac
gacagtgctttatgctgtgtgtccatcatccctatagcgcggcccaacatgccatacagaatgtagtaaaaagtatcgccattgatatataagtta
attggcagccattcaaaaccgtcaattttctgcggcactgtgtttgggttagcgataatgccaatcaccgccattagcaccagcaacatttttccgc
cgacgttcttcacctgaatcagcggtgaaaccagataaatcaccgcaatcgcgaagaaaaaccacaagtggtaaaacactggcttttgcagc
aggtttttcagcgctaactccatattgatggaggtaaacagcgcaatgtagagcagtgcgattgcgctataaaaaatcagacataagccgata
cgcaagaaatggcgcggctgggcgctgcgttcgccaaaaaagagatagccggaaatcatgaaaaatagcggcacgctgacacgagagg
cagaattcagaacattggcgatatcccatgtgacggggctaacactatgagcattggtcacataccaggtagtggtgtgaatcatcaccaccat
taaacacgctatccctcgcaggttatcaatccagtaaattttgggctgcatctgtgtctctgtatctggttaaaaaaagtctgaccgataaatcatttg
gttggcgcactggaataatctgagttttatcactacagcttatagaggcttaaggaaattcgtaagatatcagccactataccgatataaataata
agactcacctgcaaaccagacggtaatttaatgatgatgaacgctttctttccggcaatggcgcttatggtgctagtgggttgttctataccgtcac
ccgtgcagaaagcacaacgggtaaaggttgatcctctgcgttcgttgaatatggaagcgttatgcaaggatcaggcggcaaaacgttataac
accggcgagcaaaaaatcgacgtcaccgccttcgaacagttccagggaagctatgaaatgcgcggttataccttccgtaaagagcagtttgtc
tgttcttttgacgcggatggccatttttgcatctttccatgcgttaagccctgcttttcccgtttcgtactgtatatcttccatccagcgggtatactgatc
ccttcctttaaatccacacgtatccagcacgaaataatatgcaaaagtttgataccaggaccttccagggcttgatcctgaccttacaggattact
gggctcgccagggctgcaccattgttcaaccattggacatggaagtcggcgcgggaacctctcacccaatgacctgtctgcgcgagctgggg
ccagaaccgatggcggctgcttatgttcagccttctcgtcgcccgaccgatggtcgctacggcgaaaccccaaccgtttacagcactactatc
agttccaggtggtcattaagccatcgccggacaatattcaggagctgtacctcggttctctgaaagagctgggcatggacccgactattcacga
catccgtttcgtggaagataactgggaaaacccgacgctgggtgcctggggactgggctgggaagtgtggctgaacggcatggaagtgacg
cagttcacttacttccagcaggttggtggtctggagtgtaaaccggttaccggcgagatcacctacggtctggaacgtctggccatgtacattcag
ggcgtagacagcgtttacgacctggtctggagcgacggcccgctgggtaaaaccacctacggcgacgtgttccatcagaacgaagtggagc
agtccacttacaacttcgaatacgcggatgtggacttcctgttcacctgcttcgagcagtacgagaaagaagcgcagcagctgctggcgctgg
aaaatccgctgccgctgccagcctacgagcgtattctgaaagccgcccacagcttcaacctgctggatgcgcgtaaagccatctccgtcaccg
agcgtcagcgctacattctgcgcattcgcaccctgaccaaagcagtggcagaagcatactacgcttcccgtgaagccctcggcttcccgatgt
gcaacaaagataagtaagaggcggctatgtctgagaaaacttttctggtggaaatcggcactgaagagctgccaccaaaagcactgcgcag
cctggctgagtcctttgctgcgaactttactgcggagctggataacgctggcctcgcacacggcaccgttcaatggtttgctgctccgcgtcgtctg
gcgctgaaagtagctaacctggcggaagcgcaaccggatcgtgaaatcgaaaaacgcggcccggcgattgcccaggcgttcgacgctga
aggcaaaccgagcaaagcggcagaaggttgggcgcgtggttgcggtattaccgttgaccaggctgagcgtctgactaccgataaaggcga
atggctgctgtatcgcgcccatgtgaagggcgaaagcaccgaagcactgctgccgaatatggttgcgacttctctggcgaaactgccgatccc
gaaactgatgcgttggggcgcaagcgacgtgcacttcgtgcgtccggtgcacaccgtgaccctgctgctgggcgacaaagtcattccggcaa
ccattctgggcattcagtccgatcgcgtgattcgcggccaccgctttatgggcgagccggaattcaccatcgataacgccgatcagtatccgga
aattctgcgtgagcgtgggaaagtcatcgccgattacgaagaacgtaaggcgaagattaaagccgatgccgaagaagcagcgcgtaagat
tggcggtaacgctgacttaagcgaaagcctgctggaagaagtggcttcgctggtggagtggccggtcgttctgaccgcaaaattcgaagaga
aattcctcgcggtgccggctgaagcgctggtttacaccatgaaaggtgaccagaaatacttcccggtgtatgcgaacgacggcaaactgctgc
cgaactttatcttcgttgccaacatcgaatcgaaagatccgcagcagattatctccggtaacgagaaagtcgttcgtccgcgtctggcggatgcc
gagttcttcttcaacaccgaccgtaaaaaacgtcttgaagataacctgccgcgcctgcaaaccgtgttgttccagcaacagttggggacgctgc
gcgacaaaactgaccgcatccaggcgctggctggctggattgctgaacagattggcgctgacgttaaccacgctacccgtgcgggtctgctgt
ctaagtgcgacctgatgaccaacatggtcttcgagttcaccgacacccagggcgttatggggatgcactatgcgcgtcacgatggcgaagcg
gaagatgtcgcggtggcgctgaatgagcagtatcagccgcgttttgctggtgatgacctgccgtccaacccagtagcttgtgcgctggcgattgc
tgacaagatggataccctggcgggtatcttcggtatcggtcagcatccgaaaggcgacaaagacccgtttgcgctgcgtcgtgccgcgcttgg
cgtgctgcgaattatcgttgagaagaacctcaaccttgatctgcaaacgctgaccgaagaagcggtgcgtctgtatggcgataagctgactaat
gccaacgtagttgatgatgttatcgactttatgctcggtcgcttccgcgcctggtatcaggacgaaggttataccgttgacaccatccaggcggta
ctggcgcgtcgtccgactcgtccggctgatttcgatgcccgtatgaaagcggtatcgcatttccgtaccctggatgcagctgctgcactggcggc
ggcgaacaaacgtgtatctaacattctggcgaaatctgacgaagtgctgagcgaccgcgtgaatgcctctaccctgaaagagccggaagaa
attaaactggcgatgcaggttgtggtgctacgtgacaagctggagccgtactttacggaaggtcgttaccaggatgcgctggtcgaactggctg
agctgcgtgaaccggttgatgctttcttcgataaagtgatggtcatggttgatgacaaagaattgcgtatcaaccgtctgaccatgctggagaaa
ctgcgcgaactgttcctgcgcgttgcggatatttcgctgttgcaataataacgccgttattaaatagcctgccatctggcaggctttttttatcgctaaa
taatacagcaacctttaataatcttctgctgaataaagattatctcatatattaattttatgagatttttttaggattatatcaaggagaagaaacaaac

ttattaagctagaatagccacgggtgcttgagactgtttgtctcaggtattcaccgaaaggcagacagagaaaagccccacctgactataaatc
aaagtttgtactgaccccaaaaagttggacagttaaacacgaggcatataggtctgattccgatattcaattggagtcagaccttttaatttcagg
ctaattcttctgctgttgtagtattcaatatattccgtaacagcatccttcagttcgcttatattactgaactcatcaagataaaaacactccgactttaa
ggttccaaagaaacactccaccacagcattatccagacaattgccttttctggacatgctttgtttaataccatgttctttaaggatattttgatatcttc
tcatacgatactgccatccctggtcagagtgcagaacaggatgctcgtgaggattaagctttttgaatgcctgatcgagcatattctcaaccatgtt
catcactggtctttccgaaaggctgtaagaaataacttcgttgttgaagagatctattactggagacaaatacagcttgcgcccattgactgcaaa
ttcagtaacatcggtaacccacttctcgtttggccgcgtagccttgaaatctctttggagaacattaggggcggtttgccctacctctcctctgtaag
agcggtatcgcttgaccttaatcgctgctttaagtgagagggttcccatcaggcgctgaacagctttatggttaatctgtttcccttctcgatgaaga
gacagcgttaccctacggtatccgtatcggcctctattctcgtgataaatctcactaatacgctttttaacgtccgcatacttgtcaggcttgctgaga
gcctttagatgataataaaacgtactgcgcggtatctccgcagccctgagaagctcatcaagaggataaaactgccttagctcgttgagtacttt
cactttttcgtgggatgagctaaggctttcagctttttagatacataagccgcgtttcaagaaatcgaacttgcctttcaagatcctcaatgcgtcgg
tcttttgacagctccaatgctgatgccgctttttctggatcaactgatattgcaatgtttcttttggtgccaatcttgagcgcgcgtaaaccagcttctcc
gcgctcttcatagaccttcagccacctggctacagaaccactaccagcaagcataaagtgagcagcagcctgattaagggacatgtgctgct
cgatcacagctttcacgaccttaatacgcaactctggatcagcactaacgcctttaggtttgggaattaaaccttttctccatgtttttcatagaggg
caacccatgtcctgacctgggttcgggggacaccaaaacgtgccgagatgatcctgtaaccatcatcagttgtgaagtagtgattcacgacttc
aaggcgcttttcaaaagggtattttggctttgacatattaggggctattccatttcatcgtccaacaaaatgggtgcagtacatactcgttggaaatc
aacacaggaggctgggaatgccgcagaaatatagattactttctttaatagtgatttgtttcacgcttttatttttcacctggatgataagagattcact
gtgtgaattgcatattaaacaggagagttatgagctggcggcgtttttagcctgcaaattgaaagagtaagagtcttcggcgggaaattattcccg
ccttacttacggcgttgcgcattctcattgcacccaaatttattcttcacaaaaataataatagattttattacgcgatcgattatttatttcctgaaaac
aaataaaaaaatccccgccaaatggcagggatcttagattctgtgcttttaagcagagattacaggctggttacgttaccagctgccgggccttt
agcgccgctttcgatggtgaaggacactttctgaccttcgtccagagatttgtaaccatcgttctggatagcagagaagtgtacgaacacatcttt
agagccatcgtcaggagtgatgaagccgaagcctttgtcagcgttgaaccattttacgataccagtcattttaccggacatagtgtattacctttaa
taattaagtgtgcctttcggcgatatggcgtgctttacagattttgaagcgttaaaggaatgtgcactacgaggggtatcaacgataactcttgaag
ggacttgccttactacactgctttaatggtctgtacgtcaaaccgttgatgtgcattaagccacgcattggcgggtgatgcaacaattatttttcatatt
tatgattaatcggttggaaaacggtcctgtcatcaggaccgtaaacagcaataaagtggataaaagtctattccatcaactgcttacttaatgccg
ggttggcttgaatcaaacgcatcaattttagttcggcacttgaaggcttcacgcgtctggattcccattcctttaccatggcgactgatacgcccaa
aacccgggcgaaatcatcgatttttaatcctgtcccttttcgtaactgctcaatttcggtacaggacgttgttctgtgcgtcagggtaattttctgcgtttc
atctttaaaaacaatctgttccaggctgctcaacagctcatgcattggatctttatattccattgaaaactcctcaaatcacactgcgggatcgtgaa
ttgcatcgaagctcattaagaatagtcgggaaaagcagacacagcggggtgaagtcagggcatgctctggctatgattcgattcagcctgtcat
ttagcgtgacttatagccacctaacgatctgataaatgccgatatatctcttaccactcctgttttgtaaagatttcgaaaatctaaccttctgcgaag
gcgtaaatctctggctacaaaaaaggcaacggcactatcatttttaacaatgaaacaacaaattaaccttgtttcattttctccatgattttaccag
gaagattacgtagaaagggtatcctgcaagcctgtcctggactatccttgtcacgtcagacacgcgtgtgtcgttgtgcgctttttttgggtgaaag
gagtaagaaaatggcgacaggaaagtcctgctctcgctggtttgcgcctcttgcggcgttattaatggtagttagcctgagtgggtgttttgataaa
gaaggcgatcagcgtaaagcgtttatcgacttcctgcagaatacagtgatgcgtagcggtgaacgtctaccaaccctgactgccgatcagaa
aaaacagtttggtccttttgtctctgattacgcgattctgtatggttattctcagcaggtgaatcaggcgatggattccggtctgcgcccggttgtaga
cagcgttaacgcaattcgcgtaccgcaggattacgttacgcaaagcggtccgctgcgtgaaatgaacggttcgctgggtgtgctggcgcaaca
actgcaaaatgcgaagttgcaggccgatgctgcgcactctgcgctaaaacagagcgatgatttgaaaccggtctttgatcaggcattcaccaa
agtggtaacgacgccagctgatgcattgcaaccgttgattccggcggcacaaactttcacgcaacaactggtgatggttggagactacattgct
cagcaaggtactcaggtgagctttgtcgcaaatggcatccagttcccgacttcacagcaggcaagtgaatataacaaactgattgcgccattac
cggcacagcatcaggcgtttaatcaggcctggactacagcagttactgcaacccaataaagagtaaaagcccgagcgattatcgggctttact
ctacgcagtcgcggcttagtccgcgacgtgcggattcacacagttcttctcaacctttccttgtaacgcatcaatcaaattatccacggcacaggc
ggccatgccataacgcgtctcatgggtggcagatccaatatgcggtactgcgacgacgttggccattgagagcaacggcgaatctacggaca
gtggctcttgttcgaagacatccagcccggcagcgtgaatttcgcctttctgcaatgctgcgatcagtgcattttcgtcaaccaccgggccacgtc
cggcattaatgaaaatggcggaggatttcattttggcgaattgttctgcgccaaacagatgatgcgtctcatcagttaacggcaggatcaggca
aacgaaatctgactcttgtaacagagtatccaaatcgcagtagcgggcgttgaagcgttcttctgcttctttatggtggcggcgcgcgttatagag
gatgggcatgttgaagccaaagtgcgcacgttgtgccagcgccatgccgatccgtcccatcccgacaatgcccagtgttttatggtgaacgtca
gtgccgtaccagtccgggcctatgctcgcggtccattcgcctgcttttacccgttctgctacctccacaacccgacgagcggtagacaacacca
gcgccatcagcgtatcggcgacggtttctgttaatacggttggcgtgtgcatcagcagaatttttcgggcggtaagcgcatcgacatcaaaattgt
catagccgacggagatcgttgatgtggcacgcagtttcggcatttttccagcaatgcggcatttacattctcgtttgaacccagtaaaccttcagct
tcggcaaaaattgctgcattttgttcgacggtttgtgggctgaggtttgccacctggtgaacggtgaaatgctcttgcaggcgttgcagtaaatcatc

aggtaaggctttgtagaggataacggacggcttcatgcttctctccattcactgataaggcgatcaccttagcctgaaatatgatgaaggaaaac
tgaagaatgccaggtgatttctgcatcacctggcatgaaagggattacagcgggcttaaggtaatttctacacggcggttttgcgccttaccttctg
cggtgctgttgctggcgattgggttagccgggccaaggccctgagtacggatgcggctggcgtccacgccctgggtgatcaacgcgctggca
acggaatccgcacgttgctgggagagacgcatgttcaggtcgtgaccacccgtgctgtcggtataaccaatcacgttaaccgccgttttcggat
actctttcagtaccattgccacgccggtcagggtgttagcgcccgccggtttcagggtcgcgctgctgctgtcgaaggtcacattgttcggcatatt
gaggataatgttatccccgctgcgggttacgctaacaccagtgccgcgcattttgtcgcgcagcttcgcttcctgcacatccatgtaataaccaac
gccgccgcccagagctgcgcctgctgctgcgccaatcagcgcgcctttaccgcgatctttcttcgaagaagagagcgcaccaatacccgcgc
ccacgagagagcccagacctgcgccgatagcagatttacctgcttcgcgttcgccggtgtaagggttagttgtgcagccagataccgccaga
gcgccactcactacggcggcaataagataaacacgtttcttcattgttaatccttaataaccttttttattctttgccacgggttccgtggcgggagatt
atgccgcgtgaacatgaagattattcctgggaatactcggaaatttgtaagtaatatttaactgctcaatacatctaacctttcaggagtcttcggttt
ggccaactcatcctcacgatattccgttctgactgccgcccattgggggcccatgctggttgaaaccgacggcgaaaccgtgtttagctcgcgtg
gcgcgttagccacaggaatggaaaactccttgcagagcgcggttcgcgaccaggttcacagcaatacgcgggtacgatttccaatggtgcga
aaaggctttcttgcgtcaccggaaaacccgcaaggcattcgtgggcaggatgaatttgttcgcgtgagttgggatgaggcgctggatcttattca
ccaacaacataaacgcattcgtgaggcttatggtccggcatcgatttttgctggttcctacggctggcgttcaaacggcgtgctgcataaggcctc
gacattattacaacgctatatggcgctggcaggcggttataccgggcatctgggggattattcgaccggcgcggcacaggcgatcatgccgta
tgtcgtgggtggtagtgaagtttatcaacagcagaccagttggccgctggtgctggaacatagcgatgtcgtggtgctgtggagtgctaaccac
tcaatacgctgaaaattgcgtggaatgcatccgatgagcaggggctttcttacttttctgcactgcgtgacagcgggaaaaagctgatctgcattg
atccaatgcgatcggaaaccgtcgatttctttggcgataaaatggagtgggtggcaccgcacatgggcaccgatgttgcgctgatgctggggat
cgcccatacgctggtggaaaatggttggcacgacgaagcgtttctggcgcgttgcaccacaggttatgccgtcttcgcctcttatttgctgggcga
gagtgacggaatagcgaaaaccgccgaatgggcagcagagatttgtggtgttggcgcagcgaaaatccgcgagctggcggctattttccac
caaaataccaccatgctgatggcaggctggggaatgcagcgccaacagtttggtgagcaaaaacactggatgatcgtcacgctggcagca
atgttggggcaaatcggcacacccggcggcggttttggtctttcttaccattttgccaatggtggtaaccccacgcggcgttctgcggtgctctcttc
catgcagggcagcttgccgggtggctgcgatgcggtggataaaatccctgttgcccgcattgttgaagcactggaaaaccctggtggcgcata
tcaacacaacggtatgaaccgacatttcccggatattcgttttatctggtgggcgggcggtgccaactttactcatcatcaggataccaatcgcct
gatccgtgcctggcaaaaaccggagctggtggtgatctctgaatgcttctggacggcggcggcaaaacacgcggatatcgttctgcctgcgac
tacctcttttgagcgtaatgatctcaccatgaccggtgattacagtaatcagcatctggtgccgatgaagcaagtggtgccgccacgctatgaag
cgcgtaatgattttgatgtttttgccgagttaagtgaacgctgggagaagggcggttatgcacgttttacggaaggaaaaagtgagctgcaatgg
ctggaaacgttttataacgttgcccgacagcgcggggcaagccagcaggttgaattgccgccatttgctgagttctggcaagccaaccagttaa
ttgagatgccggaaaacccggacagcgagcggtttattcgcttcgctgatttttgccgcgatccgctggcgcatccgttaaaaaccgccagcgg
caagattgaaatcttctcacagcgtattgccgattacggttacccggattgccctgggcatccaatgtggctggagccggacgaatggcagggc
aatgccgaaccagaacagttgcaggtactttctgcccatccggcgcaccgcctgcacagccagctgaattacagttctctgcgcgaattgtacg
cggtggcaaatcgtgagcctgtcaccattcatcctgacgatgcccaggagcgcggcatacaagatggcgatactgttcggttgtggaacgcac
gcgggcaaattcttgccggagcggtcattagcgagggaattaaacctggcgtgatttgcattcacgaaggggcatggccggatctggatttaac
cgctgacggtatttgtaaaaacggcgcagtgaacgtgctgaccaaagatctccccagctcgcggctggggaatggctgtgcgggtaatacgg
cgctggcatggctggaaaaatacaacggtccggaactgacacttacagcgtttgaaccaccggccagctcataatccatgtgggtagttgggt
ttcatcctgccatgcgcaatcgacaatgtgaaaaccctgtgcctggtaaaaatttatcgccggttgatttttttgataaacctccagcatcaggtgg
ggatggcgctgctgcacatactgcatcagcgccttaccaataccgcgcctgacggccttcggtgcgacaaacatcgctgccagaaatcggcc
ttccataatgctgacaaaaccgagaagcttaccgtcttcttcccagacccagttttgcgcgttggcaagataggcatcccgcaccagcggaatg
cagtcacgccagtaattcgcttttataaagggatgcccccaggttgtactttccagccacagttcgaggatcgcggggagttctgaacgttgcgct
tcccgaatcatggtttatttcccggatagcaacagcagccaaccacatgatcattcaccagcccacatgcctgcataaaggagtaacagattgt
ggtgccgacaaacttaaaaccacgtttttttcagtgccttagatagggcgtcggaggcggacgtagatgtgggaatttcgctcaacgttgtggcttg
tgtcacctgtggctgatgatttacaaacgaccagacaaagtcgacaaacggttcgccgttctgttccatttgcaggtacgcccgcgcattaccaa
taattgcctgaattttccctcgatggcggataatcccggcgtcctgtaccagtctttcgacatcctcttcctgcattgctgcgaccttcaccggatcga
actgatgaaagcaggcgcgatagttttcgcgtttttttgaggacggtgatccacgataatccagcctgctgcccttcaaggcagatcatttcgaac
agtttttttactgtcagtttcaggcacgccccactcattatcatggtaggcaatataaagcgggtcctgactcacccagccgcaacgttccatactttc
cctcgctatgtacccgatcgtaaaaaatttcagccgcttttcttgacgcatcggtgaaaaagtcaatatttaatacagggctactatgcccgatatcct
tcatacaatgacaataatattgccgaattcggctcttccctggagtcgtgttaatgatcataaaaaaaaagcggtggtcgctggcagctaagcctg
ctggcgagcgtggtaatcagtgcctttttttctcaacacagcttacgcctggcaacaagaatatatcgttgatacgcaacccggactttccacaga
gcgttacacctgggatagtgatcatcaacctgattacaacgatattttgtcgcaacgtattcaaagtagccaaagggcgctgggactggaagtc
aatctggcggaagaaacccctgtggatgtgaccagcagtatgagtatgggctggaattttcctttatatgaacaggttacaaccggcccggtcg

cggcattacattacgatggcacaaccacctcgatgtataacgagtttggcgacagtactaccacgctgaccgatccgttatggcatgccagcgt
gagtaccttaggctggcgtgttgactcccggcttggcgatctccgaccctgggcgcaaatcagctataaccagcaatttggcgagaatatctgg
aaggcgcaatcaggcctgagccggatgacggcgacaaaccagaacggcaactggctggatgtcaccgtaggcgctgatatgttgctcaatc
aaaatattgccgcctatgccgcgctaactcaggcagaaaataccactaataatagcgactatctgtatacgatgggggttagcgccagatttta
acgtaacagtcacaattgaaaccattaaataacaatagttgtggcgatagtgggtgctaacttaccaaataataaatttggtgaataattgtcgcg
tcattcattcctgaactaaggcatttcattccgttctgatggcatttcatgccgtttttccccaggcataaagtgcacttcgttatggttgtcggcagaga
tttttcctttttattactgcaggaatactgccatgacaccttcaaattatcagcgtacccgctggctgacactcatcggtactatcattacccagtttgc
gctggggtcggtttatacctggagcctgtttaatggcgcgctttccgccaagctggatgcgccggtaagccaggtcgctttctctttcggcttgttaa
gtctggggctggcaatttcgtcttctgttgcgggcaaattacaggaacgttttggcgttaaacgcgtcaccatggcttccggcattttgctgggatta
ggcttcttcctgacagcgcattctgacaacctgatgatgctgtggttaagcgccggtgtgctggtgggactggcagatggcgcgggttatctgctg
acgctctctaactgtgtgaagtggttcccggagcgtaaaggtctgatctccgcgttcgctatcggttcttatggtctgggtagcctgggtttcaaattt
atcgacacgcagctgctggaaacggtcggtctggaaaaaacctttgtgatttggggagcgattgcgctgttgatgattgttttcggcgcaacgtta
atgaaagacgcaccaaaacaggaagtgaaaaccagcaatggtgtggtggagaaagattacacgctggcagagtcgatgcgtaaaccgc
agtactggatgttagcggtaatgttcctgaccgcctgcatgagcggcctgtacgtgattggggtagcgaaagatatcgcccaaagtctggcaca
ccttgatgtggtttccgcagccaatgcagtcactgttatttccatcgccaacctttcaggtcgtctggtgctgggtattctgtctgacaaaatcgcccg
tatccgtgttattaccattggtcaggtgatatcgctggtgggtatggcggccctgctgtttgcaccattgaatgcagtgacgttctttgcagcgattgc
ctgcgtggcatttaactttggcggcactattaccgtctttccgtcactggtcagtgagttctttggcctcaataacctggcgaaaaactacggtgtgat
ttatctcggtttcggtatcggtagcatttgtggttcgattatcgcctcactgtttggcggcttctatgtgaccttctacgtgattttcgccctgctgattctgtc
attggcgctttctacgacgattcgtcagccagagcagaaaatgttgcgtgaggcgcatggctccctttaatgctcagccgcgagctactgggctt
cagcttccgtgtgcatcaaacgcatcaacgaaatgtataaacaatgactcgtaaaatttgggccaattaactggccctttttactgctttttgtaacg
ctaaagtctcttttcaaacttgcatttttgtaaatttgtgcttcatgcacactctttccccacactttttccctttgctgtggtctacttattcgcgcgtgtagat
tttacttatctgactacctccgcactttttccctgccgggcctgaaaagccactaagcagggtgttatcacctgtttgtccagggtttgtttgcatgaga
tacatcaaatcgattacacagcagaagctgagctttttgcttgcaatctatattggcctttttatgaatggcgcggtttttaccgccgcttcggcagct
atgcgcacgattttaccgtctggaaaggcatttctgctgttgttgaactggccgccaccgtactggtgaccttctttttactacgtcttctttcgctgtttg
gccgccgcagctggcgtattctggcatcgctggtggtgctcttttccgcaggtgccagctattacatgaccttccttaatgtggtcattggttatggca
tcatcgcttccgtcatgaccaccgatatcgacctgtcaaaagaagttgttggtctgaactttattctctggttaatcgccgttagtgcattgcctcttatc
cttatctggaataaccgctgtcgctacaccttgctccgacaactgcgaaccccagggcagcgtattcgcagcctggcggtcgtcgtactggcgg
gtattatggtttgggcaccgattcgtttgctggatatccagcagaagaaagtggagagggcgaccggcgttgatttgccgagttatggcggtgtc
gtagcgaactcttatctgccatcaaactggctttctgcgttggggctgtatgcctgggcgcgggtcgatgaatcttccgataataattcattgcttaat
ccggcgaagaaattcacttatcaggcaccgcaaaacgttgatgacacttatgtcgtgtttatcatcggtgaaaccacgcgttgggaccatatgg
gtattttcggctatgagcgtaataccacgccgaaactggcccaggagaaaaatctggcggcgttccgtggttactcctgtgataccgcaaccaa
actctcactgcgttgcatgtttgtacgtcagggggcgcggaagataatccgcagcgcacattaaaagaacagaacattttcgcggttctgaag
cagttaggattcagttctgacctctacgctatgcagagcgaaatgtggttctacagcaacacgatggcggacaacattgcttatcgtgagcagat
tggtgcggagccacgtaatcgtggcaagccggtagatgatatgttgctggtagacgaaatgcagcaatcgctagggcgcaacccggatggta
agcatctgatcattctgcataccaaaggttcgcattttaactacacccagcgttatccgcgtagcttcgcgcagtggaagccggaatgtattggtgt
tgatagcggctgtaccaaagcgcagatgatcaactcctatgacaactcggtgacctatgtggatcactttatctccagcgtgattgatcaggttcg
cgataagaaagcgattgtgttctacgcagctgaccacggtgagtcaattaatgaacgcgagcacctgcacggcacgccgcgtgaactggca
ccgccggagcagttccgcgtaccgatgatggtctggatgtcagataaatatctggaaaatccggccaatgcgcaggcgtttgcgcagctgaa
aaaagaagccgacatgaaagtgccacgccgtcacgtagagctgtacgataccatcatgggttgtcttggctatacttcaccggatggtggaatt
aacgaaaacaacaactggtgtcacatcccgcaggcaaaagaggcagcggctaactaagcggcctgctgactttctcgccgatcaaaaggc
attttgctattaagggattgacgagggcgtatctgcgcagtaagatgcgccccgcattcggtgattggcgcagcctggtagcgcacttcgttcgg
gacgaaggggtcggaggttcgaatcctctatcaccgaccaaattcgaaaagcctgctcaacgagcaggctttttgcatctgcagctcatgagg
atgagaacctccgggggcagggaggttcgactcgagcgaagcgagagaacgttgcgccagcaacggcccgcagggcgagccacgaag
tggcgagtaatcctctatcaccgaccaaattcgaaaagcctgctcaacgagcaggctttttgcatctgcagctcatgaggatgagaacctccg
ggggcagggaggttcgactcgacgtaggcctgataagacgcgtcagcgtcgcatcaggcaaatgtgccggccattgtcggatgcggcgcg
accgccttattcgacctacacgacacacatttccctgccaaatttgcgccccatatctcactatccattaacggatttgtgacagaaaccattgtctt
tttttatatttgcttaaatcttttttttgcgttacttatggctggccctggcattttccgctgtgcgttttagcgttcatggtattaatcgctcagatatccacctttt
cctcagattttttcattaaatgcataaaacttaatcaccaaatgttgctaaatattaagctgattgttctggtcatgttggagtagcacaaatttctctg
ctgcaaatagctgtttgaagttttttaatctttgtgtgtggattctcaccgttggtgtaaatcccggttggctgtattgacgttttcacattctgttgacaga
ttgtaggtcacgaggggcattttatggaggatccgcactgttacactgatgttaattagtacggcatccccacctcataacgttgacccgaccggg

caaaaaacaaaaaaggtcaggcagcgacaacccactgcaaagggttaaaacaacaaacatcacaattggagcagaataatgcgtatttc
cttgaaaaagtcagggatgctgaagcttggtctcagcctggtggctatgaccgtcgcagcaagtgttcaggctaaaactctggtttattgctcaga
aggatctccggaagggtttaacccgcagctgtttacctccggcaccacctatgacgcctcttccgtcccgctttataaccgtctggttgaatttaaa
atcggcaccaccgaagtgatcccgggcctcgctgaaaagtgggaagtcagcgaagacggtaaaacctataccttccatctgcgtaaaggtgt
gaagtggcacgacaataaagaattcaaaccgacgcgtgaactgaacgccgatgatgtggtgttctcgttcgatcgtcagaaaaacgcgcaa
aacccgtaccataaagtttctggcggcagctacgaatacttcgaaggcatgggcttgccagagctgatcagtgaagtgaaaaaggtggacga
caacaccgttcagtttgtgctgactcgcccggaagcgccgttcctcgctgacctggcaatggacttcgcctctattctgtcaaaagaatatgctgat
gcgatgatgaaagccggtacaccggaaaaactggacctcaacccaatcggaaccggtccgttccagttacagcagtatcaaaaagattccc
gtatccgctacaaagcgtttgatggctactggggcaccaaaccgcagatcgatacgctggttttctctattacccctgacgcttccgtgcgttacgc
gaaattgcagaagaatgaatgccaggtgatgccgtacccgaacccggcagatatcgctcgcatgaagcaggataaatccatcaatctgatg
gaaatgccggggctgaacgtcggttatctctcgtataacgtgcagaaaaaaccactcgatgacgtgaaagttcgccaggctctgacctacgcg
gtgaacaaagacgcgatcatcaaagcggtttatcagggcgcgggcgtatcagcgaaaaacctgatcccgccaaccatgtggggctataac
gacgacgttcaggactacacctacgatcctgaaaaagcgaaagccttgctgaaagaagcgggtctggaaaaaggtttctccatcgacctgtg
ggcgatgccggtacaacgtccgtataacccgaacgctcgccgcatggcggagatgattcaggcagactgggcgaaagtcggcgtgcaggc
caaaattgtcacctacgaatggggtgagtacctcaagcgtgcgaaagatggcgagcaccagacggtaatgatgggctggactggcgataac
ggggatccggataacttcttcgccaccctgttcagctgcgccgcctctgaacaaggctccaactactcaaaatggtgctacaaaccgtttgaag
atctgattcaaccggcgcgtgctaccgacgaccacaataaacgcgttgaactgtacaaacaagcgcaggtggtgatgcacgatcaggctcc
ggcactgatcatcgctcactccaccgtgtttgaaccggtacgtaaagaagttaaaggctatgtggttgatccattaggcaaacatcacttcgaaa
acgtctctatcgaataattaaaagccatacaagactgatggcaaaggcaaaaatgcctgatgcgctccgcttatcaggcctacgaaaattctg
caatgtattgaatttgcacgattttgtaggccggataaggcgttaacgccgcatccggcataaacaaagcgcactttgtcaacaatctgtatacc
cggtggcgctgtgcctctgcgtgcagcgccatccggcagcaatacttcattccctgtccgataagggcgggaattgatttgtgagcaatacaga
cacgcagttccaggctgcgggtcactatagagaatccgggttatgttgcagtttattctccgacgtttgggactcgtcatccccacgtttatcggtatt
acccttctcacatttgcctttgtccacatgatcccgggcgatccggtgatgatcatggcgggcgaacgtgggatctccccagagcgtcacgcgc
agctgctggctgaactcggcttagataaaccgatgtggcagcagtatctccattacatttggggcgttatgcatggcgatctaggcatttcaatga
aaagccgcatcccggtttgggaagagttcgtgccgcgcttccaggccacgctggaacttggcgtctgcgcgatgatttttgctacggcagtcggt
attccggtcggcgtgctggctgcggttaaacgcggttccattttcgatcacacagcggttggcctggcgctgacaggttattcaatgcctatcttctg
gtggggcatgatgctgatcatgctggtttcggtgcactggaacctgacgcccgtctccggtcgcgtgagcgatatggtgttcctcgatgactccaa
tccgttaaccggttttatgctaatcgacaccgccatctggggtgaagacggcaactttatcgatgccgtcgcccatatgatcttgcctgccattgtg
ctgggtactattccgctggcggtcattgtgcgtatgacacgctcctcgatgctggaagtgctgggcgaggattacatccgcaccgcgcgcgcca
aagggctaacccgcatgcgggtgattatcgtccatgcgctgcgtaacgcgatgctgccggtggtgaccgttatcggcctgcaggtgggaacatt
gctggcgggggcgattctgaccgaaaccatcttctcgtggcccggtctgggacgctggttgattgacgcactgcaacgccgcgactatccggt
agtgcagggcggcgtattgctggtggcgacgatgattatcctcgtcaacttgctggtcgatctgctgtacggcgtggtgaacccgcgtattcgtca
taagaagtaaggggacatcatgtcacaggttactgaaaataaagtgattagcgcaccggtgccgatgaccccgttacaggagttctggcacta
ttttaaacgcaacaaaggcgcggtcgtcgggctggtttacgtcgtcatcgtgctgttcatcgcgatctttgccaactggattgcaccctataacccg
gcggaacagttccgcgatgcactgctcgccccgccagcctggcaggaaggcggcagcatggcgcacttgctgggcaccgatgacgtaggc
cgtgatgtgctgtcgcgcctgatgtacggtgcgcgcctgtcgctgctggttggctgtctggtagttgtgttatcgctgattatgggcgttattctcggcc
tgatcgccggttactttggcggcctggtcgataacatcattatgcgcgtggtcgatatcatgctggcgctgccaagtctgctgctggcgctggtgct
ggtggcaattttcggcccgtcgattggtaacgccgcgctggcactgaccttcgttgccttgccgcactatgtgcgcttaacccgcgccgccgtgct
ggtggaagttaaccgcgattacgtcaccgcgtctcgcgtggcgggtgccggggcgatgcgtcagatgtttattaacatcttcccgaactgccttg
cgccgctgattgttcaggcgtcgctcggtttctctaacgccattctcgatatggctgctcttggtttcctcggcatgggggcacagccgccaacgcc
tgagtggggcaccatgctctccgacgtgttgcagttcgcgcaaagcgcctggtgggtcgtgaccttcccgggtctggcgatcctgctgacggtg
ctggcatttaacctgatgggtgacggtctgcgtgacgcgctcgatcccaaactgaagcagtaagaggttcgagatggcgttattaaatgtagat
aaattatcggtgcatttcggcgacgaaagcgcgccgttccgcgccgtagaccgcatcagctacagcgtaaaacagggcgaagtggtcggga
ttgtgggtgagtccggctccggtaagtcggtcagttcactggcgattatggggctgattgattatccgggccgcgtaatggcagaaaaactgga
gtttaacggccaggatttgcagcgtatctcagaaaaagagcgccgcaacctggtgggtgccgaagtggcgatgatcttccaggacccgatga
ccagccttaacccgtgctacaccgtgggtttccagattatggaagcgattaaggtgcatcagggcggcaacaaaagtacccgccgtcagcga
gcgattgacctgctgaatcaggtcggtattcccgatccggcatcgcgtctggatgtttacccgcatcagctttccggcggcatgagccagcgcgt
gatgatcgccatggcgattgcctgtcggccaaaactgctgattgccgatgaaccgaccaccgcgctggacgtgaccattcaggcgcaaatca
tcgaactactgctggagctacagcagaaagagaacatggcgctggtgttaattacccatgacctggcgctggtggcggaagcggcacataa
aatcatcgtgatgtatgcaggccaggtggtggaaaccggtgatgcgcacgccatcttccatgcgccgcgtcacccgtatactcaggcattgctg

cgtgcgctgccagaatttgctcaggacaaagaacgtctggcgtcgttgccaggtgtcgttcccggcaagtacgaccgcccgaacggctgcctg
cttaacccgcgctgcccctatgccactgacagatgtcgcgctgaagaaccggcgctgaatatgctcgctgacgggcgtcagtccaaatgccat
tacccacttgatgatgccgggaggccgacactatgagtacgcaagaggccaccctgcaacaaccgctgttgcaggctatcgacctgaaaaa
acattatccggtgaagaaaggcatgttcgcgccggaacgtctggttaaagcgctggatggcgtttcgtttaaccttgaacgtggcaaaacgctg
gcagtagtgggcgaatctggctgcggtaaatcgaccctcggtcggttgctgacgatgattgaaatgcccaccggtggcgagctgtattaccag
gggcaggatctgcttaagcacgatccgcaggcgcagaagctgcgtcggcagaaaatccagatcgtcttccagaacccttacggttcgctgaa
tccgcgtaaaaaagtcgggcaaattcttgaagagccgctgctgatcaacaccagcttaagcaaagaacagcgtcgggaaaaagccctgtc
gatgatggcgaaagtcggcctgaaaaccgagcactatgaccgctatccgcatatgttctccggcggtcagcgtcagcgtatcgccatcgcccg
tggtctgatgctcgacccggatgtggtgattgccgatgaaccggtttccgcgctggatgtttcagtgcgcgcgcaggtgctgaatctgatgatgga
tttgcagcaggagttggggctgtcttatgtctttatctcccacgacctgtcggtggtggagcacattgctgatgaagtgatggtgatgtacctgggcc
gctgcgtggagaagggaacgaaagaccaaatcttcaataacccgcgccatccgtacactcaggcgctactttccgcgacgccgcgcctgaa
cccggacgatcgccgcgagcgcatcaagctcagcggtgaactaccaagcccactgaatccaccgccgggttgcgccttcaacgcccgctgt
cgtcggcgcttcggcccctgcacccagttgcagccgcagctaaaagactacggcggtcaactggtagcttgttttgctgttgatcaggatgaaa
atccgcagcgttaactcacaaaaccggggccatgcgctccggtttactcaatcagcttcaacagtggcgacaagcatagcaaaatgccgtac
agcagaatcagccaggctttaaatccgcgaagtttttccagttgtgcgactttatagatcaggaaaaacggaatgaggcacgacacaatacca
tataacgggcttcccaactgaaagaacaccagcaccgatacacgaaacgaaacccaaatcgtcaacgtaatgacgataaaagcgcagat
cgccagagtcagcacgcgtgagttaattttcttggtatcaataattcggcttaacagattgagaataatgcctttaatggcctcgtggaaaccgag
ataaatgccaaagaatgcggtcagtacggcaaagatattaagcaccgtagaggtgatatgaatgatatgcccagggatcacctgcgcggcc
agcgccagtgctgagatattttgttcaaaggcagaaacggcttcttcgtggctaattgagaaggtaaacgaaaaggcaaaaaacaggatcac
cgcgatgagcgtgatataactaattcggtgggtacgcagcgcgagccgggttgccagtactttatccgcttcccgtttacgataggcaatattcat
tgggttaagtacctgaataaatactgcagaaaagaagcaaaatggaatggtaagcaagacatcgcggaaaaagacggaggcttgcggga
aggcggttatattggcgaaattccagtgcgggatcatcgcaaaaccgaacacgacaataatccctactttgaccaccaccattggcccggaa
atcttaaacagtaatcgttcaccaccagacgcaatcgccaccagtacggcgaaaatagcgactttataaagtagagattgtgaaagatcggc
atcggttaaaccgaaggttttcaggtacgaggcgctgtcgaaaaccacggagagagagtagataaaaatcccgtggataatcatcaaaaag
tagataaccccgaggaaaattccccagttcttccccaggtaatgactgataatatcggtgtagtcattacaggaatcgctttcagaaagggttttta
aataaatgtcctgcactacccaggtggcaggataagcaatgatcgctgcggtaataaatacccaaattcccttcaagccaatttgtactggcatc
agcacggttccggcaccaatcgccatgcctatgcataataaaacccagccgaagtcgtagcgtgtaaacggcaatttctgattgtgtttcgatag
tgtgttgtgctgcataaagtcctgtccagaaagaagtcggcctcgcaaaaatgaataaacccagtttctcttgcgcaacaggtggagaagtcga
atacgcagtatgtaaaaaatgaggcaacagattcactggggaggtcgcgaggaggcgcaattctacaaaattgcgcatttcactaccagtctg
gagtcagcccggatatgaactgcaactattgcaaataaagaaattaagctggaaggcatcgcgcaatgaggaagattgccgggtgctgaca
gtaaaaagcaatcgtagaatcagcggcaagccggaaaggttccggtgaggcgcaatgttgcgggggctttatccctggtggcattggttgctg
gagagagaaaacccccgcacgttgcaggtatgcacctgacaacaccacgggggctaatcttgactctagaccactcaagaatagccgcga
aacgttgtcattacaacacaggcggctatatgacgttcgcagagctgggcatggccttctggcatgatttagcggctccggtcattgctggcattct
tgccagtatgatcgtgaactggctgaacaagcggaagtaacgtgtcatgcgggcgtcaggctgccgtaatggcaatttgcgcccggaccagg
ccgcagggggggaaactctgcggcctttttcgttcttactgcgggtaaggcacccagtcgccgccgttcaggcgaacgtacggtttatcctggtatt
gaataactactgcatttgagttctcggagaccggtgctgtttgtggcaacccactggtgagtttttttccagtcaacattgtcttcggtgaaaatcttgc
catcgagaacgcgaaccaccagatcggagatagccaggaagctgctcggttgttcgatgacaatcggtgccccctgatgcggtgccttcatg
ccgaagaatttcaccccaacggggacgtcggtgatagacgggctagggatatcacgtaggccagatacctgcattctgtcgcccttcagcgc
gccgccgtgttccggcaccacgaccaccatcactttacgacccgatttctcaagttcagtaaagaaggcgtccagttcatcaaagaatttctgcg
cccgcgctttgtaatccgctgttttgctgacccccggataatggttgccgtcatgcagtggaagcgtgttgtagaacgtggcactacggctgtttttat
ctttttcggtaacgtccagccagcggttaagcacagcggtatcgtcataaaccggcgaaccatcaaagcccagcaaaataaccggcagattt
gtttgatccatcaattcgctctgcatgccgccattttcgcgaacttctttcaaaaaaccgccgaactggccgttatgtcccatcatcaggtgctgggt
aaagcccagtttcgaaaggttatcaaacagatagcagtcgttatttgccggttgatacagattagtgtgcgaagtctgcccgcagctggcgcgc
agtaaacggatcgccgccgggccactgtaggaggtggcggagttaaagttcttgaactcaatatcgaaatgcgaccacagtggatgcgacat
caacccggcggcttctatatccgaccaggaaagcgaacagatgttaatcaccagtagttcaaatggctgagcatcagcgggcagcgaagac
gggaaggtcgatttacgtttcgcctccgcgttatagaaattattcagccaggcgttaaggttcgccgttgttggcggtgcagtttgtgcgggcatatc
acccactaccggtgcgccacccgtcgccgcaacggttgccgctgcgttaccacccgtcgttgttacagtggtcgtcggttgtccggctggccac
aaggagaaacttggtcccgccagggtaagtacgttcagccatagcagtatggcaaccacaaaaacggtaatgcgaatccattgtgacagga
ataaccaggccactaataaaacaaaaatggccccaatcatctgccagttaataaagcgtgtgacaaggtcgattaaataatcggtactgaac
cccgccacctgcgaaccctggctcattatgctttccgggccaggcaaccaggtgtcatgccagaacaaagcaaagccgatcggcagggca

atccagtggcgcaagcgatgcaggctgtagcgcggaaggggcatcagcagaaacgcggcaaacaccaaattgaggagcggatggaagt
taagatatcccgcccacaacaggccgaacttaaccagaaaatagaagttccagccggaaaggccgcgccagtattgccagagggaagaa
ggcatggcggtattttgcgtaaattgagtcatttttggttgccctggctttttccgtgcggcgtaacgtcccggccggtttaacataacgaggtttagc
aaagaacaaacgtaaggtgtcgcgaatgcgtcgcaaagagtgccgcgccagatagcccagcgggaaaaatatcagtgcgcaaacgaca
ataatttcaatgatatcgctgatggtcatcatgatgagcgctccacagcatcatctaacagtcgcatgggttctggtattcgtcgccagtggcgacc
atcgtgctcggcattgatgaccggtttagaactttgcgttaaaggcagcggcatgccccattgttctggggcaagcaagcgcatctgcaccagct
cggcactgatttgatcatcttcaaaccagaccatacggtttgagaaaatgtcgccagtaggcaatgggaaaatatgattcaacgcggtatccag
atcgttaatccgacagaatgagagaaacagcaccagccgattaccgccaatggtcatgatatcgccggtacggttagggcgacacagcgtc
agggcttgttcaacgcggatacccggtaccggacgtagggcaaccagaacgcctttaccgtgggcaggtaatagagggttattcatcatgttgt
tgacggcattacagaacacatcccacttctggaaaccacgcagtttgagcggctgggtcattgacagcaaggtagtgatatcttccggcacata
gcgactaaacttctgcccttgcacgctttcgatcatcgtcagacaacgggagagtggcgcattccacggaataaccatatttgcaccgcaggcc
aataacaaacgttcatcggtggcgcgcaggctagcggtattttcccgcacgaggattttcatcgcactaccgcgctggcgacgcagggtatga
atgctgcgggccagtggctcgatttgcgcattttgctgtaaagaaaagaccaccgtcgccgcctgagcggtacgggcttcattgaacaggactt
cgttattgttgaacagttgccagtgttccgatagcggcggcgcaccttccagtacagcaacattactcagaatgcgtttttcgtcgctgcgtggttgg
atctccgcctcttcgctttgaactaatgtccagataccattttgttgctgaacgctaagctgctgacgggcgctgacccctttttcgttgcaccagaag
gcaatatccagcaaatgttggtcaccctgaaaacgcaaactggcaagaccaaaaagtgaacggtattcctcaagcaacaatgaaaattgttt
atcgttattatttccgggattaattaccaaaagcgaacaatggtttaacctgctccatttattcattttatccaaccatgagcgaagccgctcggcag
gaatgttttgccatgcgttatttgcgcaaacaagaataaaaaggtaattatggggatcaatagaacactgcaaatcacgggtcaaatagtatag
accttttcatgatttagcattgaaaataattttatttttccggcccttgagaatcatctaattgaaagattttcgccggatcgctgtccatgctaatgac
cgcgacgtgtgcggtttcagcctgggatgcaattgtttgattcgccagactgatagcatcttcatggcgatcgacgttaaaccaccagacgccgc
ctgctggcatatgtcgcagctcatcccataatgatgagataccgatagagaatacagggtccacaatgtccctcatgtcaccattttgtctgattat
cagtttactagcgaaagctaagaaataaacctaccattgaaattaaaacttatcagatttagcatgtgaatcaatttagaaggtcacaagacgtg
tgattttagtcttttcgtttttaactttttctgttgcgtaacggaaagtcaaaaagtgagcaaattcccgtctcgccgtaaacaataaaccggcgaaat
gctcacggttagaattgtctcatgaacggtacggttatttcatagggatcaagcaaaatgaataacaatgaaccagatactctgcctgatcccgc
gataggctatatcttccagaatgatattgtggcgttaaagcaggcattttcactgcctgatattgattatgccgatatttcccaacgcgaacagttgg
ccgcggcattaaaacgctggccgttgctggcagagtttgcgcaacaaaagtaggggattggtgaatggccgtactgggatagcagggggtg
cggggaggcgtggggacaacaaccatcaccgccgcattagcctggtcattacaaatgttgggagaaaatgtcctggtggtcgatgcctgccc
ggacaacttgttgcgcctgtcatttaatgttgattttacccaccgtcagggctgggccagagcgatgctggatggccaggactggcgtgacgctg
ggttgcgctacacctcgcagctcgatttgctgccttttggtcagttatccattgaagaacaagaaaatccacagcactggcaaacccggctgag
cgatatttgctccggcttacagcaactaaaagccagcgggcgttaccagtggattttaatcgacttaccgcgtgatgcctcgcagataacccac
cagctgctgagtttgtgcgatcactcgctggcaatcgtcaatgtggatgccaactgccatatccgactgcatcagcaagcgctgccggatggcg
cacatattttgattaatgacttccgtattggcagtcaggttcaggacgatatttaccagctttggttgcaaagccagcgccgattactgccgatgctc
attcatcgtgatgaagcgatggctgaatgcctggcggctaagcaaccagtaggtgaatatcgcagtgatgcgctggcggctgaagagatact
gacgctggcgaactggtgcctgttgaactactccgggctgaaaacgccagtcgggagtgcatcatgagtatcctgacccggtggttgcttatcc
cgccggtcaacgcgcggcttatcgggcgttatcgcgattatcgtcgtcacggtgcgtcggctttcagcgcgacgctcggctgtttctggatgatcct
ggcctggatttttattccgctggagcacccgcgctggcagcgtattcgcgcagaacataaaaacctgtatccgcatatcaacgcctcgcgtccg
cgtccgctggacccggtccgttatctcattcaaacatgctggttattgatcggtgcatcgcgcaaagaaacgccgaaaccgcgcaggcgggc
attttcaggtctgcaaaatattcgtggacgttaccatcaatggatgaacgagctgcctgagcgcgttagccataaaacacagcatctggatgag
aaaaaagagctcggtcatttgagtgccggggcgcggcggttgatcctcggtatcatcgtcaccttctcgctgattctggcgttaatctgcgttactc
agccgtttaacccgctggcgcagtttatcttcctgatgctgctgtgggggggtagcgctgatcgtacggcggatgccggggcgcttctcggcgcta
atgttgattgtgctgtcgctgaccgtttcttgccgttatatctggtggcgttacacctctacgctgaactgggacgatccggtcagcctggtgtgcggg
cttattctgctcttcgctgaaacgtacgcgtggattgtgctggtgctcggctacttccaggtagtatggccgctgaatcgtcagccggtgccattgcc
gaaagatatgtcgctgtggccgtcggtggatatctttgtcccgacttacaacgaagatctcaacgtggtgaaaaataccatttacgcctcgctgg
gtatcgactggccgaaagataagctgaatatctggatccttgatgacggcggcagggaagagtttcgccagtttgcgcaaaacgtgggggtg
aaatatatcgcccgcaccactcatgaacatgcgaaagcaggcaacatcaacaatgcgctgaaatatgccaaaggcgagttcgtgtcgattttc
gactgcgaccacgtaccaacgcgatcgttcttgcaaatgaccatgggctggttcctgaaagaaaaacagctggcgatgatgcagacgccgc
accacttcttctcaccggacccgtttgaacgcaacctggggcgtttccgtaaaacgccgaacgaaggcacgctgttctatggtctggtgcagga
tggcaacgatatgtgggacgccactttcttctgcggttcctgtgcggtgattcgtcgtaagccgctggatgaaattggcggcattgctgtcgaaac
cgtgactgaagatgcgcatacttctctgcggttgcaccgtcgtggctatacctccgcgtatatgcgtattccgcaggcggcggggctggcgacc
gaaagtctgtcggcgcatatcggtcagcgtattcgctgggcgcgcgggatggtacaaatcttccgtctcgataacccgctcaccggtaaaggg

ctgaagtttgctcagcggctatgttacgtcaacgccatgttccacttcttgtcgggcattccacggctgatcttcctgactgcgccgctggcgttcctg
ctgcttcatgcctacatcatctatgcgccagcgttgatgatcgccctattcgtgctgccgcatatgatccatgccagcctgaccaactccaagatcc
agggcaaatatcgccactctttctggagtgaaatctacgaaacggtgctggcgtggtatatcgcaccaccgacgctggtggcgctgattaaccc
gcacaaaggcaaatttaacgtcaccgccaaaggtggactggtggaagaagagtacgtcgactgggtgatctcgcggccctacatcttccttgt
cctgctcaacctggtgggcgttgcggtaggcatctggcgctacttctatggcccgccaaccgagatgctcaccgtggtcgtcagtatggtgtggg
tgttctacaacctgattgttcttggcggcgcagttgcggtatcggtagaaagcaaacaggtacgccgatcgcaccgcgtggagatgacgatgc
ccgcggcaattgcccgcgaagatggtcacctcttctcgtgtaccgttcaggatttctccgacggtggtttggggatcaagatcaacggtcaggcg
cagattctggaagggcagaaagtgaatctgttgcttaaacgcggtcagcaggaatacgtcttcccgacccaggtggcgcgcgtgatgggtaat
gaagttgggctgaaattaatgccgctcaccacccagcaacatatcgattttgtgcagtgtacgtttgcccgtgcggatacatgggcgctctggca
ggacagctacccggaagataagccgctggaaagtctgctggatattctgaagctcggcttccgtggctaccgccatctggcggagtttgcgcct
tcttcggtgaagggcatattccgtgtgctgacttctctggtttcctgggttgtatcgtttattccgcgccgcccggagcggagcgaaacggcacaac
catcggatcaggctttggctcaacaatgatgataacgcgatgaaaagaaaactattctggatttgtgcagtggctatggggatgagtgcgttccc
ctctttcatgacgcaggcgacgccagcaacgcaaccactgatcaatgctgagccagctgtagccgcccagacggaacaaaatccgcaggt
ggggcaagtgatgccgggcgtgcagggcgctgatgcgccagtcgtggcgcagaacggtccttcgcgtgatgtgaagctgacctttgcgcaa
attgcaccgccgccgggcagcatggtgctacgtggcattaacccgaacggcagcattgagtttggtatgcgcagcgatgaagtggtgacgaa
ggcgatgctcaacctcgaatacaccccatcgccatcgttactgcctgtccagtcgcagttaaaggtttatctcaatgatgaactgatgggcgtgct
gccagtgaccaaagaacagttgggtaaaaaaacgctggcgcaaatgcccattaacccactgtttattagcgacttcaaccgtgtacggctgg
agtttgtcggccattatcaggacgtgtgcgaaaaaccggccagcaccacgctttggctggatgttgggcggagcagtggactggatctgacct
atcagaccctgaatgtgaagaatgacctgtcacacttcccggtgccattctttgacccgagcgataaccgcaccaacaccttgccgatggtcttt
gcgggtgcgccggatgttgggctgcaacaagcctctgccattgtcgcctcgtggtttggttcgcgttctggctggcgtgggcagaacttcccggta
ctctataaccaactgccggatcgcaatgccattgtctttgcaaccaacgacaaacggccggacttcctgcgcgatcatccggcggtaaaagcc
ccggtgattgagatgattaaccatccgcagaatccttacgtcaaactgctggtggtgtttggtcgtgacgacaaagacctgttgcaggcagcga
aaggtatcgctcagggtaacattctgttccgtggtgaaagcgtggtagtgaatgaagtgaaaccgctgctaccgcgtaagccgtacgatgcgc
cgaactgggtacgtaccgatcgtccggtcacatttggcgaactgaaaacctatgaagaacagttacaatccagcggtcttgagccagcagcg
attaacgtttcgctaaacctgccgccggatctctacctgatgcgcagtaccggcattgatatggatattaattaccgctacaccatgccgccggtg
aaagacagttcgcggatggatatcagcctgaataaccagttcctgcaatccttcaacctgagcagcaaacaggaggcgaaccgcctgctgct
gcggattccggtattacaaggtttgctggatggcaaaacagatgtctctattccggcgctgaaactgggcgcgaccaaccagctgcgcttcgac
tttgagtatatgaacccgatgccgggcggttcggtggataactgtattaccttccagccggtgcagaatcatgtggtgattggtgacgactccacc
atcgacttctcgaagtattaccacttcatcccgatgccggatctacgcgcctttgctaacgcgggcttcccattcagccggatggcggatctgtcg
caaaccatcaccgtgatgccgaaagcgcctaacgaagcacagatggaaacgttgctgaatactgttggttttatcggcgcacagacgggcttc
ccggcgattaatctgacggtgaccgatgatggcagcaccattcagggcaaagatgccgacatcatgatcatcggtggtatcccggacaaact
gaaagacgataagcagatcgacctattggtgcaggcgaccgaaagctgggtgaaaacaccgatgcgccagaccccgttccccggcattgt
gccggacgagagcgatcgcgcggcagaaacccggtcaacgctgacctcttccggtgcgatggcggcggtgattggcttccagtcgccgtat
aacgaccagcgcagcgtgattgcgctgttggcagatagcccacgcggttatgaaatgcttaacgatgcggtgaacgatagcggcaaacgcg
ccaccatgttcggttcggtcgcggtgatccgcgagtccggtatcaacagcctacgtgttggcgacgtttattacgtaggtcatctgccgtggttcga
gcgcgtgtggtatgcgctggcaaaccatccgattctgctggcggtgctggcggctatcagtgtgatattgctggcatgggtactgtggcgtctgct
gcgaattattagtcgtcgtcgtcttaacccggataacgagtaattgaagatgaatgtgttgcgtagtggaatcgtgacgatgctgctgctggctgc
ctttagtgttcaggcagcctgtacctggcctgcctgggagcagtttaaaaaggattacatcagtcaggaagggcgcgtcatcgaccccagcga
cgcgcgcaaaatcaccacctccgaagggcaaagttacggcatgttctctgccctggcggctaacgaccgtgcagctttcgataatattctcgac
tggacgcagaacaatctcgctcagggttctttaaaagaacgtttgcccgcctggctgtggggcaagaaagagaacagtaagtgggaagtgct
ggacagcaattcggcctccgatggtgatgtctggatggcctggtcgttgctggaggcggggcgtttgtggaaagagcagcgttataccgacatc
ggcagcgcgttgctaaaacgtatcgcgcgggaggaagtggtgacggtgcctgggctgggttccatgttgttaccgggcaaagtgggttttgctg
aggataacagctggcgttttaaccccagctacctgccgccgacgctggcgcagtatttcacccgctttggcgcgccgtggaccacgctgcgcg
aaaccaatcaacgtttattgctggaaaccgccccgaaaggtttttcgccagactgggtgcgctatgagaaagacaaaggctggcagctaaaa
gccgaaaaaacattgatcagcagctacgacgctatccgcgtttacatgtgggtaggcatgatgcctgacagcgatccgcaaaaagcgcgga
tgctcaaccggtttaaaccgatggcgacattcactgagaaaaacggttatccgccggaaaaagtggatgtggctacggggaaagcgcaggg
taaaggaccagtcggtttttctgccgccatgctgccctttttacaaaaccgcgatgcgcaggccgttcagcgccagcgcgtggccgataactttc
ccggcagcgatgcctattacaactatgtgctgaccctgtttggacaaggctgggatcaacaccgtttccgcttctcgacaaaaggtgagttattac
ctgactggggccaggaatgcgcaaattcacactaaacatattcacgctttccctcggtctggccgtcatgccgatggtcgaggcagcaccaac
cgctcagcaacagttgctggagcaagttcggttaggcgaagcgacccatcgtgaagatctggtgcaacagtcgttatatcggctggaacttatt

gatccgaataacccggacgtcgttgccgcccgtttccgttctttgttacgtcagggcgatattgatggcgcgcaaaaacagctcgatcggctgtc
gcagttagcgccgagttcaaatgcgtataaatcgtcgcggactacgatgctactttccacgccggatggtcgtcaggcactgcaacaggcacg
attgcaggcgacgaccggtcatgcagaagaagctgtggcgagttacaacaaactgttcaacggtgcgccgccggaaggtgacattgctgtc
gagtactggagtacggtggcgaaaattccggctcgccgtggcgaagcgattaatcagttaaaacgcatcaatgcggatgcaccgggcaata
cgggcctgcaaaacaatctggcgctattgctgtttagtagcgatcgccgtgacgaaggttttgccgtcctggaacagatggcaaaatcgaacg
ccgggcgcgaaggggcctctaaaatctggtacgggcagattaaagacatgcccgtcagtgatgccagtgtgtcggcgctgaaaaaatatctc
tcgatctttagtgatggcgatagcgtggcggctgcgcaatcgcaactggcagaacagcaaaaacagctggccgatcctgctttccgcgctcgt
gcgcaaggtttagcggcggtggactctggtatggcgggtaaagccattcccgaactacaacaggcggtgcgggcgaacccgaaagacagt
gaagctctgggggcgctgggccaggcgtattctcagaaaggcgatcgcgccaatgcagtggcgaatctggaaaaagccctcgcactggac
ccgcacagcagcaacaacgacaaatggaacagtctgctgaaagtaaaccgctactggctggcgatccagcagggcgatgctgcgctgaa
agccaataatcctgaccgggcagaacgcctgttccagcaggcgcgtaatgtcgataacaccgacagttatgcagtgctggggctgggcgatg
tggcgatggcgcgaaaagattatcccgccgccgaacgttattatcagcagaccttgcgtatggacagcggcaacactaacgccgtgcgcgg
gctggcaaatatttaccgccagcaatcgccagaaaaagctgaagcgtttatcgcctcgctctctgccagtcagcggcgtagcattgatgatatc
gaacgcagcctgcaaaacgaccgtctggcacagcaggcagaggcactggaaaaccagggcaaatgggcgcaggcggcagcacttcag
cggcaacgactggcgctggaccccggcagcgtatggattacttaccgactttcgcaggatctctggcaggccggacaacgcagccaggccg
atacgttaatgcgcaatctggcgcagcagaagtcgaacgacccggagcaggtttacgcttacgggctgtacctctctggtcatgaccaggaca
gagcggcgctggcgcatatcaatagcctgccgcgtgcgcagtggaacagcaatattcaggagctggttaatcgactgcaaagcgatcaggt
gctggaaaccgctaaccgcctgcgagaaagcggcaaagaggcagaagcggaagcgatgctgcgccagcaaccaccttccacgcgtatt
gacctcacgctggctgactgggcgcaacaacgacgtgattacaccgccgcccgcgctgcatatcagaatgtcctgacgcgggagccagcta
acgccgacgccattcttggtctgacggaagtggatattgctgccggtgacaaagcggcggcacgtagccagctggcgaaactgcccgctac
cgataacgcctcgctgaacacacagcggcgcgtggcgctggcacaggcgcagcttggcgataccgcagcagcgcagcggacgtttaata
agttgatcccgcaggcaaaatctcagccaccgtcgatggaaagcgcgatggtgctgcgtgatggtgcgaagtttgaagcgcaggcgggcga
tccaacgcaggcgctggaaacctacaaagacgccatggtcgcatccggtgtgactacgacgcgtccgcaggataacgacacctttacccga
ctgacccgtaacgacgagaaagatgactggctgaaacgtggcgtgcgcagcgatgcggcggacctctatcgccagcaggatcttaacgtca
cccttgagcacgattactggggttcgagcggcaccggtggttactccgatctgaaagcgcacactaccatgttgcaggtggatgcgccgtattct
gacgggcggatgttctttcgcagtgatttcgtcaatatgaacgtcggcagtttctccactaatgccgatggcaaatgggatgacaactggggcac
ctgtacattacaggactgtagcggcaaccgcagccagtcggattccggtgccagcgtggcggtcggctggcgaaatgacgtctggagctggg
atatcggtaccacgccgatgggcttcaacgtggtggatgtggtcggcggcatcagttacagcgatgatatcgggccgctgggttacaccgttaa
cgcccaccgtcggcccatctccagttctttgctggcctttggtgggcaaaaagactccccgagcaataccgggaaaaaatggggtggcgtac
gtgccgacggtgtggggctaagtctgagctacgataaaggtgaagcaaacggcgtctgggcatcgcttagtggcgaccagttaaccggtaaa
aatgtcgaagataactggcgcgtgcgctggatgacgggctattactataaggtcattaaccagaacaatcgccgcgtcacaatcggcctgaa
caacatgatctggcattacgacaaagatctgagtggctactcactcggtcagggcggttactacagtccgcaggaatacctgtcgtttgccatac
cggtgatgtggcgggagcgcacggaaaactggtcgtgggagctgggtgcgtctggctcgtggtcgcattcacgcaccaaaaccatgccgcgt
tatccgctgatgaatctgatcccgaccgactggcaggaagaagctgcgcggcaatccaacgatggcggcagcagtcagggcttcggctaca
cggcgcgggcattacttgaacgacgtgttacttccaactggtttgttggcacggcaattgatatccagcaggcgaaagattacgcacccagcca
tttcctgctctacgtacgttattccgccgccggatggcagggtgacatggatttaccgccgcagccgctgataccttacgccgactggtaagttttc
agatagcgcctctcttaatgccgctgcgatcgggtatactcgggcggcaatctgggatttccgggggagacaatttgcgcgtaagtcgctcgtt
aacaatcaagcagatggcaatggtggcagccgttgtcctggtgttcgtttttattttttgcaccgttttgctgttccatctggtccagcagaatcgctat
aacacggctacgcaactggaaagcattgctcgctctgtccgcgaacccttatcttcagctattttgaaaggcgatattcccgaagcggaagctat
tcttgccagcattaaaccggcaggcgtggtcagccgtgccgatgtagtgctgcctaaccagttccaggcgctgcgtaaaagttttattccagagc
gcccggtgccggtaatggttactcgcctgtttgagctaccggttcaaatctcgctgggcgtttactcgctcgaacgtccggcaaacccgcagcca
attgcctatctggtactacaggcggattccttccgtatgtataagttcgtgatgagcaccctctcaacgttagtgaccatttacttacttttgtcgcttatc
ctgaccgtcgccatcagctggtgcattaaccgcctgattttgcatccgttacgcaatattgctcgcgaacttaacgccatcccagccaaggagctt
gttggtcaccaactggcattaccgcgtctgcatcaggacgatgaaatcggtatgttggtgcgcagttacaacctcaaccagcaattgctgcagc
gccattatgaagaacagaacgaaaatgcgatgcgcttcccggtgtcggatttgccgaacaaagccttgctgatggagatgctggagcaggttg
tcgcgcgtaaacaaaccaccgcgctgatgatcatcacctgtgaaaccctgcgtgatactgcgggcgtgctgaaagaggcgcaacgagaaat
tctgctgctgacgctggtggaaaaactcaaatcggtactgtcgccacgtatgatcctcgcgcagattagcggttatgactttgctgtcattgccaac
ggtgtacaggaaccgtggcacgcaatcaccttaggtcagcaagtgctcactatcatgagcgagcgcctgccgattgaacgtattcaactccgt
ccgcactgtagcattggcgtggcgatgttctacggcgatctcaccgccgaacagctttacagtcgcgctatttctgcggcatttaccgctcgccat
aaaggcaagaatcagattcagttctttgatccgcagcagatggaagccgcccagaagcggttgacggaagagagcgatatccttaatgcact

ggaaaatcatcagtttgctatttggttacagccacaggtcgagatgaccagcggtaaactggtcagtgcggaagtgttactgcgtatccagcaa
ccggatggcagttgggacctgccggatggcttaatcgatcgcattgagtgctgtgggctgatggttaccgtcggtcactgggtgctggaagagtc
ctgtcgattgcttgcagcctggcaagagcgcggcattatgctgcccttgtcggtaaacctctctgcgctgcaactgatgcacccgaatatggtgg
cggatatgctggaactgttaacccgctatcgcattcagccgggaacactgattctggaagtgacagaaagccgacgtattgacgaccctcatg
ctgcggtggcaatcctccgtccgctgcgcaatgccggagttcgggtggcgctggatgatttcggcatgggctacgcagggctgcgtcagctgc
agcatatgaaatcgttgccaatcgacgtactgaaaatcgacaaaatgtttgttgaaggcttgccgggagatagcagcatgattgctgcaattatc
atgctggcgcagagcctgaacttacaaatgattgccgaaggcgtggagactgaagcacaacgcgactggctggcaaaagcgggcgttggt
attgcccagggcttcctttttgctcgcccactccctattgaaatcttcgaagagagttacctggaagaaaagtagctaccccaaactgattacaaa
actttaaaaagtgctggtttgtgcgagccagctcaaactttttaacctttttgtttcaattatgatccaggtacatttctgtgatgttgtctgggtgttattta
aggccgcaggtaccccataaccttacaagacctgtggttttactaaaggacaccctatgaaaacctctctgtttaaaagcctttactttcaggtcct
gacagcgatagccattggtattctccttggccatttctatcctgaaataggcgagcaaatgaaaccgcttggcgacggcttcgttaagctcattaa
gatgatcatcgctcctgtcatcttttgtaccgtcgtaacgggcattgcgggcatggaaagcatgaaggcggtcggtcgtaccggcgcagtcgca
ctgctttactttgaaattgtcagtaccatcgcgctgattattggtcttatcatcgttaacgtcgtgcagcctggtgccggaatgaacgtcgatccggca
acgcttgatgcgaaagcggtagcggtttacgccgatcaggcgaaagaccagggcattgtcgccttcattatggatgtcatcccggcgagcgtc
attggcgcatttgccagcggtaacattctgcaggtgctgctgtttgccgtactgtttggttttgcgctccaccgtctgggcagcaaaggccaactgat
ttttaacgtcatcgaaagtttctcgcaggtcatcttcggcatcatcaatatgatcatgcgtctggcacctattggtgcgttcggggcaatggcgtttac
catcggtaaatacggcgtcggcacactggtgcaactggggcagctgattatctgtttctacattacctgtatcctgtttgtggtgctggtattgggttc
aatcgctaaagcgactggtttcagtatcttcaaatttatccgctacatccgtgaagaactgctgattgtactggggacttcatcttccgagtcggcgc
tgccgcgtatgctcgacaagatggagaaactcggctgccgtaaatcggtggtggggctggtcatcccgacaggctactcgtttaaccttgatgg
cacatcgatatacctgacaatggcggcggtgtttatcgcccaggccactaacagtcagatggatatcgtccaccaaatcacgctgttaatcgtgt
tgctgctttcttctaaggggcggcaggggtaacgggtagtggctttatcgtgctggcggcgacgctctctgcggtgggccatttgccggtagcg
ggtctggcgctgatcctcggtatcgaccgctttatgtcagaagctcgtgcgctgactaacctggtcggtaacggcgtagcgaccattgtcgttgct
aagtgggtgaaagaactggaccacaaaaaactggacgatgtgctgaataatcgtgcgccggatggcaaaacgcacgaattatcctcttaatc
tcatcacttatgcccgcactcccttccccgagtgcgggtgtttgcgcataattgacccctgtgcctgaacttaccacctgggtgagttcaggtttattt
cacttcttccgtgacattttcatgttcttgcggtctaacacgaagtgtttttaacgtcatttagactgaccgacaacgggcagtttttttattaccaggat
agttgatcaggggttcacatgcagggcacaaaaattcgacttttagcgggcggtttgctgatgatggccactgctggctatgtgcaggcagatgc
gctccagcctgatccagcatggcaacaggggacgctttccaacggtttacagtggcaagtgctgaccaccccccagcgtcccagcgatcgtg
ttgaaattcgcctgctggttaataccggttcgctcgccgaaagtacacaacagagcggttacagtcacgccatccctcgtattgcgctaacgca
aagcggtggccttgacgcagcacaggcgcgttcattgtggcagcaggggatcgaccctaaacgcccgatgccgccggtaattgtctcttatga
caccacgctgtttaatctgagtttgcccaataaccgtaacgatttgctgaaagaagcgctctcttatctggcaaatgccactggcaaattgaccat
cacaccagaaaccatcaaccacgcgctgcaaagtcaggacatggtggcaacctggcctgccgatactaaagagggctggtggcgctatcg
tctgaaagggtcaaccttgttaggtcacgatcctgccgatccgctgaaacaacccgttgaagcggaaaaaattaaagatttctatcagaaatgg
tacaccccggatgcaatgacgctactggtggtgggaaacgtggatgcgcgctcggttgtcgaccaaataaacaaaacgtttggcgaactgaa
aggcaaacgtgaaacgccagctccggtgccgacgctttctccgctgcgtgcggaagcggtgagtattatgactgacgcggtgcgtcaggacc
ggttatctatcatgtgggatacgccgtggcagccgattcgtgaatcagccgcactgctgcgctactggcgtgcggacctggcccgcgaggcgc
tgttctggcatgttcagcaagcgttaagtgccagtaacagcaaagacatcggtcttggatttgactgccgtgtgctgtatctgcgtgcgcagtgtgc
catcaacatcgaatcaccaaacgacaagctgaacagcaaccttaatctggtggcgcgtgaactggcgaaggttcgcgataaaggtctgccg
gaagaagagttcaatgcgttagtggcgcaaaagaaactggagctgcagaaactgtttgccgcctatgcacgagctgataccgatattctgatg
ggtcagcggatgcgttcgttgcaaaatcaggttgtcgatatcgcgccggagcaatatcagaaactgcggcaggatttccttaatagcctgacgg
tagagatgttaaatcaggatctgcgtcaacagttgtcgaatgatatggcgttaatactgctgcagccgaaaggcgagccggaatttaacatgaa
agcgttgcaggcggtctgggatcaaatcatggccccatctaccgccgctgcgaccacctctgtcgccacggatgacgtacatcctgaagtgac
ggatattccacctgcacagtaatgcctgatggtgatgagttagcacaaccatcaggtatttatggatgagctggatagaggggtaacactttatc
cagccttttgcatatgctgcgtttacgctggcatcgcctcacgcgggataatcgcgccgcgatactgaataacggtacttgcggtcaggtgccca
cgtttcgccgcgtcttccgcgctgccgcctgtcagacgtaccgccagataaccggcactgaaagagtcgccagctgcggtggtatcgatcactt
tttctttcggcagtttcaccgccggaacatccactaacccttcgccagcaatggacaccaggcaagaatccgccccgcgtttcaccaccacttct
ttcacgcccgcgttatgggtgcgcgcaatgacgtcttccaccggctgttgaccccacagcgcgtcttcgtcgtccagcgtcaggaaggcgatat
ccgtgcattccagcatttgttggtacacctgctgtgtctcttctttgctggcccacaggcgcggacgatagttattgtcgaaaatcacttttccgccgtt
ggcgcggcattcgcgcagcagggaaagcagcttttcgcggctggtcgggcttaagatcgccaggctaatcccgctcaggtagagataatcga
aattcgccagctcttcgcaaatcgccgcagactgctcactctccagccagaatttggcggcggcttcgttccgccagtagtagaacgtacgctc
gccggtgctgtcggtttcaatgtagtaaaggcccggcagacggttttccatccgttgggtcagggaagtatcaacgttctcgccgtgccaggcgt

ccagcatctgctggctaaaactgtccgttcccagcgccgttacgtaatgaacggttaatgccgcaggatcgacctgacgggcgatatagacgg
aagtgttcagggtatcgccgccgaaaccgcgcttaacgtccgcgcctttctcggaaagctcaatcatgcattcgccaatcacggcaatctttttgg
acatagtcgtgaacctgatctgtaaaaaattagcgttagtttgcgctgtggtggtttgctggtcaaccatattaaaacagtgttccattattttttgagc
cagaacgtattcctgaaagattccgttgtggagtgaggaaatttaccccggccaggcggtaaagttctgcccttacgcgccgataatctttgtcg
agtccgggcagcatcacttttaaacacaggacatctttgatgataaggcaggttatccagcgaataagcaaccctgaagcaagcatcgagag
cttgcaggaacggcgtttttggttgcagtgtgagcgtgcttacacctggcagccgatctatcaaacatgcgggcggttaatggccgtggagctatt
aacggtggtcacgcatcccttgaacccttcgcaacgcctgccgccggatcgctattttactgaaatcaccgtcagccatcggatggaggttgtga
aagagcagattgatttgctggcgcaaaaagccgacttctttatagagcacggcctgctggcatcggtcaatattgatggccctacgctcatcgcc
ctgcgtcagcaaccaaaaatcctgcgccagattgagcgtcttccctggctgcgtttcgaactggtggagcatatccgtctgccgaaagattcaa
cctttgcctcgatgtgtgaatttggcccgctgtggctggatgattttggtaccgggatggcaaatttctctgcgctaagtgaagtgcgttatgactaca
tcaaaatcgcgcgagaactgtttgtgatgctgcgtcagtcgccggaaggacgcacactcttttctcagcttttacatctaatgaatcgctattgtcgc
ggggtgattgtcgagggcgtagaaacgccggaagagtggcgtgatgttcagaactcgcccgcattcgccgcacaaggctggtttctttcacgc
ccggcaccgatagaaacgctgaatacggcggttctggcgctataagctgcctcattttccgcctggctggactatctttaggactggcacagga
agaatatgaggaagcgaacgatgagcaaggcaggcaaaataaccgctgcgatttcaggggctttcttgttgttgattgtcgtggcgatcattttg
attgcaacttttgactggaatcgactcaaaccgactatcaaccagaaagtctctgcggagttgaatcgtccgttcgctatccgtggcgatctgggc
gtggtgtgggagcggcaaaaacaagaaactggctggcgcagctgggtgccgtggccccatgtacacgcggaagacatcattcttggcaatc
caccggatattcccgaagtcacgatggtgcatttgccacgcgtagaggcaacgctggccccgctggcgctgctgaccaaaacggtctggctg
ccgtggatcaagctcgaaaagcccgacgcgcgcctgattcgcctctctgaaaagaacaataactggacgtttaatcttgccaacgatgataac
aaagacgcgaatgcaaagccgtcggcatggtcgtttcggctggataatattcttttcgatcaagggcggatcgccattgatgacaaagtaagca
aagcggatctggagatttttgttgatcccttaggcaagccgctgccgttcagcgaagttactggatcgaaaggtaaagcggataaagaaaagg
tgggcgattacgtttttggcctgaaggcgcagggacgatataacggtgaaccgctcacgggtacgggaaaaataggcggtatgctggcgctg
cgtggcgaagggacgccgtttccggtacaggctgatttccgctctggtaacacccgtgttgcttttgatggcgtcgtgaatgacccaatgaagat
gggcggtgtcgatttacggcttaaattttctggcgattcactgggtgatctctatgaactgacgggcgttctgctgcccgataccccgccgtttgaaa
cggatggtcggctggtagcgaaaatcgacactgaaaaatcgtcggtctttgattatcgcggttttaatgggcgaattggtgatagcgatatccac
ggttctctggtctacaccaccggaaagccacgaccaaaactggaaggtgatgtcgagtcgcggcaattgcggctggcggacctgggaccgtt
gattggcgttgattccgggaaaggggcagaaaagtcgaaacggtctgaacagaagaagggcgaaaaaagcgttcagcctgcgggcaaa
gtgctgccttatgaccgcttcgaaaccgataaatgggacgttatggatgccgatgttcgcttcaaagggcggcgcattgagcatggcagtagcc
tgccgattagcgatctttctactcatatcatcctcaaaaatgctgacctgcgcctgcaaccgctgaaatttggcatggcgggcggcagcattgcg
gcgaatattcatctggaaggcgataaaaagccgatgcaggggcgggcagatattcaggctcgtcgactgaaactgaaagaactgatgcccg
atgtggaactgatgcagaagacgctgggggaaatgaacggtgacgcggaactacgcggtagcggtaactcggtggcggcacttttaggca
acagtaacggcaacctgaaactgttgatgaatgacgggctggtgagccgcaacctgatggagattgttggcctgaatgtcggcaactacattg
tcggtgcgatatttggtgatgatgaggtgcgggtgaactgcgcggcggcgaatctgaatattgccaacggcgtggcgcgcccgcagatttttgct
ttcgatactgagaacgcgttgattaatgttaccggcacggcaagttttgcttcggaacagctggatttgactattgatccggagagtaaaggaatt
cggattatcacactgcgttcgccgctgtatgtgcgggggacgtttaaaaatccgcaggctggggtgaaagccggaccgctgattgcccgtggt
gctgttgctgcggcactggcaacgctggtaacaccggcggcggcgttactggcactgatctcaccttccgaaggggaggctaatcagtgtcgg
acgattttgtcgcagatgaagaagtgatgataattgccggatgtctgacatccggcaattaaacagatttagattgttggcaaagtgtgctttgttca
tgccggatgcggcgtgaacgccttatccggcctacaaatccttgcgaattcaatatattgtatggaaattcaggcctgataagcgtagcgcatca
ggcttttcactcttattacaacgactgatgtcgcgtctcatgggtcagcagcagggcgattaacgtcaagccagccatcgccgccagatataac
cccaccgcacctagcccgtagttagtctgcaaccaggctgcgatatatggcgcaacggaagccccgagaatcgacgctacgttgtaagaga
acgatgctccggtgtaacgcacttctgtcggaaacagctctggtaacagcgcacccattggcccgaaggtcagacccatcagacttaacccc
agcagcaggaaggcaaaaaccagaatcgggttgccagaaccgagcagtgggttaaaggcgaacagcgcgaacaggatgatcagcgtg
gtgatgattaccatgcttttacggcgaccaaaggcatcagccagtaatccagcgactggcaccatcacgccaaaaccaataactgccatcatc
aacatccacaacacttcgttacgcggcaggccaagcccaactggcgcggcggcggtactaaaggtcatagagtagaccgtcatgatgtaaa
acagcgtataggttgccagcataatgaacgtacccagtacggttacgcgaacatgtttggtcagcagcgtacccagcgggatcttcacctgttttt
tcgctttagcgactttctcaaacaccggcgactcatgcagcgacacgcgaacatacaggccgataatgaccagcaccgccgagaagataa
atggcacacgccagccccagctcataaactgctcgtcggtcagtagccaggaaagcagcaagaaagtgccattggcaaagaagaagcca
atcggtgcgcccagctgcggaaaggagccatacagtgcacgtttgcgcggtggggcgttttcagtcgccagcagcgccgcgccgccccattc
accgcctaagcccagaccctgaccaaatcgagccagcgccagcagcagcggggcgaaaataccaatcgtggcatagcccggcagcag
accaatcaccacggtcgaaatccccatcgttagcaacgaggcgaccagcgtcgctttacgcccaacgcgatcgccaaaatgaccaaaaac
ggcagagccaatggggcgcgcgacgaaggcgatggcgaaggtggcgagcgactgtagcgttgctgccgtaggatcgccctgcgggaag

aagatatgcggaaacacaataacggccgcagtggcgtaaatgtaaaagtcgaagaactcaatggctgtgccaatgagagaggcgacaag
gactttattacgcgagttgatcggcgtgtattcttgctcgtggtcgagtgttgtggctgttgcttgcataatcttttcttattttttggctaacgaatagccat
attacgcacagcaaaagtgatatttcaatgtgtaacaaatcgcggaaatggtcaaaaatcaggcaaaaaggggataatgtttaggccagtga
tttttcttctatgaaatgcttcacataattttataaatagtggataaaactggtttcaggttaaataaaagttaccaactggatttatatgctggggttatg
agttggaatgaaattgtttgctcccaaatagatggctgaagaggctctggggacgagtttcaagaggttatgtgcgcattatagccggatgcgat
gctggcgcatcttatccggcctacggggagccaggccgaataaaatcgtatgccggataaggcgtttacgccgcatccggcctattttaaggct
gcgttttccccggcattcgcgggtcgtctttatattcggcggtggcaatccacgccgcgcaaaacagcgtcaaacgggcgaagaagtagaaa
aatgccatcagccccagcacggaaccaaatgctgcgccagaggggggatttcatcaacgatggcagggtgtaggtcatcacgattttaatcact
tcaaaaccaatagcagccagaaatgttccgcgaatcagggctttttttgcgcggacggtggcgcggcaaccgccagaagatccagaagaaa
agcagatagttggcgaagatggaaatcgccaggccaatcaatcgccacgtcggtttcagccactcaatgctgttcaggtgcagggcgctaat
aatcatttgctgcgccgaaccggcaaccgaggtgatcgaaagcgtcacaatcagcgcaatcaacaaaccaatcagcgaaataaaatctcg
cagatattttacccagaacttctcctgatcttgcggcgagcgttcccagacatcgcgcgactgggcacgaatcgcttcacgcaggttacccatcc
agttgatgccggaataaagtgccaccgccaggccgacaagccctacagtcgtacgctgctgaacggcggtgttgatggtgtttttcaacgtggc
ggctagcgtcggatcgctgatgttttgcagaattttgtcgaagatatcctgtagcaacatcggatgggaggccagcacaaagccccctgcggc
aaacgacaccatcaaaattggtatcatcgacaaaaatgagaaataggtgatagccgcaccaaactggttacccagccgatcgttaaagcgtt
ctgtcgcgcgaatcaggtgcgcaataaccggttggcgttggactttttcggcagtggtggtgacggtttccagcgcctggctaactttgctgcctttc
tcgctattatccaacggcttaatcggctcgtgctccagatcctggatgggacgtttgatctcgttttcctgcgtcatccgctcttttgtccttctgtcgtggt
cattcttaaaaagtatagtcggtcagtccaggtactctttcattacgccagccagccactgcataaacaggtttacacgccgggaaagctcccg
acgctgcggataaaccagcgaaagggagagcggctcggcacggtagccaggtaatacttcaataagccgcccggcacgcagggcttcgc
gcacggcaatgcgcgggatctgaataatccccagaccggcaagaccggcggtgagatagttttcgctactgtttaccgtcaacatgccgcca
gacttaaaccactggacgccattaacgctggcaacctcaaaacctaacggatgtacacccaggtgcggtgtataacgcactattgcgtgtgaa
gtcagatcatcggggctttgcggataaccaaagcgcgtcagatagtgcggactggcacagttgaccatggtcagtttgccgaggggacgggc
gataacgccatcttccggtaacgcgccagtgcgtatcacgcaatcaaaaccatcatgaagaatatctaccggacggtcatggctactcagttc
cagctcaattcccggatgcagatagagaaattccgacaggcgcggcagtaacaggcttttcgcgattcctggcgggatgtcgatgcgtaattta
ccgctgatactggtggcatcctgttgaaacagaccgtccagttcgctgagattactcaacacatcttttgctcgttgataataggtcatgccttctgg
cgtgagtttgacccgtcgcgtggtgcgctggagaagctgggtgccaagttgatgttccagtgcctgtatctggcgcgaaacacttccctttggcaa
agcaaagaaatccgctgcgcgggaaaaactttccagctccgcgactttgatgaacaactgcattgcgtgaattttatccatttctttgaccgattgt
tgttcacagcgaaacagtaacgcgcatatcgcattgtttattgtttttctaacatctaatgccgggtttcacaatgttgtcatgccgaatgtggcgattt
ctcagcaggaaaagggcggaatagctttctgagaaaggtgaatccccttaaacagagcgtatttttcagcgattccctttaaagtgaacagag
gacgtgttggctatttgtgcttatttttgtctccttgccgtttgttattttggcttgtggtcgcggaatttcatttttaattgcatgcgtactaaccaccaatata
aggaaatcctcaaaattgttttaaatagtgccctcgacatgtaatttcaggcgtttagctgtaactatttatatcggtaactattttctacacgtcatctct
gtagaatggccaccgccaaatggcgaattcttatttttctcgcgggtaatacccttccactacttaagtctttttaacgtattcaggtggagattcagt
tatgcaaatagtcatgtttgacaggcagtcaatatttattcatggaatgaaaatcagtttacagcagcgtattccaggagtgagtattcaggggc
cagtcaggcagacgagttatggcaaaagctggaaagttaccctgaagccttagttatgctcgatggcgatcaggatggtgagttttgctactggt
tgctgcaaaaaaccgtggtgcaatttcctgaggttaaggtgttaattacggcgacggattgcaataaacggtggttacaggaagttatccatttta
atgtgctggccattgtgcctcgtgattcaaccgtcgagacatttgcgttggcggtaaatagtgcggcgatggggatgatgtttctgcctggagactg
gcggacgacgccggaaaaggacattaaagatctcaaatcgttaagcgcccgccagcgggagattttaaccatgttagcggcgggcgaatc
aaataaggagatcggcagagcgctgaatatcagtaccggaacggtaaaagcgcatctggaatccctgtatcgccgtctggaagtgaaaaat
cgcacccaggcggcaatgatgttaaatatctcctcctgaagaggataattttacagccagcaggaagttagcggctggctgtaaaaatattatg
gttcgccgtacaaaccaattaaacggcgtaccacaccgttagtccagccaaacccatcctgcaacggatactcgccaccgccgccttcgcgg
ggaacaccatcggcaatatggtatttttcgatcagtttgtgctgttccagatagaactgattcaccgtcttcagccagcttcgcgcgatttcatcacc
cagaaggtcatcgccgtacattttaaatccctgaatcgccatccattgtaacggtgcccagccgttgggtttatcccactgttcaccggtttcgtact
cgcttgccagaatcccgccaggtgtcagtaaccgactgcgcacggcgtttgccagacgatcggcctgttcatggttcgccataccgacataga
gtggcacaatggcggcagcggaaaacagcgccagttgttcgcgtcgccagtcgtaatcgcggtagatgccgttttcatcatcccagaggtaac
ggtttaccgcatcgcgacgggcactggctttctggcggaacagtgcttctgtctctttctcgcctttcagcgccgagatgttggcgatggcgctctcc
agtttaaacaggaaggcattcagatcgatggggatgaactgggtggtacgaatgctcgccagacgaccagtatcacgcagccaacgggaa
gagtaatcccaaccggaggccgcccccgcgcgtaaatcgcggtacacctcgttgggcgggcgaccagaatgtttcgcggtttcaacgtcctc
aagccaggattcgtcacgcggcgtgtcgcgatcgtcccagtaacggttgagcagcgatccgtccggcatccgcacaacatggcgataggcct
gattagggattaacgattctgcaccgtccatccagaaggcatattccattttaaggtggtcgagatagcggcgcgcaccgcgtacaccatcttctt
caaacaactccaccatcagcgcaaaaaccggtggttgcgagcggctcaaataataggtgcggttgccgttggggatgtgaccgtagttttcga

tcatccaggcgaagttatcggccatgcatttcagcaaatcttcccgaccactttccgccagccccagcatggtgaaataggaatcccaatagta
ggtttcgctaaaacggccgcccgggacaatatatgactgcggcagcgccagcagagaagaccacggaatgtgatcctgtggttcgcgggtt
agcaccggccacagctggtcgatatgctctttcagggaattttgcgggtccgatacatactcgctggagtagacctccggcagccagaagtggt
tttcaacaaacttgcgcaagtcaaaatcacgatgacggcgcactttacggtagcggattaagatatccagcgggtccattttaggtgcgcagtc
gggaaaggttttactgtcggggaaaattttcgccgactgaacatgctcgaacagttcgagatagcgatcggcaggcgtcagcgcatcagagg
caggcagcccttcaatcatctcgggttccggttctgcctcgatcatctcatccagttttaattcatacgggtccagctcatagcagagatcgacttcg
atcatcagttcgtctggattagggttttgaattttctgattgagcataaggatccaacctccgaaattcgtcgctaattatcggcttttctttaagggtag
tcattcgctccgcctggtgggcgacaaagcgtgcggtggatcacgtttattaacctgtcaaaaaatataacgcactaatatcaatggaatttataa
aaataccgatttggtgttgtttttcattttcagaacattcaatcctgctctcgccgcaatcatgatatgccattcctgatctgggtaataggcacaggct
atcttattgatagtttatattcatgtaattgattgattcctatcaaaaagcgtatctccctgtttttatgatggctttgtctcgcagagacgtttgcagcatt
ctggaaaattttctgggtcacaataactcaccaccgtgagggaagaataatgaaaatggtctcacgtattaccgcgatcggcctggctggcgtc
gcgatttgctatttagggttatctggttatgtgtggtaccacgataataaacgcagtaaacaggccgatgttcaggcatctgctgtcagtgaaaata
ataaggttttaggctttctccgcgaaaaaggatgcgactattgccacacgccttcggcagaattacccgcctattattatattcctggcgcgaaac
agttgatggattacgacattaagcttggatataaatcttttaaccttgaggccgtgcgtgcggcactgctggctgataaacccgtttcgcaaagcg
atttgaataagattgaatgggtgatgcagtatgaaactatgccaccaacgcgttataccgcgctacactgggcgggtaaggtgagtgatgaag
agcgggcggaaatactggcctggattgcaaaacagcgcgcggaatattacgccagcaatgatactgctccggaacatcgcaatgaaccgg
tgcagcccatcccgcaaaaactgcctaccgatgcgcaaaaagtggcgttgggttttgcgctgtatcacgatccccgtttatcggctgatagcacc
atttcatgcgctcattgccatgcgttgaatgcgggggcgtcgatggcagaaaaacatcgattggtgttggtggcgcagttgggccgattaacgc
gccgacggtatttaactcagtatttaacgttgagcagttctgggatggtcgtgcggcaacattgcaggatcaggctggtggaccgccgttgaacc
cgattgaaatggcgtcgaaatcctgggacgaaattattgctaagctggaaaaagatccgcagcttaaaacgcagttcctcgaagtctatccgc
aaggtttcagtggcgaaaatattactgatgccattgctgaatttgagaaaacattaattacgccggattccccatttgataaatggttgcgcggag
atgaaaatgcgctgacggcgcaacagaaaaaaggctatcaattatttaaagataataaatgtgcaacttgtcatggtggtattattctcggcgga
cgttcctttgaaccgttggggctgaaaaaagactttaactttggggaaattacggcggcggatattggtcgtatgaatgtgactaaagaagagcg
tgataaattgcgtcagaaagtacccggtttacgtaacgttgctttaacggcaccgtacttccatcgcggtgacgtgccgacgctggacggggcg
gtgaaactgatgctgcgctatcaggtaggcaaagagctgccgcaggaggatgtggatgatatcgtagctttcctgcacagtctgaacggggtg
tacacgccgtatatgcaggataaacaataattaatttgatcgcccgaacagcaatgtttgggcgatttttattacgataataaagtctgtttttaatatt
atcatgttaaatgtttatattataaaaagtcgtttttctgcttaggattttgttatttaaattaagcctgtaatgccttgcttccattgcggataaatcctacttt
tttattgccttcaaataaatttaaggagttcgaaatggaccagaagctgttaacggatttccgctcagaactactcgattcacgttttggcgcaaag
gccatttctactatcgcggagtcaaaacgatttccgctgcacgaaatgcgcgatgatgtcgcatttcagattatcaatgatgaattatatcttgatgg
caacgctcgtcagaacctggccactttctgccagacctgggacgacgaaaacgtccataaattgatggatttgtcgatcaataaaaactggatc
gacaaagaagaatatccgcaatccgcagccatcgacctgcgttgcgtaaatatggttgccgatctgtggcatgcgcctgcgccgaaaaatggt
caggccgttggcaccaacaccattggttcttccgaggcctgtatgctcggcgggatggcgatgaaatggcgttggcgcaagcgtatggaagct
gcaggcaaaccaacggataaaccaaacctggtgtgcggtccggtacaaatctgctggcataaattcgcccgctactgggatgtggagctgcg
tgagatccctatgcgccccggtcagttgtttatggacccgaaacgcatgattgaagcctgtgacgaaaacaccatcggcgtggtgccgactttc
ggcgtgacctacaccggtaactatgagttcccacaaccgctgcacgatgcgctggataaattccaggccgacaccggtatcgacatcgacat
gcacatcgacgctgccagcggtggcttcctggcaccgttcgtcgccccggatatcgtctgggacttccgcctgccgcgtgtgaaatcgatcagt
gcttcaggccataaattcggtctggctccgctgggctgcggctgggttatctggcgtgacgaagaagcgctgccgcaggaactggtgttcaacg
ttgactacctgggtggtcaaattggtactttgccatcaacttctcccgcccggcgggtcaggtaattgcacagtactatgaattcctgcgcctcggt
cgtgaaggctataccaaagtacagaacgcctcttaccaggttgccgcttatctggcggatgaaatcgccaaactggggccgtatgagttcatct
gtacgggtcgcccggacgaaggcatcccggcggtttgcttcaaactgaaagatggtgaagatccgggatacaccctgtacgacctctctgaa
cgtctgcgtctgcgcggctggcaggttccggccttcactctcggcggtgaagccaccgacatcgtggtgatgcgcattatgtgtcgtcgcggcttc
gaaatggactttgctgaactgttgctggaagactacaaagcctccctgaaatatctcagcgatcacccgaaactgcagggtattgcccagcag
aacagctttaaacacacctgataacataacgttgtaaaaaccgaatgcccagcctttaaaaaaacagctgggcattcggttgcttattaatcgc
aataatatattggctgtctattcatcgtgttgataagatatatgttatatgaatgtttattaaatgtagcgattacctttaatgtcacgaaaagggattattt
gcttactattaatttccctgtgttcttaaacacgtttttgtattataaatccagtcatcctgcccgtagaacatcacacattatcatcctgttctcccgcttc
gtttaaatttatttatcaatcaatttgacttaagagggcggcgtgctacattaataaacagtaatatgtttatgtaatattaagtcaactatgcaatcac
tacatgggaattgtctaattgcgtatgcaagacataaatatattctcaccatggttaatggtgaatatcgctattttaatggcggtgacctggtttttgc
ggatgcaagccaaattcgagtagataagtgtgttgaaaattttgtattcgtgtcaagggacacgctttcattatttctcccgatgctcaaggaggag
gcattaaatcttcatgcacataaaaaagtttcttcattactcgttcatcactgtagtagagatattcctgtttttcaggaagttgcgcaactatcgcaga
ataagaatcttcgctatgcagaaatgctacgtaaaagagcattaatctttgcgttgttatctgtttttcttgaggatgagcactttataccgctgcttctg

aacgttttacaaccgaacatgcgaacacgagtttgtacggttatcaataataatatcgcccatgagtggacactagcccgaatcgccagcgag
ctgttgatgagtccaagtctgttaaagaaaaaattgcgcgaagaagagacatcatattcacagttgcttactgagtgtagaatgcaacgtgctttg
caacttattgttatacatggtttttcaattaagcgagttgcagtatcctgtggatatcacagcgtgtcgtatttcatttacgtctttcgaaattattatggga
tgacgcccacagagtatcaggagcgatcggcgcagagattgtcgaaccgtgactcggcggcaagtattgttgcgcaagggaatttttacggc
actgaccgttctgcggaaggaataagattatagagttttactcagacataaaaaaaacccggcataggggaccgggaagaggatagtctgc
cgtctccagactaataaaccgttataacactccctgttggcacgggaaactttgtgctctcagtaagttaaatataactttactggaaataagatc
agccattttttataaacataagctatacgctgtgcgaaaatatattcgtgctgcatttacttattatcaattaactgttatgcaaaactactttgtggata
aattttggtcctaccaaatctggcagtttttgcgctaagaaacagtctggcatcatttcattagtatactgaaattgaaataatcgcagtatgaaatat
aagggataatcatgactcatgtctgctcggtgatcctcattcgtcgttcattcgatatttatcatgaacagcaaaaaatatcgctgcataacgagag
tattctgctgctggagaaaaaattggcagacgattttgcgttttgttcaccggatacgcgacgactggatatcgatgagctgacagtttgccattactt
acaaaatattcgtcagctaccacgcaatttagggttacacagcaaagaccgtttgttaattaaccagtcaccccccatgccgctggtgacggcg
atttttgatagcttcaatgaatccggggtaaattcaccgatactgagcaatatgctctacctttcctgtttatcgatgttttctcataagaaagaactgat
ccccttactttcaatagcatcagcactgtttcaggaaaagttgaacgccttattagctttgatatcgccaaacgttggtatctgcgcgatatcgcgg
aaagaatgtataccagcgagagtctaatcaaaaaaaagttgcaggatgaaaatacctgtttcagtaaaatattactcgcctccaggatgtcgat
ggccagacgattactcgagttacgtcaaattcctctgcatactattgcggaaaaatgtggctatagcagtacatcgtactttataaacacatttcga
caatattatggtgtaacgccacatcagtttgcgcaacattcgccaggtaccttttcctgacatatttcgcatttgaatattggtcaggatctcacacct
gcttcatgtgaaactcttccctgatgatttctgccgggctaccggctagttctctttcgcagtttgccaggcggtatcggtattttcaggaggaaaag
actcggcatgtttgggattattaagctgacaattcatacgattacgggaatgtgggtcagtattgtgttgttcaaattgatgacgaatggctggagtg
gtttctactttcagtgctgtgtactaagcctggtattcctgacggttagctggttgttgagtggtgaatggttagcaggaaagagtaaggctgaacctt
catgttcaaccttactctcatttacacgttacgctttttaaagcgggcaaagagatgttccactacaacgaaaaagaccggaacgaagtaaatt
gccagcactgttgcggcaaacatcccgcccatcacgccggtacctaccgcgttttgcgcgccagaaccggcaccatgactgataaccagcg
gcagcacgccgagaataaaggccagagaggtcatcaggattgggcgtaaacgcatccgcgccgcttcgatgattgcctctatcggcgttttcc
cttctttctgcatcatctcaacggcaaattcgacaatcaggatggcgtttttggcggaaagcccgatggtggtcagcaaaccaacctggaagta
gacgtcattacttaagccgcgcagatcggtggccagtaatgcgccaacgacgcctaacggaacaaccaacatcaccgagaacggaattga
ccagctctcatagagtgcggcgagggcgaggaacaccacgaccagtgaaatcgcatacagcgcaggagcctgatttgaggataacgcttc
ctgatacgatagtccggtccatgagtagccgacgcctgccggaagtttagcgaccaggtctgccataaatttcatggcgtcaccggtacttttcc
cggccgccgcttcacctaaaatctccattgacgggatgccgttgtagcgttccagtcgcggtgaaccataggtccattcggtagacgagtaggc
agaaagcggtgccatcgtgccagaggcgttgcgtacataccattggttgatgttatccggcaacatacggaacggcgtgcctgcctggacata
cactttttcacccgcccctggttgaggaagtcgttcacgtagctgctgccgaaggcggtggaaattgtctggttgatatcagacagcgccacgc
ccatcgcttcagctttcgcagcgttgacgttcactttgaacatcggcgtatcttccaggccgttcgggcgtaccccggtgacctgattcggtgattgc
gctgccagtgataacagctcgtttcgcgcctgggttagtttttcgtgccccaggttaccgttgtccagcagttccatatcaaaacctgacgcggtac
ccagttcagccaccgcgggtaagttgaacgggaagacgacggctttattgatactgcttaacgcaatcattgcccgctgaatgatcgcggtaac
cgagttttcctcaccgacacgttcagaccacggcttgagactgataaacgccaggccgttgttttgcccctgaccgctgaagccaaagccgcc
aacggtaaacaccgactggacattatctttctctttagtcagataataatccgtcacctgttgcagcactttcgtggtgttaaccatggtggcaccgg
aaggtaactgcgcggtggtcataaatacccccctgatcctcttctggtaagaaagaggtcggcgtgcgcaggaacagcaccgccatcccggc
gcaaatcagcaggtagaccaccatgtagcgaccggtacaacgcaacagcgagcgggtgctatcggtatagtgttgagttgatttttcaaacag
cgtgttgaagcgtgcgaacagggcgttaggtttgtgaccgccttccggcgcggctttcagaatggtggcgcacagggcaggggtcaggctcat
tgccacaaatactgaaagcagcatggaggagatcagcgtgatggagaactggcggtagatctccccggttgcaccgctcataaaggccatc
ggcataaacactgcggaaagaacaacggcaataccgaccagcgcacgttggatctgccccatcgatttatgcgtcgcttccttcggcggtag
cttatcttccgcaatgacacgctcgacgttctccaccaccacgatggcgtcatccaccagtaaccctatcgccagcaccatcccgaacatagtc
aacgtgttgatggtgaaaccgaccgccgacaagatcgcaaacgtcccgagaataaccaccggtacggcaatcgtcgggatgattgtggcac
ggaaattctgcaaaaacagatacatgaccaggaagactaggatgatagcctcaaccagtgttttgaaaacttcctgaatagaaatttcgataa
acggcgtggtgtcgtaaggataaaccgtcttcagacttgccgggaaataggctgataagcggttcagttcctctttgactgcccgcgaggtatcc
agggcgtttgctccggcagccagtttgatggcgatcccggcagcaggtttgccgttatagcgtgccacggtggaataatcttccgccccaagttc
gacgcgagcgacatcacgcagcagcacttgcgaaccatcttgctgaactttcaacaggattttgccaaattcttccggcgtttgcagacgcgtct
gcacaatgatcgaggcgtttagctgctggtctgccgcctgtggcatgccacccagttgaccaccggaaatctggttgttttgcaccttaatctggg
aaataacatcggaaggtaccaggttatatttattgagtttttgcgggtccagccagatacgcatggcatactcggaaccaaagagttgtacgcta
ccaacgcccgcggtacggcttagcgggtctttgatattagacgctacatagtccgcgatatcgtactggttgaggctgccgttatcagaaataaa
cgccgctaccatcaggatattactgctcgacttatcgacgctaatcccctgctgctgcactgcttcaggtaatgaaggcatagcgagttgcagttt
attttgcacttgaacctgtgcgatatcaggagatgtcccagtctcgaaggtcagagtgatagaggcattgcccgccgcatcactggttgaagac

atgtacatcaggccatcaagcccattcatattttgctcaatcacctgagtgaccgagtcttctaccgtttgcgcatcggcacctggataggtagcg
ctgacggtaatggtcggtggcgcaatctgcggatactgcgcaaccggtaagttcatgatcgccagaccacctgcaagcatcataataatggca
agtacccaggcaaaaaccgggcgatcaataaaatagttagccatgaaagtccccttaagcctgcaacgttattgtttcgattcggtgctggcgtt
ttcctggctggaggaaattgctcgtgctttgatacccggacgaatgcgttgcaaaccggaaacgatcacccgatcgccagcctgcaagccag
aggtgacgacccactggtcgccgatggctttgctggcttcaatttcgcgtagctgcacgacatcgtctttatccagaatgagcgccgttgctttacc
ctgggcgttgtgggtgacgccttcctgcggcaccagtaatacattctggcggctaccttcatccactaatgccgtgacgtacatgccaggcagca
agtcaccatttgggttggggaaaatcgcccgtaacgtcacggagcccgtggtttcatccactgtcgggtcggagaatttcagcgtgccggtctgg
ctgtagcgtttaccattttccagattgagctgtactggcgtactgccctgaacctgtttgatttgcccactggcgacctcttctttcatgcgtaagaaat
cttgcaccgactgcgtgagatcgacataaatcgggtccagacgttgtacggtaaccagcgaatctgcctgattagcggtaacgagtgcgccga
cggtcaccgacgatttcccgctgacgcccgtaatcggcgaggtgacattcgcgtattgcagattgatcgtcgcctgttcaacagccgctttggcg
acggtgacattggcttctgcttcattcaactgggtgcgcgcggtgtcgtaatcctgacggctaacgtagttggtcttcagcaacgatgcctggcggt
taaaggtgatgcgggcattgctggcggtagagagcgctttcgccagcgagcctttggcggagtttagctcggcctgtaaaggtgcaggatcaat
ctgatacagcgaatcgccctggttcactttatcgccttcgataaagttgcgtttaatgataataccgcccacctggggacgtatctcggcaacttca
taaggaacggttctaccgggcaattcgctcaacacattgaccgaaccgggggagagtgtgacgacaccgacctcaggcgtcatggcggcg
gcgttttccgccgatttgtcatcgcaggcggtgagcatcgcgccgcagaataacaacggtattaacagctttcttcttctgttcattttagtccctgaa
aattcttgagaaataacggttctttaaattgcgcgcgaggagagatatgagggatttacagaccagcggcatagtggggttgtcagccagtaag
gtgggatacaggcacagtgatcgacatggtgaggtcaacgacaaaaaaaatgttgccgttctgccaacagttcctgccagcattcgggcaac
gaagggttctactggtggatacacataccaggggaataaagtcgttttagcctctcctttagtaatcaccgggggtaatgaaatcagcatctgcat
ccctcgtcatgccagccatcaatttcagttgcttatgtcctgactaaaaataagatgtgatacccagggtgacgatgtcgctcatacgtcttagtttc
agcttgtacataatgttgaatttctgaatctttaatgtttgttgcgaaatattgagcgtcgacgtgatattgctttcatttttatttttcagaatcagttgcata
attttgaactgattatgtgaaaaacataattgtgaattcttatggggcaagtgtttaccataagtaatctcaagaagatttttgatactttctttgcggct
aacaaaataaataggcgctttagcttttagtttactgatgtgataaggtgccaaaacaataattctctcggcatctaatttctccagaaatttaatcg
cttcttcatcaaggatatgattgtgataggtatcaatgataaccttgaagggtttattgcctactttttgatctctgacagggaattgatttttatcatttcg
tgattatctttcaactgccaaaagccctgtaaaagaaaagaatctttcgtcatgagaaaaatcataacttgctccttagccgttatcgtttcgaatat
gtcatccttttcattatttacatccttgtccgaatcgttgttcaatatagtaaacgcctataagaactttacccttcgtgaactaatacgtctattaacact
aatcaatacttcagatttatccatcacgcactccatatattgatcctaagtaagattcctggttgttatcagcttgtaaatagcaaacaaaaagttaa
cgtttgcggattactgcccaagaataagttatcaataaccagtttgtgatctctgaagaatattactaaagttaaaatgtaatccgatttaaatatcg
agtctccttgtttcgacttaagctggcaattggattgccagctttcttttaacgcattgaaattacctgagtaaataacgcattcatgtcttcaggtttttat
atagcgtctggagacacggatatttatgcaatgtaatcaacgccagttgcaaaaaaatgaacccggattacttatttaaccgcccatagcatca
acgcttacggaactctttgttcactttcccacagcacttttaaaacattaaacctgactacggaaaatatcagccatgataatgtttgaaatggcta
atttgccatagagtgaaaaaaattagatgaaattcagtaggttgaaataatcactagcaggtaattatttcaatgatagtgcgcaattgatctaca
acactgcgtagcggagagagtattaatcggatcatagtcacatcaagtgactatgatccgggtgacaaccggggtaattattgctgcttaacga
acaaactggcgaagctgaacaggctggcggcgctaaatatcagttcaattcccaccagtgtggaaaccagcgttacagacaccatcggcgtt
gcaccaaggaatatccaggcaatgacgatatccagcacaccaataacgagctgtagccagctgcctttcattgaacgctgacgataccaact
catcaggcgaataacccctgcaacacagaacaaaccggcaataaatgccgcaatggcaaaaatgcccagctccggtgcgcggatgaag
aaatagccgatcaataaataggcgactgcgacgaggaaaccggataataccggccagaaattatgactgcggttgctgaataacccgaca
ataagcgcaatacccgagcagattaataatgcacccactactgtgcttaaaatatcgccagagacgaacgggaaactgatacacagcaacc
cgacgataaacagcagcacggcaataaactggattgctctgcgatgtttttaagcatctccagatcaaacttcaaaattgttgccttatctatatat
aacatagaaccaccctataaaattaagaagaaaatcccctgctatcaatctatgccaaaaacgcgtctaagaatgcagtcgatttaataaaaa
tttcctaattgcagtatctgatgcatctgtaactcattgtattgaaataaaaatatctgattttgatattttccatcaacatgacatatacagaaaacca
ggttataacctcagtgtcgaaattgattcgtgacggctctttcactttatagttgaggatattacgatgaaaaaagtattaggcgttattcttggtggtct
gcttcttctgccagttgtgagcaatgcagcggatgcgcaaaaagcagctgataacaaaaaaccggtcaactcctggacctgtgaagatttcct
ggctgtggacgaatccttccagccaactgcagttggttttgctgaagcgctgaacaacaaagataaaccagaagatgcggttttagatgttcag
ggtattgcaaccgtaaccccagctatcgttcaggcttgtactcaggataaacaagccaactttaaagataaagttaaaggcgaatggacaaa
attaagaaagatatgtaattccgggaatgcgttacatcgtacttccttgcatattgaacaggccggaatatcttctttaaaagcagctattcctcctgt
tcatatataatctctatattgaatgggttacaaaatgaatatttcatctctccgtaaagcgtttatttttatgggcgctgtagcggctttgtcactggtgaa
cgcacaatctgcgttggcagccaatgaatccgctaaagatatgacctgccaggaatttattgatctgaatccaaaagcaatgaccccggttgca
tggtggatgctgcatgaagaaacagtatataaaggtggcgataccgttactttaaatgaaaccgatctcactcaaattcctaaagtgatcgaata
ctgtaagaaaaacccgcagaaaaatttgtataccttcaaaaatcaagcatctaatgacttgccgaattaatgaggtgcaagtaaaaaggagta
gcaagttgagccatcttgctgctccttttttgcatttttatatgacagcagaatttattatacgtcttatactcgcggcaattgcctgtggcgctattggcat

ggaaaggcaaatgcgcggcaaaggagcagggttacgcacacatgtattaattggcatgggaagcgccctgtttatgattgtttcgaaatatggt
tttgctgacgtgctgtctttagatcacgtcggactcgaccccagccgtattgctgctcaggtggtgacgggcgtcgggtttatcggtgcaggtaaca
ttctcgttcgtaaccaaaatattgtaggtctgacgacggcagcggatatctgggtgaccgccgccataggtatggttattggcagcggtatgtacg
aactgggtatttatggttcagtgatgaccttgttggtgctggaagtcttccatcaattaaccttccgcctgatgaacaaaaattaccatttacagctga
cattagtgaacggtaatacggtatcaatgctcgactggtttaagcagcaaaaaataaaaactgatttagtttcgttgcaggaaaatgaagatcat
gaagtggtcgccattgatatccagcttcatgccacaacgtcgatagaagacctgttgcgattattaaaaggtatggcaggggtgaaaggcgttt
caattagctaatctgtgtggcagcgtagccagactcaccgtaagcctgaaattcacaccagataatcaatatgctgatggcgtaccagctcattg
atccgcttaagattgaggatcatcatgatattgtgtttatggcagtagaacgtttttagcgatattttcagtttctccagaatatcttccgttgtcatttgatt
cgcaatgcagctcagaacctgatgttgcttgcgcgaaagtgacacagccgcttcaggcacagagcagtaactgccattgatgagtgcgacaa
aatcaatgatagtgatagtccgcggaacaaaggtcaccggcactttccccaggcagcgtttaatattaaaggggggagaaaataatgacatgg
acaggttttaatcgttcaatttgagtgagaaaattagaatgaaatacattattcattaatgtatcaataatgacgaaggcattctgatgcaacattac
gtcgatctcttcttcgttctgtatatgaacgacattacctttactcagaatgtttttcatcgcggtgaagaacatcgtatccctggtaattataagaaac
ataaccgactcctggtgcggacattaactttataatcagtttgccaaaggtaattaacaggacagggcttataataataatagttagcttactgtgt
attaatttgatgtaatgcatgatgcagatgctgagaaagcatctgcatctttcggtggtgttatttgaccagctcaggtgttacctgactcaccgcatt
ggtgtagtaaggcgcaccccagcccggttccggccagaatgcaccatagccgtaatcccacaggttataggtggtattaaccacttctctcaa
atgccacacctggatgccctgcatattcacttccaggaagttatacgggactttattgataaagccaggttgttcaccctgaatggtgccgaggat
ggtaacaaagtgattacgatagttcactggatcaaggaagccgctttgtctggcgagcagtcggccctgatagttggcttcaatatcaggcttcg
catagctatccaacggtaatacagagatttctaacaacgtatccgttttgccattgataacgttgataaccttcccaccaaagcgcgcttgttgacc
aacatataaccccggctggttatgaacagcaacaaaactttttgaatatcaggttggttattgcctttgatattttgcggaattgaactacatgcgg
caagcaaaaatgaaaggctgaggatgagtgcaccttttgtcatgttcatgttactatccttatcaacaaatattaaaaccataatagatatttctttat
agttttcatctgattctgaggtgatatgcctgatagtaacgtaaacctgtctgatgtttatatcctttctgtttaggtgaactcagttaattttggggcgtga
catgaatatccatcaccccataatatagtgcattgttttaaaagtacataaaatctatttttattcaatgattataaagtgctaataatcaatattatggt
attatattctggtaatgatttgataagcgaattcttttaccagatggtatttctatttccaccccataatttatatctttttcattaggcagtggcctctttaaa
taaggcgtgtgtgacatttttttgggttaagcgtctcgtattgaaatacttcgaattgatattcagacatttctgcccatgtttgaattttattgtatcggga
gagtgtcgtaaaggctttaacttgcttataacattgtctttggaattcttgctgatattttaatatgtcattgtatgaaagattggttatcctggcctctaaa
aatttaattattgtcgaagcaatttccgcttttgctcgtaaaaggttgtcattaatggttaataatgttgaaaataaaccttcgatttgtttgtcaagatatt
gtccatctttatataatttgggggtagggtgttctttatgtaaaaaaatagtgtcacctaaggaaggtgcgaacaagtccctgatatgagatcatgtt
tgtcatctggagccatagaacagggttcatcatgagtcatcaacttaccttcgccgacagtgaattcagcagtaagcgccgtcagaccagaaa
agagattttcttgtcccgcatggagcagattctgccatggcaaaacatggtggaagtcatcgagccgttttaccccaaggctggtaatggccgg
cgaccttatccgctggaaaccatgctacgcattcactgcatgcagcattggtacaacctgagcgatggcgcgatggaagatgctctgtacgaa
atcgcctccatgcgtctgtttgcccggttatccctggatagcgccttgccggaccgcaccaccatcatgaatttccgccacctgctggagcagcat
caactggcccgccaattgttcaagaccatcaatcgctggctggccgaagcaggcgtcatgatgactcaaggcaccttggtcgatgccaccatc
attgaggcacccagctcgaccaagaacaaagagcagcaacgcgatccggagatgcatcagaccaagaaaggcaatcagtggcactttg
gcatgaaggcccacattggtgtcgatgccaagagtggcctgacccacagcctggtcaccaccgcggccaacgagcatgacctcaatcagct
gggtaatctgctgcatggagaggagcaatttgtctcagccgatgccggctaccaaggggcgccacagcgcgaggagctggccgaggtgga
tgtggactggctgatcgccgagcgccccggcaaggtaagaaccttgaaacagcatccacgcaagaacaaaacggccatcaacatcgaat
acatgaaagccagcatccgggccagggtggagcacccatttcgcatcatcaagcgacagttcggcttcgtgaaagccagatacaaggggtt
gctgaaaaacgataaccaactggcgatgttattcacgctggccaacctgtttcgggcggaccaaatgatacgtcagtgggagagatctcacta
aaaactggggataacgccttaaatggcgaagaaacggtctaaataggctgattcaaggcatttacgggagaaaaaatcggctcaaacatga
agaaatgaaatgactgagtcagccgagaagaatttccccgcttattcgcaccttccctaaatttgcgtcagtgaaatttatattaagtagatcttcg
aaataaaaccttgacaaggttagaacgaacccggacaggttctgtcctgtcatattgaaatcaataaagttggttccggcaaagactttcatcct
gtttttattttcactgtgctcaatatataacttcgtcatttttgatacaggctttgaatatcttttatattagttggtaagtcagctcgttgttcagcaataaaa
ttagtatctgaaaacatggtttggacactgaatgttatgggtattccctcttctacgctaattttagttatgattgccttgtcgggtttaggattagcgata
acaatgattcgacattcagcacttttgtttttttcatatctaatgttattaatctgattaagtattgattgtgtctgttgcgtaagttgtgtcgaacttatcgtttc
aatgttagtaataagacttacaagttgagccacaacttttgagtagtctgcatttgtatatgtagtgttcgtgtggcacgcattaatttgttgaagttggt
gttgcgctaaatcatattgatcagagatatatccttctccagagaatgtggaattaatgggttgttgttcttcccaattatcgcaaggccacttcggtgt
gtctggcgttttagcattatcggtatttgttgtgcagttataaatatcgccgtgatatgtatttattatacctggggtaaagtcaattgacataaaattcctt
attcatcatttgttttatgctaagatgtttaaattggataattagaacaggaatggatttcttctacacacaataaatgtaatgcgttatatcttcctgat
ataattacctttgatttcccttttatctccacccggtaatatttgtgtgtaaaaaaagggtagtgatagtatcgtaaaggaaatcgttaacaactatca
aaatgcgctcattttaaaaagttattatgttctctgatagtgtgtgaagtgatttttttgtgatgttttagatgtgtaataataaaatgggttaaggtatctt

ataattatttaataatttgttgtgtaaaccactgataaatcataatccagtacgtctttaaactactgcgctgtatcgctgataactaagcagctcactg
atacaccatgctggacgcgataattggaatctcacgcagggagtactgtgtaaatagcaaaatcgcctgatgcgctacgcctgtcaggcctac
aaataacatactattcatcgaattttcataactttgtaggtcggataagacgcgccagcgtcgcatccgacataaataaagcgcacttttctaaca
acctgtgggggggatatcgccgctttatttcaggcgcttacccgcttcatcaaccactttctcgccatcttccttggagaatgcgcctttttgcgcatct
ggcagaatttccagcaccacttctgaagggcggcacaggcgagttcccagcggcgtcaccacaatcgggcgattaatcagaatcgggtgct
gaagcataaagtcgattaaccgatcgtcagtaaatttatcttccgcaaggcccagctcctcatacggttcgacgtttttacgcagcagcgcgcgt
acggaaatccccatatcggcaatgagtttgaccagttcatcgcgcgttggcggagtttccagataatggataatagtcggttctgtgccgctgttg
cggatcatctccagcgtattacgcgacgtgccgcaggccgggttgtgataaatggtaatgttgctcatatcagtatctcattacaaagtgaaaga
gagacgtagcgccagcgcagccagcgtcacaaacagcacaggcagggtcatgataatccctgtacggaagtaatatccccagctgatagt
catattcttctgcgaaagtacgtgcagccagagtagcgtagccaggctaccaattggggtaattttcggtcccaaatcgcagccaatcacattgg
cataaaccatcgcttctttgataacgccagatgccgtgctgccatcaatggacaacgcgccaaccagtaccgtcggcatattgttcataataga
agagaggaaggcggtgaggaatccggtgccgagcgtcgcggcccacaggccgttatccgccagcacgttgagtacgccagaaagatattc
cgttaatccggcattgcgcaggccataaaccaccagatacatgccgagcgagaagatgacaatctgccagggggcaccgcgcaggacttt
acccgtattaatcgcatgaccgcgtttagcgacgacaaataatatcagcgcgcccacagctgcaatggcgctcaccggaatgccgagcggtt
ccaggacgaaaaatcccaccagcagaagcagtaaaacaacccagccagttttgaacgtagcaggatctttgatcgcttctgcgggagatttc
agcagcgccatatcgtagttctgcggaatatctttgcgaaaatagagatgtaacatcaccagcgtggcaacaatcgcggcgatatccaccggc
accatcaccgaggcgtattcgcgaaagccgaggccaaagaaatcagcggaaacgatattcaccaggttggagacaataagcggcaggct
ggcggtatcggcaatgaatccggccgccatcacgaacgccagcgtagtgcctttactgaaccctaaagccagcagcatggcgatgacaatc
ggtgtcaaaataagcgccgcgccatcattggcaaacagggcggcaacggcagcaccgagcaggacaatccaggtaaacagcaagcga
ccacgaccattaccccagcgtgagacgtgcagcgccgcccattcaaaaaagccggactcatccagcagcaggctgatgataatgacggcg
ataaacgcagccgtcgcgttccagacgatattccacaccaccggaatatcacccggatggaccacgcccgtaactaacgccagtactgcgc
cgagcgttgcactccagccgatgcctaaacctttcggctgccagataaccaatacgatggtcaggacaaagatagcgcctgccagtaacata
atgcctcccggataaaacacatctgaaaattcatatgtgtttagctaaatttttaactgcaaatgttcttactgtccccggaacagttttgtcgagcca
ggttgcggacaatcgcctgaaccttttcctgttcacatcgccaggcctcatcaataattttcgccgcccatgctggaatatgcggtgataagcggt
aatgaacccacttaccttgcttgcggtccagcaatagcccgctttcacgcagcaatgccaggtggcgggagatcttgggctgcgactggtcga
gagcagtgcagagatcgcagacgcataactctcccagttcgctgagcagtaaaacgatgcccagacgggtttcatcagcaagaattttgaac
aattggatgggtaacagaaatgacatattgcgctcctgattgttgcaggtagtgtctctcttcgaagcggataagtcaaaaacatatatgacttaa
cgaatgtgtaagtgcagaggaaggtaataggtgtgaattttgagttggctattcatttgaaaggaggtctgaatcaggacgtaaaaaaaaccgac
tttgcgtcggtttttttactttccagccctgagttggtggctctggctggagtgagagagttcttatctaacagctcaatactattagccagtggctaaca
ttgcaagaattagcgatttcttcagctggcggagggagcacttcggtatatatcgcagtgacgatttatatactttcactgggtcatcgtcatattaa
gccttgtccgaatccggatgtaaaaaaaccgactttgcgtcggtttttttactttccagccctgagttggtggctctgactggagtgagatagccatc
atctaacgcatcaatgctaataccatactgaaactattgcaaggacgtgctggttttataacctgcatgtactgtatgattatccagttagctctgag
gcattttcactctggcaatgcgcataaacgctttcaaagtcctggtcagaagtacgggtggtgccgttaactgatgctctggccggagtgagaga
gttcttatctaacaatgagacatgcgccgtgacaggcagtggatgagtaagcggatgcattctcactccatcgcatggagaaaacgggtgattg
ataaagcaatcatcgttctaggggcgttaattgcgctgctggaactgatccgctttctgcttcagcttctgaactgatagcggaaacgtaattaagg
gctaagagcacactactcttagccctttaacatttaacgcattgtcacgaactcttctgccgccgttgggtgaatggcgacggtattgtcgaagtctt
ttttggttgcccccatcttcagcgccaccgcgaagccctgcaacatttcgtccataccaaagccaatgccgtgaataccgacaatcttctcttccg
atccaacgcacaccagcttcatgcggcacggctggcggtgagtggtgacggcggtatacatcgcggtgaaagaggatttatacactttcacct
gatcgtcgccatactgctcgcgcgcctgcggttccgttaaaccaacagtaccaatcggcggatggctgaagaccacggtcggaatgttgctgt
aatccagatgctcatccggcttgttattaaacaggcgttcagagagacggcgacccgctgcaactgccaccggtgtcagctccactgcacccg
tgttatcgcccaccgcgtaaataccttcaatattggtgttttgatatttatcgacgacgatatagcctttttcgttagttttaacgccagcggcttccagg
ttgatgttgtcattggcaggctcgcgaccaatcgcccaaatcaggcaatccaccgtttcactgcgaccatcttccagctccagcgtcaggctacc
atcggtatttttcactaccgctttcgggatggcgttggtgtgcagctgcgggccttcggcgttcatcacttcgaccagcgtttcggaaatcatcgggt
cgaagctgcgcagcggcgcatgtttacgcacaaacagatgcgttttcgcgccgaggccgttaatcacgcccgccagctcaacggcgatgtaa
cccgcgccaacaaccgccacgcgctctggcaaagcaggaagggcgaagaagccatcagaatcaataccgtattccacgcccggaatatc
cgggtggctcggacgaccgcctgtggcgatcagaatatgatcggccgtgatggtttcgccgtttacctccagcgttttggcatcaacgaagcgg
gcaaagcctttgattacatcaacgttatttttaccgagcacgttttcataggaagtatgaatacggtcgatataggcggtacggctggcgatcaac
gtttcccagttgaatttattgatagtggtatcaaaaccataatccgggccgtacatatggatcgcttcacggatttgcgccgcgtgccacatcactttt
ttcggcacacagccaacatttacgcaggtgccgcccagctctttggcttcaatcagcgcacatttctggccgtacatagccgcgcggttgatgga
ggcgataccgccgctgccgccgccgatggcgatgtaatcatagtgtttagtcatgacaaagtgtccttatcgttgattaccgcgattgtagcgcga

gacgtaataggtgccagcaatggctgcaattactccggcacgatccagcttacggtggcgtgcccggtgcctgccggaaccagtttgctgtgc
agccacggcagcacgttattcatctgttgttccagtttccacggcgggttaatcacaatcatgccggaagcggtcatgccacggcgatcgctgtc
tggcagtaccgccagttcaatttgcagaattttgcgaataccggtcgcttccagatcgtggatcatgcgcttaatttgctgacgcagcaccaccgg
ataccacagtgcgtaaataccagtggcgaaacgtttgtaaccttctgctatcccgctgaccaccgcttgatagtcagttttcatttcatacggcggg
tcgataaggattaaaccacggcgggaaaccggcggcagtttggccttaagctgctggaaaccgtcggctttttcgacacgcgcacggctatctt
tctgaaattcagaacgcaacaacgggtaatcgctcgggtgcagttcggtcagttgcaggctgtcctgttcacgcagtagcaggcgagcaatca
acggcgaaccggggtagtaacgcaactgaccgctacggttgaagtgttttaccacattgatgtacgcctccagttctgcgggcaaatcgtcctg
ctgccagatacgggcgatgccttcgagatattcgccggtacgctcggcatgttcgctgcctaactgataacgcccggcccctgcgtgggtgtcg
agatagagaaacggtttatctttctctttcagcgactcgatgatcaggctctgaacggtatgtttaaggacgtcggcgtggttgccagcgtgaaag
ctgtggcgataactgagcatgggtaaaggtgttccggtaagtaaaaatcggcccgtattcggggcgcacaaaagcgtatcaggacagtatac
cgaaaagaggccgccgccgcgaaagcgtaacgtttctcattgaaattcactacacttaaccccatgctacacacattatgtaaagcgcctgttg
agcgcttccttaacctctttaaccaggactgcgcgaatgacgaatccgttactgactccctttgaattgcctccgttttctaaaattctcccggaacat
gtcgttccagccgtgactaaggcattgaacgactgccgcgaaaacgtggagcgcgtagtagcgcaaggggcaccgtacacctgggaaaat
ctctgccagccgctggcggaagtggacgatgtgttggggcgtatcttctccccggtcagccacctgaactcggtgaaaaatagcccggaactg
cgtgaagcctacgaacaaaccctgccgctgctgtcggaatacagcacctgggtagggcaacatgaagggctgtataaagcgtatcgcgacc
tgcgcgatggcgatcattacgccacgctgaacacggcgcagaagaaagcggttgataacgcactgcgcgactttgaactctctggcataggt
ctgccgaaagagaaacagcagcgttacggcgaaattgcgacccgtctttctgaactgggcaaccagtacagcaacaacgtcctcgatgcga
caatgggctggaccaaactcgttaccgacgaagcggagctggcggggatgccagaaagcgcgctggctgcggcaaaagcccaggccga
agcgaaagagctggaaggttatttgctgacgctggatatcccaagctacttgccggtaatgacctactgcgacaaccaggctctgcgtgaaga
gatgtatcgcgcttacagcacccgcgcctccgatcaaggcccaaacgccggtaaatgggataacagcaaggtgatggaagagatcctcgc
gctgcgtcacgaactggcgcaactgctgggctttgaaaactacgcctttaaatcccttgccactaaaatggcagaaaacccgcagcaggtgct
ggatttcttaaccgatctggcaaaacgcgcgcgtccacaaggcgaaaaagagctggcgcaattgcgtgcctttgccaaagccgaatttggcgt
cgatgagttgcagccgtgggatatcgcttactacagcgaaaaacaaaaacagcacctctacagcatcagtgacgaacagctgcgtccgtact
tcccggaaaacaaagcggttaacggcctgtttgaagtggttaagcgtatttacggcatcaccgctaaagagcgtaaagatgttgatgtctggca
tccggatgtacgtttcttcgaactgtatgacgaaaataacgaactgcgcggtagcttctacctcgatctgtatgcccgtgaaaacaagcgcggcg
gggcgtggatggatgactgcgtaggccagatgcgtaaagctgatggttctttgcaaaaaccggtcgcgtatttgacttgtaacttcaaccgcccg
gtaaatggtaaaccggcgctgttcactcacgacgaagtgatcaccctcttccacgagttcggtcacggcctgcaccatatgctgacccgcatcg
aaaccgctggtgtttccggtatcagcggtgtgccgtgggatgcggtcgaactgccgagtcagtttatggaaaactggtgctgggagccggagg
cgctggcgtttatctctggtcactatgaaaccggcgaaccgctgccgaaagagttgctggataaaatgctggcggcgaagaactaccaggcg
gcgctgtttattctgcgtcagctggagttcggcctgtttgatttccgccttcatgccgagttccgcccggatcaggggcaaaaatcctcgaaactc
tggcagaaatcaagaaactggttgccgtggtgccatctccgtcctggggccgtttcccgcacgctttcagccatattttcgccggtggttatgccgc
aggttactacagctacctgtgggctgacgtactggcggcagatgctttctcgcgctttgaggaagagggcattttcaaccgtgaaaccgggcag
tcgttcctcgacaacattctgagccgtggcggttcagaagagccgatggatctgttcaaacgcttccgtggtcgtgaaccgcagctggatgcgat
gctggagcattacggcattaagggctgatcattcagtgaaaatctgcttaattgatgaaacaggcaccggagacggtgccttatctgttctggcg
gcccgctgggggctggagcacgatgaagacaacctgatggcgctggtgttaacgccggaacatctggaattgcgcaagcgtgatgagcca
aaacttggcggcatctttgttgattttgttggcggagcgatggcgcaccgacgcaaattcggcggtggtcgcggtgaggcggtggcgaaagcg
gtgggcattaaaggcgattatttgccggatgtggtggatgccactgcaggacttgggcgcgatgcctttgtactggcttcagtcggctgccgcgtg
cggatgctggagcgtaatccagtggttgccgcgctactcgacgacggcctggcgcgtggttatgcggatgcggaaatcggcggctggttgca
ggagcggttgcagttaattcacgcctccagcctgacggcgctgactgatattaccccgcgcccgcaggtggtttatctcgacccgatgttcccgc
ataagcagaaaagcgcgctagtgaaaaaagagatgcgtgtctttcagtcgctggtgggaccggatcttgatgccgatggattactggagcctg
cacgcttgctggcgaccaaacgcgtggtggtaaagcgcccggactacgcgccaccgctggcgaatgtcgccacgccaaacgcggtagtca
ctaaagggcatcgctttgatatctatgcaggtacgccagtgtaaaactaaaacagggtagcgataacgctaccctgctttctgcaagaataatta
atggctttccggcgtcgcaatcatgcgtttcagccacggcaccatcaacagcatcactactgcaacgcccagcgtgaccagaccaatcttacc
aaacacgttggtatagacgggcaacgtctcaagcggatcggtaatgttgtccggcaccgcggtaaatgttgccacatagccgcccagcaaga
acgcggcagcctgcgtcaggaaccacatcccgagaataaagcccatcaaatgctgcggcaccagggcagcaatcatcgccaggccaag
ggcgctaataaacagttcacctaagctctggaataagtacaccagcacgataaaccatggcgatgtcagcccttgtgcatccgcaaaccaca
ttcccgcagctgccgccgtcaaaaagcccagtgagcacataaacatgccgagagtaaatttcatcggcatcgagaggtctttgcctttgttacc
cagatgcgtgtaaatgcctgccagtattgggctggcgagtaccacccagaacgggttaagcgcctggaagctgaccgggttgatggaaaaa
ccgagaatttcatgatgcacgttgttgatggcaaagaagttcagcgatgttggcatctgggcgtagagaatgtaaaacaccaccgcttcgagca
tcaggacaaaggcgacaaacattttattgcgcccggttttatccagcttgaatgcctgacgaaagaagatgatggtgacgacgatggagaga

acaatcagcaccagattggcgacttctacgttgtgcatcagccatgcgcatacgaagatcatcaccacgctgccaagtaacacgtacaacag
tttgctgaagctcattggccggaagtcgggttcagaaccaatgtctttcaccattccacgacaggcgatgtaaaccagtaatgcgataattaacc
ccgccccgcacaggttgtaggtgactgaataaccgaatctatcagcgatcacaggggccagcgataacgctatcaacgagccgatgttgatc
gacatatagaacagggtgaatgcgccatcaagccgcggatctttcggcggatagcacttcgaaagcaagctggctgggttagctttaaacag
gccgttaccgacagcgatagtccccagggcgatgaaaatcaggtcaggcttaagtagcgacatgccggtcatgaagtagccaatcgccagc
acaagtgctccaagaacaatggtgcgtttggtccccagcaggtggtcgccgacatagccgccaatggaaatgaggccatagaccagcgca
gcaaaagcaccaaaagtgacaaaagcctgctcttgcgagaatccaagctgtttaacgaagaaaaccgccagtacgccctgcacgccgtag
tagccgaatcgctcccataactcgacaaaaaagatcatgaaaaatgggcgaggttgctgcagcatccccatgggtgttgttgtattcatatttatt
aaccttccaataccatcctgaaaagtatgaacgcgtaagggataacgcgcgaaaaagtttacatctgtcagagattcttaatattgatgcggtg
aatagattaatacactattacaacagaaaataaccagatgatcacactaatgatggaaaatatgatggtgttaaatgttgtattggctggggatttt
gattgcgtacgcagaccgtaggccagataaggtgtttacgctgatcaggtaacaaggcgcgccaaaaagcaaaacgcccgcatatgcggg
cgttcaggaacacgtcgtagagggaagattattcttcttcgtcgcgcagcggaacaatcagcatatcaacgtgaacggtgttgatcagctgacg
tgcggaagacatcagtttgctccagaagtcctggtggtgaccacaaaccaccaaatccatatcgtatttcttgattgcatcgaccagaacctggc
ccaggtcgccgctgccgctcagggtttcagtgattgggtagcctgcattagtggaaagctcggtcagtgcatgatgtgtctcttcagagatgcgttt
ctgcatatcacccagattcacatcaataagcccggtgtataggtcagagtagtttacatctacgtggatcagagaaactttcgcattgtaggggc
gagccatagagactgctttctctaccagaactttgctttccggggagaggtcgaccgcgatgagaatgtgtttataagccatagtgttactccttcc
ataaagttgtcgatgactggccagctagcgtttcttgtgccgttacgtcacccgcgtcctgcgaactacgtgcgcggggctaacccagccgtaa
agattcagtgttaagaccatccttaccttatagcgacccggatgatccgtcaatccgccttgcttactcgttagataaacaaattttcgacatgaata
tattgatagtggttaaccttctggaaaaaaacaacctgatctcctacactatctatagagccgctcgtatgttggtcaccacaaaagcggtttctc
caggacctggtgttaactgattggcagcgtatcgggagcggtagccccggggtggaactccgcgggcaggtcgccggggaggaggtatgat
aagcaccgtcgcattattttgggctttatgtgtcgtttgcattgttaacatggcgcgctatttctcatcactacgcgcgttgttagtggtactgcgtaact
gcgatccattgctctatcaatatgttgatggagggggctttttttacctcacatggccaacccaacaaacaggtgcgtctcgtttggtatatctatgcc
caacgttatcgcgatcatcacgatgatgaatttattcgccgctgtgagcgggtgcgtcggcagtttattctgaccagcgcattgtgtggtctggtggt
ggtcagcctgattgcattgatgatttggcattaagcacttcagcaataataacgcataaaaaaagcgggccagtaaactgacccgctcgagac
tgattacaaacgcaaaattgcctgatgcgctacgcttatcaggcctacataatctctgcaatctattgaatttgcatgtttttgtaggccggataagg
cgttcacgccgcatccggcatgaacaaagcgcactatgtcacaatctgaagcgggccagctgacccgctcgttttggtgcgtacgattacagg
aactgcaaggagagccagtacagcccgccggaaagcagtaccgcagccggaagggtaaacacccaggccatcagaatgctggtcacg
gttttacgctgtaagccgccaccatctaccaccatcgtccccgcgacagaagaggagagtacgtgagtagtggaaaccggcatcccggtata
actcgccaggccgatagacactgccgccgtcatctgggcagacatcccctgagcgtaggtcatgcctttcttaccgattttctcaccgatagtcgt
tgccacacggcgccagccaatcatcgtaccgatacctaacgccagcgcgaccgccatgatgatccacaccggtgcatactcgatggtgcta
agcatgtcggacttcagtttcttcaacaggcgctgatcgtcagcactcacgccaggcatcttcaccactttgtcgatagtgtcagaaacgcacag
cataatgcggcgcatctggctacgttgatcaagcgacagcttgtcgtagctttccacatcggtggtcaacatacctttcaggcggttgagcgcgtt
aatggtattcgacggatggcagtggaactccgcaggttgcgttgcgccagcttccggagccggtactaactgatcagcaccggtagcctgtttg
agcagcgcaggatgctgctcaaagtaagcttcgacgttgttgatggcatcacgggtacgggtgatttcgtagccagtggcattcatgttcaccac
gaagcctgctggcgcgacgccaatcaataccaacataaccagaccaatgcctttctgaccatcgttcgcgccgtgcgaaaacgccacgccg
atagcggaaaggatcagcgcaatacgcgtccagaacggcggctttttcttgccgtctttcttttcacgctccgctggggtcaggtggatacgggc
gcgtttcttggtgccgctccagtagcgacgcagcaagaaaatcagaccgccagcaaacaccaggccgacaataggggaaacgatcagag
aaccgaaaatacttaatactttcgggatattgagtgcatccaccactgacgtcccggtcatcaacgcattggttaaaccaatcccgatgatcgcg
ccaatcagcgtatgagagctggatgcaggtaaaccaaagtaccaggtacccaggttccagataatcgccgccagcaacatagagaacacc
atggcaaggccatgagacgatcccatattaagcagcagatccgtcggcagcatatgcacaatggcataggcaacactcagaccacccagc
aaaacacccaaaaagttgaataccgccgccataaccacggcgagctgagaacgcatcgcgcgggtatagataacggttgccacggcgttg
gctgtgtcatggaaaccattgatggcttcgtagaacagcacaaaagccagtgcaagcaataataacagcccggtatgcaaatccaggccag
caaacaaatgtagcataggacgttacgccattttgaggacatgaacgcggcgcattatcagtgactttcgcggcgcgggcaaagtgaaatata
gactttttttgatttgccccctgtatggatttcactcaaaaaataattatcttatataattcaggcaaatacttccttttagtaatattgatgctggtgcgac
cactgaggaatctttacaattcacgcccgtttttttctaagaggagcgcaacgtggaaaggtttgatgccattattataggcgctggtgcggcgggt
atgttctgttctgcgctggcaggtcaggcaggacgccgggttctgctgatcgataatggtaaaaaaccagggcgcaaaatccttatgtctggcg
gtgggcgctgcaactttaccaacctttatgtcgaaccaggcgcttatctgagccagaatccgcatttttgtaagtctgcactcgcacgttttaccca
gtgggatttcattgatctggtcaataaacacggcatcgcctggcacgagaaaacgttagggcaactcttctgcgatgactccgcgcagcagatt
gtcgacatgctggtggatgagtgcgagaagggcaatgtgaccttcagattgcgtagcgaagtgctgagtgtggcgaaggatgaaacaggctt
cacgcttgatctgaacggcatgactgtcggttgcgaaaagctggtcatcgcgactggtgggctgtcaatgccggggctgggcgcgtcgccgttt

ggttataagattgccgaacaatttggcctcaacgtgctgccgacccgcgcgggtctggtgccattcactctgcataaaccgttgctcgaagagtt
acaggtgctggcgggcgtggcggtgccttccgtgattaccgctgaaaacggcaccgttttccgtgagaacttactcttcacccaccgcggcttgt
ctggaccggcggtgttgcagatttcaagctactggcaaccgggggaatttgtcagcatcaatctgctaccggatgtggacctcgaaaccttcctg
aatgagcagcgtaacgcacatccgaatcaaagcctgaaaaacacactggcggttcatctaccgaagcggttggttgaacgcttacagcaact
cgggcaaatcccggatgtttcgctaaaacagctcaacgtgcgtgaccaacaggcactgattagcacattgaccgactggcgcgtacaaccc
aacggcactgaaggctatcgcactgccgaagtgacgctcggcggcgtggacaccaacgaactctcttcacggacgatggaagcgcgcaa
agtgcctgggctgtacttcatcggcgaagtgatggacgtcaccggctggctgggggctataacttccagtgggcgtggtcgagtgcgtgggct
tgtgcgcaggatttgattgcagcaaagtcgtcctgacctatttcctaaagatgaaaattattagagtcgcattaaaaatgagctaattttgatagtgg
ttatcttgtgattattttctaatgagcccgtgaactgaaaccctccatgcttaatataaggtggatggaaaggtgattgaaaactcactcagtggcc
agatctttatgaaatatgaacaggttgaaaaaaacagtaactttgctgtttttatacttaaccactatgcattaatgctgcgttatttcatgatgcctaa
gaaaaaccagcgttacgcaaatggtcaacgctggttttatccggtacgttgcaattattttttagcagaacccgcttctaatattgaaacgattgag
aacaacgtaaagcataccgctcccaaaccgaccagaactcgcgaaggaataaaatatcccatgtcggtctgcgccatttccgcaagaaac
gatgccaggaaaaggcaaaacaggcaggtgaagacgggaatcatcggtattctgttggctaacgaacaggtacgtctccataccagtgcca
gtagccagacttttgagaatatgctgtaacagatcattccgaggcaaataagaataatgccgggagccagtcgggcgcttgcatcggaactga
ctaagacgtatatcccctgcagtaccgtgattgagccgagaaaaataacccaataacaccacagccagtgttctttagttgaaaacgtattacg
tgtttgatggacaattgtggcaacaaggccaattagacaggcacagattgcggttaacccaagcaatacgtgacccgcgacataatgcggag
ttatgtcagcactacgtaacagcgtaatagaccaaatgaaaccgagcagggtgagtaaaacgggaacggcaatgaggcagttaccgatta
atgaagaataagcctgtaccggtgttccgtcgctcttgctccccgctgcatttttgggaatgagcagaaagtgaccggatgacgctgccaccgtc
gatacacaggcggcaatcataccaacgccgaaaataacatggccggcgacaaactcgtctgccatcacatcattgcctgctaacagtgccc
aaccccatatcatggtgataattgaccccgcatagccaataatggggaacaatgtattgtaaaacgtattaacgccgcgcgtgagctgcgaaa
tgataataaatgcggtagtgaataatgccaggcatatggccgccagagaaatcagcacatgacccgcgacgaaatactcgctttgccctgaa
ctttccagcacaaagccacctaacgcgatacaaattaagcccataatcaaagggattaatttgaaaagccacccgatatatatgttcactttaa
actcctcaggtatccctgctaatattgaaatatgcattaatgataatttcatcctgttaatataatgacgagatatcattaaagacaaatgcatagca
taactatagcggggtttactttgcagagcgctacttttcagcgttcaaaaggccatgataaataaaaatttatcctaagattaattcttatgctgttgttt
tttacaattatcttccagggagggattattcttcattatttggtaaaagaccggatagtctcggtgcttaaaatcagcgaacatataagtattattatttt
atatcgtcgtaaatctgtttttcagacaagtatcatttattttgttacaaacaattcaaaaatatcacaggttatttatgaaagcaattgacaatcagatt
agaaacatctcttcaagccatcaagataagcattcagataaagttaacagtcatcaacaccatggcaaggttgataaaacacatcgtgcaaa
gatagttgaatttgataaactggataatgattcgcaaatagataacgattttggcctgcatatcatctatttcctgcaacatggtcactggaaggtaa
atgatcgtagtcaccagatggagaaagtatggttttataatagtgaaccttctatagatattcaagaatataatagattcgccgacaatactactga
tacgtttatctttacaattataccagacaataaccatgtgttaaaattatcgtctcccattaccgttactgttgagtgtaagggcggttattattttattaat
tcctctggagataaatcagatataatttataaagttgatgggctttctatcatagcgagaaatttctttactcttcttagtggtaatttcaaacctgactg
gcgttgggatgtctctaaagagacgtttactaaggaaaagtttgatagctatgttaaatctgtgttttcaaaaatagacttctataaacagtgtggtgt
gattaacccacaaaatgccaacacggcttattttggcgatacagatggaagagtaggcgctgtactttatgcattgctcgtttcagggcatatagg
catcagggaaaagggttggagtttattatgtgaattattaaaacacgaagagatggcttcatctgcatacaaacataaaaataataaagtgctct
acgatttattaaatacgagagacatgatcttgaatgagttgcatcaacatgtttttttaaaggacgatgcgataacccccttgtatcttccttggggatc
atacaggcgatagatttagtactatttttggtgataaatatatccttactttgctaaattctatgcgaaatatggagggtaataaagatagccgtatta
ataaaaatgtcgtcgttctggctggaaatcatgaaatcaattttaatggtaactatactgcacgactagcaaatcataaactctctgccggtgatac
gtataacttaattaaaacgctcgatgtttgtaattatgactcagaaaggcaagttttaaccagtcatcacggaataataagagatgaagaaaaa
aatgttattgtctcggtgctttgcaggttccatttaatcagatgaaaaatcctactgatcctgaggaattagccaatatttttaataaaaaacataaag
aacacatggatgatcctctattccatctaatccgaagcaatactttgaaacccacacctgtatatgctaattattttgataacactactgatttcagac
cagcacgtgaacgtatcttcatatgtggggagacactcaaaggtgaagaccccagcaaatacattagacaaaaatatgggcatcatgggcc
cggggtagatcataatcaacaatttgataatggtattatggggctaaattccctgaaggaagcacgtgataaaaataataaaataatttattcctc
agggcttagttgttttcaactacattaaaactccatgaatacggtggtatgtctttttaatcctgccagtatttcataggttttcttaatcgattgtagagg
catgttctgatgacgcctctacaatctttagggactttctctgctatgagataagaaatttcatattatgctttgaggctgaatgatttgtgcaaaatata
attattttcccatcgaacatgaggtgtatgtcataagataaaatgttaactatgtattcctcgcggtaaataatatgaggtaaaatatgaaaattgga
actgtggcaggcactaacgacagtacaacgacaatcgcaacaaatgatatggtgcaagagcatgttaccaacttcactaaagaactgtttgg
gtatattgcaaatggtataggtgatgatatatcaagtattgccaggacaatgctcggtgaggttgtggaaaaaatagatgattggcaaatagaac
ggttccaacaatctattcaagatgacaaaatttcatttaccatacaaacggatcattcagaaaaatatagtatgctatcgggcatgcgtgctcatat
tctacgtaggaataataattatcagtttattgtgacaattaatagcaaaaattacggttgctctcttgataataccgatattaattggtgcagtattgttt
atttacttaataatatgacagtaaatgataatgctaatgatgttgcagttactgagtcgtacaaacccatctggaactggaaaatatcgcaatataa

tgtatctgatattaaattcgaaacaatgataaaaccccagttcgctgacagaatctatttctccaattgtttacccgttgatcccacaagtaccagac
caacctattttggtgataccgatggctcagtcggtgcagttttgttcgcgcttttcgcaacgggacatttgggaatcatggctgaaggagaaaatttc
cttagccagttgcttaatatagaagatgaagttctcaacgtattgctaagggagaactttaatgagcaacttaacactaatgttaatacaataatca
gtatcctgaacagaagagacattattttagaaagtcttcagccttatttagttattaataaagacgctgtgacgccatgtacgtttctgggcgacca
aaccggcgatcgatttagcaatatctgtggtgatcaatttatcatcgatttgttaaaacgcataatgagtatcaatgaaaatgttcatgtattagctgg
aaatcatgaaacgaattgcaatggtaactatatgcagaacttcacgcgtatgaaaccactcgatgaggatacctactctgggataaaagattat
cctgtatgtttctatgaccccaaatacaaaataatggcaaatcatcatgggataacttttgatgatcagcgaaaacgttacattataggaccgata
acagtctctattgatgaaatgaccaatgctctcgatccggtcgaattagcggcaattatcaataaaaaagcatcatgctataattaatggcaaaaa
gtttaaaactagccgggctatctcttgtcgatcctttaatcgttatttttctgtttcgacagattacagacctaaacttgaagcactattagcatgttcac
aaatgttaggtataaatcaagttgttgcacataacggaaatggtggaagagaacgcataggcgaaacggggacagttttaggtcttaacgccc
gtgacagtaaacatgctggaagaatgttcagcatgcataattgccaaattaaccctggtgcagggcctgagataaccactccctggaagtctta
ccagcatgaaaaaaacagaaatggacttatgcctttgattagaaggcgcacaatgttacagctctgaaccatagacaacatgaatattaatgtt
actatctggttgagactgttgataaaacgtaaaaaggaatgctctgttcctgcaactgaacaggcgtgaaacgctatccaacaggatggatacc
gttttgtcagccagttttatagatcacacttatgagtatgcgtgtttcatttacggccaggaatgctgatgatgacaacattactacccgttttcactaa
gccttccccgttggcgctcaatgctctgcgcgctggtcgtatttgccgtttccttcttatcccggatggaagaatccgctgaacgtcgttcggcggca
gttgtaacacgcgtcgtttgcttacagggattatggcgggtcgattatggataagagtaagcgccatctggcgtggtgggttgtcgggttactggc
ggtggcggctatcgtggcgtggtggctgttgcgcccggcaggtgtgccggaaggctttgctgtcagtaatgggcgcattgaagcgacggaagt
ggatattgccagcaaaattgccgggcgtatcgacaccattctggtgaaagaaggcaagtttgttcgcgaaggtgaagtgctggcgaagatgg
atactcgcgtgttgcaggaacagcgactggaagccatcgcgcaaatcaaagaggcacaaagcgccgttgctgccgcgcaggctttgctgga
gcaacgacaaagcgaaactcgtgccgcacagtcgctggttaatcaacgccaggcagaactggactccgtagcaaaacgtcatacgcgttc
ccgttcactggcccaacgaggggctatttctgcgcaacagctggatgacgatcgcgccgccgctgagagcgcccgagctgcgctggaatcg
gcgaaagctcaggtatcggcttctaaagcggctatagaagcggcacgcaccaatatcattcaggcgcaaacccgcgtcgaagcggcacaa
gccactgaacggcgcattgccgcagatatcgatgacagcgaactgaaagccccgcgtgacggacgcgtgcagtatcgggttgccgagcca
ggcgaagtgctggcggcaggcggtcgggtgctgaatatggtcgatctcagcgacgtctatatgactttcttcctgccaaccgaacaggcgggc
acgctgaaactgggcggtgaagcccggctgatcctcgatgccgcgccagatctgcgtattcctgcaaccatcagttttgtcgccagtgtcgccc
agttcacgccaaaaaccgtcgaaaccagcgatgaacggctgaaactgatgttccgcgtcaaagcgcgtatcccaccggaattactccagca
gcatctggaatatgtcaaaaccggtttgccgggcgtagcgtgggtgcgggtgaatgaagaacttccgtggcctgacgacctcgtggtgaggttg
ccgcaatgacgcatctggaactggttcccgtcccgcctgtcgcgcaactggcgggcgtgagccagcattatggaaaaaccgttgcgctgaac
aatatcactctcgatattccggcccgctgtatggtcgggctgattggcccggacggcgtcgggaagtcgagcttgttgtcgttgatttccggtgccc
gcgtcattgaacagggcaatgtgatggtgctgggcggcgatatgcgcgacccgaagcatcgccgcgacgtctgcccgcgcatcgcctggat
gccgcaggggctgggcaaaaacctctaccacaccttgtcggtgtatgaaaacgtcgatttttcgctcgcctgttcggtcacgacaaagcggag
cgggaagtgcgaatcaatgagctgctgaccagcaccgggttagcaccgtttcgcgatcgtccggcagggaaactctccggcgggatgaagc
aaaaacttgggctgtgctgcgcgttaatccacgacccggaactgttgatccttgatgagccaacaacggggggttgacccgctctcccgctccca
gttctgggatctgatcgacagtattcgccagcggcagagcaatatgagcgtgctggtcgccaccgcctatatggaagaggccgaacgcttcga
ctggctggtagcgatgaatgccggagaagtgctggcaactggcagcgccgaagagctacggcagcaaacgcaaagcgctacgctggaa
gaagcatttataaatctgttaccgcaagcgcaacgccaggcgcatcaggcggtagtgatcccaccgtatcaacctgaaaacgcagagattgc
catcgaagcgcgcgatctgaccatgcgttttggttccttcgttgccgttgatcacgttaatttccgcattccacgcggggagatttttggttttcttggttc
gaacggctgcggtaaatccaccaccatgaaaatgctcaccggactgctgcccgccagcgaaggtgaggcgtggctgttcggcaaccggtt
gatccaaaagatatcgatacccgccgtcgggtgggctatatgtcgcaggcgttttcgctctataacgaactcaccgtgcggcaaaaccttgagtt
acatgcccgtttgtttcacatcccggaagcggaaattcccgcaagagtggctgaaatgagcgagcgttttaagctcaacgacgttgaagatatt
ctgccggagtcattgccgctcggcattcgccagcggctttcgctggcggtggcggtgattcatcgcccggagatgttaatcctcgatgagcctact
tctggtgtcgatccggtggcgagggatatgttctggcagttgatggtcgatctctcgcgccaggacaaagtgactatcttcatctccacccactttat
gaacgaagcggaacgttgcgaccgcatctcactgatgcacgccggaaaagtgcttgccagcggtacaccgcaggaactggttgagaaacg
cggagccgccagtctggaagaggcatttatcgcctatttgcaggaagcggcagggcagagcaacgaagccgaagcgccgcccgtggtac
acgacaccacccacgcgccgcgtcagggatttagcctgcgccgtctgtttagctacagccgccgcgaagcgctggaactgcgacgcgatcc
agtacgttcgacgctggcgctgatgggaacggtgatcctgatgctgataatgggttacggcatcagtatggatgtggaaaacctgcgctttgcg
gtgctcgaccgcgaccagaccgtcagtagccaggcgtggacactcaacctctccggttcccgttactttatcgaacagccgccgctcaccagtt
atgacgagcttgatcgtcggatgcgtgcgggcgatatcacggtggcgattgagatcccgcccaatttcgggcgcgatatcgcgcgtggtacgc
ctgtggaactcggcgtctggatcgacggagcgatgccgagccgtgctgaaacggtaaaaggttacgtgcaggccatgcaccagagctggtt
acaggatgtggcgagccgacaatcgacacccgccagccaaagcgggctgatgaatattgagacgcgctatcgctataacccggacgtaaa

aagcctgccagcgattgttccggcggtgatcccgcttctgctgatgatgatcccgtcaatgctaagcgcccttagcgtggtgcgggaaaaagag
cttgggtcgattatcaacctttacgtgacccccaccacgcgtagtgaatttttgcttggtaaacagttgccatacatcgcgctggggatgctgaactt
tttcctgctctgcggcctgtcggtgtttgtgtttggcgtaccgcataaaggcagtttcctgacgctcaccctggcggcgctgctgtatatcatcattgcc
accggaatggggctgctgatctccacctttatgaaaagccagattgccgccattttcggaacggcgattatcacgttgatcccggcgacacagtt
ttccgggatgatcgatccggtagcttcgctggaagggcctggacgttggatcggcgaggtttacccgaccagtcattttctgactatcgcccgcg
ggacgttctcgaaagcgctggatctgactgatttgtggcaactttttatcccgttactgatagccatcccgctggtgatgggcttaagtatcctgctgc
tgaaaaaacaggagggatgatgcgccatttacgcaatatttttaatctgggtatcaaagagttgcgcagtctgctcggtgataaagcgatgctga
cgctgattgtcttctcgtttacggtgtcggtgtattcgtcagcgaccgttacgccaggatcgttgaacctcgcgccgatcgccattgccgatatggat
caatcgcagttatcgaaccggatcgttaacagcttctatcgtccgtggtttttgccaccggagatgatcaccgccgatgagatggatgccggact
ggacgccggacgctataccttcgcgataaatattccgcctaattttcagcgtgatgtcctcgccggacgccagccggatattcaggtgaacgtc
gatgccacgcgcatgagccaggcatttaccggcaatgggtatatccagaatattatcaacggtgaagtgaacagctttgtcgcgcgctaccgt
gataacagcgaaccgttggtatcgctggaaacccggatgcgctttaacccgaacctcgatcccgcgtggtttggcggggtgatggcgatcatc
aacaacattaccatgctggcgattgtattgaccggatcggcgctgatccgcgagcgtgaacacggcacggtggaacacttactggtgatgccg
ataacgccgtttgagatcatgatggcgaagatctggtcgatggggctggtggtgctggtggtatcgggattatcgctggtgctgatggtgaaagg
tgtactgggcgtaccgattgaaggctcgatcccgctgtttatgctgggcgtggcgctcagtctgtttgccaccacgtcaatcggcatttttatgggga
cgatagcgcgttcaatgccgcaactggggctgctggtgattctggtgctgctgccgctgcaaatgctttccggtggttccacgccgcgcgaaagt
atgccgcagatggtgcaggacattatgctgaccatgccgacgacacactttgttagcctcgcgcaggccatcctctaccggggtgccggattcg
aaatcgtctggccgcagtttctgacgctgatggcaattggcggcgcatttttcaccattgcgctgctgcgattcaggaagacgattgggacaatg
gcgtaaatacaattgcccgatgcgtaagcttatagggactacgcatcggtgcaatttattgaattttagttgtttttaggccggataaggcgtttatga
actgacccccaaaacctggacagtttcagttaaccggctttttgatactcagtcctgtattcagggcaagattacgaaagcccgctccccgcaag
gactgacgccagatagtttctgtccatggctgcttttcgcatcttacgtcttaaccctgccttgaataccttatcattcgtcaaaatattaatagcaatgt
gccgtatccctgaaaataattctgctgcatttcctcttcttattttgcagtcgtcttcattcattaccacgtccagacgccagtgcagcttattctccacgt
gccagtgatttcggatcgctgtggcgaatttctcagcggttaaatcagcagaactgatataatatctgaccgtcatttctggctctttcttttgttctgct
attatggaccgaaaggagactgccacgcataatttcttcagccctttccattcaaacgtgaaatcaataagttcatcagggacatcgcaaacaa
tatgaagacggatttcttctctgccgtgactcttttcactgattgcgtaactgtcatgctctggattatttaattctttcagcggaaatttttcctcaaaggc
tttatttagccgcccctgggttcctttaccgcgaataaataatcacctccctgtttttgtatcttctctgcaatatctttctggcaacccatcgcatcagtt
gtgatgatttttcctttaatatccagcatgttaagaagttctgggatagctgtaatctcattagatttctcatccgtcttgatctgtccgatgaccagactg
tgcattgttgagaacgcactaatgacatgaatcgctcccctgcggcgactcttgtcataagagtgccggagcgtttttccatcaattgcaatgacgt
ctttatcatctgaagaatggcagtcacgcatccagttaataaagcactcgtgaaattttgcaggactgatacaggatacaactctggcaatggtat
cgtgaacaggaataccattttcaaaatcaccatattgcttcaaaaaatcgagatgtgtttccccaaaatcctctatatcttcccaaccttctgcacc
agaaataacggcacaaatagtcaacagtagaatatccgataatttatgttccactttccaggtttgtctgtaatcggggataatagaaatatgttcc
atcaatttttttaagttccattttgttctccttaattaggtaaggagtatttgatcatgtataagcaataaaaaaacagcttcaggtaataaggaatatctc
aatttttaaacataaaatgcgaattatttagtgcaaaaagtgaacttttgatgatctttccctggccgttgactcgattgccgtagctcgaacgcaag
cactcttatcattcaatgccatattgttcaaaatattaaaaacattatcttcgcatttttttgcaaatttatatttgcccgagtataaatgctccgatatctct
acggatataatttttgtcagtgcagaattcaattgccaatctaacagcgtcttgccccccctcttaactgtaatactctggcagatgaaatccttttcaa
ggaattgcgatcatcaagtaagcgaaataattcatcatcatttagtttcttacatttattatataaaccatactctttacttgcctttcttttctggtatgtatt
tgacattgcaggaacctccataaaggctatctttttctatcgtttgactgtctggagtcctccacctactctgccatcagcgcacacgatgtttgattat
ccttacatacttattttccgtggccaaaattcagaattctcccatcatttttgcctatttctataataaaaaccccaccagcaatttggtctgagtgaatg
gtaccttcaacaacccaacttgttgttaactcagtgatttcgtatggtttttcttcttctgcggcatcctgtccataacgattcttaacatatatctcagcta
attccaatgctatagctttatttttaaccaaatttctaacggtatatttaccttcactttcatacttgcgcattattttaatattattattacactgcgcccatgt
gttgtatgtaaaaacaaacaaagatacaatgacacagttcctaaacaatattttttttacatattaccatgaactctgcagacattaggtaatcctttt
ctgtttgttatttccttcccgtggttatctattgctttaaaattaccactgggcgtacctaaataaaatgacttgatattgttatctttacttttaactattttctc
atctttacctgaaaatatttcgtcccaatattctccatgactatctgcaccatgagtatgataagctgatactttctcagtaccattagggcaagggct
attgaatggatatgatccttttctttctcctcggttaggtgctgttgagaaatatctgccactattatccttacatattaaaccagcgaactctttatcttcg
caaatagattggctatttattttatttaaggcgtcatatgcggtgctatctatagaatcaaatttatctggacctatcatagcggatagtgtgccgcaa
agaccattagtacatgcccccgattttgcatcctcccacatagataaacccagggggtcgatgtctgatatcggattcaattgatatccatacagg
ttccatcccccccttcagtccaatcggatcctgagtgatatatcgcccctgcagcgggtcataatagcggtggcggttgtaatacaggccggactc
ctcatcatactgctgccccggcagccggataagctgctgcagctgatgcgggttctcttcattcagcaggttgccccattcatcatattctgcgcac
cacgctgttgccccttccgtgctgatgagcgccagcggcaggccgcgatggtcgcagtggtacaggtggatttttcgcgccggcgtgtacaccg
ggtccatctggttttgcatctgctccacggtcaggccgcacgatgccagccagcggcggctttcctcactcacccggtcagccaggatttcacttt

ccagccggtcgagcatctgcaccagcaccggcgggaacaccacactgccaccgtcttcgccgccggactgctgaagggcatccgccagg
ctgcggcgctgcgttttcgccagctcaccggtggcagtttcgaccctgatgagtggcgtgaagctccccggctgataaatcgtctggatgcggct
cctgtcgttctgtatcgtggtcagccggtcgccgtcccagccgtaccaggtcacttgcggtttccgtgacagcgacatccagcccgtcaggtccc
gttcacgccgccacacccgttttgccaccctgcggcccagcgggtcgtaaagatagcgactttcgaccagcggctcttcatattgtgtccgcgtg
tagtgcaccagccggtgctgactgtcgtaatggtaccggtgagtccgctcatcatccgtgcggataaccccttccgggatgaggtcggttttctct
gtcagcctgccgtgacggtcataccggtaaagatagtgcgcgtcacgggcgatacggttatccggccacatgctgagggtgctgtccgggtgc
agctccgggtcgggcaggcggttacctgccgggtctgtggcatacgggatgcggatatccagattcgctgcggtggtgtgaacgccggtcagc
ctgccggtggtgctgtagctgtaactccgggtctggcgcgggctgctgatgcggatgagttcgccgttgtcgttccaggtgtaatcgcggtcagac
agcaggctgttcaggtgctggctctgtaactgcccggcaggggtataagcggtggtgagttcataacggccgaagctgcgcagcgtttcccgg
tgcaggcggtcgcgggtgtactccaccagcggtgtgtcgccgagtttcatgcctgccaggtaaccgctgccgtaggtcagccattccacggcg
ggcaggctgtccggtatacagcggttcgccagcccctgcgcgttgtacgcatgtctggtctcatgctgccagagcagtgcttccgtctgcgggtg
atgcaccgtctgacgctcaccggtcagccggcctttctcatcatacctgtaatgcaccgccacccggtgcccttcgctgatatggctgatgtctgtc
agccagccacgttcgtcatactgccaccgctctgcggtttcacccttcacggtgcggtgcgtgaggcggtctgcttcgtcatagtgccagtgggtg
accagaccctcatcctcgctgcggataagtttgccggtcaggtcgtggtgataacgctgtgtgcggccgtcaaagccggtttcctgtatcagccg
gtcaagtacatcgtaacggaaggtggtgtggctgccgttttcactggtcaggcggatgacccgtccggcagcatcgtattccatactgcgcgtca
gcccgccctgcgtggtacggacggcctttccccacgcatcgtactgtgtcccgtttctgctgccgtccggggcaatgacggcggtcaggtcacc
ggcgatgttgtattcataccgcgtttcatggccctgcgtgtctttcacggcaattaactgtccacggctgtcgtatgcgcggtactgactcagcccttc
ctcgcggtgcaccgccgtcatctgcccgaagcggtcatggtcataacgggttacataaccggaacagtcggtgaagctcagcaactgaccgt
aacggctccacgtcatggttttccggctgccggtggcatcttccgttgcgcagggtaagtcactgtgtggattatcataacggtagcgggtgatat
cgccgtcaggggcagtttcctgaatcagacggcccaattcatcatattcccggcgcaattccagcccgtcaggcccggtggctgacgttaactg
gttgtggtggttatagtaaaacgccgatgccctgccatccggcgtggttatgcgcgtgatgaggcccgtcaccacatccgggctgtactctgttgt
cctgcctgcggcatccgtctgtgccctgagcctgcccacggcgtcaaactgactctgcgtgacgctgccgtccgcgtgttcctttttcaccacccg
cttcagcccggcttcgccctgcgtgtgcagcacttcacggcggtccaggctgtcggtgatggtgatgcggtctttctcatactgatacgtgtagctt
aagcctgccgggtttagctgttctgtcacccgcccgtcgctgtcgtaacggtaacggatttccggccggcccgtgtgacggtgcgccaccatcc
ggccccggtatttatcatcgtaagtaaagctgcgcacctgtttgccactacggtcatacaccaccgccagttcgccgcgtggcgtccagccata
gcgcaccagcggcgcggcaggtaaattctccgggtattccgggtcgtgcgtcagccacacggcagacagacggatgccgttgtcccggcca
tattcggtgtaacccggcagggtatcaggaaaagcggacggttccgtcccgccggaaatggcctgctgccgggcttcttctgcccgctgcgcct
gcgtggtcagtaccagccggaagtgacgccaggcaccatccgtcacgccggtgatttcgccgctgaattcaccggcggcttcgcggtggaac
gtctgtgtgcgcccgaagcggtccaccagcccggtcagtacccggtacggcggcagcggcgcaggcagcacctcatccgcttccggcacc
cgctcacaccaaccgagcagccaccacggcccctgcggactgtttgtcgccagataacgatgcggacttaagcggagttcttccggcagcg
cctgccagagtgcggccagccggtgaccttcatccagtttcgccacgccgccgcgcaccagccacagtgactcgctgcggctgtaaccgtcc
tcaccgggaaacaggtgctcaaaatacaggcttctgccgccgttatcactgagtatcagtgtgttatcgcgcagctgtaagcggatatccgcag
gcattttccagccggggccgaggctccccaccggcgcgggcgtttttgtccggtaactgctgtaggtgcgggagaggatgaacggcagcggg
ccgggcagggcgatgtcggtttcaccgggaaggacctttgcaccgagcaggggattgaccggatggccggacgtcactccgccggggcac
accgaacaggccacgccggtgggggcaccaatacgtaccccggctgaaccctgaacaatgctaccgccatactgcgtcatgtcaccctgac
gcgccgccggttttccgctcatcgtaaaatccttttctattattttaatgacagtatcttcatacgaaaaagcgaagaaaaacttgtggtcacaatga
attgaagatttttaggaatatatttagaacgttacaaaagagaaggttcggatagggtatttagagaggaatcaggtgtgctagtggccctttgttg
atcgttcaccaaaggacccaataatagactcaatcttccttcggcaagcactgcaaatgcccgtgccgcacgccgcgctgggcgataacgtc
atcggcaaaatgctgcacgtcacccatgtcacctttcaacacggcgatttccagacagtcgtcgtggttgatgtgcacatgcagcgtggcgacg
gagaggtcgtggtgatgatgctgggtggagacaatgcggctggctaagtcgcgtttttcgtgttcatacacatacgacagcaccgcgaaacctt
gcgtgccgtgctgctgggtggcctcttgcgccagggcgctacgcagaatgtcgcggatagcttcggaacggttgttataaccacgacgctggct
caggctgtccagcgtctccagtaaatcgtcatcaagcgtgatggtgactcgttgcatttgcgttaaacctttttctgtggtgcgacggcgcacgggg
aatgcgggtaataccgcgttttgtagcacacgtccggcgtcagaggaaaaggttaatttctctcccaccacctgggtttcgacgatttgtccgttgt
ccataaccattacccgctggcaaaagcgttctaccaggcgtaagtcgtgggtgatgaacaggcaggcggtgccaaactgttgttgtagctttttc
agcaggcgaatgacacccgcctgtaacacgagatcaaggttagaaacggcttcatccagaatcagtagtttcggttcgaccgccagcgcgc
gagccaggcagacgcgctggagctggccgccgcttaactgcggtgggcgtttgtcgagaacgctgtcatcgagatcgaccgccttcagcattt
cgctggcgcgcgccagttgttcggatttttttcagtgatagcaggtggcgcatcggttcacgcaggatctcgcgcacggttttgcgcggattcacgg
cgctgatggagtcctgaaataccatctgaatatcgcggcggaacgctttacgctgggcgcgattgagtttcgccagcggttcgccacgccagct
aatattcccctgcgcgggcgattctaaacccaccagcaaccgcgcgagggtacttttcccgcagccgctgcgccccagcagggcgacggttt
cgccgcttttcagggtcagggaaacgttattcagcaccgcctgatgttgatgttttccgttaaatccaccgtgcgcatagtgatgggaaaggccg

gagatgttaagtaaagtcatgatgccagctccataccgtagagggcgagatgagcggaaaccaggctgcgcgtcactgtatgtttgggggcg
ttaaacagcgtttctacatcgccctgttcgacaatcttaccgtcagacatcaccgccacgtcatccgccagacgcgccacaacgcccatatcat
gggtcaccagcagcattcccggcgcttgtttttgcataatgctttccagcagatcgaggatgcgcgcctgtgctaccacgtcaaggtcggtggtc
ggttcatcggcgatgataaacggtgattcacacagcaccgccatcgcaatcatcatgcgctgcaacatgccgccgctcatctcgaacgggtac
agcttcagcacgcgcgcggcgttttccagccccaccgcttctatggcagcggtaagcgtagcgtcatcggcgggtttccctaacgccaggcag
gtttcccgcgcgtgggtgtgcatggtgtgcagtggattaaaggcgctgcgcgggttctgcatgatggtggcaattttgatgccgcgcagggcgca
aggcgaaaccggtttgccatcggctaaaatttcccccgccgtctggcgaacgccagcgggcaaaatgcccagcgttgcggcgcaggttaat
gatttcccgctgccgctaccgccgactaacgccagcacgcgcccgcgttgcagggttaacgatacaccgtgtaccagcggctgcgcggcctg
tagcgcgatattacgtagttcaatctgttgcggcattagtgtgcgtgctccgtcaccagatgagggtccagatgatcgcgcagtgcgtcacccac
caggttaaaggccatcacgctgataaacagcgccagccccggccagaacatttgcagcggctgggtccagatatactggcgcgcgtcgtta
atcatcacgccccattcggcggtcggcgcggtcacaccgaggccaaggaaagacatccccgcgacgtgcagcatcatatggccgatatcc
agcgttgccagcaccagcagcgaagggatcaccgcgcctgccagatgatcgacaaacacccgcacatggcccgcaccggaaagccgtg
acgccagcacaaactcgcgttggcgtagtgaaatcaccaggctgcgcaccatgcgtgcataccacgcccagtgcgacagggcgatggcga
taattacgttggtcagcccggtgccgagcacgccaaccataaagaacgacagaatcgaggtcgggaaggtcataaacatatcggcgacgc
gcatggtggcctgatcaacgcgcccgccaatcaacccggcgctgccgccaataaccagccctaatgtcagcaccagcagcaggcaggcc
attaccgaaccgagcgacacgcgggtcgctgccatcagccgcgagaaaatatcgcgacctaagtgatcggtgcccagccagtgctgcgcat
ccggcgaaagcaggcgcgacggcaaatcaatcgcctgtgggtcatacggcagccaccactggctggtgagcgcaatcagcgccagcag
ggcgataatgatcagcgccaggcgtaccgaccagcgggaagagaggaaaaagttcacgcgtgcgctccttcatgacgacgaatgcgcgg
gtccagcgcggcgttgagcaaatcgacaatcaaattacagaccacaaaaaccaccaccatcatcagcgtaaagcactggatcaccggata
gtcacggttaaaaatcgccgacaccgcatagcgcccgacgcccggccaggcaaagatgttttcgataatcatcgtcccgccaatcagttcgc
cgatatgcatccccacggcggtgatcatcggcagcgaggcattgcgcaggatgtggcgacgttcggtctgtttgtcgttcaggccgcgcagac
gcgcccaggtgacgtgacgctgaccggcgacgtccagcatactggcgcgcagtaaacgcgcgttaatcgccagcgacataaaggcaatg
gaaaccgcaggcaaaatgatgtgctgccagccgccgtaacccatcgcgggtagccattgcagatacaccgaaaacgccatcaccagtaa
aaacgccagccagaagttaggcatcgacacgccaagaaacgcgatgaaacgtacggcgaaatccggcagacggtcgcgatggcgcgc
cgcccagataccgagcggtacggaagtgagcagaattaataccagcgccgcacctgcaagttccagcgtggcgggcaggaagttcagcat
atcgtccagtaccgggcgttggctggcgaatgagataccaaagtcaagatgcagcgccttccacaaccaggtgccgtactggacgtacagc
ggctgatccagtcccagcatggtgcgggtagaggccagcatctccggcgtcggcggcaggttagacagacgcaaataatcgagcgccggg
tcgccggtaccgaggcgcagcatcagaaaaatgatcaccgaggcggcgagcaccatcgggatcagcagcagaaagcggcgtaatacgt
aacgcaacattaaggtttcaccggtttaatctgttcgaacggaatttcggtggcgatcggcgcgtaggggatgttacccagctccggttttgatac
caccatcattgagatgtaactgataggcagataaaccgcctcgtcatgcagacgggtcagaatgtcgcgatacagcgcctgacgttgcgtttcg
tcatgggtcgccagcacttcgccgatctctttatcaatcagcggtttgtcggctaatccttgctgtgcctggaagtcagcgtgtgacggtacgcgca
ttgaactgaggaaggcgtgtggatcatatggcgcgccccaggtgcggtggaaaatcatgccaaaacgaccgtcgcgctgacgagcatagat
actgctctcttcttcgccaatcagcgagacatctgcgccaatctggcgcatatcagcctgaatgatttccgccatcgatttgcttaacgcatcggtg
ccgatgaacgaaagttcaatgcgcagcggctgaccatttttctcgcggatgtctttgcccgcaggcagcgtccaaccggctttttccagcaacgc
tttcgctttttgcggatcgtactggctcggtttcaggccgaggttggcgtagggcacagaaggggcaaacagggtgtcggcgacctgctgggtg
ccatacaacgcgttatcaatcagcgatttttgtttaccgcgtaattaagagcttcacgtactgccagctcgttggtgggggctttggcggtattgag
cgccagcatcacggtttcgatcggctgtgacagttgggtgtggtaagccggattctggctaaagcgggcgaaggtatcgagcggtaataaccc
ttcgtttccgtacagcaggtcgatatcgccagtttcaaacgccaccgcgcgggtagtcgggtccgggatgacgttaaaggtgatctttttaatcgct
ggcttttcgccccagtagttttcgttacggacgaagacatcgtactgattcagtttcgattcctgcaaaatccacggtccggtgccaatcggcgcttt
aattccgttcatggtttcatggtttttaaactgcgagggagcgataaaacggaaaggacggggcagggccagttcttgcaggaaaggatagta
ggcgcttttcagggtaatttgcagctctgttttactgagtgctttaacatcaacaatctggtttgccagctccagccaggcgtgacgttgacggttatc
gagcactgcgcggaagttttctgccgccgcctcggcatcgaacggttcaccgttggagaatttcacgtcatcacgcagggtgaaggtccaggtt
ttaccatcttctgaatgagtccagctttttgccagccacgggatcaccgaaccgtctgcctgatatttcaccaatggttcataaaccatgctctgggc
gaacatctggttaggcgtgtaaaggtgcgggtttagtggcccgacattcaccggccaggcggtggtgatttcatctggtgcagcggcatggacg
ataaaagacgcacaagccagcagcgcaaatagagtgcggcggagtgtggagagcatggtaaccccaatggattaaaatagatggcgga
aataagtatgacgattttaagtattcgtcatactgattacctgttctggatcaatagatgggcagtcgggtgggcggatcagttaactgaatcgatc
cattgcacactgtcggcggtgagcgtaaagggagtaggggtgcagatcgcaaggcttaaattttcaagctgacaatggctgactgacagcga
ggagtgataggtgctgtctacgctgacgatttgccaggcgctgccgccacgctgtttaacgatggcttctttgcgcgtccagatgcgccagaaca
tttccagttgctgatcaggatgcacggcgtccatctcagcgtgttccccgaggctgaatacggcgttcgccagccagcgccagttggcgcgcgg
gcgaatcacttcgatatcgcagccgacttcgccttcatcactcaacagcagggcgatatcgtcaccgctatggcttaagttgaaccatagcggc

atttccggcgcaaatgcaggtttgccttgttcgccatagatgatctccggtagcggggaaagcgtgtgcgaaagcaatgcacgccccgccagc
cagcgttcgcgtcgtggaccttgcggtgcttgctcgcgtaaacccggtggcagtggagctgcgcttaaggtcgaaactttccccagaactatcc
gatacatatcagggccaacgtttaatggaaaatgaaagtgcgtatcgtatcacttgtcgcctcatcccggtaaccgactttcggtctgcccggcc
ccagtaaaatcgccagtttgctaccgcctttggtggtttccatccagattttacacacgctggttaatggcaccgaaagtagcatccctaccgggc
cgagcagccatccccaaattaacaatgaaagaaataccaccatggtggacatccccaggcgatggcccatcatccgtggttctaaaatattg
ccgatgaccatatggaccactaaaaacaatgcgccgaccagaatacattcgtaaacaccattaaacagcagcacctgaatcattggcggta
cggcggaaattaccgcgccgatattgggcacgtagttgagcaaaaacgccagtactgcccacatcagcgcaaactgcacccccatcagctc
cagccccagccagacgatgacacctgtccataaactgagtagcgtcttcaatgcaagatagtgcgaaacgcctttaagtgcgcggtgtaatcc
cgcgatgtgaatctgtggattattcagcgcaaaacgcattttgtaagggacgtggcgcacttcaaacagcataaaaactacggtcatcaccag
caaaagcacgctcgccattgccccggaaagcccggtcattagcgctgtggtgaaggtaaccactttttccgagtccatccgctgcagcattcgc
tccggcgacatatgcaaattaagaaaaggcaacatctcctgcaatttaaaaagtttgcgcgtcagctccttattaaacttcggcagcatagagat
aaattcgttaaacgatgccgccagtacgccgaccagcgcggttagtgcgatcagcatcaccaccactacaatcgtaatggcaacggggcgtt
gtactccccgacgaataaaccaggtgacgagcgggttgaggacgatggcaaaaaacagcgccagtaacagctgcacaatgatatctgcc
gctgcgtgaatgcccgcgaggatcactaccagcgaggccagcttgagcagaatgtgcatgcccgttttatcgggttgaggggtttccattgggg
cttccttgtgactttttgtattaagtgtagcgggagtcacgccagcaatattctgattcttactgctgattttcacgtcagcacgtggtaaaaatgaaac
actgttgtaaaaatgtggtgatcctcatgcccgaacccgtagccgaacccgcgctaaacggattgcgcctgaatttgcgcattgtctctatagtca
tgtttaacttcgccagctacctcaccatcgggttgccgctcgctgtattaccgggctatgtccatgatgtgatgggctttagcgccttctgggcagga
ttggttatcagcctgcaatatttcgccaccttgctgagccgccctcatgccggacgttacgccgattcgctgggacccaaaaagattgtcgtcttcg
gtttatgcggctgctttttgagcggtctggggtatctgacggcaggattaaccgccagtctgcctgtcatcagcctgttattactttgcctggggcgcg
tcatccttgggattgggcaaagttttgccggaacgggatcgaccctatggggcgttggcgtggttggctcgctgcatatcgggcgggtgatttcgt
ggaacggcattgtcacttacggggcgatggcgatgggtgcgccgttaggcgtcgtgttttatcactggggcggcttgcaggcgttagcgttaatc
attatgggcgtggcgctggtggccattttgttggcgatcccgcgtccgacggtaaaagccagtaaaggcaaaccgctgccgtttcgcgcggtgc
ttgggcgcgtctggctgtacggtatggcgctggcactggcttccgccggatttggcgtcatcgccacctttatcacgctgttttatgacgctaaaggt
tgggacggtgcggctttcgcgctgacgctgtttagctgtgcgtttgtcggtacgcgtttgttattccctaacggcattaaccgtatcggtggcttaaac
gtagcgatgatttgctttagcgttgagataatcggcctgctactggttggcgtggcgactatgccgtggatggcgaaaatcggcgtcttactggcg
ggggccgggttttcgctggtgttcccggcattgggtgtagtggcggtaaaagcggttccgcagcaaaatcaggggggcggcgctggcaacttac
accgtatttatggatttatcgcttggcgtgactggaccactggctgggctggtgatgagctgggcgggcgtaccggtgatttatctggcggcggcg
ggactggtcgcaatcgcgttattactgacgtggcgattaaaaaaacggcctccggaacacgtccctgaggccgcctcatcatcttaaaacttac
tgaataaccagcgtattaatgatgttttctgcggtggtctgcgctttttgctgatcgtcagcgggcagcgtaatttgcatggtcagcagttgattaccc
acgttacccagaataacggaagagtacgccgtctggcctttcgcggagataatactgtctaactgctgcattttgtgacctttcagctcaatggcttt
attggttaccacttgcagctgcggatcgcggctacgttgctgatcttccagacgcttcgccagcaccgccagatcttctttcggatcatcgcccatg
atgacgatgactgctttctgcccggtggcgtcggaccagacatgcatgttattggcctgcgttcccagcttaccgctctggtcggtcatatccgctg
gcagcgagaaacttaacttgccatcaagcaggttgacgggattcccggtagcgttactttccgcgaccgaaccctgcgccgtagcgttagtgtc
tttatcatcacaggccgcaagccccataaccagcaggccaattccgacatatttaaccagattgcgcattgacttcttcctttcgataaacggcca
taacggctcattcatccatcttatcacaactctgataacgaacctttaactcgcctgcaaagcgttgatttcggatttatctgccagtcttttcaacag
catattgagtaatacgccatacattggcaggaagaaaacgatactgattaacactttgaaacagtaatcgaccagcgcgatttccatccagtgtt
cagccataaaggcatccgggctacgccagaaggcaatgaagaaaaaggccagcgtgtcgctgacgttaccgaacagtgtggacgctgtc
ggtgccagccaccagcggcgactctgacgcaggcggttaaaaacgtgcacgtcgaggatttgccccagcgcgtaggccatgaaactggcg
gtggcgatacgggcgacaaacaggttgaagtgggcgagtgcgccgaatccctgccaggaacccatatagaatagcgacgagatgacgta
ggagattaataacgcagggatcattaccgcgaagataatgcgtcggccagcggtgcgccaaaaatacgcacggtcaggtcggtagcaag
aaaaataaacggaaagctaaacgcgccccaggtggtatggaaacccaaaatggagacgggaagctgaaccagatagttactggaggtg
atcaccagcagatgaaataacgataaccagaacaacgccttatagcgttgagtttgcgagaaaacgttcatattgtaccttttttgattaaccattg
gggtgagggaacccaatacgtacgacacgtctgttaccttaacataataacgtgcttaccctttttttcgagccgccgcatgatactgctttacgttg
acaatgcaatggttaattttcacgcaatcgttaacctagtttgcttacggactcacagggcgtaaactagcgccgtttttttaagtgatgagaagaa
aatgaccgatctcttttccagccctgaccacacactcgacgcgcttggcctgcgctgcccggaaccggtgatgatggtgcgcaaaaccgtgcg
caatatgcagcctggcgaaacgttgctgattatcgccgacgatccggccactacccgcgatattcctgggttttgtacctttatggaacacgaact
ggttgctaaagagacggatggactgccttatcgttatttgattcgtaaaggcggttgataggggctgattggcttcgatgccgccttttcccctcacc
ctaaccctctccccagaggggcgaggggaccgatcgcgctcaatgttgcgatcggtttgccttatctcctgcgcaacaatcttaacgcattcgct
gtcaccagcaccgtcgcccccgtatctgccagcactgccagccacaacccggtcatccctaacagcgtggtgacgaggaagatccctttcag
ccccagcgcaatagtgatgttctggcggatattggcgtgagtggcgcgtgccagttcaatcatttgcaccaggccgcgcaggtggttatgggtta

atgctgcgtcggcggtttccagcgccacgtctgtgccgctacccattgcaatcccgatggcggcagctttcatcgctggcgcgtcgttaataccgt
caccgaccatcgccagcggcgcatgttgattcagctcggtcaccgctttgactttatcttccggcaacaggcccgctttaaactccagccccagc
tccccggcaattgccgccgctgcgcgtggattatcgccggtgaggatcacccctttgacgcccagcgcgttcagttcactgatggcagttgcag
catcggcgcgcagggtatcctgtaacgcaatgacaccaagcacgtcatcgttacgtactaccagcactaccgtttgcccggcgctttccagttc
gttaattaaaccagtaaatgcatcagcgggatgtttcccggcagcgcaaatcaatacgcgctcaccgttaacctgcgcttcaatgccagaccc
gaccagcgcccgctgtgattcggcggtgggaatggcgagttcagcaacctgtgcttcgcgtacgatggcttgcgccagtggatgcgtcgcgcc
ttgctcgaccgccgccgccagtgtcagcagttcagattcactaatacccgttgccggatgaatcgcggtaacgcgcggtttaccgacggtcagc
gtaccggttttatcaaacgccacctgagtaacacgacccagctgttccagcgccgctccgcctttaatcaacgccccacgacgcgctgccgcc
gccagcccggaggtaatcgccgcaggcgttgagataactaacgcacacgggcagccaatcagcagcagcgtcagccctttataaatccact
cctgccagctggcggcaaacagcagcggtggcaccagcgtcaccagcagagcgacggccataatcgcgggcgtatagatacggctgaa
acggtcgataaaccgctcaatgggagcgcgacgctcttcggcttcttcgatcagtttcagaatccggtcaatggcgctggctcccggttctgaca
gcacttccaacgtcaccagacggtctacgctggtggcaccagcagggactttatcgcccgttgcgcgctccaccggaatggattcgccggtca
gggcgctttcatcaaaactggcaaacggtgagagcagtttaccgtcggcaggcaaacgcccacctgcggcgacttcaatcacatcgccagg
gcgcaggctgttaatcgccacctcttcccgctcacccttacgcaggcgcgtggcggtttctggtttcagcgccattaacgcgctaacgccctgac
gcgcgcggctggcggcccagccttccagtcgttcaccaatcaaaaacagcagcaacaccatcgcagcttcagccgttgcgccaataaaca
gtgcaccaatagcggctacgctcattaaggtttcaatggcgaagtagctgccggatttgatcaaccgtaatgcctgacgagcaatcgggtaca
gcccaaccagcgtggtcgcgataaacgccagttgcccgaacggatgattgaactgctccagaccccagctgattgccatcatcacgattagc
gtaatcagcggcagattctctttcaggcgtgatgcttgcggttcttcggcggcctgttcatcgcgcagggaatagcctgctttttgcagcgcagattc
aacttgtgcacgaatgtcattgtcggcatcgaccaccagtttttcggtggcgaacaacacctgcacctgattcacgcctgcaagctggcgcacg
gcattttctaccttgcgcgcacaggcggcgcagtccatgccgctgactttccagctatagcgggtgccggagacgttttcagagagagttggcgt
gctggaacatgcgccgtcgcagcaacagtcgttggcgttctgtaccgtggttagcggtttgaacgcagcaaattgaggggctttcttgccgtgatt
gtcaggagtcgacatggcatcctccggttaagtttttctcattaaccgaaggatacactctggagtcgactccagagtcaagttttatcagagata
cagcgagcggacgatcaggaagtgaccggcaaagtagcaggccgcagcaatcgcgttatccgcacggaagcgtcggcgatagtggctcc
ccagccagacaaagttactgataaacagcaacgaagcgccaacaaacgcagagagcgccggagcggtcggacggaagaaccacagtt
cacctgccagccacaccatcaccagcgtcatgccgataaacgtgcagataggccaacggtactcttccaggcgcgtccagataatcgccag
taacagcgcacccagaaccagcagcaccagtggtagaggccagaagaaagagagcgtcatctgactggcgaaatagatggtgtacagc
aggtgcgagaggaaaaacgcgccgatggcgtacatcagacgttgacgtggcaacagggttagcgcatcgcccagcagtgaggcgcaca
gccctgccagcaccagatagctaatggcgtcgaacatcggcgcttgccaggccagtaacagcaggagaagaagggttaacggtttaaaca
cccagcgttgccaggttggcccacgatacgatgcatccacagatagccatgcggaaagacagacagcgataaacgaccaaagcatcttag
ttccttgaattcgttatttttcgtaagcggaatgcctacggtcttgcttccagtttagtggcgagggtggacggatgacaacaccataactggcagg
gtatgcttatttccgcattttccgatgagggataaagatgagcaaaccacctcttttctttattgttatcattggcttaattgtcgtcgccgcatcgtttcgt
tttatgcaacagcgacgggaaaaagctgataatgatatggctccgctccagcaaaagctggtggtggtgagcaacaagcgggaaaaaccg
attaacgatcgccgttcgcgccagcaggaagtgactccggcaggcaccagtatacgctatgaggcaagcttcaaaccgcaaagcggagga
atggagcagacgtttcgcctcgacgcccagcagtaccacgccctgacagtgggcgataaaggtacgctgagctataaaggaacgcgctttgt
cagctttgtaggcgaacaataaggttatttttaactttaaatttcttctgccaggccagcagttcaaacacgccgaaaaggaaaatccgtaccttt
cggcggtggtcatctgcgggccatctttcggtaaagtggatttcaatagcgccagctgcataccatgcatcagcacggtaaaaatcagcgcca
cgttaacgaagatattcagcgggcgtgggaagggatgcaccaggttgaggattaaaaatccccaaacgcagagcattaacaaacgaccaa
tattaatcagcatcactttctccttgtgcttcgcgttgatacagccgataagccacctgacccgccactttttcccgatgtaatgaccagtttgctgga
acagtgggcagaccgttttcgacttcgctttcgacataaatcagggcttcgtcagccagccagccgttatcttccagtaaatttatcgtctcttctaa
caagccacggcggaacggtggatcgacaaacacgatattatgcggtgtacctttttgcgccaggaatgacatcgcgttgctgttcaccacgcgt
gcattgcctgcttttagtgtcgccagattcttaattaactgctgagaaaccgcgcgatccatctcaatcaacgttgcccccgcagcgtagcgcgat
aacgcttccagccccagcgcgccgctcccggcgaagcaatccagacattgggcgtcaacaatgaccggagccagccagttaaacaacgtt
tcgcgtacgcggtcggtggtggggcgcagacctgggctatcaggaaccgggagtttacggcctcgccactgcccgccaataatgcggatttg
gccgctgccggaatgattcggtttttcatgataaattgctcaatccgccagataacacatacttgcaggcggtgatgtgaattaagttaagtgata
gactatttcatcatttttttagctgctatgtacatagcgttaacgctgtgccatgaagcaacagcgaggagtgtagtcgcaaatggcgaaagaaa
aaaaacgtggcttttttcctggctgggctttggtcaaaaagagcagaccccggaaaaagagacagaagttcagaatgaacaaccggttgta
gaagaaatcgttcaggcgcaagagcctgtgaaggcctctgaacaagccgttgaagagcagccgcaagcgcatactgaagccgaggcgg
aaacttttgctgccgacgttgtggaagtcactgaacaggttgctgaaagtgaaaaagcgcagcctgaagcggaagtcgttgcacagccggaa
ccggtcgtagaagaaacgccggagccagtggctatcgaacgtgaagagctgccgttgccggaagacgtcaacgccgaagcggtttcgcca
gaagagtggcaggctgaagcggaaaccgtagagattgtcgaagcggcggaagaagaagcggctaaagaagaaattaccgacgaaga

gctggaaacggcgctggctgccgaagcggcagaagaggcggtgatggtggttcctccggcagaagaagagcagccggtggaagaaatc
gctcaggagcaggaaaaaccgaccaaagaaggtttttcgcgcgcctgaaacgcagcctgttaaaaaccaaagaaaatctcggttccggat
ttatcagcctgttccgcggtaaaaaaatcgacgatgatctgtttgaggagctggaagagcagcttttgatcgccgatgtgggtgtggaaaccac
acgtaaaattatcaccaatctgacggaaggcgcatcccgcaagcagcttcgtgacgccgaggcgctctatggcctgctgaaagaagagatg
ggcgagattctggcgaaagtcgatgagccgctgaatgttgaaggcaaagcgccgtttgtgatcctgatggtgggcgtcaacggtgtgggtaaa
accacgacgattggtaagctggcgcgtcagtttgagcagcagggtaaatcggtgatgctggcggcgggtgatactttccgtgcagctgcggttg
aacagcttcaggtctggggtcagcgcaacaatattccggtgattgcccagcataccggggcggattccgcctctgttatcttcgacgccattcag
gcagctaaagcgcgtaatatcgacgtcctgattgccgatacagccggacgcctgcagaacaaatcgcacctgatggaagagttgaagaaa
atcgtccgcgtgatgaagaaactcgacgttgaagcgccgcatgaagttatgctgactattgatgccagcaccgggcagaacgcggtaagcc
aggccaaactgttccatgaagccgttggcttaaccggcatcacgctaacgaaactggacggcacggcgaaaggcggggtaattttctcggtg
gctgaccagtttggtatccctatccgctacattggtgtcggcgaacgtattgaggatttgcgtccgtttaaggcggacgactttatagaggcactttttt
gcccgagaggattaacaatgattcgctttgaacatgtcagcaaggcttatctcggtgggagacaggcgctgcagggcgttacgttccatatgca
gccgggtgagatggcgtttctgaccggtcattccggcgcagggaaaagtaccctcctgaagctgatctgtgggattgagcggcccagcgccg
ggaaaatctggtttagcggccatgacatcacgcgtctgaaaaaccgtgaagttccgtttctgcgccgccagattggcatgattttccaggatcac
catctactgatggaccgtactgtctacgataacgtggcgatcccgctgattatcgccggtgccagcggtgacgatattcgtcgccgggtgtcggc
ggcgctggataaagtcgggctactggacaaagcgaagaacttccctattcagctttcgggcggtgaacaacagcgtgttggcattgcccgcgc
ggtggtgaacaagcccgcggtactgctggcggacgaaccgactggtaacctggacgacgcgctgtcggaaggcattttacgtctgtttgaag
agtttaaccgcgttggggtaaccgtattgatggcaacgcacgacatcaacctgatctcgcggcgttcctatcgcatgctcaccctgagcgatggt
cacttgcatggaggcgtgggccatgaataagcgcgatgcaatcaatcatattcggcagtttggcgggcgtcttgatcgcttccgtaaatcggtcg
gcggctcaggcgacggcggtcgtaacgcaccaaaacgcgcgaaatcctcgccaaaaccggtaaatcgcaaaaccaacgttttcaacgaa
caggtgcgctatgccttccacggcgcattgcaggatctgaaaagcaaaccgttcgccacgtttttaacggtgatggttatcgccatttctctgacg
ctgcccagcgtctgttatatggtgtacaaaaacgttaaccaggcggcgacgcagtattatccgtcaccgcaaatcactgtttatctgcaaaaaac
gctggacgatgacgctgctgcgggcgtggtggcacagttgcaggccgagcaaggcgtggagaaagtgaactatctttctcgtgaagacgca
ctgggtgagttccgtaactggtctggttttggtggtgcgctggatatgctggaagaaaacccgcttccggcagtggcggtggtgatcccgaaact
cgatttccaggggacggaatcactgaatacgctgcgtgatcgtatcacgcagattaacggcattgacgaagtgcggatggatgacagctggttt
gcccgtctggcggcgttgaccgggctggtcgggcgcgtttcggcgatgatcggcgtgttgatggtggcggccgtgttcctcgtcatcggtaacag
tgtgcgtctgagtatctttgctcgccgtgactccattaacgtacagaaactgattggtgcgacagatggattcatcctgcgcccgttcctgtatggtg
gcgcactgctgggattttctggcgcattgttgtcattaattttgtcagaaattctggtgctgcgattgtcatcggcggttgcggaagtggcacaggtttt
cggaacgaagtttgatatcaatggcttatcattcgatgaatgcctgctattgctgctggtatgctcgatgattggctgggtggcagcgtggcttgcca
cggtacaacatttacgccactttacgcctgaataataaaagcgtgttatactctttccctgcaatgggttccgtagcagggaaagagaccccgttg
tctcttcccggtatttcatctctatgtcacattttgtgcgtaatttattcacaagcttgcattgaacttgtggataaaatcacggtctgataaaacagtga
atgataacctcgttgctcttaagctctggcacagttgttgctaccactgaagcgccagaagatatcgattgagaggatttgaatgactgacaaaat
gcaaagtttagctttagccccagttggcaacctggattcctacatccgggcagctaacgcgtggccgatgttgtcggctgacgaggagcgggc
gctggctgaaaagctgcattaccatggcgatctggaagcagctaaaacgctgatcctgtctcacctgcggtttgttgttcatattgctcgtaattatg
cgggctatggcctgccacaggcggatttgattcaggaaggtaacatcggcctgatgaaagcagtgcgccgtttcaacccggaagtgggtgtg
cgcctggtctccttcgccgttcactggatcaaagcagagatccacgaatacgttctgcgtaactggcgtatcgtcaaagttgcgaccaccaaag
cgcagcgcaaactgttcttcaacctgcgtaaaaccaagcagcgtctgggctggtttaaccaggatgaagtcgaaatggtggcccgtgaactg
ggcgtaaccagcaaagacgtacgtgagatggaatcacgtatggcggcacaggacatgacctttgacctgtcttccgacgacgattccgacag
ccagccgatggctccggtgctctatctgcaggataaatcatctaactttgccgacggcattgaagatgataactgggaagagcaggcggcaa
accgtctgaccgacgcgatgcagggtctggacgaacgcagccaggacatcatccgtgcgcgctggctggacgaagacaacaagtccacg
ttgcaggaactggctgaccgttacggcgtttccgctgagcgtgtacgccagctggaaaagaacgcgatgaaaaaattgcgtgctgccattgaa
gcgtaatttccgctattaagcagagaaccctggatgagagtccggggtttttgtttttgggcctctgtaataatcaatttcccctccggcaaaacgc
caatccccacgcagattgttaataaactgtcaaaatagctattccaatatcataaaaatcgggtatgttttagcagagtatgctgctaaagcacgg
gtagtcatgcataaaacgaaataaagtgctgaaaaacaacatcacaacacacgtaataaccagaagaatggggattctcaggatgaacat
aaagggtaaagcgttactggcaggatgtatcgcgctggcattcagcaatatggctctggcagaagatattaaagtcgcggtcgtgggcgcaat
gtccggtccggttgcgcagtacggtgaccaggagtttaccggcgcagagcaggcggttgcggatatcaacgctaaaggcggcattaaaggc
aacaaactgcaaatcgtaaaatatgacgatgcctgtgacccgaaacaggcggttgcggtggcgaacaaagtcgttaacgacggcattaaat
atgtgattggtcacctctgttcttcatcaacgcagcctgcgtctgacatctacgaagacgaaggcattttaatgatcaccccagcggcaaccgcg
ccggagctgaccgcccgtggctatcagctgatcctgcgcaccaccggcctggactccgaccaggggccgacggcggcgaaatatattcttg
agaaagtgaaaccgcagcgtattgctatcgttcacgacaaacagcaatacggcgaaggtctggcgcgagcggtgcaggacggcctgaag

aaaggcaatgcaaacgtggtgttctttgatggcatcaccgccggggaaaaagatttctcaacgctggtggcgcgtctgaaaaaagagaatat
cgacttcgtttactacggcggttatcacccggaaatggggcaaatcctgcgtcaggcacgcgcggcagggctgaaaactcagtttatggggcc
ggaaggtgtggctaacgtttcgctgtctaacattgcgggcgaatcagcggaagggctgctggtgaccaagccgaagaactacgatcaggttc
cggcgaacaaacccattgttgacgcgatcaaagcgaaaaaacaggacccaagtggcgcattcgtttggaccacctacgccgcgctgcaat
ctttgcaggcgggcctgaatcagtctgacgatccggctgaaatcgccaaatacctgaaagcgaactccgtggataccgtaatgggaccgctg
acctgggatgagaaaggcgatctgaaaggctttgagttcggcgtatttgactggcacgccaacggcacggcgaccgatgcgaagtaatcatt
aatcggcaactttgggttgccgccaaattgctaatatcgagtacgttgcttcatgccggatgcggcgtaaacgccttatccggcctacaagatcc
aaagaaatcagtaaattgcaacacacattgtaggcctgataagcgtagcgcatcaggcaatatcacttttgaaatcaaacttgacgattaacac
ttctcccagccgccctgttgtgccgtaaaccccagcgcctgcataaacgccgtcatcacaccgcgatcttccacgcctgcatccgccatccacc
agcatgaaacgccaggattgttacgcaaaacctcttccagcagatattgccccacaccgcgacggcgggtgacttcccgcacgcgcaggga
atccagtgctccctcggtgccgcttaaggttacccgcacggcagcgagcaggcgctcgttaaaacgcgcggcgtagatacggtggttatcgtc
aacctgtaacgaggaaggggaatactccggccagatcttttgcaggtcaatccggtcttggtcgctaaatttttctaatcgaatgatggtcagcttc
atgggtaacccgtgtaaatcacaaaagtggaaccagtgtagcgaaataatttaatcggaggctttctctttttttatttcttttggcaggtgattaatttttt
aacagcaataattacaaaattaaaacattagagaatgaaaaatgtccagcataatcccctgaatgatagtgaattattccgccctttgtgccgtt
attttatgctgacaaaggcactttttttctgtttatctatcaataaattcagaatattatctgttcttaatcgactgaaaaatagggattttaatcgctattatc
acaaaatactgcgctaaccccttaatcagacaggcaaaaacagtgcagtataaaaaaagaacagtctgatttgttaacacataaaaacaaa
gcaacacaacatcacgaatggggatttttgactatgaaacggaatgcgaaaactatcatcgcagggatgattgcactggcaatttcacacacc
gctatggctgacgatattaaagtcgccgttgtcggcgcgatgtccggcccgattgcccagtggggcgatatggaatttaacggcgcgcgtcag
gcaattaaagacattaatgccaaaggggggaattaagggcgataaactggttggcgtggaatatgacgacgcatgcgacccgaaacaagcc
gttgcggtcgccaacaaaatcgttaatgacggcattaaatacgttattggtcatctgtgttcttcttctacccagcctgcgtcagatatctatgaaga
cgaaggtattctgatgatctcgccgggagcgaccaacccggagctgacccaacgcggttatcaacacattatgcgtactgccgggctggact
cttcccaggggccaacggcggcaaaatacattcttgagacggtgaagccccagcgcatcgccatcattcacgacaaacaacagtatggcg
aagggctggcgcgttcggtgcaggacgggctgaaagcggctaacgccaacgtcgtcttcttcgacggtattaccgccggggagaaagatttc
tccgcgctgatcgcccgcctgaaaaaagaaaacatcgacttcgtttactacggcggttactacccggaaatggggcagatgctgcgccaggc
ccgttccgttggcctgaaaacccagtttatggggccggaaggtgtgggtaatgcgtcgttgtcgaacattgccggtgatgccgccgaaggcatg
ttggtcactatgccaaaacgctatgaccaggatccggcaaaccagggcatcgttgatgcgctgaaagcagacaagaaagatccgtccgggc
cttatgtctggatcacctacgcggcggtgcaatctctggcgactgcccttgagcgtaccggcagcgatgagccgctggcgctggtgaaagattt
aaaagctaacggtgcaaacaccgtgattgggccgctgaactgggatgaaaaaggcgatcttaagggatttgattttggtgtcttccagtggcac
gccgacggttcatccacggcagccaagtgatcatcccaccgcccgtaaaatgcgggcgggtttagaaaggttaccttatgtctgagcagtttttg
tatttcttgcagcagatgtttaacggcgtcacgctgggcagtacctacgcgctgatagccatcggctacaccatggtttacggcattatcggcatg
atcaacttcgcccacggcgaggtttatatgattggcagctacgtctcatttatgatcatcgccgcgctgatgatgatgggcattgataccggctggc
tgctggtagctgcgggattcgtcggcgcaatcgtcattgccagcgcctacggctggagtatcgaacgggtggcttaccgcccggtgcgtaactc
taagcgcctgattgcactcatctctgcaatcggtatgtccatcttcctgcaaaactacgtcagcctgaccgaaggttcgcgcgacgtggcgctgc
cgagcctgtttaacggtcagtgggtggtggggcatagcgaaaacttctctgcctctattaccaccatgcaggcggtgatctggattgttaccttcct
cgccatgctggcgctgacgattttcattcgctattcccgcatgggtcgcgcgtgtcgtgcctgcgcggaagatctgaaaatggcgagtctgcttgg
cattaacaccgaccgggtgattgcgctgacctttgtgattggcgcggcgatggcggcggtggcgggtgtgctgctcggtcagttctacggcgtca
ttaacccctacatcggctttatggccgggatgaaagcctttaccgcggcggtgctcggtgggattggcagcattccgggagcgatgattggcgg
cctgattctggggattgcggaggcgctctcttctgcctatctgagtacggaatataaagatgtggtctcattcgccctgctgattctggtgctgctggt
gatgccgaccggtattctgggtcgcccggaggtagagaaagtatgaaaccgatgcatattgcaatggcgctgctctctgccgcgatgttctttgt
gctggcgggcgtctttatgggcgtgcaactggagctggatggcaccaaactggtggtcgacacggcttcggatgtccgttggcagtgggtgttta
tcggcacggcggtggtctttttcttccagcttttgcgaccggctttccagaaagggttgaaaagcgtttccggaccgaagtttattctgcccgccatt
gatggctccacggtgaagcagaaactgttcctcgtggcgctgttggtgcttgcggtggcgtggccgtttatggtttcacgcgggacggtggatatt
gccaccctgaccatgatctacattatcctcggtctcgggctgaacgtggttgttggtctttctggtctgctggtgctggggtacggcggtttttacgcc
atcggcgcttacacttttgcgctgctcaatcactattacggcttgggcttctggacctgcctgccgattgctggattaatggcagcggcggcgggct
tcctgctcggttttccggtgctgcgtttgcgcggtgactatctggcgatcgttaccctcggtttcggcgaaattgtgcgcatattgctgctcaataaca
ccgaaattaccggcggcccgaacggaatcagtcagatcccgaaaccgacactcttcggactcgagttcagccgtaccgctcgtgaaggcgg
ctgggacacgttcagtaatttctttggcctgaaatacgatccctccgatcgtgtcatcttcctctacctggtggcgttgctgctggtggtgctaagcct
gtttgtcattaaccgcctgctgcggatgccgctggggcgtgcgtgggaagcgttgcgtgaagatgaaatcgcctgccgttcgctgggcttaagcc
cgcgtcgtatcaagctgactgcctttaccataagtgccgcgtttgccggttttgccggaacgctgtttgcggcgcgtcagggctttgtcagcccgg
aatccttcacctttgccgaatcggcgtttgtgctggcgatagtggtgctcggcggtatgggctcgcaatttgcggtgattctggcggcaattttgctg

gtggtgtcgcgcgagttgatgcgtgatttcaacgaatacagcatgttaatgctcggtggtttgatggtgctgatgatgatctggcgtccgcagggct
tgctgcccatgacgcgcccgcaactgaagctgaaaaacggcgcagcgaaaggagagcaggcatgagtcagccattattatctgttaacggc
ctgatgatgcgcttcggcggcctgctggcggtgaacaacgtcaatcttgaactgtacccgcaggagatcgtctcgttaatcggccctaacggtg
ccggaaaaaccacggtttttaactgtctgaccggattctacaaacccaccggcggcaccattttactgcgcgatcagcacctggaaggtttacc
ggggcagcaaattgcccgcatgggcgtggtgcgcaccttccagcatgtgcgtctgttccgtgaaatgacggtaattgaaaacctgctggtggc
gcagcatcagcaactgaaaaccgggctgttctctggcctgttgaaaacgccatccttccgtcgcgcccagagcgaagcgctcgaccgcgcc
gcgacctggcttgagcgcattggtttgctggaacacgccaaccgtcaggcgagtaacctggcctatggtgaccagcgccgtcttgagattgcc
cgctgcatggtgacgcagccggagattttaatgctcgacgaacctgcggcaggtcttaacccgaaagagacgaaagagctggatgagctga
ttgccgaactgcgcaatcatcacaacaccactatcttgttgattgaacacgatatgaagctggtgatgggaatttcggaccgaatttacgtggtca
atcaggggacgccgctggcaaacggtacgccggagcagatccgtaataacccggacgtgatccgtgcctatttaggtgaggcataagatgg
aaaaagtcatgttgtcctttgacaaagtcagcgcccactacggcaaaatccaggcgctgcatgaggtgagcctgcatatcaatcagggcgag
attgtcacgctgattggcgcgaacggggcggggaaaaccaccttgctcggcacgttatgcggcgatccgcgtgccaccagcgggcgaattgt
gtttgatgataaagacattaccgactggcagacagcgaaaatcatgcgcgaagcggtggcgattgtcccggaagggcgtcgcgtcttctcgc
ggatgacggtggaagagaacctggcgatgggcggtttttttgctgaacgcgaccagttccaggagcgcataaagtgggtgtatgagctgtttcc
acgtctgcatgagcgccgtattcagcgggcgggcaccatgtccggcggtgaacagcagatgctggcgattggtcgtgcgctgatgagcaacc
cgcgtttgctactgcttgatgagccatcgctcggtcttgcgccgattatcatccagcaaattttcgacaccatcgagcagctgcgcgagcagggg
atgactatctttctcgtcgagcagaacgccaaccaggcgctaaagctggcggatcgcggctacgtgctggaaaacggccatgtagtgctttcc
gatactggtgatgcgctgctggcgaatgaagcggtgagaagtgcgtatttaggcgggtaataacacgttgattgatagggagtcaaaagactc
ctttgagacaggtgacaaatgtaaaattgcctgatgcgctgcgcttatcaggcctactgggtgagtggcaatatgttgaatttgcacgatcttgtag
gcctgataaggcgtttacgccgcatccggcatgaaacgatgagcaatctgtagagtttgattcagaccttctatattttcccgcttatccgtgcccc
atctcccattttccctcacccacgccgtcaccgccttgtcatctttctgacaccttactatcttacaaatgtaacaaaaaagttatttttctgtaattcga
gcatgtcatgttaccccgcgagcataaaacgcgtgaattcgcgcattcggtacaacaagagagataaacgatgaaaccgttacattatacag
cttcagcactggcgctcggactggcgttaatggggaatgcacaggcagtgacgaccattccgttctggcattctatggaaggggaactgggta
aagaggtggattctctggcccaacgttttaacgccgaaaacccggattacaaaattgtaccgacctataaaggcaactacgaacagaatttaa
gcgcggggattgccgcatttcgtaccggcaacgcgccggctattttgcaggtttatgaagttggcaccgccaccatgatggcgtcgaaagccat
taaaccggtgtatgacgtgtttaaagaggcagggattcagttcgatgagtcgcagtttgtgccgacggtttcaggttactactccgacagcaaaa
cgggccacttactctcccagccattcaacagctcgacccccgttctctattacaacaaagacgccttcaagaaagcaggattagacccggaa
cagccgccgaaaacctggcaggatctggcggactatgccgcgaaactgaaagcctccggcatgaagtgcggctacgccagcggctggca
gggctggatccaactggaaaactttagcgcctggaacggtctgccgtttgccagcaaaaacaacggctttgacggcacggacgcggtgctgg
agttcaataagccggagcaggtgaaacacatcgccatgctcgaggagatgaacaagaagggcgacttcagctacgtcggtcgtaaggatg
aatccaccgagaagttctataacggtgattgcgcgatgaccaccgcctcttccggttctcttgccaacattcgcgagtacgccaaatttaactacg
gcgtaggcatgatgccttacgacgccgatgcgaaagatgcgccacaaaacgccattatcggcggagccagcctgtgggtgatgcagggta
aagataaagaaacgtataccggtgtggcgaagttcctcgatttcctcgcgaagccagaaaacgctgccgagtggcatcagaaaaccggttat
ctgccaatcaccaaagcagcgtatgacctgacccgtgagcagggcttttatgagaaaaacccaggggcggataccgcgacgcgtcagatg
ctgaataagccgccgttgccgttcaccaaagggctgcgtctgggcaacatgccgcagatccgcgtgattgtggatgaagagctggagagcgt
gtggaccggtaagaagacaccacagcaggcactggataccgccgttgagcgtggaaatcagttgctgcgccgctttgagaaatcgacgaa
gtcttaatcagtgtaatgtcggatgcgtttcgcttatctgacctggcatcgcgtgtaggccggataagcgaagcgcatccggcacagttcaggaa
ttaaccgtaatgtcatcatcccgtccggtgttccgctcgcgctggctgccttatctgctggtcgcgccgcagctcatcatcaccgttatctttttatctg
gcctgcgggcgaagcgttgtggtactcgctacaaagcgtcgatccgtttggtttctccagccagtttgtcggcctggataacttcgtcacgctgttc
catgacagctactatctcgactccttctggacgacgataaaattcagcacctttgtcaccgtcagcggtttgctggtatcgctgttctttgcggcgct
ggtggagtacatcgtgcgcggcagccgtttctatcaaaccttaatgttgctgccttacgccgtggctcccgccgttgccgccgtattgtggatcttcc
tgtttaaccccggtcgcgggctgatcacccattttctcgcggagttcggctacgactggaaccacgcgcaaaacagcggtcaggcaatgtttct
ggtggtgtttgcctcagtatggaagcaaatcagctacaacttcctgttcttctatgccgcgctgcaatccattccccgttcgttgatcgaagccgca
gccatcgacggtgcagggccgattcgccgcttctttaagattgcgctgccgcttatcgccccggtgagtttcttcctgctggtagtgaacctggtgt
atgccttcttcgacaccttcccggtgatcgacgccgccacgtccggcgggccggttcaggccaccacgacgctgatttataagatctaccgcga
aggttttaccggactggatctggcttcgtctgccgcacagtcggtggtgttgatgttcctcgtcatcgtgctgacggtggtgcagttccgctatgttga
aagcaaggtgcgttaccaatgattgagaaccgtccgtggctgacgatattcagccataccatgctgatcctcgggatcgcggtgatcctcttccc
gctgtacgtggcgtttgtcgcggcgacgctggataaacaggccgtctatgccgcgccgatgacgctcatccccggcacacatctgctggaaaa
catccacaacatctgggtgaacggggtaggcacgaatagcgcgccgttctggcggatgttgcttaacagctttgtgatggcgttcagcattacg
ctcggcaaaattaccgtctcgatgctctcggcatttgccattgtctggtttcgttttccgctacgtaacctcttcttctggatgatttttatcaccctgatgct

gccggttgaagtacgtatcttcccgacggtggaagtcatcgccaacctgcagatgctcgacagctacgccggtttaacgctgccgctgatggcc
tcggcgaccgctactttcctgttccgccagttctttatgacgctgccggatgagctggtggaagccgcgcggatcgacggcgcatcgccaatgc
gcttcttttgcgacatcgtttttccgctctccaaaactaatctggcggcgctgtttgtgatcacctttatctacggctggaatcagtatttgtggccgttgtt
gattattaccgatgtggatctcggcaccaccgtggcagggatcaaagggatgatcgctacaggcgaaggcaccacggaatggaactcagtg
atggtggcgatgttgttaacgcttatccctccggtggtgattgttttagtgatgcagcgtgccttcgtgcgcggcctggtcgatagtgagaaataag
atggcaggactgaaattacaggcagtaaccaaaagctgggatggtaaaacccaggtcattaaaccgctgacccttgatgtggcggatggcg
aatttatcgtgatggtcgggccgtctggctgcgggaaatcgacgctgctgcgcatggttgccgggctggagcgggtgacagaaggcgatatct
ggatcaacgaccagcgcgtgactgaaatggagccaaaagatcgcgggattgcgatggtgttccagaactacgcgctttatccgcatatgagt
gtcgaagaaaacatggcgtgggggctgaaaattcgcggcatggcaagcagcaaattgccgagcgcgttaaagaagcggcgcgcattctg
gagctggacggtctgctcaaacgtcgcccgcgcgagctttccggcggtcagcgccagcgtgtggcgatgggccgcgcgattgtgcgcgatcc
ggcggtgttcctgtttgatgagccgctctctaacctcgatgccaagctgcgcgtgcagatgcgtcttgaactgcaacagttgcaccgtcgcctga
aaacgacttcactctacgttactcacgatcaggttgaagcgatgacgctcgcccagcgagtaatggtgatgaacggcggtgttgccgaacag
attggcacaccagttgaagtctacgaaaagcccgccagcctgtttgtagcgagttttatcggcagtccggcgatgaacctgctgacaggccgc
gtgaataacgaaggcacgcatttcgaactggacggcggtattgagctgccgctaaacggtggctaccgtcagtatgccgggcgtaaaatgac
tctcggcattcgcccggaacatattgcgctaagctcgcaggcagaaggcggcgtaccgatggtgatggacacgctggagatcctcggcgca
gataacctggcgcacggacgctggggcgagcagaagctggtggtgcgactggcgcatcaggagcgcccgacggcaggcagcacgctgt
ggctgcatctggcggaaaatcagctgcatctttttgatggtgaaacaggacaacgagtatgagtaactggccttatccccgcatcgtcgctcatc
gtggcggcggtaagctggccccggaaaacaccctggcgtcaatcgacgtcggggcaaaatacggtcataagatgatcgaatttgacgcga
agttatcgaaagatggcgagatcttcctgctccatgacgacaatctcgaacgtaccagcaacggctggggcgtcgcgggtgaactgaactgg
caggatttactgcgcgtggatgcgggcagttggtacagcaaaatgtttaaaggtgagccgctaccgttgctttcgcaggtggcggaacgttgcc
gcgaacacgggatgatggcgaatatcgaaatcaaacccaccaccggcaccgggccattaacgggcaaaatggtggcgctggcggcacg
cgagctgtgggccggtatgacgccgccgctgctgtcatcgtttgagattgatgctttagaagctgcacaacaggcggcaccggaactgccgcg
cggtttgttgctggatgagtggcgcgacgactggcgcgaactgaccgcgcggctgggctgcgtctctattcatctcaatcataagttactcaataa
agcgcgagtgatgcagttgaaagacgccggactgcggattctggtttataccgtcaataaaccccagcgcgcagcagagttgctgcgctggg
gtgtggattgcatctgtaccgatgcgattgacgtgattggtccgaactttacggcccaatagttttcaacggaatgtcaggctgcggcgtgctggg
ctgattcagcatgtcgccgtttctcctctccggcagcatatgctgctgattcaacgaactatccggattacggttgctgtttaacatcccgccgttggt
gttgggcagcatttgctgccgggcgggatttcgctcccccggctgtgactgcaacacccgctgagaattattgtttatctggttttctaaatgctgctg
ttgcaactgagtttgcgttttcagttgctgattcagcatccctttttgctggatttgctgagtctgcatctgggtttgcatccgctgctggctgggtatctga
taccccggctggttagggttgttcagagtattaatgggctgtgcaaagccgacaaacggcaggagtgccgtaagaatcagaagtcgtttcatcg
cgtatcctcctctgaagatatcctttaagtttactcgcttcccgacaaaacgatgattaattcagagttatataccaggcttagctggggttgcccctt
aatctctggagaataacgatgataaaaccgacgtttttacgccgggtggccattgctgctctgctctcaggaagttgttttagcgccgccgccgcg
cctcctgcgccgcccgtctcgtatggtgtggaggaagatgtcttccacccggtacgcgcgaaacagggaatggtagcgtctgtggacgccact
gccactcaggtgggggtggatattctcaaggagggcgggaatgccgttgatgccgccgtggcggtgggctacgcgctggcggtaacgcatc
cgcaggcagggaatctgggcggtggtggttttatgttaatccgctcgaaaaatggcaataccacggctatcgatttccgcgaaatggcacccg
ccaaagcgacccgcgatatgttcctcgatgatcagggcaacccggacagcaaaaaatcactcacttcgcatctggcttccggcacaccgggt
acggtagcaggtttctcgctggcgctggataaatacggcaccatgccgctgaacaaagtcgtgcagcccgcgtttaaactggcacgcgatggt
tttatcgttaacgacgcgctggctgacgatctcaaaacctacggtagcgaagtgttgccgaatcacgaaaacagtaaagctatcttctggaaag
agggcgagccgctgaaaaagggcgacacgctggtgcaggcgaacctggcaaagagcctggagatgattgctgaaaacggcccggacg
aattctataaaggcacgattgcggaacagatcgcccaggagatgcagaaaaacggtggcttgatcactaaagaagatttagcagcctataa
agcggtcgaacgcactccgataagcggcgattatcgcgggtatcaggtttactccatgccaccgccatcctccggcgggatccatatcgtaca
aatcctcaatattctggaaaacttcgatatgaagaaatacggctttggcagcgccgatgcgatgcaaatcatggcagaagcggagaaatacg
cctacgccgaccgctcggaatatcttggcgacccggattttgtcaaagtaccgtggcaggcgctgaccaataaagcctatgccaaatctattgc
cgatcaaattgatatcaataaagcgaagccatccagcgaaattcgccccggcaagcttgcgccttatgagagtaatcaaactacccattactc
agtggtggataaagatggtaacgcggtggcggtgacctatacgctgaacaccaccttcggtacgggcattgtcgcgggcgagagcggtattct
gcttaataaccagatggatgatttctccgccaaaccgggcgtaccgaacgtttacgggctggtgggcggtgatgccaacgccgtcgggccga
acaaacgcccgctgtcgtcgatgtcgccgaccattgtggtgaaagacggtaaaacctggctggttaccggtagcccaggcggtagccggatc
atcactacagtgctgcaaatggtggtgaatagcatcgattatggcttgaacgtcgccgaagcgaccaatgcgccgcgtttccaccatcagtggtt
gccggacgagctgcgtgtcgaaaaagggtttagcccggatacgctcaagctgctggaagcaaaaggtcagaaagtggcgctgaaagagg
cgatgggcagtacacaaagcattatggttgggccggacggtgagttgtacggcgcatccgacccgcgctcggtggatgatttaacggcgggg
tactaaggttagcggccctcttcgtgggaagagggctattttgtcagggcaagccgaaggtagcctttttatttcgtaatcctgtagatattcttcca

gcggttttgccgttcccagagaatgaatttcaccactgtctttatcaataataaaaggtgcgttaccagctaagcgcgcggcctcatctccagtttc
gagaaattctcgtgcttcgaaacagaaataccagccctggctaaagcgtccatgtagagtaatgacgaccgggagatctgcatcatcaaggt
aatggttcgctttcgcgaatgcgtcgtgataagtaatcataattataataaatattattgttgagtgttatattattattcctgtgtgatacattgagcaaa
gacgcgttcatcttcgtcaaagatattattaaatgctggttttgaaaaaagttcatcggataagatatagtcacgaaataagtatctgctaatattta
tgatttctttttcagaacgtcgaacgggattttcttatttaagatatcagaatcttatgattcgggtaaaattctaaataaatcaacatgtcattaaaatc
atcatagccattagctatttcggctaaaatagagactacatgtcttcggtccatctcacttaaggagtgtagttccgttgtaagtttttccatagcttgc
actgctaaatttcgaacaaggaattttctgctggtaatctctaaaaagatggcatggtttacaatgatttttgtttcctttgattattatgaacaactgtc
catgatttcgtttaagaatgaagagaaatcactaaacgaactgaatatattttctgtgccaatattatctctaatttcaaaaaagttacttttaatgtcg
gtaatgactccaacttattgatagtgttttatgttcagataatgcccgatgactttgtcatgcagctccaccgattttgagaacgacagcgacttccgt
cccagccgtgccaggtgctgcctcagattcaggttatgccgctcaattcgctgcgtatatcgcttgctgattacgtgcagctttcccttcaggcggg
attcatacagcggccagccatccgtcatccatatcaccacgtcaaagggtgacagcaggctcataagacgccccagcgtcgccatagtgcgt
tcaccgaatacgtgcgcaacaaccgtcttccggagactgtcatacgcgtaaaacagccagcgctggcgcgatttagccccgacatagcccc
actgttcgtccatttccgcgcagacgatgacgtcactgcccggctgtatgcgcgaggttaccgactgcggcctgagtttttttaagtgacgtaaaat
cgtgttgaggccaacgcccataatgcgggctgttgcccggcatccaacgccattcatggccatatcaatgattttctggtgcgtaccgggttgag
aagcggtgtaagtgaactgcagttgccatgttttacggcagtgagagcagagatagcgctgatgtccggcggtgcttttgccgttacgcaccac
cccgtcagtagctgaacaggagggacagctgatagaaacagaagccactggagcacctcaaaaacaccatcatacactaaatcagtaag
ttggcagcatcacctttaatgtcataagtaaatattgaaatgctataatccccgatcactaaccgttcttgtagatcagggtttatgaaatcatcattat
ttctataaaattcatttacggcaaaaaggtttttaactgaaggttctggaatcgagaggctaaataatcgtaatccattaacttcgaatccatccatt
gctcttaaaaaagtaagataatcaggttgattcttaaaaagaatactaacttcattacataacttctccaggcaattagttgaattagaattaaattct
atttggattatactttcaataaatgattccttgagcggtgcttcaaatggatagtctaaatcattcatcatttttattaatttatttatttgggttttagtcatca
ttttgtgtggtccataatatttttcatgggaggataaattgagccagatggaatggcccagggagtgattttgctttcacctactaatattcccttagct
attcgtgattgcatgttagtaaaaactttatgatatggttcgttttcgattaatactaaattttcgaaatcattagtaccaccatcatctagagatagtttat
ggtgaacattataatttagaggaacattgccctttctcattcttaaaaggtcttcggcgttgaatcttccagatgcttcggaggtttttgcaagtttgata
agaaaatttttttcttacagttttattaaactcctttcgtaagttagctaagtcttttgaaggtctcttaatgtatattatttctattatgtctataccatcaagaa
gtttttgttgacctctaagtttgcctaagtaactatttatttctgtatatgtatcaacattagaacgctttgttaaaagaatagatgctgttgctatcgctatg
cccgctgtttttttcatttagtatttgatcatagccttttattgcatctccacccagtttttccgcagtatctctgaatccttcaatgttcccattgtatacgcca
ccagcagcaagtagcctgccaatagctttgctgtttaacgttttgaaacgattatttgtttggtttttttgaatttggtaaaaacaaacggttataattttca
gggagtgccaaatagctgaactcggtatttgcgataagatgttgacagaggataaattcataactaatagttattgtttctgtatccagttggttttca
atgatctggtgatgaatagccgataaaaaaagcgcctcttagttgtatataataatacttttccaatctaccaaatctatttattcgatagaagtcaaat
cccataaataattgttcattattgttaatggaattaataaataatggagtgcttttatcaattaatttacaaaatgttataccatggaactgtgaaccaa
gccccgtattattaatattatttaagagtgagaatgtaaatatttcatcctcatgcccgctctgccaacgattacctgtagactcagaagtcccacaa
cctgcggagatgcttccttgttgttctcccgttactgtcaggtaaacaatattactcatccctgaaaagtccttttatggcatgctattttatcaatggca
atattatgcttgttaattgttatttatttactccaggaaattgtaggcgtatatattaaaaaataaaatattacactttaggaagtaattatgagttaaatc
tcaatatatgtattgggcaaataaataccttaaaggaaggtatgtcgattttgaatgacacccttttttccatctcagaaaaagggcactatcttactt
cacccgcgccatataatatgcatcgacatattcaccattacgcaatgcgtacttcttaccagtcccttcaatttcaaagccgtatttttttatagacctta
attgccggggcgttatcgacaaacacggttagttcaatgcgatctacccgcaaccagttgtcgcacatttcaatcatctctcgcatcagggcgct
ggcgacgccgcggttcttccaacgagagtcgacacagataccaaaatcggcaacatgactgcggcgtgggcgctgttgcacgtcaatggtg
agatggcccacgacgtctccatcaatacaggcgacgagttgcttgatgccgggacgatcggcgagtcgctcctgccacatatgatcggaagg
atgaggcacctgtagtgtgttgcaatacacctccggctgggcgtgaatctgcctgatggcctcgtaatcccgtgtttctgcgtggcgtattactatct
cactcattcctttgtcctctttggggtaaatgtccctttcaacatcattgactttcaaatgcgagtcaaatgcatttttttttgcaaaaagtgttggacaagt
gcgaatgagaatgattattattgtctcgcgatcaggaagaccctcgcggagaacctgaaagcacgacattgctcacattgcttccagtattactt
agccagccgggtgctggcttttttttttgatctttcgttctcaatttatccacgggagtgcttgtgttcgttatgcgcactccagtaggaaccacgtccgct
ttgcgctaaggtgtaaataaccaccttaaaaaggacgaaatcatggtcatcaactgcgcctttattggcttcggcaaaagcaccacccgttacc
atctgccgtatgtacttaaccgcaaggatagctggcatgtcgcgcatatttttcgtcgccatgcgaagccggaagaacaggctcccatttattccc
atatccatttcaccagcgatctcgacgaagtactaaacgatcccgatgttaagctggttgttgtctgcacccacgcggacagccatttcgagtac
gcgaaacgcgcgctggaagccgggaaaaatgtgctggtcgaaaaaccgttcactccgacacttgcgcaggcgaaagagctgtttgcgttgg
cgaaaagcaaagggctgaccgtcacgccgtatcagaatcgtcgctttgactcctgcttcctgacagcgaaaaaagcgattgaaagtggcaa
gttgggagagattgttgaagtggaaagccattttgactattaccgcccggtggcagaaaccaaacctgggctgccgcaggatggcgcgttttat
ggccttggtgtgcatacgatggaccagattatttctctgttcggtcgcccggatcacgtcgcttatgacatccgcagcctgcgtaataaagccaat
cctgacgacacctttgaagcgcaactgttttatggcgacctgaaagccatcgtcaaaaccagccatctggtgaaaatcgattatccgaaatttat

cgttcacggtaagaaaggttcgtttattaaatacggtatcgaccagcaggaaaccagcctgaaggctaatattatgccgggcgaaccgggatt
cgcagcggatgattcggtcggtgtgctggagtatgtcaatgacgagggcgtgacggtcagagaagagatgaagccggagatgggcgattac
gggcgcgtttatgatgcgttgtatcaaaccatcacccacggtgcgccaaattacgtcaaggaatctgaagttcttaccaatctggaaatccttga
acgcggatttgagcaagcctctccctccacagtaactctcgctaagtaagtttgatggcccctcgaatagttcaattttttgaacagaggggtcaa
ttttcaccctctatcatcccaggcagatcgggtccacactaagcccatcgaaatcattcagggggcgaatacaaatgatctacttacgcaaagc
aaatgaacgtggtcatgcaaatcatggctggctggactcctggcatactttctcttttgccaactattacgatccgaactttatgggcttctccgcgct
gcgcgtgattaacgacgacgtgattgaagcagggcagggcttcggcactcacccgcataaagatatggaaattttgacctacgtgctggaag
gtactgttgagcatcaggacagcatgggcaataaagagcaggttccggcgggtgagttccagattatgagtgctggtacgggtattcgtcactc
agagtacaacccaagcagcaccgagcgtctgcatctgtatcagatctggatcatgcctgaagaaaacggtattacgccgcgttatgaacagc
gtcgcttcgatgccgtacagggcaaacagctggtgctctcgccggatgcgcgagatggttcattgaaagtgcatcaggatatggaactgtacc
gctgggcgttgctgaaagatgagcagtcggtgcatcagattgccgctgaacgccgcgtctggatccaggtggtgaaaggcaatgtcaccatta
acggcgtgaaagcctcgaccagcgatggtctggcaatctgggatgagcaggcaatctccatccatgcggatagcgacagcgaagtgttact
gttcgatctgccgccggtttaaaactcaacggcatctttgaagcctgcctttttgcaggcttcctcgctcgctccggtcgcgtgctgatgtacgcttcc
ggggttttgccgcgtcgttagcacaatgcgatgcaattgatttagtgtatatctaataattgtaaacaagcactgttttccccgggaaggttctgatgt
gtccgtgttaaactaagagaatctatctcttttgtaccttcaggacgatgaaaaagaaaagacccgtacttcaggatgtggctgaccgtgtaggc
gtgaccaaaatgacggtcagccgtttttacgcaacccggagcaggtttccgtcgctctacgcggcaagattgccgcggctcttgatgaactgg
gctatattcccaatcgtgcgcccgatatcctctctaacgccaccagccgggcgattggcgtcctgttaccttctctcaccaaccaggttttcgcgga
agtattacgcggaatcgaaagcgtcaccgacgcgcacggttatcagaccatgctggcgcactacggttataaaccggaaatggagcaaga
acgcctcgaatccatgctctcctggaatatcgacggcctgatcctcaccgaacgtacccacacgccgcgcaccttaaagatgattgaagtggc
gggtattcccgtggtggaactgatggacagcaagtcgccatgccttgatatcgccgtcggttttgataactttgaagcagcacgccagatgacc
actgccattattgctcgcgggcatcgccacattgcctatctcggcgcacgtctcgacgaacgtactatcatcaaacagaagggatacgaacag
gcgatgctggatgcaggcctggtgccatatagcgtgatggttgagcaatcttcttcttactcttccggtattgaactgattcgccaggcgcggcgg
gaatatccgcagctggatggcgtgttctgtacgaatgatgacctggcggtcggcgcggcgtttgaatgtcagcgtctggggttaaaagttcctga
cgatatggcgattgccggtttccacggtcatgacattggtcaggtgatggagccacgacttgcgagcgtgctgacgccgcgtgagcggatggg
cagtattggcgctgaacgcctgctggcgcgtattcgtggcgaatctgtgacaccgaaaatgttagatttaggtttcaccttgtcaccgggcggatc
tatttaagcccacaaatttgaagtagctcacacttatacacttaaggcatggatggatattgcttctgatattgtccggctggacaatgttaccgata
acagttacccgtaacatttttaattcttgtattgtgggggcaccactttgagcacgactaaccatgatcaccacatttacgtcttgatgggcgtatcg
ggcagcggcaaatctgcggtcgccagtgaagtggcgcatcaacttcatgccgcgtttcttgatggcgatttcctccatccacggcgcaatatcg
aaaaaatggcgtctggcgaaccactgaatgacgacgatcgcaaaccgtggttgcaggcgctgaacgacgccgcgtttgctatgcagcgcac
taataaagtgtcgctgatcgtctgttctgcattgaaaaaacactatcgcgacttgctgcgtgaaggtaatccgaatctctctttcatctatttgaaagg
cgattttgatgtgattgaaagccgcctgaaagcgcgcaaaggccatttctttaaaacccaaatgttggtgacgcagtttgaaacgctgcaggag
ccgggtgcggacgaaaccgatgtactggtggtggatatcgatcaaccgctggaaggtgttgtggcaagcaccattgaggttattaaaaaagg
caaataagtagtgactacattaacgcttgttttaacagcagtagggtctgttttactgctgctgtttttagtcatgaaggcgcgtatgcacgctttcctg
gctttaatggtggtgtccatggggggctggcctttttctggtatgccgctcgataaaatcgcagcgacgatggaaaaagggatgggaggcaccct
cggcttcctggcggtggttgtcgccctgggagctatgtttggcaagatcttacatgaaaccggcgcagtcgatcagattgccgtcaaaatgctca
aatccttcggtcacagccgcgcgcattatgccatcggccttgcggggctggtctgtgcgctaccgctgttctttgaagtggcgatagttctgctgatt
agcgttgctttctcaatggcgcgccacaccggtacgaacctggtgaagctggtaatcccattatttgcaggcgtggcggcagcggcggcatttct
ggtgcctggaccagcgccgatgctgctggcatcgcagatgaatgccgactttggctggatgatcctgattggcctgtgtgcggcaattccggga
atgattattgccgggccgctgtggggtaacttcatcagccgttacgttgagctgcatattcctgacgacatcagcgaaccgcatctcggcgaagg
caaaatgccatctttcggattcagcctgtcgctgatcctgctgccgctggtgctggtagggctgaaaaccattgccgcgcgttttgtgccggaagg
atcaactgcttacgaatggtttgagtttatcggtcatccgtttaccgcgattctggttgcttgtctggtggcgatttacggtctggcaatgcgtcagggc
atgccaaaagacaaagtgatggagatttgcggtcacgcgctgcaaccggcggggatcattctgctggtgattggtgcgggcggcgtgttcaaa
caggtgctggttgactctggcgtaggtccggcactgggcgaagcgttaaccggcatgggcctgccgattgctatcacctgcttcgtgctggcag
ctgcagtgcgcatcattcagggttctgccaccgtagcctgtttaacggcggtaggactggtgatgccggttattgaacaactgaactactccggtg
cgcaaatggcggcgctgtcgatttgtatcgctggtggttcgattgttgtcagccacgttaacgacgccggtttctggttgttcggtaaatttaccggc
gcgaccgaagccgaaacgctgaaaacctggaccatgatggaaaccatcctcggcactgtcggtgccatcgttgggatgattgcgttccagct
gttgagttaagtttgttcgcccggtagttgtgacgctaccgggttcttttcgaaaaactctcctcgttaccccttcatccacattcgaatgccgtcgag
gaacatctgggttgccatcatcaccagaatcaatcccatcaggcgttcaagtgcgttcacccctttctcgcccagcagacgtaaaaatagcgaa
gactgtagcaggatgacaaaggtgccgccccaggccagcagcagagcaatcaccagatgccccatctgattcgggtactgatgagacaac
aacatcagcgtggcgagaatagtcggcccggcgactaacggaattgccaacggcacgataaatggctcttcacctgccggaagcccgctg

ctatttcctgaagcgctggggaaaatcattttaatggcgatcagaaacagaatgatgccgccagaaatggagacggtttctgctcgtaggctaa
gaaatgccagaattttctcacccgcaaacaggaacaccagcatcaccaggagagcaataagcaactctcgcaccatgattgcccgccgtctt
ttcggttcagtatgtttcagtacggacatgaaaataggtaggtttccgagcggatccataatcaggatcaataaaactgctgcagaaatgatttca
ttcataactcaaattccctgataattgccgcggactttctgcgtgctaacaaagcaggataagtcgcattactgatggcttcgctatcattgattaatt
tcacttgcgactttggctgctttttgtatggtgaaagatgtgccaagaggagaccggcacatttatacagcacacatctttgcaggaaaaaaacg
cttatgaaaaatgttggttttatcggctggcgcggtatggtcggctccgttctcatgcaacgcatggttgaagagcgcgacttcgacgccattcgcc
ctgtcttcttttctacttctcagcttggccaggctgcgccgtcttttggcggaaccactggcacacttcaggatgcctttgatctggaggcgctaaag
gccctcgatatcattgtgacctgtcagggcggcgattataccaacgaaatctatccaaagcttcgtgaaagcggatggcaaggttactggattg
acgcagcatcgtctctgcgcatgaaagatgacgccatcatcattcttgaccccgtcaatcaggacgtcattaccgacggattaaataatggcat
caggacttttgttggcggtaactgtaccgtaagcctgatgttgatgtcgttgggtggtttattcgccaatgatcttgttgattgggtgtccgttgcaacct
accaggccgcttccggcggtggtgcgcgacatatgcgtgagttattaacccagatgggccatctgtatggccatgtggcagatgaactcgcga
ccccgtcctctgctattctcgatatcgaacgcaaagtcacaaccttaacccgtagcggtgagctgccggtggataactttggcgtgccgctggcg
ggtagcctgattccgtggatcgacaaacagctcgataacggtcagagccgcgaagagtggaaagggcaggcggaaaccaacaagatcct
caacacatcttccgtaattccggtagatggtttatgtgtgcgtgtcggggcattgcgctgccacagccaggcattcactattaaattgaaaaaaga
tgtgtctattccgaccgtggaagaactgctggctgcgcacaatccgtgggcgaaagtcgttccgaacgatcgggaaatcactatgcgtgagct
aaccccagctgccgttaccggcacgctgaccacgccggtaggccgcctgcgtaagctgaatatgggaccagagttcctgtcagcctttaccgt
gggcgaccagctgctgtggggggccgcggagccgctgcgtcggatgcttcgtcaactggcgtaatctttattcattaaatctggggcgcgatgc
cgcccctgttagtgcgtaatacaggagtaagcgcagatgtttcatgatttaccgggagttaaatagagcattggctattctttaagggtggctgaat
acatgagtattcacagccttacctgaagtgaggacgacgcagagaggatgcacagagtgctgcgccgttcaggtcaaaaaaatgtcacaac
cagaagtcaaaaatccaattggatggggtgacacaataaaacaggaagacaagcatgtccgatcgtatcgatagagacgtgattaacgcg
ctaattgcaggccattttgcggatcctttttccgtactgggaatgcataaaaccaccgcgggactggaagtccgtgcccttttacccgacgctacc
gatgtgtgggtgattgaaccgaaaaccgggcgcaaactcgcaaaactggagtgtctcgactcacggggattctttagcggcgtcattccgcga
cgtaagaatttttccgctatcagttggctgttgtctggcatggtcagcaaaacctgattgatgatccttaccgttttggtccgctaatccaggaaatg
gatgcctggctattatctgaaggtactcacctgcgcccgtatgaaaccttaggcgcgcatgcagatactatggatggcgtcacaggtacgcgttt
ctctgtctgggctccaaacgcccgtcgggtctcggtggttgggcaattcaactactgggacggtcgccgtcacccgatgcgcctgcgtaaagag
agcggcatctgggaactgtttatccctggggcgcataacggtcagctctataaatacgagatgattgatgccaatggcaacttgcgtctgaagtc
cgacccttatgcctttgaagcgcaaatgcgcccggaaaccgcgtctcttatttgcgggctgccggaaaaggttgtacagactgaagagcgcaa
aaaagcgaatcagtttgatgcgccaatctctatttatgaagttcacctgggttcctggcgtcgccacaccgacaacaatttctggttgagctaccg
cgagctggccgatcaactggtgccttatgctaaatggatgggctttacccacctcgaactactgcccattaacgagcatcccttcgatggcagtt
ggggttatcagccaaccggcctgtatgcgccaacccgccgttttggtactcgcgacgacttccgttatttcattgatgccgcacacgcagctggtc
tgaacgtgattctcgactgggtgccaggccacttcccgactgatgactttgcgcttgccgaatttgatggcacgaacttgtatgaacacagcgatc
cgcgtgaaggctatcatcaggactggaacacgctgatctacaactatggtcgccgtgaagtcagtaacttcctcgtcggtaacgcgctttactgg
attgaacgttttggtattgatgcgctgcgcgtcgatgcggtggcgtcaatgatttatcgcgactacagccgtaaagaggggggagtggatcccga
acgaatttggcgggcgcgagaatcttgaagcgattgaattcttgcgtaataccaaccgtattcttggtgagcaggtttccggtgcggtgacaatg
gctgaggagtctaccgatttccctggcgtttctcgtccgcaggatatgggcggtctgggcttctggtacaagtggaacctcggctggatgcatgac
accctggactacatgaagctcgacccggtttatcgtcagtatcatcacgataaactgaccttcgggattctctacaactacactgaaaacttcgtc
ctgccgttgtcgcatgatgaagtggtccacggtaaaaaatcgattctcgaccgcatgccgggcgacgcatggcagaaattcgcgaacctgcg
cgcctactatggctggatgtgggcattcccgggcaagaaactactgttcatgggtaacgaatttgcccagggccgcgagtggaaccatgacgc
cagcctcgactggcatctgttggaaggcggcgataactggcaccacggtgtccagcgtctggtgcgcgatctgaacctcacctaccgccacc
ataaagcaatgcatgaactggattttgacccgtacggctttgaatggctggtggtggatgacaaagaacgctcggtgctgatctttgtgcgtcgc
gataaagagggtaacgaaatcatcgttgccagtaactttacgccggtaccgcgtcatgattatcgcttcggcataaaccagccgggcaaatgg
cgtgaaatcctcaataccgattccatgcactatcacggcagtaatgcaggcaatggcggcacggtacacagcgatgagattgccagccacg
gtcgtcagcattcactaagcctgacgctaccaccgctggccactatctggctggttcgggaggcagaatgacacaactcgccattggcaaacc
cgctcccctcggcgcgcattacgacggtcagggcgtcaacttcacacttttctccgctcatgccgagcgggtagaactgtgtgtctttgacgcca
atggccaggaacatcgctatgacttgccagggcacagtggcgacatttggcacggttatctgccggatgcgcgcccgggtttgcgttatggttat
cgcgttcatggcccctggcaacccgccgaggggcatcgctttaacccggcgaagttgttgattgatccttgcgcgcggcaaattgacggggag
tttaaagataacccgctgctgcacgccggtcataatgaacctgactatcgcgacaacgccgccattgcgccgaaatgcgtagtggtggttgatc
actatgactgggaagatgatgccccgccgcgcacgccgtggggcagcaccatcatttatgaagcccatgtcaaaggattaacgtacttgcac
ccggagatcccggtcgagatccgtggcacttataaagccctcgggcatccggtgatgatcaactatttgaaacaattgggcattaccgcgctgg
aactgctgccagtggcgcagtttgccagtgaaccacgtctgcaacgcatggggctaagtaactactggggttacaacccggtggcgatgtttgc

gctgcatccggcgtatgcctgctcgccagaaacggcgctggatgagtttcgcgatgcaatcaaagcactgcataaagcgggtatcgaagtcat
tcttgatatcgtgctcaaccatagtgcggaactggacctcgacggcccgttattctcgctgcgtgggatcgataaccgtagctattattggataag
agaagacggcgattatcacaactggaccggttgcggcaacacgctcaatttgagtcatccggcggtggtggattatgccagcgcctgcctgcg
ttattgggtagaaacctgccacgtcgatggtttccgctttgatctggcggcagtcatgggccgtacgccagagttccgtcaggatgcgccgttgttt
accgctatccagaactgcccggtgctctcgcaggtgaagttaattgctgaaccgtgggatatcgctcctggtggttatcaggtgggaaatttcccg
ccgctgtttgccgagtggaacgatcatttccgcgatgctgcccgtcgtttctggctacattatgatttgcctctgggggcgtttgccgggcgttttgctg
cctccagcgatgtttttaaacgtaatggtcgtctgccgagtgccgcgattaatctcgtcaccgcgcatgacggttttacgcttcgcgactgcgtttgc
ttcaaccataaacacaatgaagcaaacggagaagaaaatcgcgacgggaccaacaacaattacagtaacaatcatggtaaagaagggtt
aggcggttctcttgacctggttgaacggcggcgcgacagcattcacgccctgttaacaacgttgttgctctcccagggtacgccgatgttactgg
ccggtgacgaacatggtcacagccagcatggcaataacaatgcctactgtcaggataaccaattaacctggttggactggtcgcaggcaagc
agtggtttaaccgcatttaccgccgcgttaatccatctgcgcaagcgcattcccgctttggtggagaatcgctggtgggaagaaggcgacggca
atgtccgttggctaaatcgatatgctcaacctttaagcacggatgagtggcaaaacgggccgaaacagctgcaaattctgctctcggatcgcttt
ttgatcgcaattaacgccacgcttgaggtaacagagattgttttacctgctggggagtggcacgccattcccccattcgctggagaggataaccc
agtgattacggctgtctggcagggacctgcacacggattgtgtgtgttccagagatgataaaaaaggagttagtcatggttagtttagagaaga
acgatcacttaatgttggcgcgccagctgccattgaaatctgttgccctgatactggcgggaggacgtggtacccgcctgaaggatttaaccaat
aagcgagcaaaaccggccgtacacttcggcggtaagttccgcattatcgactttgcgctgtctaactgcatcaactccgggatccgtcgtatgg
gcgtgatcacccagtaccagtcccacactctggtgcagcacattcagcgcggctggtcattcttcaatgaagaaatgaacgagtttgtcgatctg
ctgccagcacagcagagaatgaaaggggaaaactggtatcgcggcaccgcagatgcggtcacccaaaacctcgacattatccgccgttat
aaagcggaatacgtggtgatcctggcgggcgaccatatctacaagcaagactactcgcgtatgcttatcgatcacgtcgaaaaaggcgcac
gttgcaccgttgcttgtatgccagtaccgattgaagaagcctccgcatttggcgttatggcggttgatgagaacgataaaattatcgaattcgttga
aaaacctgctaacccgccgtcaatgccgaacgatccgagcaaatctctggcgagtatgggtatctacgtctttgacgccgactatctgtatgaa
ctgctggaagaagacgatcgcgatgagaactccagccacgactttggcaaagatttgattcccaagatcaccgaagccggtctggcctatgc
gcacccgttcccgctctcttgcgtacaatccgacccggatgccgagccgtactggcgcgatgtgggtacgctggaagcttactggaaagcgaa
cctcgatctggcctctgtggtgccggaactggatatgtacgatcgcaattggccaattcgcacctacaatgaatcattaccgccagcgaaattcg
tgcaggatcgctccggtagccacgggatgacccttaactcactggtttccggcggttgtgtgatctccggttcggtggtggtgcagtccgttctgttc
tcgcgcgttcgcgtgaattcattctgcaacattgattccgccgtattgttaccggaagtatgggtaggtcgctcgtgccgtctgcgccgctgcgtcat
cgatcgtgcttgtgttattccggaaggcatggtgattggtgaaaacgcagaggaagatgcacgtcgtttctatcgttcagaagaaggcatcgtgc
tggtaacgcgcgaaatgctacggaagttagggcataaacaggagcgataatgcaggttttacatgtatgttcagagatgttcccgctgcttaaa
accggcggtctggctgatgttattggggcattacccgcagcacaaatcgcagacggcgttgacgctcgcgtactgttgcctgcatttcccgatatt
cgccgtggcgtgaccgatgcgcaggtagtatcccgtcgtgataccttcgccggacatatcacgctgttgttcggtcattacaacggggttggcatt
tacctgattgacgcgccgcatctctatgatcgtccgggaagcccgtatcacgataccaacttatttgcctataccgacaacgtattgcgttttgcgct
gctggggtgggttggggcagaaatggccagcgggcttgacccattctggcgtcctgatgtggtgcatgcgcacgactggcatgcaggccttgc
gcctgcgtatctggcggcgcgcgggcgtccggcgaagtcggtgtttactgtgcacaacctggcctatcaaggcatgttttatgcacatcacatga
atgacatccaattgccatggtcattctttaatattcatgggctggaattcaacggacaaatctctttcctgaaggccggtctgtactatgccgatcac
attacggcggtcagtccaacctacgctcgcgagatcaccgaaccgcagtttgcctacggtatggaaggtctgttgcaacagcgtcaccgtgaa
gggcgtctttccggcgtactgaacggcgtggacgagaaaatctggagtccagagacggacttactgttggcctcgcgttacacccgcgatacg
ttggaagataaagcggaaaataagcgccagttacaaatcgcaatggggcttaaggttgacgataaagtgccgctttttgcagtggtgagccgt
ctgaccagccagaaaggtctcgacctggtgctggaagccttaccgggtcttctggagcagggcgggcagctggcgctactcggcgcgggcg
atccggtgctgcaggaaggtttccttgcggcggcagcggaataccccggtcaggtgggcgttcagattggctatcacgaagcattttcgcatcg
cattatgggcggcgcggacgtcattctggtgcccagccgttttgaaccgtgcggcttaacgcaactttatggattgaagtacggtacgctgccgtt
agtgcggcgcaccggtgggcttgctgatacggtttctgactgttctcttgagaaccttgcagatggcgtcgccagtgggtttgtctttgaagatagta
atgcctggtcgctgttacgggctattcgacgtgcttttgtactgtggtcccgtccttcactgtggcggtttgtgcaacgtcaggctatggcaatggattt
tagctggcaggtcgcggcgaagtcgtaccgtgagctttactatcgcttgaaatagttttcaggaaacgcctatatgaatgctccgtttacatattcat
cgcccacgcttagcgtagaagctcttaagcactctatcgcttacaagctgatgtttacgattggaaaggacccggtcgtcgccaataaacatga
atggctgaacgcaacgttatttgctgtgcgcgatcgtctcgtggagcgctggttacgttcaaaccgtgcccagttgtcgcaagaaactcgtcaggt
ttactacctgtcgatggagtttttgattggccgtacgctctccaacgccatgttgtcgctaggaatttacgaagatgtacagggcgcactggaagc
gatggggttaaatctcgaagagctgattgatgaagaaaatgacccaggcctcggtaacggtggcctgggacgtctggcggcttgcttccttgatt
ctctggcgacgttagggttgccggggcgcggttacggcatccgctatgactacggtatgttcaagcagaacatcgttaacggtagccagaaag
agtcgccagactactggctggaatacggtaacccgtgggaattcaaacgccacaacacgcgctataaagtccgttttggcggtcgcattcagc
aggaaggtaaaaaaacgcgctggattgaaaccgaagagattctgggagtcgcttacgatcagataatccctggttacgacaccgacgcgac

caacacgctgcgtttgtggagtgcgcaagccagtagcgaaattaacctcggtaaattcaaccagggtgactacttcgcggcagtggaagata
aaaaccactccgagaacgtatctcgcgtactgtatccggatgactccacctactccgggcgtgagctgcgcctgcgtcaggaatacttcctggtt
tcctcgaccattcaggacattttaagccgccattatcagttgcataaaacctacgataacctggcggataaaatcgcgattcatctcaatgatacc
catccggtactgtcgattcctgagatgatgcgtctgctgatcgatgagcaccaatttagctgggacgacgcgtttgaggtgtgttgtcaggtcttctc
ctacactaaccacacgctgatgagcgaggcgctggaaacctggccggttgatatgctgggtaaaattctgccgcgtcacctgcagatcatcttt
gaaatcaacgactatttcctgaaaaccttgcaggaacagtatccgaacgataccgatctgctgggacgggcgtcgatcattgatgaatccaac
ggtcgtcgtgtgcgtatggcctggctggcggttgttgtgagccacaaagttaacggtgtatcggaactgcactctaatctgatggtgcaatcgttgtt
tgccgactttgcgaaaatcttcccgggtcgtttcaccaacgtcaccaacggtgtgacgccgcgtcgctggctggcggtagcgaacccatcgcttt
cagccgtgctggacgaacacctgggccgtaactggcgcaccgaccttagcctgcttaatgagctgcaacaacactgtgatttcccaatggtta
atcacgctgtgcatcaggcgaagctggagaacaaaaagcgtctggcagagtatatcgcccagcagctgaatgtggtggtgaatccaaaggc
gttgttcgatgtacaaatcaaacgtattcacgaatacaaacgtcaattgatgaatgtgttgcatgtgattacccgctataaccgcatcaaggccga
cccggatgcgaagtgggtaccgcgcgtgaatatttttggcggtaaggcggcttcggcctattacatggcgaagcacattattcatttgatcaatga
cgtagcgaaagtgatcaacaacgatccgcagattggcgataagctgaaagtcgtgttcatcccgaactacagcgttagcctggcgcagttgat
cattccggcggcagatctgtctgaacagatttcgctggcagggacggaagcttccggcaccagtaacatgaagtttgcgcttaacggtgcgctg
actatcggtacgttggacggtgcgaatgtcgagatgctggatcatgtcggtgctgacaatatctttatttttggtaacacagcggaagaagtggaa
gaactgcgtcgtcagggctacaaaccgcgtgaatactacgagaaagatgaggagctgcatcaggtgctgacgcaaatcggcagcggtgtat
tcagtccggaagatccgggtcgctatcgcgatctggttgattcgctgatcaacttcggcgatcactaccaggtactggcggattatcgcagctatg
tcgattgtcaggataaagtcgatgaactctacgagcttcaggaagagtggaccgcaaaagcgatgctgaacattgccaatatgggctacttctc
ttctgaccgtactatcaaagagtacgccgatcatatctggcatatcgatccggtgagattgtaagttcaccaataaatagaacggggccaaagg
gtcccgtttttttccgccatcatttcggaaaagtgtccagaaggggcaaggggactcggggagtataatttcaccattcaaaaagaatggaaag
aataatgcaaaacagaaaatggattttgacctcgctggtaatgactttttttcggcatccccatactggcgcaatttttggcggtggttattgccatgct
gggtgtcggacttgccggtattattgaagtttgtaatatccttatcacgccaacaatttaccttctgctcaaaatttttatgctggcgctgggcgcatta
atgctatttttctcggggcgagtggggaacgtgcctgagttttgttacgttgggtatgatggcgtgggctttgcggtaattccctgaattaaaaagtag
gttagatttttgacaaagtgcgctttgttcatgccggatgcgacgtgaacgtcttatctggcctacaagaccatgcaaattcaatatattgcataaac
cacgtaggcctgataagcgtagcgcatcaggcagttttgtgtttgtcatcagtctcaggccagattgaaatctgacctgatcaccttacgttaattta
cgacgccagcgataacctctgctgcgtatactccaccagccactgactcacacgagattgttgatccgcatttagccacatgccttgttttgtgcg
acgccacagggcgtcgtcggcgcggcggacccattcgtgatccaccaggtatttcagctccgcttcgtagaactcatgaccgaaatcttcccc
gagatcgcttaccgttcccgcattgccgagcagcagctcgctgttgctgccgtaagtgcgagcgtaatgacgcgccagcgattcagtcaggaa
cggatagcggcggcgcaggcgagcggcataatcgtcgcggtcgccttcaatggcgccacccggtagcacactctctttcgtccatgccgggc
caataccctgataatacggcgttagtttttccagcgcatgttccgccagttttcggtaggtggtcagcttaccgccgaataccgacagcagcggtg
ctttgccattttcatcatgaatatcaagggtgtaatcacgggtaatagcctgcggcgagtcggactcatcatcacacagcggacgcacaccgga
gtaggtccagacgatatcgtcacggcttaactgctttttaaagtgcgtgttatacacattcagcaggtaattgatttcactctcttcaatcttcaccgctt
tcggatcgcctttgtactcgacatcggtagtgccgatgatggaaaactcgtccatccacgggatcacgaacacaatacgtttatcttcgttttgcag
aatgtaggcttgcttctgggtatgcacgcgcggcaccacaatatggctgcctttgatcaggcgaatgccataaggcgaaggcagatgcatccc
gtcgtcgaagaactgtttcacccacgggccggtggcgttaaccaagccgcgcgcttgccagctatatttttgccggtatcgatatcttccgcttcc
acaatccacaggccgttttcgcggcgagcagaggtggcgcgagtccgagtaagcacttcgccgcctttacgcaccaccatctgggcgttggc
gagtaccagacgggcgtcgtctacccaacagtcagaatattcgaatccgcgcttaatttccggttttaacactgaatttgcgccaaaacgcaaa
ccagttgatcccggcaagctggtgcgtttacccagatgatcgtacataaacagaccaatgcgaatcatccacgccgggcgcagatgcggac
gatgtggcaggcgaaaacgcatcgggaaggcgatatgcggggccattttcagcagcacttcacgttcagccagcgcctcgctgaccaggcg
gaattcatagtgctcaaggtagcgcaggccaccgtgaatgagttttgaactggcggaagaggtcgcgcaagcgagatcctgcgcctccagc
atcagcacggataaaccgcgtccagcggcgtctgccgcgataccagcaccattgatgccgccccctatcacaatcagatctttggtttccatgc
tgccctcattcactttcgttaaagctcataaatgttcgttatcgaacatattagcaaagaatcgcgctttaggtaacattgaaaaaacattttagagt
gatatgtataacattatggcgtttatctgccgcttcgacgtaaactgtgcggtaaatttgcccacttgtttgtaaagaaagagagacgcatggatca
gttcgaatgtattaacgttgccgacgcgcaccagaagttgcaggaaaaagaggcggtgctggtcgatattcgcgatccacagagtttcgcaat
gggacatgcggtgcaggctttccatttaaccaacgacacgctgggcgcttttatgcgtgataacgactttgacactccggtgatggtgatgtgttat
cacggcaatagcagcaaaggcgcggcgcagtatctgctgcaacagggctacgatgtggtctatagcattgacggcggctttgaagcctggc
aacgtcagtttcccgcagaggtggcgtacggcgcgtaacgctttatactgtccccttttgtgtggaataagcgacagcaacgatgttgatgattac
ctcttttgctaacccccgcgtggcgcaggcgtttgttgattacatggcgacgcagggtgttatcctcacgattcaacaacataaccaaagcgatgt
ctggctggcggatgagtcccaggccgagcgcgtacgggcggagctggcgcgttttctcgaaaacccggcagatccgcgttatctggcggcg
agctggcaggcaggccataccggcagtggcctgcattatcgccgttatcctttctttgccgccttgcgtgaacgcgcaggtccggtaacctgggt

gatgatgatcgcctgcgtggtggtgtttattgccatgcaaattctcggcgatcaggaagtgatgttatggctggcctggccattcgatccaacactg
aaatttgagttctggcgttacttcacccacgcgttaatgcacttctcgctgatgcatatcctctttaacctgctctggtggtggtatctcggcggtgcgg
tggaaaaacgcctcggtagcggtaagctaattgtcattacgcttatcagcgccctgttaagcggctatgtgcagcaaaaattcagcgggccgtg
gtttggcgggctttctggcgtggtgtatgcgctgatgggctacgtctggctacgtggcgaacgcgatccgcaaagtggcatttacctgcaacgtg
ggttaattatctttgcgctgatctggattgtcgccggatggtttgatttgtttgggatgtcgatggcgaacggagcacacatcgccgggttagccgtg
ggtttagcgatggcttttgttgattcgctcaatgcgcgaaaacgaaaataattccagggatttataaatgaaacaaacacaacgtcacaacggt
attatcgaactggttaaacagcagggttatgtcagtaccgaagagctggtagagcatttctccgtcagcccgcagactattcgccgcgacctca
atgagctggcggagcaaaacctgatcctggccatcatggcggtgcggcgctgccttccagttcggttaacacgccgtggcacgatcgcaagg
ccacccagaccgaagaaaaagagcgcatcgcccgcaaagtggcggagcaaatccccaatggctcgacgctgtttatcgatatcggcacca
cgccggaagcggtagcgcacgcactgctcaatcacagcaatttgcgcattgtcaccaacaatctcaacgttgctaacacgttgatggtaaaag
aagattttcgcatcattctcgccggtggcgaattacgcagccgcgatggcgggatcattggcgaagcgacgctcgattttatctcccagttccgc
cttgatttcggcattctggggataagcggcatcgatagcgacggctcgctgctggagttcgattaccacgaagttcgcaccaaacgcgccattat
tgagaactcgcgccacgttatgctggttgtcgatcactcgaaatttggccgtaacgcgatggtcaatatgggcagcatcagcatggtagatgcc
gtctacaccgacgccccgccgccagtaagcgtgatgcaggtgctgacggaccaccatattcaactggagctgtgctgatcctgcacggcttcc
cacgtcagaccaaaacgcgccaggtatttgcgtagccgatccgcgtcattgacgctggctttgccctggcgcgaaacgtcaaaaagctggcg
tccggctgcggaaagcgactttgcctggcggcagatagcgataacgtgttccagttgcatacggtcgaagagatcgatgttttctgcctctgcgc
ccagcaacgccgtaagcgcggaggggcggctctcctgccagttatagcgcagacggtttatctcatcttcaaccacgtccagagtgatgcgtc
cgctagtggcaaaggtggccatccgcgtgacgctggcagaaagttcgcgaaagttaccgcgccatgttgcctggggagaggtcgcaaaag
ccaaccaggcgcgccgcgcttcggtgttaaaacgcacgctgtcgccagtgagtgaggcgtggcgctccacttcataatccaggttcggttcaat
atcttcctggcgctggcgtagacccggcagggtgaaggtccagagattgatccgcgcgtacagatcttcgcgaaatttgccttcggcaaccag
ctggcgcaaatcgcgcaccgttccggcgataagctgaaaatcactgctcacctggcgatcgctgccaaacgggtaaaaggttttctcttcaatg
gctttcagcagcattgcctgttcgtctgcgcccagttcgccaatctcatcaagaaacaacattccgccgttggcgctgcgtaataaaccttcacga
gattcccgcgccccggtaaacgcgccttttacatgaccaaacagcgtcgacatggcggtatcgccgcgcagggtggcgcagttcacttccac
aaacgcgccgctaaactgatgccgcgcctgttttaactctaagatgcgtcgcgccagaaatgacttgcccgcgccggttggaccgttaagcag
aatcggcgcgcgggatttgatcgccactttttcgatctgctcaatcatgcggttgaagtgggggttacgcgtggcaatgccggacttaagaaaat
caagcgtttgctggcgttcctcggcaaagcggctggcgatggcgttataacggcttaaatcgagatcgataatcgttacttcacctgggccgcgc
ggctgttctttttgcgcggaggtgaagattgtatcagccgggcgggcaggtaacgcgcttctgccagcagaaaccagcaaatctgcgcgacg
tgggtaccggtggtgatgtgaattaaatagtcttctttttctggctgaaactcgtaaccacgggcgaaatcatgcaggcaggcgtagacctcttcg
aaatcccacgggttatgcagttcaatctcaacgctcaccacttctgtttctggcgaaacgctggcgatatcgcgtttaagcgtttcaaatagcgag
cgcgagcgggcgtcgtgcaacaattccagtcgatcgatgaccaacgattcttgctggcataaacagagtgtcggacgccattttgaccagcgc
tgactgccgcgcccggcataatccagtacggtaccgacaaagccaaaagccactgttttacgcatcttagatatccttataaaagacgatatttc
attatcggtaaattggctttgcgggaaatctccggttacggacgtaaagatataatctttttaaaacaatgaattaaaaaattaagctgcttatttaa
ttttctggcacgacggttgcaattatcaggacagcaaacaacgaaaagagaaaaacaaaatgaattacgaattactgaccactgaaaatgc
cccggtaaaaatgtggaccaaaggcgtgccggtagaggccgatgcgcgtcagcaacttattaatacggcgaagatgccgtttattttcaaaca
tattgcggtaatgcctgatgtacacctgggtaaaggttccaccattggtagcgtgatcccgaccaaaggggcgattattccggcggcggtgggc
gtggatattggctgtggaatgaacgcgctgcgtaccgcgttaacggcggaagacctgcctgaaaacctggcagagctgcgtcaggcgattga
aacggccgtgccgcacgggcgtaccactggccgttgtaaacgtgataaaggtgcctgggaaaatccacctgttaacgtcgatgctaaatggg
ctgagcttgaagccggttatcagtggttaacgcaaaaatatccccgtttcctgaataccaataactataaacacctgggaacgctgggaaccg
gtaaccactttattgaaatctgccttgatgagtcggaccaggtgtggattatgctgcactccggttcacgcggaattggtaacgccatcgggactt
actttatcgatctggcacaaaaagagatgcaggaaacgcttgagacgttgccgtcgcgtgatctggcgtactttatggaaggtacggaatacttt
gatgattacctgaaagccgtggcctgggcgcagctttttgccagccttaaccgcgatgcgatgatggaaaacgtggtaacggcattgcagagc
attacgcagaaaacggtcagacagccacaaacgctggcgatggaagagatcaactgtcaccacaactatgtgcaaaaagaacagcacttt
ggtgaagagatctacgtgacgcgtaaaggcgcggtgtctgcgcgtgctggtcaatatggaattattcccggttcgatgggagcaaaaagcttta
tcgtccgtgggctgggaaatgaagagtcgttctgttcgtgcagccacggtgccgggcgggtaatgagccgaactaaagcgaaaaaactgttc
agcgtggaagatcaaattcgtgccaccgcgcatgtggaatgccgtaaagatgccgaagtgatcgacgaaatcccgatggcgtataaagatat
tgatgcggtgatggcggcacaaagcgatctggtggaagttatctataccctgcgtcaggtggtgtgcgtaaaaggataaatgatgaaaaggat
gattgcgctggatggcgcacagggcgaaggtggcgggcagatcctgcgctcggcgctgagcctgtcgatgataaccggccagccatttacc
atcaccagcattcgtgccgggcgggcgaaaccggggctgttgcgccagcatctgaccgcggtaaaggcggcgacggaaatttgcggagca
accgtggaaggcgcggagctggggtcgcagcgtctgctcttccggcccggcaccgtgcgcggcggcgattaccgctttgctatcggtagcgc
cggaagttgtacgctggtgctgcaaacggtgctgcccgcgctgtggtttgccgatggaccttcgcgtgttgaagtgagcggcggcaccgataac

ccgtcggcgccgcctgcggattttatccgccgggtgctggagccgctgctggcgaaaataggaattcatcagcaaaccacgctgttacgccac
ggtttttaccctgccggaggcggtgtggtggcaacggaagtctcgcccgtggcatcgtttaacaccttgcaacttggcgagcgcgggaacattgt
gcagatgcgtggagaagttctattagctggtgtgccgcgccatgttgctgagcgtgaaatcgctacactggcggggagtttttccctgcatgaac
agaatattcataacctgccgcgcgaccaggggccgggtaataccgtctcgcttgaagtcgaaagtgaaaatatcaccgaacgctttttgtcgtc
ggtgaaaagcgcgtcagtgccgaggtggttgcggcacagttggtgaaagaggtgaaacgctacctggcaagcacggcggcggtgggga
atatcttgctgaccaactggtgctaccgatggcgctggcgggcgcgggggaatttacggtcgcccatccctcatgccatctgctgaccaatatcg
cggtggtggagcgtttcttgccggtgcggtttagtttgatagaaacagatggcgtaacgcgggtgagcattgaatgatgcagagatgtaagccg
gatctggcgcgttatccggctaaacttacacgccgtaccccatcatcttcagcaattgctgggcgtgttgtaccgcatcctggcgatgggccacg
ccgagtttctgatacagattgcggatatgcgttttgatggtggttgccgcgacttccagttctccggcaatttgctcattgctgtaaccagagtagatc
agccccagtacctgccattcacgttgcgtcagcgggctggtgcggatcagttcaggtacttcaggatgatttagcagacgttcaacgaaattctc
atcgaaatgggcgaatttatgccgatgatgttgattgatttctcgcagaatacgctgcgcgcgatgctgttccagttccggcagcgtattaagctga
atcagctgacgcagttgttgcgccatcgcttcgccttcgatgacaaaatggctgataaatccggtgcgattcgccagttttaatgcgtccagcaac
acgcgctgggcgtcacttttacgtccggcctgccagtacagttgattaagcagcaacaggttacggttgagatcgctcatcaaccgcagactcc
gggcattttcattgagttcttcgagaacaatttctgccggttcaaactcgcccagcaagatttgtgcacgggcaatgttgcgccattgaccttgcag
gaagtggttgttcgcaaactctggtttagccgtatgacgcaaccagttggcagcggcggctttatcgccggtcatttgccagtaaatcacccgga
ctttgttggcgttagagatccagtcgctgtgatatttgccattccccagcaggttttccagacggttcagctggctacgggcgttatctaaatcacca
cgggccagcgagcattgaatcaacattgccaggcactgaagctgttgctgtggctgataagacgacaagacttcaatcccgctacgcgccga
cgcttcggcttcatccagccgcgcccaggcccataacagctgcgcacgaatgcgcaccagaaactcatgcattggcagctgttccagatgct
gctcgttgatcagctggaatgctttttcctgcgtttcccacgcggtttgcaggaacccttgggcaaacagaatttcactttgctggattaaactccac
aaagcgtagtgccagacatcgtgctggcgtgccatctgttcggtttgctgcattagcgccagtgagcgggtcaattcgcctttgcagtgcagcact
tcacccagcaccgaggttgccacaatgcggctatagaaccagcccggcggcagctcttccagtgccagttttgccagccgttccgcttcatccg
gattaccatcgttaatcgccacctgggcgcgcagagcgttaaattctgcgtgcatggtgtcttctctgatgtccttgatttcatgttcagcacgggcta
gcagggtgttaacttcgccgtagcgatgttggctttgcatcagccacgcctgcaataacaccaactgcggattttccagcaagctgtcccacggc
agggccttaagcgactcttccagcagcgacagttcgctatggttgaacagactccaggcgtgattaagcagaatatcgcgcagcatcagcgc
atcgcctgccgccagcgcatgatgaattgcttcgctgggaaatccctgggccatccagctttctgcggcggcacggtggatttccggcagctcc
gccgccagttcccactggcagcgctggcgcaggaagttaccaaacagcgggtgatagcagaaccactcgccggtatcatccatccgctgta
aaaacagcccctgacgctcaatctcttcgaggcgcatttgcccgttttcttcgccggtcacacgggtgatgagggcatcgttcattgagcgcaaa
atggcgcttttcaacagaaaatggcgcgttgcgagatcgacgttatccaaaacctcatcgaccagataatccgaaagatggctggcattgattc
ccgccaggcggcgtgccgacttatgggctgagtgggtattctgccgggcggagagggcgattagctgtagtgccgtcgcccaaccggaaac
gtcatcgcaaatccgactgctttctgcggcttcaatcggcgatgacagacggcaatcaaaaaactgcttcgcttcctgatgggtaaatgccagtt
gctgactgccaatttccagcagttgatcacgaacacgcagattggcaatgcccagttgcggaaggttgcgtgacaacaccaccagggtgaga
ttttctggttgatggcgaataaagaagcgcattgactcgtggatcactggattagtgatcagatgatagtcatcgatgaccagataaagtgggcta
tgccattccgccagctcaatgaaaagctgggcgaagagtgacgtcaggctggcatattgccgtttttgcgccatcgtctcacatatcgcacagtg
accgttggttgcctgctgcacggcggcaatgagatagctggcgaaacgctcttgctggttatcaccttcatccagcgagtaccagccgatatcgt
ttttgcctgccgcccactgggaaatgagggtggtctttccgtagcccgcaggactcgtgatcagcgccagccggaagttgttcgcgccggaaa
gtttagccagcaggcgctcacgaaccacggtatggtcgagtcgaaccggacgacttagttttgacggaatcagcatagttaatcacttcactgt
ggaaaatgaggaaatattattttttttgcgcttcgtaattaatggttataaggtcggccagaaacctttctaatgcaagcgatgacgtttttttatgtgtc
tgaatttgcactgtgtcacaattccaaatctttattaacaactcacctaaaacgacgctgatccagcgtgaatactggtttcccttatgttcatcagatt
catttaagcaagggtttcttcttcattcctgatgaaagtgccatctaaaaagatgatcttaataaatctattaagaatgagatggagcacactggat
attttacttatgaaactgtttcactcctttacttaatttatagagttaccttccgctttttgaaaatacgcaacggccatttttgcacttagatacagattttc
tgcgctgtattgcattgatttgatgctaatcctgtggtttgcactagctttaagtggttgagatcacatttccttgctcatccccgcaactcctccctgcct
aatcccccgcaggatgaggaaggtcaacatcgagcctggcaaactagcgataacgttgtgttgaaaatctaagaaaagtggaactcctatgt
cacaacctatttttaacgataagcaatttcaggaagcgctttcacgtcagtggcagcgttatggcttaaattctgcggctgaaatgactcctcgcca
gtggtggctagcagtgagtgaagcactggccgaaatgctgcgtgctcagccattcgccaagccggtggcgaatcagcgacatgttaactaca
tctcaatggagtttttgattggtcgcctgacgggcaacaacctgttgaatctcggctggtatcaggatgtacaggattcgttgaaggcttatgacatc
aatctgacggacctgctggaagaagagatcgacccggcgctgggtaacggtggtctgggacgtctggcggcgtgcttcctcgactcaatggc
aactgtcggtcagtctgcgacgggttacggtctgaactatcaatatggtttgttccgccagtcttttgtcgatggcaaacaggttgaagcgccggat
gactggcatcgcagtaactacccgtggttccgccacaacgaagcactggatgtgcaggtagggattggcggtaaagtgacgaaagacgga
cgctgggagccggagtttaccattaccggtcaagcgtgggatctccccgttgtcggctatcgtaatggcgtggcgcagccgctgcgtctgtggc
aggcgacgcacgcgcatccgtttgatctgactaaatttaacgacggtgatttcttgcgtgccgaacagcagggcatcaatgcggaaaaactga

ccaaagttctctatccaaacgacaaccatactgccggtaaaaagctgcgcctgatgcagcaatacttccagtgtgcctgttcggtagcggatatt
ttgcgtcgccatcatctggcggggcgtaaactgcacgaactggcggattacgaagttattcagctgaacgatacccacccaactatcgcgattc
cagaactgctgcgcgtgctgatcgatgagcaccagatgagctgggatgacgcctgggccattaccagcaaaactttcgcttacaccaaccat
accctgatgccagaagcgctggaacgctgggatgtgaaactggtgaaaggcttactgccgcgccacatgcagattattaacgaaattaatact
cgctttaaaacgctggtagagaaaacctggccgggcgatgaaaaagtgtgggccaaactggcggtggtgcacgacaaacaagtgcatatg
gcgaacctgtgtgtggttggcggtttcgcggtgaacggtgttgcggcgctgcactcggatctggtggtgaaagatctgttcccggaatatcacca
gctatggccgaacaaattccataacgtcaccaacggtattaccccacgtcgctggatcaaacagtgcaacccggcactggcggctctgttgga
taaatcactgcaaaaagagtgggctaacgatctcgatcagctgatcaatctggaaaaattcgctgatgatgcgaaattccgtcagcaatatcgc
gagatcaagcaggcgaataaagtccgtctggcggagtttgtgaaagttcgtaccggtattgagatcaatccacaggcgattttcgatattcagat
caaacgtttgcatgagtacaaacgccagcacctgaatctgctgcatattctggcgttgtacaaagaaattcgtgaaaacccgcaggctgatcgc
gtaccgcgcgtcttcctcttcggcgcgaaagcggcaccgggctactacctggcgaagaatattatctttgcgatcaacaaagtggctgacgtga
tcaacaacgatccgctggttggcgataagttgaaggtggtgttcctgccggattattgcgtttcggcggcggaaaaactgatcccggcggcgga
tatctccgaacaaatttcgactgcaggtaaagaagcttccggtaccggcaatatgaaactggcgctcaatggtgcgcttactgtcggtacgctg
gatggggcgaacgttgaaatcgccgagaaagtcggtgaagaaaatatctttatttttggtcataccgtggaacaagtgaaggcaattctggcca
aaggctacgacccggtgaaatggcggaagaaagataaggtgctggacgcagtattgaaagagctggaaagcggtaaatacagcgacgg
cgataagcatgccttcgaccagatgctgcacagtatcggcaaacagggcggcgatccgtatctggtgatggcggatttcgcagcctatgtaga
ggcacaaaagcaggtggatgtgctgtaccgcgaccaggaggcctggactcgcgcggcgatcctcaataccgcccgctgcggtatgtttagct
cggatcgctctattcgcgattatcaggctcgtatctggcaggcaaaacgctaaggaagctcgatggaaagcaaacgtctggataatgccgcg
ctggcggcggggattagccccaattacatcaatgcccacggtaaaccgcagtcgattagcgccgaaaccaaacggcgtttgcttgacgcgat
gcatcaacgtaccgccacgaaagtggcggtaacgccagtcccgaatgtcatggtttataccagcggcaaaaaaatgccgatggtggtggag
ggcagcggcgaatatagctggctgctgaccaccgaagaaggaacgcagtacaaaggccatgtaacgggggcaaagcgttcaatctacc
gacgaagctgccggaaggttatcacacgctgacactcacccaggacgaccagcgcgcgcattgccgggtgattgtcgccccgaaacgctgt
tacgaaccgcaggcgttgctgaataaacaaaagctgtggggtgcctgcgttcagctttatacgctgcgatcggaaaaaaactggggtattggg
gattttggcgatctcaaagcgatgctggtggatgtggcaaaacgtggcgggtcgttcattggcctgaacccgattcatgcgctctatccggcaaa
tccggagagcgccagcccatacagcccgtcttctcgccgttggctgaatgtgatttatatcgacgttaacgccgttgaagatttccatcttagcga
agaggctcaggcctggtggcagttgccgaccacgcaacagacgctgcaacaggcgcgcgatgccgactgggtcgattactccacggttacc
gccctaaaaatgacagcattacgaatggcgtggaaaggtttcgcgcaacgtgatgatgagcagatggccgcgtttcgccagtttgttgcagag
cagggcgacagcctgttctggcaggcagcctttgatgcgctacatgcccagcaagtgaaagaggacgaaatgcgctggggctggcctgcat
ggccagagatgtatcagaacgtggattcaccagaagtgcgtcagttctgcgaagaacatcgtgatgacgtcgatttttatctctggttgcagtggc
tggcttacagccagtttgccgcctgctgggagataagccagggctatgaaatgccgattggcttgtatcgtgatctggcggttggcgtagcggaa
ggtggggcggaaacctggtgtgaccgtgaactatattgcctgaaagcatcggttggcgcgccgccggatatcctcggcccgttggggcagaa
ctggggattaccgccaatggacccgcatatcatcaccgcgcgtgcctatgaaccgtttatcgagctgttgcgtgccaatatgcaaaactgcggc
gcattacgaattgaccatgtgatgtcgatgctgcgtttgtggtggataccgtatggcgagacggcagatcagggcgcgtatgttcactatccggtg
gatgatctgctctcgattctggcactcgaaagtaaacgtcatcgctgtatggtgattggtgaagatctcggtaccgtaccggtagagattgtcggta
agctgcgcagcagcggtgtgtactcttacaaagtgctctatttcgaaaacgaccacgagaagacgttccgtgcaccgaaagcgtatccggag
cagtcgatggcggttgcggcgacacatgacctgccaacgctgcgcggttactgggagtgcggggatctaacgctgggcaaaaccctggggc
tgtatccggatgaagtggtactgcgcggtctgtatcaggatcgcgaactggcgaagcaagggctgctggatgcactgcataaatatggttgtct
gccgaaacgtgccgggcataaggcatcgttgatgtcgatgacgccgacgctgaaccgtggtttgcagcgctacattgccgacagtaacagtg
ctctgttaggactacagccggaagactggctggatatggccgaaccggtgaatattcctggcaccagttaccagtataaaaactggcgacgc
aagctttccgcaacgcttgagtcgatgtttgccgatgatggcgtgaacaagttgctgaaggatttggacagacggcgcagagctgcagcgaag
aagaagtagagtgcggttgatgccggatgcgatgcttgacgcgtcttatccggcctactaattcatcccgaatcgtaggccggataaggcggtc
acgccgcatccggcagtcagtgttatcaaatcaccatattcagcagcagacagcctaccagaccgcacaccgagataatggtttccagcatc
gaccaggatttgatggtctcgccgatagtcaggttaaagtactctttgaacagccagaagcccggatcgttcacatgagagaaaatcacacta
ccggaaccaaccgcaataaccatcagctccgggctaacacccgtcgttgcaatcagcggtgccgcgataccacccgcagtgattgccgcaa
cggttgcggaacccagcgcgatacgcagtacggcagcaatcgaccaggccatcagcagcggagaaatgttggtttcgtgcatcatggaagc
aatgtatttgtccacgccgctgtctaccagcacctgcttgaacgcaccgccaccaccgatgatcaacagcatcatcgcaatgattttgatggaag
aaaccagcgtgtcgttaatctgatccattgaacgaccacggtttagaccaaaggtgaacatcgcaatcagcacggcaatcagcgttgccatta
ccgggtcaccgaggaactccgctaccggcaggaaagcgtgacctttcggcaggatcatttcggcaatcgcacgcatcgccatcagtactacc
ggcaccagagaggtccagacgctgacgccaaagctcggcatctcttcttcgctgaaggttttcgcgctgtagagaccttccggaattggcttatc
gatacctttcaacacgcgagcgtaaaccggaccggcgagaatcacggtcgggattgccagaatagtaccgtacagcagggttttacccatat

cggcattgaaaatggtggcaatcgcagtcggacccggatgcggtggcaggaagccgtgggtcacagacagtgcagccgccattggtacac
caacatacagcagtggaatattcgcagaagccgcgatggtaaacaccagcggcagcatcagcacaaagcccacttcatagaacagggca
aaaccaacggtaaaaccggtcagtaccaccgcccactggatgtgtttttaccaaatttggcaatcagcgtggtggcgatacgttgtgcgccac
cgcagtctgccagcattttgcccagcattgcgccaaaacccatgatcagggcaaggctaccgagcgtcccgccgacaccggctttgatggag
ccaataactttatccagcggcattccttgcattaatccaacagcaagcgccacgaggacgagagcgatgaagccgttcattttgaagcggatc
atcaggagcaacaacaagattacaccgatagcaacaatgactaatggcatgatttacctggcctttcatttgttatgggtaacgtcaattttctgac
gacaaactctaattatcccaatcgggaacagagatattgcggcaccacgactgatacccactaaaactaattattgtagtcagatgtcaggagt
atgtttggtacccatgtgaatgatacgggtaacatctggcgtttgagaatcaccagagcggggtaaatttaaattatgagaggttggtcatattatc
gcggggaaacgaaccgaggatttgacaaagcaatgctgcgccaacgtctggcacatgttcaacgtaggcccgaaatgacgctttagcgtcg
catcgggcaatctacaaaagaggggataacttagtagtaggagtgttcgccgcgctggtgttcggtgagatcgcgcacacctttcagctccgg
gaattcgttcagcagctgcttctcgatcccttctttcagcgtcacatcgaccatggaacaaccgttacagccgccgccaaattgcagaatggcgt
aaccgtcttcggtgatttccatcagcgaaacgcgaccaccgtgaccagcaagctgtgggttgatctgcgactgcagcatatactccacgcgctc
catcagcggtgcatcgtctgccactttacgcattttggcgttcggggctttcagcgttaactgggaacccaactggtcggtaacaaaatcgatctct
gcatcttccaggtatggtgcgcttaactcatcaacatacgcggtcagcaggtcaaatttcagggctgtgtcggtggcttccacagcgtccggcgg
acaataagaaacgccacattcagcgttaggcgtgccagggttaatcacaaatacgcggatttgtgtcccttcttcctgatttgccagcagtttggc
aaagtgcgcttgtgcagcatcggaaatacggatcatagtaatggcctaatagttgactattttagttggttataatacgcccatcatcgaggctcta
caaggttcgacaaaggcaccagacctggacagccgccgcaccattgcgtaaaagcaactgcgcaatctctgcgacggtacttccggtggta
acgacatcatccacaatcaccatatggcgaccttgcacgggcaattcaagacgaaaggcatttttcaggttgcgcttgcgcagccgggcactg
agaaaatgctgggtcgcagtggcccgtgtacgtgtgacggcttcgctatcccattggcagtgcaaccagcgtgataacggctgacacagcaa
atcgctctgattaaatccccgacgccagtgacgccgctgccataacggaacgctgacgatgcgatccggcaattgcaacccggtggtgcgac
gagcgtgtaagacttccaatagtaacagacgtgacagggcgctggcgatttcactgcgccgggaaaatttaagctggtggataagcggactt
aacggcggcgcatagtcggcaaccgtgaccagtctttgccagggcggcggtttttgcaggcagcgaccgcagggaagatgggagtgtgtgg
cgggtaatccacattgtgggcataacgttttatctgtgcgggtggcgcgtgaacagaccgaacaaatcccccaatgacctaacgccagtggca
ttcggcatagccagcataatcccggtactgttagcatatgttcatccttgtaagtcaaaagagaacaatagcggatgaataacatctggtggcag
accaaaggtcaggggaatgttcatcttgtgctgctgcacggatggggactgaatgccgaagtgtggcgttgcattgacgaggaacttagctcgc
attttacgctgcaccttgttgacctgcccggcttcgggcgtagccggggatttggtgcgctgtcacttgctgatatggccgaagccgtgctgcaac
aggcacctgataaagccatttggttaggctggagtctgggcgggctggtggcaagccagattgcgttaacccatcccgagcgtgttcaggcgc
tggtcaccgtggcgtcgtcaccttgttttagtgctcgtgacgagtggccggggataaaaccggacgtgctggcgggatttcagcagcaactcag
tgatgattttcagcgtacagtggagcggttcctggcgttacaaaccatggggactgaaacggcgcgccaggatgcgcgggcgttgaagaaaa
ccgttctggcgttaccgatgccggaggttgacgtgcttaatggcgggctggaaatcctgaaaacggtcgatctccgtcagccgctgcaaaacgt
gtccatgccgtttttgcgattgtatggctatctcgacggtctggtgccgcgcaaagtggtgccgatgctggataaactttggcctcacagcgaatca
tatatcttcgccaaagcggcccatgcgccatttatttcgcatccggccgagttttgtcacctgctggtggcgttgaagcagagggtgtaggtggcttt
tgaaatggcgagacagaactttactgactcgccgcagccaactcttcttctgacagcccggtaaagcgcatgatgtctgcaagaggaactccg
gattcaagcattattttggctatatgcagggctttggattgttcccccctcctgccgcaatctttccgcaatagtcattaaactctccttgtgtttcggtgaa
cgttcggcaacgccgtcgataaaatcgttaaaacgtacagcgtcgccagtttgcagtatgtaattaaacagccctttgatttgtctgtcattagcgt
atccactacttaataagcaggccatttgctctaccagccccatcaggtcgcgttgacgaatatgtttttgaattaactccagcagcgccatacgtc
ggtgctgcatgatttcatcatcaggcatgacggtgacatcaatcagcggaaatgcggaggcataaagctgccttgccagtttgggatcggcga
aacaatccagccaacacagcgaatagggataggggctttcaataccgtggtaaaacaacaatggcaccaccatcggcaacgttttgtatcca
gcatcaaggtgattttgcattgcggcaatagcgtaacgcatcatgcgaaatgcgatcagtttgtttgaggtgctttgatgttcaatcagacaataga
tgtatcctggtccttgttccgttttcaccgaccacagcacatcggaatagctttcacgcagatcgtcatcaataaagctgctcgactccagcttgag
ggttttcatatcacaaagcgcgtgaatgggtgccggtaaatgaaacgcaagaaaatcacgagccgtgtccggttggcgtaaaaagagtttga
acagcgcatcgtgtggggtggaactctgctttttgctcatggcgttccttcacctgttgtttgaatgccgcgactctacgcgccactccacacctgtgt
attagcgagttttcatccctgcgagatgctttcaaaggaacttacgccgtgctgcaagagttgcatttttatggaaagccgcacgcaaaaaagcc
cggatgcagacgcataccgggctttccaaaacaggcgatggagtaaggttaacgcagcgcccaccactcgcgcagacaggcttttccttccg
ggcagcttttacaactgccagagaggcagccgtcaggttcttcctgaatccgcacggctttgcccatactttccagttgttgcagcatggcgttaat
cattggctgtggagtgttcaatgtctggcttatctgggccgcttccatacggccccgtaacgccagcaaatcgcgcacctgaataagtgaagcc
attaatggcaatcaccggtggtgctggctgcgcagcaactgcttaccgacttgcgggttgccagcagttcgatatccacccggctacgcgcgcg
gcgcagcagaccgataaccacgatgttaaacaggataaccgccagaatgcacaccaggctgtaagttggatgctgactgtagctggcgacc
tgatagaacaatgttgccagtgagtaagcgatattcagcccccacaggatggagaagcccatccagccacggcttgattcacgggcgatagc
ccccatcaccgagatacatggtacatacagcaggacgaaaatcaggtagctgtaagctgctgctgcgctaccgaatttctgatccatcacgcc

catcgccccggtacccatttcgccgtcgcctttgctggcttcaatggggttcatcagtacgctaaggctgaaggtgtctttcaggctctgccaggttt
catctatcgcactgaacagctcttcaccgaggttaaattctgccggattgaactcttcgtcctgaatattttctgcggtgtagagggtgttgagcgta
cccactaccacttctttcgccatggcacctgtaaacaggccaaccgttgcctgccagttatcttcatgcacgccaattggcttgaagaccggggt
gatcacccggctgacggacgccagcgccgagtcgttgatgttatcgacgattttcccgctcagcgagaagctgttgaaagcgctcaggaaaat
gctgacgatgatgatcactttaccagcacgcagaacgaagcctttcagacgctgccaggtctggataatcaggcttttaacgtgtggtacatgat
agaccggcaactccatgacaaacggcgtcgcttcaccgcgcatgatggtgtacttgagcatcaggccagtcagcaccgccatcacaatacc
cagcatatacagcgagaagaccgccagcgcaccgttctgcccgaagaaggcagccgcgaatactgcgaagatagccagacgcgcgccg
caggacataaacggtgccatcatgatggtcatcagacgttcacgcggtgcatcaagcgtacgtgcacccattaccgacggtacgttacaacc
gaaaccgacgatcagcggcacaaaggatttccccggcaagcccagcgcctgcatcagacggtccatcacaaacgccgcacgcgccatat
acccggagtcctcaaggaaggagaggaacaggtacatcatgccaatctgcggcaccagtggcagcacggtgttaatgccgccacccagg
ccctgggcgaggaagatagtcagccagtccgggaagtggagcgtgtagccaatccattgaataccatgcacaaatagcgccacggagccg
acgtcaaacagcggctgtaacgccccgccgatgttgatagccagcaggaacatcaggtacatcacaaagaggaaaatcggcagaccgag
gaaacggttgagcacgattttatctaccgcagtggtgaaacggctgggttctgccgtcagggtgttgcttaccacatcacagatggcagcaatg
cactggtaacgcgcatcggcaatgtgcagcgccggatcgtccatctcattacgcagacgggcgagggcggcatccagatgctgcgacgcttc
accggcgtaggcgcggctgtagatatcgccttccagcatttgcaggcccagccagcgacgttgtttcagcgggatgtcggaaggcatcactttt
gccagtgaatctgcttcgttgagcagcggctgtgcgtaatgcaccagttccacattctcgttagctttatagcgatcaatcgccagcttgagcgctt
caataccgcgaccacgggttgaaaccagcgggatcaccggacagcccagacgcgccgacagagcatcaatttcaatacgaatattttgctt
ctcggcaatgtcgagcatgttcagtgccacaatgcagggaatgccgagttccagcagttgtagcgtcaggtacaggttacgctcaaggttaga
cgcatccaccacgttaatcagcaggtcggcgtcgccactcaaaatgtagtgacaggcgatttgctcatcgagcgaggtctgcgatgagatggt
ggtcagagaataggtgccgggcaggtccaccagcgtgacctgatgatcggtggtggagaattgcccttctttacgttcgacggtaacgccagc
ccagttacctacacgctgacgtgagccagtgagctggttaaataacgttgtcttgccagaatttggattaccaattaagccaatggttaatttttcat
tgttgttatcaccgtattaacaggaaaccgcttccacttctaataaggccagatctttttgcgtaataccaggctcacacgacgggtttcgatatga
atggggtcgccgagtggagcgacgcgcaccacattaaaagaggagccaggtaacatgccaagagaaagcagtttttggcgatatgccggg
ctgatttcacgggaaaagccagtgattttccacgcagtatctggagtgtattgcataggtgcctacttgtttctcattaactggataactacctcatgg
ggcgtttgacagcgacatgagaccagatgatttgccaacaaaggagagattaaataatcaacaacacaatgataatgagaatggtttctatca
gcaatacttaaaatgtgtgcgaatgttggtttgaataataatctgactcaatgttgatgtggctcaaggttatatcgcagcgaaaatataattcgaat
agtgaattacccggactggctaatgtttaattaaggtttcagttgttaatgaaatgataaatattgccggaatatattatgagtttggctgtggaataa
aaatgccggagatattctccggcacttttatgtcaggcaaggcgttcgttgccagatgcggcgtgaacgccttatctgccctacgatttcgtgcaa
attcgaaccgtaggccagtacgggcgtttaacgtttttttgcccattgccgccgccagcgcatccatcatcgcgctattaccggcaggctgcgcttc
acgaccgcgtggtttggctgccgggcggttgttttgcgggcgttcattaccgccgccgcgacgagcgttggtttcgccaggctgctcatccaggc
gcatagtcagggcgatacgtttacgctgaagatccacttccagcactttcaccttcacaatgtcgcccgctttcaccacggtatgcggatcttcca
caaacttgttcgacaatgaagagatgtgaaccaggccgtcctgatgcacgccaatatcgacaaacgcgccaaagttggtgacgttggtcact
gcgccttcgaggatcatacccggttgcaggtcgttcattgtctcgacgccatcggcaaactgagcggttttaaattccggacgcggatcgcgac
ccggtttttccagctctttgatgatgtcggttaccgtcggcacaccgaatttctcatcagtaaagtcagacgctttcaggttacgcagttcgctgctgtt
acccatcagatctttcagtgcctgctgtgttgctgccagaatgcgttccaccaccggataggcttccgggtgaacggtagacgcgtccagcggg
ttatcaccgtggttaatgcgcaagaagcccgcgcactgctcgaaggctttcggccccagacggctcacttttaacagttgctgacggttctggaa
ctgaccgttctcatcgcgccaggcaacgatgttttgcgccatcatgcgcgtcaggcccgccacgcgggttaacagcggaacagaagcagtgtt
gagatcgacgccaacggcgtttacgcagtcttctactactgcgtccagtttgcgggccagttgcgtctggctgacgtcatgctgatactgacctac
gccgatagatttcggatcgattttcaccagctccgccagcggatcctgcaaacggcgggcgatagacaccgcgccacgcagcgaaacgtcg
agatccgggaactcctgtgcagccagctcggaagccgagtaaaccgacgcgcctgcttcgctgacgattactttctgcgcggtcactttcggga
actgcttctgcacatcgagatagaaacgctcagtttcgcgggaagctgtaccgttaccgatcgctaccagttcaacgttatgtttttcacacagcg
cagcaacggtcatcgctgcttttgcggcctgtccggtgtgcgggtaaatggtgtcggtcgccaccagtttgccagtggcatcgaccaccgccact
tttaccccagtacgcagacccggatcgaggcccatcgttgcacgcagtccggcaggggccgccatcagcagatcgtgcaggttacgggcaa
agacgttgattgcttcatcttccgcacgttcgcgcacggtgcccatcagttcggtttccagatgcatcagcaccttgatgcgccacgtccagctca
ccacgcctttgcgccagctatccgccggggcattgttcaggcgcaggccaaggtgatccatgatgatttgctcgcaatagctctctttgggcggct
catcgaactgtggatcggcattcagcgaaagctgaagtacgccttcgttacgcccacggaacatcgccagcgcgcggtgagaaggcaccgt
ggacaacggttcgtgatgatcgaaatagtcgcggaatttcgccccttcctcttctttaccgctcaccaccgtagaaaccaaatgcgcgttcttcca
cagataatcacgcactttcgccagcagcgcggcatcttcggcaaaccgttccatcaggatatagcgcgcgccatccagcgcggctttggtatct
gccacgcctttatcggcataaacatattgtgcagcggcgacttctggcgtgtgtgacggatcgctccacagcaggtcagccaacggctcaagc
cctgcttcaatggcgatttgcccgcgggtgcggcgtttaggtttgtagggcaggtagaggtcttcgagttcggttttgcttagggtggcgttgatggc

cttcgccagatcatcggtgagtttgccttgctcggaaatggacttgaggatcgcctgacgtctctcttccagctcgcgcagatagctcagacgcgt
ttccagattacgcagctgcgtgtcatccagaccgccggtgatttccttacgataacgtgcgataaacggcacggtattcccttcgtcaagcaggc
gaacggcagcgtcaacctgttccgggcgcgcctgaatttcacccgcaataatgcggcagaacgaatcattcatcatggtttgagttcatcttttcg
gatcaaaaatcaggggatagttatacggactggctggcaaaaatgccagccatcggcaggaggttaagactcttccttacggtttcacgtactc
gatagcattaacataccagctcgcttccccggcaggggtattcaccaccgccagatcgccgacttctttttcagcaatgcgcgggccatcggg
gaatcgatagagatgtaatctttacggccaaaaatttcatcgtagccgacaatacggaaacggtgagtcacgccatcgtcgttttcaatctccac
ccacgcgccaaaaaagactttgccttcctgctgaggggagtaatcgacgattttgagattttccaggcatttagtgagatagcgcacgcgacgg
tcgatttcacgcagacgctttttattatactgatagtcagcattttcgctgcggtcgcccagacttgcggcccaggtcacctttttgtgacctccgggc
gttcttcacgccagagataattaagctcttgtttgagtttttcatacccttcccgggtaaccaggggcgttttcatctcgttgattccctttgtctgtttgata
atgcgcacattgggtataacgtgatcatatcaacagaatcaataatgtttcgccgaataaattgtatacttaagctgctgtttaatatgctttgtaaca
atttaggctgaaattcataccagatttagctggtgacgaacgtgagctttttaagaatacacgcttacaaattgttgcgaacctttgggagtacaa
acaatgcaagagaactacaagattctggtggtcgatgacgacatgcgcctgcgtgcgctgctggaacgttatctcaccgaacaaggcttccag
gttcgaagcgtcgctaatgcagaacagatggatcgcctgctgactcgtgaatctttccatcttatggtactggatttaatgttacctggtgaagatgg
cttgtcgatttgccgacgtcttcgtagtcagagcaacccgatgccgatcattatggtgacggcgaaaggggaagaagtggaccgtatcgtagg
cctggagattggcgctgacgactacattccaaaaccgtttaacccgcgtgaactgctggcccgtatccgtgcggtgctgcgtcgtcaggcgaac
gaactgccaggcgcaccgtcacaggaagaggcggtaattgctttcggtaagttcaaacttaacctcggtacgcgcgaaatgttccgcgaaga
cgagccgatgccgctcaccagcggtgagtttgcggtactgaaggcactggtcagccatccgcgtgagccgctctcccgcgataagctgatga
accttgcccgtggtcgtgaatattccgcaatggaacgctccatcgacgtgcagatttcgcgtctgcgccgcatggtggaagaagatccagcgc
atccgcgttacattcagaccgtctggggtctgggctacgtctttgtaccggacggctctaaagcatgaggcgattgcgcttctcgccacgaagttc
atttgcccgtacgttattgctcatcgtcaccttgctgttcgccagcctggtgacgacttatctggtggtgctgaacttcgcgattttgccgagcctccag
cagtttaataaagtcctcgcgtacgaagtgcgtatgttgatgaccgacaaactgcaactggaggacggcacgcagttggttgtgcctcccgcttt
ccgtcgggagatctaccgtgagctggggatctctctactccaacgaggctgccgaagaggcaggtctgcgttgggcgcaacactatgaatt
cttaagccatcagatggcgcagcaactgggcggcccgacggaagtgcgcgttgaggtcaacaaaagttcgcctgtcgtctggctgaaaacct
ggctgtcgcccaatatctgggtacgcgtgccgctgaccgaaattcatcagggcgatttctctccgctgttccgctatacgctggcgattatgctatt
ggcgataggcggggcgtggctgtttattcgtatccagaaccgaccgttggtcgatctcgaacacgcagccttgcaggttggtaaagggattattc
cgccgccgctgcgtgagtatggcgcttcggaggtgcgttccgttacccgtgcctttaaccatatggcggctggtgttaagcaactggcggatgac
cgcacgctgctgatggcgggggtaagtcacgacttgcgcacgccgctgacgcgtattcgcctggcgactgagatgatgagcgagcaggatg
gctatctggcagaatcgatcaataaagatatcgaagagtgcaacgccatcattgagcagtttatcgactacctgcgcaccgggcaggagatg
ccgatggaaatggcggatcttaatgcagtactcggtgaggtgattgctgccgaaagtggctatgagcgggaaattgaaaccgcgctttacccc
ggcagcattgaagtgaaaatgcacccgctgtcgatcaaacgcgcggtggcgaatatggtggtcaacgccgcccgttatggcaatggctggat
caaagtcagcagcggaacggagccgaatcgcgcctggttccaggtggaagatgacggtccgggaattgcgccggaacaacgtaagcacc
tgttccagccgtttgtccgcggcgacagtgcgcgcaccattagcggcacgggattagggctggcaattgtgcagcgtatcgtggataaccataa
cgggatgctggagcttggcaccagcgagcggggcgggctttccattcgcgcctggctgccagtgccggtaacgcgggcgcagggcacgac
aaaagaagggtaaataaacgggaggcgaaggtgcctcccgttttgctttctataagatactggatagatattctccagcttcaaatcattacagtt
tcggaccagccgctaccagcgcggcacccgcaggggtgtcggtgtatttatcgaagttgtcgataaacagtttcgccagggtttcggctttttcct
gccactgttccggagaagcgtaggtgttacgcggatcgagaatcttcgtgtctacgcccggcagttcggttgggatcgccaggttaaacatcgg
cagagtgaaggtttctgcattatccagcgaaccgttgaggatggcgtcgataatggcgcgggtatctttaatcgagatacgtttgccagtgccgtt
ccagccagtgttaaccagataagcctgcgcgcccgccgcctgcatacgtttcaccagcacttctgcgtactgagtcgggtgcagcgacagga
atgccgcgccgaagcaagcggagaaggttggcgtcggttcggtgatgccacgctcagtaccggccagtttggcggtgaagccagagagga
agtgatactgggtttgatcggcagtcaggcgagaaaccggcggcaacacgccgaaagcatcagcagtcaggaagataaccttagtcgcgt
ggcccgctttggaaaccggcttaacaatgttatcgatgtgatagatcggataagaaacgcgggtgttctcggtttttgaaccatcatcaaagtcga
tagtgccatcttcacgcacggtgacgttttccagcaacgcatcacgacggatagcgttgtagatttcaggttccgcttctttcgacagcttgatagttt
ttgcgtagcagccgccttcgaagttaaacacgccgtcatcgtcccagccgtgttcgtcatcgccaatcaggcgacgtttcgggtcggtggaaag
ggtggttttaccggtgccggaaaggccgaagaacaccgcaacatcgcctttctcaccaacgttggcggagcagtgcatagaagcgatacctttt
cagcggcagcaggtagttcatcatcgagaacatccctttcttcatttcgccgccgtaccaggtgccgccaatcagctgcatgcgctcggtcaggt
taaacgccacgaagttttcggagttgagaccctgttctttccactgcgggttagtgcacttcgcgccgttcataacgataaagtctggtttgaaacct
gccagttcttcatcgctcgggcgaataaacatgtttttgacaaaatgcgcctgccaggccacttcggtgatgaaacggacggaaagacgagta
tccgggttcgcaccacagaaagcgtcgacaacgaacagacgtttgccggaaagctgcctggtcaccaggcctttcagatgctgccaggtttcc
ggagagagaggtttgttgtcgttcttacctttgcctttgtctgcccaccagaaagtatcgcgagtggtatcgtcacggacgatatacttatcttttggtg
aacgaccggtgaagatcccggtatcgacggcaacggcacccagattagttaacaccccgcgctcataacctgtcaggctcggatcgagctct

tcctgatacagcaggtcgtagcttgggttgtaaacgatatcatgtacgtcactgataccataagcctcgagttcttgcggggtcaaaccattgttaa
cgcgcatttcactgctccttagccaatatgtattgcctgaatagtaaagtctttttgggggtgttaaccgcgacaaggctcatagatttacgtatctgg
agaaattcatatttgacaaaaaactccgtgattcctgtcacgaaacggttgctattatcgcagaaatagcattttaggtgtggaaaatgttctcaaa
aggttaaatcttggtttatatggcgggaatattgtgagtggaatcgcattcctgaatgaaattgatgcaaattcaaccggtaaagtatcgattcacc
acgctaactttttttgattattttctgaaaaaagcaaaatactactgcccggatgcgataattaacccaccttttgattctggataaccaacatggata
acgttgaactttcacccgcaacacgctgggggatgattgctaccggattacttcagggtctggtttgctacctgctgatcgcctggctttccggaaa
aaatcacagttggattgtttatggcgtgcctgcgaccgtcgcgttttcatccgttttactcttctccgtgatctcttttaaacagaagcgcctttggggat
ggctggcgctagtgtttattgccacgctggggatgagcggctggctgaagtggcaaactgatggcatgaacccctggagagctgaaaaggcg
ctttgggattttggttgctatctgctgttgatggcaatgttgttgctaccgtggatacaacaaagccttcgcatccgcaatgacagtagccgctatcg
ctatttttaccagtcagtatggcataacgtacttatattactggtgatttttctcgccaatggcttgacgtggctggtgcttttactgtggagtgagttgttt
aaacttgttggcatcacgtttttcaatacgctctttttgcaaccgactggtttatttatctcacgttaggtctggttaccgcgctggcggtgatcctcgcg
cgaacacagtcacgtttaatcgactctattcaaaagttgttcacgttaatcgccacggggttgctgccgttagtatcattgctaaccctgatgtttatc
atcaccctgccgtttacgggcctgagcgcgatttctcgccacatctccgccgccgggttgctgttgacgctggcctttttgcaattgatcttaatggct
attgtccgcgatccgcaaaaagcgtcacttccctggacagggccgttgcgttgcctgattaaaaccgctttgctggttgctccgctgtatgtgttcgt
cgccgcctgggcgttatggctgcgggtcgctcagtacggctggactgtcgaccgcttgcagggcgtgctggcggtgctggtgttactggtgtggt
cgctggggtatttgtcagcatcgtctggcgtaaagggcaaaatcccgttgttcttcagggcaaagtgaaccttgcggtttcgttattggtgttggtg
atactggtgcttcttaattcgccggtgctggacagtatgcgcattagcgtgaacagccatatggcgcgttatcagagcggcaaaaacacgtcag
accaggtaactatctacatgctcgagcagagcggtcgctatggacgtgctgcgcttgagtcgctgaaaagcgatgccgggtttatgaaagacc
cgaaacgcgcgcgggatctgctgatggcgttagatggagagcaacatcttcagcaacaggtatcggaaaaagtattagccgataatgtgtta
attgcccctggttctgttaaacctgatgcgacattctggtcggccttaatccaggatcgctataacgtgatgacctgtattgaaaaagacgcctgcg
tcctggtcgagcaagatctgaatagtgatggtcaggcggagcggatcctgtttgcttttaatgatgacagagtcattgtctatggctttgactcaga
cagaaaagaatggacgcgcttgatatgagtttacttccgaacgaaataacgaaagaaaaattactcacagctgcgaaggatggaaaactg
gggacgaagcctaaagcgtggcgcgatcttgtagtggatggtgaaaggctgaatgtgaatctgaatgagtgacaaagcgcatcaggtgtatt
agtcgtaaaatccggcagagccctctctgccggacatactcattaatgaacttgcggatctgccggagacgcgttgttgcggatttcagcaatat
ccatcgcattgaacagatagtggttaccgcagtaatcacaatgcatgtcaatttcgccatcttccgccaggatgctatcaacttcttcatcaggca
gcgttttcagcgcatcggcgcaacgttcacgcgagcaggtgcatttgaactccacatcctgcggatcgtaaaccgtcacctcttcttcgtgataca
aacgccacaacacttcgtttgccggtaaggtcagcagttcttcggttttgatggtttcggttagcgtcgccaggtggtcaaagtcgtcctgctgggc
attttgcgcaggcattacctgcaacaacataccgcctgcagccggtttgccgtctacgtcgccggtgcgaataaacaggcgcgtcggcagctgt
tcagaacgcataaagtaatcttccaggcaggccgccagggtatcaccttccagaccaactacgccctgatagcgttcgccttcgctcggggta
atggtgatcaccacgtaaccattgccgaccagcgttttcaggtcggcattttctggaatttcgccctgcacgcgcgccacaccgcgcatctgctg
gttattgttaccgttaataaccgccagattcatcggaccgtcgccctgcagctgtacggtgatatcaccatcaaacttcagcgtagcggttaacag
gctggtcgcaaccagcagttctgccagcacgtttttaacgggctgcggataatcgtggttctcaaggatctgttgcagggtttccgaaacggttac
cagttcgccgcgcacggcaaagttttcaaacagatagcgatgtaattggtcatgttgcggcataatcatctctcttgcaggtgacagttattcactg
tcgccgtgtttaaatcgtaacaggtcgcggcgctctttttgtccgggcgtcggtccgggtgcggcatggttaaggcattaagtttacgtgccagcg
ccatttttcgcgtttctctacactttccgcagtctcttcatacagcaaggctgcctcgctggcggggcgacgctgttcagtaatcgcctttacaatca
ccgtgcgttcgtcatttccctggcgcagagtgagcgtggcattcagctcgacgattttgctcggcttgctgcgctgcccgttgtaatgcaccttaccg
ccttcaatcatttcacgggccagcgcgcgggttttataaaaacgggcagcccatagccatttatccagtcgaacctcaacagcaggtttctctttc
atggcgtctccttcacattagcgaggggatcaggcggcggtagtcattcagtgacggatggcgttgatactgtttctcggcaatcccggaatcag
gattagtcacgccgaggcagtaacgaataccaaattgcgcggcagcatcgagaatcgcttcgctgtcatcaataaacagcgttctttcagctttc
agacccgtagcttcggccaccgcatgccataaccgctgatcctctttcggataaccaaatgtgtgggtggaaagtaataaatcaaggtgtgcgt
ccagaccggtatgctcaagttttaccgccaggttgtgcggatgcgcattggtgagcaaaattcgctgcttaccgctggctttcagtgcctcaagaa
acggaatggtatcttcacgcagtacggcacgcggtcccatctcggtggtcatcgcacagatatccagacccagttgctcactccagtaatcaag
acagtaccagtttagcgtatgctgtacgtcgtgatattgctggcgcatatattccatcgcttcctgtggcgtaaccccgtttttcgcgccccatgtttca
ggcaccagcttttgccagaaatagttatcgaaggcgaggtcgagcaacgtgccgtccatatccagcagaacggtatctacgtcctgccaggc
aatgttgatatgcatgagggaaatctccagagtgaagcaatttgcgcgacagggtagcataacctgccgcgcaaacgtgttattcgataaggc
tttctgaaggggtgatcagttgcgggttcaggcagctttcataataggcctgaatctccatcatgcgagtgcgatgacgctggtaacgacgccag
gcctgtacgccattgtaaatggcgctacccagcatcgtcagtagcagcaatgtggtgctgagatagcgccacaggccggaacgatccgggat
cggatgaaggccaatatgctgcgtaccattggcgtcagtgaagatttttgtgacgataccctcggcgttaaacggcgtatgcatcagcatttgtgc
cagtttctggaaagcgttccactgttcttgcggcgggtagtcgtaaagtgatgccgaaggccagggctgatcaacaaaatcgctgccttcgtcgc
tgacaatcaggaatccgccgggggccggactgtttagtgattgtgccgctcgcgccgtttcatgcgtgataaacggcgcggtggaggttgccac

caggttatccagcgattccgcactcaccgggcgtaataacacattcacgccatcgagcttcccggcgttggcgcgttttaccagcgcgtcccag
tctttactgttgccgagattgaccagcgcatttttcagtcgcacacagtcatctttggcagaacataaatccgctgtcttcagtacaatgtcgccaaa
atcatcaagcaataccatgccggatttttgaattgctgaacgtaatgaggcactgacgcgagattcatcttccggtttagggtgcagctggcgatt
aactgcttcagtcaatgccgtcgctttgttgaccagttcagattctggtaatggcaatgagcgggcgtcgttccagatgatctgcgagcagtcaaa
cggtaaaaaaggtgaattggttttcgcgctccaggttccggaagttcgaatattacacattcccgtaccgctaatacgcaatgtatcgcctacccg
cacgccagcgtcagccagttgttttacgctggtggcttcaatggtctgcgcgcctttcatccatgagagagtgaatttcagcggcatatccagcgg
gatccagaataacagcataaacagcaccagcagcgagcccgcagcgataatcgtactgcgcagccagtgctgtaacggaaagtttttttactt
catcatgcagcgaaagatatcgtccctgacgcactacatggcggtcgagatagatatcgatatcggtttgttgaccgagatcctgagcaatgtat
ggctgccagtgtgcgggatagaccaggtcgataataccgagtgaaatattgttgatctgttcctgatcgttttcgccaaacaatccccaacgacg
gggtgtaccgcgcagacaatgaatttcccgcagggaggattttgccgggggcgcgaatagcccccacagacctgcgcccagcagcagtaa
cgcgccgcctgccagccacggaacaaatacatccggggtaatcaggcagaaaaagaacatcaggaaggaggcgacgatcagcaacgc
ttcacgcagcccgcgcggacgactcagcgcatattcttcatgcgtttctttgcgaatattgagtagctcgatttgctcactctcttccccgcgaatcg
acgcctgcgttgacggaacaggttgcaaggcatagctgcgagtttcctgcatgtactcctgcagcgtatgaccgttgagtgaaataaccagcg
gcagcgaatcggtatgaatcagttcaacggtattctcatcgttgatgtactgttcccagaaagggggcaggtggacttctaccgaatcgaggtag
taacgccatttattcggatcgtcggtagagatgccgtaacgcgtgatagcgtgtgtcagcatcatgacgttgttgctttcggcattcagcgccaga
gagatcggtgccgcgctggctcccgttgggccaggcacctgtaatacctgcgtcaggctctcaagataattttcaacggcgctacgttcttcagg
tgtgagtttacgcgtttgcgcatccgcgaaggcgttggtccagggcagctgacgccgtctggatcgcacttttatcagccatcccgcaagtagtg
agcaggccagcaaagcagctaaaaaaatcacaatggtgctcatgctttccccatcttacttagtctgtcaggcgtggtcagtcgcaccggaca
caatacatgattctgcggttggctatcggcaagcgtggagtttaacttgagcatcattaagcacatcacagtaaccgctgatcatagcaaaccct
ctgaaggcggaatatcagcaaattcctggagttatttcaacctattgacttttaatttgatttgaggacgattatttattaagttacataaatgtaaata
acatgcaattcacattgccgaaagcgtgtccgatatcgcacaataaccgctgatttcacagcatagatcccacgatgagcaaatcattacaaa
aacccaccattctgaatgttgaaactgtagcccgttcccgactgtttaccgtcgagagcgtggatctggagttcagcaatggcgtgcggcgtgttt
atgaacgaatgcgtccaaccaaccgggaagcagtgatgattgtgccgattgtggacgatcacctgatcctgatccgcgaatacgcagtggga
actgaatcctacgaattaggtttttcgaaaggattaattgatccgggtgaaagcgtctacgaagccgctaaccgcgagctaaaagaagaggtt
ggatttggagcgaacgatctgactttttttgaagaagctcagcatggcaccgtcttacttttccagcaaaatgaatatcgtggtagcgcaagatctct
acccggaatcactggaaggcgatgagccagagccgctaccacaggtgcgctggccgctggcgcatatgatggatttgctggaagaccctga
cttcaatgaagcgcgcaatgtcagtgcgctgttcctcgtgcgcgaatggttgaaagggcaggggcgagtgtaaatgcgtgaagtttgcctgatg
cacaatgtttatcaggcttatggggtttctgcaatttattgagttttcacgattttgttagccggataacgcgttcacgccgtatccggcataaacaagt
gcactttgtcagcaaactgaaaaggcgccgaagcgcctttttaatcagaacaattcctgtgcctcgccattatcgataatggtcgttcccacctcg
tgcactgcctgttgtgtcggctgcgtaccttcgatgaaatactcttcgcggctgttgccaccattagctaactgcccggtgctgcgatcgatattcac
cgtcacaatacccggtggcggcgtcagcggctgctccggcacaccttcaagaacggctttcatataagcgtcccatgcaggctgggcactctt
ggcaccgccttcgtaacctgagatctgatctttaatcgctccggaagccgttgtatgaccgagattacgacggtgatcatcaaagccaatccag
accgaggtcacaacgcccggaccgtaacccgagaaccacgcatctttcgaactgttagtggtcccggttttcccgccgatatcgcgacgctgc
aaatcacgacctgcacgccagccagtaccctgccagcctggctcaccaaagatattggtgttcaaagcactcttaatcaggaatgccagcgg
agtgttgatgacgtgcggtgcgtactcctgcgcgccagtcttcgccactaacgcctgatttgcctgctccagctgcggcattggtacagaaacatt
ctgctgctcgcgggagatagcgacatcttcaacatcgttattttccagcacgttcgatttctgcgtatcaccgtaaatcaccggaatatcgcattcc
gggcaggctactttcggtttcgcttcgaaaatcacgccgccctgatcgttttcaattttgctgataaaccacgggtccaccaggaagccgccgttc
gccatgaccgcgtagccgcgcgccacctgcattggggtgaaggacgctgaacccagcgccagcgattcggtgtggacaatgttttgtgccgg
gaagccgaagcgttgcagatattctgcagcgtagtcgacgcccatcgcccgcattgcgcgtaccatcaccacgtttttcgactgacccagcccc
tgacgtaagcgaattggaccagcatactgcggtggtgagttcttcggctgccagtcagaaccggcacttgcatcccagcgagaaattggcaca
tcgttcaacatacttgccagcgtcagacctttatccatcgccgcggtgtagaggaacggtttgatgttggaacccacctgacgcagtgcctgggt
ggcgcggttaaacttgctctgattgaaatcaaagccaccgaccagcgccataacggcaccgttttgcggattgatcgacaccagcgccgagtt
cacttccggcacttgtgccagccaccatgcatcgccaacctgacgaacccagatttgctgacccgtttgcagaacatcggtcactttacgcggc
gtcggtccttgctgagtatccgaacggtaaggacgcgcccagcgaacgccttccatactcaatgcgacggtcgacccgtccgccagcatcgc
cgtcgcttgctgaggattggcgctggtgactgcggcaggcagcagcggaccataggttggcagcgccttcagcgtatcggtaatcttgttgttat
cccacgccgactcgcccactttccacagcacatttgccgggccgcgatagccgtggcgcatgtcgtagtccagcacgttattacgtaccgcctg
ctgcgcggcctgctgcactttgcgggtgatggtggtgtaaatgcgataaccgtcttcataggcactttcgccataacggttatacatctcctggcgc
accatttcgctcaggtacggcgcagagaaagcaatctccggcgcgtgatagttagcgttaatcgcctcagtgcgtgtctgatcgaactgttgttg
ggtgatatacccttcatccagcatccgcgacagcacgacgttacgccgcgcgacggcacgatccatcgagtagagcgggttgaaggtggaa
ggcgctttcggcagcccggctatcaccgccatttcgttcagcgtcagttggtcgaccgtttttccgaaatagacttgtgccgcagcaccgacacc

ataggcgcggtaaccaaggtaaatcttgttcagataaagctcgaggatctcgtctttcgtcagcagctgttcaatgcgaatcgcgaggaagactt
ccttaatcttacgcatcagcgtgcgttctggactgaggaagaagtttctcgccagctgctgggtaatggtacttgccccttgtgacgcgtgaccgg
agaacagcgccacgcttgctgcacggaagatccccaccgggtcaacgccgtgatgctcgtagaagcggctgtcttctgtcgcgataaaggct
ttcaccatctccggtgggatttgatccaacgtaaccggaatacgacgtttctcaccgtattgagcaatcagctcgccatcggcgctgtaaatctgc
atcggaatttgcaggcgaacatcttttaatgtcgccacatccggcagttgtggctcgatgtagcggtataggccataaatcgagcctgctcccag
cagaatgcaacagactgcaaggatcaaaaaatactttacgaacttcactggaaatttcccatttagtttcatttgggcagtttataaacaaacgcg
cggtagtataaaggcaagccagacgcattgatatacccgtcagagtgacgggtgataaggagatcatcacaatggcatttaagatctggcaa
attggtttgcatttacaacagcaagaagcggtagcggttgcgatcgtgcggggcgcaaaagaatgctttttgcaacgctggtggcggttgccgct
ggagaacgacattatcaaagatgggcggattgttgatgcgcagcagctggctaaaacgttgttaccgtggagtcgcgaactgccgcagcgtc
atcacattatgttggcgtttcccgccagtcgcacattacagcggtcatttccgcgcccgtcgatgtcccttggtgagcgggagcaaacggcctgg
ctgtcagggacgatggcccgcgagctggatatggatccggactccctgcgcttcgattacagcgaagactcactcagtcccgcctataacgtg
actgccgcgcaaagcaaagagctggcaacgctgcttactctggcagaaaggttgcgtgttcatgtgagcgcgatcaccccggatgccagtgc
attacagcgattcctgcctttttaccttctcatcagcaatgtctggcctggcgtgataacgaacagtggctgtgggcgacgcgctatagctgggg
cgcaaactggcggtggggatgactagcgcgaaggagctggcggcagcgttatccgttgatcccgaaagcgtcgcgatatgtggcgaaggc
ggatttgatccctgggaggccgtttctgttcgtcagccgccgctaccgccgccaggtggagactttgccatcgcgctggggctggcgcttggga
aggcgtactgatgaacccgccaattaatttttgccctggcgacagcaacgccggaccgcttttctgcgtttctggttgctgatgttcgttgcgcctct
gctgctggccgtcgggataacgctaatactgcgtctgacaggcagcgccgaagcgcgcatagacgcagttttgcttcaggcggaacaacaa
ctcgcccgcagcttacagataacgaagccacgtttgctggagcaacagcagttacgcgaacagcgttcgcaacggcagcgccagcgacaa
tttacccgcgactggcaatctgcgctggaagcactggcggcgcttttacccgagcacgcctggctgacaacgataagctggcagcagggaa
cgctggagatcaaggggctgacaacaagcattaccgcgttaaacgcactagaaacgtcactccgccaggatgcatcttttcatctcaatcagc
ggggagccacgcagcaggatgcgcagggacgctggcaatttgagtatcagttaacaaggaaggttagcgatgaacatgttctttgactggtg
gttcgccacatcaccccgcctccgccagctttgctgggcattctggttgctgatgttagttacgctcatttttctgtcatcgacacaccatgaagagc
gcgacgcattaattcgactacgggcaagtcatcaccagcagtgggccgcactgtatcgcctggtagacaccgctcccttcagcgaggaaaa
aacgctgcccttttcgccactggattttcagttatccggcgcgcaactggtttcctggcatccatccgcgcagggaggcgagttggcgttgaaaa
cgctgtgggaagcagtgccgtcggcatttacacggctggcagagcgcaacgtcagcgtgagccgtttttcgttaagcgtggaaggtgatgatct
tttgttcacgctacaactggagacgccgcatgagggttaaacgctggttgttggcaggtattgcattgtgccttttaaccggtatgcgtgaccctttta
aaccgccggaagatctatgccggattagcgaacttagccagtggcgctatcaggggatggtagggcgaggcgagcgcatcatcggtgtaat
aaaagacgggcaaaagaaatggcgacgggtgcagcaaaacgatgtgctggaaaacggctggacaattttacagctgacgccagacgta
ctaacgctgggtaccgggacaaactgcgaaccgccacaatggttgtggcaacggcaaggagatacaaatgaagcaatggatagccgcac
tactgttgatgctgatacccggcgtacaggcggcaaagccgcaaaaagtgacgctgatggtggatgacgttccggtagctcaggtgttgcagg
cgctggctgaacaggagaagttgaacctggtcgtgtcgccagacgtcagcggtacggtgtcgttacatctaacagatgttccctggaagcagg
cactacaaactgtagtgaaaagcgccggactgataacgcggcaggaaggcaacattctctcagtgcattccattgcctggcagaataacaat
atcgcccgccaggaggcggagcaggcgcgggcgcaggcaaatctgccgctggaaaatcgcagtataaccctgcaatacgccgacgcgg
gagaactggcgaaagcgggggagaagctactgagtgccaaagggagtatgaccgtcgataaacgcaccaatcgccttttgctacgagata
acaaaacggcgttaagcgcgcttgaacagtgggtagcgcaaatggatctgccggtcgggcaggttgagctgtcggcgcatattgtcaccatta
atgaaaaaagtttgcgtgagttaggcgtgaaatggacgctggccgatgcgcaacacgctggtggcgttgggcaagtcaccacgcttggtagc
gacctctccgtagcgacggcgacaacgcatgtcggttttaacattgggcgcatcaacggacgcttgctggatcttgagctttccgcgctcgaac
aaaaacagcagctggatattatcgccagtccgcgtctgctggcctcacatcttcagcctgccagcattaaacaggggagcgaaattccatatc
aggtttccagcggggaaagtggcgcgacgtcggtggaatttaaagaggccgtcctggggatggaggtcacgcccacggtgttacaaaaag
gtcgcatccggctgaaattacacatcagccagaacgttccggggcaggtgctacagcaggccgatggcgaagtgctggcgattgataagca
ggagatcgaaacgcaggtcgaggtcaaaagcggagaaacgttggcgctgggcggcatttttacccgtaaaaataaatcgggtcaggatag
cgtaccgttgcttggcgacattccctggttcgggcaattatttcgtcatgacggaaaagaagatgaacgacgcgagttagtggtgtttatcacgcc
acgactggtttccagtgagtaaacagccgtaaaagcggtaatgtttttacgctgaacgtgtttcatctatttgacgcgcgcaggtatttagcataca
aggagtaccgatttgagagttggtgctcttcgctgcctgcgttccatgatgatgatttatcattcaggcggcattttgctgtctttttacgctaatcttac
ccggtgatttatcgccagagcggtggtagcaaggcagcgcgcttgcagcgaccagatatgcagagggatgggtgatttattcagttgccaaac
ccgctggagtattgagataattttcagtctgactctcgcaatatcttatgaggtttcagttcatgtcctgcggcgctctctgagcgaagcgggtttatc
attaacgaatagtcttagtagtaccgaaaaaatggcagagaaacgcaatatctttctggttgggcctatgggtgccggaaaaagcactattggg
cgccagttagctcaacaactcaatatggaattttacgattccgatcaagagattgagaaacgaaccggagctgatgtgggctgggttttcgattt
agaaggcgaagaaggcttccgcgatcgcgaagaaaaggtcatcaatgagttgaccgagaaacagggtattgtgctggctactggcggcgg
ctctgtgaaatcccgtgaaacgcgtaaccgtctttccgctcgtggcgttgtcgtttatcttgaaacgaccatcgaaaagcaacttgcacgcacgc

agcgtgataaaaaacgcccgttgctgcacgttgaaacaccgccgcgtgaagttctggaagcgttggccaatgaacgcaatccgctgtatgaa
gagattgccgacgtgaccattcgtactgatgatcaaagcgctaaagtggttgcaaaccagattattcacatgctggaaagcaactaattctggct
ttatatacactcgtctgcgggtacagtaattaaggtggatgtcgcgttatggagaggattgtcgttactctcggggaacgtagttacccaattacca
tcgcatctggtttgtttaatgaaccagcttcattcttaccgctgaaatcgggcgagcaggtcatgttggtcaccaacgaaaccctggctcctctgtat
ctcgataaggtccgcggcgtacttgaacaggcgggtgttaacgtcgatagcgttatcctccctgacggcgagcagtataaaagcctggctgtac
tcgataccgtctttacggcgttgttacaaaaaccgcatggtcgcgatactacgctggtggcgcttggcggcggcgtagtgggcgatctgaccgg
cttcgcggcggcgagttatcagcgcggtgtccgtttcattcaagtcccgacgacgttactgtcgcaggtcgattcctccgttggcggcaaaactgc
ggtcaaccatcccctcggtaaaaacatgattggcgcgttctaccaacctgcttcagtggtggtggatctcgactgtctgaaaacgcttcccccgc
gtgagttagcgtcggggctggcagaagtcatcaaatacggcattattcttgacggtgcgtttttttaactggctggaagagaatctggatgcgttgtt
gcgtctggacggtccggcaatggcgtactgtattcgccgttgttgtgaactgaaggcagaagttgtcgccgccgacgagcgcgaaaccgggtt
acgtgctttactgaatctgggacacacctttggtcatgccattgaagctgaaatggggtatggcaattggttacatggtgaagcggtcgctgcgg
gtatggtgatggcggcgcggacgtcggaacgtctcgggcagtttagttctgccgaaacgcagcgtattataaccctgctcaagcgggctgggtt
accggtcaatggccgcgcgaaatgtccgcgcaggcgtatttaccgcatatgctgcgtgacaagaaagtccttgcgggagagatgcgcttaa
ttcttccgttggcaattggtaagagtgaagttcgcagcggcgtttcgcacgagcttgttcttaacgccattgccgattgtcaatcagcgtaacaaca
agaaaggtcaggccgcttatcaagcggtctattagcttcaggttaattgcaacgtggtaagcattaaccttttagtggggtgttaaatggatgaatt
caaaccagaagacgagctgaaacccgatcccagcgatcgtcgtactggtcgttctcgtcaatcttctgaacgttctgagcgtactgaacgtggc
gaaccgcagatcaattttgatgatattgaacttgatgacactgacgatcgccgtccgactcgtgcgcaaaaagagcgcaatgaggaaccgga
aatcgaagaagaaattgacgaatccgaagatgaaaccgtggatgaagagcgcgtagagcgtcgtccgcgtaagcgcaaaaaagcagcc
agtaaacccgcttctcgtcagtatatgatgatgggcgtcggcattctggttctactgctgttgatcatcggtatcggttctgcgctaaaagccccctc
gaccacttccagcgatcaaaccgcgtctggcgagaagagtattgatcttgctggcaatgcgaccgatcaggcgaatggcgtgcagccagcg
ccgggaaccacgtctgcggaaaatactcagcaggatgtttctctgccaccgatctcttctacgccgactcaagggcaaaccccggtggcaac
ggatggtcaacaacgtgttgaagtgcagggtgacctgaacaatgcgctgacccagccacaaaatcagcaacagctgaacaatgtggcggt
caattccacgttgccgactgaacccgcaacggttgcgcctgttcgcaatggcaatgcatcgcgtgacacggcgaaaacgcaaaccgctgaa
cgtccgtccactacgcgcccagctcgtcagcaggcggtgattgaaccgaaaaaaccgcaagcaaccgtgaaaacggagccgaagccggt
agcacagacgccgaagcgtactgaaccagctgcccctgtggcgagcacgaaggcaccggctgcgacttctacgccagcaccaaaagag
acggcgactacggctccagtacagacggcatccccggcgcaaaccacagcaacacccgctgctggggcgaagaccgcaggtaatgttgg
ttcgttgaaatcggcaccgtccagccattacactctgcagctgagcagttcctctaactacgacaacctgaacggttgggcgaagaaagagaa
tctgaaaaactacgttgtctatgaaacgacgcgtaatggtcagccgtggtatgtcctggtttctggcgtgtacgcttcgaaagaagaggcgaaa
aaagcggtatctacattgccagcagatgtccaggccaaaaacccgtgggcgaaaccgctgcgtcaggtacaggccgatctgaagtaatcaa
ggttatctcccgcaatggtttatcgttgcgggagttgcctgaagcgctggatgctgtcggagctttctccacagccggagaaggtgtaattagttag
tcagcatgaagaaaaatcgcgctttttttgaagtgggcagggggcaagtatcccctgcttgatgatattaaacggcatttgcccaagggcgaatg
tctggttgagccttttgtaggtgccgggtcggtgtttctcaacaccgacttttctcgttatatccttgccgatatcaatagcgacctgatcagtctctata
acattgtgaagatgcgtactgatgagtacgtacaggccgcacgcgagctgtttgttcccgaaacaaattgcgccgaggtttactatcagttccgc
gaagagttcaacaaaagccaggatccgttccgtcgggcggtactgtttttatatttgaaccgctacggttacaacggcctgtgtcgttacaatctg
cgcggtgagtttaacgtgccgttcggccgctacaaaaaaccctatttcccggaagcagagttgtatcacttcgctgaaaaagcgcagaatgcct
ttttctattgtgagtcttacgccgatagcatggcgcgcgcagatgatgcatccgtcgtctattgcgatccgccttatgcaccgctgtctgcgaccgcc
aactttacggcgtatcacacaaacagttttacgcttgaacaacaagcgcatctggcggagatcgccgaaggtctggttgagcgccatattccag
tgctgatctccaatcacgatacgatgttaacgcgtgagtggtatcagcgcgcaaaattgcatgtcgtcaaagttcgacgcagtataagcagcaa
cggcggcacacgtaaaaaggtggacgaactgctggctttgtacaaaccaggagtcgtttcacccgcgaaaaaataattctcaaggagaagc
ggatgaaacagtatttgattgccccctcaattctgtcggctgattttgcccgcctgggtgaagataccgcaaaagccctggcagctggcgctgat
gtcgtgcattttgacgtcatggataaccactatgttcccaatctgacgattgggccaatggtgctgaaatccttgcgtaactatggcattaccgccc
ctatcgacgtacacctgatggtgaaacccgtcgatcgcattgtgcctgatttcgctgccgctggtgccagcatcattacctttcatccagaagcctc
cgagcatgttgaccgcacgctgcaactgattaaagaaaatggctgtaaagcgggtctggtatttaacccggcgacacctctgagctatctggat
tacgtgatggataagctggatgtgatcctgctgatgtccgtcaaccctggtttcggcggtcagtctttcattcctcaaacactggataaactgcgcg
aagtacgtcgccgtatcgacgagtctggctttgacattcgactagaagtggacggtggcgtgaaggtgaacaacattggcgaaatcgctgcgg
cgggcgcggatatgttcgtcgccggttcggcaatcttcgaccagccagactacaaaaaagtcattgatgaaatgcgcagtgaactggcaaag
gtaagtcatgaataagtttgaagatattcgcggcgtcgcttttgatcttgatggtacgctggtcgacagtgctcctggtcttgctgctgcggtagatat
ggcgctgtatgcgctggagttgcccgtcgcaggtgaagaacgcgttattacctggattggtaacggcgcagatgttctgatggagcgcgcattg
acctgggcgcgtcaggaacgtgcgactcagcgtaaaacaatgggtaaaccgcccgttgatgacgacattccggcagaagaacaggtacgt
attctgcgtaaactgttcgatcgctactatggcgaggttgccgaagaggggacgtttttgttcccgcacgttgccgatacgttgggcgcgttgcag

gctaaaggcctgccgctaggcctggtcaccaacaaaccgacgccgttcgtcgcgccgctgctcgaagccttagatatcgccaaatacttcag
cgtggtgattggtggtgatgatgtgcaaaacaaaaaaccgcatccggacccgctgttactggtggctgagcggatgggaattgccccacaac
agatgctgtttgtcggcgactcacgcaatgatattcaggcggcaaaagcggcaggttgcccatcagttggcttaacctacggatataactacgg
cgaggctatcgatctcagccagcctgatgtaatttatcagtctataaatgaccttctgcccgcattagggcttccgcatagcgaaaatcaggaatc
gaaaaatgactaagcccatcgtttttagtggcgcacagccctcaggtgaattgaccattggtaactacatgggtgcgctgcgtcagtgggtaaa
catgcaggatgactaccattgcatttactgtatcgttgaccaacacgcgatcaccgtgcgccaggatgcacagaagctgcgtaaagcgacgct
ggatacgctggccttgtatctggcttgtggtatcgatcctgagaaaagcaccatttttgttcagtcccacgtgccggaacatgcacagttaggctg
ggcactgaactgctatacctacttcggcgaactgagtcgcatgacgcagtttaaagataaatctgcgcgttatgccgagaacatcaacgctggt
ctgtttgactatccggtgctgatggcagcggacatcctgctgtatcaaactaatctggtaccggtgggtgaagaccagaaacagcacctcgaac
tgagccgcgatattgcccagcgtttcaacgcgctgtatggcgagatctttaaggtgccggagccgtttattccgaaatctggcgcgcgcgtaatgt
cgctgctggagccgaccaagaagatgtccaagtctgacgataatcgcaataacgttatcggcctgctggaagatccgaaatcggtagtgaag
aaaatcaaacgtgcggtcactgactccgacgagccgccggtagttcgctacgatgtgcagaacaaagcgggcgtttccaacctgttggatatc
ctttcagcggtaacgggccagagcatcccagaactggaaaaacagttcgaaggcaagatgtatggtcatctgaaaggtgaagtggctgatg
ccgtttccggtatgctgactgaattgcaggaacgctatcaccgtttccgcaacgatgaagccttcctgcaacaggtgatgaaagatggcgcgga
aaaagccagcgcgcacgcttcccgtacgctaaaagcggtgtacgaagcgattggttttgtggcgaagccgtaattcactattgctggcaaattg
cgctttgttcatgccggatgcggcgtgaacgccttatccggcctacaaagtaatgcacatattgcaaggaaatgtaggcctgataagcgtagcg
catcaggcaattttgcttttatcatcactctgaaaccggggaaacccggttttttatcctcatttgcaataatccgaaaaaatgtgaagtgcctcgcc
gtttccacatctcgctgttgcgccttatcttcaaattcctgaagatataaatattcaatttactgaaaatttattatgcgtcgaacgtttatcaaaaaaga
aggcgtcgtcatcacgacgctggcccgttatttgttgggtgaaaagtgcggtaatcgattgaaaaccatagatgagctggcaaatgaatgccgt
tcatccgttggcctgacgcaggccgcgttgaaaacgctggaatcaagcggagcgatacggattgaacgccgtgggcgcaatggcagttatct
ggtcgagatggataacaaagcattgctgactcatgtggatatcaacaacgtggtatgtgcaatgcccttgccctatacccgtttgtacgaaggcc
tggcgagcggattgaaagcccagtttgatggcattcctttttactatgcgcacatgcgtggcgcggatattcgcgtggagtgtctgcttaatggcgt
gtatgacatggcggtggtttcgcgactagcggcggaaagttatctcacgcaaaaaggcttatgcctcgcgctggagttggggccgcacaccta
cgttggcgagcaccagttgatttgccgtaaaggcgagtccgcaaacgtgaagcgcgtggggctggataaccgttcggcggatcagaaaatc
atgaccgatgtttttttggcggtagtgatgtggaacgagtcgatctctcttatcacgagagtttacaacgcattgttaaaggcgatgtcgatgcggt
gatctggaacgtggtggcggaaaaacgaactgaccatgctgggattagaggcgacgccgctcacagacgatccgcgatttttacaggccacc
gaagccgtggtcctgacgcgagtcgatgattacccaatgcaacaactgctgcgtgccgttgtagataaacacgccctgctcgcccatcaacaa
cgggtagtgagtggggaacaggaaccgagttattaaacgaaaggcatctgatatggaaaccagactcaacctgctttgcgaggcaggcgtt
attgataaggacatctgcaaaggcatgatgcaggtcgtcaacgtactggaaacagagtgccatctgccggtgcgcagtgagcaaggaacga
tggcgatgacacatatggcgagtgcactgatgcgcagtcgccgtggtgaagaaatagagccgctggataacgagttgctggcagaactggc
gcaatccagccactggcaagccgttgtgcaattgcatcaggtgttgttgaaggaattcgcactggaagttaacccgtgtgaagaaggctatttgc
tggccaacctttatggattatggatggctgctaacgaagaggtttgaagctcccgacaaatccacgtcgcacaacctaaatattcaaaaaaatg
aatatttaattcaaggaacagaaacgaggcaacaagatgtttgtagaagcattgaaacgccagaacccggcgctgatttccgccgcactaag
cctgtggcagcagggcaagatcgccccggacagctgggtgatcgacgtggatcagatactggaaaacggtaagcggctgattgagacggc
gcggctttacggcattgaactgtatctgatgaccaagcagtttggtcgtaatccgtggctggcggaaaaattgctggcattaggctacagcggca
ttgtggcggtggattacaaagaggcgcgagtcatgcgcccgcgctggtttgcctgtggcgcatcaggggcatctggtacaaatcccttgtcatca
ggttgctgacgccgttgaacagggcaccgacgtcatcaccgtgtttactctcgacaaagcgcgggaagtttctgcggcggcggtgaaggccg
ggcgaatccagtctgtgctgcttaaagtttatagcgacgatgatttcttatccgggccaggagagcggttttgccctcaaggtgttgccggagatt
gtcgccgaaatccagaatctgccagggctgcatttagccggacttacccatttcccttgcctgctttgggatgaggctgtcggaaaagttttgccg
acaccgaatcttcacacgctgatacaggcacgggatcaactggcgaaatctggtattgcacttgagcaactgaacgcgccttcagcgaccag
ctgcacttcgctgccattactggcgcaatacggtgtgactcatgccgaacccggtcatgcgctgacgggcactattccggcaaaccagcaggg
cgatcagcctgaacgtatcgcgatgctctggttaagtgaaatctcccatcatttccgtggcgacagctactgctacggcggcggttactatcgtcg
tggtcatgcgcaacatgcgttggtgtttacgccagaaaatcaaaagattactgaaaccaatctcaaaactgtggatgacagcagtatcgactac
accctgccgctggcaggcgagtttccggtaagcagtgcagtggtgctctgttttcgcacgcagatttttgtcacccgtagcgatgtggtgctggtgt
ccggtattcatcgtggcgaaccggaaatcgtcggtcgttatgacagtcttggaaactctctgggggcgtaatggcgcgatttgtggtgttagtgatt
gatagctttggcgtaggggcaatgaaagatgtcacgctggtgcgtccgcaagatgcgggagcgaatacatgtggtcacatcctgagccagttg
ccgcatttgcagctaccaacgctggagacgctggggctaatcaacgcattgggttatgcgccaggcgatatgcagccgtcagattccgcaac
ctggggcgtggcagagctgcaacatgaaggtggcgataccttttatggggcatcaggaaattttaggcacgcgcccgttaccgccgctgcgga
tgccttttcgcgatgtgattgaccgtgttgagcaggcattagtttccgctggctggcaggtggagcgccgtggcgatgatctgcaatttctgtgggtc
aatcaggcggttgcgattggcgataatctcgaggcggatttaggccaggtctataacattaccgccaatctctctgtgatctcttttgacgacgca

atcaaaattggtcgtatcgtgcgtgagcaggtacaggtcggtcgggtcattacatttggtggcctgttaaccgacagtcaacgcattctcgatgcc
gcagaaagcaaagaagggcgctttattggtatcaatgcgccgcgttctggcgcttatgacaacggtttccaggtcgtgcatatgggctatggcgt
cgatgaaaaagtgcaggtgccacaaaaactgtatgaagcaggcgtgccaaccgtgctggtgggtaaggtggcagatatcgtcaacaatcctt
atggcgtgagctggcaaaatctggtggatagccagcggattatggatatcaccctcaacgaatttaacacccatccgacggcgtttatttgcacc
aacattcaggaaaccgacctcgctggtcatgcagaagacgtcgcacgttatgccgaacgtttgcaggtcgttgaccgtaaccttgcccggcttg
ttgaggcgatgcagccagatgattgcctggtcgtgatggcggatcacggcaacgatccgaccattggtcacagccaccatacccgcgaagtg
gtgccagtgctggtttatcagcaagggatgatcgctacgcagctcggtgtgcgcaccacgctttctgatgtgggggctaccgtgtgtgaatttttcc
gcgcgccaccgccacaaaatggtcgctcttttctttcctccctccggtttgcaggagacaccctatgagttttgatccgacgggttacaccctcgcc
catgagcatctgcatattgatctctccggctttaaaaacaacgtggactgccgccttgatcagtatgcgttcatttgccaggagatgaacgacctg
atgacccggggcgtgcgtaatgtgattgagatgaccaaccgttacatggggcgcaatgcgcaatttatgcttgatgtaatgcgcgagacgggg
atcaacgtggtggcctgtaccggttattaccaggacgcgtttttcccggaacatgtggcgacccgcagcgtgcaggaactggcgcaggagatg
gtcgatgaaattgaacagggtatcgatggcacggagctgaaagccgggatcatcgcggagatcggcaccagcgaaggaaagattacgcc
gctggaagagaaggtatttattgccgctgcgctggcgcataaccagaccggacgcccgatctccacgcatacgtcgttcagcacgatggggc
tggagcaactggcgttgctacaagcccacggggttgatctttcgcgcgtcaccgttggtcactgcgatctgaaagacaacctcgacaacattttg
aagatgatcgatctcggcgcgtacgtgcagttcgacaccatcggcaagaacagttactacccggacgaaaagcgtattgcgatgcttcatgcg
ctacgcgaccgtgggttgctgaaccgcgtcatgctgtcgatggatattacgcgccgctcccatttaaaagccaacggtggttatggctatgactat
ttactaaccacctttattccgcaattgcgccagtcaggattcagtcaggccgatgtggatgtgatgttacgtgaaaatccctctcaatttttccaataa
ggacagactcatgaaaaagattggcgttgcaggcttacagcgtgagcagattaaaaaaactattgaagcgacggctcctggctgttttgaagtt
ttcattcacaacgacatggaagcggcaatgaaggtgaaatccgggcaactggattattacatcggcgcgtgtaataccggtgcgggcgcggc
attgtcgattgccatcgcggtgataggctataacaaaagttgcaccattgccaaaccaggcattaaagcgaaagacgagcatatcgccaaaa
tgatcgctgaaggaaaagtggcgtttggcctttccgttgagcacgtcgaacacgcgattccgatgctgattaaccatctgaaataaaaggcacg
actatggatctgtatattcagattatcgtggtggcgtgcctgacgggtatgacatcgcttctggcgcatcgctcggcggctgtttttcatgacggcat
ccgcccgatcctgccgcaactgattgaaggctatatgaaccgtcgcgaggcggggagtatcgcttttggtctgagcattggttttgtggcctcggt
ggggatctcttttaccctgaaaaccgggctgctcaacgcatggttactctttcttcctaccgatatcctcggcgtcctggcgataaacagcctgatg
gcgtttggtcttggcgctatctggggcgtgttgatccttacttgcctgttgccagtaaaccagctgctgaccgcgctgccggtggatgtattaggtag
cctgggggaattaagctcgccggtggtttcagcttttgcactgttcccgctggtggcgattttctaccagtttggctggaagcaaagtctgatcgccg
ccgtggtggtactgatgacccgtgtggtagtcgtgcgctatttcccacatcttaaccctgaatccatcgaaatctttattggcatggtgatgctgctgg
ggatcgcgataactcacgacctgcgtcatcgtgatgaaaatgacattgatgccagcgggctttcggtgtttgaagaacgcacgtcacggattat
caaaaacttaccctatatcgccatcgtgggagcattgattgccgccgttgccagcatgaagatttttgctggcagtgaagtgtcgatcttcacactg
gagaaagcatattccgcaggcgtaacgccggaacaatcgcaaacgctgattaatcaggcggctctggcagaatttatgcgcggactggggtt
tgtgccgttgattgccaccaccgcgttagcaacgggtgtgtatgcagttgcgggctttacctttgtttatgcggtggactatctctcgccgaatccgat
ggttgcagcggtattaggcgcagtggttatttcggcggaagtcttgctgcttcgttcgatcggcaaatggctgggacgctacccgtcggtgcgtaa
tgcgtcggataacatccgtaacgccatgaatatgctgatggaagtggcgctgctggtcggttcgattttcgcagcaattaagatggcgggttatac
cggattctctatcgcggttgccatttacttcctcaacgaatccctgggccgtccggtacagaaaatggcggcaccggtcgtggcagtaatgatca
ccggtattctgctgaatgttctttactggcttggcctgttcgttccggcttaaggaaggcacctatgaagacgtttcctctgcaaagcctgacgattatt
gaggcgcagcaaaagcagtttgcgctggtggatagcatttgtcgccatttccccggcagcgagtttcttactggcggtgatttaggcttaacgcc
aggactgaatcaaccgcgcgttacccagcgtgtggaacaggtgctggctgatgcatttcacgcacaggctgcggcgctggtgcagggcgcg
gggactggcgcgattcgcgccgggctggcggctttgctcaaaccggggcagcgtcttctggtgcatgacgcgcctgtttacccgacgacacgg
gttattattgagcagatggggctgacgcttattactgttgatttcaatgacctgtcggcactgaagcaggtcgtcgacgagcaacaaccggatgc
ggcgctggtgcagcatacgcgccagcagccgcaggacagctacgtgctggcagatgtgctggcaacgttgcgcgcggcaggtgttccagc
gttaaccgatgacaactatgcggtgatgaaggtggctcgaatcggctgtgaatgcggcgcgaatgtctcgacattttcctgcttcaagctatttgg
gccagagggtgttggtgcagtggtcggcgatgctgatgttatcaaccgtattcgcgccacgctttactccggcggtagccagatccagggcgca
caggcgctggaagtattgcgtggtctggtgtttgcgccagtgatgcacgcggtgcaggcaggggtatctgaacggttgctggctttgcttaacggt
ggtgcggtgccggaagtgaaaagcgcggtgattgctaatgcgcagtcgaaggtgttgattgtcgagtttcatcagccgattgccgccagagtgc
tggaagaggcgcaaaagcgcggtgccttgccttacccggtgggtgcagagtcgaaatatgaaatcccgccgctcttttatcgcctttccggaac
gtttcgccaggcgaatccacaatcagaacattgtgcgattcgcattaacccgaatcgcagcggtgaagagacggtgctgcggattttgcgtga
gagtattgccagtatttaatgctggatagcaccgtcaagctaaattccgtactgaacggtcccctcgcccctttggggagagggttagggtgagg
ggaacgtacgggatgtaaaattcaacgcgccagcatcgccggatgcgatgctggcgcatcttatccggcctacggttaatgtcttttattatcaat
agttaaggtgatgcgattggcgcggcagaagagttcggagacgtgcaccggtttgtcattctgatcaaaggcgattttgctgatgcgaaacaac
ggttcgcccagttcgcatttcagccattttgcctgcgggcgggtggcggcaaagatatcgatggtctttttgtcgctgaccactcgcgtgtcaaaac

gctcctgaaataactgataggtggagcttccttcgacgtaaatctcatcaaagtcaggataacgcgacagcgggatccatgaactatcaataa
acagcggctctttatcgagatacatcacccggcagagatggaacacttcgctgtttccggggatgttcagcttttcgcaaaacggcgcggcgct
gacccgttcctgttcgatcactttctctttcgtcgccttgccttgtgagacgccaaaatcggtaaaaccactgacagtaagcagggcgttttcaactt
tctggctttgtacaaaggtgccttttccctgccagcggatcagtacgccgtctgccactaagtcgctgatggctttgcgaatggtaatgcggctgac
gttatattgtgtacaaagctcgttttcggtagggatctgttgcccggcctggtaaacccccctgcgcgatatcatccagcagtcgctggcggacggt
agcgtagaggagttgatgagagtagcggtccgtagctgacataatcgaccctgaaataaagaatgaattactcggattatagggtcgttttccg
gcaagtaacagcacgttggcgtgaagatgctcacgccaacgttatacctaccaggcaccgtggtactgaatggttttcgccgcgcacgccgtt
ccctgcgctatcgcctgcggtaatgtcatccccgcagaccagccgcaaaggaatccggcaatgaacgaatctccggcacccatggtgtcgat
aaccgtcaccggttcaggagcctgacgccagaactgcgcgccatcccaggcaatgctgccgttttcacccagcgtgacaatcactgttcctgc
gccacgggcaacaatcgctttcatcttcagacgcagcgtttcgtcttcttgcggtgcggaggcaaaggcaaaatcgagatgcggcaccagtgt
ctgccagagcgggctgtcccacttgtcggagaagtcgaaagcggtaagtttgcccgcagcgtgcagctgtgggaatgcgtcttccgcatgtcc
ccagattgccgcgtgcacaatgtcatactgcgccagccaggcgtaatcctcttcactcagggcaaagtcggccatcacgccttcggtgtagtcg
ccaaaaacgcgatcattgtcgtgcagttccacctgagtttgtgcggtaacgccgtgtttcgtatggacatggctgatatcgacgcccatgcgggc
gagatcctgcttcagctttgtgccgtagtcatcgtcacccacccaggtaatgcatcccggctgtatgccgtagcgagtgcagtacaccgccacat
tgaccgcattaccgccagaaaacgctttattcagttgcgggtagatatcgacgcagttatcgccgattgtcgccagggttttcatctctcatcctcc
ggcagcagcgcgcgaaagcgttccagcgcctggcgggcatagagtctgggctcgttcatatacatcgtcaccagctccaccgtacagtaacc
ctcatagccccgctcaataatatcgcgcatcagttcccgcagcggcattttgccttctccaggaatgtaatgcgtgtcgctggccccgtcgctgtc
gacaatatgcagatgacgtaatttatcgcccagtttgtcgaaataactcatcaccggttccgcctggacatacggcgcgcaaatgtcgaccatg
ctgaacaggcgcggcgaaggcaccagcgccagcgcatgaagcacatcattagcattacacacgacgttcgattcatacggcgttaacggtt
cgaggattagatccatgccgatgttttccgcgtactcacacagttcgcttaggttctctgccagccgtccccagataacattaggtggcgtgagat
agcccgcgtgggccgcggaaatcagcgtataacccgcgttcatctcttttgccatatccatcgccagcttgatcatgtcgaggctttcgcgacgc
atatgttcatcgcccagcatcatgttatacggatagccgttggtttctggcgtatagccgataatcggcatctgatacgtctgcgccagcgccttgat
ttgtttgatgccgcccgcttttaagtccggcgcgaacgcgtgcgggcgaccgccccaaatttcgatgccgtcgtaacccagctcgcttgcatcac
gaaatgcgtgttcaataggcagccgctggtggccgcaggtaaacatacctgttttcatcgggtaactccttgcgtaaatccggcccgttgccggg
ccgggagagggattaatattccaccagaccaccgtaatagcggcgttcatccgggttgtgatctttgtaaatagacaggtagtagcagagcca
ctccattggcacgaacatcaggaacggtgccagccacgggtgcagcccttgcgaaatttcggcgtaatcgatgacgatcacgttgtcagtacg
ctgtttaacaaagttaatggcgcgttcggtggtgtggcgactttcatcattgccgagcaggaacaggaacggaacgcccggttcgacaatctcc
agcgggccatggcggaactctccgctctcaatcacgcagccgtgcgtccaggtaaattccatcagcgttacaatgccttctttgtaacccagcg
gacgcagcggacccgcagcaacggtataaatcatcggccactggctggccagttcaccaagctggcggcctttttcttcccaggtgcgtacca
gatgaccgagcgcattcggcaactgcttgagatcgtttttgatcttgccgatttccgcgttcggcgcgaggcgggtgatcatctccagcaccacg
ctgtagcagagcagcaggtgaatttcccagatacagtcggcctgataatcaatgctaaattccgccgccgaggtaatcgggctatccgcgcgtt
tggtgaacgccgcagtgagtgcgccgcaggcccggcccagctccagcgctttgattacctcttcggttttaccgtagtcagaaacgccaattact
gcgcaacgatcgtcgaggcgatacggggtgttatcgcagaactcccagccggaaatggcgtagacctgaagatcggaaaagcgatccgcc
agatgtttcgccgtttgcgccgcgttgagtggcgatccgcaggcaacgaaataaatacgatcaatgtcgcgcttcaccatctcttccacgatggc
gtgcaccagcggaacgtcatggctgagaactttttccacttcctgaaccatattttcggtgaccagaaagtccacggtgcttttatcaatatccaac
attttattttctccagataacattacgacaaagcgttgagctggcggctacgcttcgcccagaaggcgtaagcaggcagaccagtagcaataa
cgatgacggcgcagataaggccgggaatcggtgcccagacaaaggttgaggcgaccagaatgaggcttgacgcaatggcgagggtggtc
atcagcccgaaagccggagtacgccacagcggtttgtaatcgtcgcgtttacgacaccagatgatggagccgaaggtgagggtatttttgaaa
cacatcaccagggtgaaataacccagcaggctggtgagatcggaaacgaagatgaagaagatccccagcgccccttgcaggatgatgga
gacatccggcgtgttgtatttcggatgcacatggccgaagcatttaaagaacaggttgtctttcgccatcgcgtattccagccgcggctggtacat
cacgcagctggaaagcgaaccaagaatgacgatcatcgccgtgatggcaacaaagatcccagcggtgctgccgagtgcggggatccagg
tcagggcgtcggaaatcggcgtttcagaattggcgagtttgtcgaagggcatcaggccggaaatcaccagcgccagcagggtgtagagcac
cagaaccagcagacaggaaccaatcagcgctcgtggcatggtttttccggggtttttaatttcgccggtcatataacagatagaggccatgccg
gtatacgaccaactggtggcagagatccccgccagcagcgccataaagctgcccgttgcgccaatcgcagtggtggtaggggcggcaaaa
ttctccgctttaaaccagaagatcccaaggccaatgacgatagtgaacgggataattttggcgatggtaattagcgtctgaaacgctgcgccgc
cttcaacggagcgcaggtgtagcaacataaaggcgataattaatccggcggcgataaatttaccgagcaacggatcgataggcgttaaaaa
gccaagattgctgacaatcgccagcgccataatcgacaatgacggcgcatcgttggcccagaagctggcccagccggagaggaaagcca
gcggtcggcttccggcatttttcagataaacataatctgcgccattttccggataagcggtggatagttccgcatagacgcacatttgcgggatca
caattaacccgccaatgacaaacgcgagcaccgtaagccacggcgtgcccgctgcttttgccacttcacccacagatacaaaaataccgga
gccgacggttgtcccgacggcgattgcaagaacggcccaaaatccgagcttgcgttggagttcctggcttcccatagcgataccttttttcttgtcc

aatacgttgtaggggagagtttgcagtcatttaaaaggtaacatgacaatgcatgatgaatataacaacatatgatgttatgtaatgtggaggaa
gatcacaaaatttcgcacaggatcgcgctgtggctaatggatgtagttatcaaattgaatttaaagtgaaaatatttttacgggcgggggcaaga
aggacatagaaacaaatacgccctcggaaaatccagagggcgtcgggcaattaaaccggtgttagccgatttctgtcagagacttactgtgc
agtaggaccgcaaccgccaatgattttagaaatagaaattgccgggtgcagcaggtagtcgtaagagcagttcttatcacggttttcaatatga
ccagtacatgcagttaacgtagccagtactgcacctaccagtgcaactttaataaatttgttcatttacattccctatgtattatatattaattaatattc
attgattatatgaatataagcagactgctcgttaagaacgatacaggaatatttaattggcgtcagtgagcgaatgacttaattttgtaagtcgctgt
aacggtgaatataacccattagcagataatatttattaaggggctatgttttatttgctgaatatataaaaagagtcattaagaaggccagaattct
ctggcccgggttaaattaatggttggagaaccagttcagtttatcgcgcaggccaacaacccgaccaataataattagcgatggactgttcattt
gctgcgccagttctcccagctgtgtgagcgtaccgtcaatcacgcgctgcgtgactgccgtaccgttttcgacaattgccaccggcatttcgcctg
gcattccgtgttcaatcagcttttgctgaatagtcgcggcctgattcaaccccatatagaacaccagcgtctgtttttctgccgccaggttttcccagt
ccagctcgccaccggtttttaagtgtccggtaattaagcgtacgctctgggcataatcgcgatgcgtgagtggaatacccgaataggcagagc
aaccagaagctgcggtaatacccggaaccaccgagaacggaatacccgcgttgcacagtgtttccagctcttcgccaccacggccaaaaat
aaacggatcgccacctttcagccgcaccacgcgtttgcctttttgcgcttcccgcagcaggatctggttaatctcttcctggggtacgcagtggtat
cccgcgcgtttgccgacgaaaacacggtccgcatcgcggcgtaccagattcataatatcgtcagaaaccagacggtcgtagaccaccacat
ctgcctgctgaatttgttgcagtcctttcagtgtcagcagcccggcatcgcccggacctgcaccaaccagcaccacttcaccgcgatggtcgag
cggttcgttgattaactgttcggtcgtttcagtaatggctttctgatcgttgtttgccagcgactgcgccaggcggtcgttaacgaacaatttctcccag
aaacggcgacgctcacccatcgtggcgaactgttgtttcactcgcccgcgtaattgcccggcgtatttcgctacctggcccagatgtaacggca
gcagtgattcaagtttttcgcgcaacaggcgtgccagaaccggagaggtgccgccagaggagaccgctaccatgagcggtgagcggtcaa
taatcgacggcataataaagctggcggctttcggcgcatcgaccacgttacagaagatgcgacgagcttcagcggcttcgctgacgcgctggt
taagcgcgtcatcatccgtcgctgcaatcgccagccagcaggtgtcgagaaggctttcatcaaatggcccttcgacgagggttaacatgcctg
catctgcccatgcggtgaactgtggaataaacgctaatgcattcaccgttaagcgagcgcctgcgtctaacagcaaccttgctttgcgttccgcg
acatcaccaccgccgacaatcagacagtcgcgatcgcgtaattggcaaaatataggcaaatgatccacgtaaaaaccccttagtaattaacc
ggcagccgtttcagtttgattaaatttgtccgcaaccggacgattcgctttcggcgtagcataccaataacccaatcccatgaatacggcacctg
ataaagtattacccagcgtcacccacagcaggttatgaccaatacccgccagcgtgtaggcttcgctgtggttgccgaaccaggagagcgcg
aacagcgtcatgttagcgatagagtgctcgtagccggacgcgataaatgccagcagacaccaccagatagcgataaatttcgccgccccttc
agtgcgcagcgccatccagattgccaggcaaaccagccagttacacaatgcacctttgaagaagagtaccattgccggtgcagtggttttagc
cagcgcgacggagtgaacgatgctggtatctaccggcagcaggctaccgccgccccagctatagagcatggcaacgaagacggaaccg
accaggttacccagccaggtttgcggcaggattgcccacatttgcccgtggctgatgctgcccgcttttaccccaaaggtgaggaacatggtgt
gtccggtgaacagttcagaaccggcgataatcaccagcgttaaggcgataccaaaggtcgcgcccatcaccaaagggcgtacggatggat
cgagcaaattaccgagcgtgaaaatcaggatgatcccaagacccacatacgcgcccgccatggcggagctgacccaaaagccgagcgg
gttatttgccgacaggcgtgcaatgcgcgcagcgttagccgcacacttattaatagtgtctgtaaacatttgattatcctttaaaaaataaaaacaa
aaaataaaacaaaaagtttaaaaaatgtcctgcaatataccgaagattattacaggaccatgcaaaaaggggaggcattgcgcctcccgtta
aaacattaaccgcgcagctgcaccacgccgtctttcactcgcgcttcgtaatgtttgacggaaaactgttcgtcttccatgcacaagccgtcgctt
aagcgaaaacgctgtttttcagcgggctggcgacccacagctcgccctggtgttccgcaatcagtccgcgtgacagcacgctggactcgaag
aacgggtcgatgttgctgatcgcaaacacctgatcgctgtgatacgggcggaaaatcgcgacctgctcgtcacctaacagcgcgcagacgc
cggtttcaggcaggatgtcatcgattttgcagatgtctttccactggctcatgcgttgtcctccaccagagttactgggatacgttcatacggcgttgc
cggacggtgctgttcgcgctctggcaccatctgcacgttcgggtcacgcttgtcgctgttgatgaagtgtttgaagcgagtctgcgcagacggcgt
attgaccgtttcagtccactcacacagtaccgcttcacgcaggcgcgccatctcttcttccagatgtgcgttcagccccagtttgtcgtcaatgatc
actgctttcaggtaatcgatgccgccttcgaggttttctaaccacggtgcggtacgcgtcagtttgtcggcagtacggatgtagaacatcatgaag
cggtcgagatatttgatcagcgtttcgcgatcgatatccgccgccagcagatccgcatgacgcggtttcatgccgccgttaccgcaaacataca
ggttccagcctttttcagtggcgataatacccacgtctttaccctgagcttctgaacattcacgggtacagccggagacaccgaacttcattttgtg
cggcgtacggatgcctttgtagcggttttccagttccacgccgaggccgacgctgtcgccaacgccgtagcggcaccaggtgctacccacgc
aggttttcgccatacgcagtgctttcgcataggcatgaccggtttcgaagccggcttcaatcagctgacgccagatctccggcagatcgtctttct
gtgcgccaaacatcgccagacgctgggagccagtgatcttggtgtagagattaaattcacgcgcgatacgacctaccgccatcagcccttccg
gggtgatttcaccgcccggagaacgcgggatcaccgagtaggtgccgtctttctggatgttagcgaggaagttgtcgttagaatcctgcagcgg
agtatgttccggcttcagaatgtattcgttccagcaggaggccagcagcgaaccgacggttggtttacaaacttcacaaccgtagcctttgccgt
gtttcgccagcagttcttcgaaggttttaatgccttcaacgcggatcaaatggaacagttcctgacgcgaataagcaaagtgttcgcacaggttgt
tgttaacttcgatgccctgtttcgccagttccgcgttcagtacctgagtgaccagcgggatacagccaccgcagccagtacccgctttggtttcag
ctttcagcgccgcaactgtgtggcagcctttgttgatggcagcaatcagatcacctttggtgacgtcgaagcaggagcagatttgcgcgctgtcc
ggcagtttatcaacaccgatagacggcttgccgctacccgagtgtgctggcaggatcagggaatccgggttttccggcagttcgatagcgttca

gcaccagttgcagcaggttaccgtagtcgctggtatcgcccaccagtaccgcaccgagcagggttttgttgtcttcgctgacaatcaggcgtttgt
agatctctttactttcgtcgaggtaaacgtagctacgtgcgccaggcgtgcgaccgtgcgcatcaccaataccgcctacgtctacgcccagcag
tttcagcttggcgctaaggtcagcaccttcaaaggcgttttcgctaccgagaatatggtcaacggcgacctgcgccattttgtagccaggtgctac
cagaccaaatacacggttgttccagcttgcgcattcaccgatggcgtagatatccggatcggaagtctggcaggaatcattaatgacaataccc
ccacgcggagcaacgtccagaccacactgggttgccagcttatcgcgcggacggataccggtagagaagacgataaagtcgacttccagtt
cgctgccgtcggcaaaacgcatggttttacgcgcttcaacaccttcctgcacaatctcaagggtgtttttgctggtgtgaacgcgcacgcccatac
tttcgattttgcgacgcagctgctcgccgcccatctgatcaagctgttctgccatcagcataggggcaaattcgataacgtgggtttcaatacctaa
gtttttcagcgcgcctgcggcttccagacctaacaggccgccaccaacaacggcaccgcgtttgctgcgacgggcgcaggattcaatggcgtt
gaggtcttcaatagtgcgatagacaaagcagtcctgagtatcagaacctttgattggcgggatccacgggtaggaaccggttgccatgatcag
cttgtcataaaaaacggtacgtccggcgctggagtgaatcaccttctcctgacggttgatggtgatagcgcgttcgccgaccagaactttgatgc
cgtgtttctcgtagaagccttcgcgcaccagcgacagctcttcggcggtgtggtgagagaagtaagacgagaggtgtacgcggtcataagcg
atgcgcggttcttcacagaaaacggtaatatcaaagttggccgcatcagatttatcaagaagatcttcgataaagcgatggccgaccataccgt
taccgataattgcgagtctgactttgctcatttttgcctcgatttcttttctattaccgcctaccttaacgattcagcaaccccgcttattgatgtaaatca
aattcacctttatatactccttaatgggtatattgctgatttgtataaattttcttaagtcacggaattgtctgatatttcatgtttgtgcaaaaaatagaca
aaatgagcgggtttatagaaatagatgcacgtagcgggggaagagaggaaaaggccggacgacgatagtgccgtccggcatgagcaatt
aatgagaggtcagggtgttatgctgacggtgacggctgacgaaacctaacaggaagcacatcacaaagacgacagcgtacagaccgtttg
ctgtaagcagtgccgcctgcggaccgctatgttcaacgatcgggccggtaaccacaaaggtcaacatagtaccgatggttccgcaggtcagg
acaaagttaaccagttttggcgacggtactttggtctgctgtgaacccagggtgatgatggtggtatagatcgcgctggagaagaagcccaga
gcgagaattgaccacgccatatgtgctggtgttccggtgttaaagacgtacatcagaatcgcagccagaccagccagtacggtcagaatgcg
ttgcaaatcaaagaagcgaagaataaagctgaacgcccacatgccgaccatgtatgacatccagaagttactcaccagcgtgcccgcgtcg
ttcaggctcatgcccaggcctttggcatactcaggcacccaggagataaaacctaactgaccgaggatgtagcacaacgccgcaacagag
agaaacagtacgccgatcccccacttttctttctctaccggagcatccgtttttggcgcatgtttgcccagcgccgggaactcacagccgaaggt
cagaataaaaatagcgacatacaccagcccgatgcaggcataaacccagtaccactcaatgctgcgcgccagtagaaacgcggcgatca
ttgggaaaatcatcccagccatactgaagaaggagtcggtaaataacaggcgggaaccgcgctgacgcccttcatacatttgtgttaccagg
aatgtaccaatcgacatggtgatgccgctgaccaccccgagaatgaacatcgccgccgagaacagcgccaggctgtggctgaacatcaaa
ccggcaaccgccagcaccatcaggagaaagccaaaacgtaactgcgttttcaacgggacgatttccatcagccaggcgttgaggaagata
gagattaaaatgccggcgttgaggaaggtgaaggtattactcatactggaaacaggcagattgaaataatcggcgatatttcccatcaccatc
ccggtgacaataaccaacgcaccagtcagtgcgtaggagagaaagctaatccatgtgagcttgatgcgattgctgttagtcatgattggcctgc
gttcaaaaataaaatggcatagcgggatatgccgcgagcgggcgattttaggtgattttgtgatctgtttaaatgttttattgcaatcggttgctaaat
tgcattttaagaggtgattttgatcacggaataaaaagtgatcgtcaggttacatatatttcagatacgtaaaattaggtaaagggatggccttgttc
ttgaaggctatttagaatctcttcacttgctttttttctgctctgtttgttaaggaaatctcatgttcaaatcgaccctggcggcgatggctgctgttttcgct
ctttctgctctttctcccgcagcaatggcagcgaaaggggacccgcacgtattgttgacaacctcagctggtaacatcgaactggagctggata
aacaaaaagcgccagtgtctgtgcaaaactttgtcgattatgtgaacagcggtttttataacaacactacctttcaccgcgtcattcctggctttatg
attcagggcggcggtttcaccgagcagatgcagcagaaaaaaccaaacccgccaatcaaaaatgaagccgataacggcctgcgcaaca
cgcgtggcaccatcgcgatggcacgtaccgctgacaaagacagcgccaccagccagttctttatcaacgttgccgataacgccttccttgacc
atggtcagcgtgatttcggttacgcggtatttggtaaagtggtgaaaggcatggacgttgccgataagatttcccaggtgccgactcatgacgttg
gtccgtaccagaatgtgccgtcaaaaccggtagttatcctttccgctaaagtcctgccgtaatgatttctcgcgcgggcaatcttgcccgcgcttct
gctctcccggcgtaacccggatttgccgcttatacttgtggcaaatggacacgttcagggaggcatcaagtgaagaaactcaccgataagcaa
aagtcccgtctctgggagcttcagcgtaatcgtaattttcaggccagtcgccgtcttgaaggcgtcgagatgcctttagtcactcttactgccgcag
aggctttagcgcgccttgaagagctgaggagtcactatgagcgataaattcggcgaagggcgcgatccgtatctttatccaggccttgatatcat
gcgtaaccggctgaacatccgccagcagcagcggctggaacaggccgcttacgaaatgacggcgctgcgtgctgcgaccattgagcttggt
ccgctggtgcgcggtttaccgcatttgcgaactatccatcgccagctgtatcaggatattttcgactgggcagggcaactgcgtgaagttgatattt
atcagggtgatacgccgttctgccactttgcttatatcgaaaaagagggcaatgccctgatgcaggatctggaggaagaaggttatctggttgg
cctggagaaagcgaagttcgtcgagcggctggcgcattactattgtgaaatcaacgtgctgcatcccttccgggtgggaagtggtctggcaca
gcggatcttcttcgagcaactggcgattcatgccggatatcaactgagctggcagggtatcgaaaaagaggcctggaatcaggcaaatcaga
gtggggcaatgggggatctcaccgcactgcagatgatatttagcaaagtggtaagcgaagccggggaatctgagtaaaatagcgcggttcttt
tgtaccggagccgccatgatcctgcttatagataactacgattcttttacctggaacctctaccagtacttttgtgaactgggggcggatgtgctggt
taagcgcaacgatgcgttgacgctggcggatatcgacgcccttaaaccacaaaaaattgtcatctcacctggcccctgtacgccagatgaag
ccgggatctcccttgacgttattcgccactatgccgggcgcttgccgattcttggcgtctgcctcggtcatcaggcaatggcgcaggcatttggcg
gtaaagttgtgcgcgccgcaaaggtcatgcacggcaaaacctcgccgattacacataacggtgagggcgtatttcgggggctggcaaatcc

acttaccgtgacacgctaccattcgctggtggtggaacctgactcattaccagcgtgctttgacgtgacggcctggagcgaaacccgagagatt
atggggattcgccatcgccagtgggatctggaaggtgtgcagttccatccagaaagtattcttagcgaacaaggacatcaactgctggctaattt
cctgcatcgctgatttctgattgccatttagtgatttttatgcatattttgtggttataatttcacatttgtttatgcgtaacagggtgatcatgagatggca
attgaacaaacagcaattacacgcgcgactttcgatgaagtgatcctgccgatttatgctccggcagagtttattccggtaaaaggtcagggca
gccgaatctgggatcagcaaggcaaggagtatgtcgatttcgcgggtggcattgcagttacggcgttgggccattgccatcctgcgctggtgaa
cgcgttaaaaacccagggcgaaactctgtggcatatcagtaacgttttcaccaatgaaccggcgctgcgtcttgggcgtaaactgattgaggc
aacgtttgccgaacgcgtggtgtttatgaactccggcacggaagctaacgaaaccgcctttaaactggcacgccattacgcctgtgtgcgtcat
agcccgttcaaaaccaaaattattgccttccataacgcttttcatggtcgctcgctgtttaccgtttcggtgggtgggcagccaaaatattccgacg
gctttgggccgaaaccggcagacatcatccacgttccctttaacgatctccatgcagtgaaagcggtgatggatgatcacacctgtgcggtggt
ggttgagccgatccagggcgagggcggtgtgacggcagcgacgccagagtttttgcagggcttgcgcgagctgtgcgatcaacatcaggca
ttattggtgtttgatgaagtgcagtgcgggatggggcggaccggcgatttgtttgcttacatgcactacggcgttacgccggatattctgacctctgc
gaaagcgttaggcggcggcttcccgattagcgccatgctgaccacggcggaaattgcttctgcgtttcatcctggttctcacggttccacctacgg
cggtaatcctctggcctgtgcagtagcgggggcggcgtttgatatcatcaatacccctgaagtgctggaaggcattcaggcgaaacgccagcg
ttttgttgaccatctgcagaagatcgatcagcagtacgatgtatttagcgatattcgcggtatggggctgttgattggcgcagagctgaaaccaca
gtacaaaggtcgggcgcgtgatttcctgtatgcgggcgcagaggctggcgtaatggtgctgaatgccggaccggatgtgatgcgttttgcaccg
tcgctggtggtggaagatgcggatatcgatgaagggatgcaacgtttcgcccacgcggtggcgaaggtggttggggcgtaaatacgaagcat
caggcaaaagatgtcggatgcggcgtgaatgccttatccgatggtttagcctgcattacgccttcgaatcccgcaacttgcgactcagccaaat
cccgtgatgcggtcgctgacgccatgccatcgacgaaatggtgtgcatggtgttcagatgaccaataacccgctgtaaatgctgctccagcgta
cctgccgcgccttcgtgcggctgcatctccggcgcatccatgatattggcatcgccagaactacccggttcgtcatactccagtcgctgctgaca
acgctgaatggcgatttcacaagactgtaaatactcttgtgccagttcaggtggcaatgcccggtgttcccgcgccagcgtggtcatggcattaat
atgctcaacaataaactggctgtgcgttacccacagtttcatatctgccagataatggctgttaaacgccggttcctgcatcgcctgattcaatgag
ttatacagagtgttatgtgcctgatttacccgcattcgctgccaggccagtggcgtaggttgcggatcctcgctaagaatcaagcgaatcgcttcct
gataggcttctaaagcatcatgggcgtttttacgcaataacccgctctgccactgcggccacagccagacagtaccgccgaaagcaattaaa
caaccaataatggtatcgataagacgcggaaggatgtattgctcgccgttcaaccacaatagttgcagggtatacactgcggtaatagtaaaa
ccgaccgtcgcccagccgtagtttttgcgcaatatcaggtagctggcgagggtggtaatcagcatcaacgtcagggtgtaaccttcgggaatttt
aaagtgcagcgccacgcccgcaatgattaacccgaccacggttcccacggagcgattcacaatcctcagacgggttgcgccatagccatttt
gtgtcaccagcaataccgtcatcaggatccagtacgacttcggcagatgcagcgcggtgcccatcaggctggcaacgcttaacatcacactg
agtcgtccggcattgcgtagcgccggagactttagtgacagataacttttcagcgccggaagtaatggcattcgccgctgtttatcggccagtaa
gtcacgggcatagagcggtttttgggtgcgcagcacgcgggcgatgcggctgaaatgccagtagcagaattgcccaaccggattatccggat
gctggcgggcgatttttccagtgcgccaatttgcttttccatcgtaaaacgcgttggcaggcgatggtaaagaatgtcatcagccagcacgcgc
aggcgagcggcgacggtttgcgcattccagcggataacttcttccgcatggctacgctcgaccagcttttgcacctcttccggctgatgcaaact
gaccgaaatatgttcctgtaaatccagcgcctcctggaaaatacgcagcatccgcttgtagtcagtattattttgcgcggaaagcatatgcatttg
ctgatagcactgggtaattagatcgaccgctttttgctggcgcaccagcagcggcggcagcgctttttcagggtcggtgtgctgggtaagcagg
ctgtatttggcttcacaataatctgccagttcacggtacagcagacttagtgactcgcgcagcggttgttcgcgccagatccagaaccaaaacc
agttaaacaatccgtaccagagagtgcccaacgcatagatgagcaacggttcccagaccggcatgtatcccgccaaactgagggtaaaaat
ggcggcgagcagcgatgcaggaagcaattttgcgtgcaatggccccagctcagcagtgacgccaagtaccagcgttaatccggtcagcaa
aaagggcaggggaacatcttttgccagtagtagctgtgtcagcaagctacaggtggcaaacagcgacgcaccaatgattaagcgtttgaaa
aaacgtttatgaggcgtatcaaggcccgcaatattgcaacaggcaggaacgagggagaagagcagaccgaatcgtaattcgccaagcatt
aacccaacggccacgggcaaacatagcaccagcgtttgtcgaagtgcatagttgatatcggggtgataaatcagtctgcgccacatcgggg
gaaacaaaatggcgcgctaccaggtaacgcgccactccgacgggattaacgagtgccgtaaacgacgatggttttaccgtgtgcggagatc
aggttctgatcttccagcatcttcagaatgcgtcccacggtttcacgagaacagccgacaatctgaccaatttcctgacgggtaattttgatttgcat
accgtccgggtgagtcatagcgtctggttgttttgccagattcagcagagtctgtgcaatgcggcccgtcacgtcgaggaacgccaggttgccc
actttctctgaagtgacttgcagacgacgcgccatctgtgcagacaaacgcatcagaatgtccgggtttacctgaatcaattggcgaaatttttgt
acgaaatttcagccacttcacaggcggttttcgcacgtacccatgcgctacgttcctggccctcttcaaacaggcccagttcgccaataaaatca
ccctgattcagataggagaggatcatttctttaccctcttcgtctttgatcagcactgccacagagcctttaacgatgtagtacagcgtttccgcttttt
caccctggtgaataagcgtgctcttggatgggtacttatgaatgtggcaatgagacaagaaccattcgagagtcgggtctgtttgcggtttgcca
agcaccatgcgcggttatcctctgttataagctttctccagagccagataacgccgctgtctctggattgccgaaatatgcttcccgctacctggga
aggggctatcaactgtactgcacggtaatgtgacgtcctttgcatacatgcagtacatcaatgtattactgtagcatcctgactgttttagcatagctt
tcgctttgtgtctcctggtgtctcgcttcagcatgacccaggtcgccttccgttgcgcgatttggttagtacgcgtactctgtcaggaaaattgacgca
gtggagtagcaaaaatgcaagcgcgagtgaagtgggtcgaagggttaacttttctgggcgaatccgcctctggtcatcagattttaatggacgg

caactcaggcgataaagcaccaagtccgatggaaatggtgttgatggcggcgggtggttgcagtgccatcgatgtggtttcgatcctgcaaaa
agggcgtcaggatgtggtcgattgtgaagtaaaattgacctctgaacgccgcgaagaggcaccacgcctgtttacgcacattaatctgcattta
tcgtcaccggtcgcgacctgaaagacgcagcggttgcgcgtgcggttgatctctctgccgagaaatattgttcagtggcgttgatgctggaaaa
agcggtgaatattactcactcgtatgaagtggttgccgcgtgacttaactatcccgttgtgaagccggatagtattttatccggcgtaatagcagag
ttactcgattttctttccttccatcaatcgttgcaccagcggcagcataattaattccattgccagacccattttgccgcccggtaccactaacgtattg
atgtgggaaatgaatgagccttgcaacatcgccagcagccaggggaaatcgatcccttccagattacgaaaatggatcaccacaaagctttc
atcgagcgacgggataccttttgccgcgaacgggtttgaagtgtcgacggtgggaacgcgctggaagttaagatgggtgcgggaaaactgc
ggtgtgatgtagttgatatagtcttccattgaacgcactactgagtccatcactgcttctcgtgagtgcccgcgctcgctggtgtcgcggatcagttttt
gaatccactcaaggttaacgataggcaccacgccgaccagtaagtccacatgctgcgcaacgttatgctgtggcgtgactacgccgccgtgt
aaaccttcataaaacagtacatcagtgggttccggtaaaggttgccaggggggtgaatgtccccggtacctgattccacggtacggcttcgtcgt
aggtatgcagatatttgcgagatttccctttgccgctctgaccgtattcaatgaaggtttgttccagcaggccgaagtcgttagcctcggggccga
agtagctgatatgccgcccggcgtcgcgcgctttgcggatcgccatgtccatttccgggcgggtgtaacggtgaaaactgtcgccttccacctca
gctgcatgcagatttaactgcgcgaatattttacgaaacgcgaggctggtggtggtggtccccgcgccgctggatcctgttaccgcaatgaccg
gatgtttggcagacatagtaactccatgacttagctatcattattatctcgtccgccgacggttgcaggttattcgcgaaactgactgcgcggcata
atattgaccgtttcgtgcagctccgaccataccagcaccgcttctccgcactgtagctggcgtttgacgtcggcgactttctgttcaagtgtacgctc
atgttcaccataatcggtgccttcacgtaacacaaagctttcaattaaattttccagcgtttcgggggagaggtcttgccacggaatcaacatgatt
tcgcctccagatacgttgttaaccagtcaggaatgcgtgactccagccacatttgcggatgaagtaatgtaccgccaataaagccaacatgac
cgccatgttcagtcagttgatactccacctgcggggggagactttccggtttcgggatcacctgatgatccataaacggatcgtctttggcgtgaat
aatcagcgtcggtttggcgatccggttcagcatcggcatggcgctacactgacgataatagtcgatagcgtcggcgtagccgtgaattctggcg
gtgatgagatcgtcaaattcacggatgcgacgtaccgattttaactgcgcgagattaatcggcagcgttccggggtaggctgccagcttgcgcg
cggcattggcttttaacaggttcagcaagtaacgctgataaacgcgggaaaagcccttttccatatgatagctacaggcttccagcataaacgg
cgcagagacaatcaccgccgcatcaaccgggagatcattgccttcctttgccagcaaacaggccagcatattaccgccgagcgaatagccg
acggcagccgttggcgcatgaccaaattcgcgttgcagccagcgtaaaaaccaactggcgtcttcggtttcgcccgaatggtaaatgcggtgc
atacggtttggttcaccgctgcatccgcgaaaatgcatcaccacgcccagccagccgcgcttttgcgccgcctcaaccagaccgtgggcgtaa
gggctgttgagactgccttccagcccgtgaaacaccaccagacgaggtttatgttgcgcctgtgcagggttttcactccacgcaagatcgacaa
aatcgccgtcgggcaactccagccgctgccagtacggggtgaatttcacctgacgacgaaacagacgcggcagcatggtttgcagatgaca
attgctaaagccgcgcatggggatgaattcagcactgctgctgaattcattggcatcggtcgtcgttatctgcgccatcagttgctttggccttccag
cagcatctgctcaagctgctcctgggcttccagccatgccatttcgcactcttccaggccggatttggcgctggcttgctgttgcaggcaggcggt
caactccgctttacggctctggtcatacagttcgctgtcgccgagtttctcttccgcctgcgccagttgcgcgttcagcttctccatctctttttccagac
gggcaatctctttacgcagtggctgggtttgcgcacgcagctcagcttcccgacgcttctgatctttacgtgcctgggcgctgttcgcattctcttttgg
cgcttcgtcggtctggttttcctgcttttgtacgtcgctcaaccactgttgataatcttccagatcgccgtcgaacggttcgactttacgatcgtgaacc
aggtagagatcgtcagtggtggaacgcagcaaatgacggtcgtgcgaaacgacaaccagcgcgccttcaaactcgattaatgcttcggtga
gtgcctgacgcatgtcgaggtcaaggtggttagtcggttcgtcgagcagcagcagattcggccgctgccagacaattaatgccagcaccagg
cgggctttttccccaccggagaagcggcgcgtttcttcggttactttatcgccctggaaaccaaagccgccgaggtagtcacgcagtttttgttcca
gctcctgcggcgctaaacgtgccagatgttgaataggtgattcgtcggcgcgcaggtattcaagttgatgctgggcgaagtagccgagtttgatc
cctttcgccagaccaatttcaccgctgactggcgcaagttcaccggctaacagtttgattaatgtcgatttacccgcgccattgcggcctaacaga
ccaatacgcgagccgggcaccaggttcagtttaatcgagtcgagaataatgcgatcgccatagcccgcgctgactttttccatcttcagtaacg
gatttggcaggctttccggcgcgcggaagctaaagcggaacgggttgtcgacgtgcgcgggggcaattagctccatacgctcgagcatctta
atgcggctctgggcctgcttcgctttggtggctttggcacggaaacggtcgatataactttgcagatgcgctacgcgttcctgctggctttcgtacat
cgcttgttgctgcgccagacgggtggcgcgctgtacttcaaacgaactgtagttgccggtgtactcgaacatgctttgttgttcgatatgaataatttt
atcgacgatcggatcgaggaagtcgcggtcgtgagagatcaggatcagcgtgccctgatagctcttcagccatttttccagccagataacggc
atcgagatcgaggtggttagtcggttcgtcgagcagcagcaagtctgaacggcaaatcagcgcctgggcaaggttaagacgcatacgccag
cccccggaaaaatcacttaccgggcgctccagttgttcattgctgaaaccgaggccgtgcagcaggctggcagcacgggagcgaatactcc
atgcgtcaatagcatccagcttgccatgaatggtcgcaatggcgtgcccgtcgttacgttcgttggcgtcgtgtagctgcgcttctagttgacgatat
tcacggtcgccgtcaatgacatattccagcgccgcttgcggtaacgccggcgtttcctgattcacccacgccagttgccagcttcccggaaagg
tgtagctgccgccgtcggcgctgatttcatttttcagcaatgccagcagggtagatttaccacagccgtttttacccaccaggccgactttctgccc
agggttgatggtggcggtggcattatccagcaggacgcgcacgccgcgacgaatttgtaacgaggagaaaacaatcataaggtgccgtatg
ttcagactatgttaacttatcattatgataatgtaatgtatgggcgagctgccgcaccgggcgcaatggtagcccaaaacagcgactatacaca
aaaaccataccgggagggggatgatgtctcagccagcgaaagttttgctgctgtatgcccatccggaatctcaggactcggtggcaaaccgg
gtactgcttaaaccggccacgcagctcagcaatgttaccgtgcacgacctttacgcgcactatcccgatttttttattgatatcccccgtgagcagg

cattactgcgcgagcacgaggtgattgtctttcagcatcctctttatacctatagctgcccggcgctactgaaagagtggctggaccgggtattaa
gtcgtggttttgccagcgggccgggaggaaaccaactggcgggaaagtactggcgtagcgtgattaccaccggcgagccggaaagtgctta
ccgttatgacgcgctgaatcgctacccgatgagcgatgtgctgcgcccctttgaactggcggcgggcatgtgccggatgcattggttaagtccc
atcattatttactgggcgagacggcaaagcgcacaggagctggcgagccacgccagagcctacggtgactggctggcaaatccgctgtctc
caggaggccgctgatggaaggttccgatttttactcgcaggagtgctgtttctcttcgcggcggtggctgcggtgccgctggcatcgcggctggg
tattggcgctgtgttgggatatttgctggcagggattgcaattggcccgtgggggctggggtttattagcgacgtcgatgagatcctccactttcgg
aactcggcgtggtattcctgatgtttatcatcggccttgagttgaatccctccaaactttggcaactgcggcgttcgatttttggcgtaggcgcggca
caggtgctgttaagcgcggcgttgctggcgggattattgatgctgacggatttcgcctggcaggcggcggtggtcggtggcattggccttgcgat
gtcttcaactgcaatggcgttgcaattgatgcgtgagaaagggatgaatcgcagcgaatccggccagctcgggttttcggttctgctgtttcagga
tctggcagtaatcccagcactggcgttagtgccgttgttggcggggtcggcagacgaacatttcgactggatgaaggtcggcatgaaggtgctg
gcgtttgtcggcatgctgattggtgggcgctatttactgcgtccggtattccgctttattgcagcttctggcgtgcgggaagtgttcaccgccgcgac
gctgctgctggtgttgggttccgcattgtttatggatgcgctggggctgtcgatggcgctcggtacatttattgcgggcgtgctgctggcggaaagtg
aatatcgccatgaactggaaacggctatcgatcccttcaaaggcttgctgctcggtttgttctttatctctgtcggcatgtcactcaacctcggggtg
ctttatacccatctgttgtgggtagtgataagcgtggttgtgctggtggcggtgaaaattctcgtgctgtatctgctggcgcgattgtatggcgtgcgt
agctcagagcggatgcagtttgctggcgtgttgagtcagggtggtgagtttgcctttgtcctcttttctaccgcttcttcacaacgcttattccagggcg
accagatggcgttgttgctggtgacggtgacgctttccatgatgaccacgccgttgctgatgaagctggtggataaatggctatcccgccagttta
acggaccggaagaagaagacgaaaaaccgtgggtcaacgatgataaaccccaggtcattgtcgtgggcttcgggcgttttggtcaggtgatt
ggtcgtttgctgatggcaaataaaatgcgcattaccgtgctggagcgggatatcagcgccgttaacctgatgcgtaaatacggctacaaagttta
ttacggcgacgccacgcaggtcgatcttttacgttctgcgggtgcagaggccgctgagtctatcgtcattacctgtaacgagccggaagacacc
atgaagctggtggaaatatgccaacagcactttccgcatttgcatattcttgcgcgagcgcgcggacgtgtggaagcgcatgagttattacagg
caggggtgacgcagttttcccgtgaaacattctccagtgcgttagagctggggcgcaagacgctggtcacgcttggcatgcatccgcatcagg
cacagcgagcgcaactgcattttcgccgcctggatatgcggatgctgcgagagctcatcccaatgcatgccgataccgtacaaatttctcgcgc
cagggaagcccgacgcgaactggaagagattttccagcgtgaaatgcaacaagaacgacgccagctggacggctgggatgaatttgagta
gagggtaaagatggcaatccgaaaacgttttattgcgggcgcaaaatgcccggcctgtcaggcgcaggattcaatggcgatgtggcgcgaa
aataatattgatattgttgaatgtgttaagtgcggacatcagatgcgagaagcagacaaagaagcccgcgatcacgttcgcaaagatgagca
agtgatcgggatttttcatccggactagcgatatgcgccgagtttttttaagctagtgagtacacggctgcagaattccgctacaatctgcgccact
attcttcccatgctcaggagatatcatgaaagtagcaaaagacctggtggtcagcctggcctatcaggtacgtacagaagacggtgtgttggttg
atgagtctccggtgagtgcgccgctggactacctgcatggtcacggttccctgatctctggcctggaaacggcgctggaaggtcatgaagttgg
cgacaaatttgatgtcgctgttggcgcgaacgacgcttacggtcagtacgacgaaaacctggtgcaacgtgttcctaaagacgtatttatgggc
gttgatgaactgcaggtaggtatgcgtttcctggctgaaaccgaccagggtccggtaccggttgaaatcactgcggttgaagacgatcacgtcg
tggttgatggtaaccacatgctggccggtcagaacctgaaattcaacgttgaagttgtggcgattcgcgaagcgactgaagaagaactggctc
atggtcacgttcacggcgcgcacgatcaccaccacgatcacgaccacgacggttgctgcggcggtcatggccacgatcacggtcatgaaca
cggtggcgaaggctgctgtggcggtaaaggcaacggcggttgcggttgccactaataccgaaaaagtgacaaaaaagcggggaatcccc
gctttttttacgcctcaataatgtggcggtggcgtttcttcagcctgcgacgcgatgttcgacggctggctggcttttaacttctcggtcagcagacgc
agatgatcgcgcagtttcgccatctccatttcatgagcggtcaccgtgacgttcagttcttcaatggtgatttcctgaaaagccagtcggctctcca
gctctgccaggcgtgcttccaatgataaatcctgcatgattcacctcttttgtcgaatggtcgccgcggattctacttaactttgctgcccgagacag
cactcatttcgcggtcatcgaaactaatttaaacaaaaagagtctgaaaatagatgataatagggcgtgtctgtatgtagatttgttcgacaacgc
tttatagtacccttctgataatagttaaccctggggtgagatgccccgatcctggagatatggatgaaatcactgtttaaagtaacgctgctggcga
ccacaatggccgttgccctgcatgcaccaatcacttttgctgctgaagctgcaaaacctgctacagctgctgacagcaaagcagcgttcaaaa
atgacgatcagaaatcagcttatgcactgggtgcctcgctgggtcgttacatggaaaactctctaaaagaacaagaaaaactgggcatcaaa
ctggataaagatcagctgatcgctggtgttcaggatgcatttgctgataagagcaaactctccgaccaagagatcgaacagactctacaagca
ttcgaagctcgcgtgaagtcttctgctcaggcgaagatggaaaaagacgcggctgataacgaagcaaaaggtaaagagtaccgcgagaa
atttgccaaagagaaaggtgtgaaaacctcttcaactggtctggtttatcaggtagtagaagccggtaaaggcgaagcaccgaaagacagc
gatactgttgtagtgaactacaaaggtacgctgatcgacggtaaagagttcgacaactcttacacccgtggtgaaccgctttctttccgtctggac
ggtgttatcccgggttggacagaaggtctgaagaacatcaagaaaggcggtaagatcaaactggttattccaccagaactggcttacggcaa
agcgggtgttccggggatcccaccgaattctaccctggtgtttgacgtagagctgctggatgtgaaaccagcgccgaaggctgatgcaaagcc
ggaagctgatgcgaaagccgcagattctgctaaaaaataagcattaagaaccgccgcctgaccaggcggcggtttttttattacaggccggat
ataattagtgctggaaagcggaacctccgctgtattaatttagttacccgcatcattaatgagcctgccctgaaaagttaacgacaggctcctga
aaaggagtgttttttttcatgtccaggtcgcttttaaccaacgaaaccagtgagttggatttactggatcaacgtcctttcgaccagaccgattttgat
attctgaaatcctacgaagcggtggtggacgggttagcgatgcttattggctcccactgtgaaatcgttttgcactctttgcaggatctaaaatgttc

agccattcgcattgctaacggtgaacatacaggccggaagattggttcgccaattactgacctggcgctacgtatgctgcacgatatgacggg
agcggatagcagcgtttctaaatgctactttactcgcgccaaaagcggcgtattaatgaagtccctgactatcgcgattcgtaaccgcgaacag
cgtgtaattggtctgctgtgcatcaatatgaatcttgatgttcccttctcgcagattatgagcacctttgtgccgccagaaaccccggatgtcggttca
agcgtcaactttgcctcttctgttgaagatctggttacccaaacgctggagttcaccatcgaagaagtgaatgccgatcgcaatgtttctaataac
gccaaaaatcgtcagatcgtgctgaatctctacgagaaagggatcttcgatattaaagatgcgatcaaccaggttgctgaccgcctgaacatct
ccaaacacactgtctatctctacatccgccagttcaagagcggtgatttccaggggcaagataagtaatgcgttttgccatcgtggtgaccgggc
cagcatacggtacgcaacaggcgagtagtgcttttcagtttgcgcaggcgctgatagcagatggccatgagttaagcagcgtctttttctatcgg
gaaggggtctataacgctaaccaattgacctctccggcaagtgacgaatttgacctcgtacgggcctggcaacaactgaatgcgcaacatggt
gtggcgctgaatatctgcgtagcggcagcattacgccgtggcgttgttgatgaaacggaggccggaagactggggctggcttcgtcaaacctt
cagcagggatttaccttaagcggacttggggcgctggcggaagcctcgctgacctgtgacagggtggtacagttctgatgaaacgaattgcgtt
tgtttttctactgcacctcatggtacagccgcaggccgggaaggtttagatgctttactggcaacttccgcattaactgacgatctggctgtcttcttt
atagctgatggcgtttttcagctgctgccaggacaaaagcccgatgcagtgctggcgcgtgattacattgccacttttaaattgttgggtctgtacg
acattgaacagtgctgggtttgtgcggcttcactgcgcgaacgcgggttagatccgcagacaccctttgttgtcgaagccacgccgctcgaagc
agatgccttacgccgcgaactcgccaactacgatgttattttgaggttttgaggcgtttatgctgcacacattacatcgctcaccctggctgacgga
ttttgctgcgctgctgcgtctgctcagtgaaggagacgaactgctattattgcaagatggcgtaactgccgcagttgacggtaaccgctaccttga
aagtctgcgtaatgcccccattaaggtctatgccctgaacgaagaccttattgcccgcggtttgactggtcaaatttcgaacgacatcattctcatt
gactatactgatttcgtcagacttacggttaagcaccccagccagatggcctggtgatggcgggatcgttgtatatttcttgacaccttttcggcatc
gccctaaaattcggcgtcctcatattgtgtgaggacgttttattacgtgtttacgaagcaaaagctaaaaccaggagctatttaatggcaacagtt
aaccagctggtacgcaaaccacgtgctcgcaaagttgcgaaaagcaacgtgcctgcgctggaagcatgcccgcaaaaacgtggcgtatgt
actcgtgtatatactaccactcctaaaaaaccgaactccgcgctgcgtaaagtatgccgtgttcgtctgactaacggtttcgaagtgacttcctac
atcggtggtgaaggtcacaacctgcaggagcactccgtgatcctgatccgtggcggtcgtgttaaagacctcccgggtgttcgttaccacaccg
tacgtggtgcgcttgactgctccggcgttaaagaccgtaagcaggctcgttccaagtatggcgtgaagcgtcctaaggcttaatggttctccgtta
agtaaggccaaacgttttaacttaaatgtcaaactaaactcgtagagttttggacaatcctgaattaacaacggagtatttccatgccacgtcgtc
gcgtcattggtcagcgtaaaattctgccggatccgaagttcggatcagaactgctggctaaatttgtaaatatcctgatggtagatggtaaaaaat
ctactgctgaatctatcgtatacagcgcgctggagaccctggctcagcgctctggtaaatctgaactggaagcattcgaagtagctctcgaaaa
cgtgcgcccgactgtagaagttaagtctcgccgcgttggtggttctacttatcaggtaccagttgaagtccgtccggttcgtcgtaatgctctggca
atgcgttggatcgttgaagctgctcgtaaacgcggtgataaatccatggctctgcgcctggcgaacgaactttctgatgctgcagaaaacaaag
gtactgcagttaagaaacgtgaagacgttcaccgtatggccgaagccaacaaggcgttcgcacactaccgttggttatcccttcggagttttagt
caccaggcgggcgcttccagtaagcagcccgctttgggctacttaaattgaacgcctaaaagataaacgaggaaacaaatggctcgtacaa
cacccatcgcacgctaccgtaacatcggtatcagtgcgcacatcgacgccggtaaaaccactactaccgaacgtattctgttctacaccggtgt
aaaccataaaatcggtgaagttcatgacggcgctgcaaccatggactggatggagcaggagcaggaacgtggtattaccatcacttccgctg
cgactactgcattctggtctggtatggctaagcagtatgagccgcatcgcatcaacatcatcgacaccccggggcacgttgacttcacaatcga
agtagaacgttccatgcgtgttctcgatggtgcggtaatggtttactgcgcagttggtggtgttcagccgcagtctgaaaccgtatggcgtcaggc
aaacaaatataaagttccgcgcattgcgttcgttaacaaaatggaccgcatgggtgcgaacttcctgaaagttgttaaccagatcaaaacccgt
ctgggcgcgaacccggttccgctgcagctggcgattggtgctgaagaacatttcaccggtgttgttgacctggtgaaaatgaaagctatcaact
ggaacgacgctgaccagggcgtaaccttcgaatacgaagatatcccggcagacatggttgaactggctaacgaatggcaccagaacctga
tcgaatccgcagctgaagcttctgaagagctgatggaaaaatacctgggtggtgaagaactgactgaagcagaaatcaaaggtgctctgcgt
cagcgcgttctgaacaacgaaatcatcctggtaacctgtggttctgcgttcaagaacaaaggtgttcaggcgatgctggatgcggtaattgatta
cctgccatccccggttgacgtacctgcgatcaacggtatcctggacgacggtaaagacactccggctgaacgtcacgcaagtgatgacgagc
cgttctctgcactggcgttcaaaatcgctaccgacccgtttgttggtaacctgaccttcttccgtgtttactccggtgtggttaactctggtgataccgt
actgaactccgtgaaagctgcacgtgagcgtttcggtcgtatcgttcagatgcacgctaacaaacgtgaagagatcaaagaagttcgcgcgg
gcgacatcgctgctgctatcggtctgaaagacgtaaccactggtgacaccctgtgtgacccggatgcgccgatcattctggaacgtatggaatt
ccctgagccggtaatctccatcgcagttgaaccgaaaaccaaagctgaccaggaaaaaatgggtctggctctgggccgtctggctaaagaa
gacccgtctttccgtgtatggactgacgaagaatctaaccagaccatcatcgcgggtatgggcgaactgcacctcgacatcatcgttgaccgta
tgaagcgtgaattcaacgttgaagcgaacgtaggtaaaccgcaggttgcttaccgtgaaactatccgccagaaagttaccgatgttgaaggta
aacacgcgaaacagtctggtggtcgtggtcagtatggtcatgttgttatcgacatgtacccgctggagccgggttcaaacccgaaaggctacg
agttcatcaacgacattaaaggtggtgtaatccctggcgaatacatcccggccgttgataaaggtatccaggaacagctgaaagcaggtccg
ctggcaggctacccggtagtagacatgggtattcgtctgcacttcggttcttaccatgacgttgactcctctgaactggcgtttaaactggctgcttct
atcgcctttaaagaaggctttaagaaagcgaaaccagttctgcttgagccgatcatgaaggttgaagtagaaactccggaagagaacaccg
gtgacgttatcggtgacttgagccgtcgtcgtggtatgctcaaaggtcaggaatctgaagttactggcgttaagatccacgctgaagtaccgctgt

ctgaaatgttcggatacgcaactcagctgcgttctctgaccaaaggtcgtgcatcatacactatggaattcctgaagtatgatgaagcgccgagt
aacgttgctcaggccgtaattgaagcccgtggtaaataagcctaagggttaataccaaagtcccgtgctctctcctgaaggggagagcactat
agtaaggaatatagccgtgtctaaagaaaaatttgaacgtacaaaaccgcacgttaacgttggtactatcggccacgttgaccacggtaaaac
tactctgaccgctgcaatcaccaccgtactggctaaaacctacggcggtgctgctcgtgcattcgaccagatcgataacgcgccggaagaaa
aagctcgtggtatcaccatcaacacttctcacgttgaatacgacaccccgacccgtcactacgcacacgtagactgcccggggcacgccgac
tatgttaaaaacatgatcaccggtgctgctcagatggacggcgcgatcctggtagttgctgcgactgacggcccgatgccgcagactcgtgag
cacatcctgctgggtcgtcaggtaggcgttccgtacatcatcgtgttcctgaacaaatgcgacatggttgatgacgaagagctgctggaactggt
tgaaatggaagttcgtgaacttctgtctcagtacgacttcccgggcgacgacactccgatcgttcgtggttctgctctgaaagcgctggaaggcg
acgcagagtgggaagcgaaaatcctggaactggctggcttcctggattcttatattccggaaccagagcgtgcgattgacaagccgttcctgct
gccgatcgaagacgtattctccatctccggtcgtggtaccgttgttaccggtcgtgtagaacgcggtatcatcaaagttggtgaagaagttgaaat
cgttggtatcaaagagactcagaagtctacctgtactggcgttgaaatgttccgcaaactgctggacgaaggccgtgctggtgagaacgtaggt
gttctgctgcgtggtatcaaacgtgaagaaatcgaacgtggtcaggtactggctaagccgggcaccatcaagccgcacaccaagttcgaatct
gaagtgtacattctgtccaaagatgaaggcggccgtcatactccgttcttcaaaggctaccgtccgcagttctacttccgtactactgacgtgact
ggtaccatcgaactgccggaaggcgtagagatggtaatgccgggcgacaacatcaaaatggttgttaccctgatccacccgatcgcgatgg
acgacggtctgcgtttcgcaatccgtgaaggcggccgtaccgttggcgcgggcgttgttgctaaagttctgggctaattacacgttaattagttttg
aattgaaaagggcgcttcggcgcccttttttgcatttgttgacggcatatcttattcgtactgtcagaacgaataaattattcatatttaaaatatcaatt
caatttaacattttaaatatgaatgtttctgtttttaaaatgcttcatttgtcaggctttttttcttatttttaatgccgtaatttaattcatattgaagggttctcgt
aaaacgtaaataattcctgcgtaggacttttgttttgcagtttttacgtcacaagggattataatgaaattaaatatatttactaaatctatgattggtat
ggggctggtgtgttccgctctgccagcattggcaatggaagcatggaataaccaacaaggtggtaataaatatcaggttattttcgatggcaaa
atttatgaaaatgcctggtgggtttcttctacaaattgcccgggaaaagcgaaagcaaatgatgcaactaacccgtggcgtttaaagcgtaccg
caacagctgctgaaattagtcagtttggcaatacactttcctgcgaaaagagcggcagctcatcttcttcaaattcaaatacgcctgcatccaata
cgccggctaatggcggttcggctacaccagcacagggcactgttccgtctaattcttctgtagttgcctggaataaacagcagggcggtcagac
ctggtatgtcgtctttaatggtgcggtatataaaaatgcctggtgggtagcctcttctaactgtccgggtgatgcgaaaagcaatgatgccagcaa
cccatggcgttatgttcgtgccgctacggcaacggaaatctcagaaaccagtaatccacagtcctgtacttcagcaccacagccttcaccggat
gtgaaaccggcaccggacgttaaaccggctcctgatgttcagccagccccagctgataagtcaaacgacaactatgctgtagtagcctggaa
aggtcaggaaggttcttctacatggtacgttatctataacggcggcatttataagaacgcctggtgggtaggcgcggcaaattgcccaggcgat
gcgaaagaaaacgatgccagtaacccatggcgttatgttcgcgcggcaacggcaacagaaatcagccagtatggtaaccctggctcctgttc
cgttaagccggataataatggcggtgctgtgactccggttgatccaactccggaaacaccggtgaccccaactccggataacagcgagccat
caacaccagcggatagcgttaacgattactcattgcaagcgtggagcggccaggaaggtagcgaaatttaccatgttattttcaatggtaatgtt
tacaagaacgcctggtgggttgggtctaaagattgcccacggggtaccagcgctgaaaactccaataacccatggcgtctcgagcgtacagc
taccgctgcggaattgagtcagtacggtaacccgactacctgtgaaattgataacggcggcgtcattgttgcggatggtttccaggccagcaaa
gcgtacagcgcggacagcatcgtagattataacgatgcacattataaaacttctgtcgatcaagacgcatggggctttgtcccgggcggcgat
aacccgtggaagaaatacgaaccggcgaaagcatggtccgcatccactgtgtacgtgaaaggtgatcgcgttgttgttgatgggcaggcttat
gaagcgctgttctggacgcaaagtgacaaccctgctctggtggcgaaccaaaacgccaccggtagcaatagccgcccgtggaagccgtta
ggtaaggctcagagctatagcaacgaagagctgaataatgcgccgcagtttaatccagaaacgctttatgccagcgatacgctgattcgcttta
acggtgtgaactacatttctcagagtaaagtgcagaaagtttctccttctgacagcaacccgtggcgtgtttttgttgactggaccggaaccaaag
agcgcgtaggtacgccgaagaaagcgtggccgaaacacgtttatgcaccgtatgtcgactttacgctgaatacgatcccggatctggctgcgc
tggctaagaatcataacgtcaaccacttcacgctggcgtttgtggtgagtaaagatgcgaacacctgtctgccgacatggggtaccgcttacgg
tatgcagaattacgctcagtacagcaaaatcaaagctctgcgtgaggctggcggcgatgtgatgctgtctatcggtggtgctaacaacgctccg
ctggctgcttcctgtaagaacgtagacgatctgatgcagcattattatgacatcgttgataacctgaacctcaaagtcctggacttcgatatcgaa
ggcacctgggttgcggatcaggcatctattgaacgtcgtaaccttgctgtgaagaaagtgcaggataaatggaagtcagaaggcaaagatatt
gctatctggtacaccttgccaattctgccgactggcctgacgccggaagggatgaatgtcctgagcgatgccaaagcgaaaggtgttgagctg
gcgggtgtgaacgtgatgacaatggactacggtaacgcgatttgtcagtctgcaaataccgaaggccagaacattcacggtaagtgtgcaac
gtctgcgattgccaacctgcattcacaattgaaaggcctccatcccaataagagcgatgcagaaattgacgctatgatgggtaccacgccgat
ggttggcgtgaacgacgttcagggcgaggtgttctatctctctgatgctcgtctggtcatgcaggatgcgcagaagcgtaatctcggtatggttggt
atctggtcaatcgcgcgcgacctgccgggcggcactaacctgtctccggaattccacggcctgactaaagaacaggcaccgaagtacgcatt
tagcgaaatcttcgcgccgtttactaagcaataaatgtgttggcgataccttccagtatcgccgcatttttttgtcaatgggctacttttggtagcccatt
atttttattcatatttctattctttgcttaaaaaagagtctttccgtaattgaaataagaactattttcatttatttttgaagaaagtatacgggagtctctatg
tacgtttgtctttgtaatggtatcagcgataaaaaaaattcgtcaggctgtgcgccagttttcccctcactcgttccagcaattaaaaaaatttattccg
gtcggcaatcagtgcggtaaatgtgtccgggccgcgcgtgaagtgatggaggatgaattaatgcagctgccggagtttaaggagtccgcata

aacggagtgtgtttttgacttactcgtaagccgttctactcttcaaagagtggaagcgaaggagtcaaaaaatgaaaggtgatactaaagttat
aaattatctcaacaaactgttgggaaatgagcttgtcgcaatcaatcagtactttctccatgcccgaatgtttaaaaactggggtctcaaacgtctc
aatgatgtggagtatcatgaatccattgatgagatgaaacacgccgatcgttatattgagcgcattctttttctggaaggtcttccaaacttacagg
acctgggcaaactgaacattggtgaagatgttgaggaaatgctgcgttctgatctggcacttgagctggatggcgcgaagaatttgcgtgaggc
aattggttatgccgatagcgttcatgattacgtcagccgcgatatgatgatagaaattttgcgtgatgaagaaggccatatcgactggctggaaa
cggaacttgatctgattcagaagatgggcctgcaaaattatctgcaagcacagatccgcgaagaaggttgagccgtgtcgggagggagaac
cctcccggttaataagagatccaggccagataccccaggtttatcatgctgccgagcgcaagccacgggccgaatgcgatagtggttatagtc
cggcgttgcagaagttgtgaaacgatggcgtaaccaatgccacccagcgaggcaatcagcagtatcattggcaacgtttgccagccacacc
atgcgcctgcagcggccagtagcttgaaatcaccgtagcccaatccctctttgtgacaaactaaccagacgccccagtaaacacaccacag
caccccatagcccgctaccgcgccgtaaaccgcatcatgtaaatcaattaatccatactgcgcattaaataccaagccaagccagagcagc
ggtaaggtgagtttgtcgggcaggagctgagtgcgaaaatcaataacactgagcgtgagtacgaaacagaaatagagaaagagtgcgcc
agtcacgatgggcgtgaaaggtgccagggcgcaggcgatagcagcagcaacacaaagtatcaggggcactgtatcatgccaggcatatttt
ttttgcctgaaatgaaccaatacctggcgaaacgttaacgccgttttatcctccaacggcgagaggtgataggcgatggcattaacaaaataat
ctgcaataaaaccaaccagaatgaagagcggcagtagcattgtcattattgttgctccctgaaggacaaatgcctgagggtgatctcttcgtttc
cggcaagagtaaactgaaggtcttcaataacaataccgtatgttacctgtgccgtagtgagccagcgggagacgttttcgaggggagctgtga
caggatgaattgtcagagtattgtccgggccgttttccagcgtgatagccaaattctcacgttttgcttcctcgagaaggatatttttgatgggctgcg
taagcgcaggatgaccgagtaaaccatgggtgctcgcttgatcctgcatccatttgatgtccttcttaatttttgcgcgtgggactgatgctctgca
atgtaggtatctatcggttttatcaccccaagccaacaaaaaacgccgagcgagagaaccgccagtgccgcaacgatctgtttttccgatgtgg
atttttctgcccaccatgatttaatcatttccctgagttccttactgtccatacaccactgaccggatctttttctgtttttccatcggaagccaggactgt
gttaactcacagaaacgatcgatattagcttcactcttagcactcatttttaaggtcagcgacttttgcttctggctatagttcgcctccattaactgca
gttcagggtgttccagcagaagtgtttgtaaatgatagagcaacggcacagcttcaggatattgctgagcgagttgctgcttaaagtaaaaacg
aaaattgtgggtgcgtttgatctgcgggaaataacgttgccaggtttcttgctgttgttgctgaagttgatcttcaatcttaagcgtatgccagagcgc
gatgctccggctgccaacaaaagaaagaatggcgagaacgagacagcttactgccaggcgggcgagccattttcccgatttgcttacagtttt
ctgtttacgaaaaataccatgcagcatgtcatagcgctggaatgcaatatcggcagaatacaaactcagtgatggtatttcaggatgctggata
agtggattggccgctgcaacgccgtgaggtactacaccataacaaagcatattctcaggcggaaactgggcagcaagatgctgtaaccagt
gttcatccagttcgttgaaggcatgtgcggcactgcgaatgagccagcttgtttgctggttaaccagagtccagctatctacttcccaggaagat
agcagccatctggcaaaacccgcgtcacgttgagtccggcagtatgtaaacgctcaagatagcgactgagtttctccgactgaatgcctatca
cctcgacactttcgttttgtttatcgacaaccgtccagtgccagtcctgggtgttgtcgggtaacgtctcctctgccagccattgtaatgtttgtgcggt
gagtttgtatttggcgttgggcagcgtgagggaacgatataaaaggccctctggtgggatcaaaagatgaacatttccggcaccgggctgatc
ggccagcgactccagcggaagatcgtccgtgagcgtatgtaccgaagatacgctatctgcggagaacgtcatccattcccaatgcttacgca
gcgtcgaggaagaacgtatgaccatcaacgattcaggcatcacgatccttatgattagtagagttgcagcgtacgccgatagatttgggcgctc
tgctgtttacggttgaaaaaaatatggctttgccatccctgggattgttcgtcgctgatactttccgtactcagcgtaaaatagcggctatagggaaa
gagatgccttttcacctgagcaaccagtggcttcacaccggagaaatcctgctgcgcccagtaaagaaatgcatccgtggttagccagccttcc
ctcggcctttttgcaataagacccgagcgtcagcatccgtgatgttattgaggaataacgctctgaagagcggaatatcgttctccgttagcatat
taagattgatgctaagttccgtggtcgggagaacgcagacatacgggataagccgctgatagatgttttcggttatgccttttatctgacgtaattc
gccagtcagaaaaagcatctgattggcactatgccggggcggcgtctggctttggtaaaagctatcttctgcgccgtgaaagcgtggtgagtca
tcgacatcaatatagtcagcgattgattgcacgatctcatcggtgtttccgcgatcgataccggcgttaatcagcagcgcactgaatacctgggc
ggggaaatcaggggaagccagcgggtcgtcagagatttttgctaacgcattgagattaaaacagtgctgggcatcgcgtaactgccacttcac
tgtattgccatcttccagctgcaactgttgtggctgtgcccagtactgctgcaacgtggtctgtctgtcgttatctttaacgtcctgggtgaggctggca
agcgcaagtttctccgccagttcaatatcatattgccgttgcagatgatggtttacctggcgggttcgctgaagctggctgtgaaaactgagcgtc
atgtctgctgccagagcagccatgagcgccagtaacatgagtacaatgagcagcgcgacgccgcgttgttcattattcatggttattattcccgg
cctgcgtctgagctggcgactcttctctcgctaactgctcaggtaatgcaaaacggcgctgtaacccccatattgctgagtctgcagatgtaacg
tgactgagagaggaagttcctggctttccccgctgttactttccagaagaaagctctcgacatgttccagcatcgcctgggcgggttgatccttcct
gccatccacagaagtacgaacagcacggtagagcgtatgatttcgcagataccagtgaacagtgagtaaggtttgtgcttcactgagaggag
ccagcggatcgcgactttgcgtggtaaaaatagcttcttcacccaacaccatgattttgtccgtactccgattcctccgggcgaccagttgcagga
gatcgttatcaagaatggtgatggtgcgctgtagctgattgaactgctggatttctttctgtgagcgttgatgcaactcggaagcttgcgaaaaaat
cataaacgccagcacgctaagcattgagaaaatcgccagcgccgccatgacctccagcaatgtgaaaccctgctggcgattaatcatcttgt
gcctgcccggatttattatcgatgctttgataacgcgtaagcgaggttgtctgaccgctgggtaatgtcaccgttatggtgcgttccagaagcgtgc
cgtctttgcttgagtggatatcgctgcgccagttccactcttcgccatttattaactctttgccagaactggacgtgttttcctcgccgaatgaatcttgt
gactgaagctggttatccgctatccacaatgccagcgtctcgttacgcattcgctcgatggcatttcgttgcccttgcatactgctcattaaagtcag

agcaacagcggtgaaaatactcatcgccagtaagacttcaagcagtgtcatccctgattgtttgttcatgggcgggtatctctggcgagcgtttga
tccaggggccatgagcctgaacagactaacgtcaggagcggctcaccggtcccggcgttagccatcaacaaagagaagggcgtgatttgtc
cgtcggccagaatcacaacctgtggctggttgctgtcatcaggtttaaatggctgaagatgaattgagagttcttcatcccagtcattttggctctcg
tctgcggcatcttcttgtaatggaacccagcgccaggctgatggtgttttgcccggtaccataatgcgccatgctgaatcagagaaatggatacc
gacaggctgaccactgagcgtggcgcggtcgatggctaattcaagtgcagcggtaaaccgcgccgctgtttcgcgcgcacgagtgctggcg
acatcctcccggccatatgtgaagagcaccacgcttgccgtgatagcgaccagcgccagcaccagcatcatctccagcagggtaaacccgc
gttgctgattcatcgctgacttactttttcttcttgctcaaaccccagttggtgatgtcgtcctcggttcccatttcaccatcgggccctgctgaaagcag
atcgtatgcaccatgttcaccaggattaacgaggacataatcattgccccagggatcggcaggcagacgcttgatataaccttccttgttatagtt
tgcggccagcggtggcagtgtcggcgcttctactaaggattcaagcccctgatttgtggttggatagtgatgattgtcgagtttgtacatatcaagg
gcgttttccagcgccacaatatcgctgacggctttttgcttatccgccttttctttattgcccattaggttaggaaccaccaggctggcaagtacgccg
ataatgacgatcaccaccataatttccagtaatgtaaaaccgcgttgcttatccgttgcgcgcatatgctttccttaattaatcattgagttaagttga
agaagaggttggagtaccgacacgacaataaacaggacgatcagtgccatcgtaataatgagtgctggctcgaagatggagagcgttaag
gcgatccgattttgttggagtgtctcctggttatctgcggctctgaccattaatgtgccgagctgcccgctttttttcgccagaggccaccatgtagag
catcatcggcgggaaaattgcggtttgttccagcgaaagatgaatgctgttaccctggcgaacgttctctgccgcatttgccagacgctggcgaa
tttcgaggttgttgaggctttcggtggacaaattcatcccatccagcagagggacgccgctggattgcaaaatacttaaagtgcggagatagcgt
gcgctgttaatggcgcaaatcagcgggccgatgagcgcaacgcgcagcaacatggcatgaaaacggtggcggttattgccgcgttttaacc
agagccagaaacctacagcgacaataaacactgtcgctaataatgtcgggccggtacgttgcaacgtgtcgctcagacctaaaagaatgcg
tgtactcagcggcagttgctgcttcatatgcacgaactgttcggtaattttgggcacgacagcagtgaggagaataatcacgaccccaatcgcc
accgtagtgagcatacaggggtagatcagtgactgaatgagcttgctgcggattttctgccggttttcattgtaatcagccagcttttccaacaccg
gggccagcagcccgctcttttcgcccgcttttaccagggtacgatagagcgaatcgaaaagcgtgggaaaatgctgtaatgcatcggaaagg
ggatgcccttcaaggatggcgctgcgtacctgatttaacacgtcacccagtcgtttattactgctttgttgaccgattacggcaaggctctcttccag
gggtaatgccgctgcgcttaaggttgccaactgccgggtgaaaagcgtcagttcactatggctgatcctcgggcgacgtgtttttactcccgaact
tttttgggggcgaatatccagcaggaaaagcccttcttcacgcagccgcagtcgtgcctgacgttcatcgttagcatcaatgatcccttgcaattttt
gaccatcctgggtcatggcgcgatagcgataattcatgcttcgctctccagcgtggcactggcgacgcgcataacctcatcccaggaggtgtca
ccgcttatcactttttgcaggccatttttgattaaggccttgtgttgttgccggactagtcgctccagtgcttgttcatccacattttcatgaatagcggcc
cgtaattccggcgtcaccaccatcatttcgtggatcgccatgcgcccctgatagccggattgatggcaatgagggcagcctacgggagtgcca
attgttgtcaccgcgagctgatgatatttaaacatctgcgcttgttggggtgatacgggcgtgaattgtcggcattgcggacacaggcgacgaac
cagacgttgcgcgataatccctgccagggaagacgaaagcaggaatgattcgacgcccatgtcgcggagccgggtcactgcgcctgatgc
actgttagtgtggagtgttgagagtaccagatggccggtgagcgaggcctgaaccgcaatctgcgcggtttctgtatcacgaatttcccccacca
tgacgacatccgggtcctggcgaagtatggcgcgcaggccgcgagcgaaagacatatccacacgggtattcacctgcgtttgcccaatgcct
tccagctcatattccacgggatcttctaccgtcagaatattgcggccgggagtattcagcgccgaaaggatggcgtagagcgtggtgcttttacc
ggagcctgtcggccctgtcaccaggataataccgtgcggaagctgaatgagattttctaaatcctgcttatccgctgccgtcatccccaggttgtt
caatgaaagctggaggctgtttttatccagcaggcggagtacggcgcgttcaccatagatggacggcagtgtggatacgcggacatctatgtta
cgtcgcccgatacgcaaactaattcttccatcctgtggaatacgtttttcggcgatatcaagacgagccatgaccttaattcgggagataagcagt
gccgccagtttttttgtttggctgtaaaattgtccgcaaaacgccgtcgatgcgaaaacggatcgacattgtttttttcataggtttcaatgtggatatca
gaggcggtttctttgatggcctcactcaaaatcgcattgatcaagcggataaccggtgccgctgaatcttcattcaggagatcttcatttgcgggc
atctcttccgaaagcgagagaagatcgacggattgatcgatatcctgcgcaatctgttgcgactcaccggtattctgatggaataccgcttccag
ccgagcttcaaaatcagcctgtgacaagctggtcatggtctggcactggccaacctgacgttgaatatcgagtaaccgctcgaatggcgtatcg
gcgagataaataatctcgccctctgaatagagataaccaattcgttgtgccagcgcccaactggcggggtacggtgagtgaattctcatatgaa
tgcctcaccgtgacgatggcgcaggggcgtgactgttgaacgtgttttcatccagcaccggcaaatcttccgagccaaccagcgcttttgatttcc
cgtcgatccgctgttgttgctcctgacggtaacgggtgtatttctcttttgacagtgagcgataaacatcatcgtcacggataatcgtcggacggat
aaataccatcaggttgcgtttagcgcgctcggtggaggtatagcggaagagttgccccactaaaggaatatcgccaagcagaggaacctttg
acacttgctctttagaaaaatcatccagcaatccgcccaggaccaccgtttcaccggttttgaccagcacggcgttttgaatagtacgggtattaa
acgtcgggccgagcgtcgagttagaggaagagtcaacgctggagacttcctgctctatttcgagcaacaccgcgtcgccttcattgacctgcg
gagtaactttgagttttgtccccaccgttttgcgttcgacggtattaaagacgttatcccctgaagtggtctgtgacccggatagcaccggcacatc
ctgccccacgttgaaggacgcgagtttattatccagcgttacgatgcttggggtggcgaggatgtcatttttattgttactggccagcgcggtaagc
agtactccccagtcgccattgaagaagcctgcggccatgccattgtaggcgctaaacatatcccaggcaggattcgcgctggtgatcccacca
ttctttttataatcagccacaccttgcgcagcgttaaaaatcggtaatccggtattggtaaattgctgtgcgccaacgtttttattcgcccattgcacgc
cgaggtttagtccatttccatcctgaacttcaacgatgattgcctcaaccagcacctgtgcacggcgaatgtccagacgcgcaattaccgtggcg
agtttttcctggacggactggtcagcggtaatgaccagagagttagtctgttcatcggcggtaatggcgacgttatccatcgcgccagaagagg

agggcttacgcgcattccctttttcatctttcagcttttcggaaacaccggttagcacttccaccagattcgtggctttagcatatttcaggtaataaac
ccgggtatttccttcctcgctctcttcgacatcaaggcttttcagtaatgaagtgatgcgctggcgtgctttttccggtccactgatgataagagagttg
gttcgcttatccgccacaatcttcgcggagaggagggctggcatctggcttttaccgtggctttcgctgattaattgattaagaatctcggcgagat
cttccgctgacgcatattccagatgaataatttgctgcttctctgtgccgatgacatcaacgcgctttatgacttcaatcagtttattaatggtggagg
cacgaccggtcagaataagaacgttggagggttcataatgcacaacattaccgacgctacccgcatccatcatctggcggagcagggggc
caggtcacgagcaggaacgttctcaagcggtacaattcgggtgaccaactcatcacctacgcctggacgagaactgtcagcaatcatccctg
gcgatgtttttacattagctgagcgaaccactttcagaaaaccattgtccagcgtgatcacggaataaccgtaaagatcaagaatacttaaaaa
gaactggtagtactcctgttggctaaacgtatcattactgcgtacggaaatggttccctgtaccgaagggtcgatcaggatcgttttgccaagatg
ctgacccactatttccacgaactggcggatatcggcgttattgaagttcgcgccgtattgttcgttctcagcgtgtcctgcacaaggcatgagtagt
gctgccagcaagcagcaggtgattttattgagtcctttcatgacgcagtccattcattcctgtaagttccccgacggaaacatttatcaagcggggt
acgccgttgcgacgaattgtaaactgagcactttgttgagttagcagtaaacttaatgcctgtgaaacctcatcagggtgggtcagcgaaagatt
attgatcctgagggcgatatcccctggttgtagcaagctggtggtaaatgcattgaggcccttacgagggttcaatcgaagcccgtatatctgttct
ccatcacgtataggcgtggcgataatataatcagcgagtacattttgactttctgaatcaggctgcttgatgatgtcaggtagccctggatggaga
gttactttttcgtaatgcccgtgagtttcaaacacaacgctggttttatttatttttctaataaacgttcccggcgctgactcaagggcttcgttgaggga
gtatcgcttttgttcgctcccctcattaattaagacaaaagaagtctcatcattcgatgtgagtacaatgccattaagcgctatagagagcttaggtg
tttcctgattaactgccacgggatttgcatcttttaccccagcatgattttcgttacgccataaagcggcaaggctgaaatcagactgaaatgcgttt
gttggttgattaatgatctgtccatttttaactatagttgtatggaaataattcacattaaaaataatcgaccctatgcttataaatataatcaatatattg
atggcagcatctttattatgattcgccagatggaaggggaaacgtagtgtgggcacgatgtatgttctaataaaatagattgggtgggttgtatatt
gacgttagtataaatagtattaatgccgcttgcatgaatcaagcaaaatttatttatattagtaatatgtatttaattaataaatacatacattatttttatg
attaaggataatcaataattaaaacaggaagttctatgtctacgagaagagaagttattctttcctggttgtgtgagaaacgtcaaacctggcgtct
atgctatttgttgggtgaggctggaagtggaaaaacctggctggcgcagcaactgcaaaaagataaacatcgccgtgtgattactttaagcctc
gttgtttcctggcaaggtaaggccgcatggatcgttaccgacgataacgcggctgaacagggctgccgtgacagcgcctggacgcgagatg
agatggcggggcaattactgcatgcgcttcatcgaactgatagtcgctgccctcttattattattgaaaatgctcacctgaatcaccgccgcatact
tgatgatctccaacgcgcaatatctcttattcctgacggtcaatttttgctgatcggcagacccgatcgaaaagtcgaacgtgattttaaaaaaca
gggcattgaacttgtctcgataggacgtctgacggagcacgaacttaaggcaagcatccttgaaggtcagaatattgaccaacccgatctcctt
ttaaccgccagagttctgaaacggatagctttattatgtcggggcgatcgcagaaagctggcgcttgccggcgaaacaattcgcttattacagc
aagccgagcagaccagcgttttcacagcaaaacaatggcgaatgatttaccgcatattgggtgataaccgcccccggaaaatgcagcttgcc
gtggtgatgagtggaaccattatcgctcttacctgtggttggctactgctgtcctcttttacagctacgttacccgttcctgcgtggctgatacccgtaa
cgccagtagttaaacaagatatgactaaggatattgctcatgtggtgatgcgcgatagtgaggcgctaagcgtgttatacggcgtatgggggta
cgaggtcccagctgattcggcctggtgcgatcaggcagtaagagcggggctggcatgtaaaagtgggaatgcttctttacaaacattggtaga
tcagaatttgccctggatcgcatcgcttaaagtcggggacaaaaaattgcctgttgtggtcgttcgcgtcggtgaggctagcgttgatgtcctcgta
ggtcagcaaacgtggacgctgacacataaatggtttgagtcggtatggacgggtgattatcttctgttgtggaaaatgtcgccggaaggggaga
gtaccattacgcgtgatagcagcgaggaagagattctctggctggaaacgatgttaaaccgcgcattgcatatttcgacagaaccttcggcgg
agtggcgtcccttgttggtagaaaaaataaagcaatttcaaaaaagccaccacttgaaaactgacggtgttgttggcttttcaacgttagtacatc
tctggcaggtggcaggggagagtgcctacttatatcgggatgaagcaaatatttccccggaaacaacagtgaaggggaaatgacatgtttga
attctatatcgccgcgcgtgagcagaaagaaacaggtcaccctggtatcttctcccgacaaaaacacagcaccataatatatgtaatttgtttatt
actgatttgcctgtggtttgcaggaatggtgctggttggtgggtatgccaggcagctatgggtgctttggatagtaaaagccgaagtcactgtaga
ggctgaaacgcccgcttttaaacaatcaacacaacactatttcttcaaaaaacagccactccccgtcgtcgaatccgtcgaggaggaagatg
acccaggcgtcgcggttgagaatgccccctcttcttctgaagacgaagaaaatactgtggaagaaagcgaagagaaagccggtttaagaga
gcgtgtcaaaaatgcactgaacgaacttgagagataacccgaaggctgtttactttactagcaatacgcttgcgttcggtggttaagtatgtataa
tgcgcgggcttgtcgtagttgacagcaggttcaatctgaacctcagttagccgaatttggctacctaacaatgctcccaatcggggagctacgta
agaacggttacactctcccatcaatcgtaatgggtctgaggagtaatcattttcgtttataaaataattggagctctggtctcatgcagaaccaaag
aatccgtatccgcctgaaagcgtttgatcatcgtctgatcgatcaagcaaccgcggaaatcgtcgagactgccaagcgcactggtgcgcaggt
ccgtggtccgatcccgctgccgacacgcaaagagcgcttcactgttctgatctccccgcacgtcaacaaagacgcgcgcgatcagtacgaa
atccgtactcacttgcgtctggttgacatcgttgagccaaccgagaaaaccgttgatgctctgatgcgtctggatctggctgccggtgtagacgtg
cagatcagcctgggttaatcaggtcattgagcgattgagaggttgaaacaatgattggtttagtcggtaaaaaagtgggtatgacccgtatcttca
cagaagacggcgtttctatcccagtaaccgtaatcgaagttgaagcaaaccgcgttactcaggttaaagacctggctaacgatggctaccgtg
ctattcaggtgaccaccggtgctaaaaaagctaaccgtgtgaccaagcctgaagctggccacttcgctaaagctggcgtagaagctggccgt
ggtctgtgggaattccgcctggctgaaggcgaagagttcactgtaggtcagagcattagcgttgaactgtttgctgacgttaaaaaagttgacgt
aactggcacctctaaaggtaaaggtttcgcaggtaccgttaagcgctggaacttccgtacccaggacgctactcacggtaactccttgtctcacc

gcgttccgggttctatcggtcagaaccagactccgggcaaagtgttcaaaggcaagaaaatggcaggtcagatgggtaacgaacgtgtaac
cgttcagagccttgacgtagtacgcgttgacgctgagcgcaacctgctgctggttaaaggtgctgtcccgggtgcaaccggtagcgacctgatc
gttaaaccagctgtgaaggcgtaaggagatagcaatggaattagtattgaaagacgcgcagagcgcgctgactgtttccgaaactaccttcg
gtcgtgatttcaacgaagcgctggttcaccaggttgttgttgcttatgcagctggtgctcgtcagggtactcgtgctcagaagactcgtgctgaagt
aactggttccggtaaaaaaccgtggcgccagaaaggcaccggccgtgcgcgttctggttctatcaagagcccgatctggcgttctggtggcgt
gacctttgctgctcgtccgcaggaccacagtcaaaaagttaacaagaagatgtaccgcggcgcgctgaaaagcatcctgtccgaactggtac
gtcaggatcgtctgatcgttgtcgagaagttctctgtagaagcgccgaaaactaagctgctggcacagaaactgaaagacatggctctggaag
atgtgctgatcatcaccggtgagctggacgaaaacctgttcctggctgcgcgcaacctgcacaaggttgacgtacgcgatgcaactggtatcg
acccggttagcctgatcgccttcgacaaagtcgtaatgactgctgatgctgttaagcaagttgaggagatgctggcatgattcgtgaagaacgtc
tgctgaaggtgctgcgtgcaccgcacgtttctgaaaaagcgtctactgcgatggaaaaatccaacaccatcgtactcaaagttgctaaagacg
cgaccaaagcagaaatcaaagctgctgtgcagaaactgtttgaagtcgaagtcgaagtcgttaacaccctggtagttaaagggaaagttaaa
cgtcacggacagcgtatcggtcgtcgtagcgactggaaaaaagcttacgtcaccctgaaagaaggccagaatctggacttcgttggcggcgc
tgagtaagtcggaggagtaatacaatggcagttgttaaatgtaaaccgacatctccgggtcgtcgccacgtagttaaagtggttaaccctgagc
tgcacaagggcaaacctttgctccgttgctggaaaaaaacagcaaatccggtggtcgtaacaacaatggccgtatcaccactcgtcatatcg
gtggtggccacaagcaggcttaccgtattgttgacttcaaacgcaacaaagacggtatcccggcagttgttgaacgtcttgagtacgatccgaa
ccgttccgcgaacatcgcgctggttctgtacaaagacggtgaacgccgttacatcctggcccctaaaggcctgaaagctggcgaccagattca
gtctggcgttgatgctgcaatcaaaccaggtaacaccctgccgatgcgcaacatcccggttggttctactgttcataacgtagaaatgaaacca
ggtaaaggcggtcagctggcacgttccgctggtacttacgttcagatcgttgctcgtgatggtgcttatgtcaccctgcgtctgcgttctggtgaaat
gcgtaaagtagaagcagactgccgtgcaactctgggcgaagttggcaatgctgagcatatgctgcgcgttctgggtaaagcaggtgctgcac
gctggcgtggtgttcgtccgaccgttcgcggtaccgcgatgaacccggtagaccacccacatggtggtggtgaaggtcgtaactttggtaagca
cccggtaactccgtggggcgttcagaccaaaggtaagaagacccgcagcaacaagcgtactgataaattcatcgtacgtcgccgtagcaaa
taattttagaggataagccatgccacgttctctcaagaaaggtccttttattgacctgcacttgctgaagaaggtagagaaagcggtggaaagc
ggagacaagaagcccctgcgcacttggtcccgtcgttcaacgatctttcctaacatgatcggtttgaccatcgctgtccataatggtcgtcagcac
gttccggtatttgtaaccgacgaaatggttggtcacaaactgggtgaattcgcaccgactcgtacttatcgcggccacgctgctgataaaaaag
cgaagaagaaataaggtaggaggaagagatggaaactatcgctaaacatcgccatgctcgttcttctgctcagaaggttcgccttgttgctgac
ctgattcgcggtaagaaagtgtcgcaggctctggatattttgacctacaccaacaagaaagcggctgtactggtcaagaaagttctggaatctg
ccattgctaacgctgaacacaacgatggcgctgacattgacgatctgaaagttacgaaaattttcgtagacgaaggcccgagcatgaagcgc
attatgccgcgtgcaaaaggtcgtgcagatcgcatcctgaagcgcaccagccacatcactgtggttgtgtccgatcgctgagactctggagact
agcaatgggtcagaaagtacatcctaatggtattcgcctgggtattgtaaaaccatggaactctacctggtttgcgaacaccaaagaattcgctg
acaacctggacagcgattttaaagtacgtcagtacctgactaaggaactggctaaagcgtccgtatctcgtatcgttatcgagcgtccggctaa
gagcatccgtgtaaccattcacactgctcgcccgggtatcgttatcggtaaaaaaggtgaagacgtagaaaaactgcgtaaggtcgtagcgg
acatcgctggcgttcctgcacagatcaacatcgccgaagttcgtaagcctgaactggacgcaaaactggttgctgacagcatcacttctcagct
ggaacgtcgcgttatgttccgtcgtgctatgaagcgtgctgtacagaacgcaatgcgtctgggcgctaaaggtattaaagttgaagttagcggcc
gtctgggcggcgcggaaatcgcacgtaccgaatggtaccgcgaaggtcgcgtaccgctgcacactctgcgtgctgacatcgactacaacac
ctctgaagcgcacaccacttacggtgtaatcggcgttaaagtgtggatcttcaaaggcgagatcctgggtggtatggctgctgttgaacaaccg
gaaaaaccggctgctcagcctaaaaagcagcagcgtaaaggccgtaaataaggagcgtcgctgatgttacaaccaaagcgtacaaaattc
cgtaaaatgcacaaaggccgtaaccgcggtctggcgcagggtacggatgttagcttcggcagcttcggtctgaaagctgttggccgtggtcgt
ctgactgcccgtcagatcgaagcagcacgtcgtgctatgacccgtgcagttaagcgtcaaggtaagatctggatccgtgtgttcccggacaaa
ccgatcactgaaaagccgctggcagtgcgtatgggtaaaggtaaaggtaacgtggagtattgggttgccttgattcagccgggtaaagtcctgt
atgaaatggacggtgttccggaagagctggcccgtgaagcattcaagctggcagcagcgaaactgccgattaaaaccacctttgtaactaag
acggtgatgtaatgaaagcaaaagagctgcgtgagaagagcgttgaagagctgaacaccgagctgctgaacctgctgcgtgagcagttca
acctgcgtatgcaggctgcaagtggccagctgcaacagtctcacctgttgaagcaagtgcgtcgcgatgtcgcacgcgttaagactttactgaa
cgagaaggcgggtgcgtaatgaccgataaaatccgtactctgcaaggtcgcgttgttagcgacaaaatggagaaatccattgttgttgctatcg
aacgttttgtgaaacacccgatctacggtaaattcatcaagcgtacgaccaaactgcacgtacatgacgagaacaacgaatgcggtatcggt
gacgtggttgaaatccgcgaatgccgtccgctgtccaagactaaatcctggacgctggttcgcgttgtagagaaagcggttctgtaatacagta
cactctctcaatacgaataaacggctcagaaatgagccgtttatttttctacccatatccttgaagcggtgttataatgccgcgccctcgatatggg
gatttttaacgacctgattttcgggtctcagtagtagttgacattagcggagcactaaaatgatccaagaacagactatgctgaacgtcgccgac
aactccggtgcacgtcgcgtaatgtgtatcaaggttctgggtggctcgcaccgtcgctacgcaggcgtaggcgacatcatcaagatcaccatc
aaagaagcaattccgcgtggtaaggtcaaaaaaggtgatgtgctgaaggcggtagtggtgcgcaccaagaagggtgttcgtcgcccggac
ggttctgtcattcgcttcgatggtaatgcttgtgttcttctgaacaacaacagcgagcagcctatcggtacgcgtatttttgggccggtaactcgtga

gcttcgtagtgagaagttcatgaaaattatctctctggcaccagaagtactctaaggagcgaatcatggcagcgaaaatccgtcgtgatgacga
agttatcgtgttaaccggtaaagataaaggtaaacgcggtaaagttaagaatgtcctgtcttccggcaaggtcattgttgaaggtatcaacctggt
taagaaacatcagaagccggttccggccctgaaccaaccgggtggcatcgttgaaaaagaagccgctattcaggtttccaacgtagcaatctt
caatgcggcaaccggcaaggctgaccgtgtaggctttagattcgaagacggtaaaaaagtccgtttcttcaagtctaacagcgaaactatcaa
gtaatttggagtagtacgatggcgaaactgcatgattactacaaagacgaagtagttaaaaaactcatgactgagtttaactacaattctgtcatg
caagtccctcgggtcgagaagatcaccctgaacatgggtgttggtgaagcgatcgctgacaaaaaactgctggataacgcagcagcagac
ctggcagcaatctccggtcaaaaaccgctgatcaccaaagcacgcaaatctgttgcaggcttcaaaatccgtcagggctatccgatcggctgt
aaagtaactctgcgtggcgaacgcatgtgggagttctttgagcgcctgatcactattgctgtacctcgtatccgtgacttccgtggcctgtccgcta
agtctttcgacggtcgtggtaactacagcatgggtgtccgtgagcagatcatcttcccagaaatcgactacgataaagtcgaccgcgttcgtggt
ttggatattaccattaccactactgcgaaatctgacgaagaaggccgcgctctgctggctgccttgacttcccgttccgcaagtaaggtagggtt
actaaatggctaagcaatcaatgaaagcacgcgaagtaaaacgcgtagctttagctgataaatacttcgcgaaacgcgctgaactgaaagc
gatcatctctgatgtgaacgcttccgacgaagatcgttggaacgctgttctcaagctgcagactctgccgcgtgattccagcccgtctcgtcagcg
taaccgctgccgtcaaacaggtcgtccgcatggtttcctgcggaagttcgggttgagccgtattaaggtccgtgaagccgctatgcgcggtgaa
atcccgggtctgaaaaaggctagctggtaattgtcaccaattgaatcacgggagtaaagacagatgagcatgcaagatccgatcgcggatat
gctgacccgtatccgtaacggtcaggccgcgaacaaagctgcggtcaccatgccttcctccaagctgaaagtggcaatcgccaacgtgctga
aggaagaaggttttattgaagattttaaagttgaaggcgacaccaagcctgaactggaacttactctgaagtatttccagggcaaagctgttgta
gaaagcattcagcgtgtcagccgcccaggtctgcgcatctataaacgtaaagatgagctgccgaaagttatggcgggtctgggtatcgcagtt
gtttctacctctaaaggtgttatgactgatcgtgcagcgcgccaggctggtcttggtggcgaaattatctgctacgtagcctaatcggaggaaaaa
atgtctcgtgttgctaaagcaccggtcgttgttcctgccggcgttgacgtaaaaatcaacggtcaggttattacgatcaaaggtaaaaacggcga
gctgactcgtactctcaacgatgctgttgaagttaaacatgcagataataccctgaccttcggtccgcgtgatggttacgcagacggttgggcac
aggctggtaccgcgcgtgccctgctgaactcaatggttatcggtgttaccgaaggcttcactaagaagctgcagctggttggtgtaggttaccgt
gcagcggttaaaggcaatgtgattaacctgtctctgggtttctctcatcctgttgaccatcagctgcctgcgggtatcactgctgaatgtccgactca
gactgaaatcgtgctgaaaggcgctgataagcaggtgatcggccaggttgcagcggatctgcgcgcctaccgtcgtcctgagccttataaag
gcaagggtgttcgttacgccgacgaagtcgtgcgtaccaaagaggctaagaagaagtaaggtaacactatggataagaaatctgctcgtatc
cgtcgtgcgacccgcgcacgccgcaagctccaggagctgggcgcaactcgcctggtggtacatcgtaccccgcgtcacatttacgcacaggt
aattgcaccgaacggttctgaagttctggtagctgcttctactgtagaaaaagctatcgctgaacaactgaagtacaccggtaacaaagacgc
ggctgcagctgtgggtaaagctgtcgctgaacgcgctctggaaaaaggcatcaaagatgtatcctttgaccgttccgggttccaatatcatggtc
gtgtccaggcactggcagatgctgcccgtgaagctggccttcagttctaaggtagaggtgtaagatggctcacatcgaaaaacaagctggcg
aactgcaggaaaagctgatcgcggtaaaccgcgtatctaaaaccgttaaaggtggtcgtattttctccttcacagctctgactgtagttggcgatg
gtaacggtcgcgttggttttggttacggtaaagcgcgtgaagttccagcagcgatccagaaagcgatggaaaaagcccgtcgcaatatgatta
acgtcgcgctgaataacggcactctgcaacaccctgttaaaggtgttcacacgggttctcgcgtattcatgcagccggcttccgaaggtaccgg
tatcatcgccggtggtgcaatgcgcgccgttctggaagtcgctggggttcataacgttctggctaaagcctatggttccaccaacccgatcaacg
tggttcgtgcaactattgatggcctggaaaatatgaattctccagaaatggtcgctgccaagcgtggtaaatccgttgaagaaattctggggaaa
taaaccatggcaaagactattaaaattactcaaacccgcagtgcaatcggtcgtctgccgaaacacaaggcaacgctgcttggcctgggtctg
cgtcgtattggtcacaccgtagagcgcgaggatactcctgctattcgcggtatgatcaacgcggtttccttcatggttaaagttgaggagtaagag
atgcgtttaaatactctgtctccggccgaaggctccaaaaaggcgggtaaacgcctgggtcgtggtatcggttctggcctcggtaaaaccggtg
gtcgtggtcacaaaggtcagaagtctcgttctggcggtggcgtacgtcgcggtttcgagggtggtcagatgcctctgtaccgtcgtctgccgaaa
ttcggcttcacttctcgtaaagcagcgattacagccgaaattcgtctgtctgacctggctaaagtagaaggcggtgtagtagacctgaacacgct
gaaagcggctaacattatcggtatccagatcgagttcgcgaaagtgatcctggctggcgaagtaacgactccggtaactgttcgtggcctgcgt
gttactaaaggcgctcgtgctgctatcgaagctgctggcggtaaaatcgaggaataagtagcagatggctaaacaaccgggattagattttca
aagtgccaaaggtggcttaggcgagctgaaacgcagactgctgtttgttatcggtgcgctgattgtgttccgtattggctcttttattccgatccctgg
tattgatgccgctgtacttgccaaactgcttgagcaacagcgaggcaccatcattgagatgtttaacatgttctctggtggtgctctcagccgtgctt
ctatctttgctctggggatcatgccgtatatttcggcgtcgatcattatccagctgctgacggtggttcacccaacgttggcagaaattaagaaaga
aggggagtctggtcgtcgtaagatcagccagtacacccgctacggtactctggtgctggcaatattccagtcgatcggtattgctaccggtctgc
cgaatatgcctggtatgcaaggcctggtgattaacccgggctttgcattctacttcaccgctgttgtaagtctggtcacaggaaccatgttcctgatg
tggttgggcgaacagattactgaacgaggtatcggcaacggtatttcaatcattatcttcgccggtattgtcgcgggactcccgccagccattgcc
catactatcgagcaagcgcgtcaaggcgacctgcacttcctcgtgttgctgttggttgcagtattagtatttgcagtgacgttctttgttgtatttgttga
gcgtggtcaacgccgcattgtggtaaactacgcgaaacgtcagcaaggtcgtcgtgtctatgctgcacagagcacacatttaccgctgaaagt
gaatatggcgggggtaatcccggcaatcttcgcttccagtattattctgttcccggcgaccatcgcgtcatggttcgggggcggtactggttggaa
ctggctgacaacaatttcgctgtatttgcagcctgggcaaccgctttatgtgttactctatgcgtctgcaatcatcttcttctgtttcttctacacggcgtt

ggttttcaacccgcgtgaaacagcagataacctgaagaagtccggtgcatttgtaccaggaattcgtccgggagagcaaacggcgaagtata
tcgataaagtaatgacccgcctgaccctggttggtgcgctgtatattacctttatctgcctgatcccggagttcatgcgtgatgcaatgaaagtacc
gttctacttcggtgggacctcactgcttatcgttgttgtcgtgattatggactttatggctcaagtgcaaactctgatgatgtccagtcagtatgagtctg
cattgaagaaggcgaacctgaaaggctacggccgataattggtcgcccgagaagttacggagagtaaaaatgaaagttcgtgcttccgtca
agaaattatgccgtaactgcaaaatcgttaagcgtgatggtgtcatccgtgtgatttgcagtgccgagccgaagcataaacagcgccaaggct
gatttttcgcatattttcttgcaaagttgggttgagctggctagattagccagccaatcttttgtatgtctgtgcgtttccatttgagtatcctgaaaacg
ggcttttcagcatggaacgtacatattaaatagtaggagtgcatagtggcccgtatagcaggcattaacattcctgatcataagcatgccgtaatc
gcattaacttcgatttatggcgtcggcaagacccgttctaaagccatcctggctgcagcgggtatcgctgaagatgttaagatcagtgagctgtct
gaaggacaaatcgacacgctgcgtgacgaagttgccaaatttgtcgttgaaggtgatctgcgccgtgaaatcagcatgagcatcaagcgcct
gatggatcttggttgctatcgcggtttgcgtcatcgtcgtggtctcccggttcgcggtcagcgtaccaagaccaacgcacgtacccgtaagggtc
cgcgcaaaccgatcaagaaataatcggggtgattgaataatggcaaaggcaccaattcgtgcacgtaaacgtgtaagaaaacaagtctctg
acggcgtggctcatatccatgcttctttcaacaacaccatcgtgactatcactgatcgtcagggtaacgcgttgggttgggcaacagccggtggtt
ccggtttccgtggttctcgcaaatccactccgtttgcagctcaggttgcagcagagcgttgcgctgacgccgtgaaagaatacggcatcaagaa
tctggaagttatggttaaaggtccgggtccaggccgcgaatctactattcgtgctctgaacgccgcaggtttccgcatcactaacattactgatgtg
actccgatccctcataacggttgtcgtccgccgaaaaaacgtcgcgtataacgcctcgttttccaggtttgttggagaaagaaaatggcaagat
atttgggtcctaagctcaagctgagccgtcgtgagggcaccgacttattccttaagtctggcgttcgcgcgatcgataccaagtgtaaaattgaa
caagctcctggccagcacggtgcgcgtaaaccgcgtctgtctgactatggtgtgcagttgcgtgaaaagcaaaaagttcgccgtatctatggtg
tgctggagcgtcagttccgtaactactacaaagaagcagcacgtctgaaaggcaacaccggtgaaaacctgttggctctgctggaaggtcgt
ctggacaacgttgtataccgtatgggcttcggtgccactcgtgcagaagcacgtcagctggttagccataaagcaattatggtaaacggtcgtgt
tgttaacatcgcttcttatcaggttagtccgaatgacgttgtaagcattcgtgagaaagcgaagaagcagtctcgcgtgaaagccgctctggagc
tggctgagcagcgtgaaaagccaacctggctggaagttgatgctggcaagatggaaggtacgtttaagcgtaagccggagcgttctgatctgt
ctgcggacattaacgaacacctgatcgtcgagctttactccaagtaaagcttagtaccaaagagaggacacaatgcagggttctgtgacaga
gtttctaaaaccgcgcctggttgatatcgagcaagtgagttcgacgcacgccaaggtgacccttgagcctttagagcgtggctttggccatactct
gggtaacgcactgcgccgtattctgctctcatcgatgccgggttgcgcggtgaccgaggttgagattgatggtgtactacatgagtacagcacc
aaagaaggcgttcaggaagatatcctggaaatcctgctcaacctgaaagggctggcggtgagagttcagggcaaagatgaagttattcttac
cttgaataaatctggcattggccctgtgactgcagccgatatcacccacgacggtgatgtcgaaatcgtcaagccgcagcacgtgatctgcca
cctgaccgatgagaacgcgtctattagcatgcgtatcaaagttcagcgcggtcgtggttatgtgccggcttctacccgaattcattcggaagaag
atgagcgcccaatcggccgtctgctggtcgacgcatgctacagccctgtggagcgtattgcctacaatgttgaagcagcgcgtgtagaacagc
gtaccgacctggacaagctggtcatcgaaatggaaaccaacggcacaatcgatcctgaagaggcgattcgtcgtgcggcaaccattctggct
gaacaactggaagctttcgttgacttacgtgatgtacgtcagcctgaagtgaaagaagagaaaccagagttcgatccgatcctgctgcgccct
gttgacgatctggaattgactgtccgctctgctaactgccttaaagcagaagctatccactatatcggtgatctggtacagcgtaccgaggttgag
ctccttaaaacgcctaaccttggtaaaaaatctcttactgagattaaagacgtgctggcttcccgtggactgtctctgggcatgcgcctggaaaac
tggccaccggcaagcatcgctgacgagtaaccggatcacaggttaaggttttactgagaaggataaggtcatgcgccatcgtaagagtggtc
gtcaactgaaccgcaacagcagccatcgccaggctatgttccgcaatatggcaggttcactggttcgtcatgaaatcatcaagacgactctgc
ctaaagcgaaagagctgcgccgcgtagttgagccgctgattactcttgccaagactgatagcgttgctaatcgtcgtctggcattcgcccgtact
cgtgataacgagatcgtggcaaaactgtttaacgaactgggcccgcgtttcgcgagccgtgccggtggttacactcgtattctgaagtgtggctt
ccgtgcaggcgacaacgcgccgatggcttacatcgagctggttgatcgttcagagaaagcagaagctgctgcagagtaatctgaagcaacg
taaaaaaacccgccccggcgggttttttatacccgtagtatccccacttatctacaatagctgtactctttttgttcatcccctggagtatttatgtggtt
acttgaccagtgggcagagcgccatatagcagaagcgcaagcgaaaggtgagtttgataacctggcaggtagcggcgaaccattgatactg
gatgatgattctcacgtgccaccggaattacgtgcggggtatcgcttgctgaagaatgccggttgcttaccgccagaacttgagcaacggaga
gaagcaattcagcttctggatattctcaaaggtatccgtcacgatgatccgcaatatcaagaggttagccgtcgattgtcattactggaattgaag
ctgcgacaagctggattgagtaccgatttttttacgcggcgattatgctgacaagttgttggacaaaatcaacgataactagtggagtatgtatgtat
cgcattggtgagctggcaaaaatggcggaagtaacacccgacacgattcgttattacgaaaaacagcagatgatggagcatgaagtgcgta
ctgaaggtgggtttcgcctatataccgaaagcgatctccagcgattgaaatttatccgccatgccagacaactaggtttcagtctggagtcgatcc
gcgagttgctgtcgatccgcatcgatcctgaacaccatacctgtcaggagtcaaaaggcattgtgcaggaaagattgcaggaagtcgaagca
cggatagccgagttgcagagtatgcagcgttccttgcaacgccttaacgatgcctgttgtgggactgctcatagcagtgtttattgttcgattcttga
agctcttgaacaaggggcgagtggcgttaagagtggttgttgatttttgcactggcaggattacactcgcgccgttaaataaccaactggagtttt
tatgagtcgatatcagcatactaaagggcagataaaggataatgcgatagaagcattactacatgatcccttattccgacagcgcgtagagaa
aaataagaaggggaaaggcagttacatgcgaaaaggaaaacatggcaatcgggggaactgggaggccagtggcaagaaagtaaatca
ctttttaccactggtcttctgctttcaggcgcttgttaagagcggttattctgctctttcagcaaatcacgaatttctgtcagtaatacttcttctttagttgg

tgcaggtgcggctgctggttcttctttttccgattcagtttgttgattagcttaatcgccataaagatggcaaaggccacaatcagaaaatcaaaga
cgttttgaatgaagacaccgtaatgcatcacaacagcagggatatccccctgcgcatcgcgtagcgtgacagcaaactgtttaaaatcgatcc
cgccaattaataagcccagaggaggcatgatgatatcggcaaccagtgaagagacaatcttcccgaatgccgcaccgataatgacaccca
ccgccaaatccaccacgttcccgcgcatcgcaaattcgcgaaattctttaataatgctcatgttattctccctatgaagccgacaaccataagtct
aacaaatgttaagccattttcctgccaggaatgaatttaattaatccttgataatcaaagggaaataataaggcgtcattagacgccttattaatta
caagaagaaagggcttggctggaagagtctttcgacgtcggtaataaactttttatctgtgaggaacataattacgtgatcgccttgctcaatgcg
cagattgtcattggcaatcatcacgtcgtttccacgtaccaccgctccaataatcgttcctggcggtagcttgatttcgtcaatgactctgccgacaa
cgcgtgaggtgctttcatcaccgtgagcaacggcttcaatagcttctgctacgccgcggcgcaatgaggaaacaccaacaatatctgcttttcg
cacatggctaagcaacgcagaaatagttgcttgttgtggtgaaatcgcaatatcgataacgctcccctgaaccagatccacataagcgcgacg
ctggatcaataccatcacctttttcgcacccatacgtttggcaagcatggcggacatgatattggcctcgtcatcgttggtgacggcaataaacag
atcaacttgatcgatatgttcttcggccagtagttcttgatccgacgcatcaccaaaaaagacgatcgtattctgtaacttttccgccagttcggcag
cgcgctgctgattacgttcgatgagtttaacgctgtaatctttttccagacgacgcgccagccctgcaccgatattaccgccaccaaccagcatg
atccgcttatacggtttttccagtcgctgtaattcactcatcaccgcgcggatatgctgtgaagcggcaataaagaacacttcatcaccagcttca
acaatggtcgaaccttgcggacgaatggggcgatcgtggcggaaaattgctgccacacgagtatcgatatgtggcatatgttcgcgcatggtc
gaaagtgcattaccaatcagcgggccgccataataggctttaaccacagccaggctgactttaccctcagcgaagttcaccacctgcaatgcg
ccgggatactcaatcagtcggtaaatattatcgataaccaactgctctggtgcgatcagatgatcaatcggcacagcatctgaatgaaatagctt
atcggcatcgcgcacgtagtctggtgagcggatacgagcgatgcgattaggggtgttgaaaagtgagtaggctacctggcaggcaaccatat
tggtttcatctgaactggttacagcaaccagcatatcggcgtcgtcggcacctgcctcccgcaatacgcgtggatgagagccatgcccctgcac
gacccgcaggtcaaatttatcctgtaaggtccgcagacgctcaccgttggtatcgacaacagtaatatcgttgttctcgccaaccaggttttccgc
cagtgtgccgccaacctggccggcacccagaatgataattttcatcagtcgcgacccgttatctcatcactttttgattagcttagcgtaaaagaa
gccgtcgccctcttcggcaccaggtagattttgtttacccggttgctctggtgttcctgtttcgcaaagttcggcatcagcggtacgttgcaaaaagg
ctttaatctgcaggctattctcttccggtaacaccgaacaggtggcatagaccagagttccaccggtttttaaatgcggccaaatggcgtcgaga
atttcagactgcaattgcgcgagttccgggatatcgcgatcgcgacgtaaccatttaatatctggatggcgacgaatcacaccggttgctgaac
aaggcgcatctaataaaatgcgatcaaactgttgctcgccacaccattgagaagggtaacggccatcaccttgtttcacggtcgccttcatacc
aaggcgttttaaattgtcgtaaacccgagagaggcgctgttcgtcgatatcaaccgcaacaacctgcgcttctggtgccacctcaaggatatgc
gttgttttaccgccgggggcggcacaaagatccaaaatgtgttcaccgttttgtggcgcaagccaggtcatgcaaccttgtgctgatgcatcctga
acggtaacccatccgtcttcaaaaccaggtagcgcatgaacaggtgcaggtgtttccagacgtacagcatcagggtaatccgcatgcgggaa
acctttcattcctgcttcatccagcaatgcaagccagctgtcgcgggaatgatgcgtacgattaatacgcagccacattggcggacgctggttatt
ggcttcgacgatggattgccactgctctggatacgctttttgcagacgcttcagcaaccaggaaggatgcagataacgtgcatcactggcatta
aactcggctaataactcttcttgctgacgctggaactggcgtaatacaccgtttatcaaccctttaagttgcggacgcttaattgcgatagcgccttc
aaccgtttcagccagcgcagcatgaggtggaatgcgggtataaagcagttgatacaaaccaaccataatcaggtaatgcacggtccgctgttt
gccggtcatcggacgggccattaacttattaatcagccagtctaactgcgaaagcgtacgcagtacgccaaagcacaactcttgaagaagtg
ctttgtctttatcggaaactttttgctggagcggtggcagaatgttgcttaatgattgcccttgctcgacgacttgttcaacggcctgggccgccatgct
acgtaaattacgttgtttttcataagtataaaaacgcccggcaagaccgggcttagaagagtggactatcagaccagacggttgcccggaac
aaaccattcccgacgagagttcaggaggtcttgcgcgctcatcgctttcttacccgcaggttgtaacgagagcaggttcaggatgccatcacca
gtcgcaacctgaatgccttgtttgttggcttcaaggatcgttcctggtgcagcgttggttgccgtatcaatgaccgatgctttccagactttaaccggc
tgtccttcaatttccagccagctcattggccatggattgaaagcgcgaatgcagcgttcaagctgtgctgccgaaagtgaccagtcaatacgcg
cttcttctttactcaacttctcggcgtaagtgacaagagtttcgtcctgaacttctggtttcgccgtgccgtctgccagttgtttcaacgtggtgataagc
ccttgtgggccaagctctgccagcttgtcgtacagcgtaccactggtatcttctgcagtaatcgggcaggagagcttatagagcatatcaccggt
gtctaaaccgacatccatttgcataatggtcacaccagtttctgcatcacccgcccatagtgagcgttggattggtgcagcaccgcgccagcgtg
gcagcagtgaaccatgaacgttgatacagccaagacgcggcatctccagcactgctttcggcagaattaaaccataggcgacgacgaccat
aacatcagcctgcagttcggcgaccagttgctggttttcttgtggacgcagggaaacaggttgaaaaacgggcagacctttttcctcagccaga
actttaaccgggctgggcatcagtttttaccgcgtcctgccggtcggtctggctgggtgaacacgccaacgacgttatgaccagaagacaaca
gcgcgtcgagatgacgcgctgcaaagtcaggtgtacccgcaaaaataatacgtagtgattctgacacgttagttcttatccttaagcccgggctt
tcagacgatccagtttttcaactttctgacgaatacgttgttgtttcagcggtgacagataatccataaacagtttgccgaccaggtgatccatctca
tgctgaatacagatggctaacagaccgtctgcttccagttcaaatggtttaccgtcgcggtcaagggcgcgaattttaactttctctgcgcgcggc
actaaagcacgttgttcagggatcgacaggcaaccttcttcaatgcctgtttcgccgctttttctaaaagctctggattgattaacactagccgttcg
tcacggttttccgaaacatcaataacaatgatacgttgatggatatcaacctgggttgccgccaggccaataccttcttctgcgtacatcgtctcga
acatatcatcgacgatacgctgaatttctgcattcacttcttctaccggtttagcaactttgcgaagccgctcgtccggaatatgtaacacttgcaaa
actgacataaatctccagagatgtgttcaggagttagaaagattatttcttctattctagacaaatccccctctgattgacagcatcactgaccaat

cgcaaagattgctaaggctgcttatggcagggagataaggatggtcgatacagaaatttggctgcgtttaatgagtatcagcagcttgtacggc
gatgatatggtccgtatcgctcactgggtggcaaaacagtcgcatattgatgcggttgtattgcagcaaacagggcttacattgcggcaggcac
aacgctttctttcatttccacgaaagagtatcgaaagctcactttgttggttggagcaacccaaccatcatttaattcctgcggacagcgaattttatc
ctcctcaacttctggcgacgacagattaccccggcgcactgtttgttgaaggagaactgcacgcgctgcattcatttcagcttgccgtagtgggg
agtcgggcgcattcatggtatggcgagcgatggggacgattattttgcgaaactctggcgacgcgtggagtgacaattacgagtggactggcg
cgtggaatcgatggtgtagcgcataaagcagccttacaggtaaatggcgtcagcattgctgtattggggaatggacttaataccattcatccccg
ccgtcatgcccgactggctgccagtctgcttgaacagggggcgctctcgtctcggaatttcccctcgatgttccaccccttgcttacaatttccca
cgaagaaatcgcattatcagtggtctaagtaaaggtgtactggtggtggaagcggctttgcgtagtggttcgctggtgacagcacgttgtgcgctt
gagcaggggcgagaagtttttgccttgccaggtccaatagggaatccgggaagcgaagggcctcactggttaataaaacaaggtgcgattct
tgtgacggaaccggaagaaattctggaaaacttgcaatttggattgcactggttgccagacgcccctgaaaattcattttattcaccagatcagc
aagacgtggcattgccatttcctgagctcctggctaacgtaggagatgaggtaacacctgttgacgtcgtcgctgaacgtgccggccaacctgt
gccagaggtagttactcaactactcgaactggagttagcaggatggatcgcagctgtacccggcggctatgtccgattgaggagggcatgcc
atgttcgacgtactaatgtatttgtttgaaacctatattcacacagaagctgagttgcgtgtggatcaagacaaacttgaacaggatcttaccgac
gcaggttttgagcgagaagatatctacaatgccctgctatggctggaaaaacttgctgattatcaggaagggttggcagaaccgatgcaactg
gcctcggatcctctctccatgcgtatttatacaccggaagagtgtgaacgactggatgccagctgccgtgggtttctgcttttccttgagcagattca
ggtgctcaaccttgaaactcgtgaaatggtgatagagcgagtgctcgcgctggataacgcagagttcgaactggatgatctgaaatgggtgat
cctgatggtgttgttcaatattccgggctgcgaaaatgcgtaccagcaaatggaagaattactctttgaagtgaatgaaggtatgctgcattaatc
atttctttaattcagcataagttgttatggcgaaatcagcactgttcacggtgcgtaataatgagtcctgcccaaagtgcggggctgaactggttatt
cgatccgggaaacacggtccgtttcttggatgctcacagtatccggcgtgtgactacgtccgtcctctgaaatcttcagcggatggacatatcgtc
aaagttctggaggggcaggtttgccctgcatgtggcgcaaatctggtattacgccagggacgctttggtatgtttattggttgcattaactaccctga
atgcgaacataccgaacttatcgataaaccggacgaaacagcaattacatgcccccaatgtcggacgggccatctggtccagcgccgctcc
cgttatggcaaaacatttcactcttgtgatcgctacccggagtgtcaatttgccattaacttcaaacccatagctggagaatgccctgagtgtcatta
tccgctactcatcgaaaagaaaaccgcgcagggtgtaaaacacttttgtgccagtaaacaatgtggaaagccggtttcggcggaataataac
gtgaataataacctgcaaagagacgctatcgcagctgcgatagatgttctcaatgaagaacgtgtcatcgcctatccaacggaagccgttttcg
gtgttgggtgcgatcctgatagcgaaacagcagtgatgcgactgttggagttaaaacagcgtccggttgataaggggctgattttaatcgcagc
aaattacgagcagcttaaaccctatattgatgacaccatgttgactgacgtgcagcgtgaaaccatttttcccgctggccaggtcctgtcaccttt
gtctttcccgcgcctgcgacaacaccgcgctggttgacgggccgctttgattcgcttgctgtacgagtcaccgaccatccgttggtggttgctttgtg
ccaggcttatggtaaaccgctggtttctaccagtgccaacttgagtggattgccaccttgtcgaacagtagacgaagttcgcgcacaatttggcg
cggcgttcccggttgtgcctggtgaaacgggggggcgtttaaatccttcagaaatccgcgatgccctgacgggtgaactgtttcgacaggggta
acataatggaaacctatgctgtttttggtaatccgatagcccacagcaaatcgccattcattcatcagcaatttgctcagcaactgaatattgaac
atccctatgggcgcgtgttggcacccatcaatgatttcatcaacacactgaacgctttctttagtgctggtggtaaaggtgcgaatgtgacggtgc
cttttaaagaagaggcttttgccagagcggatgagcttactgaacgggcagcgttggctggtgctgttaataccctcatgcggttagaagatgga
cgcctgctgggtgacaataccgatggtgtaggcttgttaagcgatctggaacgtctgtcttttatccgccctggtttacgtattctgcttatcggcgctg
gtggagcatctcgcggcgtactactgccactcctttccctggactgtgcggtgacaataactaatcggacggtatcccgcgcggaagagttggc
taaattgtttgcgcacactggcagtattcaggcgttgagtatggacgaactggaaggtcatgagtttgatctcattattaatgcaacatccagtggc
atcagtggtgatattccggcgatcccgtcatcgctcattcatccaggcatttattgctatgacatgttctatcagaaaggaaaaactccttttctggca
tggtgtgagcagcgaggctcaaagcgtaatgctgatggtttaggaatgctggtggcacaggcggctcatgcctttcttctctggcacggtgttctg
cctgacgtagaaccagttataaagcaattgcaggaggaattgtccgcgtgaatcaggccatccagtttccggacagggaagagtgggacga
gaataaaaaatgtgtatgttttcccgctctcgtgaatggtatgcaactgacatgcgcgatctctggcgagagtctggcgtatcgctttactggagat
acgccagaacagtggttagcgagttttcgtcagcatcgctgggacctggaagaagaagcggaaaacttaattcaggaacaaagtgaagatg
atcaaggctgggtctggttaccctgatccagatattcgtccttccatttcacgtaattattcgcggaatagcgtaacccagccttctcttcatcactta
acgggcggatctgtttgacggggctaccgagatacagatatccgctctccagccgtttattttgtgggaccagactacccgcaccaatcatcac
atcatcttctactattgcgccatcaagtaaaattgagcccatcccaaccaaaactcgattgccaatggtgcagccgtggagcatcaccttgtgac
caacagtgacatcttcgccaatggttaatgggttgccatctgggttgtacgaggatttatgagtgacatgcaacatactgccatcctggatattggt
gcgtgctccgatctgtacataatgtacatctccacgaatcacaacgagcggccagatccccacatcatcagccagacgaacgtcaccaatca
cgacactgctatcgtcgatcattacgcgctgaccgatttgtggaaaaagatcgcggtatgggcgtaaaacatcagacatacttacctcagcaat
aaatgatttactaatgactttgggggcattattggccttgtgcaagtcttttagtatgcaaaaaagcaccgttttgtgtgcgattgcagcaaaaaggg
tgaaaaaacaacaaacagaaaaaaagatcaaaaaaatacttgtgcaaaaaattgggatccctataatgcgcctccgttgagacgacaacg
tgaaacacttcacaggatggtcggaacaacgaagagaaaaaaatcctgaaattcagggttgactctgaaagaggaaagcgtaatatacgc
cacctcgcgacagtgagctgaaagccgcgtcgcaactgctctttaacaatttatcagacaatctgtgtgggcactcgaagatacggattcttaac

gtcgcaagacgaaaaatgaataccaagtctcaagagtgaacacgtaattcattacgaagtttaattctttgagcatcaaacttttaaattgaaga
gtttgatcatggctcagattgaacgctggcggcaggcctaacacatgcaagtcgaacggtaacaggaaacagcttgctgtttcgctgacgagt
ggcggacgggtgagtaatgtctgggaaactgcctgatggagggggataactactggaaacggtagctaataccgcataacgtcgcaagac
caaagagggggaccctcgggcctcttgccatcggatgtgcccagatgggattagcttgttggtggggtaacggctcaccaaggcgacgatcc
ctagctggtctgagaggatgaccagccacactggaactgagacacggtccagactcctacgggaggcagcagtggggaatattgcacaat
gggcgcaagcctgatgcagccatgccgcgtgtatgaagaaggccttcgggttgtaaagtactttcagcggggaggaagggagtaaagttaat
acctttgctcattgacgttacccgcagaagaagcaccggctaactccgtgccagcagccgcggtaatacggagggtgcaagcgttaatcgga
attactgggcgtaaagcgcacgcaggcggtttgttaagtcagatgtgaaatccccgggctcaacctgggaactgcatctgatactggcaagctt
gagtctcgtagaggggggtagaattccaggtgtagcggtgaaatgcgtagagatctggaggaataccggtggcgaaggcggccccctgga
cgaagactgacgctcaggtgcgaaagcgtggggagcaaacaggattagataccctggtagtccacgccgtaaacgatgtcgacttggaggt
tgtgcccttgaggcgtggcttccggagctaacgcgttaagtcgaccgcctggggagtacggccgcaaggttaaaactcaaatgaattgacgg
gggcccgcacaagcggtggagcatgtggtttaattcgatgcaacgcgaagaaccttacctggtcttgacatccacggaagttttcagagatga
gaatgtgccttcgggaaccgtgagacaggtgctgcatggctgtcgtcagctcgtgttgtgaaatgttgggttaagtcccgcaacgagcgcaacc
cttatcctttgttgccagcggtccggccgggaactcaaaggagactgccagtgataaactggaggaaggtggggatgacgtcaagtcatcatg
gcccttacgaccagggctacacacgtgctacaatggcgcatacaaagagaagcgacctcgcgagagcaagcggacctcataaagtgcgt
cgtagtccggattggagtctgcaactcgactccatgaagtcggaatcgctagtaatcgtggatcagaatgccacggtgaatacgttcccgggcc
ttgtacacaccgcccgtcacaccatgggagtgggttgcaaaagaagtaggtagcttaaccttcgggagggcgcttaccactttgtgattcatga
ctggggtgaagtcgtaacaaggtaaccgtaggggaacctgcggttggatcacctccttaccttaaagaagcgtactttgcagtgctcacacag
attgtctgatgaaaatgagcagtaaaacctctacaggcttgtagctcaggtggttagagcgcacccctgataagggtgaggtcggtggttcaag
tccactcaggcctaccaaatttgcacggcaaatttgaagaggttttaactacatgttatggggctatagctcagctgggagagcgcctgctttgca
cgcaggaggtctgcggttcgatcccgcatagctccaccatctctgtagtgattaaataaaaaatacttcagagtgtacctgcaaaggttcactgc
gaagttttgctctttaaaaatctggatcaagctgaaaattgaaacactgaacaacgaaagttgttcgtgagtctctcaaattttcgcaactctgaag
tgaaacatcttcgggttgtgaggttaagcgactaagcgtacacggtggatgccctggcagtcagaggcgatgaaggacgtgctaatctgcgat
aagcgtcggtaaggtgatatgaaccgttataaccggcgatttccgaatggggaaacccagtgtgtttcgacacactatcattaactgaatccata
ggttaatgaggcgaaccgggggaactgaaacatctaagtaccccgaggaaaagaaatcaaccgagattcccccagtagcggcgagcga
acggggagcagcccagagcctgaatcagtgtgtgtgttagtggaagcgtctggaaaggcgcgcgatacagggtgacagccccgtacacaa
aaatgcacatgctgtgagctcgatgagtagggcgggacacgtggtatcctgtctgaatatggggggaccatcctccaaggctaaatactcctg
actgaccgatagtgaaccagtaccgtgagggaaaggcgaaaagaaccccggcgaggggagtgaaaaagaacctgaaaccgtgtacgt
acaagcagtgggagcacgcttaggcgtgtgactgcgtaccttttgtataatgggtcagcgacttatattctgtagcaaggttaaccgaatagggg
agccgaagggaaaccgagtcttaactgggcgttaagttgcagggtatagacccgaaacccggtgatctagccatgggcaggttgaaggttg
ggtaacactaactggaggaccgaaccgactaatgttgaaaaattagcggatgacttgtggctgggggtgaaaggccaatcaaaccgggag
atagctggttctccccgaaagctatttaggtagcgcctcgtgaattcatctccgggggtagagcactgtttcggcaagggggtcatcccgacttac
caacccgatgcaaactgcgaataccggagaatgttatcacgggagacacacggcgggtgctaacgtccgtcgtgaagagggaaacaacc
cagaccgccagctaaggtcccaaagtcatggttaagtgggaaacgatgtgggaaggcccagacagccaggatgttggcttagaagcagcc
atcatttaaagaaagcgtaatagctcactggtcgagtcggcctgcgcggaagatgtaacggggctaaaccatgcaccgaagctgcggcagc
gacgcttatgcgttgttgggtaggggagcgttctgtaagcctgcgaaggtgtgctgtgaggcatgctggaggtatcagaagtgcgaatgctgac
ataagtaacgataaagcgggtgaaaagcccgctcgccggaagaccaagggttcctgtccaacgttaatcggggcagggtgagtcgacccc
taaggcgaggccgaaaggcgtagtcgatgggaaacaggttaatattcctgtacttggtgttactgcgaagggggggacggagaaggctatgtt
ggccgggcgacggttgtcccggtttaagcgtgtaggctggttttccaggcaaatccggaaaatcaaggctgaggcgtgatgacgaggcacta
cggtgctgaagcaacaaatgccctgcttccaggaaaagcctctaagcatcaggtaacatcaaatcgtaccccaaaccgacacaggtggtca
ggtagagaataccaaggcgcttgagagaactcgggtgaaggaactaggcaaaatggtgccgtaacttcgggagaaggcacgctgatatgt
aggtgaagcgacttgctcgtggagctgaaatcagtcgaagataccagctggctgcaactgtttattaaaaacacagcactgtgcaaacacga
aagtggacgtatacggtgtgacgcctgcccggtgccggaaggttaattgatggggttagcgcaagcgaagctcttgatcgaagccccggtaa
acggcggccgtaactataacggtcctaaggtagcgaaattccttgtcgggtaagttccgacctgcacgaatggcgtaatgatggccaggctgt
ctccacccgagactcagtgaaattgaactcgctgtgaagatgcagtgtacccgcggcaagacggaaagaccccgtgaacctttactatagctt
gacactgaacattgagccttgatgtgtaggataggtgggaggcttagaagtgtggacgccagtctgcatggagccgaccttgaaataccaccc
tttaatgtttgatgttctaacgttgacccgtaatccgggttgcggacagtgtctggtgggtagtttgactggggcggtctcctcctaaagagtaacgg
aggagcacgaaggttggctaatcctggtcggacatcaggaggttagtgcaatggcataagccagcttgactgcgagcgtgacggcgcgagc
aggtgcgaaagcaggtcatagtgatccggtggttctgaatggaagggccatcgctcaacggataaaaggtactccggggataacaggctga
taccgcccaagagttcatatcgacggcggtgtttggcacctcgatgtcggctcatcacatcctggggctgaagtaggtcccaagggtgatgctgt

tcgccatttaaagtggtacgcgagctgggtttagaacgtcgtgagacagttcggtccctatctgccgtgggcgctggagaactgaggggggctg
ctcctagtacgagaggaccggagtggacgcatcactggtgttcgggttgtcatgccaatggcactgcccggtagctaaatgcggaagagata
agtgctgaaagcatctaagcacgaaacttgccccgagatgagttctccctgaccctttaagggtcctgaaggaacgttgaagacgacgacgtt
gataggccgggtgtgtaagcgcagcgatgcgttgagctaaccggtactaatgaaccgtgaggcttaaccttacaacgccgaagctgttttggc
ggatgagagaagattttcagcctgatacagattaaatcagaacgcagaagcggtctgataaaacagaatttgcctggcggccgtagcgcggt
ggtcccacctgaccccatgccgaactcagaagtgaaacgccgtagcgccgatggtagtgtggggtctccccatgcgagagtagggaactgc
caggcatcaaatttagcgtgctgatatggctcagttggtagagcgcacccttggtaagggtgaggtccccagttcgactctgggtatcagcacc
actttttaggttaaagttcggcagattagaaaagaatttgtctggcggcagtagcgcggtggtcccacctgaccccatgccgaactcagaagtg
aaacgccgtagcgccgatggtagtgtggggtctccccatgcgagagtagggaactgccagacatcaaataaaacaaaaggctcagtcgga
agactgggccttttgttttatctgttgtttgtcggtgaacactctcccgagtaggacaaatccgccgggagcggatttgaacgttgcgaagcaacg
gcccggagggtggcgggcaggacgcccgccataaactgccagacatcaaatcaagcgaaaggccatccgaaaggatggcctttttgctttt
cgaactaacattcaattaatggattacctgcgataaaaatgccctcgtacgctctgatttaggatgcgcaaaaaattcatcaggtgcagcttgctc
cactatttccccacgatccataaaaattacccggtcagcgacggttcgtgcaaaccccatctcatgtgttacacacaacattgtcatacccgact
gcgccagcccaatcatcgtatccagcacctctttcaccatctcaggatcgagcgccgacgttggctcatcaaacaacataattttcggcttcata
cacagcgaacgcgcaatggcaacgcgttgctgctgaccacctgaaatctgtccgggaaacttatgcgcatgttcggcaattctcacccgctcta
ggtaatgcaccgccagatcttcagcctctttcttaggcatcttgcgtacccaaatcggtgccagggtacagttctgtaaaacggtcagatgaggg
aagagattgaaatgctgaaagaccattcccacttcctgcctgacgcgctcaatattgcggatatcttcattaagttcgatgccatctaccacgatc
cgtccctgttgatgttcttccagatgattaatacaacgaatggttgtcgatttaccggaacctgaagggccacacagaacgatccgttctcccggtt
gcacggttaaatttatatttttcaaaacatggaattgtccataccatttattgacgttttccagcgtaatcatcgcgttagcaggttgcagtaaaatttg
gctcatagtgtcctcaatgcggtgtacgcccggtgttaaaacgtttttccagatactggctatagcgcgacatgctgaaacagaagatccagtag
atcagcgcggcgaaaacataccсttccgtcgacatacccaaccaggcgggatcaacggttgcctgctgaacgctactgaaaagatcgaac
aacccgataatgatcaccaggctggtatctttgaagagtgcgatgatggtatttaccagcccaggaattaccagcttcaacgcctgtggcagaa
taaccagcccctgagttttccagtaacccaacgccagcgactctgccgcttcatattgccctttaggcagcgcctgtaatccacctcgcacgactt
ccgcaacatatgctgactgaaacaggatcacgccaaccagcgcgcggatcaatttgtcgatactggtgccttctgccataaacaacggcagc
atgaccgaagacataaacagaacggtaatcagcggcacgccgcgccagaattcgataaaaatgaccgataagatacgcacaatcggcat
atgggagcggcgacctaacgccagtaagatcccccacggtagcgcccccgcaatcccaactgatgcaataattaaagttagcgtcagcccg
ccccattgccgggtttcaacccgctcaagagcaaaaaaaccgccatacatcagccaccagacaatcagtgggtaaatcaccgcccaggcc
gcaatatagcgaccgcgatgcgggagtattttccagaacattggtgcgatcgacacaagcccaatcagtaatgcgaggttaattcgccagcgt
tggtcgtgtgggtaaagcccatacataaactgaccaaatcgttcgtggatgaagacccaacaggcaccggcttttgtgcagtcggcacgcgtt
gagccaacccagttagcctgcaaaaatgcccagttcagcaacggtgggatcagttcccacatcaaccatatgcagccaatagtcagcaggc
tattgctccagctggagaacagatttttcgcacccacaccatcgcgcggcttgagttatggctcgccgggcgcgggggatgagacagcaata
cttttgtcatcatgactccttagcgttcaacgatcgcgatgcggcggttatagatattcatcagcagcgagatagtcaggctgataatcagataga
ccgacatggtcatggctatcgtctcgatggcttgccccgtctgattcagcacggtgccggcaaacagcgaaaccatatcgggatagccaatag
cggcggcaagagaggagtttttgacgatgttgagatactggctggttaacggtggaataatcacccgcaatgcctggggaataatgacctggc
gtagcgtaaccgggttgggtaatcccagtgaccgagccgcttcatgttgaccataaggcactgcctggatcccagcgcggataatctcggcga
taaatgcagatgtataaaccgaaagtgccagcgttaaggctgccagttcaggaattaaaaccatcccgccgcggaagttaaagcctcgtagg
gctgggacatcccagtgtagtgctgcgccaaaaagccattgcgccagtaaaggcaaaccaatgatcaacactgcggcgatcggccaggtg
cggcgcagttgtccggtctttatctggtatgttttattaaagcggaatagcccaacagaaaggactatagccataacaacagccaggataaacg
caataaatccgtctcccagctgcggtgacggaatataaagcccacgattgctcaaaaaggccagatcaaacgcgctcacagcctggcgtgg
tccgggcaaattgcgcaacacggcaaagtaccagaagaagatttgcagcagcgggggaatattacggaagatctcaatataaattgtggaa
agctttcgtagcagccagttatccgaaagtctcgccagaccgataaagaagcccaaaacagaagcgaacacaatacacaatgcagaaac
cagtagcgtattgagtaagccgacaataaaaacgcgtccgtaggtgtcgccctgctggtaatcgatcaaatgctggacaataccgaagccag
cgccgcgatccagaaaggcaaaacctgaagtaatgccacgattattgagattcgttacagtgttgtgaaataaccaaccaacaatgccgaca
acagcaacaacggcaaggatctggaataaccaggcgcgaaccgtagggttggcaaaagagagtgagcctttaacggttgagcggcgatg
agacatagaaacctcggaaagcataacgctgaagagagcgccgccgtagcgacgctcgttacctcacaccgacttaacgcacgggcggt
gcgtactgaataccgccgttattccagagattattttgcccacgtttaattttcagcgggctttctgaacctacgttacgctcaaaaatttccgagtagt
tacccacctgtttgatgatgttataggcccatttattatccagcttcagatccttgccgtaatcgccttctttacccagcagatgtgccatatcaggcgt
tgctggattagccgctttttcatcgacgttctgggaattgatgcccatctcttcagcattcagcatggcgaaaagcgtccagcgtacaatcgagaa
ccattcatcatcgccacgacgaactaccggaccaagcggttcttttgagataacttccggtaagacaatccattcagctgggttgcttaatttgatg
cgcagggcatacagttgtgattgatccgaggccagcgtatcgcagcgaccagattccagtgccttcgctgattcgtcagagcgatcgaaagtc

accggtgtgtacttcatattgtttgccttgaagtagtcggcgacgttgagttcggtatcagtacccgcctgaatacagacggtagcgccatccagtt
ctttcgcgctttttagccccgctttatcgtgcgtcaggaagccaatgccgtcgtaataagtgacgccggtaaatgccattcccatcccggcatcgc
gagatgaagtccaggtcgtattacgggagagcaaatccacctcccctgactgtaaagcggtgaagcgttcttttgcagtgagcggggtatatttc
actttcgtgtcgtcaccaaatacagcagcggcaacaccacgacaaatatcaacatcaatacctgaaaacttaccgtcagcatcggcataaga
gaacccaggtaatccatcactgatcccgcattgcacaaaacctttttctgaacggcatcaagcgtcgcgccagcagcgcctgatttgcaacgg
caagcagcacgctggcggcagccagtgtggctatcatcatctttttcataatgcttcctgtgaggcgaaaattatcgttataggacgtttgggaaa
cgtcttttacctgtctgtggccatcctcttataagcaaaagtagtgccagcttttgcgcacgatttcgcgtcgcaataagctgagtgtagacaggaa
ataataaaaacagcgccccgcaatggtgcgaaaatgaaacatgcgccacattttggcgcaaaatatttacgcgtgttttactaagcagaataat
gattgttagcgtgagtatatttctggtgggaagtgtgaaaattaaattgatgcgttatttcgaaagcagcgaaataacgcgatgtaacatgctaaat
cagcagggaattacccagcagaatatagcaatattttatcttatggagagatactgtatcgttaaaaagagcgcggaactccgcgccctgttca
gttatttcgatagagcgatgatccccatcgtcaggccagcaacagcagctgccccgccggcaatagccccagccgtttcccagttatcctgagt
cgtgcctttcatacaggtattggtgtgaaccagacgaccctgatcgtcataaaccggaacgcagggcgaatcgtgagcacagcccgccagc
aatgcggtgagcaatgccacaggaattaatcttttcatggtgttacctcagtaataaacgctaacgcttaataagttaactctcttattaacgagctg
aatgccattttatcaccggaggttataaatcttgcggaatgaataatgacaaattatgtgaattgtaaaaatagtgaagggagcgggaactatag
aaagcttttaaagagtgagcagaagaatatcttattaatttttatgggtttcactggaaataataaaggcacccgaaagcgcctttatgtttctgattt
atcctttaaagcaacggcggatcaccacaaagaacaccggtacgaagaagattgccagcaacgttgcagagaccattcctcccattacccc
gatacccacagcgttctgcgcgccactgccggcaccgttactgatagctagcggtaatacgccgagaataaaggcgagagaggtcatcagg
ataggacgcagacgcatacgtactgccatcagtgtcgcttcaacaacacctttaccctctttctccatgagatctttagcgaactcaacgatcaaa
atagcgtttttggccgacaagccaattgtcgttagcaagcccaccataaagtagacgtcatttttttgattaaagagtgtcgccgccagcagcac
gccgacaatccctaacggcactaccaacataaccgagacaggaattgaccagctttcatagagtgcagcaaggcacaggaaaacaacca
caaaggaaattgctaccagagcgggagcctggtttcccgataagcgttcctgatacgacatacccgtccagtcataaccaatgcccgcaggt
aattttgacgcaaggttttccatcaacgccatggcatcgccggaactggttcctggcgcggcttccccctgaatctccattgacggcagaccgttg
tagcgttccagtcgcggagagccatacacccaatgtgaagtggtaaaggccgagaatggcaccatttcgccgttggcgctgcggacataaa
gtttatcgacatcttctggcagcatacggaatttggcatccgcctgaacatacaactttttcacgcggccacggtcgatgaagtcgttaacgtaagt
cccacccagcgccgttgaaatggtctgattgatgtcagaaagtgaaacacctaatgcctgcgccttttcctggtcaacttccagtttaaactgcgc
ggtgtcttccaggccattagggcgcacgctgactaagctggcaggatgttgcgccgccataccaagcaactggttacgggcctgggttagggc
atcgtgacccagcccagcctgatcaattaactcaaagtcgaaacccgttgccgtgcccagttcaacaatggctggcatattgaatggaatgac
aaaaccgtcgcggatcttgcccaattccattttggcacgatggattaccgcttccgcactgttttcgtcaccattacgctcttcccacggtttcagact
gacgaaggccataccggcgttttgtgcctggccgctgaagctaaagccgttaaccgtaaagacactttcaacgttcgctttctcgttcttcagata
gtaatccgtaacttgatccaacactttttgcgtccgctcttgcgtcgcgccagcgggtaactgaatcatggtcagaaagacaccctgatcctcttca
ggtaagaaggaagacggaagacgtaaaaacaacaccaccattcctgcaacaatcagcgcatagatcagtaaatatcgtcctgtggatccg
aggattttgccgacgctgttggtgtagtggttaacgctatgatcgaaggtggtattaaaccaaccgaagaaaccgcccttattttcgtgatgctcag
cagagacgggtttaagcagcgttgcacataacgcaggggtaagaatcaatgccaccagaacagaaagcgccattgccgaaacgatggtg
atagagaactggcgataaattgccccagtagaaccgccgaagaatgccatcggaataaataccgctgacagcaccatcgcgatacccacc
agtgcgccctgaatttgcgacatcgatttttccgtcgcttcttttggcgggagcttatcctccatcatcacgcgctcgacgttctccaccaccactatc
gcatcatcgacgagcagcccgatggcaagcaccatcccgaacatcgttagtgtgttgatggagtaaccaaaagcggcgaggatggcaaac
gtccctaacaacaccacgggtaccgcaatggtggggatcagcgttgctcgcatattctgcaagaacagatacatcaccaggaacaccagca
taatggcttcgaacagcgtttttaccacttcgtgaatagaaagctggacgaatggcgtggtgtcataagggtagagaaccttcattccctgcggg
aagaatggctgtaattccgccagttttgccttaatggctttcgcggtatcgagagcattcgcgccggttgccagcttaatccccaggcccgccgcc
ggttttccgttgatacgagcgataacgttatagttttcaccgccaagttcaacccgtgcgacatctttcaggcgtaccaccgagccgtcactgttta
cgcgcagggtcactttgccgaattcttccggatttttaaaccgcgtctgagcaataatcgaggcgttcaattgttgccctggtaacgctggcgttcc
gcccaactgtccggcagcgatctgatcgttctgtaccttcaactggttaatcacatcaaccggtgtcagtttatatttgtttagcagatcggcatcca
gccagatacgcatcgcatactgtgcgccgaaaagctgtacgtcaccgacgccattcagacggctaagcgtatctttaacgttagaggccacat
agtccgagatatcgtcctgtgtggtgcctgggttatcagagacaaagcccgccaccatcaaatagctgctactggacttttcaacactgatcccc
tgctgctgaacctcctgcggcagcaacggcgtggcgagctggagtttgttctgcacctgcacttgcgcgatatcaggatcggtcccggactgga
aggtaagggtaattgtcacgctaccggcggaatcgctggtggaggacatatacatcaggttatcgataccgttcatattctgttcgataacctgcg
tcaccgtatcctgcacggtctgcgcatcagcgcccggatagtttgctgaaacagaaaccgcaggcggtgcaattgttggatactgagcgacgg
gcaattgtaggatcgccagtgcgcccgccatcatcagaataatggccagcacccatgcaaatatcggtcgtcgaataaaaaagtttgccatgt
cagattaccttacttcgatgcagtatctgcgggggtatcggtagtggctttaacctgctctcccggacgcgctttttgcaggccgctgacaatgactt
gatcgccagatttcagtccttcactaatcaaccatttatcgccaatcgcctgactggcaacgacagggcgcgcttcaacctgacttttatcgttaac

aatcagcacggttgcatcaccacgcggtgtgcggctaacgccttgttgcgggataagaatggcgtcaggttggacgccttcatcaatccgtgca
cgcacaaacatacccggcaaaagcgtatgttgcgggttagggaagacagcacgtagggttatggagccggtgctttcatcaacggtcacatc
ggagaattgcagcgtacctttcaggggataggtttgaccgttttccatgaccaactctacgttgctggtggcgttttccttatgcaaatttccttgctct
acggattgcttcagcctcataaaatcgttgctggattgggtcacatcaacgtagataggatcgagctgctggacagtcgccagttcagtcgtttgc
ccattagtgacaagagcgccttcggtcacagtcgatttgccgatacgtccgctaattggcgcagtgactttggtataagcaagattgatgcgagc
gctttcgactgtggcttttgcggcaatcacggcggcatcggcctgacgagcatcagcaatggcctggtcgtactcctgctggctgatgtatttcgta
cccacgagcggaacgtaacgttttaccgtcaaatgcgcgatggcggcggcggcttcacttttcgccagttcgcctttcgcgctgtcataatttgcct
gataggtcgcgggatcgatctggtacagggactggcctgcttgcacatcgctgccttcagtgaaattgcgattcagtacgatcccgctaacctgt
gggcgaacttcggctatacgataagcattggtgcggcctggtaattcagtcttaacttctaacggggccgtttttacaatatgaacggtaacctgc
ggttcaccgacgtgagctttctcttctcccttatcgttacaaccggcgattaaagccgcggagatcagaataaaggagggcaggaggaaaaa
cctggcatgtttcgtcattactattcctcaaaaaaccaaaagcgcgttatttacccaaaaaggcaacgcgttaactcgcagaaagaaaaatac
agttcgctatcctacaaattatcattcgtcgatgtaaggaatagttatgaatacaggcatctcaaggcacataaacacaaaaaaagattaatatt
ctactgttttattttgacgcgggttgaaagaggcagaattaaaacctcgtaaattgaaatatatattgatgtagtgaatgtatcttaggtaaataatat
atattatttatttaacgatttatagcaactcaatattagcctctcgtataaatacacattaggtgatagattaaccttcgctattttctcactctgtgtcgaa
tatatttatttcctgaataattaatcatggcaaaaagaaccaaagccgaagctctgaagacccggcaagaactgattgaaactgccatcgccc
agtttgcgcagcatggcgtaagcaagacgacgctcaacgacattgccgacgccgctaacgttacgcgtggcgctatctactggcacttcgaa
aacaagactcaactgtttaatgagatgtggttgcaacagccttcattgcgggagttaatccaggaacacttgacggctggattagagcatgacc
cgtttcaacaattgcgtgaaaaattgattgtcggcttgcaatatattgccaaaattccccgccagcaggcgttgctgaaaatcttatatcacaaatg
tgaatttaatgatgagatgctggccgagggagtgatacgcgaaaagatgggctttaatccgcagactctccgcgaagtattgcaggcgtgtca
gcaacaaggttgtgtagcaaataacctcgatttagatgttgtgatgattattattgatggtgccttcagcggaattgttcaaaactggttaatgaatat
ggcgggttatgatctttataaacaagcccccgctctggtcgataacgtattaagaatgttcatgccagatgaaaacataacgaaattaattcatca
aacgaatgaattaagtgtcatgtaaattcacattctgtcctgaaaattcagttcgtcacagttaaccagttttagtggattaactgagttcatattgcg
gcatttgtctgtttctgttgccagcagttcaatccactgcatcagtaaagtatcaaacagaaaaacgaagacagcgaaaacgacgagccacc
aatacttgcgaatcattacctgagcctgtgcatgtttaaccaagtctggcaggaaatatacacgaaatgaataatctgaaaaccaggcttacac
atcctttactttgtaatgagatcggggtcaacttcgcttaaccggcttcgttttgacaggttgccggtctttctttttttcactttcgccagttcttccgcaga
gtaatgcgacgcgacatccagccgtcgaagccccattttgatgtactcgctgttgatctcaataccaatgaattttcgtccgctggcgatggctac
ggcaccggtagtaaagctaccagcaaacgggtcgagaacgatatcgcctgggttggaagaggcgagaataatgcgtttcagtaaggcttcc
ggtttttgcgtcgggtggttttcatattcatccatcaaataacgcacgcgcggaaaatcccaaacgttacccggtactttttgatgattgtaaggctgt
ggaggatttttgcgataatcgatcaacgcgcgctgcgatccggttttggcttctaccagaatagcatcaccgttgaatgtgtagttctttgcgtctttc
accatcatcaggatgggttcgtacatggagccgtagtgtttttttcgcctgcactccagaactgtcatatgaccagacgatgcgacttttgatggtaa
aaagcttgcggcactggagatcgataaagggcatgttttccgtactgttcatgatgtacatgctgccctgctttttcagaacgcggtggcactctgc
aatcacttcaaacagccagtcgataaacagatcttctttccaggcttcgatcagaccatcaaaatttttaccgatgttatatggtgggtcggcaaa
gatcagatcgacactttcggcggggatctttttaagttcggcaagcgcatcaccgtgaataatggtcttagcttcattaccaaaccgggtcggttc
acatcctgttctcatggtcactccctttgtgacacctataaaaaaggcgcttccccatgccgagtagcgcctttttaatcaagcatttagctaacctg
aattagttcatgccgtatttttttcaatttttttacgcagcgtaccacggttgatgcccatcatcagcgcagcacgggtctggttaccacgggtgtattgc
atcaccatgtccaacaggggctgttctacttcagccagtaccagctcatagaggtcattcacatcctgaccattcagttgagcaaaatagttcttc
agtgcctgtttaaccgagtcacgcaggggtttttgggttacctgatcctgagagttaacggtagaaacggtcagtacgtcagaatttacgcgttgtt
cgaacatagttctgtcagctctttatttctgtttacgcaaaattttcgaagtatgcctccaacgcctccagctgttcgctggcatcctcaatggcgttga
atgtgcgccgaaactggtcatttggagcgtgttcctggagataccaggaaacgtgtttacgtgcaattcggtacccttttgccggaccataaaagt
catgcagttcccgaacgtgcgcgcaaagcaagcgcttaacctctgccaaaggcagcgggggcagcaactccccagtgtccagataatgctg
gatttcccgaaagatccagggtcttccctgagctgcgcggcctatcatcagggcatccgcccctgtatagtcgagcacagctctggctttaagcg
ggtcagtaatgtcgccattcgcgataaccggaatggaaactttctgcttaactgcccgaatactgtcgtactcagcttctccattgaacaaacagg
cgcgtgtacggccatgaatggtcagagcctgaatgccacagtcttcagccagttgggcaatctcttcgcagttacggtgttccggtgcccagcc
ggtgcgaatcttcagggtaacaggaacgtccactgcattgacgacctcggtaaggatcgatttaacgacatccgggtactgcaagagggctg
aacctgcgagcttgcgattcactttttttagccgggcaacccatattgatatcaataatctgggcaccgctttccacgttaatacgtgctgcatctgcc
atttctttcggatcgctaccagcaatttgcacggtgcgaataccgggttcatcaatgtgcaccatccgtaaacgagatttgtcgctttcccaaacct
gtgggttagaagacatcatctcggatactgtcaatccggctcccatctcgtagcacaacgtccgaaaaggtctgtctgtaatgccagccatggg
cgctgcgatcaggcgatttctgagctgatattgtccgatgcgcatgagttaagaaatgaccatactgtgactgcaaggcggcgtatattacgcatt
ttttgcacgagatgaaaggccaaactttgaccaatcctctgagatggatcaaagaattgcatttaaaatgagcgtggtgcgataattactcataa
aaatcatcatattagaaaatagtgactaaaatttacactcaaagaaatttgagtaagttctcaatttttctttatgaatgaaaattttggcacgcaaat

ttgcgtaaataatcggcaaatttacgtgccttttgtgagcttgctcgcacttcgccccgcgtcaccctacggcgatgcgaaggttaattcttacgac
cggtaatacggcaccactcttctttttccacgaccgggtccagtgcgaagctatcggcataagcttcacaaacgctctctgcctggcttgccaga
ataccggaaaggcccagcaaaccgcctgaaaccggcaggacgctgattaacggtgccagttcacgtaatgggcctgcaaggatgttagcg
accaccacgtcggctttcatttcttctggctgatctttcggtaagtagagttccagacggtcagaaacgccattacgttcggcgttatcgcggctgg
cctgaatcgcctgcggatcgatatcaataccaatggcttttgctgcacccagtttcagcgccgcgatcgccagaatgccggaaccacagccaa
agtcgatgactgttttaccggttaaatcgaggctgtcgagccattgcaggcacagagaggtggttggatgggtacccgtaccaaacgccagcc
ctggatctaacatcacgttgacggcgttttcgtccggcacatcacgccagctagggcagatccacagtcgttcaccaaagcgcatcgggtgga
aattatccatccattcgcgctcccagtctttatcttctagttgttcgattttatgcgcgaagcctgcgccgagcagcggatggttttccagaatcgcca
ccacgtcgttcatatcggtttcagcgtcgaacagaccaatcacatcggtgtcgccccacaggcgcgtttcgcccggcagcggttcaaatactgg
cgtatcgtgggtatcctgaaaagtgatagaaacggcacccgcttccatcagcgcatcgctaagatcttccgcgttcgcgccggtggtgttcagttt
cagttggatccaaggcatggcaaaactctttatttatcagtagtcaaaacggtagcttgcgggacggatgtaccgaaacggtttccgaccagga
aagccagcaaacttagtagtaacgagggcacgatagggtggaagcccaggtactgaatattcagcgtcgcgagtacggcatacagcacgc
cgccaacgatcatcgcacttagcgcgcctttggcgttggcgcgttcccagtaaagacccagcaccagcggccacaggaaaacggcttccag
cccaccgaaggccaacaaattcagccagatgatcatttctggcggcttccaggcggcaagcagcagcaacgcgccgagaactaacgtaat
taccgccgacatccgcttcagacgcgtctcgttttgcatttgatccggacggatattcagatagagatctttaatgatcgtagcggaactttgcagc
agttgggcgttaattgtcgacatgatcgcagccatcggtgcagccaggaagatcccggcagcaaacggtggcagcacttttaccattaacgtt
gggatcaccaggtccggtacggtgagatcggggatcaccgcccgacctaacgctccggccaggtgcataccgaacatcagaattgcgacc
acaatcgtaccgatgatgatcccccgatgtacggctttgctgtctttataagagatacagcgcaccgcagtatgcggcaggccaatcacgcca
aaacacaccagtacccagaacgacgtcataaaggcaggcgacagaatatcgtcagcgccttgtggcgtaaccagttgcggatcgatggttt
gcaaggtctgtactgcgttacttaagccgccagcggcatgtactacgccaataagcagcacaacggtgccaatcagcatcacaagcccttgc
atggtgtcgttcagcacgctggcgcgaaagccaccaaaggcggtatataacgcaatgctgataccaaaaatcagcagcccggtttcataag
gaatacccgccgcggtttccagcaggcgcgcaccgccgataaactgcacggtcatcgcaccaacgaacgcaaccagcaaactcaaactc
gccagccacaccagaagacgactctggtagcgggcaaacagcatatcgttcagcgtcactgcattgtagcggcgcgcaagaatcgcaaac
ttcttgccgagaataccgagtgaaagccagactgcaggaagctgaatcatcgccagcaatacccagcccagcccgtatttataagcagctcc
tggcccgccgataaacgaactggcactgatataggtcgcggtgagcgtcatcgccagcacaataccgcccatagagcggctgccgaggaa
atactcattaaggaaggtgccggtgctccgtttacgcatcgcataaaccgagataccgaacaccaccaccagataggcgaccagcggtaga
attacttcaagctgcatcgtcatcctccagtgggatatcgcgatagataaatttcaccatcgcccagcacagtccaataaacagcagcggcgtc
aggatgcaggccatctcaaaccagcgcggaaagccggtaaaaccgggggcaacgccagataagtaagcggctactaaccaaactgcca
gatacaaaagggtcagccccagcgcccagcgcgcctctttatgggcctgaacaaaacgagtgtccatttttttgtccctgatgggtgaagaaag
cggggattgtaccttatggggggttgtcgatccccagtaataaaaaaggccggaaaatccggccttttgacgctttagcagtcttatttttcctgaag
accgagtttttttctccagatagtggatgttagtgccaccatgctggaagttctcgtcattcatgatgcggatctgcagatcaacgttggttttgatacc
gtcgatgatcagctcctgcagcgcattcttcatgcgggcaatcgccacgtcacggttttcaccgtagcaaatcagcttaccgatcattgagtcata
gtacggcggtacggtgtagcccgcgtagatatgagactcccaacgtacgccaaaaccgccaggtgcgtggaaacgggtgattttgcccgga
cttggcaggaaggtgttcggatcttcggcgttgatacgacattccaccgcatggccgcgaacgtgaacttcttcttgcttgatcgacagcggttga
ccggcagcgatacgcagctgttctttgatcaggtcaacgccggtgatcatttctgtaaccgggtgttctacctgaatacgggtgttcatttcgatga
aatagaactcgccgttttcgaacaggaactcgaaagtacctgcaccgcgatagccgatatcaacacacgctttagcgcaacgttcgccgatgt
agcgacgcagttccggggtaatgcccggtgctggcgcttcttcgaccactttctggtggcggcgttgcatggagcagtcacgttccgccagata
gatagcgttgccctgaccgtcagccagtacctgaatctcgacgtggcgaggattttccaggtatttctccatgtaaaccatatcgttgctgaaagc
agctttcgcttccgcacgggtcatggagatggattgtgccagttcagcgtcgccgcgcactacgcgcataccgcgaccgccgccgccgccgg
aggctttgataatcaccggataaccaatgcgtttagcaatggcacggtttttatccatatcgtcgcccagcgggccgtcagaacccggtacgca
agggacgcccgctttttttcatcgccgcgattgcggatactttgtcgcccatcaggcgaatggtttctgctttcgggccaatgaagataaagccgga
gcgttcaacctgctcggcaaagttggcgttctcggagaggaagccgtaacccggatggattgctactgcgccggtgatttcagcggcgctgatg
attgccgggatgttcagataactttttactgacggagcagggccaatacagaccgtttcatctgccagtaatacgtgttttagatcgcgatccgcg
ctggagtgcacagcgacagtcttgatgcccagttctttacaggcacgaagaatacgcaatgcaatctcgccgcggttggcaataacaattttatc
cagcatgttcgcctcgttactcgatgacgaccagcggctcgtcaaattctaccggttgtccactttcgaccagaattgctttcacggtaccggatttg
tccgcttcgatctggttcatcattttcatggcttcaacgatgcacagggtatcgcccacgttgactttctgacccacttcgatgaacgcttttgcgtccg
ggcttggggtgcggtagaaagtaccaaccatcggggaacgtacgatgtgaccactgatttccgctgctgctggcgcttccatggaaggaacg
gtcgccggagcggctgcgttagattgagctggctgctgcatcattggtgcagcgtaagcttgttgcatcacagggaaacttgcggcaggagctg
cacggctaatgcgtactgactcttcgccttcagaaatttccagttcggagatgcctgattcttcaaccagctcgatcagtttttttaatcttacgaatatc
catgagtgggttccgtactctttgtttagtgtgattgtgacaggcgtttcaccgccgtctgtaaagcgtatgccccgacagctgtatgcatagcgata

aattccagcaggccgggacgccggtctattttgccccagagtgtcaatgttagacttgacggacattgtgcagcgatctgctgctatcctccggc
aaaaaacaaaatataccttcgttgcgccaatgtcaccttttccgcagcgcaaaaactgcgtcagggaggacgaggtcgcacattataacgatt
tcgtagcaattggcagctaaatactggtcttatcagggaagataatcaacagctaacatgtaaataaccttcaacaccgtgtaatttgcaacaag
ccgcacaattcacgaaattagcgccatcatcgacggaacttcttataacgtaaggtgaaaagctgaaaagccagccctgcgaagatgaggg
gctgcaatgagataatcttcacagaccacaaataatgtagggatgccaggatcgcgacaagatagacgacattacgcagcggttgctgggg
attacccaattttcactgcatcgtctggatagatgtgaatgctaaagcacgcaaaattaaccgattgacaatgctcagcattaaataggttcgagt
gattaatttgtagtcaaacaaaatcagattattcaccccatggtaccagaaataagtaaggggctaagcgtagcgtcgcccaggcaaagcaa
cataaacctaacaggcgacggaggcgtatcaataatagctgtttagtggagtatcaatgcgcatcaatttgtggtcagtcgaaatcatgactttcc
agtttggatgatgttgtataacttcctgaccaaggtgttcgacgaaagctgtccggctaaccgcttttcccgttaaccgattgaataatgatttcaaa
atgagttccctctcttattattccctgctaaatggttagttaaccttcaccagcgtgcgaccctggatctggttattaatgatggcctcggcaaagttcg
gtgcctctgacagagatatctcttttgccgcctgggtatagaatgattccggtaaatcggcgaccagtcgctgccaggcttgtgcgcggcgttctg
gtggcgtcattactgaatccaccccttgcaaacggacattacgcagaataaatggcatgaccgtggttggcagagtaaaaccacccgccaga
ccacaggccgccacgcagccgccgtaattcatttgcgccagcacttttgccagcactttgtcgccaacggtgtcaattgccccagcccagactt
gtttttccagaggacgggattcggcaaactcatcacgagggagaacacggctagcacctaaacttttcagatattcatgggtactttcgcgacc
ggaaacggcaacgacctgataacccaacttatgcagcagcgccacggcggtactgccgacgccaccactggcacccgtcacgacaatctc
cccgtcctgcgggcgaacaccggcatcttccagcgccatcacacacagcatggcggtaaaaccggcagtaccgataatcattgctttacgcg
cgtccagcccttgcggcatggcaaccagccagtcacctttcactcgcgcctgctccgccagcccaccccagtggttttcaccaacgccccagc
cagtgagtaacacctcctgaccggcatgaaaacgcggatcttcgctggtgcgtacagttccggcaaaatcgatcccaggaatcatcggaaaa
ttacggatgatttttcccttaccggtaatcgccagcgcatctttatagttcaggctcgaccagtgaacatcgaccgtgacatcgccctccggcagg
cgactttcgtccagagtctgtactgatgcgagagttttgccgtcctgctgttctaaaagtaacgcctgcataagtggtcctcatgtgcatgatggatt
ggaaaataatttctgaagactatactcgctaatggaaaagcaaagccgatgaagcgcaataaattgcgagataaatctgatttgctagtatgcc
cgcttcctcactatcggagttaacacaaggatgagattaacgacgaaattttcggcctttgttacgctgctcaccgggttaacaatttttgtgactttg
ctgggctgttcgctaagtttctacaacgccattcagtataagtttagtcatcgcgttcaggcggtggcgacggcgattgatacccaccttgtgtcga
atgacttcagcgtattaaggccacaaattaccgaattaatgatgtcggcagatatcgttcgtgtagacctgctccatggtgataaacaggtttatac
cctggccagaaatggtagttatcgtccagttggctccagcgatctgtttcgcgaactgagcgttccgttgataaagcatccggggatgtcgttgcg
tctggtttatcaggatccgatgggcaactatttccattcgttgatgaccaccgcgccgctcacgggggcgattggctttatcattgttatgctcttcctg
gcggtacgctggttacaacggcaacttgccgggcaagaattgctggaaacccggctactcgtatcttaaacggtgagcgtggctctaatgtgt
tgggaaccatctatgaatggccgcccagaaccagcagtgcgctggatacgctgcttcgtgaaattcagaacgcacgcgaacaacacagcc
gtcttgatacgctgatccgctcttatgccgcccaggacgtgaaaaccggcctcaataaccgactctttttcgataatcagttagcaacgttactgg
aagatcaggagaaagtaggtacccacgggatcgtgatgatgattcgtctgccggatttcaatatgttgagcgatacctgggggcacagccag
gttgaagaacagttcttcactctgacgaatctgctgtcgacatttatgatgcgctaccctggcgcactgctggcgcgttaccaccgcagtgattttg
ctgcgctgttaccgcaccggacgttaaaagaggcagagagcatcgccggtcagttaatcaaagccgttgataccttgccgaacaataaaatg
ctcgatcgcgacgatatgatccacattggtatctgcgcctggcgtagtggtcaggataccgagcaggtaatggaacatgcagagtctgccacg
cgtaatgcgggattgcagggcggcaatagctgggctatttacgatgactcgttgcctgaaaaaggacgcggtaatgttcgctggcgtacgcttat
cgagcaaatgctcagtcgcggcggcccgcgcctttatcaaaaaccggcggttactcgcgaaggtcaggttcatcatcgcgaactcatgtgccg
catcttcgatggtaatgaagaggttagctcggcggagtatatgccgatggtcttgcagtttggcttatcggaagagtatgaccgtctgcaaatcag
ccgtcttattccactattgcgttactggccagaggaaaatctggcgattcaggttaccgttgagtcgctgattcgcccgcgttttcagcgttggctgc
gcgatacgttaatgcaatgtgaaaaatcacaacgaaaacgcataattattgaacttgcagaggccgatgtaggtcaacatatcagtcgtttaca
acctgttattcgtttagtgaatgctttaggggtacgggtagccgtcaaccaggctggtttgacgctggtaagtaccagttggatcaaagaacttaat
gttgagttactcaagctccatccggggctggtcagaaacattgagaagcgaacggagaaccagctgctggttcaaagcctggtggaagcctg
ctccgggaccagcacccaggtttacgccaccggcgtgcgttcgcgaagcgagtggcagaccctgattcagcgcggtgttacaggcgggcaa
ggggatttttcgcgtcctcacagccacttgatactaacgtgaaaaaatattcacaaagatactcggtttaacctgccgtttaatccgttttcacgta
gaataatgcgcgctgcgtctcatgggagtgtgcttgtctgctcgccagattgttgcagcacatatgcagatgaatgaccttacgcggttgcaaac
aggcgaggaatgctgctgatgcattaagcctttctggactcaggcagagatttgtaacaaaggaaacgaactgcactaattttcaccgtagcag
atgatttttgcgccttgtcgctgctgcgtgtggttggtaaagtaagcggattttcttttccgccccagctttcaggattatcccttagtatgttgaaaaaat
ttcgtggcatgttttccaatgacttgtccattgacctgggtactgcgaataccctcatttatgtaaaaggacaaggcatcgtattgaatgagccttcc
gtggtggccattcgtcaggatcgtgccggttcaccgaaaagcgtagctgcagtaggtcatgacgcgaagcagatgctgggccgtacgccgg
gcaatattgctgccattcgcccaatgaaagacggcgttatcgccgacttcttcgtgactgaaaaaatgctccagcacttcatcaaacaagtgca
cagcaacagctttatgcgtccaagcccgcgcgttctggtttgtgtgccggttggcgcgacccaggttgaacgccgcgcaattcgtgaatccgcg
cagggcgctggtgcccgtgaagtcttcctgattgaagaaccgatggctgccgcaattggtgctggcctgccggtttctgaagcgaccggttctat

ggtggttgatatcggtggtggtaccactgaagttgctgttatctccttgaacggtgtggtttactcctcttctgtgcgcattggtggtgaccgtttcgacg
aagctatcatcaactatgtgcgtcgtaattacggttctctgatcggtgaagccaccgcagaacgtatcaagcacgaaatcggttcggcttatccg
ggcgatgaagtccgtgaaatcgaagttcgtggccgtaacctggcagaaggtgttccacgcggttttaccctgaactccaatgaaatcctcgaa
gcactgcaggaaccgctgaccggtattgtgagcgcggtaatggttgcactggaacagtgcccgccggaactggcttccgacatctccgagcg
cggcatggtgctcaccggtggtggcgcactgctgcgtaaccttgaccgtttgttaatggaagaaaccggcattccagtcgttgttgctgaagacc
cgctgacctgtgtggcgcgcggtggcggcaaagcgctggaaatgatcgacatgcacggcggcgacctgttcagcgaagagtaatcggatg
caggcaggggaagtgtctgtttaccctgcctggtctgatacgagaatacgcataacttatgaagccaatttttagccgtggcccgtcgctacaga
ttcgccttattctggcggtgctggtggcgctcggcattattattgccgacagccgcctggggacgttcagtcaaatccgtacttatatggataccgc
cgtcagtcctttctactttgtttccaatgctcctcgtgaattgctggatggcgtatcgcagacgctggcctcgcgtgaccaattagaacttgaaaacc
gggcgttacgtcaggaactgttgctgaaaaacagtgaactgctgatgcttggacaatacaaacaggagaacgcgcgtctgcgcgagctgctg
ggttccccgctgcgtcaggatgagcagaaaatggtgactcaggttatctccacggttaacgatccttatagcgatcaagttgttatcgataaaggt
agcgttaatggcgtttatgaaggccagccggtcatcagcgacaaaggtgttgttggtcaggtggtggccgtcgctaaactgaccagtcgcgtgc
tgctgatttgtgatgcgacccacgcgctgccaatccaggtgctgcgcaacgatatccgcgtaattgcagccggtaacggttgtacggatgatttg
cagcttgagcatctgccggcgaatacggatattcgtgttggtgatgtgctggtgacttccggtctgggcggtcgtttcccggaaggctatccggtc
gcggttgtctcttccgtaaaactcgatacccagcgcgcttatactgtgattcaggcgcgtccgactgcagggctgcaacgtttgcgttatctgctgc
tgctgtgggggcagatcgtaacggcgctaacccgatgacgccggaagaggtgcatcgtgttgctaatgaacgtctgatgcagatgatgccg
caggtattgccttcgccagacgcgatggggccaaagttacctgaaccggcaacggggatcgctcagccgactccgcagcaaccggcgaca
ggaaatgcagctactgcgcctgctgcgccgacacagcctgctgctaatcgctctccacaaagggctacgccgccgcaaagtggtgctcaac
cgcctgcgcgtgcgccgggagggcaatagtggcgagctatcgtagccagggacgctgggtaatctggctctctttcctcattgcgctgttgctgc
aaatcatgccctggccggataacctgattgttttccggccaaactgggtgttactcatcttgttgtattggatcctggccttgcctcatcgcgtaaatgt
gggcacaggttttgtgatgggtgccatactggatctgatcagcggctcgacgcttggcgtacgcgtattggcgatgagcatcattgcttacctggt
ggcgctgaaataccagcttttccgcaacctcgcattatggcagcaggcgctggtcgtcatgttgctttcgctggtggtggatattattgttttctgggc
agagtttttagtgattaacgtctctttcagaccggaagtgttctggagtagtgtagtcaatggggtgctctggccgtggattttcttgctgatgcgcaa
agtccgtcagcagtttgcagtgcaataaaggtttctatgacttctctgtatttagcttccggttctccgcgtcgtcaggagttacttgcgcaacttggcg
tgacctttgaacgtattgttacgggcattgaggagcagcgtcagccgcaggagagcgcgcagcagtatgttgtgcgtctggcgcgcgagaaa
gcacgggcaggtgtcgcgcaaacggcgaaggatctcccggtgctgggtgcggatactatcgttatcctgaacggagaagtgctggagaaac
cgcgcgacgcagagcatgcggcgcagatgttgcgcaaattatcgggtcagacccatcaggtgatgacagcagtggcgttggccgacagcc
agcacattctcgattgcctggtggtcaccgatgtgactttcagaacgttaacagacgaagacatcgcgggctatgtcgccagcgatgaaccgtt
agataaagcaggtgcatacggtattcaggggctgggtggctgttttgtcaggaagataaatggcagctatcacgccgtagtcggcttaccgctg
gttgaaacgtatgaattattaagtaattttaacgcactgcgtgagaaaagggataaacatgacggctgaattgttagtaaacgtaacgccttcgg
aaacgcgagtggcgtatattgatggcggtattctgcaggaaattcatattgaacgtgaggcgcgacgcggaatagtaggcaatatctacaagg
gtcgtgtaagtcgtgtacttccgggtatgcaggcggcttttgtagatattgggctggataaagccgcgtttcttcatgcatccgacatcatgccgca
caccgaatgtgtggcgggtgaagaacaaaagcaattcacggtgcgcgacatctcggaactggttcgtcaggggcaagatctgatggtgcag
gtggtgaaagatccgcttggcactaaaggtgcgcgcctgaccaccgatatcacgctcccttctcgctatctggtgtttatgccaggggcttctcac
gttggggtttcccaacgtattgaaagcgaatcagaacgtgaacgcctgaaaaaagtggtcgcagagtattgcgacgagcagggcgggtttat
catccgtaccgcagcggaaggggttggcgaggctgaactggcctccgatgccgcttatctgaaacgcgtctggaccaaagttatggagcgta
aaaaacgcccgcagacccgttatcagctgtacggcgaactggcgctggcgcagcgtgttctgcgtgatttcgccgatgccgaactggaccgc
attcgcgttgactcacgcctgacttacgaagcgttacttgagttcacctcggagtacattcccgagatgacaagcaagctggagcattacacag
gacgccagccgattttcgatctctttgatgtcgaaaacgaaatccagcgagcgctggaacgcaaagtagaactgaaatccggtggttatctcat
tatcgaccagaccgaagcgatgaccaccgtggacatcaataccggagcgtttgtcggtcatcgcaatctggacgacaccattttcaataccaa
tattgaagcgacgcaggctatcgctcgccagttacggttgcgtaatctgggcgggattatcattattgatttcatcgatatgaataatgaagatcac
cgccgccgagtgctgcactcgctggagcaggcgttgagcaaagaccgggtgaaaaccagcgttaatggtttttcggcgctggggctggtgga
gatgacgcgtaaacgcacccgcgaaagcattgagcacgtactgtgtaacgaatgcccaacctgccacggtcgcggaacggtgaaaaccgt
ggaaacggtatgctatgaaatcatgcgcgagattgttcgtgtccaccatgcttacgactccgaccgtttcctggtctatgcttctccggcagtagct
gaagccttgaaaggcgaagagtcacactcgctggcggaagtggaaattttcgttggcaaacaggttaaagtacaaattgaaccgctctataa
ccaggagcagtttgacgtcgtaatgatgtaaacagatgctgggccgccatccggcaaagggtttttgagtcacatttttagcagacaaggagtg
acgggtgaggcgattgccggggattttactgcttactggagccgcgctcgttgtgatcgctgccctgctggttagcggcctgcgtattgctttaccg
catcttgacgcctggcgtccggaaatcctcaacaaaatagaatccgcgactggcatgccggtagaagccagtcagctctcagccagctggca
gaattttggcccgacgcttgaagcacacgacatccgtgcagaactaaaagatggcggcgaattttcggttaaacgcgttactctggcgctggat
gtctggcagagcctgttacatatgcgctggcagtttcgcgacctcactttctggcagctgcgctttcgcaccaacactcctatcaccagcggtggt

agtgatgacagtctggaagccagtcacatcagcgatctgtttcttcgtcaatttgaccatttcgatcttcgcgacagtgaagtcagtttcctgacgcc
atccggtcagcgcgccgagctggcgatcccacaactcacctggctgaacgatccacgtcgacaccgtgcggaaggcctggtaagcctctcc
agccttaccggacagcacggcgtgatgcaggtgcgcatggatttgcgcgatgatgaggggttgttaagcaatggtcgcgtctggctccaggcg
gatgacatcgacctgaagccgtggctcggtaaatggatgcaggacaatattgcgctggaaacggcacagttctcccttgaaggctggatgac
gatcgacaaaggcgatgtaaccggcggtgacgtctggctgaaacagggcggtgccagctggttgggcgagaagcaaacgcatacgctgtc
ggtggataatctgaccgcgcatattacgcgtgaaaatccgggctggcagttctctattcccgatacacggatcacgatggacggcaaaccctg
gccgagcggagcattgacgctggcctggataccggaacaggacgttggcggcaaagacaataaacgcagtgacgaactccggattcgcg
ccagtaatctggagctggcaggcctggagggcatacgcccgctggccgcgaaactttcacctgcactgggtgatgtttggcgctccacacaac
cgagcggcaagattaacactctggcgctggatatcccgcttcaggcggcagacaagacccgttttcaggcatcgtggagcgatctggcctgg
aagcaatggaaattattaccgggtgcggaacacttctccgggacgctttccggcagcgttgaaaatggtttgcttaccgcgtcgatgaagcagg
caaagatgccttacgaaacggtattccgtgcgccactagaaatcgccgacggccaggcaactataagctggctgaacaataacaaaggtttc
cagctggatgggcgtaatattgacgttaaagccaaagccgtccatgcgcgcggcggttttcgttacctgcaacctgctaacgatgaaccctggc
tgggtattctggctggcatcagtaccgatgatggttcacaagcctggcgctatttcccggaaaacttgatgggtaaagacctggttgattacttaag
tggcgcgattcagggcggtgaagcggataacgcgacgctggtttatggtggcaatccgcaactcttcccctataaacacaacgaaggtcagtt
tgaagtgctggtgccgctgcgcaacgcgaagtttgccttccagccggactggcctgcattaactaaccttgatattgaactggactttattaacga
cggtttatggatgaaaaccgatggcgttaatctgggcggcgtgcgcgcgagtaatcttaccgcagtgatccctgactactcaaaagaaaaact
gctgattgacgctgacattaaaggtccgggtaaagccgttggcccttactttgatgagacaccgctgaaagattctctgggtgcgaccctgcaa
gaactccagctcgacggcgatgtgaatgctcgcttacatcttgatatcccgctgaacggcgaactggtaaccgcgaaaggtgaagtgacgctg
cgtaataacagtctgtttatcaaaccactcgacagcaccctgaaaaatttgagcggtaaattcagctttatcaatagcgatctgcaaagtgaacc
actgacagcaagctggtttaatcagccgttgaacgtggatttttccaccaaagaaggggcaaaagcctaccaggtagcggtaaacctcaacg
gtaactggcaaccggcgaaaaccggcgttctgcctgaagcggtgaacgaagcattgagtggcagcgtggcgtgggatggtaaagtgggca
ttgatctgccttatcatgctggtgcgacctataacatagagctgaacggcgatctaaagaatgtgagcagtcacttaccttcaccgttagccaaac
ctgcgggtgaaccactagcggtaaacgttaaggttgatggcaatctcaacagctttgaattaaccggacaggctggtgcggataatcatttcaat
agccgctggttgctcggtcaaaagctgacgctcgatcgtgctatttgggcggcagacagtaaaacgctcccgccgttgccggaacaaagtgg
cgttgaactcaatatgccgccgatgaatggtgccgagtggctggccctgttccagaaaggcgctgcggagagtgtcggtggtgcagcgagttt
cccacaacacataacgttacgtacgcctatgttgtcactgggaaatcagcaatggaataacctgagtattgtttcgcaaccgacggcaaatggc
accctggttgaagcgcaagggcgtgaaatcaacgccacgctagcgatgcgtaataacgcgccgtggctggcgaatatcaaatatctttattac
aacccgagcgtggcgaaaactcgtggtgattcaacgccgtcatcacctttcccgacaacggagcgcattaacttccgtggctggccggacgc
ccaaatacgatgcacagagtgctggttctgggggcaaaaattcggtcgcattgacagtgatatcaccatttctggcgatacgttaacgctgacc
aatggactgattgatactggtttctcgcggcttactgccgatggtgaatgggttaataatccggggaatgaacgtacctcgctgaaaggaaaact
gcgcgggcagaaaattgatgccgccgcagaatttttggtgtcacgacgcccatacgccagtcgtcatttaatgtggattacgatttacactggc
gcaaagcaccctggcagccagatgaagcgacgttgaatggcatcattcatactcaactgggtaaaggcgaaattaccgaaatcaataccgg
acatgccgggcaattgctgcgcttattgagcgtagatgccctgatgcgtaagctgcgttttgatttcagagacacttttggcgaagggttctattttga
ctccattcgcagcaccgcgtggattaaagacggcgttatgcacaccgacgacacgctggtggatggcctggaggcggatatcgccatgaaa
gggtcggtaaatctggtacgtcgcgacctgaatatggaagcggttgtcgcaccagagatttctgcgacggtgggcgtggctgcggcttttgcggt
taaccccattgttggcgcggcagtgtttgccgccagtaaagtgctggggccgctgtggagcaaagtctccattttgcgctatcacatttcgggtcc
gctggacgatccgcaaatcaacgaagtgttgcgccaaccgcgtaaagaaaaagcgcaatgatttgacgagggcgcgtaattgccccaatct
cataggataatcgttgccaaaggccaacgagccagaacataaccgtaggtcggatagggcgttcacgccgcatccggcagccgttgcctga
tgcgacgctgtgcgtcttatcaggcctacaaacggaacataaccgtaggtcggataaggcgtttacgccgcatccggcagccgttgcctgatg
cgacgctgtgcgtcttatcaggcctacaaacggaacataaccgtaggtcggataaggcgtttacgccgcatccgacagccgttgcctgatgcg
acgctgtgcgtcttatcaggcctacaaaccggaacataaccgtaggctggatagggcgttcacgccgcatccggcagccgtaaaaaatcctc
tactgcagtaactaacgagtagcaaaaacgatgagtcttaacctggtaagtgaacaattgctagcggcgaacggcctgaaacatcaggactt
gttcgcgatcctcggtcaactggccgaacgtcgccttgattatggcgatctctattttcagtcgagctatcacgaatcctgggttttagaagaccgc
attattaaagatggttcttacaacatcgatcagggcgttggtgtgcgtgcaatcagcggtgaaaaaaccggatttgcttacgctgaccaaatcag
cctgctggcgctggaacagagtgcgcaagcggcgcgcaccatcgtccgtgatagtggtgatggtaaagtacagacgctgggcgcggtaga
gcatagcccgttgtatacctcggtagatccgctgcaaagcatgagccgtgaagagaagctggatatcctgcgtcgcgtcgataaggttgcccg
cgaagcggacaagcgcgtacaggaagtgactgccagcctcagtggtgtctatgaattaattttggttgcggccaccgacggcacgctagcgg
cggatgtccgtccgctggtgcgtctttccgtgagcgttctcgtcgaagaagatggcaaacgcgaacgcggtgccagtggcggcggcggtcgttt
tggttatgaattcttccttgccgatctcgacggcgaagtccgtgcggatgcatgggcaaaagaagcagtgcgtatggcgctggtcaatctttctgc
cgttgctgcaccagcgggcaccatgccggtagtacttggcgcaggttggccgggcgtgctgttgcatgaagcggttggtcacggtctggaagg

cgacttcaaccgccgtggcacttcagtatttagtggacaggtcggggagctggtggcttcagaactgtgtaccgtggttgatgatggcacgatgg
tcgatcgccgaggttcggtggcgattgatgacgaaggtacgccaggccagtacaacgtgctgattgagaacggcattctgaaaggctacatg
caggataaactcaacgcgcgtttgatggggatgacgccgactggcaacggtcgccgtgaatcctacgcccatctgcccatgccgcgtatgac
caacacctatatgctgccgggtaaatcgaccccgcaggaaattattgaatccgttgagtacggtatctatgcaccgaactttggtggcggtcag
gtggatatcacctccggcaaattcgttttctccacttcagaagcatatctgattgaaaacggtaaagtaacgaagccggtgaaaggcgcaacgt
tgattggttccggtatcgaaaccatgcagcagatttcgatggttggcaacgacctgaaactggataacggcgtgggtgtctgcggtaaagaag
ggcaaagtttgccggttggcgtgggccagccaacgttgaaagtcgataacctgactgttggcggtactgcgtaataatttcactattttcagaagg
ataattaatctttctacgtgcacgaacggtcccctcgcccctttggggtgagggttagggtgaggggaacccgttggcacaggtttgtacaacgt
aacagtacaatatgaattacttctctttcccgcgcccgtgcatctcctgaaacaatttaccgacctcaacaaaataatccgtcagcgagttgatca
cgacctgtaccttcagcggcagcttatctttttcggtatataacgcataaaccgggcgtggatctgactggtaacgcggcagcaggatctccagc
tccccacgattgatctcgttgatcacccacatcagcggcacgtaggcgatcccggcacccgccgtcagccagcgcaccagcgtcatcggatc
attagtcacaaatcttccttgtgggatcaggcgagtcgagatcccttccggtgcgatcagttcaaattcattgtcgggccgcacgctgtattcaagc
catgaatgactactcaaatcggcgggtttttccggtatgccgtattgtgtgagatagcttttcgcggcgcacaccaccattggcatcgcgcccaga
cggcgggaaaacaggctggaatcctgcaacgcgccgacgcggatcaccacatccagaccgtcggcaatcaggtcgggggctggaattcc
ggtaaccagattgacgctcaaacctgggtattctttcagcattttggctgtcagcccggcgagaacattttgtgccatagttgaagaacagccaat
gcgtagcgtcccgatgggggtgttattgaaggcatacagttgctcatgaacatcctgcacttcatgaagcatacgacggcagccctggtagtaa
attctaccggcttcggtcaggccaatgctgcgtgtgctacggtttaacagctttacctgcaactcatcttccagttttgataccgtctgactgatggac
gaaacgctcatctgtagctgtctggcggcggcggtaaaagagccaaattcaactactttggcaaacaccgacatgcgttttagtcgttccattatt
cactctgacttaaaagtgatttagatcacataatatagataacagcataacagttacgctaatatattaaatatcaatctacagcaatgttgctctc
gcccggctttccatgcccttctctgcggcgacagatgctgaaaataataacgcctgctctctctttacaaccaaggtcaacatgagtctgtttcccg
ttatcgtggtgtttgggctgtccttcccaccgatattttttgaattgcttttatcactggcgattttctggctggtgcgccgggtacttgtgccaacaggtat
ctacgactttgtctggcatccggcgttgttcaacaccgcgctctattgctgcttgttttatttgatatcgcgactgttcgtttgaggttgaagtgaaaaca
ctaataagaaaattctcccgtacggccatcacggtcgtattagtcattctggccttcatcgcaatttttaatgcctgggtctattacaccgaatccccc
tggacgcgtgacgcgcgctttagcgctgacgtcgttgcgatcgcgccggacgtttctggactcattacccaggtgaatgttcatgataaccagct
ggtgaaaaaaggacagatactgttcaccatcgaccagccgcgctatcaaaaggcgcttgaggaagcgcaagccgatgttgcttattatcagg
tactggcacaggagaaacgccaggaggccggacgtcgtaaccgtctcggtgtgcaggcgatgtctcgcgaagagatcgaccaggccaac
aacgtactacaaacggttctgcatcagttagcgaaagcgcaggcgacccgcgatctggcaaaactggatcttgaacgcacggtgatccgcg
cgccagcagatggctgggtgaccaacctcaacgtctataccggtgagtttattactcgaggatcaacggcggttgcgctggtgaaacagaact
ccttctatgtactggcctatatggaagaaactaagctggaaggggtgcgtccggggtatcgtgcagagatcacgccgcttggcagtaacaaag
tgctgaaagggactgttgatagtgttgccgcaggggtcaccaacgccagcagcacgcgtgacgacaaagggatggcgactatagactctaa
ccttgaatgggtgcgtcttgcgcaacgtgttccggttcgtattcgtctcgacaaccagcaagagaacatctggcctgcgggcaccactgctaca
gtggtggtcactggcaaacaagatcgcgacgaaagccaggattcgttcttccgtaaaatggcccatcgcctgcgtgagtttggttaatcacgat
gggtattttctccattgctaaccaacatattcgctttgcggtaaaactggcgaccgccattgtactggcgctgtttgttggctttcacttccagctggaa
acgccacgctgggcggtactgacagcggcgattgttgccgccggtacggcctttgctgcgggaggtgaaccgtattctggcgctattcgctatc
gtggctttttgcgcatcatcggcacatttattggctgtattgccggactggtgatcatcattgcgatgatccgcgcaccattattgatgattctggtgtg
ctgtatctgggccggtttttgtacctggatatcctcgctggtacgaatagaaaactcgtatgcgtgggggctggccggttataccgcgctgatcatt
gtgatcaccattcagccggaaccattgcttacgccgcagtttgccgtcgaacgttgtagcgagatcgttatcggtattgtgtgtgcgattatggcgg
atttgctcttttctccgcgatcgatcaaacaagaagtggatcgagagctggaaagtttgctggtcgcgcaatatcaattaatgcaactctgtatcaa
gcatggcgatggtgaagttgtcgataaagcctggggcgacctggtgcgacgcaccacggcgctacaaggcatgcgcagcaacctgaatat
ggaatcttcccgctgggcgcgggccaatcgacgtttaaaagcgatcaatacgctatcgctgacgctgattacccaatcctgcgaaacttatctta
ttcagaatacgcgcccggaattgatcactgatactttccgcgaatttttgacacgccggtagaaaccgcgcaggacgtccacaagcagctca
aacgcctgcggagagttatcgcctggaccggggaacgggaaacgcctgtcaccatttatagctgggtcgcggcggcaacgcgttatcagctt
ctcaagcgcggcgttatcagtaacacaaaaatcaacgccaccgaagaagagatcctgcaaggcgaaccggaagtaaaagtagagtcag
ccgaacgtcatcatgcaatggttaacttctggcgaaccacactttcctgcattctgggcacgcttttctggctgtggacgggctggacttccggca
gtggtgcaatggtgatgattgcggtagtgacgtcactggcaatgcgtttgccgaatccacgcatggtggcgatcgactttatctacgggacgctg
gccgcgctgccgttagggctgctctactttttggtgattatccctaatacccaacagagcatgttgctgctgtgcattagcctggcagtgctgggatt
cttcctcggtatagaagtacagaaacggcgactgggctcgatgggggcactggccagcaccataaatattatcgtgctggataacccgatga
ctttccatttcagtcagtttctcgacagcgcattagggcaaatcgtcggctgtgtgctcgcgttcaccgttattttgctggtgcgggataaatcgcgcg
acaggaccggacgtgtactgcttaatcagtttgtttctgccgctgtttccgcgatgactaccaatgtggcacgtcgtaaagagaaccacctcccg
gcactttatcagcagctgtttttgctgatgaataagttcccaggggatttgccgaaatttcgcctggcgctgacgatgattatcgcgcaccagcgcc

tgcgtgatgcaccgatcccggttaacgaggatttatcggcgtttcaccgacaaatgcgccgcacagcagaccatgtgatatctgcccgtagcg
atgataaacgtcgtcggtactttggccagttgctggaagaactggaaatctaccaggaaaagctacgcatctggcaagcgccaccgcaggtg
acggaaccggtaaatcggctggcggggatgctccataagtatcaacatgcgttgaccgatagttaagtcaaaaccgacgccaaaagcgtcg
gtttttcatggctatacttagcgatgcacggcagaactcgccgcgaaacgtgacggtggcaacagatgaatatttatacctttgattttgatgaga
ttgagagtcaggaggatttttatcgtgactttagccaaacctttggtctggcgaaagataaggtacgcgatctcgactcactatgggatgtgttaat
gaacgatgtcctgccgctaccacttgagattgaatttgttcatctgggagagaaaacgcgtcgccgttttggcgcgttaatattgctgtttgatgagg
cagaggaagagctggaagggcatttgcgttttaatgttcgtcattagcgcaaaaaaaagcccccgaaccgggggcaatatcgtcggacaag
acgatgagggtttatttgtacagttcagccgtagcgtgccaggtgtcaccgctacgagcttcagtaatctggtaggccgttgcgcctttctcttccgc
tttttgttcagcatttcacgcatatccattggcgaagacgccacaccacttacggatacggtcccgattgcttcacgattttgtgcttgtgcagcatc
aatggagtcggcagcgaatgcaccgaaagagagaacagaaagtacgcttaatgcagcaacagtggttttgattttcatgatttttacctcgaca
taatcttttagctgggcctttgtttcgtgacccatatcacaaaatcaagtatacactaattattgagctaattaataccgcgctaactgtttcagttgata
ctaagtgttaaaaatgtgaattttaataacaaaaaagaattaaaaatacggttatttatcgttaattctagtttttcaattagataatgaattttgcata
atgtgcttttttaccttatgtattcattgtgtgaatgacatgtcgcagtaaaacgcactattcgttaaattaatcgcaggggaaagcagggggttgag
agggataagcaacattttccccgccgtcagaaacgacggggcagagattaaagctcctggtcgaacagctctaaaatcgcttcgtacaggtct
ttgactgtgaaaccgttagcaggggtggtaaagatggtgtcatcgccagcgatggtgcccagaataccttctgctttgcccagtgagtccagca
ggcgagcaattaactgcgccgcgccagggctggtatgaatcacgacaactgcatcgttgtagtcgatatccagcaccagattcttcaatggact
ggaggtggttggtacacccagttcagctggcaggcagtaaaccatttccattttggcattgcgtgtacgtacagcaccaaacttggtcaacatcc
gcgagactttagactgattaatattgtcaaagccttgctcctgcaacgcggcgacgatttcgccctgggagctaaatttctcttctttaagtaatgctt
taaatgctttaactagttcttcttgcttagccgagcttcgcataagtcacccgatatggtggttgatacaacattattgtgcatacagatgaatttttatg
caaacagtcagccctgaagaaggctgaaataatgttatgaaagagcgggattttatcaaatttcgttattgagaaacatgcctgcgtcacggca
tgcaaattctgcttaaaagtaaattaattgttatcaaattgatgttgttttggctgaacggtagggtatattgtcaccacctgttggaatgttgcgctaat
gcataagcgactgttaattacgtaagttaggttcctgattacggcaattaaatgcataaacgctaaacttgcgtgactacacattcttgagatgtgg
tcattgtaaacggcaattttgtggattaaggtcgcggcagcggagcaacatatcttagtttatcaatataataaggagtttaggatgaaagtcgca
gtcctcggcgctgctggcggtattggccaggcgcttgcactactgttaaaaacccaactgccttcaggttcagaactctctctgtatgatatcgctc
cagtgactcccggtgtggctgtcgatctgagccatatccctactgctgtgaaaatcaaaggtttttctggtgaagatgcgactccggcgctggaa
ggcgcagatgtcgttcttatctctgcaggcgtagcgcgtaaaccgggtatggatcgttccgacctgtttaacgttaacgccggcatcgtgaaaaa
cctggtacagcaagttgcgaaaacctgcccgaaagcgtgcattggtattatcactaacccggttaacaccacagttgcaattgctgctgaagtg
ctgaaaaaagccggtgtttatgacaaaaacaaactgttcggcgttaccacgctggatatcattcgttccaacacctttgttgcggaactgaaagg
caaacagccaggcgaagttgaagtgccggttattggcggtcactctggtgttaccattctgccgctgctgtcacaggttcctggcgttagttttacc
gagcaggaagtggctgatctgaccaaacgcatccagaacgcgggtactgaagtggttgaagcgaaggccggtggcgggtctgcaaccctg
tctatgggccaggcagctgcacgttttggtctgtctctggttcgtgcactgcagggcgaacaaggcgttgtcgaatgtgcctacgttgaaggcga
cggtcagtacgcccgtttcttctctcaaccgctgctgctgggtaaaaacggcgtggaagagcgtaaatctatcggtaccctgagcgcatttgaac
agaacgcgctggaaggtatgctggatacgctgaagaaagatatcgccctgggcgaagagttcgttaataagtaattgattagcggataataa
aaaaccggagcacagactccggtttttgttttgagcgcacgacttaattggttgccggatattcctgaatggtgacctgcagcgttaactgcttatc
atcacgcatcactactacagggatcaccgaaccagggcgaatttccgccacctgatccatcgtctccagagcagagatggccggtttgttatcc
accgaaataatcagatcgttgacctgaatacccgcattcgccgccgggccgtcaggtgacacttcattaaccacgatcccttgcagttgatctat
accaccgccctgcgcgtgcagtggtgcgatctcacgtccgccgataccaatgtagccgcggatcacgcgaccatcgcggatcagcttatccat
aattttggttgctaactggaaaggaatcgcaaagccgataccttccggcgtttcgccatcgttactcttatcaaacgacagcgtattaatgcccatc
agttcgcccagcgagttcaccagcgcgccgccagagttaccgtggttaatggaagcatcggtttggaggaagttttgccgcccggtcgggttca
gaccgattcgacccgtggcactaataatcccctgggtaatggtctgcccgaggttgtacgggttaccgatcgccagtactacgtcgccaatgtg
cggtacgcgacgtgcattaattggaatggtaggtaaaccgccagtggcattaattttaagtaccgccagatcggttagagagtcagatcccacc
agcaatgcttcaaatacacgtccatcctgtaaggcgacgatgatctgatcggcgtcgttgatgacgtgtttattggtgatgatataaccgcgttgat
ccatgattacaccggatcccagggtgcggatctcaagctggttgtgagagttggtgttcaaaccacggttgtaaacgttaaccaccgctggcgc
ggcgcggcgaaccgccagattatagctggcaggcgtctcatcggtactgtcaaattgcggagtggaaagcgggttaaggctgcgcagcgaa
ggcatggcaaccagcagaatagcgccgacaattaatccaatcgcaacggaacgtaagagcttcacaaacatgatggaggcgtcattaaaa
aagggaacggcagcagcataccacgagttaaccggacatcacacgtaagcctgatgcccggtttacgacattaacgcatcagcagatagat
gctttcattgccgcgtacaatttgcagggcgatgatggccggttttgccgccagcactttacgcatttcagcaatcgagttcacccgatcgcggttg
acgccaatgatcacatcgtctttttgcaagccagcctgagcagctgggcttcctttgacaacttcatcgattttaatacctttgccgccatcttttagct
gaccatcgctcaacgttgcaccttccagcgctggcgtgatcatttcagcgctggccgacgaagaggtgctggtatcgagcgtcacttctacttcc
agtggtttgccgttacgcagcaggccaagcttcactttcgtgcccggctcggtggtcgcgatacgagagcgcaactcagcaaagctattcagc

ggtttgccgttgaggctggtaataatatcgcccgctttgacgcccgcttttgccgagccagaacctggcaacacttcgctgacaaacgcgccac
gctgcacgtcaaggttgaaggctttggcgatatcggcactcatctcggtgcctttgatgcctaacaaaccgcgtttgatttcaccaaagtcgataa
gctgctgcgccagtgttcgcgccatattactggggatggcaaatccaatcccgacgctcccgccgccaggcgcaaggattgcagtgttgatgc
caattaactcaccgttaaggtttaatagtgcaccgccggagttaccgcggttaatggaagcatctgtctggataaagttttccagaccttcaagatt
caacccgctgcggcctaatgcggaaacaatgccagaggtggcggtttgcccaaggccaaatgggttaccgaccgctacggcaaaatcacc
gacgcgcaatttatcggagtcggcaatagcgatttgcgttaatttgctcgggttttgaatttgtaacagggcgatatcgctctggtcatcgctaccaa
tcagttttgcatcaaactcgcgcccatcattgagctgaatactgattttctgtgcctgattaatcacatggttgttggtcagcacatagcctttactggc
gttgatgatgacaccggagcctaaaccttcgaagggttgtgcaggttgatccggtaaatcatcaccaaaaaactttttgaattcttccgggattttct
gtccctgactggccgttccttccacccgtacgctcaccactgccggaagcactttttccagcattggagccagactggggagaggggcctgatc
ggcaacctggcctggaatcgacgcgacggcctgaaatgacgccgagagagttaacccgacacttaacgctaatgcactcaacagctgggtt
tgtttttcattattcctgctctcgtacctgaatgataagaaaagagattcaaaacagtttgatgttattgaattttcagacggctaacaatgagacac
cagattaaatgatagtcggggagggagagaagaggagggcgcaatggctgcgcccgaaaaataaattagtcgcgcttcgcgccagtacg
cagcaggccggatgcgccttcagaatagtcgcgaggcatctgcaccggtgcctgatcgttgctggcttcagactctgccagacgattacggaa
cgggtttgcttcagcagacagttccggcagcaggctgctggagctttttgccatgtgctgatacagctggcgatagtcgtgcgccatggtatccag
taattccgcgctgcgggcaaagtggctaaccagctcttcgcgatactcgtccagttcagctttattcttttccagttcgtactgcaacgcctgttgctg
gcgtagtttacgattaccaaaacgcatggccacagcaccaataatgatgccgacgactaacccaattagcgcatattcccaggtcatgaacat
ctcccgttgtcttttgtttccgtagggtgttggcttcaggctccatgcctgcggctgattatgccactataaccgttaattccacagaagtggaatccc
gactgcatatcgcgtagtgtagaacggcctttttttcgtcaaccgtgaacaacggcgcaccgattattcaaggaataacaataagatcatgcaa
agcgttaccccaacatcgcaatacctgaaggcgcttaatgaaggcagccatcaacccgacgacgttcaaaaagaggccgtcagccgcctg
gaaattatttatcaggaactcatcaatagcacgccaccagcccccaggacgagtgggctaatggcgcgggtcggtaagctgtggggtaaac
gcgaagacacaaagcatacgccagtgcgtggcttatatatgtggggcggtgtaggacgcgggaaaacctggctgatggaccttttctatcaaa
gcctgccgggagagcggaaacagcgcctgcactttcaccgttttatgctgcgggtgcatgaagagctaactgccttacaggggcagaccgat
ccgctggaaattattgccgatcgctttaaagccgaaactgacgtgctctgttttgacgaatttttgtttctgatattaccgatgccatgctacttggcg
gtctgatgaaagccctgttcgctcgcggtattaccctggtagcgacgtcaaatattccgccggatgaactttatcgaaatggcctgcaacgtgcg
cgttttctgcctgcaatcgatgccattaaacagcattgtgatgtaatgaacgtggacgctggtgttgattatcgtctgcgtacactcactcaggcgc
atctgtggctttcgccacttcacgatgaaacccgggcgcaaatggataaactatggttggcgctggcggggggaaacgagaaaattcaccg
acgttagaaatcaaccatcggccattagcaacaatgggcgtcgagaaccagacgctggcggtctcttttactacgctgtgcgtcgacgcccgc
agtcagcatgactatattgcgctctcacgtctctttcatacggtcatgttgtttgatgtaccagttatgacgcggttgatggagagcgaagcgcggc
gctttattgcgctggtggatgagttttacgagcgccatgtcaaattagtggtgagtgcagaagtgccgctgtatgaaatttatcagggcgatcggct
gaagtttgagttccagcgttgcctgtcacgtctgcaagagatgcaaagcgaagagtatctgaagcgcgagcatttggcgggttaaaacctgtca
caaatcacaaaaaggggtcgatctttgaccccgacttctctataatcctgcgaccccacgttacaagaaagtttttttcccaaaactttttgtgtgct
ggcataggctattcgaaggggtaggtttgccggactttgtcgtgtgaacctcaacaattgaagacgtttgggtgttcaccaacgtgtaactatttatt
gggtaagcttttaatgaaaacttttacagctaaaccagaaaccgtaaaacgcgactggtatgttgttgacgcgaccggtaaaactctgggccgt
ctggctactgaactggctcgtcgcctgcgcggtaagcacaaagcggaatacactccgcacgtagataccggtgattacatcatcgttctgaac
gctgacaaagttgctgtaaccggcaacaagcgtactgacaaagtgtactatcaccacaccggccacatcggtggtatcaaacaagcgacctt
tgaagagatgattgctcgccgtcctgagcgtgtgattgaaatcgcggttaaaggcatgttgccaaaaggcccgctgggtcgtgctatgttccgta
aactgaaagtttacgcgggtaacgagcacaaccacgcggcacagcaaccgcaagttcttgacatctaatcgggattataggcaatggctga
aaatcaatactacggcactggtcgccgcaaaagttccgcagctcgcgttttcatcaaaccgggcaacggtaaaatcgtaatcaaccaacgttc
tctggaacagtacttcggtcgtgaaactgcccgcatggtagttcgtcagccgctggaactggtcgacatggttgagaaactggacctgtacatc
accgttaaaggtggtggtatctctggtcaggctggtgcgatccgtcacggtatcacccgcgctctgatggaatacgacgagtccctgcgttctga
actgcgtaaagctggcttcgttactcgtgacgctcgtcaggttgaacgtaagaaagtcggtctgcgtaaagcacgtcgtcgtccgcagttctcca
aacgttaattggcttctgctccggcagaaaacaattttcgaaaaaacccgcttcggcgggtttttttatagctaaaatctgaatcagcgtaaaaact
ggaaagttgctttttgctgccacctgacagacaggtaaaacaaaaaccacatcgccaataagggactaagtcaactatttcagactaaagcg
catctcttttttccccatttccggcatcgactcaccacaatggtcgcaaaatctggtaaactatcatccaattttctgcccaaatgtcgggtattgctcat
tttttgtttgattttcgaacaaagagagtcgttccttattgggtaacacaacttctgactggccacctggtggctggtagcagtaaaaattctgactat
acctggaggttttcatggctgtcgctgccaacaaacgttcggtaatgacgctgttttccggtcctactgacatctatagccatcaggtccgcattgtg
ctggctgagaaaggtgtaagtttcgagatcgaacacgtggaaaaggacaatccgcctcaggatctgattgacctcaacccgaatcagagcgt
tccgaccctggtggatcgtgagctgaccctgtgggaatctcgcatcattatggaatatctggatgagcgtttcccgcatccgccactgatgcctgtt
tacccggtagctcgcggtgaaagccgtctgtacatgcatcgcatcgaaaaagactggtacacgctgatgaacaccatcatcaacggttcagct
tctgaagcagatgccgcacgtaagcaactgcgcgaagaactgctggcgattgcgccggtcttcggtcagaagccgtacttcctgagcgatga

gttcagcctggtcgattgctatcttgctccgctgctgtggcgtctgccgcaactgggcatcgagttcagcggcccgggtgcgaaagagctgaaa
ggctatatgacccgcgtctttgagcgtgactctttccttgcttctttaactgaagcagaacgtgaaatgcgtctgggccggagttaatctgtatggatt
tgtcacagctaacaccacgtcgtccctatctgctgcgtgcattctatgagtggttgctggataaccagctcacgccgcacctggtggtggatgtga
cgctccctggcgtgcaggttcctatggaatatgcgcgtgacgggcaaatcgtactcaacattgcgccgcgtgctgtcggcaatctggaactggc
gaatgatgaggtgcgctttaacgcgcgctttggtggcattccgcgtcaggtttctgtgccgctggctgccgtgctggctatctacgcccgtgaaaat
ggcgcaggcacgatgtttgagcctgaagctgcctacgatgaagataccagcatcatgaatgatgaagaggcatcggcagacaacgaaacc
gttatgtcggttattgatggcgacaagccagatcacgatgatgacactcatcctgacgatgaacctccgcagccaccacgcggtggtcgaccg
gcattacgcgttgtgaagtaatacaaaacaggcccaggcggcctgttttgtctttttaatgacttaataaaatagcgtgattgtggcaatcatattca
ccacgaaaccgactgccatggggatcgctgtccgcttcaccaccgcaaaaggcgaaacgcctgcaattcctgacgtaacgaccaccaccg
cagtaatcggcgaaaccgcgcgcgctagcgtcgtggcaaaatgcatcggcataatcattacaaccgctggtacatgtagtccggctgcgatat
tcggaataagactggcaaatgacataaacggcgcattgccagagcccatcacaatggcacaaatggcaatgaccagcgccataataatca
tcacgccaataccgcccagaccagaatgctccgcccccctgataaccgcatcgacagtgccaatcgtcgttaagcctttcgcaaatatttcacc
cgcgaccaccagcgttaccacgttggcaaactgcgtacccatgccgtcaaaaaacgcctgcacatcgtccattgtttcgcgcaagttatgcttg
cggaagaactcaacaaacatcgtcacagtcaaactcagtaacatcaccaccaccagatgcagttctgactgcatcagcccgacgtgggcga
ggaacagcgagccgagcatcaggattaacggcatcacaggtaaaatggcgtaatagagcggcgggacattatcgagagctttttgctctgcc
tgttcgtgattgatatttttatctttttgtcaaaagcgcgttgcacgaaaaagtgggagatcgccaccgagataatgacgcaagaggcgaccgg
aagctggtagtggaagaagtatgtggcaattttcattcccgctacctgggcagcaaaaatggagttcgtttccagaatcccccactcaatggac
atcgtggttgcgataactgccaccgcagagagacgacttactcccagactcaccagcgtcggaaataaggtgaccatcagcaacatacccа
gaccggaggcgctggtaataaactgcgccatgatttggccgatgacgtaagttgccgacagaataatatacggcgagcgaatgagttttaac
gggcggcttaacaagctcaccatcgcgcgactggcccctatgcgctccatgtagcgggcataaccgcccaccgccataatcgacagcccca
atccggcaatgcggttggacagcatgcgcaggatttcattataaatatcgacgatcaagtagccgctgcttttggtgggatcgagtacaccacc
gaaccctaaccagacaccgcacatcatcaggaagatacctgcggcagccagcaccacctgaggtttgtagtttttcaggatcaaatagccca
tcgtaattaatacgatgacagatataattatgccgaacattgttgaactccgtgtcaaaagaaaacggtcaatcccataaacggcagattgaaa
acaacgatgttatatttttgcaaggctatttatggtgcggatgtcgtgtttttaattgtaggtgaggtgatttttcattaaaaaatatgcgcttatgattatt
tgtaagaacacattcataatattcataatgctcgtgaatagtcttataaataattcaaacgggatgtttttatctgcgttacattaatttttcgcaatagtt
aattattccgttaattatggtaatgatgaggcacaaagagaaaaccctgccatttcccctactttcaatcctgtgataggatgtcactgatgatgtta
atcacactgaccttacagaatgggccttatgaacgcatttgattcgcaaaccgaagattcttcacctgcaattggtcgcaacttgcgtagccgcc
cgctggcgcgtaaaaaactctccgaaatggtggaagaagagctggaacagatgatccgccgtcgtgaatttggcgaaggtgaacaattacc
gtctgaacgcgaactgatggcgttctttaacgtcgggcgtccttcggtgcgtgaagcgctggcagcgttaaaacgcaaaggtctggtgcaaata
aacaacggcgaacgcgctcgcgtctcgcgtccttctgcggacactatcatcggtgagctttccggcatggcgaaagatttcctttctcatcccggt
gggattgcccatttcgaacaattacgtctgttctttgaatccagtctggtgcgctatgcggctgaacatgccaccgatgagcaaatcgatttgctgg
caaaagcactggaaatcaacagtcagtcgctggataacaacgcggcattcattcgttcagacgttgatttccaccgcgtgctggcggagatcc
ccggtaacccaatcttcatggcgatccacgttgccctgctcgactggcttattgccgcacgcccaacggttaccgatcaggcactgcacgaaca
taacaacgttagttatcaacagcatattgcgatcgttgatgcgatccgccgtcatgatcctgacgaagccgatcgtgcgttgcaatcgcatctcaa
cagcgtctctgctacctggcacgctttcggtcagaccaccaacaaaaagaaataatgccactttagtgaagcagatcgcattataagctttctgt
atggggtgttgcttaattgatctggtataacaggtataaaggtatatcgtttatcagacaagcatcacttcagaggtatttatggcaacgaatttacg
tggcgtaatggctgcactcctgactcctttgaccaacaacaagcactggataaagcgagtctgcgtcgcctggttcagttcaatattcagcagg
gcatcgacggtttatacgtgggtggttcgaccggcgaggcctttgtacaaagcctttccgagcgtgaacaggtactggaaatcgtcgccgaag
aggcgaaaggtaagattaaactcatcgcccacgtcggttgcgtcagcaccgccgaaagccaacaacttgcggcatcggctaaacgttatgg
cttcgatgccgtctccgccgtcacgccgttctactatcctttcagctttgaagaacactgcgatcactatcgggcaattattgattcggcggatggttt
gccgatggtggtgtacaacattccagccctgagtggggtaaaactgaccctggatcagatcaacacacttgttacattgcctggcgtaggtgcg
ctgaaacagacctctggcgatctctatcagatggagcagatccgtcgtgaacatcctgatcttgtgctctataacggttacgacgaaatcttcgcc
tctggtctgctggcgggcgctgatggtggtatcggcagtacctacaacatcatgggctggcgctatcaggggatcgttaaggcgctgaaagaa
ggcgatatccagaccgcgcagaaactgcaaactgaatgcaataaagtcattgatttactgatcaaaacgggcgtattccgcggcctgaaaac
tgtcctccattatatggatgtcgtttctgtgccgctgtgccgcaaaccgtttggaccggtagatgaaaaatatctgccagaactgaaggcgctggc
ccagcagttgatgcaagagcgcgggtgagttgtttcccctcgctcgcccctaccgggtgaggggaaataaacgcatctgtaccctacaattttc
ataccaaagcgtgtgggcatcgcccaccgcgggagactcacaatgagtactacaacccagaatatcccgtggtatcgccatctcaaccgtgc
acaatggcgcgcattttccgctgcctggttgggatatctgcttgacggttttgatttcgttttaatcgccctggtactcaccgaagtacaaggtgaatt
cgggctgacgacggtgcaggcggcaagtctgatctctgcagcctttatctctcgctggttcggcggcctgatgctcggcgctatgggtgaccgct
acgggcgtcgtctggcaatggtcaccagcatcgttctcttctcggccgggacgctggcctgcggctttgcgccaggctacatcaccatgtttatcg

ctcgtctggtcatcggcatggggatggcgggtgaatacggttccagcgccacctatgtcattgaaagctggccaaaacatctgcgtaacaaag
ccagtggtttttgatttcaggcttctctgtgggggccgtcgttgccgctcaggtctatagcctggtggttccggtctggggctggcgtgcgctgttcttt
atcggcattttgccaatcatctttgctctctggctgcgtaaaaacatcccggaagcggaagactggaaagagaaacacgcaggtaaagcacc
agtacgcacaatggtggatattctctaccgtggtgaacatcgcattgccaatatcgtaatgacactggcggcggctactgcgctgtggttctgcttc
gccggtaacctgcaaaatgccgcgatcgtcgctgttcttgggctgttatgcgccgcaatctttatcagctttatggtgcagagtgcaggcaaacgc
tggccaacgggcgtaatgctgatggtggtcgtgttgtttgctttcctctactcatggccgattcaggcgctgctgccaacgtatctgaaaaccgatct
ggcttataacccgcatactgtagccaatgtgctgttctttagtggctttggcgcggcggtgggatgctgcgtaggtggcttcctcggtgactggctg
ggaacccgcaaagcgtacgtttgtagcctgctggcctcgcagctgctgattattccggtatttgcgattggcggcgcaaacgtctgggtgctcggt
ctgttactgttcttccagcaaatgcttggacaagggatcgccgggatcttaccaaaactgattggcggttatttcgataccgaccagcgtgcagcg
ggcctgggctttacctacaacgttggcgcattgggcggtgcactggccccaatcatcggcgcgttgatcgctcaacgtctggatctgggtactgc
gctggcatcgctctcgttcagtctgacgttcgtggtgatcctgctgattgggctggatatgccttctcgcgttcagcgttggttgcgcccggaagcgtt
gcgtactcatgacgctatcgacggtaaaccattcagcggtgccgtgccgtttggcagcgccaaaaacgatttagtcaaaaccaaaagttaatc
ctgttgcccggtctatgtaccgggcctttcgctaagggaagatgtatgtcgttacttgcacaactggatcaaaaaatcgctgctaacggtggcctg
attgtctcctgccagccggttccggacagcccgctcgataaacccgaaatcgtcgccgccatggcattagcggcagaacaggcgggcgcgg
ttgccattcgcattgaaggtgtggcaaatctgcaagccacgcgtgcggtggtgagcgtgccgattattggaattgtgaaacgcgatctggagga
ttctccggtacgcatcacggcctatattgaagatgttgatgcgctggcgcaggcgggcgcggacattatcgccattgacggcaccgaccgccc
gcgtccggtgcctgttgaaacgctgctggcacgtattcaccatcacggtttactggcgatgaccgactgctcaacgccggaagacggcctggc
atgccaaaagctgggagccgaaattattggcactacgctttctggctataccacgcctgaaacgccagaagagccggatctggcgctggtga
aaacgttgagcgacgccggatgtcgggtgattgccgaagggcgttacaacacgcctgctcaggcggcggatgcgatgcgccacggcgcgt
gggcggtgacggtcggttctgcaatcacgcgtcttgagcacatttgtcagtggtacaacacagcgatgaaaaaggcggtgctatgaccacact
ggcgattgatatcggcggtactaaacttgccgccgcgctgattggcgctgacgggcagatccgcgatcgtcgtgaacttcctacgccagccag
ccagacaccagaagccttgcgtgatgccttatccgcattagtctctccgttgcaagctcatgcgcagcgggttgccatcgcttcgaccgggataa
tccgtgacggcagcttgctggcgcttaatccgcataatcttggtggattgctacactttccgttagtcaaaacgctggaacaacttaccaatttgcc
gaccattgccattaacgacgcgcaggccgcagcatgggcggagtttcaggcgctggatggcgatataaccgatatggtctttatcaccgtttcc
accggcgttggcggcggtgtagtgagcggctgcaaactgcttaccggccctggcggtctggcggggcatatcgggcatacgcttgccgatcc
acacggcccagtctgcggctgtggacgcacaggttgcgtggaagcgattgcttctggtcgcggcattgcagcggcagcgcagggggagttg
gctggcgcggatgcgaaaactattttcacgcgcgccgggcagggtgacgagcaggcgcagcagctgattcaccgctccgcacgtacgcttg
caaggctgatcgctgatattaaagccacaactgattgccagtgcgtggtggtcggtggcagcgttggtctggcagaagggtatctggcgctggt
ggaaacgtatctggcgcaggagccagcggcatttcatgttgatttactggcggcgcattaccgccatgatgcaggtttacttggggctgcgctgtt
ggcccagggagaaaaattatgatgatgggtgaagtacagtcattaccgtctgctgggttacatcctgcgttacaggacgcgttaacgctggcatt
agctgccagaccgcaagaaaaagcgccgggtcgttacgaattacagggcgacaatatctttatgaatgtcatgacgtttaacactcaatcgcc
cgtcgagaaaaaagcggaattgcacgagcaatacattgatatccagctgttattaaacggtgaggaacggattctgtttggcatggcaggcac
tgcgcgtcagtgtgaagagttccaccatgaggatgattatcagctttgcagcaccattgataacgagcaagccatcatcttaaaaccgggaatg
ttcgccgtgtttatgccaggtgaaccgcataaaccaggatgcgttgtcggcgagcctggagagattaaaaaggttgtggtgaaggttaaggctg
atttaatggcttaaaacttagttcgttttaaagattctgcagacactgtctgcagaattcgaaaccgctgaaattaacggcgtctatagccttcctcta
gcaccgcctgtgttcgaacgagttcatctgcgagtcgtttctcatcaggtagttgcatcttatattcgcttgccagaacggtgttaggcaaacctgcc
agagcataatgcgcttctccggctcctttctctgcacagagaaccagaccgatgggcggattttcatccggtagcgtccagtgctctttggcgtag
ttgagatacatattcatctgtccggcatcgctatagctgaatttgcccacttttagatcgacgattagcaggcagcgtaaacggcggtggaaaaa
cagcagatcgacacgaaaccagttgtcatcaatgcgtaaccttcgctgccgaccaacaaaggcaaaatcatccccaagttccagcatgaaa
tccatcaggtggttgatcagcgcctcctcaaaatcggattctgaatattcatctttcaattccagaaattcgagcacaaagggatcgcgtatcgcct
gttgcggaagaatatgcgtctcggcaggagcgtgttgttgcagcatggctgatttgtcatgtgacagtagtgtccgctcataaaactgggtcgca
atttgccgctctagctggcgaacagaccagccacagcggagcgtctctttttcataaaagctgcgggcgtcagcgtttttaacggaaagtaaac
ggacgtaggtggacctgggcaaggggaaagatttggcagacagcgtctgccaaatctttgccgatgggaattctgcagacggtgtctgcgga
attcccaatggtgttaattcgccagacattgtctggtgaatttcaacatgttgaaaaaagaggtaaaaaagcctcatttggcgtaagtttttttgcaga
gaacccacgcttataccttagacataaatcctttgatagtcgcttgattagctgcgcaccatacgcagccctggcctcgccaccttgttcaaattcg
acaattcgtcggccaatttcctggtacgtcgcggtcattaacgcgttaacgctacgtaccgtttccgtccgggcgctatcgaccagatgaataata
ccgtcgtggatttgctggtagcctgctgtggttccttctgagagcgattccattatctgtccctttatatggagctaagttgaataatggaatgagctat
actctgggggtgagcactgaacaattaagaacagcgtattacatttactttccccaagatatgcgaagagctttcctgaaacattaaccgatcg
tataaaacgaatgcctgatagtactcctgtccggcattgttatcacaagtagactacgcttatcacagctttcccatcaagttcgatttttttctgaacc
gcctaaatcattcgcgatagatgcgctcacctgaagcggtataatggcgttcgttacaggagttggggttgtttcacctggttttacaaaggtgtaa

taatagctttgatcgactgcaagcggataagcggtgagcgtttgccatgctctttttgccctgttatgacggcatctgcacctgctattgatacagct
aacgtctggctaccattgctggcactaatgttgttgctatcaattaacacggcataactaccgatatttttattatcttttgttttccctaagccgaataa
gcgcgttgtctggctaatataacctccgccatttaaggtatattttacaccactctgacccccactatcttttccggtggtcatactatccattcgtgtat
cacgagcggtaatcgcaatgccggtagcggattcgcaagttatagtcatattaatattacgtgttccaatctgtgtgtagtgctgcgttgaaagtct
attgactgaatgactaccgaagttaacaactccgccgttgcttagtattggagtacaagctgcagcttccagagtcaccttaacgtcaattgatac
tctatcagcggcttgcgctgacatggcaaaaaaggcacttccggcaatcagtaattttacattgttcattttgcctccaccaggaccgtcagattgc
ctgtaatatctgtcatgtcggttagttccccttcatccttaaaagctggagccattgtcagtgtgaggctaacgcttttaccgctatcaattaccacttct
tgaccattttgcataaacaccagccaattattgggggaagtactaaggaaggtgcgaacaagtccctgatatgagatcatgtttgtcatctggag
ccatagaacagggttcatcatgagtcatcaacttaccttcgccgacagtgaattcagcagtaagcgccgtcagaccagaaaagagattttcttg
tcccgcatggagcagattctgccatggcaaaacatggtggaagtcatcgagccgttttaccccaaggctggtaatggccggcgaccttatccg
ctggaaaccatgctacgcattcactgcatgcagcattggtacaacctgagcgatggcgcgatggaagatgctctgtacgaaatcgcctccatg
cgtctgtttgcccggttatccctggatagcgccttgccggaccgcaccaccatcatgaatttccgccacctgctggagcagcatcaactggcccg
ccaattgttcaagaccatcaatcgctggctggccgaagcaggcgtcatgatgactcaaggcaccttggtcgatgccaccatcattgaggcacc
cagctcgaccaagaacaaagagcagcaacgcgatccggagatgcatcagaccaagaaaggcaatcagtggcactttggcatgaaggcc
cacattggtgtcgatgccaagagtggcctgacccacagcctggtcaccaccgcggccaacgagcatgacctcaatcagctgggtaatctgct
gcatggagaggagcaatttgtctcagccgatgccggctaccaaggggcgccacagcgcgaggagctggccgaggtggatgtggactggct
gatcgccgagcgccccggcaaggtaagaaccttgaaacagcatccacgcaagaacaaaacggccatcaacatcgaatacatgaaagcc
agcatccgggccagggtggagcacccatttcgcatcatcaagcgacagttcggcttcgtgaaagccagatacaaggggttgctgaaaaacg
ataaccaactggcgatgttattcacgctggccaacctgtttcgggcggaccaaatgatacgtcagtgggagagatctcactaaaaactggga
taacgccttaaatggcgaagaaacggtctaaataggctgattcaaggcatttacgggagaaaaaatcggctcaaacatgaagaaatgaaat
gactgagtcagccgagaagaatttccccgcttattcgcaccttccctaattgatgctgttcctgtgctgcccggattgtcatttttgccactggctaac
gccaggtcgagattgttactacctgaaaatgcatcgctggcgatcagatttaatgaaccattcgggccaaatccaaatccgttgttggaaatatta
gcggtattcaactgaacacgaatatgagcgtcctgatcgcattgcattaccagcgtgattgttctgtctgctaattgtgttgttttaccccggtcaactt
ggcgcatggcagcacttcgtttgccatagtcgatattttgtatgctactggagatattacactctgcctgagcggccatggcgaagttcagcagca
ggcatcctgttattatccgtttcattggcactctcctgttgctgtctcatagaacacatcttttggtgcatcttctgggaatgtaaggtgaatacggcatg
attgctgctcatcttttatatctagcaccatgtccggtttgatgttattgaggaatacaacaccgtcatccactgatgttgtcagatagttgccttcacta
tcttcaatcgcaacgcctctgggcagtttttttgccgtcggaaagtgtgatattgagtaatgcacggcgttgtgttatcgcactgaatccgactttccct
actgcaccacggccttgtttgatcatttttgtgccgttaccgatatcgacatttttttggcaagctggcggtatcgatctcaactcctgatgttcgccacg
gctggattgatggaatgacggcattaccccgaaaatctgtccatactgggcctgcctgagatgtaattctgacacctgcaatattgttatccattttt
gcgatggcgaaagtgtcattgatagtccacggtgaaaaggtcactccctgatcatgtacggcaataccacctgacatcgtaccgttgtaagtac
ggctatcactgccgcttgctcctgcggcaaggctgacttgggtgtaatggagattggaactgatactgccgttcaagctcgtttcacgttcgtcgtg
atctcgttcagcattcacatagtaggacatctcatctgagacgactcccatgacaccagtaccatagtgggttttatgatcgtcatggcgagtatac
agtgtggcggtgtttgatctgccaaatggaatactgatgttgacgtagaacagatcaccatcgtcttcattattttctgaagcactaagttgatgctg
ccagttgacggaaaccgtggcgtgtttgaacgctttgttccagttaatattaaggttgcttgagtcattgtcaccgtcgtaactatatgttttatagcca
ctgatgttgaaaccacccagaatggagttagaccagtttaaaccaagcgcataagtgcttttagtcggatcggtataatcatcatcaatcgcttca
gacagttcgcgataatggcgatcagatcgagtgaggctggttgttagcccaagtgaaaatgggagattgtagttcgcgtcaagacgatatttctg
tccttgcagtgagtctttcgtatcctgcgatgtactaatttggctgctcactgttaaatcgggcaggggaaccagactcgaccggattgatgccgcc
tgataattttctgctacgataacgccgccgttcattgcactccagcgtgtcagattccatccgctcgatgcactaattacccaaggtgattcgtcata
gtcatcgtctacccgccctatcgcgaagcgataaccctggaagctttctacatgctgattaaacagggtggagggaacaatatagttgtgcgaa
ctaccgtctgtttcgacaacggtgacattaagatcactattaccattgcgaacaggtacatcagggatagtgaatgcgcccgcaggaaccaga
atggaatgaattaaaactccttgttgacgaatctcgacacgagcctgagaggtgttggctataccagtaacttgcacaccactgccgctggtttg
caatgcgttgtccggtgcgatttcgataccgtaaatactggctccttccaacacgctattattgaggttaacttcacctgctcgcatgagtgttttaag
atctgtaaaggtacgttgaaggtaggttgacgagttctggttactgaatgtgccatttgtttgtgtaaggaactgatggctgcgtaacatccagtcatt
aatattaatcccgccttcaagtgcagcctgggagtagtccgaactcccattagaaaattctgcacggctgctcatcagagagtagtttagcagcg
cagctgttccaccagttgctgcgtttgtgagatccaaccctatgggaatgatcgcctgtggtgaaacgataatatctaacgcttcctggttaggtaa
tggtgtgattgtggtacccgggtaggacaatatatagtcgtaacagccattttttttcttcagaaggaatttttaaaccggcttgttgcagaaatgcctg
atcaagacaaatgtcgccattttcatcaaagcgcgtagcaatgttgccttttttttcgccatttactgatactatcagtgaatattcacctggcaaaaa
acgggctgaacgggaaaaataatgcgatatattaggatctacacctagtgattttaatgtttcccgatcaaaatcaacagcggcaatctctatac
catctgcattggctgggggagaaaaggcaaaaccaatggtgtaggccagtaacgtttttttaacatccttgtcgctccaggcaaagttattttaa
gtcagtgacatatttattaccgcgataaccatatcggctggctgggtaaaactcgactttgtatccgtagcgtttgttaatgcagtagttgtactggt

gtgtggtagaaaataggtcttatttatagcccccgtttttcctgaagggagcgttttaaattgtaaggtcatacgaacgacataattactggggttgct
aatctcaatttggtcgccatttttacgccattccaggaatttccaggtctctcgctcttccggcagggaagccgggtggatcagtacaggtagatcc
tgacgaattgaaacagtaactctgctgctcttgtcatcttttggtggaataccttcgaacgttacgcgtttcagctcttctgattgcagaggaacagtc
gcctgaagtaaaaatcgaacctgttgtacttgaccggcttcaacacgaataacgggctgagtcgggattaaacgaattgatttattgctttcaggc
aaatcaacaatggtggtgtaaagcaatgaaggatgatcatctgtattcttaatattaatactagcttcaccacgcttttcatcaaccagcaatactg
atgtttcaggaaccatccctgtggctaaggtattcattgatgttgttataaatacagtgaatgtaatgtgtctgagcatttaaattaaaactcctgtaat
aatgataaataacgcagaaacatattgttaatatatgttatgcactgtttgcgatgagattgattaagacagaccatgtcatagggcataatctgtc
cgagttatagctaactggttaatgcgtaaaaataaaaactgtattacaagttcttataaatactgaacgctgatagtgacgttaccatccagtttgat
cgtttcatcagaaccaagaatagtattgtcatttaccgcagcatcaatcagtaacgggaaggtgaccgctgtaatagcaacaggtgtcagttcc
cctgtttttgcaacagaataaccgcgctcgattgctgagcaacttgtcagcgagcagaatgcgccattagatgaggctttccatgtgggggtcgc
tgctgaccagtggtcagtttgaatcaggtcgacaggaatttcacctgtatcgtccgccgcgtgaatcccaccattagacctgtcaataccaatgg
atgcagcgccaatttttgtccattaggcgcagaacctaaaccaaaggcgtttttcgttcccttcatatctacgagagtatcataagctttttcaatat
aggatgtgtcgtttaacgctacaatggtcgactgacggttatccttggaggttacagcaattagcgttggtgctgtgcaggttatatttaatgtgctgtt
tttgcggcccagttgaaccagtttattacttttaccggtcggattcagcgcactgttatgatgtttcccatagtcaacaatgccgccgcccgtaacaa
ctggggtacatgcaccaggggtatactcgccgatgatagttaattctgtatcagttgaatctgttgccatggcactgaatgctgcaacagaaagtg
ctgcacaaatagcagctgttgtgaggttctttttgaaaaacatatgaacatatccttaataacaaatttatttttaaaataaaaagctgaagcagta
tatatatctgtttgtgaatgtaaataaataactcgttcagaaatagccaacttgctgggatgtatttatttaatgaattttatagttacgatcatgtgaata
ataaggtgctggtgaggtggtttgtggtgataattattcattacatgaatgtttgcgctgaaaaataggaaaatatatagataattaaggataatata
ttggtttttatattcatattttgtatattaatatcgagacaatttacatggaatgaaaagactgaatagcttgcagaatcggtattgtcagaggtgttaa
caagtaattgagacggattgtaaatgagaaattgcttgatgtgcttcactcatcagacttggtgaacaaagcacaatttattgaaaattatccctatt
ataggaaaggtcaaacgctcatgccgcatttgccagaaaacaacgcgcacttcgtcagtaatttgattcgccagacacagtctggcgaattcat
tgttacctcgcttaaacttccagccagttcataataccgtctgccgccttacgaccttcggcaatagcggtcaccaccagatcggaaccacgga
cgatatcgccgccagcaaagattttcgggttgctggtctggaaggcgttgtcgctgccttccggggcgatgatgcggccttgtgaatccagctcg
acgctgtgttttgccagccattccatgttgtgtggacggaaaccaaacgccatgatcaccgcatctgccggaacgatatgttcggaacctgcaa
cgatctccgcgcggcgacggcctttggcgtccggttcgcccatttcggtacgcaccatttttacgccgctgactttgccgttaccgttcacttcaata
cccagcggctggacgttgaatttgaactctacgccttcttcccgcgcgtttttcacttcgcggcgggaacccggcatgttctcttcatcacgacgat
aggcacaggtaacgtgcttcgctccctggcgcacggacgtacgcacgcagtccatcgcagtgtcgccaccgccaaggaccaccacgcgttt
gccttccatgctgacgaacggttcgtcgcgggtttcaccaaagcccattaactgtttggtgttggcgatgaggaacggcagcgctgcgtacacg
ccatcggcgtcttcgttttccagcccgccgcgcattgactgataagtcccgacgccaaggaacacggcatcgtaatcactcagcagatcgtcc
agctgtacgtcgcggcccacttcggtattgagtttgaattcaatacccatgccggtgaagatttcacggcgacgcgtcattacctctttttccagctt
gaaggccggaataccgaaggtcagcagcccgccaatttctggatgacggtcgaagacaacggcttttacgccgttacgcgtcaggacatcc
gcacacgccagacctgccgggcctgcgccgataatcgccactttttttaccggtctgtttcacaccagacatatccggacgccagcccatctcga
acgctttatcgttgatatagcgctcaatgttgccgatggtcaccgcgccaaactcatcgttcagagtgcaggaaccttcgcacagacggtcttgc
gggcagactcgtccgcaaacttccggcagggtgttggtctggtgcgacagttccgccgcttcaaaaatacgcccctcgttggcgagcttcagcc
agttcgggatgtagttgtgtaccgggcatttccactcgcagtatgggttgccgcacgacaggcagcggtcagcctgcgctttggcctggccttcg
gaaaacggctcgtaaatttcaacaaactcaattttgcggatcttcagcggtttctttggcggatcaacgcgctgcaggtcgataaattgataaaca
ttctgactcattgttgctaccccttactgcgcctgcacgcgcaactcagctgcgctacgactacggtgacccagcagtgcttttacatcactggact
tcggtttaaccagcgcaaatttagtggcgaaggttgaccagttcgccagaatctcttcaccgcgctgagagccggtatgctgcacatgctcggtg
ataagaccacgcagatgctcttcatggatcgccagagcgtcaacgcttaagacctcgaccagttccgggttaacgcgtttgcggaaatcgccg
ctttcatcgagaacgtaagcgaaaccgccggtcatgcccgcaccgaagttaacgccggttttacccagaatgcagacgataccacccgtcat
atattcacaaccgttgtcgccaatgccttctaccacggtgatagcaccggagttacgcacgccgaaacgttcacccgcgcggcctgcggcata
cagacgaccaccggtcgcgccatacaggcaggtgttgccgataatgcttgcttcatggctgcggaaggcggaaccaaccggaggacgaat
ggcgattaagccgcccgccatgcctttaccgacatagtcgttggcatcaccggtcaggtacagttccacgccgcccgcgttccacacgccgaa
gctctggcctgcggtgccgttgaagtacgctttgataggatcggctgccagcccctgatcgccgtgcgtctgggcgatatagcctgaaagcgac
gcgccgacagaacggtcggtgttgcgaatatcgaaccagaaggttttgctctggcgctcatcgacaaacggtttcgcctgttgcagcaactgcg
cgttcagcaggccgttatcaaacggcgggttgttttcggtgcagtagagtgccttacctggatgcggttcggcagtctccagcagcttcgacagc
gccagcttctgctgtttggcggtgaaaccgtccagctctttcagcaggtcggtgcgaccaatcagatccaccagacgtgttacgccaagctgtgc
catcagctcgcgggtttcacgggcgataaactcaaagtaattcgtcaccttgaatggcaggccgtgatagtggttcttacgcagtttgtcatcctg
agttgctacacccgttgcgcagttgttcagatggcaaatacgtagatatttacagccgagcgccaccatcgggccagtgccgaagccgaagct
ttctgcgccgagaatcgccgccttgatgatatcgacacccgttttcaggccgccatcgacctgcaaacggatcttgtgacgcaagccgttagca

accagcgcctgctgggtttcaacaagccccagctcccacggacagcctgcgtatttcaccgatgaaagcggacttgcgccggtgccgccgtc
atagcctgcgatggtgatcaagtccgcataagcttttgccacgccagtcgcgatggtgcctactcccggttcggaaaccagcttcacggagatc
atcgctttcgggttaacctgcttgaggtcgaaaatgagctgcgctaagtcctcgatagagtagatatcgtggtgcggcggcggggagatcagcg
tcactccgggcaccgaatagcgcagtttggcgatgtaaggcgtgactttatcacccggcaactgaccgccttcgcctggcttcgcgccctgggc
gactttaatctgaatgacgtcggcattgaccagatacgccggagtaaccccaaagcgaccggaagccacctgcttgatgcgcgacactttgtt
ggtgccgtagcgcgccgggtcttcgccgccttcaccggagttcgaattaccgccgatgctgttcatcgcttccgccagcgcctcgtgggcttccg
ggcttaacgcgccgatagacatcgcggcggtatcaaagcgtttaaacagttcgcttgccggttcaacatcagcaatgttgaccgcgttttcaccc
ggcgtaattgccagcagatcgcgcagcgtggttgccggacgctcattaaccagcttcgcgtattcctgatagtcgctgtactcgccgctttgtacc
gcttgttgcagcgtgcgcaccacgtccgggttgtaggcgtggtattcgccgccgtggacgtatttcagcagaccgccctggctgatgggcttacg
cgccagccaggcacgtttcgacagattcagcagatcctgctggaagtcttcaaagcttgctccaccaatgcggctgaccgcccccctggaagc
acaggcccactacatcatcgtgtagaccgaccgcttcaaacagtttcgagcagcggtaagaggcgatggtggagatgcccattttggacatg
attttgtacaagcctttgttgatgccgttacggtagttgagcatcacggtacgataatctttggcaatcgcatgggtgtctaccaggcggcccagcg
tttcataggcaaggtatggataaatagccgtcgcgccgaagcccagcaacacggcgaagtggtgcggatcgcgggcgctggcggtttcgac
gatgatgttggcatcgcaacgcaggctttgatcgaccagacgggtctggatcgcgccaaccgccatcggggctggaaccggcaggcgatctt
tagcgatattccggtcggagagcaccagcagcacggtgccgctacgtaccatttttcggctttgtcgcacagctctttgactgtcgcttcgagcgt
ggttttagtgacgtcaaaggtgatatccagcgtatctgcgcggtagtgctcctctttcatcgtcgtgagctgtttgaaatcggagtagagcagaatc
ggcgatttaaagcttaaacggtgcgcctggccctctgcttcgcaaaagacgttcatttcacgaccgatactggtggcgagcgacataacatgcg
cttcacgcagcgggtcgattggcgggttagtcacctgggcaaactgctggcggaagtagtcgtaaataatgcgcggctgactggagagcacg
gcgaatggggtatcatcgcccatcgaaccgaccgcttcctgaccgttttcgcccagtacgcgaattacggagtccagctcttccgcgctgtagtt
aaactgtttctggtagctggcaagcgtgtcgtcgtccagttcgcggctacccacttcttcatcgggcagatcttcaaacggtaccagtcggcgga
cgtttttctccatccactctttatatggatggcggcttttcagatcgtcatcggtttctgccgagtgcagaatacgcccactgcgggtgtcgataacca
tcagttcgcctggcccgacgcggcctttttcgaccacttcgtcaggctggtaatcccagataccgacttcagaggcgcaggtgatgagcttatcttt
ggtgatgacgtagcgcgccggacgcagaccgttacggtcgaggttacaggcggcaaaacgaccgtcggacatcacgatgcccgccgggc
catcccacggctccatatgcatggagttaaagtcaaagaaggcacgcagttccgggtccatatccgggttgttctgccaggcgggtggtactaa
tagacgcatggcgcggatgatatccatcccgcctgccagcagcagttccagcatgttatccatcgaactggagtcagagccggtttcgttgacg
aacggtgcggcgtcgtgcaggtcagggataagcggtgtctggaatttataagtacgcgcacgcgcccattggcggttaccggtgatggtgttga
tttcaccgttatgcgccagatagcggaacggttgcgccagcggccagcgcggtacggtgttagtggagaagcgctggtggaacaggcaaat
ggccgattccagacgcaggtccgcaagatccagataaaagcgcggcagatccgtcggcatacacagacctttatagatgttcaccagattcg
acaggctacagacgtagaagtctttgtcggcttcgagacgcttttcaatgcggcggcgggcgataaacagacggcgctccatatcgcgtggac
gccagcctgccggggcgttcacaaaaatttgctcaatgcgtggcagagaggagagggcgatttcacccagcacgccttcgttagtggggaca
tcacgccagcccacaatcgacaaggtttcgcgttgcagttcttcttcaacgatgcggcgtgcggcagcggcgagttcaggatctttattcaggaa
gagcatcccgacagcgtagtttttttgctaaacgccagccgcgctcctgcgcaacgatgcgaaaaaagcgatccggtttttgtaacagcaagcc
gcaaccgtcgccggttttaccatcggcgagaatcgcgccacggtgctgcatgcgggccagtgcgtgtattgcagtacgcactaccttgtggcta
ggttcgccttctatgtgggcgatcaggccgaaaccacagttatccctctcaagggatttatcgtacaacatatcgtgaacctcccccaggctctgc
gggaaccccacttccgaaccgctgcgcacgggcacagaaagagcgtggcgacggggtttgcgtgtcatacgcgcgcctcgcattcgcccc
cttgttgtccttttcgcatcggttaatacggtgaagaggggatctcaattactgcataaatatgatgagcaggctgctcatccagaaagcttccagc
ggatttccaacttatcgggaaagctgatacaggtcaaatggcaagcttattggtacagaaatgtgctttagcgcaaaattctttattaaatgactga
aattaaaatggattttatctttaaggctaaaagggaagtcgttagagaaacagtcaattcctgactgtcataaaggtgtgttaacccttatcctgcct
tgaactgacgacaaaaccagcattttatactgccttaattggtaatgacgtttcatgctactgtttttgcctaaaatccatcaggagataaaaaaatc
tgacacaagataagcaaaagtgatttatgtgattatgcatgaaatttcggtaattttgattgattatgcagttgataaaagcaggccgggacgattg
atccaggtcatcgccgaattgagctaaaaatggcaggctttggcctcttttcaaggcgcggtctatcagattatgcagttacagaaattagtcaat
atgtttggtggtgatctcacccgtcgttatgggcaaaaggtgcataagctgacgctgcacggcggttttagctgccctaaccgtgacggtaccatc
gggcgtggtggctgcacattctgtaatgttgcctcgtttgccgatgaagcgcagcagcatcgttccattgccgagcaactggcgcaccaggcga
atttagttaaccgcgctaaacgctatctggcctactttcaggcgtataccagcacctttgcggaagttcaggtgctgcgttctatgtatcagcaggc
ggtgagccaggccaatattgtcggtttgtgtgtgggtacccgcccggactgcgtgccggatgcggtgctggatctgctttgcgaatataaggacc
agggttacgaagtgtggctggagctggggctacaaaccgcccacgacaaaacactgcatcgcatcaaccgcggccatgattttgcctgttatc
agcgtacaacccagctggcacgtcagcgcgggctgaaggtgtgttcccacttaattgtcggcctgcctggcgaagggcaggctgaatgtttgc
aaacgctggagcgagtggttgaaaccggcgtggacggcataaagctgcatccgctgcatattgtgaaaggcagcattatggcgaaagcctg
ggaagcggggcgtttgaacggtattgaactggaggattacacgctcactgccggagaaatgattcgccatacgccaccggaagtgatctacc
accgcatttccgccagcgcccgtcgcccgacgctgcttgctccgctgtggtgcgaaaatcgatggacgggaatggtcgagctggatcgctatct

gaatgaacatggcgtacagggatcggcgctgggacgtccgtggctacctccaacggagtaggtcgttgaggggaattccgcatttctcacac
aatttataacgtaactgtcagaattgggtattattggggcaggttgtcgtgaaggaattccctaatgaagcaaattcgtctgctggcgcagtattatg
ttgacctgatgatgaagttaggtctggtgcgcttctcaatgttgctggcgctggccctcgtcgttcttgccattgtggtacaaatggcggtaaccatg
gtgctgcatggtcaggtcgaaagcattgatgttattcgttctatcttctttggtttgctgattacgccgtgggcggtctactttctatcggtggtcgtcgag
caactggaggagtcacgacaacgtctgtcacggctggtgcaaaaactggaggagatgcgcgagcgcgatttgagcctcaacgttcagttaa
aagataatattgcccagctaaatcaggaaattgccgttcgtgaaaaagcggaagcagaactgcaggaaaccttcggccaactgaaaattga
aatcaaagagcgcgaagagacacaaattcagctcgagcagcaatcctcattcttacgttccttccttgatgcttcacccgacctggtttttatcgt
aacgaagataaagagttttccggctgtaaccgcgcgatggagctgctgaccggaaaaagcgaaaaacaactggttcacctgaaacctgctg
atgtttactcaccggaagccgccgcaaaagtcattgaaaccgatgaaaaagtgttccgtcataatgtgtcactgacctatgaacagtggctgga
ttacccggacgggcgcaaagcctgctttgaaatccgtaaagtgccgtactacgaccgcgtgggtaaacgtcacggtttgatgggctttggtcgc
gacattaccgagcgtaagcggtatcaggatgcgcttgaacgggccagccgcgacaaaacgacgtttatctccaccatcagtcacgaattgcg
tacaccgctgaacggtatcgtcggtctgagccgcattctgctggataccgaactcaccgccgagcaggaaaaatatctcaagaccatccatgt
ttcggccgtcacgctggggaatatctttaacgatattatcgacatggataagatggaacggcgcaaggtccagcttgataatcaaccggttgattt
caccagcttccttgccgatctggaaaatctctccgcattgcaggcgcaacaaaaaggattgcgctttaacctggagccgacgctgccattaccg
catcaggtcattaccgacgggacgcgtttacggcagatcctgtggaacctcatcagtaacgccgtcaaattcacccagcaaggccaggttacc
gtgcgcgtgcgctacgatgaaggcgatatgctgcattttgaagtggaagactctggtatcggcattccgcaggatgagctggataaaattttcgc
catgtattaccaggtgaaagacagtcatggcggtaaacctgccaccggcaccggtattggtctggccgtttctcgtcgtctggcgaaaaatatg
ggcggcgatattacggttaccagcgaacagggcaaaggttcaacctttacgttgacgatccacgcaccgtcggtagcagaagaggtcgatg
atgcgtttgatgaagacgatatgcctttaccggcgctgaatgtgctgctggtggaagacattgaactgaacgtgattgttgcgcgttctgtgctgga
aaaattaggtaacagcgttgatgtcgccatgaccggcaaggcggcgctggagatgtttaaaccgggcgaatacgacctggtgttgctggatat
tcagttgccagatatgaccgggctggatatctctcgtgaactgacgaaacgttatccgcgcgaggatttaccgccgctggtggccttaaccgcta
acgtgctgaaagacaaacaagagtacctcaatgctggaatggatgatgtgctgagtaagccgctttctgttccggcgctaaccgcgatgatca
agaaattctgggatacccaggatgatgaggagagtacggtgacgacagaagagaacagtaaatcagaagcattgctcgatattcccatgct
ggaacagtatctcgaacttgtaggaccgaagctgatcaccgacgggttagcggtgtttgagaagatgatgccgggctatgtcagcgtgctgga
gtcgaatctgacggcgcaggataaaaaaggcattgttgaggaaggacataaaattaaaggtgcggcggggtcagtggggttacgccatctg
caacagctgggtcagcaaattcagtctcctgaccttccggcctgggaagataacgtcggtgaatggattgaagagatgaaagaagagtggc
gtcacgacgtagaagtgctgaaagcgtgggtggcaaaagccactaaaaaatgaccccggctagaccggggtgcgcgaatactgcgccaa
caccagggaaatcttggctgcgccgtaaattattatgatgagttacaagggcacagcactgtttttcaggccgcacgcagttaagatagcaaaa
cttaaatgatttgttacatgaatcagttaaatgtgtgatgcgtaccattttattaaaaaaattaatgtgtttcagtcaattgcgaaaggatgatcacaa
tgaagaaaattggcgtaattctgagcggatgcggcgtctatgacggttctgaaattcatgaagcggtgttgacgttgttagctatttcacgcagcg
gtgcgcaggcggtctgctttgcaccggataagcagcaggttgatgttatcaaccatttaactggcgaagcgatgacggaaacgcgcaatgtgc
tgattgaagcggcacgaataacgcgcggtgaaatccgtcctctggcccaggccgatgccgctgaactggatgcgttgattgtgccgggggg
tttggcgcggcgaagaatttaagcaattttgccagtcttggtagcgaatgcaccgttgaccgtgaattaaaggcgctggcacaagcgatgcatc
aggccggaaaaccgcttggttttatgtgtattgccccggcgatgctgccgaaaattttcgatttcccgctgcgtttgaccatcggtactgatatcgat
accgcagaagtgctggaagagatgggcgcggagcatgtgccgtgtcctgtcgatgatatcgtggttgatgaagacaataagattgtcaccacc
ccagcatatatgctggcgcagaacattgcagaagcggcgagcggcattgataagctggtttcccgcgtgctggttctggctgaatgagtaaaa
gccgcttaacggtgtttagtttcgttcgccgttttcttttgcggttaatggttgtcctcgccgttttctggggcgggggcatcgcgttgtttagcgttgcgcc
tgttcccttctcagcggtaatggtcgagcgacaggtcagcgcctggctgcatggcaattttcgttacgtggcacattctgactgggtcagtatggat
caaatctcgccgtggatgggactggcggtgattgccgcagaagatcagaaatttcctgagcactggggctttgatgtcgcttccattgagaaag
ccctggcgcacaacgagcgcaatgaaaaccgtattcgcggtgcttcaacgatttctcaacagacagccaaaaatctctttttatgggatgggcg
tagctgggttcgaaaagggctggaagccggattaacgctggggatagaaacggtctggagcaaaaagcgtatcctgacggtttacctgaata
tcgccgaatttggcgacggtgtgtttggcgtcgaagctgcggcacaacgttatttccacaaacccgcgagcaaacttacccggtcggaagctg
cattactggcagctgtattacctaatccacttcgtttcaaagtctcctcgccatcgggctacgtgcgtagccgtcaggcgtggattttacggcagat
gtatcagttaggcggtgagccgtttatgcaacaacaccagctggattaagcttcgcgcgaagtgagggccagcgttttgctctcacgaggttgc
agatctttctgcagggcaatagccagcgccggttgggcgataaatcgtttccataaccgctcttgcttacgcagacaacaccacttcgaccagg
tcgccagtggaatcaatgtgctttcaccaatgttgtcgcagacgaccgggataacctctgactcactgatgcggtgacagaggatattctgcggc
ttcagcgacatcgtcacgatacggttatcctgcaaataacgcttcagctgtttcagtagctggcgcagttgtgcaatgtcttcttcgtaacggcattgt
tcagcaaattcggtcagcgtaatggaaggcttgccgtcaaaatctgcaatcacatcgtagacataaccggttccgcaatccgtctcaacggtac
cgtgatagcgcggtattccactccagtcttttaaccggcgaccaagatgcgcgtagtactttaactcacggcggatctctttatcgccaccatcgc
cacgatggtagacaatcttgatacagcgttgggcatcttccggatgcgcataacatttccgatgtcttccggtgcccaggggactttgttcagata

agcgaatcatgccatctcctggaaatattttctgacgatctccagtatggtcgattaacttaaacaaaacctaaacaatgcctcttaaggttttctta
aggttcttctgaaagtgaaaagcgcacaaaatgtacaaatgacgttgattgagaacgatttcagtacgtgattttcttattgtcggggggagtttga
agggagttgtatgtcaaagtgatgaagattaatcttcatcaaaaccagaattaaagagggcgataacggcggccagtgcttcctcttcctgtgg
accggtcgcttcaacttcaatctgccgtcctttggcagaatccaacatcagcagcgcaataacgctgttggcttcagcctcggtgccttcgtcatta
cgtaagagcacttcagcgtcaaaaccctgcattaattcaaacagcttcattgcaggccgggcatgcatgcccagcttgtttgtgatttcaacagttt
gcttgacggtcatggtttacgtttttccagcgtacgatggcgtgactggacgtttttaccgcgcgagcggaagtagtctgccagttgctctgcaatat
acaccgaacggtgcttcccgccggtacaaccaatggcgaccgtcaagtagctacggttgttggtttccagcataggtaaccatagctcaagat
agcttcgcgtctggtagataaaattgtgtacttctgtgtggcggtcgaggaacgcggcgacaggtttatcaagacctgtcattggacgcagtttcg
gatcccagtgcgggttcggcaagaagcgcacgtcaaagacgtaatctgcatcgatagggataccgtgtttgaagccgaaagactcaaagac
catggtcagttcgcgttcacgtttacccagcagacgggtacgcagcatttcagccagctcgtgaacggacatttctgaggtatcgacaatcagat
ccgctcgcgaacgcagaggctccagcaaatcgctttctttgtcgatagcactttccagcgacaggtttttgctggaaagcggatgcagtcggcg
cgtgtcactgtaacgacgaattaaggtattacggtcggcatccaggaacaggagttgcggtgagaaagcgtcaggcaggttactcatcgcctg
ttcgaatatttctggtgactccggcatattacgaacatcaatgctgacggcggcagaaatctcgcgatcggccagggttcgagccagatcgggt
aacaacactacgggaaggttatccacgcagtaaaaacccatatcttccagcgcacgcagggcgacagatttacctgaacctgaacgtccgc
tgacgatcatcagtaccatgtactgtttctcctcacaacgtctaaaagagacattaccgaataactacgcttcatccggagtaccttcggtatccgt
aatgatttgatacagctcttcatcgctctgggctgcgcgtaaacggcggcagatggttttgtccgccagacgtttcgccaccagcgacagagtat
gcaggtgcgttttagtttggtctgccggcaccagcagggcaaaaagtaagtccaccggttggttgtcgatggcatcgaaagctataggcgtttcg
agctgaacgaaaacgccaacggcgcgcagagtatcttcttccagtttgccatgcggaatggcaataccattgccgataccggtactgcccattt
tttcccgcgtcaggatagcttcaaaaaccacctgaggcggcaggctaagttgtttcgccgccagctcgctgatgatttccagggcgcgtttttttgct
ctgacagtggacgcggcttcgcgtacattccctgttaagaacactgctaagctgtagagttgtatcattatttgtcataatttcacctaagaacctgc
ccactcaaacagtggaacaggctcggagcattaaccgtacaaatggcccgttgtgccgcacaacagaccgccatgcacatgctaattgccc
ggacaattagtgttgtttcagtttatctttatgtttggtcaactggcgtgccagcttatcaattaagccatcaatggcagcgtacatgtcctgaccttcc
gcgctggcatgaatttcaccaccgtttacatgcagtgttgcatctgaggtgtgggtgactttctccactttcagaacaacatagacctggttgattcg
gtcaaaatattgctcaagtttggcaaatttggctgtaacaaattcgcgcagtgcctcggtgatctcgacgttatttccggtaatgttgagctgcatagt
gtcttccttatcggttgggtcaaacgagttgtttacgctggtttgacggcggaatggataaagactctcggtacttcgcaacagtgcggcgtgcca
ccatgataccttgttccgacagcaaagaggttaacttgctgtcgctcaacggtttcgctgggttttccgccgcgattaatttcttcaccagcgcacg
aatcgccgtggaggaagcttcgccgccgccctcggtattgacgtgactggaaaagaaatacttcagttcaaaaatgcctcgtggactatgcag
gtatttttgcgtggtcacgcgagatatcgtcgattcatgcatttcgacagcctgggcgatatcggccagtaccatcggtttcatatattcttcaccttgc
tcaaagaaggcttgctgctgttcaacgatacagcgactcacgcgcagtagcgtatcgttacggctttccagactcttgatcaaccatttggcatcc
tgcagattgctgcggataaactggctgtcaccatcgttgcgcgcgttattgcacatcgaggcgtagtgctggttgatttgcagacgcggaatgctg
tcactgttgagttctaccgtccagtgaccgttatgcttacgcaccagcacatctggaatgacatactcaggttcgccagtctggatcgactgcccg
gggcgcggatcgagcgactggatcagattgacggcttctttcagcacatcttctttcagacgcgtgacgcgcattaaagtgcggaagtcgtgatt
ggctaacagatcgagatgatcgctaatgatcagtctggcctcttccagccacggcgtggtcttatcgaattgggagagttggatcagcagacag
tcacgcagatcttttgccgccacaccgaccggatcaaaccgttggatccgcttaaggacggcttcaacttcgtcgatgtcgatctcttcatcgcct
atactttcgagaatatcttccagcgggacagtcagataaccggtttcatcaacggcatcgacgatagaggtagcaatagcgcggtcagtgtcg
gaaaacggtgtcagctcaacctgccacatcaggtaatcctgcaaggtctgcgtcgtttcgccctggtagaccggcagctcgtcgtcaatgtagt
caccgctggtgccggatggtgtaccagcggtgtaaatggtgtcccaactggcatcgagcggcagctcttccggcatctctttttgttcgagcgcgt
cggcggtgtccagcgtttcactgtcttgcgtttcgcgggtgtcgatttcttcatgagtgtcgatttgctcaagcagcggattactctccagcgcctgct
gtagctcctgctgaagttccagcgtcgacaactgcaacagacgaattgcctgttggagctgtggcgtcatcgccagttgttggctaagcctgagtt
gcaaaccttgcttcatgttcagaatcgtactctcctgctaaaacgtcgcaaacttctaccctatcagagtctgaagtcttccccaaggtatacacgc
ttaacgtgttcgtcttgtaagatttctgtaggcgtgccgtgggcgatcaaatgcccctgactgacgatataagcgcgttcacaaaccgccagtgttt
cacgcacgttgtggtcagtgatcagcacgcccaggccgctgtcgcgcaggtgctcaatgatgcgtttaatgtcgataaccgagatcgggtcaa
ccccggcaaacggttcgtcgagcagaataaatttcggattcgcagccagtgcgcgggcaatttctacacggcgacgttcacccccggagagt
gactgccccatgctgtcacgcaggtgctcaatgtgaaactcttccatcagctcgttcgcgcggtcttcacgttgttcagcagacaagtcgtcacga
atttgcagtaccgccatcaggttatcgtaaacgctgaggcgacggaaaatggaggcttcctgtggcagatagccgataccgcggcgcgcgcg
tgcatgcagaggcagcagactgatatcgtcatcatcaataatgatgttgcccgcatcgcgcggcacaatgcctacaaccatgtagaaagtggt
ggtcttaccggcaccgtttggccccagcagaccgacaatttccccggagttgacggtcaggctgacgtcttctaccacgcgacggcctttatag
gcttttgcaaggttctttgcagttaatgttgccataacgaattaattacccttcttctgtgccggggtctggcctttgttgtttttgtcctgcagctgcgacg
gcaccagaacggttgttacgcgcttgcctttgtcgctgaaagcctgcattttctgctctttcaccaggtaagtgatcttatcgcccttaatgttgctatc
gacctgctgcagataagcattacccgtcagaacgacaaaatcttttgccagttcgtagtgcatctgggaagcgtgaccttcaacgggtttaccgtt

gtcctgcatctggtagaacgttgccggtttaccgtagccgtcaatcacttctttaccttgttcgccgcccggacgggtaacgaccactttgtcggcat
taattttgatggtgccctgggtgacgatgacattaccggtaaaggtaaccacgttgccttgcatatcaagagattgctggtccgattcaatgtggat
cggctgatcagtgtctccggttacggcaaatgccggaatgctggcggccagaagtgagctggcaagcacaagattaaggctgagtttgtttgttt
tgaatttcataggatgttctaaccttttcaatcagctcggcgttcttgctgcgtaagttgccgcgcattttcagaccgctggagttaaatgttgttccgta
taacgtgacgaggtcttcagaggtaacatcctgcgtcaccagattgatctgcgcgttatccgtcgtgattctgcgaagttgagagtctggcacaa
gtgcgttgacttcaacgtgtccatataaatagagcatccggtcattggtcagcttggctttatctgcttttacggaccatgtcgggattttatccttatca
aacgtggtaagtaccggctgcgtaaaccacgaaacggcctgatcggaataatattcaacgtgttgagcaatcaatcgatagcttagtgcccctt
ctgggttatagacgagcgtgtccgtatgctcgcttttataggtgggatcattgttgttgacgaccacctgggcggtatcgtctttttcggccatattaat
gccgatcatcaccagaaccgccagtgatagcacaatgataacccaacgtctggctttactcatatcgattgccctttggcttcatccagtttgccct
gcgccaggagtaataagtcgcaaacttcgcgcactgcgccacgaccgccagcaatgcgcgtcacgtaatcggcgcgcgggatcaacagtg
gatgcgcatcggccacggcgacgcttaaacccactttttccattaccggccagtcgatgagatcatcgccgacataagccacattttccggggc
aatcgccagtttttccagcagatcgctaaaggcgatcagtttgtttgactgcccctgatacaagtgagtgatccccaatgtggcacaacgatcttct
accagtttagcctttcgcccggtaatgatagcgacttcaatatcagaggtgagcgcacaacgaatgccataaccgtcacgaacattgaacgctt
tcagctcttcgccattattgcccatataaatcaggccatctgacagtacgccatcgacatcgaggatcagcagacgaatgttctctgcttttgctat
aacgtcggcgctgacagggccgtaacaggtcgcaagcgacgcacctgctttgctcattgttgtttatccttgaatctttacactacgcctgcacgc
agtaaatcatgcatatgtaacacaccgagtaaatggtcgccatcggcaaccatcacggaggtgatatggcgggactgcattaagttcagtgcc
tcaacggccagaatgccagggcgcacacgtattcccccggcgtcatcacatcggcaatacttaactgacgaacatccacgcccatatcga
agacacggcgtaaatcaccgtcggtaaagatgccttcaatcatcatattgtcatcgcaaatgacagtcataccaagatttttgcgggtaacttcc
agcaacgcgtcacgcagactggccgttttcttaacatgcgggatctcatcgcccgtatgcataatatcgtttacgcgcagcagaagtttacgacc
cagtgcgccgcctgggtgtgagagcgcaaaatcttcagcagtaaagccgcgtgcttttaacagcgcgacagcgagggcatcgcccataacc
agcgtggcggtggtgctgctggtcggtgccagccctaacggacaggcttctttcgctactttaacacacagatgcacatctgcggcgcgcgcca
tgctgctctccgggcgaccggtgatgcagattaacggtacgtgaagacgcttaagcactggaattaaggccgtgatttcgctggattcaccaga
gttagagatagcaatcaccacatcctgtggggtaaccatgcctaaatcaccatgcgcggcttcaccaggatggacgaaaaatgaaggtgtac
cggtgctggcaaacgttgccgccatttttcgcccaatatgccccgattttcccatccccatgacgacaactttccctttacaccagaacatcttttca
caggcaagcgtgaaattctgattgatgtattgatcaagctccgccaggcattcacgttcaatcgccaggacttctttacctgcttgctgaaagtcaa
aacccggttgtaactctacgtgcgacataatgcgtttccagttattcaacgagtattggcgataaccagtacaacatcgccagccatacgataaa
tccgccagttaataataccccctacaccacggcccggttgcggggagcgccgccagcacagcaacgcaaaaataatgctcaccagcaacat
cacgctgtagtcacgactgtacgccagtggatcaatctctcctggcgttatcagcgcgggtaaacccaacacgatgacaatattaaaaatgtttg
cgccaatgatatttccgacagcaatgtcgttttcacctttgcgaacccccgctattgcggttgccagttccggcaggctggttccgatagcaattgc
cgtcagacccatcgtcaactcgctgatggcaaagtaattcgccagcaccgtggcgttatcaaccaccatccgcgtggccactggcatgatgat
aagcgcaatgccgagccataaaaacgcgacgggcaatccgccgtcacgcggcagctctgcaagctgctctctggtcaggctgtcagtcccc
tgacgttcagcctgacgtgcaagtttaacaatgaacagcagccatagcacagccagaaagaggagaaagataccatcgctgcgactaagtt
gtccgtcatagagtacggaaccggccaccacgctgaccaacaacattaagggtaattcacggcgtagaacatcagaatggacggtaaaag
gacgaaccagcgcagccagaccgaggatcagcaatatattgataatgtttgagccgagggctgtaccaacggctaaatcgcgttgttcgtgc
agagacgcggcaagcgagacgatgacttctggtaacgatgtaccaatactgaccaccgtcatgccgatgatcagcggcgggatgccaaag
gttcggcaaagaatagacgcggcaaaaaccaggcggtcggcactgtaaacgaccaaaagtaaaccaacaattaacagtgccgtagctaa
aagcatctaacgtcctttcttcaggtatactcgccggtccgctgaagattttcagaaagccgtaacggatgcttaattttgactttatgcggctaaaa
agtaaaacaaatgccagctttcgctaaccacggcgggtaatattctgtaaatatgttgggttcaaggttaaattgagcgccatgcttagaaaatc
aacgcaagacgaagggtgaattatggagcagtctgtggcgaatttagtcgatatgcgcgatgtcagttttacgcgtggcaatcgctgcatcttcg
ataatatttccctgaccgtgccgcgagggaagatcacggcgatcatggggccatcgggcatcggtaaaacgacgctactccgtctgattggcg
ggcaaatcgcaccagatcatggtgagatccttttcgatggtgagaatattccggcgatgtctcgttcgcgcctgtatacagtgcgcaaacggatg
agcatgttatttcagtccggggcgttgttcactgatatgaacgtatttgacaacgtcgcctatccactgcgcgaacatacccaacttcccgcgccat
tgttgcatagtacggtgatgatgaagctggaggccgtggggctgcgtggagcggctaaactaatgccttctgaactttccggtgggatggcgcg
gcgtgcagcgctggcgcgtgcgattgcgctggagccggatctcatcatgtttgatgaaccttttgttgggcaagatcccatcaccatgggcgtact
ggtgaagctgatttctgagctgaacagcgcgctgggcgtgacttgtgtggtggtttctcacgatgtgccggaagtgttaagtattgcggatcacgc
ctggatcctggcggacaaaaaaattgtcgctcatggcagtgcccaggcgttgcaggcgaatcctgatccgcgcgtacgtcagtttctggacgg
gatagctgacgggcctgttccgttccgctatcctgccggcgattatcacgctgatcttttaccagggagttaagccactcatgctgttaaatgcgct
ggcgtcgctcggacataaagggattaaaaccctgagaacgttcgggcgggccgggttaatgttattcaatgcgctggtcggcaaaccggaatt
tcgcaaacatgcgccgctgctggtgcgccagctctataatgtcggcgtcctgtcgatgctgattattgtggtttctggcgtgttcatcggaatggtgtt
ggggctgcaaggttatctggttctgaccacttatagtgcggaaaccagtctgggtatgctggtggcgttatcgctactgcgtgaactggggccggt

ggttgccgcgttgttgtttgccgggcgtgctggttcggcgctaaccgcagaaatcggcctgatgcgcgctacagagcaactctccagtatggag
atgatggcggtggatccgctgcgtcgggttatttctccccgtttctgggctggggttatttcattaccactgttgacggttattttcgtcgccgtggggat
ctggggcggatctctggtcggcgtcagttggaaaggcattgatagcgggttcttctggtcggcaatgcaaaatgccgtcgactggcgtatggatc
tggtcaactgtctgattaagagcgtggtgttcgccatcacggtgacgtggatttcgttgtttaacggctacgacgccatcccgacgtctgccgggat
tagccgggcaaccactcgcaccgttgtccactcgtctctggctgttctggggctggattttgtgctgaccgcattgatgtttgggaattgagttcatgc
aaacgaaaaaaaatgaaatttgggtgggtatctttttattagcagcactgctggcggcgctgtttgtttgcctgaaggcggcgaacgtgacgtcc
atacgtactgaaccgacctacacgctttatgcgacgttcgataacattggcggcctgaaagcgcgctctccggtcagtattggtggcgttgttgtg
ggtcgggtggcggatattacgctggacccgaaaacctatctgccgcgcgtaacgctggaaattgaacaacgttataaccacattccagatacc
agttcgctgagcattcgtacttccggcctgctgggggaacaatatctggcattaaacgtcggttttgaagacccggaactggggactgctatcct
gaaggatggcgatacaattcaggacactaagtctgcgatggtgctggaagatctcattggtcagttcctttacggtagtaaaggcgatgacaat
aagaatagtggcgatgcgccagctgctgcgccaggtaataatgaaaccactgaacctgtgggtacaacgaaataatttcaggagaaccgac
gcatgtttaaacgtttaatgatggtcgctttgctggtgattgcacctctgagtgcggcaaccgcggcagaccagaccaatccgtataagctgatg
gacgaggcggcgcagaaaacgttcgatcgcctgaagaatgagcaaccgcaaattcgggccaacccggattatctgcgtaccattgttgatca
ggaactgctgccatacgtacaggtgaaatacgccggtgcgctggtgctgggccagtattacaagagtgcgacccctgctcaacgtgaagcct
actttgccgctttccgtgagtacctgaagcaggcttacggtcaggcgctggcgatgtatcacggtcaaacctatcagattgcgccagaacagcc
gctgggcgataaaaccattgtgcctattcgcgttaccattattgacccgaatggccgtccgccggtgcgtctggacttccagtggcgtaaaaact
cccagacgggcaattggcaggcttacgacatgattgctgaaggcgtcagtatgatcaccaccaaacaaaacgagtggggaacgctgctgc
gtaccaaaggtatcgacggcctgactgcgcaactgaaatcgatttctcaacagaaaatcactctggaagagaaaaaataatgagcgagtca
ctgagctggatgcagacgggtgacacgctggcgttatccggagagctggatcaggacgttttgctaccgctttgggaaatgcgtgaggaagcg
gtgaaggggattacctgcatcgatcttagccgtgtctcccgcgtggatacgggggactggcactgctgctccatcttatcgatctggcgaaaaa
gcagggcaacaatgtgacgcttcaggggggtaaacgacaaagtgtataccctggcgaaattgtataatttgcctgctgatgttctgcctcgttaaat
tttttcaggccggatatggcgttaaccgcctgtccggcaaacctgcctgcaagtcgagccttgtctgatttatcagcgaggctttttgcttatttatgcc
agcgcaactttgctctaagatgtttcgctggtttattaactgatgattgaagatcccatggaaaataatgaaattcagagcgtgttgatgaacgctct
ctccctccaggaagtccacgtttccggcgatggcagccactttcaggttattgccgtgggtgagttgtttgacggcatgagtcgggttaaaaaac
agcagacggtctatggtccgctgatggaatatattgcggataaccgcattcatgctgtgtcgatcaaagcgtataccccctgcggagtgggcgcg
cgatcgcaaactgaacggcttttgagctatgggcgattcgccggtagcggatatgaattgttaactgagaacaaactaaatggataaatttcgtg
ttcaggggccaacgaagctccagggcgaagtcacaatttccggcgctaaaaatgctgctctgcctatcctttttgccgcactactggcggaaga
accggtagagatccagaacgtcccgaaactgaaagacgtcgatacatcaatgaagctgctaagccagctgggtgcgaaagtagaacgtaa
tggttctgtgcatattgatgcccgcgacgttaatgtattctgcgcaccttacgatctggttaaaaccatgcgtgcttctatctgggcgctggggccgct
ggtagcgcgctttggtcaggggcaagtttcactacctggcggttgtacgatcggtgcgcgtccggttgatctacacatttctggcctcgaacaatta
ggcgcgaccatcaaactggaagaaggttacgttaaagcttccgtcgatggtcgtttgaaaggtgcacatatcgtgatggataaagtcagcgttg
gcgcaacggtgaccatcatgtgtgctgcaaccctggcggaaggcaccacgattattgaaaacgcagcgcgtgaaccggaaatcgtcgatac
cgcgaacttcctgattacgctgggtgcgaaaattagcggtcagggcaccgatcgtatcgtcatcgaaggtgtggaacgtttaggcggcggtgtc
tatcgcgttctgccggatcgtatcgaaaccggtactttcctggtggcggcggcgatttctcgcggcaaaattatctgccgtaacgcgcagccaga
tactctcgacgccgtgctggcgaaactgcgtgacgctggagcggacatcgaagtcggcgaagactggattagcctggatatgcatggcaaa
cgtccgaaggctgttaacgtacgtaccgcgccgcatccggcattcccgaccgatatgcaggcccagttcacgctgttgaacctggtggcagaa
gggaccgggtttatcaccgaaacggtctttgaaaaccgctttatgcatgtgccagagctgagccgtatgggcgcgcacgccgaaatcgaaag
caataccgttatttgtcacggtgttgaaaaactttctggcgcacaggttatggcaaccgatctgcgtgcatcagcaagcctggtgctggctggctg
tattgcggaagggacgacggtggttgatcgtatttatcacatcgatcgtggctacgaacgcattgaagacaaactgcgcgctttaggtgcaaat
attgagcgtgtgaaaggcgaataatcgtctgagagctggcagccccgttcgcggggctaccgccagaccactcatttttcggtttagtgtagct
acgcatcaactgcgttctgtcgataaactcatgggtctgcggatcatggtagcgtgatggccagataacccaggggtcagttcccagggctttcg
caataatcatctctcctttcggccatgggcgcgataatgcattcgccagcgttgaggaactcaaaccatttctgcgagattccgccgccattgacg
ttcccttcttgcgcaaacccgcaatgatgtcagcgggatgccagtcaatgaaattactttccatttttcctcactccttaagttagtcgctttcattcag
aacacttcttctgtcgagcatcactatactcctgatggcctattgctctaaaaagttcctgtaaagagtgtgaatattcagaatatataaagatgcac
atccctatttttcaggtgttatagagctgaataagtgtttttatggaacttactggaaccatcccgcgcgctgcgcgggatgaggggaggggatta
acgatcgcgttgaacagcgatgtgcgcgaggccgatgagtgcttctcgccaaggggtgtccgggagcacctgtaacgctgcgatggctttgtct
gcttcttcctcggcacgctgacgcgtccattcaagagatccacaagcgttcattgcttccagaaccggttccagaagatggcgaccgttaccctg
ttcgatggcggtacggatcatctgtgcctgttctggtgtgccatgatgcatcgcatgcagcagcggcagcgtcggtttaccttcgttcagatcgtcg
ccgacatttttacctaactgttcgccatcggcattgtaatcgagtaaatcgtcgatcaactggaaagcagtgccgagatagcgcccataatcctg
caggcctttctcctcctccggcgtacagccagccagaatcccggaacactgcgcggcagcctcaaacagacgcgcggttttgctatagataa

cgcgcatgtagttttcttcagtgatgtccggatcgttaacgttcatcagttgcagaacttcaccttctgcgatgacgtttacggcttctgacatgacttc
cagcactttgagcgaaccgaggctggtcatcatctggaaagcgcgggtataaataaaatcgcctaccagcacgctggcggcattgccaaatg
cggcgttggcggtagctttacccctgcgcatatctgattcatccacaacgtcgtcgtgtagcagagtcgccgtgtggataaactcgatcagggca
gcaatggtgacatgcgcatttccctcatagccaacagctcgtgcagccagtacagcaatcatcggacgaatacgtttaccgccgccgctgacg
atgtaatagcctaactgattgatcagttggacgtcggaattaagctgctcaaggattgccgcattaacacccgccatatcttgcgcggttaactca
ttgatttttctaaattcatcgcaaaagccgggcttttcgccccgattaccttactgtgaacaaaggcaacttagggtttatggttaggaatatagggt
gattgtactgaaaaaatggcacagataaacgttaccgtacaagttgtgtttttttcttcgtgtattgactgtagcacttgtcaaaggcgtgcgttttgc
gtaatattcgcgccctattgtgaatatttatagcgcactctgaatcattgaaaaggtgtgcgcggaagcggagttttatatgtacgcggttttccaaa
gtggtggtaaacaacaccgagtaagcgaaggtcagaccgttcgcctggaaaagctggacatcgcaactggcgaaactgttgagttcgctga
agtgctgatgatcgcaaacggtgaagaagtcaaaatcggcgttcctttcgttgatggcggcgtaatcaaagctgaagttgttgctcacggtcgtg
gcgagaaagttaaaatcgttaagtttcgtcgtcgtaaacactatcgtaagcagcagggccatcgtcagtggttcactgatgtgaaaattactggc
atcagcgcctaagacctgaggagagatttcaaatggcacataaaaaggctggcggctccacacgtaacggtcgcgattcagaagctaaac
gcctgggcgttaagcgtttcggtggcgaatccgttctggcgggtagcatcatcgttcgtcaacgtggtaccaaattccacgctggcgctaacgta
ggttgcggtcgtgaccacactctgtttgctaaagcagacggtaaagtgaaattcgaagttaaaggcccgaaaaaccgtaaatttatcagcatcg
aagctgaataagtttttcgcgtcccggtaacggatgaaagccccgcaacacgttgcggggctttttacattcgataaccggtaaaaatgcgcat
ggtgcgacgttaatccgcaaatgcgctttgcagggaatacggtatgaagcagcaggcaggcattggcattcttttggcgctcaccacagcaatt
tgctgggggcgttgccaatcgcaatgaagcaggtgctggaggtgatggaacctccgacaatcgtgttttaccgtttcttgatggcgagtattggc
ctgggtgccattcttgcggtgaagaagaggttgccgccattacgcgtgtttcgtaagccacgctggttgattttgttggcagtggcgaccgccggg
ctgtttgggaacttcatcctgttcagctcatccttgcaatacctgagtccgaccgcttcgcaggtgattgggcaactctcgccagttggcatgatggt
tgccagcgtatttatcctcaaagagaaaatgcgcagcactcaggttgtaggggcattgatgctcctgagcggcctggtgatgtttttaacaccag
tctggtcgagatatttacaaagctcaccgattacacctggggagttatctttggggtcggtgcggcgacggtttgggtgagttatggcgtggcgca
aaaggttttattgcgtcggctggcctcaccgcagatcctgtttttactgtacactttatgtacaattgcgctcttccctctggcaaagcctggagtgata
gcgcagcttagccactggcagctcgcatgtttaatttttgcggactgaataccttggtaggatatggcgccctggcggaagcgatggctcgctg
gcaggcagcgcaggtgagcgcgatcatcacgctcaccccactgtttacgctgtttttttcagatcttttatcactggcctggcccgatttcttcgcca
gaccgatgttaaaccttttaggttatctcggtgcgtttgtcgtggttgcgggcgcgatgtattccgccattggtcatcgtatttggggcggattacgtaa
gcatacaacggtggtatcgcaaccccgcgcaggcgaatgatttacggagaataaaatgaagtttgttgatgaagcatcgattctggtcgttgca
ggtgatggcggtaatggttgcgtgagcttccgccgcgaaaagtatattccgaaaggcggcccggatggcggcgacggcggtgatggtggtg
acgtatggatggaagccgacgagaacctgaacacgcttatcgattatcgttttgaaaaatctttccgtgcagagcgcggtcagaatggcgcaa
gccgcgactgtaccggtaagcgcggtaaagacgtgacgattaaagtgccggtaggtacgcgtgtaatcgaccagggtactggtgaaaccat
gggcgatatgaccaaacacggtcagcgtctgctggttgctaagggcggctggcacggtctgggcaatacccgtttcaaatcgtccgttaaccgt
acaccgcggcagaaaaccaacggcacgccgggcgataagcgcgagctgctgctggagctgatgctgctggctgacgtcggtatgttgggg
atgccaaacgcgggtaaatcgacctttattcgtgcggtatcggcggctaaaccgaaagtggcggattatccgtttaccactctggtgccaagtct
gggtgtggtacgaatggacaacgaaaagagcttcgttgttgccgatattccaggactgattgaaggcgctgcggaaggcgcaggtctgggca
ttcgcttcctgaagcacctggaacgttgccgcgtcctgttgcacctcatcgatatcgatccgattgacggcaccgatccggttgaaaacgcgcgt
attattatcagcgagctggaaaaatacagccaggatctggcgacgaaaccgcgttggttagtgttcaacaagatcgatctgctggataaggta
gaagccgaagagaaagcgaaagcgatcgctgaggcgctgggctgggaagataaatattatctgatctctgcggcgagtggactgggcgtg
aaagatctctgctgggatgtgatgacctttatcattgaaaacccggtcgtgcaggctgaagaagcgaaacagccagagaaagtcgaattcat
gtgggatgattatcatcgccagcagcttgaagagattgctgaagaggatgatgaagactgggatgacgactgggacgaagacgacgaaga
aggcgttgagttcatttacaagcgttaatcactgatgataaacgccaaatcagcctgatgcgctacgcttatcaggcctacaagagaattgcaat
atgttgaatttgcacgattttgtaggccggataaggcgtttacgccgcatccggcataaacaaagcgcactttgtcaataatctgaagccccggc
catgtgccggggtttctttttgactaattgttctgataaatatctttatacaaacggctttcaaaacgcactaacggaatacggcgattacgctgatcc
gcaggttctactgcatagccagaaagatattgcacaaacgccattcgttgcccgctcgctgtggtaatgaatcccgccaggttatataccccctg
caacgaaccggttttcgctgagacttttccatccacgcccgcctgatgcagacctgcacggtactgcaaagagccgtcatagcccgccagcg
gcagcatggagataaagttaagttcattgtcgtgttgggcaatgtattgcagcacctgcatcatggtggcgggggcaatcaggttatgccgcga
aagccctgaaccatcggcaataatggtgtttccaatatcgacaccggcttgctggcgcaggatctgacgcacggcgtccgacccggcccgcc
atgttccaggcacattgaagcgcgcatggcctatcatgcggaaaaccgtatcggcgatcatgttgtccgactttttcagcataatcttaagcagat
cgtgcagcggggccgactgtttactggcaactaccgttccaggttcgttaacctgagtctggcgcagcagtgttccgctccaggtgatacccgcc
tgttttaactcatctttcagaattgcaccggcatagctggctccatcctgcacggcaaaagccaacgggagcggctcagaacgttgtggcaggc
atcccgtcagcgtaaagcggttcaggtcgcctggcaccacatccagttcgcagtattgcgcttcggcagaaccacgggggagggtgcgtacc
tggctgaacatcgtaacggggtaataagatgccacgcgtataaaagccatatcaccaggctttggggcactgtagagcgagacggagaaac

agttgcggtcaactatggcggcggcaggcggagcgctaaagcattgtgtcatgtcattccatggccagccgggggctttatcgtggctggcga
aaatggaggtatctatcaacacattgccatcgatttggttgacgccagattttttcaaagtcgcgaccatattgcgaatatcctgacgttttaacgtc
ggatcggcaccaaatcgcgccactaagtcacccttaagtacgccgttttccacattgcctttggtttcaagcgtcgtggtaaaacgaaaatcggg
gccgagttgaatcaacgccgccagcgcagtaatcactttctgggtactggcaggcagcgccatctgctgactgtggtaatcaatagcgggggc
cgacgcgccgactttttgcaccatcagggcaaggttggcaccagcggggagttgagtaatgtactcatcaacatttgcggcctgaacactgaa
cgctatacagctggtcaatccgatgataaatctggaaaatcgcataatctcgcgctaacaacctggaatcgagccgtcatactacggcgcaac
gccctataaagtaaacgatgacccttcgggaacttcagggtaaaatgactatcaaaatgtgaattgtagctgacctgggacttgtacccgggtc
ggtattttttgcttctggtcccggtaaggagttatgccgggcaggccgaacagccggggtgggtgaagacttgccctatcaggaatattcaaga
ggtataacaaatgcaagctattccgatgaccttacgcggcgctgaaaaattacgcgaagagctggattttctgaaatctgtgcgccgtcctgaa
atcattgctgctatcgcggaagcgcgtgagcatggcgacctgaaagaaaacgccgaataccacgcagctcgtgaacagcagggtttctgcg
aaggccgtattaaagacatcgaagccaagctgtcgaacgcgcaggtgattgatgtcaccaaaatgcccaacaatgggcgcgttatttttggtg
ctaccgtaacggtgctgaatctggattctgacgaagaacagacttatcgcatcgttggcgatgacgaagctgactttaaacaaaacctgatttct
gtaaactcgcctattgctcgtggcctgatcggcaaagaagaagatgatgttgtggtcatcaaaacgccgggcggcgaagtagaatttgaagta
attaaggtggaatacctgtaagaattacccaatactcaagatgttgatgtattgtaaagaaaggaaaaaggccgctatgcggccttttatcaacg
aacagagcgtggcattttgctctcctgcctgcggaaaacccctcgttttacacagcaaatgtgtgtaactttaggataatcttagcgtggcagcga
gattttacgttctttagttgggcgataaagcaccagcgttttaccgatgacctgtacattacaggcgccggtttcgcgcacgatagcttccacgatc
aaggttttagtttcgcgatcttcggtggcgattttcaccttgatgagttcatggtgctctaacgcttgttcaatctcggccagcacccccttcggtcaaac
cattactgccaagcagaacaactggcttgagcggatgtgccagacctttcaggtgctgtttttgtttagtactcagattcatcgtatttttttgcttacgtt
gggattgaaaacgggtcattctaccgccatctcccatatatcaccaaataggcgcgtaaaaatttacgcaattggttacgatgagttatccccat
gggaaagttaaatgacaggtaagaagcgttctgccagctccagccgctggcttcaggaacactttagcgataaatatgttcaacaggcacag
aaaaaggggttacgttcccgtgcctggtttaaacttgatgaaatacagcaaagtgacaaactctttaaaccgggaatgacggttgtcgaccttg
gtgctgctccgggtggttggtcacaatatgtggtcacccaaattggcggcaaaggccgcatcatcgcttgcgatcttttacctatggatcctatcgtt
ggtgtggactttcttcagggcgattttcgtgatgaactggtgatgaaagcactgctggagcgcgttggcgacagcaaagtccaggttgtcatgtcc
gatatggcaccaaacatgagcggaacaccggcggtggatatcccccgtgccatgtatctggtggaactggcgctagaaatgtgtcgtgatgta
ttagcgccaggtggcagttttgtagtgaaggtgttccagggcgaaggtttcgatgagtatctaagggaaattcgctccctgtttacgaaggtcaaa
gttcgtaagccggactcttctcgtgcacgttcgcgggaagtgtatattgtagcgaccgggcgtaaaccctaaccgggagatttcagacgaaagt
ttgaaagatgctggatatagagtatcctgacgctgtttttaacacagttgtaataagaggttaatcccttgagtgacatggcgaaaaacctaatact
ctggctggtcattgccgttgtgctgatgtcagtattccagagctttgggcccagcgagtctaatggccgtaaggtggattactctaccttcctacaag
aggtcaataacgaccaggttcgtgaagcgcgtatcaacggacgtgaaatcaacgttaccaagaaagatagtaaccgttataccacttacattc
cggttcaggatccgaaattactggataacctgttgaccaagaacgtcaaggttgtcggtgaaccgcctgaagaaccaagcctgctggcttctat
cttcatctcctggttcccgatgctgttgctgattggtgtctggatcttcttcatgcgtcaaatgcagggcggcggtggcaaaggtgccatgtcgtttgg
taagagcaaagcgcgcatgctgacggaagatcagatcaaaacgacctttgctgacgttgcgggctgcgacgaagcaaaagaagaagttgc
tgaactggttgagtatctgcgcgagccgagccgcttccagaaactcggcggtaagatcccgaaaggcgtcttgatggtcggtcctccgggtac
cggtaaaacgctgctggcgaaagcgattgcaggcgaagcgaaagttccgttctttactatctccggttctgacttcgtagaaatgttcgtcggtgt
gggtgcatcccgtgttcgtgacatgttcgaacaggcgaagaaagcggcaccgtgcatcatctttatcgatgaaatcgacgccgtaggccgcca
gcgtggcgctggtctgggcggtggtcacgatgaacgtgaacagactctgaaccagatgctggttgagatggatggcttcgaaggtaacgaag
gtatcatcgttatcgccgcgactaaccgtccggacgttctcgacccggccctgctgcgtcctggccgtttcgaccgtcaggttgtggtcggcttgcc
agatgttcgcggtcgtgagcagatcctgaaagttcacatgcgtcgcgtaccattggcacccgatatcgacgcggcaatcattgcccgtggtact
cctggtttctccggtgctgacctggcgaacctggtgaacgaagcggcactgttcgctgctcgtggcaacaaacgcgttgtgtcgatggttgagttc
gagaaagcgaaagacaaaatcatgatgggtgcggaacgtcgctccatggtgatgacggaagcgcagaaagaatcgacggcttaccacg
aagcgggtcatgcgattatcggtcgcctggtgccggaacacgatccggtgcacaaagtgacgattatcccacgcggtcgtgcgctgggtgtga
ctttcttcttgcctgagggcgacgcaatcagcgccagccgtcagaaactggaaagccagatttctacgctgtacggtggtcgtctggcagaaga
gatcatctacgggccggaacatgtatctaccggtgcgtccaacgatattaaagttgcgaccaacctggcacgtaacatggtgactcagtgggg
cttctctgagaaattgggtccactgctgtacgcggaagaagaaggtgaagtgttcctcggccgtagcgtagcgaaagcgaaacatatgtccga
tgaaactgcacgtatcatcgaccaggaagtgaaagcactgattgagcgtaactataatcgtgcgcgtcagcttctgaccgacaatatggatatt
ctgcatgcgatgaaagatgctctcatgaaatatgagactatcgacgcaccgcagattgatgacctgatggcacgtcgcgatgtacgtccgcca
gcgggctgggaagaaccaggcgcttctaacaattctggcgacaatggtagtccaaaggctcctcgtccggttgatgaaccgcgtacgccgaa
cccgggtaacaccatgtcagagcagttaggcgacaagtaagttcccgcatcagatgactgtatttgtaccgaaaaccccggggcgtgctccg
gggtttttttcttatcaattcataccaggataacatcatgaaactctttgcccagggtacttcactggaccttagccatcctcacgtaatggggatcct
caacgtcacgcctgattccttttcggatggtggcacgcataactcgctgatagatgcggtgaaacatgcgaatctgatgatcaacgctggcgcg

acgatcattgacgttggtggcgagtccacgcgcccaggggcggcggaagttagcgttgaagaagagttgcaacgtgttattcctgtggttgag
gcaattgctcaacgcttcgaagtctggatctcagtcgatacatccaaaccagaagtcatccgtgagtcagcgaaagttggcgctcacattattaa
tgatatccgctccctttccgaacctggcgctctggaggcggctgcagaaaccggtttaccggtttgtctgatgcatatgcagggaaatccaaaaa
ccatgcaggaagctccgaagtatgacgatgtctttgcagaagtgaatcgctactttattgagcaaatagcacgttgcgagcaggcgggtatcgc
aaaagagaaattgttgctcgaccccggattcggtttcggtaaaaatctctcccataactattcattactggcgcgcctggctgaatttcaccatttca
acctgccgctgttggtgggtatgtcacgaaaatcgatgattgggcagctgctgaacgtggggccgtccgagcgcctgagcggtagtctggcct
gtgcggtcattgccgcaatgcaaggcgcgcacatcattcgtgttcatgacgtcaaagaaaccgtagaagcgatgcgggtggtggaagccact
ctgtctgcaaaggaaaacaaacgctatgagtaatcgtaaatatttcggtaccgatgggattcgtggtcgtgtaggggatgcgccgatcacacct
gattttgtgcttaagctgggttgggccgcgggtaaagtgctggcgcgccacggctcccgtaagattattattggtaaagacacgcgtatttctggct
atatgctggagtcagcactggaagcgggtctggcggcagcgggcctttccgcactcttcactggcccgatgccaacaccggccgtggcttatct
gacgcgtaccttccgcgcagaggccggaattgtgatatctgcatcgcataacccgttctacgataatggcattaaattcttctctatcgacggcac
caaactgccggatgcggtagaagaggccatcgaagcggaaatggaaaaggagatcagctgcgttgattcggcagaactgggtaaagcca
gccgtatcgttgatgccgcgggtcgctatatcgagttttgcaaagccacgttcccgaacgaacttagcctcagtgaactgaagattgtggtggatt
gtgcaaacggtgcgacttatcacatcgcgccgaacgtgctgcgcgaactgggggcgaacgttatcgctatcggttgtgagccaaacggtgta
aacatcaatgccgaagtgggggctaccgacgttcgcgcgctccaggctcgtgtgctggctgaaaaagcggatctcggtattgccttcgacggc
gatggcgatcgcgtgattatggttgaccatgaaggcaataaagtcgatggcgatcagatcatgtatatcatcgcgcgtgaaggtcttcgtcagg
gccagctgcgtggtggcgctgtgggtacattgatgagcaacatggggcttgaactggcgctgaaacagttaggaattccatttgcgcgcgcga
aagtgggtgaccgctacgtactggaaaaaatgcaggagaaaggctggcgtatcggtgcagagaattccggtcatgtgatcctgctggataaa
actactaccggtgacggcatcgttgctggcttgcaggtgctggcggcgatggcacgtaaccatatgagcctgcacgacctttgcagcggcatg
aaaatgttcccgcagattctggttaacgtacgttacaccgcaggtagcggcgatccacttgagcatgagtcagttaaagccgtgaccgcagag
gttgaagctgcgctgggcaaccgtggacgcgtgttgctgcgtaaatccggcaccgaaccgttaattcgcgtgatggtggaaggcgaagacga
agcgcaggtgactgaatttgcacaccgcatcgccgatgcagtaaaagccgtttaaagcgttagataactggctaaaaaggcggcgattgtcg
cctttttctcagcgaacacatcggttttgctgtttttttccgcagttgatacaatgcgataaaattgcccttgcgaaggtcattcgctttggttagtattca
cacccgcttcagtgggaaagattaaaaactcccgctttattggttgaagcattggtacgcggcaactccgcaaggaacaggttgattatgtatga
agctcttttagtagttttccttattgtggcaattggccttgttggtctgatcatgctgcagcaaggtaaaggcgctgatatgggagcctccttcggagc
aggcgcttccgctacgctgtttggttcaagtggttctggtaacttcatgacccgcatgacggcgctgctggcaacgttattcttcatcatcagtctggt
gctgggtaacatcaatagcaacaaaaccaataaaggtagcgaatgggaaaatctgagtgcaccggcgaaaaccgaacaaactcagcca
gctgctccggctaagccgaccagcgatatcccgaactaaaaagtagtatccgtgccgaggtggtggaattggtagacacgctaccttgaggt
ggtagtgcccaatagggcttacgggttcaagtcccgtcctcggtaccaaattccagaaaagagacgctgaaaagcgtctttttcgttttggtcct
ggtctgaggaatggttgtcaggtatctgtatctcgtccagttgctttactcgtagctggtaccttgaaacgatggtgccggtacgccttagttataaatt
tttcccttctactgacataattggcactttattgtgtcaactccagttccggtacgaatacggatatgtttacttctcgataaaccatcctgctcctgtca
gtataacgactgatggctacgttctgcgtaatgtttagagacgcattgataatgttatttaattactgagaacgcaatggccgcatcatttaaaaag
aaaaaactaacagtgtttcgttctgatatggatcagagttgaaaaacatgtctgatataaaattaaacattgttgataattgtttatattggttgtgaac
ttggatttctggatgacagtattcaacaaatttgctagaacttttaaatctcattggttgttgtatctttgtgttattgtttttggtattacgaacttagtcgcttc
ttccggagcgcatatggttcagcgcttgctgttcttcgttctgaccatcctggttgtaaaacgtatatcatcccttccgcttcgcctgcttgttgccgcac
catttgtgttactgactgcggcagacatgagtattagcctctattcatggtgtacctttggtacaactttcaatgatggatttgcgattagtgtgctccag
agtgatccggatgaagttgtcaaaatgctggggatgtatatcccttatctatgtgcctttgctttttatcccttcttttttggcagtaataataaaatatg
atgtttccttgccgacaaaaaaagtgacaggaatattattgctgattgtcatttcgggcagtttattttccgcttgtcaatttgcttataaagatgcaaa
aaataaaaaagcgttcagtccatatatactagcgtcgcgatttgctacctatacgccgtttttcaatctcaactattttgctttagcagcgaaagagc
atcaaagattactctcaattgcaaacacggtgccgtattttcaattatcagtcagggatacaggtattgatacctacgtgttgattgtgggggagtct
gtacgtgtcgacaatatgtctttgtatggatatacacgctctacgacaccgcaagttgaagcacaaagaaaacagatcaaactgtttaatcaag
caataagcggcgcaccttacactgcgctgtcggttcccctttctttaactgctgattctgttttgagtcatgacattcataattaccccgacaacattat
taatatggctaatcaagcaggatttcagactttctggctaagctcgcaatccgcttttcggcagaatggtacagcagttaccagtatcgccatgcg
cgccatggaaacagtttatgtcagaggatttgatgaattgttgttgccgcatttatcgcaagcattacagcaaaatacgcagcaaaagaaactg
attgttcttcatttaaatggaagccatgaaccggcttgtagcgcctatccgcaatccagcgccgtgtttcaaccgcaggacgatcaggatgcctg
ctatgacaactccattcattacacagatagtttgctaggtcaggtttttgaattattaaaagatcgccgcgcctcggtcatgtattttgccgaccacg
gcctggaacgtgaccctacgaagaagaacgtctattttcatggaggcagggaggctagccagcaggcatatcatgtcccgatgtttatctggta
tagccccgttcttggggatggcgtggatcgcacaacggaaaacaacatcttttcgacagcttacaataattaccttattaatgcgtggatgggggt
aacaaagccggaacagccgcaaacgcttgaggaagtgattgcacactataaaggagactcacgggttgtggatgcaaaccatgatgttttcg
attatgtgatgctcagaaaggagtttacagaggataagcaaggtaaccccacccctgaagggcagggttgatgtcgaattactggcctttgtttt

ccagattctccacctgcggcacgcctgaagcggcagaggaggaaagcaggccagttttggcataaccgaaaagtttctcgcgggtatcagtg
atatccaggttacgcatggtcaattgaccaatacgatcatctggcgagaacaccgagtcgcctttttccatcgtcagacgctctggcttgtaggtc
aggttctctgagacggtattcaggattgaataatcgttcccacggcgcagctccagggtaacttcaccagtgatctggctggcaacccagcgttg
cagagagtcacgcagcatcagcgcctgggagtcaaaccaacgcccctggtacagcagacggcccaactgacgaccatgcgcgtgatact
gctcaatggtgtcttcgttgtgaataccggtcaacaggcgttcatacgcaatgtgcagcagtgccatccccggagcttcgtaaataccacggcttt
tcgcttcgatgatacggttttcaatctggtcgctcatgcccaggccgtgacgaccgccgatgcggttagcttccagcatcatttctacgtcgtcgcta
aaggttttaccgttcagcgccaccggatgaccttgttcaaagcgtactgtgacttcttctgccgggatcttcacgctctcatcccagaatttcacgcc
cataatcgggttgacgattttgacgctggagttgaggtattccagatccttcgcttcatgcgttgcaccaagcatgttggagtctgtggagtaggcttt
ttcgacagacattttgtagtcgaaaccgcaggcaatcataaattcagacatctcatgacggccgcccagttcatcaataaagtcagtatcaagc
cacggtttgtaaatctgcagttcagcattggtcagcagaccataacgatagaaacgttcgatatcgtttcctttgtaggtgctaccgtcaccccaga
tattcacgccatcttctttcatcgcagcaaccagcatggtaccagtcacggcgcggcccagcggcgtcgtgttgaaataggtcaggccgccggt
ggtgttatgaaatgcgccacactgaatagcggcaataccttcggccaccagttgtttgcggcagtcgatcagacgtgcgttctccgcgccgtatt
ccatggcacgacgagggatcgcatcataatcctcttcgtctggctggcccaggtttgcagtatatgcataaggaaccgctccctttgtcgcatcc
acagcagtgcggcactggtgtccagaccgccagaaaaagcgataccaatacgttgacctaccgggagatgcttgagaatcgtcgtcataaa
ataacaccctgcttaattaactgatgatgagcctggatttccgctctcactgaatttttatgcaaaataaatgagttttcatttaatcatcttttatcggag
acaggaagagtttagtgtgtttttttgtaaaataatgcgcttaagggagagcaggagaaggcaaaagtattcaacaaatgaaagtgaactggat
attcattcacatgattagcaataaacgttgacaaaatgtggcgtggatcactataatgcctgcagattttacgtcccgtctcggtacaccaaatccc
agcagtatttgcatttttttacccaaaacgagtagaatttgccacgtttcaggcgcggggtggagcagcctggtagctcgtcggctcataacccg
aagatcgtcggttcaaatccggcccccgcaaccactttcccttagagtcctttttcaaatatactgtgaagacttcggccttcgtagtgggatttgaa
aaaatccttctggaaagtgctccagaccgcagttgcggttatagggttcagttatataaagccccgatttatcggggtttttttgttatctgactacaga
ataactgggctttaggcccttttttttatgtcttgggggtgggcttgtccacattagagcaaaaattaacagagatgattactgcgccagttgaggccc
tgggttttgaactggttggcatcgaatttattcgcggtcgcacatccacactgcgcatctatattgatagtgaagatggcatcaatgttgatgattgtg
ctgatgtgagccaccaggtaagtgctgtgctggatgttgaagatcccatcaccgttgcttataacctggaagtctcctcaccgggtctcgatcgcc
cactgttcacggctgaacactacgcccgttttgtcggagaagaggtgactctggttctccgtatggcggtacaaaaccgtcgtaaatggcaggg
cgttatcaaagcggtagacggtgaaatgatcacagttaccgtcgaaggtaaagatgaagtgttcgcgctgagtaatatccagaaggcgaacc
tggttccccacttttaatagtctggatgaggtgaaaagcccgcgatgaacaaagaaattttggctgtagttgaagccgtatccaatgaaaaggc
gctacctcgcgagaagattttcgaagcattggaaagcgcgctggcgacagcaacaaagaaaaaatatgaacaagagatcgacgtccgcgt
acagatcgatcgcaaaagcggtgattttgacactttccgtcgctggttagttgttgatgaagtcacccagccgaccaaggaaatcacccttgaa
gccgcacgttatgaagatgaaagcctgaacctgggcgattacgttgaagatcagattgagtctgttacctttgaccgtatcactacccagacgg
caaaacaggttatcgtgcagaaagtgcgtgaagccgaacgtgcgatggtggttgatcagttccgtgaacacgaaggtgaaatcatcaccggc
gtggtgaaaaaagtaaaccgcgacaacatctctctggatctgggcaacaacgctgaagccgtgatcctgcgcgaagatatgctgccgcgtg
aaaacttccgccctggcgaccgcgttcgtggcgtgctctattccgttcgcccggaagcgcgtggcgcgcaactgttcgtcactcgttccaagccg
gaaatgctgatcgaactgttccgtattgaagtgccagaaatcggcgaagaagtgattgaaattaaagcagcggctcgcgatccgggttctcgt
gcgaaaatcgcggtgaaaaccaacgataaacgtatcgatccggtaggtgcttgcgtaggtatgcgtggcgcgcgtgttcaggcggtgtctact
gaactgggtggcgagcgtatcgatatcgtcctgtgggatgataacccggcgcagttcgtgattaacgcaatggcaccggcagacgttgcttcta
tcgtggtggatgaagataaacacaccatggatatcgccgttgaagccggtaacctggcgcaggcgattggccgtaacggtcagaacgtgcgt
ctggcttcgcagctgagcggttgggaactcaacgtgatgaccgttgacgacctgcaggctaagcatcaggcggaagcgcacgcagcgatcg
acaccttcaccaaatatctcgacatcgacgaagacttcgcgactgttctggtagaagaaggcttctcgacgctggaagaattggcctatgtgcc
gatgaaagagctgttggaaatcgaaggccttgatgagccgaccgttgaagcactgcgcgagcgtgctaaaaatgcactggccaccattgca
caggcccaggaagaaagcctcggtgataacaaaccggctgacgatctgctgaaccttgaaggggtagatcgtgatttggcattcaaactggc
cgcccgtggcgtttgtacgctggaagatctcgccgaacagggcattgatgatctggctgatatcgaagggttgaccgacgaaaaagccggag
cactgattatggctgcccgtaatatttgctggttcggtgacgaagcgtaataaactgtagcaggaaggaacagcatgacagatgtaacgattaa
aacgctggccgcagagcgacagacctccgtggaacgcctggtacagcaatttgctgatgcaggtatccggaagtctgctgacgactctgtgtc
tgcacaagagaaacagactttgattgaccacctgaatcagaaaaattcaggcccggacaaattgacgctgcaacgtaaaacacgcagcac
ccttaacattcctggtaccggtggaaaaagcaaatcggtacaaatcgaagtccgcaagaaacgcacctttgtgaaacgcgatccgcaagag
gctgaacgccttgcagcggaagagcaagcgcagcgtgaagcggaagagcaagcccgtcgtgaggcagaagaatcggctaaacgcgag
gcgcaacaaaaagctgaacgtgaggccgcagaacaagctaagcgtgaagctgctgaacaagcgaaacgtgaagctgcggaaaaagac
aaagtgagcaatcaacaagacgatatgactaaaaacgcccaggctgaaaaagcccgccgtgagcaggaagctgcagagctcaagcgta
aagctgaagaagaagcgcgtcgtaaactcgaagaagaagcacgtcgcgttgctgaagaagcacgtcgtatggcggaagaaaacaaatg
gactgataacgcggaaccgactgaagattccagcgattatcacgtcactacttctcaacatgctcgccaggcagaagacgaaagcgatcgtg

aagtcgaaggcggccgtggccgtggtcgtaacgcgaaagcagcgcgtccgaagaaaggcaacaaacacgctgaatcaaaagctgatcg
tgaagaagcacgcgcagcagtacgtggcggtaaaggcggaaaacgtaaaggttcttcgctgcagcaaggcttccagaagcctgctcaggc
cgttaaccgtgacgttgtgatcggcgaaactatcaccgttggcgaactggcgaacaagatggcggttaaaggctctcaggtcatcaaagcgat
gatgaaactgggcgcaatggcaaccatcaaccaggttatcgatcaggaaaccgcacagctggttgctgaagagatgggccataaagttatc
ctgcgtcgtgaaaacgagctggaagaggcggtaatgagcgaccgtgacacgggtgctgcggctgaaccgcgcgcgccggttgtgaccatc
atgggtcacgttgaccacggtaaaacctctctgctggactacattcgttcaacgaaagtggcctctggcgaagcgggcggcattacccagcac
attggtgcataccacgttgaaactgaaaacggcatgatcaccttcctggacaccccggggcacgccgcgtttacttcaatgcgtgctcgtggtg
cgcaggcaacggacatcgtagtcctggttgttgctgccgacgacggtgtgatgccgcagaccatcgaagcaatccagcacgcgaaagcgg
cgcaggtaccggtggtggttgcagtgaacaagatcgataaaccagaagctgatccggatcgcgttaagaacgaactctcccagtacggcat
cctgccggaagagtggggcggtgaaagccagttcgtacacgtatctgcgaaagcgggtaccggtatcgatgaactgctggacgctatcctgc
tgcaggcggaagttctggagctgaaagcggtacgtaaaggtatggcgagcggtgcggttatcgaatccttcctcgataaaggtcgtggtccgg
ttgctaccgttctggtacgtgaaggtactctgcacaagggcgatatcgttctgtgtggcttcgaatacggtcgtgttcgtgcgatgcgtaacgaact
gggtcaggaagtgctggaagcgggtccgtccattccggtggaaatcctcggcctgtccggcgtaccggctgcgggtgatgaagttaccgttgt
acgtgacgagaagaaagcgcgtgaagttgcactctatcgtcagggtaaattccgcgaagttaaactggcgcgtcagcagaaatctaaactcg
agaacatgttcgccaacatgaccgaaggcgaagttcacgaagtgaatatcgtcctgaaggcagacgtacagggttctgtcgaagcgatctcc
gactccttgctgaaactgtctactgacgaagttaaagtgaagatcatcggttctggcgtaggtggtatcaccgaaaccgacgccaccctggctg
cggcgtccaacgccatcctggttggctttaacgtacgtgctgatgcctctgcacgtaaagtgattgaagcggaaagcctggatctgcgttactact
ccgtcatctataacctgattgacgaagtgaaagcggcgatgagcggtatgctgtctccggaactgaaacagcagattatcggtctggcggaag
ttcgtgacgtgttcaaatcgccgaaatttggtgccatcgcaggctgtatggttaccgaaggtgtggttaaacgtcacaacccgatccgcgttctgc
gtgacaacgtggttatctacgaaggcgagctggagtccctgcgccgcttcaaagatgacgttaacgaagtccgtaacggtatggaatgtggtat
cggcgttaagaactacaacgacgtccgcactggcgatgtgatcgaagtattcgaaatcatcgagatccaacgtaccattgcttaaggtttttagg
gttccatgcgggataaggcgtttacgctgcatccggcatggattgcctgatgtgacgctggcgcgtcttatcaggtcaacgtgaacagcccttcc
acctgaaattaattttaaaaaggggctaacagccccttttttgtcaggagaatttattatggcgaaagaatttggtcgcccgcagcgcgtagcgca
ggaaatgcaaaaagagatcgctctcatcctgcagcgtgaaattaaagatcctcgcctgggcatgatgaccaccgtttccggtgtcgaaatgtct
cgcgacctggcgtatgccaaagtatatgtgacgttcctcaacgacaaagatgaagacgcggttaaagcgggcatcaaagcgttgcaagaag
cttctggtttcatccgcagcctgctggggaaagcgatgcgcctgcgtatcgtgccggaactgaccttcttctacgacaactctctggttgaagga
tgcgcatgtcaaacctggtgaccagcgtggtcaaacatgacgaagaacgtcgtgttaacccggacgacagcaaggaggactaatgagtcgt
cctcgtcgtcgcggtcgcgacattaacggcgttttgttgctggataaacctcagggtatgtccagcaacgatgcgctgcaaaaagtgaaacgta
tatataacgccaaccgtgccgggcataccggtgcgctggacccgctggcgaccggcatgttgccgatttgcctcggggaagcgacgaagtttt
cccagtatctgctggactccgacaaacgctatcgggtcattgcgcgtcttggacagcgtaccgatacttctgatgccgacggacagatcgttga
agaacgtccggtaacctttagcgcagagcagctggcagcggcactggatactttccgtggcgatatcgaacagatcccttcgatgtattcagca
ctgaaatatcagggcaaaaaactgtacgaatatgcgcgtcagggcattgaagttccgcgtgaagcgcgtccgattaccgtttatgaattgctgttt
attcgccatgaaggcaatgagctggagctggaaattcactgctcaaaaggcacttatatccgcaccatcattgatgacctgggtgaaaaactc
ggctgtggcgcgcatgttatttacctgcgccgtctggcggtaagtaaatatccggttgaacggatggtgaccctggagcacctgcgtgaacttgtt
gagcaagctgaacagcaggatattccagccgcggagttacttgatccattactgatgccaatggacagtccagcttcggactatccggtggtg
aatcttccgttaacgtcttctgtttacttcaaaaatggtaacccggttcgtacatctggtgcgccactggaaggactggttcgcgtcacggaaggtg
agaacggcaaatttatcggtatgggcgaaattgacgatgaaggccgcgttgcgcctcgtcgcctggtggttgaatacccggcgtaatgttaacc
gtcttgcgataacaggtcgctacgagtagaatactgccgcttaacgtcgcgtaaattgtttaacactttgcgtaacgtacactgggatcgctgaatt
agagatcggcgtcctttcattctatatactttggagttttaaaatgtctctaagtactgaagcaacagctaaaatcgtttctgagtttggtcgtgacgc
aaacgacaccggttctaccgaagttcaggtagcactgctgactgcacagatcaaccacctgcagggccactttgcagagcacaaaaaagat
caccacagccgtcgtggtctgctgcgcatggtttctcagcgtcgtaaactgctcgactacctgaaacgtaaagacgtagcacgttacacccagc
tcatcgagcgcctgggtctgcgtcgctaattcttgcgagtttcagaaaagggggcctgagtggcccctttttttcaagctgacggcagcaattcact
ggaaactaatgtattgttgctatgaatgatcttccgttgcagaggttcgcgcggctaatgagaggctttacccacatagagctgggttagggttgtc
attagtcgcgaggatgcgcagaagatcgggtattaacaccagtgccgtaaggtactgtctaagaaagagaaaggatattacattgcttaatcc
gatcgttcgtaaattccagtacggccaacacaccgtgactctggaaaccggcatgatggctcgtcaggctactgccgctgttatggttagcatgg
atgacaccgcggtattcgttaccgttgttggccagaaaaaagccaaaccaggtcaggacttcttcccactgaccgttaactatcaggagcgtac
ctacgctgctggtcgtatcccgggtagcttcttccgtcgtgaaggccgcccaagcgaaggcgaaaccctgatcgcgcgtctgattgaccgccc
gattcgcccgctgttcccggaaggcttcgtcaacgaagttcaggttatcgccaccgtggtttctgttaacccgcaagttaacccggatatcgtcgc
gatgattggtgcttccgcagcgctgtctctgtctggtattccgttcaatggcccgattggtgctgcccgcgtaggttacatcaatgaccagtacgtac
tgaacccgactcaggacgagctgaaagagagcaaactggatctggttgttgccggtactgaagccgctgtactgatggttgaatctgaagctc

aactgctgagcgaagaccagatgctgggcgcagtagtgttcggtcatgaacaacagcaggttgttattcagaacatcaatgaactggtgaaa
gaagccggtaaaccgcgttgggactggcagccggagccggtaaacgaagcgctaaacgcgcgcgttgctgcactggctgaagctcgcctg
agcgatgcttaccgcatcaccgacaaacaagagcgttatgcgcaggttgatgtcatcaaatctgaaaccatcgcgacgctgcttgctgaagac
gaaaccctggacgaaaacgaactgggtgaaattctgcacgcgatcgagaaaaacgttgttcgtagccgcgtactggcaggcgaaccgcgt
atcgacggtcgtgaaaaagatatgatccgtggtctggatgtgcgtactggcgtgctgccgcgtactcacggttctgcgctgttcacccgtggtga
aacgcaggcgctggttaccgcaacgctgggtactgctcgtgacgcgcaggttcttgatgaactgatgggcgaacgtaccgataccttcctgttc
cactacaacttccctccgtactccgtaggcgaaaccggcatggtcggttctccgaagcgtcgtgaaattggtcacggtcgtctggcgaagcgcg
gcgtgctggcagtcatgccggatatggacaaattcccgtacaccgtacgtgttgtgtctgaaatcactgaatccaacggttcctcttctatggcttc
cgtgtgcggcgcgtctctggcgctgatggacgcaggtgtgccgatcaaagctgccgttgcgggtatcgcaatgggtctggtgaaagaaggcg
acaactacgttgtactgtctgacattttgggcgacgaagatcacctgggcgatatggacttcaaagttgcaggttcccgcgacggtatctctgcac
tgcagatggatatcaaaattgaaggtatcaccaaagagatcatgcaggttgcgctgaaccaggctaaaggtgcgcgtctgcatatcctgggcg
taatggaacaggcgatcaacgcgccgcgtggcgatatctctgagttcgcaccgcgtatccataccatcaagatcaacccggacaagatcaa
agatgttatcggtaaaggcggttctgtaatccgtgctctgaccgaagaaactggcaccaccatcgaaatcgaagatgacggtactgtgaagatt
gcagcgaccgacggcgagaaagcgaaacatgctattcgtcgtatcgaagagatcactgcagaaatcgaagtgggccgcgtctacactggt
aaagtgacccgtatcgttgactttggcgcatttgttgccatcggcggcggtaaagaaggtctggtccacatctctcaaatcgctgacaaacgcgtt
gagaaagtgaccgattacctgcagatgggtcaggaagtaccggtgaaagttctggaagttgatcgccagggccgtatccgtctgagcattaa
agaagcgactgagcagtctcaacctgctgcagcaccggaagctccggctgctgaacagggcgagtaaggttgccatttgccctccgctgcg
gcggggggcttttaaccgggcaggacgccttgttagcaaccgggaacaggacgttcattcaaccgtggtcttcgggagtgggaaatgaagcc
ttttttgcgctggtgtttcgttgcgacagcacttacgcttgcaggatgcagtaatacttcctggcgtaaaagtgaagtcctcgcggtaccattgcaac
cgactttacagcaggaagtgattctggcacgtatggaacaaatccttgccagtcgggctttaaccgatgacgaacgcgcacagcttttatatga
gcgcggagtgttgtatgatagtctcggtctgagggcattagcgcgtaacgatttttcgcaagcgctggcaatccgaccggatatgcctgaagtatt
caattacttaggcatatatttaacgcaggcaggcaattttgatgctgcctatgaagcgtttgattctgtacttgagcttgatccaacttacaactacgc
gcacttgaatcgcgggatcgcattatattacggcggtcgtgacaagttagcgcaagatgatctgctggcgttttatcaagacgatcccaatgatc
ctttccgtagtctgtggctttatctcgccgagcagaagctcgatgagaagcaggctaaagaagtgttgaaacagcacttcgaaaaatcggataa
ggaacagtggggatggaacattgtcgagttctacctgggcaacattagcgaacaaacgttaatggaaaggctcaaggcggacgcaacgga
taacacctcgctcgctgagcatctcagtgaaaccaacttctatttaggtaagtactacctaagtctgggggatttggacagcgccacggcactgtt
caaactggcggttgccaacaacgttcataactttgttgagcaccgatacgcattgttggaattatcgctcctgggccaggaccaagatgacctgg
cagaatcggaccagcaatagctgacgtacacatcagcccgtaatctttttttgattgccatcaccttaacgggtgagggcgttgttgttcgttaatac
acctactttgagccggttcacacttttcaatgaaaattgctgatcaatttcatgatgagttatgtagactggccgccattaattttgaggcacacgtac
tacatggctgaattcgaaaccacttttgcagatctgggcctgaaggctcctatccttgaagcccttaacgatctgggttacgaaaaaccatctcca
attcaggcagagtgtattccacatctgctgaatggccgcgacgttctgggtatggcccagacggggagcggaaaaactgcagcattctctttac
ctctgttgcagaatcttgatcctgagctgaaagcaccacagattctggtgctggcaccgacccgcgaactggcggtacaggttgctgaagcaat
gacggatttctctaaacacatgcgcggcgtaaatgtggttgctctgtacggcggccagcgttatgacgtgcaattacgcgccctgcgtcagggg
ccgcagatcgttgtcggtactccgggccgtctgctggaccacctgaaacgtggcactctggacctctctaaactgagcggtctggttctggatga
agctgacgaaatgctgcgcatgggcttcatcgaagacgttgaaaccattatggcgcagatcccggaaggtcatcagaccgctctgttctctgca
accatgccggaagcgattcgtcgcattacccgccgctttatgaaagagccgcaggaagtgcgcattcagtccagcgtgactacccgtcctgac
atcagccagagctactggactgtctggggtatgcgcaaaaacgaagcactggtacgtttcctggaagcggaagattttgatgcggcgattatctt
cgttcgtaccaaaaacgcgactctggaagtggctgaagctcttgagcgtaacggctacaacagcgccgcgctgaacggtgacatgaaccag
gcgctgcgtgaacagacactggaacgcctgaaagatggtcgtctggacatcctgattgcgaccgacgttgcagcccgtggcctggacgttga
gcgtatcagcctggtagttaactacgatatcccgatggattctgagtcttacgttcaccgtatcggtcgtaccggtcgtgcgggtcgtgctggccgc
gcgctgctgttcgttgagaaccgcgagcgtcgtctgctgcgcaacattgaacgtactatgaagctgactattccggaagtagaactgccgaacg
cagaactgctaggcaaacgccgtctggaaaaattcgccgctaaagtacagcagcagctggaaagcagcgatctggatcaataccgcgcac
tgctgagcaaaattcagccgactgctgaaggtgaagagctggatctcgaaactctggctgcggcactgctgaaaatggcacagggtgaacgt
actctgatcgtaccgccagatgcgccgatgcgtccgaaacgtgaattccgtgaccgtgatgaccgtggtccgcgcgatcgtaacgaccgtggc
ccgcgtggtgaccgtgaagatcgtccgcgtcgtgaacgtcgtgatgttggcgatatgcagctgtaccgcattgaagtgggccgcgatgatggtg
ttgaagttcgtcatatcgttggtgcgattgctaacgaaggcgacatcagcagccgttacattggtaacatcaagctgtttgcttctcactccaccatc
gaactgccgaaaggtatgccgggtgaagtgctgcaacactttacgcgcactcgcattctcaacaagccgatgaacatgcagttactgggcgat
gcacagccgcatactggcggtgagcgtcgtggcggtggtcgtggtttcggtggcgaacgtcgtgaaggcggtcgtaacttcagcggtgaacg
ccgtgaaggtggccgtggtgatggtcgtcgttttagcggcgaacgtcgtgaaggccgcgctccgcgtcgtgatgattctaccggtcgtcgtcgttt
cggtggtgatgcgtaatcatcgctgaacagcgaacacaatctgtaaaataatatatacagccccgatttttaccatcggggctttttttctgtcttttgt

actcgtgtactggtacagtgcaatgcataacaacgcagtcgcactatttttcactggagagaagccctcatggcaacactaaccaccacccaa
acgtcaccgtcgctgcttggcggcgtggtgattatcggcggcaccattattggcgcagggatgttttctctgccagtggtcatgtccggggcgtggt
ttttctggtcaatggcggcgctgatctttacctggttctgtatgctgcattccggcttgatgattctggaagctaacctgaattacagaatcggttcgag
ttttgacaccatcaccaaagatttgctgggcaaaggctggaacgtggtcaacggcatttccattgcctttgtgctctatatcctgacctatgcctatat
ttctgccagtggttcgattctgcatcacaccttcgcagagatgtcactaaacgtcccggcacgggcggcgggttttggttttgcattgctggtagcgt
ttgtggtgtggttgagcactaaagccgtcagtcgcatgacagcgattgtgctgggggcgaaagtcattaccttcttcctcacctttggtagcctgct
ggggcatgtgcagcctgcgacattgttcaacgtcgccgaaagcaatgcgtcttatgcaccgtatctgttgatgaccctgccgttctgtctggcatc
gtttggttatcacggtaacgtgccaagcctgatgaagtattacggcaaagatccgaaaaccatcgtgaaatgtctggtgtacggtacgctgatg
gcgctggcgctgtataccatctggttgctggcgacgatgggtaacatcccgcgtccggagtttatcggtattgcagagaagggcggtaatattga
tgtgctggtacaggcgttaagcggcgtactgaacagccgtagtctggatctgctgctggtcgtgttctcaaactttgcggtagcgagttcgttcctc
ggcgtaacgctgggtttgtttgactatctggcagatctgtttggtttcgacgactcggctgtgggccgcttgaaaacggcattgctgacctttgcccc
gccagttgtgggggggctgttgttcccgaacggattcctgtacgccattggttatgctggtttagcggctaccatctgggcggcaattgttccggcg
ctgttagcccgtgcatcgcgtaaacgctttggcagcccgaaattccgcgtctggggtggcaagccgatgattgcgctgattctggtgtttggcgtc
ggcaacgcactggtgcatattttatcgagctttaatttactgccggtgtatcagtaatcagcggtgccttatccgacatttctgctgcctacacaatgc
ctgatgcgcttcgcttatcaggtctatgtaggacagcgttgccagctcggataaggcttcccgcgttaagacacactatcccaacaactcttcctta
acatccatcgccagctcaaacgaatgcagccgcgcctggtggtcgaaaatctgcccgttgaccataatctcatcggcgtcggtttcgcgcagg
atcgactgcaagccatgacgcactttcgctttatcacctaccaacgacatactcagcgcctgctgcacgccatactgctcagacggtgaccag
aactgatccatattttgaatcggcggcggcagttgcccggtttcgccacggcgcagcttcacaaaggcttgctgcattgaggtaaacagaaattc
agcgtcgcggttgctgtcggcggcgataatattgatgcacaccatcgcgtatggtttttccagccgtgctgacggtttgaagttgctgcgataaag
atgcagcgcctggaacagcatatccggcgcgaagtgtgaggcaaacgcaaacggcagaccaagctgcgccgccagttgcgcgctgtaaa
ggctggagcctaacaaccacacggggattttctcgccatagcctggtaccgggcgcacatgcggattgggatcgcgggcgtcaaaccagtc
caccagctccgccacatcgcgggggaaattatcaatatcgccgctcatatgacgacgtagcgccatcattgtccgttggtcactacccggagc
acgacccagccccaaatcgattcgccccggatagagtgtattaagcgtgccgaactgttctgcaatgaccaacggtgagtggttaggcaacat
cacgccgccagaccccagatgcagcgtggtggtattcgccgccagatagccgatcaataccgacgtggcagcactggcaatgccagtcata
ttgtggtgttctgccagccagtagcgatgatagccgcgcttttcagccagacgggcgagatcgagagagtgggagaatgcttctcgcgctgaa
gaaccttcgggaatgggggccagatcgagtagcgaaaacgcaatggttttatcagtcataacagctcactttgttaccgattaatctttaatactg
cattaaagagtagttaaagttgttaacaaagtgagctatttacttaggcttgcagctccagtcctgccagccgccgccagtagccgttacagtcgc
tgttcgccgtcaacggcagtggtgcagcgccattttcattagcgcggaaggcgtcgagcatcgcgaaagtgtcagtaccctgcggtgacaggc
ggaccacatcaaccaggccctgcatggatgccagctcgttaccgaggttgtaaacgtagccgctcatggtctgaatgccattgagtacaaaca
cttgttggttttcctgcgacagcacgttgcgcccgttcggatacttaatgcagcaggtttcacactcatctttcgggcggtcttccgaacgcgcggta
aagcagcgggcggagtaggccagcggcagatgaccgtagctcaggacttccacttcaaactggttacgaatgcccagctcatcgcactgatt
aagcagattcaccagccagtcgcgggaaagctccactggcatacaccagcgcatcatgccctgtttgagcaatattttcagtgtcaccgcgttg
tagcagttcagcgcgtgcccggcgacgaacggcagtttgcgttcagcgcacatattcaccacgccgagatcgctggcttcaatcagaaactca
ccgttctcaacatagcgtttcagttcgcccagttcagatgatgcctgcaccagcgccagcgtggagagcacaatctgcttaccactcccggcga
gcgattttgccatctccagccagtcgccaactttggttgcccgacgcttgctgcataccgcttcaccaagataaatcacgtcggcgctgctggtgg
cggcctgctgataaaattcttccagcgtctcttttggccagtaccacagcactggccctaaggaatatttcattgcttttctcactgccatttacggtg
atatgcgccaagagtggtttgcgtgccttcggacatcgacccgagcgtctccatccacgcgctttgcggtacaaagttttgcggatcggccttac
aacggtcgatagcctgacgccagactttcgccacctggctgacatacgccgggctacgctggcggccttcaattttcaccgaagcaatattcgc
cgccattaactccggcagcagttccagggtattgagactggttggttcttccagcgcgtgatagcgctcgccgtccaccagataacgccctttac
atagcgtcggataacctgcgttttcgccgtcctgataacggtcgatcagcacttcgttcaggcgggattccagcccctgcggcgtttgttgccagc
gcacgaaacgggccggagaacacgcgcctatggtgttgggcgactcacccgtcagatacgacgacagatagcaacgaccttccgacata
atgcacaggctgccgaaagcaaagacttccagtggtacaggtgtgacccgtgccagttgtttcacctgatgaatcgacaacacgcgcggcag
caccacgcgagcaacgtcaaaatggcgatgataaaagttaatcgcctcttcattggtcgccgaagcctgcaccgatacgtggcgctcaatatg
cggataacgctcggcggcgtactccagcatggcgaggtcggcgaggatcagcgcgtcggcacccagctgcgccgccatatccacggcgc
gctgccaacgggcgtaaccgtccggatgcgcaaaagtgttaatcgcgatgtgaagtttgcggcgatgttgatggacaaaactcaccgcttcct
gcaatttttctcggtaaagttaaggccggcgaagtgacgggcattggtatcatcttttagcccgatataaacagcatctgcgccgttttcgatggc
cgccttaagcgccgggagatttccggcagggcagagcagctccataatttttcctgaaggttgcgctatcaaaacgcaaaaatgttaacgaac
tgggattttagttaaccttgctgcgacaatttttgatttaaggcagttaaagttgtattggtggtagcagcaatttcatatggaattgttgatttataccgc
tatgttatttttctgatatggcaaaataggatgattgttgaaacagggagtaaaactcgtgttggataaactgcgttcccgtattgtgcatttggggcc
atctctgttgagtgtaccggtaaaactgacgccatttgcgctaaaacgccaggttcttgagcaggtcttaagctggcaattccgccaggcgctgg

atgatggcgagctggagtttcttgaaggccgctggttaagtattcatgtgcgtgatattgacctgcaatggtttacctcggtggtgaatggcaaact
ggtcgttagccagaacgcgcaagctgatgtgagttttagtgccgacgccagcgatctgctgatgattgcggcgcgtaaacaagatccggatac
gctcttcttccagcgtcggctggtgattgaaggcgatacggagctggggctgtatgtgaaaaacctgatggacgccattgaactggagcaaatg
ccgaaagccttacgcatgatgctgctgcaactggcggattttgttgaggcgggaatgaaaaccgcgcctgaaaccaaacagacatcggtag
gtgaaccatgctaattcgagtagaaattcccattgatgcgccgggtattgatgccctgctgcgtcgctcattcgaaagtgatgcggaagcgaag
ctggttcacgatctgcgtgaagatggctttctgacgctggggctggtggcgacagatgacgaaggtcaggtcattggctatgtggcatttagtccg
gttgatgtgcagggcgaagacctgcaatgggtcggcatggcaccgttggcggttgatgaaaaataccgtggacaggggctggcacgccaac
tggtctatgaaggactcgattcgcttaatgagttcggctatgccgcagtggtgacgctgggcgatccggcgctgtacagtcgtttcggctttgaact
ggcggcgcatcacgatctgcgttgccgctggccgggcactgaaagcgccttccaggtacatcgtttagcggatgacgcgctgaatggcgttac
cggcctggttgagtatcacgagcatttcaatcgcttttaatctccggggtttgcagactgcttaacagctctgcaaaccctgcgccttccgctacca
gacgctctttttgccgtttggtcagttgcttaacgcgatattccgcccgtaacgccagcgaacgatcgcccactggcgcggaaaacaccagcgtt
agttctcctttcccgcgcagtgctttcgcccctttgccgctttggtgctgctgatagcggcgttcgacatccgtggtgatcccggtataaagcttattgt
cggcggtacggatcaagtaaagaaaccagggtgtcatcgtctgcgtcgcatgttaaggtcaggcccacagtataaaagagaacagcaatgg
aaacactcatcgccatcagccgttggctggcaaaacaacatgttgtcacttggtgtgtgcagcaggaaggggaactctggtgcgctaatgcctt
ttatctttttgatgcgcagaaagtcgccttctacattttgacggaagaaaaaacgcgccacgcgcagatgagtggccgcaggctgcggttgcc
ggaacggtaaacggtcagccgaaaacggtagcgttaattcgcggtgtgcagtttaaaggtgagatccgcaggctggaaggtgaggaaagc
gacctcgcgcgcaaggcgtacaatcgtcgctttccggttgccagaatgctgtcggccccggtgtgggaaatccggctcgatgaaatcaaattc
accgacaacacgctgggctttggtaaaaaaatgatttggctacgcgactcaggcaccgagcaggcgtaacgcctcgcggttaaacgctggc
agatcgtccggtgtccggctggtaaccagctgatctttatcaaccacgacttcctgatcgtaaaattccgcgcccgcatttttaacatcaataatga
tcggtttaactgcggtcagtttgcgcccccgaatcacatcggcgctgatcagcaactgcgggccgtgacagatggcaaacaccggtttgccact
attcacaaaatcacgggtaaaggtgacaaaacggttgtccccacgcagataatccggtgaatggccgcccggtagcagcagggcatcaaa
ctccgcaggcgtcacttcatcgatggatttatcgatggtcacgctggcttctcctttttgcctttcaccgttttacccgcttgtttttcaatggtaatcactt
cgtgtccggctttacggaactcgtctgcgggtgaagtaaattctgaatcctcaaattcatcagtgattaaaacggcaattttcttactcatgcttcctc
cgcgttgttgcattggatgaggcgaatttcccacaatcggggataattacctcatggtctattaagcctggtgcatgagccgtggagcgcaaggc
agacaattctgtaaaggagcgaagatgagtcaggtactgattacaggcgcaacggggctggtgggcggtcacctgctgcggatgttgattaa
cgaaccgaaagttaacgccattgctgcgccgacgcgacgtccgttgggcgatatgcccggggtgtttaatccccatgatccgcaactgagtga
cgcgttggcgcaggtcaccgatcccatcgacattgtgttttgttgtctcggcaccacgcggcgagaagcggggagcaaagaggcgtttattcat
gccgattacacgctggtagtggataccgcattaaccgggcggcgactgggcgcgcagcatatgttggtagtcagtgcgatgggtgccaatgc
ccactcgccgtttttctataaccgcgtcaaaggggagatggaagaagcattaatcgcccagaactggccgaaattgaccattgctcgcccgtc
gatgttactgggcgatcgtagcaaacagcggatgaacgaaacgctttttgcgccgctgttccgtttgttaccaggtaactggaaatccattgatgc
gagagacgtggcgcgggtcatgctggcagagtcgatgcggcctgagcatgaaggcgtgacgattttaagctcttcagaattacgcaaaaga
gctgaataattattcccaagaattgatagtgagcgtaatattcacgcgctcaattatcataacttattcctggggaatgctatgactggtcagtcttca
tctcaggcggcaacgcccattcagtggtggaaacccgcgcttttctttctcgttgtcattgccggtctctggtatgtgaaatgggaaccttactacgg
caaagcgtttactgctgccgaaacccacagtatcggtaaatctatccttgcgcaggcggatgctaacccatggcaggcggcgttggattacgc
gatgatctatttcctcgcggtatggaaagcggcggtgctgggggtgatcctcggttcgttgattcaggtgctgatcccgcgtgactggttgttgcgta
cgcttgggcaatcgcgctttcgcggcacgctgctgggaacgctgttttcgttgccgggcatgatgtgtacctgctgtgcggctccggtcgcggcg
ggaatgcgtcgccaacaggtgtcgatgggcggtgcgctggcattctggatgggcaatccggtgttaaacccggcgacgctggtgtttatgggct
ttgtcctcggctggggttttgcggcgattcgtctggtggccgggctggtgatggtgttgctgattgcgacgctggtgcaaaaatgggtgcgtgaaa
caccgcaaacgcaggcaccggtcgaaattgacataccggaagcacagggcgggtttttagccgctggggcagggcgctatggacgcttttc
tggagtacgatcccggtttacatccttgcagtactggtgttgggtgccgctcgcgtctggttattcccccatgccgatggtgctgtcgataacagcct
gatgtgggtggtggcgatggcggtagcaggatgcttgtttgtcattcccacggcagcagaaattccgattgtacaaacgatgatgctggcaggt
atgggaaccgctccggcgctggcattgttgatgacgctcccggcagtgagtttgccgtcactgattatgctgcgcaaagcgttcccggcgaaag
ccttatggctgacaggggcgatggtggcagtgtctggtgtgattgtcggcgggctggcgctgttgttctgatttgagggaaaacatgtcggatgcg
gcgtaaacgccttatctgacctacggtttgtatcgttgtaggcctgataagacgcggcaagcgtcgcatcaggcattacaaggggctgctattta
ataaacgtaaacgccgtagttacccgcttcacgccgctcacccggctggcaatatctgccgccgctttcgcttcacgttcagtcaccagccccat
caggaacacttcaccgttttcggtggtcactttcacgttggacgatttcaccaggtcgctggttaagagctgcgaacgcactttggtggtgatccac
gtatcgttagatgcttcgcccagaccaatcggctggccctgacgaatctcgttatacacttcgttggcaccgtctacgcccatagcaatctgtttgg
cgcgagccgaaagttcagcatttggtgactgcccaaccagcagcactttgccctgataggccgttacattaatgcgcgcttctttcttaatctgttc
gtctttcgacaatgcgctgttcacgcgcacttccagggtaccatcgtccacctgggtgccgacactgcgtgggtcagttgcggctttggtacccac
agcagcggtacccactacggcagcggcaacacaaccttgcaacagcagcgcggaaataaggactgcgattggcgataatgccttcatgtat

tctccttaatcatcctggtgagggaaaagcgtgttatcgatcagatcgcacaggcaatttaccgtcagcatatgcatttcctgaatgcgagcacta
cgatgcgaaggaatgcggatctccacatcctgtggccctaacaaacctgcaagttcgccgccgtcatagccggtcaatgccacaatggtcata
tcacgcgtaacggcggcttcaactgctttaacaatatcgcggctgttgccacgggtggaaatggctaacaatacatctcccgcatgacccagc
gcccgcacctgttttgcatacacttcatcatgtaagcgatcgttggcaatcgccgttaagacaacattatcagtatttagtgcaatggcaggtaag
ctgggccgctccgtttcgaaacggttgatcatgctggcagcaaaatgctgtgcattggcagcggaagttccattaccacaacagaggattttgtt
gccattgagcagagactgaaccagcgtcatggctgcacgggagatggcatccggaagcgcctctgccgccgcaatttgagtttgaatgctttc
agtgaagcaagctttaattctttcttgcacgctaatccttaaaccttaattatgagtggtcattaaaggcatccttaatccactcaacctcattcccggt
gaaggctaccacatcgaaccggcaatccacagtatcaaaactcccattatgacgcgcgagccacaagcgggcagtctgtaataatttgtgttg
tttgctgcgggtcacactggctgccgcgccgccataaagcgcagagcggcgatagcgtacctcgacaaaaatggtggtccggccttcacgca
ttatcagatcgatctcgccgccacgctcgttcacgttagcggcgataaaccgcagtcctttgccttccagccagcgacgcgcttgtgcttcccacg
catcgccggtctgtttggtggttaactgacggggactacctgaccttgttggtactgtagccatgataagttcctgttaatcacgcaatccgggttag
ccgtcaggcttccggtattaccgttgatttcaaaaccctgaacctggcgcatttgtgagaaatgatttgccagcgaccaggcatcgacgcccatc
gcatacatgcgagccagtgaataatcgttattcaccgcgctgagtgcctgctgcattaacggtagattaccgcctgccagcatcgggatttcgct
gtactgcaagccttccatctccagtcggaaatccgggccagcggtcccttgcgcactgcgggagctggcgtacagcgttgcaccgctctggct
accgttacgcatggcgatcatcggtttgataaaagcgatttcacccggcgttgccacaatgtacaccgcatcgacacgaccgccattattggtg
aactggtcatcggttggcgtggtttgcagcgttggattgttggtcgtcatgccggagtcggttgtcgctctgagagtaatcgggctacccgttaaag
caataccagaaccgccgttaacacccgcgcgtaattcgctggtggaaccaaatttttgttgcagaacggtgccgccgcccagtttctgccactct
tgcgcaaacgcattggctacgcgatcgcccaatgaactgcgtgggatcagcaccagcggcgcttgtttaccctggtcacgaatatgacgcgct
gcatcgcgcgcttcgtcttccggtgaaagcgcgaagtaacaaatattgacgcgattttcgatattctccggctggttcagtgccagtacgttcagc
ggagtgttgctcttcagcaactcttcaacgttatttttcagcaacggaccgaccacaatactcgcgccatcctgctgaacctggcttaagatctggc
taagtggttgtgatgaggtatcgtagattttcagctctgcggatggatttgcgggtgcgcttaccgctgcggtgcttgtcgccggggcgcttacagg
caccggctgggctgcaggctgttcaccggtcagatcgctaaccgaggcttgtgccgggctggcaacgccatccacggtttgcggctgaggttg
ttcagctacgtctgcggcaggtgcggcagctacctgagctgccactggctgagtgccgatatttttcgccgcttcaaagccttgctgaatagtgcg
accaaataccgctgcctggccattcagtggcaacagcagggcgattttgttggtcgaggctggtttaaacgcttttacgttaaccaactgcgttgg
cagcattttcgcgcccggattgttcggataacgtttctgccagtcggcgatcccggctttcatcatgtcgggatcgttacggttatcaaaccagacg
cgctgcagatccagccagccttgcagaatattttcgtcggcgttgatcaccagcgtattcgcctgttcctgagtcatggaggagagcgcctgcca
ggtggcatcaatattctgctgtttttctttcgcgccaagcagcggttcctgagcaattaacgcgcgcagtaaatcaatggaaggacgcccctggct
ggcatcgatttttgcctgccagtaacgcgcttgctggttttgttctaaatcggcaggtgtgattttcgccagcaagttttgcgcgccagcaaaatctttc
tgcgccagtttaatctctaccgccagcagtgttttctcgcgacgctgagcatcgttcagttcttgcggtagttggttaaacaactcaaccgcctgccc
ggttttaccttctttcaccagtgcacgaatggcgagtaattgccagttgatcctggtatcatcagagctttgctgcatctgctgaagataaaaggca
gaatcagcctgcgccgtgccctgcatataagcagtggactgatcgggagtatgggtgccacaaccggcgaaaatcagggctgccagaaca
acaggcagacaacgcgcggctttcaaacgagaaaatgttgagggtaccataatgtatccagtgatattttttttacgcaatgctcaatattaaatc
ggcaatacggacgacacaatgaaacaacaccaatcggcggataattctcagggccagctttacattgtaccgacgccaatcggcaatctgg
cggatatcacccagcgtgcgttagaggtattacaggccgttgatctgattgccgccgaggatactcgtcacaccggtttattgctgcaacattttgg
gattaatgcccggttgtttgcgctgcacgaccataacgaacaacaaaaagctgaaacgctgctggcgaagctgcaagaggggcaaaacatt
gcgctggtttccgatgccggaacgccgctaattaacgatcctggctaccatctggtgcgtacctgccgtgaagcggggatccgcgtggtgccgc
tacccgggccgtgtgctgctatcactgcgttaagcgcagcgggtttaccctctgaccgtttctgttacgaaggctttttacctgccaaatcaaaagg
ccgccgtgatgcgctaaaagccattgaagcggagccgcgcacgctgatttttatgaatctacccaccgtctgttagatagcctggaagatatcg
ttgcggtattaggcgaatcccgctacgtggttctggcgcgtgagctgaccaaaacctgggaaaccattcacggcgcgcccgttggcgagctgc
tggcgtgggtaaaggaagatgaaaaccgtcgcaaaggcgaaatggtgctgattgtcgaaggtcataaagcacaggaagaagacttacccg
ccgatgccctgcgcacgctggcgctgctacaggcagaactgccgctgaaaaaagcggcggcgctggccgcagaaattcacggcgtgaag
aaaaatgcgctgtataagtatgcgctggagcagcaggggtaacagtgccgaagcgttgcgttaatcaaaacgcaacgtcttcttttaccgcac
gatgacggtaatggtggctgtgccctgaaattttcctgtttccagctcaccaccgaccagattaactggccaggcgcgcatattgacggtgccat
gcccggaaggatcaacgggaaggataccgttctgaaaaggaatatttgcaactccgccgccgagattttcaatacgaataccgacatcggg
cacattgtctgaacttcttcgcctggcgaccaggttgtactggtctactaccccagttgtaccgtcgatgcgcatttgcaacgagtttttaatctttgaa
gtatcaccacagtcataagtgatatcaaagtctactggagtaaaaccgtctggcatggcattgcgggtggtaaacttctgaccattgatgaatcc
aaaattaaccttaataacatcgccctgattaattttgcacgactgtggggcggtgagatttccctgaatcgataacttgactaaatcatggaagtca
cctgtagacgggcttcccattccatcgacccacgcgcctttaatgacggcaatatccgttttcggaatggtcatcgagccgagaaatggcttggt
gacatacagggtaaaggtggtgttcgcaccaatattaacgggtccataacgagacgtatcaccctggcaaacacccgtttgggttgcagcag
aatataccgtgttgtttttatctgtgtgaatcgggtgttaacagagacaacctggtcggttgtgagtcctggaatgtcgtttgcctgtaatgtggttttaa

tatccaggctgtcattgagtttgtaatatcctgtctgatgtccagtggtggtaagtacggggctgaccatatagacaagtttaactgccttacccgca
gagcaggaacaggcgtattgccagttagtgctacttagcgttgcagaggtaaactcccgtcctgtcacgttatctgtctggttcaccgtaacatcat
caaccacactgctgtaatttgtcgcaccgctagtgctgtcggctccacaccctcctgaggaggcataagcgcaggatgtggatatcaacaaaa
gacctgttataagaggcgctcttttcattttttatccttaatggcactgcgctgtgccttgtaatacagaggttgtaggttctgaatccggtgtactgaa
agcaaactgacattgttgatctgcctgatttccccagcgtacagtccagctttgtgatttactcgatatcccagagagatatagtatgccgccctcat
cgacgatactttgttgccccgtatcatcgttgctggcaagagcgccaaagggcaacggtttaccgttgcgatcgctgactgtaacaagtacgcg
ataaccgatattggcgttgaaacgcgccgctaccattgcacctctgttgggcaccacaaactgtgttgtttgttcaagatcgacgttatcgggcag
ctgcgtagtatctaccgagagacggttttcctgataagttgtgagataaggaaccactgcatagccaaaaggatcggtagcaatccccggata
gttttgtatcctcacgccagatgctccgttagcatcgataagcgcaaaagcgttgcccagatattgcgagagcgtcacgccatgaggatggata
accacgccgccggtaacgccgtaattgtattggctgctgtcattaccgtagctgtatccggcactgaaggtgccataaggtgaacggtaactgg
cgttcaggctactgttatggttgttatcgtcatccagactttgttccaggctatagctcaggcgaccatcatccagcagtgagccatcaagacgcat
ttcatgttgggttgggcgatctttctggctggtcatctgataggaaacccggctacgcggtaaccagcgttctaacggaatactgagattcaaaga
gagtgcgcggtcattattttgatcagtgtagcggctgtattgataattcaggctgtaaccaactccccattgctggccggaaaccccaacagaga
tattcctgtttttatcgttattgccccaatagtcttgctgcgaaccggaggcatacagacttaccccatcaaatattgtctggctgatgttgaattgaatt
tcactttttggcgactgttatagtcccaattgcgggtattggcttcattaaaactaaaatagccatcgttggtatagcgatagtagctgacagcgat
attggtgttggtttccgggatatctttgatgtactgcgtacgccattgatagccatgaaaagagtgctggttatcgaattgggtgtcagctctgttgata
tccatcgacaacgcgcccagtgcgccaagtgtgccgccgataccgatccccagcgcataataatcttcagaaccgagcaggccgccataca
gcgtaaaagtattattcaaaccatatattgccgtggcttcagcaaattcgggttccttgctatcactgtttgcatcagcgcgatagcgtccagcggtc
gcgctatactttagatgcccaggtcgctgcatcatgggtaatgaagaataaggctggataaagcgacgttgtgtaccgtcactttcttcaatcgtg
acttctaaatcgccgctgttggaagaggggtagagatcgttaatttcaaaggcacccgctgacacgttgctttgatagatcacataaccattttgc
ctgatagtcacgattgcactactgtttgcgataccgcgtaccgttggggcaaatccgcgctggctgtttggcaacatattatcgtcggaagcgagt
tgcacgccagtaaaggtgtagctggaaaaaatactgccgctggtggcgctttctcccagaagcaactgagacttcaacgccttgatatcacgtt
gtaaataactactgatggtattccagcttgatgtctgatcgttgcgtgtccatgtggaatagttgcgtaatcgccaggggccaaaattagcaccattt
tgcatattcaggtactgtcgttggctacgattgccctggcggtaacggttatcagaacctgtaaacgagtagttggtaaacagcgttggtataccat
cgtcccaacgagaaggggagacgtaacctcttgcatcacggtacagtgcaatttggggaatgctcaaattaagttgctgatgattgaaatcaa
attcagcagctgtaccgggaatgatttgttcaagcgagttgatcacgctatcgtcatctttttcagccagcgccgggatttcatccaccttaataccc
agctcacgtagttgttctaccgtaaactgtggctgcagaagttgctctgcattggcggtaaatgtaattttttctgtgaaaccttctttttattcagccag
atatcaacctgataagttcctggtagttgtgcatattttttgaaaaacgcgacagatcggttgtttgcataccactttttgtggtttccagcataatggg
gtcgaaataatactcttcggcgttagcggtagtgcaaagcatgccgctgccagtgagcaccatcggcaaacaacgtttgatgatgagcgcca
actgtttcggtgtgcgtttatgtccctggtggtgtcgttgtggcattccctgatgacctgtgtttttatatttttttattgcagtggacgagtttctgtcccggaa
ctgccgccataatcattgatcgcagcccatttcacgttctttccgccagatgcaggaagtggtaactgtttatgttctccgggcgcgatatattcaatt
ttgcttgtgagatcggttttatcgactgccagtccggcaaatacgacgtaatattcagttgggttatatatattgagtgaatggtcactacgtttaaatt
ccagtgatttccacgcatcgtttacccgtcccttcaggtgtgcggggcgataaaacattttgatccgtgttttaaataccagcgtcaactcgttagca
ttaacatcatctgatttttctttgctggaatggcacgaacattaatgtagaacagcgactctttattttcaggcaggttatcagctgttttaataattcgc
agtgatgagtcatcgccagcctcaagacgaaatagcggtggggttataataaatggggcacggcttttaccatctatattatctacccatgattgc
agtaaataaggcgtttctgcgcctttgtttgcgacaggtaacgcagcctctttgcggctggcatcatagatcactcgcgttcgctgtaaaacgattc
ctcctgcaagcgcctggccaatacagaagctacacaacaaggttaatatcaccgcgaatgttcgttttgacatagttatttttcaacctgatacaa
cgagaaagaggaaggaaaagggcgttatctgacaggtcagataacgccctaacgtattaacggtaatccagcgttacgttaacgatagcgt
ctgcttcccctggggtgatttctgatggaactacggttgatacgtagtaagcgatgaggtcaacattgacaataccattggattccgccgggatct
gataaaccgtgttatcattgcccatagcattcagtggcataatgtttccgctttccgcatcgtcaatacggatacccacacctgttgccgctatgcca
gcaaaagagcctacttttgccagattagggaagttagggacggtgccattatctcgcgtttccagccatagacggaaagacgcttcaacgttgc
cgttatcgtgctcccacccagtcattgggcagttaactaaaggaatagtgaatggaatttttgggctacgctcgccaatattacgaaactgttcgg
cattatagtgaccaagagcaacttcaattttcttatcagagtcattgatttcacaagcggattttaaaatctcgccatcaaaaactatttcaccatcg
gttgcagcggcattacctgcgaaaagcgcaagtaagcccgcaatagctgtttttgtaactttattcatatataaccttatccttgtaacaagtacata
atcatatttaatgtgaatatatgttcaacatctttaaagattatacaggcagaaatatttcattgaacattaaatagactgaactttaagattatatttat
atgcaaatatttgatattttcattatattgatatcaattttctatgtattcaaaagagtataaatgcctgactttgctgcgccaggggggcgagtcagaat
acgaggtgatttattaatagaacaagttatatgtatatctaaaattaaattgttgaacataactccttttgctatcggcgttatgttcaacccagtcaaa
tgttatttaatataatgcgtgactgttacataagttaatttaaacattttgagcaaatttttcatctggttaggtgttaattaaacaaataaagttactgtgc
agccataaaaatgaagccgggatagcggtagagactttagccgtgagaaaacgatctgtcgcatcctgctttccttcgccagtcaccagtaac
aaaacttcgcgggcattgagaatatccttcaggcctaaggtgatcccacgagtcacggggcgacccgcggtttttaacatctcatgttgctgtgtt

ctggcatcaagttgactgatatggcaggccggttgcaggctttctcccggttcgttcagcccaagatgaccgtttttccccaatccgagaacgcat
aaatccagaccgcctttgcgcgcaatcaggttcgttacccgttcgcactctgtctcatttatctcttcggagcgaaagctgatgagctggtcttcacg
tagccccagcggctgcacgatatgctgttgcaggaaagtttcgcaggtgcctggcatcgttaatggcagatccacccattcgtcgagcttcacg
aaggtgagctggctgacatcaacctgctgctggtggatttttctaccagataatgatacgtcagtaatggcgtggctccggtcgccaggcaaat
cacggcattcggtttgctacggatcacggccaataaatattcgctggcacgttcacttaacgccgtatagttttcaacttgctgaagggtttgcaca
ggatgaaattccttaagtaaagaggcaccaccagacgcagtgcctcgttccatttacaaaatgccgaatgcggaacaaacaatagagagca
caaaagtcacgccgattaacagcaccggatgcgctttttcacccgcaggaagtaatacatcagcagggtgtaggccatcggtaaaatgttcg
ggaagactttatcgaagaaatcctgctgcagcgcaacgctgtgggtattgtcgatggcaaacgatgtcaccacgttaatatgcacatacgaag
cgatcagcccgccgattaccgtgatcccgaggatggttgccgaacgggcaatcatctgcgagttctctcgcactttatcgatcgccttcacgccg
actgaataaccgacgtgggtccagccgacgcgcaggaaaaagataagcaggtaaacggcgaaaaatagaatcggccccagcaggtttc
cctggctggcaaatgatgagcaaattcccgccataatcggcaacaaagtaaaccagaaaatcgcatcgccaatcccggcgattgggccaa
acagtgccactttgaggcctttaatggtgtcgcggttttctcctttttcttccatcgaaattaataaccccatcaggaatccgaccagattcgggtggg
tattaataaattcgaggttatctttcatcgccgcgcttaagcccggtttgtcgtccttataaatctttttcaggatcggcaacatcgcccaggtaaaac
cgcccgcctgcatcctttcgtagttaaagctcgcttgcagcagcgacgaacgaaagcccagacgggtgatatctttttactgatttcagatccca
ttgctgtagtcctcttcttcatttttactggcaacgggctgcggttgcgcttgctgccgggatttcgcattgaaaaactcatacaccgcaaagcctgc
gcccagtacggcaaccggcaacaggttgctgacctggatgtagcaaacaaacaggaaaccggcgatcaggtaagggatatattgcgctttg
aacattacgcgcagcagcaagccaaaaccaacggcaggcagaataccgccagccacttcaaagccgtgggtcagccaggcgggcatcg
ctttcaccagcgcctgcatcgccccctgtgccaggtaagtacagaggaaagcaatcaccgcatacgctgaagcgacgatgagcatcgttgtc
cagttaagccgggaaaacgctgccgtatccgcctctttcgcgcatttatcggctttggtcataaataatgagaaagcggaatagaagaacaga
atgacgtactgcattaacaaactaaacggcaggccaagaccaattgctgttttggcatcaacgcccgtagaccatgcaatgacggtggtcatc
agacccgccataatcgggttgggcggctgaacaccacctgcaggggttaatccggcgaaagccaactctgtcagaccaccggtaattaagc
cagtctgaatatcaccgagaatagcgccagttagggtacaaacgattatcgggcggaataaaaataaggcttccagccaaaaatcaatccc
cagaacaaaaactaacgccgccagggataatccctgaagtagggttatttcatgcatttttattacctcaaaccaatattaaggcgattttagattt
agtcagggatttgttctttttgatcgccagggacgtcctgaatgaaaacattcacgccacgttgtttaataaaacgtaagtccgtgagatcctggtc
atcgacataaactttactgctgatttgctttttcccttccgagaaatgcatattgccgacgttgacatctttcaggtcaataccaccttctaccaatttac
gtaccgtttgtggcgtacggcaaatcaggaagatcttctgatgtggtgcagctttgccgatgacgttaatggtttttcgatagtaaagaaacgaat
gccaaagccgtaggtttccgcggtaatacccattaatttctgttgaatatcatcgttagcgacaacatcatccacgactaccagcagatttgcacc
gatggtggatgtccaggtcacgccaacctgaccgtgaaccagacggttatcaatacgggttaagagaatatttggactggtcattgtgtaatcct
caccataaagttgaataatataatttttttaaatatattattgttgcgatattgtagtgatgtatttattaaggtttagttatctccttttccattaaggcactc
cccgatattaatcgggaagtacattgggataaatcataaaaaaactaatgattatgctgaaattcgattcgctgaaccacagacattaattttatttct
gacaacttctttcatcgcatccattccgacgcgcatataataacgaggatcattaccctgcggattttccgcaaaccaggctttaaccgcgccag
cgaaggctatttttaattctgtggcaacgttcacttttgtgacgccaagttcaatagtgcgacggacaaattcatccggaacatcgctggcaccat
gcagcaccagaggaacatccaccacttcacgaatttccgccagccgctggaaatcaatcttcggcgttttgctgtataagccgtgcgccgtacc
aatcgctaccgccaggctgtcgacgccagtcagttcgacaaagcgtttagcttcttgtggatcggtcaggaatgcactttcggcgtcaacgctca
tgtcatcttcaacaccgcccaggcggcccagttctgcttccacgctgcaatcttgtgagtggcagaagtcaacaaccgatttcaccagcttcacg
ttctcggcaaacgggaagtggctgccgtcgatcatcgcactgcgcacacctgcgtggactttacggcgaatatcatccagcgattcgtggtggt
cgagatgcagcgccagtggcatgttgtaggttgtggaataggcgctacacagggcgtagatctcttccagcgcgatgtgtttaaaggtccccgg
cgttccggcgaggatcaccggcgatcgcatttcactgcacacttcgaggatcgcttggatcgtctcggcgttatgaatgttaaaagcaggcacc
gcgtagccattggcctgggcgtcctgtaacagatatttagtggagataatgctcataatgcgatcctcttatgcctgccacggatgaatgattacg
ccttgcaccacgcggttaacggtgccactggctgatggggtatccggcgtattgcccatgtgcagcgactgcatcagtgcaaacgtctgggcgt
acatcaggaagcaaaatgcctgctcaacgtcgataaagtgacgtgacggtggcaggatgatatgtggaccggcagcgacgatgtcgctgctt
tccgcggcgatggcgattacacgcattgcctggttgtcacggcgaagttcagccagcagatcaagatcatactgacgggtgtaagggtggctg
gagacaaataccaccaccagcgtttcgtcatcgaccagcgattttggtccatgacggaatccggttggagaatcataaaaggccgccagttta
cccgccgtcagttccagcactttcagcgccgactcgcgtgctgcgccctgtaagccaccgctaccgagataaacgatccgtttccacggtgcgt
aaccaaacacaccttcgctgaaatcgcccagtgaggtcaggatcgcctggcaacgatccgccacgtcgcggaaggtttggctgttgatcgtct
caggtgcgaaaaccgcgaggcagctggccatcatggtggtaatgctgctggtcatcgcaaagccgcgatcgtgcgtttctgcgggcatcagc
agggcaaacgcgttatcgctgttgatcgcgttttggtaaagagcgcccgcttcgttgcaggtgatcggcaggtgatagcattccggtacaaattg
atttgccagttccacggctgcgacgctttccgggctgttgccggatcgaccgaaggagatcaacagcagcggatgagctgggttcaggtagtc
catcggattggtgaccagatcggtggtcggtacggcgctgaagtttttaccggtatggctggcgagccacggcgcgatgatgtcaccgataaat
gccgacgttccggctccggtcaggatgatccgcagattctctttgcgcagtaacggttcaaggaagttattgagcgcggaacgtagcgcgtcga

tgttggtgagtgaacggatccatgcgcgaggctgatggcggatctcttcttcagtccatgtaccggttgcggcggcagcaggggtgtaattttctg
gcataactcaatccttagtctgatgaacttgacgtcgggcgttgtcgctttcacttgcctttcgtttcatttcacttggtgatgtctttcgttgattaaaatct
atagaaaagaaactgaaagttcaatggagaaaaaagaaataaaacgaaatttgtgatcgttgaaagaaaagataaaggaagcaaaagg
gattgctggatgcggcgcaaacgctttatccggccttgcggtcaccaactgtaggtcggataagacgcgttagcgtcgcatccgacaccgctg
cacagatcaaaacgaaagaaaaagaaaggccttacgggggaagtgaaggcccaataaaggagcaaactatcaaaacgaagctaattg
accctgaatccagatttgttgcacatgtagcccgctatccagcgcaacgacgctggcgcgtttgcccggtttaagcgatcccagaacaccatca
acacccagcattcgcgccggatgcagcgacgccatatggatggcttccgcaggcgttacgcccgtcaactcgaccatattgcgcactgccgc
atcaacagacagcgtactgcccgccagcccaccagacgcggtacggacaacgccaccgtgcatctgcacttcttcaccacataacgtatag
cgaccatccggcatcccagctgcctgcatcgcgtcggtgatcagtacgattctctctttcgcacagcaacaacacagcgacattgccgccggat
gcacatgatgaccatcggctatcagttccagccaggcgcgcttgtccgttaatcccgcgccaaccattcccggttcgcggtgatgtaaacctgtc
atcccgttatagcaatgcaccaggccgtcggcaccagcatcaaatgcggcgcgggtttgttgccaggtcgcagcgagacatcacgccggac
atcaccgagaacaggaaggtaatccccatctgtactgcgacagcgaaaggtacggcgacacctactgcgacatcgggtttcacgccagtag
taatggcaaacgccgtgccgacgatagtaccgataatcacgttaggcggctgtgcgcccgccagcggggccagccccatccacaccagttc
cagcgtaccgccggttaaaattccggtatgcagatcgccaagtaccagcccgaccaacgggccgaggaccaccgggcggtgcatatgggt
taggccgttaaacatatccaggccagcgataaaggcgataatgcccaacgcaaatgcctgcaacaggctgatttccatgatgaatccctcaa
agtaatttaaagaggtccacagcaggctctgtcgggacgccctgaacgaagcattccaccccagcggttttcaggtcgttaaatgctgcgatat
cgcccgcatccacagaaaccgttttggcgatttgttgtttgccattggcgtagtgcatattgccaacgttaatgcgattcaccggaacgccaccttt
caccagcgtcaggaaatcggcgggtgttttacaaaccagcaggattttctgtcgatcggcggcgcgatgaatgttgtcgataactttttgcagcgt
ccagaaacgtacggcgatcccttctgccagtaccatttccatcaggttttgttgtaccggatcttcggcaacctcatcgttggctaccagcaccag
atttgcccccgcaaatccgacccattgaacgccgacctgaccgtgaatcaagcgttcatcaatccggcttaaaacaatatttggcatagcgtgtt
cctctttgttttgcttattggccttcacaggctgtgtggtactgcgccaggatgtcctgaatatggttgataatgagttcccgtggcgttggctgcagct
cgccggagcgaactttcacgtactgcagcggcagatactggctgatcagcggcagcggaattggtgaatccgccagattacgtaccagatga
gcgaaagcgtcatcaatctggctgtccggccagtaatagcgcacgcgatccgagtagctataaccacgcgccagacgacgcgcgttgccgt
caccgtggtagtggctttgccagtattccgggcggtcgagcatcacgtcttccagcacctgacgcagaccagaacaggctttcgctggcacca
gttcttcttcaatcgccgccagagagaacagagcttcacgcagggcgaaggtcagcgctgggccaactttcagaatggcaaagtggtcaatc
accagctggcgcagcgattgcggcgtttgataatcggtagagtgcgcttcgaaaatcagcgtttcgtagttttcgaccatctggcttaaggcgctc
gctttggcgggctgataatcaataacgttggtgtgatcgaattcgacgccgggttgaaccaccagggcaatgatgcgtggccagatggcattca
aaccttgcttttcaaaggcgtgacgatgggcttccagcgtggcgcgggcggcatccggcgtggtgaccgccagctcgcttaaggtttcatgcgc
gccgccaggtaccggcacttcggtaccaatgacatactccagatcggcttcgccaaagtgttcaagacaggtttcttccgccactttcgccaga
cgggcggcgcgttcagccacgatgtcatcagttaagggaatcggatcgtcctgacaggacatgctgcaatcaaggtggatttttttgaatcctgc
cgcaacgtagcttttaatcaaatcatcggcattggccattgcctgagcggccggcaggttttgccagcggtttggccccagatggtcaccaccca
gaatcaacgcatcctgcgggaaattcaacgagtcggcgagctgacaaacaaagccgcgaaaatcggcgggcgtcattccggtataaccgc
cgaactggtctacctgattggaggttgcttcaatcagtaacggcgtttggtttgcactggcgtagcggattgcagcttccagcaccagcggatgtg
cggaacaaacggcataaattccatttgttttgcccgctttgtgctgtctcaccatttctgtcagatgtttcactttcctctccagttcagattactgcgata
ttaatctttcgttttgtttcagttgatactgcgtcatggtgagataaaaataaaacgaaagataatggttttctgatctgattcacaaaagaaacataa
tgaaagtcactgaaacgagagtagcttgcgtcaatgggcaaggtgggcttgcatttgcttaatagaaaggcgttaataggcaaaacgaaatg
aaacgaaagttttacgaaaggacttactatgagtaataccgacgcttcaggtgagaagcgagtgactggcaccagcgagcgacgagaaca
gatcattcagcgtctgcgacagcaagggagtgtgcaggttaacgatctgtcggcattgtatggcgtatctactgtgacgatccgcaacgatctgg
cgtttctggaaaagcaggggatcgctgtgcgtgcctatggtggcgcgttgatctgcgatagcacgacgccgtcagtcgagccatcagtggaag
ataaaagcgcactgaacaccgcgatgaaacgcagcgttgcgaaagctgccgttgagttgattcagccaggtcatcgggttatcctcgattccg
ggaccaccacttttgagattgctcgtctgatgcgcaagcacactgacgtaattgcgatgaccaacggtatgaacgtggctaatgcgttgctgga
agcggaaggcgttgagctgctgatgaccggcgggcatttgcgccgtcagtcgcaatctttttacggcgatcaggctgagcaatcgctgcaaaa
ttaccacttcgatatgctgtttcttggtgtagatgcgatcgatctggagcgcggcgtcagcacacataatgaagatgaagctcgtttaaaccgtcg
gatgtgcgaagttgcggaacggatcatcgtggtcaccgattccagtaagttcaatcgttccagtttacataagatcattgatactcaacgtatcga
catgatcattgttgatgaaggcattcctgcggatagcctggaaggactgcgaaaggctggggttgaagtgattctggtcggggagtgagaaag
gatgcccgctggtcaacacgaatgcgttcggcgggcattgtgaacaccatcaatgggtttcttctgtttctcgggtgagggtttcccagtcggcag
gaggatgtcctctttttaacattttgctgaatacggtataggcatctgttttttttaccgtaggtgcgcagagtgttttcatcgttcatccatcccagaatga
tgactttctctttttcactataacgaaagaagagacgataacgaccagcaccaaatttacccgtgaccagtctttatttttcccagagcctaacga
cttgccatgacggaatgccggtgatgatggattgaccgtgatatgctcctcaatcactttatggactaccgccaataactttgtggcggcttttctctg
ataattttcaggatcttttccctttaatgtctcgacttcggcaactaaagcgtcgtaggtttcctgaaaacagggatgagcatatagcgcccaacca

ttaaccctttgtggaaaatccattattcgtcgtcgccaatctcatcatcaatgttgacgtccatgccagcgacaagtttctttccttgttgaatgttgaat
ggacgagtttttgcgggttgttctggatatctgcatccagaaaacgcaaaaatgcattcatagtatgatcctcctgttcatctcccagtcggcacat
aaatacttgcccaccaggcagaatttcgtaatgaatgctgtcctggcctggcttcagttttaaggcctcacgcactggcgcggggatagttgtttgt
ccgcgtatcgtgacctttgattcagtggtcagtacagcgtgagagcgagcattagcgggcatgattcactgtccttttacagcctgttttctgctcaa
attataagcttgaactaaggtaatgcaaatgcattattaatggacgtgggggctttaaacaataaaaacatggcactaaggccatgttttgcgag
ggacgttccaaaagaaatcaggtcaccggtgccgggttaaacaccgccagctggttatgcagcccccattgatccgagaaggttttcttcttcc
cgctggcgacgtcgagaataaagtggaacaacttccagcccacctcttcaatagtttcttcgccggtagcgatggtgcccgcattaatatccatt
aaatcaaaccagcggttcgccagctcggtgcgggttgccattttaatgacgggtaccgccatcaggccgtacggcgtaccacgaccggtcgt
aaacacttgcactgtgatacccgaagccacctgttgcgtgccacagacaaaatcgctggctggcgtcgcggcgtaaattaatccgcgtttagtc
gggcgttgaccgggcgacagcacttcaacaattgcgcttttacccgatttagcaatggagccgagtgccttctctaccacgtttgccagaccgcc
tttcttgttgcccggcgaagggttggcgctgcggtcggtttttcccatattgagatagttatcgtaccactccatctcctccagcagccgtttgccgac
ctcttcgttcactgcgcgtggtgtcagcagatggatcgcgtcacgcacttccgttacttctgaaaacatcaccgttgcgccgcagcgcaccagta
gatcagacgcatagccaaccgccgggtttgccgttacaccagaaaacgcatcgctgccaccgcactgcataccaacgaccagttctgaagc
cgggcaggtttctcgctgccgttgattcagtttttgtagatggcgttcggcgatctgcaaaatatcctcgaccatggactgaaaaccgacatgctttt
catcctgcaaactgacaatgctggcgctttctactggaatagcttgcacatcatccgttccagtcagcaggcgctcaggctgcaacttttcacaac
ccaggccaatcaccattacttcgccgccaaagttaggattcagcgaaatattgtgaatggtacggataggtacaactgccgccggtgcgttaat
cgccacgccacaaccgtacaaatgattcagccccaccacgccatcgacgttcgggtatttcggtagcagatcgcgttcaatgatttttactacat
agtccaccacgcctgccacacagtggacgctggtggtgataccgagcaggtttttggtgcccacgctgccatcggcattgcgatagccctcaa
aggtgtatccttccagcggcggtaagggttccgggactttggttgccagtggcagcgtgtgtaacggcggcgcttccggtagtacaaccattgat
tcgtcgatccagcttccgcgtgggattgcacgcacggcgtaaccaatcacttcgccataacgaataatttcaccattagccggaatgtccagca
atgcgactttatgcccctggggaatatgttcaattaattccagcccatccggaaaacgcgttcctgctttcaggccattatcattaacaataattgcc
acattatctgtgtcgtgaacttttatataaaacgcagttggcgtttcttgtctgatttcgatgttggccattgtgcaatattctccagccaggggttattaa
aatgaggacctggtctcatttaaaaataaatgacttcattgggtgaaataagaatgtcaggagtttaaacgcagaaatgtgatcaatgtcaatca
tttcagggattgcagtgccgcccggtaaggtaaagcgcaaatctgtgctttagcgcgcaaccttatgtgcatatgctcaaaataatatttaaatagt
attaattaaattagcatttgcacaatgattattattctcgcgctgcttatactaattcccgagttcattgtcgttacggcataaatgaaactcataaata
acaccttccccataataataaaaatcagcataagtacccgaggtaaataaaatgattctggacaccgttgacgaaaaaaagaaaggcgtgc
atacccgctatttaatattactgattatttttattgttaccgccgttaactacgccgatcgtgcaacgctgtctattgctggtaccgaagtggcaaaag
agttgcagttaagtgcggtttcgatgggttacatcttctccgcttttggctgggcctacttgctgatgcaaatccccggcggctggctgcttgataagt
ttggctcgaaaaaagtttacacctacagcctctttttctggtcgctattcaccttcctgcaaggctttgttgatatgttcccgctggcctgggcagggat
ctccatgttctttatgcgctttatgctcggcttctcggaagcgccatcattcccggcgaacgcccgaattgtcgccgcctggttcccgacgaaaga
acgtggtactgcctccgccatctttaactcggcgcaatatttctcgctggcgctcttttcgccgctgcttggctggctgactttcgcctggggctggga
gcacgtctttaccgttatgggggtgattggttttgtgctgacggcgctgtggatcaagttgattcataacccgacagatcacccacgtatgtctgcg
gaagagctgaagtttatctctgaaaatggcgcggtggtcgatatggaccacaaaaagccgggcagtgcggcagcaagcggacccaaactg
cattacatcaagcaattgctctctaaccgcatgatgctgggcgtatttttcggacaatattttatcaacaccatcacctggttcttcctcacctggttcc
cgatttatctggtgcaggaaaaaggcatgtcgattctgaaagtgggtctggtcgcctcgattccagcactgtgtggttttgcgggcggcgtgctgg
gaggtgtcttctcggattatctgatcaaacgcggtttatccctgaccctggcacgtaagctaccgattgtgctgggaatgttgctggcttccaccatc
atcttatgtaactacaccaacaacaccacgctggtggtcatgctgatggcgctggctttctttggcaaaggatttggtgcgctgggctggccggtg
atttctgacaccgcgccgaaagagattgttggcctctgcggcggcgtctttaacgtctttggcaatgttgcctccattgtcactccactggtgattgg
ctacctggtaagtgaactgcactccttcaatgcagcactggttttcgtgggatgttcagcgctgatggcgatggtctgctacctcttcgtagttggcg
acattaaacgtatggaattgcagaaataagcaaaggtataagcgatgaataacgatgttttcccgaataaattcaaagccgcactggctgcga
aacaggtacaaattggttgctggtcagcactctctaacccgattagcactgaagttcttggtttggctgggtttgactggctggtgctggatggcga
acatgcgccaaacgatatctccacgtttattccgcagttaatggccttgaaaggcagcgccagcgcgccagtagtgcgagtgccgaccaacg
agccggtaattattaagcgtcttctggatatcggtttctataacttcctgattccttttgtagaaacaaaagaggaagcagagctggcggtggcatc
aacccgttacccaccggaaggcattcgcggcgtctccgtttctcaccgcgccaatatgtttggcaccgtggcggattatttcgctcagtcgaaca
agaacatcactattctggtccagatagaaagtcagcagggcgtagataacgtcgatgccattgccgctaccgaaggcgtagacggcatcttc
gtcggccccagcgatctggccgcggcattaggccatctcggcaatgcatcacacccggatgtacaaaaagcaattcagcacatttttaaccgt
gccagcgcgcacggcaaacccagcggtatcctcgcgccggtcgaagccgatgcgcgtcgttatctggaatggggcgcgacgtttgtggctgt
cggcagcgatctcggcgtcttccgctctgccactcagaaactggctgatacctttaaaaaataaccaccaccgcaacaagagagatgattgat
atgactatgaaagttggttttattggcctggggattatgggtaaaccaatgagtaaaaaccttctgaaagcaggttactcgctggtggttgctgacc
gtaacccagaagctattgctgacgtgattgctgcaggtgcagaaacagcgtctacggctaaagcgatcgctgaacagtgcgacgtcatcata

accatgctgccaaactcccctcatgtgaaagaggtggcgctgggtgagaatggcattattgaaggcgcgaagccaggtacggtattgatcgat
atgagttctatcgcaccgctggcaagccgtgaaatcagcgaagcgctgaaagcgaaaggcattgatatgctggatgctccggtgagcggcg
gtgaaccgaaagccatcgacggtacgctgtcagtgatggtgggcggcgacaaggctattttcgacaaatactatgatttgatgaaagcgatgg
cgggttccgtggtgcataccggggaaatcggtgcaggtaacgtcaccaaactggcaaatcaggtcattgtggcgctgaatattgccgcgatgt
cagaagcgttaacgctggcaactaaagcgggcgttaacccggacctggtttatcaggcaattcgcggtggactggcgggcagtaccgtgctg
gatgccaaagcgccgatggtgatggaccgcaacttcaagccgggcttccgtattgatctgcatattaaggatctggcgaatgcgctggatactt
ctcacggcgtcggcgcacaactgccgctcacagctgcggttatggagatgatgcaggcactgcgagcagatggtttaggaacggcggatcat
agcgccctggcgtgctactacgaaaaactggcgaaagtcgaagttactcgttaatgacgtcgtgcccggatggcgtattgcaatccgggcctg
gaatccaggccgaataagagaaacgccctccggcgtcatgtggtaacaggcataggtatgaaaatcgtaatcgccccagactcttataaag
aaagtttatctgccagcgaggttgcgcaggcgatagaaaaaggatttcgggaaatttttcctgatgcacagtacgtttctgttccggttgccgacg
gtggcgaaggaacggtggaagcgatgattgcagccacccaggggggctgaacgtcacgcctgggttacagggccgctgggcgagaaagtg
aatgccagttggggggatctccggcgatggcaaaaccgcgtttattgaaatggcggcggccagtgggctggagctggtacctgcggaaaaac
gcgatccactcgtgaccacttcacgcggcacaggcgagttaatcctgcaggcgctggagagcggtgcgacaaacattattatcggcattggc
ggcagcgctacaaatgatggcggcgcaggcatggtacaggcgctgggggcgaaattatgcgacgccaacggcaatgaaattggttttggcg
gcggtagtcttaatactctgaatgatattgatatttccggcctcgatccgcgcttaaaagattgcgtcattcgcgtcgcttgtgatgtcaccaatccgc
tggtgggcgataacggcgcatcgcgcatctttggcccacaaaagggagccagtgaagcgatgattgttgagctggacaataacctctctcact
atgccgaggtcattaaaaaagcgctgcatgttgatgtgaaagatgtccccggtgcaggagctgcgggtggtatgggcgcggcgctaatggcg
tttcttggtgcggaactgaaaagtggtattgaaatcgtcactacggcgctgaatctggaggaacatattcacgattgtacgctggtgatcaccggt
gaagggcgtattgacagccagagtattcacgggaaggtaccgattggtgtcgcaaacgtggcgaagaagtaccataaaccggtgattggca
ttgcgggtagcctgaccgatgatgttggcgttgtacatcagcatggcattgatgcggtcttcagcgtattgaccagcataggtacgttggacgaag
cattccgcggggcttatgacaatatctgccgtgcttcacgtaatatcgccgcgacactggcgattggaatgcgcaacgcggggtgacaagggc
gcgcaaaccctctatactgcgcgccgaagctgaccagacagtcgccgcttcgtcgtcgtcctcttcgggggagacgggcggaggggagga
aagtccgggctccatagggcagggtgccaggtaacgcctgggggggaaacccacgaccagtgcaacagagagcaaaccgccgatggc
ccgcgcaagcgggatcaggtaagggtgaaagggtgcggtaagagcgcaccgcgcggctggtaacagtccgtggcacggtaaactccacc
cggagcaaggccaaataggggttcataaggtacggcccgtactgaacccgggtaggctgcttgagccagtgagcgattgctggcctagatg
aatgactgtccacgacagaacccggcttatcggtcagtttcacctgatttacgtaaaaacccgcttcggcgggtttttgcttttggaggggcagaa
agatgaatgactgtccacgacgctatacccaaaagaaagcggcttatcggtcagtttcacctggtttacgtaaaaacccgcttcggcgggttttt
gcttttggaggggcagaaagatgaatgactgtccacgacactatacccaaaagaaagcggcttatcggtcagtttcacctgttttacgtaaaaa
cccgcttcggcgggtttttacttttggaggggcagaaagatgaatgactgtccacgacactatacccaaaagaaagcggcttatcggtcagtttt
acctgatgtacgtaataaaccgttccggcgggtttcagattgttgagtgcgctttattcatgccggatgcggcgtgaacgccttatcctgcctacaa
aagcacgcaaattcaatatattgcagagatggcgtaggcctgataagcgtagcgcatcaggcatttttgcggttgccatcagtttcaaacccgc
gtcagtgagtttttgcttttggtgcacagggtaagatgaataaatatctacggtattaataatagactttgtaagaagttattaagtctatattgcattta
aaaggtcgtaatcatacgtatagtcctctctggtaaaatattgcttctctgagttatatgcttcatatgtgttatattccccgacctcaaagccattgtcc
ggagaattttcgtctttctcgagggtattatctgaaagtaactcttctgagctagagtcttctatgtttaaatccatagtataattaatttgggggacgac
cagataagcattttctttggtattttcataatcttcaatgattatatcgatgtaatcatatattttttctgatgagaattctttcatctctgttaatttaattctgttc
aagcaattgatgattttcgatgagcttttatatatttcttttgttaattcattttctaatttatctgtaagagttgcccttggtatagattttataattgcaaatct
cataatttttaattgttcttcggtaatgtctccaggctcatttaaacaactttcgacaaacttgatttttttcttcagaatacatatcgctaatgcttattaaaa
caggcgttatttttagtaaataatcccaggagattgtggcgttttgtgttgaactaacaaaattaacctgaaacatgttggcattagtaaaatttgttg
aattcaatttaaaaggatttaaaatagtattagccatatgtttatctgtgaaatcataataatctaaatcgacattacgtaagtccagagaggatag
atcaacgatatctccagtcccacaactaaaaattacgtttaacctcctgatttcattgtaaatatcttttaatgggcaattaatattgatattacctgtgg
atatttcctggtttttttaacggttcattaatggtaagttcacaagtattgtccgatcgttgaataatttgggcgttataacctaaaaaatccagacgaag
agacgagacattataaagaattacattttgtaagtacgctaattctttttgaaaatcaccagtgataatgcattttccaccaggttgtaacgtgttaag
taaagtgaccatttcaatagaggtctgttttagtttttctatacctggtgctagtgcttcgaggttatttcctgttgggctttcaggaaggtcattcatttttc
gacggactaaatcactactaatatcattaccaattgatgaaacagggaacattattttatcctttataaataatcaataaaaatcattattaaaatga
tcgggtgaaaaaaagagaatttgccaacaatctgaagtaatgctggttttattaaagtgcacaagggggggttaagatggcttcttttcgggtgaat
tttgcacaatacttagaaatcgtctccttaatacattccagctctttttttattgacggctgagcaagtacattgttcaccaaaaggtgcaaaagtgcct
acatctaatgtttgtatgatataatcatcatgttctcctgtagaaatatctataatgaatggactcatattttccggttggattcggatcatgacagaag
gaacatcgccatgatagaaattcaatgcagttattgcagataatactctccgcagttcattacttatactgtaaattttattaacgtcattggtggaga
caatttctgcaaactttctttttaccgtccaataatcaatatttctattgtgattgttaaccactgcgtgcattggcgggattgaagggtgaaaaatatg
cgcaattgggaagcctttcattgatgcctgccttgtcatcatgacaaacaataatagatgcttattttcctcgcagggttctttcgcactatcaaaaa

acgctcatttacccggcgttacagtaaacgaagcaacattctgtggcctacgccgatcaaaagctctgtgaaaatttcccttgttgtctttgtctgac
cttatcgcggcgatcagcaattattcgttttgtgatcctcaacacaaaatgctattcgtcgcctatgaggggagaagaatttatggaaaacgattgc
attcacaaaatgacgtcctgtcagaagagaaaaagttgacgttaatcaattgttcgagatgatcaatacatgtattcgaaattttaaaatattgata
aaataaatgtttgttctccaacaaatagcgtgaaaaaatgtgacatgccgcattatttatcactgaaaatggtttatttatataataaaatttgagaactt
gctcattaaatttaactcaaattttgcctggtaattatccggtaattgcttgaaatatggtctcagccccttttttgtattaaccacataacgaatgatgtt
atcgccataaaatatggttatccccgtcatttttttgacaaaaatcagggtttatgctgattttatactttaacttgttgatatttaaaggtatttaattgta
ataacgatactctggaaagtattgaaagttaatttgtgagtggtcgcacatatcctgttcatttcattttgatacacttcatgccgtcaatgaggtaatt
aacgtaggtcgttatgagcactattcttcttccgaaaacgcagcacctggtagtctttcaggaagtcattagaagtggttctatcggctcggctgca
aaagaattagggttaactcaaccggccgtcagtaaaatcattaacgatattgaagattattttggtgtggaattagtggtgcggaaaaataccgg
tgtaacattaacacctgccggtcaattgttactctcccgttccgaatccattacccgtgaaatgaaaaatatggttaatgagataagcggtatgtctt
ctgaggcggtggtggaagtctcatttggttttccttcattgattggttttacttttatgtcagggatgatcaacaagttcaaagaggtgttcccgaaagc
gcaggtttctatgtatgaagcgcaactgtcttcgttcttaccggcaatccgcgacggtcgactggattttgcgattggtacgttaagtgcagaaatg
aagcttcaggatttacatgttgagccgctgttcgagtccgagtttgtgctggtagccagtaagtcccgaacatgcaccggcaccaccacgctgg
agtcgttgaagaacgaacagtgggtgttgccacaaactaatatggggtactacagcgaactgcttactacgttacaaagaaatggcatcagta
ttgaaaacatcgttaaaaccgactcagtcgtgacaatttataatcttgttctcaatgctgatttcttaactgtaattccttgtgatatgacgtcaccttttg
gttctaatcaatttattactattccggttgaagaaacattacctgtggcacaatatgccgcggtatggtcgaaaaaattatcgtattaaaaaagcagc
atcggttttggtggaattagccaaagagtattcatcttataatgggtgtagacgaaggcaattaatagaagttggttagttatttgtttttatttaaacat
aaataatccacctgtctgtcttgcaggtgtcggttacggttacctacatatttaattcaggcgaagaggttttataatgcatattacatacgatctgcc
ggttgctattgatgacattattgaagcgaaacaacgactggctgggcgaatttataaaacaggcatgcctcgctccaactattttagtgaacgttg
caaaggtgaaatattcctgaagtttgaaaatatgcagcgtacgggttcatttaaaattcgtggcgcatttaataaattaagttcactgaccgatgcg
gaaaaacgcaaaggcgtggtggcctgttctgcgggcaaccatgcgcaaggggtttccctctcctgcgcgatgctgggtatcgacggtaaagt
ggtgatgccaaaaggtgcgccaaaatccaaagtagcggcaacgtgcgactactccgcagaagtcgttctgcatggtgataacttcaacgac
actatcgctaaagtgagcgaaattgtcgaaatggaaggccgtatttttatcccaccttacgatgatccgaaagtgattgctggccagggaacgat
tggtctggaaattatggaagatctctatgatgtcgataacgtgattgtgccaattggtggtggcggtttaattgctggtattgcggtggcaattaaatc
tattaacccgaccattcgtgttattggcgtacagtctgaaaacgttcacggcatggcggcttctttccactccggagaaataaccacgcaccgaa
ctaccggcaccctggcggatggttgtgatgtctcccgcccgggtaatttaacttacgaaatcgttcgtgaattagtcgatgacatcgtgctggtcag
cgaagacgaaatcagaaacagtatgattgccttaattcagcgcaataaagtcgtcaccgaaggcgcaggcgctctggcatgtgctgcattatt
aagcggtaaattagaccaatatattcaaaacagaaaaaccgtcagtattatttccggcggcaatatcgatctttctcgcgtctctcaaatcaccg
gtttcgttgacgcttaattaattcgttgaggataggatatgagtacttcagatagcattgtatccagccagacaaaacaatcgtcctggcgtaaatc
agataccacatggacgttaggcttgtttggtacggcaatcggcgccggggtgctgttcttccctatccgcgcaggttttggcggactgatcccgatt
cttctgatgttggtattggcataccccatcgcgttttattgccaccgggcgctggcgcgtctgtgtctttctggctctaacccttccggcaacattacgg
aaacggtggaagagcattttggtaaaactggcggcgtggttatcacgttcctgtacttcttcgcgatttgcccactgctgtggatttatggcgttacta
ttaccaatacctttatgacgttctgggaaaaccagctcggctttgcaccgctgaatcgcggctttgtggcgctgttcctgttgctgctgatggctttcgt
catctggtttggtaaggatctgatggttaaagtgatgagctacctggtatggccgtttatcgccagcctggtgctgatttctttgtcgctgatcccttact
ggaactctgcagttatcgaccaggttgacctcggttcgctgtcgttaaccggtcatgacggtatcctgatcactgtctggctggggatttccatcatg
gttttctcctttaacttctcgccaatcgtctcttccttcgtggtttctaagcgtgaagagtatgagaaagacttcggtcgcgacttcaccgaacgtaaat
gttcccaaatcatttctcgtgccagcatgctgatggttgcagtggtgatgttctttgcctttagctgcctgtttactctgtctccggccaacatggcgga
agccaaagcgcagaatattccagtgctttcttatctggctaaccactttgcgtccatgaccggtaccaaaacaacgttcgcgattacactggaat
atgcggcttccatcatcgcactcgtggctatcttcaaatctttcttcggtcactatctgggaacgctggaaggtctgaatggcctggtcctgaagtttg
gttataaaggcgacaaaactaaagtgtcgctgggtaaactgaacactatcagcatgatcttcatcatgggctccacctgggttgttgcctacgcc
aacccgaacatccttgacctgattgaagccatgggcgcaccgattatcgcatccctgctgtgcctgttgccgatgtatgccatccgtaaagcgcc
gtctctggcgaaataccgtggtcgtctggataacgtgtttgttaccgtgattggtctgctgaccatcctgaacatcgtatacaaactgttttaatccgt
aactcaggatgagaaaagagatgaatgaatttccggttgttttggttattaactgtggttcgtcttcgattaagttttccgtgctcgatgccagcgact
gtgaagtattaatgtcaggtattgccgacggtattaactcggaaaatgcattcttatccgtaaatgggggagagccagcaccgctggctcacca
cagctacgaaggtgcattgaaggcaattgcatttgaactggaaaaacggaatttaaatgacagtgtggccttaattggccaccgcatcgctcac
ggcggcagtatttttaccgagtccgccattattaccgatgaagtcattgataatatccgtcgcgtttctccactggcacccctgcataattacgcca
atttaagtggtattgaatcggcgcagcaattatttccgggcgtaactcaggtggcggtatttgataccagtttccaccagacgatggctccggaag
cttatttatacggcctgccgtggaaatattatgaagagttaggtgtacgccgttatggtttccacggcacgtcgcaccgctatgtttcccagcgcgc
acattcgctgctgaatctggcggaagatgactccggcctggttgtggcgcatcttggcaatggcgcgtcaatctgcgcggttcgcaacggtcag
agtgttgatacctcaatgggaatgacgccgctggaaggcttgatgatgggtacccgcagtggcgatgtcgactttggtgcgatgtcctgggtcgc

cagccaaaccaaccagagcctgggtgacctggaacgcgtagtgaataaagagtcgggattattaggtatttccggtctttcttcggatttacgtgt
tctggaaaaagcctggcatgaaggtcacgaacgcgcgcaactggcaattaaaacctttgttcaccgaattgcccgtcatattgccggacacgc
agcttcattacgtcgcctggatggaattatattcaccggcggaataggagagaattcaagcttaattcgtcgtctggtcatggaacatttggctgta
ttaggcttagagattgatacagaaatgaataatcgctctaactcctgtggtgagcgaattgtttccagtgaaaatgcgcgtgtcatttgtgccgttatt
ccgactaacgaagaaaaaatgattgctttggatgccattcatttaggcaaagttaacgcgcccgcagaatttgcataatttagttgaagtattgta
gagagattatttttcatgaaggtagatattgataccagcgataagctgtacgccgacgcatggcttggctttaaaggtacggactggaaaaacg
aaattaatgtccgcgattttattcaacataactatacaccgtatgaaggcgatgaatctttcctcgccgaagcgacgcctgccaccacggaattgt
gggaaaaagtaatggaaggcatccgtatcgaaaatgcaacccacgcgccggttgatttcgataccaatattgccaccacaattaccgctcatg
atgcgggatatattaaccagccgctggaaaaaattgttggcctgcaaacggatgcgccgttgaaacgtgcgctacacccgttcggtggcatta
atatgattaaaagttcattccacgcctatggccgagaaatggacagtgaatttgaatatctgtttaccgatctgcgtaaaacccataaccagggc
gtatttgatgtttactcaccggatatgctgcgctgccgtaaatctggcgtgctgaccggtttaccagatggctatggccgtgggcgcattatcggtg
actatcgccgcgtagcgctgtatggcatcagttatctggtacgtgaacgcgaactgcaatttgccgatctccagtctcgtctggaaaaaggcgag
gatctggaagccaccatccgtctgcgtgaggagctggcagagcatcgtcatgcgctgttgcagattcaggaaatggcggcgaaatatggcttt
gatatctctcgcccggcgcagaatgcgcaggaagcggtgcagtggctctacttcgcttatctggcggcagtgaaatcgcaaaatggcggcgc
gatgtcgctgggccgcacggcatcgttcctcgatatctacattgagcgcgactttaaagctggcgtactcaatgagcagcaggcacaggaact
gatcgatcacttcatcatgaagatccgtatggtacgcttcctgcgtacaccggaatttgattcgctgttctccggcgacccaatctgggcgacgga
agtgatcggcgggatggggctggacggtcgtacgctggtgaccaaaaactccttccgctatttgcacaccctgcacactatggggccggcac
cggaacctaacctgaccattctttggtcggaagaattaccgattgccttcaaaaaatatgccgcgcaggtgtcgatcgtcacctcttccttgcagt
atgaaaatgacgatctgatgcgtactgacttcaacagcgacgattacgcgattgcctgctgcgtcagcccaatggtgattggtaagcaaatgca
gttctttggtgcacgcgctaacctggcgaaaacgctgctctacgcaattaacggcggggtggacgagaagctgaagattcaggtcgggccga
aaacagcaccgctgatggacgacgtgctggattacgacaaagtgatggacagcctcgatcacttcatggactggctggcggtgcagtacatc
agcgcgctgaatatcattcactacatgcacgacaagtacagctacgaagcttcgctgatggcgctgcacgatcgtgatgtctatcgcactatgg
catgcggcatcgcgggcctgtcggtggcgacggactccctgtctgccatcaaatatgcccgcgtgaaaccaatccgtgacgaaaacggcctg
gcggtggactttgaaatcgacggtgaatatccgcagtacggcaacaacgacgagcgcgtagacagcattgcctgcgacctggttgaacgctt
tatgaagaaaattaaagcgctgccaacctatcgcaacgccgtccctacccagtcgattctgactatcacttctaacgtggtgtacggccagaaa
accggtaatacgccggacggtcgtcgcgccggaacaccgttcgcgccgggcgctaacccgatgcatggtcgtgaccgcaaaggtgccgtg
gcctcattgacgtcggtggcgaaactgccgttcacctacgccaaagatgggatctcgtacaccttctcaatcgttcctgcggcgctgggcaaag
aagatccagtacgtaaaaccaaccttgtcggcctgctggatgggtatttccaccacgaagcggatgtcgaaggcggtcaacacctcaacgtc
aacgtaatgaatcgggaaatgctgctggatgccatcgagcacccggaaaaatatcctaacctgacaatccgtgtctctggctacgccgtgcgc
ttcaacgcactgacccgtgaacagcaacaggatgttatttcacgtacctttacccaggcgctctgacgccggaggatgtatgaaaaagattatc
gaaacgcaacgtgccccaggcgcaatcggcccttatgttcagggcgttgatttaggcagcatggtcttcacctccgggcaaataccggtttgcc
cacagaccggtgagatcccggctgatgtgcaagatcaggcgcgtttaagcctcgaaaacgtcaaagcgatcgtggttgctgccgggctgagc
gtgggcgatatcatcaagatgaccgtgtttatcaccgatctgaatgattttgccaccatcaacgaagtctataagcagttcttcgatgagcatcag
gcgacctatccgacccggagctgtgtgcaggtcgcgcgtttgccgaaagatgtgaagctggaaattgaagccatcgcagtacgtagtgcgta
aagcctcgtgagcgggacggtcgtaaggtcgttccgctccacttcactgaacggcaatccgagggtgtggatatgattagtgcattcgatattttc
aaaattgggattggtccctccagttcgcataccgtggggccaatgaatgccggaaaaagttttattgatcggctggaaagtagcggcttattaac
cgcgacgagccatattgtggtcgatctgtacgggtcgttgtcactgacgggcaaaggccatgccacggatgtcgccatcatcatgggactggc
aggaaacagtccgcaggatgttgtcattgatgagatccctgcatttatagagttagtaacgcgcagcgggcggctgccagtggcatctggtgcg
catattgttgattttcctgtagcaaagaacattatcttccatcccgaaatgttgcctcgccatgagaacggaatgcggatcactgcctggaaggga
caggaagagctattaagtaaaacctattactctgtcggcggcgggtttattgtcgaagaagaacacttcggcctgtcgcacgatgtcgaaacgt
ccgtaccttacgatttccactcagcaggtgaactgctgaaaatgtgtgattacaacggcctgtctatatctggtctgatgatgcacaacgagctag
cgctgcgcagcaaagcggaaattgacgccggttttgcccgtatctggcaagtgatgcatgacggtattgaacgtgggatgaacactgaaggc
gtgctgcctggtccgctcaatgtgccgcgccgtgccgtagcgctgcgtcgtcagctggtttccagcgataacatctctaacgatccgatgaatgtc
atcgactggatcaacatgtacgcgctggcggttagtgaagaaaacgcagctggcgggcgcgtggtaacggcaccgactaacggtgcgtgc
ggcattattccggcagtactggcttattacgataagttccgtcgtccggtaaacgagcggtcaattgcccgctattttctggccgcgggggctattg
gcgcgctgtataaaatgaacgcctccatctctggcgcggaagtcggctgtcaggggggagattggcgtggcctgttcaatggcggcggcaggg
ttaactgaactactgggcggcagtccggcgcaggtatgcaatgcggcggaaatcgcgatggagcataaccttgggctgacctgcgatccggt
tgccggacaggtacaaatcccgtgcattgaacgtaatgccattaatgccgtgaaagcagtaaacgccgcgcggatggcgatgcgccgcacc
tcggcaccgcgtgtttcactcgataaagtgatcgagacgatgtatgaaaccggcaaagatatgaacgataaataccgcgaaacatcacgcg
gaggactggccattaaagtggtctgcggctgacattgctgtcagctttcatccttgggtgcacaaatgtgcaccttttttttgtgatctgccgcaaata

gagaatgaatattctcctgcgattaatttagtagaaaattcttatccatctccgttatttcaaagtcttgattgtgaagattttctctgacctcgcgcgtat
catttctttattctaataactcgcctgcaaaaacgtagaccgcatatcgccttgatgcggctcggaaaaaaataataagccctacaaaatattga
gcgagaaattatggaaattgcatcgaataaaggcgtcattgcagacgcttcgaccccggcgggtcgtgctggaatgagtgagagcgagtgg
cgagaagcgatcaaattcgacagtactgacaccggctgggtgattatgagtatcgggatggcgattggcgcggggattgtttttctcccggtgca
ggtcggtttgatgggattgtgggtatttttgctctcatcggtgattggttacccggcaatgtatctgtttcagcgattgtttattaatacgctggcagaatc
accagaatgtaaagattacccgagcgtcattagcggttatttaggtaaaaactggggcatcctgttaggtgcgctctatttcgtaatgctggtgattt
ggatgttcgtctattccaccgccatcaccaacgatagtgcttcctacctgcataccttcggcgtgacggaagggttgctgtcagacagtcccttttat
ggtctggtactgatttgcattctggtggcgatctcctcacgcggcgagaaattgttattcaaaatttcgaccggcatggtgctgaccaagctgctgg
tggtcgcggcgctgggcgtgtcgatggtgggaatgtggcatctgtataacgtcggttcgctaccgccgctggggctgctggtgaaaaacgccat
tattacgctgccgtttaccctgacgtcgattctgtttatccagacgttaagtccgatggtgatctcttatcgctcgcgggaaaaatcaattgaagtgg
cgcggcataaagcattgcgggcaatgaatatcgcgtttggcattttgtttgtcaccgtctttttctacgccgtgtcgttcacgctggcgatgggacat
gacgaagcggtaaaagcctatgagcagaatatttccgcgctggcgattgccgcacagtttattagcggtgacggcgcagcgtgggtgaaagt
ggtcagcgtcattctcaatatctttgctgtcatgactgcattctttggcgtctatttaggctttcgcgaagcaacgcaagggatcgtaatgaacatcct
gcgtcgcaagatgcctgccgagaagattaacgaaaatctcgttcagcgcggcatcatgattttcgccattttgctggcctggagcgccatcgtac
tgaacgcaccggtgttgagcttcacctctatctgtagcccgattttcggcatggtagggtgcctgatcccggcgtggctggtttacaaagtaccag
cattgcacaaatacaaagggatgtctctgtacctgattatcgtcactggtttgttgctgtgtgtttctccgttcctggcattttcttgatttacctgaaatttt
aaggtttttaatatgtttgattcgactttaaatccgttatggcagcgttacatcctcgccgttcaggaggaagtaaaaccggcgctgggatgtactg
aaccgatttcactggcgctggcggcggcggttgctgcggcagaactggaaggtccggttgaacgtgtagaagcctgggtttcgccaaatctga
tgaagaacggtctgggcgtcaccgttcccggcacgggaatggtggggctgccgattgcggcggcgctgggggcgttaggtggaaatgccaa
cgccgggctggaagtgctgaaagacgcaacagcgcaggcaattgccgatgccaaagcactgctggcggcggggaaagtctccgttaaga
tccaggaaccttgcgatgaaatcctcttctcacgcgccaaagtctggaacggtgagaagtgggcgtgtgtcaccatcgtcggcgggcatacca
acattgtgcatatcgagacgcacgatggtgtggtgtttacccagcaggcgtgtgtggcagagggcgagcaagagtctccgctgacggtgctttc
cagaacgacgctggctgagatcctgaagttcgtcaatgaagtcccgtttgcggcgatccgctttattctcgattccgcgaagctaaattgtgcgtta
tcgcaggaaggtttgagcggtaagtgggggctgcatattggcgcgacgctggaaaaacagtgcgagcgcggtttgctggcgaaagatctctc
ttcatccattgtgattcgtaccagcgcggcatccgatgcgcgtatgggcggcgctacgcttccggctatgagtaactccggctcgggtaaccag
gggattaccgcaacaatgcctgtggtggtggtagcagaacacttcggagcggatgatgaacggctggcgcgtgcgctgatgctttcgcatttga
gcgcaatttacatccataaccagttaccgcgtttgtctgcgctgtgtgccgcaacgaccgcagcaatgggggccgccgccgggatggcatggc
tggtggatgggcgttatgaaaccatctcgatggcgatcagcagtatgatcggcgatgtcagcggcatgatttgcgatggtgcgtcgaacagctg
cgcgatgaaggtttcgaccagtgcttcggctgcgtggaaagcggtgttaatggcgctggatgataccgccgtgaccggcaatgaagggattgt
ggcgcatgatgttgagcagtcgattgccaacctgtgtgcgttagcaagccattcgatgcagcaaacggatcggcagattatcgagattatggcg
agcaaggccagataaacgccaggttttggggatcgtgccggatgcgatgctggcgcatcttgtccggcctacgggttgtttaattcgttaatgttgt
tggattttgtaggccggataaggcgttttcgccgcatccggcaacctgtctttacgcggtagcttcatcctcggctaaacgcgtttcagcatccgcg
acgatagcttccagctcgctcaacatctcttcatagccaaaatttttggcggtgcgagcaggttctttcgccaccacgggcttcaccggctggcgc
tttttggccaatttttactcatcatcatctccctgtaaaaacaactagacaggcaaatctatcagcaaagcgcgcagtggggaatcggcaacca
gcgtaatgttagcctcgtcacgaataaacgccccatcaccgcaggtcagcgccgctttctcttcatgatgcgtaagcgcatgaaatttcccgtga
atcgattgcaaatacgcgcgtggcccatgcaactggaaattcgcactttcgcctttgtcgagcacgatatggtgcagccacacttgctggcgtaa
ttgcaggcttcccatcgcaccctctggcgaggcgattaattgctgcttgcccatattaagcgccagcttttgaatcagcggattctctcgctgcggg
caggcgtccagccaaagctgcattcgcgttaacggtttgtctttgctgagattgtgttcgctatagctgacacccggctgggtagagagcagcaa
cgcctctccggcgctggcctgaacatgattgccttcgctatcgcgatactctgcttctccatccagaatcacatttaaaatatcgactttgggatagg
ttcgcggctgaaaggcggcacctggggccagcacttcctggttaagcacacgcagggaggcatagcctaacaatttcgggtcgaagtagtgt
ccaaaggaaaaagtataccgggcctgcaaccatccgtagtctgcttgtccacactgcctggcagttcgggtagtaatcataattcttgacctctct
ttacttccttttatggtaaggggctggacgctacattgttagccagatattctgcccggtatgttcaaatttcctgaatgagaacgaaatggccaaag
aaagggcattaacgctggaagcactacgggttatggatgcgatcgatcgccggggcagttttgcggcggcggcggatgagctgggacgcgt
gccttccgcacttagctacaccatgcaaaaactggaagaagagctggatgtggtgctgtttgaccgctcgggccatcgtaccaaattcaccaat
gtcgggaggatgttgttggagcgagggcgcgttttgctggaagccgcagataaactgactactgatgcggaagctctcgcgcgcggttggga
aacgcatctcaccattgtgaccgaagcgctggtaccgacacctgccttttcccgttaatcgacaaactggcggcaaaagccaatacccaact
ggcaatcatcacagaagtgctggcgggggcgtgggaacggctggagcaggggcgggcggatattgttatcgcgccggatatgcattttcgttc
ctcgtcggagatcaactcgcgcaagctctatacgttaatgaacgtctacgttgccgcgcctgatcacccgattcatcaggagccggaaccgttat
ctgaagtgacgcgtgtgaaatatcgtggaattgcggtggcggataccgctcgtgagcgcccggtgttgaccgtacagctgctggacaaacag
ccgcgcttaacggtgagcacgattgaagataaacgtcaggcattactggcggggcttggcgtggcgacgatgccgtatcctatggtcgaaaa

agatattgcggaagggcggttgcgtgtcgtcagcccggaatcgaccagcgagatcgatattattatggcctggcgtcgtgacagtatgggga
agcgaaatcctggtgtctgcgggaaattcccaaactttttaacggaaaataatatttattattgttaataaaaaggggcgaaatatcgccccaatt
ctaagattaatttgaaccaaacttcggatcgtttccgtaacgattgctgccagaagtaccttctgtgcagaaaaaaaccagaagcacgagcca
gccaataaacggaacaaaaaagagcaaggcccaggcacctgaacggtcggtatcgtgtaagcgacggatagcaagcgcaaggacggg
aaggaaggttgccagcaaataaagcatagacagatatggaagctccagacctaaaatcaattgaatgacattgataatagcgccgacgatg
gcgttaatcagagtaaacatccagtactctttacgacgtgcgcgaccagagaaaccaacataatttttaatacggacaaataccactgcataa
aaacatccctttaattaaaagtgacgaaagtctattccgggtattgtgtaaatgctattcgtatctttctttttcggatgttataaaggttttgatatttaag
ataaatacatggttattttttataggtaatttatataacgacattgaaatattttaaataaacattagtaaattattcattcatcaacataatgataaaag
ccggatgagtcagacatccggcgagcagatttgttttaaggttccagttttggatctggtccaaagcggttttcgcctggcgtacccgcctggcagt
taaagacgatgataataagccagccgataaacgggattaagaatagcagtgcccaccacgccgagcggtcggtgtcgtgcaggcggcgg
aactgaaccgcccaccacggtaaaaacaccagaataccgtagattgtcgtcaggatgccttcgccaccagcgcgttgccagcctaacatttta
tccagcagccccagcacgaacgtaaagatgatgttgaccagaataaacatccagtactctttgcgccgcgcacgaccccggaaaccgacat
aatttttagtactttcagataccagtccatttttgcctcaattttgctcaatcacttgtttttaaagcagcaaattaagaatagacgtgaatgaaatggc
agtaaatattcgttcaaggaattaataaagtgccgtgaaggcgcgctaagactgatgacaagcgcaaaatcgccagatgcgctacgcttatct
ggtctgcgcgggttatgcaatctattgaatgtacaatgttttgtatgtcagataaggcgttcatgccacatccgacatgaacaacatgcacttagct
aattatcagaggcacctcgtaaggtgcctttttttaaccgaagcgaacatctcgtccatgcggttcatcgagatcctgccacggaccaattgaaat
aatccccgtagggttgatggtcttatggctgcggaagtaatgattacggatatgatcgaaattgactgtttcggcgatccccggcatctggtagat
atcgcgcagaaaaccgtacagattcaggtaatcgctgatgcggtgcttatcacacttgaagtgggtcacatacaccggatcaaaacgcacca
gcgtagtccacaggcgaatatcggcttcggttagctggttgccggtcaggtaacgatgctggcctaaaatctgctccagtcgtgccagcgattca
aacactttcgccaccgcctcgtcgtaagcttcctggctggtggcaaaaccagctttatacacgccgttgttaacggtgtcataaatccagccgtta
agttcgtcaatttttgtttgcagggctggagggtagtaatcacccgctttcgcgcccagcgcatcaaacgcggtattgaacatgcggatgatttctg
ctgattcgttgctgacgatggtgtggttctttttgtcccacagcacgggaacagtaactcgtccgctgtagtgtggatcggcgtggagataaagctg
atacagaaattcattttgatagagcgtgtctccggttgctcccggaaaactgtcatcaaaggtccaaccgttttccagcatcagcgggttcactac
ggaaacggaaataaacggttccagtcctttgagtttgcgcatgatcagcgtgcggtgcgcccacgggcaggcgagtgaaacatagagatgat
aacgatctttctctgcgataaaaccgcctgtgccagtggggccaggagcgccatcggcagtgagccagttacgaaatgcggaagctgaacgt
tgaaatttaccgccggtagatttggtatcgtaccaggtgtcatgccagacgccgtcaatcagttgacccatttttctctcctccaatagcaaaaacg
aggagcttcctcctcgtttttatcgttaagtatagcgcgtgcttaccactttttattcagcaggcggtcgatgctatacgcgcccggaccggtaattg
ccagcagcaggaatccgccagaaattgtcaggtttttcatgaacatcagcgagttgacgccttcagcaaagttgctgtgaaataaaaatgccgt
cagcagcgtaaagcccgcagtaaacagggctgtggtgcgagtcaggaaaccgaacaggattgccagaccaccaccaaactcaagcaga
atcaccagtggcagcataaaacccgggacgcccattgcttccatatattgttgggtacccgcgtagccagtaatttttccccagccagcggtaat
aaacagaatcggcattaaaatgcgcgctaccagtacaccaacatcttctaatttttttcatcatactctccactgcgccactcaggctgctgattgttt
ttttgtgcaatggcgcggtattagcgtcgttgctgtcgatggagagaatcataaacgtggtgaatgatgattgttagcaaggaaaactgtcaaaa
atcttcaaaaaatttgagggataaggccggaatggctccggccagagggaagttaaccgcgaagctgttgctgcttgagggtcgttttaacca
gacgccaggcgctccatacgccaaaaccgcgtctggcccagcggaccagcatattaggatggcgaatcgtccagatcgccatcacgctact
gccaaccagcgcccaggagcgcagacttagcagcatattccagcgacgatcgtaagcgcctgttgtctccagccattcacgacgactggcg
gaaagatccagccgttgttgctggatctggctaagcagttgcgccttacgtcgttcacgttcgactttactgctcacgggactcctcctcgagcagc
tgccgatcgtttgctaactcatggcgtgtatggcgcagcaacgtagacttacgcgatttacgtagcgtccagataccgccaatcagtgccagtag
cagcaacaccacggtggtggcaatcatcgcattcaggcgatattgcgggtcaaccgcccaaataattagcaccatcaggctcataagacca
aatgcagcgaaaagcatcgtcaggcccagcatcagtaaaagttgaaagagattcgctttttcctcttccagctccaccaccgccagccgcaga
cgtgtctctaccatttcaaccatgatagaaacaattcgctgcccgatgcccagaacgcttttaccgggcccttgtgcgtgatgagtgtccgccata
attaacgacgcgacagcagaacgccgagcactacaccgattgcagcgccaatgcccacgcccgtccacggattttcgcgcacatactcatc
ggcacgcgccgccgcgacacgggtttgtttggcaatggcatcaccggtttcacccaggcgataacggctctgtttcagtgcctgctccgctttgct
acgaatcttactcaactcttctttcgacttctcgccagatgagctaagcacctcttccagcgtatcggaaagggatttcaactcagcacgcagatg
ttccgtagtgtgttctttcgacatagttttctccaggtgagtaagtagtgactattgtgagttagtagtcgcgcgcttccagctttttcagctcttcctgcg
cttctgccagtttccgttcgcgtttggcaatcttatcggcatcgcctttttgcttcgcctcggctaaatcttgctggcgttccgccacctcatctttctgcttt
gcgattttcttctgatgatcggcacgcagctggctatctgaacagttggcccggacttcactcagggctttattcagaccgtcaatacgattctggttt
tggtgtttttcggcatagctgatctccttaaggatattttgctccttttcctgacacagggtagtggcataactaccggcactaagagcaaagagag
aaacagctaaagcgatgcggtatttcattcttgaaccttccattgtgattcggacctattacaaaggaacgattgtccagacaacgaagcggtag
tgttaaccaccgcctcattaatcatagacagttacggtgaaatcaccaaagtgagtgatatgtttcggataacaggaagttatccgaagcgatg
agagttatcccgtaaccgggtcagccactgcatagcctgactgttattgtcctgctgcgcaatgatgtagccatggggcaatgttctgtcgaggac

cgcttttgcggcggcgctctggtcgctggagtcaaatgtaattaacagagtgtcgtttttgggggtgatacttttgaaaggaatgccatgagcgtca
aggtgatgccagattgaaaaaccgtctggcatcgttgtgccttgatgaacggcacgaatcgccagcgtagactcttgctggcgaaccgcggac
caggccagcaacagcgtgccgaccagaagtaaaacagcgccggaccaggctagctggcgaagcgacatgcgaggtatttgcatccctta
cccccgatttccatatttctttttccataacacgaccagagaacctgccaggccaaacaccagcagcaccaccgggagcagcatcaggcatg
acatcagctggtcctcgtactttaaaaataccggcgttttgccgagcatgtaacccagagttgtcaggatcaatacccacagcagaccgctcat
ccagttgaaaaactgaaagcgcgcgttattcagccctgataacccggcaatcgtcggcagcagtgttctgacaaacgcaataaagcgacca
attaacagcgccgataaaccgtgtttatgaaaaagatggtgtgcgcgttgatgataatgcgcgggtaaatgagatagccagttttgtacggtgcg
ggtattgcccagccatcgcccctgaatatagctgacccagcagccgaggctggcggcaacggtcagcagcagaatcgtttgcggatagccc
atcgcgcctttcgcaatcaacacgccgaccaataccagtaaactgtcgcccggtaaaaaggccgccggaagcaagccgttttcaaggaaca
aaattacaaacaagacaaaatacaacatgccaatcatcgatggattggccagggtttcaaaatcctgcgcccacagggcttgcagcaattgg
gtcaaaagttccattcattattcctggtgaatcggttaacgccacgctggttttaaaagatgcactaccgcaatgttattgctgttttggtatgttctgcg
ggtttgatacatcaacacagcctgaactattccctgttaaatcgtgagataagaaagcattcaaaaacataaattcaaattgaatctaattgtaac
aaaggtcaacgttgttcgttatcaaaattacatgtccgtggagtctgattttgctgaatgcaggggagggtggcgagaggatttacacaggctga
cactttatacttttcgcttaccgacgagacaaacaggcgcattgcagcgcctgtcggacaggaatttcatttactgccgctggtggctgtatcgag
atgcaccaccgggttttcggcgaatagatagcggtcggcattgaactcgaagtcgtcgctggtttcgttaaataacatgatcttggtgttttccagat
gctgccacattgccagcttagcagcatgaggatctttgcgaatcagcgccttgaggatttgatcgtggtcatcacaccagttatcgacggtacgg
gaatcaatgtgttcgtgcagttttttccagtacgggttatgactacgctgggtccacatttttccacgatagccgccagggcggagttctgcgtcgc
cagagcgacctgaatgtggaactgcaaatcccactcggaatcacggaagcattgttcgccgcgcgcctgttcctgaatggccatcagtttcatg
atgtcctgtttcgttacctgagttgccgcgaattcggcaatattactttcgatgagctggcgagcctgaagcaactcaaacggaccgtaattggcg
aattccatattattgtcagccgcctgctgatggcgcggctggttggaaaccacatgaatacctgaacctttacgcacttcgacatagccttcaactt
ccagcatgatgatggcttcacgaactaccgtacggctgacgttcttttcatcggcaataaagcgttctgcaggcagtttatcacccaccagatag
acgccctgttcgatgcgctctttcaggtcagcggcaagttgttgatacaaacggcgtggttcagtgatttccatatgcgctccaggcatgatacgg
cagactatatttgttataccacttttgcgagtttagctctactcgcagtgatgaaaaagccgcctgagaaggcggctttgaagggaaactaaaact
tacacgaaacgttaatgttgcggtgcgggatcgttatgtgtttcctgcgccacctcaatcgccggtttgttctgcaacacggtccagataaccagc
gcacctaacaggtcgaacactgccagaactgcgaacagcgggctgaagccgatggtgtcagccagtgcaccgacaaccagcgcaaaca
gcgtacttgccagccatgcggacatcccggttaaaccgtttgccgttgccacttcgttacgaccaaacacatcggaagagagcgtaatcagcg
cgccagacagtgcctggtgggcaaaaccaccgatacacagcagcataattgcgacatacgggttggtgaacaggccgatcatacccgggc
caatcatcagcactgcacccagcgttacgaccatcttacgggaaacgatcaggttcacaccaaaccaacgctggaacagcggcggcaggt
aaccaccgaggatacaaccgaggtcagcaaacagcatcggcatccaggcgaacatcgcgatctctttcaggttaaagccgtaaactttaaa
catgaacagcgggatccacgcgttaaaagtaccccaggccggttctgccaggaaacgcggcagcgcgataccccagaactgacggttac
gcaggatctgaccaacggacattttcttcgccgtgctcacctggtgctgggcttcctgaccattaataatatagtcgcgttcttcatcggtcagatgc
ttctggtcgcgcggatgtttatagaaaatcagccatgccatcgcccagataaagctcaatgcaccggagatgataaatgccatctgccagctgt
gcattacgattgcccataccaccagcggcggcgcaatcatcgcaccaatcgaagaacctacgttaaagtagcctactgcgatggaacgctctt
tcgccgggaaccattcggagctggctttcagacccgccgggatcatcgctgcttccgcggcaccgaccgcaccacgagcaacagccaggc
caccccagctacctgccagcgcggttgcaccacagaacacggcccacagcacagcaaacattgcataaccgattttcgtacccagcacatc
cagtacataacctgctaccggttgcatgaccgtataagcagcagaataggctgcgatgatataggaatactgttgggtggagatgtttaactctt
ccatcagagttggcgcagctgccgccacagtgttacgcgtcaggtaaccaagcacggtgccgagcgtcaccagtgcgatcatataccaacgt
aaccctttaattttacgcatgtaaaacctcatcgttttgttatatccgcaggaagcgcggccatcatcccgccagaggctgaagatgttagttacga
ccgggtaaccgaaaaaaaaccggcacgccccgaacgttgccatgaaagtttcgtgctaattcggcttccgtaccggttaacccgattcatcta
acaaatgaatgaatcggcaatttgcattccgtcgacttatgatttgcgacggcagaaagataacttgtcatacaactttaaaggtgagagccat
cacaaatgtgggaatatttgtagggacattacctgacgacagcaaggccagtactggcgcggcctgcagcgagatttaccactttgagagtaa
tttttttaactacgtttattgatctaactcacgaaaatatcttcggactctggaaattggtgtgataactttgtcagcatcgcaccataagcaagctagc
tcactcgttgagaggaagacgaaaatgactccgtttatgactgaagatttcctgttagataccgaatttgcccgccgtctgtatcacgactacgca
aaagaccagccgattttcgattaccattgccatttgccgccgcagcagattgcggaagactatcgttttaaaaacctgtatgacatctggctgaa
aggcgatcactacaaatggcgcgctatgcgtaccaacggtgtggccgagcgtctgtgtaccggtgatgcgtctgaccgtgaaaaatttgacgc
ctgggcggcgactgttccgcacactatcggcaacccgttataccactggacgcacctcgaactgcgccgtccgtttggtatcactggcaaattg
ctttctccgtcaactgccgatgaaatctggaacgaatgtaacgaactgctggcgcaggataacttctccgcgcgcggcatcatgcagcagatg
aacgtgaaaatggtcggcaccaccgatgacccgatcgattctctggagcatcacgcagagatcgccaaagacggctctttcaccatcaaagt
gctgccgagctggcgtccggacaaagccttcaacatcgaacaggcgacctttaacgactacatggcgaagctgggcgaagtttccgatacc
gacattcgccgctttgctgacctgcaaactgccctgaccaaacgtctggatcacttcgccgctcacggctgtaaagtgtctgaccacgcgctgg

atgtggtgatgtttgctgaagcgaacgaagcggaactggacagcattctggcgcgccgtctggctggcgaaaccctgagcgagcacgaagt
ggcgcagttcaaaactgcggtactggtgttccttggtgccgaatatgcacgtcgcggctgggtacagcagtaccacattggcgcgctgcgtaat
aacaacctgcgtcagttcaaactgctggggccggatgtaggctttgactccatcaacgaccgtccgatggcggaagagctgtctaagttgctga
gcaagcagaatgaagaaaacctgctgccgaaaaccattctgtactgcctgaacccgcgcgataacgaagtgctgggcaccatgatcggtaa
cttccagggcgaaggtatgccgggcaaaatgcagttcggttccggctggtggtttaacgatcagaaagacggtatggaacgtcagatgaccc
aactggcgcagctcggtctgctgagccgctttgtcggtatgctgactgacagccgtagcttcctgtcatacacccgtcacgaatacttccgccgc
attctgtgccagatgatcggtcgctgggtggaagcgggcgaagcaccggcggacatcaacctgctgggcgagatggtgaaaaatatttgcttt
aacaatgcgcgtgactacttcgccattgaactgaactaaggtctggggttgatatgcaatacatcaagatccatgcgctggataacgtcgcggt
cgctttagcagatttggctgaaggcacagaagtcagtgtcgataaccagactgttacgctgcgccaggatgttgctcgtggacataaatttgcgtt
aacggatatcgcaaaaggggccaatgtcatcaaatatggcctgccgattggttatgcattggcggatattgcggcaggtgttcacgttcacgcc
cacaatacgcgcacgaatctgagcgatctggatcagtatcgctatcaacctgattttcaggatctgcctgcgcaagcggcagatcgtgaagtgc
agatctatcgtcgcgctaacggcgatgtcggggtgcgtaatgagctgtggatcctgccaaccgtgggctgtgtcaacggcatcgcgcggcaga
tccagaaccgttttctgaaagagaccaacaacgccgaaggtactgacggcgtgttcctcttcagccacacctacggctgctcacagctgggcg
acgatcacattaatacccgcaccatgctgcaaaacatggtgcgccacccgaacgcaggcgcagtgctggtgattggtctgggctgtgaaaac
aaccaggttgccgcattccgtgaaacgctgggcgatatcgatcctgaacgcgttcatttcatgatctgccaacagcaggatgatgagatcgaa
gccggaatcgagcatttgcatcagctgtataacgtgatgcgcaacgataaacgcgagccaggcaaactcagcgaactgaagtttggtctgga
gtgcggtggttctgacggtctttctggtattactgctaacccgatgctggggcgtttctctgactacgtgattgctaacggcggtactaccgtactgac
cgaagtgccggagatgtttggcgcagagcagttgctgatggaccattgccgcgacgaagcaacgtttgaaaaactggtcaccatggtcaacg
acttcaaacagtactttattgcccatgatcagccgatctatgaaaacccatcgccggggaacaaagcgggcggtatcaccacgctggaagac
aaatcacttggctgtacccagaaagcgggttccagcgtcgtggttgacgtgctgcgttacggcgagcgtctgaaaacgccagggctgaacttg
ttaagtgcgccgggtaacgatgccgtagcgaccagcgccctggcgggtgcgggctgccatatggtgctgttcagtactggtcgtggcacgccg
tatggtggatttgtgccgacggtgaaaatcgccaccaacagtgaactggcggcgaagaaaaaacactggatcgactttgacgcgggtcagtt
gatccacggtaaagcgatgccgcagttgctggaagaatttatcgacaccatcgttgagtttgccaacggtaagcaaacctgtaacgagcgtaa
cgacttccgcgaactggcgatcttcaaaagcggcgtaacgctataaagtcgaaatgagttgttaataaaaaacggcgtttcataatgtgaagc
gccgtttgcttttccatacctgctaaggatttatcatgaccgcgtattggctggcccagggcgtgggtgtcatcgcctttctgattggtatcacaacatt
tttcaatcgtgacgaacgtcgcttcaaaaagcagctttcggtctatagcgccgttattggcgtacatttttttcttctgggcacctatcccgctggtgcc
agtgccatccttaatgccattcgtacattgattaccttacgcacgcgcagcttatgggtaatggcgatttttattgtgctgactggcggaattggcctc
gcgaagttccatcatcctgtcgaactattgccggttatcggcacgattgtcagtacctgggcgctattctgctgtaaagggctgaccatgcgctgc
gtaatgtggttttcaacgtgttgctgggtgattcacaacttctgggcggggtcgataggcggcacgatgattgagggtagttttctgcttatgaatgg
cctgaatatcattcgtttctggcggatgcagaaaaggggaattgatccgtttaaagttgagaaaacccccttccgccgtagacgaaaggggttaa
acaattaattacgcagggcgctatttgccagacgatcgtcttctgcctggcaagctgccgcagtgaacagcacgtcagttgaagagttcagcgc
ggtttcgcaagagtcctgcaatacgccgatgataaagccgacggcaaccacctgcatggcgatatcgttcgaaataccgaacatattacagg
ccagtgggatcagcagcagagacccccccgccacgccggatgcgccacaggcacacagagaagccaccacgctcaacagcagcgcc
gtgggcagatcgaccggaatacccagcgtattaaccgcagccagcgtcaacacggtaatagtgattgctgcgcccgccatattgatggtggct
cccagcggaatagaaacggaataggtatcgcgatccagattcagcttttcacacagcgccatattcaccggaatgttcgctgcagagctgcgg
gtgaagaaggcatacacaccgctttcgcgcaggcacagcagcaccagcgggaacgggttacgacgaattttccaccacaccagcaatgg
gttaaccaccagcgccaccagtaacatacagccaaccagcacgaccagcagttgcgcgtagccccacagtgtggagaaaccggtggttgc
cagggtagaagaaaccagcccaaaaataccaatcggtgcgaagcgaatgaccagtttcaccataaaggtaacggcattcgacatatcgtta
accaggtttttggtggtctcgttaccgtgacgcagtgcgaagccgaggccgatcgcccacaccagaatcccgatgtagttacctttcagcagcg
cgtcgatggggttggaaaccatgctcattaccagcccgcgcatcacttcgacaatgcctgacggcggcgaaatatcacccgcgctactggata
aatgcagggtagaagggaaggcaaagctgaagactactgcggccagagcagcagagaaggtgcccagtagatagaggaacaaaatag
ggcggatattggttttctgcccgtgctggtggttagcaatagatgccatcaccagcatcaacaccaggatgggggcaacggctttcagtgcgcc
gacgaacaaagtacctaacagaccaacagcttccgccgcgggttttgagatccatgccagaagaatccccagaacaaggccgaccagga
tttgttttaccaggctgccatgagccagacgccggaatagccccggtgaacgttgcgtagtcatttttcgatcctttcattgtgttgtcgcacatccct
ggtgcgttctgtcgacgcatctttcaggatgtaacaaagtgtttgcaaggtcgagtataaggaaagttacagcttcgggaagcgaaaaatgctg
gattttaaagagaggtcatatttgaaacttaatggttacatttctttgacataaaaataaagccaacgtgcggcttccagactgatgacaacatca
aaaaaggactcggtcagtcccctcgccccgtttggggagagggttagggtgaggggaacaggccagcatcggtgcgaaaaattaccctca
ccccgaccctctccctggaagggagagggagaaaacggtgctcatgttaatgacagggtacacggcttaggcagattaaaaattatccaccc
cgctgcttatcatgccgataattcacccaggcgttgataataaacgtcatcaccagaatgccaaacaccacgcccagcgagacggcgattgg
aatatggtagaagtcgacaatcagcatcttgataccgataaacaccagaatcaccgccaggccatatttgagcatcgagaaacgctctgctac

gcccgccagcaggaaatacatcgcacgcaggccgaggatcgcaaacaggtttgaggtcagcacaatgaacgggtcagtggtcacggcga
agatagccggaatgctatccacggcgaaaatcacgtcgctcaattccaccagaatcagtaccagcatcagcggtgtggcgtacagcaatcc
attcttacgcacaaagaaatgctcgttgtcgatggtgtcggtcatgcgcaaatgaccgcgtagccagcgcaccagcggcttgtcgccaatgcct
gattcatcttcatgggcgagcgccatcttcacgccggtaaacagcaggaaggcaccgaagatatacaggatccagtcgaactgcgaaatta
gccagctgccagtgaagatcatgatggtacgcagaacaatcgccccgagcacgccatacaccagcacgcggcgttgtaatgccgccggaa
cagagaaatagctgaacaacatcagccagacaaagacgttatcgaccgccagcgatttctcaatcagataacctgtgagaaaggccagtg
cctgtggatcggcaacggcgcgaccttcggtttgcaccagataccaccagaaagcggcgttaaacagtaacgacagcgtcacccagacca
gcgaccaggccgcagcctgtttcatggtcatggcatgtgccccacgacgcccctgcaacaacaggtcgatagccagcataatggcgacaac
aacagcgaatccgccccatagcaacggcgtgccgacagtattcatagaagttccttacacataaaaaaacggccaacgtcggaagacgtc
agccgctgctttttatgcatagacctcgccttccggcaaggtctcacttacaacaaaaaaggaatacgtctgcgtattcctttcgggttgcccggtg
accggatgtgtttttcacacatcgtaatgacgatcgaccggcaatgaagttactcccctttgcaggtaacaaagtatgacagaccattcagcca
gtcaatggtctgttacataccttttacactgttttactttggcaattatagttttacgctatctgccggaaaaatcaccccagtctggcggcggatctcg
gtcagcagtttggcggtgatgcgactgaccgccagccctggatgatccaccagatgctcatccaccagggtagcgaacagctctgcttcgtag
agcatggtattaatatgctgcggctgggtgagatcctgcatttggctgccacgcggcacgaagcacactttctggcattcagacagtttttcaatc
accagcgatcctgcttcgccctgaatctcgctcgccaggacagaatcactgacttttgagtgctgcaaggtgacgctgaaatcaccgtaatccat
caccaccacgccctgggcgtcaacgccgcttgccagcaaactggcggttgcctgcacgcttttcggctcaccaaataacgccaccgccgac
gccagacagtaaaaaccgatatccataattgaaccgttagagaatgccggattaaaggtgttgggattctcaccatccaggtaacgttgatacc
gcgaggaatactggcaatagttgaaaaagactttccgcagtttgccgactttcggcagcgcctggcgcaacaaatgaaagttcggcaggcag
gcggttttaaatgcctcaaacagcaccacctgattttcccgcgcacaggcaatggcggcatccacttccgccagattcgacgccagcggtttct
cgcaaatcacattaattttatggctaaggaaaagttgtgtctgggaaaaatgcagggaattcgggctggcaatatacaccgcgtcaatggcatc
gctttccgccatcgcttccagcgaggtaaacagatgctcgacagaaaaatcattggcgaagtgctgggcctgttcaaggctgcgggaatatac
ggcggttaacttgtatttaccgctctcatgggcggcctcgacgaactggcgagtgatccagttcgtaccaatcacagcgaaacgtatcataacg
cgtggatactccataaagggattctgtcgtcaatgtagcacgcctttttcatcttcgacgtgcgcagatacgcattatttctgcgccggacagccat
gctcactaaacggcccaatcatgaccgaaggcccgagatatttcggctggcgatggaggattttggcgtcaatcacggcgctgacacgagcg
agtttctcccgcagttgagtataaaaaccgtgcttcatattgaggtctggcgcgttaaaaccgatagcgtcgatgccgtactgttttgccagccag
atggcgcgttgattatgaaattcttgcgagataatggtgatatggttttcaccaaaaacctttttggcacgcactaccgaatccagcgttgagaatc
cggcatagtcgcagaagatcacttttgctggtacgcctttagcgatcaaagcctgctgcatacctgatgcttcatcgtaatttttacgtccgttatcgc
cacttaccagcagccatttcacttttccggcgtggtacaacgctgctgcggtatcaatacgtcgggtaaagtagcgattacccggcctcgccccc
agcaataaacccacgttacgcgccggaacggcgttgacatcgctccaggtcagctgtttgctggcgttgaccattacgcgatcggcgataagg
atggtcgctcctgccacaagtaacagtaagcaggcaatctttagagtccggcgagaaaaacaaatacggagaagaaggcgggcaaatgc
gcgcagcatggtcgttatccttaacgaaagggggaggtcgattttaggcgctaaagaaagaaaagataacgcctgacatcattttcagatttt
gttctgcccaagcgacaactgcgttagccacagtcgggtgtcgaactcaagctggtggtactggggttccatatgacaacacaactggtaaaa
cgctttgttatgctctttctccttcaggtgcgccagctcgtgcaccacgatcatgcgcaaaaacggttccggcgcgttgcgaaacacggtagcga
cgcggatctccacctttgctttcagcttgccgccctgcacacgagaaacagcggtatgtaacccgagcgcgtttttagcacatggatcttattgtc
atacatcactttattgatcggcggggcattacgcagaaactgatttttcagatcctgagtatattgccagagggctttatcggtggcgtagtcgtgtg
ttcccggatagcgttttgccagcacatcgcccagacgctgttcgttaatcagtgttcgcacctgggaaagtagctgctcgggataaccctgaaga
taagtaagattgctcataaccgcccacgaaaaagagaaaaagggtatactcacgcaccccttttcaggggcaatgtcgaaattttatcattcagg
agggccgatgagccacttagacaacggtttccgttcactgacactacaacgttttccggcgacggatgacgttaacccgctacaggcgtgga
agcggcggatgaatatttgctgcaacagttggacgacacagaaatccgcggcccggtgttgatcctgaatgatgcctttggtgcgttaagctgc
gcactggcggaacataagccgtacagcattggcgactcatacatcagtgaactggcgacgcgcgagaatttacgcctcaacgggattgatg
agtcgagcgtgaagtttctcgacagcaccgccgactacccgcaacagccgggtgtggtgctgatcaaagtgccgaaaacactggcattgctg
gaacagcaactgcgtgcgctgcgcaaagtggtcacgtcggatacacgtattattgccggtgccaaggcccgtgacattcacacttccacgctg
gaactgttcgaaaaagtgctcggcccaaccaccaccacgctggcatggaaaaaagcgcgcctgattaattgcactttcaatgaaccgcagct
ggccgatgcgccgcagaccgttagctggaagctggaaggtactgactggactatccacaaccatgcgaatgtcttctcccgcaccgggcttg
atatcggcgcgcgcttctttatgcaacatctgccagagaatctcgaaggggagattgtcgatctcggttgcggtaatggcgttattggtctgacgct
gcttgataaaaacccgcaggcgaaagtggtgtttgtcgatgaatcgccgatggcggttgcttccagccgtttgaacgttgaaaccaacatgcca
gaggcgttggatcgctgcgagtttatgatcaacaacgcgctctccggcgtggagcctttccgctttaatgctgtgctctgcaacccgccgtttcacc
aacaacatgcgctgaccgataacgtcgcctgggagatgttccaccacgcccgccgctgcctgaaaatcaacggcgagctgtatatcgttgcc
aaccgtcacctggattacttccataaactgaagaagattttcggcaactgcaccactattgcgacgaataataaatttgtggtactgaaagcggt
gaagctgggacgtcgtcggtaagattgggagttacctgccggatgcggcgtaaacgccttatccggcctacgttcagcacgttactccaggcc

ggataagacgcggcaagcgtcgcatccggcaatgtctgcccagttctggaatcgcgcttccataaaacaaaaaatccctgtcaccatcgtcta
cattctcttgtttagcgtttttctacgtttattcttccgtcacacagataaattcatccgttgccaatctggcaacggatgttatcatattgccaatttgtcaa
cggacgtgatgaagaatgcacctgataactcaaaaagcattgaaagatgctgcggaaaaatacccgcaacataaaacggagttggtggct
ctggggaacacgattgctaagggatatttcaaaaaacctgagtcattaaaagcagtattcccatctctggataacttcaaatatctggataagca
ttatgttttcaatgttgggggcaatgaattacgtgttgtagcaatggtctttttttgaatcgcaaaagtgctacatacgtgaagttatgacgcataaaga
atacgatttctttaccgctgttcatcgtactaaggggaaaaaatgattgcgattgccgacatcttgcaagcaggagaaaagctaactgctgtggc
accttttctggcgggtattcagaacgaggaacaatacacccaggcgctggaactggtagatcatctgctgctcaacgatcctgaaaaccccttg
ctggatctggtgtgtgccaaaataaccgcgtgggaagaatcagcgcccgaatttgcggaatttaatgccatggctcaagccatgcctggcggt
atagccgtgattcgtacccttatggatcaatatggtttaacccttttccgatctgccggaaattggcagtaaatctatggtgtcacgcgttttgagcgg
gaagaggaaattaacgctggaacacgctaaaaaattggcaacgcgattcggcatttctcccgccttgtttattgattaacgcgacgtgccggat
gcgttgcatccggcgcttcaaataacgatttaaatctccagcgccagccgtgttccctgggcaattgcccgtcgtgcgtccagctccatagccac
atcgcagccgccaattaaatgcaccgttttcccgctatcaatcagcggttgcgccagcgcgcggtttggctcttgccctgcgcagatcaccacat
tgtccactgctaatacctgcgtttcgccgttgatcaccacatgcagcccgtcatcgtcaatcttctgataacttacgcctgggatcattttcacacccc
gcgagagcagggtggtgcgatggatccagccggtggttttgcctaacccctgtcctggtttgctggctttgcgctggagcatcacaatctgccgtg
ggctacgggggatctgcattccctgcgggcttaagccaccagcctgttgtaggctactgtcgatcccccattcattacagaacccggcgatattct
ggctggtggattcgcccggctgacttaaatacatcgccgtatcaaaaccaatcccgccacaaccgatgatggcaactttgttgccaaccggcg
ctttgtcgcgcagtacatcgagataactcaataccttcggatgatcgatcccgtcgatgggcggagtgcgcggcacgatcccactggcgagga
tcgtttcatcgaaagcctgtaactgatccgccgtcacggtgtgattgagttttagcgtcacgcccgtcacttcgatcatccggcggtaatagcgca
gcgtttcgtaaaactcctctttgccggggatctgtttggcgatattaaactgcccgccaatctcgctatgagcgtcaaacaatgttacctgatgccc
acgcgccgccgcgttaatggcaaacgccagcccagcaggtcccgcaccgaccaccgccagatttttttctgcacggcgggaaggattggc
attttggtttcgtggcaggcgcgaggattcaccaggcacgaggtgactttgccaacgaagatttgatcgagacaggcctgattgcagccaatac
aagtgttgatctcatcggctcgtcccgattgcgcttttgacagcagctccgcatcagcaagaaacggtcgcgccatcgataccatatcggcatcg
ccgcgcgagagaatatcgtcggcaacctgcggatcgttaatccggttggtggttaccagcggcagcgagacgtggcctttcagtttgcgcgtga
cccagctaaatgcgccgcgcggcacgggcgtggcaatggtcggaatacgtgcttcatgccagccaatgccggtgttgataatggtcgcgccc
gccgcttcaatggcctgcgccagctctaccgtttcggcaaaagtcccgccgtcttcgaccaggtcgagcatcgacagtcggtagataataatga
agtcgttgccgacgcgttcgcgcaccgcacgcactacttctacggcaaatcgcatccggttgcggtaatcgccgccccactggtcactacgctg
attggtgcgcagcgtcagaaattcgttgatcaaatacccttcggaacccatcacctctacaccgtcgtatcctgcctcccgcgccagttgcgcgc
agcgggcgaaattgtcgatcagttgcaggatctcttcatggcttaactcatggggcacgaaacggttgatgggggcctgcaatgcggacgggg
cgaccagatgcggttggtagctgtagcgcccggtatgcaaaatttgcagggctattttgccgccttcctgatgtaccgcttcggtaatggtgcgat
ggtgtgggatctggctggcgtcgttgagcattgcgccaccttccatgccaacgcctgttaaatctggtgcaatgccgccgctgacaatcagcgcc
acgccgtgacgggcgcgttcggcataaaacgctgccagccgctcggcaccgtccgggtattcctccagcccggtgtgcattgagcccatcaa
cacgcggtttttttaacgtggtaaaacctaaatccagcggggcgaacagcgacgggtagctcataaaattgtccagtatgtaaaataattgttatg
tggtcggatgagttctaatttagccgtcgccggagaaaagggaaaaggggagtgcggggtttgtgatgggattcaaaaagaatacgcccggt
gataacgccggatgcgacgcttgtcgcgttttatccaggttacggaattgcacatgtagggcggataaggcgtccacgccgcatccgccagtg
gcgcggtgcagatgccggatgcgacgcttgtcgcgtcttatccgacctacgggaacacacatgtagggcggataaggcgttcacgccgcatc
cgccagtggcgcggtgcagttgccggatgcaacgcttgccgcgttttatccggccaggaggattgcacatgtagggcggataaggcgtttacg
ccgcatccgccagcagttgcaaattattgcttacggaaaaaatcgttatacaacatatacaaatgcgcggcactccaggagaaatttggtgcg
ccttgctgtgcgcctgtcagcgggttgtaattttcctgaattggtccatcggcggttaaccctttggcgtgccggaagaacgtatccgccagcttca
gggcatcatcacgataaccgtaacgctccatccctttcagaccaaaccagaactgatccacccatacgcgcccacgccagtagatatcagcg
ccaaaagccggattggttaacgccgccgttcccagcgggacaaaggtgttgaactctttaggatcgagcatcaccttcaccaccgcgtcggca
ttggcctgcgttgccgcaccgttaaacagcggcgaccagccttccggccctttaccgcgctcaacaatcggtttgcccgcgcagccgttcgcca
gtggtttatcttcaatacgtacgtcatagtagaactgggtagtcgggtcgaacatacaggtgttgatgtagtccgcgagctgctgtgccaactggc
gatagcgtttggcctcttctggcttaccgaggatggttgccatctccgccagataatggttatcgctgtacatatagctggcctgatccaccgactc
ctgcaatagcgagtagcccagcaacgttccgtcctgactgcggttttcggcgaatttcaccgtccagtcgctacgtttgccgccattagcgacata
tttatccaactgttctttgtcgataaacccaaagacggcggcatcatcacgccccgattcccacgatgcagcaacctgtgccggaatttccagac
tgtcgtactggcctttctccaccacgcgggcgtagttgttcagcccagactgcgtctcttctttatcgcctttttttcaccgtaaacagcatctcgccgct
ctcagtgttgtgggctttgtcgcgggtcgcgccatattccggcacgccgttgccgttgtgatcgcggttacgtaaccaccagtcgtgataggccac
cagtttcgggtacatctctgccacccaggttttatcttgggtgacgttatacacttccatcaccgaccaggcggcaaggctgggtttggtattacgtt
cgttccagttgccgccatcgccaccacgctcggggctaaggttccacgctatcaggtcggggacaaagcccacatcctgcggacgcacgcta
tcgccaggctggatctgccaggagaagaccgcgcggatattctctttggcgatgtccggattgaaatgcgccatcgcaaacgcctgcttccag

gtatcccacggccaggtctgattgccggagaaccagcgcccggtcaccgacggtgtaacggtgttaaatttcaccgcaccgcccggtgagcg
ccagttaccgttgagcgtttcgatcgctttcaccgcgacgcgcgtctgttccggcgtcgcatccggattggttaaccctttcttcaggtattcttccca
gcgttgctgcgaggcggtgagataaaacgccggacgcgccagaatatcgcggatctgcatttgctctttgctaacttcctgagcggtcagcaga
tgggaataggtggtgtagagcgtggtcgaaccgttgatatgcgccttactggtaaagcgattaccgttgatttcagtctgcaccggcagggattta
tgcacctgatattccgattcgccggaggtcagcagatcccaggtggcgcgcactttgccaaaggtgactttcaggccatcacgggtggcgctg
attttgcgctgatagtcagggtattcgccagcaatggttttatcggaaagcggtttcccttctttcgcttccagtttttccagcagttcgccgtcccaca
ccagatccagcggtttattgctggtgattttggtttccagtagtgacgtgcgcggcgtggcgaagcgcagagtcatttcgacttgcacatcttttgct
gtcagtttttgcaccagcgcaccgggaatactgtatgcctccagcgtaaagtcgactttcttgccgtcctgccagacggtcaggcggtcgaaatt
gctggccataaagttgatgtactcttccgtcagcagcgcaacgcccggaaagccgcccatggtgttagggccgtctggcaacagatgaccat
gccaggctccgagatcgaaaaacggattaaaacgctggtgatcgtcgtaatcgtaatccttcatgtactgcggcgcgccagtacggttaatcac
gtttttataattgtcggcgttagcggcatgggcggaaaaacttatcagcagagcgcaggttactggcgttaaaatagtttttattttcatgaaattaca
gtccttattaccagtagaaatattgcatcaggaagatttggtcgctgggatcgtgagaggcatttttcatgtagaagcggctatcaaaagccgtgg
cgaaacgtccgccgccgtattcgtaccagataccaaactgcatgaaagaggtggtttcatcgtcagcgtggtcagggcgatgtttggcgtaggt
gtaagcggtggagagataaacgccctttaacgcttcatacatggcgctcaccatcatcccactttcacttcccggtacgtccagcccgtcggcg
cgtccggtatggtgccaggcgcgagcggcaaagttaggagttagcaggcccgtgagatagagctgtcctgtgccgtcggtgatttccagacc
gttcatccaggtgatgccttcggcaaggtcatactggatgtaaccgttcatcattgccggataggtgtatttgtcgtcatagcgatcgtatttgccaa
aatgtagccaggctttgctttcatcatgatgtccggcaggagtcgcggtgacgctataacgaaagttgccgcttaagttctgtaccttcagcgcag
tcataatgtcgcgggtattgggaatgacgtagcccagatcgggggtgaaatcgccccaccattgcaggtcatccagcgaggaatcctgacgc
agactgagcatgatttcggtgccatcattggtacgataaccgccatagaaacggttaattccgccctcgaaaccaccccagctatggtctggca
cccaggcattgccgtcgtggtcggcctgcacggtccagccttcgccgtaaatcaggccgaaccaggagccgtacttcatctccaggccacctt
caatataggtaccgtcgctgtatttatgtttattctcaccctctaaatccatatatccaccaacccctaattcgccgtaaaaacgaaatgaaggaga
agatgtcgaggtaattgcgggtgtttttgtgtcgtcatgttctgccggacgtgcttcttctcctgccgcagaaaaggcgtagcaaaacggcagagc
aagtaatgctctgcatggtgcagtgattaatttcattgtgttaatccatgagctaaagaaaaagtacagccttttatcgttgtaattataaaaatataa
aggcagaaactattttccctctcctcatccaggaagagggaaaatgcctgctcatttttattattctgcttaatcaacattgctggctggcgtcgcttc
aatatggcgctcttcagccgttaatccctttatatatttactgtatttccaccacgcaaagccaaggaagataacaataccgccgacgttataaaa
aatgatggtcagaatattcgcgccagtcgggaatgtcgaagcgacaaaccctacggcaaatatcgcaatcagcattgaaacaacgataata
ccggtgcggcgggagcccatacggaaatcgcgtggcaggtgatctaatttggcgcgtaaattcaggtaagccagcatgatgaataacggcg
gaagcatggacgctgcggcggtcatattaataatagtattcatcagatcctgcacggtattggaaccgagcatcgggataatcatcagcgggat
gacgatcaggaactggatccacgctgcgcgcgccggaacgccgttttcgttcagttcgacggttttcttaccaaagatgccttccgggatttcgg
agaagaaaattttcaccggcgttgcggtccacatcagcagggaaccgaacatcgcggtaaaggacaccagaccgacaaagcgattcatca
acgcttccggtagaccaaaatacgccgccatgccgtggaatacctgcaccgatccgccggtaaatttcaactctttgctgctgacgaagacgtt
aatcagcaccgaggagacggaatacagtacgccgataaaaatcccggcgaggatgatcactttaacgaacgatttcgaaccgcctttgacg
tcgttaacgtacacagcgacggactccgcgccacctgcggccataaagatccaggtggtaacgccgaggaacgcccagttgaagttcggg
atcatcgcgtcaacggtgatggcgtcagcaggctgtacgccgccaaccagcgccgtacctgccagtaaaatgtaggagagcgttaacagca
gcatcagcgttgaagtgacggaggtaattggccccaacattttcgccccgttggtggaaacccaggtggagaaggcgaacagcaccatact
gataatggtggtggcaaccggcgtcataatatattcgtatccgaggaaggcatacgaagcataagcaataacgcgcggcaacagtgaggtg
aaaaagaacaggtttacgaaccagtaggtataggcagtaataaatgcccaacgtccgcccagcgaactttttacccacgcgtagacaccgg
cttctgagtttttatttaacgaaacaaattctgcgatgatcagacagaagggaataaaataaaaaatcgtcgcgaggaaaaacatcggtgccg
aggccagtccaagctcaatattattattgataacgttgttaaagctgtaaacggcggcaaaagtcagcgagagcaagccgaatttgccgattgt
attacgtttggtatcagacataacacatctccgaaaatgagtagggtagcggtggcggcaaactccttttgccgccgtagttgttatttgttatgga
aataaccatcttcgatggtgactttgagaaccactttttgaccgcgttattgcagataaaccgatacgcttcatggatatcgcaaataacgatttgc
ccttcgtggacctcaacggtttctccgcagccttttaaatattcacggtcagtttcatcacgataatattccactacctgtaatgtctctttcggcgcata
ttcaatttttgctgcccttgcaggtagtaatgcacttcaaaatagcgtcgatgcccggtaaatagcgcatcggtcgcggagtctgtctccacgcgg
taagtcaatgagtcaccgatggagtgggcgacgccaggctgaatgttgtcgatattttcaatcgcttcaacgcagcgttgccacttcttgccaga
ggcgtaaatctggcggaactgttctaagttatcgatgatcctcattgcttattctcgctgtgcaaattcgtggaaaagaaccctgcgccgaactcat
acgacgccaggctttgcgcggtagcttctccgccagaaaccggcagcaacgtaaagccgtagctgaagtcacggaaccagacgcgccag
gagtccagcacctcgctgccccaggagttggagccgaggccaagcagctggtgatcgaggttcagggtgatgtcatcactgcgctgcagctc
gttacagtgctgggcagcgtggatgttttcctgggtatagtgccaggcgctgaagttaattgggcgctgcggaaccaccagcagaccgttaccg
tggcggttagtcagtgccgtccagcggacatgctgacggttaccgttgttctgcgggaagggatagttctcgaacatggcatcgacggtgctgc
gccagatatcgatgatgttagcctgctggctgtcggcgtagttttcgcccggtccacgaccgtaatacgccacctgatcgtattcgccgttaattcc

catggtgaaaccgatgcacggaatgatgtgcggatagtcgccgtaacgctcgccggaaagcgccacgttaacctggccatcggcagcgatg
cgccagatgtaggtgcagcgcatcccgaagtcaaacaccggcggggcaataactgtgcggctgatgatcagcacttcaccatcgctctgttct
acggcaaagtcgcgcagatgttcctgcatgatctgcaaatgattcggttgccacagcccttcgtactcctgcttgtggttgtcgatcatcggcttga
agaagttgatctttggctcgcgagtcagcagcgattcgccattcacctgccaggatgtcggtttgccactcatttttgagaaggtgatcgcgaagtt
gtagccgcgaacggtgcagctcaaacgatcgtcttccagcgtcagcggacgcgcattatttggtgcgaaaggcactggctgcgcggtgttttcc
ttcagcgggaactgataagtggcgatagggtgtccggcttcgctgtagcgggtgcgggaatctttggtcaccgtaatgttgaggaacgcttcgcg
ggcgtccagctgcggcagcgtgatctgcaaggggggcttcgctgttcggcgcaacgtcgcgcagtttaatctgctgcgtcgcgagcgtttcacctt
cggcgcgcacctctgcgtgcagggtgtagtcatcaagcgtggtaaaccacagtttattttcgactttcaactcgccgcgagtcagatcccgcgcg
tggatttttaccggcgcgataacctgtttgtactctttcaggcccggtcccggcgtctgatcggaatagatcaaaccatcaagacagaagttatag
ttgttgggatagtcgccgtagtcgccgccgaatttataccagacattgccgtggtcgtcctgtgcctggatcccgtggtcgcaccactcccagaca
taatgaccctgaatgcaatcgtgcttatagaagacgttctggtactccgtcagcccgcccggtccgttccccatcgcatgagcatattcacagatg
atgcgcggcttcggatgcgggtattcaccaaactcattcatcagcggcacgcgggtgtacatggtggaaataatatcgaccacttcagcatcgc
gatcttcttcgtaatgcaccagtcgcgtgtcatccagcgctttcgccgcatggtacatcgcgcggatgttacagccatagccggattcattgccca
gcgaccagatgatgatcgacggatggtttttctgcgcgtggatatggcgaacaatgcgctcgacgtagactttttcccactgcggatcgtcggta
atacggctaatatcgccgacattagcaaagccgtgcgattcgacgtcggtttccgccatcacaaacaggccgtagatatcacacagttcgtaa
aaacgcggatcgttcgggtagtgagcggtacgcacggagttgatattgtgctgcttcatcaactggagatctttctcgacgcgatccattccaac
ggcgcggcctttgcgatgatcgttgtcgtgacggttgacgccgtgcagcatcacataacggttattgatccagaacagaccgtcgcgcactttga
tatcacggaagccaacgcgttgtggcaccacttccagaacgttgccgttggcgtctttcagcgtcatgaccagatggtaaagataaggggattc
tgctgaccattgctgcggctgttcgacagtaaaagcaaagctggcgctggtcagtttttcaattgccaaatgatcaatggcgctgctgtgcaccac
gcgttcgccatcaaacagggtatattccagcgtcgtgacgacaggggaggcggcgagattttccagcaccacttcgcaggaaagcgtggcat
cgcaataggcttcgtcaaagtcggtacgcacagtgaaatcgttaatatgcgttaggtgttttccgaccagataaacatcgcggaagatccccgc
tgaccaccacatatcctggtcttccacgtaggtagagtccgcccactgcatcacgcgcacacacaacaggttgtcgccggttttaaccatcgcg
ctgatgtcaaactctgcggtcaggcgactgcccttgctgaaacccacatactgaccgttaacatagacttcaaaataggtttcgacgccgtcaa
atttaatcagcgtctgtttaccctgccagccgtcgctgagggtgaaaatacgttgataggcaccggttgggttatcgctggggacaaacggcac
atcgatggggaacggaaaaccttcgtcggtatattgcagtttgccgtgaccttccatttgccacatggcggggacggtaatatgcccccagtcag
ccattaactcagaggtgaaggcttctggtacttgcagcggatggtcaaaaaagtggaaattccactgaccgcttaagggcagaaacaggctg
ctggtttcgcgggcaaaggtacgcgcttgcgcaacagaatcatatgaaaaaaagtacgcacgcggcgcaagtcggttttcgtgggtgagctg
aatgttttcccagcgattcatagggcctcccggtagagagaaatcggaaccattcatggtgatggtagtttgtgtaattgctggggagtttagtaaa
atttttactaaaaagtagcaggtaaatgcatttactttaactggatcacgaaaataaacggcagcactgtccggcaaaaacgattgccggaca
gtgtgtaaggggatttaacgggtcgtgccgcgcagttttaatttgctgggaacgaagactaacagcggcagcgcgcgaccatcgcgggcttttt
catacaccaggttaacgccctgacttcccatcatctcggaatggatgcgcacggtggagagcggcggaaaggtaaatcgcgcggtggggat
atcgttaacgctgataagcgaaatatcctgtgggatgttcaggcctcgttcatgaattgcccgcagtacgccgatagcaatggaatcggaagca
acaaacagtgccttcggatagtcttcccgcgccagcatttgttttgccagttcataacccgacgaactggaaaaaccgccgcgccagatatcct
cttcgcgtaccacttgtttcagtcggccatattccgcaaaggcgacctcacgaatatccgccttgccaggctcatcttcaccgccaataaaacca
atacgattaacgccctggttgatatagaagtcgatgatttctttactgatgcgtgccagatcgatatccaccgcatcgtaaccgctgccgggttcgt
gaaagtcgataaaacagatattgtcggtcaacgcgctggcagcggcgcgcagggcgggcgtgggtttgccgacaattaaaataccggtgac
gtttttaatgtctggtaagccgctgtgttcataacagttggtgagctcgatgcccagcttttcgcactgggtttcaatgccgtggcggatcgccagat
agtaaggatcgttgatctccagctcctgctggtagctgtagatagccagaatatggtgttggttgactgcacctgtctggagtttacgggcactact
ggtcttgtactccagcttttcggcgatctcgagaatgcgatgtttcgtctcttctttcacattcaatgtcggatcgtcatttaagaccctggatactgtcg
ccagggatacgccagcttcgattgcgatgtcttttagtgttgccatgtttaccttttttactttccctgatgtgcgcccccattgtagaggaagtgcttatc
gggtcacctgttttcagtaaataaagcgcgtttctttgacgctgtaacggtaacacagcggctcacgaatgtgatcacgcctgcattctccattttta
gcctacaggattgagaaaaacttttgctatagtttagtaaaattttaccctaagggaggaagcaatggaaaccgtggcttacgctgattttgcacg
tctggaaatgcgcgtcggaaagattgtggaagtgaaacgccatgaaaacgccgacaagctgtacatcgtacaggttgatgtggggcaaaaa
acgctgcaaaccgtgaccagcctggtgccgtactacagcgaagaagagctgatggggaaaacggtggtggtcttatgcaatctgcaaaaag
cgaaaatgcgtggtgaaacgtcggaatgcatgctgttgtgtgcggaaaccgatgacggcagtgaaagcgtgctgttaacgccggagcggat
gatgcctgcgggcgtacgcgtcgtataaaaagatcggatggcgacgtcgtatcgccatccgatttgatattacgcttcttcgacacttactcgcat
ggccgccagcgccttacgcgccgctttgatcaccagttcacactgttcaatggtcagtgtcagtggcggttcaatgcggatcgttttggcgttattg
agcgttccggccaccagtacgcgctggcggaacatctcgctggcaaagttatagccgatttcgttatcaacaaactcaatcgccatcaacatcc
ctttaccacgcgcttcctgtaccagatcgggatattcccgcgccagttgacggaaaccgtccagcaacatatcgcctttttgctcagcctgagcc
ggtaagttctgctccagcaacacattgatggtcgccagcgccgccgcacaggccagcgggttgccgccaaaggtggtggtatgcaggaatg

ggttgtcgaacagaactgaaaacacctcttcagtggcgatggtcgcgccaatcggcatcacgccgccgccgagcgctttggcaaggcagag
gatatccggctgtacgttctcatgctcgcaggcgaacatcttgcccgtgcgccccatgcccgtttgtacttcatcgaggatcatcagtgcgccgaa
ctcatcgcatagcttacgtacggcggtgagatagcccggcggcggcagaattacgccaccttcaccctgaatcggttcgaggatcaccgcag
ccacatcatcaccggttttttgcactcgttaagagccgtgcgcatggcttcgatattgccaaacggcacatgacggaagcccggcagtaacgg
cataaacggtttgcggaaggtcgatttcgccgtggccgacagcgcgccaagtgatttaccgtggaacgcgccgctggtggcaataaaagtaa
acttgccgcgcggtgactggtaagctttcgccagcttcagcgctgcttcaacggactcggtgccgctattacagaagaagctgtatttcagtttac
cgggcgttagcgcagcaagggttttcgccaacatcgcccgtaacggatcgagcagctcctggctgtgcagcggttgtttcgcaagttgattctgt
acggcggaaaccacaactggattacggtgccccacgttgaaaattccaaaacctcccaggcagtcgataaactcctggccctgggtgtcgac
aagcgtatttaaacttcccgcttgccactctacggctccgtaatccccgccggcggtaacagatttgcgatactctaaaaaccccggattgacat
gctctttgaagtattcaatcacctctcggttaagtgctttcatctcctcatgatccagcgttcgcttctcaatgagattcagggcgtgggcgctgcacg
ctaaagccgatgcgctcgaaggtaacctgttcaaaatatgctccggaggctcgcgtatcacatgataccgatttaagtattgcagggattgcgc
cactccggctgcgatcgcgaaaatcgggataaacacaaattcggttatcatttgtgcaatatttaatgctatctgttaacatttgttgttattttgcacg
gtttttgtcgggtatggatttgatgattgctgcgcaaatgcacgttttcagggcaataattgcctgatttttgggcaagataattaacgttttcgtatagt
aacagagtgaaagtattttaaatcaaggaattagatttgtgtttaaatcgcaaattgcgatctaaatcaaattaatcggttaaagataaccgcagc
ggggccgacataaactctgacaagaagttaacaaccatataacctgcacaggacgcgaacatgtcttctcatccgtatgtcacccagcaaaa
taccccgctggcggacgataccactctgatgtccactaccgatctgcaaagctatatcactcatgctaatgacacttttgtgcaggtgagcggct
ataccttgcaagagttacaagggcagccgcacaacatggtgcgtcacccggatatgccaaaagcggcgtttgcggatatgtggttcaccctga
aaaaaggggagccctggagcggcatcgtgaaaaatcgccgcaaaaatggtgaccattattgggtgcgggccaatgcggtaccgatggtgc
gcgagggaaaaatcagtggctatatgtcgattcgtacccgggcgacggatgaagagatcgcggcggtggagccgctgtacaaagcgttga
acgccggacgtaccagtaagcgtattcataaaggcctggtggtgcgtaaaggctggctgggtaaactgccttcattaccgcttcgctggcgggc
gcgtggagtgatgaccctgatgtttatcttgctggcggccatgctttggtttgttgctgccccggtggtgacgtatatcctctgtgcgttagtggtattgtt
ggcaagcgcctgttttgaatggcagattgtgcgcccgatagaaaatgttgcccatcaggcactgaaggtggcgaccggagaacgtaatagtgt
tgagcatctgaatcgcagcgatgagctggggctgacattacgtgcggtagggcaacttggcctgatgtgccgttggctaattaacgatgtctcaa
gccaggtgtccagtgtcagaaatggcagtgagacgctggcgaaaggcaccgatgaactgaacgaacatacccagcagacagttgataac
gttcagcaaacggtggcgaccatgaaccaaatggcggcgtcggtgaaacagaactctgccacggcgtcggctgccgataaactgtcaatca
ctgccagtaatgcggcagtgcagggtggggaggcgatgaccacggtgatcaagacaatggacgatatcgccgacagtacccagcgcattg
gcaccattacttcgctgattaacgatattgcgtttcagaccaatattctggccctgaatgcggcggtggaagcggcgcgtgccggcgaacaggg
caaaggttttgcagtggtggcaggggaagtgcgtcatttagccagccgcagcgctaatgctgccaacgatattcgcaagctgattgatgccagt
gctgataaggtgcaatccggttcgcagcaggtacacgccgccggacggacgatggaagatattgtggcacaggtgaaaaacgtcacccag
ttgatcgcccagattagccattcaacgctggaacaggccgatgggctttccagcctgacccgtgcagtggatgagcttaacctgatcacccag
aaaaatgccgagctggtggaagagagtgcgcaggtgtcggcgatggtgaaacaccgcgccagccgactggaagacgcggtgacggtact
gcattaatcggtatgccggatccggcggttggagcacaatgcctgatgcgatgctggcgcatcttatcaggcctgcggggtgtgtagcgggtgt
aggccggataaggcgcgtcaagcgtcgcatccggcaattgcaccgcgccactggcggatgcggcgattaatccagttgcgtaatatcaaaa
gcggcgcggtcgatcaccgcaatgatctttttatttgtgcatcactgatatcgctctggttgacgcgtaaatccagcaccgctttaaaattatccag
cgcccgcttcatttgcgggttctggcgcagcgccgcgccaacgcaacgcgctttgatgcgttcttcaatcatctccacctgttcgcggttttcttcca
gccactgcgcgccttgttcggtcagcgcaatctgcttcttacctccttcctcttcgcggatggtaatcagcgactgctcctgcagaaaatccagcgt
tgggtagatgacgcccgggcttggggtgtaattcccctgggttagattctcaatcgctttaatcaattcgtaaccgtggctgtcatcgcgcgagag
aatatccagaatcaccagacgtaattcaccgtgaccaaagaaacgttgccgacgaccgccgccgcgaccgtggcgaccaccgcagcattg
accgtgttcatgctggtgaccgtgtccgcagtgctcgtgttctgacttctcacctttgcagcagccctcatggcgtggctggccttcatgtttacaaca
cccttcgtgatgatggctcatatcagcctccttttgcttaattagatatatctaatttatatctgatatgagcgttcttgcaagtgtaaaaatgatttttaaa
cgtggactcatctcacacgtaggccggataaggcgttcacgccgcatccggcgttgagtaatgatgcctgagaagaggcggttcgtcttatcag
actatagacatatcagttctacaaatcgcttgcatttatcatgattaacaatcattatcatttgcgagttttatttagatatatctgattaccatcacgaa
ggcgataacaatgaataacacccccccgctacccgcagcgcgttcgcaatgatctgcgcttccgtgaactgactgtgttacgcgttgagcgcatc
agcgccggttttcagcgcattgtcctcggcggcgaggcgctggacggttttacatcgcgtggctttgacgatcacagcaaactcttctttcctcaac
ctgacgctcactttgtgccgccaacggtaacggaagagggcatcgtctggccggaaggaccacgcccaccgtcgcgtgactatacgccgct
gtatgacgaactacgccatgaactggcgattgatttctttattcacgacggtggggtcgccagcggctgggcgatgcaggcgcaaccgggcg
ataaacttacggtggcaggtccgcgcggttcgctggtggtgccggaagattacgcgtatcagctgtatgtctgcgatgaatccggaatgcctgc
attgcgccgccgcctggaaacgttgagcaaacttgccgttaaaccgcaagttagtgcgctggttagcgtgcgggataacgcctgtcaggattat
ctcgcgcaccttgatggttttaatatcgaatggctggcacatgatgagcaggcggtagatgcgcgtctggcgcagatgcaaatccctgccgatg
attacttcatctggataaccggcgaaggtaaagtcgttaagaatttaagccgccgctttgaagcggaacagtatgacccacagcgggtacgtg

cagcggcttactggcacgcaaagtaactgatatataaaaagaaaaaggctgacgatttctcgtcagcctttgctatatctggtggcccctgctgg
acttgaaccagcgaccaagcgattatgagtcgcctgctctaaccactgagctaaggggccatggtagcggattataaagtaactccgtgtcgc
aatccagccattaccgcgcgcctgctgtttttataaacaatgtattttcaatccgttatacttttcctggtgatgttagaaaggagtaaatatggttgag
gatattttggctccagggttacgggtcgtgttttgcggtatcaaccctgggctttcatccgccgggactggttttccctttgctcatccggcaaatcgct
tctggaaggtgatatatcaggccgggtttaccgaccgtcagttgaagccgcaggaggcacagcatctgctggattatcgttgtggcgtcaccaa
actggtagaccgtccaacggtgcaagccaatgaagtttcaaagcaggagctacacgcaggcgggcgtaagctgattgaaaaaattgaaga
ttatcagccgcaggcgttggcgattctgggcaaacaagcatatgaacagggattcagccagcgcggtgcacagtgggggaaacaaacgct
caccattggttcgacgcagatttgggtgctgccaaatcccagcggtttaagtcgcgtttcactggagaaactggttgaagcgtatcgcgagctgg
accaggcgctggtagtgcgtgggcgataaaaaacgccaccgaaaaggtggcgtttgtgcagagggcctagtgccgggtgcggcgtaacgc
ctgatccggcctaccgattaatcgtccaggaagctacgcagcacttcagaacggctcgggtgacgcagtttgcgcagcgccttcgcttcgatct
gacggatacgttcgcgggtaacgtcgaactgtttacccacttcttccagcgtgtagtcggtgttcatatcgataccgaaacgcatacgcagaactt
ttgcttcacgcgcggtcaggccagccagcacgtcgtgcgttgccgcacgcaggctttcggtggtcgcagaatccagcggcagctcgagggtg
gtatcctcgatgaaatcccccagatgcgaatcttcatcatcaccgatcggcgtttccatggagattggctctttggcgatcttcagcactttgcggat
cttgtcttccggcatcagcatacgttcagccagttcttccggcgtcggttcacggcccatctcttgcagcatctggcgagaaatacggttgagcttgt
tgatggtctcaatcatatgcaccggaatacggatggtgcgcgcctgatccgcgatagagcgggtgatcgcctgacggatccaccaggttgcgt
aggtggagaacttgtaaccacggcggtattcgaatttatcaaccgctttcatcagaccgatgttgccttcctgaatcaggtcaaggaactgcaag
ccacggttggtgtatttcttagcgatagaaataaccagacgtaagttcgcttcaaccatctctttcttcgcacggcgggctttcgcttcaccgatgga
catacgacggttgatatctttaacctgctcgatggtcaggccggtttcttcttcaatctgctgcagtttttgcagggcgcgatgcacttcttcagagac
atcgtgcagtttttccgaccacggcttgttcatcgcaattgccgcgttgaaccaggtatcgctggtttcgttgccggtaaacagggtaatgaagttttt
cttcggcattttgcactgctcaacgcagagcttcatgatcagacgttcttgcgtacgaacgcggtccatcatgacgcgcatgctgttgaccaggta
gtcaaactgcttcggcaccaggcggaactgtttgaatacttcagacagtttcaggatctcttcctgagcggtagcgtgactgcgacctttcgctttg
atggtgtcacgcgttacaacgtactgagcgcgtagttccgcaaatttttcgcgagccagttccgggtcgatgctgttgtcatcatcggcgctgtcgt
catcgccatcttcttcgtcttcatcttcgtcatcgtccagatcttcctgggaaagctcagaaccgacgtgagtggcggtaggtgccagatcttcttctg
cgttcgggtcaacaaagccggtgatcagatcggacagacgcgcttcttctgcttcaacacgatcgtactgttccagcagataggtgatcgcttcc
ggatattcagcaacggagcattgaacctggttgatcccgtcttcaatacgcttagcgatgtcaatttcgccttcgcgggtcaacagttcaacggtg
cccatttcacgcatgtacatgcgtaccgggtcagtcgtgcgcccgatttcagattccacgctggaaagcacctgcgcggcggcttcggcagcat
cttcgtccgcggtgttttcagccagcatcagatcatcggcatccggtgcttcttccatcacctgaatgcccatgtcgttgatcatttggatgatgtcttc
gatctgatctgaatcgacgatatcttccggcagatggtcattgacctcggcataggtcagatagccttgctccttaccacgggtgacaagaagttt
cagctgtgactgcgggttttgctccataagacggtatccacacttatttcatgaggttggtgttgggcgattgacccgatgtccttcagccgttaacg
ctgaaatccaacggttgtttaccgccaacgataattacgagggcgtacttatatatttgccgctgcctctcagtgcggctgtcgggggcttcccgat
cgctcttcggcacttaagccgttaaatcactttttcgccagctcctggtttaatgtccagagctccaggcgttcttcgttgcttaaaccatgcgtgcgct
cacgagcgattaactcttcctggcgcagttcaagcagcgaatcaaacatatggttgagtgagtcggtgaaggtttgctcagcaatattcttatctg
ctatatcgtcccacatcgacagtttttcaagggtggcagcattatttgtaccacgatagtgctctaaaagttgcccggtggtcagacctggctggg
agagacaagtgttgaccagttctctgaataagccaagtccagggagcttattttcatccagattctcaagcggcgggaccaacgtcgctaattct
ggattttgcaccagcaaccctataagtatacgcatggtcgtgcgttttagctgcggaacagggcgagaaacgccgctctctgccgcttttggcatt
aatcgttcaagctggctgtcatcaagtatgcctaatttgttgcctaattcctgacgaagatatattcgcagcgtttcgcccggcacttgcgatatcaat
ggtagtgccagcgtactcaaacgtgcgcgcccgtcaggggtactcagatcaacttgcggcatcagactgttaaacagaaatgcggagagtg
gcatcgcctgctccatccgcgcttcaaacgcttctttaccttcttttcgtactagcgtgtcagggtcttcgccatcaggcaaaaacataaagcgtag
ctgacggccgtctgtcatgtaaggcagcgccgtttccagcgctcgccaggcggcatcgcggcctgcacggtcgccgtcataacagcaaatga
cattgttggtcgcgcggaacaacagttgtatgtgatcggcggtggttgacgtacctaacgacgcaacggcgtaattaatgccgtattgcgccag
cgccaccacgtccatatagccttcgaccacaagcagacgattgggttcagcgttatcctgctgcgcttcataaagaccgtaaagctggcggcct
ttatggaaaatgtctgtttccggcgagttcaggtatttgggggtatcgttgcccagcacgcgcccgccaaaaccaatcacccgaccgcgtttatc
gcgaatggggaacatcacccgctcgcggaaacgatcgtaactgcgtccctgatcgttagtgaccaacatccccgcatcaatcaatgactggc
gattttctggattgccgccaaaccgcttcaggacgttgtcccagccggggggcgcaaaaccaatcgcaaagcgagcgataacctcgtggctt
aatccgcgtttttccagatactggcgcgcagacgtggcaacaggttgttgtaaagattgttggtaaaacgtattcagaccgtccatcaactgataa
agcgtttgcctctgatggcgctcgatctggctggggccgctgcctgcttcaaatggcacttcaagattgtgcattgctgccagctcttcgaccgtttc
gacgaactcgagcttgtcgtagttcatcaggaagtcgatcgcgttgccgtgcgcgccacatccaaagcagtggtaaaactgtttctcaccgttaa
cggtgaaggacggggttttctcgttgtggaatggacaacacgcgtggaaattcttgccctgctttttcagcttcacacgggcatcgatcagatcga
cgatgtcagtgcgtgccagcagatcattaatgaatacgcgtgggattcgtccagccataggccccgatttttagcaattcataaacgaaaataa
gccgcgcattcctttcggaagcacggccttacaactacaactcggtctgatcggagagcaacgctctcggggaattagtacagacgagtgcg

gcgtgcgttttcgcgagccagtttcttcgcgtgacgtttcactgcagaagctttagcgcgcttacgttcggtagtcggtttttcatagaactcacgacg
acgaacttccgccagaacacctgctttttcgcaggaacgcttgaagcgacgcagagctacgtcgaacggctcgttttcacgtactttaattaccg
gcatgtgcctctcacctttgattaattcggtttgccgctggcatcaacgccagcttatttcaaaatggtgcggaattttactgcaattgctgctgctttgt
aaagcaccgcggcctttttttgaaagggacttttataagggtgaggagtatacacgaggctttctccaggggcgaacaaagttttacatcaaccc
gcattggtcctacactgcgcggtaataaagcgaggtaaaacaagtcatgcgtgtactgggtattgaaacttcctgcgatgaaaccggcatcgc
catttacgacgatgaaaaaggtttgttagccaaccaattgtatagtcaggtgaaattgcacgctgactacggcggcgtcgtgcctgaactggcct
cccgcgatcatgtgcgtaaaaccgtaccgttgatccaggcggcgctaaaggagtctggtttaacggcaaaagacattgatgctgtggcctata
ccgcaggccctggattagtcggcgcgctactggttggcgcgaccgtggggcgttctctggcgtttgcctgggacgttccggcgatccctgtacac
catatggaagggcatctgttagcgccgatgctggaagataacccgccggaatttccgtttgttgcgctgcttgtttccggcggtcatacgcagtta
atcagcgtgactggcattggtcagtacgagctgctcggcgagtctatcgatgatgccgccggggaagcgtttgataaaaccgcgaagctgctg
gggctggattatcctggcgggccgttactgtcgaaaatggcggctcagggtactgccgggcgctttgtcttcccgcgtccgatgaccgaccgtcc
ggggctggatttcagcttctccggcctgaaaaccttcgcggcaaataccattcgtgacaacggcaccgacgaccagacgcgtgctgatatcgc
ccgcgcctttgaagatgcggtggtcgatacgctgatgattaagtgcaagcgggcgctggatcagacgggctttaagcgactggtcatggcggg
cggcgtgagtgctaaccgtacgttacgggcgaagctggctgaaatgatgaaaaaacgccgcggcgaagtgttctacgcgcgtccggaatttt
gtactgataacggcgcgatgatcgcctatgccggaatggtgcggtttaaagcaggcgcgacggcggatctcggcgttagcgtgcgtccgcgct
ggccgctggcggagttaccggctgcgtaaaactattgggtgccggagagcaatttccggcaccgtcctcacttaaagcaacaccacgggcat
ccacagtaagccggtaatcaccagcaatacgaggaatatcagcccaaaaatcgcccccagtcgccagtaatccgccgttggcagataacc
actgccgtagtaaatcggactggggccggtggcgtatggtgtaagaatgctccccagaccaattgccgcaccaaccatcaggcagaacacc
ggcagcgggatttccggcatcgccagcgcagcagcaatcatcatcggtgcgagagcggaggtatacgccgtggcgctggcgaaaaagta
gcgcagtagataaaacaccacaatcaacgccaccatcaccatcgttggcgaataaccgcttaagctgcctgctaacagtttgccaaaccagc
taataaaaccggtgttgttgagtccggtggcgagggtgataagcgaggccagccagaagaaaacgttccacgccgctttattactgacaatgt
cgtcccaactgataatgcgcagaagcagcatcagtgccaccacgctgtaaccgaccatcgcggcatcgatataatcaccgccgaaaatcca
cagcaccagcgcgcctaccatcagccccagcatccgtttttcacgcgaacagagcgggcccattgcctgcagttccgtctctgcccagcgcg
gcacctgatcgccagacttcagtaccggcgggtacagcacgtaagccagccagggaaccagcagaaccagtaaaatgctgagcggcaa
catcccgaggaaccagtcgccccaactcagcgtggcgtgagatgcgcttttcatcagtccaattaacagcaagtttggtgccatcgccgtcaag
aaaatggcgctggtcacgcagtcggcaacaatccccatccacatgatgtacgagccaatgctgcgcgaactgctgtcgtttggttgtgattgata
gagcggtggcaggttacggatgatgggatagataatccccgcaccacgcgccgagttggacggtgttacaggtgcgaggatcagctcggag
aacatcaccgcatagccgagaaacagcgtgcgatgtcccatctttttcaccagaatcagcgcaatgcggcgtccaagcccggtttttcatagc
ctgtgccaaacataaaggcggcgaaaatcagccagataaccgaattagaaaaaccggaaacggcccacgagagggattttgcagtgaatt
taaagcctggctgagcgagctgctccgggctgaacagcagccagggagagagtatggcgatgatggatatacccaccatcgccaccacgg
cacccggcacgggttcgaggatcagtccaacgatcacgccagtaaaaacggcaaagtagagccaggtatggttctccagccctgcgggaa
ccggaagtagagcaataatggcgatgaccgccagcggcgcaagatatcgccaccattcagtggaaggtttcatcgtgttatgcctgcgaaaa
aggagaggggtgagcctctccgggagggttatttgatgtaatggacgtgctcgcagatctcttccacgatgggatcgcggcgttcggcgaaca
gctttttgttttcggctatcaggttgttgccgtgggtatcaatagagacaatcagcgggccgaactctttgacccggcagacccacagtgactccg
gcattccgagctctgtccagtgcacttcttcaatctcttccacctgggttgccgccagcaccgcgcagcctgccgggaaaatcacatgtagcgcc
ttgaatttctggcagccttcttcggtcagcggccccataccacctttgccaaccaccagtttcacgccggtctgctcaataaattcacgttcaaaac
tttccatacgcatactggttgtcgggccgacggagaccatctcccatttgtcgccgtttttgcgcacgatggggccagcgtggaaaatcgctttgc
cgttgagatcgtaagggatcggacgtttcagttcgatcaaacggcggtgacaaacgtcgcggcaggtcaccagcgtaccggtcaggtagatc
acatcgccgacgcgaatatcttgcagatcttcagctttgatcggggttgtcaggatctttttcataacgcgctccgggtgtgagacagattttcaaa
ggtgagatcggcatgaaccagcagcgtgccgcgacgatgcgcccagcagccggtagagacagcaacgccgatggttgacggatggcgg
gcggcagattcgatatgtacgcccatcactgaactgttgccggtcagcccttgtggaccaatccccagacggttgagtccttcttccaggcgtag
ctccagttctgccgcttttggattgggatggcggctgccaatcgggcgcaaaatggctttacgcgagagtacggcggcggtttccaccgaggtg
gcgatgcccacgcccaccagtaccggtggacaggcgtttacggcgagggtggagatattttcgaagacgaatttcaccacgccttcgtagcct
tctgacggcattaacactttcgagcggccaggtagcgtgcagccgccgcctgccatgtaaacttcgatttccgcatcgtcattgtcggggatgat
gtcccaggtgacccacggtacgccgctaccggtatttttgccggtgtttacttcgtcaaaaatttctaccgcattgtgacgtagtggcgctttgacgg
ttgcctcttccacggcttgtttgagtatgctttgcagctcgccaagcagtgggaagcgggaaccgactttaacgaagaacataatctccccggtgt
cctgacaggcaggacgattcaggtcaatcgctttttgcatgttgtcgaacatcgtatggtagataattttccccatcgacgacgtttcggcatccttta
gctgttttagtttatccaccacgtcatcaggcattcgggtagaaatcatggcggtaaagttagcgacaatctctgtcaacttattcactgcctgttgct
tattactttcgctcatcatggcaactccagtttttgtatttgtctggaaaaattcaccgccacaaactaccactaaaaatgtggttttatatttttgccac
cgtgaatatgtaattaacagttcgtgaatcaacgcgaaaataagaatgaatattgcgccagagcagggaatggcaatgttttgcgaaatacact

gcgcttatcacaactgcggataattaacgatgctgaatagctggcctttagccaaagacttgcaggtactggtagaaatcgttcacagcgggag
ttttagcgccgcagcggcgacgcttgggcagacgcctgcatttgtcaccaagcggatccaaatccttgaaaatacgctggcaacaacgctgct
gaaccgctccgctcgcggcgtggcgctgactgaaagtggccaacgttgttacgaacatgcgttggaaatcctcacccaatatcagcgactggt
cgatgacgtcacgcaaatcaaaacgcgcccggaggggatgattcgtattggttgcagttttggttttgggcgcagccatattgcgccagctatta
ccgaactgatgcgcaattatcctgagttacaggtgcattttgaattgttcgatcggcaaattgatttagtgcaggataatattgatctggatattcgtat
taatgacgaaattcctgattattatattgcgcatcttttgacaaaaaataaaagaatattatgcgcagcacctgagtatctgcaaaaatatccgca
gccacaatccctacaggaattaagtcgtcatgactgtctggtgaccaaagagcgcgatatgacccatggaatatgggagttggggaacggtc
aggagaaaaaatcggtgaaggtaagcgggcatctttcctccaatagcggcgagattgtgctgcaatgggcgctggagggcaaagggattat
gctgcgttccgagtgggatgtgttgccgtttctggagagtggcaaactggtgcaagtattgccagagtatgcacaaagcgccaatatctgggctg
tttaccgggagccgctctatcgcagcatgaaattacgtgtctgcgtggaatttctggcggcatggtgccagcaacggctgggcaagcccgatga
aggctatcaggtcatgtagatccaccagaaatcactcgggatccttttcgcgctttcttttgaatttcgtccagatttttgtctcctgacgacgccaca
gacgttggatgttgtcatgatgacgcagcaggatcaggcaagagagcatcgaaaccgggaaggtgaattgtggcttaaaccaccagacata
aaacggagcaatcagtgcactgacaatcgctcccagcgacgagtatccgctcaatagcacggtcagtaaccaggttcccgccattactccgg
tgagatcccagccaatgggtgcgatggcaccaaaagcggtagcaacgccttttcctcctttaaatccgaagaaaacgggccagatgtgtcca
agacaggcggcgatggcaattaagcctagccagaaggggctgacacctaattcatacgcgccccagacgggcaacattcctttcagaacgt
cgaaaatcagtactgctacggctgctcccttgccaccgatacgtaacacattggttgcgcctggattgccggagccgctggttcgcggatcggg
cagcccacacaagcggcaaaccagaatggcactggaaatggagccgcagaggtacgcgatgaggatcattccaggcgcgattgcactca
taagctgttccgttttgaaaattcgtgttcaacgatgaatctgtggataatacgcacatttcgccggaagtggtatccggttagccaaaaagcagg
caggacgtgatggatattgtatttatagagcaactttcggtaatcaccactattggtgtttacgactgggaacagaccatcgaacagaagttagtg
ttcgatatcgaaatggcgtgggataaccgtaaagcggcgaaaagtgatgatgtggcggattgcctcagttacgctgacattgcagaaacggtg
gtcagccacgtcgagggggcgcgttttgcgctggtggaacgcgtggctgaagaggtggcggagctgctgttagcacgcttcaactcgccgtg
ggtgcgtatcaaactcagcaagccaggcgcagtggcgcgggcggcgaatgttggcgtaatcattgagcgtggcaataatctgaaagaaaat
aattaattttacagctgttaaaccaaacggttataacctggtcatacgcagtagttcggacaagcggtacattttaataatttaggggtttattgatga
gcgatatgcactcgctgctgatagcggcaatattgggtgtggtcgaaggattgacagaatttctgccggtatccagcacgggccatatgattattg
tcggtcacttgttggggtttgagggcgacacggcgaaaacctttgaagttgtgatccagttaggatcaattctggcggtagtagtgatgttctggcg
gcgtctgtttggcctgattggcatccactttggccgcccgttgcagcacgaaggtgaaagcaaaggtcgtttaacgctgatccacattttgctggg
gatgattccggcggtggtattggggctgttgttccacgacacgattaagtcattgtttaacccgataaatgtgatgtatgcgctggtcgttggcggttt
gttgctgattgccgccgaatgcctgaagccgaaagagccgcgtgcgccgggtcttgatgatatgacctatcgtcaggcatttatgattggctgttt
ccagtgtctggcgctgtggccgggtttctcccgttccggggcgaccatttcaggtgggatgctgatgggggtgagccgttacgctgcttccgagttt
tcgttcctgctggcggtgccgatgatgatgggcgcaacggcgctcgatctctacaaaagctggggcttcctgacaagcggcgatatcccgatgt
ttgccgttgggtttatcaccgcttttgtggtggcgctgatagcgattaaaaccttcctgcaattgattaagcgcatttcgtttatcccgttcgccatttatc
gctttattgtggcggctgcggtgtatgtcgtgttcttttaattgaggaatagtgtcggatgcttggcgttgtcaacgattgcgtaggccgaataaggcg
ttcacgccgcatccggcaatcaacgcctgatgcgacgcttgcgcgtcttatcaggcctacaactgtcgttgacatgtagccggataaggcgttta
cgccgcatccggcaaaatgcccaatactcattcaggctttgggcaacgttgttccttccagctggctaccgccgcaatccgccgtcgggtcagct
cctcgcgaatctccacacctttaaatcccgcttcaacgacggcttttgtcggcactgactgcgccacttcccaggcttcgcgcaaccagcggcct
tgcgggtagtccgctgattcaaaaccggttctgccgcgcacgtcagcctcgctggtcagcgccagttgctcgacacgctgcggtttacgccagg
catcgatggaatcaaataatttgacgatggtttttgggttcagcattgggaaggtgtggatgagatcgtgaaactcagccaccagtctggctaaat
cgcgaatttcatttggcacgcgcagacgctggcatagttgttccactaacttaacacccgccgggccatgaccatgatgacgcggccagagttc
tggcggcgtcagccctttaccgagatcgtggcataaagttgcgaaacggacatcgacctgcggactcagcatcgccgccattgagagcgtca
ttaaggtatgaatacccgtatcgatttccggatgccacttggcaggggccggaacgccaaacagtgcgtcaatttccgggaataaaacgcgc
agtgcgccgcaatcgcgcagtacctggaagaacacctgtggattgcgggtggtgagggcgctttccgtctctttccatacccgttcaggcgtca
ggtgttccagttcacccgcatgggtcatctcgcgcatcaacgccagagtttcatcggcaatacgaaaaccgaggtgggcataacgcgcagca
aaacgcgccacgcgcaatacgcgtaacggatcttcgccaaaagcgggggaaacatggcgcaacagacgattttgcagatcgcccagacc
gttgtacgggtcgataatctcgccgttatcgtcctgggccagcgcattaatggtcagatcgcgacgcttaagatcatcttccagcgtgacatccgg
tgcggcatagcaagtaaaaccggtgtaaccggaaccggatttccgttcggtacgtgccagcgcatactcttcatgcgtttgcggatgcagaaac
acaggaaaatcgcggcctacctgctggtagcccgcgtcgagcatctcctgtggcgtactgccgaccaccacccaatctctgtctttgaccggta
gccctaacaatgcatcccgaacagcaccaccgaccagataaatcttcacgccatcatcccttcgtcaaatacacaattcctaataataagaca
gtgcaacgaagaaggcgatttagttcatccagcgatctttgcgtttgcggcttgggatcaggtgcggcagtaccagaccgagcagcaagcca
agccccagcacgccaccgccatacataaaccattgcatgatgatggtgcgctgtttgtcatccagctgtacgctggcggcatcgacctttttctgc
gcgacaatcagctcgttttcagtttctgattttcttcttttaacccgttgatcacgctgtcgctctgcgccactttttgctgcatttctgcggtgcgctgattc

caggtgttatcgatattggtgagtttatcggtcagggttttgacctgattttccagatctggcacacgggagcgcaggcttggctcagtgctaagttg
tttcaacgggatccaggcggtacggccagagctgtctttcacctgggcataattggtgttggcgtcagtttgtaataaggtcacttcctcgccggcg
ttaaccgtgcccacgaggcgataatgatctcccggaccgctgcggacccaggtattcagttcgtcggaaacatagcgcgtttcttcagcgtgtg
agacggcagtcgcgctaagtgcgagtaaagttaatccgatcaggcgtaattttggcatcaggctgtcgttattgtcatggaaagtggaacgata
gtagtggcatcagtgtcgctacgcaaagcattcgacatcaatcggaatcctctgtgtcaggtcttgccactgcttcaaacttttacgcccgtcaaat
tgcccgttgcatggcaatttggcgcaaaatactatctactgacaaaaaagatcgcattaagttcgccgttcatcgtcacttatgtgacataaccga
taagtaaaggccatggctcaggaaatcgaattaaagtttattgttaatcacagtgccgttgaggcgttgcgtgaccatctcaatacgctgggcgg
cgagcaccatgaccccgtgcagttgctgaatatttactacgaaacgccggataactggctgcgtgggcacgatatgggcttacgtattcgtggc
gaaaacggtcgctatgagatgaccatgaaagttgcaggaagagtgacaggcggcttacatcagcgcccggaatataacgtggcgttgagc
gaaccgacgctcgacctggcgcagttaccgacggaagtctggccgaacggcgaattgcccgccgatctcgcctcccgcgtgcagccgctgtt
cagcaccgatttttatcgcgaaaaatggctggtggcggtcgatggtagccaaattgaaatcgccctcgaccaggggggaagtgaaagcgggtg
aatttgctgaacctatctgtgagctggaactggaactgcttagcggcgacacgcgcgcggtgctgaaactggcgaaccaactggtatcgcaa
accggattacgccagggcagcctgagcaaagcggcgcgtggctatcatctggcgcagggcaatccggcgcgtgaaatcaaaccgaccac
cattttgcatgttgcggcaaaagccgatgtggaacaggggctggaagcggcgctcgagctggcgttagcgcaatggcagtatcatgaagaac
tgtgggtacgcggcaacgatgcggcgaaagaacaggtgctggcagccattagcctggtccgtcatacgctgatgctgttcggtggtattgtgcc
gcgtaaagcgagcactcacttacgtgatctgctgactcaatgcgaggcgaccattgcttctgcggtgtctgccgtgacggcggtctactctaccg
aaacggcaatggcgaagctggcgttgaccgaatggttggtaagcaaagcatggcagccattttagatgccaaagcgcagggcaaaatca
gcgactccttcaaacgctttgccgatatccatctttcccgccatgccgctgaactgaaaagcgttttctgccagccgttaggcgatcgctaccgtg
accagttgccacgcctgacgcgtgatattgactcaatactgttgctggcgggttactatgatcctgtcgtcgcgcaagcctggctggagaactgg
caggggctgcatcacgctattgcgaccgggcaacgcatcgaaattgaacatttccgtaatgaggcaaacaatcaggaaccgttctggttgcac
agcggaaaacgttaatcaggcaaggataaaatcgcttatgaagccgctctcttcaccgttacagcagtactggcagaccgttgttgagcggct
gccagagcctttagccgaggaatcacttagcgcacaggcgaagtcagtacttacttttagtgattttgtgcaggacagcgtgattgcgcatccag
agtggctgacggaactggaaagccaaccgccgcaggccgacgaatggcagcattacgcggcatggttgcaggaggcgctctgtaatgtga
gtgacgaagccgggttaatgcgcgagctgcggctattccggcggcgcattatggtgcgcatcgcctgggcgcaaacgctggcactggttactg
aagagagcatattgcagcagctcagctatctggcggagacgctgattgttgcggcgcgtgactggctgtatgacgcctgctgccgcgagtggg
gaacgccgtgcaatgcgcagggcgaagcgcaaccgctgctgattttaggcatgggtaagctgggcggtggggagctgaatttctcctctgata
tcgatctgatttttgcctggccggaacatggttgtacgcagggtggacgccgggaactggataacgcgcagtttttacccgcatggggcagcg
gctgattaaagtgctggatcaaccaacgcaggatggcttcgtctatcgcgtggatatgcggctgcgtccgtttggcgaaagtggcccgctggtg
ctgagctttgccgcgttggaagattattaccaggagcaggggcgcgactgggagcgttacgcgatggtcaaggcgcggattatgggcgatag
cgaaggcgtctatgctaacgagttgcgtgcgatgctgcgcccgtttgttttccgtcgttacatcgatttcagcgtgattcagtcgctgcgcaacatga
aagggatgattgcccgtgaagtgcgtcgacgtggtttgaccgacaatatcaaactcggcgcaggcggcattcgcgaaattgaatttatcgttca
ggtgttccagctcattcgcggcggacgcgaaccgtcgctgcaatcgcgctctttactgccaacgctcagcgccattgccgagctgcatctgctttc
tgaaaacgatgctgaacaattgcgagtggcgtatctgttcctgcggcgtctggaaaacctgctgcaaagcattaacgacgaacaaacccaga
cgcttccttctgatgagcttaatcgtgcgcggctggcgtgggcgatggactttgctgactggccgcaactgaccggggcgctgaccgcacatat
gaccaatgtgcgccgggtgtttaatgaattgattggcgacgatgaaagtgaaactcaggaagagtcgctgtcggaacagtggcgtgagctgtg
gcaggatgcgttgcaggaagatgacactacgccagtgctggcgcatcttagcgaggatgatcgcaaacaggtgctaacgctgattgccgattt
ccgcaaagagctggataagcgcaccatcgggccgcgaggacgtcaggtgctcgaccatctgatgccgcatctgctaagtgatgtctgtgcgc
gtgaagacgctgccgttacgctgtcgcgcattaccgccttgctggtggggattgttacccgcaccacctatttagaattgctcagtgaattccccgc
ggcgcttaaacatttgatttctctgtgtgccgcgtcgccgatgattgccagccagctggcgcgttatccattattgctggatgaattgctcgatccaa
acaccctttaccagccgacggcgaccgatgcctaccgcgatgagttgcgccagtatttgctgcgcgtgccggaagatgacgaagagcaaca
gcttgaggcgctgcgtcagttcaaacaggcgcagctgttacgcatcgccgcagcggatatcgccggtacgctaccggtgatgaaagtgagcg
atcacttaacctggctggcggaagccatgatagatgccgtcgttcagcaggcgtgggttcaaatggttgcccgctacggtaagccgaatcacct
gaacgaacgcgaagggcgtggttttgcggtggtcggctacggcaagctgggcggctgggagttaggctacagttccgatcttgaccttatcttc
ctccatgattgcccaatggatgcgatgactgacggtgagcgggaaatcgacgggcggcagttttatctgcgtctggcgcaacgcattatgcatc
tgttcagtacgcgtacctcttccggcattttgtatgaagtggatgctcgactgcgtccgtccggggcggcgggaatgctggtgacatccgcagaa
gcatttgccgattatcagaaaaacgaggcctggacgtgggaacatcaggcgctggtgcgtgcgcgtgtagtgtacggcgatccgcagctcac
cgcgcactttgacgcagtgcgtcgcgagattatgacgctgccgcgtgaaggtaaaactctgcaaacggaagtgcgggaaatgcgcgagaa
aatgcgcgctcatctcggcaataaacatcgcgatcgctttgatatcaaagctgatgaagggggaattaccgatatcgaatttattacccaatatct
ggtgttgcgctacgctcatgaaaaaccgaagttaacgcgctggtcagacaacgtgcgtattctggaactactggcgcaaaacgacattatgga
agagcaggaagcgatggcgctgacccgtgcttacactacgcttcgcgatgaacttcatcatctggcattacaggaattgccgggccatgtgtcg

gaggattgcttcaccgcagagcgtgaactggtgcgggcaagctggcagaagtggctggtggaagaatgaagtatggtattatcgcgcgcaa
attttgaatctctcaggagacaggaatgaaagtaacgctgccagagtttgaacgtgcaggagtgatggtggttggtgatgtgatgctggatcgtt
actggtacggccccaccagtcgtatctcgccggaagcgccggtgcccgtggttaaagtgaataccatcgaagaacgtccgggcggcgcggc
taacgtggcgatgaatatcgcttctctcggtgctaatgcacgcctggtcgggttgacgggcattgacgatgcagcgcgcgcgctgagtaaatctc
tggccgacgtcaacgtcaaatgcgacttcgtttctgtaccgacgcatccgaccattaccaaattacgggtactttcccgcaaccaacagctgatc
cgtctggattttgaagaaggtttcgaaggtgttgatccgcagccgctgcacgagcggattaatcaggcgctgagttcgattggcgcgctggtgctt
tctgactacgccaaaggtgcgctggcaagcgtacagcagatgatccaactggcgcgtaaagcgggtgttccggtgctgattgatccaaaagg
taccgattttgagcgctaccgcggcgctacgctgttaacgccgaatctctcggaatttgaagctgttgtcggtaaatgtaagaccgaagaagag
attgttgagcgcggcatgaaactgattgccgattacgaactctcggctctgttagtgacccgttccgaacagggtatgtcgctgctgcaaccgggt
aaagcgccgctgcatatgccaacccaagcgcaggaagtgtatgacgttaccggtgcgggcgacacggtgattggcgtcctggcggcaacg
ctggcagcgggtaattcgctggaagaagcctgcttctttgccaatgcggcggctggcgtggtggtcggcaaactgggaacctccacggtttcgc
cgatcgagctggaaaatgctgtacgtggacgtgcagatacaggctttggcgtgatgaccgaagaggaactgaagctggccgtagcggcagc
gcgtaaacgtggtgaaaaagtggtgatgaccaacggtgtctttgacatcctgcacgccgggcacgtctcttatctggcaaatgcccgcaagctg
ggtgaccgcttgattgttgccgtcaacagcgatgcctccaccaaacggctgaaaggggattcccgcccggtaaacccactcgaacagcgtat
gattgtgctgggcgcactggaagcggtcgactgggtagtgtcgtttgaagaggacacgccgcagcgcttgatcgccgggatcttgccagatct
gctggtgaaaggcggcgactataaaccagaagagattgccgggagtaaagaagtctgggccaacggtggcgaagtgttggtgctcaacttt
gaagacggttgctcgacgaccaacatcatcaagaagatccaacaggataaaaaaggctaaccggaaagcggttcacagatctcgcttcgt
acatggcaggcctattgacgctgttgtttttcaacctagagtaaaggaacaagggtaagggaggatttctccccccctctgatgagttgttagtaa
gtcgggaaacttaacagtaacaacacaaccagtatgatgacgagcttcatcataaccctttccttatacaaggccccttcttcgggaggggcttt
cccgtttcagccccttgctgcccccccgattgttgatgagcgccggaccccgcgcaatgctttcccagaggcaattttgccttccccgagcggtca
cgcaaaacgctgcaacgtcctgagcatttgcaccttttagcggaacgcagatataaaaaatccccggctttttgggccagggatatgtgttaatg
aaaccttcttgacgctgattttttttcaacctaaagtaaaggaacaagggtgagggaggatttctccccccctctgattggctgttaataagctgcga
aacttacgagtaacaacacaatcagtatgatgacgagcttcatcataaccctttccttctgtaaggccccccttcttcgggaggggctttcccgtttc
agccccctgctgactccccgattgttgatgagcgccagaccccacgcaatgctttctctgatgcagttttgccttccccgagccgtcacgcaaaat
gctgcaacaacctgatcatttgcactttttagcggaacgcagatataaaaaatccccggctttttgggccagggattattcctggaagagggttta
ctctgctttttcttcgacgggagttgttgaggttaagggtgaagtcaatgccgccagtgagccgcctgaaacgccaatttcattgagcaaggagtc
aatcagcggtgcctgtgtgcggtaagagagagcggctgataatgcttgttccgccaggtttccgccccccacattacccgtgtttgcatcacccg
cagcgccgccacgattcagaccatcgacctgaataatcttgataccgtcgattgacttcatcggctcaacggatttctctattaccgcaggcagc
gcctgcaacaaggccagtttgaatttaaggctggtttgttcatcagaaagtacgttgatagcatcgttcagcgcacgttgtgcttctgcttccgcca
gacccttttttacgtgtagcttcggctaactcaacgatagccgccgcctgcatctctgcggcttctttttctgctttcgcccgcacggtcagttcaaccg
ctttggtttctgcatcctgcgcggcagcgattagggcaacttgtttagcacgatcggcttcggcagtctggcgagtggtttctacgttttgttgggcgc
ttaccgcttctgcaagtgcgagattagcacgcgcttctgcctgggactgttgttccgatttggcggcaatagcgatcgatttcgtctggttagcgattt
cggtgacctgctgctgttcgatctctttaatgcgaacttcacgttcagcttcaacctttcttgagcggacggcctgttcgcggtcgatttctgtttcctga
atctgtcgttcagccagaattcgtgtctgctctgcttcacgacgacgttcagcttcaaaagccgcaatacgtgcattctgttcggcagtacgggtttt
aacctgctgctcctgctcaagcgtcataaacgcttcttgctgttcaatctccagcttgcgcgatagcgcatcgcgatttttttcacgcaccgcaactt
ctacatcctgttcaacttcgttacgttcgcggcgacggcgctctgtttcctgagtcagtttggttaaaccttcggcgtcaaaggcattgttcggattga
aatgttctttcgaggtctggttaaagttggtaagtgaaacgctctccagttccagaccgtttttcgacaggtcttctgccactgtattttgcacccccctg
cacaaagttctcgcgggtatcctgtaactcatgcatggtcatttgcgcagctgttgcacggagggcatcgacaaatttatcttcaaccaacatacg
taagtcttcaggcgacagggtgcgttgccccagcgtctgggcagcggtggcaatcccttctactgaaggttttacccgcacaaagaaagcgac
tactacatcgacgcgcatacgatctttcgtaatcaggctatcaatggttgagcggctgacttccagcttcagagtattcatattgatggggattatttc
atgaaagatcggcatcacgattgcgccaccgctcattaccactttttgcccacctaaaccagtacgaacaaaagcttgctctgccgaagcgcg
acgatagagcctggcgaaaataattccaataataaacagaatgcatacggcaataattgcggtaaacatccaggagggcacagaattaac
aatatcatccattattaaatttccttttgattaataaattacaatatttggggccagggattattttccgccaaatatcgcgttgcagaaagtcggcaa
ataattaacactttgtctcctttagtgaaaatttttccttcttcaggctccagtagtaaataatgtatttggccaaattgatcggtgagttttccttcgcagg
gattacctgaagtggcttgatgaccggtaattaacgccatgctaccaatatattcttcttctgtaatagctgaactatggtcgcgtggaatccaggg
cgcaactattttaccggtataatgcaccgcaataatcgtaaacagtaaactgacaggaaccacgaacaggttcgacagtggtgattgccaga
ccatgatgcaggcatgctgcaacaggatgccaataagaccaaaaaaaccagccagtaagcagagaacgacgagagctggcaatcttcc
aatattgagataatgaagtgcctggctaatatgacctgtggttatagaatcataatggtcaagatgtgcatcaagagcaccggagagcatgtga
ccgcagataagcgcaaaaatttctaacaggccgatcaacagtacaaaggaaatagcgaaaagataaggggtgttatagtcggcgaataaa
atcatatatcactccaatgtccatttagtgacgcaatatttactttgtaatagtttccttgcttacatttatctgtgttaataataacacaggttagagaag

ttctggaagagaaagtcttttaatatggtttttatttttcactaaaaagtgtgatcggggacaatatatttacgcacgttatgtttaaaggcactacact
gattggggaatactgaaatcagaaaaagcatatggccttaaaaggtatgcgataaccggaggatgtgcttatggatcatagtcttaattctttaa
ataatttcgatttcctggcgcgtagttttgccagaatgcacgcagaaggtcgcccggtcgatattctggccgttactggtaacatggatgaagaac
atagaacctggttttgcgcacgttatgcctggtattgtcaacagatgatgcaggcaagagagctggagttagagcactgatataacggcctga
tggcccgttttagtgtttgtattagatactagttataggtgattttcaggattacgtctttactaaacggtccctcaacaaccgtttctgccgggttcgca
ctcagttctgccctgaatggtgcggtgaccgaaggcgcactagacgatgaaaaagaagcaaattgtgagaagcggttaaattccgttgctgttc
catcgttatgaaatatcttcaatagcagaccattacctatatccagatggctataatcactcaaaatatccgtagtgaaaaaactccctaaaatatt
aaactgctcagtacaggccgcacccggatctttggtcatgactacgctaaacgtctcactcacgctggtatttttaattgttcgggaatttattttacc
aaagtcgacaacgccatcagctggttccagatcaacattaacaaagcagtcaaggacatgaatgttatttaatccgcgaatcctgaaggtcag
atttttatctacttctatagcacctgctccgctcatacctttaaattcaagtgctatatattcgtcaggtaatacaagtttttttatttactttattggcatttttag
ctagcttaatatatagctgcactcgtactggaaaagttactgataattgcggttcaggaccgccactacagactgcgggctttttgtagccattccc
gaaggttccggctccctttatatcaatacatacaggtgctgcaataggtagtggagatgtagaatcataatcaaccccattataagttacaccga
aagtataagagttttgattataaaagtcttgacctgatgctaacaacatcattatatttgcccatgcagatgggtattcttctcctggagatgggcctg
gtagtgcattattacagaaaacattcataaaatattcactgctttgccagattttatcacctttttttaccatcgctggggatagagattcttggtacgta
accaatgacaacggtatttccaccttcatgttcagcatagcagtccattgcccatgcaggtaatgaactaaaacccataaaaaaagtaataac
aattaacaagtagcgcatattattgatctcggttggctacagaaatattacaaatgttatttactacagggcagtttatttcataaaaacttaatccac
caaagtcattgatataacccacctgaaaagttgacggaacatttttaaactgcgtatttgcatagctaaaaggggttaccattaagctttgcgtttgt
ggtaacatcgttaccccatttgttccgatataacccagcgtaacgtgataaggcatcttattgttaaagcggatcgaaccgtcatttctacgaatga
tttcaactttttcgtaagtcattaccccatcgtcttctaattcaatggcttttggccgccagaatagtttcaatgcgtgctgcatagtgacttgcatcacat
tttttttatctgttttgggcggtatttctcgcacgttaaaatagaagagcgtttctctgtctttaggaagttgagcaatttttttcctgagttatcattcttatttga
atttgctcactgggatttaaacgcaccattggaggaacagtgacaataaagtcacgcgatttattgcctttcgtatcttctatccatgtttgggccag
atagggcaggtttttactttgattagtaagttttaatgcttcgcctttatctgactcattcattatgatacgggtacgatccgggatgatggcaccccag
gcaatgctgctcatacaggagagtaatactcctgctgcaatgatattctttgtatttaagtagcgcattagtttcatcctgaattaattttggctaatac
acggtaaaatagccgttccggtggttaagttgctgctattgggtggttgaataaaacaagatttgtcaccccagcgtaaagcaagtttgttatcatc
ctgaatgccgttgaggtatacaaagccgccatcagcaaccagtcctaattctttgtggctggtttcatcttttacgctaatccccagtggtggatgac
taccatctgccaggcgaataacccctatgatttggtagcccttgcgggtatcaagtttggcgtagccaatagcaccctcggttaaggtggtggtg
ataaccgagttttcgatatccacattttctggcagatttcgggtatcaacacttaatgatgttgttatgtaactgctgactgatggaataactccgatgc
cataccgattagttaccgcgcgtttattgttcaacggaatatcttcagtgccgtctgcgtcgatcataaaacgcgagtcagcactaccgctatagtc
gtggaaagcggcaccgtggcgagtcgcagtgaatgaaccattccaactggcattcagagaataataactgtctcgctgtgatgtaccggaga
ggtagagtcgaccgttctcggtattatgctgataactggcgcgtagtgacgctttcatatctttgaattcatcattgtcgcctgaagcagaaacgctc
caggaattatttcgatcggaagagtcataccacgaagcagtatgcgaaatctcattatcctgatttcgttgcataccatagctcaatgttctactagt
tccccagggaatcgaaatatttaggtacagttgatcctggttgtcttcttcccagtttattcggctaaatgttagagaagtggagacgtttttttaatggc
ccgatatcaaaatcgcggctaagtgataatgaataattcacattactgctggcgtcccagtaagtattacgagccaaacttacgtatgtatttaac
cttaatgattcaagatactggttaaacgtgacaatataactctctttttcattctgataattcgtgtaatggttagtgtcattgatatattcattcattgtcac
aaagtttttatcagagaaacggtaaccagcgaaagtgagctggctaccggtagattcaaaacgttttgaatagttagcgcggtagctataaccc
gtttctttatcctgattatgcaactgagcatcagatcgtgtgacatcaaaagataatgaacctaatgagttaagattaaaaccaacgccggcagc
cagagattgataatcacggtttgttaaaactgaaccaccatacagggatacattgttcagccaaccccatgagaattcaccagtccagaagaa
gggttgttgtaaggaatcaccaccaacgctggtcggtttacccatagcagtggtgtagcgaatctgccctttacgcgtcagataaggaatactatt
agatgcaacttgccattgcgtaacgcgtccgtcctcttcttcaaccttgacgtcaagttgcccctgtaatgtattgaataaattggtaatagcaaatg
ggcctggaggaacattttcttgataaataatacggttgttctgtgagacagtgacctttgcattcgtttgtgcgacacccgtgatttgcggtgcgtaac
cacgtaggtctggcggtaacatattctcatcgcttttcaacgaaatgccagaaaaatggaaagagtcgaaaatagaggattcagtatcgtattg
acctatagttagttttgcattcattgctgggattgggcgaaacaaataaatacgcggaaaatccaggttggtcgcctggcttttgccattgttaaatg
actgatcgtactggtaatcagtacgcaggcgccaggccccaagattaaccccagcctgcccataggaacttatattttgcgaattatcgccctg
atgtggtgcataatgagaggcatataaattgtagtcaagaaatgccccagcaataccatgattccaaagttcaggaggcgtccagtttgcatctt
tgtattttaaccatgcctgaggtgcagatatggataattgcattttccctttatcgagataagttgtaatttgtttttctttttcgatagggtaacaaccatc
gatcaggttttgctgagttttttcaataaattcatcagtaaatcctaataatgttaattgctctggtgtcacgcaaactgcagtttggttatcaattaatac
ccattggaggtcaaggtggattggcgtcctcttattattaatcagagtgcttagtgaatatttgccgacgggtaattggccatcagtttcaaattggg
aaaggttaacgttttcacgatcttcggcttcgattaaatctttgttgaattcaacggcataagctgacgcacaaccaatgatgataactggatttgcc
cctatatttccagacatctgttatcacttaacccattacaagcccgctgccgcagatattcccgtggcgagcgataacccagcgcactatgcgga
tgccattcgttataatgctcgaacgcctctgcaaggttctttgctgccgttaacccgtctggtttgggcatgatactgatgtagtcacgctttatcgttttc

acgaagctctctgctattccgttactctccggactccgcaccgccgtgttcttcggttcaagtcccaacatccgggcgaactggcgtgtttcattag
cccggtagcatgaaccattatccgtcagccactccactggagacgacggaagatcgttgccgaagcggcgttccaccgctcccagcatgac
gtcctgtactgtttcactgttgaagccgccggtagtgaccgcccagtgcagtgcctcacgatcacagcagtccagcgcgaacgtgacacgcag
tctctctccgttatcacagcagaactcgaacccgtcagagcaccatcgctgattgctttctttcacggccactctgcctgtatgtgcccgtttcgatg
gcggtacagcaggttttcgctcaagcaacagcgcattctggcgcatgatccggtaaacacgtttggcattgatcgcaggcataccatcaagttct
gcctgtctgcgaagcagcgcccatacccgacgataaccatacgttggcagctctccgataacatggtgtatacggagaagcacatccgtatc
atcagtgtgacgactgcggcggccatccatccagtcatcggttcgtctgagaatgacgtgcaactgcgcacgcgacacccggagacaacgg
ctgactaagcttactccccatccccgggcaataagggcgcgtgcgctatccactttttttgcccgtccatattcaacggcttctttgaggagttcatttt
ccatcgttttcttgccgagcaggcgctggagttctttaatctgcttcatggcggcagcaagttcagaggcaggaacaacctgttctccggcggcg
acagcagtaagacttccttcctggtattgcttacgccagagaaataactggctggctgctacaccatgttgccgggcaacgagggagaccgtc
atccccggttcaaagctctgctgaacaattgcgatcttttcctgtgtggtacgccgtctgcgtttctccggccctaagacatcaatcatctgttctcca
atgactagtctaaaaactagtattaagactatcacttatttaagtgatattggttgtctggagattcagggggccagtctaataacggataatgaac
aataaatatttttaatcaattcgcttattgtggttaattttaatttttttatacatctgatccacttatcattaatccgttaatgatgtggccgggcgaacccg
gccaaatgcgattactcgtaagcaatcttgtagtcagcctgagcgtaaacgttgcccggggtggcatctttgcttggggattccatacgagctttg
aaattcagttcctgatagctgttggtattgttgaacgtaattactaccggagtacctaaagttacttcgccgctctggctgtccagaatacgtacgcc
aacgccagtggcgccgccgatttcagtttctttggtatttttcagaagatcagactgacccgtcatggtatttgctgtagtgaaagtaaccttcgctttt
gaataagtagttccaccctgactcagcatgcagtcctgcaggtgaatagtgaaaggttcagccaaagatttctgatcgcttttcagtacggtatct
gctacttcacccagatttacagttaaatcttcgtcacctggtttaatagaacatggagcagagataacttcacctttaaatttaacaataccgtgac
cttcatcagcaaacacgttattggaaagaatcattgctacgccagcaacaagaagtgactttttaaaagcggacataatattcctttatataaag
ataagtagaaaagagaatgattagtaactgttatttaaggactactaatcattgaaatatgatattttattttgtgtttttttataaaattagtttatctaaat
atcgattaagtaaaacttaaaaaatacattgcaattatactttcaattgtactcattcatttttgtgatggttttctgaatgggattacgcgataaggtaa
tagaaagcactataaattagaggattaattcataatttacagtgcattaaagacgaatgtttacagcgttgggggggagagtctctggatctggctg
ctttttgatctcagtactgcgattttccagttcgctgattctttgttcaagcagcgccagtttttcacgagtccgtaacaggacttgcgtttggacgtcga
actcttcacggcttacaaggtcgaggcgcgtcagctgcgcttgtagggtttggcggatttttttctccacatcttccccgaactccctgattcctttagg
cattgattcgtgaacctggcgagcgatttgctcaatttttttcgggtcaatcattgtagtttccctgtactgataggtgttgagtgccattgtagtgcgat
aagggtaagtcataaaccagaattatgtgaagctatgcgttgctgccgctaatcattagcgttatagtgaatccgcttattctcagggcggggcg
aaattccccaccggcggtaaatcaactcagttgaaagcccgcgagcgctttgggtgcgaactcaaaggacagcagatccggtgtaattccgg
ggccgacggttagagtccggatgggagagagtaacgattctgtcgggcatggacccgctcacgttattttggctatatgccgccactcctaaga
ctgccctgattctggtaaccataattttagtgaggtttttttaccatgaatcagacgctactttcctcttttggtacgcctttcgaacgtgttgaaaatgca
ctggctgcgctgcgtgaaggacgcggtgtaatggtgcttgatgatgaagaccgtgaaaacgaaggtgatatgatcttcccggcagaaaccat
gactgttgagcagatggcgctgaccattcgccacggtagcggtattgtttgcctgtgcattactgaagatcgccgtaaacaactcgatctgccaat
gatggtagaaaataacaccagcgcctatggcaccggttttaccgtgaccattgaagcagctgaaggtgtgactaccggtgtttctgccgctgac
cgtattacgaccgttcgcgcagcgattgccgatggcgcaaaaccgtcagatctgaatcgtcctggccacgttttcccacttcgcgctcaggcag
gtggtgtactgacgcgtggcggtcatactgaagcaactattgatctgatgacgctggcaggctttaaaccggctggtgtactgtgtgagctgacta
atgacgatggcacgatggcgcgtgcaccagagtgtattgagtttgccaataaacacaatatggcgctcgtgactattgaagacctggtggcata
ccgtcaggcacatgagcgtaaagccagctgaaaaccgctgcttaatttactgccttaatcaagaaaccgaagttgtagcaggcttcggtttttatt
tttccctgctatgcaagatgttaattccgcttctctttctatgagaaaatttcattaatatcaggcattctttttcattatgatcaaaatcatataattgttccg
ttattatgcgaccatatgaatatatccgaaaaaggatatgtgaaatttatacgctaaatgtttagtattgttaaatattaagggtaaaaggatgtttat
cgcctggtactggattgtattgattgctctggttgtggtgggttatttcctgcatttgaaacgttattgtcgggcgtttcgccaggacagagacgcactg
cttgaagcgcggaacaaatacttaaacagtacgagagaagagacagccgaaaaggtagaataaggctttcgtgaagctggcagaagttgt
ccatgcgctggacaacttctgatgttgtttttaaccaattcccgccgtttgtagcagcactaaactgaatcccatcactgacattccacatagtacgc
catagctggggttattattagggtcaatctctttggcgagcggcattaattcatcaaccgagagcgccaccataattcctgcaaccgccgccatg
attgccgccatgaccaccggggaaatcatgctaccgaggattaaccacgccagcacaccaccaagaatttctgctagtccagaaatccccg
cccacagaatcgcggtgcgtttagaccccgttgccgcataaaccgggcctgccactgccagaccttcagggatattgtgcaacgcgacggcc
agtgcgatgccaaatcccagctccaggttgctgctcgccgtgacaaaggtggcaatcccttccgggaagttatgcagactgatgccgagagtg
agcagaatggctgtgcgcttgatcgattttggcaacggctgcaccgatttttgcattaaatcctgcggatgagcatgtggcaacatgcggtccag
gccaaaatagccaagcagaccgaagataaacattccataacccaacacaggcgacattccttcagctgctagtgcggcaggaagcatttcc
attaatgagatgagcaacatgatccccgccgcaaaacctagcgaaaacgccagtaagcggttcgagggtttttgcccgagaacgccgaga
aacgcgccaataaacgtggctgcccccgccagtatggtcagaatgagaggtactgacatcgacaactccttataaatcttcaccttcaggcag
atcgtcaaaataactgatgatcatcatcattgttatccgggttcttactccgccgaacaagcgtattgtgaggatatcaaaatgacacctttagttaa

ggatatcatcatgtcttcaacacgtatgccagcattgtttttaggtcacggtagtccgatgaacgtgctggaagataatttgtatacccgcagctgg
cagaagttggggatgacattgccacgcccgcaagcgattgtggtggtttcggctcactggtttacccgtggaacaggagtgaccgcgatggag
acgccgcccacgattcatgactttggtggcttcccgcaggcgctgtacgatacgcattatcctgctccgggttcgcctgcgctggcacagcgtct
ggttgagctgttagcgccgatcccggtgacgctggataaagaagcctggggctttgaccacggctcgtggggcgtgctgattaagatgtatcct
gacgctgatatcccgatggtgcagttgagtatcgacagtagcaaacctgccgcctggcatttcgaaatggggcgcaaactggcagcgctgcg
agatgaaggaataatgttggtcgccagcggtaacgtggtgcataacctgcgcacagtgaagtggcacggtgatagttcaccgtatccgtgggc
gacgtcgtttaatgagtatgtgaaagcgaatctgacgtggcaagggccagtggaacaacatcctctggtgaattacctcgaccatgaaggtgg
cacgttatcgaacccaacgccagagcattatttgccgttgttgtatgtgttaggtgcgtgggatgggcaggagccaattaccattccggttgaggg
tatagaaatgggcagcctgagtatgctgtcggtgcagataggctaacaattaccggatggcgagtgccatccggtatcgtggcttattcaacaa
aaatatgtggataaaaccgagacatatcctgggtgatcaatgcacggtcttcacgaatgccaattccggcgggttgatcgttcaccagccagct
accaatcagcatatagctgtcgccgaatttcggtaacgggtggaattgctgaacaatcatcccttcttcgccatacggaccttccgctgcttcaat
ggttttgccgttctcaatgatcgacacgtttgcgccttcacgggagaagatcggtttaaccacatatttttccatttgcggatgatcatcttccgcaaa
ataagcgggcagcaggttcgggtgattcgggaacatctcccacagtagcggtagaagtgccttgttggagataatgctcttccacgccggttcc
agccagcgtacgcctgcatcctccagcttggttgagaacatctcacgcaacataaattcccacggatacagtttgaacaggttggaaattacct
gatcctgtaaatccgtgaactgaccttttttcacctaacccgatatcatcgatgtagaggaactcagtagcaatttcagcttccgttgcgcagtcctg
caaatactgaatggttccgcgatcttccaccgtgtcgcgacagcaggtgagatgcagcaactggaagccatactgttcacgcagctcaacga
agcgatcgatcagtttttcttgcagactgttaaactggtcgctgccctccggcaagttaccggcgttaagctgatcttccagccagatccactgaa
agaacgccgcctcgtatagtgacgttggcgtatcggcgttattttccagaagtttaggttcaccagtgccatcccacgccagatcaagacgcga
ataaagcgatggctggtgcgtcagccatgactggcgcacaaaactccaggtgtgttttggaatgcggaatttggtcatcagctcatcgctggcg
atcactttttccaccactttcaggcacatctggtgcagttcggcggtgacttcttccagcttttcaacctgggcgagggtcaacttgtagtaagcatct
tcacaccagtacggctcgccgtacatggtgtgaaaattgaaaccgtattcgtgggctttctcacgccagtccgggcgctcggtaatactgactctt
tccatcggtatcagccacccattgaacgagaagaggtaccggttgcactacgctgcatagtgctttgtttggcaacagattcaccaaaaccgcc
acgggtaacggtagtggtggtcgccggttttggtgccattgccgtcttcggtacggtcatggtgcggcctggctgggctgcgccatagtttttaccc
gtcgcgtcggtatatttaccgtaagccggactggctgggtttttcgaggagaacagcggctgctgtgcaaatcccgcgccgccgcccatcaga
cgccccatcatgtaaccggccatcagcggcatccagaaactcccgctggattgctgggcctgcgcctggttttctggtgccatgccagcctggg
ctggtgcctgctggcactgaccttcaccaaattcagcaacacagtcttcacgggtggcgtatttcggcgcagtacgttcggcttctttcagcgcatt
gttgtacgcggtggtacattcggcgcttttgcctgggtttgcagctgaacagtcgtcagcattttgatagagagacactgtttcatcactcttttcaca
gccagccagcataaaaacagtggcaaccgcgagagcgactggtgtcagatggcgtgcgctccagtttttgcggaacgatgcgtggcgtatg
gattttgtccgtttcatttttgtcttccgggaccagtggtaaatacccatcagaatagaggatggctggtcgaaattgaagcgagaagggggaag
aatgcggcagataacccgtatctttacgttgccttacgttcagacggggccgaagccccgtcgtcgtcatcagttacggaaagggttatgaccgt
tactggtggtagtgcgtgcggatgtttgctgaacgactggtgccgggctatcaggcgcataaccatcagcaatagcattctgttccggcgtttgcg
gtgcaacgttttccggattagtggaaaccggtttgctcagcgcattgttcagtgccagcagatcctgctcgttcaacgtacccagagctgacttaat
attcagctgattaatcaggtagttataacgcgcattcgccagctcttgcttggcgttgtacaacgtggtggtcgcatccaacacatcaacaatggt
acgcgtaccgaccgagtagcccgcttccatcgcgtctaatgagctttgagcggaaactacggcttgtttgtaggcgttaatgctactgatagatgc
attaatgttgttgaaggaggaacgcacggtctgcacgacgctacgatgggcactttccagttgctcgctggcaccgacaaagttgtactgtgcct
gtttcacctgcgagttaaccattccgccctgataaatcggcagcgagaagctcaggccaactttgttctggcccatattgctatcgtcatactgggt
accagcggcaccacgggttttcgaaccgctataagaggtgtcagaaatcccggtagaagccgttaaatccagagtcggtaagtgaccatcct
gcgcctggcgaatttgctcgcgcgccaggtcctggctcaagcgtgcctgtaacagcgacaggttgcgtttttcggcttctttcagcagcgcgttaa
ccggctgtggtttgtcggttttaaagttttcgacattcagcgcagccagttccggatagtagttaccggtgatctggcgcagctgctctaccgcgttat
caaggttattacgtgcggtcacttcgttcgccagcacggtatcgtactgtgcgcgggcgttctgcacgtcggtgatcgctaccaggcccacgtta
aaacgttgggtggtttgatctaattgacggtagatcgcttctttttgtgcctgtgtataggaaagaacgtcaatagcattcaacacgttgaaataagc
ggtcgcggtgttgaggatcaaggtttgctgatcggtctgatacgtgacgtcctgaatccctgctgctttttcctgcagcgttaacgcacgccatttcg
acatatcaaaaatggattgagttaactgcaaggacgcactggtcgcgttagagttgatgccgttcgcgtcgcggtagccgttgctataggtgtaa
tctgcacctaaacctagctgtggcagtaatggactgcgcgcttcattaatttttcaaaggcagcatcacgatcggcggcagacttacgcaattcc
gggttactaaggcgtgcttgctgataaacttgcatcaggttctcggcctggctcaacgaactgaacccagaaaggctcaggccgataagaatg
gggagcaatttcttcatttgcattccttgtggtgaagcagtatttagcgcgatcaaactgtaaaattattgcggattctagcagaagccgctaccgc
aaaaggttggcgttacgtgccatagggcgttaatgtgcctcaatttaacacatgactgctcaaacggcagtcaaacgtcgctgaaattcacattt
aattcactattagtgccaggacattaacaatgcttaagccagacaacctgcccgttacatttggcaaaaacgatgtagaaattattgcacgaga
aacactttatcgcggctttttttcattagatctttatagatttcgtcatcgtctattcaacgggcaaatgagtcatgaggtgcggcgggaaatttttgag
cgcggtcacgccgcagtcttgctaccctttgacccagtgcgtgatgaagttgtgctgattgagcagattcggattgccgcgtacgacaccagcg

aaaccccttggctactggagatggttgccgggatgattgaagagggtgaaagtgtggaagatgttgcccgtcgcgaagcgattgaagaggcg
ggactgatagtcaaacggaccaaaccggtgttaagtttcctggcaagcccgggggcaccagtgagcgttcgtcaattatggtgggcgaagt
ggacgccacgaccgcaagcggtattcacggtctggctgatgaaaacgaagatattcgcgttcatgtggtaagccgggaacaggcataccag
tgggtagaagaggggaaaatcgacaacgcagcgtcggtcatcgctttgcaatggctgcagctgcatcatcaagcgttaaaaaatgagtggg
cataaatgaagcgttacacacctgactttcctgaaatgatgcgcctgtgcgagatgaacttttcacaattgcgccgtttgttaccgcgcaatgacg
cacccggcgaaactgtaagctatcaggtggcaaacgcacaatatcggctgacgattgtggaatcgacccgatacactaccctggtgacaata
gaacagactgcgcccgcgatcagttactggagccttccgtcaatgacggtgcgtctgtatcatgacgcgatggtggctgaagtgtgttcaagcc
agcagatttttcgcttcaaagcgcggtatgattatcctaataaaaagttgcatcaacgcgacgaaaagcatcaaattaatcagtttttagcggact
ggttgcgatactgtttagcacatggagcgatggcgattccggtttattagcgtcgtgaaacctaaggacaccatttggaaagcctgttaacccttc
ctctggctggtgaggccagagtcaggattttacaaattaccgacactcacctgtttgcacaaaagcacgaagccctgttaggggtaaacacctg
ggagagttaccaggcggtgctggaggcgattcggccacaccagcacgaattcgacctgattgtcgcgacaggtgatttagcgcaggatcaat
cctctgcggcctatcagcatttcgctgaaggcatcgcaagttttcgtgcgccctgcgtctggctgccgggcaaccacgatttccagcccgcgatg
tacagcgcgttacaggatgcgggtatctccccggcgaagcgcgtgtttattggtgagcaatggcaaatcctgttgctggatagccaggtgtttgg
cgtgccgcacggtgagctgagcgagtttcagcttgagtggctggaacgtaaactggccgatgcgccagaacgccatacgttgctgctgctgca
tcatcatccgctacctgcgggttgtagttggctcgatcaacacagtctgcgtaacgcgggcgaactggataccgtgctggcgaagtttccgcac
gtcaaatacttgctgtgcggtcatattcatcaggagctggatctcgactggaatggtcgccgcctgctggcaacgccgtcgacctgtgtgcagttt
aagccgcactgttccaactttacgctggataccatcgcgcccggctggcgtactctcgagttacatgctgatggcacgctgaccaccgaggtgc
atcgcctggcggacacacgtttccaacctgataccgcttcagaaggctactgatgtctacgcttctttatttacacggtttcaacagctcgccgcgc
tctgcaaaagcgagcttgttaaaaaactggctggcggaacatcaccctgacgttgagatgatcattccgcagttgccgccgtatccttccgacg
cggcagagctgctggaatccattgtcctggaacatggcggtgattcgctgggtattgtcggttcgtcactgggggatattacgccacctggttgt
cacaatgttttatgctgcccgcagtggtggtaaacccggcggtgcgcccgtttgaactgctgacggactatctcggtcagaacgagaacccctа
caccgggcagcaatatgtgctagagtcacgccatatttacgatcttaaagtcatgcagattgacccgctggaagcgccggatttgatctggctgc
tgcaacagacgggagatgaagtgctggattaccgccaggcggtggcgtactacgcttcctgccgccagactgtcatagaaggcggcaacca
cgcattcacgggcttcgaagattatttcaacccgatcgtcgattttcttggtctgcaccatctctgacgatcaactcgaattactaacttaaaccatg
acgcaaacttataacgctgatgccattgaggtactcaccgggcttgagccggttcgccgccgtccggggatgtataccgataccactcgcccta
accatttggggcaagaagtcattgataacagtgtggatgaagcactggcgggtcacgcaaaacgcgtggacgttattttacatgctgaccagtc
gttagaagttattgacgatgggcgcgggatgccggtggatattcacccggaagagggtgtaccggcggttgaactgattctttgccgtctgcatg
caggcggtaaattctctaacaaaaattaccagttctctggcggcctgcatggcgtggggatttcggtggttaacgccctgtcgaagcgcgtaga
agttaacgtgcgccgcgatggtcaggtttataacatcgcctttgaaaatggcgaaaaggtgcaggatttacaggttgtcggcacttgcggtaaa
cgcaatactggtaccagtgtgcacttctggccggatgaaaccttctttgacagcccgcgattttctgtttcacgcctgacgcatgtgctgaaagcc
aaagcggtattgtgccctggcgttgagatcacttttaaagatgagatcaacaataccgaacaacgctggtgctatcaggacggtctgaatgatt
acctggcggaagcggtaaatggtctgccgacgctgccggaaaaaccgtttatcggtaatttcgctggtgatactgaagctgtggactgggcgct
actgtggctgccggaaggcggtgaactgctgaccgaaagctacgtcaaccttatcccaacgatgcagggcggtacccatgttaatggtctgcg
tcagggcctgttggacgcgatgcgtgagttctgtgaataccgcaatattctgccgcgcggtgtaaagctgtcggcggaagatatctgggatcgct
gcgcctatgtgctgtcagtaaaaatgcaggatccgcagtttgccgggcagacgaaagagcgtctctcttcgcgtcaatgcgcggcattcgtttct
ggcgtggtgaaagatgcctttatcctgtggctgaaccagaacgttcaggcggctgaactgctggcggagatggcgatttccagcgcccagcgc
cgtatgcgtgcggccaaaaaagtggtgcgtaaaaagctgaccagcggcccggcgttgcctggcaaactggctgattgtaccgcgcaggacc
ttaaccgtaccgagctgttccttgtggaaggtgactccgcaggcggatctgccaagcaggcgcgcgatcgcgaatatcaggcgatcatgcca
ctgaaaggtaagatccttaacacctgggaagtctcttccgacgaagtgctggcttcgcaggaagtgcacgatatttcggtagcgatcggtatcg
atcctgacagcgacgatctgagccagcttcgttatggcaaaatctgtatcctcgcggatgcggactctgatggtctgcacattgccacgctgctct
gcgctttgttcgtaaaacatttccgcgcgttggtgaaacacggtcacgtttacgtcgcactgccaccgctctaccgtattgatctcgggaaagagg
tttattacgcgctgacggaagaagagaaagagggcgtacttgagcaattaaaacgcaagaaaggcaagccgaacgtccagcgttttaaag
gtctgggggaaatgaacccgatgcaattgcgcgaaaccacgcttgatccgaacactcgccgtctggtgcagttgactatcgatgatgaagac
gatcagcgtactgacgcgatgatggatatgctgctggcgaagaaacgctcggaagatcgccgcaactggttgcaagagaaaggcgacatg
gcggagattgaggtttaaaggaaagaacattgcccggtcaggatggccgggcaaacaaggcaggattaaatccctggctgcagaatacgg
atattcatctccagcacgtcaccttttacggcttcgctatacgccttcatgtgcggggtttgcagatgcgcttcaagatgcgcgatgctttcccactgc
tcaatcatcacgatagaatccggtgccatagactggaaactcacgccagctgcgcaatccaccattggcgcatagccgtggcaaccttcttcttt
cagtacggttggaacgattttagcaaactgatccaataccgcctgacggtgatgttgaccaggacgagtacggatttctgcgattacggtaagc
atggttaactccttctaaagcctgagctctagttaaccaaaaatttccacaagatgcttgcgatattcttcagtatagcggggaacatcaggcatttt
tatcacgtcattagcgataaatgtcggcagcggttccataccgaggaattggtttgctttatggaacggcagatacacaccgtcaacgccaacg

ccgtggaagaactgatctttttcggtgaaggcttccattggtgcgttccaggtcagagaaagcatatattttttgccctgtaccaggccgccggaa
ccgtatttttttcgacggatctttgcgggtacgaccatcgctggcatacagcgtcccgtgaccttcggtgaatacatcatcaatgtattttttcactgtcc
acggcgcgcccatccaccagcctggcatctgccagatcaccacatcagcccagagaaagttttgtacttccgctttgacatcgtagtcgctgtc
ggcgcgaacgatgcggacatcatgcccaaggtcgcgcagtgtgccatccgcgacttcggtcagggtgtcgttcagttgaccattggagtgggc
gaattttttcgcgccgttgataatcaggatgttgctcatttttttaacctcaaggtgagacgtttgccgactatagtacgtcacagagcggtacggtga
aattagcaaaatgtgcaaagtcttttgcgaatttagcaaaagtctgatcttactctgactcaccgatgcaggataaatattttctgaaaatgacaat
actgatagccattcctcataattcttactcgcaccggaataatttacacgtttggttcggttttttttggggattgaaaatatggctttgaataagtaaaa
gatgaaagtatcaacgtgtcatgttgataattataaatggatttttatgagcgtactgatatatgttaaagcagaaaattaaaacaatatttgaagc
attgctatatataatgcttacttattggttaatagattcttttttttgctttcaataagtatgactggatgcttgagtcgggaggaaatatttgcagtatacctt
ccgtttcaggcgaagatcgtatattgcaagcaatgattgcagcattttttcttcttacgccacttattattcttatcttaagaaaactctttatgcgggaa
atgttcgagttttgggtatatgtattctcgctcggtatttgtctggtttgtggttggtggctttttttggggacggtttatattttgctattagtaaaatctatgag
agtcgttttaacggctctcatagacagagaagttaccagcttaccttcgcctcaaatccaccttgttccgcattcccaaattcaacattcatgccatg
caatttggcgattcgctggacaatcgatagcccaagcccgctgccggtagcggtttgtccgggtgggcgatagaagcgttcgccaattcgcgc
cagtgcctctggtgtcacaccggggccgttatccctcacgatgaaattatcagcattcagcgtgacgtctaccacgctgccctgtggactgtagc
gcacggcgttatccagcaaatttcgcaccaacaaacttagcaatagcggttgcccggtgcgtttgatgctgtgggcattgagtgtcagtcgcacg
tcaattttcgcctgctgcgccgtgtggtaaatatccatcaccgacgattgcaggagatcttcaagcgggatctccgcgacgtcctgaaggttatcc
agtgagtccagccgcgatagcgtgagcagttgatcaaccagacgagtagcgcgatcgatcccggaatgtaattggagcagtgctttttttccgc
gcctgcggatcatcgtcagagagctgcgcaacttcggtttgcactttcagcgccgttaacgggctacgaagttcgtgagctgcgtcggaggtaa
agcgtcgttcacgaaccatcatcgcatgtgtgcgggcgaacagttgatttagcgactcaaccagtggccgcacttcgctgggtacgccagtcg
cgtttagtggtttttccgagtcagggtcacgcatacgtagtgccagcgccagtttgttcagcggcgcgagttcacgacccagtagtaccatcatga
tgattaacataatcggcagtgcgaccagccacgggatcaattgcccggcaacaatcgccagcgccatgtcttcacggtattcccattcctggcc
aacaacgatgcgatatttgccatcaggtgaggtcatccagacaaaacgccaaggatcgtcttcaccgaccagttgcccgtcagcaaaacctt
cccgttgatagctatagggaatatcttctccgttatcgccatcattaaggaccattctgccgtcgtgggtaaagatggcaaaggtcagcgcatcgt
catcaacatgaccgtgttttaatctatttggcgtctgtgccatgcgatccgccgcgttgatttcgttgagatcgagcgtacttaaccgcttggcaaac
agcatcagttgggtgtcgaacaattcatcgacgttatccgttgtttgtttccaggcgacaaagctggaaagcagccaggtcaccgaggccaga
attaaaaagattagcgtcagcctgacgcgcagactaagacgttgggtaaatttcatttctcacctaatgtgtaaccaataccatgcacggtacga
ataaaatcactaccgagtttgcgtcgcagatgatgcacatgcacttcaacggcattactggtgacctcttcgtcccaggtatacagtttctcttcaat
cagtttgcgcgacagtacccgaccagcgttacgcatcagtaattccagcagggcaaattcttttggtttcagtgttaagggttcgccagccagcgt
ggcgatacgtttgccggggtcgagcatgacgttaccgtggcgcagctcgttgctggcctggccgttggttcggcgcatcagagcttccagcctg
gcggcgacttctatcaacgcaaaaggtttacacagataatcgtcagctcccagacgcagcccttctacacgttccgccagcgcatcgcgcgc
ggtcaggatcagtaccggctcacgctgacctttttctcgccattcgcgcaaaatatcgcgaccatccatgcctggtaaggttaaatccaggatca
ccgcatcataaggtgcgctataaagcgcctcttttccctgacgaccttgtgtaaaccagtcgacgctaaaacccattttactaaggcccgttttgat
gccgtcgccaatcagcatgtcatcttctatcagtaaaattcgcatttttttcatccctgcgataaccgtatttgctgccagtaaaccgtgttgtaacgcg
ctctgtacagcactaaattttattttttttcccttaagaagttgttaaggactatcttgttaaatgctcggaaacagacattaaagggagtaataaacat
gaaaaaattcgcagcagtaatcgcagtaatggccctgtgcagcgcaccggtgatggcagcagagcagggcggtttttctggcccatcggca
acgcaaagtcaggccggaggattccaggggccgaacggcagcgtaacgactgtagaaagcgcaaaatccctgcgtgacgacacctgggt
aaccctgcgcggcaatatcgttgaacgcatctctgacgatctctacgtgttcaaagatgccagcggtactatcaatgttgatatcgaccacaaac
gctggaacggcgtgacggtgacgccgaaagatacggttgagattcagggtgaagtcgataaagactggaattctgttgaaattgacgtcaaa
cagatccgcaaagtaaatccgtaattgttaccgctcccgggacgcgttcccgggaataatttcgcagggaggcaaaatgacaaacctgacac
tggatgtaaacattatcgatttcccatcaatacctgtggcgatgttgccgcaccgctgtagccctgaattgctcaactacagcgtggcgaaatttat
catgtggcgtaaagagacggggctttctcctgttaaccaaagccagacttttggcgtcgcctgggacgaccctgccaccaccgcaccggaag
cgtttcgctttgatatctgcggcagcgttagcgaaccgattcccgataatcgttatggtgtgagcaatggtgaacttaccggtggacgttatgccgt
ggcccgccacgttggcgagctggacgatatttcacacacggtatggggcatcattcgccactggctgcctgcaagcggcgagaaaatgcgta
aagcaccgattctgtttcactacaccaatcttgccgaaggggtgacagagcagcgactggaaacggatgtttatgtgccgttggcgtgatgcct
gactccagcttcccttataaattcctgcttcaaactggcgctactaatgcgccactttcttatatggtcaatcactttccttaaagaatacgttttgtgtg
gtcactatctccgtacatctaactaaccttttaggtgctttttcattgctgtaattaaccttttaggttataactaaagtaacagggaggcgggggttat
ggaaaaacgcacaccacatacacgtttgagtcaggttaaaaaacttgtcaatgccgggcaagttcgtacaacacgtagtgccctgttaaatgc
agatgagttaggtttggattttgatggtatgtgtaatgttatcattggattatcagagagcgacttttataaaagcatgaccacctactctgatcatact
atctggcaggatgtttacagacccaggcttgttacaggccaggtttatcttaaaattacggtaattcatgacgtactgatcgtctcgtttaaggaga
agtaatatgaaatgtccggtttgccaccagggagaaatggtttctggcattaaagatattccatacaccttccgtggacgaaaaacagtattgaa

aggtatccacggtttatattgtgtccattgcgaagagagcatcatgaataaagaagagtcagatgctttcatggcgcaagtaaaggcatttcggg
cttcggtgaatgccgaaacagtggcacctgaatttatagtgaaggttcgaaaaaagctctctcttacccaaaaagaggcaagcgaaatttttgg
gggaggtgtaaatgcgttttcgcgttacgaaaaaggcaatgcccaacctcatccttccacaatcaaacttttacgtgttctggataagcatccag
aactattgaatgaaatccgttaattaatggaaaagcgggggcaactcgcttttttctttaccggagtgtgagccaaatcactcgcgataagtttga
gcgaacaatgccacaggcgtattgttgttgggcttataatttctccggtaaggaataccatgtatacgcgaaatttattatggctggtcagcctggt
aagtgcggctcctctctacgctgctgacgttcccgccaacacaccgctcgccccgcaacaagtctttcgttacaacaatcatagcgacccaggt
acgctcgacccgcaaaaggtggaggagaatactgccgcgcagattgtgctggatctgtttgaaggtctggtatggatggacggtgaaggcca
ggtgcagcccgctcaggctgaacgctgggagatactggacggcggcaagcgctatattttccatctgcgtagcggtttgcagtggtccgacggt
cagcctctgacggcagaggattttgtcctcggctggcagcgcgcggttgacccgaaaacggcaagcccttttgctggctatctggcacaggcg
cacattaacaatgccgcagctattgttgcgggtaaagcagatgttacatcgctgggtgtcaaagcgacggatgatcgtactcttgaagttacgctt
gagcagccggttccttggttcacgacgatgctcgcctggccgacgctgttcccggttcctcatcatgtcatcgctaaacatggcgatagctggagt
aagccagagaacatggtttacaacggtgcctttgtgcttgatcagtgggtagttaacgaaaagattactgcacgcaaaaatccaaagtaccgc
gatgcgcaacatacagtattgcaacaggttgagtatctggcgctagataattcggtcaccggctataaccgctatcgcgcgggagaggtcgat
ctcacctgggttccggcgcagcaaattcccgccattgaaaaatcactgcctggcgagctacgaattattccgcgtctgaacagcgaatattaca
acttcaaccttgagaaaccgccatttaacgatgtgcgagtgcgtcgggcgctatatcttacggttgatcgacagcttattgcgcaaaaggtactg
gggttgagaacgcccgcaaccacgctgacgccgccagaggtaaaaggctttagcgcgacgacgttcgatgaactgcaaaagccaatgagt
gagcgcgtcgcgatggcaaaagccttgctgaaacaggcgggatacgacgcctctcatccgctacgctttgagctgttctacaacaagtacgat
ctgcatgaaaagaccgcgatagcgttgtcttccgaatggaaaaaatggctgggtgcacaggtgacgctgcgcacaatggagtggaaaacct
atcttgatgcccgacgagccggtgatttcatgctgtctcggcagtcgtgggatgcgacgtacaatgatgcttccagcttcctgaacacgctcaaa
agcgatagtgaagaaaacgtcggtcactggaaaaatgcgcagtatgacgccttactaaaccaggccacgcagatcactgatgcgacaaag
cgtaatgcgttgtatcagcaggcagaagtgatcatcaaccaacaggcaccgctgattcctatctactatcagccgttaatcaaactgcttaaacc
ctacgttggcggttttccgctgcataatccccaggattatgtctacagcaaagagttgtatatcaaggcacattgatgcctttatcgccgtcggagc
acgtccggcggcgctattcacgttgatctcctgtgactcgacgcggcagataatgtagtatctccggcaatattgccccttgaaggctggcgaat
aagttgaggaatcagaattaatgagcgatatggcagagcgccttgcgctacatgaatttacggaaaacgcctacttaaactactccatgtacgt
gatcatggaccgtgcgttgccgtttattggtgatggtctgaaacctgttcagcgccgcattgtgtatgcgatgtctgaactgggcctgaatgccagc
gccaaatttaaaaaatcggcccgtaccgtcggtgacgtactgggtaaataccatccgcacggcgatagcgcctgttatgaagcgatggtcctg
atggcgcaaccgttctcttaccgttatccgctggttgatggtcaggggaactggggcgcgccggacgatccgaaatcgttcgcggcaatgcgtt
acaccgaatcccggttgtcgaaatattccgagctgctattgagcgagctggggcaggggacggctgactgggtgccaaacttcgacggcactt
tgcaggagccgaaaatgctacctgcccgtctgccaaacattttgcttaacggcaccaccggtattgccgtcggcatggcgaccgatattccacc
gcataacctgcgtgaagtggctcaggcggcaatcgcattaatcgaccagccgaaaaccacgctcgatcagctgctggatatcgtgcagggg
ccggattatccgactgaagcggaaattatcacttcgcgcgccgagatccgtaaaatctacgagaacggacgtggttcagtgcgtatgcgcgcg
gtgtggaagaaagaagatggcgcggtggttatcagcgcattgccgcatcaggtttcaggtgcgcgcgtactggagcaaattgctgcgcaaat
gcgcaacaaaaagctgccgatggttgacgatctgcgcgatgaatctgaccacgagaacccgacccgcctggtgattgtgccgcgttccaacc
gcgtggatatggatcaggtgatgaaccacctcttcgctaccaccgatctggaaaagagctatcgtattaaccttaatatgatcggtctggatggtc
gtccggcggtgaaaaacctgctggaaatcctctccgaatggctggtgttccgccgcgataccgtgcgccgccgactgaactatcgtctggaga
aagtcctcaagcgcctgcatatcctcgaaggtttgctggtggcgtttctcaatatcgacgaagtgattgagatcattcgtaatgaagatgaaccga
aaccggcgctgatgtcgcggtttggccttacggaaacccaggcggaagcgatcctcgaactgaaactgcgtcatcttgccaaactggaagag
atgaagattcgcggtgagcagagtgaactggaaaaagagcgcgaccagttgcagggcattttggcttccgagcgtaaaatgaataacctgct
gaagaaagaactgcaggcagacgcgcaagcctacggtgacgatcgtcgttcgccgttgcaggaacgcgaagaagcgaaagcgatgagc
gagcacgacatgctgccgtctgaacctgtcaccattgtgctgtcgcagatgggctgggtacgcagcgctaaaggccatgatatcgacgcgcc
gggcctgaattataaagcgggtgatagcttcaaagcggcggtgaaaggtaagagcaaccaaccggtagtgtttgttgattccaccggtcgtag
ctatgccattgacccgattacgctgccgtcggcgcgtggtcagggcgagccgctcaccggcaaattaacgttgccgcctggggcgaccgttga
ccatatgctgatggaaagcgacgatcagaaactgctgatggcttccgatgcgggttacggtttcgtctgcacctttaacgatctggtggcgcgta
accgtgcaggtaaggctttgatcaccttaccggaaaatgcccatgttatgccgccggtggtgattgaagatgcttccgatatgctgctggcaatc
actcaggcaggccgtatgttgatgttcccggtaagtgatctgccgcagctgtcgaagggcaaaggcaacaagattatcaacattccatcggca
gaagccgcgcgtggagaagatggtctggcgcaattgtacgttctgccgccgcaaagcacgctgaccattcatgttgggaaacgcaaaattaa
actgcgcccggaagagttacagaaagtcactggcgaacgtggacgccgcggtacgttgatgcgcggtttgcagcgtatcgatcgtgttgagat
cgactctcctcgccgtgccagcagcggtgatagcgaagagtaatatgccggggagggcgctgtttcctcccgcttgcaaggaacgccggatg
aaacgttaagtcgtcatccggcagcgtattcaaccccccggaaacgataaaaattcccttgaaaccgttgtttattcatgcgttgcgattaacaata
cgcttttccagagagcggcttttaacaatgccctaacctgatttcaggtgacgtacaatgccagtctctcagaccttcagagggtgttatgctatata

tctttcgtcttattattaccgtgatttacagcatcttagtctgtgtattcggctccatttactgccttttcagcccgcgtaacccgaaacatgtggccacctt
tgggcatatgtttggccgtcttgcgccgctgtttggcctgaaagttgagtgccgtaaacctacagacgctgaaagctacggcaatgctatctatat
cgctaaccaccagaacaactatgacatggtgacagcatcgaacatcgtgcaaccgccgacggtgacggtaggtaaaaagagcttgctgtg
gatccccttcttcgggcagttgtactggttaaccggcaacttattgatcgacagaaacaatcgcactaaagctcacggcaccattgcggaagta
gtgaatcacttcaaaaaacgccgtatttccatctggatgttcccggaaggaacccgcagccgtggtcgcggcctgctaccgttcaagactgga
gcatttcacgcggcaattgcggcgggcgtcccgattattcccgtgtgcgtctctacaacttcgaataagattaatcttaatcgactgcacaacggt
ctggtgattgtcgaaatgctgccgccaattgacgtcagtcagtatggcaaagatcaggttcgtgagctggctgcccattgtcgttcgataatggaa
caaaaaatcgccgagctcgataaagaagtcgcagaacgcgaagccgccggaaaagtttaagtcggcaatctgtatttttgcggggaacactt
tcctgcacggtattactttagccagttttacatggagcaaatatgtcactcagtcggcgtcagttcattcaggcatcggggattgcactttgtgcagg
cgctgttcccctgaaggccagcgcagccgggcaacagcaaccgctacccgttccgccgctacttgaatctcgccgtgggcaaccgctgtttat
gactgtacaacgtgcgcactggtcatttacgccagggacacgcgcgtcggtctggggaatcaatggtcgttacctggggccgactatccgcgt
ctggaagggcgacgatgttaagcttatttacagcaaccgcctgacagaaaatgtctcaatgacggtggccgggctacaggtaccaggcccgc
tgatgggcggtccggcacggatgatgtcgccaaacgctgactgggcacccgtactgcccattcgccagaacgcagctactctgtggtatcacg
ccaatactcccaaccgcacggctcagcaggtctataacggccttgccggaatgtggctggtggaagatgaagtcagcaagtcgctgcctatc
cccaaccattatggtgtggatgattttccggtcattatccaggataaacggctggataactttggtacgccagaatacaacgaaccgggaagcg
gcggctttgttggtgatacgctgctggttaacggtgtacaaagcccgtacgttgaagtctcgcgtggctgggtgcgcttgcgactgctgaacgcgt
cgaactctcgtcgctatcaactacagatgaacgatggtcgcccgttacatgtgatttctggcgatcagggattcctgcctgctcctgtatcggtgaa
gcaactttcgctggcaccgggcgagcgccgcgagattctggtggatatgagcaacggcgatgaagtgtcgatcacctgtggcgaagcggcg
agcattgttgatcgtattcgtggcttctttgagccatccagtattctggtttctaccctggtgctaacgctgcgcccaaccggccttctgccgctggtca
cagacagtcttccgatgcgcttgctgccaactgaaatcatggctggttcgccaattcgcagtcgcgatatcagtctgggtgatgacccgggtatta
atggacagctgtgggacgtcaaccgtattgatgtcaccgcgcagcaaggaacgtgggaacgctggacggtacgcgcggacgagccgcaa
gcgttccatattgaaggcgtaatgttccagatccgtaacgtgaatggcgcgatgccgttcccggaagacagaggctggaaagataccgtttgg
gttgacggacaagtggagctgcttgtttatttcggtcagccttcctgggcgcacttcccgttctacttcaacagtcagacgctggaaatggcggac
cgtggctcgattgggcaactgttggtcaatccggtaccgtaatcttcatactcgcccgaggagggcgagtatggaatacctttcgatttctctttcgtt
ccggcttataatccccggcctttgattattttttatcatttctggaagcaaaatgagctctatctccctgatccaaccggatcgcgacctgttctcctgg
ccgcagtactgggccgcctgttttggaccggcaccgtttttgccgatgtctcgtgaagagatggatcaacttggctgggatagctgcgacatcattt
tggttactggcgacgcgtatgtcgatcacccaagcttcgggatggcgatttgcggtcgtatgctggaagcgcagggctttcgcgtcgggatcatc
gcccagccggactggagcagcaaagacgactttatgcgtctgggtaaaccgaatctgtttttcggtgttactgctggcaacatggattcgatgat
caaccgttataccgccgatcgccgtttacgtcatgacgatgcctacacgccggataacgtcgcgggtaagcgcccggatcgcgccacactgg
tttatacccagcgttgtaaagaggcgtggaaagatgtaccggtgatcctcggcggtattgaggctagtctgcgccgtaccgcgcattatgattact
ggtccgataccgtgcgccgttccgtgctggtggattcgaaagccgacatgctgatgtttggtaacggtgagcgtccgctggtggaagtggcgca
tcgtctggcgatgggcgagccaattagtgaaatccgcgatgtgcgtaataccgcgattatcgtgaaagaggcgctgcctggctggagcggcgt
ggattccacccgtcttgatacccctggaaaaatcgacccaatcccgcatccgtatggtgaagatttgccgtgcgcggataacaaaccggtggc
accgaaaaagcaggaagccaaagccgtaaccgtgcagccaccgcgcccgaaaccgtgggaaaaaacctacgtgttgctgccttctttcga
gaaagtgaagggcgataaagtgctgtacgcccatgcttcgcgtattctgcaccacgaaaccaacccaggctgtgcccgcgcattgatgcaaa
aacacggcgaccgctatgtgtggatcaacccgcctgctattccgctttctaccgaagagatggacagcgtttttgcgctgccatacaagcgcgt
gccacatccggcctatggcaatgcccgtattccggcttacgaaatgatccgtttttcggtcaacattatgcgtggctgctttggcggctgctctttctg
ttctatcaccgagcacgaagggcgcattattcagagccgttccgaagattcgatcattaatgagatcgaagcgatccgcgacaccgttccaggt
tttacgggcgtgatttccgatcttggtgggccaactgccaacatgtatatgttgcgctgcaaatcgccacgcgctgaacaaacttgtcgccgtttgt
cgtgcgtttatccggatatttgtccgcacatggacacgaaccacgaaccaacgatcaacctctatcgccgtgcgcgtgatctgaaaggcattaa
aaagatcctgattgcctctggtgtgcgttatgacatagccgtagaagatccgcgctatatcaaagaactggcgacccatcacgtcggcggttatc
tgaagattgccccggaacataccgaagaagggccgttatcgaagatgatgaagccgggcatgggcagctatgaccgctttaaagagctgttc
gatacttactcaaaacaggcaggtaaagagcagtatctgatcccctatttcatctccgcgcaccccggtacgcgtgatgaagatatggtgaatct
ggcgctgtggctgaaaaagcaccgcttccgcctcgaccaggtgcagaacttctatccgtcgccgctggcgaactcaaccaccatgtattacac
cgggaaaaacccgctggcgaagattggttataagagcgaagacgtcttcgtaccgaagggcgacaaacagcgtcgtttgcataaagcgttgt
tgcgttaccacgatccggcaaactggccgttaatccgccaggcgctggaagcgatgggcaaaaagcatctgattggcagccgtcgcgattgc
ttagtgcctgcgccaaccattgaagagatgcgtgaagctcgtcgccagaaccgcaatacccgtccggcgttgacgaaacatacgccgatgg
cgacccagcgtcagacgcctgctacggcaaaaaaagcgtcgtctacgcaatctcgtccggtgaatgctggtgcgaagaaacggcctaaag
cggcggttggacgttaaaaaatatccccggcaactgacacgctaccggggatttttttatcattctgcgtacatacgcaagcagcgcaggcaat
caaaatagtcctgagcatcaagacgcgtattggctctgttagcttcggatttggcagcatagatttgtgggcgtagtgctttcatatcctgctcaagc

cgggcgattttggcatttaatgtttgcacattgctcgccagctgattgaccttttgctcattgacgcagccactgagcaagcaggttgccagaacaa
cagctcccacggtgaaaatcgttttcatatagtgttcccttttatttattgttccggctaattgttatttaattcagctcattgctttaatctggcggcagttttt
gcctcaatctgcttaagtaggctgccgctgttcgggcggataaaatcagcgatttcccacttgccttcgcgccgctgtaaaaccagttcttgctcttc
gttgttgccattggtcagcgtaaaacgaacatgaatgcgggcggaatcggcggttttctccaggactcgaatttcatcaacgcgtgaaaatagc
gactcttcgccaaaacagatcggattgtcgtaaataaattgccgttctatttgcatatcttcatcgggattattggccgtcttgaaggtggttttataga
aggcattaaccgcctggcggtaagagtcggaaaccacccacgtccacggccagctataatcagcaatatgttcaaagagttcggcaacaaa
ggcttccggctgttctttttgcaacgaagctaaagcggccagcgtttttcattctcgctattaactgcttgtaagacgctgccatgattgctgacaata
tcgtcaatgacccaacgaccattttccgccaccattttcagtgtctgcgtggtcttttctttatcatctttaaagatacgaaagcgcacaacggcatc
ggcatgatcggcgtcagtttgggttatcgcaacgctttctagcaccagatcgccaaaatcctgacaatcacaaaccggatcataatccagcca
gccaatattgcccggcagcgtaagattgtcgttcagggtaagcgcctgttgaatacgcgcagaagttatcgcccgctctccggtttcaccaaaat
aaggggcagtggcatctgatttatagttctgataaatatgccggactgtttgttctacggtctgtgagggggctgtgcgtgtacggtaaaacttgc
cagggctgctaaaaacagaagtattattttcatctgtttttcacctctccgttgcgtttatcccggatatagatgagttgttcttttaagagcttccggctc
tgcatgatgatgtccttatatttggcattcctgaacaaggaagagtaacaacgggcgggacgcgaggggaataaatgatttctgaaaagtccg
gtagcggaacattaccgccaccgggagaatttgcatgttagccgccgaactggtcaggatcgggaccgagacgcttgccctgatcgagttttg
caatttcgccgagttcgtctttgtcgagacggaaatcccagacatcaaagttttcggcaatacgtgaaggtgtgaccgatttcgggatcaccacc
aggccgctatccagatgccagcggataacaatctgcgccggggttttgccgtatttatctgccagatcgcgaatgactttctgatcgaaaacgcc
tttccctccttgcgctaatgggctccaggattcggtctggattttgtgtgtcgcgttccaggcgtgtagctggcgttgttgcatcagcggatgaagttc
gatctggtttatcacaggcgtcacgccagtttcatcaatcaggcgttgcaggtgatggatctggaagttgcacacgccgatgcttttgattaatccc
tctttttgcaattcgatcatgcctttccatgcttcgacataatggtcgatagcgggaacgggccagtgcattaagtagaggtcgatataatcaagct
ggagtttttttcaggctgtcgagcagggcttcgcgggggcgcttgtggtcgtcgttccacagcttagtggtgatgaacagttcttctctgttgactgag
gcatttttcagggctttgccgacaccttcttcgttcttgtaggccgcggcggtatcaatcgagcgataacccacttctaacgctttttgaatggcggtg
attacttcctcattacttgcttgccagacgcccagtcccagctggggcatgacattgccatcctgtagcttaataacggttggattagccatacgttc
ctcctttatatgaactcaccggagcaggctccggtgaggtgttgtgaacttaagtctggacgaaatgcccgaaaacgaaagtttgaggcgtaaa
aagcttagcgggcggcttcgtatatacggcggctgacatccaacgtaatgtcatgattttcgcccagttgggtcatgccgtgctcttccagtttttca
gcaaagccgggatggagctgccgtccagaccgtagtcggagaggtgggtcggcacgcctaattgctcaaagaaattgcgggttgcggcaat
cgcggcgtcaatacgctcatcatcggaaccttcagtgatgttccagacgcgttcagcatattgcagcagcttagcgcgcttggtatcgcgtttttca
ttccacagtgcaggcaggacgatagccagtgtttgcgcgtgatccagaccgtgcatcgcagtcagttcgtggcccagcatatgcgttgcccagt
cctgcggtacgccagcgccaatcaaaccgttcagcgcctgagtcgccgcccacatgacgttggcgcgcacatcgtagttttctggctctttcag
ggctttcggaccatcttcgattagcgtcagcaaaatgccttctgcgaaacggtcctgaattttggcatcaaccggtttggtaacatactgttccacg
gtgtgtacaaaggcgtccactacgccgttagccacctgacgcggcggcagggtgtaggtataaaccggatcgagcacggcaaataccggct
gaacatgggcagaatggaacgcctgcttgtcgcctgtggttttacgggagatcaccgcgcctgcgttggattctgaaccggttgctggcagcgtc
agcacacagcccatcgggatggcgcttttaatctctttaccgcccgtttgcagaatgtgccacggatcgatattttccggatagttagccgctgcg
gcgataaatttggtgccgtccagtacagaaccgccgccaaccgccagcaggaaagtcactttctgttcgcgaaccagtttcacggcgttcatca
gcgtttcataagccgggtttggctcaataccgccaaattccagcacgtccatgcctttcagggcatccagaacttgatcgagaacgccggttttttt
cacgctgccgccgccgtaggtaatcaatacgcgagcatcgtgaggaatttgttcgcgtaaaccagcgattgcgcctttaccaaacagaatgcg
ggttggggtgtgcagattaaagttgttcattacttgctccctttgctgggccaatatgagggcagagaacgatctgcctgatgtttttcattgtgatcg
ccagcgccctggctctcaatgctcatttctgccaatgtcttgcctatttctccagagtgctggagaaatgctacaaaattgcgcacaatcaaattgc
cgcattattcctaagaaattacgcgatatgaaacgtgaagagatttgccgcttgctggcggataaagttaataaactgaaaaataaagaaaat
agtttgtcaggactgttgcccgatgtgcgtttgttgtatggcgagacgcctttcgcacgtacaccggtgatgtacgagcctggcatcataattctcttt
tccgggcataaaatcggttatatcaatgaacgcgtgtttcgttatgatgccaatgaatacctgctgctgacggtgccgttgccgtttgagtgcgaaa
cctatgccacgtcagaggtgccgctggcagggttgcgtctcaatgtcgatattttgcagttacaggaactgttgatggacattggcgaagatgag
catttccagccgtcgatggcagccagcgggattaactccgccacgttatcagaagagattttatgcgcggcggagcggttactcgacgtgatgg
agcgaccactggatgcgcgtattctcggcaaacagatcatccgcgaaattctgtactacgtgctgaccggaccttgcggcggcgcgttactgg
cgctggtcagtcgccagactcacttcagtctgattagccgcgtgctgaaacggattgagaataaatacaccgaaaacctgagcgtcgagcaa
ctggcggcagaagccaacatgagcgtatcggcgttccaccataattttaagtctgtcaccagtacctcgccgttgcagtatttgaagaattaccgt
ctgcataaggcgcggatgatgatcatccatgacggcatgaaggccagcgcagcagcgatgcgcgtcggctatgaaagcgcatcgcaattta
gccgtgagtttaaacgttacttcggtgtgacgccgggggaagatgcggcaagaatgcgggcgatgcaggggaattaaatacgttcccggtac
agcaaccgggaacggggaaaatcgtcaggcgttacagtatttttttttaatcaccacaaacagcgtgcctaacaagccagcggttaacaagg
caattggcaggatcatcaggaacgtcattacctgatcttcatggcgtttaacgaacggaatcatacttaaggcatagccaaaactggttaccac
gctgacccacagcaatccacttaaccagttgaaaaactggaagcggcggtttggcagaccggaaattcccgccatggttggcagcagcgta

cggacaaatgcaagaaaacgtccagccagcagcgccagcagaccgtggcggtcaaacatgcaggtggcgcgctggtgatatttagcagg
aagctgtgccagccagcctttcaccgttttggtattccctaaccagcgcccctgaatataacttagccagcagcccagacttgctgcggcggtca
gaatcgcaatcgtaggcagaaaatccataacccccctgggcaatcaatgcgcctgccagtatcaacaagctgtcgcctggcaaaaatgaggc
gggcagcaggccgttttctaaaaacagcgtggcaaacatgacaaagtaaacaacgctaacaatatgaggatccgccagcgcggcaaagt
cgtgttgccagagcgcagcgatgatatcttgaataacagccatggactttcctgtggaacagcgtttaagcctttatctgcctattgtactcctgaat
cgtccgggacgccttgatcccggacgcaacaatttaagacttttcacaataaaatgtctgcaaaattgtccaaaagtggcaatgttatacaattc
gcgcaaaaccggcgtccagatcggcaatcagatcgtcgacatcttccagaccaatatgcaggcgaatcaaggtcccgctaaaatcgatctc
gccttgtgggcgaatggcggcgatatgttctggttgatttgccaggatcaacgattcatacccgccccacgagtaggccatgctgaataaactg
aagttatccagatagttcgccagctcttcattattgagtttttcttaagcacaaaggaaaatagcccgctgctgcctgtaaagtctcgtttccagaat
tcgtgacctttactgccaggcagagcagggtggttaactcgcgcaacttgcggatgttctgccagccattcagccactttcagactgctttcatgat
gttgacgcaaacgcacacctaatgtgcgcaggccacggctggttatataggcggtatcggcatcgaccatctggcccatcagataggcattttc
ccgtagctgctcccagcaacgggcattgcacacggcagtgccaatcatcgcatctgaatgcccaaccagatatttggtggcggcttgaataga
aacatcgatgccaaaatccagcgccttaaacagcacaccggctgcccaggtgttgtcgatcataatgatggcatccggcaccacactgcgta
cggcggcaacaatcgccggaacgtcgtggacttccatggtgatggagcctggcgattccagaaacacgattttagtgtttggctgcagatgctt
aacgatatcggcaccaatcagcggatcaaaccatgatgtcgttacgcccagtttgctgaggattttgctacagaaatcctgactcggttcatagg
cggtgttggtcatcaacacatgatcgccctgttcgataaaagcaagaatggaattagcaaccgctgccgccccgcagggaaatagcacgca
gcctgcgccaccttccagttcacacatcgcttgttgtaaggagaaatgggttaacgttccgcgccgtccatagaacaactctccattggcgcgatt
acgtgtcgcgtgtttttggcttctacactgtcaaagaccagcgaagaagcgcgctgaatcacgctatttaccgcgccgagagtgtatttttgctgc
gtcctgcattcaccagttgagtatcaagctttttgtccgccatgtcgggattcctgtttttatacgtctggatgtctaaactagcatgaatattcgcggg
cgcatcctgaagaattggcataaagcagaaaattttcgcaaatggcgtgctggtgactttttactgcgtaaacgcaaggaaaagaaagcaaa
ggtacggcagtatgcaaatagtaatgagaacgactatcaattcgacgtcgttttgatattattatgcgcagattttgtgacttgcgtcctggagatac
acagtgggtaataatttaatgcagacggacctttccgtctggggtatgtatcagcacgccgatattgtcgttaagtgcgtgatgattgggcttattttg
gcctccgtagtcacctgggcaatcttcttcagtaagagcgtagagttcttcaatcagaagcgtcgccttaagcgcgagcagcaactgctggctg
aagcgcgttccttaaaccaggccaacgatatcgccgctgattttggtagcaaaagcttaagcctgcatttgctcaatgaagcgcagaatgagct
ggaactgtcagaaggcagcgacgataacgaaggtattaaagaacgtaccagcttccgtctggagcgtcgggtcgccgcagtgggtcgtcaa
atgggccgcggtaacggctatctggcaaccatcggcgcgatttctccgttcgttggtctgtttggtacggtctggggcatcatgaacagctttattg
gtatcgcgcaaacgcagaccactaacctggcagtcgttgcgccgggtatcgcagaagctctgttagcaacggcaatcggcctcgtggcagc
gattcctgcggtcgttatctataacgtatttgcacgccagattggcggctttaaagcgatgctgggtgatgttgcagcgcaggtattgttgctgcaaa
gccgtgacctggatctggaagccagcgccgctgcgcatccggttcgtgtcgcacaaaaattacgcgcaggataatatccgatggcaatgcat
cttaacgaaaacctcgacgataacggcgaaatgcatgatatcaacgtgacgccgtttatcgacgtgatgttggttctgctgattatctttatggtgg
cggcaccgttagcgacggtagatgtgaaggtgaacttgcctgcttctaccagcacgccgcagccgcgcccggaaaaaccggtttatctgtcg
gtgaaggcagacaactcgatgtttatcggtaacgatccggtcaccgatgaaacaatgattacggcgttgaatgcgttaaccgaaggcaagaa
agacaccaccatcttcttccgagcggacaaaaccgtcgattacgagacgttgatgaaggtaatggatacgctgcatcaggcgggttacctga
agataggtctggtcggcgaagaaaccgccaaagcgaagtaaagtagaattgcctgatgcgctacgctcatcaggcctacaaaatctattgca
acatgttgaatcttcgtgcgtttgtaggccggataaggcgttcacgacgcatccggcattaggtgctcaatgcctgatgcgctacgcttatcaggc
ctacaaaatctattgcaacatgttgaatcttcatgcgtttgtaggcggataaggcgttttcgccacatcaggtaagagtgaattcacaatgatgccc
ggttgcttttcacaaccgggcatttttttaacctaaatgctcgccgccgcacacgccgtgcacttctgcggtgacgtagctcgactcctgacttgcc
agataaacatatacaggggccagttccgccggttgccccgcacgtttcatcggcgtttgctgaccaaactgcgggatcttatcctgcgtttgtccg
ccggaaatttgcagtgctgtccagatcgggcctggcgcgacaatattcacccgaatacctttctccgcgacctgttttgccaagccacggctgta
gttcagaatcgccgccttcgtagccgcatagtccagtaaatgcggacttggctggtatgcctggattgacgaagtggtgatgatacttgcacctttt
cggtagcaggggggatcgcttcctgggttagccagaacagcgcgaaaacgttaatggcaaaggtcttttgaaactgttcgctggtgaggtctgca
atatccggaatggcaacctgtttcccggcgaccagcgccataatatccagcccgcctaacgccttgtgcgcttcgtgaaccagcgaacgggc
aaatttctcatcgcttaaatcgcctggcagcagaacggctttgcgtccgcattcttcaatgatctttttcacatcctgagcgtcttcttcttccacggga
agataactgatcgccacgtcagccccttcacgcgcgtaagcgatggcggcagcgcgacctattccggaatcgccccctgtcaccagtgcttta
cgatctttcaggcgaccgctaccaacataggttttctcgccgcaatccggtaccggtgtcatcttcgcctggatgcctggcgtcggttgtttctgtttg
ggatattcaccagtgtaatactgcgtggtcgggtcttttaaatgagacatcgtttttctcccttcaggttcaacgtcctttaagggtagacgttctcgat
gcgttgataagggaaccaggaagatccctaaacctcagaattatgcgacaaaggtttaacgtatatgttgatttgctgttgcgtgctgtttactcaa
ttgcgatatactgttgcccgttttaactacacgacaggaatgtatggaacgttttcttgaaaatgcaatgtatgcttctcgctggctgcttgcccccgt
gtactttggcctttcgctggcgttagttgccctggcgctgaagttcttccaggagattattcacgtactgccgaatatcttctcgatggcggaatcaga
tttgatcctcgtgttgctgtcgctggtggatatgacgctggttggcggtttgctggtgatggtgatgttttccggttatgagaatttcgtctcacagctgg

atatctccgagaacaaagagaagctgaactggctggggaaaatggacgcaacgtcgctgaaaaacaaagtagcagcgtcgattgtggca
atttcttccattcacttactgcgcgtctttatggatgcgaaaaatgtcccggataacaaactgatgtggtacgtcattatccatctgacgtttgtgctttc
tgcatttgtgatgggctatcttgaccgactgactcgtcataatcactgatctgattcgggcgcggttcgcgcgcccgttattaacaggtcatttatcgg
aagacgcctgccacagattcagctcgccatcggcgatatgctgatcaatctgcgccagctccttggtgctaaatgtcagattattcagcgcctgc
acgttctcctctagttgctccgcgcggctggcaccaatcaataccgacgtcacgcgatcatctttcagcaaccagcttaacgccatttgcgccatt
gattgtccacgctgctgtgccatttcattcaataagcgcaggctgttgaggttggcttcggtaagcattttcggtgtcagaccacgaactttattccctt
cacgatgcatccgtgaatcttgcggaatgccgttgagatattttccggtcagcaatccctgagccagaggagtaaaggcaatacagcccacgc
cgttattttgcagggtatccagcaggccgcttttatccacccagcggttcagtaaattgtacgaaggttgatgaattaacagcggaattttccactc
gcgcagcaactcgaccatttttgcgtccgctctggcgagtaagaggagatcccgacatacagcgccttaccgctttgtaccgcatgagccagc
gcagaggcggtttcttccatcggcgtattttcatcgacgcgatgagagtaaaagatatcgacatactcaagccccatacgcttcaggctttggtcg
aggctggcgagcaggtatttacgtgaaccgccagagccgtaagggccgggccacatatcgtagccagccttggtagagataatcagttcatc
gcgataagcggcaaaatcctcccgcagcaggcgaccaaagttctcttctgcgcttcctggaggcggcccgtaattgttggctaaatcaaagtg
cgtaatgcccaaatcaaacgctttacgcaggatcgcacgctgtgattccagcgcgttaacgtgaccgaaattgtgccataaaccgagcgataa
cgcgggcaggcgtaaaccactttttccgcaatagcggtattgcatctgcccgtaacgttcgggattcgctaaccagaccatgacctctcctttcca
ccgttcaatttcgaaacaatgtttctagtttagcgattcgccagcgcgtatcccgtagtctggctcacagagtgacgaaaaactgggcaaaaata
cgtgcttatgctttgcttaaaaaaacaccaactgaggagtgcaacgatgccgcgtttaaccgccaaagatttcccacaagagttgctggattact
atgactattacgctcacgggaaaatctcgaaacgtgagttcctcaaccttgcggcgaagtgcggtaggcgggatgacggcattagcgttgtttg
atttgctcaagccaaattatgcgctggcgactcaggtagagtttaccgacccggaaattgttgctgagtacatcacgtatccttcgccaaatggtc
acggcgaggtgcggggttatctggtgaagcccgcaaagatgagcggcaaaacgccagccgtagtggtggtgcatgagaatcgtggactga
atccgtatatcgaagatgtggcacggcgagtggcgaaggcggggtatatcgccctggcacctgacggcttaagttccgttggaggttatccgg
gaaatgatgataaaggtcgtgagctgcaacagcaggttgatccaacaaactgatgaatgatttctttgccgcaattgagtttatgcaacgctatc
cgcaagcgacaggcaaagtgggtattaccggattttgctatggcggtggcgtatcgaacgcggcggctgtcgcgtatccggaactggcctgc
gcggtgccgttttatggtcgtcaggcacccactgccgatgtggcgaagattgaagcgcctttactactccacttcgcggaactggacacccgaa
tcaacgagggctggcctgcttacgaggcggcgttgaaagccaataataaggtttatgaggcgtatatctatccgggggttaatcacggattccat
aatgattccacgccccgttatgacaaatctgccgccgatcttgcctggcaaaggacactgaaatggttcgataaatatctctcctgataggtttatc
tcttacgggattacgtcttaaacaagcatgaaaaaaatagcgtgcgcaaaagtcgttctttgcctaaaatatcgctatatatgacaatatatagcga
atgaggtgaacgatgaataaccattttggtaaaggcttaatggcgggattaaaagcaacgcatgccgacagtgcggttaatgtgacaaaattct
gtgccgattataaacgcggttttgtattaggctactcacaccggatgtacgaaaagaccggagatcgccagcttagcgcctgggaagcgggta
ttctgacgcgccgctatggactggataaagagatggtaatggatttctttcgtgagaataattcctgttctacgttgcgcttttttatggccggttatcgc
ctcgaaaattgatcaaacatacgtattatcttgctttaattaattacactaatgcttcttcccttcgttttagcgccccgccgcagtatcatgatatcgat
aaccataataaatgtgtggtaaatggcgcatcgatcgcattattgattttgcgattgaggcaaaatatatgccaggtcttcgcaacggaataacta
taaatgactggagataacaccctcatccattctcacggcattaaccgtcgtgatttcatgaagctttgtgcagcattagccgccaccatggggtta
agtagcaaagccgctgcagagatggccgaatcggttactaacccgcagcgtccgccagttatctggattggcgcgcaggagtgcaccggttg
tacggaatctctgcttcgtgcaacgcatccaacggtagaaaacctcgtactggagactatctctctggagtatcacgaagtgctttccgccgcctt
cggtcatcaggtcgaagagaacaaacataacgctctcgagaagtacaaagggcagtatgtgttagtggtggatggttccatcccattaaaag
ataacggtatttattgcatggttgccggtgagccgattgtggatcacatccgcaaagcggcggaaggcgcagcagccattatcgctatcggttc
ctgctctgcgtggggcggtgttgccgcagctggagttaacccaactggcgcagtcagcctgcaagaagttctgccaggcaaaaccgttatcaa
tattccgggctgcccgccgaacccgcacaacttcctcgcgaccgttgcgcacatcatcacttacggcaaaccgccgaaactggatgacaaaa
accgtccgaccttcgcctatggccgtctgattcacgaacactgcgaacgtcgcccgcacttcgatgctggtcgttttgccaaagagttcggtgatg
aaggccaccgcgaaggctggtgcctgtaccacctcggctgtaaagggccagaaacttacggcaactgctcaacgctgcaattctgcgatgtt
ggcggtgtgtggccggtggcgattggtcacccttgctatggctgtaacgaagaaggtatcggcttccataaaggcatccatcagcttgccaacg
tcgaaaatcaaactccgcgttcacagaaaccggatgttaacgctaaagagggcggcaacgtctctgcaggcgctattggtttgctcggcggtg
tggttgggttggttgccggtgtcagcgtgatggcggtgcgtgaactgggtcgtcagcaaaagaaagataacgctgactcacggggagaataa
ccgtgaacagacgtaattttattaaagcagcctcctgcggggcattgctgacgggcgcgctgccgtctgtcagtcatgcggctgctgaaaaccg
cccgccaattccgggatcgctggggatgttgtacgactcgaccttgtgcgtaggctgccaggcttgcgtcaccaagtgtcaggatatcaatttcc
ctgaacgtaacccgcaaggggaacagacctggtcgaacaacgacaaactgtcgccgtataccaataacatcattcaggtgtggaccagcg
gcacaggggtcaacaaagaccaggaggagaacggctacgcgtacattaagaaacagtgtatgcactgcgtcgatccgaactgtgtctctgt
gtgcccggtctctgcactgaaaaaagatccgaaaaccggcattgtccattacgacaaagatgtgtgcaccggctgccgttactgcatggtcgc
ctgtccgtacaacgtgccgaagtacgactacaacaacccgtttggtgcgctgcataagtgcgagctgtgcaaccagaaaggtgtggaacgtct
cgataaaggcggtctacctggctgcgtagaagtgtgcccggcgggcgcggtgatttcggtacgcgtgaagagctgatggcggaggcgaaa

aaacgtctggcgctgaagcctggcagcgaataccactatccgcgtcagacgctgaaatctggcgacacttacctgcatacggtgccgaaata
ttatccgcatctgtacggcgagaaagagggcggcggtactcaggttctggtactgacgggtgtgccttatgaaaatctcgacctgccgaaactg
gacgatctttctaccggtgcgcgttccgaaaatattcaacacaccctgtataaaggcatgatgctaccactggctgtgctggcggcttaaccgt
gctggttcgtcgcaacaccaaaaacgaccatcacgacggaggagacgatcatgagtcatgatccacaaccgctgggcggcaaaatcatca
gtaaaccggtcatgatttttggaccgttaatcgtcatctgtatgctcctgattgtgaagcgtctggtgttcggtctgggctctgtctctgacctgaacgg
cggcttcccgtggggcgtgtggatcgcgtttgacctgctgattggcaccggctttgcctgtggcggctgggcgctggcgtgggcggtatacgtcttt
aaccgtgggcaataccatccgctggtgcgtccggcgctgttggcgagtctgtttggttactcactgggtggcttgtcgatcactatcgacgtgggtc
gctactggaacctgccgtacttctacattccgggtcacttcaacgtgaactcggtactgttcgagacggcggtctgtatgaccatctatatcggcgt
gatggcactggagtttgctccggcactgtttgaacgtctggggtggaaggtgtcgctacagcgactaaacaaggtgatgttcttcatcatcgcgct
cggtgcgctgctgccgaccatgcaccagtcttcaatggggtcgctgatgatctcggcgggctacaaggtgcatccgttgtggcagagctatgaa
atgttgccgctgttctcgctgctgacggcgttcatcatgggcttctcgattgtcatctttgaaggttcgctggtgcaggcgggtctgcgtggcaacggt
ccggatgaaaagagtctgtttgttaagctgaccaacaccatcagtgtgttgctggcgattttcatcgtgctgcgctttggcgagcttatctatcgcga
caagctgtcgttagcgtttgccggtgacttctactccgtgatgttctggattgaagtcctgctgatgctcttcccgctggtcgttctgcgtgtggcgaag
ctgcgtaatgattcccgcatgctgttcctgtcagcactgagcgcactgttaggttgtgcaacctggcgtctgacctattcgctggtggcattcaaccc
gggcggcggttacgcctacttcccgacctgggaagaactgttgatttctattggttttgtggctattgagatttgcgcttacatcgtactcattcgtcta
ctgccgatacttcctcctttaaaacaaaacgatcataatcgtcatgaggcgagcaaagcatgagccagagaattactattgatccggtaacccg
tattgaggggcatttacgcatcgattgcgaaatcgaaaatggcgtcgtttcgaaagcatgggcttccggtaccatgtggcgcggcatggaaga
gatcgtgaaaaaccgcgatccgcgcgatgcatggatgattgtgcaacgtatctgtggcgtatgtactaccactcacgcgctgtcttccgttcgtgc
ggcagaaagtgcgctgaatatcgacgttccggttaacgcgcaatacatccgtaacatcattctggctgcgcacaccacgcatgaccatattgtt
catttctatcagctttcggcgctggactgggtggacatcacttctgcactgcaagctgacccaaccaaagcctccgaaatgctgaaaggcgtttc
gacctggcacctgaacagtccggaagagttcaccaaagttcagaacaagatcaaagatctggttgccagcggtcagttgggtattttcgctaat
ggctactggggtcatccggcgatgaaactgccgccggaagtgaacctgattgcggtagcgcactacctgcaagcgttggagtgccagcgtga
cgctaaccgcgtcgtggcgctgctgggcggtaaaacgccgcacattcagaacctggcggtaggtggtgtcgcgaacccgatcaacctcgac
ggtttgggcgtgctgaaccttgagcgcctgatgtacatcaagtctttcatcgacaaactgagcgactttgttgagcaggtttataaggttgataccg
cagttattgccgcgttctacccggaatggctgacacgcggtaaaggtgcggtgaactacctgagcgtgccggaattcccgaccgacagtaaa
aacggcagcttcctgttcccgggcggctacattgagaatgcggatctgtcctcgtatcgtccgatcacttctcattccgatgaatacctgatcaaa
ggcattcaggaaagcgcgaagcactcctggtataaagacgaagcgccgcaggcaccgtgggaaggcaccaccattccggcttatgatggtt
ggtctgacgacgggaaatattcctgggtgaaatcaccgactttctacggcaaaacggtagaagtggggccactggctaatatgctggtgaaac
tggcggcaggtcgcgaatctacccagaacaaactgaatgaaatcgttgcgatttatcagaaactgactggcaacacgctggaagtggcaca
gctgcactccacgctgggccgtattattggtcgtaccgttcactgctgtgaattgcaggatatcctgcaaaaccaatacagtgcactgatcacca
atatcggcaaaggcgatcacaccacctttgtgaagccgaacattccggcaacgggtgaattcaaaggtgttggcttcctcgaagcgccgcgc
ggtatgctctctcactggatggttattaaagacggtatcatcagcaactaccaggcggttgttccatcaacctggaactctggtccgcgtaacttca
atgatgacgtcggtccttacgagcagtcgctggtgggtacaccggttgccgatccgaataaaccgctggaagtggtgcgtaccattcactccttt
gacccgtgcatggcctgtgcggtacacgtagtggatgctgacggcaacgaagtggtttcagtgaaggttctgtaatgcgtattttagtcttagggg
tcggcaatattttgctgaccgatgaagccatcggtgtgcggattgtcgaagcgttagagcaacgatacattctgccggattatgttgagatcctcg
atggcggcacggcgggaatggagctgcttggcgacatggcaaatcgcgatcatttgattattgcggatgccattgtctcgaaaaagaacgcgc
cgggaacgatgatgatcctgcgggatgaagaagttccggcgttgtttaccaacaaaatctctccgcatcagcttggcctggccgacgtcttgtcg
gccctgcgcttcaccggcgagtttccgaaaaagctgaccctggtcggcgtgatcccggaatcgctggagccacacatcggcttaacgccgac
ggttgaagcaatgattgaacctgcgcttgagcaggttctggctgcgctgcgtgaatctggcgtggaagccatcccacgggaggcgattcatga
ctgaagagatagcaggtttccagacctccccgaaggcgcaagtacaggcagcgtttgaagaaattgcccggcgttcgatgcacgatctctcttt
tctgcatccttcaatgccggtgtatgtttctgattttacgctgttcgaaggtcagtggacggggtgtgtgatcaccccgtggatgctgagtgcagttat
cttccccggcccggatcaactctggccgctgcgcaaagtgagtgaaaaaattggtctgcaactgccgtatggcactatgacctttaccgttggc
gaactggacggtgtttcgcaatatctctcctgttcgctgatgtcgccgctttcgcacagcatgtcgattgaagagggccaacgcctgacggatga
ctgcgcacgaatgatcctttcgctgccagtcacgaatccggatgtaccacacgcagggcgtcgcgccctgctgtttggtcgcaggagtggcga
aaatgcatgagttgtctctttgccagagcgccgttgaaattatccaacggcaggcggagcagcacgatgttaagcgcgtcaccgccgtgtggct
ggaaattggcgcgctctcctgcgttgaggagagcgccgtccgttttagttttgaaattgtctgccacggaacggtggcgcaagggtgcgatttac
atatcgtctataaacccgcccaggcttggtgctgggattgcagccaggtggtggagattcatcagcacgatgcgcagtgtccgctctgtcacgg
cgagcggttgcgtgtcgataccggcgattcgctgatcgtcaaaagtattgaagttgaataaccggagttaataatgtgtattggcgttccaggcc
aggtgctggctgtcggtgaagatattcaccagcttgcgcaggttgaagtatgtggtatcaagcgcgatgtgaatatcgccctgatttgtgaaggta
accctgccgatctactgggccagtgggtgctggtacacgtcggatttgccatgagcatcatcgacgaagatgaagccaaagccacattagac

gcactgcgccaaatggattacgacattaccagcgcgtgatgattagctttcgtaggcctgataagacgcggcagcgtcgcatcaggcattgtgc
acgattgccggatgcggcgtgaacgccttatccggcctggatggcttgctgcgacgaacaccaacccttacccctgacgcttatcttccgtattc
gtctcgaaatcactggcgtcatggcgctcatgcaactgctcattcagcggtccgttggtgcggttaacaatacgcccacgtttcaccgccggacg
ttcgcctacttctttcgcccagcgttgtacatgcttataactgcccgcatcaagaaactcagcggcatcatacacaccacctaacaccacgttgcc
aaaccacggccaaatcgccatatccgcaatggtgtactcatcgcccgcaacaaacttatgctgcgccagttgcttatccagcacgtcgagcag
acgtttggcttccatggtaaagcggttgatggcgtactcaatctttaccggtgcgtaatggtaaaagtgaccaaaaccaccgccgaggaacggt
gccgcgccctgtaaccagaacagccagttcatcgtttcagtacgctttgccaaatcctgcggcaggaagtagccaaatttctccgccagataaa
gcaggatcgaaccagattcaaacacgcggatcggcggattatgcgtatgatcgcgcagcgccgggatcttcgagtttgggttcacttcgacaa
agccgctggagaattgatcgccatcgccaatacgaatcagccaggcgtcgtactctgcaccagtaacgcccagcgccagcagctcctcaag
cataatcgttactttctgaccgttcggcgttcccagcgaataaagttgcaatgggtgtttgccaacgggcagcgtttttcatgcgtcggaccagaa
accgggcgattgatattggcgaacgcgccgccagcggatttatcccacgtccagactttcgcgggctgataagtattgtctgtcatagtgagttgc
cttctgagtggtagtgttgaagcagtgtagcaggtcacttgctgtacagaatgtgaatttttaaccctttgcgccagccgctgcgatgaggtgtatg
attagtctgacctctgtccggttaatgtcgctgagcaggtaatcagagcacaacaattacgccaactgcttaggttgtattgttaaaggtaaagtg
atgagcaaaggaacgaccagccaggatgccccgttcgggacattattgggctacgccccaggtggggtagcaatctactcttcagattacagt
tctctcgatccgcaggaatacgaagatgacgccgtattccgtagctatatcgacgacgaatatatgggccacaagtggcaatgcgttgaatttg
ctcgccgttttctctttctgaattacggtgtggtctttactgacgtgggtatggcgtgggagattttctcgctgcgcttcctgcgagaagtggttaatgac
aacatcctgccattgcaggcatttcctaacggctcgccgcgtgcgccggtcgcgggtgcgcttcttatctgggataaaggcggtgaatttaaaga
cactggccatgtcgccatcattacccaattgcatggcaacaaagtccgtattgcggaacagaacgtgattcattccccgttgccgcaagggca
acagtggacgcgcgagctggagatggtggtcgaaaacggctgctataccctgaaagacacttttgatgacaccaccattctgggctggatgat
ccagacggaagatactgaatacagcttaccgcagccggaaattgcaggcgagctgctgaaaatcagcggagcgcgcctggaaaacaaa
ggccagtttgacggtaaatggctggatgaaaaagatccgctgcaaaacgcctatgtgcaggccaacggtcaggtgatcaatcaggatccttat
cattactacaccattaccgagagtgccgagcaggagctaattaaagccaccaacgagctgcacctgatgtatcttcacgcaaccgacaaggt
gctgaaagatgacaacctgctggcgctgttcgacatcccgaaaatcctctggccacgtttgcgtctctcctggcagcgtcgccgtcaccatatga
tcactggtcgtatggatttctgcatggatgagcgtggcctgaaggtttacgagtacaacgccgactccgcctcctgtcataccgaagcgggcttg
atcctcgaacgttgggcggagcagggctataaaggcaacggcttcaatccggcggaagggctgattaacgaattggctggtgcctggaaac
acagtcgtgcacgtccgtttgtccatatcatgcaggacaaagatatcgaggaaaactatcacgcgcagtttatggagcaggcgctgcaccag
gcgggctttgaaacgcgtatcttgcgtggattggatgaactgggctgggatgctgccgggcaactgattgatggggaagggcgactggttaact
gcgtgtggaaaacctgggcgtgggaaaccgcgtttgatcagattcgtgaagttagcgaccgtgagtttgctgcggtgccaatccgtaccggtca
tccgcaaaacgaagtgcgtcttatcgacgtattgctgcgcccggaagtgctggtctttgagccgctgtggacggtgatccccggcaacaaagc
gattctgccgatcctctggtcgctgttcccgcaccatcgttacctgctggataccgatttcactgttaatgatgaactggtgaaaacaggttacgca
gtgaaaccgatcgccggtcgctgtggcagcaatatcgacctcgtcagccatcatgaagaggtgctggacaaaaccagcggtaaatttgccga
gcagaaaaacatctatcagcaactgtggtgtttgccgaaagtggacggtaaatacattcaggtatgtaccttcaccgttggcggcaactacggt
gggacgtgtttgcgcggtgatgaatcactggtcatcaaaaaagagagtgatattgaaccgttaattgtggtgaaaaagtaatacatgttgtagca
accccgtactgattatgaagaataatcagtacggggattaatcaaaatattcaatatttatatattccaatatttattcatttcatatgtgaatatattaa
ccagtggaatacctgtgttttgattatttctaaaggttttgaatgaatgcttattgtctgatacacaagaaataacactctttttatcgttaaaaaatgat
atttcactttgcccatgccgttaaagtcactgataatgcgtccgttcgtaaattcaaaatggcgtaatctaatatatgctaaatttatttgttggccttgat
atatacacagggcttttgttattgcttgctctggcatttgtgttgttctacgaagcaatcaatggttttcatgacacggcgaatgcggtggcagccgtta
tttatactcgtgccatgcaaccacaacttgctgtggtgatggcggcatttttaactttttggcgtgttattgggcggacttagcgttgcctatgccattg
tccatatgttgccaaccgatttgttgctgaatatggggtcaacccacggcctggcgatggtcttttccatgctgctggcggcgattatctggaacctg
ggaacgtggttcttcggtttaccggcctccagttcgcacaccttgattggtgcgattatcggcatcggtttaaccaacgcgctgttaaccggctcat
cggtgatggatgcgttaaacctgcgtgaagtgaccaaaattttctcctcgctgattgtttcccctatcgtcggcctggtcattgcgggaggcctgata
ttcctgctgcgacgctactggagcgggacgaaaaagcgtgaccgtattcaccgcattccggaagatcgcaaaaagaaaaaaggcaaacgt
aaaccgccattctggacgcgtattgcgctgattgtttccgctgcgggcgtggcgttttcgcacggcgcgaacgacggacaaaaagggatcggc
ctggtaatgctggtactggtggggattgcccctgctggcttcgtcgtcaatatgaatgcgtccggctatgaaattacccgtacccgcgatgccgtta
ccaacttcgaacactacctgcaacagcatcctgaactgccgcagaagttgattgcgatggaacctccattgcctgcagcatcgactgatggca
cgcaagtaacagagtttcactgtcatccggcaaatacctttgatgctattgcgcgcgttaaaacgatgctgccaggcaatatggaaagttacga
gccgttaagcgtgagtcagcgcagccagctgcgccgcattatgctgtgcatctctgatacctccgcgaagctagcgaaactgccaggcgtcag
taaagaagaccagaacctgctgaaaaaacttcgcagcgatatgttaagcaccattgagtacgctccggtgtggatcatcatggcggtagcact
ggcgctcggcattggcaccatgattggctggcgtcgtgtagcgatgaccatcggtgagaagattggtaagcgcggcatgacgtatgcgcaag
gcatggcggcacaaatgacggcggcagtgtctatcggtcttgccagttatattgggatgcccgtctccacaacacacgtcctctcgtctgcagtt

gcagggacgatggtggtggacggcggtgggttacagcgtaaaacggtaaccagcatcctgatggcgtgggtatttactttaccggcggcaatt
tttctttctggtgggctgtactggatagcattgcagttgatttaaccttcctgaaaatgcccggtcctggcgatcgggcatttccatttttgactagtgat
aaccacgcgcggtcataaaatccgtaatcgctttttctgcatcaaccaacacctgttccagtggctgattggcatcgatatcaaccagttgtgcac
caccaaaggttaactgtggcgttatggcaatcttcctcgccagcgattcccgtttatggtcgggtttacgtgcacaggcaacttcaaggtcaacatt
gagtttgatgaccagatcaggcttatggctcgccatccagtgaaacgctttacgttcctggcttgccagccatgagacaaaacgaccaccttca
acgttaggtgggaacaccgtaccatcgtaagcgccaggaatttggtcctgaggataacggtcggttagaacaattaacccgcgacgacgac
aggcaagcatatgacgaaagcgcagtaagcgacgggcgacaaacgctgtaattaccagcgccggaactggtccaggcaattttttgctgttt
tcacctgatttcgttcaattgttttatgtaaggattttcccatcaacggtaattttgtcactgcacgaccgacatttccggcctgttttcctaaatgaactct
ttcggcagcaccatatttttcgacaacggtaataagatgttcacacaccgttgacttgcctgaaccatcgctaccaataacggcaattaatggag
gtgttattgactgcatgaatatatcctttaatttaaatatccattaaaaatatttatttggttaatatgtttttatgaaagcgtaattcaggtcaatgtcaca
attaaccattgtcacaataaggttgaacggatattctgagcacggaccacgtaatattcaatacattatttactgccgggtttttcgtgaatcaacgt
aatatgtcaataattaattccacgccagtgcgtgttattgccattgtaggatgtgatggttcaggtaaatcgaccctcacggcaagcctggtaaatg
aactggcagcaagaatgccaacagaacacatttatctcgggcaatcgtccgggcgaattggcgaatggatttcacagctccctgttattggcgc
accttttgggcgttatctgcgaagtaaagcggcacatgtgcacgaaaagccctcaacaccgcctggcaatattactgcactggttatctatctgct
ttcctgctggcgggcgtacaagtttcgcaaaatgttgtgtaaaagccagcaaggctttctgctcatcaccgaccgctacccgcaggttgaagtgc
cggggtttcgctttgatggcccgcaattggcaaaaaccacgggcggtaacggttggataaaaatgttaaggcagcgcgagctgaagctgtac
caatggatggcatcttatttgcccgtattgttgattcgtcttggcattgatgaacaaaccgcgtttgcgcgtaaacctgaccaccaactggcagcgtt
acaggaaaaaatcgccgttacgccgcaactgacattcaatggcgcaaagatccttgaactggatgggcgacaccccgccgatgaaattctg
caagcgtcactacgcgcaattcacgccgcgctttcctgatcaccaaaatggataggggatgatttaacgaatggatgcactacaaactcaaac
tgttaatagtacaaccgcgccgcagcccaactacattccggggctgattgcggtggtcgggtgtgatggcaccggtaaatccacactgaccac
cgacctggtgaaatcgctgcaacaacactggcaaaccgagcggcgctatctggggctgctctccggcgaagacggcgacaaaatcaaac
gattgccgttggttggcgtctggctggaacggcgactggcggccaaatcctcgaaaacccaaagcatgaaaaccaaatctccggcgctatgg
gcggcggtgattatgtactgcttctcgctgcgaagaatggcgaatctacgcaaggttcagcgactggcgcaaagtggcgttctggtggtcagcg
atcgcttcccgcaggctgaaatttcgggcttttattatgatggaccggggattggcgtcgaacgtgcgaccgggaaaatcagcatgtttctggcg
cagcgcgaacggcgtttataccaacaaatggcgcaatatcgcccggaattaattattcgcctgggcattgatattgagactgccatctcccgca
agcctgaccatgactatgccgagctgcaggacaaaatcggtgtcatgtcgaagattggctataacggcacaaaaattcttgagattgattccag
agcgccctacagcgaagtgctggaacaggcacagaaggcggtttctctggttgccatcgtttctgaccgccgaagtttaacttaaggctggcgg
gatactttttactatcaacgataaggtcaggctgaattggcgggttttaacatcaaacattggtttgcagatggcgcgtttcgcaccattattcgcaat
agcgcctggttaggctccagtaatgtcgtgagcgccttgctgggtctgttggcgctctcgtgtgccggtaaagggatgacgcccgccatgtttggc
gtactggtgattgtgcaatcgtacgccaagtcgatcagcgattttattaagtttcagacatggcaactggtggttcagtacggaacaccagcatta
accaacaataatccgcagcaattccgcaatgtcgtctcattttccttctcgctggatatcgtcagcggcgcggtggcgattgtcggtggcattgcct
tactgccattcctttcccattcattaggtctggatgaccaaagtttttggctggcagcgctctattgcacgctcattccttcaatggcttcctccacgcc
gaccggcattctgcgtgcggtagatcgcttcgatttaattgctgtacagcaggcgacgaaaccttttctgcgcgcagcggggagcgtcgtagcct
ggtattttgactttggttttgcgggttttgttattgcctggtacgtgtcgaatctggttggcggcaccatgtactggtggtttgccgcgcgcgaattacgc
cgccgaaatatccataacgccttcaaattgaatctgtttgagtctgcccgatacattaaaggcgcgtggagttttgtctggtcaaccaacattgccc
actccatctggtcggcgcgtaactcgtgcagcacagtgttggtggggatcgtgttaggacccgctgccgccgggttatttaaaatcgccatgaca
ttcttcgacgccgccggaacgccagcgggtttgctgggtaaaagtttttacccagaggtgatgcgtttagatccgcgcaccaccagaccgtggtt
gttaggtgtgaagtccggtttactggcgggcggaatcggtattctggtggcgctcgcggtgttgatcgtcggcaagccgctcatttcgctggtgtttg
gcgttaagtatctcgaagcgtatgacctgatttagggaaggtgcgaataagcggggaaattcttctcggctgactcagtcatttcatttcttcatgttt
gagccgatttttctcccgtaaatgccttgaatcagcctatttagaccgtttcttcgccatttaaggcgttatccccagtttttagtgagatctctcccact
gacgtatcatttggtccgcccgaaacaggttggccagcgtgaataacatcgccagttggttatcgtttttcagcaacccccttgtatctggctttcacg
aagccgaactgtcgcttgatgatgcgaaatgggtgctccaccctggcccggatgctggctttcatgtattcgatgttgatggccgttttgttcttgcgt
ggatgctgtttcaaggttcttaccttgccggggcgctcggcgatcagccagtccacatccacctcggccagctcctcgcgctgtggcgccccttg
gtagccggcatcggctgagacaaattgctcctctccatgcagcagattacccagctgattgaggtcatgctcgttggccgcggtggtgaccagg
ctgtgggtcaggccactcttggcatcgacaccaatgtgggccttcatgccaaagtgccactgattgcctttcttggtctgatgcatctccggatcgc
gttgctgctctttgttcttggtcgagctgggtgcctcaatgatggtggcatcgaccaaggtgccttgagtcatcatgacgcctgcttcggccagcca
gcgattgatggtcttgaacaattggcgggccagttgatgctgctccagcaggtggcggaaattcatgatggtggtgcggtccggcaaggcgcta
tccagggataaccgggcaaacagacgcatggaggcgatttcgtacagagcatcttccatcgcgccatcgctcaggttgtaccaatgctgcatg
cagtgaatgcgtagcatggtttccagcggataaggtcgccggccattaccagccttggggtaaaacggctcgatgacttccaccatgttttgcca
tggcagaatctgctccatgcgggacaagaaaatctcttttctggtctgacggcgcttactgctgaattcactgtcggcgaaggtaagttgatgact

catgatgaaccctgttctatggctccagatgacaaacatgatctcatatcagggacttgttcgcaccttccttaataaaaatgaccttaaggcattt
atcgcttttccgtcatcactttatcccgacgatccaaactggatacccccttattcattgaacgcaacgagcatttgtctgcgaaaaatccgggga
cggaccatatcatctggcaggcgtgggtggcaaaaaaagcggggcagatagttgggcgtattaccgcgcaaatagataccttgcaccgcga
gcgttacggcaaagataccggtcatttcggcatgattgacgccattgatgatccgcaggtttttgccgcgttgtttggcgcagcggaagcgtggtt
gaagtcacaaggtgcaagtaagatcagcggtcctttcagcctgaatatcaatcaggaaagcggattactgattgaaggttttgacacaccacc
ctgtgcgatgatgccacacggcaaaccgtggtatgccgcgcatattgaacaactgggttatcacaaaggcattgatttactggcgtggtggatg
cagcgaaccgatctcactttctctccggcgctaaaaaaactgatggatcaggtgcgcaaaaaggtgaccattcgctgcatcaatcgtcagcgg
tttgccgaagagatgcagatcctgcgtgagattttcaactctggctggcagcacaactggggatttgtgccgtttaccgaacatgaattcgcgac
catgggcgatcaacttaaatatctggtgccggacgatatgatctatatcgctgagattgattctgcaccctgcgcgtttattgtcggcttgccgaac
atcaacgaggcgattgccgatctgaacggatcgctctttcccttcggctgggcaaaattgctgtggcgtttgaaagtcagcggtgtgcgaactgc
gcgagtaccgctgatgggcgtgcgcgacgagtatcagttcagccgcatcggcccggtgattgcgctgttattgattgaagccttacgcgatccgt
ttgcccgccggaagatcgatgcgctggagatgtcatggatcctcgaaaccaatacaggcatgaataacatgctggaacgcattggggcgga
gccgtacaagcgttaccgattgtatgaaaagcagatttaataccagctaactcaggttcatctccagcggcaccgccgagcgaatcaaatggt
ggccttccagctcgatatcgtggagattctttttcacggtccgcacatgatcgcgtgctgcgcgctgggcgacgtgcggcagccgctgcaacac
cgcgttgtagatccgcgcatgctggcgatcgatctgctttttttgctgtggtcgatggtagagattatttacgctggcaaacactgaattaaacatca
gatcggtcaatgattgcagcgtaaacaccagcacctgattgtgagaagcctgacaaatggcgagatggaaagcgtgatccaactgcgcatgt
tcgatcagcgaaatctctttgttgttctcactggcggcgagcatttttttcataacagcgggttatcacaacaaaatcagcctgcgttcccagcgttgc
cgccagccttgccgattcgccctccagtaatgcgcgaacgtcgagcagatcgtacagcgttcgcggctgcgtactgaacagatggatcagcg
ggctggtgtcctgcacccgattaagccgtgcgacacgagaatcgcgaccctgcgccgtttcaataatcccgcgcccgcgcagcacggtcagc
ccttcacgcagtgcggagcgtgagaagccgagcttttcacacagtcgacgttccgagggaagcggctgaccgaccttcagtacgccgtcgat
aattaaccgttcgatactctctgcaaccacttcgcaaatagggcgacgttcatctttcattcccggacctcgtgcacaggtaggaccaatttttctgt
tcttaactcgcaaaacacgcacatcacgtaagtgattatgttacatcaatttaacattgagttaaccaagacaaggtcacagagctggaaaaaa
aatggtctgaccggtagctaaagagatagacgaaaacgaaaagcccgcttaataactgttcacagaagcagcgcgcaaaaatcagctgcc
acacaacacaacaaagcgaagcctactcatgagcatcttgtacgaagagcgtcttgatggcgctttacccgatgtcgaccgcacatcggtact
gatggcactgcgtgagcatgtccctggacttgagatcctgcataccgatgaggagatcattccttacgagtgtgacgggttgagcgcgtatcgc
acgcgtccattactggttgttctgcctaagcaaatggaacaggtgacagcgattctggctgtctgccatcgcctgcgtgtaccggtggtgacccgt
ggtgcaggcaccgggctttctggtggcgcgctgccgctggaaaaaggtgtgttgttggtgatggcgcgctttaaagagatcctcgacattaacc
ccgttggtcgccgcgcgcgcgtgcagccaggcgtgcgtaacctggcgatctcccaggccgttgcaccgcataatctctactacgcaccggac
ccttcctcacaaatcgcctgttccattggcggcaatgtggctgaaaatgccggcggcgtccactgcctgaaatatggtctgaccgtacataacct
gctgaaaattgaagtgcaaacgctggacggcgaggcactgacgcttggatcggacgcgctggattcacctggttttgacctgctggcgctgttc
accggatcggaaggtatgctcggcgtgaccaccgaagtgacggtaaaactgctgccgaagccgcccgtggcgcgggttctgttagccagctt
tgactcggtagaaaaagccggacttgcggttggtgacatcatcgccaatggcattatccccggcgggctggagatgatggataacctgtcgat
ccgcgcggcggaagattttattcatgccggttatcccgtcgacgccgaagcgattttgttatgcgagctggacggcgtggagtctgacgtacag
gaagactgcgagcgggttaacgacatcttgttgaaagcgggcgcgactgacgtccgtctggcacaggacgaagcagagcgcgtacgtttct
gggccggtcgcaaaaaatgcgttcccggcggtaggacgtatctccccggattactactgcatggatggcaccatcccgcgtcgcgccctgcctg
gcgtactggaaggcattgcccgtttatcgcagcaatatgatttacgtgttgccaacgtctttcatgccggagatggcaacatgcacccgttaatcct
tttcgatgccaacgaacccggtgaatttgcccgcgcggaagagctgggcgggaagatcctcgaactctgcgttgaagttggcggcagcatca
gtggcgaacatggcatcgggcgagaaaaaatcaatcaaatgtgcgcccagttcaacagcgatgaaatcacgaccttccatgcggtcaagg
cggcgtttgaccccgatggtttgctgaaccctgggaaaaacattcccacgctacaccgctgtgctgaatttggtgccatgcatgtgcatcacggt
catttacctttccctgaactggagcgtttctgatgctacgcgagtgtgattacagccaggcgctgctggagcaggtgaatcaggcgattagcgat
aaaacgccgctggtgattcagggcagcaatagcaaagcctttttaggtcgccctgtcaccgggcaaacgctggatgttcgttgtcatcgcggca
ttgttaattacgacccgaccgagctggtgataaccgcgcgtgtcggaacgccgctggtgacaattgaagcggcgctggaaagcgcggggca
aatgctcccctgtgagccgccgcattatggtgaagaagccacctggggcgggatggtcgcctgcgggctggcggggccgcgtcgcccgtgg
agcggttcggtccgcgattttgtcctcggcacgcgcatcattaccggcgctggaaaacatctgcgttttggtggcgaagtgatgaaaaacgttgc
cggatacgatctctcacggttaatggtcggaagctacggttgtcttggcgtgctcactgaaatctcaatgaaagtgttaccgcgaccgcgcgcct
ccctgagcctgcgtcgggaaatcagcctgcaagaagccatgagtgaaatcgccgagtggcaactccagccattacccattagtggcttatgtt
acttcgacaatgcgttgtggatccgccttgagggcggcgaaggatcggtaaaagcagcgcgtgaactgctgggtggcgaagaggttgccggt
cagttctggcagcaattgcgtgaacaacaactgccgttcttctcgttaccaggtaccttatggcgcatttcattacccagtgatgcgccgatgatgg
atttacccggcgagcaactgatcgactggggcggggcgttacgctggctgaaatcgacagccgaggacaatcaaatccatcgcatcgcccg
caacgctggcggtcatgcgacccgctttagtgccggagatggtggctttgccccgctatcggctcctttattccgctatcaccagcagcttaaaca

gcagctcgacccttgcggcgtgtttaaccccggtcgcatgtacgcggaactttgaggagcaggctatgcaaacccaattaactgaagagatgc
ggcagaacgcgcgcgcgctggaagccgacagcatcctgcgcgcctgtgttcactgcggattttgtaccgcaacctgcccaacctatcagcttc
tgggcgatgaactggacgggccgcgcgggcgcatctatctgattaaacaggtgctggaaggcaacgaagtcacgcttaaaacacaggagc
atctcgatcgctgcctcacttgccgtaattgtgaaaccacctgtccttctggtgtgcgctatcacaatttgctggatatcgggcgtgatattgtcgagc
agaaagtgaaacgcccactgccggagcgaatactgcgcgaaggattgcgccaggtagtgccgcgtccggcggtcttccgtgcgctgacgc
aggtagggctggtgctgcgaccgtttttaccggaacaggtcagagcaaaactgcctgctgaaacggtgaaagctaaaccgcgtccgccgctg
cgccataagcgtcgggttttaatgttggaaggctgcgcccagcctacgctttcgcccaacaccaacgcggcaactgcgcgagtgctggatcgt
ctggggatcagcgtcatgccagctaacgaagcaggctgttgtggcgcggtggactatcatcttaatgcgcaggagaaagggctggcacggg
cgcgcaataatattgatgcctggtggcccgcgattgaagcaggtgccgaggcaattttgcaaaccgccagcggctgcggcgcgtttgtcaaag
agtatgggcagatgctgaaaaacgatgcgttatatgccgataaagcacgtcaggtcagtgaactggcggtcgatttagtcgaacttctgcgcg
aggaaccgctggaaaaactggcaattcgcggcgataaaaagctggccttccactgtccgtgtaccctacaacatgcgcaaaagctgaacgg
cgaagtggaaaaagtgttgcttcgtcttggatttaccttaacggacgttcccgacagccatctgtgctgcggttcagcgggaacatatgcgttaac
gcatcccgatctggcacgccagctgcgggataacaaaatgaatgcgctggaaagcggcaaaccggaaatgatcgtcaccgccaacattgg
ttgccagacgcatctggcgagcgccggtcgtacctctgtgcgtcactggattgaaattgtagaacaagcccttgaaaaggaataacaaaatga
aaactaaagtcattcttagccagcaaatggcgagtgcaattattgccgcaggtcaggaagaggcgcagaaaaataactggtctgtttccattgc
tgttgccgatgacggcggtcatctgctggcgttaagtcgcatggacgattgcgcgccgattgcggcttatatctcccaggagaaagcgcgtacc
gccgcgctggggcgtcgtgaaactaagggctatgaagagatggtgaacaacggacgtaccgcgttcgtgactgcgccgttattaacgtcgct
ggaaggcggcgtaccggttgttgtggatgggcaaattattggtgccgtgggcgtttctggtttaaccggagcacaggatgcgcaggtcgcgaa
agcggcagcagcggtgttggcgaaataagcgaaaacgaggagataaacaatgagtcaaaccataacccagagccgtttacgcattgacg
ccaattttaaacgttttgtggatgaagaagttttaccgggaacagggctggacgctgcggcgttctggcgcaattttgatgagatcgttcatgatct
ggcaccagaaaatcgtcagttgctggcagaacgcgatcgcattcaggcagcgcttgatgagtggcatcgcagcaatccggggccggtaaa
agataaagcggcctataaatctttcctgcgtgaactgggctacctggtgccgcaaccggagcgcgtgacggtggaaaccacgggcattgaca
gcgaaatcaccagccaggcggggccgcagctggtggttccggcaatgaacgcccgctacgcgctgaacgcggcgaacgctcgctggggc
tcactgtacgatgcgttatacggcagcgacatcatcccgcaggaaggggcgatggtcagcggctacgatccgcaacgcggtgagcaggttat
cgcctgggttcggcgtttcctcgatgaatctctaccgctggaaaacggcagctatcaggatgtggtggcgtttaaggtggttgataaacaattacg
catccagttgaaaaatggtaaagaaaccacgttacgtactccagcacagtttgtcggttaccgtggcgatgccgctgcgccgacctgcattttgc
tgaaaaataacggcctgcatattgagctgcaaatcgatgccaatgggcggattggcaaagacgatccggcgcacatcaacgatgttatcgtc
gaagctgctatcagtaccattctcgactgcgaagattcggtcgcggcggttgatgcggaagataaaatcctgctgtaccgcaacctgctgggcc
tgatgcaggggactctgcaagagaaaatggagaaaaacggtcggcaaatcgtgcgtaaactgaatgacgatcgtcattacaccgccgccg
atggctctgaaatttctctgcacggacgctcgctgctgtttatccgcaacgtgggtcatttgatgaccattcctgtgatttgggacagcgaaggcaa
tgaaatcccggaaggcattcttgatggcgtcatgactggcgcgattgccctctatgatttaaaagtgcagaaaaactcgcgcactggcagcgtc
tatattgtgaaaccgaaaatgcacggtccgcaggaagtggcgttcgccaacaaactgtttacccgcattgagacaatgctcggtatggcaccg
aataccctgaaaatgggcattatggatgaagaacgtcggacctcgctgaacttgcgtagctgtatcgctcaggcgcgcaaccgcgtggcgttc
atcaataccggtttcctcgaccgtaccggcgatgaaatgcattcggtgatggaagctggcccgatgctgcgtaaaaatcagatgaaatcgacg
ccttggatcaaagcctacgagcgtaataacgtgctttccggtctgttctgtgggctgcgcggtaaagcgcaaattggtaaaggcatgtgggcaat
gccggacctgatggcagacatgtacagccagaagggcgaccaactgcgtgccggggcaaacacagcctgggttccgtcaccaaccgctg
ctacgctccatgcgctgcactaccaccaaaccaacgtacagagcgtacaagccaacattgcccagaccgagttcaatgctgaatttgaaccg
ctgctggacgatctgctgactattccggttgctgaaaacgctaactggtcggcgcaagagatccaacaagagctggataacaacgtgcaggg
gattctggggtacgtggtgcgctgggtggagcaggggattggttgttcaaaagtgccggatattcacaatgtggcgttgatggaagaccgcgca
acgctgcgtatctccagccagcatatcgccaactggttacgtcacggtattctgaccaaagaacaggtgcaggcgtcgctggagaatatggcg
aaagtggttgatcagcaaaacgctggcgatccggcttatcgtccgatggcggggaatttcgctaactcgtgtgcttttaaagctgccagcgattta
atcttcctcggcgtgaaacagccaaacggctataccgaaccgttattacacgcctggcgtttacgcgaaaaagaaagtcattaatagcgtcaa
aaaagccccggcaagttttattgtcggggctatgatagtaactgcggtagtacatttatcccggaaataaccggagcgattatcagggtttaaca
atattaaatacccgtagcgcattactagcgggcctaaaacaacaatatggacatcttaacgatactctgcgtctgcgtgtgcggaacagaaac
ccttacccgatatacttgttatgccagaggtagcaattgcgttagcggcgtactgttgctgtaaagtataacgcctatattttacagttcagtcctctttt
gcttattacaaccttaagtatatcaagcatataaagataataagagactgaacaatatggttacctggacccaaatgtatatgccgatgggagg
actggggctatccgctctggtcgccctgatcccgataatattcttcttcgttgcactcgcggtattacgtctgaaaggacatgtcgctggagcaata
acccttatattatctatcctgattgcaatattcgcctttaaaatgccgattgatatggcatttgctgctgcgggctatggctttatttatggattatggcca
atagcgtggattattgtcgcggcggtgttcctgtataaattaaccgttgccagcgggcagttcgatattatccgcagctcggttatctccatcaccga
cgatcagcgtttgcaggtgttactgattggtttctcctttggtgcgttgctggaaggagcggctggctttggtgcgccggtggcgattaccggtgcgc

tgctggtgggcctgggcttcaaaccgttatacgcggcggggctgtgtctgattgccaatactgcgccggtggcgtttggtgcgttgggcgtgccg
attctggtcgccggtcaggtaacgggaatcgatccgttccacattggcgcaatggcgggacgtcagttaccgttcctgtcggttcttgtgccgttct
ggctggtagcaatgatggacggctggaaaggggtgaaagagacgtggccagcggcgctggttgctgggggaagcttcgctgtcactcagtt
ctttacctctaactatattggtccggaactgccggatattacttcggcgctggtgagtatcgtctcactcgctttattccttaaagtctggcggccgaa
aaataccgaaacggcaatcagcatgggacaatccgcaggtgcgatggtggtaaataagccatcttctggcggtcccgtgccttcagaatata
gtctggggcaaatcattcgagcgtggtcaccgtttttaatcttaacggtgctggtcaccatctggaccatgaagccgtttaaagcgttatttgctccg
ggcggcgcgttttattcactggtgattaatttccagatccctcatttgcatcaacaagtgttgaaagcggcacccattgtcgcccaaccaacgcca
atggatgcggtgtttaaattcgacccccctctcggctggcggcaccgctatttttattgcggcgattatctctatcttcatcctcggtgtggggatcaag
aaaggtattggcgtctttgccgaaacgctaattagcttgaagtggccgatactgtcgattggcatggtgctggcgttcgccttcgtcaccaactatt
ctggcatgtccaccacgctggcgctggtactggcaggtacaggcgtgatgttcccgttcttctcaccgtttctcggctggctgggcgtattccttacc
ggctcggacacctcctctaacgccctgtttggttcactgcaatcgaccacggcgcagcaaatcaacgtctctgacaccctgctggtggcagca
aacaccagcggcggcgtaactggcaagatgatctccccgcaatctatcgccgtggcctgcgccgcgacgggcatggtgggccgagaatctg
aactgttccgctacaccgtgaagcacagtctgatttttgccagcgttatcggcattatcaccctgctgcaggcgtatgtgtttaccgggatgttagtct
cgtaaatcaataaagccggatgatcgttaatatcatccggcttcatttacattattctgtcttattaattgaaaatcatttctcgctttattatccatgcag
caataatgttttgcgttcagataacctgctaaataattaagagcgtgagaacatctattttttatcacgcaaaacaagtctgtattaattatgttcgctt
gatgagcaaacaccgttctatctgttgcttttgcaatcttaatcgttaaaactaccaacaaatttatatagttacgttgtgaaatgttgttaatttttgtttg
ttgaaatttcgtaaaaatagccagatagacatcgtataggggttattcttataaatttcacctgttttctatcaatgatgtcgttttcttaagaatggagg
aagttattagagaagataaagcataaaactatttctcccagttacgaatttttaacattgttttgtcacttgcgttattaatgaataagaaatttaaata
taagaaatcgcttttagcggctattttgagcgcaaccctgttagccggttgtgatggcggtggttccggatcttcctccgatacgccgcctgtagatt
ctggaacagggtctttgccggaagtgaaacctgatccaacaccaaacccggagccgacgcctgagccaacgccggacccagagcctacg
ccagaaccgatacctgatcctgaaccaacaccagaaccggagccagaacctgttcctacgaaaacgggttatctgaccctgggcggaagc
cagcgggtaactggtgctacctgtaatggtgaatccagcgatggctttacatttaaacctggcgaggacgttacttgcgtggcgggtaacacga
caattgccaccttcaacactcagtcagaagctgcgcgtagcttgcgtgcggttgaaaaagtgtcgtttagccttgaggacgcgcaagaactggc
gggctccgatgacaagaaaagcaatgcggtttcgctggtaacgtccagtaacagctgtccggcgaatacagaacaggtttgtctgacgttctc
ctcggtgatcgagagtaaacgcttcgactcgctgtataagcaaatcgatctggcaccggaagagttcaaaaagctggtcaatgaagaggtgg
aaaacaatgctgcgaccgataaagcgccatccactcatacttcaccggtcgtgcccgtcaccacgccgggaacaaaaccggatctgaacg
cttccttcgtgtcggctaacgcggaacagttttatcagtatcaacccactgaaatcattctctctgaaggtcgactggtcgatagccagggatatgg
tgttgctggcgtcaactactacaccaattcaggccgtggcgtgacaggggaaaatggtgaattttcctttagctggggcgaaaccatctcctttgg
tatcgatacctttgaactgggttcagtgcgcggcaataagtcgaccattgcgctgactgaactgggtgatgaagttcgcggggcgaatattgatc
agcttattcatcgctattcgacgaccgggcaaaataatacccgtgttgttccggacgatgtacgcaaggtctttgccgaatatcccaacgtgatca
acgagattatcaatctctcgttatccaacggtgcgacgctgggggaaggtgagcaagtcgttaatctgcctaacgaatttattgagcagtttaata
cgggtcaggccaaagagatcgataccgcgatttgtgcgaaaaccgatggttgtaacgaggctcgctggttctcgctgacgacgcgcaatgtta
atgacggccagattcagggcgttatcaacaagctgtggggcgtggatacgaactacaaatctgtcagcaagttccatgtattccatgactccac
caacttctatggcagcacgggtaatgcgcgcggtcaggcggtggtgaatatctccaacgcggccttcccgattctgatggcgcgtaatgataaa
aactactggctggccttcggcgaaaaacgcgcctgggataaaaacgagctggcgtacattacggaagcgccttctcttgttgagccggaaaa
cgttacgcgcgataccgccacctttaacctgccgtttatttcgctggggcaagtcggtgagggcaaactgatggttatcggtaacccacactaca
acagcattttgcgttgcccgaacggttacagctggaacgggggcgttaataaagatgggcagtgtacgctcaacagcgacccggatgacatg
aagaacttcatggagaacgtgctgcgctatctgtccgacgataaatggaagccggacgcgaaagccagcatgaccgtaggcaccaacctg
gatactgtctattttaaacgtcatggtcaggttacaggaaacagcgctgcgttcgacttccatccggattttgcgggcatctctgttgagcatttaagt
agctatggcgatctcgatccgcaggaaatgccgctgctgatccttaacggctttgaatatgtgactcaggtgggtaacgatccttatgcaatcccg
ctgcgtgcagataccagcaaaccgaagctgactcagcaggatgtgaccgatctgatcgcctatctgaacaaaggtggatcggtgctgatcat
ggaaaacgtgatgagcaatcttaaggaagagagcgcgtctggttttgtgcgtctgttggatgccgcaggtctgtcgatggcactgaacaagtcg
gtagtaaataacgatccgcaagggtatccgaaccgcgttcgtcagcagcgcgcaacgggcatttgggtctatgaacgttatcctgccgtagat
ggtgcgctgccgtacaccatcgatagtaagacaggggaagttaagtggaaatatcaggtagaaaacaaacctgatgacaaaccgaagctg
gaagttgccagctggctggaagatgtagatggcaaacaggaaacgcgttatgcctttattgatgaggccgatcataaaacagaggattctctg
aaggctgcgaaggagaaaattttcgccgcgttcccggggctgaaagagtgtactaatccggcatatcactatgaggtcaactgcctggaatat
cgtcctggcacgggggttccggttactggtggcatgtatgttccacagtatacgcaactaagccttaacgccgacactgcaaaagcgatggtgc
aggctgcggatttaggcaccaacattcagcgtctgtatcagcatgagctctacttccggaccaatggtcgcaaaggtgagcgtctgagcagcgt
cgatctggaacgtctgtaccagaacatgtcggtctggctgtggaacgatacgagctatcgttatgaagaaggcaaaaatgacgagctgggctt
taaaacgttcaccgagttcctgaactgctacgccaatgatgcctatgcaggcggcaccaagtgttctgcagatctgaaaaaatcgctggtcgat

aacaacatgatctacggtgacggtagcagcaaagcgggcatgatgaacccgagctacccgctcaactatatggaaaaaccgctgacacgc
ctgatgctgggccgttcctggtgggatctgaacatcaaagttgatgtcgagaagtatccgggagcagtatcggaagagggacagaacgttact
gaaaccatcagcctgtactcgaatccgaccaaatggtttgcaggtaacatgcagtcaactggcctgtgggcaccggctcagaaagaggtcac
cattaagtccaatgcgaacgttcctgtgaccgtcaccgtggcgctggctgacgacctgaccggacgtgagaagcatgaagttgcgctgaacc
gtccgccaagagtgactaaaacgtactctctggacgctagcggtacggtgaagttcaaggtgccttacggtggcctgatttatatcaagggcaa
tagctctaccaatgaatctgccagcttcacctttactggcgtggtaaaagcaccgttctataaagacggcgcatggaaaaacgatctgaactca
ccggctccgctgggtgagctggaatcagacgctttcgtctataccacaccgaagaagaacctgaatgccagcaattacactggcggactgga
gcaattcgctaacgatctggatacctttgccagctcgatgaatgacttctacggccgtgatagcgaagacggtaagcaccggatgtttacctata
aaaacttgccgggccacaaacatcgtttcaccaacgatgtgcagatctccatcggtgatgcgcattcgggttatccggtaatgaacagcagctt
ctcgccgaacagcaccacgctgccgacgacgccgctgaacgactggctgatctggcatgaagtcggtcataacgccgcagaaacgccgtt
gactgtaccgggtgcaactgaagtcgctaacaacgtgctggcgctgtacatgcaggatcgttatctcggcaagatgaaccgtgtcgctgacga
tattaccgtcgcaccggaatatctggaggagagcaacaaccaggcatgggcacgcggcggtgcgggtgaccgtctgctgatgtacgcacag
ctgaaggaatgggcagagaaaaactttgatatcaagaaatggtatccagatggcactcctctgccagagttttacagcgagcgtgaagggat
gaaaggctggaacctgttccagttgatgcatcgtaaagcacgcggcgatgaggtcagcaatgacaagtttggcggcaagaattactgtgctg
aatccaacggtaacgcagcggacacgctgatgctgtgtgcctcctgggtcgcccagacggatctttcggagttctttaagaaatggaatccggg
cgcgaatgcttaccagctgccgggggcgagcgagatgagcttcgagggcggtgtgagccagtcggcttacaacacgctcgcgtcactcgat
ctgccgaaaccggaacagggaccggaaaccattaatcaggttaccgagcataagatgtctgccgagtaagcccgtaggccggataaggc
gctcgcgccgcatccggcatgaaataaggcgcaccgggttaacacatttgcccgatgcgcctgcttatcgggcctacagacggggaaagcc
gcgattatttgcgctggcagacacccgtaggacggataaggcgttttacgccgcatccagcaagttacaacaaacaacctttaaccatgcttttt
gatgtttttcagcaataccccacggcgatgcccgtcctggcaaccgtcggaggattgatcatcggcagtttttttgaatgtggtgatttggcgttaccc
aatcatgctgcgccaacaaatggcggagtttcacggtgaaatgtcgagtgcgcagtcaaaaataagccttgcgctaccgcgttcgcactgccc
acattgtcagcagaccatccggatacgtgacaatattccgctgttctcctggctgatgctcaaagggcgctgccgcgactgtcaggcgaaaatc
agcaagcgttatccgctggtggagttattgacagcacttgctttttttgctggcgagtctggtctggccagaaagtggatgggggctggcggtgatg
atattatccgcctggctgattgccgcgagtgtcatcgacctcgatcaccaatggctgcccgatgtttttactcagggcgtattgtggacaggactga
ttgcggcatgggcgcagcagagtccgctcacgctacaagatgcagtcaccggcgtcctggtgggatttatcactttttactctctgcgttggatag
ccggaatagttctgcgtaaagaagcattaggcatgggcgatgtattacttttcgccgcgttaggtggctgggtgggggcattgtcgctgcccaatg
ttgctttaatcgcctcatgctgcggcctgatatatgccgttattacaaaaagaggatcaaccacactgccttttggaccgtgtttaagtctgggcggt
atagcaacactttatctacaggcattgttttaatgataaccacgtcattatcaaagtgacattttaactcttattaataaccttagagattatttaccatg
tcgataaaacaaatgccagggagggtattaatatcgctattgttgagcgttacaggattattaagtggctgtgccagccataatgaaaatgccag
tttactggcgaaaaaacaggcgcaaaatatcagccaaaacctgccgattaaatctgcgggatataccttagtgctggcgcaaagtagcggca
caacggtaaaaatgaccattatcagcgaagcgggtacacaaaccacgcagacgcctgacgcctttttaaccagctatcaacgacaaatgtg
cgctgacccgacggtgaaattaatgatcactgagggaattaattacagcataacgattaatgatacacgtacaggtaaccagtatcagcgga
aactggatcgtaccacctgtggaatagtcaaagcataacgtcgggtagatataaattggcgcgggttgtttttcgtgacgcacgaatttatctcatt
caatggctgacaaaaattcgtcacactcttaaccagagacaatctcttaatacagacaaagagcatctgcgaaaaattgcacgcgggatgttc
tggctgatgctgcttattatttctgcaaaaatggcgcattcactctggcgctatatctccttttctgcggaatatacggcggtttctcaaccggtgaata
aaccgagccgtgtggatgcaaaaacgttcgataaaaatgacgtgcaattaatcagccagcaaaactggtttggcaaatatcagcctgtcgcc
gcgcaggtaaaacaacccgaacctgtgcctgtggcagaaacgcgtcttaatgtggtgctgcgtgggatcgcctttggtgccagacccggcgc
ggttattgaagaaggcggtaaacagcaggtctatttgcagggtgaaacgcttggctctcacaacgcggtgattgaggaaatcaaccgcgacc
atgtgatgctgcgttatcagggaaaaatagagcgtctgagtctggcagaagaggaacgttccactgttgctgtgaccaacaaaaaagccgtc
agtgacgaagcaaagcaagctgttgctgagcctgctgtcagtgtgccagttgagatcccggctgccgtgcgtcaggcactggcgaaagatcc
gcagaaaatttttaactatatccagcttacgcctgtgcgtaaggaggggattgtcggttatgcagcgaagccgggggcagatcgttctctgttcga
tgccagcggtttcaaggaaggcgatatcgccattgcgctaaatcagcaggatttcactgatccacgagcaatgattgctctgatgcggcagtta
ccttcaatggattccattcaacttacggttttacgcaagggtgcgcgccacgacatttccatcgcgctgcgctaccgactattctgcccgccttact
ggaagaagagctgaaccaggatatcgatgaactgcatttcgccccgttgaatatcgaccagcaactggcaaccgtagcagtgattcaccaac
agcatatgcgcaacattgcgcagtggttgcaggaaaacggcatcacccgcgctaccgtcgcgccggactggatgtccattccttgtgggtttat
ggcttgcgatgcgcaacgggttatctgccgcatcgatgaatgccggggatggagcgccgggctggcgctggctccggtcatgttccgcgccc
agctcaatgagcaggatttaccgctttcgctaaccgtagtcggcattgcaccggaaaagctatcagcatgggctggcgcagacgctgaacgc
ctgaccgttacagctctgcccgccattaccacttatggcgaaccggaagggaacctgctgacagggccgtggcagcctcgcgtcagctaccg
aaaacagtgggcgcgctggcgggtgatgattctgccgatattgctgattctggttgcgctggcagtggagcggggcgtgacgttatggagcgtc
agcgaacaggtggcgcaaagccgcacccaggcggaggaacagttcttaacgttgttcccggagcagaagcggattgtgaatttacgctctc

aggtgacgatggcgctgaaaaaatatcgcccacaggccgacgatacccggctgctcgccgagttgtcagcgatagccagcaccctgaaat
cagcgtcactttccgacatcgaaatgcgtggttttacctttgatcaaaaacgccagatacttcacctccagctacgggccgcgaactttgccagttt
cgacaaactgcgtagtgtactggcaaccgattatgttgtgcaacaggacgcgttacagaaagagggtgatgcggtttccggcggcgtaacgtt
gcggaggaaataacatgttacgcgataaatttattcactattttcagcaatggcgtgaacgccagttaagccgtggcgaacactggctggcaca
acacctggcggggcgttctccgcgtgaaaagggcatgttactggcagcggtggtgttcctgtttagcgtcggatattacgtcctcatctggcagcc
gttgagcgaacggattgagcaacaggagacaatattgcagcagctggtggcgatgaacacccgactgaagaacgccgcgccggatattatt
gcagcgcgaaaatctgccacaacaacgcctgcgcaggtatcgcgggtcatcagtgacagtgcttccgcgcactcggtggtcatcaggcgga
tagccgatcgtggggagaatattcaggtctggatagaacctgtggtgtttaatgacctgctgaaatggttaaacgcactggatgaaaaatatgcg
ctgcgggtgacacaaattgatgtcagtgctgctgagaagcctgggatggtgaatgtgcagcggctggagtttggacgggggtaaagtatcagg
agattaatatcaataaaattactctcgttaacgtggcgagagtaattcccccagactttataagctaattttttttcgtgaaggttattgtcagatccaa
aagcaaaaacccgccttgtgggcgggttcttaagaattagtggtgcccggactcggaatcgaaccaaggacacggggattttcaatcccctgc
tctaccgactgagctatccgggcaacggggcgcattaaacctgattcgcctcgtctcgtcaatcaaatttcttcaatttactgcagactgcacaatc
tatcatcactttgccgatattgcacgcattgtcaggcaaaccaggcggtccaggcggcggaaagcggcattgccagaaggagtgctggaag
catgttgaccaccgggaacattttaatgccacagatgcgtaatccggttgccagcagcaataaaccgcctacggcgctgaagtctgccatcat
cgacggtgtggtcagcggtaaaatcagcgcggcagcccacgccagcgttaactggatgatcagtaatgggatactaatcaccgataccgca
atacccagcgagcaggcgaagatcatcgccgtaaagaaatcaagaaatgacttggcgattaaaatactcggatcgccggtcatcccttcgttc
atcgccccgaagatcccggtgccgctggcgcaaaacaggacaataatcgcgacataattctgaataaaagattcatgcgctggcttcttacgt
gagtgacgaaacagattttgtgctttggcgactgctgtattgacacctttctccagcaaacaaatttcgccaattagagcgccaagtagcgtagct
aaaaccatcgctggaaggttggcacatttcaccaccagtaaaataccaatccccagcgatgccagaccaaaaattgatgtcatggagacgc
ggatacgttccggtaagcgttggctgagcagtgcgccgagaacgccacccagtaagactgcactagcgttgataaaagggccgatgaccac
aagagttcctgttagctgtattgtttgattttcttattatgaccgtttggtgccgtcgatggcttgtttgttcgcaaagtcctggcttgcacgctttagcgaa
aggtgccatgattgcgcgaattttctcctctctgtacggagtttgcccgatgcacgccatctccttacattctctcgcttatcgccgtttcgcgcgaaa
cgtttccctttctatgttactgctcatgcggtgaagttaacgcacgctcactgcaggacaacagtaaaatcagagcgtttctgcttttactgatgtctg
gcggtcggagctggtgaccagtttgacccatatctcatggggcagggttttccaccttgtccggtattcttacttccccgaaacgggtttgcgcttat
gaaatcaatgaatattgccgccagtagtgaactggtatcccgactttcttctcatcgtcgcgtggtggcgttgggagatactgattttacggacgtc
gcggcagtcgtcattaccgctgcggatagtcgcagtggcattcttgcgttgcttaagcgcaccggttttcatctaccggtgtttttgtattccgaacat
gctgttgaattacctgcgggcgttacggcggtaatcaacggcaacgagcagcagtggctggagctggaatccgcagcctgtcagtatgaaga
gaatttgctgccaccgttttatgacacgctgacgcagtacgttgagatgggcaacagcacctttgcttgccctggacatcaacatggtgcgtttttta
aaaagcatcctgccggacgccatttttacgatttctttggtgagaacgtctttcgcgccgatatgtgtaacgctgacgtaaaattgggcgatctgctt
attcatgaaggatcggcgaaagatgcgcagaaattcgcagccaaagtctttcatgccgataaaacctattttgtgctgaacggcacatcggca
gcgaataaagtggtgacgaatgcgctgttaacgcgtggcgatctggtgctcttcgaccgtaacaaccataagtcgaatcatcacggcgcgctg
attcaggcgggggcgacgccggtctatctggaagcttcacgcaacccgtttggtttcattggcggtattgatgcgcactgttttaatgaagagtatc
tgcgccagcaaattcgcgacgttgcgccagaaaaagccgacctgccgcgcccgtatcgcctggcgattattcagctgggaacctatgacggc
actgtctataacgcccgtcaggtgatcgataccgttgggcatctgtgtgattacattctgtttgattccgcgtgggtcggttatgaacaatttatcccg
atgatggcggatagctcgccgctgctgttagaacttaacgaaaacgatccggggatctttgtgactcagtcggtgcacaaacagcaggcggg
attctcacagacgtcgcagatccataaaaaagataaccatatccgcggacaggcgcgtttttgcccgcataagcggttgaataacgcctttatg
ctccatgcttctaccagccctttctatccgctgtttgctgcactggatgttaacgccaaaattcatgaaggggagagtgggcgtcggctgtgggctg
agtgtgttgagatagggattgaagcgcgcaaggctattcttgcgcgctgtaagctgttccgcccgtttatcccgcccgttgttgatggcaaattgtg
gcaggattatccgacatcagtgttagccagcgaccgccgtttttcagttttgagccgggggcgaagtggcacggctttgaaggatatgccgcg
gatcagtattttgttgatccgtgcaagctgttactcactacaccaggtatcgatgccgaaaccggcgaatatagcgactttggcgttccggcgacg
attctggcgcactatctgcgtgagaacggcattgtgccggagaagtgcgatctcaactccattctgtttttattaactccggcggaaagccacgag
aagctggcacaactggtggcgatgctggcgcaatttgaacagcatattgaggatgactcgccgctggttgaggtgttgccgagcgtttataaca
agtatccggtgcgctatcgcgactacaccctgcgccagttgtgtcaggagatgcacgatctgtatgtcagtttcgacgtcaaagacctacaaaa
agcgatgttccgccagcagagtttcccgtcagtggtgatgaacccccaggatgcgcatagcgcttatattcgcggtgacgtggagttggtgcgg
attcgtgatgccgaagggcgaattgcggcagaaggggcgttgccttatccacctggcgtgctttgcgtggtacccggggaagtctggggtggg
gcggttcaacgttatttccttgcactggaagaaggggtgaatttgttgccgggattttcgccggagctgcaaggtgtttatagcgaaaccgatgcg
gatggcgtgaaacggttgtacggttatgtgttgaagtaagaataaaaaaaacgggtcaccttctggcgacccgtttttctttgcgtaattagtggct
aaccgtctgtgtgcctgtcgggacacgaacgtgtttatatttgaacatcgccatgaacgcgaaggccagaaccacggagtaaccagcgaaa
atcaaccatacggtctgccagtcggtaatgccgttttgggtgtacatctcaacaactttaccgctcacgatgccgccgaggatacagccgaagc
cgttagtcatcatcaggaacatcccttgtgcactggcgcgaattgccgggctaacttctttttcgacaaacaccgaaccagagatgttgaagaag

tcgaatgcgcaaccgtaaacgatcatcgacagtaccagcagtacagtaccgaacggagtcgggtcgccgtaagcaaacagcgcaaaacg
caggatccacgccacaatactgatcatcattacgttcttaataccgtagcggcttaagaagaacgggatggtcagaatgaacagggtttcaga
gatctgcgaaatcgacatgatgattgacgcatgctgcacaataaagctgctggcaaacatcggatctttgtcgaagctgtgcaggaaggtatta
ccgaacatgttggtaatctgcagttccgcgcccagcagcattgagaagataaagaagattgccatacgcttgtttttaaacagcgcgaatgcat
cgaggcccagcagggttgtccagctctgattcgcttgctgtttagcaaccggaatatgcggcagagtcagggtaaacagaaccagaatggcg
gaaagtgctgcgccaatatacagctgcatgtggcttaattcgaagccagacaggctcaccacccacattgccatgataaagccgatggtgcc
ccagatacggattggcgggaagtcagtaacgatatccatcccggcattttgcaggcgatagtaagagatggtgttgattaacccaagcgttggc
atataagcaaacgagttaatcaatatcacaaggaacatcgcttccggtgtcgtgacctgtgccgccatgaacagcgtgatagcgccaatggtg
tggcaaatggcatatacccatttcgcacttaaccatttgtcggccacaatccccagcagcgcaggcataaagaccgctgcgatacccagtgag
ctataaactgcgccaatagaagcaccgtcaaacttcagggtaacaaacatataggagccgagggtcgtcagccaacttccccacagacag
aactgcagaaaagagaggattttcagctgcagcttaagattcatgttaatttcctcacatcgtgatgcggatgagtgtttgaaaaagcacatttga
ccgtgcgagatgatgggattctatcaacttcgcggactggtttttgttacctgtggcaaataattgcaaacattcccaatttgcaaaacgaaattgt
gatgtggataacatttttgcccctgagcatcgtcaggggcggttaatggaattatcggcggcggtaggatttttgcgcggtattcactttataaagat
aacggcgagattccgcagagggatggcgggtcgtcagcgtctgataaacatcgcccggcgtcatggtgttaataatattggcagcctgaatctt
atcattcgaaaagactcgcagcacgctgcctgcgccgccgttataggcggtgatgacggcataacgccgcgatgttgggttatcaattccgcc
gagataaacattgttcagcatcgccagatacgcggtgccggtatcaatattgctggcaggatcaaacaagaaactgcggctcggcgtgccgg
atttcccctgcgagcggaacacatctttcccggcagtatgttgtaccacctgcattaatcccagcgcatcggaacggctgaccgcatacgggtta
aaggaagattcggtctgcataattgccagaatcagcgactcatcaacgccatatttccgtgacgcctggcggaccatgccgagatatttgtgcg
cacgtttatcaaggtggttcggcaccatgttaatggtgacgctgtagatgatacgcagcccgttgctgcggctcttcagacggtttttcagcagata
atccgcgaagttgcttgcgcgaccttcccagcgaatcggctgcccggtgttgtccaccacctgaccgtaaaggaaaggttctttcgaaatcgta
atatcatcaacgtcggaatagagatcgaccgaactcggatcgtcacccatcaataacgttttgataattgcccggcgcaaatgcgcggcaggtt
ctgtcccggcgatggtttcgatagtaatcgtaccgtcatcgaagttgatgtggctgcgggtctgatattgatcggtgtatttcacgtagtccttaggac
cagcgatcaccacctctttgaagccccagatgttctcaatattgtgggcaaattgccccatcagaatatcaaaaccgttggtatctttgacccagg
cttcgttataggtatcgcctttttggtcgtcgaacaggagatgagcaacggcgcaatcaaagccagcgcgagatatttttcatcattccgggtgc
gtgttgtgtttgtttttatgcaagttgccggaggcgtgctccggcactgtttttatttttatcttccggcgtatagccctcgatatgcacctctttaccctcg
aacaggaagttgaccatctcctgctcaagcagcttgcggtgctcggcattcatcatgttgagtttcttttcattaatcagcatggtttgcttgtgctgcc
actgcgcccaggcttctttggagatctcgttatagatgcgttttcccagctcgccggggtacagctgaaaatcctgaccttctgcttcacgttgcag
gaaagtacaaaaaatcgttctgctcataaatcatcctctttatcgactcacgcgctaaaccggcgcgccagtgcgtaactgctgtaacaaacgc
tccacgggagccgctaggccaactgacggcggttgcgctaagttataccagagcgcattgccttcatccatgcagccggtgaatgacgacac
gggaagccacataggcacaatatctaagtggaaatggctgaaggtatgccgaaacgcggtcagttgcgtcaggttatcggcagcaatctgcc
gttgcgccagccactgccgcaaactttcttcgtcggcaaactgcgggaaacagtataaaccgccccacaatccgctcggcggacgctgcgcc
agcaatacttcatcttcgtgctgtaatagcaaaaagtagccggtgcgctccggcagcgtctgtttcggtttttgcccggataaagcgcccagctat
tgttggcggcggcaatacatccgttttgtagcggacagagcgaacatttcggtttcgagcgcgtacaaatcatcgcacccaaatccatcatcgc
ctgattaaaccgttccacgccaaccgcgggcgtcacctgctcgctcaaactccataatttattctcgacctctttttcccaggccagccgcttaca
gcatagcagcgcgccagcacgcgtttgacgttaccgtcgagaatcggaaagtgcttacccagagaaagcgagagaatcgcgcctgcggtg
gaacgcccgacgcccggcagtgctgcaacttcctcaaaggtttccgggaatttaccgccgtgtaaggtcgccacttgttgtgccgctttatgcag
attgcgcgcgcgggcgtaatagccaagcccggtccacaagtggagaacttcgtcgagcggcgcattggcgagatcggtcaccgtcgggaa
gcgcgccataaagcgttcaaaataggggataacggtcgcaacctgagtttgttgcaacatcacttctgagagccatactttgtagggcgtcttgt
caatttgccagggcagagtttttcgcccgtatttatcgtaccagtccagaacctgggctgaaaattgcgacgcttgcatggtcaccgaattcactgt
tgttgggggcaagattgcagcacagcgacggcagggtgtaaaccggaactttccgcagccacggccttatcatgcttgcatccggcaactaa
ctttggataatgcccgttttcagaacactttcacaagcgactaaacctttatgaaaaacgacgtcatttcaccggaatttgatgaaaacggccgcc
cactgcgccgtatccgtagttttgtgcgccgccaggggcgactgaccaaaggccaggaacatgcgctggaaaactactggccggtgatggg
cgttgagttcagcgaagatatgctggatttccccgcgctttttggccgtgaagcgccggtgacgcttgagattggttttggcatgggggcgtcgctg
gtggcaatggctaaagatcgccctgagcaggacttcctcggcattgaagtgcattcaccgggcgttggtgcgtgcctggcttctgcgcatgaag
aaggtttaagcaacctgcgcgtgatgtgtcacgatgcggttgaagtgctgcataaaatgattcctgacaattcattgcgcatggtgcagctctttttc
cctgacccgtggcacaaagcgcgccataataaacgccgtatcgttcaggtgccgtttgccgaactggtaaaaagcaaactgcagctggggg
gcgtattccatatggcgaccgactgggaaccttatgcggaacatatgcttgaagtgatgtcttctattgacggttataaaaacctgtcagagagca
atgattacgtaccgcgtccggcatcacgtccggtgacgaaatttgaacaacgtggtcatcgtcttggtcacggagtatgggacttaatgttcgag
agggtgaaataatggcaaagaaccgtagccgtcgtctgcgtaaaaaaatgcacatcgacgaattccaggaattaggattttcggtggcatgg
cgattcccggaaggtacatcggaagaacagattgataaaaccgttgatgattttattaacgaggttatcgaaccgaacaaactggcctttgacg

gcagcggttatctggcctgggaaggtctgatctgcatgcaggaaatcggcaaatgcaccgaagaacatcaggcgattgtgcgtaagtggctg
gaagagcgcaaactggatgaggtacgcaccagcgaacttttcgacgtttggtgggactaagaaagcatacgggcgatgacaaatgcaaaa
ctgcctgatgcgctacgcttatcaggcctggaaagatgcacgatcgagtaggcgggataaggtgtttacgccgcatccggcatggaaaacgc
gtactttgttatcaatctggggccagcaaatgctggcctgatttttcttgagggaagactatgatgcgcaaaatgctgctggcggcagcactttca
gtgacggcaatgaccgctcacgccgactaccagtgcagcgtcacgccgcgtgacgatgtgattgtcagcccgcaaaccgtgcaggtgaag
ggcgaaaacggcaatctggtgatcacgccagacggcaacgtgatgtataacggtaagcaatattccctgaatgccgcccagcgcgagcag
gcgaaggattatcaggctgaactacgcagcacgctgccgtggattgatgaaggcgcgaaaagccgcgtcgagaaagcccgtattgctctgg
ataaaattatcgttcaggagatgggcgaaagcagcaaaatgcgcagccgtctgaccaaacttgatgcgcagctgaaagagcagatgaacc
gcattattgaaacgcgcagcgatggcctgacgtttcactataaagccattgatcaggttcgcgccgaaggccagcaattagtgaatcaggcaa
tgggtggaattttacaggacagcattaatgaaatgggcgcgaaagcggtgctgaaaagcggcggtaacccattacagaatgtgctgggaag
cctggggggctgcaatcctcaatccaaaccgagtggaaaaagcaggaaaaagatttccagcagtttggcaaagatgtttgtagccgcgttgt
gactctggaagatagccgcaaagccctggtcgggaatttaaaataatcctctattttaagacggcataatactttttatgccgtttaattcttcgttttg
ttacctgcctctaactttgtagatctccaaaatatattcacgttgtaaattgtttaacgtcaaatttcccatacagagctaagggataatgcgtagcgtt
cacgtaactggaggaatgaaatggagtttttcaaaaagacggcacttgccgcactggttatgggttttagtggtgcagcattggcattacccaat
atcaccattttagcaaccggcgggaccattgccggtggtggtgactccgcaaccaaatctaactacacagtgggtaaagttggcgtagaaaat
ctggttaatgcggtgccgcaactaaaagacattgcgaacgttaaaggcgagcaggtagtgaatatcggctcccaggacatgaacgataatgt
ctggctgacactggcgaaaaaaattaacaccgactgcgataagaccgacggcttcgtcattacccacggtaccgacacgatggaagaaact
gcttacttcctcgacctgacggtgaaatgcgacaaaccggtggtgatggtcggcgcaatgcgtccgtccacgtctatgagcgcagacggtcca
ttcaacctgtataacgcggtagtgaccgcagctgataaagcctccgccaaccgtggcgtgctggtagtgatgaatgacaccgtgcttgatggcc
gtgacgtcaccaaaaccaacaccaccgacgtagcgaccttcaagtctgttaactacggtcctctgggttacattcacaacggtaagattgacta
ccagcgtaccccggcacgtaagcataccagcgacacgccattcgatgtctctaagctgaatgaactgccgaaagtcggcattgtttataacta
cgctaacgcatccgatcttccggctaaagcactggtagatgcgggctatgatggcatcgttagcgctggtgtgggtaacggcaacctgtataaa
tctgtgttcgacacgctggcgaccgccgcgaaaaccggtactgcagtcgtgcgttcttcccgcgtaccgacgggcgctaccactcaggatgcc
gaagtggatgatgcgaaatacggcttcgtcgcctctggcacgctgaacccgcaaaaagcgcgcgttctgctgcaactggctctgacgcaaac
caaagatccgcagcagatccagcagatcttcaatcagtactaatcgcctcgccccggtatcgtgccggggctttttcacttcagactcacgtcca
ttgccaattttaattaccctaatgataatcaccggaataaattattccgcgtgaggtttttcgggtgaaaaagcaatggattgttggcacggcactg
cttatgttgatgactggtaatgcctgggcagatggcgaaccgccaactgaaaatatcttaaaagatcaattcaaaaagcagtatcacggcattct
caagcttgatgctatcaccttaaaaaatcttgatgctaagggtaatcaggccacctggtcagcggaaggcgatgtctcttccagtgacgatctct
atacctgggtcggtcagttggcagattacgaactgctcgaacagacctggacgaaagataaaccggtaaaattctcggcgatgttaaccagta
aaggaacgcccgcgtctggctggtcggtgaacttttactcttttcaggcggcagccagcgatcgtgggcgggtggtcgacgatatcaaaacga
ataataaatatctgatcgtgaatagcgaagatttcaattatcgctttagtcagcttgaatctgcgttgaatacccagaagaactcgattcctgcgctg
gaaaaggaagtgaaagcgcttgataagcaaatggtggcggcgcagaaagcagcggatgcatattggggaaaagatgcaaacggtaagc
aaatgacccgcgaagacgcctttaaaaaaattcaccaacagcgtgatgagtttaataaacagaacgatagcgaggcgttcgcggttaaatat
gacaaagaggtttatcaaccggcgattgcggcatgtcataaacagagtgaagagtgttatgaagtgccgattcagcagaaacgagatttcgat
atcaacgaacaacggcgacagacttttctgcaatcacaaaaacttagccgtaaattgcaggatgactgggtaacgctcgaaaaagggcaat
atccgttaacgatgaaagtttcagaaataaacagtaaaaaagtcgcgattttgatgaaaattgacgatatcaaccaagctaatgaacgctgga
aaaaagataccgaacaactgcggcgcaatggggtgattaagtaagtttgatatagtgcacgatttgtagcaggatgcggcgttgaccccgcat
ccggcaatcaacgtctgatgcgacgcttgcgcgtcttatcagacctacaaccgctgccacatgtcggctggataaggcgttcacgccgcatcc
ggcaatacaagtttactcagccagaaaaagctccagcagcgaatttaaaaacagcttcccatgttccgttatctgccagtaatccgcacattcg
gtgagataaccctgggcaatcgcctcgtctaactgtgggcgaatcacatcttcgcaaagcccggtatacgcaataaactccacgcgcggcgc
ggcctccagcagacggaagcgattcataaagaactcaaacggcttatctgtggcttcgacatcacgctggctttccagataccttccttgcataa
aaccacgcggatgacgcgttttggtggtacgcagaatgcgcccatccgggaaggtcactttgccgtgtgcgccgcagccaataccgatgtagt
caccaaagcgccagtagttgagattgtgctggcactgataaccgggtttggcgtaagcggaagtttcatattgctgataacccgctgcggttaat
aactgatgcccctgttcgaatatatcccacaacgcgtcatcgtccggcagcaccggtggtcgcgaaccaaacagcgtattgggttcgatggtc
agttgataccaggaaagatgcggcggattcagttcaatggcctggcgtagatcgccaagcgcctcttccagtgattgatccggcagcccatgc
atcaaatcaaggttaaagctacgtaaccctaaaccgctcgccagcttcgccgcgcgtttcgcttcttgcgggccatgaatacgcccaagtcgttt
cagcttttcttcgctaaaactctgcacaccaatagagatgcggttcacaccagcacgctgataatcgacaaagcgatcggcttctaccgtgcca
gggttcgcttccatagtaatttctgcatccgctgccagcggcaaacgcgcacgcacgccgtccagcagcgtttgcatcgccgggccggaaag
caggctcggcgtaccaccgccaataaagattgtctttacttcacggccctgagcgtaagccacatcgttgtccagatcgttaagcagatgctga
acataatcgtcgtgcggcacttctcctttcaacgcgtgagagttgaaatcgcagtacgggcatttctgcacgcaccacgggatgtgaatgtaga

gactcagcggaggtaatttaaccattacgtaaagcgtccagcagcagtttcaacgcctgaccacggtgggaaatggcgctcttttcttcgcgggt
cagttcggcggcggttttcccttcggaaggtacgaagaagattggatcataaccaaagccaccagtgcccgctggttcacgagtaatcacgcc
cggccagctaccgtggcataccagcggagtgggatcttccgcgtgacgcagatataccagcacgcagtggaaacgcgcctgacgttggtcg
tccggtacgtctttcattgtttccagcagtttttgcagattcttttgatcggtcgcgtcttcaccggaataacgcgcggagtaaatgcccggcgcgcc
gccaagcacatctaccgccagaccagagtcgtcggcaattgccggtaaagcggtcacttttgccgcatggcgcgctttcagaatcgcgttttcg
ataaaggtcaggccggtttcttcagcggaatcaacgccgaggtctgtttgggccacgatatcaagaccgaagtcgctaagcagcgacgccag
ctcacgcactttaccgacattgccggttgcgaggacaactttttgcataggatacctaattaattaacgccgcgatttctggcgggatttgttgcgg
attaatgattttaatttgtttgtggcggccaagttcgcctttttcaatcaccacctggcttttggcaacccggaattgcttaccgagaaacttcaccaga
tgactgttggcctggccgtcaaccggcggcgcggtaatggcgactttaacttcgtcgccatgtaaaccgacaatagaatcacggctggctttcg
gctgaatatagagccgtaaaaccagaccgtcatcattaactgttacggcattcataacgccatccacagccctggcagcagcatatttccggtt
gcctgtaatacttctgcgacacccatattgatgacatacagcagcagaacgaggatcatcggcgagaaatcaatcccacccattgccggtag
caggcggcgaatcgggcgcagcagcggatcggccagctgaatcagcacgtattcaatcgggctacgcccctggcttacccagctcataatc
gccatcaccagcaggacccagaaaatcagcaggccgatggttttcagcagaatcagtaaaccggcaatccagatgattggcaggaaggtc
accactttaaacagcacgatggctttgataaaactgagaatataggcaaccagcagcgaggcgctgtcaattggccccattgccggaataac
gcggcgcagtggcccgataattggctgcgttaccttcactacaaactgtgagaaggggttgtaaaaatcacaatgagcccactgcatccagat
gcgtaataacagcaccatggtatacagctcaatgaccgttgaaagcaggaaagtcaacgtattcatggcgttcctttaattccttatttttagagta
atcacgcgcaccaaaaattgcagtgccgatacgaaccatcgtgctacctgccgcaatggcggcttccatatcgtccgacattcccagagaga
gcgtgtcgatatgcgggtagcgcgttttcagtccggcaaatgctacagccatttggcgtgcaacttcaaactgccttacatattctgactcaggcg
cagggattgccatcaacccgcgcagacgtaaacgcggtagttcagcgaccgcagctgccagctcgtccagttcagccagttgaatcccgga
cttactgttttcatcactaatgttaatttgaatcagaacgttaagaggggggaagttctgccgggcgctggtcgttgagacgggtagcgatgcgcaa
acggtcgatggtatgacaccagtcgaaatgctctgccaccaggcggcttttattagactgcaacgggccaataaaatgccattctaatcctgtta
cgcccagttcctgaaagtggcgaattttatctacccctttcctgaacgtagttttcaccaaattgacgctgcccggcatcaatggcttctgcgatggc
gctcgcaggttttgttttactgactgcaagcagcgtaatttcttctggagaacggccgcaacgcgttgcagccgctgagattttgtcccggacctgt
gccaggttatgcgcaatatcgttcattttccgaggatcttagtatgaatatggaagaaattgtggcccttagtgtaaagcataacgtctcggatcta
cacctgtgcagcgcctggcccgcacgatggcgcattcgcggcagaatggaagctgcgccgtttgatacgccggacgtcgaagagctactgc
gggagtggctggatgacgatcagcgggcaatattgctggagaatggtcagctggattttgccgtgtcgctggcggaaaaccagcgattgcgtg
gcagcgcattcgcgcaacggcatggcatttcgctggcgttacggctgttaccttcgcactgcccgcagctcgaacagcttggcgcaccaacgg
tattgccggaattactcaagagcgagaatggcctgattctggtgacgggggcgacggggagtggcaaatctaccacgctggcggcgatggtt
ggctatctcaatcaacatgccgatgcgcatattctgacgctggaagatcctgttgaatatctctatgccagccagcgatgtttgatccagcagcgg
gaaattggtttgcactgtatgacgttcgcatcgggattgcgggccgcattgcgggaagatcctgatgtgattttgctcggagagctgcgtgacagc
gagacaatccgtctggcactgacggcggcagaaaccgggcatttggtgctggcaacattacatacgcgtggtgccgcgcaggcagttgagc
gactggtggattcatttccggcgcaggaaaaagaccccgtgcgtaatcaactggcaggtagtttacgggcagtgctgtcacaaaagctggaa
gtggataaacaggaaggacgcgtggcgctatttgaattgctgattaacacacccgcggtggggaatttgattcgcgaagggaaaacccacca
gttaccgcatgttattcaaaccgggcagcaggtggggatgataacgtttcagcagagttatcagcaccgggtgggggaagggcgtttgtgacg
ttgccatgcccgatgcgcaagcttatcgggcctacaaaggcaataaatgtctgatgcgacgctaacgcgtcttatcagacctatagccgctgcc
agatgtgtgctggataaggtgttcacgccgcatccggcaataagacgcgatgatattatttacactccgccaggcgtttaaatcgccttaatatcc
ctgctcgaaatagctttcgagaataataaccgcagaggcagagtcaactttgcctttgttgagcgcccgatagccgccctgttcaaacagaccg
gaacgggcttccacagtgctaagacgctcgtcatggagctttacttcaacaccgaaacggccatgaatacggttggcaaatttacgcgctctgg
cagtcaatggttgctcggtgccgtccatattcagcggcaaaccgacgatgatttcgtccggctgccactctttcagtaaacgctcgataatgttcc
agtccggcgtaccgtcctgtgctttaattgcaggcaaagggcgagcggtgccggtaatgcgttggccgaccgctacgccaatgcttttggtgcc
gaagtcgaaggcgagtaaggttccactcatcaggcgtgtcctgccacaccaggcatggtgagaatatccacaccaatcagttttgccgcctcg
cgccagcggtcggcaatcggcgttttgaacagaatattcagatctgccggggccgttagccacgcgttatcgagaatttcttgttccagttgacctt
tctcccaggaggcataacccagagccaccaatacgtcagacggttgtttatcggtgccgagcgtttccagcacatcgcgggaagtggtcatta
ccgtgttgtctgaaatgcgaatgctggaagcaaaattggagggcggagtatgcaaaataaacccgcgatcttcagccagcggaccgccgag
cataaccggtttatccagacggattgattcatcacgcggctccggcgtgatcttcagcttttccagaatcccttcaattttgagattttccagcggctt
gttgacgatgatccccattgcaccattggtattatgttcgcaaatgtagaccacggaacgacggaaaatcggatcctggagagcaggcatggc
aataagaaagtgatgctgtaaattcattgtcagaggttctgtttcctggttcaaaaaagcaacagcgcccagtatgcgtggaaagaagggcgct
gtcactcagagtctcaacgagatccttctcgctaaggtgggttactgctgctgtaaacgtgcttcgatggcatccattaacattccggtgatcgaca
ccggaaactctgcttcaatctcacgaatacaggttgggctggtgacgttaatttcagtcagacggtcgccgatgatatccagaccaacaaaaat
cagccctttttctttcagcgtcggcccgatctgacgggcgattttccagtcactttccgtcagcggacgaggttcaccgcgaccaccggcagcca

gattgccacgggtttcgccccctgcggaatacgcgccaggcagtacggtaccggctcgccatccaccaccagcacgcgtttgtcgccatcttt
aatggctggcaggtaattttgcgccatgcagtagcgagtgccatgctcagtcagggtttcggcaatcacgccgaggtttggatcgccttctttcac
gcggaaaatcgacgcgccgcccataccgtccagcggcttaagaatgatgtcgctgtgtttctcccagaacgcttttagctgcgctttattgcgcgt
aaccagcgtttctggcgttaagtcagagaaccaggcggtaaacagtttctcgttacagtcgcgcaggctctgcggcttgttaacgatcagcgtcc
ctttctcttcggcacgttccagaatataggtcgcgtagataaactcggtatcaaacggcgggtctttacgcatcaggatcacatcgagatcggcc
agcggcagatcctgttcaccgacgaacgaaaaccactcttcgtagttctgcttcacgttcagcgtgcgggtatgggcgcgggcttcaccattgat
cagatacagatcgcccatctccatatagtgaagttcgtaaccacgacgctgtgcttccagcaacatagcaaaactggaatctttcttgatgttgat
gtttgcgatggggtccatcacgatgccgagcttgatcattattcttctccgttagcccaaatcgccaaatcgtacttgtagcgcggtaatggcggtg
agcgcagttgtctctgtacgcaaaacgcgaggtcccaacaggatatcagtaaattgatagcgggcagtcatggcaatttcatctgccgataaa
ccgccttccgggccaatcagcaggcggacgcgttcaaccggtaacggcaacgtattgatgctgttactggcgcgcgggtgaagattcagtttc
agtccttcatcctgctctgcacaccaggcttccagatccatcgctggacggatttccggcacccggttacgaccacactgctcacaggcagcaa
ttgcaatcttctgccactgctgaagcttcttgttcagacgttcactatccagtttaacgccgcagcgctcagaaaaaagtggcgtaatgaggcttac
accgagttcgatcgatttctggatagtaaattccatttttcaccacgcgacatcacctgaccgaggtgaatatgcagcggagattcgcgatcgtc
gatctggccttccagcaccttcacttccacgctttttttgctggcgctggtaatttcggcgtcaaagacctggttgctaccgtcaaacaattgcaacg
cctgccccggccccatgcgcagtacgcgcccgatatggttggcggcatcttcgcaaagcgcgatgtgagaatggctggtcagtggttcaggat
gataaatgcgggggatacgcatagttaaaaatccgcgtcgtctccccacgcggttgtacgcgtggggtaggggttaacaaaaagaatcccgc
tagtgtaggttagctctttcgcgcctggcaagcgcgttgcacatacgggttatgattgccctgcaccttcgcgatgcgttcatcgcgctcgcactcc
cagtcggtaaccggatacatcttgttccatgcgttgaacagctgcgtttgctggcgagagagtgtcaggttgtattggtcgcgcatatagaagtag
gtgcgcgcaatggcaccgcgtgcacgcgctggtggttcggcagctttttctttgaaatcgaccttcatggcgcattgaccgtactggccttcaccg
ccattccactggctgtacataaagttgccgcgatcgccattcacctcaccgactgacggctgcaggttatgcatatcgctttccatcttgcgataga
ccggatctttagcgcagtttttacgtccaccgtcctgccagcactggcgctggtgaccgaactgccaggcgggaacgacatgttcccactctac
gcggctggcgcggttttcatttttgcgcacctgatagccgcacgattgcagatcaacaacgcctttttttgccctgccagttaattttacatccgcaat
aaaacgtaccgggcgcgtcagcgtggacttttaccgccgcggctttcgcctgagaaaaactattgataccttcggccaacgccgggccggaa
aatgctgcgctcagtaccaccgcagcaatagacaaataacggtacatcacttactccgtgttaaaacgagccagcaacgtagcgaaagctgt
tttggtatgcaatcagccagatcaggaaagttcctgatagttcagatggttatttcgcaaccagctgttcaccgcagtgaacacagcgatagacg
gcctcgccacgaactacgcgattatggcggcgtacggtaagctgatgctcctggcacttgcagcggtaggggaaggtgttgcgacgcacgga
ttgcagttcgaactgatgcgtacgacgggcgggaacacccagcacgttttccatcatccacttccactctttgccatgtggcgctacgcggccga
aatgtttccataccagcaaatgtgccagttcgtgcggtaccacttcttcaataaaagcttcactgttttccaacagcaaaacgggattgaggcga
atttcatagctttccagccaggccgttccggcggaggttccgcgctgggtgtaagagagttttggctccgggtagttacgccctagcttcaggttgg
cctgggcgagtttttcccgcaggcgacgcataacggcctgttggatggcgatagggagacgggatgttttcatagcggcagaggatagagcg
aagaaaagatgactgcaagaggtggcttcctccgcgatgggaggaagcttggggagattaatcgtgagcgcctatttcgcgcagtttacgacc
tttcatcagattacgttcaatatgttccagcgaaacgtgtttggtttccggtaccagccacaatgtcagcaggataaacagtacgttcagagccgc
atacacccagaaggtgttggcgttacccagcgtgttgagcatggtcaggaacgttgcgccaacgatcatgttggcaatccagttggtggcagtg
gagcaggtgatgccaaaatcgcggcctttcagcggctgaatttcggagcacagtacccaaatcagcggaccggcactcatggcaaaaccg
acaataaacatcagcagcatggcgatggcgaaatactgcgccgacggagagtgaataccgatatgcatcattgtaccgagtacgcccatgc
cagcagccatcaccaggaagcccagcgttagcgttggtttacgtccccagcggtcaacaaggccgattgcgataaaggtggcaagtacgttg
gtcaggccgacaatcacggtcccccacatttgctcggtagtgttggtataacccgccagttcgaagattttcggcgcgtaatacatgatgacgttc
atcccggtgaattgctgcattacctgcaacagtacgccaaggaacaccgcgcggcggaagttgctgttctctttaaacagcgcccagccactct
gtttaacctgcaaactttcacggatttcatccagttcgcgtttcgcttccgcgctggtgtcacgcaggcgtagcagcacgcgttcggcatcaacaa
aacggcgtttggcggcaaaccaacgtgggctgtctggcaggaagaagacaccaatcagcagcaaaattgccgggatgataatcacaccc
agcatccagcgccatgcaccggtgtagctgaaggcggtatcagaaagataagcaccgaggatcccgatagtgatcatcaactgatacatcg
agatcatactgccacgaatttttttccggcgcaatttcagagaggtacagcggtgcggtataagaggccacacccaccgccagccccagtaga
acgcgggaaagaatcagtacttcaacgtttggcgcagccgcagagaacagcgaaccggcaacaaacaaaattgcgccgatcatcaggct
ctttttgcgcccgagtttaaaggagagccagccgctgcccaccgcaccgactgccgcaccgaacatcatggagcttacgacccattcttgcgt
gtgcgaagtaatctggaattcatctgcaataaacggcagtgcgccagcaattacaccgatatccaggccaaagagtaatcccgccagagcg
gcaaggaagcagacgaaaaacgtcattgccttgtttgaccgcccctgtttttttagcgtcaggcatgatgccctccaatatggttatttttttattgtgaa
ttaagataggtgagtacgacgtaaaaagatgtgaagcaaatcacatcagtgtaatcgcttacactaacatttatcttagttatgatttaagtgcatg
aaaataattgagttatatcatcatagattaaacgctgttatctgcaattaagactttactgaaaagaaatgtaacaactgtgaaaaccgaataata
atatgaaaatattacaaatctttgtctgttgtggttggtgtaatcggtttcagttgtgatgataatctgcggtgaaacgtgatcaggaagaatgatgtta
ttgtgcgggcggtttttttgccggatgcggcgtgaacgcctatccaaaagaaaaaggccagcgcgaggctggcctttgaacgcaggtgaaga

aagattacttcagaccggcagcatcgcgcagcagctgcgctttgtcggtttttcccacgggaaatgttcacgaccaaagtgaccgtatgctgcg
gtttctttgtagatcgggtgcagcagatccagcatctgaatcagaccgtatgggcgcaggtcgaagaactcacgtaccagcagggtcagttgtt
cagaaggcactttctcagtaccgaaagtttctaccatgatggaggtcggttcagccacgccgattgcgtaggaaacctgaatttcacaacgatc
ggccaggccagcagcaacgatgtttttcgcgacataacgtgctgcgtaggctgcggaacggtccacttttgatggatctttaccagagaatgca
ccgccaccgtgacgcgccatgccgccgtaggtatcaacgataattttacgaccagtcagaccgcagtcacccattgggccaccgataacga
aacgaccggtcgggttgatgaagaatttggtggcagaagtcagccattcagcgggcagaattggcttgatgatctcttccattaccgcttcttgca
gcgatttctggtcgatctcttcagagtgctgagtggaaagcacgacagcatcgataccaacgattttgccgtcgtcatactgaaaagtcacctgg
cttttcgcgtccgggcgcagccacggcagagtgccgtttttacgcacttcagcctgacgctgtaccagacggtgtgcataggtgataggtgctgg
catcagcacgtcggtttcattagttgcgtagccaaacatcagaccctggtcacccgcgccctgttccagcggatcggcacggtcaacgccctg
gttgatgtcaggagactgtttgccgatagcgctcagaaccgcacaggagttagcgtcaaagcccatgtcggaatgcacatagccaatttcgcg
aacggtgttacgggtgatctcttcgatgtctacccaggcgctggtggtgatttcgccgccaactaaaaccatgccggtttttacgtaggtttcgcaa
gcaacgcgtgctttcggatcctgttcgaggatcgcgtctaaaacggcatcagaaatttggtcagcaattttgtcaggatgcccttcagagacgga
ctcggacgtaaaaaggtgttttgccatatttaatatcacctaaagagaatttggttagctcaaactgttgtgtggattttctgtggtagcggatcctac
cacgactctgcaggttaaaaacactggcagtctgagtgttaatcggtatggatggattaacatctggatggctattttaggtcaattcttcaccctatt
tccactttttttgaatcgtgtctcattctgttaaaaacgtggctggaaatttttcctgacaatgccggcattctgcgtatttatcttttgcaattttctgccatt
gtggggtataaaacgcggcgcgcggcttaaataaaaagcacacgacgtttctttcgtgttgccacttccagccgggttgaaatcagagttttggc
ttgtgggttcgtcttaacaggcggccgtggaggtgatacgaaataatgaaccgttgtctgctgcttaacctgtctcaccgttctggtgaagattcgtt
ccccgcactctgcatctctgctttgcatacctgccgatgttatacccatctcggcgcttctcaggattcaagagctggttacagttactgaggactga
acaagggcgctcttgtaaaaacaagagttttctcgtggtttcgccgaactttcacacttacgttcggttatgtgcttaataatgttatgaaaaagaaa
ccggttgcgcagttggagcgtcagcattcactgctggaaaatccatgtgcttatgggttgttatcgcagttccaggctgcgatagtcgttaactgtttt
acacttaataaaataatttgaggttcgctatgtctgacgacatgtctatgggtttgccttcgtcagcgggcgaacacggtgtactacgctccatgca
ggaggttgcaatgagctcccaggaagccagcaagatgctgcgtacttacaatattgcctggtggggcaataactactatgacgttaacgagct
gggccacattagcgtgtgcccggacccggacgtcccggaagctcgcgtcgatctcgcgcagttagtgaaaactcgtgaagcacagggcca
gcgtctgcctgcactgttctgtttcccacagatcctgcagcaccgtttgcgttccattaacgccgcgttcaaacgtgcgagggaatcctacggctat
aacggcgattacttccttgtttatccgatcaaagttaaccagcaccgccgcgtgattgagtccctgattcattcgggcgaaccgctgggtctggaa
gccggttccaaagccgagttgatggcagtactggcacatgctggcatgacccgtagcgtcatcgtctgcaacggttataaagaccgcgaatat
atccgcctggcattaattggcgagaagatggggcacaaggtctatctggtcattgagaagatgtcagaaatcgccattgtgctggatgaagca
gaacgtctgaatgtcgttcctcgtctgggcgtgcgtgcacgtctggcttcgcagggttcgggtaaatggcagtcctccggcggggaaaaatcga
agttcggcctggctgcgactcaggtactgcaactggttgaaaccctgcgtgaagccgggcgtctcgacagcctgcaactactgcacttccacct
cggttcgcagatggcgaatattcgcgatatcgcgacaggcgttcgtgaatccgcgcgtttctatgtggaactgcacaagctgggcgtcaatattc
agtgcttcgacgtcggcggcggtctgggcgtggattatgaaggtactcgttcgcagtccgactgttcggtgaactacggcctcaatgaatacgcc
aacaacattatctgggcgattggcgatgcgtgtgaagaaaacggtctgccgcatccgacggtaatcaccgaatcgggtcgtgcggtgactgc
gcatcacaccgtgctggtgtctaatatcatcggcgtggaacgtaacgaatacacggtgccgaccgcgcctgcagaagatgcgccgcgcgcg
ctgcaaagcatgtgggaaacctggcaggagatgcacgaaccgggaactcgccgttctctgcgtgaatggttacacgacagtcagatggatct
gcacgacattcatatcggctactcttccggcatctttagcctgcaagaacgtgcatgggctgagcagctttatttgagcatgtgccatgaagtgca
aaagcagctggatccgcaaaaccgtgctcatcgtccgattatcgacgagctgcaggaacgtatggcggacaaaatgtacgtcaacttctcgct
gttccagtcgatgccggacgcatggggatcgaccagttgttcccggttctgccgctggaagggctggatcaagtgccggaacgtcgcgctgt
gctgctggatattacctgtgactctgacggtgctatcgaccactatattgatggtgacggtattgccacgacaatgccaatgccggagtacgatcc
agagaatccgccgatgctcggtttctttatggtcggcgcatatcaggagatcctcggcaacatgcacaacctgttcggtgataccgaagcggttg
acgtgttcgtcttccctgacggtagcgtagaagtagaactgtctgacgaaggcgataccgtggcggacatgctgcaatatgtacagctcgatcc
gaaaacgctgttaacccagttccgcgatcaagtgaagaaaaccgatcttgatgctgaactgcaacaacagttccttgaagagttcgaggcag
gtttgtacggttatacttatcttgaagatgagtaagtcctgtgttacttgaatccgcttaatttagcggtgataatccgccacaatttattgtgacaaatc
caacccttcctcgtcgggcctaacgacgcggaagggtttttttatatcgactttgtaataggagtccatccatgagcaccttaggtcatcaatacga
taactcactggtttccaatgcctttggtttttttacgcctgccgatgaacttccagccgtatgacagcgatgcagactgggtgattactggcgtgccgtt
cgatatggccacttctggtcgtgcgggtggtcgccacggtccggcagcgatccgtcaggtttcgacgaatctggcctgggaacacaaccgcttc
ccgtggaatttcgacatgcgtgagcgtctgaacgtcgtggactgcggcgatctggtatatgcctttggcgatgcccgtgagatgagcgaaaagc
tgcaggcgcacgccgagaagctgctggctgccggtaagcgtatgctctctttcggtggtgaccactttgttacgctgccgctgctgcgtgctcatg
cgaagcatttcggcaaaatggcgctggtacactttgacgcccacaccgatacctatgcgaacggttgtgaatttgaccacggcactatgttctat
accgcgccgaaagaaggtctgatcgacccgaatcattccgtgcagattggtattcgtaccgagtttgataaagacaacggctttaccgtgctgg
acgcctgccaggtgaacgatcgcagcgtggatgacgttatcgcccaagtgaaacagattgtgggtgatatgccggtttacctgacttttgatatc

gactgcctggatcctgcttttgcaccaggcaccggtacgccagtgattggcggcctgacctccgatcgcgctattaaactggtacgcggcctga
aagatctcaacattgttgggatggacgtagtggaagtggctccggcatacgatcagtcggaaatcactgctctggcagcggcaacgctggcg
ctggaaatgctgtatattcaggcggcgaaaaagggcgagtaagcaccagatgcgatgcgcacgggtaaaacgtgccattaatgtcggatgc
ggcgtgaacgccttatccgacctacgttcggcacccgtaggccggataagatgcgccagcatcgcatccggcaatgcgcacaaggtaacaa
atgtgccattcatgtcagatgcggcgtgaacgccttatctgacctacgttcgacaccaccaggctttacttaatcccatccgcgctcatgcgatcg
cgaatatgctgggcgcgttcggcggatgcaggatggtcgtcaaacattgagctttggcgaccttcttccagttttgccagtttttcaaagctggtgg
caagacctgccgggctgatgccgcgttggcgcagaagatcgtaagaataatcatcggcttctgcttcctggcgctgggagaattgcgaattga
ctaatttctcgcccagattaccaagttgtgattgagataaacttccgacaatcccgcccgcagaggccgcagctactcgcacggcatttgtacca
agtgccacctgcattcctttttcacatggcctaacgccacgtgccccatttcgtgaccgatcaccgcttcgacttcgttatccgtcatcatatccatc
agcccgctatagacgcggatacagccgttagccattgcaaaggcgttcacatccttcgccatatacactttgtaatttaccggctgaccgttgata
ttgttgcctagcgcattggcaatagttgtcagacgtttagcgtattcgctattggctggcgcaatcgtcgccttgctgtccatctcctgacatgcctgat
cgctcagggttttcacctgcgcatcactcaaactgtaagcctgaaaagcttccgctcctgatgagagcagtccgttggagtccatattctggcaa
ccagtcagtaccgttgccacgctcattgctaccaataaggcgcgaattttcatgttttctcttccgcacaaggtcaggattattctaaaaatgcgcc
gtttgcaggtgaatcgacgctcagtctcagtataaggaaaagcgcagcggacgggcgagtagattgcgcaacatgcgagcatgatccagag
atttctgaagcggcaaaaggatgttccatgtacatgacgcgcggcttgcggtaaattgttggcaaattttccggcgtagcccaaaacgcgctgtc
gtcaagtcgttaagggcgtgcccttcatcatccgatctggagtcaaaatgtcctcacgtaaagagcttgccaatgctattcgtgcgctgagcatgg
acgcagtacagaaagccaaatccggtcacccgggtgcccctatgggtatggctgacattgccgaagtcctgtggcgtgatttcctgaaacaca
acccgcagaatccgtcctgggctgaccgtgaccgcttcgtgctgtccaacggccacggctccatgctgatctacagcctgctgcacctcaccg
gttacgatctgccgatggaagaactgaaaaacttccgtcagctgcactctaaaactccgggtcacccggaagtgggttacaccgctggtgtgg
aaaccaccaccggtccgctgggtcagggtattgccaacgcagtcggtatggcgattgcagaaaaaacgctggcggcgcagtttaaccgtcc
gggccacgacattgtcgaccactacacctacgccttcatgggcgacggctgcatgatggaaggcatctcccacgaagtttgctctctggcgggt
acgctgaagctgggtaaactgattgcattctacgatgacaacggtatttctatcgatggtcacgttgaaggctggttcaccgacgacaccgcaat
gcgtttcgaagcttacggctggcacgttattcgcgacatcgacggtcatgacgcggcatctatcaaacgcgcagtagaagaagcgcgcgcag
tgactgacaaaccttccctgctgatgtgcaaaaccatcatcggtttcggttccccgaacaaagccggtacccacgactcccacggtgcgccgct
gggcgacgctgaaattgccctgacccgcgaacaactgggctggaaatatgcgccgttcgaaatcccgtctgaaatctatgctcagtgggatgc
gaaagaagcaggccaggcgaaagaatccgcatggaacgagaaattcgctgcttacgcgaaagcttatccgcaggaagccgctgaatttac
ccgccgtatgaaaggcgaaatgccgtctgacttcgacgctaaagcgaaagagttcatcgctaaactgcaggctaatccggcgaaaatcgcc
agccgtaaagcgtctcagaatgctatcgaagcgttcggtccgctgttgccggaattcctcggcggttctgctgacctggcgccgtctaacctgac
cctgtggtctggttctaaagcaatcaacgaagatgctgcgggtaactacatccactacggtgttcgcgagttcggtatgaccgcgattgctaacg
gtatctccctgcacggtggcttcctgccgtacacctccaccttcctgatgttcgtggaatacgcacgtaacgccgtacgtatggctgcgctgatga
aacagcgtcaggtgatggtttacacccacgactccatcggtctgggcgaagacggcccgactcaccagccggttgagcaggtcgcttctctgc
gcgtaaccccgaacatgtctacatggcgtccgtgtgaccaggttgaatccgcggtcgcgtggaaatacggtgttgagcgtcaggacggcccg
accgcactgatcctctcccgtcagaacctggcgcagcaggaacgaactgaagagcaactggcaaacatcgcgcgcggtggttatgtgctga
aagactgcgccggtcagccggaactgattttcatcgctaccggttcagaagttgaactggctgttgctgcctacgaaaaactgactgccgaagg
cgtgaaagcgcgcgtggtgtccatgccgtctaccgacgcatttgacaagcaggatgctgcttaccgtgaatccgtactgccgaaagcggttact
gcacgcgttgctgtagaagcgggtattgctgactactggtacaagtatgttggcctgaacggtgctatcgtcggtatgaccaccttcggtgaatct
gctccggcagagctgctgtttgaagagttcggcttcactgttgataacgttgttgcgaaagcaaaagaactgctgtaattagcatttcgggtaaaa
aggtcgcttcggcgacctttttattaccttgatatgtccgtttgcggacaagcaatagataaggcgtgttgtagatcacaaatatttatatgcaataa
atatcaattatgtaatatgcatcacgatatgcgtattgacatttgttgttatatctataactcaatgttatataagaaattaataattcactgttttcaaaa
caccggtttccctgctcaattgctttcattaaaccgcacgagtattttatttttaccgccggacggtaccctaaaaataaaataaggataatttatgc
ggcttagtgattattttccagaatcatcaatctcagttatacattcagcaaaagattggcaggaagctatcgatttctcgatggtatcattgctggata
aaaactatatcagcgagaattacattcaggcaattaaagattccaccattaacaatggcccttattatattctcgcaccaggcgtggcaatgcctc
atgcgcgaccggaatgtggggcgcttaaaaccgggatgtctttgacattacttgaacaaggtgtttattttccggggaatgacgaaccaattaaa
ttactcatcggactctctgctgccgatgccgattcgcacattggcgctattcaggcgttaagtgaattactgtgcgaagaagaaatactcgaaca
actcttaacagcatcatcagaaaaacaattagcggacattatcagccgcggataattactctctcctctacaggataatattatggaaaacaagt
ctgctcgtgcaaaggtccaggcttttgggggctttttgactgcaatggtcatccccaatattggtgcttttattgcctggggttttattactgcgttatttatt
cccaccggttggctgcctaacgaacatttcgccaaaattgtcggcccgatgattacctatttattgcccgtgatgattggttctacaggtggtcatct
ggtcggcggtaaacgcggggcggtcatgggcggaataggtactattggtgtgatcgttggcgcagagatcccgatgttccttggctcaatgatc
atggggccgctcggtgggttggtcataaaatatgtcgataaggcactggaaaaacgcatacctgccggttttgagatggttatcaataacttctc
attaggtatcgcggggatgctcctttgtctgctgggttttgaagttatcggcccggcggtgttaattgccaatactttcgtcaaagagtgtattgaggc

gctggtacatgcgggttatctgccattgttgtcagtcatcaatgaaccggcgaaagtgcttttcctcaataatgcgatcgatcagggcgtctattatc
cgctgggaatgcaacaggcttcggttaacggtaaatccatcttctttatggtggcctctaacccaggtccgggcctggggctgctgctggcgttta
ccttgtttggtaaagggatgagtaaacgttctgcgcccggggcgatgattattcacttcctcggtgggatccacgaactgtatttcccgtatgtgctg
atgaagccgctgaccattattgccatgattgcgggcggtatgtctggcacctggatgtttaacttactggacggtggtctggtggctggcccaagt
ccggggtctatctttgcttacctggcactgacgccgaaaggctcgttcctggcgacaattgccggtgttacggtaggtaccctggtgtcctttgctat
tacttcgctgatactgaagatggaaaaaacggtggaaacggagagcgaagatgagtttgctcagtcagccaatgcggttaaggcgatgaaa
caagagggtgcattctcgttaagcagggttaagcgtatcgcctttgtttgcgatgcggggatgggctccagtgcgatgggcgcgaccaccttcc
gtaaacgcctggaaaaagcggggctggcaattgaagtaaaacattacgccatagaaaacgtgcctgcggatgcggatatcgtcgttactcat
gccagtctggaagggcgcgtgaaacgtgtgacggataaaccactgatattgattaataactatattggcgatccaaaactcgacactttatttaa
tcaattaaccgccgaacataaacactgattacagaggtaaaaatgaaaaccaaagttgctgctatttatggcaagcgggatgtccgtctgcgc
gtatttgaactgccagaaattaccgataatgaattactggtgagtgtaatttctgacagcgtctgtttatcgacctggaaagcggcgttactcggta
gtgaacataaacgcgtacccgacgatttagaaaatcatccggtcattaccgggcatgaatgtgccggggttattgtcgaagtgggtaaaaatct
cactggcaaatataaaaaaggccagcgttttgtattgcaaccggcgatggggttaccaagcggatattcagcgggctacagctacgaatatttt
ggcggcaatgccacttatatgattattcccgaaatcgccattaatttgggctgcgtattaccgtatcacggctcttattttgctgcggcgtcgctggca
gagcctatgtgctgcattattggtgcttatcatgccaattatcacaccacgcaatatgtttatgagcatcgcatgggcgtcaaacctggcggcaat
attgcactgctggcgtgtgcaggtccgatgggcattggcgctatcgattacgccattaacggcggcatacaaccgtcgcgggtggtggtggtcg
atatcgacgacaaacgtctggcgcaggtacagaagctgctgccggtggaactggcggccagtaaaggcattgagctggtgtatgtgaatacc
aaagggatgagcgatcctgtccagatgctgcgggcgctgacaggagatgccgggttcgatgacatttttgtttatgcggcggtgcctgctgtcgtt
gagatggctgatgaattactggcggaagatggctgtctgaacttctttgccgggccgacggataaaaacttcaaagtgccgtttaatttctacaac
gtccattacaacagcacgcacgtcgtcggtacatctggcggttcaacggacgacatgaaagaggcgattgcccttagcgccactgggcagtt
acagccgtcgtttatggtgacccatatcggtggcctggatgcggtgccagaaaccgtgctcaatctgccggatatccctggcggtaaaaaactc
atttataacggcgtgaccatgccgctcactgccattgccgattttgccgaaaaaggcaaaaccgatccgctgtttaaagagttggcgcggctggt
tgaggaaacgcacggcatctggaatgaacaggccgagaaatatctgctggcacaatttggcgttgatatcggggaggccgcgcaatgatgtc
cctggcgtggccattatttcgcgttacggaacaggcagcgttggctgcctggccgcaaaccggatgtggcgacaaaaataaaattgatggcct
ggcggtcaccgcgatgcgccaggcattaaacgacgtcgcttttcgtggacgagtggttatcggcgaaggagaaattgaccatgcgccgatgc
tgtggattggcgaagaggtgggcaaaggcgatgggccagaagtcgatatcgcggttgatcccattgaaggcacgcggatggtggcgatggg
gcagagcaatgcgctggcggtcatggctttcgcccctcgagatagcctgttacatgcgcccgatatgtatatgaaaaagctggttgttaatcggc
tggcggcgggcgcgattgatctgtctttgcctctgacggacaacctgcgcaacgtggcgaaagcgttaggcaaaccgctggataagctgcgc
atggttacgctggataaaccgcgcctgagtgccgcgattgaggaagcaacacagctgggcgtgaaggtttttgccctgccggatggcgatgtc
gccgccagcgtgctgacctgctggcaggataatccttacgatgtgatgtacaccatcggcggtgcgccagaaggcgtgatttctgcctgtgccg
ttaaagcattaggcggggatatgcaggccgagctgattgacttttgccaggctaaaggggattacacggaaaatcggcagattgcagagcag
gagcgcaagcgttgcaaggcaatgggcgttgacgttaaccgtgtgtactcgctcgatgaactggtgagggggaacgatatcctctttagcgcta
cgggcgtgacgggcggtgagctggtgaacggtatccaacagacggcgaatggggtgcggacgcagacattactgatcggcggcgcggac
caaacgtgtaatataatagactccctgcattgatggtgatctatcggaagaccaatggcgacgctgacagaagatgatgtgcttgagcaactg
gatgcacaggacaatttattttcatttatgaaaactgcgcataccattttgctccaggggatacgccagtttctgccgtcgctgtttgtcgataacgat
gaagagatcgtggaatatgcagtgaagccgttactcgcccaaagcggcccgcttgacgatattgatgttgcactgcgtttgatttatgcgttgggg
aaaatggataaatggttgtacgccgatatcacgcatttttcccagttctggcattacctgaacgaacaggatgaaacgcccggatttgccgatga
catgacctgggattttatcagcaacgtcaatagcataacccgcaatgcgatgctctatgacgcgttaaaagcgatgaagttcgctgatttctccgt
ctggtcagaggcgcgttttagcggaatggtcaaaacggcgctgacgctggcagtaacgaccaccttaaaggaattaacgccgtgaaaattga
attaacggtgaatgggctgaagatacaggcacagtaccagaacgaagagattgaaaatgtacataaaccgttgctgcacatgttggcggcct
tacagacggtaaatccgcagcggcgtacggtggtttttctttgcgctccgccggggacgggtaaatcaaccctaaccaccttctggaatatctc
gcgcaacaagacccggaactgcccgctattcagacgctcccgatggatggttttcaccattacaatagctggctggatgcgcatcaattgcgcc
ccttcaaaggcgcaccagagacatttgacgttgcgaaactgacggaaaatctgcgccaggtcgtggaaggggattgtacgtggccgcagta
cgatcgacaaaagcatgatcctgttgaagatgcgttgcacgttaccgcaccactcgtcatcgtcgaaggaaactggttgttactggatgatgaa
aagtggctggagttggcatctttctgcgatttttctatttttattcatgcacctgctcaaattctgcgggagcgcttgattagtcgtaaaattgcgggag
gattaacgcggcaagtagccgaagcattttatgcccgcaccgatggccccaatgtcgagcgtgtgctgatgaatagccgtcaggccaatctga
tagtagagatgactgaagaaggccggtatcacttcacaagctgacaatttattccagttattgtagggttattattacgccagcctggaataaagc
ttcccacaaaaggggataaaagtgtgatgtgagtcagataaatgtcttcttcggctggacaaacattccttttattccacgtttcgcttatcctagctg
aagcgtttcagtcgattaaatgttcgacaattaaccaatcagtcgcagtttgcgacaggtaaggtttccccggacgatttgctggattactctgtctg
gcaaatttgctcagggaaaaccttgcaggagatctatgaccgtacgcgtagcgataaatggcttcggtcgcatcgggcgtaatgtggttcgtgct

ttgtatgaatccggacgccgggcggaaattaccgtggtggcaatcaacgaactggcggatgctgcgggcatggcgcatttgttgaaatatgac
accagccatggccgttttgcatgggaagtacgacaggaacgcgatcaactttttgttggtgatgacgccatccgcgtattgcatgaacgttcact
gcaatcgctcccctggcgtgaacttggcgttgatgtagtcctcgactgcaccggcgtatatggctcccgcgagcatggcgaagcgcatattgcc
gccggggccaaaaaagtgctcttttcacatcctggcagtaacgatctcgacgcgaccgttgtttacggcgtcaatcaggatcaacttcgtgcgg
aacaccgcatcgtttctaacgcttcctgtaccacgaattgcataattcccgtcatcaaattgttagatgatgcgtacggtattgagtccggcactgtg
accacaattcactccgccatgcacgatcaacaggttattgatgcataccatcctgacctgcgtcgcacccgggcagccagccagtcgatcattc
cggtcgatactaaactggccgccggtatcacacgatttttccgcaatttaacgatcgctttgaagcgattgcggtacgtgtgccaaccataaatgt
gacggcaatcgatttaagcgtgacggtgaagaaacctgtaaaagccaatgaagtcaacctgttgctgcaaaaagcagcacaaggtgcatttc
atggtatagttgactatacggaattgccgttggtctctgtagattttaaccacgatccgcacagtgccattgtcgatggcacccaaacccgggtca
gtggcgcacacctgatcaaaacgttggtctggtgcgataacgaatggggctttgctaaccgaatgctcgacacgacgttagctatggctactgtt
gctttcaggtaagacgcaagcagcgtctgcaaaacttttagaatcaacgagaggattcaccatgtctgtaattaagatgaccgatctggatcttg
ctgggaaacgtgtatttatccgtgcggatctgaacgtaccagtaaaagacgggaaagtaaccagcgacgcgcgtatccgtgcttctctgccga
ccatcgaactggccctgaaacaaggcgcaaaagtgatggtaacttcccacctgggtcgtcctaccgaaggcgagtacaacgaagaattctct
ctgctgccggttgttaactacctgaaagacaaactgtctaacccggttcgtctggttaaagattacctcgacggcgttgacgttgctgaaggtgaa
ctggttgttctggaaaacgttcgcttcaacaaaggcgagaagaaagacgacgaaaccctgtccaaaaaatacgctgcactgtgtgacgtgttc
gtaatggacgcattcggtactgctcaccgcgcgcaggcttctactcacggtatcggtaaattcgctgacgttgcgtgcgcaggcccgctgctggc
agctgaactggacgcgctgggtaaagcactgaaagaacctgctcgcccgatggtggctatcgttggtggttctaaagtatctaccaaactgac
cgttctggactccctgtctaaaatcgctgaccagctgattgttggtggtggtatcgctaacacctttatcgcggcacaaggccacgatgtgggtaa
atccctgtacgaagctgacctggttgacgaagctaaacgtctgctgaccacctgcaacatcccggttccgtctgatgttcgcgtagcaaccgagt
tctctgaaactgcaccggctaccctgaaatctgttaacgatgtgaaagctgacgagcagatcctggatatcggtgatgcttccgctcaggaactg
gctgaaatcctgaagaatgcgaaaaccattctgtggaacggtccggttggcgtgttcgaattcccgaacttccgcaaaggtactgaaatcgtgg
ctaacgctatcgcagacagcgaagcgttctccatcgctggcggcggcgacactctggcagcaatcgacctgttcggcattgctgacaaaatct
cctacatctccactggcggcggcgcattcctcgaattcgtggaaggtaaagtactgcctgcagtagcgatgctcgaagagcgcgctaagaagt
aaaaaatcacagggcagggaaacctgcccttgtttcagcgcgcttttagagcacgcaatttttcatactctaaataattcgagttgcaggaaggc
gacaagagagtgaatccccaggagcttacataagtaagtgactggggtgagcgaacgcagacgcagcacatgcaacttgaagtatgacg
agtataaggcccgacgatacaggacaagagacatgtctaagatttttgatttcgtaaaacctggcgtaatcactggtgatgacgtacagaaagt
tttccaggtagcaaaagaaaacaacttcgcactgccagcagtaaactgcgtcggtactgactccatcaacgccgtactggaaaccgctgcta
aagttaaagcgccggttatcgttcagttctccaacggtggtgcttcctttatcgctggtaaaggcgtgaaatctgacgttccgcagggtgctgctat
cctgggcgcgatctctggtgcgcatcacgttcaccagatggctgaacattatggtgttccggttatcctgcacactgaccactgcgcgaagaaa
ctgctgccgtggatcgacggtctgttggacgcgggtgaaaaacacttcgcagctaccggtaagccgctgttctcttctcacatgatcgacctgtct
gaagaatctctgcaagagaacatcgaaatctgctctaaatacctggagcgcatgtccaaaatcggcatgactctggaaatcgaactgggttgc
accggtggtgaagaagacggcgtggacaacagccacatggacgcttctgcactgtacacccagccggaagacgttgattacgcatacacc
gaactgagcaaaatcagcccgcgtttcaccatcgcagcgtccttcggtaacgtacacggtgtttacaagccgggtaacgtggttctgactccga
ccatcctgcgtgattctcaggaatatgtttccaagaaacacaacctgccgcacaacagcctgaacttcgtattccacggtggttccggttctactg
ctcaggaaatcaaagactccgtaagctacggcgtagtaaaaatgaacatcgataccgatacccaatgggcaacctgggaaggcgttctgaa
ctactacaaagcgaacgaagcttatctgcagggtcagctgggtaacccgaaaggcgaagatcagccgaacaagaaatactacgatccgc
gcgtatggctgcgtgccggtcagacttcgatgatcgctcgtctggagaaagcattccaggaactgaacgcgatcgacgttctgtaagatattcct
ttctgcttatctcaaggcccgctctgcgggtctttttttcgccaaaagcaaaattgcctgatgcgctacgcttatcaggcctacacgtatcctgcaata
tattgaatttgcaggtttttgtaggccggataaggcgttcacgccgcatccggcatgacaaagcgcacattgatatctgtctgagccccgctctgc
ggggatttttttcgccaaaagcaaggtgattcagatgagaaatctgtgatctatttggcaaaattatgctttattgtttacccttgtcagactgcccgtc
ataaggcggcggagtgtatttctccattttgagtcagttgaaaaggaatattgaatggaagatttgaatgttgtcgatagcataaacggcgcggg
aagctggctggtagctaaccaggcgctgctgctaagttatgcagtaaacatcgtggcggcactcgcgatcatcatcgttggtttgattatcgcgc
ggatgatttccaacgcggtgaatcgcctgatgatctcccgtaaaatcgatgccactgttgctgattttctttctgcattagtccgttacggtattatcgc
ctttacgctaatcgctgcactgggacgcgtgggtgtacaaaccgcgtcagtcattgctgtactcggtgccgcaggcttagctgttggtctggctttg
caggggtcactgtctaacctggccgctggcgtgttacttgtcatgttccgcccgttccgtgccggagaatatgttgacctgggcggcgtagccggt
actgtgctgagtgtgcagattttctccaccaccatgcgtactgcagacggtaaaattatcgttattccgaacggtaaaattattgccggaaatattat
taacttctcccgcgagccagttcgccgtaacgaatttattattggcgtggcgtatgattccgatatcgatcaggttaagcagatcctgaccaatatt
atccagtctgaagatcgcattttgaaagatcgcgaaatgactgtgcgcctgaacgaacttggtgcatcgtcgattaatttcgtggtccgcgtctgg
agcaacagcggcgatctgcaaaacgtgtactgggatgtgctggagcgtattaaacgtgaatttgatgccgccggtatcagcttcccgtacccgc
aaatggatgtgaactttaagcgggtgaaagaagacaaagctgcgtaatcaacgctgaggccagataatactctctggcctctctcttattagtttt

tctgattgccaattaatattatcaatttccgctaataacaatcccgcgatatagtctctgcatcagatacttaattcggaatatccaacgtgttttcttatt
actttcaaggtcttgcacttggggcggctatgatcctaccgctcggtccacaaaatgcttttgtgatgaatcagggcatacgtcgtcagtaccacat
tatgattgccttactttgtgctatcagcgatttggtcctgatttgcgccgggatttttggtggcagcgcgttattgatgcagtcgccgtggttgctggcgc
tggtcacctggggcggcgtagccttcttgctgtggtatggttttggcgcttttaaaacagcaatgagcagtaatattgagttagccagcgccgaagt
catgaagcaaggcagatggaaaattatcgccaccatgttggcagtgacctggctgaatccgcatgtttacctggatacttttgttgtactgggcag
ccttggcgggcaacttgatgtggaaccaaaacgctggtttgcactcgggacaattagcgcctctttcctgtggttctttggtctggctcttctcgcag
cctggctggcaccgcgtctgcgcacggcaaaagcacagcgcattatcaatctggttgtgggatgtgttatgtggtttattgccttgcagctggcga
gagacggtattgctcatgcacaagccttgttcagttaggcgctatctgatggaaaaataaaacagaggcgctaagcttgcctccagaggtcctg
aattttgggcaagtcaattaacacggagagactaacgtgaagttcaaagttatcgccctggcggcattaatgggtattagcgggatggcagcg
caggctaacgaattgccggatggaccgcatattgtcacctccggtacggcaagcgtggatgcggtgccagacattgccactcttgcgattgaa
gttaacgtggccgcgaaggatgccgctactgccaagaaacaggcagatgagcgcgtcgcacaatacatttccttccttgaactcaatcagatc
gcgaaaaaagatatcagctcagcgaacttacgcacccagccagattatgattatcaggatggtaaaagtatccttaaaggctaccgcgctgtg
agaacggtggaagtcacgctccgtcagttagacaaactgaattccttgctggatggcgcgctgaaggcgggtcttaacgaaattcgttctgtgtc
gctgggcgtggcgcagccggatgcctataaagacaaagcgcgtaaggcagcgattgataacgcgattcatcaggcgcaggaactggcga
acggctttcatcgtaaactggggccggtatatagcgtgcgctaccatgtttccaactatcagcccagcccaatggtgcggatgatgaaagccga
tgccgcgccggtgtccgcccaggaaacttacgagcaggccgctattcagtttgatgatcaggtcgatgtggtcttccagttagaacctgtggatc
aacaacccgctaaaacacctgcagcacaataatatttgtagcccgacgtattcgtcgggctaatgcctttacccgatattgcctcctttttctgtaat
tttctgtaaaaccatgacgtaatcacctatctttgctcgcggtttcatcaaggataggctgactgaaacggttaagcggacaaggatgcgggga
gggggatggacatttttatttcgaaaaagatgcgcaatttcattttattagcgcaaaccaataatattgcccgggcggcagaaaaaatccatatg
acggcttcgccatttggtaaaagcattgccgcgctggaagaacaaattggctatacgctatttacccgcaaagataataatatcagcctcaata
aagccggacaggaactgtatcaaaaactgttcccggtttatcaacggctttctgcgattgataatgaaatccataactcagggcgtcgttcacga
gaaattgtgattggcatagataatacttatccgacaattattttcgatcagttgattagtctcggagataagtatgaaggcgtgaccgcccagcca
gttgagttcagcgaaaatggcgttatcgataatctttttgaccgccagttagattttattatctccccacagcatgtatctgcacgagttcaggaactg
gaaaacctgactatcagtgaactaccaccgctgcgattaggctttctcgtttctcgccgttatgaagagcgacaagagcaagagctgcttcagg
agctgccctggctacaaatgcgctttcagaaccgggccaacttcgaagcaatgattgatgcgaatatgcgaccttgtgggatcaatccaaccat
tatttatcgtccctacagttttatggccaaaatcagcgccgtggagcgcggacattttctgacggtgatcccgcattttgcctggcgactggtgaatc
cggcaacgctgaaatattttgatgcaccgcacaggccgatgtatatgcaggaatatctttattcaatcagaaatcatcgttataccgccacgatg
cttcagcatattgctgaagatcgtgacgggacgagtcattaacccagcatcgagccggttgcaattaaattacggtgtaagtcgaagacgtggc
taagatcgtggtgaatatgtccgccaggcgcattttccagatagcgatgcagataatcccgatacataggatgtgcacaattatcaatgatagtg
cgggcgcgttgaagcggggaaagaccgcgcagatcggcgatcccttgttcggtgatgatcactttgacgctgtgttcgctgtgatcaacatggc
tgcacattggcacgacggttgagatcttgccttctttagcaatcgacggggccataaagatcgacagatacgcgttgcgttcaaaatcaccgctg
ccgccgatgccgttcatcagatcgaccccggctacgtgtgttgagttggcatgcccgtaaatatcaaactccaggccgacgttcagagcgatg
acgcccagacgacggatgatttccgggttattggaaatctcctgcggacgcaacacaatgcggctggcaaagtaatccatattgtcgtaaatct
tgcgcagggaatcggccgagattgtcaggctggaggcgctggccccgctgattttgccggtttccagtaaatgcaccaccgattcctgtagcac
ttccgaatacatcataaacggaggaattaccgggtttcccccagacgcgccattaccgcattattgatattgcccacgccactttgcagcggca
gaaattccggcggaatacgcccatgcgccatttcctgcaataagaacgtgaccacgttatcggcaatctgctggcacatgggattttgcttatcc
agcatattaccggcgtcgggcaagttggtttccacgacggcgacaatctttttcggatcgatttgcacatagcgggtaccgacgcgatccattgc
atggaagatcgacacgctattgcgccgcggtggcgcgccaggaatcacaatatccgccagttctgcaacgcgcggatcgtgatagtgattga
gttcaatgatcactttcttcgcccgcagcagccaggtcggcgcattaccgatcccgctggttaaccagactcgaccatccggtgccagtgccga
tgcttcaatgacggcaacatcaatgtcgccgaagaaaccgtaattgaccatttgcgccacttcgctcaaatgcaggtcaacgaaactcaccgc
gccctgattgatctttttacgtaaaccggacgatgtttgatatggcgcacgccaggaaacagcatcggcgtcagaaagtacatcgtcagcggc
ggcgctgattgacgcacccgtcagaaggcgaatttgatacggctttttggcctcatgctgttcgttagctctgcgggcaatcgcggtgggtaggg
ctttcggcgaacccgccggggtaaagccgctaaatgccaccatgtcgttatgctggataatttctgccgcttcattggcggtcatccttgtccactg
agtttccatttttactccttcgttcgcagggattaatgaccaacgaaattaggtttacgtttttcgaggaaagcgttcatcccttcctggtaatcttcgct
gtcatacaccgcgcggcgcatcccctgaatacgttcaaattcatcggagttcatggtgtgtgcttcgcccagtacacgcagctcttctttgataacg
gcaatggctaacggcgctttctcagagatgtggtgcgccatttgtaaggtgaaatcttccagttcttccacttccacaacatggttgaggatgccg
acagccagcgcgcgctgggcggtgattggcgaagcggtaaaaatcagctctttgacaatgtggaagcccgcgtcgcgggtcaggttgtgaat
gccgaccaggttatacgggacgccgaggtttacaggcgtcattgagaaggttgaggtactggcggcgatgatcagatcggaactcatgatcat
ttcaaatgcgccaccccaaacactaccttccaccatcgaaatgatcggtttcgggaatttttggatcatgcgggtgatttgacgcaatggatcatc
ataggagagcggatcgcgaccgccagacggcagttcgtgaatatcgtgacctgcggagaagactttggatccactcggtgcgcgcaaaatg

atacagcgaatttccggccggttgagatcgcttaacgcctgcataagatcatcaataaagactttacttaaggcattaagttttcggccatagttaa
actcaatgaccgccactttgttgatagtgacaacgttaacatactgataagacataaaaattcctttaatcaaaatattgcgtctggataaattcact
gagctgccgcaggccggtgcgcggtgagagcgtattgtttttgaccgctaaaagcgtctggcggtaatagcgatcgaaatcttcattcgcgaac
aggtgattcagtacttcttcttcggtctgcttacgcagccattccaccgattgttgttgccgcacttgttgtaaacgaccactggcagttagcgcggttt
tgaagtcgatgatggcgtgccagatctcatcgattccacgtttttccagtgcgctacaagtcagaacccgtggctgccattcgtcgtatttacgtcg
cagaatatgcagggcactctcgtacatatgccgggcaatggcgacattggtatggttatcgccatcgtctttgttgataacgatcagatcagcca
cttccatcagcccttttttaatgccctgcagatcatcgccgccaccggcaatttgcaacgagataaaacagtccaccatgcgggcgacttctgttt
ccgactgcccgacgccaaccgtttcgacaatcactacgtcataacccgctgcttcgcataacagcattaattcccgcgctcgctgactggcacc
gcccagatgaccggaggatggtaccgggcgaataaacgccgcttcggcacgcgccaggtcattcatgcgggttttatccccgagaatgctac
cgccagtgaccgggctgctgggatcgaccgcaataaccgcgacctttaatccctctcgaatcaacaacatgccaaaggcctcaagaaaggt
acttttccccgcgccgggggtgccggtaacgcccagtcgcagggtgttaccgcagtacggcataatggcatcaagcagctgcgtacttagtgc
ctgatgacgcgggtgacggctttccaccagcgtcatggcctgggcgagtgtggcacgctcaccctgacgtaagcggcgaatactttctgccag
cgtggcttcattaatcatgatgctggcttatcagattcagtacgtcgcgcacactgtcgagcataggtgtacctggaccataaatcgccgccacg
ccgcgctcttgcaggaaggcgtaatcctgcggcggaatgacgccacccgcgaccacgcagatatcttcgcgtccccatttttcagcgcttcga
ccagttccgggatcagcgttttatgaccggcagccagtgaggatgcgcccactacgtgaacgtcgttttctacggccaggcgggcgatctcttc
aggtgtagagaacatcgggcttaaatctacgtcgaaaccgagatcggaataggcgctggcgatcactttcgcgccgcgatcgtgtccatcctg
gcccatcttagcgatcagaatgcgcgggcgacgaccattgtcggcaaggaactgctccgtttgcgcaacaatggcatcgaactcggaggcc
gatttctcagactgatgatagctttgcgcaatcacgccggtaacacactggcttggcaccagataacggtcgaaagcgacttccagcgcatcg
gaaatttcacccagggtggcgcgaacgcgagcggcattaacagcggcagccagcaggttttcgttatgctgtgcggcgtgagtcagggcgtt
caacgcggcggttacggcggcatcatcacgggtggcgcgaatgcgttccagcgaagcaatttgctcgttacgcaccatcacgttgtcgatctc
aagtacatcggtttcgtcttcgtgatccagtttgtacttgttgacaccaacgatgacacgcttgccctggtcgatcagcgactgttcgcgcgctgag
gcctcttcgatcattcgttttggcagacctgcttcgatcgctttcgccatgccaccggcttcgtcgatctgttggataatagctctggcttgtttgacgat
ttgatcggtcagcgactcaatgtaataggatccggccagtggatcgacggtgcggcagagttctgattcttcctggatgatgatctgggtgttgcg
ggcaatgcgtgctgagaaatcggtaggcaaaccaagcgcttcgtcaaaggcgttggtatgcagtgactgagtaccgcccagcgtcgcagcc
agcgcttcaatggtggtgcggataacgttgttatacggatcctgttcagtcaggctccagcctgaggtctggcagtgggtacgcagcgccagtg
atttcgggtcctgtgcgccaaatccactgaccgcttcgctccataaataacgtgccgcacgcaacatggcgacgttcataaacagatccatgcc
gatgccgaagaagaacgacaggcgaggagcgaagtcatcaattttcagtccggcagagattgctgctttgatgtactcaatcccatcagcga
gcgtaaatgctacctgctgcacgcagttggcacccgcttcacccatgtggtaaccgctgatactgatggtattaaatcgcggcatgttgccggaa
caccaggcgatgatgtcggcgataatgcgcattgacggttttggtgggtaaatataggtgttgcggcagaggtactctttgagaatatcgttttgaa
tggtgccggtcagtttatcaggtgtaacaccttgctcttctgcggcgacgatataaaacgccagtactggtagcactgcgccattcatggtcatcg
aaaccgacattttatccagcgggatctggtcgaacaggactttcatatcttccacggtgtcgatagcgacgcccgctttgccgacgtcgcccgcc
acgcgcgggttatcggagtcgtagccacggtgggtggcaaggtcaaacgcaacggaaagacctttttgcccggcggccaggttacggcgat
aaaaagcgttggactcttttgctgttgaaaaaccagcatactgacggatggtccacggttgggcggtatacatagtggcacgcgggccacgaa
cgtagggcggcaaaccaggaagggtacctgtcacctccagattatcgagatcggcttcggtatacagcggcttgatggcgatcccttccgcgg
tttgatgaaccagcgagtcgacagttttctcccgacggctcaattccttgttggcaagctgttgccactcctgcacgttagacattatttgctccgtaa
aacgcaatcaaaatgagggtatttgcgcctacggtgagctttttacgtgggtttgtcgccagcaaaaaaaaatgcattcagacgtcgtttttttgccgc
ctgagatttgtctattttgtgatgtgattcaaggaatacaaaaagaagtgaggacaacgcctgatatgtgctatcaggcattatttgatggattaatc
ctgacgaaggactttgtgaccataatcgagtaacgcatcagtgactttacgcatcatgcggctttcaggagcaaagcggtgccagtagagcat
ccgtcgttgaaatagcccaggcgttaagtcaatcagttcaccgctggccagctctttctcgatttgcaggtgcgggatcatacagcaggtggtgc
cctggcgagcaagttgtacgaacgcttctgaagaattaacgatatggcagggcacgctgcctggaggcagatcgaagttttgctgcaaaaag
gcctggtgcatatcgtcaagatggtcaaacgcgaccactggcgctttcagtaatgccgaacgcgttacgccgttagggaaatatttttcggcaa
agggttttgagctgacgaacagatagtcgagcgcaccaagtttatcgacaagacaactcggcagcgcctgatgttgaatactcaccgcgccg
accacttcgccgcggcgcagacgttcctgagtgcgggtttcatcttctacctgcaagttgaggcggataggcgaatcagccaacacaggagc
cagtgcaggaagcaaccacgtcgccagactgtcggcgttgaccgccagtgaaagcagcagcggagtcgaaccggtttgttcatcgcccag
ccactcttcttccagcaactccacctggcgcagcagtgccagcagtttttgcccttgttccgtcgggcgcggcggtacggtacgcaccaacagc
ggctgcccgaacatattttccagttgcttaatgcgctgtgagacggctgattgtgtaatgcacagcttttgtgccgcgcgctcaaatcctcgttcacg
tatcaccgcatccagtgcctgtaatgttctgtagtccgggcgtttcattgctgttgtctgctcctggtaaatgctttttcctgcactatgacacaattttgtg
tcaggttgcatataccgaagccgtcggcgggtaacatttgtatccgtggttaaaagcgcagcttaaaagatcagggatttgcgctaatgcatttcg
ctcagcgagttcgcgccctggttgtgttgaatggcgtggcgttattgcctcaatttgcctgtaaacaggggcttgcgaacggtgaactggtgcgcc
tgtttgcaccgtggagcggcatacccagaccgttgtatgctttatttgcggggcgaaaggggatgcctgccattgcgcgatattttatggatgagtt

aaccacgcggcttgccaacggggtctgaatcgctttttttgtatataatgcgtgtgaaatttcataccacaggcgaaacgatcatgacgcaggat
gaattgaaaaaagcagtaggatgggcggcacttcagtatgttcagcccggcaccattgttggtgtaggtacaggttccaccgccgcacacttta
ttgacgcgctcggtacaatgaaaggccagattgaaggggccgtttccagttcagatgcttccactgaaaaactgaaaagcctcggcattcacg
tttttgatctcaacgaagtcgacagccttggcatctacgttgatggcgcagatgaaatcaacggccacatgcaaatgatcaaaggcggcggcg
cggcgctgacccgtgaaaaaatcattgcttcggttgcagaaaaatttatctgtattgcagacgcttccaagcaggttgatattctgggtaaattccc
gctgccagtagaagttatcccgatggcacgtagtgcagtggcgcgtcagctggtgaaactgggcggtcgtccggaataccgtcagggcgtgg
tgaccgataatggcaacgtgatcctcgacgtccacggcatggaaatccttgacccgatagcgatggaaaacgccataaatgcgattcctggc
gtggtgactgttggcttgtttgctaaccgtggcgcggacgttgcgctgattggcacacctgacggtgtcaaaaccattgtgaaatgatctgacggg
ggaacctcccccgttaaaaaaattctcttcattaaatttggtgacatgtgtcacgcttttaccaggcaattgtcgattgctctaaataaatcctctaaa
ccagcatattcatccaagaattacctttgcgtgatatttcctcaacatcgcgacgcaaacgttcatattgccgcaatattattttttgatatgttgaaag
gcggatgcaaatccgcacacaacatttcaaaagacaggattgggtaaatggcaaaggtatcgctggagaaagacaagattaagtttctgctg
gtagaaggcgtgcaccaaaaggcgctggaaagccttcgtgcagctggttacaccaacatcgaatttcacaaaggcgcgctggatgatgaac
aattaaaagaatccatccgcgatgcccacttcatcggcctgcgatcccgtacccatctgactgaagacgtgatcaacgccgcagaaaaactg
gtcgctattggctgtttctgtatcggaacaaaccaggttgatctggatgcggcggcaaagcgcgggatcccggtatttaacgcaccgttctcaaa
tacgcgctctgttgcggagctggtgattggcgaactgctgctgctattgcgcggcgtgccggaagccaatgctaaagcgcaccgtggcgtgtgg
aacaaactggcggcgggttcttttgaagcgcgcggcaaaaagctgggtatcatcggctacggtcatattggtacgcaattgggcattctggctg
aatcgctgggaatgtatgtttacttttatgatattgaaaataaactgccgctgggcaacgccactcaggtacagcatctttctgacctgctgaatatg
agcgatgtggtgagtctgcatgtaccagagaatccgtccaccaaaaatatgatgggcgcgaaagaaatttcactaatgaagcccggctcgct
gctgattaatgcttcgcgcggtactgtggtggatattccggcgctgtgtgatgcgctggcgagcaaacatctggcgggggcggcaatcgacgta
ttcccgacggaaccggcgaccaatagcgatccatttacctctccgctgtgtgaattcgacaacgtccttctgacgccacacattggcggttcgac
tcaggaagcgcaggagaatatcggcctggaagttgcgggtaaattgatcaagtattctgacaatggctcaacgctctctgcggtgaacttcccg
gaagtctcgctgccactgcacggtgggcgtcgtctgatgcacatccacgaaaaccgtccgggcgtgctaactgcgctgaacaaaatcttcgcc
gagcagggcgtcaacatcgccgcgcaatatctgcaaacttccgcccagatgggttatgtggttattgatattgaagccgacgaagacgttgcc
gaaaaagcgctgcaggcaatgaaagctattccgggtaccattcgcgcccgtctgctgtactaattccccttctctgaaaatcaacgggcaggtc
actgacttgcccgttttttttatcccttctccacaccgttgccgcgttgcgcattctactttgcgagtctgctccagaaaataaatcaaagtcagatcaat
gcgttgtgtttatttaatggggatttacgatggcagggcagcatggggctgtaacggtaaagccctcaccgaagcgagggcttgaaggagaag
ggttatgatgcgacttgtcatcatactgattgtactgttactcataagtttcagcgcttattaacagtcagtctcaggggaggagcaatcctcccttac
ccttactcactaaattaggtcaaagaatcaacgatgtcaatcagggcgatgcgggtgtatcgcccttaccactcccagactttcgacggtgtaac
caccgcaggaagagggatatcccactcttcaacggggagtttttccaccaactgacaatcatgcgcataacccaccggttgcgttttatagtgct
gccagttttgtaaggtccgatcataaaaaccaccgcccattcccaggcgctgaccgtactcatcaaaggcgaccagcggtgtgatcagcacgt
ctaatcgggaaaggggtagcacgtcacgcacatccaattttggctcatggatcttcaacctgttcatcaccagttcgctttgcggatggtaattcag
gaacagcaaattaccggcactaaagggatgcaaaactggaagatatacgcgcttaccggcgcgccagagttgttctatgagtggctgggtgt
cgagttcgccatcaaaagagaggaatacagcgaccgtatgtgccatcaccaccgggggataagtcatcatccgggtagcggcttgttgaccc
atttcctgctgttgttccggcgttaacgcacgacgacgttgccgaatcattttgcggatttcttgtcgggataatgtcagtgggagttctggtagttgcg
tcatggctggtgccagataagaagggaatctccgagatgccgccgcaggctgtaacccttgaacccttggttcaaggtgaatgtgtcgtcgca
gttttaaggcttctcggacggaccgagcatgctcaccaaccgcggagcgccacattcttgtggtatgaaatatcggctcaggggactggcccg
cttgcgaacatctcagagaaattttgtcttcacggttactctaccacagtaaaccgaaaagtgtcattcaaagttttggttagtttttcggtgatgcga
ccttgttcaagtaacgcttgttctatggtctgctgcagcatccgaatacgctgttccatacttgccgcgtagtcacgagtctttgctttttcttgcgctaa
ctcatagctgatattcaatgcggcaatgaagaccaactgttcagtatttgtgactctagtgcgttctttcagatcttgcaaccgttggttcagatcgtc
cgctgcctgattcaacgcatccctttggtcaggcgggcagttcacacgcagtgaacggccaaaaatttggatatcgacgggttgtgcagacatg
ccaccttcctgctgattgactgcgctgccttcgtctttagacccatggtctgcgaaggggcgacactatagctaccctgatgagaagagacaag
ccctttctggtccaccagggcccaaagtggtagcatatcatgaatattcctccctttgacgacgaatgcttatgtctatacagaacgaaatgcctg
gttacaacgaaatgaaccagtatctgaaccaacaagggacgggtctgaccccagctgagatgcatggtttaatcagcgggatgatatgtggc
ggtaacgatgacagctcatggctaccgctacttcacgacctgacgaacgaaggcatggctttcggtcatgagctggcacaggcactgcgtaa
aatgcactctgccaccagcgatgccctgcaggatgacggcttcctttttcagctttatctgcctgatggcgatgatgtcagcgttttcgatcgggctg
atgcattggcaggttgggtcaatcacttcctgcttggtcttggcgttacgcaaccgaagctggataaagtgaccggcgaaaccggtgaagctat
cgacgatctgcgtaacattgcgcaactgggttacgacgaagacgaagatcaggaagagcttgaaatgtcgcttgaagagatcatcgaatac
gttcgtgttgccgcgctgttatgccacgacacctttactcatccgcaaccgaccgcgccagaagtacaaaaaccgactctacactaaaaacaa
aaaacgtaaggagagtgttatgagtgagatatcccggcaagagtttcagcgtcgccgtcaggccctggtggagcaaatgcaacccggcagc
gccgcgctgatttttgctgcaccagaagtaacacgtagcgccgacagcgaataccccctatcgtcagaacagtgacttctggtacttcaccggctt

taacgaaccggaagcggtgctggtgctgattaaaagcgatgacactcataaccacagcgttctgtttaaccgcgttcgcgacctgacggcgga
gatctggtttggccgtcgcttaggccaggatgccgcgccagagaaactgggcgttgaccgcgcactggcattcagcgaaatcaatcagcaac
tttatcaactacttaacggcctggatgtggtttaccatgcccagggcgaatatgcatatgctgatgtaatcgtgaacagtgcgctggaaaaactgc
gtaaaggttcgcggcaaaatctcaccgcaccggcaacgatgatcgactggcgtcctgttgttcatgaaatgcgcctgttcaaatcgccagaag
agattgccgtactccgccgcgcgggagaaatcaccgccatggcacatacacgggcgatggaaaaatgccgtccgggaatgttcgagtacc
atctggaaggcgaaattcaccacgaatttaaccgccacggtgcgcgctatccgtcctataacaccattgtcggcagcggtgaaaacggctgc
attctgcactacaccgaaaacgagtgtgaaatgcgcgacggcgacctggtgttgattgacgcgggttgtgaatacaaaggttacgctggcgat
attacccgcaccttcccggtcaacggcaaattcacccaggcccagcgtgaaatctacgacattgtgctggagtctctcgaaaccagcctgcgc
ctgtatcgtccgggaacttccattctggaagtcactggtgaagtggtgcgcatcatggttagcggcctggtaaaactcggcatcctgaaaggtga
tgttgatgaactgatcgctcagaacgcccatcgtcctttctttatgcatggccttagccactggttaggactggatgtccatgacgtgggtgtttatgg
tcaggatcgctcgcgcattctggaaccgggcatggtactgaccgtagagccagggctgtatattgcgccggatgcagaagtgccagaacaat
atcgcggtatcggcattcgtattgaagacgacattgtgattaccgaaaccggtaacgaaaacctcaccgccagcgtggtgaaaaagccgga
agaaatcgaagcgttgatggttgctgcgagaaagcaatgagcgtaatcatcgtcggtggcggcatggcgggcgcgacgctggcgctggctat
ttcccggttaagtcacggggcgctgccggtacatttgattgaagcgactgcgccagagtcacatgctcatccgggctttgatggacgagcgata
gcgctggcggcgggtacctgtcagcaactggcgcgcatcggcgtctggcaatctctggcggattgcgcaactgccatcaccaccgtgcatgtc
agcgatcgtggtcacgctggatttgtcaccctcgccgcagaagattaccaactggcggcgctgggacaggttgtcgaattgcacaatgtcggg
caacggctgtttgcattgctgcgtaaagcacctggcgtaacgctgcattgccctgatcgcgtggctaacgttgcccgtactcagagtcacgttga
agtgacgctggagagtggcgagacgctgacgggccgcgtgctggtagcagctgatggcacccattcagcgttagccaccgcgtgcggcgtt
gactggcagcaggagccttacgaacaactggccgtgattgccaacgttgctacttccgttgcgcatgaagggcgcgcttttgaacgctttacgc
aacatggcccgctggcgatgttgccgatgtctgacggacgctgttcgctggtctggtgtcatccactggaacggcgcgaagaggtgttgtcgtg
gagtgacgagaagttttgccgtgaactccagtcggcctttggctggcgacttgggaaaattacccacgctggtaaacgcagtgcttatccgctg
gcgttaacccacgccgccagatctattacccatcgtaccgtgctggtgggcaatgcggcgcaaactctgcacccgattgccgggcaagggttt
aacctcggtatgcgagatgtgatgagtcttgcggaaaccctgactcaggcgcaggagcgcggagaagacatgggggattacggcgtattgt
gccgttatcagcagcgtcgacagagcgatcgcgaagcaaccattggcgtcacggacagccttgtacatctttttgccaaccgttgggcaccgct
ggttgtcgggcgcaacatcgggctgatgacgatggaattattcaccccggcacgcgatgtgctggcgcagcgcaccctcggttgggtggcgc
gttgaaggcggttttaaggaataaacaatgcaaagtgttgatgtagccattgttggcggcggcatggtggggctggcggttgcctgtggcttaca
ggggagcggcttacgcgttgccgtactggagcagcgcgtacaggaacctctggcggcgaatgcaccaccacaactgcgcgtttcggctatc
aatgccgccagcgaaaaattactcacccgtcttggcgtctggcaggacattctctctcgtagggccagctgttatcacggtatggaagtgtggga
caaagacagctttggtcacatttcgtttgacgatcaaagcatgggctatagccatcttgggcatatcgttgaaaattcagtgattcactacgcgctg
tggaacaaagcgcatcagtcgtcagatatcactctgttagcccccgcagaattacagcaggtcgcctggggagaaaatgaaaccttcctgac
gctgaaagatggcagcatgttaacggcgcgtctggtgattggcgcggacggcgctaattcctggttgcgcaacaaagccgatattccgctgac
tttctgggattatcagcatcacgcgctggtagcgaccattcgcacggaagaaccgcatgatgcggtggcgcggcaggttttccatggcgaagg
cattctggcctttttaccgcttagcgatccgcatctttgctcgattgtctggtcactgtcgccagaggaagcgcagcggatgcagcaggcaagtga
agacgaatttaatcgcgcgttaaatatcgcttttgataatcgcctgggcttatgcaaggttgagagcgcgcgtcaggtgttcccactgacggggc
gttatgcgcgccagtttgcctcgcaccgtctggcgctggtgggcgacgccgcacataccattcacccgctggcggggcagggggtaaatctcg
gctttatggatgctgcagagctgattgccgaactgaaacggttgcatcgtcaggggaaagacatcgggcagtacatttatctgcgtcgctatgag
cgtagccgcaagcacagtgcggcgttgatgctggctggtatgcagggattccgcgatctgttttccggtaccaatccggcgaaaaaactgctgc
gtgatattggtttgaaactggccgacacgcttcctggcgttaagccgcaacttatccgccaggcaatgggattaaacgatttgcctgaatggctg
cgttaaaaatttctcctctgttgtttatttgatacccatcacactttcatctcccggtttttcgccgggagattttcctcatttgaaataaactaatttcacct
ccgttttcgcattatatttctaatgccattatttttgatttagtgtttttgacatttttttagctcttaatattgtcttattcaaattgactttctcatcacatcatctt
tgtatagaaactggtgtatttttggtttttattctgtcgcgatttttgcatttttaaccataagctaatgtgatgatcaattttaccttatggttaacagtctg
tttcggtggtaagttcaggcaaaagagaacgattgcgttggggaccgggagtggctccgatgctgggtttcgtggtgataatttcaccatgaaaa
agttgtcagccccgcttattcaatgaggacaagatggcacaacagactcctttgtacgaacaacacacgctttgcggcgctcgcatggtggattt
ccacggctggatgatgccgctgcattacggttcgcaaatcgacgaacatcatgcggtacgtaccgatgccggaatgtttgatgtgtcacatatg
accatcgtcgatcttcgcggcagccgcacccgggagtttctgcgttatctgctggcgaacgatgtggcgaagctcaccaaaagcggcaaagc
cctttactcggggatgttgaatgcctctggcggtgtgatagatgacctcatcgtctactactttactgaagatttcttccgcctcgttgttaactccgcc
acccgcgaaaaagacctctcctggattacccaacacgctgaacctttcggcatcgaaattaccgttcgtgatgacctttccatgattgccgtgca
agggccgaatgcgcaggcaaaagctgccacactgtttaatgacgcccagcgtcaggcggtggaagggatgaaaccgttctttggcgtgcag
gcgggcgatctgtttattgccaccactggttataccggtgaagcgggctatgaaattgcgctgcccaatgaaaaagcggccgatttctggcgtg
cgctggtggaagcgggtgttaagccatgtggcttgggcgcgcgtgacacgctgcgtctggaagcgggcatgaatctttatggtcaggagatgg

acgaaaccatctctcctttagccgccaacatgggctggaccatcgcctgggaaccggcagatcgtgactttatcggtcgtgaagccctggaag
tgcagcgtgagcatggtacagaaaaactggttggtctggtgatgaccgaaaaaggcgtgctgcgtaatgaactgccggtacgctttaccgatg
cgcagggcaaccagcatgaaggcattatcaccagcggtactttctccccgacgctgggttacagcattgcgctggcgcgcgtgccggaaggt
attggcgaaacggcgattgtgcaaattcgcaaccgtgaaatgccggttaaagtgacaaaacctgtttttgtgcgtaacggcaaagccgtcgcgt
gatttactttttggagattgattgatgagcaacgtaccagcagaactgaaatacagcaaagaacacgaatggctgcgtaaagaagccgacg
gcacttacaccgttggtattaccgaacatgctcaggagctgttaggcgatatggtgtttgttgacctgccggaagtgggcgcaacggttagcgcg
ggcgatgactgcgcggttgccgaatcggtaaaagcggcgtcagacatttatgcgccagtaagcggtgaaatcgtggcggtaaacgacgcac
tgagcgattccccggaactggtgaacagcgaaccgtatgcaggcggctggatctttaaaatcaaagccagcgatgaaagcgaactggaatc
actgctggatgcgaccgcatacgaagcattgttagaagacgagtaacggctttattcctcttctgcgggagaggatcagggtgaggaaattta
tgcctcaccctcactctcttcgtaaggagagaggttcacaattcactgcacgtttcaggaaccatcgctcatgacacagacgttaagccagcttg
aaaacagcggcgcttttattgaacgccatatcggaccggacgccgcgcaacagcaagaaatgctgaatgccgttggtgcacaatcgttaaac
gcgctgaccggccagattgtgccgaaagatattcaacttgcgacaccaccgcaggttggcgcaccggcgaccgaatacgccgcactggca
gaactcaaggctattgccagtcgcaataaacgcttcacgtcttacatcggcatgggttacaccgccgtgcagctaccgccggttatcctgcgtaa
catgctggaaaatccgggctggtataccgcgtacactccgtatcaacctgaagtctcccagggccgccttgaagcactgctcaacttccagca
ggtaacgctggatttgactggactggatatggcctctgcttctcttctggacgaggccaccgctgccgccgaagcaatggcgatggcgaaacg
cgtcagcaaactgaaaaatgccaaccgcttcttcgtggcttccgatgtgcatccgcaaacgctggatgtggtccgtactcgtgccgaaacctttg
gttttgaagtgattgtcgatgacgcgcaaaaagtgctcgaccatcaggacgtcttcggcgtgctgttacagcaggtaggcactaccggtgaaatt
cacgactacactgcgcttattagcgaactgaaatcacgcaaaattgtggtcagcgttgccgccgatattatggcgctggtgctgttaactgcgcc
gggtaaacagggcgcggatattgtttttggttcggcgcaacgcttcggcgtgccgatgggctacggtggcccacacgcggcattctttgcggcg
aaagatgaatacaaacgctcaatgccgggccgtattatcggtgtatcgaaagatgcagctggcaataccgcgctgcgcatggcgatgcaga
ctcgcgagcaacatatccgccgtgagaaagcgaactccaacatttgtacttcccaggtactgctggcaaacatcgccagcctgtatgccgtttat
cacggcccggttggcctgaaacgtatcgctaaccgcattcaccgtctgaccgatatcctggcggcgggcctgcaacaaaaaggtctgaaact
gcgccatgcgcactatttcgacaccttgtgtgtggaagtggccgacaaagcgggcgtactgacgcgtgccgaagcggctgaaatcaacctgc
gtagcgatattctgaacgcggttgggatcacccttgatgaaacaaccacgcgtgaaaacgtaatgcagcttttcaacgtgctgctgggcgataa
ccacggcctggacatcgacacgctggacaaagacgtggctcacgacagccgctctatccagcctgcgatgctgcgcgacgacgaaatcct
cacccatccggtgtttaatcgctaccacagcgaaaccgaaatgatgcgctatatgcactcgctggagcgtaaagatctggcgctgaatcaggc
gatgatcccgctgggttcctgcaccatgaaactgaacgccgccgccgagatgatcccaatcacctggccggaatttgccgaactgcacccgtt
ctgcccgccggagcaggccgaaggttatcagcagatgattgcgcagctggctgactggctggtgaaactgaccggttacgacgccgtttgtat
gcagccgaactctggcgcacagggcgaatacgcgggcctgctggcgattcgtcattatcatgaaagccgcaacgaagggcatcgcgatatc
tgcctgatcccggcttctgcgcacggaactaaccccgcttctgcacatatggcaggaatgcaggtggtggttgtggcgtgtgataaaaacggca
acatcgatctgactgatctgcgcgcgaaagcggaacaggcgggcgataacctctcctgtatcatggtgacttatccttctacccacggcgtgtat
gaagaaacgatccgtgaagtgtgtgaagtcgtgcatcagttcggcggtcaggtttaccttgatggcgcgaacatgaacgcccaggttggcatc
acctcgccgggctttattggtgcggacgtttcacaccttaacctacataaaactttctgcattccgcacggcggtggtggtccgggtatgggaccg
atcggcgtgaaagcgcatttggcaccgtttgtaccgggtcatagcgtggtgcaaatcgaaggcatgttaacccgtcagggcgcggtttctgcgg
caccgttcggtagcgcctctatcctgccaatcagctggatgtacatccgcatgatgggcgcagaagggctgaaaaaagcaagccaggtggc
aatcctcaacgccaactatattgccagccgcctgcaggatgccttcccggtgctgtataccggtcgcgacggtcgcgtggcgcacgaatgtatt
ctcgatattcgcccgctgaaagaagaaaccggcatcagcgagctggatattgccaagcgcctgatcgactacggtttccacgcgccgacgat
gtcgttcccggtggcgggtacgctgatggttgaaccgactgaatctgaaagcaaagtggaactggatcgctttatcgacgcgatgctggctatc
cgcgcagaaattgaccaggtgaaagccggtgtctggccgctggaagataacccgctggtgaacgcgccgcacattcagagcgaactggtc
gccgagtgggcgcatccgtacagccgtgaagttgcggtattcccggcaggtgtggcagacaaatactggccgacagtgaaacgtctggatg
atgtttacggcgaccgtaacctgttctgctcctgcgtaccgattagcgaataccagtaattcactgattcgactattttctaaaggcgcttcggcgcc
tttttagtcagatgacaaagtacaaaagtgctcagacagtcccctcgcccctttggggagagggttagggtgaggggaacaggccggcactg
gcgcgaatatttaccctcaccccggccctctccctgaaagggcgaggggaaaagcgtgccaacattgaagattgagccagtttgttagcaat
ctcaaagatacgtcaacgaattaatttttctcggaaaaacaaatggctatagcacttgtgactggtggcagtcgcggcatcgggcgggcaactg
cattactgttggcgcaagaagggtatacggtggcggttaattatcagcaaaacctccacgcggcgcaggaagtgatgaacttaataacgcaa
gccggtggcaaagcattcgtgctccaggcggatatcagcgacgaaaaccaggtcgttgcgatgtttacagcaatcgatcagcacgatgaacc
gctagcagcgctggtcaataacgccgggatcttgtttacccagtgcaccgttgaaaaccttaccgcagagcgaatcaaccgagtactttccacc
aacgtgacgggatattttctctgctgccgcgaggcggtaaaacgcatggcgcttaaaaatggtggcagtggcggcgctatcgtcaatgtctcttc
ggtggcctcacggttgggttcgccaggggaatatgttgattacgcggcatcgaaaggggcgattgatacgttaaccaccggactatcgctgga
agtcgccgcgcaggggatccgcgttaactgcgtgcggccagggtttatttataccgaaatgcacgccagcggcggcgagcctggacgcgtc

gatcgcgttaagtcgaacatccccatgcagcgtggtggacaggcagaagaggtcgcgcaggccattgtctggctactaagtgataaagcctc
ttacgtcacgggaagttttatcgatttggcgggcgggaaataaaacagggaagttgtctgaccggatgcaacaagtattgcatccggtacttcat
cgacttaaagcttctcgccgttgctggcaatcacctctttgtaccagttaaagctcttcttacgtgaacgcgacatatcgccagtaccgtcgtcatgtt
tattcacatagataaagccgtagcgtttgctgtactgcccggtggtgaacgacacgcagtcgatgcagccccacggtgtgtagcccatcagatc
cacgccatcgtaagtcaccgctttttcatctcttcgatatgggcgcgcaggtagtcaatgcggtagtcgtcgttgatgctgccatcttcttccactttg
tcgtaagcgccaaaaccgttttcgacaataaacagcggcctctgataacgctcatacagttcgcaaagtgcatagcgcagacctactggatca
atctgccagccccagtcagatgctttaacatacgggtttggtacgctgccttcaaaaccagagatcgcatcgccggtgccgccttcggccttcac
tgcattggtcatgtaatagctgaaaccaagataatcgcaggtgccttcacgcagcacatccagatcgccgtcttccattttgatgttaaatccgcg
acgctcccactcgttcaacacataggacgggtaatagccgcgtagctgcacatcggtaaagacgtagcgttcgcgcatcgactcctgagcga
acatcacatcgtccgggttacaggagtaaggatagagcggcaccatcgccagcatacagccgactttcatctccgggttaatgcgacgcgca
gctttcaccgccagggcgctggcgacaaactggtgatgcagcacctgatacatcgtctcttccgggttttcatgctcggtatacaccacgccgga
gcagcagtaaccgaacagcggtgcacgccagttacgctggttgttaatttcgttgaaggtcatccagtatttgactttgtgcttatagcgttcaaata
ccacttccgcgaaacgtacaaagaaatcaaccactttacggttggtccagctaccgtattgctgcaccagatgcagcggcatttcaaagtggg
agagggtgatcaccggttcgatgttgtatttcagcagttcatcgaacatatcatcgtagaacttcagcccttcttcgtttggctgagcttcatcgcctttt
ggaaaaatgcgcgtccaggcaatggatgtacgaaaacatttgaagcccatttcggcaaatagcttgatgtcttccttatagtgaccataaaaatc
aacggcttcatggtttggatagtattttcctggcaagacttctttggtgatttcgcgcggcacgccgtgtgcgccaccggtcagaacgtcacaaatg
ctcggcccttttccgcctttgttccagccgccttcgacctgatgagcggcaactgcgccgccccataagaaatctttcggtaaggtgagtttttcac
tatcataggctccagatgagtcaattcttacaggaaatagtaccatgcagccaaacgacatcactttttttcaacgtttccaggatgacattctggc
tgggcgtaaaaccatcaccatccgcgacgagtctgaatcgcacttcaaaacgggtgatgtgcttcgtgtcggacgttttgaagatgacggttattt
ttgcacgattgaagtcaccgcaacctcaaccgtaacgctggatacgctgactgaaaaacatgcagagcaggaaaatatgaccctgactgaa
ctgaaaaaagtcattgctgacatctatcccggtcagacacaattttatgtgattgaatttaaatgtctttaaattgaactcacgcctgttagttgaaatt
tcgcatttaatttcatgatttttaggagattagtctcaaatcagaatttattttagctaacaggtgttcactggaactattctcagttacgctggagaggt
atacatgtgcgtgtacgtttctccggagtaagttatggttcagaagcccctcattaagcagggatattcactggcagaggaaatagccaacagc
gtcagtcacggcattgggttggtgtttggtatcgttgggctggtgttgctactggttcaggcggtggatcttaatgccagcgccacagcgataacca
gctacagcctctatggcggcagtatgatcctgctgttcctcgcttcgacgctctatcacgccattcctcatcaacgggcaaaaatgtggctgaaga
aatttgaccattgcgctatttacctgttgattgccggaacctacacgccgtttttgctggtggggctggattctccgttagcgcgcgggttgatgattgt
tatctggagcctggcattgctgggtattctgtttaaactgaccatcgcgcaccgattcaaaattttatctctggtgacctatctggcgatgggctggct
gtcgctggtggtaatttatgaaatggcagttaagctcgcggcgggcagcgttaccttactggcggtaggcggcgtggtttattcgctcggggtgat
tttctacgtctgcaaacgcattccatacaaccatgccatctggcacggcttcgtgctcggcggtagtgtgtgccactttctggcgatctatttgtatatt
gggcaggcgtaatgggtatgcctttgtaggcctgataagacgcgccagcgtcgcatcaggcatcggagcacaactgccggattcggcgtga
acgccttatccgatctacgggcctcgtccctgttgtgataaaggcatcaaatcccagacatgataagacgcgccagcgtcgcatcaggcgata
aatgcctgatgcgataaagagatttactcttcgagcgaatacggcagcggctcgatatgcaatgtattcgcatcgtcgcgtacgcggaagatgc
tatccggttccatatcgttattcatgacgacctgtaccacgacctgaccatcttccagttttaccgcagccagtaccgtaccggtacgacgccagtt
ctcgcccattttcagctctaagtcttcaccagcttccggcagtcggctggcgctacctgccagcaaccagagcgcacgtttattggcaccacgga
attttgctcgcgccaccatctcttgtccggtataacagcctttcttaaagctgataccgcccagcgcctggagattggtcgcctgtgggataaactg
cccgctgttggcggcatcaatcaccgggaaacccgcttcaatgtttaatgccagccactgttggctattgttcagttccgcttcaccgcgcagtttat
cggtcagcatattagcagtagcttcatcggttacgatcaggaaacgttctgccgggtgttcaaaccatagcaaagtggtcgcgccttctttgacta
cctgtttttctttcgaaggcagttcgctaaagagatttgccagcgcggcgcgcgcctgaaaaccggcaacaccaagcagcacacgctcgtcgt
ctggcgcgatggtcactttagagaataccgcatatttttcagttcagtcagctgcggttcacgcacgctgcgccgttcaatccatgcaaagccat
cgccgtcgcggaacagacgtaaattgctccacattttacctttggcgtcgcaatgggcggcgagcaggtgctgatcttctgccatctggctgaca
tctgctgtcacctgaccctgcatatatttttcgctgtccgcgccagtaatggtggcaagcgcccagtcatcaagcgtcatcagcgtcagcggtaaa
cgggcagaagccgtaggctgacggggaggaaaaggtgtaaaagccataatagagtcctgagttgcttaacggcgtattaatgactaatggta
aaagagctatgaggcaatgcaagtgctttcactacgcgatctgtgccttctgcgaggcgacttccaaaaaaatgaagccattgaattgttgtgaa
ttaatttcgcgatcgcgatggtgtttactgatattcccgtaaaagaaggcgaaaaacacgttacaatagccgggtactggattttcgtgagacag
gaagaagatggacattaacaacaaagcccgcattcattgggcatgccgccgtggtatgcgcgaactcgatatttcaatcatgccgtttttcgaa
catgagtacgacagcttaagcgatgacgaaaaacgcatctttattcgtctgctggaatgtgacgatccggacctgtttaactggctgatgaatca
cggtaaaccggccgatgcagaactggaaatgatggtccgactcatccagacacggaaccgggaacgtggtcctgtggcaatctgatttgcgc
gtctcctggcgcgcacagtggctttccttgctgattcatgggctggttgccgctgttattttactcatgccctggccactcagttacaccccgttatgga
tggtgttactttcgctggtggtgtttgattgcgttcgcagccagcggcgtattaatgctcgccagggggaaattcgcttgttgatggacgggcgtttgc
gttggcaagggcaggagtggagcatcgtcaaagcaccgtggatgattaagagcggcatgatgctgcgtttacgttctgatggcggtaaacgg

caacatttatggctggcagccgacagcatggacgaagctgaatggcgggatttacggcggattttgttgcaacaagagacgcaaagataaa
gatgacggcaaagcatatgcgctaagccgtcagtgagatcaggcgtaatgctctgccatttcgttgaggatttgctcgcaccagctctgaatac
gctcgtcgctaaggtcatactggttagtttcatccagcgccagacccacgaacagttgcccgtcagcaatcaccggtttcgggctggtaaattca
tatccttccgttggccagtagccgacgaacttcacgcctttggtcgagagtttgtcatgcagcataccgagcgcatcgaggaaccactcgccgta
tcccagttgatcgccaagcccatacagcgcaacaattttaccttcaaggttcaggtcgtcgagctgatcccagacggcttcccagtcttcctggat
ttcaccaaaatcccaggtcgggatacccagaatgagcacatcgtactgctccattaatttcggggagtcgtccttgaggttatgtaaggtcacca
gttctgggccgataatatcgcggatttttctgccgccatttcggtgtaacaggtgctggaaccgtaaaaaagacccatattcataacgtaagcttc
tcgattctgctgtggcttttgcggctagtgtatcagaatcgctttagtcacggcacaataaggcataatggatcaaatgagtgaccataaggggc
gcaagtgaaacaggatctggcacgcatcgagcagtttcttgatgctctgtggctggaaaaaaatctggctgaaaatacgttgaacgcttaccgt
cgcgatctgtcaatgatggtggagtggttgcatcaccgcgggttgacgctggcgacggcgcaaagtgacgatttgcaggcattactggcagaa
cggctggagggcgggtataaagccaccagctcagcgcgtttgctgagtgcagtgcgccgattgttccagtatctttatcgcgaaaagtttcgtga
agacgatcccagtgcgcatctcgcttcaccgaaattgccccagcgtttgccaaaagatttaagcgaagcgcaggtcgaacgtttattacaggc
accattaatcgatcagccactggagctacgcgataaagccatgcttgaagtgttgtatgctaccgggctgcgtgtctctgaactggtcggactga
caatgagtgatatcagcctgcgtcagggcgtggtacgggtcattggtaaaggcaacaaagagcgtctggtgccgttaggtgaagaggcggttt
actggctggaaacctatctggaacatgggcgtccgtggctgctgaatggtgtgtcaattgacgtgttgtttcccagccagcgtgcgcagcagatg
acgcgacagaccttctggcaccgtattaaacattatgctgtgctggcgggtatcgacagcgaaaagctgtcaccgcatgtgttgcgtcacgctttt
gccactcatttattaaatcatggtgcggatttacgcgtggtgcagatgctactgggccacagcgatctctccaccacgcaaatttatacgcatgtc
gctaccgagcgtctgcggcaacttcatcaacagcatcacccgcgggcgtgatgtctgaaaagaacgggaagatttatgaagaaaggttttatg
ttgtttactttgttagcggcgttttcaggctttgctcaggctgatgacgcggcaattcaacaaacgttagccaaaatgggcatcaaaagcagcgat
attcagcccgcgcctgtagctggcatgaagacagttctgactaacagcggcgtgttgtacatcaccgatgatggtaaacatatcattcaggggc
caatgtatgacgttagtggcacggctccggtcaatgtcaccaataagatgctgttaaagcagttgaatgcgcttgaaaaagagatgatcgtttat
aaagcgccgcaggaaaaacacgtcatcaccgtgtttactgatattacctgtggttactgccacaaactgcatgagcaaatggcagactacaac
gcgctggggatcaccgtgcgttatcttgctttcccgcgccaggggctggacagcgatgcagagaaagaaatgaaagctatctggtgtgcgaa
agataaaaacaaagcgtttgatgatgtgatggcaggtaaaagcgtcgcaccagccagttgcgacgtggatattgccgaccattacgcacttgg
cgtccagcttggcgttagcggtactccggcagttgtgctgagcaatggcacacttgttccgggttaccagccgccgaaagagatgaaagaattc
ctcgacgaacaccaaaaaatgaccagcggtaaataattcgcgtgaaacaacagatacaacttcgtcgccgtgaagtcgatgaaacggcag
acttgcccgctgaattgcctcccttgctgcgccgtttatacgccagccggggagtacgcagtgcgcaagaactggaacgcagtgttaaaggtat
gctgccctggcagcaactgagcggcgtcgaaaaggccgttgagatcctttacaacgcttttcgcgaaggaacgcggattattgtggtcggtgat
ttcgacgccgacggcgcgaccagcacggctctaagcgtgctggcgatgcgctcgcttggttgcagcaatatcgactacctggtaccaaaccgt
ttcgaagacggttacggcttaagcccggaagtggtcgatcaggcccatgcccgtggcgcgcagttaattgtcacggtggataacggtatttcctc
ccatgcgggggttgagcacgctcgctcgttgggcatcccggttattgttaccgatcaccatttgccaggcgacacattacccgcagcggaagcg
atcattaaccctaacttgcgcgactgtaatttcccgtcgaaatcactggcaggcgtgggtgtggcgttttatctgatgctggcgctgcgcaccttttg
cgcgatcagggctggtttgatgagcgtaacatcgcaattcctaacctggcagaactgctggatctggtcgcgctggggacagtggcggacgtc
gtgccgctggacgctaataatcgcattctgacctggcaggggatgagtcgcatccgagccggaaagtgccgtccggggattaaagcgctgctt
gaagtggcaaaccgtgatgcacaaaaactcgccgccagcgatttaggttttgcgctggggccacgtctcaatgctgccggacgactggacga
tatgtccgtcggtgtggcgctgttgttgtgcgacaacatcggcgaagcgcgcgtgctggcaaatgaactcgatgcgctaaaccagacgcgaa
aagagatcgaacaaggaatgcaaattgaagccctgaccctgtgcgagaaactggagcgcagccgtgacacgctacccggcgggctggca
atgtatcaccccgaatggcatcagggcgttgtcggtattctggcttcgcgcatcaaagagcgttttcaccgtccggttatcgcgtttgcgccagca
ggtgacggtacgctgaaaggttccggtcgctccattcaggggctgcatatgcgtgatgcgctggagcgattagacacactctaccctggcatga
tgctgaagtttggcggtcatgcgatggcggcgggtttgtcgctggaagaggataaattcaaactctttcaacaacggtttggcgaactggttactg
agtggctggacccttcgctattgcaaggcgaagtggtatcagacggtccgttaagcccggccgaaatgaccatggaagtggcgcagctgctg
cgcgatgctggcccgtgggggcagatgttcccggagccgctgtttgacggtcatttccgtctgctgcaacagcggctggtgggcgaacgtcattt
gaaggtgatggtcgaaccggtcggcggcggtccactgctggatggtattgcttttaatgtcgataccgccctctggccggataacggcgtgcgc
gaagtgcaactggcttataagctcgatatcaacgagtttcgcggcaaccgcagcctgcaaattatcatcgacaatatctggccaatttagcgtca
tcttctctataaaaaagagcgtggattgggtacaatcccgctcttatcaccgcattttgactagctcaataaaagaaatcagaccatgtttgaaatt
aatccggtaaataatcgcattcaggacctcacggaacgctccgacgttcttaggggtatctttgactacgacgccaagaaagagcgtctgga
agaagtaaacgccgagctggaacagccggatgtctggaacgaacccgaacgcgcacaggcgctgggtaaagagcgttcctccctcgaag
ccgttgtcgacaccctcgaccaaatgaaacaggggctggaagatgtttctggtctgctggaactggctgtagaagctgacgacgaagaaacc
tttaacgaagccgttgctgaactcgacgccctggaagaaaaactggcgcagcttgagttccgccgtatgttctctggcgaatatgacagcgccg
actgctacctcgatattcaggcggggtctggcggtacggaagcacaggactgggcgagcatgcttgagcgtatgtatctgcgctgggcagaat

cgcgtggtttcaaaactgaaatcatcgaagagtcggaaggtgaagtggcgggtattaaatccgtgacgatcaaaatctccggcgattacgctt
acggctggctgcgtacagaaaccggcgttcaccgcctggtgcgtaaaagcccgtttgactccggcggtcgtcgccacacgtcgttcagctccg
cgtttgtttatccggaagttgatgatgatattgatatcgaaatcaacccggcggatctgcgcattgacgtttatcgcacgtccggcgcgggcggtc
agcacgttaaccgtaccgaatctgcggtgcgtattacccacatcccgaccgggatcgtgacccagtgccagaacgaccgttcccagcacaa
gaacaaagatcaggccatgaagcagatgaaagcgaagctttatgaactggagatgcagaagaaaaatgccgagaaacaggcgatgga
agataacaaatccgacatcggctggggcagccagattcgttcttatgtccttgatgactcccgcattaaagatctgcgcaccggggtagaaacc
cgcaacacgcaggccgtgctggacggcagcctggatcaatttatcgaagcaagtttgaaagcagggttatgaggaaccaacatgtctgaac
aacacgcacagggcgctgacgcggtagtcgatcttaacaatgaactgaaaacgcgtcgtgagaagctggcgaacctgcgcgagcagggg
attgccttcccgaacgatttccgtcgcgatcatacctctgaccaattgcacgcagaattcgacggcaaagagaacgaagaactggaagcgct
gaacatcgaagtcgccgttgctggccgcatgatgacccgtcgtattatgggtaaagcgtctttcgttaccctgcaggacgttggcggtcgcattca
gctgtacgttgcccgtgacgatctcccggaaggcgtttataacgagcagttcaaaaaatgggacctcggcgacatcctcggcgcgaaaggta
agctgttcaaaaccaaaaccggcgaactgtctatccactgcaccgagttgcgtctgctgaccaaagcactgcgtccgctgccggataaattcc
acggcttgcaggatcaggaagcgcgctatcgtcagcgttatctcgatctcatctccaacgatgaatcccgcaacacctttaaagtgcgctcgca
gatcctctctggtattcgccagttcatggtgaaccgcggctttatggaagttgaaacgccgatgatgcaggtgatccctggcggtgccgctgcgc
gtccgtttatcacccaccataacgcgctggatctcgacatgtacctgcgtatcgcgccggaactgtacctcaagcgtctggtggttggtggcttcg
agcgtgtattcgaaatcaaccgtaacttccgtaacgaaggtatttccgtacgtcataacccagagttcaccatgatggaactctacatggcttacg
cagattacaaagatctgatcgagctgaccgaatcgctgttccgtactctggcacaggatattctcggtaagacggaagtgacctacggcgacg
tgacgctggacttcggtaaaccgttcgaaaaactgaccatgcgtgaagcgatcaagaaatatcgcccggaaaccgacatggcggatctgga
caacttcgactctgcgaaagcaattgctgaatctatcggcatccacgttgagaagagctggggtctgggccgtatcgttaccgagatcttcgaa
gaagtggcagaagcacatctgattcagccgaccttcattactgaatatccggcagaagtttctccgctggcgcgtcgtaacgacgttaacccgg
aaatcacagaccgctttgagttcttcattggtggtcgtgaaatcggtaacggctttagcgagctgaatgacgcggaagatcaggcgcaacgctt
cctggatcaggttgccgcgaaagacgcaggtgacgacgaagcgatgttctacgatgaagattacgtcaccgcactggaacatggcttaccgc
cgacagcaggtctgggaattggtatcgaccgtatggtaatgctgttcaccaacagccataccatccgcgacgttattctgttcccggcgatgcgt
ccggtaaaataagcattacgttatgctcacaaccccggcaaatgtcggggtttttttatttaagctgggtaaatgcagataatcgttttctggcttcgc
gatttgtcgcctgcatcaccatccacggactgaacgcccacggcgtggcatcaataccgtgtaatacatctgctaaatcacaccattgataatcc
atcacttcatcatcattgatctgtaacgcactagtggtgcgtgcggcaaataccggacacacttcattttccacaatgccactcggatcggtggcg
cggtagcgaaagtcaggatagatagattcaggaggcgtaatttccacgccaagctcataacggcaacggcggatcactgcgtcttcgttgcttt
ctcccagttgtgggtgcccacaaaccgagttagtccacacgccaggccatgctttttttgctcagtgcgcggcgggtaactaataattgtcctttgg
cattaaacagccaactggagaacgcgagatgtaagcgggtgtctgccgtgtgtgcggcatacttttccagcgtacccgtgggaactccctgtgc
attcaataaaatgacgtgttccgtttgcataatttctcacatgtaattctgatcactcatcattatagataattgaagagtgaaatgattaaccaagtg
agcgtttatcgacaaccgcccgttttgagcggatgccgacaggtaaaaaccatttaaaaaagtagcatcatatatttgaagcgactttcaatggc
cattgcaacacagtcagaaatgacaggaatagagagagtaaaaagcaacaacagactgaatatagcgcgcattaaaaaattcatattattct
ccatgctcatttttcttcagcttgcgcatcagaagccacatacgtactgtcctgacttttaaatctttatcagagacggattccttcaccaggtcagca
tgatgctgatagccattaaagaagacgttcagcacaacggcgctaagggttgcgagcataatgccgctatgtagcagcggttgcagtacggc
cggtaacttagaaaagaaatcgtgagagagcgtcggagtcatgccaacgccgagactgatcgccacaatatagaggttgtaacggttggtg
gtgtagttacagcgcgacagaattcgaatccctgtcgccagtaccatgccgaacatcactagaccagcgccgcccagcacaaattgcggaat
ggaggctaccagcaccgccatttttggcaccatgccaaacaggattaatataattcccgaagaaatacacacccagcggctatgaacgcgc
gtcacgctaaccaggccaacgttttgagaaaatgacgtgtgggggaagctgttaaacgtgccgcctatcattgtccctacgccatcgacacgc
agcccgcgaataatatcgtgcgaagagagtttacgaccgactatttcacccagtgccaggaacatccccattgactcgataaacacgatgatt
aacacggcagtcatggtcagaatggaaacgggatcgaaaatcggcataccaaacgacatcggcgtaacaatcgcaaaccatgaagcatc
atgtagcccggataaattgacttcattcatcatccacgaaagtaaaaagccaaatacaatccccagtaatacggcgacgttggacataaatcc
tttcgcatagcgagtaatgagcaagataaaaattaagacggcaaaggagatacctaaataaacgggattaccatattgcggattccctttacct
ccggcggcccagtcaatacccacctgaatgatgctaagcccgatagaagtaataaccacaccggtaaccagtggcgggaataaaggcatc
aagcgaccgataagtggcgctaataatgtggtgataaaacccgcggcgatagtggcaccaaatatccccagcaggccgatatccgggttca
tcccaatggctatcattggtgttacagcagcaaaggtcaccgacataatcaccggcaggcggatccccataaagcggccgataccgatacat
tgcaataatgtgacgatcccgcagcaaaagagatccgagctaatgagcatcgcaatagcttcttttgagaggcccagtcggtcaccaatcata
agaggaacagcgactgcacctgcgtacatgacaaggacgtgttgcagaccgagtatgatcagctttcctggtgataatatgcggtcaacctca
tcagttgggcggtcttgcccagaagatgagggaagttgggaatctatggcgctcatcagtgtttctcctttcgcccgccagagtaaaaagcaac
cgtttacgttaatgaaactgtctaaacacatgctcgtggagtgccagtttttgcgaggaaggggagttagagacaggaattgcggaggcaatca
cataatcgtggttaaaccacgaaaatgagaagagaacctgacaatggaattatctgtaattttctcatattgatatttcattaatcattctggcatgtc

gcactctcgcatttaatcgtttttatctggatagcgctcttttgatcggcaatgttgaaataatgaaagcggcatagattgaggggcaataatttgaa
ctcagtcgcaaaattataattaaaacggtcatgctacctaatatatgcccgtttattgcatcgcgaaatttgagaagacataactcattaaatgaa
agggatgcaaatgaataagtttattgctgctgaagctgcggaatgtataggctgtcatgcttgtgaaattgcctgtgcggtggcacacaatcaag
aaaactggccgctgagtcacagtgactttcgaccgcgtatccacgttgtagggaaaggccaggctgcgaatccggtggcctgccatcactgc
aacaatgccccttgcgttacggcttgtccggttaatgctctgactttccagtccgatagcgtacaactggacgagcaaaaatgtattggttgtaaa
agatgcgcaatcgcttgcccctttggcgtcgttgagatggtcgatacgattgcgcagaaatgcgacctttgtaaccagcgcagttccggcacgc
aagcctgtattgaagtttgcccaacacaggcgttacgactgatggacgataaagggttgcagcagataaaggtggcccgccagcgcaaaac
ggcagcaggaaaagcgtcatcagacgctcagccatctcgcagtgcagcgttgctccccgttaactcgcgtaaaggcgcagataaaatttcag
cgagtgaacggaaaacccactttggcgaaatatattgcgggctggatccacaacaagcgacttatgagagtgaccgctgtgtttattgtgccga
aaaagctaactgcaactggcattgcccgctgcataacgctattccggattacatccgtctggtacaggaaggaaagattattgaagcggcaga
actttgccaccagaccagttccttaccagaaatttgcggcagggtatgtccacaggaccgtctttgtgaaggcgcatgtactttgaaagatcactc
gggcgcagtctctatcggtaatctggaacgctacatcaccgataccgcgctggcgatgggctggcgtcccgatgtcagcaaagttgttccccgt
agcgaaaaagtggcggtgattggcgctgggcctgcagggttagggtgtgctgatattctggcgcgcgcaggagttcaggtcgatgtctttgatc
gccatccagaaattggcggtatgctgacttttggcattcctcctttcaaactcgataaaacggtattaagccagcggcgagagatattcaccgca
atgggaatcgactttcatcttaactgtgaaattggccgcgatattacctttagcgatttaacttctgaatatgatgcagttttcatcggcgtggggactt
acgggatgatgcgagcagatctgccgcatgaagatgcgcccggtgtcattcaggctctaccgttcctgactgcccatacccgccagctcatgg
gattgccggagtctgaagagtatccgctgacggacgtggaaggtaagcgagtcgtggtattgggcggtggcgatacgacaatggattgtttgc
ggacttccatccgcctcaatgccgccagcgtgacctgcgcgtatcgtcgtgatgaagtcagtatgccgggctcgcgcaaagaggtggttaatg
cgcgcgaggaaggtgtcgagtttcagttcaatgttcaaccgcaatatatcgcttgtgacgaagatgggcgtttaactgcggtgggcctgattcgta
ccgccatgggtgagccggggccggatggtcgccgtcgtcctcgtccggtagcgggttcagagtttgaattgcccgccgatgttctgattatggcc
tttggtttccaggcgcatgccatgccgtggttgcagggcagcggaattaaactcgataaatggggcctgattcaaaccggtgacgtcgggtattt
acctacccagacgcatctgaaaaaagtctttgctggtggtgatgcagttcatggcgcggatctggttgtcactgcaatggccgcaggaaggca
ggcggcgcgcgatatgttaactctgtttgatacgaaggcatcgtgatgaaatcgttaattatcgttaatccggctgactgtattggctgccgtacttgt
gaagtagcctgtgtggtcgctcacccttcagaacaggagttgaatgccgatgtctttttaccccggctgaaggtacagcgactggacagcatca
gtgcgccggtgatgtgccatcagtgtgaaaacgccccttgtgttggcgcttgccccgtgggggcgctgacgatgggcgagcaggtggtgcag
accaattctgcccgttgtattggctgtcagagttgcgtcagtgcgtgcccgtttggaatgatcaccattcagtcgttgccgggggatacccgacaa
caaattgtgaaatgcgatctctgtgaacagcgggaagaggggccagcctgcgttgaatcttgcccaacacaggcgctgcagttgcttactgaa
agagaactcaggcgagtccgccagcagcgtattgttgccagcggtgagaatcctctctgaaagaaatctccgcgctgaaaggcgcggaga
atctattagattgcccaaccacccgcgtaaaatgcgaccagtgcggcagtaatgatcaccgtaccaatattcagcttttgccattcgcgtgcaaa
gacgcggcctacgaccagtgtcacaaagcccagcataataccggtaacgatattacaagtcagaacgatgaacacggcacacaccaggc
cagccatagcgtcaataaaatcattgaaatccagcttcgagacgttacttaacatcagcaaacctacgtacatcagagcgggtgcagtggcgt
aaccagggatcaaaaatgacagcggtgataaaaacagaattaacaggaataacgcccccactacggttgcggttaaacctgttttacccccc
ggcggcggttcctgccgctgattcgatataaaccgctgcgggcgctgcgcccaccaggccggagaatattgaacttactgagtcactggtcag
ggctttgccgccgttgatgatctggttgtctttatccaacaaattcgcctgaccggcgacggcacggatggtgccagtagcgtcgaacactgcgg
tcatcaccaatgccagtacactcggaagtacagttggctggagtgcacccataatatcgaggctgaaaatcagagacttaccatcttcgccagt
caggcttggcatcgccaccagaccgtggtatttcaccgctgggtcaaagattaagccgatgatcgaaattgcaataatcaccaacaagatccc
gccgggtacgcgacacttctccaggccgaagatgaccgccagccccagcaagctcatcatcaccgggaaggaggtaaacgcaccgagc
gccactggcaagccttcaatcgggtttttgataaccatacccacaccgttagcagcaatcagcagcagaaacagcccgataccgatacctgta
ccgtgagcgatacccatcggcaaattacgtaagatccaggtacgcacaccggttacggaaatggcggtgaagatgacgcccatcagaaat
accgcgcccagtgcgacaggaacgctaatttgttgcccgagtaccagactgaatgcggtaaacgccgtcaaggaaatcgcgcaaccaatc
gccattggcaaattagcccataatcccatcagcaacgagccgaagcccgcgaccagacaggtggcaacaaacacagctgcgggaggaa
aacctgctttgcccagcattcccggaacgacgataacggaataaaccatggccagaaaggtcgttaagccagccagtacttcctgacgaac
ggtactgccacgagcggtaattttaaaataattatccagcgcgccctgtggctttggtgcgtccggtgtttgtaggatgtctccagacataatgatgt
cctcacgaggatgttaaagttttattattagttgcgttcgtacaccagacgaccatcaacgtaggtgcggtagatcgaacggtcatcgcccaacg
tcatcatcacgaacaatttgtcgactaaagaaacagagttgtcatagcgcagctgctgtagcggagtggcggtgggttccatcaccacgaaat
cagcctctttgccaggtaaaaagttgccaatcaaatcgtcaaggcccagagatttcgctccgccgagcgtggccaggtaaaacgcttcatatg
ccgagaggcgatagccttgtaattgcaatactttgtaggcttcgttcagcgtttgcagcatgttgaaagtggttccggcaccgatatccgttcccat
gcccactttaactttcttctgccatgctttttcaagttgaataagccgctgccgaggtaaaggttggaggtcggacagaaagcaatgctggatttg
gtttcgctgagacgatcccactctttttcttcgagatggacgcagtgagcaaagacacagtttttaccggtcaggccgtactgatggtaaacatcc
agataaccatcatggtcaggataaagcgatttcacccaggcaatttcatctttgttttcacagagatgggtatgtacccacgtatccggatattcttc

tttcaggcgttgcgccatcgccatctgttcaggagatgaggtcggggcgaagcgtggcgtaatcgcatatagcagacgaccatttttgtgccag
cgttcgatcagttctttgctttggtgatagctgctttcggcagtgtcgagcagataatccggtgcgttgcggtccatcatcaccttaccggcaatcata
cgcatattgatatgactggcggcttcaaacagcgcatcaacagattgcggatgaacagtgccaaacaccagcgccgtggtggttccgttacgt
aaaagctgcttgatgaagaacgccgacatttcgcgggcgtactctaaatcctcataacgacgttcagtagggaaggtgtgtttattcaaccactc
cagcaattgctcaccataggcccccaccatttcactttgcggataatggatatgtgtatcgacaaagcccggtactatcagtttgccgcgatagtc
gcgcacgcgaatggtgtcaggaatttgatgctttccgttttcccattcgccaaaccattccacttttccctgtttaatgagtaataaaccatcctcaat
aaaccgcagcgcagaggcaatctcttccgggttatcgatcgtacgggtgacatcaataaaactgcctcgtaccgcttttaacgtgtgttctcctga
catcattgactcctttactgttaatccatctcttccgctgaggtaataccaggcagaacgttttcctgtcggtagccaccagggaggataatatttaat
aaaatcgcagttaacccgccagcacaaatagggttttcaactaatacataaatagaggctggcaatattttaaaaatttcgggatcgtaggaga
cgccaagccctaaacctaaagaagtggcgacaataagtgtttcacggcgctttaaaccgttggtgatgatgatgcgaatccctgcgatggcaa
tcatggaaaacatcaacgtcattgcgcctcccagaactgccgagggaatggtcgtgaagaagccgccaatcatcggaaataagccgagga
taaccagcattaccgcgatggttcgcccgacataacgtgaagcgacgccagtcatctgaataaccccattattttgcgcaaacgtggttaatggt
aatgaaccgacagcggaggcgataacagaaaccagaccatctgccagcacgccgcctttcagccgggactgatactcttccccctgaatgg
ggcggcgggaaaccattgccgtggcggtgatatcgccgactgcttccagcacgctaagcagataaatcgtgccaaccaccaggaactgatg
gaagctaaaactaaagccgtatttgaacggatgcgggatggtgattaacggcaaattgcgcatactgctgaaatccaccatgcccaggcata
acgatgcgatatagccgacacatagcccaatggcgatccctcccatgcgtagcaacggactgcgacagcagttaaagccgatcaccacaat
taaaaccaataaaccaacgccgagatgttcgtaattgccgaacgtaccgctgcttttggctgcaaatcctccaccaaaatcgataatgccgact
ttaatcaggcttaagccgatcatcagtaccacaataccgctgacggtaggcgtaataacccgacgtaaatagggcaagataaatgaagatcc
gacaaccagaaatgcgccaacgaaggagacgccgagaagcgacgacatgattaactcttcgtgaaaaccgtcgcttttcatgctgctgccc
agcgcaatcatgaccgtaacaaatgaaaaattgactgactgaattgagagtaggccagaaccgacgatgccgtagcggtttacttgtaacca
ggtaccaataccagaggcgatcatcgccatagaaacaagataggcagttgtttcagcggaaagctgtaaggccgcacccacgattaacgc
gggggttaccatcggaacgaaaattgccaacagatgggtaatggcaccaacgagagcctgatgaaagggagggcgatcctccagttcaaa
tataaggtcagaacctgcatggtttatatcagacatcccttcactccttttaaagtaaatttgatcatctgaattagtaaaataaattcccacgaatttg
gggcgcaggacgccttgagaacggcttatctcattttcgcagtcactatttagtcatcgttcaaacagagaaacaggccgccgaagcggccatt
tattatttatatttttccagcgcagtgagtattttctccggtgtgaaatgccattcgcgtaaccagatgccgcatgcatcgtgaattgcggtagcaatc
gccggagctgcgccatttacaccgatttccgagatcgatttcgccccgaacgggccgactttatcgtcgctcggcaccagcacagcgcggaa
atcacgcggaatgtcaccaattttcggtgcgccgtaactgcgtaaatcacgcgttaacgggtgaccttcggcgtcataaatgatctcttcgctcat
actgtggccgatagcacgcagggtggcaccgtagatttgtcccaacgctaactctggattgaccggtgtaccgcagtccagcaaggcgtaga
atttatccaggcggatttcacccgtacgcgtgttgacggcaacttcagcgaagtttgcgccatacgggaaggcgaaatcaggcgtgatataact
gccagttcccaccagtgaaccaaagccggtgccggtttcgcctttatgggcaatatccccgaaactaacttcgcctttcttgccgcgcacgacgc
ccggcgttgccagttgaacatctgccactggctcacccaacatttgcgcgccgtggaacagaattttctcccgtagattttccgctgccaaacgc
gccgcgttacccgagaagcaagtaccggacgaggcatatgcgcctttatcaaacaacgcatgatcggtatcaccggagataacatgcacgt
cctgcggtgggcagtgcagcacttctgctgccagtttcgtcactacagtatccagaccagtaccaatatccgcaccgccagaatgaacgataa
aggtgccgtccgattccagtttgatcatgcagttagcctgatcgatatccgggatacccgatttctgcatgataatggcgacgccgcgaccgatgt
gccagtcaccattttgcggtttcggtgacgaccattggatcatctcgcgaccctgacgcaggatctcttccagtgcacagctggcggcggaagg
aacggaggtcggcgctttaccttcaccgattgcaccgagaattttcagctcttgcccttcgtgtacccggttacgttcgataatttccagttggtcgat
ctgtaactgttcagccagttccgctaatgccatggtgatagcgaagttacctttcggtgcgccataaccctgataagcaccatttgggcaaatgttg
ctgtagtaggtggtgacctggaaatcgacgttatcgcacggatataacggcagcgacagcgccggtccgttacacggtacggtgagtgagtg
gttgccgtaagggccagtgttggcgcggaaatccatcttcactgccgtcaggcgaccatctttttttcgctcccagtttgacggtgactttcgcgacg
tgacgagaggtgttagcaataaactcttcttcacgggtgtagcggaacagtaccggacgcccggtcacgcaggttgcccaggcgcacacttct
tccagcaggatgtcctgtttggaaccaaaaccgccgccaactcgctctttaatgacatgaactttatgctgtttcatgcccacgaggcgcgcgac
ctggcggcgtaagtgccatggtacctgggtggaggcgtggataaccagacgatcgccgtccatacgagtaaagcagatatgtgtttcagtcgg
gcactgctgcgcttgcgttgagttataggttcgctcaatgatcacatcggcatcggcaaagcctttgtccatatcgccaatatgaccatgaatactg
gcggcgatatttttgcgagggcgagaaccgatcgggaagttgatgatcatatgctcgccgcgctgggctgcatggctgttatcgtcttccagagt
atctggcgcaccagcaacatacaccaccggttcatcgtgcacgacaggcgcatcttccgccattgcttcgtcgatcgacattaccggcttaagc
acttcatattcaacgtcgatgagcttcagtgcttcgagcgcaatttcttcactttctgcgacgaccgcagcaacacgatcgccgacgtgacgcattt
tcttgccgaacatacggcggtcaagcggtgacggttccggtgcgctctgaccacccggggtatagtagatatccgggcaattcaggtgagtaa
taacgtgaacgacgcccggtaaggcttcagctttgctgacatccagatgagtaatcagtgcgtgagcgtgtgggctacgtaacattttaatgacg
caggcgtcagccgttacgcggtcttcaacatagcagggtttcgcctgcaccattttcgcggcatcagttttaggataatgcttaccaatgacttcta
ggtcgtcacggaaagtcggagcgatatcaatggtggcctgcggattatttttacgtgcaaccgccagttcaatgacctgatagtattgctgccag

ccagcatcgcggctgaacaaaccagaaagcgcgtcgtcgatctcttcgcgggtaggtgcggcgatgcgatcgagaagatcggtgataatca
gagctgcagctggatcgttataaccagactgcaccacgccaacatcaaccattgcctgttgaaccagacttaactcgttccatttgcccagagat
tctgcggtacgaatatctgccttctctaactgtgcggcaataagcaaggacgcgttaacgatattaccgttaaagattattgcgtcagaaccggc
aaacccgaaaccatcatcactgttgcgtacagagtgcattcccatgttaaacaacagcttttgcacgttttcgcctggattaacggttagctcctga
ggcgcgccatttaaagtaaagtggatgatcatacggcttcctccccagcctgttggcagtcggcatacaggtcggctaccagaactcccgtgat
atagcgtttataggccacgctgccccgcaaatcttcctgcgggaaaatggcgttggcgacagcctgttcaagtgcattgccttccagattttgttttt
cgacatcatgcagacgcagtgctttactggcgacgccatccagcgcaattcgcataccgtcgtggtctgtcattgcaacggctgccgtcacgac
ggttaaacctgcctgagaacggctaattttgcgggtcgcacaggtgcgatacggatctttaatgataatttcggttagcaggcgatcgcatgggc
aggccaggtagtcctcgattgacagcgtttcgccgttgccaaaaaccagttcagcatccagtgccaacaggacgggaagcagcaccgactc
ttcctggcgggcggcgatttcaccaccaatggtcgactgattacgaacatggcgtgagtaaacaaaaccgagggcttcacgcagcgctgcag
gaataaatcgcgcatcacgcagtggctgcaagcgagacattgcgccaatccgcagtgcaccgttatcccagtcaacccagtccagttccaga
tcctgtaaggaaatggcaatctttttatcggtacgggttggtgtagcattgagtttgctgcccccggcgaaccagacggcttcatcctggtagcgg
cgcttcagttccagcgcctgttcgacagagtcgggcctgaaaaattgttcaatcatcttctttccttatttaaccctcttccctgcgggaagagggca
tctgtcatttatgccagcgcatccatccgacgccacatactggcagcggcttttctggcttgtgcataaatggaatcgcaatcgaagttaaactga
cggtcttcatagaccatcacaccattgaccatcacgctgtgaacgctgcctgagcccataccgaaagcgatatgcccggcgatattgtctgcca
gcagcggcgtcggcgagttgtaatcgcaaatggttaaatcagctttgtaaccggcttccagaagcccaaatttcgcgccaaagttgcggctcat
cagttcgttaccgttagtcagggctttggcaaaactgtcaggccacagcggaccacccgcatcgcgatgtttaaagaaggcaaatttcatctctt
caaacatgtccgaaccaatgccgtccgttcccaacgccagattgcggatgtcgctaaggtgatggttgtagccgacatggttgttcatgtttgaac
gggcgttatgcaccaggaacgcatcgcgctgattgagtagggtgatgtcatctttcgacaagtacagcccatgagcgaccagcgttttgctgtc
gatgagatcgaattgcgccagtcgtgccagcaggtctttgccgtaccagtggtgactgtaggaaacgtcgtaaaggtcttccgcagcgtgaata
tgcaaaccacggcctgtggctttcacggcttcacgcagcatctccagaccggcatccggcacggtaaacggcgcgtgagcaccgatatgtgc
ttccaccagatacggctcgcttgtcgctttcttcgcctcatcaatcaaacgggcgaaacggatgttttcttctacaccttcctgcaactctttgatgcc
gttgttacggtcagtagtttcaaaacaggtcatcgcgcgcaggccaacttttaaaaatgcgtcgcgcaatgtggagagcgacccgccgatata
cgccggagaggcatggtgatcgataaccgatgtacatccgctcttaatcgcttccagggaacaaatcagtccgctgtaatagagcgactcttca
tcaagggcgcgatcgagccgccaccagagatttttcagcgttgagatgaaatccgggcaaggggcgatgtttgccataattccgcgggaaag
ccccgagtaaaaatggttgtgcgagcagacaatccctggcatcacaatccggccatgcatctctttgaagctggcgtcggggtagcgttgcgtc
agggcatcgccgatagcgacaatcacatcgttttcgatggcgatatcaacgccttcctgcacttttgccgggtgtagctgtaccgcagtgacattc
ttcagaatcaacatgattacacctccacgcggcccagcagataatgatgatgctggtggacatggctgatgatgcggcacatatcgttcagctc
cggtggtacgttgttaaactgacctttgctgtcgatgtttaacacccagctttggttattcagacgtactcgtacccggcaatcttccacaaggaagc
ctgggttgctgctgttatcaaagtcttgcgccaggctgaagacggtgattttgtctttgtacggtttaccgttccacggacagaactgagcgcagttg
ccgcattcgttacagtaagcgtcgaggtgcagcgtctggaaacggttctggaagcctgggaccgcaatggagacgttggcgcggttcgggca
gacatccacacacttgctgcaaacgtagttacattcgaggcagcgagcggcttcctgggcgacaaacgcgtcacgatcgtcactgttcaccag
cgtgatagagatatcgcctttacgttgatagatttccgctggattgacgttgttccagtatttatcgttctggtgggaacggatattttcccgactaagg
atggcatcggtcgcccgacgcgcggttccgacagcagcgacaatggaggatggtccgcgctgtacgtcgccgatcataaagacgtcagtca
gacgagtttcgccattatggtcgacgtctggccagccgtttttgtccagcggcacgcccatcgcattcagggcttcagtatcctgctgttcaccaat
ggcggtgatcaggctgtctacaagcagtgttactgtttcattggtttcaaccggacgacgacgacctttctcatccggttcgccaagcgacataac
gcgcaaggttaaggtgccatcagcatcgaaacgttccggattattcaggaaacggaactctacgccgtcgtgcaacgcttcttcatactcttcgc
gccatgcgggcatctcttgtagtgaacgacggtaaacgatcgttgctttttctacgcctggaacgcgtaacgccgcacgagcgcagtccattgc
ggtgttacccgccccgacaacgaccacatgtttgcccagcttgagcgctgtacccttgttgtattcacgcaggaaggggagtgatttccagacat
tttggttgtcgcccgccagtttcacaccgctatttttatcagtgccggtggcaatcagaacatagtggaagccctgatttttaactgctcaatggtta
aatcgggtgagcagccatactcaaatttcacgccgtgagcggcaacaaaatcgatatcgtgctgaattaactctgcaggaatacggaactga
ggaatgatatttttcaccacgccgcccgcattggcttcgcgttcaaacagcgtaaccggatggcccgctctggcaaggaagtaacctgctgcca
gacccgccggacctgcaccaatcacggcaaccggatggcgtgaaccagaaccggctggtttgtgccagcgttgcttatattcatcccaaccttt
ttccagcgcgactttttttcagttcgcggatattcagcgcactgtcgtaatccaggcgggtacagttgtattggcactggtgatcgcaaatatgaccg
gtaatggcgggcagagcgttgcgttggtagatgagttccagcgcgtcggcatagcggtgttcgccaagcagacggatgtattccggaatatctt
gcttgatagcgcaggcagtaacacagggggcaacgtagcagtcggtcagcggcaggtcttctgccacttcaatacgctcttctggcttccagtg
tttctgggtgtattccatggttaacgcatctgctgccagtctgttcagtcgttcgacgtcaacatggtcaagtccccaggcgtcggagccttccagct
cgcgcatgcaggcacttaagcgcagatagccgccaggtttcagcaggtcggttgccatagtaatagggcgaatacctgtatcaaaaatatcg
cggatagtcagctgactggcaccaccggaataagaaatgggcagtttgccgtcaaaggcgcgagagagaactgctgcaacattgatggag
agcgggaacagcgcacggcctgacatatacatctcttcaccaggcagtgcgcctttattgttgatggtgccgagagtgttagtcagttttacgcca

aagccgagtgatttttcttttgccagtgccatcaggcgttccagcatttccagtgcttgcgtcagcttgaggtcgtgatcaaatgactcttcttttaagc
ctatgtaaccgaaaccgcagacatcgaggatctcacgaacacgcgcgtaccccagtaaggtcgggttaagtttcacaaaggtgttgagccctt
tttcttccagcatgtagcggcaaatggcttcgatttcatgcggaggacagccgtgcatggtggagagggtgacgccatgcaccatactggtggg
gatgcgagcgggtaaggcttgcaagctttcgcgtttttcctgcaatccgtggcgagctaaaaatgcgtcatcctggagtaatttattcagcgtatcg
cgatattgagcgaatttcggatggtcagatgcgtccatcatattgtcgatgaactgttgcatcggcggttgcttaataccttcgaggttgtaaccgac
gctcatattaaagatgaacgatttaccggaatcagaaggctggaacatcgcttcgagaaggtgcagggcaaaccaggctttgaggtattcatc
ccaggctttaagcagggtaaactcggtagaccattcggtgttaaagcactcgtcttcggcatcgatacagggcttttccagctccaggcggtcaa
gaatttggacggtttttagttcgatgaagcgtccgccagtcagccaggaagtgacaatattttgcgcgagctgcgtgtgcgggccagcggcagg
gccgacgggagtggcacaggtttcaccgaagacgctaacagttttaccttttacaggtgagtaaaactgttgctcgggaataccaaagattgag
cgttgttgttggtattcatcaaatatgcgcgtcaaaagttcctcaaacggaatgggacgcataatatcccccataaccctctccttagcttatacag
atatcattgagtgaccggtgttgagaaatccggtggtttagggggatagagcaacaatcgcaccactcttgccaggtttggcgggaaatataaa
aaaatgtgaggcggctcgaaacttaagggctttttgtgaatgaactcacaggtgctaaatgagaattaaaatcactcctgaaaattattattgcca
gcccatttatcaatttttctcttttctcaaaatcagtgattccttatcattatgataatgagtgccggaaggattaaatatttgaacgcaatcgtttaacta
cgttttctaaatttcagtatacctttccttcgctgtaataaagtcgtccggagtatcaatatctaaaattatttctgcattttctatttcgacggaataatgat
ctcccataaggagtgcctgacgcatattggtgacattgggtcgttggattgcctgcatcaggcatggttttgacactaaaatcggatggccgggg
atgccattatggagtggcagtattgcgccatcatttcgtaacgaccagatttttctaaaaatatcgatggtgagggttggcatgtcaccgtgggtga
gaaaacaatgttctgtttgcactgcgggtactgcggccttgacggatgttagtaaaccctgcgcataatctgggttgtgaataatagtgatattgct
ctggttcgcataacgttcgtgcagttcattaccacgatagccggtgactaaaataattcggctacaaaactgcaacgcatttttgatacttgtatca
agaattgttccctgttcccagggtaacatcattttccattgccccattcttgatgataatccggcagcggtaattatacagtcgatggctgacatagt
ggttccattatttataggggctaataacgcctgataattgcgttacttcctgatggattaagttggggataatttatcccagagaggtcataaagact
cgtgaaaagtatagttgacccatcggcattagtcattgatttaggtgcgcagaaacgtcccactgtgatttctgttgttggtgctgggggaaaac
cagcttgctttttggctggcagaactgctccaggctagcggcaggcgtgtgttaatcacgacgacgacacatatgtttatgccaacgtctcactg
gcccgtggttttctgtcgtgatcccgccatgcttcctcatgcgtctcttacatcccccatttcattttgttttcacagctggaaagcgaaccagggaaa
agtgcagggatttacgccagaagcgattgatgcactggtgcaacgaccagagtgtgacgtaattctcattgaagcagatggctcgcgtggaat
gccgttaaaagcgcctgatgagcacgaaccttgcatacccaaaagcagttgctgcgtgattgctgtgatgggaggacatactttgggcgcgaa
agtgagcacagaaaatgtccatcgctggtcgcagtttgctgatattaccgggttaacacctgatgcaaccttgcaactgagcgatctcgttgcgct
ggttcgccaccctcagggggcgtttaaaaacgtaccgcaaggttgtcggcgagtctggttcattaaccgtttttctcaatgtgagaatgcgattgc
acaaagcgagctccttcaaccgctgcaacaacacgacgtagaggcaatctggctgggcgatatacaagaacatcctgcaatcgcgcgcag
atttgtgaattagcggcccgggtattccgggttactgaaaggatatccattgaggttggtatgaatattttcacagaggctgcaaaactcgaagag
caaaattgtccgtttgctatggcgcaaattgttgatagccgaggctcgactccccgccattctgcacaaatgttagtgcgcgccgatggttctatcg
tcggtacaattggtggcggaatggttgagcggaaggtgattgaagagtcgcttcaggcattgcaggaacgtaagccgcgattattccatggac
gtatggctcgtaacggtgcggatgctgtcgggtcagattgtggaggtgcaatgtcagtatttatcagcgtccatggtatgcgtccacgtctggtgtt
gattggcgcggggcatgtcaaccgggcgatagcccagagtgcggcgctattaggatttgatattgccgttgctgatatttatcgcgaaagcctca
atcctgaactattcccaccatcaaccacgcttctccatgctgagtcgtttggtgcggcagtggaagcactggatattcgccctgataattttgtcctg
attgccacgaataatcaggatcgtgaagccctcgacaaactcattgaacagcccattgcatggttggggttgctggcaagtcgtcgcaaggttc
agcttttcctgcgtcaattgcgtgagaaaggcgtggctgaagaacatattgcccgtttacatgcgcccgttggttacaacataggtgcggaaacg
ccgcaggagatcgccatcagcgtgctggcagaaatattacaggtgaaaaataacgcgccgggtggctgatgatgaaaccttctcatccttcc
ggacaccagctggtggtgattcgcggtgcgggagatatcgccagtggtgtggcgctacgtctgtatcatgcgggttttaaagtgatcatgctgga
agtggaaaaaccgacagtgattcgttgtaccgtggcgtttgcccaggccgtgttcgatggcgaaatgacggtcgaaggcgtcactgctcgcct
ggcaaccagctctgcggaagcgatgaaacttaccgaacgcggattcattcctgtgatggtagatcccgcctgttcattgcttgatgaactgaaac
cgctttgcgtggtggacgctattctggcgaaacagaatttgggaacgcgggcagatatggcaccagtaacaatcgcgcttgggccgggcttta
ctgctgggaaggattgtcatgcggtaattgaaacaaatcgcgggcactggctcggtcaggtgatttactctggttgtgcgcaggagaataccgg
tgttcctggcaatattatggggcataccacccgacgggtgatccgtgctcctgctgcaggcattatgcgatccaacgtgaaattaggcgatctggt
gaaagagggcgatgtgattgcctggattggtgagcatgaaattaaagcaccgttgacggggatggtgcgtggcttgttgaacgacggcctggc
agtggttggtggttttaaaattggtgatatcgatcctcgtggtgaaaccgctgatttcaccagcgtttctgataaagcccgggcgattggcggcggc
gtacttgaggcgttaatgatgttgatgcatcagggcgtgaaagcgacaaaagaagtgctggaagtggcttaaatgaaaatggccggatggtat
tacccacccggcctgagactgatgacaaacgccaaattgcctgatgcgctacgcttatcaggcctacaagtgcattgcaatctattgaatttgcg
atgttttgtaggacggataaggcgctcgcgccgcatccggcatcaacaaagcgcacttacttgacaatctttggccggatggtattacccaccc
ggcctttaaattactgacaaatcaccgttcctgttttgccttcaatcccttctttcgctttgctcaatacggtaatcagtgcttcacgacctgcgcgaga
gcgagcaaacgaggcggcagcttccactttaggcagcatagaacctttcgcaaaatgaccttcttcgataaagcgttcagcatcacttaatgac

aggcgatcgagccactgttcattctctttaccgaagttaatggccactttttctaccgcagtcagaatgatcagcatatcggcatcaatcatttctgct
aaacgggcgctggcccagtctttatcgataaccgcgctggcaccacgcagatggttaccttcacgaataacagggataccaccaccgccaa
cggtgatcaccacctgacctgcatctaccagagctttaaccgtttctttttcaatgatatcaaccggttttggcgaggcaacaacacggcgatagc
cgcgaccagcatcttctttcagagtgtaaccctgttttgtcagttgctctgcttcttgctcggtaaagaacgaaccgatcggcttggtcgggttgagg
aatgccggatcgttagcgtcgacttcgacctgagtaaccagcgttgctacaggtttattaatgccccgagaaagcagttcttctcttaaagcgtttt
gtagatcgtaaccaatataaccctggcttaatgcaacacagacagacatcggcagcattggcgagtgcgcttcagtttttgcggcagcttcaaa
ggcctggttaatcatgcccacctgcggcccgttaccatgagtgacgataacttcatgtccctgggcaattaaatcaacaattgcctgagaagtaa
tttttaccgctttcatttgcccggccaggtcatcgcccagcgcattcccgcccagggcgagaacaattttcttactcatttttcctcactcatcattcttg
attgtgtatagatccctggcagtggttagagcacgggagggacaaacggtttgcgtcgcaggaaacgacctcgcccggctttgcctgtaaagg
tgccgtcacagaaaatcgtttcaccacgggataaggttctgacaatcgcgccctgacaggtaaaaccctcccatggcgagtagtcggcgttgt
cgtggagatggcgatgctgaatttgttggctctgacgtgggtcgataatcaccacgtcgccgtcggaaccgggcgctaataatcctttttgcggcc
acaggccaaacaacctggcgggcattgcgctggttaattcaacaaagcgttccggtgttatacgtcccgtcatcacgccactggaaaacagta
actgcatgcggttttccacaccgggtaagccatttgggcagcgactgaaatcgcctttagaaatttgcaggcgttgagccatcgagaaggtgca
gtgatcggttgccaccacgtcaatcgcaccatcgctgatgccacaccacagtttgtcctgctcgcgtacgttacgcagcggtgggctaagaatg
aacttcatgccatcttctgtatcgtaactgcgttcgtccaacaggagatattgtgggcaggtttcaacccagactggctggtgattcgcacgggca
agacgcagataatccagacctaagccgttagacaggtgcacgatatagagcggggcgttaccggcaatttgtgccaggttaatcatgcgggc
gatggcttccgcttcgcattccagagggcgactcaaggcatgatagcgcggcgcggttaaccctgcggcgataaattccgcccgcttgctggc
gatagccgcatcattttccgggtgcacggtggtcagcgcgccggattcatgcaagcggcgtaatgcctgcaaaacctcgtcatcgttgagtttgt
attgataggttaaatagagtttaaaactgctcagtccttcctcgactatcatcgggatttcgtcgaggattgcgtgattaatgtgctggatcacaccgt
gaaagctgtaatcgatgaccgctttatgggcggcataaccacgataaacctccagttgatggcgtaaccgacagccgtttgggccaaatccca
tatggtcaataatggttgttgtaccgccacacgcagctgcgcgggtaccggtaaaaaaatcatcacaactgcgcgcgatgccgacatcaatatt
gaaatgcgtatggacatccacgccgccagggaaaacgtaacagccagtggcatcaatttcttcatacgggagctgcggcgaaatattgttgc
ccaactggcgaacaatcccgctttcaatcagcaaatcctgtttggcttgtccatctgcgttaacgacagtgccgtttttgatcaatacgcgcatagc
aaactccaaaaaccctccggcgaaccggaggggaataagtgaagagtaattactctgttgccagccagcttaacgggattgcagcgtacat
cgcggcacaggtcaccaggtgagatttccaggtttttcgttaggtgcatgcgcttcaggttctttacccgggccaaagccgatcaccggaatgc
cgtgacggcccatgatagatacgccgttagttgagaaggtccacttatcaacaaccggcgctttgccaaacagcccttcgtaggcattcacca
gtgctttaacggtgaagtgatcttcttccactttccaggtcgggaagtagcattcggttgggtaaaccaggccagtccaggacggacggtcgtag
ttgtacatagaaacaaccgcgttagctttctgtactgcaggcagggcgcggatttcgtccagcgcgccttcccaggtttcgccccaggtcagacg
acggtcgattgaaactgcgcagctgtctgctacagcgcaacggcttggggatgtgaagaagatttcagaaacggtgagggtgcctttgccgag
gaattcgtcataacccagacgttgggagagttcttgtaattcgccaagaatcggacccattttgaaaatggcgttatcaccgcgttctggtgcaga
accgtggcagctaacaccctgaacatcaatacgaatttccatacgaccgcgctgaccacggtatacctggcagtcggttggttcggtactgacc
acaaattccgggcgaatgccggattgttcaataatgtactgccagcacagaccgtcgcagtcttcttcctgcaccgtaccggtaaccagcagg
gtatattcatcttccagaccgaggtctttaatgattttaccggcataaaccatagaggccatgccaccttcctggtcggaagtaccgcgaccacc
aatcagttcatcagtttccatgccttcgtacggatcgaagtcccagtttttgatgttgccaatgccgacggtatcgatatgagcgtccattgccacca
gacgcgggccgtggccgatataaccgagaacgttgcccatcgggtcgatttcaactttatcgaagccgactttttccatctcttctttaatacgatgt
actacgcgtttctcgtcgcagctttcactgggaatagcaaccatgtcgcgcaggaagcgagtcatatccgcctggtaatcttttgctttttcaagaat
cagtttgaatggaatattcttagccattatttttctccagatttttttactccccgttttgcagggagcagttccaatagggtgattaaggtgctacagcgt
gtttgccttcccagacaacttcgcggtagtgcttcacgtcggtgtcgccttcagtgctgataaccagcaccacggcatctttgttcagcgccagttttt
ccatcaggctttgacgttgcgggtgataatgaaccgctgcgagaacgcccaaaccgacagcgccggattcaccggagatgatgcgcgggtc
gttgccgtacggattacccagcacgcgcatacctaatgcggcaacgctgtcctggcaggagatgaattgggtggcgcagttacgtaggatttcc
cagcccagcgggttaggttcgccacaggccaggcctgccatgatggtggccatatcaccgccaacgttgacgatgtcgcctttgacgccgga
gcgataaatacagtcagctttgtcaggttcaacaataatgctgtgcagattttgcgggctatagacgtcgaccagataacccagcacaccaccg
gccattgctccgacaccggcttgcagcagaacgtgcgtcggggttacgcccatttcacgcatttgctcgacggcttcatctgccagggttgcgta
gccttgcatgatccaggttgggattttggtgtaaccttcccatgccgtgtcctgtaccacttcccagccgtgctgctgcgcgtgttgcatggtcaggc
gaacggtatcgtcatagttcatatccgtgacgatgcactcggcaccgaggttcagaatggcgtcaacgcgttcctgagcagaacctttcggcat
gtaaatcaccgcattctgtccgagttgctgtgctgcccacgccacaccgcgcccgtggttgccgtcggtggtggtcgcgaaagtcatttttttcgcc
gatggcatttttcaggtgctcaaatgacagcgtttcgatatcaagatgatatttttcacacaataattgagcgatggcgtacgcaccgccaagcatt
ttgaaggcgttcagaccgaatcgttttgattcgtctttgacgagaattttcttcacaccaaaaaggtttgcgagatcgtccagcgcacaaagcggt
gttgggcgataaccagctattttctggtggaactggcgcgccagtttggcctggctttgcgaaaagagcggtgatgtttcgccgttgaaaaatttgt
tatcggcgatatcaatcttcaatgagaaaacggacatatcctatccttttggcctctggaaatagagggccgaccagctgatcggcccgaggg

gttatttcacgcgttcttgcgcttctttcagcagttgctcgagcagtgcacctggtttggcgtatttacgggacaggatcatcgcggcgatgatataa
ggcttccagctggcttctttgtaggtagcgatacggtatttttcgaatacgccttcagtcacttcaccttctttacaggatacgccgctgatatcagctg
gcaggcagtgcatgtacagggcttcgccatcacgggtcagttccatcatctcttcagtacaatgccagtctttgtgttgcgcgttctgtgccagaca
ctgtttttccagtgcttttaagccttcgtgatcgttcgcacgcagcaattcagtacgctcttccatcactttgtaaggtgcccatgacttcggataaacg
atgtctgcgtctttgaaggcttcttccatgctggtgacctgacggaagctaccaccggaggctttagcattgtttttcgccacttcaaccacatccgg
gatcaggtcgtagccttccggatgggccagggtgacatccataccgaagcgagtcatcagaccgatgatgccttgtggtacagagagcggttt
gccatagcttggagagtaggcccaggtcatggcgattttttacctttcaggttttccagtgaaccaaagtgttcacgtaaccacgcgaggtcagc
cattgactgagtcgggtggtcaatatcgcattgcaggttcactaaagccggacgctgtggcagtacaccctgcttgtaaccgtcatcaagtgcag
cgccaacttcacgcatataggcgttgcctgcgcccagatacatatcgtcgcgaataccaatagcgtcggcgcagaaggagatcatattggcg
gtttcacgcacggtttcgccgtgagcgatttgtgatttgccttcgtcgagatcttgttgtgcaaggccgagcaggttaagcgcggaagcataagag
aagcgggtacgggtggagttgtcgcggaatacggaaatacctaatccactattaaagactttggttgagatgttttcagcacgcagtgcttttaat
gctgcggcaacgtccagtacttgtttcagttcatctggcgtctgttcccacgttaacaaaaaatctttctcgtgaaggtgagaggtcagcgaattga
tatccttaatcagctcattaacagttttcatcataaagtcccggtagtggaatgacaggcaaagtggtttatttcactgtgacttgccaaagtgattg
gtgttttcacagctatacctgggactaacaataagtgtgccagaattatccgcgcggctgcaaaagactaatttgacggataatgtgtggggttg
atcacaaattgatgccagcttctggttgtttgtaaaatacgaaaacgtttggcttttttatcgcgttatttatttgctgctaagtatcagaaataagataa
aggggcaggctatctctcttcagtttgataaactcaccgccgtaattctcatattgatatagaaatgtgatatctcttccataatttcattagaaaatga
taaaaatcttatgtttttatcaaagccaatttcgcctgagttaatatcatcatgatctgtgaaccatcaacgtcttcaatgatcgacttttaagtaagtct
ggaaggtaaagctatggagcttgctactacgcagtcagtattgatgcaaattcaaccgacaattcagcgttttgccagaatgcttgccagcgtttt
gcagcttgaggttgagatcgttgatgaaaacttgtgtcgcgttgccggaacgggcgcgtatgggaagtttcttggtcgccagttgagcggcaact
cacgcctgctccgccacgtcctggaaacgaaaactgaaaaagttgtgacacagtctcgcttcgatcccctttgcgaaggttgcgatagtaaag
aaaattgccgcgaaaaagcatttctgggtacgcctgtcattttacaggatcgttgtgttggggtgataagtttgattgccgttacccacgagcaaca
agagcatatcagtgataatttacgcgaattttctgattatgttcgccatatatccaccatttttgtttcgaaacttctggaggatcaggggccaggag
ataacatcagtaaaatatttgcgaccatgatcgataatatggatcagggcgtattagttgtagatgatgaaaatcgggttcagtttgttaatcagac
tgccttaaaaacacttggtgttgtgcaaaataatattattgggaaacctatccgtttcagaccattaacatttgagagtaattttacccatggacatat
gcagcatattgtttcatgggacgataaaagtgaattaatcattggtcaattgcataacattcagggccgacaattatttttaatggcgtttcaccaat
cgcataccagtttttctgtagcaaatgcacctgatgaaccgcatattgaacaattggttggcgagtgccgtgttatgcggcaattaaaacgactca
ttagccgtattgcacccagcccatccagcgttatggtggttggtgaaagcggcacgggtaaagaagtcgtcgcccgtgcaatccataagttga
gcggaagacggaataaaccctttattgctatcaactgtgccgcgattccggagcagcttctggagagcgaactgttcggttatgttaaaggtgca
tttactggcgcttctgccaacggtaaaacagggttgattcaggcggcgaatacgggcacgctgtttctcgatgaaataggtgatatgccattaatg
ttgcaggctaaattactgcgcgctattgaggcacgtgaaattctgccgattggtgccagtagcccaatacaagtcgacattcgcatcatttctgca
actaatcagaatttggcccagttcattgccgaaggtaaattccgcgaagatctcttctaccgacttaatgttatcccgataactctgccaccgctgc
gtgaacgtcaggaagatattgaactattggtgcattactttttacatctgcatacccgtcgtctgggatcggtttatcctggcattgctcccgatgtcgt
cgaaatattgcgtaagcatcgttggcccggaaacctgcgcgagttaagcaatttgatggaatatctggttaacgtggttccttcaggtgaagttat
cgacagcacgctattgccgccaaatctgctgaataatggcacaacggagcaaagtgatgtaacagaggtcagtgaggcgcacctgtcactc
gatgatgcgggcggcacggcgctggaggagatggaaaagcaaatgatccgcgaggcgctttcacgtcataacagcaagaagcaagttgct
gatgaactgggcatcggcattgctacgctctatcgcaagattaagaaatatgagttgttaaacacataacctcagtgaatccggcctgaattcag
gccggatatccttttacttcgttttctcgcaatccagaactgtatttacaatcatctgataccccgtgcagcgacaaagatttcccgccagtccgcga
cgaatttccgtaatggttaatggcttctcgcgtggtttcgccagcattgccgtggtagccataatcaggccaggcgtacaaaacccgcactgcac
tgcgccggatttcgcataagcctgctgaacatgagaaagttttccgcctttcgcttcaccttccagcgtgcggatctcttttccttcagcccaggcgg
caaggtataagcaactgtctattgctgtgccgtcgaccaacaccgtacaggcaccacattcacccacgcagcacccttgtttgacacttagcag
tccttgttcgcggagtaattccgagagcggcgtgcctggtgcggcgtgaagctgaaaaggcatcccgttaatggtgcattcgatggtaattgtttc
gctgtgattcattgcaattttccccccgccgcggcgacggcttcgctaatcactttttttggtcatcgtctggatgagatgcagacgaaactctttactg
gcccgccatgaagaacgcggggcgacatcttgcaggacagattcgctgatagcttccagcgtttgcaggtttaatggcgcattttgtgcagtctg
ttcggcatgttggcagcgaatcggcgttggcgcggcaacaccaaatgccaggcgtaattcgctgaaattgccgttatccagtcggcaatgtgcg
gcgcagccaatcgttgaaatatccattgcgtcgcgcatggcatatttaaaatgcgcgctgcccgcgtgttctttcggctgtggcggaaaatgaaa
ggcgacgaggatttcgtcatgctcaagagacactttgcccggcccggtgtgaaagccattaatcgggacgaaacgaacaccgcgcgggga
gtggatctccagtttcgcgtcataaattagcgttggcgtggcagaatctgcgctggtggcaccgttgcaaatatttccaccgtaggtagcgacgtt
acggatctgcggtccagcaatggacgtggccgcagcacataacgccgggagatgacgttgagttataggatcttctattagctgggtaaatgtc
gttgcagagccgatacgtagcgagccatcttccgccagcgtaattccccgcagctccgccagattatgaatatcaacaatatggcgataacgg
tcattgtgatggtggagctgaatcagtacgtcagtgccaccggcgagcagtttggcctgcgggttgtcagccagcaggttgatggcatcggcaa

gggttgctgcgcgatggtaagaagcaaaatcaaacatgatgttatcctcaaatcaatcctgccagatggaactcttcatataaccgttttggcgtc
agcggcagtgtattgattgcaacaccggtagccatcttcaccgcgttacgaatagcagcggcaacaggaattattggtggctcacccagtgact
tatgtccgtatgcggattgcggctcattgatttcgacgaacgcgctttccagttgtggcagatccggcatggtcggcattttgtaatccagcagattg
gggttacggaccacgccgcttttagcatcgatgatcatctcttcaaatagcgcccagccaatgcccattcccattccgccgtgtacctgaccttctg
ccagcagtggattaagaatatgccctgaatcatgaacgttgaggatgcggttgatggtgactttgcacagcgcaatatcgaccgtcagatcaac
aaaggtacagccaaacgccggtgggttagtggtggttttgatggagctttcagcagagagctgcccgccgcgttcagggtggtagaaagcgtc
catcgccaaatcttttaacgacattaacggctcttccggtcgttcaaccagcacgatatggccttttatcagggtcagattcatcgctgactgatgta
gcatgactgcggcgtgagcgatgattttctcttttaataatagtgccgcactgcgcagcgcaggcgcggcaacatagctctggcgtgaggcaa
atgcgccgggatcgaacggcgtaacgtcggtatcttgagttgaaataacgcgaacgtcgctgaccggaacccccacggtttctgccaccattt
gcgagaagacggtgtcggcaccctgaccgatttccgtcgcgccgctttgcacgttgatggttccatcctgattcatcagaaggcgcgcgcctgct
atttctacgccgacaggccaggtgttagaggtgtagctaaaacaggcgacgccaacgccgcggcgcaaattgccttgctggttctggcattctg
cacggcgtttttcccattcaaagattttccggcctttttcaagacactccggcaaccctgcgctgtaaatacgtttgcccgtgagcggattagcatct
ccttcgcgggcggcgttgcgtaaacgaatttcaacaggatcaatacctaacgctgtcgcggcgtcatcaagcatagactcaacggcaaatac
gacttgtggcgcgccataaccacgcatcgcaccagccgaggggaggttggtatagcaggtctttgaactgtaagcgtaggcacaacgagga
taaaggtaagcgactttattccccccgcagaagcgatggagtgcccgtgagatgcataagcgccggtgttagacagaacatccagactata
acctttcaatgttccgtcgcggttcacgcccatttgcccgtcaatggtaaaagcgtggcgggtacgggttgcgaggaaacactcttcacggctaa
gggaaactttcaccggaatgccgccaagcttgctggtcaggaatgccgccattggctcttccagtacatcctgtttattaccaaaaccgccaccg
acaaatggtttgatgactcgtacgcatgaccagggaatatccagcgcctgaccaaccacgcggcgaacaatgtgcgggatctgggtgctgg
aaacgatggtaattcgcgagtcatcctccatccacgccagcgatgttacgctttccatgtgacaatgttgaataacgggggtctgatagtgcccct
gtacctggtagtcggcggcatcgattgtttgttggacattacccgtcgacatcgtgctttgtttcagtaaattgccaccgttatggattggtgcagcgt
cttctgccagcgccgcttctggcgtggtgataacgggtaattcttgccactcaatgctgaccaattgcgccgctttttctgccgtgagttcatcgcgg
gccacgacgatggcaacggcgtcaccatgatgacgaacatggcgagttagcagtgcgcgatcggcggtatcgcgcttgttttcgtcaagtgtc
caggcatgcccagctgtagcgaatggaatatcaggcacatcttcccaggtaaaaatcgccagtacgcccggcaaacttctggcttgttcatcat
taatacttacggcataaccatgtgcgatagggctacgtacatatttcgcataacacatgcccgccataacataatcgtcagtatatcgtgcccgc
ccggtgaccttagcaatggcatcgacgcgcatgcatgattcacccgtagcggttgcttcccgcgcttccatagaaatcccctcgatttgaacattt
ggttcttatggcaaagatttagtctaagcaggcaagatttacgccagaaacgacgataagcgttaggggaggtaggaagtgtgatgacgttaa
aaaaacacagatatgatacgaaacaggactctgaataggggcagtgatacgtatcaatatgagtggcgataaaatcaatgtgataaattggg
attattacgcatcaggatgggaaatagggtaaaaagtcaggaagttcgatacctctacagcgaatattttgctgatgatacttccctttgccaaat
gaaattaacctctttctctattaccacgtttttccagaagcaagagattgggtatcaagaggctggctgctatgataaggcgtcttgtttttttaagcga
ggaaagattttgagtgcgggacgcctgaataaaaaatctctgggtatcgtgatgttgttatcggttggactgcttttggcgggctgttcgggtagca
aatcatccgatacaggaacgtattccggctccgtttacaccgtgaaacgggggggatacgctatatcgtatttcgcgcaccacgggaaccagcg
taaaagaactggcgcgactgaacggcatttcccccccttacaccattgaagttggtcagaaactaaaactgggtggggcgaaaagtagcagt
attacacgtaaatcaaccgccaaatcaacgaccaaaaccgcatcggttacaccgtcatcagcggtaccgaaatcatcctggccgccagtag
ggcaacgttgttggttatggccaacgacagggaaagttatcatgccgtattcgacagcagatggcggcaataaagggattgatatctcagctcc
acggggtacacctatttacgccgcgggtgcaggaaaggtggtgtatgtgggcaaccagctgcgtggctacggtaatctcatcatgattaaaca
cagtgaagattacattacggcttacgcccataatgacacgatgctggtaaataatgggcaaagcgtgaaggctgggcaaaaaatcgccacta
tggggagcacggatgcggcatctgttcgcctgcatttccagattcgttaccgtgcaacggcaattgatccgctacgttacttgccgcctcagggc
agcaagccaaaatgctgatggcgaattaatcagcagtcagcagcgtggcacttgctaaggagagcgtaaggtttataatgccttacgcatctc
gaagcgggcgtagttcaatggtagaacgagagcttcccaagctctatacgagggttcgattcccttcgcccgctccaatttatcttcgccgtaata
cttccccattttgttgtaaataaaaagcgtttacgcaatgcaatccttttgtaacaacccaggtaaaacaccttattttcaagttgttaaataagtggtt
atctacgcttttaacaaacgattgatggaatattttatgaaagggaaatctgcattaactcttctgctcgctggtatattttcgtgcggcacatgccag
gcgacaggggcagaagtgacgagtgaatctgtatttaacattcttaattccacgggggcagcaacagataaaagttatttatcgttaaatccag
ataagtatcctaattatcgtcttctgattcactcagccaaattacaaaatgaaataaagagccactataccaaagacgaaattcaaggattgcta
acattaacagaaaacacacgaaagttaacgttgacggagaaaccctggggaacgtttattcttgcttctactttcgaagatgacaaaacagcg
gcagaaacccactatgatgctgtctggttgagggatagtttatggggctatatggcattagtatcagatcagggaaatagtgtagccgcaaaaa
aggttctgctcaccctgtgggattatatgtctacgcccgaccagattaagcggatgcaggacgtcattagcaacccaaaacgactggatggaa
ttcccgtgcaaatgaatgctgtgcatatccgatttgacagcaactcccccgtgatggcagatgtgcaggaagaaggcaagccgcagctgtgg
aaccataaacaaaatgatgctttaggtctttaccttgatctccttatacaagcaatcaacactggtactattaacgctgaagattggcaaaaaggt
gaccgattaaagtcggttgcgctgctgattgcttatctggataaagccaatttctatgttatggaagattcctgagcatgggaagaagatgcccga
ttaaacacatcttccgttgcactggtgacctctggactggaacgtttgtcaaacctactctcgaagaaagactctgtttttgtctctgatttactacgcg

aagctaaagtcaatgaactggatgaaactctttcaacaacgaggttgaaccacctcatagataaaggttatgaaagaataacgttacagcttg
atttagggggagaatccccaggttatctggaaaaagataaacattacagagaagccgatgcggctttacttaatgttatttatcctacaaatctttc
aaagataaacaccagaagaaaagagcaagtttagactggccccctgaatctccagacaaccaatatcacttaaataagtgatagtcttaata
ctagtttttagactagtcattggagaacagatgattgatgtcttagggccggagaaacgcagacggcgtaccacacaggaaaagatcgcaatt
gttcagcagagctttgaaccggggatgacggtctccctcgttgcccggcaacatggtgtagcagccagccagttatttctctggcgtaagcaata
ccaggaaggaagtcttactgctgtcgccgccggagaacaggttgttcctgcctctgaacttgctgccgccatgaagcagattaaagaactcca
gcgcctgctcggcaagaaaacgatggaaaatgaactcctcaaagaagccgttgaatatggacgggcaaaaaagtggatagcgcacgcgc
ccttattgcccggggatggggagtaagcttagtcagccgttgtctccgggtgtcgcgtgcgcagttgcacgtcattctcagacgaaccgatgact
ggatggatggccgccgcagtcgtcacactgatgatacggatgtgcttctccgtatacaccatgttatcggagagctgccaacgtatggttatcgtc
gggtatgggcgctgcttcgcagacaggcagaacttgatggtatgcctgcgatcaatgccaaacgtgtttaccggatcatgcgccagaatgcgc
tgttgcttgagcgaaaacctgctgtaccgccatcgaaacgggcacatacaggcagagtggccgtgaaagaaagcaatcagcgatggtgctc
tgacgggttcgagttctgctgtgataacggagagagactgcgtgtcacgttcgcgctggactgctgtgatcgtgaggcactgcactgggcggtc
actaccggcggcttcaacagtgaaacagtacaggacgtcatgctgggagcggtggaacgccgcttcggcaacgatcttccgtcgtctccagt
ggagtggctgacggataatggttcatgctaccgggctaatgaaacacgccagttcgcccggatgttgggacttgaaccgaagaacacggcg
gtgcggagtccggagagtaacggaatagcagagagcttcgtgaaaacgataaagcgtgactacatcagtatcatgcccaaaccagacggg
ttaacggcagcaaagaaccttgcagaggcgttcgagcattataacgaatggcatccgcatagtgcgctgggttatcgctcgccacgggaatat
ctgcggcagcgggcttgtaatgggttaagtgataacagatgtctggaaatataggggcaaatccaataaacaattggaatttacttccattgatct
gtttactcagtggttgtcacttttatcgagaaaggtttgcagaacgcgggtttttttataaggttcctgatgtactaagggattatttatcagctataccg
ctggaaataaatgaaaaggcacgatacaaacccggcatcgctaactatcacaatatcatcacatgtggtttttcaacgttgttaccctatataag
gcaacaaccattggcaatgcagcaacgatttaatttattatttcctgattttgttgaccacatacaatcgccgttaccacttgcatcaacacttttaga
gagaattacattttatgcgaaaaaaaaatagagatgagcttgataaaatctcctgcaaatggtgttgtgattaaaagaaaaatttctgatggattga
aagaaatagtttctcttaaagaaaaaatccttcttgaaaccaccgcaaaaattcagagtattgaggaaaagagagaagaaaaatttattcaag
gttattatgatggctatacaaaaggcattattgatgagatggataattttatacccttgataagcttgctatgctcagaacttgaaaaaaaagaata
aacatgataaacgatctgaagagtatattgttaaaaccttcagaagaagttgatgtttttatcaaaatttttgaaagttgggtaacaaaattaccatc
aatatcaggtcctgtaaatctccatatacctaccagtttcaaagataagtctcttgaagtagagtcatattttgtcgacaaatcaatatggaatgtcc
atattacatttcatgatgataagcgttttgtgtttttttaccgatcaatttattgctgaatttagtccgcaggaatttgtggataattgtgagcaatatttaatt
aataatcattgtttctcccctgacaaggttaacgaaatctgcgaacaagccagacattatttagttgaaaaaatgtttgaaacacattctttggatat
gaataattctgttttggcaagccctgaggacttatagctccctggctcaagtttaatgatttgaatataatctaattgtaatattttaatcatctcatgattt
tttaatgcctgtggtatttttttacgcaaaaattttatttttaattatatttcattattatttcatcaatgtattctttggattttgcttataataataatggaagga
tgattataaatgaatgcgttaaagcatttacactcaggatgaatatatgggaaaaattaaaattgtagtttcagatcagcagccgtttatgattgatg
ggataattggatttctcggacattatcctgatttatatgaggttgttgggggctataaagatctgaagaaagctatagcggagtgtaacaaatctac
agcctaaatatttattttaggagaatttgcaggtggaatgatgggggccgaactcgtaaaatgggttaaatcgcataagatagatgctcatattatt
acatttgtagcaaagatgccgtatattgattcaataaaattgcttgaagcaggtgcgaaaggatgcgtatggaaaaccagtcacccggcgaaa
ctaaatcgtgctattgattcgattagtaatggttacacttattttgatagtgtacatatggattgtgaaaaaatatcttccaggtattcctctgataatcaa
cttacaaatcgtgagtcagaaatattacaactcatagctgatggtaaaacaaacaaagaaatcgctaatttcttgcagttaagcaggaaaaca
gtcgaaactcatagactcaatatcatgaagaaattagatgttcacagtggtatcgagttgatcaaaacagctttacgtatgggtgtttgcactatat
aaaaaaaaagaggctttaggctcattttgctgaagccttacgattgtctagttcattaagcctggtatatacaatatatattttggggcatattgctgtct
ctttttttggttatccattgcaaaacccgcattataagtccccaggcattgccacgttactccacaacgtgaaaaatggttatataaaatctcggttcc
tattttaatgttcagacaagggttatctaacaaatctcgttcatcttttattattccacgttttttttaacaggggaatatgcatctggtttatctgcattaacc
aataatctaaactctttatattgttattcttatctctgttcttgcctatgctatcctttttttagattgctttcaacaagagcgatagctttaattaaatttggttcg
attctgaacattgttcctgcttcattagagcaagagttgctggcgaaagacatttttgtaataaatatacaaaacgtgaagatggttaagaatttaa
cttttggtgagcgcatatttttttcatgtgtgaggattgaatgaaaggaacgagatactattataaaagtatctcgttatgatttatttggtcaatcgtcta
ccactcaattttatagtaaggtgagataggattatcttctgaatacatatcattttgaaaaggcgccatccatgttgaaactataaatgcttgtatttga
tctgtatcagtagcactggtgttgatgtgtttatctaatgatacgacttcatcacccgttgcattcgctatatttgaactttgagataaattattgataccg
attggattagtcataaagctccctttttttaattgtccaggccaatggtctttacatcatacttttaactctagagcattttaaatatcttgcaataaggtga
atatcttagatatttttatctctgttgtttaaataacaagttgcatacatctaaataataaaattgcgtcaataaaaatttatcgcatacaaattctcgtca
gctgctttaatttcaaaatgttgtcaattataactcgatggctttcactctcagtttctttatagtagagtttaatgttgtttttgagcgggttaggcacatac
atatctacacattcttttatcacaactgcttttcgtcttctgggttcgagttgtagtgatttgtctatacaaatccttgcctcttcaatttttttcattatgaaagt
ggactagtgccctatagtagtagagagaggctatatcagttgagtgtatcttagcctgttcaaataagatatgagatactttttgcttgtccagacag
accagttatcagtcccataatagctaaaatttttccatcgacagtggttatgtctgatacataatctaaaagctctaatgctttttgagcagcaagttc

aagttcactttttccatgaagtgccaaagacatatggcattccgctagaaggcaataacattctgtttttagcgtctgtatatcacttttattttgtaatct
atcaaatatagtcatggccctgtaaatgctttcaggggtgaagtcgtaaagttctttctttccggctaacatttcatctgataaataatgattcttatatc
cttggtcctgcttagctaatcttacagatcgttctggtttatgtaccgtttgaaggatattatctataataaattgtgatgtattatgtagttcatcaacagg
gagatgattactggcgatgaggaaaaggtttttagcgtcgatcaactcaatgtataaagtgttcactgcattattctggtttattcttcctgtaacgaa
ataatctggcttgaatctgctcaagaatgaattttgagatatgtgatcattacaaaaatttgtcgcagccatcggataggtatagagaccatcgattt
ttttatttgaaatgatttgcactaattcctgattaagtattaatggatccagtgtattcaatgaagtagtgaaagggaatatagctatggaatagtctga
agtattatcttcattaattttagttttgaaaacctgcccgctgaaacgataacctttccggtagattgtttctatgcaacgatcatagccaatttttttcaaa
aatgcagcgcaaagaatagatacatcttgtcagggactcgtcactaacaataatatttttccatactgattcgatgatcatatctttcagtacaacat
gaccagcggattcaagtaatactattaatacacctaactctttctgtggaatataaacttgctcatttccttgagtaagaattccatcttccgtgagcg
ttaatccctcaagaaaatgatatgagaatttattttctaagtccattagttaacctctatgaaaaaattggtttcttgcatcctgcttaagcaatagaaa
ttatggctattcatatttatgtgtcattaacgctacaggaaattacatttgacatatctatggttgtgttcactaatttatcttaggaattcagtgatgtaatc
tgaaatagccttattcaattttgataaagtagattgttctctaacgcattgaaaaataatgatttatagtatgctcttaactgttttaagatgctgatttaa
gaaaacataagaggtgctggttatagtggttaactgcactataaccagcaaatttaaccaaatgattgtaatgcttagccattatcttctgaattatc
gtttaagagttcaatatatgcctgcgctttactcttaacactatcatcctcacaattgttaataatgtgatagaaacattgcattgcgttatcgatattttt
agccccaatctgacattgacccatacgataaataactgaatagtcatcatacgggaagtaattgtaacttagtttgtaaaggtcatatgcttgttga
tatttctttttgggctgacatactgatgcaaaatcttttagatactcatgattttcgaacgcataaatgcacaagaatttataataaatttctgcgtccgtt
atattaccctttttcatggtagtcgaatgcatatgcatatattctatcaaggatatcaggagttaaaccatgtagtaatgccagattttctcctttcgata
atgcgtgttttagttgatttgcccattcatcactattattgaatatttcaattgtttctgtgctcataatattcctctgtgatgtgtctcgtgatgccaaattatt
attgattgactcattgatatttttatatttatctttttttaaattatgagttttaagcttgcattgcttatggtatatttttcattattggtaaaagtactgttcactac
aaactaataacatcattagttgttttactaatattcaccttattagttatcttaattacgacattacataaatgccttaatgtgtatgacctggattcatagt
atcaattgccatttttttcaatgaaaattaaaaaggggatttttgagaagggtagaatgtcgtttgtttttttctttattatgtctggtgacgcggggggata
gaacgtctgtgcatatagtagtgaatttattcaggtctacattattctctattttggtcagttttatgagttcatttacagccatactcattaaatcatctgtt
atcgaaaatttatatatgctcatagcagataatattgcttgctgaattatctcttcagaaatatccgctatctttattgacaaatcatgtgttagatattttc
ctccaagagattcttgttcaatataatttaaaccagaccccattgtgattttaactggattaacggatttctcttcatttttcttatttttgttctcctgatcag
gaactaatggttgcagtggtaggctattattaatatttcgtggcttcattttacatttctctcatttgattagagcaaaatccgaattcgcagcttctgata
atgtcaaatatcaccttatggagacgttacgtagaatgactatatttgttatcggcacattaggggtattaattttagtattgatggaaggtgattattg
aacagtaacatcgagtcacggtaaaaatgtgaggtatacaatggacattgaattttcgcagattcatgaaatggtctatatgcatgatattgtaaa
ttcggactcgaaaaagaaaccaaggattccgttaaaaaaattccttaatgctgagaatgttctcacgcaaacaacatcatggacattaaattca
agatatgtgaatgttaattctgtcaataaagtcaacgtaaagagtaaagttaaaaacagttatatctcacgatctgtcaatgatgagtttagtttaac
agatgatgaaatcaattcttttaaagaaacgttagtcctttcctctattgattcactgagtaaactggttcttaataatcctctttctgtattgtttacatcg
actgtacgaaggaataataatcgagctaagatgaatgtcgagttcgactcatggatttgcaccagatgttgttgatacatgatattttacattgcaa
ggagatgtctaaaatgaaagatgttgatcaaatctttgatgctttagactgccatatactgcgagaatatttaattttattgttttatgattaacgaggtt
ccatctttttgttatctattttagtttaccttgcacaatgcattattgctctgttaccaatgctcaaaataaaagcatgcttaccttgctttagtatattatagc
ttacactttttttctggaaagacataaaaatcaggaataagttgtgcaagtctatcatcatcaacttgatccataatctcaaacttattaactttctttgat
gttaaatgggctgtgtcatagttttcaatccatgtgtattcaatatttcgccgtattgatttactttctctgttaccttgttgccaggttccactcaacgcaa
cagttcctgttttattttttttgttatacataaagtcgatggtaagatttgctcgtccctggtcattaaaaattacccaatttgcagtgcatgtttcgttatata
tcatttttttctgcagtgtaagaatagacagtgaagagaataaacccactgataaaaactaaaataaataataagttttttttataatcaatcacgatt
aacctcacggtaagacagggttaaacagtttgccacttcatgttggttatagtttttattgcatgccataacaatcatccccggtgaggattttgcaa
gagggaagtataaccacggatgttttttacagttaatccccgaatccagtatccgtgttttatatttatcgaactctttcaacccatcaattgtatcttct
gttacattaaaatggcagccattaatttccgcaacagtcttgtaatccttgaaaaatggcttcggttgattattattccatagccaatatgcagaatat
gcgcctatgacaaatgcgctcaacataattagatgactttttatatgtttgaggattttgttattgaaattctttgcgccaccaatgctctctttgtatccc
gcattaacttttatggcaggtggggcattgtgtgtatgtgcgtctgttgacgagtttattacatgattttgtatgttaatgtcattatggatcttgaatcctct
tctcggcacggttgcaattagcgagtgggtagtatcaccaacagcgcgaaatccgcgtctgataatggatatgttctgatataacgtgttagtag
gcacacgcataccttcttcttcccagactctggtgaaaaaatcttgttgtgcaaccacatcaggaaaagcttcaagtagaagtgataaacatcg
actggctggcgttgtcagaacgacgttaagatcggcgttatgtaccgatattaattttcggtgctcaggccagaactcgatattatcgttaataatcc
agtacatacgtcaataaacactaaatcaatcgaaatggagatcacatagtttgctaagtatattggtatttacggcataaatatatattaattttatat
ttatcatgatgattgaaatgaggctttaatgttgcaacgtaaaactttacgtaaattaacatggttaacatttatgccactattgtttgtaaattcatattt
cgtaatgcttctgaatttttcgtgtgatggttttaaatactatggtgtttactcttgaggggacggcctatttataaaatacggacatttcaataaatgcc
cgtataaacagagtatgattctggctggtcgttgagtatcaatgttggaccgaatgtgaacgaataaataattcgtttattttaccacccattctctttt
cgattcgttgcttgtacgcgtagatgctacggatatccatatccagaacttcagcaatctgatatggattataaccagaaaccatataccccattat

ggtgaattccatctgagttaatgtgttacctccggtaaatgataaataacgaccaataaatagttgtctgatcttttcattggcaacatcaagaccat
catcattatatatgacagcagatattgctgagttgtgttttcgccagtaattagctaacgattgcatattgcggtcgctgactaaaacaatccccatgt
cagtacttgcaagatagtcaatccagtctttttcaaggaatagacgtaaaaaacttactgaaaaatcgacaaaaatatatccgcttaatctcgcat
tgtggtttttgttaagataccttctaccacaccacgattaaaatatgaactttcttcgggccacatacaaaaacacgagtttgcgcaatactgcaa
acgcggtgactcttctggcattatttggcaatgcattgagcaactattgcatgtaagacttttataacgactacaaagccccatgttaagaacctcc
ctatccgtatataattgcataatttcattggttattgatgatgtcaatacaccatgattttgtaattttgccgggaaaaattgcgattaatctgtatttgctat
aattctgagtaaggcatttaaaaatgcatttctcgttatcaaatatgattatgtttcactgatttaatcttgcatggttgagagcggtccagataactatc
tcatggtataagaatatctatatcgcaaattgtttttgaatgaccagaagatatcttgcgaatctaagttatgcatcttaaatagtcttatttaagatgtt
cagatctctttgtgtgtttaactaactgataatactaggatatgacgagagaagtgatagggtggaataataacaaatgaatattatgaaaaatg
gaatggttcaaaattgtagcaattttgaaccatccacaactgatattagctaaagaacattaccgatacgcacaggatacccacgatcagcgta
atcagatttccgatggaacgccagggtttaagcgccgggatgagatacgttgacagcgtaggcatgatgaaaagtatcatggcaatgagcgg
gccgctgatcgcgtaaatcatcgaaatcgcgttcgggttaatgcaacaaacaatgaaggtaatcagcgataccaacataattgatagtgcgcg
gttaaatgcacgacttttctttacaccaacctgctgtagtgttgttttgacgacctctgtggcaccttcaataacgccaaagtacgtacccaggaatg
atttcgacatcgcaactactgcgacaataatcccggaaattgacaaccatgctggggcgttcggcagcatagaaagcgccgataaaatggtg
accccttcttctttagcagcttcaatatacgaaggtggaatagaaagcaggcagctaaagacaaagaacagtacacttatgcagatgatgag
ataagcgactttcataatttttttgcatttatccatagcgtgttcgccatatttttcacgtctgtctatggcaaacgtagaaataatgggcgtatggctaa
aggcgaaaaccatcacgggaatcgatatccatatctggtgaagggtattctgattgaactctacctgggttgttaatagatcaggttgccaactac
cgacaaggtaaatggaaagaaataagaaataggcaatcaatgggaataccaaaaatcccattacccgaatagtggcatgacgtcccatca
gaaaaatgagattcagaattaatacaacacccagactcaccaacatacggatgcgaagatcaataaccatatgctttgccagctgttccgtaa
gtgagttggtaattgccactgcatatatcaacacgacgacaaaaaaggcgatgaagtacagcgtggtaatcagattaccaatcttcttgccata
gtagtgtgttaccgcgcccgttatcccttcacctgctgatgtttttgatgagaggatgaactggcacaaggctttatgtggccaatatgttaaaggcc
aggcgaccagagcagtaataaacaggaccacagcccctgccgaacctaactgaataggaaggaagagcgtgcctgcaccaacggctgt
gccgtagagcgcgaaactccacagagtttcttctttagaccaaatattagacattatctgaacgtatcatctacaaattaaacaaaatggagggc
aatttaccataaaccaggttaagtatgggagattaacgccctatctacccctctattggtgggttagtggttgcaaaccttacgtgccagttatgtct
ctgactgctgctcaggcatatttcctttgtgataaaaatcactttttttaaacaattaagataactgtaattaacaatcatttactattgcactgttaattg
gtgcgaggttgtgatgaaagacgttgtgattgtcggggcgttacggacacctatcggctgctttcgtggtgcgttagcgggtcattccgccgtgga
acttggtagtctggtcgtgaaagcgttaatagaacgtaccggcgttcctgcatatgcggtggatgaagtaattcttggtcaggtgttgactgcagg
ggcagggcagaatccggcaaggcaatcggctattaaaggtggtctgcctaatagcgtttctgcaatcactattaatgacgtttgcggttccgggc
ttaaagcactgcatctggctactcaggcgatacagtgtggcgaggctgatattgtcatcgccggtggccaggaaaacatgagccgcgcacca
catgttctgactgatagccgcaccggtgcacagcttggcaatagccagttggttgacagtcttgtgcatgatgggttgtgggatgccttcaatgatt
atcatattggtgtcaccgccgaaaatctggctcgcgaatatggcatcagccgtcagttgcaggatgcttacgcacttagctcgcaacaaaaagc
gcgagcggcgattgacgccggacgatttaaagatgagatcgtcccggtaatgacccaaagtaacgggcagacgttggttgttgataccgatg
aacagccacgcactgacgccagcgcagaaggcttagcccgtttaaatccttcatttgatagtctcggttctgtgacagcgggtaatgcatcatcc
ataaacgatggcgcagctgcggtaatgatgatgagcgaagccaaagcacgagcgttgaatttacccgtgctggcccgcattcgcgcatttgcc
agcgttggtgtagatccggcattgatgggaattgcgccggtgtatgcgacccgccgttgcctggagcgtgtaggctggcagttggctgaagtcg
atcttatcgaggctaatgaagcgtttgctgcacaggcgctttcggttggcaagatgcttgagtgggatgagcgtcgggtcaatgtcaatggtggc
gcgatcgcactcggtcacccgataggcgcttccggttgccgaatcctggtttctctggttcatgaaatggtgaaacgtaatgcccgcaaaggact
ggcaacgctttgtatcggcgggggccagggtgtggcattgaccattgaacgtgacgaatagctctgcatttcctccttaccgataaatatctgccg
tcattaatctgttgaataatgatggctggcaagtcgacatacattttctgggctccctcatttttccattctcatgacatattcgtgatcgacactgcactt
tttttaatccttaacttaaaaatgaaacattgttttatttttaattgaaaaccctagcccatgggcatatcatccctttatagagaaaacgaaacagtgt
ttcactatcgttttctattttcacgcttcactgattatcggaggttgatgtggacgtaagacagagcatccacagtgcgcacgcaaaaacgctggat
acccaagggctgcgcaatgaatttttggttgaaaaggtatttgtcgccgatgagtacaccatggtttacagccacattgaccgaattattgttggcg
gcattatgccgataactaaaacggtttccgttggcggggaagttggtaaacaactcggcgtaagctatttccttgaacgtcgcgagttaggtgtta
tcaatattggcggtgccggtacgattactgtcgatggccaatgctatgaaatcggtcaccgcgacgccctgtatgttggtaaaggtgcaaaaga
agttgtctttgccagtattgataccggcactccggcgaagttttattacaattgcgcacccgcgcatacgacgtatcccaccaaaaaagtcacac
cggacgaagtatctccagtcacgttaggcgataacctcaccagtaaccgtcgcacgattaacaaatattttgtcccggatgtactggaaacctg
ccaattgagtatggggctgacggagctggctccgggtaacttgtggaacaccatgccgtgtcacacccacgagcgccggatggaagtttatttc
tatttcaatatggatgatgacgcctgcgttttccacatgatggggcagccgcaagaaacgcgtcatattgtgatgcataacgagcaggcggtgat
ctccccgagctggtcgatccattccggtgtcggaaccaaagcttatacctttatctggggcatggtcggtgaaaaccaggtctttgatgatatgga
ccatgtggccgttaaagatttgcgctagttgtgggcataaacgaataaggtattgttatgattttaagtgcattttctctcgaaggtaaagttgcggtc

gtcactggttgtgatactggactgggtcaggggatggcgttggggctggcgcaagcgggctgtgacattgttggcattaacatcgttgaaccga
ctgaaaccatcgagcaggtcacagcgctggggcgtcgtttttttaagcctgaccgccgatctgcgaaagattgatggtattccagcactgctggat
cgcgcggtagcggagtttggtcatattgatatcctggtgaataacgccggattgattcgccgcgaagatgctctcgagttcagcgaaaaggact
gggacgatgtcatgaacctgaatatcaagagcgtattcttcatgtctcaggcagcggcgaaacactttatcgcgcaaggcaatggcggcaag
attatcaatatcgcgtcaatgctctccttccagggcgggatccgtgtgccttcttataccgcatcaaaaagcggcgtgatgggtgtgacgcgattg
atggcgaacgaatgggctaaacacaacattaatgttaatgcgatagccccgggttacatggcgaccaacaatactcaacaactacgggcag
atgaacaacgtagcgcggaaattctcgaccgcattccagctggtcgttggggactgccgagtgacctgatggggccgatagtgttccttgcctc
cagcgcttcagattatgtgaatggttataccattgccgtggatggcggttggctggcgcgttaattcattcttcttacttttatgaccctgccgcatggc
agggtttttttatacctgtagatcatcataatccatatcatggttatgaaataatccatattaattatcaattaatgaactttatgaattttatctgctgtaaa
attaggtggttaataataatctcaataattcaacttaatttgaaaattggaatatccatcacataacgacatgtcgcagcaatttaatccatatttatg
ctgtttccgacctgacacctgcgtgagttgttcacgtattttttcactatgtcttactctctgctggcaggaaaaaatggttactatcaatacggaatct
gctttaacgccacgttctttgcgggatacgcggcgtatgaatatgtttgtttcggtagctgctgcggtcgcaggattgttatttggtcttgatatcggcgt
aatcgccggagcgttgccgttcattaccgatcactttgtgctgaccagtcgtttgcaggaatgggtggttagtagcatgatgctcggtgcagcaatt
ggtgcgctgtttaatggttggctgtcgttccgcctggggcgtaaatacagcctgatggcgggggccatcctgtttgtactcggttctatagggtccg
cttttgcgaccagcgtagagatgttaatcgccgctcgtgtggtgctgggcattgctgtcgggatcgcgtcttacaccgctcctctgtatctttctgaaa
tggcaagtgaaaacgttcgcggtaagatgatcagtatgtaccagttgatggtcacactcggcatcgtgctggcgtttttatccgatacagcgttca
gttatagcggtaactggcgcgcaatgttgggggttcttgctttaccagcagttctgctgattattctggtagtcttcctgccaaatagcccgcgctggc
tggcggaaaaggggcgtcatattgaggcggaagaagtattgcgtatgctgcgcgatacgtcggaaaaagcgcgagaagaactcaacgaa
attcgtgaaagcctgaagttaaaacagggcggttgggcactgtttaagatcaaccgtaacgtccgtcgtgctgtgtttctcggtatgttgttgcagg
cgatgcagcagtttaccggtatgaacatcatcatgtactacgcgccgcgtatcttcaaaatggcgggctttacgaccacagaacaacagatgat
tgcgactctggtcgtagggctgacctttatgttcgccacctttattgcggtgtttacggtagataaagcagggcgtaaaccggctctgaaaattggtt
tcagcgtgatggcgttaggcactctggtgctgggctattgcctgatgcagtttgataacggtacggcttccagtggcttgtcctggctctctgttggc
atgacgatgatgtgtattgccggttatgcgatgagcgccgcgccagtggtgtggatcctgtgctctgaaattcagccgctgaaatgccgcgatttc
ggtattacctgttcgaccaccacgaactgggtgtcgaatatgattatcggcgcgaccttcctgacactgcttgatagcattggcgctgccggtacg
ttctggctctacactgcgctgaacattgcgtttgtgggcattactttctggctcattccggaaaccaaaaatgtcacgctggaacatatcgaacgca
aactgatggcaggcgagaagttgagaaatatcggcgtctgatttcacgggccggatgtgctgtacatccggcccttttttcgttaatagagattgg
gcacttggccgttgaggcgtttgtctcgttccttattcagccttgttgcggtaacacacatcaggagagaggaatgaaaacaattggtttgctggga
ggaatgagctgggaatccaccattccttactatcgtttgataaatgaaggcattaaacagcggcttggtgggcttcactctgcgcaagtgctgcta
catagcgtcgattttcatgaaatagaagagtgccagcgtcgcggggaatgggataaaaccggggacattctggctgaggcggcgcttggctt
acagcgggcgggcgcagaaggtattgtgctatgtaccaatacgatgcataaagtggcggatgccattgagtcacgttgcactctgcctttcttac
acattgcggatgccaccggacgtgcaattaccggggccggaatgactcgtgtggcgctgctgggtacgcgttacaccatggaacaggattttt
atcgcgggcggctgacggaacaattttccatcaattgtcttattcctgaagcggatgaacgggcgaaaattaatcagattatttttgaagaactgt
gtctggggcaatttaccgaagcgtcacgcgcttattgtgcgcaagtgattgctcgccttgcagaacagggcgcacagggcgtcatttttggctgc
acagaaattggtttactggtgccagaagagcgcagtgttttgcctgtgtttgataccgcggcgatccatgccgaggatgctgtcgcttttatgctgtc
gtagctgacgacaaaatagcgtcaagagaagtgaccagtttcggtaaccccgcttgtaaatgcccactaaacgcctgaaccagcgctgatga
cgggcggtgcaggggcggatcaggctgacggtgaacggaaccgcaatactgaaccgccgcaccactaaaccgcttgccgcataatcta
gtgcggtgagcgggttaaccaccgaaatacctaccccgcccgtaccattgcgcagactgacgcggcgctgtgggtttctacgatcatgcgtc
gtttaacctgatgttcagtaaatagctgatccagcaactggcgatagctgtcagtacgggaaaggctgatgtagttctcaccctgaaaatcatcc
ggcgttaatacctttttaccgccagcggatgacccggaggtaacacacacacttcatctaaagagagtaattcggtacgttctgttcccgcagg
cgtatggagcgtttcagtgagtcctaaatcatgacgctgggccgagagccactcttcaagtagcggtgattcctggggcacgatatttaagctga
catcgggataacgtgccagaaagggttgcaggagctgcggtaaaaaagattgcgaaaagaccggcaggcaggcaatagacagttctccct
ggcgaaactcgcgcagactttctgcggcgctgacaatgcgatccagtccgtaccaggatcgttgcacttcttcaaacagacgcagtccttgcac
ggtaggatgtaatcgcccacgtacgcgctcaaacaatttcagcccgatcaccttctcaaagcgcgcaagttcgcggctgacggttggctgtga
ggtgtgtagcaggtgtgccgcctcagtcaggcttccggcggtcattaccgcatgaaaaatttcaatatgacgtaagttaacggcggccattagc
gctctctcgcaatccggtaatccatatcatttttgcatagactcgacataaatcgatatttttttattctttttatgatgtggcgtaatcataaaaaagcact
tatctggagtttgttatgccacattcactgttcagcaccgataccgatctcaccgccgaaaatctgctgcgtttgcccgctgaatttggctgcccggt
gtgggtctacgatgcgcaaattattcgtcggcagattgcagcgctgaaacagtttgatgtggtgcgctttgcacagaaagcctgttccaatattcat
attttgcgcttaatgcgtgagcagggcgtgaaagtggattccgtctcgttaggcgaaatagagcgtgcgttggcggcgggttacaatccgcaaa
cgcaccccgatgatattgtttttacggcagatgttatcgatcaggcgacgcttgaacgcgtcagtgaattgcaaattccggtgaatgcgggttctgt
tgatatgctcgaccaactgggccaggtttcgccagggcatcgggtatggctgcgcgttaatccggggtttggtcacggacatagccaaaaaac

caataccggtggcgaaaacagcaagcacggtatctggtacaccgatctgcccgccgcactggacgtgatacaacgtcatcatctgcagctg
gtcggcattcacatgcacattggttctggcgttgattatgcccatctggaacaggtgtgtggtgctatggtgcgtcaggtcatcgaattcggtcagg
atttacaggctatttctgcgggcggtgggctttctgttccttatcaacagggtgaagaggcggttgataccgaacattattatggtctgtggaatgcc
gcgcgtgagcaaatcgcccgccatttgggccaccctgtgaaactggaaattgaaccgggtcgcttcctggtagcgcagtctggcgtattaatta
ctcaggtgcggagcgtcaaacaaatggggagccgccactttgtgctggttgatgccgggttcaacgatctgatgcgcccggcaatgtacggta
gttaccaccatatcagtgccctggcagctgatggtcgttctctggaacacgcgccaacggtggaaaccgtcgtcgccggaccgttatgtgaatc
gggcgatgtctttacccagcaggaagggggaaatgttgaaacccgcgccttgccggaagtgaaggcaggtgattatctggtactgcatgatac
aggggcatatggcgcatcaatgtcatccaactacaatagccgtccgctgttaccagaagttctgtttgataatggtcaggcgcggttgattcgcc
gtcgccagaccatcgaagaattactggcgctggaattgctttaactgcggttagtcgctggttgcatgatgacttgcctccagcgacggagttga
cactgaatgacgacgtaccagcgtcggactaaagacattagtgatttccgggagagggcgattatccgccagcgccaaagccagttcggca
gcctgggtcgccatcgtcacgattgggtaacgcacggtggtcaggcgcggacgcacatagcgtgacaccagcacatcatcaaagccaatta
acgaaatctcacccggtacatcaataccattatcattgagaacgcccatcgcacccgccgccattgaatcgttataacaggctaccgcagtga
aatttcttcctcgtcccaaaagctcggtcattgcctgttcgccgccgctttcgtctggttcgccaaatgtcaccagccggtcattggccgcaatacca
ctttcagcaagggcatcgtaatacccttgcagacgatcttcggcgtcagaaatagagtggttagagcacagataaccaatgcgggtatgacctt
gctgaattaaatgacgcgttgccagccaggcaccgtaacgatcgtccagagcaatacaacggttttcaaagccaggcaggatacggttgatc
agcaccataccgggcatttgtttcattaatgaggctaaatcagcatccgggatcattttggcatggacgaccaacgcagcacagcgatggcgg
atcagttgctcaatggcctgacgctctttttgttcgttgtggtaaccgttgccaatcaataaaaaattaccggtgtgataagccacctgttcgaccgct
ttcaccattgcaccgaaaaacggatcggaaacatcaccaacgaccagaccgaccgtttcagtggtctgctgcgccagcgcacgggcgttgg
cgttcgggtgatagctaagagactccattgcactatgcacagccagccgggaagcttcgctggctttgggtgaattattaatgacgcgggaaac
ggtggcgactgaaacgcctgccagtcgggctacatcctttatggtcgccatgaaaataccttagtgggtaaacgcttacattctttcagtgttacg
gaaaacgcccgttggttcaaggggtggcgtgttttcatcgtgggaatgtgagccaaacgcaaataagtaagacaaaaaaagagattgcaaa
cctttggttacactttgcgaaacgctgttgcgattgaccgctggtggcgtttggcttcaggttgctaaagtggtgatcccagaggtattgataggtga
agtcaacttcgggttgagcacatgaattacaccagcctgcgcagatgcgcaggttttttttgccggtcatcaatctgtaacagtaaccgacaattt
acacacctcgttgcatttcccttcattcctttgcgttttctcgctggcgaagagtcgtcgtgcagaccacaatcaagatcccagaggtattgattggt
gagattattcggtacgctcttcgtaccctgtctcttgcaccaacctgcgcggatgcgcaggttttttttcgcacctaatttactgtcgctcgcgttcttat
ctcgtgtaatctccctccattcgcttttactgaatcagagcaaagggagttggaatgctttttagcttttttcgaaatttgtgccgtgttttgtatcgcgttc
gcgttacgggtgacacccaggcactgaagggcgagcgcgttctaattacgcctaatcacgtctcttttattgatggcattttgcttggactgtttttac
ctgtgcgtccagtgtttgccgtttacacctcaataagccaacagtggtatatgcgttggctgaaatcatttatcgactttgttcctctcgacccgacgc
aacctatggctattaaacatctggtacgtctggtggaacagggccgaccagtggtgattttccctgaaggacgcatcaccacgacaggctcgct
gatgaaaatctacgatggcgcgggttttgtcgcggcgaagtctggtgcaacggttattcctgtgcgtattgaaggggcggaacttacgcacttca
gccgcctgaaaggtctggttaaacgtcgcttgttcccgcaaattactctgcatattttgccaccaacgcaggtggcgatgccggatgcgccgcgt
gcccgtgaccgtcgcaaaatcgctggcgaaatgctgcatcaaataatgatggaagcgcgaatggcggtgcgcccgcgtgaaacgctgtacg
aatctttactgagtgcaatgtaccgcttcggagccgggaagaaatgtgtcgaagacgtcaactttaccccagactcctatcgcaaattgcttacg
aaaacgctgtttgttggacgcatccttgaaaaatacagtgttgaaggcgaacgcatcggcttaatgctgcccaatgcaggcatcagtgcggca
gtgatttttggggccatcgcccgtcgccgcatgcccgcaatgatgaactacactgccggggtaaaagggctgaccagtgctattacggcggct
gaaatcaaaaccatcttcacttcccgccagtttctcgataaaggcaaactctggcatctgccggagcaacttactcaggtgcgctgggtctatct
ggaagatttaaaagcagatgtcaccactgccgacaaagtatggatcttcgctcatttgctgatgccgcgtctggcacaggttaaacagcagcc
ggaagaagaggcgctgatcctttttacctccggttctgaaggccatccgaaaggcgtcgtccatagccataaaagcattctggcgaatgtcga
gcagattaaaacgattgccgacttcaccaccaacgatcgctttatgtcggcgttaccgctgtttcactcctttgggctgacggtaggcctgtttacg
ccactgcttacaggtgcagaagtgttcctttatccaagcccgctgcattaccgcattgtgccggagttggtgtatgaccgcagttgcaccgtgttgtt
cggcacctcgactttcctcggtcactacgcgcgtttcgccaacccgtatgacttctatcgtctacgctatgtggtggcaggcgcagaaaaattaca
agaaagtaccaaacagctttggcaggataaatttggcctgcgcatccttgaaggctacggcgtgaccgaatgcgcgcctgtcgtttctatcaac
gtaccgatggcggcgaaacccggtacggtagggcgtattctaccaggaatggatgcgcgcctgttgtcggtccctggtatcgaagagggcgg
acgcctgcaactgaaagggccgaacataatgaacggctatctgcgggtggagaagccaggtgtactggaagtgcccaccgccgagaatgt
tcgcggcgaaatggagcgcggctggtatgacactggcgatattgtgcgttttgacgagcagggctttgtgcagattcagggccgcgcaaaacg
ctttgccaaaattgcaggcgaaatggtgtcgctggaaatggtggaacaactggcacttggtgtttcgccagataaagtccatgccactgcgatta
agagcgatgccagcaaaggcgaggcactggtgcttttcaccacagataacgaactgacgcgcgataagttgcaacagtatgcccgcgagc
acggcgtgccggagcttgctgtaccgcgcgatattcgctatctgaaacagatgccattacttggcagcggcaaacctgactttgtcacgttgaaa
agctgggtagacgaagcggaacaacacgatgagtgagtcagtgcacactaacacttcgttgtggtcgaaggggatgaaagcggttatcgtg
gcgcagtttctctctgcgtttggcgataatgccctactgtttgccactctggcgttactgaaagcgcagttctatccggagtggagccagcccatcct

gcaaatggtgtttgtaggtgcttacattctttttgcgccgtttgtcgggcaggtggcggatagcttcgccaaaggccgggtgatgatgtttgccaacg
gcctgaagctgctgggcgcagccagtatctgctttggtatcaatccgtttctcggctatacgctggtgggtgttggtgctgcagcctattcaccggc
gaaatacggtattctcggcgaattaaccacgggtagtaagttagtgaaagctaacggtttaatggaagcttctaccatagcggcgattttgctcg
gttccgtagccggtggtgtgctggctgactggcatgtcctcgtcgccctggccgcatgcgcactggcctacggtggtgcggtcgttgccaatatct
acattcccaaactggcggcggcgcgtccggggcagtcctggaatctcatcaacatgacccgcagtttcctgaatgcctgcacctcgctatggc
gcaatggtgaaacgcgtttttcgctggtgggcaccagtttattctggggagcgggtgtcacgctgcgtttcctgttggtgctgtgggtaccggtggc
gctgggcattaccgataacgctacgcccacctatctcaacgcgatggtagcgattggtatcgtggttggcgcaggtgcggcagcgaagttagtt
acgctggaaaccgtgtcacgctgtatgccagccgggattttgattggcgtggtggtactgattttttccctgcaacacgagctgctgccagcctatg
ccttgttgatgctgattggcgtgatggggggctttttttgtcgttccgctcaatgcgttgctacaggagcggggtaaaaaaagcgtcggggcgggg
aatgcgattgcagtacaaaaccttggcgaaaacagcgccatgttgttgatgctgggcatttactcgctggcggtaatgataggcatcccggtcgt
gcccattggcattggcttcggtgcgctgtttgcgctggcaataacggcgctgtggatctggcagcgccgtcattaatatttaacgccggttttaacc
ggcgttaatcttatggtgccggataagtataaacctgatgcaccgcttcaatttcagctaatacgtcttcgcttaactccagatgcaaactttcgatg
ttagttttcagctgatccatcgtggttgcgcccagcagagtgctggcaacaaacggttgacggcgtacaaacgcgagcgccatctgagcaggg
tccaggccatgacgtctggcgatatcaacatacgccgcgacggctttttgcgtttgctcaccgctatagcgggtgaaccgactaaagagcgtatt
acgtgcgccagcgggttttgcaccattgagatatttcccggtcagcgtgccgaaacccaggcacgaataggccagcagttcgaccccttcata
ctggctgacttctgccagacctacttcaaaactgcggtttaacagactgtaagggttctgaatggtgacaatacgcggcagatcgtgtttgtccgc
cagatgcaggtagcgcattacgccaaatgcagtttcgttcgacacgccgatataacgaatttttcccgcgcgttggtactctgccagtgcgtcca
gcgtatccagcagcgaaaccgcaggcgcagaatccgtccagctataaccgagtttgccgaagcagttggtcgggcgctgcggccagtgca
cctgataaagatcgaggtaatcagtctgtaggcgcttgaggctgtcatgcagcgcttcgcggatattcttccgatccagcgcctgatccgggcgg
atgcccttgtcattattgcgcgacggtccgctcactttggaggcgataattaacttttcgcggctgccatgtttcgccagccagttgccgacgtaggt
ttcggttaacccttgcgtttcggggcgcggaggtactgggtacatttcggcaacgtcgataaggttaatgccctgagcgacggcatagtcgagtt
gtgcgtgggcgtcggcttcgctgttctgttcaccaaacgtcatcgtgccaagccccagcgtgctgacttccagcgaactgtggggtatacggtga
tattgcatagccgtttcctttttataatcacgacataaggaatataaaaatggcagagggaagggaaaagggaaagagaaaaatcttaaggc
cagccggatgctgaccttatttgttaacgttcaataatttgcgaaacatcatcacggttaatttgcatcgcgttaccttgctgatcgtgataactcacc
agcccggtatcatcatcaatttcaggttttccatcggtcaaaatcatacggccatctttggtcgccatgacgtaatcactggaacacccggaaac
agcaaacgccagtccgactgcagaaattattactgcccatttttttcatccggttatcctcatttggcctacctatgataatagtctgtttataaaccga
cgttatatatcttgaaagcaggaaaatcttaaactgtgagggtaaaaggagtgagtacgccctcccgcaggagagcgtacggagtatcagag
cggattctttttgttgcgaatcaagttgaggctttcaaccgcaatagagaagaacatcgcgaagtagatgtaacctttcggtacgtggatgtcgaa
actttccagaatcagggtaaagcccaccagaatcaggaaagagagcgccagcatttttaccgaaggatggcgttcgacaaaatcaccaatc
gagcgcgcggcgaacatcatcacgcctacggcaatcaccacggctgccatcataataaacaggtgatctgacagacccacagccgtaatc
accgagtcgaggctaaagataatatccagcagcataatctgcacgatagcgccgaggaatgatgaaacgcgtgttttcagcccttcttcttcac
cttcaatggattcgtggatttccttgctggctttccagataaggaacaagccacccagaagcagaatcaaatcacgggcagaaatttcctgact
gaatattgtaaaaagcggatttgtcaggcgcgtaacccaggcgattgatgccagcagcgccagacgcataaccatggctcccgccaacccc
agacggcgcgcatgagcacgttgtgctgtgggaagctttgccaccaccagagaaaggaaaataatattgtcgatcccaagaacgatctcca
gcagcgtcagcgtaccgagcgcaagccaggcgttaggatcggttatccatgcaaataacatctaaaaagccctgccaaaaaaatgaagcg
gtaattatatgcccggaaagcggcaggtcaaagcgatggctactggatcagaaaatgacgggccagtagtgcactggtgaaattcttcttcaa
ataaaagccgcgtggcagcgtcagaatccgttcgccccgggcaccaatggcttcggtaagcgctttcgcattcgctgctttcggtcgtatctgta
aatactccccgtgacgagcggtaatccgctcaacctgaccgagaacaatcatatccattaattcttcccagtcttcgcgtagctgccggtcttcct
cttcattcgggctccacagcaacggtgatccaacgcgacgctgcgccagcgggatgctgcgctcgccttcaaccggtatccacagtacgcgtt
tgagtttgtggcgcacgtggctggtttcccaggtcaccccgctattgcccgttaacggggcaacacaaacgaatgttgtttccagcggacgacc
aagactatccacagggatagttttaagttccacgcccagagcagcaaaatcttgctcaggtttactccctgcgctggcacctagccagatctcca
gtaacacgccaatccagcctttatcgcgttttaaattctctggcgtaaccagcccgacaagtgccgccagttctcccaatgtataaccagaaagtt
gctgtgcttgcgctaacaactgttcttcagtttcgggaggagagagcagtgggcgaggttgggacatgtcatgataccttgattaaaaaacaaa
cagcaattatgtgccgaatattcgcagtgtaactttttccacatgcgttgttcaattctctgatttatattgctttttaggttgccggtaatcgagttgcgca
acactgcaacgggttttccaaatctggtcactgaaaataaacaggatcttacaccatgttatccacagaaagctgggataactgtgaaaaacct
ccactactgtttccatttacagccttgacgtgcgacgaaactccagtttttagtggttcagtgcttaacttagtggcacattctgtggataaatacggc
attggttgatctttcgccaacccgtaaatgagaggtgtgacctgggtcacgaattactctttttcattactaaaattacatgtaatgcattgactgata
atgaatattttaaaatgtctatatcgggcttattcagaatgctccgggttttcccgtagtaattccgtagttcttcacaactctatccacagaaaaggtg
aataaaaacggctatccacccccttcctctgtttataactctgcacataactgtgagttattcaattgttattagtgtgtaatgccctgcagagagtggtt
taccgtcttcctggagtatgaaacaatcattcgtatataaagctttattttgaggtagtccggtgattgatgacgatggctaccgcccaaacgtaggt

atcgtgatttgtaatcgccaggggcaggtaatgtgggcccggcgatttggtcagcactcctggcaatttccgcaaggcggaatcaaccccgga
gaatccgcagagcaggcgatgtaccgtgaattgtttgaagaagtaggattaagccgcaaagacgttcgaatccttgcttcaacgcgtaactggt
tgcgctacaaattaccgaaacgtttggtgcgttgggacacgaagccggtttgtatcggccaaaaacaaaaatggtttctcttgcagctggtgagc
ggcgatgcagaaatcaatatgcaaaccagcagtacaccagagtttgacggctggcgatgggtaagttactggtatccggtcagacaggtggt
gtcatttaaacgtgatgtctaccgtagggtaatgaaagagttcgcgagtgtggtgatgtcactgcaggaaaatacgccaaaaccacaaaacg
catctgcttatcgacgtaaaagaggttaagtcacgccaattatgctcactcgcctgcgcgaaatagtcgaaaaggtagccagcgcaccacgc
ctgaatgaggcgttaaatattctggttaccgacatctgtcttgcgatggataccgaggtctgttcggtctacctggccgatcatgatcgacgttgtta
ctacctgatggcgacccgggggctgaaaaaaccacgcggtcgcactgtaacgctcgcgtttgatgaagggatcgtcggcctggttggcaggc
tggcggaaccgataaaccttgcagatgcgcaaaagcaccccagcttcaaatacatcccctccgtaaaagaagaacgtttccgcgcgtttttag
gcgtaccaattattcaacgtcgccagttgcttggtgtactggtggtacagcaacgagagttgcgccagtatgacgaaagtgaagaatccttcctg
gtgacgcttgccacccagatggcagctattctttctcagtcgcagttgactgccttgtttgggcaatatcgccagacgcgaatccgcgcattaccg
gcagcacctggtgtggcgattgccgaaggctggcaggatgccacgttacctttaatggaacaggtgtatcaggcatcaacgctggatccggct
ctggaacgcgaacgactgaccggggcgctggaagaagcggcaaacgagtttcgccgctacagcaaacgctttgccgccggtgcacaaaa
agaaacggcggctattttcgatctttactcgcacctgctttcggacacccggctgcgtcgcgaattgtttgccgaggttgataaaggctcggtggc
agagtgggcggtaaaaacggtcattgaaaaatttgccgaacagtttgccgcgctaagtgataactatctcaaagagcgggctggcgatttacg
tgcgctgggtcagcgattgctgtttcatcttgatgacgctaatcaagggccgaacgcctggccggaacgtttcattctggtggcagatgaactgtc
agcgacaacgcttgctgagctgccccaggatcgcttagtcggtgttgtcgtgcgagatggcgcagccaactcccatgctgcgatcatggtacgt
gcgctggggatccctaccgtgatgggcgcggatattcagccttcggtgctgcatcgtaggacgctgatcgttgatggctatcgcggtgaattgct
ggtcgatccggagccggtactgctgcaagaatatcagcggctaattagtgaagagattgagcttagccgtctggcggaagatgacgtcaattt
acccgcgcagttaaaaagcggtgagcgtataaaagtcatgctcaatgctggtttaagcccggaacatgaagaaaaactgggcagccgtattg
atggcatcggtctttatcgcactgaaatcccattcatgctgcaaagtggtttcccgtcggaagaagaacaggtggcgcagtatcaggggatgct
gcaaatgttcaatgataaacccgtcaccttgcgtacgctggatgtcggagcagataagcagctgccttacatgccgatcagcgaagagaatcc
atgcctgggttggcgtgggattcgcattacgctcgatcagccggagatcttcttgatccaggtgcgggcgatgctgcgtgctaatgccgctacgg
gcaacctgaatattctgttgccgatggtcacaagcctcgatgaagttgatgaagcacgccgcctgattgaacgtgccggacgtgaagtcgagg
agatgatcggttacgaaattcccaaaccacgtatcggcatcatgctggaagtgccgtcaatggtatttatgctgccgcatctggcaaagcgggt
cgatttcatctctgttggcaccaacgatctgactcaatacattctggccgttgatcgcaacaatacccgggtggcgaacatttatgacagtcttcat
cctgcaatgttacgagctctggcgatgatcgcccgggaagcggaaatacatggaatcgatctccgtttgtgcggtgaaatggcgggcgatccc
atgtgcgtggcaatcctcattgggcttgggtatcgccatctgtctatgaacggacgttctgtagcgcgggcaaaatacctgctgcggcgcattgat
tatgccgaagcagaaaatcttgcgcagcgtagtctggaagcgcaactggcgaccgaagttcgccatcaggttgcagcctttatggagcgtcgc
ggcatgggcgggctgattcgcggagggttatagcgcggatcatatacatatcttttaacggtatccggcaaccagccaggtccccttgtgctatt
attcgcacctttggagcgcctgaaacctgcggcgcgcatttcaatcgctgttctctttcagcgaaataacaagaacttgtggtgacagatgacca
gtagctatctgcattttccggagtttgatccggtcattttctcaataggacccgtggcgcttcactggtacggcctgatgtatctggtgggtttcatttttg
caatgtggctggcaacacgacgggcgaatcgtccgggcagcggctggaccaaaaatgaagttgaaaacttactctatgcgggcttcctcggc
gtcttcctcgggggacgtattggttatgttctgttctacaatttcccgcagtttatggccgatccgctgtatctgttccgtgtctgggacggcggcatgtc
tttccacggcggcctgattggcgttatcgtggtgatgattatcttcgcccgccgtactaaacgttccttcttccaggtctctgattttatcgcaccactca
ttccgtttggtcttggtgccgggcgtctgggcaactttattaacggtgaattgtggggccgcgttgacccgaacttcccgtttgccatgctgttccctg
gctcccgtacagaagatattttgctgctgcaaaccaacccgcagtggcaatccattttcgacacttacggtgtgctgccgcgccacccatcaca
gctttacgagctgctgctggaaggtgtggtgctgtttattatcctcaacctgtatattcgtaaaccacgcccaatgggagctgtctcaggtttgttcct
gattggttacggcgcgtttcgcatcattgttgagtttttccgccagcccgacgcgcagtttaccggtgcctgggtgcagtacatcagcatggggca
aattctttccatcccgatgattgtcgcgggtgtgatcatgatggtctgggcatatcgtcgcagcccacagcaacacgtttcctgaggaaccatgaa
acagtatttagaactgatgcaaaaagtgctcgacgaaggcacacagaaaaacgaccgtaccggaaccggaacgctttccatttttggtcatc
agatgcgttttaacctgcaagatggattcccgctggtgacaactaaacgttgccacctgcgttccatcatccatgaactgctgtggtttctgcaggg
cgacactaacattgcttatctacacgaaaacaatgtcaccatctgggacgaatgggccgatgaaaacggcgacctcggcccagtgtatggta
aacagtggcgcgcctggccaacgccagatggtcgtcatattgaccagatcactacggtactgaaccagctgaaaaacgacccggattcgcg
ccgcattattgtttcagcgtggaacgtaggcgaactggataaaatggcgctggcaccgtgccatgcattcttccagttctatgtggcagacggca
aactctcttgccagctttatcagcgctcctgtgacgtcttcctcggcctgccgttcaacattgccagctacgcgttattggtgcatatgatggcgcag
cagtgcgatctggaagtgggtgattttgtctggaccggtggcgacacgcatctgtacagcaaccatatggatcaaactcatctgcaattaagcc
gcgaaccgcgtccgctgccgaagttgattatcaaacgtaaacccgaatccatcttcgactaccgtttcgaagactttgagattgaaggctacgat
ccgcatccgggcattaaagcgccggtggctatctaattacgaaacatcctgccagagccgacgccagtgtgcgtcggtttttttaccctccgttaa
attcttcgagacgccttcccgaaattttgcaacgtcctgcaacggcgtaaatagtccggaagatgcgccgaagaaatagaaactggccctcgt

cttttttctcctcgctccatactgccggcatgaaaacacaacgtggttatacgctgattgaaacgctggtcgcgatgctgattttggtcatgctaagc
gcaagtggactctatggctggcaatactggcagcagtcgcaacggctatggcaaaccgccagccaggcgcgggactatttgctctatttacgt
gaagatgccaactggcataaccgcgaccacagtatcagcgttatcagggaggggacgttatggtgccttgtgagttccgctgctggggccaat
acctgtcatggcagttcaccattggtctttgtgccacgctggcccgaagtcgaaatgagcgacctgacaccttcgcttgctttctttggcctgcgca
ataccgcatgggccgggcatattcgcttcaaaaactcaacgggcgagtggtggctggtggtttcgccgtggggaagactccggctttgtcagc
aaggagaaacagaaggatgcctgtaaaagagcaaggtttttctctgctggaagtgttgattgctatggcgatcagtagcgtattgttgctgggg
cggcacgctttctgcctgcgttacagcgtgaaagtttaacgagcacccgtaagctggcgctggaagatgaaatctggctgcgggtatttaccgtc
gcgaagcatctccagagggcgggttattgtcatggcatctgtaccggcgaagggctggaaattgtcggacagggtgactgtgtcattgtgcagt
gggatgcgaacagtaacggtatctgggatcgcgaaccggtaaaagagtccgaccagattggatttcgtctgaaggagcatgtgctggaaac
gctacgcggtgcgacatcctgtgaaggtaagggctgggataaagtcactaatccggatgccatcattatcgacacttttcaggtcgtacgtcag
gatgtcagcggcttctcgccggtgttgacggttaatatgcgtgctgccagtaagtctgaaccgcaaaccgtggtgaatgccagctatagcgtga
caggattcaacctgtgaaccgcgaaaagggtgtttcgtcactggctctggtcctgatgctgctggttttgggtagtttgctattacaaggaatgagc
caacaggatcgcagttttgcctctcgcgtgagcatggaaagtcagtcattgcgccgccaggccatcgttcagtcggcgctggcgtggggaaaa
atgcactgctggcagacgcagccagcagttcagtgctcgcagtacgctgaaaccgatgcccaggtttgtttgcgtttactggcagataatgaag
ccttattgattgccggttatgaaggcgtttcgttgtggcgaacaggcgaagtcattgatggaaacattgtttttcgccacgcggctggagcgattttt
gtccgctgaaagagagggcgttatgtcagcttccctgaagaatcaacaaggctttagcctgccggaggtaatgttggcgatggtgttgatggtg
atgattgtcactgcgttatcgggtttccagcgaacattaatgaacagtcttgccagcagaaaccagtaccaacagctctggcggcatggctggc
agcaaacgcaactgcgcgcgatttcgccacctgccaactggcaggtcaaccgaatgcagacatcgcaggcgggatgtgtcagcatcagcg
ttacgctagtttcacccgggggcagagaaggcgagatgacccgcctgcattgcccgaatcgtcagtagtcaggagccgctatgttaagggtct
accattccaatcgtctggacgtgctggaagcgttgatggagtttattgtcgaacgcgaacggctggacgatcctttcgaaccagagatgattctg
gtgcaaagtaccggtatggcacagtggctgcaaatgaccctgtcgcaaaagtttggtattgcggcaaacattgattttccgctgccagcgagctt
tatctgggatatgttcgtccgggtgttaccggaaatccccaaagagagcgcctttaacaaacagagcatgagctggaaactgatgactctgctg
ccgcaattgctggagcgcgaagactttaccctgttgcggcattatctgactgacgatagcgacaagcgaaaactgttccagctttcctcaaaag
cggcggacctgtttgaccagtatctggtctatcgtccggactggctggcacagtgggaaacaggacatttggttgaagggctgggagaagcac
aggcctggcaagccccgttgtggaaggcgctggtggaatatacccatcaactcgggcaaccgcgctggcaccgcgccaatctctatcagcg
ctttatcgaaacgctggagtccgcgacgacctgcccgccggggttaccttcgcgcgtctttatatgcggtatttccgcgttaccgcctgtttatctcc
aggcgctacaggcgctgggtaaacatattgaaatccatctcctgtttaccaacccctgccgttattactggggcgatattaaagatcctgcttatct
ggcgaaactgctgacccgtcagcgtcgacacagttttgaagatcgcgaattgccgctatttcgtgatagcgaaaatgccgggcagctctttaac
agcgatggtgaacaggatgtcggcaacccgctgctggcttcatggggtaagcttgggcgcgactacatttatctcctttctgacctggagagca
gccaggagctggacgcctttgtcgatgtcacgccagataacctgctgcataacattcagtctgacattctggaactggaaaaccgcgccgttgc
tggtgtgaacatcgaagagttttcccgtagcgataacaaacgcccgcttgatccactggatagcagtatcaccttccacgtttgccatagcccgc
agcgtgaagttgaagttttacacgatcgcctgctggcgatgctggaggaagacccgacacttactccgcgcgacatcatcgtgatggtggctg
atatcgacagctacagtccgtttattcaggctgtgtttggtagcgcacctgcggatcgttacctaccttacgccatttccgaccgtcgtgcgcggca
gtcacatccggtactggaagcgtttatcagcctgttatcactgcctgacagtcgttttgtgtcagaggatgtgctggcgttgctggatgtgccggtgc
tggcggcgcggtttgacatcaccgaagaagggctgcgttatttacgccagtgggtcaacgaatccggcattcgttggggcatagatgacgaca
acgttcgcgagctggaactccccgccaccggacaacacacctggcgatttggcctgacgcgtatgttgttgggctacgcgatggagagcgcg
cagggcgagtggcaatcggttctaccttatgatgaatcgagcggcttaattgcagaactggtggggcatctggcttcactgctaatgcagctaaa
catctggcgtcgcgggctggcacaggagcgtccgctggaagagtggttgccggtttgtcgcgatatgctcaacgccttcttcctgccggatgcg
gaaaccgaagcggcgatgacgctgatcgaacaacaatggcaggcgattatcgccgaaggtttaggtgcgcagtatggcgacgcggtgccg
ctgtcactattgcgtgatgaactggcacagcgtctggatcaagaacgtatcagccagcgttttctcgccggaccggttaacatttgtactctgatgc
caatgcgttcaattccgttcaaagtggtttgcctgctgggaatgaacgacggcgtttatccacgtcagcttgcgccattgggctttgacctgatgag
ccagaaaccgaagcgtggcgaccgtagccgtcgcgatgacgaccgctatctgttcctggaagcgttaatttccgcgcagcaaaaactctatat
cagctatatcggtcgttccattcaggataacagtgagcgtttcccgtcggtactggtgcaggaactgatcgactacatcggcaaagtcattatct
accgggcgatgaagcgctcaactgtgatgaaagcgaggcaagggtaaaagcgcatcttacttgcctccatacccggatgccgtttgatccac
aaaactaccagccaggcgaacgacaaagctatgctcgtgaatggctacctgcggccagccaggctggtaaagcacattctgaatttgttcag
ccgctgccgtttaccttaccggaaaccgtgccgctggaaacgctacaacgattctgggcacatccggtgcgggcatttttccagatgcgtttgca
ggtgaacttccgtactgaagacagcgaaatccccgacaccgagccatttattctggaaggacttagccgttatcaaatcaatcagcagttattg
aatgcactggttgagcaggatgatgccgaacgcttgttccgccgcttccgagcggcaggggatttaccgtatggcgcttttggtgaaattttctgg
gaaacacagtgccaggagatgcagcagcttgccgacagagtcattgcctgtcgccagccggggcagagtatggaaattgatctcgcctgca
acggtgtgcagataactggctggttgccgcaggtgcagccggatggcctgttgcgctggcgtccctctttattaagtgtggcccagggaatgca

actttggctggaacaccttgtctactgtgccagcggtggtaatggtgaaagtcgcctttttctacgcaaagacggcgagtggcgttttccgccgctt
gcagccgaacaggctttgcattacctctcacaactgattgaggggtatcgtgaaggaatgtccgcgccattgctggtgttacctgaaagtggcg
gcgcgtggctaaaaacctgttatgacgcgcaaaacgatgccatgctggatgacgattccacgttgcaaaaagcccgtacgaaattccttcagg
cttacgaaggcaacatgatggtgcgtggcgaaggtgatgatatctggtatcaaaggctctggcggcaattaacaccagagacaatggaggc
catcgttgaacagtcgcaacgtttcctgttaccgctgtttcgctttaatcagtcatgagtgctgtataaaaattgcgcaatctatccgcttactttatgat
gcgcaccagtcacggactgatggttatataaacataggctgactcgtgcagcacaagattaaattctggcagatgatttgcgttaacgtgttgaa
tctggacagaaaattaagttgattatgaggtccgtgaatgccccgcagcacctggttcaaagcattattgttgttagttgccctttgggcacccttaa
gtcaggcagaaacgggatggcagccgattcaggaaaccatccgtaaaagtgataaagataaccgccagtatcaggctatacgtctggataa
cggtatggtggtcttgctggtttctgatccgcaggcagttaaatcgctctcggcgctggtggtgcccgttgggtcgctggaagatcccgaggcgta
ccaggggctggcacattaccttgaacatatgagtctgatggggtcgaaaaagtacccgcaggctgacagtctggccgaatatctcaaaatgc
acggcggtagtcacaatgccagcactgcgccgtatcgcacggctttctatctggaagttgagaacgacgccttgcctggtgcggtagaccgcc
tggccgatgctattgctgaacctttgctcgacaagaaatatgccgaacgtgagcgtaatgcggtgaacgctgaattaaccatggcgcgtacgc
gtgacgggatgcgcatggcacaggtcagcgcagaaaccattaacccggcacaccccggttcaaagttttctggtggtaacctcgaaactttaa
gcgacaaacctggtaatccggtgcagcaggcgctgaaagatttccacgagaagtactattccgccaatttgatgaaggcggttatttacagtaa
taaaccgctgccggagttggcaaaaatggcggcggacacctttggtcgcgtgccgaacaaagagagcaaaaaaccggaaatcaccgtgc
cggtagtcaccgacgcgcaaaagggcattatcattcattacgtccctgcgctgccgcgtaaagtgttgcgcgttgagtttcgcatcgataacaac
tcagcgaagttccgtagtaaaaccgatgaattgattacctatctgattggcaatcgcagcccaggtacactttctgactggctgcaaaagcagg
gattagttgagggcattagcgccaactccgatcctatcgtcaacggcaacagcggcgtattagcgatctctgcgtctttaaccgataaaggcctg
gctaatcgcgatcaggttgtggcggcaatttttagctatctcaatctgttacgtgaaaaaggcattgataaacaatacttcgatgaactggcgaat
gtgctggatatcgacttccgttatccgtcgatcacccgtgatatggattacgtcgaatggctggcagataccatgattcgcgttcctgttgagcatac
gctggatgcagtcaatattgccgatcggtacgatgctaaagcagtaaaggaacgtctggcgatgatgacgccgcagaatgcgcgtatctggta
tatcagcccgaaagagccgcacaacaaaacggcttactttgtcgatgcgccgtatcaggtcgataaaatcagcgcacaaactttcgccgact
ggcagaaaaaagccgccgacattgcgctctctttgccagagcttaacccttatattcctgatgatttctcgctgattaagtcagagaagaaatac
gaccatccagagctgattgttgatgagtcgaatctgcgcgtggtgtatgcgccaagccgttattttgccagcgagcccaaagctgatgtcagcct
gattttgcgtaatccgaaagccatggacagcgcccgcaatcaggtgatgtttgcgctcaatgattatctcgcagggctggcgcttgatcagttaa
gcaaccaggcgtcggttggtggcataagtttttccaccaacgctaacaacggccttatggttaatgctaatggttacacccagcgtctgccgcag
ctgttccaggcattgctcgaggggtactttagctataccgctacggaagatcagcttgagcaggcgaagtcctggtataaccagatgatggattc
cgcagaaaagggtaaagcgtttgagcaggcgattatgcccgcgcagatgctctcgcaagtgccgtacttctcgcgagatgaacggcgtaaa
attttgccctccattacgttgaaagaggtgctggcctatcgcgacgccttaaaatcaggggctcgaccagagtttatggttatcggcaacatgacc
gaggcccaggcaacaacgctggcacgcgatgtgcaaaaacagttgggcgctgatggttcagagtggtgtcgaaacaaagatgtagtggtc
gataaaaaacaatccgtcatctttgaaaaagccggtaacagcaccgactccgcactggcagcggtatttgtaccgactggctacgatgaatac
accagctcagcctatagctctctgttggggcagatcgtacagccgtggttctacaatcagttgcgtaccgaagaacaattgggctatgccgtgttt
gcgtttccaatgagcgtggggcgtcagtggggcatgggcttccttttgcaaagcaatgataaacagccttcattcttgtgggagcgttacaaggc
gtttttcccaaccgcagaggcaaaattgcgagcgatgaagccagatgagtttgcgcaaatccagcaggcggtaattacccagatgctgcagg
caccgcaaacgctcggcgaagaagcatcgaagttaagtaaagatttcgatcgcggcaatatgcgcttcgattcgcgtgataaaatcgtggcc
cagataaaactgctgacgccgcaaaaacttgctgatttcttccatcaggcggtggtcgagccgcaaggcatggctattctgtcgcagatttccgg
cagccagaacgggaaagccgaatatgtacaccctgaaggctggaaagtgtgggagaacgtcagcgcgttgcagcaaacaatgcccctga
tgagtgaaaagaatgagtgatgtcgccgagacactagatcctttgcgcttgcccttacagggtgagcgcctgattgaagcctctgccggcaca
ggcaaaacctttacgattgcggcgctctatttgcgcctgttacttggactaggcggttccgccgcctttccccgcccgctgaccgttgaagaactg
ctggtggtcacctttaccgaggctgccacggcagaattgcgcggtcgtatccgtagcaatatccacgagttgcgcatcgcctgtctgcgtgaaa
ccaccgacaatccactgtacgaacgcctgctggaagagatcgacgataaagcgcaagccgcgcagtggttgttgttagccgaacggcagat
ggatgaagcggcagtctttactattcacggcttttgccagcgcatgctcaacctgaatgcctttgaatccggcatgctgtttgagcagcagctgatt
gaagatgagtctctgctacgctaccaggcctgcgccgatttctggcgtcgccactgctacccgctgccgcgtgaaatagcccaggtcgtctttga
aacctggaaagggccgcaggcgttgctgcgcgatattaatcgttatctgcaaggcgaagcgccggttatcaaagcaccgccgcccgatgatg
aaacgctggcttcccgtcacgcgcaaattgtggcgcgtattgatacggtaaaacagcagtggcgcgacgcagtgggtgaactggatgcgctg
atcgaatcttctggtattgatcgacgcaagtttaaccgtagcaatcaggctaaatggatcgacaagatcagcgcctgggcagaagaagagac
aaacagttatcagttgccggagtcgctggaaaaattctcccagcgtttcttagaagatcgcacgaaggccggggggaaaccccgcgacatc
cactgtttgaggcgatcgatcaactgcttgcagaaccattgtcgatccgcgatctggtgatcacccgcgcattggctgagatccgcgaaacagt
agcgcgtgaaaaacgccgccgtggcgaattgggttttgatgacatgttaagtcggctcgattccgcgctgcgtagcgaaagcggtgaggtgttg
gcagcggcgatccgtacgcgattcccggtggcaatgatcgatgaatttcaggataccgacccccagcagtaccgaatttttcgccgtatctggc

accatcagccggaaaccgcattgttgctaattggcgacccgaagcaggccatatatgcattccggggtgcggatatcttcacttatatgaaggc
gcgtagcgaagttcacgcccactacactttagacaccaactggcgttccgcaccaggaatggtgaacagcgtgaataagcttttcagccaga
ctgatgacgcgttcatgtttcgcgaaataccgtttattccagtgaaatcagccgggaaaaatcaggcgttacgttttgtatttaaaggtgaaacac
agcctgcgatgaaaatgtggctgatggaaggcgaaagctgcggcgttggcgattatcaaagtaccatggcgcaggtatgtgctgcgcaaatc
cgcgactggctacaagccggacagcggggcgaagcgttgctgatgaacggcgacgacgcgcgtccggtgcgtgcttcggacatcagtgtg
ctggtgcgcagccgccaggaggccgcccaggtgcgcgatgccttaacgttgctggaaatcccttccgtttacctttcgaaccgcgacagtgtttt
gaaactctggaagcgcaggaaatgctttggttgttgcaggcggtgatgacgcccgaacgtgagaacaccctgcgtagtgcgctggcaacgtc
aatgatggggctgaacgcgctggatatcgaaacgctgaacaatgacgaacatgcgtgggatgtggtagtcgaagagttcgatggttatcggc
aaatctggcgcaaacgtggcgttatgccgatgctgcgggcgctgatgtcggcgcgtaacattgctgaaaacttgctggcaacggcaggcggt
gagcggcgtcttaccgatatcttgcatatcagcgaactgctacaagaagccggaacgcagctggaaagtgaacatgcgctggtacgctggtt
atcgcaacatatcctcgagccagacagtaatgcctccagccaacaaatgcgtctcgaaagtgataaacatctggtgcagattgtcacgatcca
caaatcgaaagggctggaatatccattggtctggctgccgtttatcaccaatttccgcgtccaggagcaggcgttttatcacgatcgccactcgttt
gaggcagttctggatcttaatgctgcgccagaaagcgtcgacctcgcggaggccgaacgtctggcggaagatctgcgtttgctttacgtggcgc
tgacacgttcggtttggcattgcagtctcggcgttgcaccgctggtgcgccgtcgtggcgataaaaaaggtgacaccgacgtccaccaaagtg
cgctcgggcgtttgctgcaaaaaggggaaccgcaagatgcggcagggcttcgcacctgtattgaagcgttatgcgatgatgatattgcctggc
aaacggcacaaactggtgataaccaaccctggcaggttaatgatgtttctacagcagagctgaatgcgaagacgttacaacgattgcccggc
gataactggcgcgtcaccagctactctggtttgcaacagcgtggtcacggtatcgcccaggatttgatgcctcggctggatgtcgatgctgcagg
cgttgccagcgtcgttgaagaaccgacgttaacaccacatcagtttccgcgcggtgcgtcaccggggacgttcttgcacagtttgtttgaagacc
tggattttacccagccggttgacccgaactgggtgcgggaaaaactggaactcggcggctttgaatcgcagtgggaaccggtattgaccgagt
ggatcacggctgtcctccaggcacctctcaatgaaaccggcgtaagcctgagtcaactttccgcccgcaataaacaggtggagatggagtttt
atctgccgattagtgaaccgcttatcgccagtcagcttgatacgttaatccgccagtttgacccgctatccgcaggctgcccgccgctggagttca
tgcaggtacgtggcatgttaaaaggctttatcgacctggtgttccgccacgaagggcgttattacctgctcgactataaatccaactggttgggtg
aagacagttcggcttacacccaacaggctatggcagcggcaatgcaggcacaccgctatgatctgcaatatcagctttataccctggcgctgc
atcgttatctgcgccatcgcattgctgattacgactatgagcaccactttggcggcgttatttatctgttcctgcgtggcgttgataaagaacatccgc
aacaggggatttacacaacccgacccaacgccgggttgattgccctgatggatgagatgtttgccggtatgaccctggaggaggcgtaatga
aattgcaaaagcaattactggaagctgtggagcacaaacagctacgcccgctggatgtgcaatttgccctgaccgtggcgggagatgaacat
cctgccgtcaccctcgcggcggcactgttaagtcatgatgccggagagggacacgtttgtttgccgctttcacgactggaaaataacgaggcgt
cgcatccgctgttggcgacctgtgtcagtgaaatcggtgagctacaaaattgggaagaatgcttgctggcttctcaagcggtcagcaggggag
atgaacccacgccgatgatcctctgtggcgatcgtctttatttgaatcgcatgtggtgtaacgagcgcacagtggcacgctttttcaacgaagtga
atcatgccattgaggttgatgaagctctactggcgcaaaccctggacaaactttttccagtaagcgatgaaattaactggcaaaaagttgcggc
ggcagtggcgctgacgcggcggatctcggtgatttccggcggccctggcaccggtaaaacgaccaccgtagcgaagttgctggcagcgtta
attcaaatggccgacggcgaacgctgccgtatccgtctggctgcaccaacgggtaaagctgccgcgcgcttaaccgaatctctcggcaaggc
tttgcgacagttaccgctgaccgatgaacaaaagaaacgcattccggaagatgccagcactttgcaccgattgctgggcgcgcagccgggta
gccagcgtttacgtcatcatgccggtaacccgctgcatcttgatgtgctggtggtagatgaagcgtcaatgatcgatctgcctatgatgtcgagac
tgatcgacgccttgcccgatcatgcgcgagtgatctttctcggcgatcgtgatcaactggcctcggttgaggctggggctgtgctgggcgatatct
gcgcttatgccaacgcgggctttaccgccgagcgtgccaggcagctaagccgcctgacggggactcacgttccggcaggaaccggcacag
aagcggcatctttgcgcgacagtctctgcctgctgcaaaaaagctatcgtttcggcagcgattctggcattggtcagttagctgcggcgatcaac
cgtggtgataaaacggcagtgaaaaccgtttttcagcaggattttactgatatcgaaaaacggcttttacagagcggcgaagattatattgcgat
gcttgaggaagctcttgcgggttacggacgttatctggatctgctgcaagcgcgtgccgagccggatttaatcattcaggcgttcaatgagtacc
agcttttgtgcgccctgcgggaagggccgtttggcgtggctggactgaatgagcgaattgagcagtttatgcaacagaagcgcaaaattcatcg
tcatccgcactctcgttggtacgaaggtcgaccggtgatgattgcccgtaatgacagcgcgcttgggttgtttaatggcgatatcggtattgcgctg
gatcgcgggcaggggacgcgcgtctggtttgcgatgccggacggcaatattaagtctgtgcaaccgagtcgcttgccagagcacgaaactac
gtgggcgatgacggtacataaatcgcagggatcggagttcgaccatgcggcgttgattttgccgagccaacgcacgccggtagtaacgcga
gagctggtttataccgcggtgacccgcgcgcgtcgccgtctgtcgctgtatgccgatgagcgcatattaagtgcggcaatcgccactcgtactg
agcggcgcagtggtctggcggcgttgtttagttcacgggaataaacgtaattgccggatgcgacgtacgagtgttacgcatgtcgcatccgacg
attttcatcgcttaccctaaatccgccatcaacactttggatttacgctggtagttgtacaactgctttttgctctcgggcagtaaatcaatatccactg
gggtaaatccacgttcctggaaccagtgaatactgcgcgtggtcagcacaaacaatttgcttaagccgctctgcttcgcctgagcggcaatgcg
ttccagcagaacttcaccccttgatgaactgcggtaatccgggtgaactgccacacaggccatttccccaatcttctcttccgggaacggataga
gcgcggcgcaggcaatagtcgtgttatcgcgctgaataatggtgaatttgtcgatttccatctccagctgctcgcgagaacggcgtaccagaat
accttgctgctccagtgggcgaatcaactccagaataccgccaatatcgttgattgttgcgcgacgaatctgctcggcgctttccatcacaatctg

cgtaccgataccgtcgcgtgagaacaactcttgcaacagcgcgccatcttcctgataactgattaaatgacagcgacgcacgccgctgcggc
aggctttcactgcgccacgcaaaaagcgcaccgtaccggagttgtaatcgcctttctcttcctgggcttctacccgcgcttgcgcttcgttaggga
aaagttcggagacaatatcaccgtcgtcattagtgacgccctgggaagagcaaaaaccaatcatcttttcagctttcagtttgatggccagttga
gtggcaatctcttccgaggtcaggttaaagctctcgccagtgactgaaacagcgaccggccccattagcactattgcaccgctgtccagttgac
gatggatcgcgtcttcatcaatccgccggatacgcccgctatggcagtaatccacgccgtcatcgacgcccagcggctgggcaataataaaat
tgccactgacgacgttgatatgcgcgccctgcagcggcgtgttattgagactcatcgacaggcgagcagtaatatccagttgcaatgttcccgc
agcctgcttcaccagttccagtgttttggcgtcggtcacacgtatattcttgtgatacagcggttcgtggtgatgcgcagccagatttgcgtcgatctg
cggacgtgcgccatagaccaccaccagacggatgccgaggctgtgcaacaacccgatatcattaacgatactggagaaattctcatgctca
atggcttcaccgccgagcatgatgacaaacgtttttccccggtgggtattgatatagggaaccgaatggcggaatccctcgaccaactcggtttt
acgttcctttaccacggcacacctctttgcatgattattcgaaattagtgtatttttattctgtttttcgcagggtgcaagtgtaaattttatgcgagagcg
actttttatcagtaatgccgtgaatataaaaagaatgtttaccgtttattagatgacagattatgcgtctttcgctaaagtttccggtcaaattagtcgtt
tacttgttacacagcttagatttcttttgctcgggagaggcatgtcaggatccaacactgcaatcagccgtcgtcgtttactgcaaggcgcgggtgc
catgtggctattgagcgtaagtcaggtcagtctggctgcggtcagccaggtcgtggcggtgcgcgtctggcctgcgtccagctacacccgcgtg
acggtggaatcaaatcgtcagctgaaatataagcagttcgcgttgagtaaccctgaacgcgtggtggtggatatcgaagatgtaaacctgaac
tcggtactcaaggggatggcggcacaaattcgtgcagacgacccgttcatcaagtcggcgcgcgtcgggcaatttgacccgcaaaccgtac
ggatggtttttgaattaaagcaaaacgtaaaaccgcagctgtttgcccttgcgccggtcgccgggtttaaagagcgtctggtgatggacctctatc
cggccaatgcacaggatatgcaggacccgctgctggcgctgctggaggattacaacaaaggcgacctcgaaaagcaggtgccgccagcg
caaagtggtccacaaccgggtaaagcagggcgcgatcgtccgattgtcattatgcttgaccctggccacggtggcgaagactccggtgcggt
ggggaaatacaaaacgcgcgaaaaagatgtggtattgcaaatagctcgccgtttgcgctctctgatcgagaaagagggcaatatgaaggtgt
acatgacgcgcaatgaagacatcttcattccattgcaagtgcgcgtagcaaaagcccagaaacagcgtgctgacctgtttgtctctatccatgc
cgacgcctttaccagtcgtcagccgagcggttcctctgtgtttgcgctctcaaccaaaggtgcaaccagtactgcggcaaaatatctggcacaa
acccagaacgcctcggacttgattggtggcgtgagcaaaagcggtgaccgctatgtcgaccacaccatgttcgatatggtacagtcgctgacc
attgccgacagcctgaagtttggtaaagcggtactgaataagctcggtaaaatcaacaagctgcataaaaatcaagttgaacaggccgggttt
gccgtactaaaggcaccagatattccctccattctggtcgaaacggcgtttatcagtaacgttgaggaagagcgtaaactgaaaacggcgact
ttccagcaggaagttgcggagtctattcttgcggggattaaagcgtattttgccgatggggcgacgctggcgagaaggggatgataaaaaggc
gctgaatggcgctttttttatgggagaccagaaacaaaaaaacacccgttagggtgttcaataattggttgcgggggccggatttgaaccgacg
accttcgggttatgagcccgacgagctaccaggctgctccaccccgcgtcaccgtactgcttttacttcatcaaatttgattggttgcgggggccg
gatttgaaccgacgaccttcgggttatgagcccgacgagctaccaggctgctccaccccgcgtcaccgtactgctttacttcatcaaattttaattg
gttgcgggggccggatttgaaccgacgaccttcgggttatgagcccgacgagctaccaggctgctccaccccgcgtccgtggatgcgcactat
actccgctcgcgttttcatgcaaccttttttttaccccgatcacggatttggcatgattttgaacaaaaacaaatcactaaagcctattttttgtgcaaa
atttgcgtcagggagaggctgctcatttcattaaacgcggcggtttgttatcttcgttgcgccttattttttaacctgaagaagagaacaatgaaagg
acgttgggtaaagtaccttcttatgggcacggttgtggcaatgcttgccgcctgctcttccaaaccaaccgatcgcggacagcaatataaagac
gggaaatttacccagcctttctctctggtgaaccagccagatgccgttggcgcgccgattaacgccggtgattttgccgagcaaattaaccatat
ccgtaattcgtcaccgcgtctgtatggcaaccagagtaatgtttataacgcggtgcaagagtggctgcgcgcaggcggtgatacccgcaatat
gcgccagttcggcattgatgcctggcagatggaaggtgccgacaactatggtaacgtgcagtttaccggttattacacgccggtaattcaggcg
cgccatacccgccagggcgagttccagtatcctatttaccgtatgccgccaaaacgtggtcgtctgccgtctcgtgcggagatctacgcggggg
cattgagtgataaatatattctcgcttacagtaactccctgatggataacttcattatggatgtgcagggtagtgggtatatcgactttggtgatggca
gtccgcttaactttttcagctatgcagggaaaaacggtcatgcctatcgcagcattggtaaggtgctgatcgaccgtggcgaagtgaaaaaaga
agatatgtcgatgcaggcgattcgtcactggggcgaaacacacagtgaagccgaggttcgcgagctgctggaacagaacccgtctttcgtctt
ctttaaaccgcaatcttttgctccggtgaaaggggcaagtgcggtgccgctggttggtcgcgcgtcagttgcctctgatcgttccattattccgcca
ggtactaccttgctggcagaagtgccgttgctggataataacggcaaatttaatggtcagtacgaactgcgtctgatggtggcgctggatgtcgg
tggtgcaatcaaaggccaacacttcgatatctatcaagggatcgggccggaagccggacaccgcgcaggttggtacaaccactatggacgt
gtctgggtgctgaaaaccgccccgggcgcaggtaacgtctttagcggctgatgtgatattctgtatgccgtttttacggatatgacagggtgaggg
gtaacctcacatcctgattattgactaagtgcgcgttgttcatgccggatgcggcgtgaacgccttatccggcctacaaatcatgcaaatccaaa
atattgcaggagttatgtaggcctgataagcgtagcgcatcaggcatttatgcgtttagggtgagggataacctcgcccttttttaattctgaggtgct
atgtctgtggtaattagtgatgcatggcgtcagcgttttggtggcacagcgcgtctgtatggtgaaaaagcgttgcaactgtttgctgacgcgcata
tttgtgtggttggtatcggtggtgtcggttcctgggcggcggaagcgctggcgcgcacggggattggcgcaatcacgcttatcgatatggatgat
gtgtgcgtcaccaataccaatcggcaaattcatgccctgcgcgataacgttgggctggcaaaagcggaagttatggcggagcgtattcgcca
gattaacccggagtgccgtgtaacggtggtggatgatttcgtgacgccggataacgtagcgcagtatatgagcgtgggttattcgtacgtgattg
atgccattgatagtgtacggcccaaagcggcgctgattgcttattgtcggcgcaataaaatcccactggtcacaaccggtggcgcgggtgggc

agattgatccgacgcagattcaggttaccgatctggcgaaaacgattcaggacccgctggcggcgaagttgcgcgagcgcctgaaaagcg
attttggcgtagtgaaaaacagtaaaggtaagctcggcgtggattgcgtgttttctactgaagcgctggtgtacccgcagtcagacggtacggtg
tgtgcgatgaaagccacggcagaagggccgaagcggatggattgtgcatctggatttggcgcggcaacgatggtgaccgccacctttgggttt
gttgcggtttctcatgcgctgaagaagatgatggcgaaagcggcgcgtcagggttaaacaatcaggtcggatgagacgcgaacgtcttatccg
acccggttctgtgcttaaacctgcttcgtcgcagcgataatcgcctcgcttaacgcatttaacccctggctgcgtgaggcgctaagctgcgcacgt
aatcccagctcatcaaacaatgccagtggtgactgtgcctgcaactcggcggcggttttcccctcaacggcagtcaacaacaccgccagcag
gccgcgcacaatgcgcccttcgctgtcgccaaagaaatgcattttgccgttttcagccactgtatatcccagccagacgcggttttcgcatccgg
caatctctttagcctgcgcttttaactcgtctggcaatgccggaagctgtttccccagcatgatcaactggcgatatttatcttcccattgcgtcaacg
gtgcgaaggtattgcgtaacgtttctgcggttacggttgtgccgaacggatgtccggcgaattgcgggtttgtcattaatccaccaataattccagc
gcgcggtcaacggcattcaccagcgcatccacatcactctttgtattatatggcgcaaaagaggcgcgcagtgtgccggttacgcctaattctg
ccagtagcggctgagcgcaatgctgcccggcccgcagggcaataccgtactccgccagcagcgtcaccatatcgctatgatgaacgccag
caaaatcaaaggccagcaggctggaatcctggcagcggaatgaacgaaagccgggacgtttcgccagcgcatcttccgccagcgttgcta
agctacggctccagctttcggcctggttgatatcgtaatctgccagccattccagcgccgcgcttaatcctatgacaccagcgacatttggcgttc
cagcttccagtttccacggcgcagattgagtcgtgaagccgtcaaaactcacttcgtgaaccattttgccgccgcccagccagggcgacatcg
cctccagcagttctgatttaccatacagcacgccgatacctgtcgggccatacagtttgtgacctgaaaaagcatagaaatcaatatccagttgc
tgaacatccgcggggaaatgcactgccccctgagcaccatcaaccatcaccaccatcccggctgaatgagcaaaggtaatcgctcgcgcc
agatccgggcaaccgccagtaacgttcgacatctgacccaacgccagaatccgactacggggagtaatcagttctggcaacaaatcgacat
ccggcagtcgctgcgcattaagcggcaatttcaccactttggctccagtttgttgggcgaccatcagccaggggacgaggttggcgtggtgttct
gccacgctgacaataatctcatcgcccggttgcagacgcggacgcgcatagcattgtgccaccatgttgatggattcagtggtgccgcgcgtcc
agacgatagttttatcatccggtgcattcagtaattgcgccactttctctcgtgcagcttcataacgcgcggtcaggcgttgggcttcggcaaactg
gctgcgatggacgtttccggcgctcagactgtaaaactgttgggtggcttcaaccacggcttcaggtttaagcgcggtcgcggcgctgtcgagat
agacgcccgcatcctgtagtgcgggaaactgggcgcgaaactgcgcgggattaaaaacgttcatggtactcctcggcttgatgcaccgatcgt
gacgcaaaattcactttgatacaaggagagtgctaaccatcagaattatctggatatcctggcgaagccgcgtaagtaacatatgctgaatagt
gttaggcgttaattttagcctgttaatcaatactattaagatttattaatcgctttaaggagaagatgatgaaaaagactgccgcaattatttctgcctg
tatgctgacttttgccctgagcgcctgttccggttcgaactatgtgatgcacaccaatgacggacgtaccatcgtctctgacggcaaaccacaga
ctgataacgataccggtatgatttcgtataaagacgctaatggcaacaaacagcagatcaaccgtactgacgtgaaagagatggtcgaactg
gatcagtaattcgcgatcgcaaggtaaaaaaaagcaccgcaattaggcggtgctacattaatcactatggacagacagggtaaatgtacag
gaagtgaaaaaaggtagctttgctaccatggtctgaatcgcagaccaattgcaaacacaacaacacaacatcacaaccgtaagccaaaag
ttcaccagaacacgcattccgataaaacttttcgttccggctcaggaagtgccgccactataggtatttgctggtagaagctcaacggacaattta
taatggctcagattaaaaaaactaataggttacatagtgtgatctaattgttaaattcatttaacatcaaagtttaatagccatgtctaaacgattacc
accgctaaatgccttacgagtttttgatgccgcagcacgccatttaagtttcactcgcgcagcagaagagctttttgtgacccaagccgcagtaa
gtcatcaaatcaagtctcttgaggatttttggggctaaaactgttccgccgccgtaatcgttcactcctgctgaccgaggaagggcaaagctattt
cctcgatatcaaagagatattttcgcaattaaccgaagcgacgcgtaaactccaggcccgtagcgccaagggggcgttgacggtcagtttact
ccccagtttcgccattcattggttggttccgcgactttccagctttaattcagcttatccgggaattgacgttcgaatccaggcggttgatcgtcagga
agataagctggcggatgatgttgatgtggcgatattttatggtcggggcaactggccggggctacgggtggaaaaactgtacgccgaatatttat
tgccggtgtgttcgccgctactgctgactggcgaaaaacccttgaagacaccggaagatctggctaaacatacgttattacatgatgcttcgcgc
cgtgactggcagacatatacccgacagttggggttaaatcatatcaacgttcagcaagggccaatttttagccatagcgccatggtgctgcaag
cggctatccacgggcagggagtggcgctggcaaataacgtgatggcgcaatctgaaatcgaggccggacgtcttgtttgcccgtttaatgatgt
tctggtcagtaaaaatgctttttatctggtttgtcatgacagtcaggcagaactgggtaaaatagccgcctttcgccaatggatcctggcgaaagc
cgctgctgaacaagaaaaattccgctttcgttatgaacaataatttacgtagggtacgaccatgaccagccgttttatgctgattttcgccgccatt
agcggcttcatttttgtggctctgggcgcttttggcgcgcatgtgttaagtaaaaccatgggggccgttgagatgggctggatccagaccggcctc
gaataccaggcgtttcatacgctggcgatcttaggtctggcggtggcaatgcagcgtcgcatcagtatctggttttactggagtagcgttttcctcg
cgttaggcacggtgttgttcagcggcagcctttattgcctggcgctgtcccatctgcgtttgtgggcgtttgtcactccggttggcggcgtgagcttcc
tcgcgggctgggcgttaatgttagttggtgctatccgtttaaagcgcaagggcgtaagtcatgaataaggttgtattgctgtgccgtccgggctttg
aaaaagagtgcgccgcagaaattaccgataaagccggccagcgggaaattttcggttttgcccgcgtgaaagagaatgcgggttatgtcattt
atgaatgttatcaacctgatgatggcgataagttaatccgtgagctgccgttcagttcattaatttttgcccgccagtggtttgtggtgggggaactcc
tgcagcatttgccgccagaagatcgtattacccccattgtcggcatgttacagggcgtagtagagaagggcggtgaactgcgtgttgaagttgc
cgataccaacgaaagcaaagagttactgaaattctgccgtaaatttactgttccgctacgcgctgccttgcgcgatgccggggtgctggcgaac
tatgaaacgccgaagcgtccggttgtgcatgtattctttattgcaccaggctgctgctataccggttactcatacagcaacaataattcgccgttcta
tatgggcattccgcgcctgaaatttccggcagatgcgccgagtcgttccacgctcaaactggaagaggcatttcatgtgtttattcctgcggatga

gtgggatgaacgcctggcgaacgggatgtgggcggtggatttaggcgcttgccctggcggctggacctaccaactggtgaagcgcaacatgt
gggtttattccgtcgacaacggcccgatggcgcaaagtctgatggataccggacaggtgacgtggctgcgggaagacggtttcaaattccgtc
cgacgcgcagcaatatctcctggatggtatgcgatatggttgaaaaaccggcgaaagttgcggcattgatggcgcagtggctggttaatggct
ggtgccgtgaaaccattttcaacctcaaactgccgatgaaaaaacgctacgaagaagtgtcacacaatctggcgtatattcaggcacagcttg
atgaacatggcataaatgctcagattcaggcgcggcagttgtatcacgatcgcgaagaagtgacggtgcacgtccgccgcatctgggctgcg
gtgggtggtcgtcgcgacgagcgataacaggcaaggccggggcgtcgtctccggcctgctaccctttccgtcaggctgttaccaaagaagttg
caacctggcgctcatcagaaataatgtgcgttacctcatccagatgcccgatgcgggcttcccctgaacgattaaatttacttttatcaatcaataa
caacgattgcgcggcacgttttaatagcatcgatttgtaatcagcgttgatcgcattggagtcccacagtgcgccgctgctatcgatcccttcaca
agaaaaaataaacagatcgatttccagcgatttaagttgggaaatcagcgagggattgacgtaacagccatatttgcgctcaagtgtgccgcc
ggaactgatcagttgaatgcgttcgcgtttaccgagttcatggcaaatcggatggctattggtgaagacctgaatgttgatgtcaggcaactggc
gtgccagataccagcaagttgaactggcgtctaaggctatcaccatcccttcttcaatccacgcgagcgcctcgcgcgcgatatctgctttatgc
gcataatggcttttcagcctgatgtgaaagggatcgccactgtcttgattttgacggtggatatatttagcgcgtccatgattgcgcagaattttaccc
tgcgtctgtaattcattgagatcgcgacgaatggtttctttactgacctttagctgttcagagagagcttccgtggtcaggctggtatggttcagcagc
aggtcgactatcgcttgctggcgtgccgctttcatatttttcaccccatcattcgataaccgcccgcgtcatgcgggcgtaccggtatgagattacg
gtgttaccccttttttaaaagaatattcccgtacttcgctcgttcgcctgtgataacgatcgcaaaggctttttgcgcccgttcataaaacgcaaaac
gattgatgcggatgatgtcaggacacggggcttgtagtgaaagcgcattacggtaacgtcgttctacttcaggatcgagagtgtcaccttctacc
gccgccatcatcaccagcggcggtgcataactgtccagttcaaataacgggataatcgcctggagcaagtcgctcaccaacaggccatcag
cgcggatcacctgcggtcccatcgaatgggcgggaaagtgagcatcggaaaaaataatttcatctccatgtcccatctctgccagcactttcaa
tagttcgggagaaattaacggcgaaattgttttcagcatttcacacttcctctataaattcaggttcagtttgcgggtagaaataacggtactgataa
tgaatctgtgcgcgggcttcttccgggctgttaaattcccctacgccataccaaccgaacagcgcagctcctgcgacggtcgtttcggcgtcgtc
gagaacttttaccggaatatcaagcatattggctttaatctgattccacaatgtgttgcgacttcctccaccgactaacaataattcagaggcctta
aagtgcccgatttttccagcatctgtagattgcgctgtaattgcgcagttaacccttccagcgccgcgcgatagaaatgcccccgcgtggtatta
agcgtcactccttgccagccagcgttctgacacgacaataaatcacactgcatttttacgccatccgcgccaggcgcgatcagacgagcttctt
caatcaacatttgccagggtgtttcagccgtccagaacagttttctcacccattccagcacgccggatgccagccattgcatacctgggttataca
accctgcctggctatccagttcgcaggtggaaccggcgtactgacttaacagcgaagtatcaacctgggcgctgcgaaccattaaaatttccc
atgtaccggaagagagcacgggttcattttgttcagcaccagcgccaaaaagggcgaactgggtatcgtgacctgcggaaatcaccggtatg
ccaacgggtaagccgagcattgctgcggcgctgttctgtagcgtaccaatctgttcacccgcttccaccagacgagggaagagtcggcgtgg
aataccggtggcttgtaaaatttgcggactgaaatcgcgttgctggatatccagcatctggctggttccggccatcgtgatatcagtagtgaattcg
ccggttaaacggtggttaatcagcgacgaaataaagagccaggcgtgcgcgcgttccagcagttgtggatgatttctttcaaccacaccaact
tatataacgtattgaaactaaaggctccgacgccagaaatagcctgcaaccgctgtgcggagattaaccgttcaatattgtccataaccgctgct
gttcgcggacatttccagctaataatcggatagagcagattgccttgcttatctaccagagcgccatccacaccaaaggtggtgacggcgatac
cgcggatgtggcattcagtcagttcactattgatttgccgacagcaatcagcaaagcgttgcaaaatggcgtctaaagaccactggtgccaggt
gttgttttccatcgcgatatcgctggcattaggcgttgaggcgcgggcaacaattttgccctgccgattaaccgcgatggccctgacattggtcgc
gccacagtcgagtaccaggataacttcttgtttcataatcgctcctgcaatatagccggataacattgcttatccggctaaccactcttgcttgttca
ggcccggtagccggagcgaccgggcatcacatcagggagtaatgtattaacgcttgtacaacggaccgtagttctggcaagcgcggtaatcc
tggccttcaatatccatgccgtgcgcagcccaggcagaaggacgatacactttggtctcttcaacgttgtgcatacataccgggatacgcagca
tggaggcgagagtgataaagtctgcgccaacgtggccgatggtcagaaccccatggttagcgccccagttcgccattaccgagtacacatcc
gtaaacggccctttaccggtgaggcgcggtgcaaaccaggtggttggccaggttgagttggtgcgtttgttgaggatgtcatgcacatccttcgg
caattccacgctccagccttccgcgatttgcagtaccggtcccaggcctttgatgatgttgacacgagtcatggtgaacgggacgccgccttcgg
taaggaagcgggaagagtaaccgccgccacggaagtattcgtggatcgccgggcaccattcggtagcggcgaggcaagcgtcagcctctt
gctgagagatttcccagtgtggcttcatcgtcgggttaccttcgctgtcgcgttgtttacaggaaccgtccagcgcagcagaaccggagttgatca
aatggatgatgccgtgttctgccagtccatccagtttatgccccgttacacgctcaattgcttctggtgaccagtaggtacgcacatcggcaaata
cctgagcggtgccggtgagctggtgacccattagcattgccacgccgttaagactgtcgttttcggtcgccacgacaaagggttcgcgcacgcc
attccagtcaaatgaactgttgaggatcgcttcggcggtgtcaccattgggatattgatcggtccagtgacgttgcccctggaagcccgcagcga
tggcgttgtagccaagtgattcttccacgcgaccaatatcggccagtttgctgttgccttgcatcatgtcgcggatacacatcgccatcagtaaact
ttcgcgcagaactgcgcggctttgctcggcattacgttgatactgtttgttattttcatcttcgccatagcggaagtttttatcagcccaggccagtgcc
atttccaattcggcttcgtcgtaaatcttctgatcgatacggcgacgcagttcggtcatatccaccgcctggactttcattcccagccaggattcaaa
gaagttgtgatcaacaatggaaccggcgatacccatcgaaacgccgcccagcgacagatagcttttacctttcatgctggcgacggccaaac
cggcgcgggcaaagcgcagcagtttttcttcaacatcggcaggaatcgatgtgtcatcggcatcctgaacgtcatgaccgtaaatggagaatg
ctgggatgcctttctggctgtgagctgccagagccgctgccaggtaaacagcgccggggcgttcagtgccgttaaagccccaaatggccttcg

ggcgggttggatccatgtcgatggtttcactgccatagcaccagcaaggcgttaccgtaatggtgaggcctacattctgactgctgaatttttcttc
gcaagcagcggcttcagccatacccgcgatacaggtatcggaaatgacacactcgacggcagctccgcaggcatggcgcagtttctcggtc
agcagtgcggccgtagctttcgccatattcattgtttgttcttcaagcgactcacgaacacccatgcgacgaccgtcaataaccgggcggatacc
aattttcggtaagctgattttttcattacagattcctcactttattcggaaaatatgttcagttagttgccgtttgagaacggaaacgggcaaagataa
agatgaccgcgaagcagagtgcggggatcagttcagcagtggggatgttgcccgccgcgtcactgacaaaacccatgaccggagtgaca
ataccgccgccaataatggtcataacgatgaaggacgaaccatatttggtgtcctggccgagattcttaatgcccagcgagaagattgttgggt
actgaatcgacataaaggcgctgcataaagtcagggctattaagcccacatgaccgccagcgaaggctgagatcaggcacagtgccatag
cgattaatgcgtaggcggccaggactttgtgtggtgcgaagcgactgatgagccaggtaccggtgaaacgaccaataaagaagcacaccat
ggttccggttaaatagttagcggcaaagcctgcagtcatacctggaatttcttctacagcgtagcgaatcaaatagctccagcaggccgtttgtg
cgccgacatagcagaattgcgctaataccgcccagcgccagtggcgaatacgcgccaggcgagaaagcgatgcggagaacgatccttgtt
tggcgtcactgtgattatcactctgcaatgccgggaatttcgtcagcatgatcagcagggcgaccagtaacacgatagccacgatgatcatata
aggtgtctgtaccgataataccaggctgtgtttatacgcactcaattgctctggagacattttatcgagaacgtcttgcgattgatgtggcacgttag
acaaaataagactttgcccaaagacaaccgcgataattgcgccaaacgagttaaatgtttgcgcaagatttaagcggaagtgaccactacttt
ccggccctaataccgtaacaaaagggtttgcggcagtttccagacaacctaatccggctgcaataataaataggccaactaaaaacaaggt
gtagttcattatttctgcggcgggccagaataatgcagcacccaaggcatataaaaataacccggtaataatccctgctttataactgagtttttc
atcaatatcccagcagggattgggataatgaaataaccaaagtaaaaggccgattggatcaggccagcctggaaatttgtcagcgtaaaag
cctgctggaattgaggtaataaaatgtcgttaaggttattggctaccgcccaaagaaaaaacagtgagcacagcagcgcgaatggaataatg
taacttctgctttgccctgcatctttatctaccgcacggtaactctgcgtttgtattgatgtgtttcccatagcatcctcttaggttcagaagcttaagcac
gtaatcggttatgttttcaaaagtgcaaaaccagcgagttcgggcttcatatcagtgaatatttgcctgtctgaaatgaactcatggattcattttcaa
ataaaaaacgctctgtaatatgacggcggtcacacttattagctagaaggtgactttatgtgactaccatcactttaataatcgaaatttcatgccc
gtttcagaaaataaatcataaaaaacacaaatcagatgaccgtattaattgtttgtgccgaaaacggtcattccgggcttggaggaatagaaaa
atgaccgttttgataaatatttttaagagcggtcatttttaatggggttataaaaagagaacttgcagttatttactgtgattagttgaaccaggtcaca
aaatataagcttgtgaatttctttcttgttaatcaggaagaagatggtgggtaattaaacggctaattcaatagtgtgaaaggaacaacattattgc
cctgttttgaatcagagagaggtagctaatggaacgaaataaacttgctcgtcagattattgacacttgcctggaaatgacccgcctgggactga
accaggggacagcggggaacgtcagtgtacgttatcaggatgggatgctgattacgcctacaggcattccatatgaaaaactgacggagtcg
catattgtctttattgatggcaacggtaaacatgaggaaggaaagctcccctcaagcgaatggcgtttccatatggcagcctatcaaagcagac
cggatgccaacgcggttgttcacaatcatgccgttcattgcacggcagtttccattcttaaccgatcgatccccgctattcactacatgattgcggc
ggctggcggtaattctattccttgcgcgccttatgcgacctttggaacacgcgaactttctgaacatgttgcgctggctctcaaaaatcgtaaggca
actttgttacaacatcatgggcttatcgcttgtgaggtgaatctggaaaaagcgttatggctggcgcatgaagttgaagtgctggcgcaactttac
ctgacgaccctggcgattacggacccggtgccagtgctgagcgatgaagagattgccgtagtgctggagaaattcaaaacctatgggttacg
aattgaagagtaatttcgtaaagcaacaaggagaaggatgatggctaacagaatgattctgaacgaaacggcatggtttggtcggggtgctgt
tggggctttaaccgatgaggtgaaacgccgtggttatcagaaggcgctgatcgtcaccgataaaacgctggtgcaatgcggcgtggtggcga
aagtgaccgataagatggatgctgcagggctggcatgggcgatttacgacggcgtagtgcccaacccaacaattactgtcgtcaaagaagg
gctcggtgtattccagaatagcggcgcggattacctgatcgctattggtggtggttctccacaggatacttgtaaagcgattggcattatcagcaa
caacccggagtttgccgatgtgcgtagcctggaagggctttccccgaccaataaacccagtgtaccgattctggcaattcctaccacagcaggt
actgcggcagaagtgaccattaactacgtgatcactgacgaagagaaacggcgcaagtttgtttgcgttgatccgcatgatatcccgcaggtg
gcgtttattgacgctgacatgatggatggtatgcctccagcgctgaaagctgcgacgggtgtcgatgcgctcactcatgctattgaggggtatatt
acccgtggcgcgtgggcgctaaccgatgcactgcacattaaagcgattgaaatcattgctggggcgctgcgaggatcggttgctggtgataag
gatgccggagaagaaatggcgctcgggcagtatgttgcgggtatgggcttctcgaatgttgggttagggttggtgcatggtatggcgcatccact
gggcgcgttttataacactccacacggtgttgcgaacgccatcctgttaccgcatgtcatgcgttataacgctgactttaccggtgagaagtaccg
cgatatcgcgcgcgttatgggcgtgaaagtggaaggtatgagcctggaagaggcgcgtaatgccgctgttgaagcggtgtttgctctcaaccgt
gatgtcggtattccgccacatttgcgtgatgttggtgtacgcaaggaagacattccggcactggcgcaggcggcactggatgatgtttgtaccgg
tggcaacccgcgtgaagcaacgcttgaggatattgtagagctttaccataccgcctggtaaatgcgctgatgtgataatgccggatacgacgttt
gcggcgtatccggctcgccgttaccgtaccaaccgcaattgctgtaaattgccgtcgatatgcaaatcggtttgtaagcgggcaatatcgcggc
acagaaacgccatctctttatgggtttctaatttttgcgccacttttcggcaaccgcatccagattctcatatatcccttccagactctgaaactcgac
cagcagctgcgtggcgctttttggtccgattcccgcaacacccggtacctttgaactgctgatccccgcaagtccccagtaatcgggcaactgct
gcggttgaacgccaaattctttatcgataaatggcgcatccagccaacgtttctggaagtaatcacgaatacgtaatgtcggtgaaagtaactga
cagtagcctttatctgtcgaaacaatcgttgcctgatgcccggcctgtgtcactttgaccgccagcgtggcggctaagtcatcggcttcgttgccgc
tggttgaccagcacgggacgccgcgttgctcaaaggcggcgcgtaatgcaggcatctcgtcgtgcaactcttccggcattggcggtcgacccg
ctttgtaatctggtaaacgctgatgacgccagccgctactgcggttttcatcatcaaaaacggcgaccgcgtgggttggctggctgtgcataatg

agctgatcgagcgcatgctggcaggtttcgacacagggcgacccctgaacggcatgaatgcgacgaataagattcagtgcatcgacaataa
gcaaatgaacagccacgataacccccttaaagatcaggcgataagggtaacattcgatgccggaacaggctacggctggatgagaaatcg
ggaagaggcctcgcaaaacgaggcctttggagagcgattaatcgcaggcaacgatcttcattgccaggccgccgcgagaggtttcgcggta
cttggcgttcatatctttacctgtttcgtacatggtttcgataactttatcgaggcagacgcgcggctcgctggtacggcgcagcgccatacgtgcg
gcgttcaccgcttttactgccgcaatggcgttacgctcgatgcatggcacctgtacctgtccggcgaccgggtcacacgtcagaccgaggttgtg
ctccatggcgatttccgccgcgatgcacacctgcgccgggcttgcgcctaatagttctgccagaccagccgccgccattgagcacgccacgc
caacttcaccctggcaacccacttcagcaccagaaatcgacgcgttcatcttataaagagaaccaatggcgctggctaccagcaggtaacga
gccagtgagttagcgttcacttcgcggataaacttgtcgtagtacgccagaactgccgggataatcccgcacgcaccgttagtcggcgcagtc
accacgcgaccgccagcagcgttctcttcgttcactgccagtgcaaacatgttgatccagtcaacaaccgccatcgggtcagtggtggttttatc
ctggctgaccagcatccggcgtagtgccgcagcacggcgtggaacgcgcagtttgccaggcaacacgccttcggtggaaataccgcgctca
ataccgccgcgcatgacttcccagacgttcgccaggtgctgttccagctcttctttgctgtgcagcgccagctcgtttttcatcatcaggccagaga
gtgacagcccggtttcctgacaatgtttttgcagatcggctgctgaactgtacggataaggaacttcaaccggtgcgctatcctgctggccaaaat
gctcttcatcaacgataaagccaccgccaatagagtagtaagtctggctgtaaacgactttatcgcccgccagcgcggtaatgcgcataccgtt
ttcatgcagagaaaggttgtcggcgtggaagttcatgcactgatcaaccgggaactccacttcatgctgaccgtttgccagcatcaggcgacca
tgagtattcacatcctgaataaaactggggatggaatcgatatccacggtatccggcaggttacccgccaggcccataataatggcgatatca
gtgtggtggcctttaccggtcagagagagcgagccgtacacgtcaaccaccacgcgggtcacgtctttaagcaggttacgggcaatcagatc
gtcggtaaattgtttacccgctttcattggtccaacggtatgagaactggaagggccaatgccgattttgaaaatatcgaatacgctaatcatagg
aaatacatcgcgttaaaacggaggaagcgccgcccgaaagcggcgcgaaaggacttagctgaacagagagtagaagattgcggagatt
gcaatcagacccatcacgacaacgaatacgttgctgatgtgaccgctgtacttacgcattgccggtactttctgaattgcgtacatcggcatcag
gaacaggatcatcgcgatgattggaccgcccagggtttcaatcatacccaggatgctcgggttcagggtggcaacaatccaggtcgttaccag
catgaacagcgcagtgatacggttcagcttgttgatttcgatagacttacctttaccacgcagagatttaatcaccataccgttgaagccttcacgt
gcgcccaggtagtgaccgaggaaggatttggtgatagcgataatcgcgataatcggagccatccacgcgataaccggtgcgttaaagtggtt
agccaggtaagacagaatcgagatgttctgctctttagccgcagccaggtctgccggagtcaggctcagtacacagctgaagacgaagaac
attacggtcagcaccatcatgatgtgtgcgaatgccaggatcttcgagcatttctgttctgccatatcgccgtactcttcacgcttcgcaacggcga
aagaagagatgatcggagagtggttgaacgagaacaccattaccggaattgccagccacagggtcatccacagaccgtttccggttgcaga
tgcagtgtccagagacagcgtttccagtgcagcgccgttccactgcgggatcaggtacagagccagcagcatcagtacgccaacaaacgg
gaataccagaatactcatcgctttaacgatcatctgctcaccgaagcgaacgatggtcatcatacccacgatcaggatcagcgacagaatcg
cacgcggcggtggcgtcatacccagctggtgagacatgaagctttcaacggtattggtgattgccacgctataaaccagcaggatcgggtag
atagcgaagaagtagagcagggtaatcagtttacctgcgccaataccaaagtgttcttctacaacctcggtgatgtcttcgcccgggtttttacca
gacagtacgaagcgagtcaggccgcggtgagcaaaaaacgtcatcgggaacgcaaggatagccatgatgatcagcgggatcataccgc
caacaccggcgttgattggcaggaacagcacgcccgcgccgattgccgtgccgtaaaggcccagcatccacatggtgtctgtcttgcgccag
gcactacgagagtctttcgacgcaatcgtgctggtttgagtcgtttccatctatttctcctggaggaatgcaaataattcagcttttagccagttcagc
ccgggaaggtgcctgaccgtaaaaatgaaatctgtctatcggcgatggcttaatcatttaccgccgtaataatttgagggcggaaagatacaca
caagttatgtatctatcagtgatcttgatctcaaatacaataatctacttggaagatagattatcccaacgctttactctgtaaatcaaaagtaaaag
agaatttaagggcaatatttgcccattaaacgacatctgacttcttccggggcgcgcaatatatgtaggttgaactgaaaaaatcaatacttttagt
taatcatggcgataatttctttttcatagcgataaaatacggtttatgaaaatagggaaacgtttgcgttgcaacataaattgtatgacatattaactg
tgatgatatttgtcgcgtgagggtggtaagaaacattcatataacaaatgaaatgagttgatgagtaagtgaccaaaaacgagaaaacccgg
caacaaacgtcaccgggggagcggagattacgtgcagatttcgtagcaagggatgtaggctgagcctggcaacttcatacgatgctgggca
acaaaaccctgtagcaggtcgtccatacgacgcataatctctttatcgccgttgattttgtaaggaccaaactcttcaatggcgcgaataccgact
tcttttacgttacccgccacaataccggagaacgcacggcgcaggtcggcagccagcacttcaacaggttgatccgggtaaagcttcagatta
gccatattctcgtgagacggctcaaacggcatttgcaaatctggcgcaatgcgcattgaccagttaaagctgtaggcatcgcctgtatcacggc
gattttctttcaccagcggcatcgattttttcatctgacgagcgacttcagcggcgtcatcaatgatgatgcggtaatggcggcgcgcgttttcaccc
agcgtatgcacgacaaactcgtccagtacgcggaagtagtcggcgctctctttcgggccggtgaggatcaatggtaaaacctgatctttgttgg
ccgggttcattaaaattcccagcaaatagagcaactcttctgccgtacccacaccgccagggaagataatgataccgtgagcgatacggaca
aacgcttccagacgtttttcgatatctggcatgatgatcaattcgttgaccagcgggttaggcggttcagcggcgataatcgacggctctgtcata
ccaataaaacgactgtctttgtaacgctgctgcgcgtgtccgaccgcagcacctttcatcggcgcttccatcgctcccggaccacagccggtgc
agatattcagctcacgcaggcccagctggtttccgacgcgacgggcatacaaatactcgttttcgttaattgagtgaccgccccagcagaccac
catatttggcgcttcacccacatgcagcgcgcgagcgttacgcaagatggaaaagaccaggttagtgatgtggacggagttgtcgagattcag
gttgggaaaacgaacggtgttatggatttgcccgtaaacgaagagaatgtcacgcaggaccgcgaacaagttggcctgcaaagcgcgaat
aattcgcccatcgacaaaagcctcttccgggggattaatcagttccagctttacgccgcgttcacggcgcaagacgttaatatcgaaattttcaa

aacgagacagcaattctttgctgttatcggtcaaactaccggagttcagtacggcaagtgaacagttgcgaaacagttgatagaggtcgctgct
ggcggtgcgtttaagcatatccacttccagctgcgacaacatatccatggagccaagcgggctaatatgtgtaatcaagaaaaactccttatgg
gacgaatatcgtcattccctttgattacaatagccctgcctgatgacctttcacaaccttctgcgtgaatccagcacgcaaaattgagaaaaaattt
attgccgaaccagtcttgtggtcgatgggacaaaatcgctattactgcgccacgggttaatgtccagaccgccacgacgggtataacgtgcgta
aacgctcaatttttctggctggcagaagcgtaacaggtcattaaagatgcgttccacgcactgttcgtggaactcgttgtgatgacggaatgaga
ccaggtaacgcagcagtttttctctgtcaa...

Supplemental Table 2, Python scripts

Script 1

Searches through DNA sequences to identify spacer sequences and records matching results along with relevant metadata:

```
import os

import csv


def reverse_complement(sequence):

    complement = str.maketrans("acgt", "tgca")

    return sequence.translate(complement)[::-1]


def hamming_distance(seq1, seq2):

    """Calculate the Hamming distance between two sequences."""

    return sum(el1 != el2 for el1, el2 in zip(seq1, seq2))


def allowed_mismatches(sequence, query, max_mismatches):

    """Find all positions where the query matches the sequence with allowed mismatches."""

    matches = []

    query_len = len(query)


    for i in range(len(sequence) - query_len + 1):

        subseq = sequence[i:i + query_len]

        mismatches = hamming_distance(subseq, query)

        if mismatches <= max_mismatches:

            matches.append((i, subseq, mismatches))  # Include mismatches for concatenation

    return matches


def process_fastq(input_fastq, output_csv, max_mismatches):

    search_sequence = "tatgtttagagtgttccccgcgccagcggggataaacc".lower()


    results_found = False

    all_results = []  # List to store all results before writing to the CSV
```

```
    try:
        with open(input_fastq, 'r') as fastq_file:
            while True:
                header = fastq_file.readline().strip()  # Read and store the sequence header
                sequence = fastq_file.readline().strip().lower()
                fastq_file.readline()  # Skip the plus line
                fastq_file.readline()  # Skip the quality score line

                if not sequence:
                    break

                # Search in forward strand
                forward_matches = allowed_mismatches(sequence, search_sequence, max_mismatches)
                for position, mismatched_seq, mismatches in forward_matches:
                    following_33bp = sequence[position + len(search_sequence):position + len(search_sequence) + 33]
                    concatenated = mismatched_seq + following_33bp
                    all_results.append([concatenated, mismatches, "Forward", following_33bp, header])
                    results_found = True

                # Search in reverse complement
                rev_comp_sequence = reverse_complement(sequence)
                reverse_matches = allowed_mismatches(rev_comp_sequence, search_sequence, max_mismatches)
                for position, mismatched_seq, mismatches in reverse_matches:
                    following_33bp = rev_comp_sequence[position + len(search_sequence):position + len(search_sequence) + 33]
                    concatenated = mismatched_seq + following_33bp
                    all_results.append([concatenated, mismatches, "Reverse", following_33bp, header])
                    results_found = True

        if results_found:
            # Sort the results: forward strand at the top, reverse strand at the bottom
```

```
        all_results.sort(key=lambda x: x[2])  # Sort by the 'Strand' column ("Forward" before "Reverse")

        # Reassign the count after sorting
        for count, result in enumerate(all_results, start=1):
            result.insert(0, count)  # Insert the count at the beginning of each row

        # Write the sorted and updated results to the CSV file
        try:
            with open(output_csv, 'w', newline='') as csv_file:
                writer = csv.writer(csv_file)
                # Updated headers based on the new requirements
                writer.writerow(["Count", "Leader-Repeat-Spacer Unit", "Number of Mismatches", "Fastq
Strand", "Spacer", "Metadata (Fastq Header)"])
                writer.writerows(all_results)

            print(f"Results successfully written to '{output_csv}'.")

        except IOError as e:
            print(f"Error: Unable to write to the file '{output_csv}'. {e}")
    else:
        print(f"No matches found for '{search_sequence}' in '{input_fastq}'.")

  except FileNotFoundError:
    print(f"Error: The file '{input_fastq}' was not found.")
  except IOError as e:
    print(f"Error: There was an issue with reading the file '{input_fastq}'. {e}")
  except Exception as e:
    print(f"An unexpected error occurred: {e}")

def process_directory(directory_path, max_mismatches):
  if not os.path.isdir(directory_path):
    print(f"Error: The directory '{directory_path}' does not exist.")
    return
```

```
    # Loop through all files in the directory
    for filename in os.listdir(directory_path):
        if filename.endswith(".fastq"):
            input_fastq = os.path.join(directory_path, filename)
            output_csv = os.path.join(directory_path, filename.replace(".fastq", ".csv"))
            print(f"Processing file: {input_fastq}")
            process_fastq(input_fastq, output_csv, max_mismatches)

if __name__ == "__main__":
    try:
        directory_path = input("Enter the directory path containing FASTQ files: ").strip()
        max_mismatches = int(input("Enter the number of allowed mismatches: ").strip())

        if not directory_path or max_mismatches < 0:
            print("Error: A valid directory path and a non-negative number of mismatches must be provided.")
            exit()

        process_directory(directory_path, max_mismatches)

    except ValueError:
        print("Error: Invalid number of mismatches provided. It must be an integer.")
    except Exception as e:
        print(f"An unexpected error occurred: {e}")
```

Script 2

Converts csv files generated from script 1 into FASTA format:

```
import os
import pandas as pd

def csv_to_fasta():
    # Prompt for the directory path containing the CSV files
    directory = input("Enter the directory path containing the CSV files: ")

    # Iterate through each file in the directory
    for filename in os.listdir(directory):
        # Process only .csv files
        if filename.endswith('.csv'):
            # Define the full path to the input file
            csv_file = os.path.join(directory, filename)

            # Create the corresponding output FASTA file name
            fasta_file = os.path.join(directory, filename.rsplit('.', 1)[0] + '.fasta')

            # Read the CSV file
            df = pd.read_csv(csv_file)

            # Clean column names (strip whitespace and convert to lowercase)
            df.columns = df.columns.str.strip().str.lower()

            # Verify columns
            print(f"Processing {filename} with columns: {df.columns}")

            # Extract base name of CSV file for FASTA headers
            base_name = filename.rsplit('.', 1)[0]

            # Open the FASTA file for writing
            with open(fasta_file, 'w') as fasta_out:
```

```
        # Iterate through each row in the DataFrame
        for index, row in df.iterrows():
            # Get the sequence and count
            sequence = row['spacer']  # Updated to match the correct column name
            count = row['count']  # Assuming the count is in the 'count' column

            # Create the FASTA header without .csv in the name
            header = f">{base_name}_{count}"

            # Write the header and sequence to the FASTA file
            fasta_out.write(f"{header}\n{sequence}\n")

    print(f"Finished writing to {fasta_file}")

# Run the function
csv_to_fasta()
```

Script 3

Concatenates multiple FASTA files:

```
import os


def concatenate_fasta_files(directory, output_file):
    fasta_files = [f for f in os.listdir(directory) if f.endswith('.fasta')]


    if not fasta_files:
        print("No FASTA files found in the directory.")
        return


    with open(output_file, 'w') as outfile:
        for fasta_file in fasta_files:
            with open(os.path.join(directory, fasta_file), 'r') as infile:
                outfile.write(infile.read())


    print(f"Concatenation complete! Output saved as: {output_file}")


def main():
    directory = input("Please enter the directory path containing the fasta files: ").strip()


    if not os.path.isdir(directory):
        print("Invalid directory path.")
        return


    output_file = input("Please enter the name for the output fasta file (e.g., output.fasta): ").strip()


    if not output_file.endswith('.fasta'):
        output_file += '.fasta'


    concatenate_fasta_files(directory, output_file)


if __name__ == "__main__":
```

main()

Script 4

Removes duplicate spacer sequences and orders the remaining spacers by their frequency:

```
from Bio import SeqIO

def remove_duplicates_with_count_sorted(input_fasta, output_fasta):
    sequence_counts = {}
    sequence_records = {}

    for record in SeqIO.parse(input_fasta, "fasta"):
        seq_str = str(record.seq)
        if seq_str in sequence_counts:
            sequence_counts[seq_str] += 1
        else:
            sequence_counts[seq_str] = 1
            sequence_records[seq_str] = record

    sorted_sequences = sorted(sequence_counts.items(), key=lambda x: x[1], reverse=True)

    records_to_write = []
    for seq_str, count in sorted_sequences:
        record = sequence_records[seq_str]
        record.id += f"_count{count}"
        record.description = ""
        records_to_write.append(record)

    SeqIO.write(records_to_write, output_fasta, "fasta")
    print(f"Processed sequences. Unique sequences sorted by count written to {output_fasta}")

input_fasta = input("Enter the input FASTA file name: ")
output_fasta = input("Enter the output FASTA file name: ")

remove_duplicates_with_count_sorted(input_fasta, output_fasta)
```

Script 5

Removes any spacer sequence that has an 80% or greater sequence identity to the parental spacer in the RC5311 LacZ reporter CRISPR array:

```
from Bio import SeqIO
from Bio import pairwise2

input_fasta = input("Enter the input FASTA file name (including path if necessary): ").strip()
output_fasta = input("Enter the output FASTA file name (including path if necessary): ").strip()

reference_seq = "gctttcgcagacgcgcggcgatacgctcacgca"
threshold_identity = 80  # Percent identity threshold

def calculate_percent_identity(seq1, seq2):
    """Calculate the percent identity between two sequences."""
    alignment = pairwise2.align.globalxx(seq1, seq2, one_alignment_only=True, score_only=True)
    percent_identity = (alignment / max(len(seq1), len(seq2))) * 100
    return percent_identity

filtered_sequences = []
try:
    for record in SeqIO.parse(input_fasta, "fasta"):
        identity = calculate_percent_identity(str(record.seq), reference_seq)
        if identity < threshold_identity:
            filtered_sequences.append(record)

    SeqIO.write(filtered_sequences, output_fasta, "fasta")
    print(f"Filtered sequences have been saved to {output_fasta}")
except FileNotFoundError:
    print(f"Error: File {input_fasta} not found. Please check the file name and path.")
except Exception as e:
    print(f"An error occurred: {e}")
```

Script 6

Automates a BLASTN workflow that compares the spacer sequences to a reference database, extracts PAM sequences and annotates the data with match counts and frequency adjustment:

```
import subprocess

import os

import csv

from collections import Counter


def reverse_complement(seq):

    complement = str.maketrans('ACGTacgt', 'TGCAtgca')

    return seq.translate(complement)[::-1]


def extract_pam(sequence, sstart, send, sstrand):

    if sstrand == 'plus':

        pam_position = sstart - 4

        if pam_position >= 0:

            return sequence[pam_position:sstart-1]

    elif sstrand == 'minus':

        pam_position = sstart

        if pam_position + 2 < len(sequence):

            pam_sequence = sequence[pam_position:pam_position + 3]

            return reverse_complement(pam_sequence)

    return "N/A"


def read_sequences(file_path):

    sequences = {}

    print(f"Reading sequences from {file_path}...")

    try:

        with open(file_path, 'r') as f:

            seq_id = None

            for line in f:

                line = line.strip()

                if line.startswith('>'):
```

```
                seq_id = line[1:]
                sequences[seq_id] = ''
            elif seq_id:
                sequences[seq_id] += line
        print(f"Loaded {len(sequences)} sequences.")
    except FileNotFoundError:
        print(f"Error: File {file_path} not found.")
    except PermissionError:
        print(f"Error: Permission denied for file {file_path}.")
    return sequences


def perform_blast(query_fasta, db_name):
    temp_output_file = f"{os.path.splitext(query_fasta)[0]}_temp_results.txt"
    blastn_command = [
        "blastn",
        "-query", query_fasta,
        "-db", db_name,
        "-out", temp_output_file,
        "-outfmt", "6 qseqid sseqid pident length mismatch gapopen qstart qend sstart send evalue bitscore
sstrand"
        ]
    print(f"Running BLAST command: {' '.join(blastn_command)}")
    try:
        subprocess.run(blastn_command, check=True)
        print("BLAST search completed.")
    except subprocess.CalledProcessError as e:
        print(f"An error occurred during BLAST search: {e}")
        return None
    return temp_output_file


def process_blast_results(temp_file, query_sequences, db_sequences):
    jb028_results = []
    p1656_results = []
    aligned_to_p1656 = set()
```

```
    with open(temp_file, 'r') as temp_file:
        lines = temp_file.readlines()
    for line in lines:
        fields = line.strip().split('\t')
        if len(fields) >= 13:
            try:
                pident = float(fields[2])
                length = int(fields[3])
                if pident == 100.0 and length == 33:
                    qseqid = fields[0]
                    sseqid = fields[1]
                    sstart = int(fields[8])
                    send = int(fields[9])
                    sstrand = fields[12]
                    query_sequence = query_sequences.get(qseqid, 'Unknown')
                    db_sequence = db_sequences.get(sseqid, '')
                    pam_sequence = extract_pam(db_sequence, sstart, send, sstrand)
                    if query_sequence:
                        pam_sequence += query_sequence[0]
                    pam_sequence = pam_sequence.upper()
                    if "JB028" in sseqid:
                        jb028_results.append([qseqid, query_sequence] + fields[1:3] + fields[3:8] + [sstart, send] + fields[10:] + [pam_sequence])
                    elif "p1656" in sseqid:
                        p1656_results.append([qseqid, query_sequence] + fields[1:3] + fields[3:8] + [sstart, send] + fields[10:] + [pam_sequence])
                        aligned_to_p1656.add(qseqid)
            except ValueError:
                pass
    return jb028_results, p1656_results, aligned_to_p1656


def calculate_match_counts_and_frequency(results):
    sequences = [row[1] for row in results]
    sequence_counts = Counter(sequences)
```

```
    for row in results:
        sequence = row[1]
        match_count = sequence_counts[sequence]
        frequency_adjustment = 1 / match_count
        row.append(match_count)
        row.append(frequency_adjustment)

def write_output(jb028_results, p1656_results, aligned_to_p1656, output_file):
    with open(output_file, 'w', newline='') as csvfile:
        csvwriter = csv.writer(csvfile)
        column_headings = [
            "Count", "Spacer ID", "Sequence", "Database", "pident", "length", "mismatch", "gapopen",
            "qstart", "qend", "sstart", "send", "evalue", "bitscore", "sstrand", "PAM",
            "Spacer Match Counts", "Frequency Adjustment"
        ]
        csvwriter.writerow(column_headings)
        count = 1
        calculate_match_counts_and_frequency(p1656_results)
        calculate_match_counts_and_frequency(jb028_results)
        for row in p1656_results:
            csvwriter.writerow([count] + row)
            count += 1
        for row in jb028_results:
            if row[0] not in aligned_to_p1656:
                csvwriter.writerow([count] + row)
                count += 1
    print(f"Results saved to {output_file}.")

def main():
    print("Script started.")
    db_name = input("Enter the base name of the BLAST database (e.g., JB028-p1656): ")
    db_fasta_file = f"{db_name}.fasta"
    if not os.path.isfile(db_fasta_file):
```

```
        print(f"Error: The file {db_fasta_file} does not exist.")
        return
    db_sequences = read_sequences(db_fasta_file)
    directory = input("Enter the directory containing .fasta files: ")
    if not os.path.isdir(directory):
        print(f"Error: The directory {directory} does not exist.")
        return
    fasta_files = [f for f in os.listdir(directory) if f.lower().endswith('.fasta')]
    if not fasta_files:
        print("No .fasta files found in the directory.")
        return
    for fasta_file in fasta_files:
        fasta_path = os.path.join(directory, fasta_file)
        if not os.path.isfile(fasta_path):
            print(f"Skipping invalid file: {fasta_path}")
            continue
        query_sequences = read_sequences(fasta_path)
        temp_results = perform_blast(fasta_path, db_name)
        if temp_results is None:
            continue
        jb028_results, p1656_results, aligned_to_p1656 = process_blast_results(temp_results,
query_sequences, db_sequences)
        output_file = f"{os.path.splitext(fasta_file)[0]}-blast-{db_name}.csv"
        write_output(jb028_results, p1656_results, aligned_to_p1656, output_file)
        if os.path.exists(temp_results):
            os.remove(temp_results)
    print("Script completed successfully.")


if __name__ == "__main__":
    main()
```

Script 7

Produces a radial plot from binned spacer data produced from script 6:

```
import pandas as pd
import numpy as np
import matplotlib.pyplot as plt
from math import pi

input_file = input("Please enter the name of the input CSV file (with extension): ")

data = pd.read_csv(input_file)

print("Available columns:", data.columns)

data.columns = data.columns.str.strip()

if 'Range' not in data.columns or 'Spacer Count' not in data.columns:
    raise KeyError("CSV file must contain 'Range' and 'Spacer Count' columns.")

ranges = data['Range']
spacer_counts = data['Spacer Count']

N = len(ranges)

max_value = max(spacer_counts)
scaling_factor = 0.3
normalized_counts = spacer_counts / max_value * scaling_factor

angles = [(n / float(N) * 2 * pi) + (pi / 2) for n in range(N)]
bar_width = 2 * pi / N * 0.8

fig, ax = plt.subplots(figsize=(12, 12), subplot_kw={'polar': True})

bars = ax.bar(
```

```
    angles,
    normalized_counts,
    width=bar_width,
    bottom=0.5,
    color='black',
    edgecolor='black',
    alpha=0.7
)

ax.set_xticks([])
ax.set_yticks([])
ax.set_ylim(0, 1)

ax.spines['polar'].set_visible(False)
ax.grid(False)

output_file = "radial_chart.png"
plt.savefig(output_file, dpi=600, bbox_inches='tight')

plt.tight_layout()
plt.show()
```

Script 8

Takes PAM sequences and produces a Logo plot:

```
import os

import pandas as pd

import logomaker

import matplotlib.pyplot as plt


# Function to load PAM sequences from the selected Excel file

def load_pam_sequences(filename, column_name="PAM"):

    df = pd.read_excel(filename, engine="openpyxl")

    if column_name not in df.columns:

        raise ValueError(f"Column '{column_name}' not found in the file.")

    pam_sequences = df[column_name].dropna().astype(str).tolist()

    if not pam_sequences:

        raise ValueError("No valid PAM sequences found in the selected file.")

    return pam_sequences


# Function to generate the logo from PAM sequences

def create_logo(pam_sequences, output_filename="pam_logo.png", labelled=True):

    counts_df = logomaker.alignment_to_matrix(pam_sequences, to_type="counts")

    pwm_df = counts_df.div(counts_df.sum(axis=1), axis=0)


    # Create a new figure for each logo (important for multiple logos)

    fig = plt.figure(figsize=(0.9055, 0.6772), dpi=300)

    ax = fig.add_subplot(111)

    logo = logomaker.Logo(pwm_df, ax=ax, color_scheme="classic")


    # Set up the x-ticks and labels

    logo.ax.set_xticks([])  # Only 3 positions in the sequence

    logo.ax.set_xticklabels([], fontsize=12)

    logo.ax.set_xlim(-0.5, 2.5)


    # Set the y-axis label to "Frequency" or "Relative Frequency"
```

```
    logo.ax.set_ylabel("Frequency", fontsize=12)

    if not labelled:
        # Remove x and y labels for the unlabelled version
        logo.ax.set_xticklabels([])  # Remove x-tick labels
        logo.ax.set_yticklabels([])  # Remove y-tick labels
        logo.ax.set_ylabel("")  # Remove y-axis label

    # Set custom y-ticks: 0.0, 0.5, 1.0
    logo.ax.set_yticks([0.0, 0.5, 1.0])

    # Increase the axis (spine) thickness to 2pt
    logo.ax.spines['left'].set_linewidth(1)
    logo.ax.spines['bottom'].set_linewidth(1)

    # Increase tick thickness to 2.5pt
    plt.tick_params(axis='y', which='major', width=1, length=3)

    # Remove the top and right borders (spines)
    logo.ax.spines['top'].set_visible(False)
    logo.ax.spines['right'].set_visible(False)

    # Save the figure to the specified output file
    plt.savefig(output_filename, dpi=300, pad_inches=0)
    plt.close()  # Close the figure after saving to prevent overlap
    print(f"Logo saved as {output_filename}")

# Main function
if __name__ == "__main__":
    folder_path = os.path.abspath("PAM_plots")
    os.makedirs(folder_path, exist_ok=True)

    # Ask the user for the input file using input() instead of Tkinter
```

```
input_file = input("Please enter the full path to the Excel file (with .xlsx or .xls extension): ")

# Ensure a file was selected
if not input_file:
    print("No file selected. Exiting.")
    exit()

try:
    print(f"Processing file: {input_file}")
    pam_list = load_pam_sequences(input_file)

    # Get the base name of the file (without extension)
    base_filename = os.path.splitext(os.path.basename(input_file))[0]

    # Generate the output file name with the same base name but .png extension
    labelled_output_image = os.path.join(folder_path, f"{base_filename}_labelled.png")
    unlabelled_output_image = os.path.join(folder_path, f"{base_filename}_unlabelled.png")

    # Generate logo with labels
    create_logo(pam_list, output_filename=labelled_output_image, labelled=True)

    # Generate logo without labels
    create_logo(pam_list, output_filename=unlabelled_output_image, labelled=False)

except Exception as e:
    print(f"Error: {e}")
```

Supplemental Table 3 Probability Calculations

Assumptions used in all calculations

Base composition is taken as uniform (A=C=G=T=0.25) and codons are treated as independent.

Only the spacer segment contributes variable in-frame codons. For our reporter, 30 bp of spacer sequence are in frame with lacZ, which is 10 codons.

Stop codons are TAA, TAG, TGA (3 of 64).

Start codon considered is ATG (1 of 64).

A) Theoretical detection limit of the papillation assay

A spacer–repeat insertion restores the reading frame, but the spacer itself can introduce an in-frame stop before lacZ. The probability that a given in-frame codon is not a stop is 61/64. For 10 in-frame codons, the probability that none of them is a stop is:

$P(\text{no in-frame stop in spacer}) = (61/64)^{10}$

Numerical value:

$61/64 = 0.953125$

$(0.953125)^{10} = 0.618726\ldots \approx 0.62$

So the theoretical detection fraction is about 62 percent. We rounded this to ~63 percent in the main text.

B) Probability that a 30 bp spacer contains at least one in-frame ATG

There are 10 in-frame codons. The probability a given codon is ATG is 1/64. The probability that none of the 10 codons is ATG is $(63/64)^{10}$, so:

$P(\text{at least one in-frame ATG}) = 1 - (63/64)^{10}$

Numerical value:

$63/64 = 0.984375$

$(0.984375)^{10} = 0.854291\ldots$

$1 - 0.854291\ldots = 0.145709\ldots \approx 0.146 \approx 15$ percent

C) Probability that a 30 bp spacer contains an in-frame ATG that is not immediately followed by an in-frame stop

We consider adjacent in-frame codon pairs across the 10-codon spacer: there are 9 such pairs (positions 1–2, 2–3, …, 9–10). A given pair qualifies if the first codon is ATG (1/64) and the second codon is not a stop (61/64). Treating pairs independently (a good approximation at these low probabilities), the probability that at least one qualifying pair occurs is:

$P(\text{ATG followed by non-stop somewhere}) = 1 - [1 - (1/64)*(61/64)]^9$

$= 1 - [1 - 61/4096]^9$

Numerical value:

$61/4096 = 0.014893\ldots$

1 − 0.014893… = 0.985106…

(0.985106…)^9 = 0.873679…

1 − 0.873679… = 0.126320… ≈ 0.126

Notes

Using these same assumptions, the detection fraction in A scales as (61/64)^N if the number of in-frame spacer codons is N. We used N=10 because 30 bp of the spacer are in frame with lacZ in our construct.

The ATG metrics in B and C are baseline expectations for random sequence. Any sequence bias (e.g. GC content, PAM-linked composition) would nudge these numbers slightly but not change their order of magnitude.
